# Conserved sequence motifs in human TMTC1, TMTC2, TMTC3, and TMTC4, new O-mannosyltransferases from the GT-C/PMT clan, are rationalized as ligand binding sites

**DOI:** 10.1186/s13062-021-00291-w

**Published:** 2021-01-12

**Authors:** Birgit Eisenhaber, Swati Sinha, Chaitanya K. Jadalanki, Vladimir A. Shitov, Qiao Wen Tan, Fernanda L. Sirota, Frank Eisenhaber

**Affiliations:** 1 Bioinformatics Institute (BII), Agency for Science, Technology and Research (A*STAR), 30 Biopolis Street, #07-01 Matrix, Singapore, 138671 Republic of Singapore; 2 Genome Institute of Singapore (BII), Agency for Science, Technology and Research (A*STAR), 60 Biopolis Street, Singapore, 138672 Republic of Singapore; 3 Siberian State Medical University, Moskovskiy Trakt, 2, Tomsk, Tomsk Oblast 634050 Russia; 4 School of Biological Science (SBS), Nanyang Technological University (NTU), 60 Nanyang Drive, Singapore, 637551 Republic of Singapore

**Keywords:** TMTC1, TMTC2, TMTC3, TMTC4, PMT, Dolichyl-phosphate-mannose-protein mannosyltransferase, GT-C glycosyl transferase, O-mannosylation, Membrane topology, Transmembrane region prediction

## Abstract

**Background:**

The human proteins TMTC1, TMTC2, TMTC3 and TMTC4 have been experimentally shown to be components of a new O-mannosylation pathway. Their own mannosyl-transferase activity has been suspected but their actual enzymatic potential has not been demonstrated yet. So far, sequence analysis of TMTCs has been compromised by evolutionary sequence divergence within their membrane-embedded N-terminal region, sequence inaccuracies in the protein databases and the difficulty to interpret the large functional variety of known homologous proteins (mostly sugar transferases and some with known 3D structure).

**Results:**

Evolutionary conserved molecular function among TMTCs is only possible with conserved membrane topology within their membrane-embedded N-terminal regions leading to the placement of homologous long intermittent loops at the same membrane side. Using this criterion, we demonstrate that all TMTCs have 11 transmembrane regions. The sequence segment homologous to Pfam model DUF1736 is actually just a loop between TM7 and TM8 that is located in the ER lumen and that contains a small hydrophobic, but not membrane-embedded helix. Not only do the membrane-embedded N-terminal regions of TMTCs share a common fold and 3D structural similarity with subgroups of GT-C sugar transferases. The conservation of residues critical for catalysis, for binding of a divalent metal ion and of the phosphate group of a lipid-linked sugar moiety throughout enzymatically and structurally well-studied GT-Cs and sequences of TMTCs indicates that TMTCs are actually sugar-transferring enzymes. We present credible 3D structural models of all four TMTCs (derived from their closest known homologues 5ezm/5f15) and find observed conserved sequence motifs rationalized as binding sites for a metal ion and for a dolichyl-phosphate-mannose moiety.

**Conclusions:**

With the results from both careful sequence analysis and structural modelling, we can conclusively say that the TMTCs are enzymatically active sugar transferases belonging to the GT-C/PMT superfamily. The DUF1736 segment, the loop between TM7 and TM8, is critical for catalysis and lipid-linked sugar moiety binding. Together with the available indirect experimental data, we conclude that the TMTCs are not only part of an O-mannosylation pathway in the endoplasmic reticulum of upper eukaryotes but, actually, they are the sought mannosyl-transferases.

**Supplementary Information:**

The online version contains supplementary material available at 10.1186/s13062-021-00291-w.

## Background

The family of TMTC (transmembrane (TM) and tetratricopeptide (TPR) repeat-containing) proteins in human is represented by four paralogues: TMTC1 (isoform X3 with accession XP_016875493, 875 residues (AA); see comment below why sequence Q8IUR5 (882 AA) appears doubtful), TMTC2 (Q8N394, 830 AA), TMTC3 (Q6ZXV5, 915 AA) and TMTC4 (Q5T4D3, 741 AA). Their common sequence architecture consists of an N-terminal segment with transmembrane regions and intermittent loops and a C-terminal stretch of multiple, in the order of 10 TPR repeats.

After having long been genes with unknown function, first functional information trickled in from genome-wide association (GWAS) and family (FS) studies that linked TMTCs with neurological/psychiatric diseases, sensory organ disorders but also with other conditions. Although an original, GWAS-based claim for TMTC2 in primary open-angle glaucoma in a Japanese cohort [1] could not be confirmed in several follow-up studies (for Afro-Caribbean [2], Chinese [3], Japanese [4], Korean [5], Saudi Arabian [6] and South Indian [7] cohorts), new GWAS evidence for another set of single nucleotide polymorphisms in cohorts of mixed ethnic origin reemphasizes the link [8]. Optic disc area is influenced by TMTC2 in cohorts of European and Asian ancestry [9].

TMTC1 has been related to schizophrenia (via GWAS [10]) and it is differentially expressed in inflammatory bowel disease linked arthritis [11]. The circular RNA circTMTC1 inhibits skeletal muscle satellite cell differentiation in chicken [12]. TMTC2 is associated with non-syndromic sensorineural hearing loss (SNHL; via both GWAS and FS [13, 14]). TMTC2 interactions with certain miRNAs hint towards a role in Parkinson’s disease [15]. GWAS associates TMTC2 with obesity in Caribean Hispanics [16] and Han Chinese [17], left ventricular mass increase [16] as well as with immune conditions such as eczema, asthma and ‘atopic march’ [18]. Family studies show TMTC3 mutations being causative for cobblestone lissencephaly [19] and periventricular nodular heterotopia with intellectual disability and epilepsy [20]. Genetic inactivation of TMTC4 in mice causes rapid, early postnatal cochlear hair cell death, leading to hearing loss [21]. TMTC4’s role in influencing bone mineral density is known from a transcriptome-wide association study [22].

Hence, the diversity of clinical effects hints towards human TMTCs having, most likely, very basic molecular and cellular functions with pleiotropic, context-specific effects. TMTC1 [23], TMTC2 [23], TMTC3 [24, 25] and TMTC4 [21, 25] were found to be located in the endoplasmic reticulum (ER). For all TMTCs, the TPR-containing C-terminal segment was shown to be located in the ER lumen (TMTC1/2 [23], TMTC3/4 [25]). TMTC1/2 were associated with intracellular calcium homeostasis [21, 23]. TMTC3 was reported to have a potential role in ER stress response [24], TMTC4 was linked with unfolded protein response [21].

Dramatic progress in understanding TMTC function was recently achieved by Danish researchers collaborating with several American groups [25, 26]. Knockout of all four TMTCs in HEK293 cells abolished O-mannosylation of a variety of cadherin and proto-cadherin proteins; thus, the TMTCs are members of a new O-mannosylation pathway that selectively processes cadherin-like targets [26]. Apparently, the presence of various TMTCs affects the spectrum of modified cadherins since the selective TMTC1/3 knockout (with TMTC2/4 remaining functional) produces a larger set of O-mannosyl glycopeptides in the mass-spectrometric analysis [26]. Further, TMTC3 complementation at the background of a combined four TMTC knockout in HEK293 cells rescues the O-mannosylation of E-cadherin and enhances cellular adherence [25]. TMTC3/4 knockdowns were demonstrated to delay gastrulation in frog [25]. Three known TMTC3 disease mutations in the N-terminal protein half (H67D, R71H, G384E) were shown to exhibit reduced protein half-life despite native ER localization.

Having followed the TMTC story since 2012, we were puzzled by the difficulties to consistently interpret the sequence-analytic findings in terms of biological function, a problem so nicely summarized by Larsen, Graham et al. [25–28]. It starts with something apparently simple such as the largely varying predicted transmembrane region (TM) numbers for various TMTCs due to evolutionary sequence divergence within their membrane-embedded N-terminal region and it does not end with the diversity of enzymatic activities and substrates of homologous proteins (largely sugar transferases), sometimes even with known 3D structure. In this work, we explore:

(i) To which extent can the sequence architecture of TMTCs be unified, especially with regard to their number of TMs?
(ii) What is the nature of the sequence segment homologous to Pfam model DUF1736?
(iii) Can the conservation of sequence motifs among TMTCs and known homologous sugar transferases (including those with known 3D structure) be rationalized in terms of catalysis and ligand/substrate binding?

## Methods

If not otherwise mentioned, all sequence-analytic operations were carried out with the ANNOTATOR software suite [29, 30], an in-house tool developed over ca. 20 years that integrates more than 40 academic tools (either self-programmed or used with permission of the original authors) for the prediction of protein structural and functional features. In the context of this work, the battery of programs for prediction of transmembrane regions, cellular export signals and for sequence similarity searches were especially important. In cases where completeness and recent updates of sequence and domain databases were critical, selected locally executed similarity searches were repeated on the respective websites supported by the original authors (BLAST [31, 32], HHpred [33, 34]) to make sure that no important hit from recent database additions was omitted.

Structural modelling of TMTCs by homology was carried out with Modeller (version 9.4) [35]. As it became clear during the subsequent analyses that the TMTCs harbor a binding site for a lipid-linked sugar, we used the Schrodinger suite [36] for the placement of this ligand. Subsequent induced fit relaxation and energy optimization of the complex followed published procedures [36–42].

## Results

### Collection and sequence architecture of the TMTC1/2/3/4 superfamily

Pairwise similarity searches using the BLAST tool [31, 32] and starting with any of the full-length human TMTC1, TMTC2, TMTC3 and TMTC4 sequences conveniently gather the superfamily of true TMTC orthologues in upper Eukarya and of TMTC-like proteins in other organisms including many hypothetical proteins, if at all, automatically annotated by sequence similarity.

The sequence architecture of human TMTCs is two-partite with an N-terminal segment consisting of transmembrane regions and intermitting loops (456 AA for N-TMTC1, 475 AA for N-TMTC2, 426 AA for N-TMCT3 and 462 AA for N-TMTC4) and a remaining C-terminal part comprising TPR repeats. This result was obtained by analysing human TMTC1/2/3/4 within the ANNOTATOR environment [29, 30]. We applied the suite of transmembrane prediction tools (DAS-tmfilter [43, 44], HMMTOP [45, 46], PHOBIUS [47, 48], TMHMM [49, 50] and TOPPRED2 [51, 52]) as well as comparisons with protein domain and protein repeat databases (PFAM [53], SMART [54], Miguel Andrade’s repeats [55]) via HMM searches [56, 57].

When we repeat the simple BLAST searches with just these N-terminal segments of TMTC1/2/3/4, apparently the same superfamily of TMTCs is collected (in the order of ~ 10,000 hits with E-value < 3.e-4 and above 60% query sequence coverage; details not shown). Phylogenetically, true TMTC orthologues and TMTC-like proteins are found throughout the eukaryote kingdom with homologues even among prokaryotes but the set of four paralogues per organism with full coverage of the N-terminal domain can be systematically detected only from vertebrates down to the insect level. Already in the complete genome of the worm *Caenorhabditis elegans*, just two TMTCs are known (TMTC1: Q20144/NP_509123, TMTC2: NP_504200).

**Fig. 1:**
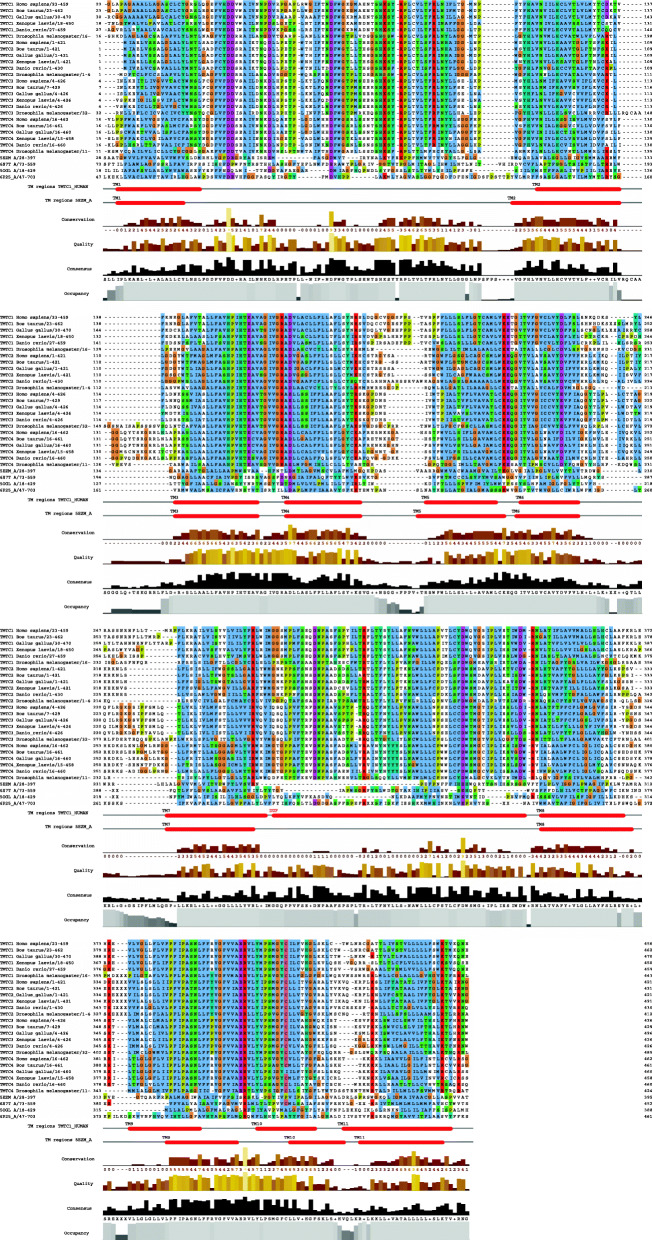
Grand alignment of N-terminal segments of TMTCs together with sequences of selected sugar transferases with known 3D structure. We show the grand alignment of the full set of the N-terminal segments of TMTCs from six organisms (*Homo sapiens* (Hs), *Bos taurus* (Bt), *Gallus gallus* (Gg), *Xenopus laevis* (Xl), *Danio rerio* (Dr), *Drosophila melanogaster* (Dm)) together with the sequences taken from 5ezm chain A [58], 6s7t chain A [59], 5ogl chain A [60] and 6p25 chain A [59]. For supporting navigation in the alignment, the location of the TMs in human TMTC1 and in 5ezm are shown. The figure was generated with Jalview [61] using an externally created and manually edited multiple alignment (in the SEAVIEW environment [62, 63]). The location of TMs in TMTC1 follows the observations from the 3D structural model created in the course of this work and, at some places, does differ slightly from the sequence-analytic predictions provided in Additional File 2. The following sequence segments have been excluded from the alignment and replaced by “XX”: in TMTC1_B, 244–304 after TM6; in TMTC1_Gg, 251–310 after TM6; in TMTC1_Dm, 358-417after TM8; in TMTC2_Hs, 337–393 after TM8; in TMTC2_Bt (G3MY32_BOVIN), 334–393 after TM8; in TMTC2_Gg (F1NPM4_CHICK), 324–380 after TM8; in TMTC2_Xl, 337–393 after TM8; in TMTC2_Dr (F1R0Y9_DANRE), 346–401 after TM8, in TMTC2_Dm, 360–504 after TM8; in 6S7T, 288–348 after TM6 and 486–535 after TM10; in 6P25, 219–261 after TM6, 312–531 after TM7 and 560–585. Please note that, as result of the excluded sequence stretches in some sequences, the residue numbering in the figure might deviate from the residue numbering in the respective entry of the sequence database. Additional information for this figure is provided in Additional Files 1 and 2 available with this article. For locating specific residues in the alignment, we recommend first finding the nearby TMs and then looking for conserved motifs next to them

We created a grand alignment of the full set of the N-terminal segments of TMTCs from six animal organisms (*Homo sapiens*, *Bos taurus*, *Gallus gallus*, *Xenopus laevis*, *Danio rerio*, *Drosophila melanogaster*; see Fig. 1 and Additional file 1) to study family-specific and superfamily-wide sequence conservation patterns.

As a first goal during the alignment creation, we wanted to understand the number and sequence localization of TM regions in the human TMTCs. In the literature, the number of TM regions in the N-terminal segment of various human TMTCs is reported to be different for various TMTCs and between 8 and 12 [25–28]. The confusion is not surprising as TM region predictors behave erratically in the twilight range of their scoring function [43]. Just one additional polar residue can bring the hydrophobicity of the candidate sequence segment below the threshold. And the boundaries of TM regions are typically heuristically determined bringing the length near 20 residues.

This variation of TM region number among TMTCs is potentially conflicting with evolutionarily conserved function as the latter requires homologous loop segments being located in the same subcellular space (in the ER or in the cytoplasm). Thus, membrane topology needs to be conserved among species within a given TMTC family and, to a large extent, also among various TMTC paralogues. As a further constraint, the C-terminal, TPR-comprising region is shown to be located in the ER for all TMTCs [23, 25].

For all 24 sequences in Fig. 1, locations of potential TM regions were identified with the full suite of the five TM predictors in the ANNOTATOR [29, 30]. In total, we find 12 regions with hydrophobic motifs that are predicted as TM regions in at least some sequences for three out of four families TMTC1, TMTC2, TMTC3 and TMTC4 (see Additional File 2). Four major discrepancies and issues are observed:

1. The most N-terminal TM region might actually be a signal peptide.
2. In the human TMTC1 sequence as in Q8IUR5, there is no hit for TM7. But it does exist in the sequence version of TMTC1 with accession XP_016875493 (isoform X3).
3. In human TMTC3, TM3 is only weakly recognized.
4. All TMTC sequences have a segment with significant sequence similarity to the Pfam domain DUF1736 (E-value < 1.e-30 for any of the human TMTCs in an HMMER search against Pfam-A [53]). The TM segment predictors suggest a TM region inside this segment for all human TMTCs except for TMTC2.

First, the most N-terminal hydrophobic region in all human TMTCs seems to be a true TM segment, maybe, a signal anchor but not a signal peptide as the sequence assessments with SIGNALP version 5 [64] show. The following loop contains the strongly conserved DD motif that, if having an enzymatic function, needs to be localized in the ER. Consequently, the N-terminus of TMTCs appears cytoplasmic. With the C-terminus in the ER, TMTCs need to have an uneven number of TM regions so that the TPR segment can reside inside the ER lumen [23, 25].

Second, we encountered serious difficulties when attempting to include the canonical TMTC1 sequence Q8IUR5 into the grand alignment, especially in the region that includes TM7 and the DUF1736 hit (which is much worse in Q8IUR5 with E-value=3.e-19 compared with other TMTCs). This would not have surprised anyone if the sequence were from a more obscure insect or fish genome but Q8IUR5 is a human protein. Searching human sequences with TMTC1 from *Bos taurus* or *Gallus gallus* delivers XP_016875493 (TMTC1 isoform X3) as the sequence that can be much easier aligned with TMTC1s from other species as well as with other TMTCs. At the same time, searching the *Bos taurus* or *Gallus gallus* proteomes with human Q8IUR5 does not deliver a better, more similar isoform than the best homologue found with XP_016875493. Thus, it cannot be excluded that Q8IUR5 has sequence errors in the region 245–312 (with the corresponding region 245–305 in XP_016875493 being the correct version). While none of the five TM region predictors finds a trace of a hit for TM7 in Q8IUR5, it is confidently predicted by the majority of them in XP_016875493.

Third, the evolutionary argument (see Fig. 1) strongly suggests that the respective regions for TM3 in human TMTC3 are just subthreshold for the TM predictors (compared with other human TMTCs, there are additional polar residues (Ser119, Ser120 and Ser124) in the respective sequence KSSVIASLLFAVHPIHT (residues 118–134) of human TMTC3).

Fourth, the sequence segment predicted to be a TM region as part of the DUF1736 hit is actually not membrane-embedded. When checking the TMTCs against sequences with known 3D structures via HHpred [33, 34] as implemented in the ANNOTATOR environment [29, 30], we find convincing statistically significant similarity of the N-terminal portions of TMTCs to structures such as 5ezm [58]. For example in the case of N-TMTC1, the E-value is 1.9e-22. Comparison with the alignment delivered by HHpred reveals that the segment FPNFFFI (261–267 in 5ezm), a small, quite hydrophobic helix at the ER side and with its axis parallel to the membrane, aligns with the segment 318–324 in human TMTC1. Notably, the segment 311–324 is the common core from TM predictions by four different TM predictors (TMHMM, PHOBIUS, DAS-tmfilter, and HMMTOP). Similar observations are available in other homologous structures. TMTC1’s segment 311–324 hits the same type of small, hydrophobic helix in the ER lumen parallel to the membrane in 5ogl (found with E-value 2.7e-15 by HHpred; segment 325–333 with sequence PEVFMQRIS [60]) or in 6s7t (found with E-value 2.4e-17 by HHpred; segment 382–389 with sequence GRFYSLWD [65]).

**Fig. 2:**
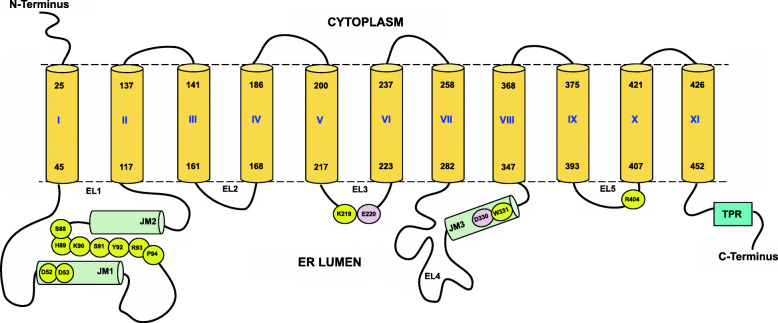
Cartoon of the membrane topology of the N-terminal domain of TMTCs and localization of important substructures and residues. The figure shows schematic representation of the overall structural elements and the connectivity of TMTCs. The TM helices are shown in yellow cylinders and marked as I to XI while the helical regions in the lumen are shown in green cylinders and are marked as JM1, JM2 and JM3. The lumenal loops are numbered from EL1 to EL5. The whole TPR region is shown as a single block colored in cyan. The figure also highlights important residues which are (i) the strictly conserved DD motif (M1, Table 4) in EL1 (loop between TM1 and TM2), (ii) conserved SHKSYRP motif (M2, Table 4) also present in EL1, (iii) conserved lysine residue of KET(Q) xxT motif (M4, Table 4) that forms a salt bridge with the phosphate group of DPM, (iv) glutamate residue from conserved KET(Q) xxT motif (M4, Table 4) in EL3 and aspartate residue of the conserved DW motif (M4, Table 4) in EL4, (v) strictly conserved arginine residue from conserved ERxxY motif (M7, Table 4) in loop EL5 between TM9 and T10. All the important residues are colored in yellow except the metal binding residues which are highlighted in pink. The sequence position numbering corresponds to TMTC1. The location of TMs in TMTC1 follows the observations from the 3D structural model created in the course of this work and, at some places, does differ slightly from the sequence-analytic predictions provided in Additional File 2

Thus, we can convincingly conclude (i) that the DUF1736-similar region in TMTCs, actually just a loop between TM7 and TM8 located in the endoplasmic reticulum lumen, does not contain a TM region, (ii) that all human TMTCs comprise 11 TM regions in their N-terminal sequence portion and (iii) that the N-terminus is located in the cytoplasm and the C-terminal TPR domain is in the ER lumen (see also Fig. 2).

Further, we wish to emphasize that the TM regions in TMTCs are largely of the complex type (the only consistently simple TMs are TM7 in TMTC3 from various species (data not shown)) [66, 67]; thus, their sequences contain evolutionary information beyond the generally not informative hydrophobic background (sprinkled-in polar residues, glycine and proline are typically rare in TMs [68, 69]) useful for sequence comparison in homology searches [70–72].

As mentioned by a reviewer, membrane topology prediction for proteins with TM regions has been attempted directly from sequence, typically following the TM segment prediction part [45, 46, 73]. As a trend, these prediction tools support the topology conclusions for the TMTCs but not always. For example, the probability for the N-terminus to be cytoplasmic was predicted by TMHMM [49, 50] as follows: TMTC1 0.61, TMTC2 0.64, TMTC3 0.89, TMTC4 0.30. We think that the predicted number of TM regions (especially their even/uneven number) critically influences the correctness of the topology prediction. For TMTC1/2/3, nine TM regions were found by TMHMM (uneven as in the case of the actual 11 TM regions) but this number was predicted ten for TMTC4.

### TMTCs are homologous to membrane-bound sugar transferases with known 3D structures

We summarized the findings related to the top hits of the HHpred searches with the N-TMTC1, N-TMTC2, N-TMTC3 and N-TMTC4 sequence segments in Table 1. The original HHpred outputs are available as supplementary material (Additional File 3). All the hits have excellent E-values (<< 1.e-10) despite low sequence identities of the respective sequence alignments (all values between 8 and 13%; e.g., TMTC1/2/3/4 align with 5ezm with sequence identities 8, 13, 10 and 12% in the HHpred-generated alignments respectively); thus, the match of the physico-chemical property pattern between the respective sequences is excellent, especially for the TM segments and some loop regions next to them.

**Table 1:** HHPred search with the N-terminal part of the four human TMTCs against PDB (PDB_mmCIF70_29_May, version 29/05/2020)

PDB ID	N-TMTC1 (1–456)	N-TMTC2 (1–475)	N-TMTC3 (1–426)	N-TMTC4 (1–462)
5ezmA/5f15A (578 AA) [58]	1.9E-22	5.9E-19	1.3E-21	4.2E-21
	Q: 1–456	Q: 1–475	Q: 2–424	Q: 1–460
	T: 7–399	T: 33–400	T: 27–395	T: 12–395
6s7tA (826 AA) [59]	1.8E-17	2.3E-15	3.1E-17	1.2E-16
	Q: 1–456	Q: 2–475	Q: 2–425	Q: 1–462
	T: 48–560	T: 75–559	T: 70–558	T: 53–559
6s7oA (705 AA) [65]	2.2E-17	4.9E-15	3.4E-17	4.1E-17
	Q: 8–456	Q: 2–475	Q: 1–426	Q: 1–461
	T: 1–479	T: 21–480	T: 12–477	T: 1–476
6eznF (718 AA) [74]	1.4E-17	3E-15	5.1E-17	1.7E-16
	Q: 9–454	Q: 1–474	Q: 2–424	Q: 3–461
	T: 1–467	T: 19–469	T: 14–465	T: 1–466
3wajA (875 AA) [75]	2.6E-17	8.6E-15	1.8E-17	1E-15
	Q: 12–456	Q: 1–474	Q: 1–425	Q: 7–459
	T: 1–490	T: 16–490	T: 9–489	T: 1–486
5oglA (713 AA) [60]	2E-16	5.7E-14	4.5E-16	1.1E-15
	Q: 11–455	Q: 2–475	Q: 1–426	Q: 4–462
	T: 1–432	T: 18–343	T: 12–433	T: 1–434
6p25A/6p2rA (817 AA) [59]	5.7E-14	7.8E-13	6.3E-14	1.8E-13
	Q: 1–453	Q: 2–473	Q: 1–257	Q: 1–303
	T: 26–703	T: 53–705	T: 45–290	T: 32–304
7bvfA (1102 AA) [76]	1.9e-12	1.2e-10	1.1e-12	2.2e-11
	Q: 33–456	Q: 7–473	Q: 13–426	Q: 26–462
	T: 263–631	T: 263–631	T: 263–633	T: 263–630
6sniX/6snhX (562 AA) [77]	1.5E-10	1.3E-08	1.8E-11	1.2E-09
	Q: 1–416	Q: 1–434	Q: 1–388	Q: 1–424
	T: 30–411	T: 54–411	T: 49–411	T: 35–411

The eight essentially full-length hits with best E-values and sequence coverage > 90% are tabulated: 5ezm, crystal structure of ArnT from *Cupriavidus metallidurans* in the apo state [58], 5f15 is the same as 5ezm but with undecaprenyl phosphate as analogue for a lipid-linked sugar substrate; 6s7t, cryo-EM structure of human oligosaccharyltransferase complex OST-B [59]; 6s7o, cryo-EM structure of human oligosaccharyltransferase complex OST-A [65]; 6ezn, cryo-EM structure of the yeast oligosaccharyltransferase (OST) complex [74]; 3waj, crystal structure of the *Archaeoglobus fulgidus* oligosaccharyltransferase (O29867_ARCFU) complex with Zn and sulfate [75]; 5ogl, structure of bacterial oligosaccharyltransferase PglB in complex with an acceptor peptide and an lipid-linked oligosaccharide analogue [60]; 6p25/6p2r, structure of *Saccharomyces cerevisiae* protein O-mannosyltransferase Pmt1-Pmt2 complex bound to the sugar donor and a peptide acceptor/without peptide ligand [59]; 7bvf_A, Cryo-EM structure of *Mycobacterium tuberculosis* arabinosyltransferase EmbA-EmbB-AcpM2 in complex with ethambutol [76]. We added also 6sni/6snh (cryo-EM structure of nanodisc reconstituted yeast ALG6 in complex with 6AG9 Fab or with Dol25-P-Glc [77]) because of the much shorter template length. For each query and each PDB structure (listed as PDB ID), we provide the E-value and the sequence ranges hit in the query (Q) and in the template (T; we also provide the length of the template in parentheses below the PDB identifier). The uppercase letter behind the PDB identifier denotes the relevant chain

Proteins with known structure discovered in these searches belong to the group of well-studied membrane-standing arabinosyl-, oligosaccharyl- or mannosyltransferases. Their annotated enzymatic domain is fully part of the alignment. Given the full-length coverage of the N-TMTCs’ sequences queried against the PDB, there is no doubt that N-TMTCs and the annotated enzymatic domains of sugar transferases detected share a common fold and have a similar 3D structure.

For all N-TMTCs, the sequence of the bacterial aminoarabinose transferase ArnT corresponding to structures 5ezm/5f15 [58] is the most similar homologue with an almost gapless alignment (with some exception for the N-terminal region of the loop between TM7 and TM8). The alignments of N-TMTCs generated by HHpred cover the first 11 of the 13 N-terminal TMs in 5ezm/5f15, nicely supporting the membrane topology consideration in the previous section (to note, TM region TM4 is missing and TM5/6 are annotated as a single large TM both in the PDB entry 5ezm and in the Uniprot entry Q1LDT6). As a result of the structural similarity, we can conclude that there are five loops between TM regions that form the structure in the ER lumen (see Fig. 2): (i) two long loops EL1 (between TM1/TM2) and EL4 (between TM7/TM8; both loops contain helical segments) as well as (ii) three short loops EL2 (between TM3/TM4), EL3 (between TM5/TM6) and EL5 (between TM9/TM10). In 5ezm/5f15 (as in other sugar transferases of this type), there are two substrate binding cavities that communicate via a channel limited, on one side, by the TMs in the membrane and, at the other side, by the long loop connecting TM7 and TM8 (i.e., EL4 in the case of TMTCs). One binding region is formed by the segments homologous to EL1, EL2 and EL4 and accommodates the sugar acceptor substrate. The other site (built by EL1 and mainly by EL4) provides for interaction with a lipid-linked carbohydrate (LLC; the sugar donor, e.g., a dolichyl phosphate or pyrophosphate with attached sugar/oligosaccharide moiety). In the zone of contact of the two substrates, a divalent metal ion important for catalysis is coordinated by amino acid residues of the transferase. Despite the vast differences in sequences and possible ligands, homology considerations suggest that the TMTCs are constructed following the same general architecture.

Most importantly, we see at the level of sequence comparison (even without any structural modelling) that some critical motifs strongly conserved among the TMTCs have a structural and/or functional equivalent (e.g., in ligand binding) in the 3D structures of enzymes found. The strictly conserved DD motif in the loop between TM1 and TM2 (e.g., D52/D53 in N-TMTC1) aligns with the known active site in several sugar transferases (e.g., D55/E56 in 5ezm_A, D77/E78 in 6p25_A or D281/D282 in 7bvf_A). All the sugar transferases found in our HHpred homology search have at least an aspartate that coincides with the first aspartate in this motif. This residue is described as binding to the polar group of the sugar acceptor and/or a divalent metal ion (e.g., for 5ezm/5f15 [58], 5ogl [60], 6s7t/6s7o [65] or 6sni/6snh [77]). Thus, these positions are absolutely critical for enzymatic catalysis since any residue substitution leads to loss of function. For example in 6p25/6p2r [59], E78 forms a salt bridge with R138 making D77 sticking out towards the cavity where it binds to the sugar acceptor substrate. Any replacement of D77/E78 abolishes enzyme function [59, 78].

In 5ezm/5f15, D158 (in EL2, N-terminal to TM4) interacts with the acceptor substrate and also forms a salt bridge with K203 (in EL3, C-terminal to TM5). The homologous residues are conserved in TMTCs (e.g., D169 and K219 in N-TMTC1) and, thus, are predicted to also play a role in ligand binding.

An arginine in the loop EL5 between TM9 and T10 close to the N-terminus of TM10 and strictly conserved among TMTCs (e.g., R404 in TMTC1 as part of the conserved sequence AERV) followed by a hydrophobic stretch of residues (from TM10) is also seen in sugar transferase structures (R459 in 6s7t [65], R405 in 6s7o [65], R404 in 6ezn [74], R426 in 3waj [75, 79], and R375 in 5ogl [60]). In all these known structures, this arginine is described as an interaction partner of the LLC’s phosphate group whereas the lipid part of the LLC is accommodated within a hydrophobic groove formed mainly by TM6 and TM7.

The sequence SHKSYRP (with H89/K90 in TMTC1) in EL1 is well conserved among TMTCs (close to the N-terminal end of second helix in EL1). At the same time, K85 in the 5ezm/5f15 sequence at a homologous position is known to interact with the LLC’s phosphate. Thus, it is reasonable to assume that one of the positively charged residues in TMTCs (e.g., H89 or K90 in TMTC1) has a similar role. This suggestions is supported by the known mutant phenotype in human TMTC3 (the mutation His67Asp introduces a charge swap and leads to cobblestone lissencephaly [19]; H67 is the position in TMTC3 homologous to H89 in TMTC1).

The limits of a purely sequence-analytic approach can be illustrated with the case of the DW motif conserved among all TMTCs in EL4 (e.g., D330/W331 in N-TMTC1) at the C-terminal end of the helix parallel to the ER membrane. It is problematic to identify the function of an equivalent motif in homologous 3D structures, even in those with a hit to DUF1736. For example, the apparently homologous sequence position R270/Y271 in 5ezm/5f15 are at the edge of a structurally unresolved loop region. In 6s7t, residues E405/H406 seem the closest to positions homologous to the TMTCs’ DW motif. E405 is directed towards R214 (a residue in the loop homologous to EL2) [65]. Thus, the function of the conserved DW motif in TMTCs (as well as of several others) cannot be unambiguously understood due to such comparisons. Interestingly, a DW motif has been described as critical for subunit interaction in pyruvate dehydrogenase kinase 2 [80].

Thus, this sequence-analytic comparison of TMTCs with known homologous 3D structures shows that a number of conserved sequence motifs can be understood in the context of ligand binding. TMTCs appear to incorporate divalent metal ions for catalysis and LLCs as donors for a sugar moiety. Given the experimental finding of TMTCs being part of a new O-mannosylation pathway [26], the LLC applicable here is dolichyl-phospho-mannose (DPM), the universal donor of mannosyl-residues in higher eukaryotes.

### TMTCs are homologous to a variety of sequence families of membrane-bound sugar transferases

When applying HHPred with N-TMTCs as input against the Pfam library of sequence domain family models, a large variety of annotated entries besides many domains of unknown function are hit with, beyond doubt, statistically significant E-values (E-value< 1.e-5, see Table 2 and Additional file 3).

**Table 2:** HHPred search with N-terminal part of four human TMTCs against Pfam-A_v33.1

Pfam domain	TMTC1 (1–456)	TMTC2 (1–475)	TMTC3 (1–426)	TMTC4 (1–462)
Glyco_transf_22 (PF03901, 388 AA)	2.1E-20	1E-18	1.5E-20	6.4E-19
	Q: 29–456	Q: 3–475	Q: 9–426	Q: 22–462
	T: 1–352	T: 1–351	T: 2–350	T: 1–350
STT3 (PF02516, 458 AA)	5.5E-19	2.1E-17	1.8E-19	9.5E-18
	Q: 26–456	Q: 1–473	Q: 5–423	Q: 19–459
	T: 3–406	T: 4–400	T: 3–401	T: 3–401
PTPS_related (PF10131, 616 AA)	1.4E-15	9.7E-14	2.5E-16	4.3E-15
	Q: 89–456	Q: 62–475	Q: 67–425	Q: 81–462
	T: 1–308	T: 1–308	T: 1–307	T:1–308
PMT (PF02366, 247 AA)	2.3E-14	1.3E-13	1.5E-14	2.1E-13
	Q: 30–285	Q: 3–248	Q: 9–259	Q: 23–293
	T: 2–242	T: 1–242	T: 2–242	T: 2–242
Mannosyl_trans2 (PIG-V) (PF04188, 432 AA)	6E-14	1.9E-12	3.8E-14	1.4E-12
	Q: 51–451	Q: 25–470	Q: 30–426	Q: 44–462
	T: 60–425	T: 60–425	T: 60–429	T: 60–427
Dpy19 (PF10034, 651 AA)	8.4E-13	1.8E-12	4.4.E-13	3E-12
	Q: 46–455	20–474	27–424	39–460
	30–502	30–503 (651)	32–499 (651)	30–499 (651)
AftA_N (PF12250, 432 AA)	3.6E-12	3.7E-11	3.1E-13	1.6E-11
	T: 27–446	T: 3–465	T: 7–399	T: 20–435
	Q: 76–430	Q: 78–431	Q: 76–402	Q: 75–402
PMT_2 (PF13231, 159 AA)	3.7E-13	1.1E-11	6.3E-13	1.6E-12
	Q: 91–276	Q: 64–234	Q: 69–250	Q: 83–284
	T: 1–156	T: 1–156	T: 1–156	T: 1–159
Arabinose_trans (PF04602, 471 AA)	6.3E-11	5.9E-09	1.1E-10	1.6E-10
	Q: 34–456	Q: 8–468	Q: 13–426	Q: 27–462
	T: 51–428	T: 51–423	T: 51–430	T: 51–427
PIG-U (PF06728, 363 AA)	9.8E-11	7.8E-09	2.1E-10	5E-09
	Q: 47–456	Q: 6–475	Q: 14–423	Q: 45–462
	T: 30–349	T: 1–350	T: 1–345	T: 35–349
Mannosyl_trans4 (PF15971, 163 AA)	9.4E-11	1E-09	6.2E-11	3.9E-10
	Q: 81–276	Q: 59–234	Q: 59–250	Q: 78–285
	T: 1–162	T: 6–161	T: 1–162	T: 6–162
Glucos_trans_II (PF14264, 312 AA)	6.4E-07	2.8E-06	7.6E-08	5.6E-07
	Q: 45–413	Q: 19–431	Q: 24–385	Q: 38–421
	T: 5–310	T: 5–310	T: 5–310	T: 5–310
GT87 (PF09594, 251 AA)	3.5E-07	6.4E-06	1.1E-06	1.5E-06
	Q: 91–389	Q: 64–406	Q: 68–360	Q: 82–396
	T: 2–251	T: 2–249	T: 1–248	T: 1–248

The functionally annotated hits with best E-values are listed: PF03901, Alg9-like mannosyltransferase family; PF02516, Oligosaccharyl transferase STT3 subunit; PF10131, 6-pyruvoyl-tetrahydropterin synthase related domain, function unknown; PF02366, Dolichyl-phosphate-mannose-protein mannosyltransferase; PF04188, Mannosyltransferase (PIG-V); PF10034, Q-cell neuroblast polarisation, function unknown; PF12250, Arabinofuranosyltransferase N terminal domain; PF13231, Dolichyl-phosphate-mannose-protein mannosyltransferase; PF04602, Mycobacterial cell wall arabinan synthesis protein; PF06728, GPI transamidase subunit PIG-U; PF15971, Dolichyl-phosphate-mannose mannosyltransferase; PF14264, Glucosyl transferase Gtr II; PF09594, Glycosyltransferase family 87. For each query and each Pfam entry (listed as Pfam entry name and ID), we provide the E-value and the sequence ranges hit in the query (Q) and in the template (T; we also provide the length of the template in parentheses below the Pfam model name)

Most of the domains found belong to the GT-C clan (CL0111) of glycosyltransferases (out of 19 known GT-C members, nine were detected: Glyco_transf_22, STT3, PTPS_related, PMT, Mannosyl_trans2, PMT_2, Arabinose_trans, PIG-U, GT87). Most informative are the sequence homologies with Glyco_transf_22 (PF03901) and STT3 (PF02516) because the E-value is < 1.e-18 and alignment of the Pfam domains and the N-TMTCs cover both query and template almost completely (coverage > 95%). Certain super-conserved residues in the sequence family alignments of both Pfam families are also conserved among the TMTCs. This includes the active site DD motif in EL1 (e.g., D52/D53 in N-TMTC1) and the arginine in front of TM10 (e.g., R404 in TMTC1) that are characteristic for both Pfam domains.

The homology with other groups of dolichyl-phosphate-mannose-dependent mannosyltransferases (Mannosyl_trans4, PF15971), glucosyl transferases GtrII (Glucos_trans_II, PF14264) and arabinofuranosyltransferase N-terminal domain (AftA_N, PF12250) not directly linked to the GT-C clan fits into the same general functional prediction for TMTCs as sugar transferases and having a similar 3D structure.

The HHPRED search results are confirmed by iterative PSI-BLAST [32] runs with standard parametrization and human TMTC sequences as input. They deliver plentiful hits within the GT-C clan and beyond (results not shown). The diversity of significant homology hits constitutes a problem for function assignment of TMTCs beyond the general prediction as GT-C/PMT-like sugar transferases. It needs to be emphasized that the GT-C clan is a very diverse sequence superfamily comprising membrane-bound sugar transferases with a large variety of different specific activities and substrate types (including the transfer of arabinose, mannose, glucose or oligosaccharides among others).

We find also other proteins including even enzymatically completely inactive ones such as PIG-U (see reference [81] for discussion of PIG-U’s function). Interestingly, the profile build on the basis of our grand alignment of TMTCs is linked by HHPred to the domain BindGPILA [81] with E-value ~ 0.03 (calculated at the background of all Pfam models). To note, this domain model is derived from homologous sequence segments with 10 TMs and intermittent loops extracted from proteins in the glycosylphosphatidylinositol (GPI) lipid anchor pathway PIG-B, PIG-M, PIG-U, PIG-V, PIG-W and PIG-Z [81]. PIG-W is an acetyltransferase for the GPI lipid anchor, PIG-U is not an enzyme at all but the remaining four (PIG-B, PIG-M, PIG-V and PIG-Z) are mannosyltransferases. All of them are united by the ability to bind phospho-lipid linked sugar/carbohydrate moieties.

Thus, the mere homology of TMTCs to the GT-C group of sequences by itself is only informative with regard to fold coincidence, to structural similarity and to a general level of functional classification. Yet, the conservation of residues known to be important for catalysis and substrate binding as detailed in the sequence analysis above indicates that TMTCs are actually enzymatically active. As we see in the 3D structure modelling exercise below, many additional conserved sequence motifs can be rationalized due to interactions with ligands and substrate molecules.

### Insights from the structural modelling of human TMTCs by homology to membrane-bound sugar transferases with known 3D structural arrangements

We attempted to create 3D structural models of all four TMTCs together with a divalent metal ion and DPM with the goal to explore whether observed sequence motifs that are conserved between TMTCs and sugar-transferases of known 3D structure come spatially together for interaction with the ligands.

HHpred scored the aminoarabinose transferase structures ArnTCm (PDB IDs: 5ezm and 5f15, chain A [58]) as by far the best hit for all human TMTCs (see Table 1) and also for five other organisms including *Bos taurus, Gallus gallus, Danio rerio, Xenopus laevis* and *Drosophila melanogaster* (results not shown). Therefore, this X-ray crystal structure was used as a template to build 3D models of TMTC1 (XP_016875493.1), TMTC2 (Q8N394), TMTC3 (Q6ZXV5) and TMTC4 (Q5T4D3) using the functions automodel and loop refine in Modeller (version 9.4) [35]. The overall structure of 5ezm (apo ArnTCm, resolution 2.70 Å) / 5f15 (UndP-bound ArnTCm, resolution 3.20 Å) [58] consists of (i) an N-terminal membrane-embedded region and (ii) a periplasmic domain (PD). For this work, only the first segment is of interest. It involves 13 TM helices and interconnecting loops including three juxtamembrane helices (JM1, JM2 and JM3). JM1 and JM2 form the first periplasmic loop between TM1 and TM2 while JM3 leads into a partially disordered flexible periplasmic loop (PL4 being homologous to EL4 in TMTCs) between TM7 and TM8.

In this study, only the membrane-embedded domain of TMTCs including the juxtamembrane helices were modelled using the most N-terminal regions of the templates 5ezm and 5f15 (the 11 TM segments together with JM1 and JM2 following 5ezm while JM3 was molded after 5f15). The major hurdles to generate the 3D structure of TMTCs by homology modelling are (i) the low percent identity (< 15%) with sequences of the template crystal structures (Table 3) and (ii) several overly long loops between TM regions without equivalent in the structure templates. As we want to understand structural detail at the lumenal side, cytoplasmic loops are not that critical but the lumenal ones are. The loop sequence segments include (i) the cytoplasmic loop between TM2-TM3 (residues 136–146) in TMTC4, (ii) the cytoplasmic loop between TM6-TM7 in all TMTCs and (iii) the lumenal loop TM9-TM10 in all TMTCs. Furthermore, the template 5ezm/5f15 does not account for a loop extension at the N-terminal side of the domain of unknown function, DUF1736 (PF08409), between TM7-TM8 for all TMTCs. Moreover, we note that TMTC2 has another unusually longer cytoplasmic loop between TM8-TM9 (residues 337–392) and, therefore, in the absence of any template, residues 337–392 were not modelled. We describe the alignment with the 5ezm/5f15 template, the regions modelled for each TMTC proteins and issues with the overly long loops in Table 3 and in the annotated alignment in Additional File 4 – Supplementary Figure 1.

**Table 3:** Modelling the 3D structures of TMTCs

	TMTC1	TMTC2	TMTC3	TMTC4
**Sequence identity with template** **(5EZM/5F15)**	9.4%	10.6%	9.5%	11.3%
**Modelled regions**	23–456	1–336 & 393–474	4–428	17–464
**Loop between TM6-TM7**	240–257	207–220	209–231	242–262
**Loop between TM9-TM10**	393–406	411–419	*365–373	401–409
**DUF1736 region** **(JM3)**	284–358 (321–335)	247–321 (284–298)	258–331 (294–308)	292–366 (329–343)

The table provides the sequence identities of template 5EZM/5F15 with TMTCs, the range of the modelled regions, the longer loops between TM6-TM7 and TM8-TM9 compared with the templates, and location of DUF1736 along with JM3 (*residues 365–369 continue to be helical with TM9). TMTC2 has another unusual, longer cytoplasmic loop between TM8-TM9 (residues 337–392) which is not modelled in the absence of any template

As we expect that certain long loops, especially those that have no equivalent in the 5ezm/5f15 structure, will not get reconstructed well, the DOPE model scoring system provided by Modeller might not be such a good choice for selecting among various model instances. We have validated our model instances based on the TM-align scores [82]. A TM-score between 0 and 0.3 suggests random structural similarity while a TM-score greater than 0.5 and less than 1.0 suggests two structures having the same fold. The TM-align scores for TMTC1, TMTC2, TMTC3 and TMTC4 (when compared with 5ezm) are 0.93441, 0.72261, 0.91499, and 0.92104 respectively.

**Fig. 3:**
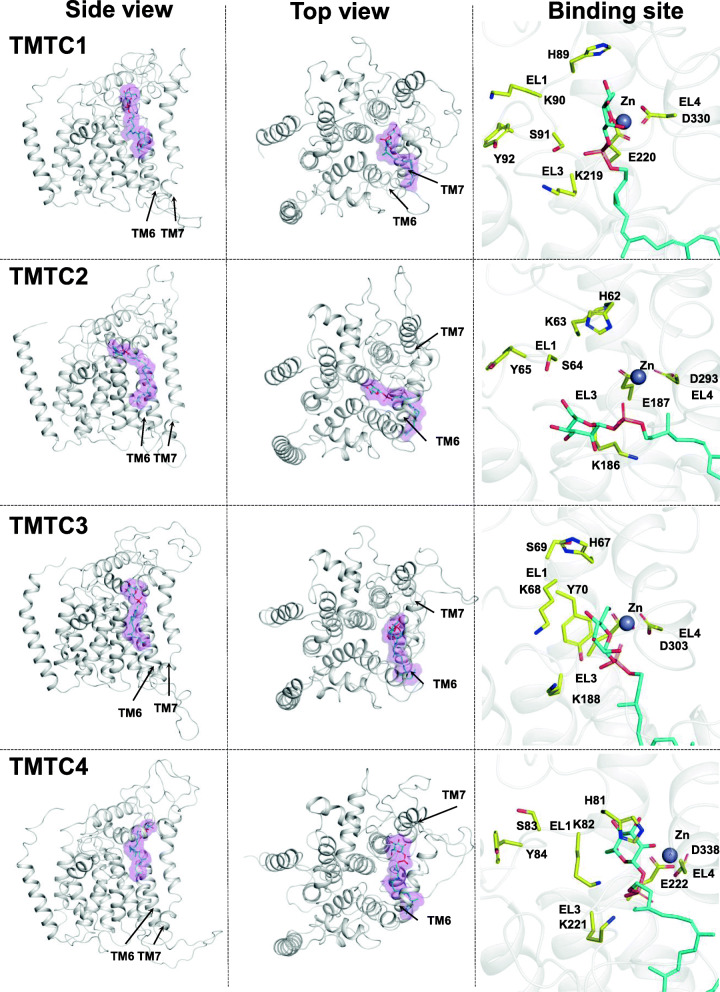
Structure models of TMTC1/2/3/4 with ligands. The cartoon representation of model TMTC1/2/3/4 (from top to bottom) with docked DPM is shown in side- (left column) and top-view (middle column). Close-up (right column) of the binding pocket of TMTCs with docked DPM (cyan color sticks) and with important residues (HKSY residues of the conserved SHKSYRP motif M2 in EL1; K and E from motif M4 in EL3) presented in yellow color sticks; the divalent metal ion (modelled as zinc) is shown in gray color

The resulting 3D structure models (see Fig. 3) were used to place a divalent metal ion (following 5ezm for initial positioning) and a DPM moiety (using crystal-bound ligand UndP in 5f15 for initial posing as reference position). We applied Zn^+ 2^ parametrization for the ion in this study although there is no clarity about the exact nature of the divalent metal ion from experiment. The crystallographic evidence speaks for zinc in 5ezm [58]; yet, Mn^2+^ is the likely ion in the case of 5ogl [60], several other reports such as the one for 6s7t [65] remain silent about the nature of the ion other than emphasizing an electronic density consistent with a divalent metal ion. To emphasize, we do not think that the exact parametrization of the ion (beyond carrying two positive charges) is critical for the outcome of this modelling study.

3D structure modelling operations including ligands were implemented with Schrodinger suite [36]. An induced fit procedure following established protocols [36–42] was applied. In brief, the Schrodinger programs “Protein Preparation Wizard” and “LigPrep” were utilized for preparing the TMTC models and the DPM. With “Glide-SP” and “Prime”, multiple poses of DPM were generated and optimized in multi-step energy minimizations (with the OPLS parameter set and a surface Generalized Born implicit solvent model) that included some stages with softened potentials and side chains mutated to alanine. The procedure was completed with a minimization that allowed all residues within 5 Å of DPM (including their backbone and side-chain) and ligand DPM itself to be relaxed. The complexes were ranked by Prime energy (molecular mechanics energy plus solvation) and those within 30 kcal/mol of the minimum energy structure were passed through for a final round of Glide docking and scoring with GlideScore. The final structures for each of the TMTCs together with the ligands are provided with their atomic coordinates (Additional File 5).

**Fig. 4:**
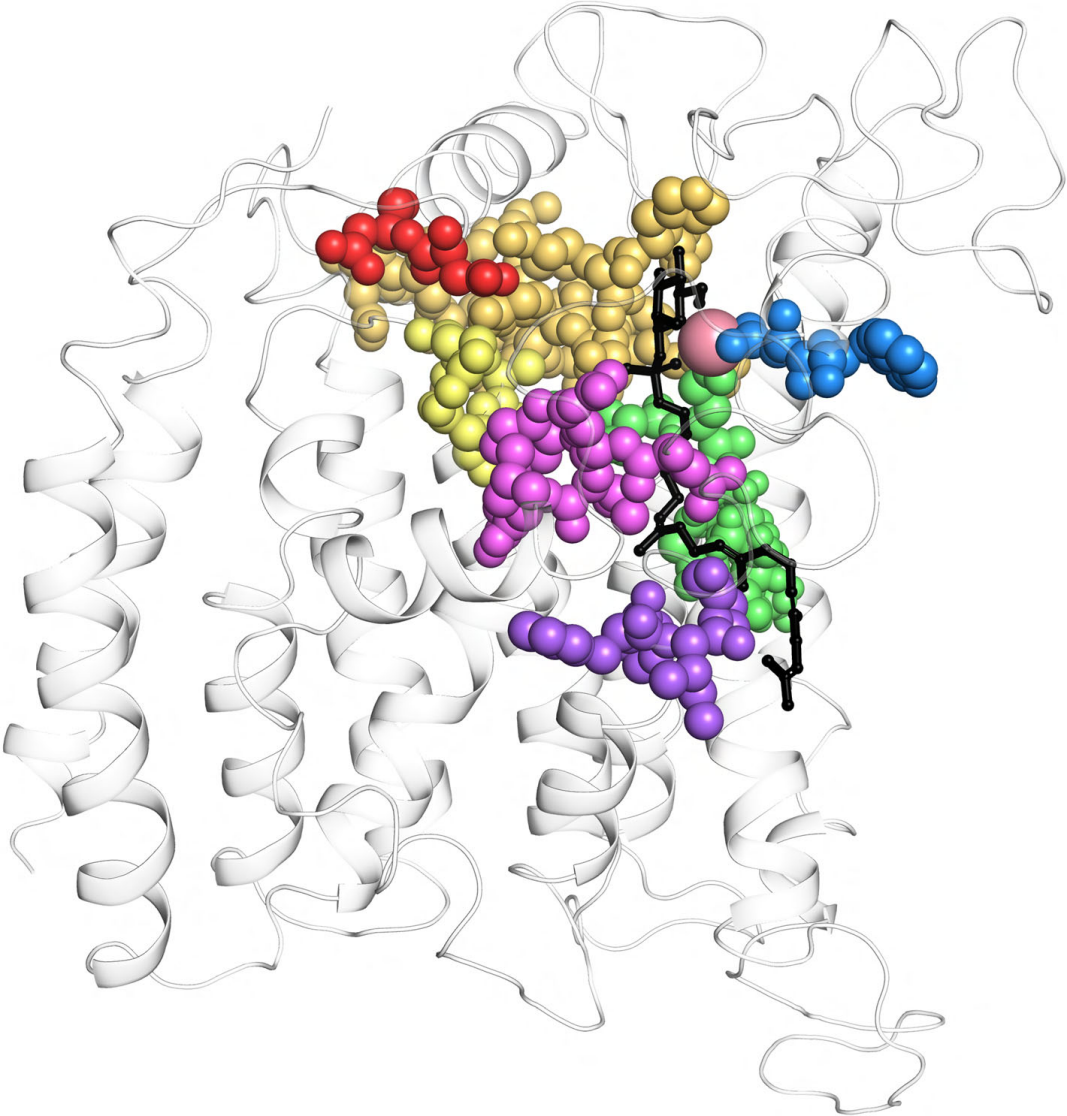
Sequence motifs M1-M7 come spatially together in model structures of TMTCs. We illustrate the spatial localization of sequence motifs M1 (red), M2 (orange), M3 (yellow), M4 (green), M5 (blue), M6 (violet) and M7 (pink, all shown in ball mode) at the background of the structural cartoon of the whole protein. DPM is presented as blackish sticks, the divalent metal ion is represented as reddish sphere. We show the case of TMTC1; the figures for the other TMTCs look very similar. To note, motif M2 in this figure is extended to the conserved region represented by SHKSYRPLCVTLTSFRLN in TMTC1 (88–103 in EL1)

As the most important outcome of the modelling effort, visual inspection of the four model structures show that, for all TMTCs, the resulting structures show consistently that seven conserved sequence motifs M1-M7 as listed in Table 4 come spatially together at the lumenal side of the TMTCs, form part of the surface of the protein structure that is homologous to the two substrate/ligand binding sites in 5ezm/5f15. They group closely around the DPM moiety and the divalent ion creating a dome region (see Fig. 4 for the case of TMTC1). We find that residues in motifs M4 and M5 are observed for coordinating the divalent metal ions. M2 and M3 are largely engaged in mannose interactions, M6 tends to contact with the dolichyl tail. Motifs M4, M5 and M7 are important for interaction with the phosphate in DPM. Thus, the observed sequence conservation can be rationalized in terms of evolutionary conserved function.

**Table 4:** Several conserved sequence motifs in TMTCs are related to DPM binding and divalent metal ion coordination

Motif	Residues	TMTC1	TMTC2	TMTC3	TMTC4
M1 (red) DD in EL1	D	52	26	31	45
	D	53	27	32	46
M2 (orange) SHKSYRP in EL1 mannose	S	88_A_	61_C_	66_B_	80_A_
	H	89_A_	62_B_	67_A_	81_A_
	K	90_B_	63_C_	68_A_	82_A_
	S	91_A_	64_A_	69_B_	83_A_
	Y	92_C_	65	70_B_	84_B_
	R	93_C_	66	71	85_A_
	P	94	67	72	86_A_
M3 (yellow) RxD in EL2	R	167	139_A_	143	172_C_
	D	169	141_A_	145_B_	174
M4 (green) KE(T/Q) xxT in EL3	K	219_A_	186_A_	188_A_	221_A_
	**E**	**220**_A_	**187**_A_	**189**_A_	**222**_A_
	T/Q	221(T)_A_	188(Q)_A_	190(Q)_A_	223(Q)_A_
	T	224_A_	191_B_	193_B_	226_C_
M5 (blue) DW in EL4	**D**	**330**_A_	**293**_A_	**303**_A_	**338**_A_
	W	331	294_A_	304_A_	339_A_
M6 (violet) PxxP in TM9	P	386_A_	404_C_	358_A_	394_C_
	P	389_B_	407_A_	361_A_	397_B_
M7 (pink) ERxxY in EL5	E	403_A_	421_A_	375_A_	411
	R	404_C_	422_A_	376_C_	412
	Y	407_C_	425_C_	379	415_B_

Conserved residues present in the vicinity of the ligand dolichyl-phosphate-mannose (DPM) are part of seven motifs M1-M7 in the TMTC family protein sequences. For each motif, the actual sequence, the location (loop number or TM number), loop coloring in Fig. 4 and the residue numbers in TMTC1/2/3/4 respectively are listed. If at least one atom of the residue is within 5 Å, 6 Å or 7 Å of any atom of DPM, the respective residue is marked with the corresponding subscript “A”, “B” or “C”. In bold, we indicate residues in M4 and M5 observed for coordinating the divalent metal ions. We find motifs M2 and M3 largely involved in mannose interactions, M6 provides for the dolichyl tail, and M4, M5 and M7 are important for interaction with the phosphate

Further, several close contacts between the DPM ligand, the metal ion and TMTC residues were observed (to note, we did not enforce any specific residue contacts during the induced fit docking procedure). Given some sequence diversity among TMTCs and also the large number of degrees of freedom in the modelling process, it is not surprising that not all contacts are found in all models. Yet, a common subset of those was detected in each of the TMTC1, TMTC2, TMTC3, and TMTC4 model structures (see Table 4) and some contacts repeat patterns seen in homologous crystal structures:

(i) The phosphate functional group of DPM interacts with the divalent metal ion. In addition, the metal binds to the glutamate residue in the conserved KET(Q) xxT motif in EL3 (e.g., E220 of TMTC1) and to aspartate residue of the conserved DW motif (e.g., D330 in TMTC1) in EL4. To note, H267 (in the motif H265-E266-H267 where the glutamate is homologous to D330 in TMTC1) interacts with the divalent metal ion held between JM1 and EL4 in 5ezm [58].
(ii) The phosphate group of DPM also forms a salt bridge with the lysine residue of the conserved KET(Q) xxT motif in EL3 (e.g., K219 in TMTC1).
(iii) The mannose moiety interacts with residues H-K-S-Y within the conserved SHKSYRP motif M2 in EL1 (e.g., S80, H89, K90 and S91 residues in TMTC1, Fig. 3).
(iv) The conserved stretch in EL1 represented by SHKSYRPLCVLTSFRLN in TMTC1 (it includes motif M2) forms the dome region of the DPM binding pocket in all 4 TMTCs. The dolichyl lipid chain of DPM occupies the cavity that is provided by hydrophobic residues of TM6, TM7 and TM9.

The structural models of human TMTCs can only be considered preliminary in many details at this stage since

- important ingredients such as the protein substrate and possibly important interacting partners are missing,
- sequence identity with the target structure is low (~ 10% in the manually edited alignments used for modelling, Table 3),
- there are loop extensions not found in the structural template, and
- the TMTCs are modelled without the C-terminal TPR domain.

The average accuracy of C-alpha atom positioning in homology modelling above 30% sequence identity is estimated 2 Å [83, 84]; hence, the error is expected to be higher for certain regions in our model structures, especially in loop regions without equivalent in the template. On the other hand, the known crystal structures (having very moderate crystallographic resolutions around 3 Å) do not resemble the complete protein complex including the correctness of certain groups of amino acid chains, some inter-TM loops, substrates and ligands needed for catalysis either.

Despite these restrictions, we see consistent features emerging from the modelling of various TMTCs, namely the arrangement of TM regions in the membrane as well as of the loops and segments that form the binding site for the lipid-linked sugar and the divalent metal ion; essentially, the major part of the structure located in the ER lumen appears functionally plausible after the conserved sequence segments got spatially united as a result of the 3D reconstruction.

Thus, it makes sense to analyze also contacts between the DPM moiety, the metal ion and TMTC residues seen only in a few of the TMTC models. In this way, we will get a more complete picture of the binding cavity and can enlarge the list of potentially relevant residues for interaction with the ligands:

(i) We found the aspartate from motif M3 in the vicinity of the mannose in TMTC2 (D141) and TMTC3 (E145). The homologous residue D158 in 5f15 [58] is also seen to interact with the arabinose moiety.
(ii) K203 in 5f15 [58] forms a salt bridge to the arabinose moiety. A similar close contact to the sugar is seen by homologous lysine residues in motif M4 for TMTC2 (K186), TMTC3 (K188) and TMTC4 (K221).
(iii) The motif M7 arginine in TMTC2 (R422) forms a hydrogen bond with the phosphate. This interaction resembles the contact between several homologous arginine residues (R459 in 6s7t [65], R405 in 6s7o [65], R404 in 6ezn [74], R426 in 3waj [75, 79], and R375 in 5ogl [60]) and the phosphates from the respective LLCs in those X-ray 3D structures. Similarly, the M7 tyrosine is observed close to the phosphate in TMTC2 (Y425) and TMTC4 (Y415) as Y345 in 5f15 [58].
(iv) Residues E84/K85 in 5ezm [58] do interact with the metal ion in the absence of a LLC molecule. We see the homologous residues HK in motif M2 also interacting with a ligand (but with the sugar moiety) in our TMTC models.

## Discussion

Despite the wealth of sequence-analytic findings available for TMTCs, the systematic analysis of their sequences and of related biomolecular data for the purpose of assigning the biological function of TMTCs has never been performed before. Several roadblocks had to be overcome. First, there are issues with sequence accuracy as, for some TMTCs, several versions of protein sequences are available in databases, some of which lack sequence pieces essential for TMTC function as this study has revealed. Second, the complex nature [66] of the TM regions sprinkled with polar residues/prolines/glycines makes their accurate prediction in the TMTC sequences difficult. This seriously hampers function discovery since localizing certain loops at the correct side of the membrane might be impossible with errors in membrane topology. Third, just the fact of finding sequence similarity with a large number of sugar transferases is helpful to establish the homology relationship but provides little guidance for biological follow-up work aimed at zooming into the exact molecular and cellular functions of TMTCs, for example with regard to actual catalytic capacity, substrate specificity and ligands bound.

This work has made significant steps forward in understanding 3D structure and biological function of the membrane-embedded domains covering the N-terminal halves of TMTC1, TMTC2, TMTC3 and TMTC4 sequences. First, we determined the exact membrane topology using sequence-analytic, phylogenetic and available experimental data. The assumption of conserved membrane topology for evolutionarily conserved molecular function was key to interpret TM prediction results for N-TMTCs in a unified manner. The finally determined membrane topology including 11 TMs nicely complies with all known constraints. The C-terminal globular TPR domain is located in the ER lumen together with the critical for function conserved sequence motifs in the loops between TM regions. The homologous sequence segments in the known 3D structures 5ezm/5f15 corresponding with the luminal loops in TMTCs have the same membrane topology. We can further conclude that TMTC sequences in the database that cannot fit to this topology are most likely erroneous.

Whereas the complex nature of TM regions in TMTCs makes TM prediction difficult, it supports establishing gene homology via searches for significant sequence similarity [66, 70]. The evidence certifying the homology of N-TMTCs with GT-C/PMT-class and other related sugar transferases is overwhelming; thus, TMTCs must have the same overall fold and resemble similar tertiary structure. Despite the huge evolutionary distance from bacteria to human representatives in this homology group, higher eukaryote TMTCs share strongly conserved sequence motifs with GT-C/PMT-class enzyme sequences. Even at the pure sequence-analytic level, we can explain a few of these conserved sites as required for catalysis or for ligand binding. Given the close relationship with ArnT from *Cupriavidus metallidurans* (the structure of which is known: 5ezm/5f15), we suggest that these ligands include a divalent metal ion and a LLC molecule. Since TMTCs are part of an O-mannosylation pathway, we conclude that this LLC is DPM.

3D-structural modelling of N-TMTCs further enhances the association of conserved sequence motifs with ligand binding. Seven conserved sequence motifs from various parts of the protein sequence (including those seen already at the level of just sequence comparison) come spatially together to form the surface of binding sites for the mannosyl residue, the phosphate group and the dolichyl tail of DPM as well as the divalent metal ion; thus, their evolutionary conservation can be rationalized as maintaining the ability to position these two ligands for catalysis. Notably, this spatial co-localization of peptide stretches corresponding to the conserved motifs is sufficiently macroscopic to be a reliable result not affected by the accuracy of the homology procedure applied here.

In addition, we derive, as a result of this homology-supported structural modelling, a further expanded list of residues taken from the set of conserved motifs that are potentially interacting with the divalent metal ion and the DPM ligand. This list comprises those critical residues previously found with combined phylogenetic arguments (sequence conservation among TMTCs and similarity with sequences of structurally and functionally characterized sugar transferases) as a subset. Thus, we can relate certain residues strictly conserved among the TMTC sequences with functions in catalysis and ligand binding. This work also clarified the nature of the DUF1736 sequence segment in TMTCs, actually a loop between TM7 and TM8 the accurate positioning of several of its functional residues is critical for catalysis and binding of ligands, especially the lipid-linked sugar moiety.

Notably, we have already established the homology of TMTCs with GT-C/PMT-class sugar transferases when we first analysed their sequences for the first time in 2012; yet, a substrate and biological context assignment as well as 3D structural modelling were not possible. With HHpred [33], significant sequence similarity with DPM-dependent mannosyltransferases (PMTs, PF02366) was detected. With RPS-BLAST [85, 86], we found the link to ArnT-like arabinose transferases (COG1807). Their respective 3D structures were not known during that time [58].

The density of hints derived from sequence analysis, phylogenetic comparisons, homology studies and structural modelling leaves no doubt that the TMTCs have enzymatic activity and perform sugar moiety transferase functions in their biological context. Thus, the O-mannosyl-transferase sought in the recently discovered new O-mannosylation pathway (via combinations of TMTC knock-outs) that selectively processes cadherin-like targets and that the TMTCs are members of [26], are actually the TMTCs.

Finding the real substrates of the various human TMTCs and rationalising the function of their glycosylation are important questions from the view-point of biological science. Additionally, this topic has a critical medical dimension as several mutations of TMTCs are compatible with survival but severely disable the affected patients in various ways due to the pleiotropic nature of their molecular and cellular functions. Laudably, first steps in this direction have been done. It can be concluded that various cadherins/proto-cadherins found as substrates for the new O-mannosylation pathway are protein substrates for O-mannosylation by TMTCs [25, 26].

BLAST/PSIBLAST [32] searches reveal TMTC proteins are present in a wide range of animals but apparently not in fungi and plants (details not shown). Interestingly, essentially full-length homologous sequences (including the sugar transferase followed by TPR segments) are also found in many, typically not yet well characterized prokaryotes besides hits in lower eukaryotes such as oomycetes and choanoflagellates. One example is protein AMJ42_05695 (from Deltaproteobacteria bacterium DG_8) that is found by a BLAST search with human TMTC3 (24% sequence identity, E-value=3.e-47, alignment of query positions 12–698 against positions 46–774 from target). Human curiosity will not be satisfied until the diversity of their organic chemistry, the related biomolecular mechanisms and the cellular phenotypes will be understood.

## Supplementary Information

**Additional file 1.** The grand alignment of TMTCs. The compressed library file AF1-2020-10-grand-aln-TMTCs.zip provides the alignment shown in Fig. 1 in the “.aln” and “.jvp” formats.

**Additional file 2.** Positions of TM regions and the DUF1736 segment in TMTC sequences. The file AF2-2020-06-TMs-TMTCs.xlsx provides the sequence positions of 11 TM regions (maximum consensus region from 5 TM predictors DAS-tmfilter [43, 44], HMMTOP [45, 46], PHOBIUS [47, 48], TMHMM [49, 50] and TOPPRED2 [51, 52]) in various animal TMTC sequences (the same sequences used in the alignment of Fig. 1) as well as the sequence segments that correspond to the DUF1736 region. We also indicate the approximate core of the hydrophobic region in EL4/DUF1736 that gives rise to false-positive TM predictions but actually represents a helix in the ER lumen parallel to the ER membrane (column “helical hydrophobic region”) if the TM predictors detect any. To emphasize, ‘exact boundaries’ of TM regions are difficult to derive with sequence-analytic methods; a flexibility of up to a few residues on either side should be considered as only the hydrophobic core of the TM is accurately predicted.

**Additional file 3.** HHPred outputs when searching TMTCs against Pfam or PDB structures. The compressed library file AF3-2020-06-HHPred-TMTCs.zip contains the outputs when running the four human TMTC sequences as input of HHPred against PDB sequences and against Pfam domains (as of 23rd of June 2020).

**Additional file 4 **: **Supplementary Figure 1**. Alignment of TMTC sequences with those of the template structures 5ezm and 5f15 used for homology modelling. The file AF4-2020-10-modeller-alignment-TMTCs.pdf shows the alignment of the four human TMTC sequences with template structures 5ezm and 5f15 that was actually used for generating their 3D model with the Modeller suite version 9.4. The sequence IDs of the human TMTCs used for this alignment are TMTC1 (XP_016875493.1), TMTC2 (Q8N394), TMTC3 (Q6ZXV5) and TMTC4 (Q5T4D3). For TMTC2, residues 337–392 are not shown in the alignment. The figure was generated with JalView (version 2.10.5 [61];) using the Clustal coloring scheme. Longer loops in TMTCs compared to template structures between TM2-TM3 in TMTC4, TM6-TM7 and TM9-TM10 in all TMTCs are boxed in blue color. The unstructured loop region hit by N-terminal part of the Pfam domain DUF1736 is boxed in black while the JM3 within the DUF1736 is boxed in magenta color. The sequence corresponding residues 337–392 in TMTC2 (between TM8-TM9) is not modelled due to absence of template region and is represented as a sequence break using the sign “/”, circled in red.

**Additional file 5.** Atomic coordinates of 3D structural models of all four TMTCs with ligands (divalent metal ion and dolichyl-phosphate-mannose. The file AF5-2020-10-3Dmodel-TMTCs.zip provides the atomic coordinates for the 3D structural models of the four human TMTCs generated with template structures 5ezm and 5f15.

## Data Availability

All supporting data are submitted in Supplementary materials (Additional Files 1, 2, 3, 4 and 5).

## Abbreviations

AA: Amino acid(s)
DPM: Dolichyl-phosphate-mannose
ER: Endoplasmic reticulum
GPI: Glycosylphosphatidylinositol
PDB: Protein Data Bank (https://www.rcsb.org/)
TM: Transmembrane (region)
TMTC: Transmembrane and tetratricopeptide repeat-containing
TPR: Tetratricopeptide repeat

## References

[1] Nakano M, Ikeda Y, Taniguchi T, Yagi T, Fuwa M, Omi N, Tokuda Y, Tanaka M, Yoshii K, Kageyama M (2009). Three susceptible loci associated with primary open-angle glaucoma identified by genome-wide association study in a Japanese population. Proc Natl Acad Sci U S A.

[2] Cao D, Jiao X, Liu X, Hennis A, Leske MC, Nemesure B, Hejtmancik JF (2012). CDKN2B polymorphism is associated with primary open-angle glaucoma (POAG) in the Afro-Caribbean population of Barbados, West Indies. PLoS One.

[3] Chen LJ, Tam PO, Leung DY, Fan AH, Zhang M, Tham CC, Chiang SW, Fan BJ, Wang N, Pang CP (2012). SNP rs1533428 at 2p16.3 as a marker for late-onset primary open-angle glaucoma. Mol Vis.

[4] Mabuchi F, Mabuchi N, Takamoto M, Sakurada Y, Yoneyama S, Kashiwagi K, Iijima H, Yamagata Z, Aihara M, Iwata T (2017). Genetic Variant Near PLXDC2 Influences the Risk of Primary Open-angle Glaucoma by Increasing Intraocular Pressure in the Japanese Population. J Glaucoma.

[5] Kim K, Heo DW, Kim S, Kim JS, Kim CS, Kang C (2014). Expansive marker analysis replicating the association of glaucoma susceptibility with human chromosome loci 1q43 and 10p12.31. Eur J Hum Genet.

[6] Kondkar AA, Azad TA, Almobarak FA, Bu-Amero KK, Al-Obeidan SA (2019). Polymorphism rs7961953 in TMTC2 gene is not associated with primary open-angle glaucoma in a Saudi cohort. Ophthalmic Genet.

[7] Rao KN, Kaur I, Chakrabarti S (2009). Lack of association of three primary open-angle glaucoma-susceptible loci with primary glaucomas in an Indian population. Proc Natl Acad Sci U S A.

[8] Choquet H, Paylakhi S, Kneeland SC, Thai KK, Hoffmann TJ, Yin J, Kvale MN, Banda Y, Tolman NG, Williams PA (2018). A multiethnic genome-wide association study of primary open-angle glaucoma identifies novel risk loci. Nat Commun.

[9] Springelkamp H, Mishra A, Hysi PG, Gharahkhani P, Hohn R, Khor CC, Cooke Bailey JN, Luo X, Ramdas WD, Vithana E (2015). Meta-analysis of Genome-Wide Association Studies Identifies Novel Loci Associated With Optic Disc Morphology. Genet Epidemiol.

[10] Mealer RG, Williams SE, Daly MJ, Scolnick EM, Cummings RD, Smoller JW (2020). Glycobiology and schizophrenia: a biological hypothesis emerging from genomic research. Mol Psychiatry..

[11] Verma A, Somvanshi P, Haque S, Rathi B, Sharda S (2019). Association of inflammatory bowel disease with arthritis: evidence from in silico gene expression patterns and network topological analysis. Interdiscip Sci.

[12] Shen X, Liu Z, Cao X, He H, Han S, Chen Y, Cui C, Zhao J, Li D, Wang Y (2019). Circular RNA profiling identified an abundant circular RNA circTMTC1 that inhibits chicken skeletal muscle satellite cell differentiation by sponging miR-128-3p. Int J Biol Sci.

[13] Guillen-Ahlers H, Erbe CB, Chevalier FD, Montoya MJ, Zimmerman KD, Langefeld CD, Olivier M, Runge CL (2018). TMTC2 variant associated with sensorineural hearing loss and auditory neuropathy spectrum disorder in a family dyad. Mol Genet Genomic Med..

[14] Runge CL, Indap A, Zhou Y, Kent JW, King E, Erbe CB, Cole R, Littrell J, Merath K, James R (2016). Association of TMTC2 With Human Nonsyndromic Sensorineural Hearing Loss. JAMA Otolaryngol Head Neck Surg.

[15] Liu X, Chen J, Guan T, Yao H, Zhang W, Guan Z (2019). Wang Y: miRNAs and target genes in the blood as biomarkers for the early diagnosis of Parkinson's disease. BMC Syst Biol.

[16] La-Morte D, Beecham A, Rundek T, Wang L, MS MC, Slifer S, Blanton SH, Di Tullio MR, Sacco RL (2011). A follow-up study for left ventricular mass on chromosome 12p11 identifies potential candidate genes. BMC Med Genet.

[17] Chiang KM, Chang HC, Yang HC, Chen CH, Chen HH, Lee WJ, Pan WH (2019). Genome-wide association study of morbid obesity in Han Chinese. BMC Genet.

[18] Marenholz I, Esparza-Gordillo J, Ruschendorf F, Bauerfeind A, Strachan DP, Spycher BD, Baurecht H, Margaritte-Jeannin P, Saaf A, Kerkhof M (2015). Meta-analysis identifies seven susceptibility loci involved in the atopic march. Nat Commun.

[19] Jerber J, Zaki MS, Al-Aama JY, Rosti RO, Ben-Omran T, Dikoglu E, Silhavy JL, Caglar C, Musaev D, Albrecht B (2016). Biallelic Mutations in TMTC3, Encoding a Transmembrane and TPR-Containing Protein, Lead to Cobblestone Lissencephaly. Am J Hum Genet.

[20] Farhan SMK, Nixon KCJ, Everest M, Edwards TN, Long S, Segal D, Knip MJ, Arts HH, Chakrabarti R, Wang J (2017). Identification of a novel synaptic protein, TMTC3, involved in periventricular nodular heterotopia with intellectual disability and epilepsy. Hum Mol Genet.

[21] Li J, Akil O, Rouse SL, McLaughlin CW, Matthews IR, Lustig LR, Chan DK, Sherr EH (2018). Deletion of Tmtc4 activates the unfolded protein response and causes postnatal hearing loss. J Clin Invest.

[22] Ma M, Huang DG, Liang X, Zhang L, Cheng S, Cheng B, Qi X, Li P, Du Y, Liu L (2019). Integrating transcriptome-wide association study and mRNA expression profiling identifies novel genes associated with bone mineral density. Osteoporos Int.

[23] Sunryd JC, Cheon B, Graham JB, Giorda KM, Fissore RA, Hebert DN (2014). TMTC1 and TMTC2 are novel endoplasmic reticulum tetratricopeptide repeat-containing adapter proteins involved in calcium homeostasis. J Biol Chem.

[24] Racape M, Duong Van Huyen JP, Danger R, Giral M, Bleicher F, Foucher Y, Pallier A, Pilet P, Tafelmeyer P, Shton-Chess J (2011). The involvement of SMILE/TMTC3 in endoplasmic reticulum stress response. PLoS One.

[25] Graham JB, Sunryd JC, Mathavan K, Weir E, Larsen ISB, Halim A, Clausen H, Cousin H, Alfandari D, Hebert DN (2020). Endoplasmic reticulum transmembrane protein TMTC3 contributes to O-mannosylation of E-cadherin, cellular adherence, and embryonic gastrulation. Mol Biol Cell.

[26] Larsen ISB, Narimatsu Y, Joshi HJ, Siukstaite L, Harrison OJ, Brasch J, Goodman KM, Hansen L, Shapiro L, Honig B (2017). Discovery of an O-mannosylation pathway selectively serving cadherins and protocadherins. Proc Natl Acad Sci U S A.

[27] Larsen ISB, Narimatsu Y, Joshi HJ, Yang Z, Harrison OJ, Brasch J, Shapiro L, Honig B, Vakhrushev SY, Clausen H (2017). Mammalian O-mannosylation of cadherins and plexins is independent of protein O-mannosyltransferases 1 and 2. J Biol Chem.

[28] Larsen ISB, Narimatsu Y, Clausen H, Joshi HJ, Halim A (2019). Multiple distinct O-Mannosylation pathways in eukaryotes. Curr Opin Struct Biol.

[29] Eisenhaber B, Kuchibhatla D, Sherman W, Sirota FL, Berezovsky IN, Wong WC, Eisenhaber F (2016). The Recipe for Protein Sequence-Based Function Prediction and Its Implementation in the ANNOTATOR Software Environment. Methods Mol Biol.

[30] Schneider G, Wildpaner M, Sirota FL, Maurer-Stroh S, Eisenhaber B, Eisenhaber F (2010). Integrated tools for biomolecular sequence-based function prediction as exemplified by the ANNOTATOR software environment. Methods Mol Biol.

[31] Johnson M, Zaretskaya I, Raytselis Y, Merezhuk Y, McGinnis S, Madden TL (2008). NCBI BLAST: a better web interface. Nucleic Acids Res.

[32] Altschul SF, Madden TL, Schaffer AA, Zhang J, Zhang Z, Miller W, Lipman DJ (1997). Gapped BLAST and PSI-BLAST: a new generation of protein database search programs. Nucleic Acids Res.

[33] Soding J, Biegert A, Lupas AN (2005). The HHpred interactive server for protein homology detection and structure prediction. Nucleic Acids Res.

[34] Hildebrand A, Remmert M, Biegert A, Soding J (2009). Fast and accurate automatic structure prediction with HHpred. Proteins.

[35] Fiser A, Sali A (2003). Modeller: generation and refinement of homology-based protein structure models. Methods Enzymol.

[36] Schrodinger LLC Schrodinger Release 2020–3: Glide, LigPrep, Prime, Protein Preparation Wizard [https://www.schrodinger.com/]. Accessed 1 Aug 2020.

[37] Sherman W, Day T, Jacobson MP, Friesner RA, Farid R (2006). Novel procedure for modeling ligand/receptor induced fit effects. J Med Chem.

[38] Sherman W, Beard HS, Farid R (2006). Use of an induced fit receptor structure in virtual screening. Chem Biol Drug Des.

[39] Repasky MP, Shelley M, Friesner RA. Flexible ligand docking with Glide. Curr Protoc Bioinformatics. 2007;18(1):8.12.1-8.12.36. 10.1002/0471250953.bi0812s18. 10.1002/0471250953.bi0812s18 18428795

[40] Repasky MP, Murphy RB, Banks JL, Greenwood JR, Tubert-Brohman I, Bhat S, Friesner RA (2012). Docking performance of the glide program as evaluated on the Astex and DUD datasets: a complete set of glide SP results and selected results for a new scoring function integrating WaterMap and glide. J Comput Aided Mol Des.

[41] Friesner RA, Murphy RB, Repasky MP, Frye LL, Greenwood JR, Halgren TA, Sanschagrin PC, Mainz DT (2006). Extra precision glide: docking and scoring incorporating a model of hydrophobic enclosure for protein-ligand complexes. J Med Chem.

[42] Friesner RA, Banks JL, Murphy RB, Halgren TA, Klicic JJ, Mainz DT, Repasky MP, Knoll EH, Shelley M, Perry JK (2004). Glide: a new approach for rapid, accurate docking and scoring. 1. Method and assessment of docking accuracy. J Med Chem.

[43] Cserzo M, Eisenhaber F, Eisenhaber B, Simon I (2002). On filtering false positive transmembrane protein predictions. Protein Eng.

[44] Cserzo M, Eisenhaber F, Eisenhaber B, Simon I (2004). TM or not TM: transmembrane protein prediction with low false positive rate using DAS-TMfilter. Bioinformatics.

[45] Tusnady GE, Simon I (2001). The HMMTOP transmembrane topology prediction server. Bioinformatics.

[46] Tusnady GE, Simon I (1998). Principles governing amino acid composition of integral membrane proteins: application to topology prediction. J Mol Biol.

[47] Kall L, Krogh A, Sonnhammer EL (2004). A combined transmembrane topology and signal peptide prediction method. J Mol Biol.

[48] Kall L, Krogh A, Sonnhammer EL (2007). Advantages of combined transmembrane topology and signal peptide prediction--the Phobius web server. Nucleic Acids Res.

[49] Sonnhammer EL, Von Heijne G, Krogh A (1998). A hidden Markov model for predicting transmembrane helices in protein sequences. Proc Int Conf Intell Syst Mol Biol.

[50] Krogh A, Larsson B, Von Heijne G, Sonnhammer EL (2001). Predicting transmembrane protein topology with a hidden Markov model: application to complete genomes. J Mol Biol.

[51] Von Heijne G (1992). Membrane protein structure prediction. Hydrophobicity analysis and the positive-inside rule. J Mol Biol.

[52] Claros MG, Heijne GV (1994). TopPred II: an improved software for membrane protein structure predictions. Comput Appl Biosci.

[53] El-Gebali S, Mistry J, Bateman A, Eddy SR, Luciani A, Potter SC, Qureshi M, Richardson LJ, Salazar GA, Smart A (2019). The Pfam protein families database in 2019. Nucleic Acids Res.

[54] Letunic I, Doerks T, Bork P (2012). SMART 7: recent updates to the protein domain annotation resource. Nucleic Acids Res.

[55] Andrade MA, Ponting CP, Gibson TJ, Bork P (2000). Homology-based method for identification of protein repeats using statistical significance estimates. J Mol Biol.

[56] Eddy SR (1998). Profile hidden Markov models. Bioinformatics.

[57] Eddy SR (2011). Accelerated Profile HMM Searches. PLoS Comput Biol.

[58] Petrou VI, Herrera CM, Schultz KM, Clarke OB, Vendome J, Tomasek D, Banerjee S, Rajashankar KR, Belcher DM, Kloss B (2016). Structures of aminoarabinose transferase ArnT suggest a molecular basis for lipid A glycosylation. Science.

[59] Bai L, Kovach A, You Q, Kenny A, Li H (2019). Structure of the eukaryotic protein O-mannosyltransferase Pmt1-Pmt2 complex. Nat Struct Mol Biol.

[60] Napiorkowska M, Boilevin J, Sovdat T, Darbre T, Reymond JL, Aebi M, Locher KP (2017). Molecular basis of lipid-linked oligosaccharide recognition and processing by bacterial oligosaccharyltransferase. Nat Struct Mol Biol.

[61] Waterhouse AM, Procter JB, Martin DM, Clamp M, Barton GJ (2009). Jalview Version 2--a multiple sequence alignment editor and analysis workbench. Bioinformatics.

[62] Galtier N, Gouy M, Gautier C (1996). SEAVIEW and PHYLO_WIN: two graphic tools for sequence alignment and molecular phylogeny. Comput Appl Biosci.

[63] Gouy M, Guindon S, Gascuel O (2010). SeaView version 4: A multiplatform graphical user interface for sequence alignment and phylogenetic tree building. Mol Biol Evol.

[64] Magro Armenteros JJ, Tsirigos KD, Sonderby CK, Petersen TN, Winther O, Brunak S, von HG NH (2019). SignalP 5.0 improves signal peptide predictions using deep neural networks. Nat Biotechnol.

[65] Ramirez AS, Kowal J, Locher KP (2019). Cryo-electron microscopy structures of human oligosaccharyltransferase complexes OST-A and OST-B. Science.

[66] Wong WC, Maurer-Stroh S, Eisenhaber F (2011). Not all transmembrane helices are born equal: Towards the extension of the sequence homology concept to membrane proteins. Biol Direct.

[67] Wong WC, Maurer-Stroh S, Schneider G, Eisenhaber F (2012). Transmembrane helix: simple or complex. Nucleic Acids Res.

[68] Baker JA, Wong WC, Eisenhaber B, Warwicker J, Eisenhaber F (2017). Charged residues next to transmembrane regions revisited: “Positive-inside rule” is complemented by the “negative inside depletion/outside enrichment rule”. BMC Biol.

[69] Baker JA, Wong WC, Eisenhaber B, Warwicker J, Eisenhaber F (2017). Erratum to: Charged residues next to transmembrane regions revisited: “Positive-inside rule” is complemented by the “negative inside depletion/outside enrichment rule”. BMC Biol.

[70] Wong WC, Maurer-Stroh S, Eisenhaber F (2010). More than 1,001 problems with protein domain databases: transmembrane regions, signal peptides and the issue of sequence homology. PLoS Comput Biol.

[71] Wong WC, Maurer-Stroh S, Eisenhaber B, Eisenhaber F (2014). On the necessity of dissecting sequence similarity scores into segment-specific contributions for inferring protein homology, function prediction and annotation. BMC Bioinformatics.

[72] Wong WC, Yap CK, Eisenhaber B, Eisenhaber F (2015). dissectHMMER: a HMMER-based score dissection framework that statistically evaluates fold-critical sequence segments for domain fold similarity. Biol Direct.

[73] Tusnady GE, Simon I (2010). Topology prediction of helical transmembrane proteins: how far have we reached?. Curr Protein Pept Sci.

[74] Wild R, Kowal J, Eyring J, Ngwa EM, Aebi M, Locher KP (2018). Structure of the yeast oligosaccharyltransferase complex gives insight into eukaryotic N-glycosylation. Science.

[75] Matsumoto S, Shimada A, Nyirenda J, Igura M, Kawano Y, Kohda D (2013). Crystal structures of an archaeal oligosaccharyltransferase provide insights into the catalytic cycle of N-linked protein glycosylation. Proc Natl Acad Sci U S A.

[76] Zhang L, Zhao Y, Gao Y, Wu L, Gao R, Zhang Q, Wang Y, Wu C, Wu F, Gurcha SS (2020). Structures of cell wall arabinosyltransferases with the anti-tuberculosis drug ethambutol. Science.

[77] Bloch JS, Pesciullesi G, Boilevin J, Nosol K, Irobalieva RN, Darbre T, Aebi M, Kossiakoff AA, Reymond JL, Locher KP (2020). Structure and mechanism of the ER-based glucosyltransferase ALG6. Nature.

[78] Lommel M, Schott A, Jank T, Hofmann V, Strahl S (2011). A conserved acidic motif is crucial for enzymatic activity of protein O-mannosyltransferases. J Biol Chem.

[79] Matsumoto S, Shimada A, Kohda D (2013). Crystal structure of the C-terminal globular domain of the third paralog of the Archaeoglobus fulgidus oligosaccharyltransferases. BMC Struct Biol.

[80] Li J, Kato M, Chuang DT (2009). Pivotal role of the C-terminal DW-motif in mediating inhibition of pyruvate dehydrogenase kinase 2 by dichloroacetate. J Biol Chem.

[81] Eisenhaber B, Sinha S, Wong WC, Eisenhaber F (2018). Function of a membrane-embedded domain evolutionarily multiplied in the GPI lipid anchor pathway proteins PIG-B, PIG-M, PIG-U, PIG-W, PIG-V, and PIG-Z. Cell Cycle.

[82] Zhang Y, Skolnick J (2005). TM-align: a protein structure alignment algorithm based on the TM-score. Nucleic Acids Res.

[83] Baker D, Sali A (2001). Protein structure prediction and structural genomics. Science.

[84] Forrest LR, Honig B (2005). An assessment of the accuracy of methods for predicting hydrogen positions in protein structures. Proteins.

[85] Marchler-Bauer A, Panchenko AR, Shoemaker BA, Thiessen PA, Geer LY, Bryant SH (2002). CDD: a database of conserved domain alignments with links to domain three-dimensional structure. Nucleic Acids Res.

[86] Lu S, Wang J, Chitsaz F, Derbyshire MK, Geer RC, Gonzales NR, Gwadz M, Hurwitz DI, Marchler GH, Song JS (2020). CDD/SPARCLE: the conserved domain database in 2020. Nucleic Acids Res.

