# Supplementary material for: Conserved sequence motifs in human TMTC1, TMTC2, TMTC3, and TMTC4, new O-mannosyltransferases from the GT-C/PMT clan, are rationalized as ligand binding sites
Source: Biol Direct. 2021 Jan 12;16:4. doi: 10.1186/s13062-021-00291-w (PMC7801869; doi:10.1186/s13062-021-00291-w)
Supplement: Supplementary file 1 — Additional file 1. The grand alignment of TMTCs. The compressed library file AF1-2020-10-grand-aln-TMTCs.zip provides the alignment shown in Fig. 1 in the “.aln” and “.jvp” formats. [file 13062_2021_291_MOESM1_ESM.zip › AF1-2020-10-grand-aln-TMTCs/index.html]

Supporting Information for Grand Alignment of TMTCs


**Supporting Information for Grand Alignment of TMTCs**
  
  
Filename AF1-2020-10-grand-aln-TMTCs.aln : download
  
  
Filename AF1-2020-10-grand-aln-TMTCs.jvp : download

Additional information for the publication in Biology Direct
  
**"Conserved sequence motifs in human TMTC1, TMTC2, TMTC3, and TMTC4, new O-mannosyltransferases from the GT-C/PMT clan, are rationalized as ligand binding sites"**
  
by Birgit Eisenhaber, Swati Sinha, Chaitanya K. Jadalanki, Vladimir A. Shitov, Qiao Wen Tan, Fernanda L. Sirota, Frank Eisenhaber
