# Supplementary material for: Conserved sequence motifs in human TMTC1, TMTC2, TMTC3, and TMTC4, new O-mannosyltransferases from the GT-C/PMT clan, are rationalized as ligand binding sites
Source: Biol Direct. 2021 Jan 12;16:4. doi: 10.1186/s13062-021-00291-w (PMC7801869; doi:10.1186/s13062-021-00291-w)
Supplement: Supplementary file 3 — Additional file 3. HHPred outputs when searching TMTCs against Pfam or PDB structures. The compressed library file AF3-2020-06-HHPred-TMTCs.zip contains the outputs when running the four human TMTC sequences as input of HHPred against PDB sequences and against Pfam domains (as of 23rd of June 2020). [file 13062_2021_291_MOESM3_ESM.zip › AF3-2020-06-HHPred-TMTCs/HHpred_TMTC1_PDB.html]

(\*) HHpred | Bioinformatics Toolkit          **We're sorry but the Toolkit doesn't work properly without JavaScript enabled. Please enable it to continue.**

Sign In

- Search
- Alignment
- Sequence Analysis
- 2ary Structure
- 3ary Structure
- Classification
- Utils

- HHblits
- HHpred
- HMMER
- PatternSearch
- ProtBLAST/PSI-BLAST

Nothing found.

###### Tools

###### Jobs

ID

Date

Tool

1780993HHPR5863267HHPR5407837HHPR8665047HHPR2161064HHPR

# HHpred

Job ID: 1780993,Created: 25 minutes ago

- Input
- Parameters
- Results
- Raw Output
- Probability Plot
- Query Template MSA
- Query MSA

>XP\_016875493.1\_TMTC1\_Homo sapiens 1..456
MVVTTSARGGGGDRTPSRRRGCGLAPAGAAALLAGASCLCYGRSLQGEFVHDDVWAIVNNPDVRPGAPLRWGIFTNDFWG
KGMAENTSHKSYRPLCVLTFKLNIFLTGMNPFYFHAVNIILHCLVTLVLMYTCDKTVFKNRGLAFVTALLFAVHPIHTEA
VAGIVGRADVLACLLFLLAFLSYNRSLDQGCVGGSFPSTVSPFFLLLSLFLGTCAMLVKETGITVFGVCLVYDLFSLSNK
QDKSYLRASSNRNFLLTMRPFLKRAILVLSYVLVILYFRLWIMGGSMPLFSEQDNPASFSPYILTRFLTYSYLLAFNVWL
LLAPVTLCYDWQVGSIPLVETIWDMRNLATIFLAVVMALLSLHCLAAFKRLEHKEVLVGLLFLVFPFIPASNLFFRVGFV
VAERVLYMPSMGYCILFVHGLSKLCTWLNRCGATTLIVSTVLLLLLFSWKTVKQNE

Paste ExampleUpload File

Protein FASTA

Align two sequences/MSAs

Select structural/domain databases

PDB\_mmCIF70\_29\_May

- PDB\_mmCIF70\_29\_May (default)
- PDB\_mmCIF30\_29\_May
- SCOPe70\_2.07
- ECOD\_ECOD\_F70\_20200207
- COG\_KOG\_v1.0
- Pfam-A\_v33.1
- NCBI\_Conserved\_Domains(CD)\_v3.18
- SMART\_v6.0
- TIGRFAMs\_v15.0
- PRK\_v6.9
- No elements found. Consider changing the search query.
- List is empty.

Select proteomes

Select options

- Euk\_Arabidopsis\_thaliana\_TAIR10\_20\_Jun\_2017
- Euk\_Bombyx\_mori\_p50T\_Dazao\_06\_May\_2019
- Euk\_Brachypodium\_distachyon\_23\_Aug\_2017
- Euk\_Caenorhabditis\_elegans\_18\_Jul\_2017
- Euk\_Capsaspora\_owczarzaki\_ATCC\_30864\_23\_Mar\_2020
- Euk\_Chaetomium\_thermophilum\_29\_Jun\_2017
- Euk\_Chlamydomonas\_reinhardtii\_27\_Jul\_2017
- Euk\_Entamoeba\_histolytica\_HM1\_IMSS\_22\_Mar\_2017
- Euk\_Dictyostelium\_discoideum\_AX4\_19\_Sep\_2017
- Euk\_Drosophila\_melanogaster\_19\_Jul\_2017
- Euk\_Giardia\_lamblia\_ATCC\_50803\_31\_Aug\_2017
- Euk\_Homo\_sapiens\_04\_Jul\_2017
- Euk\_Physcomitrella\_patens\_28\_Aug\_2017
- Euk\_Plasmodium\_falciparum\_3D7\_7\_Jun\_2017
- Euk\_Saccharomyces\_cerevisiae\_S288c\_11\_Mar\_2017
- Euk\_Schizosaccharomyces\_pombe\_19\_Sep\_2017
- Euk\_Solanum\_lycopersicum\_28\_Jul\_2019
- Euk\_Tetrahymena\_thermophila\_SB210\_22\_Aug\_2017
- Euk\_Toxoplasma\_gondii\_ME49\_10\_May\_2018
- Euk\_Trichomonas\_vaginalis\_G3\_21\_Nov\_2018
- Euk\_Trypanosoma\_brucei\_gambiense\_DAL972\_28\_Mar\_2017
- Euk\_Ustilago\_maydis\_521\_29\_May\_2017
- Euk\_Paramecium\_tetraurelia\_9\_Dec\_2018
- Arc\_Archaeoglobus\_fulgidus\_DSM\_4304\_5\_Dec\_2017
- Arc\_Halobacterium\_jilantaiense\_5\_Dec\_2017
- Arc\_Lokiarchaeum\_sp\_GC14\_75\_31\_Oct\_2018
- Arc\_Methanocaldococcus\_jannaschii\_DSM\_2661\_5\_Dec\_2017
- Arc\_Methanosarcina\_mazei\_S\_6\_17\_Mar\_2017
- Arc\_Methanothermus\_fervidus\_DSM\_2088\_5\_Dec\_2017
- Arc\_Pyrococcus\_horikoshii\_OT3\_5\_Dec\_2017
- Arc\_Sulfolobus\_solfataricus\_5\_Dec\_2017
- Arc\_Thermoplasma\_acidophilum\_DSM\_1728\_7\_Dec\_2017
- Bac\_Acinetobacter\_baumannii\_29\_Mar\_2018
- Bac\_Aquifex\_aeolicus\_VF5\_19\_Sep\_2017
- Bac\_Bacillus\_subtilis\_subsp\_subtilis\_str168\_19\_Mar\_2017
- Bac\_Bacteriovorax\_sp\_DB6\_IX\_1\_Jun\_2018
- Bac\_Bdellovibrio\_bacteriovorus\_HD100\_1\_Jun\_2018
- Bac\_Christensenella\_minuta\_2\_Apr\_2019
- Bac\_Deinococcus\_radiodurans\_R1\_19\_Sep\_2017
- Bac\_Enterococcus\_faecalis\_13\_SD\_W\_01\_1\_Jun\_2018
- Bac\_Escherichia\_coli\_K12\_07\_Mar\_2017
- Bac\_Fischerella\_muscicola\_PCC\_7414\_24\_Sep\_2017
- Bac\_Frankia\_alni\_ACN14a\_24\_Sep\_2017
- Bac\_Helicobacter\_pylori\_26695\_1\_Jun\_2018
- Bac\_Leptospira\_interrogans\_serovar\_Lai\_str56601\_1\_Jun\_2018
- Bac\_Mycobacterium\_tuberculosis\_H37Rv\_27\_May\_2017
- Bac\_Neisseria\_gonorrhoeae\_FA\_1090\_1\_Jun\_2018
- Bac\_Neisseria\_meningitidis\_MC58\_9\_Jun\_2017
- Bac\_Nostoc\_punctiforme\_PCC\_73102\_18\_Mar\_2017
- Bac\_Phycisphaerae\_bacterium\_L21\_RPulD3\_1\_Jun\_2018
- Bac\_Plesiocystis\_pacifica\_SIR1\_1\_Jun\_2018
- Bac\_Pseudomonas\_aeruginosa\_PAO1\_5\_Jun\_2017
- Bac\_Salmonella\_ent\_ser\_Typhi\_CT18\_22\_Nov\_2018
- Bac\_Staphylococcus\_aureus\_subsp\_aureus\_NCTC\_8325\_13\_Jun\_2017
- Bac\_Streptomyces\_scabiei\_87.22\_24\_Sep\_2017
- Bac\_Synechocystis\_sp\_PCC\_6803\_6\_Jun\_2017
- Bac\_Tenacibaculum\_dicentrarchi\_27\_Nov\_2017
- Bac\_Tenacibaculum\_maritimum\_NBRC\_15946\_27\_Nov\_2017
- Bac\_Thermus\_aquaticus\_Y51MC23\_24\_Sep\_2017
- Bac\_Thermus\_thermophilus\_HB8\_19\_Sep\_2017
- Bac\_Waddlia\_chondrophila\_WSU\_86\_1044\_1\_Jun\_2018
- Bac\_Yersinia\_pestis\_CO92\_10\_Apr\_2017
- Vir\_SARS-CoV-2\_31\_Mar\_2020
- No elements found. Consider changing the search query.
- List is empty.

Resubmit

MSA generation method

HHblits=>UniRef30

- HHblits=>UniRef30 (default)
- PSI-BLAST=>nr70
- No elements found. Consider changing the search query.
- List is empty.

Maximal no. of MSA generation steps

3

- 0
- 1
- 2
- 3 (default)
- 4
- 5
- 8
- No elements found. Consider changing the search query.
- List is empty.

E-value incl. threshold for MSA generation

1e-3

- 0.1
- 0.05
- 0.02
- 0.01
- 1e-3 (default)
- 1e-6
- 1e-8
- 1e-10
- 1e-15
- 1e-20
- 1e-30
- 1e-40
- 1e-50
- No elements found. Consider changing the search query.
- List is empty.

Min. seq. identity of MSA hits with query (%)

0

- 0 (default)
- 10
- 20
- 30
- 40
- 50
- 60
- 70
- 75
- 80
- 85
- 90
- 95
- 100
- No elements found. Consider changing the search query.
- List is empty.

Min. coverage of MSA hits (%)

20

- 10
- 20 (default)
- 30
- 40
- 50
- 60
- 70
- 80
- 90
- 100
- No elements found. Consider changing the search query.
- List is empty.

Secondary structure scoring

during\_alignment

- none
- after\_alignment
- during\_alignment (default)
- after\_alignment\_pred\_vs\_pred
- during\_alignment\_pred\_vs\_pred
- No elements found. Consider changing the search query.
- List is empty.

Alignment Mode:Realign with MAC

local:norealign

- local:norealign (default)
- local:realign
- global:realign
- No elements found. Consider changing the search query.
- List is empty.

MAC realignment threshold

0.3

- 0.0
- 0.01
- 0.1
- 0.2
- 0.3 (default)
- 0.4
- 0.5
- 0.6
- 0.7
- 0.8
- 0.9
- 0.95
- No elements found. Consider changing the search query.
- List is empty.

No. of target sequences (up to 10000)

250

- 250 (default)
- 500
- 1000
- 2000
- 3000
- 4000
- 5000
- 6000
- 7000
- 8000
- 9000
- 10000
- No elements found. Consider changing the search query.
- List is empty.

Min. probability in hit list (> 10%)

20

- 10
- 20 (default)
- 30
- 40
- 50
- 60
- 70
- 75
- 80
- 85
- 90
- 95
- 100
- No elements found. Consider changing the search query.
- List is empty.

Resubmit

VisHitsAln
Select AllForwardForward Query A3MModel using selectionDownload HHRColor SeqsWrap Seqs

Number of Hits: **17**

Detected sequence features:
**◾Transmembrane segment(s)**

#### Visualization

Resubmit Section

1

456

Prob=99.8%
E=1.8E-17 6S7T\_A Dolichyl-diphosphooligosaccharide--protein
glycosyltransferase subunit STT3B (E.C.2.4.99.18); N-glycosylation,
Oligosaccharyltransferase, OSTB, TRANSFERASE; HET: 0K3, KZB, NAG, EGY,
MAN, BMA; 3.5A {Homo sapiens}

#### Hitlist

Show102550100AllEntries

Search:

| Nr (Click to sort Ascending) | Hit (Click to sort Ascending) | Name (Click to sort Ascending) | Probability (Click to sort Ascending) | E-value (Click to sort Ascending) | SS (Click to sort Ascending) | Cols (Click to sort Ascending) | Target Length (Click to sort Ascending) |
| --- | --- | --- | --- | --- | --- | --- | --- |
| 1 | 5EZM\_A | 4-amino-4-deoxy-L-arabinose (L-Ara4N) transferase; membrane protein, lipid glycosyltransferase, zinc; HET: PC, DSL, PO4, MPG, EPE; 2.7A {Cupriavidus metallidurans (strain ATCC 43123 / DSM 2839 / NBRC 102507 / CH34)}; Related PDB entries: 5F15\_A ; Related PDB entries: 5F15\_A ; Related PDB entries: 5F15\_A | 99.92 | 1.9e-22 | 31.6 | 388 | 578 |
| 2 | 6S7T\_A | Dolichyl-diphosphooligosaccharide--protein glycosyltransferase subunit STT3B (E.C.2.4.99.18); N-glycosylation, Oligosaccharyltransferase, OSTB, TRANSFERASE; HET: 0K3, KZB, NAG, EGY, MAN, BMA; 3.5A {Homo sapiens} | 99.84 | 1.8e-17 | 36.2 | 397 | 826 |
| 3 | 6S7O\_A | Dolichyl-diphosphooligosaccharide--protein glycosyltransferase subunit STT3A (E.C.2.4.99.18); N-glycosylation, Oligosaccharyltransferase, OSTA, TRANSFERASE; HET: KZB, NAG, EGY, MAN, KZE, BMA; 3.5A {Homo sapiens}; Related PDB entries: 6FTI\_5 6FTG\_5 6FTJ\_5; Related PDB entries: 6FTG\_5 6FTI\_5 6FTJ\_5; Related PDB entries: 6FTG\_5 6FTI\_5 6FTJ\_5 | 99.84 | 2.2e-17 | 34.7 | 397 | 705 |
| 4 | 6EZN\_F | Dolichyl-diphosphooligosaccharide--protein glycosyltransferase subunit 1 (E.C.2.4.99.18); OST complex, oligosaccharyltransferase, N-linked glycosylation; HET: PTY, BMA, CPL, MAN, NAG;{Saccharomyces cerevisiae (strain ATCC 204508 / S288c)}; Related PDB entries: 6C26\_A; Related PDB entries: 6C26\_A; Related PDB entries: 6C26\_A | 99.84 | 1.4e-17 | 32.9 | 407 | 718 |
| 5 | 3WAJ\_A | Transmembrane oligosaccharyl transferase (E.C.2.4.1.119); oligosaccharyltransferase, N-glycosylation, Archaeoglobus fulgidus, GT-C; 2.501A {Archaeoglobus fulgidus}; Related PDB entries: 5GMY\_A 3WAK\_A; Related PDB entries: 5GMY\_A 3WAK\_A; Related PDB entries: 5GMY\_A 3WAK\_A | 99.84 | 2.6e-17 | 35.1 | 408 | 875 |
| 6 | 5OGL\_A | Peptide-binding protein, Substrate mimicking peptide; Oligosaccharyltransferase, Complex, Protein N-glycosylation, Bacteria; HET: 9UB, PPN; 2.7A {Campylobacter lari (strain RM2100 / D67 / ATCC BAA-1060)}; Related PDB entries: 3RCE\_A 6GXC\_A; Related PDB entries: 6GXC\_A 3RCE\_A ; Related PDB entries: 6GXC\_A 3RCE\_A | 99.79 | 2e-16 | 28.8 | 374 | 713 |
| 7 | 6P25\_A | Dolichyl-diphosphooligosaccharide--protein glycosyltransferase subunits (E.C.2.4.99.18); complex, TRANSFERASE, glycosylation; HET: NAG, CPL, NNM; 3.2A {Saccharomyces cerevisiae W303}; Related PDB entries: 6P2R\_A ; Related PDB entries: 6P2R\_A ; Related PDB entries: 6P2R\_A | 99.75 | 5.7e-14 | 39.4 | 402 | 817 |
| 8 | 6P25\_B | Dolichyl-diphosphooligosaccharide--protein glycosyltransferase subunits (E.C.2.4.99.18); complex, TRANSFERASE, glycosylation; HET: NAG, CPL, NNM; 3.2A {Saccharomyces cerevisiae W303}; Related PDB entries: 6P2R\_B ; Related PDB entries: 6P2R\_B ; Related PDB entries: 6P2R\_B | 99.67 | 1.8e-14 | 24.1 | 259 | 759 |
| 9 | 7BVF\_A | Probable arabinosyltransferase B (E.C.2.4.2.-), Probable; Mycobacterium tuberculosis, cell wall synthesis; HET: 95E, DSL, CDL;{Mycolicibacterium smegmatis MC2 155} | 99.61 | 1.9e-12 | 29.4 | 360 | 1102 |
| 10 | 7BVF\_B | Probable arabinosyltransferase B (E.C.2.4.2.-), Probable; Mycobacterium tuberculosis, cell wall synthesis; HET: 95E, DSL, CDL;{Mycolicibacterium smegmatis MC2 155} | 99.54 | 1.5e-11 | 28.6 | 357 | 1116 |
| 11 | 6W98\_A | F5/8 type C domain-containing protein; Glycosyltransferase, lipomannan, lipoarabinomannan, arabinofuranose, membrane; HET: PNS, 6OU; 2.9A {Escherichia coli (strain K12)}; Related PDB entries: 6WBX\_A 6WBY\_A | 99.51 | 3.2e-11 | 30.7 | 398 | 1413 |
| 12 | 7BWR\_A | Integral membrane indolylacetylinositol arabinosyltransferase EmbB; Mycobacterium tuberculosis, EmbB, cryo-EM, ethambutol; HET: F8L;{Mycolicibacterium smegmatis MC2 155}; Related PDB entries: 7BVC\_B 7BVG\_B 7BWR\_B 7BX8\_B 7BX8\_A | 99.51 | 1.4e-11 | 26.1 | 351 | 1082 |
| 13 | 7BVE\_B | Integral membrane indolylacetylinositol arabinosyltransferase EmbC; Mycobacterium smegmatis, cell wall synthesis; HET: PO4, PN7, 95E; 2.81A {Mycolicibacterium smegmatis MC2 155}; Related PDB entries: 7BVH\_B 7BVH\_A 7BVE\_A | 99.49 | 1.1e-10 | 30.2 | 357 | 1084 |
| 14 | 6SNI\_X | Dolichyl pyrophosphate Man9GlcNAc2 alpha-1,3-glucosyltransferase (E.C.2.4.1.267); Glycosyltransferase, Glucosyltransferase, GT-C, N-Glycosylation, MEMBRANE; HET: PTY, Y01;{Saccharomyces cerevisiae}; Related PDB entries: 6SNH\_X | 99.47 | 1.5e-10 | 29 | 344 | 562 |
| 15 | 7BVC\_A | Integral membrane indolylacetylinositol arabinosyltransferase EmbA; Mycobacterium smegmatis, cell wall synthesis; HET: 95E, PNS, CDL, F8L;{Mycolicibacterium smegmatis MC2 155}; Related PDB entries: 7BVG\_A | 99.33 | 2.5e-9 | 27.4 | 357 | 1088 |
| 16 | 6P25\_B | Dolichyl-diphosphooligosaccharide--protein glycosyltransferase subunits (E.C.2.4.99.18); complex, TRANSFERASE, glycosylation; HET: NAG, CPL, NNM; 3.2A {Saccharomyces cerevisiae W303}; Related PDB entries: 6P2R\_B ; Related PDB entries: 6P2R\_B ; Related PDB entries: 6P2R\_B | 75.12 | 53 | 11.8 | 107 | 759 |
| 17 | 6S7T\_A | Dolichyl-diphosphooligosaccharide--protein glycosyltransferase subunit STT3B (E.C.2.4.99.18); N-glycosylation, Oligosaccharyltransferase, OSTB, TRANSFERASE; HET: 0K3, KZB, NAG, EGY, MAN, BMA; 3.5A {Homo sapiens} | 30.95 | 650 | 25.9 | 214 | 826 |

Displaying 1 to 17 of 17 hits

- «
- ‹
- 1
- ›
- »

#### Alignments

|  |  |  |  |
| --- | --- | --- | --- |
|  | | | |
|  | Template alignmentTemplate 3D StructurePDBe | | |
| 1. | 5EZM\_A 4-amino-4-deoxy-L-arabinose (L-Ara4N) transferase; membrane protein, lipid glycosyltransferase, zinc; HET: PC, DSL, PO4, MPG, EPE; 2.7A {Cupriavidus metallidurans (strain ATCC 43123 / DSM 2839 / NBRC 102507 / CH34)}; Related PDB entries: 5F15\_A ; Related PDB entries: 5F15\_A ; Related PDB entries: 5F15\_A | | |
|  | Probability: 99.92%, E-value: 1.9e-22, Score: 190.9, Aligned cols: 388, Identities: 8%, Similarity: -0.082, | | |
|  |
|  | Q ss\_pred |  | CceecccCCCCCCCCccchhhccHHHHHHHHHHHHHHHHHHhhhcCCCcccccHHHHHhCCCCCCCCCcccccccccccc |
|  | Q XP\_016875493.1 | 1 | MVVTTSARGGGGDRTPSRRRGCGLAPAGAAALLAGASCLCYGRSLQGEFVHDDVWAIVNNPDVRPGAPLRWGIFTNDFWG   80 (456) |
|  | Q Consensus | 1 | m~~~~~~~~~~~~~~~~~~~~~~~~~~~~~~~l~~~~~~~~~~~~~~~~~~De~~~~~~a~~~~~~~~~~~~~~~~~~~~   80 (456) |
|  |  |  | +.+..+..+....................+++++.+..............+||..|...++++.+++.+.....+.. |
|  | T Consensus | 7 | ~~~~~~~~~~~~~~~~~~~~~~~~~~~~~~~~~~~~~~~~~~~~~~~~~~~De~~~~~~a~~~~~~~~~~~~~~~~~---   83 (578) |
|  | T 5EZM\_A | 7 | PTSQQRASVASSQSTQGAVGWSAATGWVVLFVAVALVVWFVSLDMRHLVGPDEGRYAEISREMFASGDWVTIRYNAL---   83 (578) |
|  | T ss\_dssp |  | ---------------------CCTHHHHHHHHHHHHHHHHHGGGSSCCCTTHHHHHHHHHHHHHHHCCSSSCEETTE--- |
|  | T ss\_pred |  | CCcccccccccCCCccchhhHHHHHHHHHHHHHHHHHHHHhccccccCCCCCHHHHHHHHHHHHHhCCceeEEECCE--- |
|  |
|  |
|  | Q ss\_pred |  | cCCCCCCCcccccchHHHHHHHHHHHhCCCchHHHHHHHHHHHHHHHHHHHHHHHHhccchHHHHHHHHHHHHCcccHHH |
|  | Q XP\_016875493.1 | 81 | KGMAENTSHKSYRPLCVLTFKLNIFLTGMNPFYFHAVNIILHCLVTLVLMYTCDKTVFKNRGLAFVTALLFAVHPIHTEA   160 (456) |
|  | Q Consensus | 81 | ~~~~~~~~~~~~~Pl~~~~~~~~~~l~g~~~~~~rl~~~l~~~~~~~~~~~l~~~~~~~~~~~a~~aa~l~~~~p~~~~~   160 (456) |
|  |  |  | .+..+||++.++.+....++|.++...|++++++++++++++|.++|+.. +++.+++++++++++|..... |
|  | T Consensus | 84 | -------~~~~~pPl~~~l~~~~~~l~g~~~~~~rl~~~l~~~l~~~~~~~l~~~~~--~~~~a~~a~~l~~~~p~~~~~   154 (578) |
|  | T 5EZM\_A | 84 | -------KYFEKPPFHMWVTVVGYELFGLGEWQARLAVALSGLLGIGVSMMAARRWF--GARAAAFTGLALLAAPMWSVA   154 (578) |
|  | T ss\_dssp |  | -------ECCSSCSHHHHHHHHHHHHHCSSHHHHTHHHHHHHHHHHHHHHHHHHHHH--CHHHHHHHHHHHHHCHHHHHH |
|  | T ss\_pred |  | -------eCCCCCHHHHHHHHHHHHHHCcCHHHHHHHHHHHHHHHHHHHHHHHHHHh--ChHHHHHHHHHHHHHHHHHHH |
|  |
|  |
|  | Q ss\_pred |  | HHhhhhhHHHHHHHHHHHHHHHHHHHHhc-----CCCCCCCCcchhHHHHHHHHHHHHHHHHhHHHHHHHHHHHHHHHHH |
|  | Q XP\_016875493.1 | 161 | VAGIVGRADVLACLLFLLAFLSYNRSLDQ-----GCVGGSFPSTVSPFFLLLSLFLGTCAMLVKETGITVFGVCLVYDLF   235 (456) |
|  | Q Consensus | 161 | ~~~~~~~~~~~~~~~~~l~~~~~~~~~~~-----~~~~~~~~~~~~~~~~~~~~~~~~la~~~k~~~~~~~~~~~~~~~~   235 (456) |
|  |  |  | ....++|.+..++.+++++++.+..++ + +.++.++++++++++.++|+....+.+...+.... |
|  | T Consensus | 155 | --~~~~~~~~~~~~~~~~~~~~~~~~~~~~~~~~~----------~~~~~~l~g~~~gla~~~k~~~~~~~~~~~~~~~~   222 (578) |
|  | T 5EZM\_A | 155 | --AHFNTLDMTLAGVMSCVLAFMLMGQHPDASVAA----------RRGWMVACWAAMGVAILTKGLVGIALPGLVLVVYT   222 (578) |
|  | T ss\_dssp |  | --HTSCCHHHHHHHHHHHHHHHHHHHTCTTSCHHH----------HHHHHHHHHHHHHHHHHHHTTHHHHHHHHHHHHHH |
|  | T ss\_pred |  | --HhhHhhHHHHHHHHHHHHHHHHHHhCCCcchhh----------ccHHHHHHHHHHHHHHHccchHHHHHHHHHHHHHH |
|  |
|  |
|  | Q ss\_pred |  | hcccccchhHhhccCchhHHHhhHHHHHHHHHHHHHHHHHHHHHHHHhcCCCCccccCCCCcccchhHHHHHHHHHHHHH |
|  | Q XP\_016875493.1 | 236 | SLSNKQDKSYLRASSNRNFLLTMRPFLKRAILVLSYVLVILYFRLWIMGGSMPLFSEQDNPASFSPYILTRFLTYSYLLA   315 (456) |
|  | Q Consensus | 236 | ~~~~~~~~~~~~~~~~~~~~~~~~~~~~~~~~~~~~~~~~~~~~~~~~~~~~~~~~~~~~~~~~~~~~~~~~~~~~~~~~   315 (456) |
|  |  |  | ..++++++...+ ......++..+...++.......... |
|  | T Consensus | 223 | ~~~~~~~~~~~~---------------~~~~~~~~~~~~~~~~~~~~~~~~~~---------------------------   260 (578) |
|  | T 5EZM\_A | 223 | LVTRDWGLWRRL---------------HLALGVVVMLVITVPWFYLVSVRNPE---------------------------   260 (578) |
|  | T ss\_dssp |  | HHSCCTTHHHHT---------------CHHHHHHHHHHHHHHHHHHHHHHCTT--------------------------- |
|  | T ss\_pred |  | HHcCChHHHhhc---------------chHHHHHHHHHHHHHHHHHHHHHCCc--------------------------- |
|  |
|  |
|  | Q ss\_pred |  | HHHHHHHHhHHhhccCCCCCCccccchhhHHHHHHHHHHHHHHHHHHHHHHHHhcccchHHHHHHHHHHHHHhhHhcccc |
|  | Q XP\_016875493.1 | 316 | FNVWLLLAPVTLCYDWQVGSIPLVETIWDMRNLATIFLAVVMALLSLHCLAAFKRLEHKEVLVGLLFLVFPFIPASNLFF   395 (456) |
|  | Q Consensus | 316 | ~~~~~~~~~~~~~~~~~~~~~~~~~~~~~~~~~~~~~~~~~~~~~~~~~~~~~~~~~~~~~~~~~~~~~~~~~~~~~~~~   395 (456) |
|  |  |  | ..................................................+++++++..................... |
|  | T Consensus | 261 | --~~~~~~~~~~~~~~~~~~~~~~~~~~~~~~~~~~~~~~~~~~~~~~~~~~~~~~~~~~~~~~~~~~~~~~~~~~~~~~   338 (578) |
|  | T 5EZM\_A | 261 | --FPNFFFIHEHWQRYTSNIHSRSGSVFYFLPLVIGGFLPWAGIFPKLWTAMRAPVEGTQARFRPALMAGIWAIAIFVFF   338 (578) |
|  | T ss\_dssp |  | --HHHHHHHCCCCCC-------CCCCTTTHHHHHHHHTGGGGGGHHHHHHHHCC-------CCCHHHHHHHHHHHHHHHH |
|  | T ss\_pred |  | --chhhhHHHHHHHHHhcCcccCCCChHHHHHHHHHHhhhHHhHHHHHHHHhhcccccCcccccHHHHHHHHHHHHHHHH |
|  |
|  |
|  | Q ss\_pred |  | CCCccccccccchHHHHHHHHHHHHHHHHHHHHhHHhHHHHHHHHHHHHHHHHHHHHHhcC |
|  | Q XP\_016875493.1 | 396 | RVGFVVAERVLYMPSMGYCILFVHGLSKLCTWLNRCGATTLIVSTVLLLLLFSWKTVKQNE   456 (456) |
|  | Q Consensus | 396 | ~~~~~~~~Ry~~~~~~~~~ll~~~~~~~~~~~~~~~~~~~~~~~~~~~~~~~~~~~~~~~~   456 (456) |
|  |  |  | .......+||.++..|+++++++.++.++.++.+++.......+++++.+........... |
|  | T Consensus | 339 | ~~~~~~~~ry~~~~~p~l~ll~~~~l~~~~~~~~~~~~~~~~~~~~~~~~~~~~~~~~~~~   399 (578) |
|  | T 5EZM\_A | 339 | SISRSKLPGYIVPVIPALGILAGVALDRLSPRSWGKQLIGMAIVAACGLLASPVVATLNAN   399 (578) |
|  | T ss\_dssp |  | HTSSSCCGGGGTTHHHHHHHHHHHHHHTCCHHHHHHHHHHHHHHHHHHHHHGGGGGGCCCT |
|  | T ss\_pred |  | HHhcccChhcHHhHHHHHHHHHHHHHHHhCCCccHHHHHHHHHHHHHHHHHHHHHHHhhhc |
|  |
| --- | | | |
|  | Template alignmentTemplate 3D StructurePDBe | | |
| 2. | 6S7T\_A Dolichyl-diphosphooligosaccharide--protein glycosyltransferase subunit STT3B (E.C.2.4.99.18); N-glycosylation, Oligosaccharyltransferase, OSTB, TRANSFERASE; HET: 0K3, KZB, NAG, EGY, MAN, BMA; 3.5A {Homo sapiens} | | |
|  | Probability: 99.84%, E-value: 1.8e-17, Score: 163.75, Aligned cols: 397, Identities: 10%, Similarity: -0.024, | | |
|  |
|  | Q ss\_pred |  | CceecccCCCCCCCCccchhhccHHHHHHHHHHHHHHHHHHhhh-cCCCcc---cccHHHHHhCCCCCCCCCcccccccc |
|  | Q XP\_016875493.1 | 1 | MVVTTSARGGGGDRTPSRRRGCGLAPAGAAALLAGASCLCYGRS-LQGEFV---HDDVWAIVNNPDVRPGAPLRWGIFTN   76 (456) |
|  | Q Consensus | 1 | m~~~~~~~~~~~~~~~~~~~~~~~~~~~~~~~l~~~~~~~~~~~-~~~~~~---~De~~~~~~a~~~~~~~~~~~~~~~~   76 (456) |
|  |  |  | +++.+.....+.................++++++++++...... ...+.. .||..|...++++.+++......+.+ |
|  | T Consensus | 48 | ~~~~~~~~~~~~~~~~~~~~~~~~~~~~~l~~i~~~~~~~rl~~~~~~~~~~~~~D~~~~~~~a~~~~~~g~~~~~~~~~   127 (826) |
|  | T 6S7T\_A | 48 | AAPPKPAPAGLSGGLSQPAGWQSLLSFTILFLAWLAGFSSRLFAVIRFESIIHEFDPWFNYRSTHHLASHGFYEFLNWFD   127 (826) |
|  | T ss\_dssp |  | ---------------CCHHHHHHHHHHHHHHHHHHHHHHTTCTTTTTTCSCCCSTTHHHHHHHHHHHHHHCHHHHHTCEE |
|  | T ss\_pred |  | CCCCCCCCCCCCCCCCCCchHHHHHHHHHHHHHHHHHHHHHHHHHHhhhhhhcCCChHHHHHHHHHHHHhCcHHHhcccc |
|  |
|  |
|  | Q ss\_pred |  | cccccCCCCCCCcccccchHHHHHHHHHHHhC------CCchHHHHHHHHHHHHHHHHHHHHHHHHhccchHHHHHHHHH |
|  | Q XP\_016875493.1 | 77 | DFWGKGMAENTSHKSYRPLCVLTFKLNIFLTG------MNPFYFHAVNIILHCLVTLVLMYTCDKTVFKNRGLAFVTALL   150 (456) |
|  | Q Consensus | 77 | ~~~~~~~~~~~~~~~~~Pl~~~~~~~~~~l~g------~~~~~~rl~~~l~~~~~~~~~~~l~~~~~~~~~~~a~~aa~l   150 (456) |
|  |  |  | .....+...... ..++|++.++.+..+.++| .+....|+++.++++++++++|.++|+.. ++..|++++++ |
|  | T Consensus | 128 | ~~~~~p~g~~~~-~~~~P~~~~l~a~~~~l~g~~~~~~~~~~~~~l~~~l~~~l~~~~~y~l~r~l~--~~~~allaall   204 (826) |
|  | T 6S7T\_A | 128 | ERAWYPLGRIVG-GTVYPGLMITAGLIHWILNTLNITVHIRDVCVFLAPTFSGLTSISTFLLTRELW--NQGAGLLAACF   204 (826) |
|  | T ss\_dssp |  | CSSSTTTCEEST-TSSCTTHHHHHHHHHHHHHHTTCCCCHHHHHHTHHHHHHHHHHHHHHHHHHHHS--CHHHHHHHHHH |
|  | T ss\_pred |  | chhcCCCCCCCC-CCCchHHHHHHHHHHHHHHhcCCCCcHHHHHHHHHHHHHHHHHHHHHHHHHHHc--CchHHHHHHHH |
|  |
|  |
|  | Q ss\_pred |  | HHHCcccHHHHHhhhhhHHHHHHHHHHHHHHHHHHHHhcCCCCCCCCcchhHHHHHHHHHHHHHHHHhHHHHHHHHHHHH |
|  | Q XP\_016875493.1 | 151 | FAVHPIHTEAVAGIVGRADVLACLLFLLAFLSYNRSLDQGCVGGSFPSTVSPFFLLLSLFLGTCAMLVKETGITVFGVCL   230 (456) |
|  | Q Consensus | 151 | ~~~~p~~~~~~~~~~~~~~~~~~~~~~l~~~~~~~~~~~~~~~~~~~~~~~~~~~~~~~~~~~la~~~k~~~~~~~~~~~   230 (456) |
|  |  |  | ++++|.++..+......+|.+..++.+++++++.+..+++ +.++.++++++++++.++|+.+.+++++++ |
|  | T Consensus | 205 | ~a~~p~~~~~s~~~~~~~e~~~~~~~~l~l~~~~~~~~~~----------~~~~~~l~gl~~gla~~~~~~~~~~~~~~~   274 (826) |
|  | T 6S7T\_A | 205 | IAIVPGYISRSVAGSFDNEGIAIFALQFTYYLWVKSVKTG----------SVFWTMCCCLSYFYMVSAWGGYVFIINLIP   274 (826) |
|  | T ss\_dssp |  | TTTCHHHHGGGSTTCCCSHHHHHHHHHHHHHHHHHHHHHC----------CHHHHHHHHHHHHHHHHHCTTHHHHHHHHH |
|  | T ss\_pred |  | HHHHHHHHHhhhccCchHHHHHHHHHHHHHHHHHHHhccC----------cHHHHHHHHHHHHHHHHhcccHHHHHHHHH |
|  |
|  |
|  | Q ss\_pred |  | HHHHHhcccccchhH---------------------------------------------------------hhccCchh |
|  | Q XP\_016875493.1 | 231 | VYDLFSLSNKQDKSY---------------------------------------------------------LRASSNRN   253 (456) |
|  | Q Consensus | 231 | ~~~~~~~~~~~~~~~---------------------------------------------------------~~~~~~~~   253 (456) |
|  |  |  | ++.++....+++++. .+ |
|  | T Consensus | 275 | l~~~~~~~~~~~~~~~~~~~~~~~~~~~~~~~~~p~~~~~~~~~~~~~~~~~~~~~~~~~~~~~~~~~~~~~~~------   348 (826) |
|  | T 6S7T\_A | 275 | LHVFVLLLMQRYSKRVYIAYSTFYIVGLILSMQIPFVGFQPIRTSEHMAAAGVFALLQAYAFLQYLRDRLTKQE------   348 (826) |
|  | T ss\_dssp |  | HHHHHHHHTTCCCHHHHHHHHHHHHHHHHHHTTSTTTTTHHHHBSSTHHHHHHHHHHHHHHHHHHHHHHSCSTT------ |
|  | T ss\_pred |  | HHHHHHHHhccCChhHHHHHHHHHHHHHHHHhccccCCCCcccchHHHHHHHHHHHHHHHHHHHHHHHhcCHHH------ |
|  |
|  |
|  | Q ss\_pred |  | HHHhhHHHHHHHHHHHHHHHHHHHHHHHHhcCCCCccccCCCCcccchhHHHHHHHHHHHHHHHHHHHHHhHHhhccCCC |
|  | Q XP\_016875493.1 | 254 | FLLTMRPFLKRAILVLSYVLVILYFRLWIMGGSMPLFSEQDNPASFSPYILTRFLTYSYLLAFNVWLLLAPVTLCYDWQV   333 (456) |
|  | Q Consensus | 254 | ~~~~~~~~~~~~~~~~~~~~~~~~~~~~~~~~~~~~~~~~~~~~~~~~~~~~~~~~~~~~~~~~~~~~~~~~~~~~~~~~   333 (456) |
|  |  |  | ..........++..+.................... ......+......... |
|  | T Consensus | 349 | -----~~~~~~~~~~~~~~~~~~~~~~~~~~~~~~~~~~~------------------------~~~~~~~~~~~~~~~~   399 (826) |
|  | T 6S7T\_A | 349 | -----FQTLFFLGVSLAAGAVFLSVIYLTYTGYIAPWSGR------------------------FYSLWDTGYAKIHIPI   399 (826) |
|  | T ss\_dssp |  | -----THHHHHHHHHHHHHHHHHHHHHHHHHTSSBCCCHH------------------------HHHHHHSSHHHHTCHH |
|  | T ss\_pred |  | -----HHHHHHHHHHHHHHHHHHHHHHHHhccccccchHH------------------------HHHHhcccccccCccc |
|  |
|  |
|  | Q ss\_pred |  | CCCccccchhhHHHHHHHHHHHHHHHHHHHHHHHHhcccchHHHHHHHHHHHHHhhHhccccCCCccccccccchHHHHH |
|  | Q XP\_016875493.1 | 334 | GSIPLVETIWDMRNLATIFLAVVMALLSLHCLAAFKRLEHKEVLVGLLFLVFPFIPASNLFFRVGFVVAERVLYMPSMGY   413 (456) |
|  | Q Consensus | 334 | ~~~~~~~~~~~~~~~~~~~~~~~~~~~~~~~~~~~~~~~~~~~~~~~~~~~~~~~~~~~~~~~~~~~~~~Ry~~~~~~~~   413 (456) |
|  |  |  | .....................+.+++........++++.............+.+.... .||.++..|++ |
|  | T Consensus | 400 | ~~~~~~~~~~~~~~~~~~~~~~~~~~~~~~~~~~~~~~~~~~~~~~~~~~~~~~~~~~-----------~Ry~~~~~p~~   468 (826) |
|  | T 6S7T\_A | 400 | IASVSEHQPTTWVSFFFDLHILVCTFPAGLWFCIKNINDERVFVALYAISAVYFAGVM-----------VRLMLTLTPVV   468 (826) |
|  | T ss\_dssp |  | HHHBSTTSCCCHHHHHHSCSSHHHHHHHHHHHHHHSCCHHHHHHHHHHHHHHHHHTTC-----------STTHHHHHHHH |
|  | T ss\_pred |  | hhcccccCCCCHHHHHHHHHHHHHHHHHHHHHHHhcCCHHHHHHHHHHHHHHHHHHHH-----------HHHHHhHHHHH |
|  |
|  |
|  | Q ss\_pred |  | HHHHHHHHHHHHHHHhHH-------------------------------------------------hHHHHHHHHHHHH |
|  | Q XP\_016875493.1 | 414 | CILFVHGLSKLCTWLNRC-------------------------------------------------GATTLIVSTVLLL   444 (456) |
|  | Q Consensus | 414 | ~ll~~~~~~~~~~~~~~~-------------------------------------------------~~~~~~~~~~~~~   444 (456) |
|  |  |  | +++++.++..+.++..+. ......+++++++ |
|  | T Consensus | 469 | ~ll~a~~l~~l~~~~~~~~~~~~~~~~~~~~~~~~~~~~~~~~~~~~~~~~~~~~~~~~~~~~~~~~~~~~~~~~~~~~~   548 (826) |
|  | T 6S7T\_A | 469 | CMLSAIAFSNVFEHYLGDDMKRENPPVEDSSDEDDKRNQGNLYDKAGKVRKHATEQEKTEEGLGPNIKSIVTMLMLMLLM   548 (826) |
|  | T ss\_dssp |  | HHHHHHHHHHHHHHTCC-----------------------------------------------CHHHHHHHHHHHHHHH |
|  | T ss\_pred |  | HHHHHHHHHHHHHHHhccccccCCCCCCCCCccchhhccccccccccccccccchhhhhhcCCChHHHHHHHHHHHHHHH |
|  |
|  |
|  | Q ss\_pred |  | HHHHHHHHHhcC |
|  | Q XP\_016875493.1 | 445 | LLFSWKTVKQNE   456 (456) |
|  | Q Consensus | 445 | ~~~~~~~~~~~~   456 (456) |
|  |  |  | ............ |
|  | T Consensus | 549 | ~~~~~~~~~~~~   560 (826) |
|  | T 6S7T\_A | 549 | MFAVHCTWVTSN   560 (826) |
|  | T ss\_dssp |  | HHHHHHHHHCCC |
|  | T ss\_pred |  | HHHHHHHHHhhh |
|  |
| --- | | | |
|  | Template alignmentTemplate 3D StructurePDBe | | |
| 3. | 6S7O\_A Dolichyl-diphosphooligosaccharide--protein glycosyltransferase subunit STT3A (E.C.2.4.99.18); N-glycosylation, Oligosaccharyltransferase, OSTA, TRANSFERASE; HET: KZB, NAG, EGY, MAN, KZE, BMA; 3.5A {Homo sapiens}; Related PDB entries: 6FTI\_5 6FTG\_5 6FTJ\_5; Related PDB entries: 6FTG\_5 6FTI\_5 6FTJ\_5; Related PDB entries: 6FTG\_5 6FTI\_5 6FTJ\_5 | | |
|  | Probability: 99.84%, E-value: 2.2e-17, Score: 160.49, Aligned cols: 397, Identities: 10%, Similarity: -0.066, | | |
|  |
|  | Q ss\_pred |  | CCCCCCCCccchhhccHHHHHHHHHHHHHHHHHHhhh----cCCCcccccHHHHHhCCCCCCCCCcccccccccccccCC |
|  | Q XP\_016875493.1 | 8 | RGGGGDRTPSRRRGCGLAPAGAAALLAGASCLCYGRS----LQGEFVHDDVWAIVNNPDVRPGAPLRWGIFTNDFWGKGM   83 (456) |
|  | Q Consensus | 8 | ~~~~~~~~~~~~~~~~~~~~~~~~~l~~~~~~~~~~~----~~~~~~~De~~~~~~a~~~~~~~~~~~~~~~~~~~~~~~   83 (456) |
|  |  |  | ++++++................+++++++.+...... .......||..|...++++.+++.. ......+...... |
|  | T Consensus | 1 | m~~~~~~~~~~~~~~~~~~~~~l~~~~~~~~~~~~~~~~~~~~~~~~~D~~~~~~~a~~~~~~g~~-~~~~~~~~~~~~~   79 (705) |
|  | T 6S7O\_A | 1 | MTKFGFLRLSYEKQDTLLKLLILSMAAVLSFSTRLFAVLRFESVIHEFDPYFNYRTTRFLAEEGFY-KFHNWFDDRAWYP   79 (705) |
|  | T ss\_dssp |  | ------CCCCHHHHHHHHHHHHHHHHHHHHHHHHCSHHHHSCCCCCSSSHHHHHHHHHHHHHHCSH-HHHSCEECSSSTT |
|  | T ss\_pred |  | CCccccccCChHHHHHHHHHHHHHHHHHHHHHHHHHHHHhcccccccCChHHHHHHHHHHHHhCCh-hhhccccccccCC |
|  |
|  |
|  | Q ss\_pred |  | CCCCCcccccchHHHHHHHHHHHhC------CCchHHHHHHHHHHHHHHHHHHHHHHHHhccchHHHHHHHHHHHHCccc |
|  | Q XP\_016875493.1 | 84 | AENTSHKSYRPLCVLTFKLNIFLTG------MNPFYFHAVNIILHCLVTLVLMYTCDKTVFKNRGLAFVTALLFAVHPIH   157 (456) |
|  | Q Consensus | 84 | ~~~~~~~~~~Pl~~~~~~~~~~l~g------~~~~~~rl~~~l~~~~~~~~~~~l~~~~~~~~~~~a~~aa~l~~~~p~~   157 (456) |
|  |  |  | ........++|++.++.+..+.++| .+....|+.++++++++++++|.++|+.. ++..|++++++++++|.+ |
|  | T Consensus | 80 | ~g~~~~~~~~p~~~~~~~~~~~l~g~~g~~~~~~~~~~~~~~l~~~l~~~~~y~l~~~~~--~~~~al~aa~l~~~~p~~   157 (705) |
|  | T 6S7O\_A | 80 | LGRIIGGTIYPGLMITSAAIYHVLHFFHITIDIRNVCVFLAPLFSSFTTIVTYHLTKELK--DAGAGLLAAAMIAVVPGY   157 (705) |
|  | T ss\_dssp |  | SCEEHHHHSCCHHHHHHHHHHHHHHHTTCCCCHHHHHHTHHHHHHHHHHHHHHHHHHHHS--CHHHHHHHHHHHHSCHHH |
|  | T ss\_pred |  | CcCCCCCcCCchHHHHHHHHHHHHHHcCCCCCHHHHHHHHHHHHHHHHHHHHHHHHHHHc--CHHHHHHHHHHHHHHHHH |
|  |
|  |
|  | Q ss\_pred |  | HHHHHhhhhhHHHHHHHHHHHHHHHHHHHHhcCCCCCCCCcchhHHHHHHHHHHHHHHHHhHHHHHHHHHHHHHHHHHhc |
|  | Q XP\_016875493.1 | 158 | TEAVAGIVGRADVLACLLFLLAFLSYNRSLDQGCVGGSFPSTVSPFFLLLSLFLGTCAMLVKETGITVFGVCLVYDLFSL   237 (456) |
|  | Q Consensus | 158 | ~~~~~~~~~~~~~~~~~~~~l~~~~~~~~~~~~~~~~~~~~~~~~~~~~~~~~~~~la~~~k~~~~~~~~~~~~~~~~~~   237 (456) |
|  |  |  | ...+.....++|.+..++.+++++++.+..+++ +.++.++++++.+++.++|+.+....+++.+..+... |
|  | T Consensus | 158 | ~~~~~~~~~~~~~~~~~~~~~~~~~~~~~~~~~----------~~~~~~~~gl~~~l~~~~~~~~~~~~~~~~~~~~~~~   227 (705) |
|  | T 6S7O\_A | 158 | ISRSVAGSYDNEGIAIFCMLLTYYMWIKAVKTG----------SICWAAKCALAYFYMVSSWGGYVFLINLIPLHVLVLM   227 (705) |
|  | T ss\_dssp |  | HHSSCTTCCCHHHHHHHHHHHHHHHHHHHHHHC----------CHHHHHHHHHHHHHHHHHCTTHHHHTTTHHHHHHHHH |
|  | T ss\_pred |  | HHhhcccchhHHHHHHHHHHHHHHHHHHHHhhC----------CHHHHHHHHHHHHHHHHhhchHHHHHHHHHHHHHHHH |
|  |
|  |
|  | Q ss\_pred |  | ccccchhH--------------------------------------------------hhccCchhHHHhhHHHHHHHHH |
|  | Q XP\_016875493.1 | 238 | SNKQDKSY--------------------------------------------------LRASSNRNFLLTMRPFLKRAIL   267 (456) |
|  | Q Consensus | 238 | ~~~~~~~~--------------------------------------------------~~~~~~~~~~~~~~~~~~~~~~   267 (456) |
|  |  |  | ..+++++. .++...+............... |
|  | T Consensus | 228 | ~~~~~~~~~~~~~~~~~~~~~~~~~~~~~~~~~~~~~~~~~~~~~~~~~~~~~~~~~~~~~~~~~~~~~~~~~~~~~~~~   307 (705) |
|  | T 6S7O\_A | 228 | LTGRFSHRIYVAYCTVYCLGTILSMQISFVGFQPVLSSEHMAAFGVFGLCQIHAFVDYLRSKLNPQQFEVLFRSVISLVG   307 (705) |
|  | T ss\_dssp |  | HHTCCCHHHHHHHHHHHHHHHHHHHTTSSSTTHHHHSSTTHHHHHHHHHHHHHHHHHHHHHHSCHHHHHHHC-------- |
|  | T ss\_pred |  | HhcCCChHHHHHHHHHHHHHHHHHHhccccCCcccCCHHHHHHHHHHHHHHHHHHHHHHHHcCCHHHHHHHHHHHHHHHH |
|  |
|  |
|  | Q ss\_pred |  | HHHHHHHHHHHHHHHhcCCCCccccCCCCcccchhHHHHHHHHHHHHHHHHHHHHHhHHhhccCCCCCCccccchhhHHH |
|  | Q XP\_016875493.1 | 268 | VLSYVLVILYFRLWIMGGSMPLFSEQDNPASFSPYILTRFLTYSYLLAFNVWLLLAPVTLCYDWQVGSIPLVETIWDMRN   347 (456) |
|  | Q Consensus | 268 | ~~~~~~~~~~~~~~~~~~~~~~~~~~~~~~~~~~~~~~~~~~~~~~~~~~~~~~~~~~~~~~~~~~~~~~~~~~~~~~~~   347 (456) |
|  |  |  | ..+................................ ............... |
|  | T Consensus | 308 | ~~~~~~~~~~~~~~~~~~~~~~~~~~~~~~~~~~~------------------------------~~~~~~~~~~~~~~~   357 (705) |
|  | T 6S7O\_A | 308 | FVLLTVGALLMLTGKISPWTGRFYSLLDPSYAKNN------------------------------IPIIASVSEHQPTTW   357 (705) |
|  | T ss\_dssp |  | --------------CCCCCCSTTHHHHSTTHHHHT------------------------------CTTTTTSGGGSCCCH |
|  | T ss\_pred |  | HHHHHHHHHHHHccccccccHHHHHhhChhHhcCC------------------------------CCceeeccccCCCCH |
|  |
|  |
|  | Q ss\_pred |  | HHHHHHHHHHHHHHHHHHHHHhcccchHHHHHHHHHHHHHhhHhccccCCCccccccccchHHHHHHHHHHHHHHHHHHH |
|  | Q XP\_016875493.1 | 348 | LATIFLAVVMALLSLHCLAAFKRLEHKEVLVGLLFLVFPFIPASNLFFRVGFVVAERVLYMPSMGYCILFVHGLSKLCTW   427 (456) |
|  | Q Consensus | 348 | ~~~~~~~~~~~~~~~~~~~~~~~~~~~~~~~~~~~~~~~~~~~~~~~~~~~~~~~~Ry~~~~~~~~~ll~~~~~~~~~~~   427 (456) |
|  |  |  | ............+.........+++++.....+++.+......... .||..+..|+++++++.++..+.++ |
|  | T Consensus | 358 | ~~~~~~~~~~~~l~~~~~~~~~~~~~~~~~~~~~~~~~~~~~~~~~---------~R~~~~~~p~~~l~~a~~l~~l~~~   428 (705) |
|  | T 6S7O\_A | 358 | SSYYFDLQLLVFMFPVGLYYCFSNLSDARIFIIMYGVTSMYFSAVM---------VRLMLVLAPVMCILSGIGVSQVLST   428 (705) |
|  | T ss\_dssp |  | HHHHHHCSSSGGGHHHHHHHHHHSCCTTHHHHHHHHHHHHHHHHHC---------SGGGGGGHHHHHHHHHHHHHHHHHH |
|  | T ss\_pred |  | HHHHHHHHHHHHHHHHHHHHHhcCCChhHHHHHHHHHHHHHHHHHh---------HHHHHHHHHHHHHHHHHHHHHHHHH |
|  |
|  |
|  | Q ss\_pred |  | HhHHhH----------------------HHHHHHHHHHHHHHHHHHHHhcC |
|  | Q XP\_016875493.1 | 428 | LNRCGA----------------------TTLIVSTVLLLLLFSWKTVKQNE   456 (456) |
|  | Q Consensus | 428 | ~~~~~~----------------------~~~~~~~~~~~~~~~~~~~~~~~   456 (456) |
|  |  |  | ..++.. .....++++++............ |
|  | T Consensus | 429 | ~~~~~~~~~~~~~~~~~~~~~~~~~~~~~~~~~~~~~~~~~~~~~~~~~~~   479 (705) |
|  | T 6S7O\_A | 429 | YMKNLDISRPDKKSKKQQDSTYPIKNEVASGMILVMAFFLITYTFHSTWVT   479 (705) |
|  | T ss\_dssp |  | HTTTSSSCC---------------CCHHHHHHHHHHHHHHHHHHHHHHHHC |
|  | T ss\_pred |  | HHhhcCCCCCCccCccccCCCCCcCHHHHHHHHHHHHHHHHHHHHhHhHHH |
|  |
| --- | | | |
|  | Template alignmentTemplate 3D StructurePDBe | | |
| 4. | 6EZN\_F Dolichyl-diphosphooligosaccharide--protein glycosyltransferase subunit 1 (E.C.2.4.99.18); OST complex, oligosaccharyltransferase, N-linked glycosylation; HET: PTY, BMA, CPL, MAN, NAG;{Saccharomyces cerevisiae (strain ATCC 204508 / S288c)}; Related PDB entries: 6C26\_A; Related PDB entries: 6C26\_A; Related PDB entries: 6C26\_A | | |
|  | Probability: 99.84%, E-value: 1.4e-17, Score: 162.23, Aligned cols: 407, Identities: 9%, Similarity: -0.073, | | |
|  |
|  | Q ss\_pred |  | CCCCCCCccchhhccHHHHHHHHHHHHHHHHHHhhhcCCCc---ccccHHHHHhCCCCCCCCCcccccccccccccCCCC |
|  | Q XP\_016875493.1 | 9 | GGGGDRTPSRRRGCGLAPAGAAALLAGASCLCYGRSLQGEF---VHDDVWAIVNNPDVRPGAPLRWGIFTNDFWGKGMAE   85 (456) |
|  | Q Consensus | 9 | ~~~~~~~~~~~~~~~~~~~~~~~~l~~~~~~~~~~~~~~~~---~~De~~~~~~a~~~~~~~~~~~~~~~~~~~~~~~~~   85 (456) |
|  |  |  | ++++++..............+++++++............+. ..||..|...++++.+++.. ......+........ |
|  | T Consensus | 1 | m~~~~~~~~~~~~~~~~~~~l~~~~~~~~~~~~~~~~~~~~~~~~~D~~~~~~~a~~~~~~g~~-~~~~~~~~~~~~~~g   79 (718) |
|  | T 6EZN\_F | 1 | MGSDRSCVLSVFQTILKLVIFVAIFGAAISSRLFAVIKFESIIHEFDPWFNYRATKYLVNNSFY-KFLNWFDDRTWYPLG   79 (718) |
|  | T ss\_dssp |  | -----CCSHHHHHHHHHHHHHHHHHHHHHHTTTTTTTTTCCCCCSSSHHHHHHHHHHHHHSCHH-HHHSCCCTTSSTTTC |
|  | T ss\_pred |  | CCcchhHHHHHHHHHHHHHHHHHHHHHHHHHHHHHHHHchhhhcccChHHHHHHHHHHHHcccH-HHhcccCCcccCCCC |
|  |
|  |
|  | Q ss\_pred |  | CCCcccccchHHHHHHHHHHH----hC---CCchHHHHHHHHHHHHHHHHHHHHHHHHhccchHHHHHHHHHHHHCcccH |
|  | Q XP\_016875493.1 | 86 | NTSHKSYRPLCVLTFKLNIFL----TG---MNPFYFHAVNIILHCLVTLVLMYTCDKTVFKNRGLAFVTALLFAVHPIHT   158 (456) |
|  | Q Consensus | 86 | ~~~~~~~~Pl~~~~~~~~~~l----~g---~~~~~~rl~~~l~~~~~~~~~~~l~~~~~~~~~~~a~~aa~l~~~~p~~~   158 (456) |
|  |  |  | ......++|++.++.+..+.+ +| ......|+.++++++++++++|.++|+.. ++..|++++++++++|.+. |
|  | T Consensus | 80 | ~~~~~~~~p~~~~l~a~~~~l~~~~~G~~~~~~~~~~~~~~l~~~l~~~~~y~l~~~l~--~~~~a~~aa~l~~~~p~~~   157 (718) |
|  | T 6EZN\_F | 80 | RVTGGTLYPGLMTTSAFIWHALRNWLGLPIDIRNVCVLFAPLFSGVTAWATYEFTKEIK--DASAGLLAAGFIAIVPGYI   157 (718) |
|  | T ss\_dssp |  | CCSSSSCCTTHHHHHHHHHHCCCCCSSCCCCHHHHHHBTHHHHHHHHHHHHHHHHHHHS--CHHHHHHHHHHHHHCHHHH |
|  | T ss\_pred |  | CCCCCCCChHHHHHHHHHHHHHHHHhCCCCCHHHHHHHHHHHHHHHHHHHHHHHHHHhc--ChHHHHHHHHHHHHHHHHH |
|  |
|  |
|  | Q ss\_pred |  | HHHHhhhhhHHHHHHHHHHHHHHHHHHHHhcCCCCCCCCcchhHHHHHHHHHHHHHHHHhHHHHHHHHHHHHHHHHHhcc |
|  | Q XP\_016875493.1 | 159 | EAVAGIVGRADVLACLLFLLAFLSYNRSLDQGCVGGSFPSTVSPFFLLLSLFLGTCAMLVKETGITVFGVCLVYDLFSLS   238 (456) |
|  | Q Consensus | 159 | ~~~~~~~~~~~~~~~~~~~l~~~~~~~~~~~~~~~~~~~~~~~~~~~~~~~~~~~la~~~k~~~~~~~~~~~~~~~~~~~   238 (456) |
|  |  |  | ..+......+|.+..++.+++++++.+..+++ +.++.++++++++++.++|+.+.++.+++.+..+.... |
|  | T Consensus | 158 | ~~~~~g~~~~~~~~~~~~~~~l~~~~~~~~~~----------~~~~~~l~gl~~~l~~~~~~~~~~~~~~~~~~~~~~~~   227 (718) |
|  | T 6EZN\_F | 158 | SRSVAGSYDNEAIAITLLMVTFMFWIKAQKTG----------SIMHATCAALFYFYMVSAWGGYVFITNLIPLHVFLLIL   227 (718) |
|  | T ss\_dssp |  | SSSCSBCCSSSTTTHHHHHHHHHHHHHHHHHC----------CHHHHHHHHHHHHHHHTTCTTGGGGGGTHHHHHHHHHH |
|  | T ss\_pred |  | HhHhccccchHHHHHHHHHHHHHHHHHHhhcC----------CHHHHHHHHHHHHHHHHhccchHHHHHHHHHHHHHHHH |
|  |
|  |
|  | Q ss\_pred |  | cccchhHhhccCchhHHHhhHHHHHHHHHHHHHHHHHHHHHHHHhcCCCCccccCCCCcccchhHHHHHH---------- |
|  | Q XP\_016875493.1 | 239 | NKQDKSYLRASSNRNFLLTMRPFLKRAILVLSYVLVILYFRLWIMGGSMPLFSEQDNPASFSPYILTRFL----------   308 (456) |
|  | Q Consensus | 239 | ~~~~~~~~~~~~~~~~~~~~~~~~~~~~~~~~~~~~~~~~~~~~~~~~~~~~~~~~~~~~~~~~~~~~~~----------   308 (456) |
|  |  |  | .+++++... ..........+.......+........................... |
|  | T Consensus | 228 | ~~~~~~~~~---------------~~~~~~~~~~~~~~~~~p~~~~~~~~~~~~~~~~~~~~~~~~~~~~~~~~~~~~~~   292 (718) |
|  | T 6EZN\_F | 228 | MGRYSSKLY---------------SAYTTWYAIGTVASMQIPFVGFLPIRSNDHMAALGVFGLIQIVAFGDFVKGQISTA   292 (718) |
|  | T ss\_dssp |  | TTCCCHHHH---------------HHHHHHHHHHHHHTTCSTTSSSHHHHCTTSHHHHHHHHHHHHHHHHHHHHTTSCHH |
|  | T ss\_pred |  | ccCCChhHH---------------HHHHHHHHHHHHHHhcchhccCCccCchHHHHHHHHHHHHHHHHHHHHHHhcCChH |
|  |
|  |
|  | Q ss\_pred |  | ---------------------------HHHHHHHHHHHHHHHhHHhhccCCCCCCccccchhhHHHHHHHHHHHHHHHHH |
|  | Q XP\_016875493.1 | 309 | ---------------------------TYSYLLAFNVWLLLAPVTLCYDWQVGSIPLVETIWDMRNLATIFLAVVMALLS   361 (456) |
|  | Q Consensus | 309 | ---------------------------~~~~~~~~~~~~~~~~~~~~~~~~~~~~~~~~~~~~~~~~~~~~~~~~~~~~~   361 (456) |
|  |  |  | ...............+................................++++. |
|  | T Consensus | 293 | ~~~~~~~~~~~~~~~~~~~~~~~~~~~~~~~~~~~~~~~~~~~~~~~~~~~~~~~~~~~~~~~~~~~~~~~~~~~~l~~~   372 (718) |
|  | T 6EZN\_F | 293 | KFKVIMMVSLFLILVLGVVGLSALTYMGLIAPWTGRFYSLWDTNYAKIHIPIIASVSEHQPVSWPAFFFDTHFLIWLFPA   372 (718) |
|  | T ss\_dssp |  | HHTTTC-----------------------------------------------------CCCCHHHHHHHSSSTTTTHHH |
|  | T ss\_pred |  | HHHHHHHHHHHHHHHHHHHHHHHHHHhhhhhhhhHHHHHhhcccccccCCCcccchhHhCCCCHHHHHhhhhHHHHHHHH |
|  |
|  |
|  | Q ss\_pred |  | HHHHHHHhcccchHHHHHHHHHHHHHhhHhccccCCCccccccccchHHHHHHHHHHHHHHHHHHHHhHHh--------- |
|  | Q XP\_016875493.1 | 362 | LHCLAAFKRLEHKEVLVGLLFLVFPFIPASNLFFRVGFVVAERVLYMPSMGYCILFVHGLSKLCTWLNRCG---------   432 (456) |
|  | Q Consensus | 362 | ~~~~~~~~~~~~~~~~~~~~~~~~~~~~~~~~~~~~~~~~~~Ry~~~~~~~~~ll~~~~~~~~~~~~~~~~---------   432 (456) |
|  |  |  | ......+++++................... .||..+..|+++++++.++..+.++.+++. |
|  | T Consensus | 373 | g~~~~~~~~~~~~~~~~~~~~~~~~~~~~~-----------~R~~~~~~p~~~il~a~~l~~l~~~~~~~~~~~~~~~~~   441 (718) |
|  | T 6EZN\_F | 373 | GVFLLFLDLKDEHVFVIAYSVLCSYFAGVM-----------VRLMLTLTPVICVSAAVALSKIFDIYLDFKTSDRKYAIK   441 (718) |
|  | T ss\_dssp |  | HHHHHHTTCCSSHHHHHHHHHHHHHHHHHC-----------STTHHHHHHHHHHHHHHHHHHHHHHSCCCC-------CC |
|  | T ss\_pred |  | HHHHHHhcCChHHHHHHHHHHHHHHHHHHH-----------HHHHHHHHHHHHHHHHHHHHHHHHHHhccccCCcccccc |
|  |
|  |
|  | Q ss\_pred |  | ----HHHHHHHHHHHHHHHHHHHHHh |
|  | Q XP\_016875493.1 | 433 | ----ATTLIVSTVLLLLLFSWKTVKQ   454 (456) |
|  | Q Consensus | 433 | ----~~~~~~~~~~~~~~~~~~~~~~   454 (456) |
|  |  |  | ......++++++.......... |
|  | T Consensus | 442 | ~~~~~~~~~~~~~~~~~~~~~~~~~~   467 (718) |
|  | T 6EZN\_F | 442 | PAALLAKLIVSGSFIFYLYLFVFHST   467 (718) |
|  | T ss\_dssp |  | HHHHHHHHHHHHHHHHHHHHHHHHHH |
|  | T ss\_pred |  | chHHHHHHHHHHHHHHHHHHHHHHHH |
|  |
| --- | | | |
|  | Template alignmentTemplate 3D StructurePDBe | | |
| 5. | 3WAJ\_A Transmembrane oligosaccharyl transferase (E.C.2.4.1.119); oligosaccharyltransferase, N-glycosylation, Archaeoglobus fulgidus, GT-C; 2.501A {Archaeoglobus fulgidus}; Related PDB entries: 5GMY\_A 3WAK\_A; Related PDB entries: 5GMY\_A 3WAK\_A; Related PDB entries: 5GMY\_A 3WAK\_A | | |
|  | Probability: 99.84%, E-value: 2.6e-17, Score: 163.64, Aligned cols: 408, Identities: 12%, Similarity: -0.022, | | |
|  |
|  | Q ss\_pred |  | CCCCccchhhccHHHHHHHHHHHHHHHHHH------hhhcCCCcccccHHHHHhCCCCCCCCCcccccccccccccCCCC |
|  | Q XP\_016875493.1 | 12 | GDRTPSRRRGCGLAPAGAAALLAGASCLCY------GRSLQGEFVHDDVWAIVNNPDVRPGAPLRWGIFTNDFWGKGMAE   85 (456) |
|  | Q Consensus | 12 | ~~~~~~~~~~~~~~~~~~~~~l~~~~~~~~------~~~~~~~~~~De~~~~~~a~~~~~~~~~~~~~~~~~~~~~~~~~   85 (456) |
|  |  |  | ++......+.......+++++++.+..... ..........||..|...++++.+++...........+... |
|  | T Consensus | 1 | ~~~~~~~~~~~~~~~~l~~l~~~~~~lr~~~~~~~~~~~~~~~~~~D~~~~~~~a~~~~~~~~~~~~~d~~~~~p~g---   77 (875) |
|  | T 3WAJ\_A | 1 | MQNAESWFKKYWHLSVLVIAALISVKLRILNPWNSVFTWTVRLGGNDPWYYYRLIENTIHNFPHRIWFDPFTYYPYG---   77 (875) |
|  | T ss\_dssp |  | --------------CTTTHHHHHHHCCCCCTTHHHHBSSSBCCCSSHHHHHHHHHHHHHHTTTCCCSEETTSTTTTC--- |
|  | T ss\_pred |  | CcchHHHHhhHHHHHHHHHHHHHHHHHHhhCccccccCCceecccCChHHHHHHHHHHHHHCcccCCCCchhcCCCC--- |
|  |
|  |
|  | Q ss\_pred |  | CCCcccccchHHHHHHHHHHHhCCC-----chHHHHHHHHHHHHHHHHHHHHHHHHhccchHHHHHHHHHHHHCcc-cHH |
|  | Q XP\_016875493.1 | 86 | NTSHKSYRPLCVLTFKLNIFLTGMN-----PFYFHAVNIILHCLVTLVLMYTCDKTVFKNRGLAFVTALLFAVHPI-HTE   159 (456) |
|  | Q Consensus | 86 | ~~~~~~~~Pl~~~~~~~~~~l~g~~-----~~~~rl~~~l~~~~~~~~~~~l~~~~~~~~~~~a~~aa~l~~~~p~-~~~   159 (456) |
|  |  |  | ....++|++.++.+....++|.+ ....|++++++++++++++|.++|+.. +++.|++++++++++|. ... |
|  | T Consensus | 78 | --~~~~~~Pl~~~l~a~~~~l~G~~~~~~~~~~~~l~~~l~~~l~~~~~y~l~r~l~--~~~~allaall~a~~p~~~~~   153 (875) |
|  | T 3WAJ\_A | 78 | --SYTHFGPFLVYLGSIAGIIFSATSGESLRAVLAFIPAIGGVLAILPVYLLTREVF--DKRAAVIAAFLIAIVPGQFLQ   153 (875) |
|  | T ss\_dssp |  | --EECCSCHHHHHHHHHHHHHTTCCSHHHHHHHHHHHHHHHHHTTHHHHHHHHHHHS--CHHHHHHHHHHHTTCCSHHHH |
|  | T ss\_pred |  | --cCCCchhHHHHHHHHHHHHHcCCChHHHHHHHHHHHHHHHHHHHHHHHHHHHHHc--ChHHHHHHHHHHHHcchHHHH |
|  |
|  |
|  | Q ss\_pred |  | HHHhhhhhHHHHHHHHHHHHHHHHHHHHh------------cCCCCCCCCcchhHHHHHHHHHHHHHHHHhHHHHHHHHH |
|  | Q XP\_016875493.1 | 160 | AVAGIVGRADVLACLLFLLAFLSYNRSLD------------QGCVGGSFPSTVSPFFLLLSLFLGTCAMLVKETGITVFG   227 (456) |
|  | Q Consensus | 160 | ~~~~~~~~~~~~~~~~~~l~~~~~~~~~~------------~~~~~~~~~~~~~~~~~~~~~~~~~la~~~k~~~~~~~~   227 (456) |
|  |  |  | .+......+|.+..++.+++++++.+..+ ++ +.++.++++++++++.++|..+.++.+ |
|  | T Consensus | 154 | ~s~~g~~~~~~~~~~~~~l~l~~~~~~~~~~~~~~~~~~~~~~----------~~~~~~l~gl~~gl~~lt~~~~~~~~~   223 (875) |
|  | T 3WAJ\_A | 154 | RSILGFNDHHIWEAFWQVSALGTFLLAYNRWKGHDLSHNLTAR----------QMAYPVIAGITIGLYVLSWGAGFIIAP   223 (875) |
|  | T ss\_dssp |  | TTSTTCCCSHHHHHHHHHHHHHHHHHHHTTSSSCCC----CTT----------TSHHHHHHHHHHHHHHHHCGGGGGHHH |
|  | T ss\_pred |  | HHhccccchHHHHHHHHHHHHHHHHHHHHHhccCCCCcccccc----------hhHHHHHHHHHHHHHHHHhchHHHHHH |
|  |
|  |
|  | Q ss\_pred |  | HHHHHHHHhcccccchhHhhccCchhHHHhhHHHHHHHHHHHHHHHHHHHHHHHHhcCCCCccccCCCCcccchhHHHHH |
|  | Q XP\_016875493.1 | 228 | VCLVYDLFSLSNKQDKSYLRASSNRNFLLTMRPFLKRAILVLSYVLVILYFRLWIMGGSMPLFSEQDNPASFSPYILTRF   307 (456) |
|  | Q Consensus | 228 | ~~~~~~~~~~~~~~~~~~~~~~~~~~~~~~~~~~~~~~~~~~~~~~~~~~~~~~~~~~~~~~~~~~~~~~~~~~~~~~~~   307 (456) |
|  |  |  | +++++.+.....++.++..+ +.........+++.++................................. |
|  | T Consensus | 224 | ~~~~~~~~~~~~~~~~~~~~-----------~~~~~~~~~~~~~~~l~~~p~~~~~~~~~~~~~~~~~~~~~~~~~~~~~   292 (875) |
|  | T 3WAJ\_A | 224 | IILAFMFFAFVLAGFVNADR-----------KNLSLVAVVTFAVSALIYLPFAFNYPGFSTIFYSPFQLLVLLGSAVIAA   292 (875) |
|  | T ss\_dssp |  | HHHHHHHHHHHTTTTCCCCH-----------HHHHHHHHHHHHHHHHHHGGGTTSSSSCCSSSSCHHHHHHHHHHHHHHH |
|  | T ss\_pred |  | HHHHHHHHHHHHHHhCCCCC-----------HhHHHHHHHHHHHHHHHHHHHhcCCCCCChhcccHHHHHHHHHHHHHHH |
|  |
|  |
|  | Q ss\_pred |  | HHHHHHH------------------------------------HHHHHHHHHhHHhhccCCCCCCccccchh-------- |
|  | Q XP\_016875493.1 | 308 | LTYSYLL------------------------------------AFNVWLLLAPVTLCYDWQVGSIPLVETIW--------   343 (456) |
|  | Q Consensus | 308 | ~~~~~~~------------------------------------~~~~~~~~~~~~~~~~~~~~~~~~~~~~~--------   343 (456) |
|  |  |  | ....... ............................. |
|  | T Consensus | 293 | ~~~~~~~~~~~~~~~~~~~~~~~~~~~~~~~~~~~~~~~~~~~~~~~~~~~~~~~~~~~~~~~~~i~e~~~~~~~~~~~~   372 (875) |
|  | T 3WAJ\_A | 293 | AFYQIEKWNDVGFFERVGLGRKGMPLAVIVLTALIMGLFFVISPDFARNLLSVVRVVQPKGGALTIAEVYPFFFTHNGEF   372 (875) |
|  | T ss\_dssp |  | HHHHHHHHHHHTHHHHTTCGGGHHHHHHHHHHHHHHHHHHC--------------------------------------- |
|  | T ss\_pred |  | HHHHHHHhcCcchHHhcCCCccchHHHHHHHHHHHHHHHHHHCCHHHHHHHhhcceecCCCCceeeeeccccccccCCCc |
|  |
|  |
|  | Q ss\_pred |  | --hHHHHHHHHHHHHHHHHHHHHHHHHhcccchHHHHHHHHHHHHHhhHhccccCCCccccccccchHHHHHHHHHHHHH |
|  | Q XP\_016875493.1 | 344 | --DMRNLATIFLAVVMALLSLHCLAAFKRLEHKEVLVGLLFLVFPFIPASNLFFRVGFVVAERVLYMPSMGYCILFVHGL   421 (456) |
|  | Q Consensus | 344 | --~~~~~~~~~~~~~~~~~~~~~~~~~~~~~~~~~~~~~~~~~~~~~~~~~~~~~~~~~~~~Ry~~~~~~~~~ll~~~~~   421 (456) |
|  |  |  | ...........++.++........+++++.....+.+++.+..+...... .||..+..|+++++++.++ |
|  | T Consensus | 373 | ~~~~~~~~~~~~~~l~~~~~~~~~~~~~~~~~~~~~~l~~~~~~~~~~~~~~---------~Ry~~~~~p~~~il~a~~l   443 (875) |
|  | T 3WAJ\_A | 373 | TLTNAVLHFGALFFFGMAGILYSAYRFLKRRSFPEMALLIWAIAMFIALWGQ---------NRFAYYFAAVSAVYSALAL   443 (875) |
|  | T ss\_dssp |  | -CTHHHHHHTTHHHHHHHHHHHHHHHHHHHCCHHHHHHHHHHHHHHHHTSSC---------GGGTHHHHHHHHHHHHHHH |
|  | T ss\_pred |  | cHHHHHHHhHHHHHHHHHHHHHHHHHHHccCCchhHHHHHHHHHHHHHHHHh---------hhHHHHHHHHHHHHHHHHH |
|  |
|  |
|  | Q ss\_pred |  | HHHHHHH------------hHHhHHHHHHHHHHHHHHHHHHHHHhcC |
|  | Q XP\_016875493.1 | 422 | SKLCTWL------------NRCGATTLIVSTVLLLLLFSWKTVKQNE   456 (456) |
|  | Q Consensus | 422 | ~~~~~~~------------~~~~~~~~~~~~~~~~~~~~~~~~~~~~   456 (456) |
|  |  |  | ..+.++. +++.......+++++++........... |
|  | T Consensus | 444 | ~~l~~~~~~~~~~~~~~~~~~~~~~~~~~~~~~~~~~~~~~~~~~~~   490 (875) |
|  | T 3WAJ\_A | 444 | SVVFDKLHLYRALENAIGARNKLSYFRVAFALLIALAAIYPTYILAD   490 (875) |
|  | T ss\_dssp |  | HHHGGGCC-----------------CCTTTHHHHHHHHHHHHHHHHH |
|  | T ss\_pred |  | HHHHHHHhHHHHHHhhhchhccchHHHHHHHHHHHHHHHHHHHHHHH |
|  |
| --- | | | |
|  | Template alignmentTemplate 3D StructurePDBe | | |
| 6. | 5OGL\_A Peptide-binding protein, Substrate mimicking peptide; Oligosaccharyltransferase, Complex, Protein N-glycosylation, Bacteria; HET: 9UB, PPN; 2.7A {Campylobacter lari (strain RM2100 / D67 / ATCC BAA-1060)}; Related PDB entries: 3RCE\_A 6GXC\_A; Related PDB entries: 6GXC\_A 3RCE\_A ; Related PDB entries: 6GXC\_A 3RCE\_A | | |
|  | Probability: 99.79%, E-value: 2e-16, Score: 153.73, Aligned cols: 374, Identities: 11%, Similarity: -0.029, | | |
|  |
|  | Q ss\_pred |  | CCCCCccchhhccHHHHHHHHHHHHHHHHHH--------------hhhcCCCcccccHHHHHhCCCCCCCCCcccccccc |
|  | Q XP\_016875493.1 | 11 | GGDRTPSRRRGCGLAPAGAAALLAGASCLCY--------------GRSLQGEFVHDDVWAIVNNPDVRPGAPLRWGIFTN   76 (456) |
|  | Q Consensus | 11 | ~~~~~~~~~~~~~~~~~~~~~~l~~~~~~~~--------------~~~~~~~~~~De~~~~~~a~~~~~~~~~~~~~~~~   76 (456) |
|  |  |  | ++.+.........+.....++++.++++.++ ..........|+..|...+++..++......... |
|  | T Consensus | 1 | m~~~~~~~~~~~~~~~~~~l~~i~~~~~~lRl~~~~~~~~~~~~~~~~~~~~~~~D~~~~~~~a~~~~~~~~~~~~~~~-   79 (713) |
|  | T 5OGL\_A | 1 | MELQQNFTDNNSIKYTAILILIAFAFSVLARLYWVAWASEFYEFFFNDQLMITTNDGYAFAEGARDMIAGFHQPNDLSY-   79 (713) |
|  | T ss\_dssp |  | -CCSSCCTTCCCHHHHHHHHHHHHHHHHHHHHHHHHHHTTCGGGEETTEECCSSTTHHHHHHHHHHHHHTCCCTTSCCC- |
|  | T ss\_pred |  | CccchhccccccHHHHHHHHHHHHHHHHHHHHHHHHHhhccchhhcCCEEeeccccHHHHHHHHHHHHcCCCCCCCcch- |
|  |
|  |
|  | Q ss\_pred |  | cccccCCCCCCCcccccchHHHHHHHHHHHhC-CCchHHHHHHHHHHHHHHHHHHHHHHHHhccchHHHHHHHHHHHHCc |
|  | Q XP\_016875493.1 | 77 | DFWGKGMAENTSHKSYRPLCVLTFKLNIFLTG-MNPFYFHAVNIILHCLVTLVLMYTCDKTVFKNRGLAFVTALLFAVHP   155 (456) |
|  | Q Consensus | 77 | ~~~~~~~~~~~~~~~~~Pl~~~~~~~~~~l~g-~~~~~~rl~~~l~~~~~~~~~~~l~~~~~~~~~~~a~~aa~l~~~~p   155 (456) |
|  |  |  | ++|++.++.+....++| ......|++++++++++++.+|.++|+.. +++.|++++++++++| |
|  | T Consensus | 80 | ---------------~~p~~~~l~~~~~~l~g~~~~~~~~~~~~l~~~l~v~~~y~l~r~l~--~~~~al~aall~a~~p   142 (713) |
|  | T 5OGL\_A | 80 | ---------------FGSSLSTLTYWLYSILPFSFESIILYMSTFFASLIVVPIILIAREYK--LTTYGFIAALLGSIAN   142 (713) |
|  | T ss\_dssp |  | ---------------TTCHHHHHHHHHHHHSCSCHHHHHHHHHHHHGGGGHHHHHHHHHHTT--CHHHHHHHHHHHHHCH |
|  | T ss\_pred |  | ---------------hcchHHHHHHHHHHhCCCCHHHHHHHHHHHHHHHHHHHHHHHHHHhC--CchHHHHHHHHHHHHH |
|  |
|  |
|  | Q ss\_pred |  | ccHHHHHhhhhhHHHHHHHHHHHHHHHHHHHHhcCCCCCCCCcchhHHHHHHHHHHHHHHHHhHHHHH----HHHHHHHH |
|  | Q XP\_016875493.1 | 156 | IHTEAVAGIVGRADVLACLLFLLAFLSYNRSLDQGCVGGSFPSTVSPFFLLLSLFLGTCAMLVKETGI----TVFGVCLV   231 (456) |
|  | Q Consensus | 156 | ~~~~~~~~~~~~~~~~~~~~~~l~~~~~~~~~~~~~~~~~~~~~~~~~~~~~~~~~~~la~~~k~~~~----~~~~~~~~   231 (456) |
|  |  |  | .++..+......+|.+..++.+++++++.+..+++ +.++.++++++.+++.++|..+. .+++...+ |
|  | T Consensus | 143 | ~~~~~s~~g~~~~d~~~~~~~~l~~~~~~~~~~~~----------~~~~~~l~gl~~~l~~~~~~~~~~~~~~~~~~~~~   212 (713) |
|  | T 5OGL\_A | 143 | SYYNRTMSGYYDTDMLVLVLPMLILLTFIRLTINK----------DIFTLLLSPVFIMIYLWWYPSSYSLNFAMIGLFGL   212 (713) |
|  | T ss\_dssp |  | HHHHTTSTTCCSGGGGTTHHHHHHHHHHHHHHHHC----------CTTHHHHHHHHHHHHHHHCGGGHHHHHHHHHHHHH |
|  | T ss\_pred |  | HHHHhhccccCchHHHHHHHHHHHHHHHHHHHcCC----------chHHHHHHHHHHHHHHhhccchHHHHHHHHHHHHH |
|  |
|  |
|  | Q ss\_pred |  | HHHHhcccccc---------------------------------hhHhhccCchhHHHhhHHHHHHHHHHHHHHHHHHHH |
|  | Q XP\_016875493.1 | 232 | YDLFSLSNKQD---------------------------------KSYLRASSNRNFLLTMRPFLKRAILVLSYVLVILYF   278 (456) |
|  | Q Consensus | 232 | ~~~~~~~~~~~---------------------------------~~~~~~~~~~~~~~~~~~~~~~~~~~~~~~~~~~~~   278 (456) |
|  |  |  | +.+...+++++ ++... +.........+......... |
|  | T Consensus | 213 | ~~~~~~~~~~~~~~~~~~~~~~~~~~~~~~~~~~~~~~~~~~~~~~~~~-----------~~~~~~~~~~~~~~~~~~~~   281 (713) |
|  | T 5OGL\_A | 213 | YTLVFHRKEKIFYLTIALMIIALSMLAWQYKLALIVLLFAIFAFKEEKI-----------NFYMIWALIFISILILHLSG   281 (713) |
|  | T ss\_dssp |  | HHHHHTTTCHHHHHHHHHHHHHHSCCCHHHHHHHHHHHHHHHHHCSSCC-----------CHHHHHHHHHHHHHHHHHTT |
|  | T ss\_pred |  | HHHHhCCcchHHHHHHHHHHHHHhhhhHHHHHHHHHHHHHHHHcchhcc-----------chHHHHHHHHHHHHHHHHhc |
|  |
|  |
|  | Q ss\_pred |  | HHHHhcCCCCccccCCCCcccchhHHHHHHHHHHHHHHHHHHHHHhHHhhccCCCCCCccccchhhHHHHHHHHHHHHHH |
|  | Q XP\_016875493.1 | 279 | RLWIMGGSMPLFSEQDNPASFSPYILTRFLTYSYLLAFNVWLLLAPVTLCYDWQVGSIPLVETIWDMRNLATIFLAVVMA   358 (456) |
|  | Q Consensus | 279 | ~~~~~~~~~~~~~~~~~~~~~~~~~~~~~~~~~~~~~~~~~~~~~~~~~~~~~~~~~~~~~~~~~~~~~~~~~~~~~~~~   358 (456) |
|  |  |  | .....................................+.-. .................++ |
|  | T Consensus | 282 | ~~~~~~~~~~~~~~~~~~~~~~~~~~~~~~~~~~~~~~~~~--------------------~~~~~~~~~~~~~~~~~~l   341 (713) |
|  | T 5OGL\_A | 282 | GLDPVLYQLKFYVFKASDVQNLKDAAFMYFNVNETIMEVNT--------------------IDPEVFMQRISSSVLVFIL   341 (713) |
|  | T ss\_dssp |  | TTHHHHHHHHHHTSCCSCCSCCTTTSCCCCCGGGGBGGGCC--------------------CCHHHHHHHHHSSHHHHHH |
|  | T ss\_pred |  | cchHHHHHHHHHhhcccccccccccccccccHHHHHHHhcC--------------------CCHHHHHHHhcccHHHHHH |
|  |
|  |
|  | Q ss\_pred |  | HHHHHHHHHHhcccchHHHHHHHHHHHHHhhHhccccCCCccccccccchHHHHHHHHHHHHHHHHHHHHhHHhH----- |
|  | Q XP\_016875493.1 | 359 | LLSLHCLAAFKRLEHKEVLVGLLFLVFPFIPASNLFFRVGFVVAERVLYMPSMGYCILFVHGLSKLCTWLNRCGA-----   433 (456) |
|  | Q Consensus | 359 | ~~~~~~~~~~~~~~~~~~~~~~~~~~~~~~~~~~~~~~~~~~~~~Ry~~~~~~~~~ll~~~~~~~~~~~~~~~~~-----   433 (456) |
|  |  |  | .+.......++++. ....+.++++.+...... .||..+..|+++++++.++..+.++.+++.. |
|  | T Consensus | 342 | ~~~gl~~~~~~~~~---~~~~l~~~~~~~~~~~~~---------~R~~~~~~p~~~i~~a~~l~~l~~~~~~~~~~~~~~   409 (713) |
|  | T 5OGL\_A | 342 | SFIGFILLLKDHKS---MLLALPMLALGFMALRAG---------LRFTIYAVPVMALGFGYFLYAFFNFLEKKQIKLSLR   409 (713) |
|  | T ss\_dssp |  | HHHHHHHHHTTCGG---GGGGHHHHHHHHHHHHHC---------GGGGGGGHHHHHHHHHHHHHHHHHHHHHTTCCCCHH |
|  | T ss\_pred |  | HHHHHHHHHHcCHh---HHHHHHHHHHHHHHHHHh---------HHHHHHHHHHHHHHHHHHHHHHHHHHHHhhcccchh |
|  |
|  |
|  | Q ss\_pred |  | -HHHHHHHHHHHHHHHHHHHHhc |
|  | Q XP\_016875493.1 | 434 | -TTLIVSTVLLLLLFSWKTVKQN   455 (456) |
|  | Q Consensus | 434 | -~~~~~~~~~~~~~~~~~~~~~~   455 (456) |
|  |  |  | +....++++++..........+ |
|  | T Consensus | 410 | ~~~~~~~~~~~~~~~~~~~~~~~   432 (713) |
|  | T 5OGL\_A | 410 | NKNILLILIAFFSISPALMHIYY   432 (713) |
|  | T ss\_dssp |  | HHHHHHHHHHHHHHHHHHHHHHH |
|  | T ss\_pred |  | HHHHHHHHHHHHHHhHHHHHHHh |
|  |
| --- | | | |
|  | Template alignmentTemplate 3D StructurePDBe | | |
| 7. | 6P25\_A Dolichyl-diphosphooligosaccharide--protein glycosyltransferase subunits (E.C.2.4.99.18); complex, TRANSFERASE, glycosylation; HET: NAG, CPL, NNM; 3.2A {Saccharomyces cerevisiae W303}; Related PDB entries: 6P2R\_A ; Related PDB entries: 6P2R\_A ; Related PDB entries: 6P2R\_A | | |
|  | Probability: 99.75%, E-value: 5.7e-14, Score: 136.69, Aligned cols: 402, Identities: 12%, Similarity: -0.049, | | |
|  |
|  | Q ss\_pred |  | CceecccCCCCCCCCccchhhccHHHHHHHHHHHHHHHHHHhhhcCCCcccccHHHHHhCCCCCCCCCcccccccccccc |
|  | Q XP\_016875493.1 | 1 | MVVTTSARGGGGDRTPSRRRGCGLAPAGAAALLAGASCLCYGRSLQGEFVHDDVWAIVNNPDVRPGAPLRWGIFTNDFWG   80 (456) |
|  | Q Consensus | 1 | m~~~~~~~~~~~~~~~~~~~~~~~~~~~~~~~l~~~~~~~~~~~~~~~~~~De~~~~~~a~~~~~~~~~~~~~~~~~~~~   80 (456) |
|  |  |  | ...........................+.+++++.+....+.........+||..+...+..+.+++. |
|  | T Consensus | 26 | ~~~~~~~~~~~~~~~~~~~~~~~~~~~l~~l~ll~~~lrl~~l~~~~~~~~DE~~~~~~a~~~~~g~~------------   93 (817) |
|  | T 6P25\_A | 26 | VRPFIVTDPSAELASLRTMVTLKEKLLVACLAVFTAVIRLHGLAWPDSVVFDEVHFGGFASQYIRGTY------------   93 (817) |
|  | T ss\_dssp |  | SSEEECCCCCHHHHHHSSCCSHHHHHHHHHHHHHHHHHTTTTTTSSCBCCTTHHHHHHHHHHHHHCBC------------ |
|  | T ss\_pred |  | CcCcccCCccHHHHhhhccCcHHHHHHHHHHHHHHHHHHHhcccCCCcceeeHHHHHHHHHHHHhCCC------------ |
|  |
|  |
|  | Q ss\_pred |  | cCCCCCCCcccccchHHHHHHHHHHHhCCCc-----------------hHHHHHHHHHHHHHHHHHHHHHHHHhccchHH |
|  | Q XP\_016875493.1 | 81 | KGMAENTSHKSYRPLCVLTFKLNIFLTGMNP-----------------FYFHAVNIILHCLVTLVLMYTCDKTVFKNRGL   143 (456) |
|  | Q Consensus | 81 | ~~~~~~~~~~~~~Pl~~~~~~~~~~l~g~~~-----------------~~~rl~~~l~~~~~~~~~~~l~~~~~~~~~~~   143 (456) |
|  |  |  | +.+.+||++.++.+....++|.+. ...|++++++++++++++|.++ |....++.. |
|  | T Consensus | 94 | -------~~~~~PPL~~ll~a~~~~l~G~~~~~~f~~ig~~~~~~~~~~~~Rl~~~l~~~l~v~l~y~i~-r~l~~~~~~   165 (817) |
|  | T 6P25\_A | 94 | -------FMDVHPPLAKMLYAGVASLGGFQGDFDFENIGDSFPSTTPYVLMRFFSASLGALTVILMYMTL-RYSGVRMWV   165 (817) |
|  | T ss\_dssp |  | -------CCCSSCTHHHHHHHHHHHHTCCCSCCCCCSTTCBCCTTSCCHHHHHHHHHHHHHHHHHHHHHH-HHTTCCHHH |
|  | T ss\_pred |  | -------CCCCCChHHHHHHHHHHHHcCCCCCCCccccccCCCCCCHHHHHHHHHHHHHHHHHHHHHHHH-HHcCCCHHH |
|  |
|  |
|  | Q ss\_pred |  | HHHHHHHHHHCcccHHHHHhhhhhHHHHHHHHHHHHHHHHHHHHhcCCCCCCCCcchh-----HHHHHHHHHHHHHHHHh |
|  | Q XP\_016875493.1 | 144 | AFVTALLFAVHPIHTEAVAGIVGRADVLACLLFLLAFLSYNRSLDQGCVGGSFPSTVS-----PFFLLLSLFLGTCAMLV   218 (456) |
|  | Q Consensus | 144 | a~~aa~l~~~~p~~~~~~~~~~~~~~~~~~~~~~l~~~~~~~~~~~~~~~~~~~~~~~-----~~~~~~~~~~~~la~~~   218 (456) |
|  |  |  | |++++++++++|..+.. ....+.|.+..++++++++++.+..+++ + +++++++|+++++++.+ |
|  | T Consensus | 166 | Allaall~~~~p~~i~~--s~~~~~d~~~~ff~~lal~~~~~~~~~~----------~~~~~~~~~l~l~gl~lgla~~t   233 (817) |
|  | T 6P25\_A | 166 | ALMSAICFAVENSYVTI--SRYILLDAPLMFFIAAAVYSFKKYEMYP----------ANSLNAYKSLLATGIALGMASSS   233 (817) |
|  | T ss\_dssp |  | HHHHHHHHHSCHHHHHH--HHSSCSHHHHHHHHHHHHHHHHHHHTSC----------SSSHHHHHHHHHHHHHHHHHHTT |
|  | T ss\_pred |  | HHHHHHHHHHcHHHHHH--HHHHhhHHHHHHHHHHHHHHHHHHHhCC----------CCCcHHHHHHHHHHHHHHHHHhh |
|  |
|  |
|  | Q ss\_pred |  | HHHHHHHHHHHHHHHHHh------cccccchhHhhccCchhHHHhhHHHHHHHHHHHHHHHHHHHHHHHHhcCCCCcccc |
|  | Q XP\_016875493.1 | 219 | KETGITVFGVCLVYDLFS------LSNKQDKSYLRASSNRNFLLTMRPFLKRAILVLSYVLVILYFRLWIMGGSMPLFSE   292 (456) |
|  | Q Consensus | 219 | k~~~~~~~~~~~~~~~~~------~~~~~~~~~~~~~~~~~~~~~~~~~~~~~~~~~~~~~~~~~~~~~~~~~~~~~~~~   292 (456) |
|  |  |  | |+.++++++.++++.+.. ..+++.+...+ ......++.+++....+...+......... |
|  | T Consensus | 234 | K~~gl~~l~~~~l~~l~~l~~~~~~~~~~~~~~~~---------------~~~~~~~~li~ip~~iy~~~~~~~f~~l~~   298 (817) |
|  | T 6P25\_A | 234 | KWVGLFTVTWVGLLCIWRLWFMIGDLTKSSKSIFK---------------VAFAKLAFLLGVPFALYLVFFYIHFQSLTL   298 (817) |
|  | T ss\_dssp |  | CTTHHHHHHHHHHHHHHHHHHHHHCSSSCHHHHHH---------------HHHHHHCCCCCHHHHHHHHHHHHHHHHCCB |
|  | T ss\_pred |  | hhHHHHHHHHHHHHHHHHHHHHhCCCCCCHHHHHH---------------HHHHHHHHHHHHHHHHHHHHHHHHHHHhcc |
|  |
|  |
|  | Q ss\_pred |  | CCCCcccchhHHHHHH---------------------------------------------------------------- |
|  | Q XP\_016875493.1 | 293 | QDNPASFSPYILTRFL----------------------------------------------------------------   308 (456) |
|  | Q Consensus | 293 | ~~~~~~~~~~~~~~~~----------------------------------------------------------------   308 (456) |
|  |  |  | ......+.+....... |
|  | T Consensus | 299 | ~g~~~~~~s~~f~~~L~g~~~~~~~~~~v~~Gs~itlr~~~~~~gyLhSh~~~yp~gs~qqqvt~y~~~d~nn~w~i~~~   378 (817) |
|  | T 6P25\_A | 299 | DGDGASFFSPEFRSTLKNNKIPQNVVADVGIGSIISLRHLSTMGGYLHSHSHNYPAGSEQQQSTLYPHMDANNDWLLELY   378 (817) |
|  | T ss\_dssp |  | CCSTTTTSCTTTGGGSBSCCSCCSEESBCBTTCEEEEEESSSSSCCEEEEEEECSSTTCCEEEEECCSCCGGGCEEEEEC |
|  | T ss\_pred |  | CCCCcccCCHHHHHHhcCCCCCCCceeeeecCceEEEEEccCCCCeeeeecccCCCCCCCccceeecccCCCCcEEEEEC |
|  |
|  |
|  | Q ss\_pred |  | -------------------------------------------------------------------------------- |
|  | Q XP\_016875493.1 | 309 | --------------------------------------------------------------------------------   308 (456) |
|  | Q Consensus | 309 | --------------------------------------------------------------------------------   308 (456) |
|  |  |  | ...+... |
|  | T Consensus | 379 | ~~~~~~~~~~~~v~~g~~irL~h~~t~~~L~sh~~~~pvs~~~~~~~evs~~g~~~~~gd~~d~w~v~i~~~~~~~~~~~   458 (817) |
|  | T 6P25\_A | 379 | NAPGESLTTFQNLTDGTKVRLFHTVTRCRLHSHDHKPPVSESSDWQKEVSCYGYSGFDGDANDDWVVEIDKKNSAPGVAQ   458 (817) |
|  | T ss\_dssp |  | C----CCSCCCBCCTTEEEEEEETTTTBCCBCCSCCCSSCCSCSSCEECBBCCBTTTBCCGGGCEEEEECTTTSCSSHHH |
|  | T ss\_pred |  | CCCCCCCCCceecCCCCEEEEEeCCCCCceeeCCCCCCCCCCCccccEEEeeccCCCCCCcccceEEEEecccCCCCccc |
|  |
|  |
|  | Q ss\_pred |  | -------------------------------------------------------------------------HHHHHHH |
|  | Q XP\_016875493.1 | 309 | -------------------------------------------------------------------------TYSYLLA   315 (456) |
|  | Q Consensus | 309 | -------------------------------------------------------------------------~~~~~~~   315 (456) |
|  |  |  | +.-..+..-... ...+-...........++..+......+++..+.......+.++... |
|  | T Consensus | 459 | ~~~~~~~t~frl~h~~~~c~L~s~~~~lP~wg~~q~EV~c~~~~~~~~~~w~ie~~~~~~~~~~~~~~~~~~~~f~~kf~   538 (817) |
|  | T 6P25\_A | 459 | ERVIALDTKFRLRHAMTGCYLFSHEVKLPAWGFEQQEVTCASSGRHDLTLWYVENNSNPLLPEDTKRISYKPASFISKFI   538 (817) |
|  | T ss\_dssp |  | HSCBTTTCCEEEEETTTCCBCEEEEEEECSSSTTEEEEEECSSBCSGGGCEEEEEEECSSSCSSCCEECCCCCCHHHHHH |
|  | T ss\_pred |  | ccEEEcCCEEEEEeCCCCeEEEECCCcCCcccccceeeeecCCCCCcCceEEEecCCCCCCCCCcccccCCCCcHHHHHH |
|  |
|  |
|  | Q ss\_pred |  | HHHHHHHHhHHh-------------------hccCCCCCCccccchhhHHHHHHHHHHHHHHHHHHHHHHHHhcccch-- |
|  | Q XP\_016875493.1 | 316 | FNVWLLLAPVTL-------------------CYDWQVGSIPLVETIWDMRNLATIFLAVVMALLSLHCLAAFKRLEHK--   374 (456) |
|  | Q Consensus | 316 | ~~~~~~~~~~~~-------------------~~~~~~~~~~~~~~~~~~~~~~~~~~~~~~~~~~~~~~~~~~~~~~~--   374 (456) |
|  |  |  | .....++..++..+|+... +.....++.+|++++.+++++..+..+.+...++.+......+++.+ |
|  | T Consensus | 539 | e~~~~m~~~n~~l~~~h~~~S~p~~Wp~~~r~i~~w~~~~~~i~~lgNp~~ww~~~~~~~~~~~~~~~~~~~~~~~~~~~   618 (817) |
|  | T 6P25\_A | 539 | ESHKKMWHINKNLVEPHVYESQPTSWPFLLRGISYWGENNRNVYLLGNAIVWWAVTAFIGIFGLIVITELFSWQLGKPIL   618 (817) |
|  | T ss\_dssp |  | HHHHHHHHHHHSCCCCCTTCCCGGGGGGTCSCEEEEEETTEEEEECCCHHHHHHHHHHHHHHHHHHHHHHHHHHHTCCCC |
|  | T ss\_pred |  | HHHHHHHHHHhhcCCCCcccCCccccceeecceeccCCCCceeEecccHHHHHHHHHHHHHHHHHHHHHHHHHHcCCCCC |
|  |
|  |
|  | Q ss\_pred |  | ----------HHHHHHHHHHHHHhhHhccccCCCccccccccchHHHHHHHHHHHHHHHHHHHHhHHhHHHHHHHHHHHH |
|  | Q XP\_016875493.1 | 375 | ----------EVLVGLLFLVFPFIPASNLFFRVGFVVAERVLYMPSMGYCILFVHGLSKLCTWLNRCGATTLIVSTVLLL   444 (456) |
|  | Q Consensus | 375 | ----------~~~~~~~~~~~~~~~~~~~~~~~~~~~~~Ry~~~~~~~~~ll~~~~~~~~~~~~~~~~~~~~~~~~~~~~   444 (456) |
|  |  |  | ++....... |
|  | T Consensus | 619 | ~~~~~~~~~~~~~~~~~gw~~hy~Pf~~~----~R~~fl~hYlpal~F~il~~~~~~~~~~~~~~~~~~~~~~~~~~~~~   694 (817) |
|  | T 6P25\_A | 619 | KDSKVVNFHVQVIHYLLGFAVHYAPSFLM----QRQMFLHHYLPAYYFGILALGHALDIIVSYVFRSKRQMGYAVVITFL   694 (817) |
|  | T ss\_dssp |  | CSHHHHHHHHHHHHHHHHHHHTTGGGTSC----CSCCCGGGSHHHHHHHHHHHHHHHHHHHHTTTSSCHHHHHHHHHHHH |
|  | T ss\_pred |  | CchhHHHHHHHHHHHHHHHHHHHHHHHHc----cCccchhhHHHHHHHHHHHHHHHHHHHHHHhccccchHHHHHHHHHH |
|  |
|  |
|  | Q ss\_pred |  | HHHHHHHHH |
|  | Q XP\_016875493.1 | 445 | LLFSWKTVK   453 (456) |
|  | Q Consensus | 445 | ~~~~~~~~~   453 (456) |
|  | T Consensus | 695 | ~~~~~~f~~   703 (817) |
|  | T 6P25\_A | 695 | AASVYFFKS   703 (817) |
|  | T ss\_dssp |  | HHHHHHHHH |
|  | T ss\_pred |  | HHHHHHHHH |
|  |
| --- | | | |
|  | Template alignmentTemplate 3D StructurePDBe | | |
| 8. | 6P25\_B Dolichyl-diphosphooligosaccharide--protein glycosyltransferase subunits (E.C.2.4.99.18); complex, TRANSFERASE, glycosylation; HET: NAG, CPL, NNM; 3.2A {Saccharomyces cerevisiae W303}; Related PDB entries: 6P2R\_B ; Related PDB entries: 6P2R\_B ; Related PDB entries: 6P2R\_B | | |
|  | Probability: 99.67%, E-value: 1.8e-14, Score: 140.45, Aligned cols: 259, Identities: 13%, Similarity: 0.067, | | |
|  |
|  | Q ss\_pred |  | CceecccCCCCCCCCccchhhccHHHHHHHHHHHHHHHHHHhhhcCCCcccccHHHHHhCCCCCCCCCcccccccccccc |
|  | Q XP\_016875493.1 | 1 | MVVTTSARGGGGDRTPSRRRGCGLAPAGAAALLAGASCLCYGRSLQGEFVHDDVWAIVNNPDVRPGAPLRWGIFTNDFWG   80 (456) |
|  | Q Consensus | 1 | m~~~~~~~~~~~~~~~~~~~~~~~~~~~~~~~l~~~~~~~~~~~~~~~~~~De~~~~~~a~~~~~~~~~~~~~~~~~~~~   80 (456) |
|  |  |  | ...........................+.+++++.+....+.........+||..+...+.++.+++. |
|  | T Consensus | 41 | ~~~~~~~~~~~~~~~~~~~~~~~~~~~l~~l~~~~~~~r~~~l~~~~~~~~DE~~~~~~a~~~~~~~~------------   108 (759) |
|  | T 6P25\_B | 41 | DERDAEDFSKEKPAAQSSLLRLESVVMPVIFTALALFTRMYKIGINNHVVWDEAHFGKFGSYYLRHEF------------   108 (759) |
|  | T ss\_dssp |  | ----------------CCHHHHHHHHHHHHHHHHHHHHHSSSGGGSCBCCTTHHHHHHHHHHHHTTBC------------ |
|  | T ss\_pred |  | cccCHhHHhhcCCcccchhhHhHHhHHHHHHHHHHHHHHHHhcCCCCceeeeHHHHHHHHHHHHhCCC------------ |
|  |
|  |
|  | Q ss\_pred |  | cCCCCCCCcccccchHHHHHHHHHHHhCCCc----------------hHHHHHHHHHHHHHHHHHHHHHHHH-hccchHH |
|  | Q XP\_016875493.1 | 81 | KGMAENTSHKSYRPLCVLTFKLNIFLTGMNP----------------FYFHAVNIILHCLVTLVLMYTCDKT-VFKNRGL   143 (456) |
|  | Q Consensus | 81 | ~~~~~~~~~~~~~Pl~~~~~~~~~~l~g~~~----------------~~~rl~~~l~~~~~~~~~~~l~~~~-~~~~~~~   143 (456) |
|  |  |  | +...+||++.++.+....++|.+. ...|++++++++++++++|.++|+. . ++.. |
|  | T Consensus | 109 | -------~~~~~PPl~~~l~a~~~~l~g~~~~~~~~~~~~~~~~~~~~~~R~~~~l~~~l~~~l~y~l~r~l~~--~~~~   179 (759) |
|  | T 6P25\_B | 109 | -------YHDVHPPLGKMLVGLSGYLAGYNGSWDFPSGEIYPDYLDYVKMRLFNASFSALCVPLAYFTAKAIGF--SLPT   179 (759) |
|  | T ss\_dssp |  | -------CCCSSCTHHHHHHHHHHHTTTCCSCSCCCSSCBCCSSCCHHHHHHHHHHHHHHHHHHHHHHHHHSCC--CTHH |
|  | T ss\_pred |  | -------CcCCCCHHHHHHHHHHHHHhCCCCCCCCCCCCCCCCcCCHHHHHHHHHHHHHHHHHHHHHHHHHcCC--CHHH |
|  |
|  |
|  | Q ss\_pred |  | HHHHHHHHHHCcccHHHHHhhhhhHHHHHHHHHHHHHHHHHHHHhc-------CCCCCCCCcchhHHHHHHHHHHHHHHH |
|  | Q XP\_016875493.1 | 144 | AFVTALLFAVHPIHTEAVAGIVGRADVLACLLFLLAFLSYNRSLDQ-------GCVGGSFPSTVSPFFLLLSLFLGTCAM   216 (456) |
|  | Q Consensus | 144 | a~~aa~l~~~~p~~~~~~~~~~~~~~~~~~~~~~l~~~~~~~~~~~-------~~~~~~~~~~~~~~~~~~~~~~~~la~   216 (456) |
|  |  |  | |++++++++++|..+.. ....+.|.+..++++++++++.+..++ + +..+.+++|+++|+++ |
|  | T Consensus | 180 | allaall~~~~p~~~~~--s~~~~~d~~~~~f~~l~l~~~~~~~~~~~~~~~~~----------~~~~~~l~gl~lgla~   247 (759) |
|  | T 6P25\_B | 180 | VWLMTVLVLFENSYSTL--GRFILLDSMLLFFTVASFFSFVMFHNQRSKPFSRK----------WWKWLLITGISLGCTI   247 (759) |
|  | T ss\_dssp |  | HHHHHHHHHSCHHHHHH--TSSSCSHHHHHHHHHHHHHHHHHHHTTSSSTTSHH----------HHHHHHHHHHHHHHHH |
|  | T ss\_pred |  | HHHHHHHHHhhhHHHHH--HHHHHHHHHHHHHHHHHHHHHHHHhhhcCCCCCHH----------HHHHHHHHHHHHHHHH |
|  |
|  |
|  | Q ss\_pred |  | HhHHHHHHHHHHHHHHHHHhcccccchhHhhccCchhHHHhhHHHHHHHHHHHHHHHHHHHHHHHHhcCCCCccccCCCC |
|  | Q XP\_016875493.1 | 217 | LVKETGITVFGVCLVYDLFSLSNKQDKSYLRASSNRNFLLTMRPFLKRAILVLSYVLVILYFRLWIMGGSMPLFSEQDNP   296 (456) |
|  | Q Consensus | 217 | ~~k~~~~~~~~~~~~~~~~~~~~~~~~~~~~~~~~~~~~~~~~~~~~~~~~~~~~~~~~~~~~~~~~~~~~~~~~~~~~~   296 (456) |
|  |  |  | ++|..++.+++.++++.+....++..++. .......+.........+++........+..........+..+.. |
|  | T Consensus | 248 | ~~K~~~~~~~~~~~l~~l~~~~~~~~~~~------~~~~~~~~~~~~~~~~~~~~p~~i~~~~~~~~~~~~~~~g~~~~~   321 (759) |
|  | T 6P25\_B | 248 | SVKMVGLFIITMVGIYTVIDLWTFLADKS------MSWKTYINHWLARIFGLIIVPFCIFLLCFKIHFDLLSHSGTGDAN   321 (759) |
|  | T ss\_dssp |  | HHCGGGHHHHHHHHHHHHHHHHHHTTCSS------SCHHHHHHHHHHHHCCCCCHHHHHHHHHHHHHHHHCCBCCTTGGG |
|  | T ss\_pred |  | HhHHHHHHHHHHHHHHHHHHHHHHhcCCC------CCHHHHHHHHHHHHHHHHHHHHHHHHHHHHHHHHhhcCCCCCccc |
|  |
|  |
|  | Q ss\_pred |  | cc |
|  | Q XP\_016875493.1 | 297 | AS   298 (456) |
|  | Q Consensus | 297 | ~~   298 (456) |
|  |  |  | .+ |
|  | T Consensus | 322 | ~s   323 (759) |
|  | T 6P25\_B | 322 | MP   323 (759) |
|  | T ss\_dssp |  | SC |
|  | T ss\_pred |  | CC |
|  |
| --- | | | |
|  | Template alignmentTemplate 3D StructurePDBe | | |
| 9. | 7BVF\_A Probable arabinosyltransferase B (E.C.2.4.2.-), Probable; Mycobacterium tuberculosis, cell wall synthesis; HET: 95E, DSL, CDL;{Mycolicibacterium smegmatis MC2 155} | | |
|  | Probability: 99.61%, E-value: 1.9e-12, Score: 126, Aligned cols: 360, Identities: 8%, Similarity: -0.066, | | |
|  |
|  | Q ss\_pred |  | HHHHHHHHHhhhcCCCcccccHHHHHhCCCCCCCCCcccccccccccccCCCCCCCcccccchHHHHHHHHHHHhCCCch |
|  | Q XP\_016875493.1 | 33 | LAGASCLCYGRSLQGEFVHDDVWAIVNNPDVRPGAPLRWGIFTNDFWGKGMAENTSHKSYRPLCVLTFKLNIFLTGMNPF   112 (456) |
|  | Q Consensus | 33 | l~~~~~~~~~~~~~~~~~~De~~~~~~a~~~~~~~~~~~~~~~~~~~~~~~~~~~~~~~~~Pl~~~~~~~~~~l~g~~~~   112 (456) |
|  |  |  | .++.+.+..+.... +.+.||.++...+++..+.+.....+...+ ....++++|++++..+..+ |.++. |
|  | T Consensus | 263 | ~~V~~~l~~w~~~g-p~~~DDg~~~~~Ar~~~~~G~~~n~~~~~~----------~~e~p~~lyY~lL~~W~~v-G~s~~   330 (1102) |
|  | T 7BVF\_A | 263 | AAVIATLLLWHVIG-ATSSDDGYLLTVARVAPKAGYVANYYRYFG----------TTEAPFDWYTSVLAQLAAV-STAGV   330 (1102) |
|  | T ss\_dssp |  | HHHHHHHHHTTTSC-CCCSTTHHHHHHHHHHHHHTSCBCSSSGGG----------CBCCTTCTTHHHHHHHHHH-CCCHH |
|  | T ss\_pred |  | HHHHHHHHHHHHhc-ccCCcchHHHHHHhhccccCcHHHHHHHHC----------CCCCCChHHHHHHHHHHhc-cCchH |
|  |
|  |
|  | Q ss\_pred |  | HHHHHHHHHHHHHHHHHHHHHHHHhccc------hHHHHHHHHHHHHCcccHHHHHhhhhhHHHHHHHHHHHHHHHHHHH |
|  | Q XP\_016875493.1 | 113 | YFHAVNIILHCLVTLVLMYTCDKTVFKN------RGLAFVTALLFAVHPIHTEAVAGIVGRADVLACLLFLLAFLSYNRS   186 (456) |
|  | Q Consensus | 113 | ~~rl~~~l~~~~~~~~~~~l~~~~~~~~------~~~a~~aa~l~~~~p~~~~~~~~~~~~~~~~~~~~~~l~~~~~~~~   186 (456) |
|  |  |  | ..|++|+++++++..++++..+..+... ++.+..++.+..+. .++.+ ....++|.+..++.+++++++.+. |
|  | T Consensus | 331 | ~LRLpSvlagl~t~~ll~r~v~~~lgr~~~~l~~~~~a~~~aal~~la-~~l~y--~~~~Rpyal~al~~~la~~~~~ra   407 (1102) |
|  | T 7BVF\_A | 331 | WMRLPATLAGIACWLIVSRFVLRRLGPGPGGLASNRVAVFTAGAVFLS-AWLPF--NNGLRPEPLIALGVLVTWVLVERS   407 (1102) |
|  | T ss\_dssp |  | HHTSHHHHHHHHHHHHCCCCCHHHSCCSSSSSSSCSHHHHHHHHHHHH-HHTTT--CCSSSSHHHHHHHHHHHHHHHHHH |
|  | T ss\_pred |  | HhHHHHHHHHHHHHHHHHHHHHHHhCCCCCCccccHHHHHHHHHHHHH-HHHHH--hcCCChHHHHHHHHHHHHHHHHHH |
|  |
|  |
|  | Q ss\_pred |  | HhcCCCCCCCCcchhHHHHHHHHHHHHHHHHhHHHHHHHHHHHHHHHHHhcccccchhHhhccCchhHHHhhHHHHHHHH |
|  | Q XP\_016875493.1 | 187 | LDQGCVGGSFPSTVSPFFLLLSLFLGTCAMLVKETGITVFGVCLVYDLFSLSNKQDKSYLRASSNRNFLLTMRPFLKRAI   266 (456) |
|  | Q Consensus | 187 | ~~~~~~~~~~~~~~~~~~~~~~~~~~~la~~~k~~~~~~~~~~~~~~~~~~~~~~~~~~~~~~~~~~~~~~~~~~~~~~~   266 (456) |
|  |  |  | .+++ +..++.+..++.++++.+++++++.+....+......+..+.+.... .... |
|  | T Consensus | 408 | ~~~~----------r~~~~al~~~~a~lal~~hptgll~laall~~~~~l~r~lr~r~~~~---------------~~~~   462 (1102) |
|  | T 7BVF\_A | 408 | IALG----------RLAPAAVAIIVATLTATLAPQGLIALAPLLTGARAIAQRIRRRRATD---------------GLLA   462 (1102) |
|  | T ss\_dssp |  | HHHT----------CSHHHHHHHHHHHHHTTSCGGGGGGGHHHHHTTHHHHHHHHHSCSSS---------------CSSH |
|  | T ss\_pred |  | HHcC----------ChHHHHHHHHHHHHHhccchHHHHHHHHHHHHHHHHHHHHHHhhhhc---------------chHH |
|  |
|  |
|  | Q ss\_pred |  | HHHHHHHHHHHHHHHHhcCCCCccccCCCCcccchhHHHHHHHHHHHHHHHHHHHHHhHHhhccCCCCCCccccchhhHH |
|  | Q XP\_016875493.1 | 267 | LVLSYVLVILYFRLWIMGGSMPLFSEQDNPASFSPYILTRFLTYSYLLAFNVWLLLAPVTLCYDWQVGSIPLVETIWDMR   346 (456) |
|  | Q Consensus | 267 | ~~~~~~~~~~~~~~~~~~~~~~~~~~~~~~~~~~~~~~~~~~~~~~~~~~~~~~~~~~~~~~~~~~~~~~~~~~~~~~~~   346 (456) |
|  |  |  | .....++.........+.+.....................+.+....+...+... ....+.. |
|  | T Consensus | 463 | ~la~vla~~~~~l~~~F~dq~l~~~~~a~~~~~~~g~~~~W~~e~~Ry~~L~~~~------------------~~~Gs~a   524 (1102) |
|  | T 7BVF\_A | 463 | PLAVLAAALSLITVVVFRDQTLATVAESARIKYKVGPTIAWYQDFLRYYFLTVES------------------NVEGSMS   524 (1102) |
|  | T ss\_dssp |  | HHHHHHHTGGGTHHHHSSSSCHHHHHHHHHHHHHSSCCCCGGGTTTTSTTTSCSS------------------CGGGCTT |
|  | T ss\_pred |  | HHHHHHHHHHHHHHHHHccCcHHHHHHHHhHHHhhCCCChhHHHHHHHHHHHhcc------------------CCCCCch |
|  |
|  |
|  | Q ss\_pred |  | HHHHHHHHHHHHHHHHHHHHHHhcccc---hHHHHHHHHHHHHHhhHhccccCCCccccccccchHHHHHHHHHHHHHHH |
|  | Q XP\_016875493.1 | 347 | NLATIFLAVVMALLSLHCLAAFKRLEH---KEVLVGLLFLVFPFIPASNLFFRVGFVVAERVLYMPSMGYCILFVHGLSK   423 (456) |
|  | Q Consensus | 347 | ~~~~~~~~~~~~~~~~~~~~~~~~~~~---~~~~~~~~~~~~~~~~~~~~~~~~~~~~~~Ry~~~~~~~~~ll~~~~~~~   423 (456) |
|  |  |  | ........+..+.........+++... ......+.+..+.++.+.+ ...+..||+....++..++++.+... |
|  | T Consensus | 525 | rr~pvLl~l~~L~~~~~~l~Rrrr~~g~~~~~~~~ll~~~~l~lvll~~-----tPsKwt~hfg~~A~~~~aLlA~~~v~   599 (1102) |
|  | T 7BVF\_A | 525 | RRFAVLVLLFCLFGVLFVLLRRGRVAGLASGPAWRLIGTTAVGLLLLTF-----TPTKWAVQFGAFAGLAGVLGAVTAFT   599 (1102) |
|  | T ss\_dssp |  | THHHHHHHHHHHHHHHHHHHSSSCCSSSCHHHHHHHHHHHHHHHHHGGG-----CSCCCSGGGGGGHHHHHHHHHHHHHH |
|  | T ss\_pred |  | HHHHHHHHHHHHHHHHHHHHHcCCCCCcccCHHHHHHHHHHHHHHHHhc-----CccHHHHHHHHHHHHHHHHHHHHHHH |
|  |
|  |
|  | Q ss\_pred |  | HHHHHhHHhHHHHHHHHHHHHHHHHHHHHHhcC |
|  | Q XP\_016875493.1 | 424 | LCTWLNRCGATTLIVSTVLLLLLFSWKTVKQNE   456 (456) |
|  | Q Consensus | 424 | ~~~~~~~~~~~~~~~~~~~~~~~~~~~~~~~~~   456 (456) |
|  |  |  | +.+...+..+...... .+++++........|+ |
|  | T Consensus | 600 | ~~~~~~r~~r~~~~~~-~~~~~~~als~~g~n~   631 (1102) |
|  | T 7BVF\_A | 600 | FARIGLHSRRNLTLYV-TALLFVLAWATSGING   631 (1102) |
|  | T ss\_dssp |  | HHHTTSSCSHHHHHHH-HHHHHHHHHHTTSCCC |
|  | T ss\_pred |  | HhhhhhcchHHHHHHH-HHHHHHHHHHHhhhcc |
|  |
| --- | | | |
|  | Template alignmentTemplate 3D StructurePDBe | | |
| 10. | 7BVF\_B Probable arabinosyltransferase B (E.C.2.4.2.-), Probable; Mycobacterium tuberculosis, cell wall synthesis; HET: 95E, DSL, CDL;{Mycolicibacterium smegmatis MC2 155} | | |
|  | Probability: 99.54%, E-value: 1.5e-11, Score: 120.3, Aligned cols: 357, Identities: 8%, Similarity: -0.049, | | |
|  |
|  | Q ss\_pred |  | HHHHHHHHhhhcCCCcccccHHHHHhCCCCCCCCCcccccccccccccCCCCCCCcccccchHHHHHHHHHHHhCCCchH |
|  | Q XP\_016875493.1 | 34 | AGASCLCYGRSLQGEFVHDDVWAIVNNPDVRPGAPLRWGIFTNDFWGKGMAENTSHKSYRPLCVLTFKLNIFLTGMNPFY   113 (456) |
|  | Q Consensus | 34 | ~~~~~~~~~~~~~~~~~~De~~~~~~a~~~~~~~~~~~~~~~~~~~~~~~~~~~~~~~~~Pl~~~~~~~~~~l~g~~~~~   113 (456) |
|  |  |  | +++...+.......+.+.||.++...+++..+.+.....+...+ ....++++|++++..+..+ |.++.. |
|  | T Consensus | 281 | ~~V~~~L~~w~~~g~~~~DDg~~~~~ar~~~~~G~~~n~~~~~~----------~~e~p~~~yY~lL~~w~~l-G~s~~~   349 (1116) |
|  | T 7BVF\_B | 281 | AVVIFGFLLWHVIGANSSDDGYILGMARVADHAGYMSNYFRWFG----------SPEDPFGWYYNLLALMTHV-SDASLW   349 (1116) |
|  | T ss\_dssp |  | HHHHHHHHHHHHHCCCCSSHHHHHHHHHTTTTSSSCBCCSSSSS----------CBCCSSCSSHHHHHHHTTT-CCCHHH |
|  | T ss\_pred |  | HHHHHHHHHHHHhCcCCCCchHHHHHhhccccccchHHHHHHhC----------CCCCccHHHHHHHHHHHHc-CCchHH |
|  |
|  |
|  | Q ss\_pred |  | HHHHHHHHHHHHHHHHHHHHHHHhcc----chHHHHHHHHHHHHCcccHHHHHhhhhhHHHHHHHHHHHHHHHHHHHHhc |
|  | Q XP\_016875493.1 | 114 | FHAVNIILHCLVTLVLMYTCDKTVFK----NRGLAFVTALLFAVHPIHTEAVAGIVGRADVLACLLFLLAFLSYNRSLDQ   189 (456) |
|  | Q Consensus | 114 | ~rl~~~l~~~~~~~~~~~l~~~~~~~----~~~~a~~aa~l~~~~p~~~~~~~~~~~~~~~~~~~~~~l~~~~~~~~~~~   189 (456) |
|  |  |  | .|++|+++++++..++++.+.+.+.. ++..++.++++++.. ...+ ....++|.+..++.+++++++.+..++ |
|  | T Consensus | 350 | lRlpSllagl~t~~ll~r~vl~~lg~~~~~~~~a~~~aal~~l~~--~lpy--~~~~Rpyal~al~~~lal~~~~ra~~~   425 (1116) |
|  | T 7BVF\_B | 350 | MRLPDLAAGLVCWLLLSREVLPRLGPAVEASKPAYWAAAMVLLTA--WMPF--NNGLRPEGIIALGSLVTYVLIERSMRY   425 (1116) |
|  | T ss\_dssp |  | HTSHHHHHHHHHHHHCCCCCGGGSCTTTSSCHHHHHHHHHHHHHH--HSSS--TTSSSTHHHHHHHHHHHHHHHHHHHHH |
|  | T ss\_pred |  | hhHHHHHHHHHHHHHHHHHHHHHhchhhcCCHHHHHHHHHHHHHH--hchh--cCCCcHHHHHHHHHHHHHHHHHHHhcC |
|  |
|  |
|  | Q ss\_pred |  | CCCCCCCCcchhHHHHHHHHHHHHHHHHhHHHHHHHHHHHHHHHHHhcccccchhHhhccCchhHHHhhHHHHHHHHHHH |
|  | Q XP\_016875493.1 | 190 | GCVGGSFPSTVSPFFLLLSLFLGTCAMLVKETGITVFGVCLVYDLFSLSNKQDKSYLRASSNRNFLLTMRPFLKRAILVL   269 (456) |
|  | Q Consensus | 190 | ~~~~~~~~~~~~~~~~~~~~~~~~la~~~k~~~~~~~~~~~~~~~~~~~~~~~~~~~~~~~~~~~~~~~~~~~~~~~~~~   269 (456) |
|  |  |  | + +..++.+.+++.++++.+|+++++.+....+......+..+++.... ......+ |
|  | T Consensus | 426 | ~----------r~~~~al~~~~a~lal~~hptgl~alaall~~l~~l~r~lr~r~~~~---------------~~la~la   480 (1116) |
|  | T 7BVF\_B | 426 | S----------RLTPAALAVVTAAFTLGVQPTGLIAVAALVAGGRPMLRILVRRHRLV---------------GTLPLVS   480 (1116) |
|  | T ss\_dssp |  | T----------CSSHHHHHHHHHHHHHTTCGGGCCSHHHHHHTTHHHHHHHHHHHTTS---------------CSHHHHS |
|  | T ss\_pred |  | C----------CcHHHHHHHHHHHHHHhccHHHHHHHHHHHHhhHHHHHHHHHhhhhc---------------chHHHHH |
|  |
|  |
|  | Q ss\_pred |  | HHHHHHHHHHHHHhcCCCCccccCCCCcccchhHHHHHHHHHHHHHHHHHHHHHhHHhhccCCCCCCccccchhhHHHHH |
|  | Q XP\_016875493.1 | 270 | SYVLVILYFRLWIMGGSMPLFSEQDNPASFSPYILTRFLTYSYLLAFNVWLLLAPVTLCYDWQVGSIPLVETIWDMRNLA   349 (456) |
|  | Q Consensus | 270 | ~~~~~~~~~~~~~~~~~~~~~~~~~~~~~~~~~~~~~~~~~~~~~~~~~~~~~~~~~~~~~~~~~~~~~~~~~~~~~~~~   349 (456) |
|  |  |  | ..++.........+.......................+......+...+... ...+..... |
|  | T Consensus | 481 | ~~la~~~~~l~~~F~dqsl~~~~~a~~~~~~~g~~~~W~~e~~Ry~~L~~~~-------------------~~Gs~arr~   541 (1116) |
|  | T 7BVF\_B | 481 | PMLAAGTVILTVVFADQTLSTVLEATRVRAKIGPSQAWYTENLRYYYLILPT-------------------VDGSLSRRF   541 (1116) |
|  | T ss\_dssp |  | HHHHTTTCTHHHHTSSSCHHHHHHHHHHHHHTSCCCCGGGTHHHHHGGGSSS-------------------SSSCHHHHH |
|  | T ss\_pred |  | HHHHHHHHHHHHHHccchHHHHHHHHHHHHHhcCCchHHHHhHHHHHHhccC-------------------CCCChHHHH |
|  |
|  |
|  | Q ss\_pred |  | HHHHHHHHHHHHHHHHHHHhcccc---hHHHHHHHHHHHHHhhHhccccCCCccccccccchHHHHHHHHHHHHHHHHHH |
|  | Q XP\_016875493.1 | 350 | TIFLAVVMALLSLHCLAAFKRLEH---KEVLVGLLFLVFPFIPASNLFFRVGFVVAERVLYMPSMGYCILFVHGLSKLCT   426 (456) |
|  | Q Consensus | 350 | ~~~~~~~~~~~~~~~~~~~~~~~~---~~~~~~~~~~~~~~~~~~~~~~~~~~~~~~Ry~~~~~~~~~ll~~~~~~~~~~   426 (456) |
|  |  |  | .....++.+.........+++... ......+.+.++.++.+.+ ...+..||+....++...+++.+...+.+ |
|  | T Consensus | 542 | ~vLl~ll~L~~~~~~l~R~rr~~g~~~~~~~~ll~~~~~~~~ll~~-----tPtKwthhfg~~a~~~~aLlA~~~~~~~~   616 (1116) |
|  | T 7BVF\_B | 542 | GFLITALCLFTAVFIMLRRKRIPSVARGPAWRLMGVIFGTMFFLMF-----TPTKWVHHFGLFAAVGAAMAALTTVLVSP   616 (1116) |
|  | T ss\_dssp |  | HHHHHHHHHHHHHHHHHHCSCCTTSCTTHHHHHHHHHHHHHHHTTS-----CSCCCGGGGGGGGTTHHHHHHHHHHHTST |
|  | T ss\_pred |  | HHHHHHHHHHHHHHHHHhcCCCCcccCcHHHHHHHHHHHHHHHHhc-----CchhHHHHHHHHHHHHHHHHHHHHHHhCh |
|  |
|  |
|  | Q ss\_pred |  | HHhHHhH-HHHHHHHHHHHHHHHHHHHHh |
|  | Q XP\_016875493.1 | 427 | WLNRCGA-TTLIVSTVLLLLLFSWKTVKQ   454 (456) |
|  | Q Consensus | 427 | ~~~~~~~-~~~~~~~~~~~~~~~~~~~~~   454 (456) |
|  |  |  | ...+..+ +......+++++.+++...+. |
|  | T Consensus | 617 | ~~~r~~~~r~~~~~~~~~~~ala~~g~n~   645 (1116) |
|  | T 7BVF\_B | 617 | SVLRWSRNRMAFLAALFFLLALCWATTNG   645 (1116) |
|  | T ss\_dssp |  | TTSCCHHHHHHHHHHHHHHHHHHTSSCCC |
|  | T ss\_pred |  | hhcchHHHHHHHHHHHHHHHHHHHhhhcc |
|  |
| --- | | | |
|  | Template alignmentTemplate 3D StructurePDBe | | |
| 11. | 6W98\_A F5/8 type C domain-containing protein; Glycosyltransferase, lipomannan, lipoarabinomannan, arabinofuranose, membrane; HET: PNS, 6OU; 2.9A {Escherichia coli (strain K12)}; Related PDB entries: 6WBX\_A 6WBY\_A | | |
|  | Probability: 99.51%, E-value: 3.2e-11, Score: 124.97, Aligned cols: 398, Identities: 13%, Similarity: 0.026, | | |
|  |
|  | Q ss\_pred |  | CCCCccchhhccHHHHHHHHHHHHHHHHHHhhhcCCCcccccHHHHHhCCCCCCCCCcccccccccccccCCCCCCCccc |
|  | Q XP\_016875493.1 | 12 | GDRTPSRRRGCGLAPAGAAALLAGASCLCYGRSLQGEFVHDDVWAIVNNPDVRPGAPLRWGIFTNDFWGKGMAENTSHKS   91 (456) |
|  | Q Consensus | 12 | ~~~~~~~~~~~~~~~~~~~~~l~~~~~~~~~~~~~~~~~~De~~~~~~a~~~~~~~~~~~~~~~~~~~~~~~~~~~~~~~   91 (456) |
|  |  |  | ..++.++...........+++++++.+..+....+.....+|..+......+.... ...+++.......++..+... |
|  | T Consensus | 1 | ~~~~~~~~~~~~~~~~~ll~~~lll~~~~~~~~~g~~~~d~~~~~~~~~~~~l~~~---~~~W~~~~~~G~~~~~~~~y~   77 (1413) |
|  | T 6W98\_A | 1 | SYVMTYRLDSSALSRRWLAVAAAVSLLLTFSQSPGQISPDTKLDLAINPLRFAARA---LNLWSSDLPFGQAQNQAYGYL   77 (1413) |
|  | T ss\_dssp |  | ----CCCCCCCCCCTHHHHHHHHHHHHHHTTSSTTCBCCTTCSHHHHCHHHHHHHT---TSSEESSSTTSEECCSSGGGC |
|  | T ss\_pred |  | CccccccCCcccchHHHHHHHHHHHHHHHHhCCCCccCCCCCCccccCHHHHHHHH---HhccCCCCCCCCCchhhhhhh |
|  |
|  |
|  | Q ss\_pred |  | ccchHHHHHHHHHHHhCCCchHHHHHHHHHHHHHHHHHHHHHHHHhccchHHHHHHHHHHHHCcccHHHHHhhhhhHHHH |
|  | Q XP\_016875493.1 | 92 | YRPLCVLTFKLNIFLTGMNPFYFHAVNIILHCLVTLVLMYTCDKTVFKNRGLAFVTALLFAVHPIHTEAVAGIVGRADVL   171 (456) |
|  | Q Consensus | 92 | ~~Pl~~~~~~~~~~l~g~~~~~~rl~~~l~~~~~~~~~~~l~~~~~~~~~~~a~~aa~l~~~~p~~~~~~~~~~~~~~~~   171 (456) |
|  |  |  | +| ...++.+...+........|+...++.+++.+.+|+++|+...+++..+++++++++++|.++....... .+.+ |
|  | T Consensus | 78 | ~P--~~~~~~l~~~lg~~~~~~~rl~~~l~~~la~~g~y~L~r~l~~~~~~~al~Aal~yalsP~~l~~~~~~~--~~~~   153 (1413) |
|  | T 6W98\_A | 78 | FP--HGAFFSLGHLLGVPAWVTQRLWWALLIVAGFWGLIRVAEALGIGTRGSRIIAAVAFALSPRVLTTLGAIS--SETL   153 (1413) |
|  | T ss\_dssp |  | CC--CCHHHHHHHHHTCCHHHHHHHHHHHHHHHHHHHHHHHHHHHTCSCTTHHHHHHHHHHTCHHHHHHHTTCG--GGTH |
|  | T ss\_pred |  | hh--HHHHHHHHHHcCCCHHHHHHHHHHHHHHHHHHHHHHHHHHhCCCChHHHHHHHHHHHHCHHHHHHhhccC--hhhH |
|  |
|  |
|  | Q ss\_pred |  | HHHHHHHHHHHHHHHHhc--CCCCCCCCcchhHHHHHHHHHHHHHHHHhHHHHHHHHHHHHHHHHHhcccccchhHhhcc |
|  | Q XP\_016875493.1 | 172 | ACLLFLLAFLSYNRSLDQ--GCVGGSFPSTVSPFFLLLSLFLGTCAMLVKETGITVFGVCLVYDLFSLSNKQDKSYLRAS   249 (456) |
|  | Q Consensus | 172 | ~~~~~~l~~~~~~~~~~~--~~~~~~~~~~~~~~~~~~~~~~~~la~~~k~~~~~~~~~~~~~~~~~~~~~~~~~~~~~~   249 (456) |
|  |  |  | ..+++.++++.+.+..++ + ++++.+++++++++...++....++.+.+.+..++..++++++ |
|  | T Consensus | 154 | ~~~llp~~ll~l~~~~~~~~~----------~~r~~~~~~l~~~l~~~~~~~~~~~~l~~~~l~~l~~~~~~~~------   217 (1413) |
|  | T 6W98\_A | 154 | PMMLAPWVLLPLILTFQGRMS----------PRRAAALSAVAVALMGAVNAVATALACGVAVIWWLAHRPNRTW------   217 (1413) |
|  | T ss\_dssp |  | HHHHHHHHHHHHHHHHTTSSC----------HHHHHHHHHHHHHHSCSSSHHHHHHHSHHHHHHHHSBCCCHHH------ |
|  | T ss\_pred |  | HHHHHHHHHHHHHHHHcCCCC----------HHHHHHHHHHHHHHhcchhHHHHHHHHHHHHHHHHHCCCCchH------ |
|  |
|  |
|  | Q ss\_pred |  | CchhHHHhhHHHHHHHHHHHHHHHHHHHHHHHHhcCCCCccccCCCCcccchhHHHHHHHHHHHHHHHHHHHHHhHHhhc |
|  | Q XP\_016875493.1 | 250 | SNRNFLLTMRPFLKRAILVLSYVLVILYFRLWIMGGSMPLFSEQDNPASFSPYILTRFLTYSYLLAFNVWLLLAPVTLCY   329 (456) |
|  | Q Consensus | 250 | ~~~~~~~~~~~~~~~~~~~~~~~~~~~~~~~~~~~~~~~~~~~~~~~~~~~~~~~~~~~~~~~~~~~~~~~~~~~~~~~~   329 (456) |
|  |  |  | ++.........+.+..+........................... ............ |
|  | T Consensus | 218 | ---------~~~~~~~~~~~~l~~~~wl~Pll~~~~~~~~~~~~~e~~~~~~~---------------~~s~~~~l~~~~   273 (1413) |
|  | T 6W98\_A | 218 | ---------WRFTAWWIPCLALASTWWIVALLIFGKISPKFLDFIESSGVTTQ---------------WTSLTEVLRGTD   273 (1413) |
|  | T ss\_dssp |  | ---------HHHHHHHHHHHHHHHHHHHHHHHHHHHHSCCCTTSSCC----------------------CCHHHHHHTC- |
|  | T ss\_pred |  | ---------HHHHHHHHHHHHHHHHHHHHHHHHhcccCHhHHHHHhccccccc---------------cccHHHHHhCCC |
|  |
|  |
|  | Q ss\_pred |  | cCCCCCCccccchhhHHHHHHHHHHHHHHHHHHHHHHHHhcccchHHHHHHHHHHHHHhhHhc----------------c |
|  | Q XP\_016875493.1 | 330 | DWQVGSIPLVETIWDMRNLATIFLAVVMALLSLHCLAAFKRLEHKEVLVGLLFLVFPFIPASN----------------L   393 (456) |
|  | Q Consensus | 330 | ~~~~~~~~~~~~~~~~~~~~~~~~~~~~~~~~~~~~~~~~~~~~~~~~~~~~~~~~~~~~~~~----------------~   393 (456) |
|  |  |  | .+.........................++.++......+++++.+......+.+.+.+..... . |
|  | T Consensus | 274 | ~w~~~~~~~~~~~~~~~~~~~~~~~~~~l~~lgl~~l~~r~~~~~~~l~~~~l~g~~l~~~~~~~~~~~p~~~~~~~~l~   353 (1413) |
|  | T 6W98\_A | 274 | SWTPFVAPTATAGSSLVTQSAMVIATTMLAAAGMAGLAMRGMPARGRLVAVLLIGLVLLTAGYTGALGSPIAQQIQFFLD   353 (1413) |
|  | T ss\_dssp |  | ----------------CCHHHHHHHHHHHHHHHHHHHSSTTCTTHHHHHHHHHHHHHTC--------------------- |
|  | T ss\_pred |  | CCccccCCCCcccchHHHhHHHHHHHHHHHHHHHHHHHhCCCchHHHHHHHHHHHHHHHHhcccCCCCCchHHHHHHHhc |
|  |
|  |
|  | Q ss\_pred |  | ccCCCccccccccchHHHHHHHHHHHHHHHHHHH-------------HhHHhHHHHHHHHHHHHHHHHHHHHHhcC |
|  | Q XP\_016875493.1 | 394 | FFRVGFVVAERVLYMPSMGYCILFVHGLSKLCTW-------------LNRCGATTLIVSTVLLLLLFSWKTVKQNE   456 (456) |
|  | Q Consensus | 394 | ~~~~~~~~~~Ry~~~~~~~~~ll~~~~~~~~~~~-------------~~~~~~~~~~~~~~~~~~~~~~~~~~~~~   456 (456) |
|  |  |  | ..........||..+..++++++++.++..+.++ .+++.+.....++++++++.........+ |
|  | T Consensus | 354 | p~~~~~R~~~Rf~~~~~l~lall~a~~l~~l~~~~~~~~~~~~~~~~~~~~~~~~~~~~~~~~~~~~~~~p~~~~~   429 (1413) |
|  | T 6W98\_A | 354 | DGGTPLRNVHKLEPLIRLPLILGLAHALSRIPLPASVPVRQWLSALARPERNRAVAFAIVLLVALAASTSLAWTGR   429 (1413) |
|  | T ss\_dssp |  | ------CCGGGSHHHHHHHHHHHHHHHTSSSCCTTTSCC------------CTTTHHHHHHHHHHHHHTHHHHTTC |
|  | T ss\_pred |  | cCchhhcChhhhHHHHHHHHHHHHHHHHHhCCCCCCCCHHHHHHhhcChHHHHHHHHHHHHHHHHHHHHHHHHhCC |
|  |
| --- | | | |
|  | Template alignmentTemplate 3D StructurePDBe | | |
| 12. | 7BWR\_A Integral membrane indolylacetylinositol arabinosyltransferase EmbB; Mycobacterium tuberculosis, EmbB, cryo-EM, ethambutol; HET: F8L;{Mycolicibacterium smegmatis MC2 155}; Related PDB entries: 7BVC\_B 7BVG\_B 7BWR\_B 7BX8\_B 7BX8\_A | | |
|  | Probability: 99.51%, E-value: 1.4e-11, Score: 119.64, Aligned cols: 351, Identities: 9%, Similarity: -0.047, | | |
|  |
|  | Q ss\_pred |  | HHHHHHHHhhhcCCCcccccHHHHHhCCCCCCCCCcccccccccccccCCCCCCCcccccchHHHHHHHHHHHhCCCchH |
|  | Q XP\_016875493.1 | 34 | AGASCLCYGRSLQGEFVHDDVWAIVNNPDVRPGAPLRWGIFTNDFWGKGMAENTSHKSYRPLCVLTFKLNIFLTGMNPFY   113 (456) |
|  | Q Consensus | 34 | ~~~~~~~~~~~~~~~~~~De~~~~~~a~~~~~~~~~~~~~~~~~~~~~~~~~~~~~~~~~Pl~~~~~~~~~~l~g~~~~~   113 (456) |
|  |  |  | .++...+.......+...||.++...+++..+.+....-+...+ ....++++|+.++..+..+ |.++.. |
|  | T Consensus | 267 | ~~V~a~L~~w~~ig~~~~DDg~~~~~ar~~~~~G~~~n~~r~~~----------~~e~p~~~yY~lL~~w~~l-G~s~~~   335 (1082) |
|  | T 7BWR\_A | 267 | GVVVGGMAIWYVIGANSSDDGYILQMARTAEHAGYMANYFRWFG----------SPEDPFGWYYNVLALMTKV-SDASIW   335 (1082) |
|  | T ss\_dssp |  | HHHTTTTTHHHHHSCCCTTHHHHHHHHHSHHHHSSCBCCSSSTT----------CBSCSSCCTHHHHHTTTTS-CCCTTT |
|  | T ss\_pred |  | HHHHHHHHHHHHhccCCCchHHHHHHHHHHhHhcchHHHHHHhC----------CCCCccHHHHHHHHHHHHh-cCChHH |
|  |
|  |
|  | Q ss\_pred |  | HHHHHHHHHHHHHHHH-----HHHHHHHhccchHHHHHHHHHHHHCcccHHHHHhhhhhHHHHHHHHHHHHHHHHHHHHh |
|  | Q XP\_016875493.1 | 114 | FHAVNIILHCLVTLVL-----MYTCDKTVFKNRGLAFVTALLFAVHPIHTEAVAGIVGRADVLACLLFLLAFLSYNRSLD   188 (456) |
|  | Q Consensus | 114 | ~rl~~~l~~~~~~~~~-----~~l~~~~~~~~~~~a~~aa~l~~~~p~~~~~~~~~~~~~~~~~~~~~~l~~~~~~~~~~   188 (456) |
|  |  |  | .|++|+++++++..++ +.+. +....++..++.+++.+.. ..+.+ ....++|.+..++.+++.+++.+..+ |
|  | T Consensus | 336 | lRLpS~laglat~~ll~r~v~~~lg-r~~~~~~~a~~~Aal~~l~--~~l~y--~~~~Rpy~l~al~~~la~~~l~ra~~   410 (1082) |
|  | T 7BWR\_A | 336 | IRLPDLICALICWLLLSREVLPRLG-PAVAGSRAAMWAAGLVLLG--AWMPF--NNGLRPEGQIATGALITYVLIERAVT   410 (1082) |
|  | T ss\_dssp |  | TTGGGTGGGTSSHHHHTTSSTGGGC-HHHHHCSHHHHHHHHHHHH--HHTTT--TTSSSTTHHHHHHHHHHHHHHHHHHH |
|  | T ss\_pred |  | HHHHHHHHHHHHHHHHHHhhHHHhc-hhhcccHHHHHHHHHHHHH--HHHHH--hcCCchHHHHHHHHHHHHHHHHHHHh |
|  |
|  |
|  | Q ss\_pred |  | cCCCCCCCCcchhHHHHHHHHHHHHHHHHhHHHHHHHHHHHHHHHHHhcccccchhHhhccCchhHHHhhHHHHHHHHHH |
|  | Q XP\_016875493.1 | 189 | QGCVGGSFPSTVSPFFLLLSLFLGTCAMLVKETGITVFGVCLVYDLFSLSNKQDKSYLRASSNRNFLLTMRPFLKRAILV   268 (456) |
|  | Q Consensus | 189 | ~~~~~~~~~~~~~~~~~~~~~~~~~la~~~k~~~~~~~~~~~~~~~~~~~~~~~~~~~~~~~~~~~~~~~~~~~~~~~~~   268 (456) |
|  |  |  | ++ +..++.+.+++.++++.+|+++++.+.+..+......++.+++.... ...... |
|  | T Consensus | 411 | ~~----------r~~~~al~~~~a~lal~~hptgllalaallv~l~~l~r~~r~r~~~~---------------~~la~l   465 (1082) |
|  | T 7BWR\_A | 411 | SG----------RLTPAALAITTAAFTLGIQPTGLIAVAALLAGGRPILRIVMRRRRLV---------------GTWPLI   465 (1082) |
|  | T ss\_dssp |  | HC----------CSHHHHHHHHHHHHTTSSCTTCHHHHHHHHHTHHHHHHHHHHTTTTS---------------CSHHHH |
|  | T ss\_pred |  | cC----------CCHHHHHHHHHHHHHHHhcHHHHHHHHHHHHccHHHHHHHhhhhccc---------------cHHHHH |
|  |
|  |
|  | Q ss\_pred |  | HHHHHHHHHHHHHHhcCCCCccccCCCCcccchhHHHHHHHHHHHHHHHHHHHHHhHHhhccCCCCCCccccchhhHHHH |
|  | Q XP\_016875493.1 | 269 | LSYVLVILYFRLWIMGGSMPLFSEQDNPASFSPYILTRFLTYSYLLAFNVWLLLAPVTLCYDWQVGSIPLVETIWDMRNL   348 (456) |
|  | Q Consensus | 269 | ~~~~~~~~~~~~~~~~~~~~~~~~~~~~~~~~~~~~~~~~~~~~~~~~~~~~~~~~~~~~~~~~~~~~~~~~~~~~~~~~   348 (456) |
|  |  |  | ...++.........+.......................+..........+... ...+.... |
|  | T Consensus | 466 | a~~~aa~~~~l~~~F~dqsl~~~~~a~~~~~~~g~~~~W~~e~~Ry~~L~~~~-------------------~~Gs~arr   526 (1082) |
|  | T 7BWR\_A | 466 | APLLAAGTVILAVVFADQTIATVLEATRIRTAIGPSQEWWTENLRYYYLILPT-------------------TDGAISRR   526 (1082) |
|  | T ss\_dssp |  | HHHHHTTSSTHHHHTSSCCHHHHHHHHHHHHHHSCCTTTSGGGSSSSCCCCTT-------------------CCCHHHHH |
|  | T ss\_pred |  | HHHHHHHHHHHHHHHhchHHHHHHHHHHHHhhcCCCccHHHhhHHHHHHHcCC-------------------CCCcchHH |
|  |
|  |
|  | Q ss\_pred |  | HHHHHHHHHHHHHHHHHHHHhcccc---hHHHHHHHHHHHHHhhHhccccCCCccccccccchHHHHHHHHHHHHHHHHH |
|  | Q XP\_016875493.1 | 349 | ATIFLAVVMALLSLHCLAAFKRLEH---KEVLVGLLFLVFPFIPASNLFFRVGFVVAERVLYMPSMGYCILFVHGLSKLC   425 (456) |
|  | Q Consensus | 349 | ~~~~~~~~~~~~~~~~~~~~~~~~~---~~~~~~~~~~~~~~~~~~~~~~~~~~~~~~Ry~~~~~~~~~ll~~~~~~~~~   425 (456) |
|  |  |  | ......+..++........+++... ......+......++...+ ......||+.........+++..+..+. |
|  | T Consensus | 527 | ~~vLl~l~~L~~~~~~llR~~r~~g~~~~~~~~ll~~~~~~~~ll~~-----tPtKwthhfga~ag~g~~l~a~a~v~l~   601 (1082) |
|  | T 7BWR\_A | 527 | VAFVFTAMCLFPSLFMMLRRKHIAGVARGPAWRLMGIIFATMFFLMF-----TPTKWIHHFGLFAAVGGAMAALATVLVS   601 (1082) |
|  | T ss\_dssp |  | HTTTTTTTSSHHHHHHHHHSSSCSSSCTTHHHHHHHHHHHHHHHTBT-----TTBCCCCCTTGGGSHHHHHHHHHHHHSS |
|  | T ss\_pred |  | HHHHHHHHHHHHHHHHHHhhcccCCCcCCHHHHHHHHHHHHHHHHhh-----CcchHHHHHHHHHHHHHHHHHHHHHHHc |
|  |
|  |
|  | Q ss\_pred |  | HHHhHHhH-HHHHHHHHHHHHHHHH |
|  | Q XP\_016875493.1 | 426 | TWLNRCGA-TTLIVSTVLLLLLFSW   449 (456) |
|  | Q Consensus | 426 | ~~~~~~~~-~~~~~~~~~~~~~~~~   449 (456) |
|  |  |  | ....+..+ +......++++..+++ |
|  | T Consensus | 602 | ~~~~r~~r~r~~~~~~~~~~~ala~   626 (1082) |
|  | T 7BWR\_A | 602 | PTVLRSARNRMAFLSLVLFVLAFCF   626 (1082) |
|  | T ss\_dssp |  | TTTCCCHHHHHHHHHHHHHHHHHHT |
|  | T ss\_pred |  | HHhcccHHHHHHHHHHHHHHHHHHh |
|  |
| --- | | | |
|  | Template alignmentTemplate 3D StructurePDBe | | |
| 13. | 7BVE\_B Integral membrane indolylacetylinositol arabinosyltransferase EmbC; Mycobacterium smegmatis, cell wall synthesis; HET: PO4, PN7, 95E; 2.81A {Mycolicibacterium smegmatis MC2 155}; Related PDB entries: 7BVH\_B 7BVH\_A 7BVE\_A | | |
|  | Probability: 99.49%, E-value: 1.1e-10, Score: 113.79, Aligned cols: 357, Identities: 10%, Similarity: -0.064, | | |
|  |
|  | Q ss\_pred |  | HHHHHHHHhhhcCCCcccccHHHHHhCCCCCCCCCcccccccccccccCCCCCCCcccccchHHHHHHHHHHHhCCCchH |
|  | Q XP\_016875493.1 | 34 | AGASCLCYGRSLQGEFVHDDVWAIVNNPDVRPGAPLRWGIFTNDFWGKGMAENTSHKSYRPLCVLTFKLNIFLTGMNPFY   113 (456) |
|  | Q Consensus | 34 | ~~~~~~~~~~~~~~~~~~De~~~~~~a~~~~~~~~~~~~~~~~~~~~~~~~~~~~~~~~~Pl~~~~~~~~~~l~g~~~~~   113 (456) |
|  |  |  | .++...+-......+.+.||.++...+++..+.+.....+...+ ..+....+|+.++..+.. +|.++.. |
|  | T Consensus | 261 | ~~V~~~l~~w~~ig~~~~DEg~~l~~ar~~~~~Gy~~n~~~~~~----------~~dapfg~yY~lL~~W~~-vG~s~~~   329 (1084) |
|  | T 7BVE\_B | 261 | GLVSAMLVWWHFVGANTADDGYILTMARVSEHAGYMANYYRWFG----------TPESPFGWYYDLLALWAH-VSTASVW   329 (1084) |
|  | T ss\_dssp |  | HHHHHHHHHHHHSCCCCSSSHHHHHHHHHHHHHSSCBCSSSGGG----------CBCCSSCSSHHHHHHHTT-TCCCHHH |
|  | T ss\_pred |  | HHHHHHHHHHHHhcccccchhHHhhhhhchhhcCchHHHHHHHC----------CCCcccHHHHHHHHHHHH-ccccHHH |
|  |
|  |
|  | Q ss\_pred |  | HHHHHHHHHHHHHHHH-----HHHHHHHhccchHHHHHHHHHHHHCcccHHHHHhhhhhHHHHHHHHHHHHHHHHHHHHh |
|  | Q XP\_016875493.1 | 114 | FHAVNIILHCLVTLVL-----MYTCDKTVFKNRGLAFVTALLFAVHPIHTEAVAGIVGRADVLACLLFLLAFLSYNRSLD   188 (456) |
|  | Q Consensus | 114 | ~rl~~~l~~~~~~~~~-----~~l~~~~~~~~~~~a~~aa~l~~~~p~~~~~~~~~~~~~~~~~~~~~~l~~~~~~~~~~   188 (456) |
|  |  |  | .|++++++++++..++ ..+. +....++...+.++++++. ....+ ....++|.+..++.+++++++.|..+ |
|  | T Consensus | 330 | LRlpSll~glat~~ll~R~vl~~Lg-~~~~~~~~a~~~aal~fl~--~wl~y--~~~~Rpyalvalla~l~~~~~~ra~~   404 (1084) |
|  | T 7BVE\_B | 330 | MRFPTLLMGLACWWVISREVIPRLG-AAAKHSRAAAWTAAGLFLA--FWLPL--NNGLRPEPIIALGILLTWCSVERGVA   404 (1084) |
|  | T ss\_dssp |  | HHHHHHHHHHHHHHHCCCCCTTTSB-STTTSCSHHHHHHHHHHHH--HHHHH--CSSSSSHHHHHHHHHHHHHHHHHHHH |
|  | T ss\_pred |  | HHHHHHHHHHHHHHHHHHHhHHHHH-HHhccCHHHHHHHHHHHHH--HHHHh--cCCCChHHHHHHHHHHHHHHHHHHHc |
|  |
|  |
|  | Q ss\_pred |  | cCCCCCCCCcchhHHHHHHHHHHHHHHHHhHHHHHHHHHHHHHHHHHhcccccchhHhhccCchhHHHhhHHHHHHHHHH |
|  | Q XP\_016875493.1 | 189 | QGCVGGSFPSTVSPFFLLLSLFLGTCAMLVKETGITVFGVCLVYDLFSLSNKQDKSYLRASSNRNFLLTMRPFLKRAILV   268 (456) |
|  | Q Consensus | 189 | ~~~~~~~~~~~~~~~~~~~~~~~~~la~~~k~~~~~~~~~~~~~~~~~~~~~~~~~~~~~~~~~~~~~~~~~~~~~~~~~   268 (456) |
|  |  |  | ++ +.+++.+++++.++++.+|+++++.+....+......+..+++.... ...... |
|  | T Consensus | 405 | ~~----------r~~~~ala~~~a~la~~~~Ptgl~ala~ll~~~~~l~r~lr~r~~~~---------------~~la~l   459 (1084) |
|  | T 7BVE\_B | 405 | TS----------RLLPVAVAIIIGALTLFSGPTGIAAVGALLVAIGPLKTIVAAHVSRF---------------GYWALL   459 (1084) |
|  | T ss\_dssp |  | HT----------CSHHHHHHHHHHHHHHTSSGGGGGGHHHHHHTSHHHHHHHHHHTTTS---------------CSHHHH |
|  | T ss\_pred |  | CC----------CcHHHHHHHHHHHHHHhhhHHHHHHHHHHHHHHHHHHHHHHhccchh---------------HHHHHH |
|  |
|  |
|  | Q ss\_pred |  | HHHHHHHHHHHHHHhcCCCCccccCCCCcccchhHHHHHHHHHHHHHHHHHHHHHhHHhhccCCCCCCccccchhhHHHH |
|  | Q XP\_016875493.1 | 269 | LSYVLVILYFRLWIMGGSMPLFSEQDNPASFSPYILTRFLTYSYLLAFNVWLLLAPVTLCYDWQVGSIPLVETIWDMRNL   348 (456) |
|  | Q Consensus | 269 | ~~~~~~~~~~~~~~~~~~~~~~~~~~~~~~~~~~~~~~~~~~~~~~~~~~~~~~~~~~~~~~~~~~~~~~~~~~~~~~~~   348 (456) |
|  |  |  | ...++.........+..................................+. ........- |
|  | T Consensus | 460 | a~~lAa~~~~l~~~Fadqsl~~~~~a~~v~~~~gp~l~w~~e~~Ry~~l~g--------------------~~~~gs~ar   519 (1084) |
|  | T 7BVE\_B | 460 | APIAAAGTVTIFLIFRDQTLAAELQASSFKSAVGPSLAWFDEHIRYSRLFT--------------------TSPDGSVAR   519 (1084) |
|  | T ss\_dssp |  | HHHHHHHHTTHHHHTSSSCHHHHHHHHHHHHHHSCCCCGGGTHHHHHHHTS--------------------SSSTTCHHH |
|  | T ss\_pred |  | HHHHHHHHHHHHHHHHhhhHHHHHHHHhhhhccCCCcHhHHHHHHHHHHhc--------------------CCCCCCHHH |
|  |
|  |
|  | Q ss\_pred |  | HHHHHHHHHHHHHHHHHHHHhcccch---HHHHHHHHHHHHHhhHhccccCCCccccccccchHHHHHHHHHHHHHHHHH |
|  | Q XP\_016875493.1 | 349 | ATIFLAVVMALLSLHCLAAFKRLEHK---EVLVGLLFLVFPFIPASNLFFRVGFVVAERVLYMPSMGYCILFVHGLSKLC   425 (456) |
|  | Q Consensus | 349 | ~~~~~~~~~~~~~~~~~~~~~~~~~~---~~~~~~~~~~~~~~~~~~~~~~~~~~~~~Ry~~~~~~~~~ll~~~~~~~~~   425 (456) |
|  |  |  | -...++.++.++.......++++... .....+++..+..+.+..+ ......+|+....+...++++..+..+. |
|  | T Consensus | 520 | r~~vLl~l~~l~~~~~ll~R~rr~~g~~~~~~~~l~~~~~~~l~ll~~----tPtKwthhfg~lag~~~~lla~~~~~~~   595 (1084) |
|  | T 7BVE\_B | 520 | RFAVLTLLLALAVSIAMTLRKGRIPGTALGPSRRIIGITIISFLAMMF----TPTKWTHHFGVFAGLAGCLGALAAVAVT   595 (1084) |
|  | T ss\_dssp |  | HHHHHHHHHHHHHHHHHHHHSSSCTTBCHHHHHHHHHHHHHHHHHGGG----CSSCCSGGGGGGTTHHHHHHHHHHHTTS |
|  | T ss\_pred |  | HHHHHHHHHHHHHHHHHHhhcCCCCCCCCCHHHHHHHHHHHHHHHHhc----CCchHHHHHHhHHHHHHHHHHHHHHHHH |
|  |
|  |
|  | Q ss\_pred |  | HHHhHHhHHHHHHHHHHHHHHHHHHHHHhcC |
|  | Q XP\_016875493.1 | 426 | TWLNRCGATTLIVSTVLLLLLFSWKTVKQNE   456 (456) |
|  | Q Consensus | 426 | ~~~~~~~~~~~~~~~~~~~~~~~~~~~~~~~   456 (456) |
|  |  |  | +...+..+.... ....++++..+.....|. |
|  | T Consensus | 596 | ~~~~r~~r~~~~-~~a~~~~~~ala~~G~N~   625 (1084) |
|  | T 7BVE\_B | 596 | TTAMKSRRNRTV-FGAAVLFVTALSFATVNG   625 (1084) |
|  | T ss\_dssp |  | TTTCCCHHHHHH-HHHHHHHHHHHHTSSCCC |
|  | T ss\_pred |  | HHhccchHHHHH-HHHHHHHHHHHHhccccc |
|  |
| --- | | | |
|  | Template alignmentTemplate 3D StructurePDBe | | |
| 14. | 6SNI\_X Dolichyl pyrophosphate Man9GlcNAc2 alpha-1,3-glucosyltransferase (E.C.2.4.1.267); Glycosyltransferase, Glucosyltransferase, GT-C, N-Glycosylation, MEMBRANE; HET: PTY, Y01;{Saccharomyces cerevisiae}; Related PDB entries: 6SNH\_X | | |
|  | Probability: 99.47%, E-value: 1.5e-10, Score: 108.55, Aligned cols: 344, Identities: 11%, Similarity: -0.031, | | |
|  |
|  | Q ss\_pred |  | CceecccCCCCCCCCccchhhccHHHHHHHHHHHHHHHHHHhhhc------CCCcccccHHHHHhCCCCCCCCCcccccc |
|  | Q XP\_016875493.1 | 1 | MVVTTSARGGGGDRTPSRRRGCGLAPAGAAALLAGASCLCYGRSL------QGEFVHDDVWAIVNNPDVRPGAPLRWGIF   74 (456) |
|  | Q Consensus | 1 | m~~~~~~~~~~~~~~~~~~~~~~~~~~~~~~~l~~~~~~~~~~~~------~~~~~~De~~~~~~a~~~~~~~~~~~~~~   74 (456) |
|  |  |  | +............................+++++.+.+....... ..+...|+..+....+...+.+...+... |
|  | T Consensus | 30 | ~~~~~~~~~~~~~~~~~~~~~~~~~~~l~~i~~~~l~lR~~~~~~~~sg~~~pp~~~D~~~~~~w~~~~~~~~~~~wy~~   109 (562) |
|  | T 6SNI\_X | 30 | PAEESFYASPMYDFLYPFRPVGNQWLPEYIIFVCAVILRCTIGLGPYSGKGSPPLYGDFEAQRHWMEITQHLPLSKWYWY   109 (562) |
|  | T ss\_dssp |  | --------------------------CCSSHHHHHHHHHHHGGGSCCTTSSCSSSCCHHHHHHHHHHHHTTSCTTSTTTS |
|  | T ss\_pred |  | CcccccCCCchhHhhhccCCCCCccHHHHHHHHHHHHHHHHHhcCCCCCCCCCCCCCCHHHHHHHHHHHHhCCHHHhccc |
|  |
|  |
|  | Q ss\_pred |  | cccccccCCCCCCCcccccchHHHHHHHHHHHhC--------------------CCchHHHHHHHHHHHHHHHHH-HHHH |
|  | Q XP\_016875493.1 | 75 | TNDFWGKGMAENTSHKSYRPLCVLTFKLNIFLTG--------------------MNPFYFHAVNIILHCLVTLVL-MYTC   133 (456) |
|  | Q Consensus | 75 | ~~~~~~~~~~~~~~~~~~~Pl~~~~~~~~~~l~g--------------------~~~~~~rl~~~l~~~~~~~~~-~~l~   133 (456) |
|  |  |  | +.+++... |||+..+...+...+.+ .+....|+.++++.+++...+ |.+. |
|  | T Consensus | 110 | ~~~~~~~~---------YPPl~~~~~~~~~~i~~~~~~~~~~l~~~~g~~~~~~~~~~~~rl~~i~~~ll~~~~~~~~~~   180 (562) |
|  | T 6SNI\_X | 110 | DLQYWGLD---------YPPLTAFHSYLLGLIGSFFNPSWFALEKSRGFESPDNGLKTYMRSTVIISDILFYFPAVIYFT   180 (562) |
|  | T ss\_dssp |  | CSTTTCCC---------SCHHHHHHHHHHHHHHHHHCTTTTCSSSSTTCCCTTCCSSSHHHHHHHHHHHHHTHHHHHHHH |
|  | T ss\_pred |  | CccccCCC---------ChHHHHHHHHHHHHHHHHhCHHHHHhcccCCCCCcchHHHHHHHHHHHHHHHHHHHHHHHHHH |
|  |
|  |
|  | Q ss\_pred |  | HHH---hccchHHHHHHHHHHHHCcccHHHHHhh-hhhHHHHHHHHHHHHHHHHHHHHhcCCCCCCCCcchhHHHHHHHH |
|  | Q XP\_016875493.1 | 134 | DKT---VFKNRGLAFVTALLFAVHPIHTEAVAGI-VGRADVLACLLFLLAFLSYNRSLDQGCVGGSFPSTVSPFFLLLSL   209 (456) |
|  | Q Consensus | 134 | ~~~---~~~~~~~a~~aa~l~~~~p~~~~~~~~~-~~~~~~~~~~~~~l~~~~~~~~~~~~~~~~~~~~~~~~~~~~~~~   209 (456) |
|  |  |  | |+. ..++++.+..++++++++|..+...+.. . .|.....+.+++++++.+.+ ...++ |
|  | T Consensus | 181 | ~~~~~~~~~~~~~~~~~~~~~~l~P~~i~~~~~~~q--~d~~~l~l~l~al~~~~~~~-----------------~~~ag   241 (562) |
|  | T 6SNI\_X | 181 | KWLGRYRNQSPIGQSIAASAILFQPSLMLIDHGHFQ--YNSVMLGLTAYAINNLLDEY-----------------YAMAA   241 (562) |
|  | T ss\_dssp |  | HHHHHHHTCCHHHHHHHHHHHHCCHHHHHHHTTTCC--CHHHHHHHHHHHHHHHHHTC-----------------HHHHH |
|  | T ss\_pred |  | HHHhhcCCCChhHHHHHHHHHHhCHHHHHhhcccch--hHHHHHHHHHHHHHHHHCCC-----------------hHHHH |
|  |
|  |
|  | Q ss\_pred |  | HHHHHHHHhHHHHHHHHHHHHHHHHHhcccccchhHhhccCchhHHHhhHHHHHHHHHHHHHHHHHHHHHHHHhcC---- |
|  | Q XP\_016875493.1 | 210 | FLGTCAMLVKETGITVFGVCLVYDLFSLSNKQDKSYLRASSNRNFLLTMRPFLKRAILVLSYVLVILYFRLWIMGG----   285 (456) |
|  | Q Consensus | 210 | ~~~~la~~~k~~~~~~~~~~~~~~~~~~~~~~~~~~~~~~~~~~~~~~~~~~~~~~~~~~~~~~~~~~~~~~~~~~----   285 (456) |
|  |  |  | +++++++.+|+..+.+.++++++.+....++++++.++ .............+.+..+...+ |
|  | T Consensus | 242 | i~~~lal~~K~~~l~~~p~~~~~ll~~~~~~~~~~~~~---------------~~~~~~~~~~~~~l~~~Pf~~~~~~~~   306 (562) |
|  | T 6SNI\_X | 242 | VCFVLSICFKQMALYYAPIFFAYLLSRSLLFPKFNIAR---------------LTVIAFATLATFAIIFAPLYFLGGGLK   306 (562) |
|  | T ss\_dssp |  | HHHHHHHTTCGGGTTSHHHHHHHHHCCCCCSSCCCHHH---------------HHHHHHHHHHHHHHHHHHHHTTTCSHH |
|  | T ss\_pred |  | HHHHHHHHhhHHHHHHHHHHHHHHHHHHcCCCCCcHHH---------------HHHHHHHHHHHHHHHHHHHHHhcCCHH |
|  |
|  |
|  | Q ss\_pred |  | --CCCccccCCCCcccchhHHHHHHHHHHHHHHHHHHHHHhHHhhccCCCCCCccccchhhHHHHHHHHHHHHHHHHHHH |
|  | Q XP\_016875493.1 | 286 | --SMPLFSEQDNPASFSPYILTRFLTYSYLLAFNVWLLLAPVTLCYDWQVGSIPLVETIWDMRNLATIFLAVVMALLSLH   363 (456) |
|  | Q Consensus | 286 | --~~~~~~~~~~~~~~~~~~~~~~~~~~~~~~~~~~~~~~~~~~~~~~~~~~~~~~~~~~~~~~~~~~~~~~~~~~~~~~   363 (456) |
|  |  |  | ............+.......+............. ................+..+... |
|  | T Consensus | 307 | ~~~~~~~~~fp~~rgl~~~~~~n~w~~~~~~~~~~~---------------------~~~~~~~~~~~~~~~~l~~l~~~   365 (562) |
|  | T 6SNI\_X | 307 | NIHQCIHRIFPFARGIFEDKVANFWCVTNVFVKYKE---------------------RFTIQQLQLYSLIATVIGFLPAM   365 (562) |
|  | T ss\_dssp |  | HHHHHHHHHSCCCCSSSCSCCSSSHHHHTTTSCGGG---------------------TSCHHHHHHHHHHHHHHHHHHHH |
|  | T ss\_pred |  | HHHHHHHHHcCCCcccccchhhhHHHHHHHHHHHHh---------------------hCCHHHHHHHHHHHHHHHHHHHH |
|  |
|  |
|  | Q ss\_pred |  | HHHHHhcccchHHHHHHHHHHHHHhhHhccccCCCccccccc-cchHHHHHHHH |
|  | Q XP\_016875493.1 | 364 | CLAAFKRLEHKEVLVGLLFLVFPFIPASNLFFRVGFVVAERV-LYMPSMGYCIL   416 (456) |
|  | Q Consensus | 364 | ~~~~~~~~~~~~~~~~~~~~~~~~~~~~~~~~~~~~~~~~Ry-~~~~~~~~~ll   416 (456) |
|  |  |  | ....++++............+..++. +...+++| +++.+|...+. |
|  | T Consensus | 366 | ~~~~~~~~~~~~~~~~~~~~l~~flf--------s~~vhekyill~llPl~ll~   411 (562) |
|  | T 6SNI\_X | 366 | IMTLLHPKKHLLPYVLIACSMSFFLF--------SFQVHEKTILIPLLPITLLY   411 (562) |
|  | T ss\_dssp |  | HHHHTSCCSSSHHHHHHHHHHHHHHH--------CSSCCSSCCHHHHHHHHHGG |
|  | T ss\_pred |  | HHHHhCCCcchHHHHHHHHHHHHHHh--------chhcCchhcHHHHHHHHHHh |
|  |
| --- | | | |
|  | Template alignmentTemplate 3D StructurePDBe | | |
| 15. | 7BVC\_A Integral membrane indolylacetylinositol arabinosyltransferase EmbA; Mycobacterium smegmatis, cell wall synthesis; HET: 95E, PNS, CDL, F8L;{Mycolicibacterium smegmatis MC2 155}; Related PDB entries: 7BVG\_A | | |
|  | Probability: 99.33%, E-value: 2.5e-9, Score: 104.16, Aligned cols: 357, Identities: 8%, Similarity: -0.083, | | |
|  |
|  | Q ss\_pred |  | HHHHHHHHhhhcCCCcccccHHHHHhCCCCCCCCCcccccccccccccCCCCCCCcccccchHHHHHHHHHHHhCCCchH |
|  | Q XP\_016875493.1 | 34 | AGASCLCYGRSLQGEFVHDDVWAIVNNPDVRPGAPLRWGIFTNDFWGKGMAENTSHKSYRPLCVLTFKLNIFLTGMNPFY   113 (456) |
|  | Q Consensus | 34 | ~~~~~~~~~~~~~~~~~~De~~~~~~a~~~~~~~~~~~~~~~~~~~~~~~~~~~~~~~~~Pl~~~~~~~~~~l~g~~~~~   113 (456) |
|  |  |  | .++...+.......+...||.++...+++..+.+.....+..-+ ......-+|+.++..+..+ |.++.. |
|  | T Consensus | 251 | ~vV~~~L~~W~~igp~~~DDg~~~~~ar~~~~~G~~gny~r~~~----------~~eapf~~yY~ll~~w~~v-g~s~~~   319 (1088) |
|  | T 7BVC\_A | 251 | TGVIGGLLIWHIVGAPTSDDGYNMTIARVASEAGYTTNYYRYFG----------ASEAPFDWYQSVLSHLASI-STAGVW   319 (1088) |
|  | T ss\_dssp |  | HHHHHHHHSTTTSCCCCTTHHHHHHHHHHSSSSSSCBCSSSGGG----------CBCTTSCHHHHHHHHHTTT-CCCHHH |
|  | T ss\_pred |  | HHHHHHHHHHHHhCCCCcchhHHHHHHHHHHHhcCHHHHHHHhc----------CCCcCCHHHHHHHHHHHhc-ccchHH |
|  |
|  |
|  | Q ss\_pred |  | HHHHHHHHHHHHHHHH-----HHHHHHHhccchHHHHHHHHHHH--HCcccHHHHHhhhhhHHHHHHHHHHHHHHHHHHH |
|  | Q XP\_016875493.1 | 114 | FHAVNIILHCLVTLVL-----MYTCDKTVFKNRGLAFVTALLFA--VHPIHTEAVAGIVGRADVLACLLFLLAFLSYNRS   186 (456) |
|  | Q Consensus | 114 | ~rl~~~l~~~~~~~~~-----~~l~~~~~~~~~~~a~~aa~l~~--~~p~~~~~~~~~~~~~~~~~~~~~~l~~~~~~~~   186 (456) |
|  |  |  | .|++++++++++..++ ..+. +....++...+.+++.+. ..| + ....|+|.+..++.+++.+++.+. |
|  | T Consensus | 320 | lRLPSllagl~tw~llsR~vl~~Lg-~~~~~~~~a~~aaal~fla~wlP----y--~~~~Rpe~~val~~~~a~~~~~ra   392 (1088) |
|  | T 7BVC\_A | 320 | MRLPATAAAIATWLIISRCVLPRIG-RRVAANRVAMLTAGATFLAAWLP----F--NNGLRPEPLIAFAVITVWMLVENS   392 (1088) |
|  | T ss\_dssp |  | HTGGGTHHHHHHHHHCCCCCHHHHC-HHHHHCHHHHHHHHHHHHHHHTT----T--CSSSSSHHHHHHHHHHHHHHHHHH |
|  | T ss\_pred |  | hHHHHHHHHHHHHHHHHHHHHHHHh-HhhcccHHHHHHHHHHHHHHHHH----h--cCCCChHHHHHHHHHHHHHHHHHH |
|  |
|  |
|  | Q ss\_pred |  | HhcCCCCCCCCcchhHHHHHHHHHHHHHHHHhHHHHHHHHHHHHHHHHHhcccccchhHhhccCchhHHHhhHHHHHHHH |
|  | Q XP\_016875493.1 | 187 | LDQGCVGGSFPSTVSPFFLLLSLFLGTCAMLVKETGITVFGVCLVYDLFSLSNKQDKSYLRASSNRNFLLTMRPFLKRAI   266 (456) |
|  | Q Consensus | 187 | ~~~~~~~~~~~~~~~~~~~~~~~~~~~la~~~k~~~~~~~~~~~~~~~~~~~~~~~~~~~~~~~~~~~~~~~~~~~~~~~   266 (456) |
|  |  |  | .+++ +..++..+.++.+++..+++++++.+..+++......+.-+++.... .... |
|  | T Consensus | 393 | ~~~~----------r~~~~a~a~~~aala~~~hPtGl~a~a~ll~~~~~l~r~~r~r~~~~---------------~~~a   447 (1088) |
|  | T 7BVC\_A | 393 | IGTR----------RLWPAAVAIVIAMFSVTLAPQGLIALAPLLVGARAIGRVVTARRAGT---------------GILA   447 (1088) |
|  | T ss\_dssp |  | HTTT----------CSHHHHHHHHHHHHHHTTCGGGGGGGHHHHHHHHHHHHHHGGGTTTT---------------CSHH |
|  | T ss\_pred |  | hcCC----------CcHHHHHHHHHHHHHHhhcHHHHHHHHHHHHHHHHHHHHHHHhhccc---------------cHHH |
|  |
|  |
|  | Q ss\_pred |  | HHHHHHHHHHHHHHHHhcCCCCccccCCCCcccchhHHHHHHHHHHHHHHHHHHHHHhHHhhccCCCCCCccccchhhHH |
|  | Q XP\_016875493.1 | 267 | LVLSYVLVILYFRLWIMGGSMPLFSEQDNPASFSPYILTRFLTYSYLLAFNVWLLLAPVTLCYDWQVGSIPLVETIWDMR   346 (456) |
|  | Q Consensus | 267 | ~~~~~~~~~~~~~~~~~~~~~~~~~~~~~~~~~~~~~~~~~~~~~~~~~~~~~~~~~~~~~~~~~~~~~~~~~~~~~~~~   346 (456) |
|  |  |  | ..+..++.........+.+..-..................+.+-. ....++. ...+...+.. |
|  | T Consensus | 448 | ~~a~~laa~~~~l~~~F~dqtl~~~~~a~r~~~~~gp~~~w~~E~-~RY~~L~-----------------~~~~~~Gs~a   509 (1088) |
|  | T 7BVC\_A | 448 | SLAPLAASVAVVFVIIFRDQTLATVAESVRIKYVVGPTIPWYQEF-LRYYFLT-----------------VEDSVDGSLT   509 (1088) |
|  | T ss\_dssp |  | HHHHHHHHHTTHHHHHTTSSCHHHHHHHHHHHHHHSCCCCTTCCT-HHHHHHS-----------------CSSCSSSCTT |
|  | T ss\_pred |  | HHHHHHHHHHHHHHHHhCCCcHHHHHHHHhHHhhhCCCChHHchh-HHHHHHH-----------------ccCCCCCCHH |
|  |
|  |
|  | Q ss\_pred |  | HHHHHHHHHHHHHHHHHHHHHHhcccch--HHHHHHHHHHHHHhhHhccccCCCccccccccchHHHHHHHHHHHHHHHH |
|  | Q XP\_016875493.1 | 347 | NLATIFLAVVMALLSLHCLAAFKRLEHK--EVLVGLLFLVFPFIPASNLFFRVGFVVAERVLYMPSMGYCILFVHGLSKL   424 (456) |
|  | Q Consensus | 347 | ~~~~~~~~~~~~~~~~~~~~~~~~~~~~--~~~~~~~~~~~~~~~~~~~~~~~~~~~~~Ry~~~~~~~~~ll~~~~~~~~   424 (456) |
|  |  |  | .-...+..+..++........+++.... .....+....+..+..+.+ ......+|+-...+....+++.....+ |
|  | T Consensus | 510 | rR~~VLl~l~~l~~~~~~l~rrrr~~g~~~~~~~~l~~~~~~~~~lL~~----tPtKWthHfGa~Ag~gaal~a~~~va~   585 (1088) |
|  | T 7BVC\_A | 510 | RRFAVLVLLLCLFGLIMVLLRRGRVPGAVSGPLWRLCGSTAIGLLLLIL----TPTKWAIQFGAFAGLAGALGGVTAFAF   585 (1088) |
|  | T ss\_dssp |  | HHHHHHHHHHHHHHHHHHHHHSSCCTTSCHHHHHHHHHHHHHHHHHTTS----CSCCCSGGGGGGHHHHHHHHHHHHHHH |
|  | T ss\_pred |  | HHHHHHHHHHHHHHHHHHHHhcCCCCCCCccHHHHHHHHHHHHHHHHHH----CcchHHHHHHHHHHHHHHHHHHHHHHH |
|  |
|  |
|  | Q ss\_pred |  | HHHHhHHhHHHHHHHHHHHHHHHHHHHHHhcC |
|  | Q XP\_016875493.1 | 425 | CTWLNRCGATTLIVSTVLLLLLFSWKTVKQNE   456 (456) |
|  | Q Consensus | 425 | ~~~~~~~~~~~~~~~~~~~~~~~~~~~~~~~~   456 (456) |
|  |  |  | .+...+..+...... ..++++..+.....|. |
|  | T Consensus | 586 | ~~~~~r~~r~~~~~~-a~~~~~~alaf~G~N~   616 (1088) |
|  | T 7BVC\_A | 586 | ARVGLHSRRNLALYV-TALLFILAWATSGLNG   616 (1088) |
|  | T ss\_dssp |  | HHHTTSCHHHHHHHH-HHHHHHHHHHTTSCCC |
|  | T ss\_pred |  | HHhccChHHHHHHHH-HHHHHHHHHHhhcccc |
|  |
| --- | | | |
|  | Template alignmentTemplate 3D StructurePDBe | | |
| 16. | 6P25\_B Dolichyl-diphosphooligosaccharide--protein glycosyltransferase subunits (E.C.2.4.99.18); complex, TRANSFERASE, glycosylation; HET: NAG, CPL, NNM; 3.2A {Saccharomyces cerevisiae W303}; Related PDB entries: 6P2R\_B ; Related PDB entries: 6P2R\_B ; Related PDB entries: 6P2R\_B | | |
|  | Probability: 75.12%, E-value: 53, Score: 33.25, Aligned cols: 107, Identities: 8%, Similarity: -0.046, | | |
|  |
|  | Q ss\_pred |  | hhHHHHHHHHHHHHHHHHHHHHHHHHhcccchHH------------HHHHHHHHHHHhhHhccccCCCccccccccchHH |
|  | Q XP\_016875493.1 | 343 | WDMRNLATIFLAVVMALLSLHCLAAFKRLEHKEV------------LVGLLFLVFPFIPASNLFFRVGFVVAERVLYMPS   410 (456) |
|  | Q Consensus | 343 | ~~~~~~~~~~~~~~~~~~~~~~~~~~~~~~~~~~------------~~~~~~~~~~~~~~~~~~~~~~~~~~~Ry~~~~~   410 (456) |
|  |  |  | +....+.....++...+.........+++..... .+.++..++..+|+... +.....+|.+|++ |
|  | T Consensus | 607 | Np~iww~~~~~~~~~~~~~~~~~~~~~r~~~~~~~~~~~~~~~~~~~~~~~g~~~~ylP~~~~----~R~~fl~hYlpal   682 (759) |
|  | T 6P25\_B | 607 | TPASTWASSVAVLAFMATVVILLIRWQRQYVDLRNPSNWNVFLMGGFYPLLAWGLHYMPFVIM----SRVTYVHHYLPAL   682 (759) |
|  | T ss\_dssp |  | CTTHHHHHHHHHHHHHHHHHHHHHHHHTTCCCCCSHHHHHCCCCCCCHHHHHHHHHHHHHHHS----CSCBCGGGHHHHH |
|  | T ss\_pred |  | CHHHHHHHHHHHHHHHHHHHHHHHHHhcCCCCCCCchhHHHHHHHhHHHHHHHHHhcchHHcc----cCcccHHHHHHHH |
|  |
|  |
|  | Q ss\_pred |  | HHHHHHHHHHHHHHHHHHh----HHhHHHHHHHHHHHHHHHHHHHHH |
|  | Q XP\_016875493.1 | 411 | MGYCILFVHGLSKLCTWLN----RCGATTLIVSTVLLLLLFSWKTVK   453 (456) |
|  | Q Consensus | 411 | ~~~~ll~~~~~~~~~~~~~----~~~~~~~~~~~~~~~~~~~~~~~~   453 (456) |
|  |  |  | ++.+++++..+..+.++.+ ++..+.....+++++++.....+. |
|  | T Consensus | 683 | ~f~il~~~~~~~~~~~~~~~~~~~~~~~~~~~~~~~~~~~~~f~~~~   729 (759) |
|  | T 6P25\_B | 683 | YFALIILAYCFDAGLQKWSRSKCGRIMRFVLYAGFMALVIGCFWYFS   729 (759) |
|  | T ss\_dssp |  | HHHHHHHHHHHHTSSSGGGGSHHHHHHHHHHHHHHHHHHHHHHHHTT |
|  | T ss\_pred |  | HHHHHHHHHHHHHHHHhcccccccHHHHHHHHHHHHHHHHHHHHHHH |
|  |
| --- | | | |
|  | Template alignmentTemplate 3D StructurePDBe | | |
| 17. | 6S7T\_A Dolichyl-diphosphooligosaccharide--protein glycosyltransferase subunit STT3B (E.C.2.4.99.18); N-glycosylation, Oligosaccharyltransferase, OSTB, TRANSFERASE; HET: 0K3, KZB, NAG, EGY, MAN, BMA; 3.5A {Homo sapiens} | | |
|  | Probability: 30.95%, E-value: 650, Score: 25.88, Aligned cols: 214, Identities: 11%, Similarity: 0.002, | | |
|  |
|  | Q ss\_pred |  | ccchHHHHHHHHHHHHCc------ccHHHHHhhhhhHHHHHHHHHHHHHHHHHHHHhcCCCCCCCCcchhHHHHHHHHHH |
|  | Q XP\_016875493.1 | 138 | FKNRGLAFVTALLFAVHP------IHTEAVAGIVGRADVLACLLFLLAFLSYNRSLDQGCVGGSFPSTVSPFFLLLSLFL   211 (456) |
|  | Q Consensus | 138 | ~~~~~~a~~aa~l~~~~p------~~~~~~~~~~~~~~~~~~~~~~l~~~~~~~~~~~~~~~~~~~~~~~~~~~~~~~~~   211 (456) |
|  |  |  | ...+...++.+.+..+.. ..... ... ...+...+.++.++.+.+...++ +...+....++ |
|  | T Consensus | 141 | ~~~P~~~~l~a~~~~l~g~~~~~~~~~~~--~~l--~~~l~~~l~~~~~y~l~r~l~~~----------~~allaall~a   206 (826) |
|  | T 6S7T\_A | 141 | TVYPGLMITAGLIHWILNTLNITVHIRDV--CVF--LAPTFSGLTSISTFLLTRELWNQ----------GAGLLAACFIA   206 (826) |
|  | T ss\_dssp |  | SSCTTHHHHHHHHHHHHHHTTCCCCHHHH--HHT--HHHHHHHHHHHHHHHHHHHHSCH----------HHHHHHHHHTT |
|  | T ss\_pred |  | CCchHHHHHHHHHHHHHHhcCCCCcHHHH--HHH--HHHHHHHHHHHHHHHHHHHHcCc----------hHHHHHHHHHH |
|  |
|  |
|  | Q ss\_pred |  | HHHHHHhHH--------HHHHHHHHHHHHHHHhcccccchhHhhccCchhHHHhhHHHHHHHHHHHHHHHHHHHHHHHHh |
|  | Q XP\_016875493.1 | 212 | GTCAMLVKE--------TGITVFGVCLVYDLFSLSNKQDKSYLRASSNRNFLLTMRPFLKRAILVLSYVLVILYFRLWIM   283 (456) |
|  | Q Consensus | 212 | ~~la~~~k~--------~~~~~~~~~~~~~~~~~~~~~~~~~~~~~~~~~~~~~~~~~~~~~~~~~~~~~~~~~~~~~~~   283 (456) |
|  |  |  | +.-...... ....+...+.++.+....++++.+ ..++..++..+......... |
|  | T Consensus | 207 | ~~p~~~~~s~~~~~~~e~~~~~~~~l~l~~~~~~~~~~~~~------------------~~~l~gl~~gla~~~~~~~~-   267 (826) |
|  | T 6S7T\_A | 207 | IVPGYISRSVAGSFDNEGIAIFALQFTYYLWVKSVKTGSVF------------------WTMCCCLSYFYMVSAWGGYV-   267 (826) |
|  | T ss\_dssp |  | TCHHHHGGGSTTCCCSHHHHHHHHHHHHHHHHHHHHHCCHH------------------HHHHHHHHHHHHHHHCTTHH- |
|  | T ss\_pred |  | HHHHHHHhhhccCchHHHHHHHHHHHHHHHHHHHhccCcHH------------------HHHHHHHHHHHHHHhcccHH- |
|  |
|  |
|  | Q ss\_pred |  | cCCCCccccCCCCcccchhHHHHHHHHHHHHHHHHHHHHHhHHhhccCCCCCCccccchhhHHHHHHHHHHHHHHHHHHH |
|  | Q XP\_016875493.1 | 284 | GGSMPLFSEQDNPASFSPYILTRFLTYSYLLAFNVWLLLAPVTLCYDWQVGSIPLVETIWDMRNLATIFLAVVMALLSLH   363 (456) |
|  | Q Consensus | 284 | ~~~~~~~~~~~~~~~~~~~~~~~~~~~~~~~~~~~~~~~~~~~~~~~~~~~~~~~~~~~~~~~~~~~~~~~~~~~~~~~~   363 (456) |
|  |  |  | .....+.+..+. |
|  | T Consensus | 268 | --------------------------------------------------------------------~~~~~~~l~~~~   279 (826) |
|  | T 6S7T\_A | 268 | --------------------------------------------------------------------FIINLIPLHVFV   279 (826) |
|  | T ss\_dssp |  | --------------------------------------------------------------------HHHHHHHHHHHH |
|  | T ss\_pred |  | --------------------------------------------------------------------HHHHHHHHHHHH |
|  |
|  |
|  | Q ss\_pred |  | HHHHHhcccchHHHHHHHHHHHHHhhHhccccCCCccccccccchHHHHHHHHHHHHHHHHHHHHhHHhHHHHHHHHHHH |
|  | Q XP\_016875493.1 | 364 | CLAAFKRLEHKEVLVGLLFLVFPFIPASNLFFRVGFVVAERVLYMPSMGYCILFVHGLSKLCTWLNRCGATTLIVSTVLL   443 (456) |
|  | Q Consensus | 364 | ~~~~~~~~~~~~~~~~~~~~~~~~~~~~~~~~~~~~~~~~Ry~~~~~~~~~ll~~~~~~~~~~~~~~~~~~~~~~~~~~~   443 (456) |
|  |  |  | ....++++++..........+...+.........+.........+......+..........+...++...........+ |
|  | T Consensus | 280 | ~~~~~~~~~~~~~~~~~~~~~~~~~~~~~p~~~~~~~~~~~~~~~~~~~~~~~~~~~~~~~~~~~~~~~~~~~~~~~~~~   359 (826) |
|  | T 6S7T\_A | 280 | LLLMQRYSKRVYIAYSTFYIVGLILSMQIPFVGFQPIRTSEHMAAAGVFALLQAYAFLQYLRDRLTKQEFQTLFFLGVSL   359 (826) |
|  | T ss\_dssp |  | HHHTTCCCHHHHHHHHHHHHHHHHHHTTSTTTTTHHHHBSSTHHHHHHHHHHHHHHHHHHHHHHSCSTTTHHHHHHHHHH |
|  | T ss\_pred |  | HHHhccCChhHHHHHHHHHHHHHHHHhccccCCCCcccchHHHHHHHHHHHHHHHHHHHHHHHhcCHHHHHHHHHHHHHH |
|  |
|  |
|  | Q ss\_pred |  | HHHHHHHHH |
|  | Q XP\_016875493.1 | 444 | LLLFSWKTV   452 (456) |
|  | Q Consensus | 444 | ~~~~~~~~~   452 (456) |
|  |  |  | .+....... |
|  | T Consensus | 360 | ~~~~~~~~~   368 (826) |
|  | T 6S7T\_A | 360 | AAGAVFLSV   368 (826) |
|  | T ss\_dssp |  | HHHHHHHHH |
|  | T ss\_pred |  | HHHHHHHHH |
|  |

---

If you use HHpred on our Toolkit for your research, please cite as appropriate:

A Completely Reimplemented MPI Bioinformatics Toolkit
with a New HHpred Server at its Core.  
Zimmermann L, Stephens A, Nam SZ, Rau D,
Kübler J, Lozajic M, Gabler F, Söding J, Lupas AN, Alva V.
J Mol Biol. 2018 Jul 20. S0022-2836(17)30587-9.

  

Protein homology detection by HMM-HMM comparison.  
Söding J. Bioinformatics. 2005 Apr 1;21(7):951-60.  
  
Fast and accurate automatic structure prediction with HHpred.  
Hildebrand A, Remmert M, Biegert A, Söding J. Proteins. 2009;77 Suppl 9:128-32.  
  
Automatic Prediction of Protein 3D Structures by Probabilistic Multi-template Homology Modeling.  
Meier A, Söding J. PLoS Comput Biol. 2015 Oct 23;11(10):e1004343.

Download

---

If you use HHpred on our Toolkit for your research, please cite as appropriate:

A Completely Reimplemented MPI Bioinformatics Toolkit
with a New HHpred Server at its Core.  
Zimmermann L, Stephens A, Nam SZ, Rau D,
Kübler J, Lozajic M, Gabler F, Söding J, Lupas AN, Alva V.
J Mol Biol. 2018 Jul 20. S0022-2836(17)30587-9.

  

Protein homology detection by HMM-HMM comparison.  
Söding J. Bioinformatics. 2005 Apr 1;21(7):951-60.  
  
Fast and accurate automatic structure prediction with HHpred.  
Hildebrand A, Remmert M, Biegert A, Söding J. Proteins. 2009;77 Suppl 9:128-32.  
  
Automatic Prediction of Protein 3D Structures by Probabilistic Multi-template Homology Modeling.  
Meier A, Söding J. PLoS Comput Biol. 2015 Oct 23;11(10):e1004343.

Loading...

---

If you use HHpred on our Toolkit for your research, please cite as appropriate:

A Completely Reimplemented MPI Bioinformatics Toolkit
with a New HHpred Server at its Core.  
Zimmermann L, Stephens A, Nam SZ, Rau D,
Kübler J, Lozajic M, Gabler F, Söding J, Lupas AN, Alva V.
J Mol Biol. 2018 Jul 20. S0022-2836(17)30587-9.

  

Protein homology detection by HMM-HMM comparison.  
Söding J. Bioinformatics. 2005 Apr 1;21(7):951-60.  
  
Fast and accurate automatic structure prediction with HHpred.  
Hildebrand A, Remmert M, Biegert A, Söding J. Proteins. 2009;77 Suppl 9:128-32.  
  
Automatic Prediction of Protein 3D Structures by Probabilistic Multi-template Homology Modeling.  
Meier A, Söding J. PLoS Comput Biol. 2015 Oct 23;11(10):e1004343.

Loading hits...

---

If you use HHpred on our Toolkit for your research, please cite as appropriate:

A Completely Reimplemented MPI Bioinformatics Toolkit
with a New HHpred Server at its Core.  
Zimmermann L, Stephens A, Nam SZ, Rau D,
Kübler J, Lozajic M, Gabler F, Söding J, Lupas AN, Alva V.
J Mol Biol. 2018 Jul 20. S0022-2836(17)30587-9.

  

Protein homology detection by HMM-HMM comparison.  
Söding J. Bioinformatics. 2005 Apr 1;21(7):951-60.  
  
Fast and accurate automatic structure prediction with HHpred.  
Hildebrand A, Remmert M, Biegert A, Söding J. Proteins. 2009;77 Suppl 9:128-32.  
  
Automatic Prediction of Protein 3D Structures by Probabilistic Multi-template Homology Modeling.  
Meier A, Söding J. PLoS Comput Biol. 2015 Oct 23;11(10):e1004343.

Loading hits...

---

If you use HHpred on our Toolkit for your research, please cite as appropriate:

A Completely Reimplemented MPI Bioinformatics Toolkit
with a New HHpred Server at its Core.  
Zimmermann L, Stephens A, Nam SZ, Rau D,
Kübler J, Lozajic M, Gabler F, Söding J, Lupas AN, Alva V.
J Mol Biol. 2018 Jul 20. S0022-2836(17)30587-9.

  

Protein homology detection by HMM-HMM comparison.  
Söding J. Bioinformatics. 2005 Apr 1;21(7):951-60.  
  
Fast and accurate automatic structure prediction with HHpred.  
Hildebrand A, Remmert M, Biegert A, Söding J. Proteins. 2009;77 Suppl 9:128-32.  
  
Automatic Prediction of Protein 3D Structures by Probabilistic Multi-template Homology Modeling.  
Meier A, Söding J. PLoS Comput Biol. 2015 Oct 23;11(10):e1004343.

- Help
- FAQ
- Privacy Policy
- Imprint
- Contact Us
- Cite Us
- Recent Updates

© 2008-2020, Dept. of Protein Evolution, Max Planck Institute for Developmental Biology, Tübingen

Template 3D Structure: 
×

Loading...
