# Supplementary material for: Conserved sequence motifs in human TMTC1, TMTC2, TMTC3, and TMTC4, new O-mannosyltransferases from the GT-C/PMT clan, are rationalized as ligand binding sites
Source: Biol Direct. 2021 Jan 12;16:4. doi: 10.1186/s13062-021-00291-w (PMC7801869; doi:10.1186/s13062-021-00291-w)
Supplement: Supplementary file 3 — Additional file 3. HHPred outputs when searching TMTCs against Pfam or PDB structures. The compressed library file AF3-2020-06-HHPred-TMTCs.zip contains the outputs when running the four human TMTC sequences as input of HHPred against PDB sequences and against Pfam domains (as of 23rd of June 2020). [file 13062_2021_291_MOESM3_ESM.zip › AF3-2020-06-HHPred-TMTCs/HHPred_TMTC1_PFam.html]

HHpred | Bioinformatics Toolkit          **We're sorry but the Toolkit doesn't work properly without JavaScript enabled. Please enable it to continue.**

Sign In

- Search
- Alignment
- Sequence Analysis
- 2ary Structure
- 3ary Structure
- Classification
- Utils

- HHblits
- HHpred
- HMMER
- PatternSearch
- ProtBLAST/PSI-BLAST

Nothing found.

###### Tools

###### Jobs

ID

Date

Tool

2161064HHPR

# HHpred

Job ID: 2161064,Created: 14 minutes ago

- Input
- Parameters
- Results
- Raw Output
- Probability Plot
- Query Template MSA
- Query MSA

>XP\_016875493.1\_TMTC1\_Homo sapiens 1..456
MVVTTSARGGGGDRTPSRRRGCGLAPAGAAALLAGASCLCYGRSLQGEFVHDDVWAIVNNPDVRPGAPLRWGIFTNDFWG
KGMAENTSHKSYRPLCVLTFKLNIFLTGMNPFYFHAVNIILHCLVTLVLMYTCDKTVFKNRGLAFVTALLFAVHPIHTEA
VAGIVGRADVLACLLFLLAFLSYNRSLDQGCVGGSFPSTVSPFFLLLSLFLGTCAMLVKETGITVFGVCLVYDLFSLSNK
QDKSYLRASSNRNFLLTMRPFLKRAILVLSYVLVILYFRLWIMGGSMPLFSEQDNPASFSPYILTRFLTYSYLLAFNVWL
LLAPVTLCYDWQVGSIPLVETIWDMRNLATIFLAVVMALLSLHCLAAFKRLEHKEVLVGLLFLVFPFIPASNLFFRVGFV
VAERVLYMPSMGYCILFVHGLSKLCTWLNRCGATTLIVSTVLLLLLFSWKTVKQNE

Paste ExampleUpload File

Protein FASTA

Align two sequences/MSAs

Select structural/domain databases

Pfam-A\_v33.1

- PDB\_mmCIF70\_29\_May (default)
- PDB\_mmCIF30\_29\_May
- SCOPe70\_2.07
- ECOD\_ECOD\_F70\_20200207
- COG\_KOG\_v1.0
- Pfam-A\_v33.1
- NCBI\_Conserved\_Domains(CD)\_v3.18
- SMART\_v6.0
- TIGRFAMs\_v15.0
- PRK\_v6.9
- No elements found. Consider changing the search query.
- List is empty.

Select proteomes

Select options

- Euk\_Arabidopsis\_thaliana\_TAIR10\_20\_Jun\_2017
- Euk\_Bombyx\_mori\_p50T\_Dazao\_06\_May\_2019
- Euk\_Brachypodium\_distachyon\_23\_Aug\_2017
- Euk\_Caenorhabditis\_elegans\_18\_Jul\_2017
- Euk\_Capsaspora\_owczarzaki\_ATCC\_30864\_23\_Mar\_2020
- Euk\_Chaetomium\_thermophilum\_29\_Jun\_2017
- Euk\_Chlamydomonas\_reinhardtii\_27\_Jul\_2017
- Euk\_Entamoeba\_histolytica\_HM1\_IMSS\_22\_Mar\_2017
- Euk\_Dictyostelium\_discoideum\_AX4\_19\_Sep\_2017
- Euk\_Drosophila\_melanogaster\_19\_Jul\_2017
- Euk\_Giardia\_lamblia\_ATCC\_50803\_31\_Aug\_2017
- Euk\_Homo\_sapiens\_04\_Jul\_2017
- Euk\_Physcomitrella\_patens\_28\_Aug\_2017
- Euk\_Plasmodium\_falciparum\_3D7\_7\_Jun\_2017
- Euk\_Saccharomyces\_cerevisiae\_S288c\_11\_Mar\_2017
- Euk\_Schizosaccharomyces\_pombe\_19\_Sep\_2017
- Euk\_Solanum\_lycopersicum\_28\_Jul\_2019
- Euk\_Tetrahymena\_thermophila\_SB210\_22\_Aug\_2017
- Euk\_Toxoplasma\_gondii\_ME49\_10\_May\_2018
- Euk\_Trichomonas\_vaginalis\_G3\_21\_Nov\_2018
- Euk\_Trypanosoma\_brucei\_gambiense\_DAL972\_28\_Mar\_2017
- Euk\_Ustilago\_maydis\_521\_29\_May\_2017
- Euk\_Paramecium\_tetraurelia\_9\_Dec\_2018
- Arc\_Archaeoglobus\_fulgidus\_DSM\_4304\_5\_Dec\_2017
- Arc\_Halobacterium\_jilantaiense\_5\_Dec\_2017
- Arc\_Lokiarchaeum\_sp\_GC14\_75\_31\_Oct\_2018
- Arc\_Methanocaldococcus\_jannaschii\_DSM\_2661\_5\_Dec\_2017
- Arc\_Methanosarcina\_mazei\_S\_6\_17\_Mar\_2017
- Arc\_Methanothermus\_fervidus\_DSM\_2088\_5\_Dec\_2017
- Arc\_Pyrococcus\_horikoshii\_OT3\_5\_Dec\_2017
- Arc\_Sulfolobus\_solfataricus\_5\_Dec\_2017
- Arc\_Thermoplasma\_acidophilum\_DSM\_1728\_7\_Dec\_2017
- Bac\_Acinetobacter\_baumannii\_29\_Mar\_2018
- Bac\_Aquifex\_aeolicus\_VF5\_19\_Sep\_2017
- Bac\_Bacillus\_subtilis\_subsp\_subtilis\_str168\_19\_Mar\_2017
- Bac\_Bacteriovorax\_sp\_DB6\_IX\_1\_Jun\_2018
- Bac\_Bdellovibrio\_bacteriovorus\_HD100\_1\_Jun\_2018
- Bac\_Christensenella\_minuta\_2\_Apr\_2019
- Bac\_Deinococcus\_radiodurans\_R1\_19\_Sep\_2017
- Bac\_Enterococcus\_faecalis\_13\_SD\_W\_01\_1\_Jun\_2018
- Bac\_Escherichia\_coli\_K12\_07\_Mar\_2017
- Bac\_Fischerella\_muscicola\_PCC\_7414\_24\_Sep\_2017
- Bac\_Frankia\_alni\_ACN14a\_24\_Sep\_2017
- Bac\_Helicobacter\_pylori\_26695\_1\_Jun\_2018
- Bac\_Leptospira\_interrogans\_serovar\_Lai\_str56601\_1\_Jun\_2018
- Bac\_Mycobacterium\_tuberculosis\_H37Rv\_27\_May\_2017
- Bac\_Neisseria\_gonorrhoeae\_FA\_1090\_1\_Jun\_2018
- Bac\_Neisseria\_meningitidis\_MC58\_9\_Jun\_2017
- Bac\_Nostoc\_punctiforme\_PCC\_73102\_18\_Mar\_2017
- Bac\_Phycisphaerae\_bacterium\_L21\_RPulD3\_1\_Jun\_2018
- Bac\_Plesiocystis\_pacifica\_SIR1\_1\_Jun\_2018
- Bac\_Pseudomonas\_aeruginosa\_PAO1\_5\_Jun\_2017
- Bac\_Salmonella\_ent\_ser\_Typhi\_CT18\_22\_Nov\_2018
- Bac\_Staphylococcus\_aureus\_subsp\_aureus\_NCTC\_8325\_13\_Jun\_2017
- Bac\_Streptomyces\_scabiei\_87.22\_24\_Sep\_2017
- Bac\_Synechocystis\_sp\_PCC\_6803\_6\_Jun\_2017
- Bac\_Tenacibaculum\_dicentrarchi\_27\_Nov\_2017
- Bac\_Tenacibaculum\_maritimum\_NBRC\_15946\_27\_Nov\_2017
- Bac\_Thermus\_aquaticus\_Y51MC23\_24\_Sep\_2017
- Bac\_Thermus\_thermophilus\_HB8\_19\_Sep\_2017
- Bac\_Waddlia\_chondrophila\_WSU\_86\_1044\_1\_Jun\_2018
- Bac\_Yersinia\_pestis\_CO92\_10\_Apr\_2017
- Vir\_SARS-CoV-2\_31\_Mar\_2020
- No elements found. Consider changing the search query.
- List is empty.

ResubmitReset

MSA generation method

HHblits=>UniRef30

- HHblits=>UniRef30 (default)
- PSI-BLAST=>nr70
- No elements found. Consider changing the search query.
- List is empty.

Maximal no. of MSA generation steps

3

- 0
- 1
- 2
- 3 (default)
- 4
- 5
- 8
- No elements found. Consider changing the search query.
- List is empty.

E-value incl. threshold for MSA generation

1e-3

- 0.1
- 0.05
- 0.02
- 0.01
- 1e-3 (default)
- 1e-6
- 1e-8
- 1e-10
- 1e-15
- 1e-20
- 1e-30
- 1e-40
- 1e-50
- No elements found. Consider changing the search query.
- List is empty.

Min. seq. identity of MSA hits with query (%)

0

- 0 (default)
- 10
- 20
- 30
- 40
- 50
- 60
- 70
- 75
- 80
- 85
- 90
- 95
- 100
- No elements found. Consider changing the search query.
- List is empty.

Min. coverage of MSA hits (%)

20

- 10
- 20 (default)
- 30
- 40
- 50
- 60
- 70
- 80
- 90
- 100
- No elements found. Consider changing the search query.
- List is empty.

Secondary structure scoring

during\_alignment

- none
- after\_alignment
- during\_alignment (default)
- after\_alignment\_pred\_vs\_pred
- during\_alignment\_pred\_vs\_pred
- No elements found. Consider changing the search query.
- List is empty.

Alignment Mode:Realign with MAC

local:norealign

- local:norealign (default)
- local:realign
- global:realign
- No elements found. Consider changing the search query.
- List is empty.

MAC realignment threshold

0.3

- 0.0
- 0.01
- 0.1
- 0.2
- 0.3 (default)
- 0.4
- 0.5
- 0.6
- 0.7
- 0.8
- 0.9
- 0.95
- No elements found. Consider changing the search query.
- List is empty.

No. of target sequences (up to 10000)

250

- 250 (default)
- 500
- 1000
- 2000
- 3000
- 4000
- 5000
- 6000
- 7000
- 8000
- 9000
- 10000
- No elements found. Consider changing the search query.
- List is empty.

Min. probability in hit list (> 10%)

20

- 10
- 20 (default)
- 30
- 40
- 50
- 60
- 70
- 75
- 80
- 85
- 90
- 95
- 100
- No elements found. Consider changing the search query.
- List is empty.

ResubmitReset

VisHitsAln
Select AllForwardForward Query A3MDownload HHRColor SeqsWrap Seqs

Number of Hits: **31**

Detected sequence features:
**◾Transmembrane segment(s)**

#### Visualization

Resubmit Section

29

456

Prob=99.8% E=1.1E-17 PF09852.10 ; DUF2079 ; Predicted membrane protein (DUF2079)

#### Hitlist

Show102550100AllEntries

Search:

| Nr (Click to sort Ascending) | Hit (Click to sort Ascending) | Name (Click to sort Ascending) | Probability (Click to sort Ascending) | E-value (Click to sort Ascending) | SS (Click to sort Ascending) | Cols (Click to sort Ascending) | Target Length (Click to sort Ascending) |
| --- | --- | --- | --- | --- | --- | --- | --- |
| 1 | PF03901.18 | ; Glyco\_transf\_22 ; Alg9-like mannosyltransferase family | 99.89 | 2.1e-20 | 30.3 | 347 | 388 |
| 2 | PF02516.15 | ; STT3 ; Oligosaccharyl transferase STT3 subunit | 99.86 | 5.5e-19 | 30.7 | 370 | 458 |
| 3 | PF09852.10 | ; DUF2079 ; Predicted membrane protein (DUF2079) | 99.83 | 1.1e-17 | 32.4 | 342 | 519 |
| 4 | PF07220.12 | ; DUF1420 ; Protein of unknown function (DUF1420) | 99.77 | 5e-16 | 31.4 | 366 | 670 |
| 5 | PF10131.10 | ; PTPS\_related ; 6-pyruvoyl-tetrahydropterin synthase related domain; membrane protein | 99.73 | 1.4e-15 | 28.2 | 300 | 616 |
| 6 | PF02366.19 | ; PMT ; Dolichyl-phosphate-mannose-protein mannosyltransferase | 99.64 | 2.3e-14 | 20.8 | 216 | 247 |
| 7 | PF04188.14 | ; Mannosyl\_trans2 ; Mannosyltransferase (PIG-V) | 99.64 | 6e-14 | 25 | 326 | 432 |
| 8 | PF10034.10 | ; Dpy19 ; Q-cell neuroblast polarisation | 99.61 | 8.4e-13 | 31.2 | 362 | 651 |
| 9 | PF12250.9 | ; AftA\_N ; Arabinofuranosyltransferase N terminal | 99.56 | 3.6e-12 | 27.7 | 341 | 432 |
| 10 | PF11028.9 | ; DUF2723 ; Protein of unknown function (DUF2723) | 99.55 | 1.5e-13 | 16.6 | 155 | 188 |
| 11 | PF13231.7 | ; PMT\_2 ; Dolichyl-phosphate-mannose-protein mannosyltransferase | 99.54 | 3.7e-13 | 17.2 | 154 | 159 |
| 12 | PF09913.10 | ; DUF2142 ; Predicted membrane protein (DUF2142) | 99.53 | 2.1e-12 | 24.1 | 326 | 405 |
| 13 | PF04602.13 | ; Arabinose\_trans ; Mycobacterial cell wall arabinan synthesis protein | 99.47 | 6.3e-11 | 27.9 | 352 | 471 |
| 14 | PF06728.14 | ; PIG-U ; GPI transamidase subunit PIG-U | 99.44 | 9.8e-11 | 27.7 | 306 | 363 |
| 15 | PF15971.6 | ; Mannosyl\_trans4 ; DolP-mannose mannosyltransferase | 99.31 | 9.4e-11 | 16.5 | 156 | 163 |
| 16 | PF09586.11 | ; YfhO ; Bacterial membrane protein YfhO | 99.14 | 3.9e-8 | 29.7 | 363 | 832 |
| 17 | PF04922.13 | ; DIE2\_ALG10 ; DIE2/ALG10 family | 99.13 | 6.2e-9 | 20.5 | 209 | 434 |
| 18 | PF03155.16 | ; Alg6\_Alg8 ; ALG6, ALG8 glycosyltransferase family | 99.06 | 3e-8 | 22.8 | 327 | 470 |
| 19 | PF14264.7 | ; Glucos\_trans\_II ; Glucosyl transferase GtrII | 98.99 | 6.4e-7 | 27.1 | 302 | 312 |
| 20 | PF05208.14 | ; ALG3 ; ALG3 protein | 98.96 | 6.2e-8 | 19.7 | 203 | 356 |
| 21 | PF09594.11 | ; GT87 ; Glycosyltransferase family 87 | 98.78 | 3.5e-7 | 17.6 | 240 | 251 |
| 22 | PF05007.14 | ; Mannosyl\_trans ; Mannosyltransferase (PIG-M) | 96.49 | 0.17 | 17.6 | 190 | 269 |
| 23 | PF16192.6 | ; PMT\_4TMC ; C-terminal four TMM region of protein-O-mannosyltransferase | 91.61 | 2.3 | 12.2 | 108 | 198 |
| 24 | PF14897.7 | ; EpsG ; EpsG family | 89.45 | 5.2 | 28.9 | 298 | 319 |
| 25 | PF09971.10 | ; DUF2206 ; Predicted membrane protein (DUF2206) | 83.62 | 13 | 17.4 | 213 | 390 |

Displaying 1 to 25 of 31 hits

- «
- ‹
- 1
- 2
- ›
- »

#### Alignments

|  |  |  |  |
| --- | --- | --- | --- |
|  | | | |
|  | Template alignmentCDD | | |
| 1. | PF03901.18 ; Glyco\_transf\_22 ; Alg9-like mannosyltransferase family | | |
|  | Probability: 99.89%, E-value: 2.1e-20, Score: 164.96, Aligned cols: 347, Identities: 11%, Similarity: -0.016, | | |
|  |
|  | Q ss\_pred |  | HHHHHHHHHHHHHhhhcCCCccccch-HHHHhCCCCCCCCCcccccccccccccCCCCCCCcccccchHHHHHHHHHHHh |
|  | Q XP\_016875493.1 | 29 | AAALLAGASCLCYGRSLQGEFVHDDV-WAIVNNPDVRPGAPLRWGIFTNDFWGKGMAENTSHKSYRPLCVLTFKLNIFLT   107 (456) |
|  | Q Consensus | 29 | ~~~~l~~~~~~~~~~~~~~~~~~De~-~~~~~~~~~~~~~~~~~~~~~~~~~~~~~~~~~~~~~~~Pl~~~~~~~~~~l~   107 (456) |
|  |  |  | .++++.++............+..||. .+...+++..+++.........+. ....+||++..+......++ |
|  | T Consensus | 1 | lil~~~~~l~l~~~~~~~~~~~~De~~~~~~~a~~~~~~~~~~~~~~~~~~---------~~~~~p~~~~~~~~~~~~~~   71 (388) |
|  | T PF03901.18 | 1 | YLLLFTIALRILNCFLVQTSFVPDEYWQSLEVSHHMVFNYGYLTWEWTERL---------RSYTYPLIFASIYKILHLLG   71 (388) |
|  | T ss\_pred |  | CHHHHHHHHHHHHHHhhhcCCCchHHHHHHHHHhhhccccCcCCCcccccc---------cCCHHHHHHHHHHHHHHHcC |
|  |
|  |
|  | Q ss\_pred |  | CCCc----hHHHHHHHHHHHHHHHHHHHHHHHHhccchHHHHHHHHHHHHccccHHHHHHHHhHHHHHHHHHHHHHHHHH |
|  | Q XP\_016875493.1 | 108 | GMNP----FYFHAVNIILHCLVTLVLMYTCDKTVFKNRGLAFVTALLFAVHPIHTEAVAGIVGRADVLACLLFLLAFLSY   183 (456) |
|  | Q Consensus | 108 | g~~~----~~~rl~~~l~~~~~~~~~~~l~~~~~~~~~~~a~~aa~l~a~~p~~~~~~~~~~~~~~~~~~~~~~l~~~~~   183 (456) |
|  |  |  | |.++ ...|+.+.+++++++.++|.++||.. +++.+++++++++++|....+ ....++|.+..++.+++++++ |
|  | T Consensus | 72 | ~~~~~~~~~~~r~~~~l~~~~~~~~~y~l~~~~~--~~~~a~~a~~l~~~~p~~~~~--~~~~~~~~~~~~~~~~~~~~~   147 (388) |
|  | T PF03901.18 | 72 | KDSVQLLIWIPRLAQALLSAVADVRLYSLMKQLE--NQEVARWVFFCQLCSWFTWYC--CTRTLTNTMETVLTIIALFYY   147 (388) |
|  | T ss\_pred |  | CCCHHHHHHHHHHHHHHHHHHHHHHHHHHHHHHc--ChhHHHHHHHHHHHhHHHHHH--HhhhchHHHHHHHHHHHHHHH |
|  |
|  |
|  | Q ss\_pred |  | HHHHhcCCCCCCCCcchhHHHHHHHHHHHHHHHHHHHHHHHHHHHHHHHHHHhcccccchhHhhccCcchHHHhhHHHHH |
|  | Q XP\_016875493.1 | 184 | NRSLDQGCVGGSFPSTVSPFFLLLSLFLGTCAMLVKETGITVFGVCLVYDLFSLSNKQDKSYLRASSNRNFLLTMRPFLK   263 (456) |
|  | Q Consensus | 184 | ~~~~~~~~~~~~~~~~~~~~~~~~~~~~~~la~~~k~~~~~~~~~~~~~~~~~~~~~~~~~~~~~~~~~~~~~~~~~~~~   263 (456) |
|  |  |  | .+..+++ +.+.. ++++.+++.++|+.+..+.++..++.+...+++ +++... . |
|  | T Consensus | 148 | ~~~~~~~----------~~~~~--~~~~~~l~~~~k~~~~~~~~~~~~~~~~~~~~~-~~~~~~---------------~   199 (388) |
|  | T PF03901.18 | 148 | PLEGSKS----------MNSVK--YSSLVALAFIIRPTAVILWTPLLFRHFCQEPRK-LDLILH---------------H   199 (388) |
|  | T ss\_pred |  | HHHhCCC----------CcHHH--HHHHHHHHHHhcchHHHHHHHHHHHHHHcChhc-HHHHHH---------------H |
|  |
|  |
|  | Q ss\_pred |  | HHHHHHHHHHHHHHHHHHHhcCCCCccccCCCCcccchhHHHHHHHHHHHHHHHHHHHHHhHhhccccccCCCccccccc |
|  | Q XP\_016875493.1 | 264 | RAILVLSYVLVILYFRLWIMGGSMPLFSEQDNPASFSPYILTRFLTYSYLLAFNVWLLLAPVTLCYDWQVGSIPLVETIW   343 (456) |
|  | Q Consensus | 264 | ~~~~~~~~~~~~~~~~~~~~~~~~~~~~~~~~~~~~~~~~~~~~~~~~~~~~~~~~~~~~~~~~~~~~~~~~~~~~~~~~   343 (456) |
|  |  |  | .....++..+..........++.................................... |
|  | T Consensus | 200 | ~~~~~~~~~~~~~~~~~~~~~~~~~~~~~~~~~~~~~~~~~~~~~~~~~~~~~~~~~~----------------------   257 (388) |
|  | T PF03901.18 | 200 | FLPVGFVTLSLSLMIDRIFFGQWTLVQFNFLKFNVLQNWGTFYGSHPWHWYFSQGFPV----------------------   257 (388) |
|  | T ss\_pred |  | HHHHHHHHHHHHHHHHHHHhcchhhhhhhhhhhhcccccccccccCchHHHHHhhhhh---------------------- |
|  |
|  |
|  | Q ss\_pred |  | cHHHHHHHHHHHHHHHHHHHHHHHHhccccchHHHHHHHHHHHHHhHhccccCCCccccccccchHHHHHHHHHHHHHHH |
|  | Q XP\_016875493.1 | 344 | DMRNLATIFLAVVMALLSLHCLAAFKRLEHKEVLVGLLFLVFPFIPASNLFFRVGFVVAERVLYMPSMGYCILFVHGLSK   423 (456) |
|  | Q Consensus | 344 | ~~~~~~~~~~~~~~~~~~~~~~~~~~~~~~~~~~~~~~~~~~~~~~~~~~~~~~~~~~~~Ry~~~~~~~~~ll~~~~~~~   423 (456) |
|  |  |  | ................+++++ ....+.+............. +||..+..|+++++++.++.+ |
|  | T Consensus | 258 | ---------~~~~~~~~~~~~~~~~~~~~~----~~~~~~~~~~~~~~~~~~~~-----~ry~~~~~p~~~l~~~~~~~~   319 (388) |
|  | T PF03901.18 | 258 | ---------ILGTHLPFFIHGCYLAPKRYR----ILLVTVLWTLLVYSMLSHKE-----FRFIYPVLPFCMVFCGYSLTH   319 (388) |
|  | T ss\_pred |  | ---------hHHhHHHHHHHHHHHchhhhH----HHHHHHHHHHHHHHhhcCCC-----cchHhcHHHHHHHHHHHHHHh |
|  |
|  |
|  | Q ss\_pred |  | HHHHhchhhHHHHHHHHHHHHHHHHHHHHHhcC |
|  | Q XP\_016875493.1 | 424 | LCTWLNRCGATTLIVSTVLLLLLFSWKTVKQNE   456 (456) |
|  | Q Consensus | 424 | ~~~~~~~~~~~~~~~~~~~~~~~~~~~~~~~~~   456 (456) |
|  |  |  | ..++.+++........++..............+ |
|  | T Consensus | 320 | ~~~~~~~~~~~~~~~~~~~~~~~~~~~~~~~~~   352 (388) |
|  | T PF03901.18 | 320 | LKTWKKPALSFLFLSNLFLALYTGLVHQRGTLD   352 (388) |
|  | T ss\_pred |  | hhcccchHHHHHHHHHHHHHHHHHHHhhccchH |
|  |
| --- | | | |
|  | Template alignmentCDD | | |
| 2. | PF02516.15 ; STT3 ; Oligosaccharyl transferase STT3 subunit | | |
|  | Probability: 99.86%, E-value: 5.5e-19, Score: 159.45, Aligned cols: 370, Identities: 12%, Similarity: 0.015, | | |
|  |
|  | Q ss\_pred |  | HHHHHHHHHHHHHHHH-----hhhcCCCccccchHHHHhCCCCCCCCCcccccccccccccCCCCCCC-cccccchHHHH |
|  | Q XP\_016875493.1 | 26 | PAGAAALLAGASCLCY-----GRSLQGEFVHDDVWAIVNNPDVRPGAPLRWGIFTNDFWGKGMAENTS-HKSYRPLCVLT   99 (456) |
|  | Q Consensus | 26 | ~~~~~~~l~~~~~~~~-----~~~~~~~~~~De~~~~~~~~~~~~~~~~~~~~~~~~~~~~~~~~~~~-~~~~~Pl~~~~   99 (456) |
|  |  |  | ....+++++++....+ ..........||..|...++++.+++....+.+....+.+....+.. ...+||+++++ |
|  | T Consensus | 3 | ~~~~l~~i~~~~~~~r~~~~~~~~~~~~~~~D~~~~~~~a~~~~~~~~~~~~~~~~~~~~~~~~~~~~~~~~~~p~~~~l   82 (458) |
|  | T PF02516.15 | 3 | SRIETAELKGMNTADRAYFTDENGLPYMYEPDSYYNYRLTANILDHGHPGDKIINGTPWDLHSNYPPGNRVNYPPLILWI   82 (458) |
|  | T ss\_pred |  | hhHhHHHHHhhhhhhHHhCCCCCCCCcccCCCcHHHHHHHHHHHHhCCCCCcccCCCCCCchhcCCCCCCCCCCchHHHH |
|  |
|  |
|  | Q ss\_pred |  | HHHHHHHhCCCc-----hHHHHHHHHHHHHHHHHHHHHHHHHhccchHHHHHHHHHHHHccccHHHHHHHHhH--HHHHH |
|  | Q XP\_016875493.1 | 100 | FKLNIFLTGMNP-----FYFHAVNIILHCLVTLVLMYTCDKTVFKNRGLAFVTALLFAVHPIHTEAVAGIVGR--ADVLA   172 (456) |
|  | Q Consensus | 100 | ~~~~~~l~g~~~-----~~~rl~~~l~~~~~~~~~~~l~~~~~~~~~~~a~~aa~l~a~~p~~~~~~~~~~~~--~~~~~   172 (456) |
|  |  |  | .+....++|... ...|+++.++++++++.+|.++||.. +++.|++++++++++|.+... ...++ +|.+. |
|  | T Consensus | 83 | ~~~~~~~~~~~~~~~~~~~~~~~~~~~~~l~~~~~y~l~~~~~--~~~~a~~a~~l~~~~p~~~~~--~~~~~~~~~~~~   158 (458) |
|  | T PF02516.15 | 83 | SLLFHNFINLFIPFSLIETCFWLPAIIGPLAGIVMFFMVRRYA--GDLPGLLSGVLLVLAPVYFSR--TVPGFFDTDMFN   158 (458) |
|  | T ss\_pred |  | HHHHHHHHHhhCCCcHHHHHhHHHHHHHHHHHHHHHHHHHHHc--CcHHHHHHHHHHHHcHHHHHh--hCCCCCCchHHH |
|  |
|  |
|  | Q ss\_pred |  | HHHHHHHHHHHHHHHhcCCCCCCCCcchh--HHHHHHHHHHHHHHHHHHHHHHHHHHHHHHHHHHhcccccchhHhhccC |
|  | Q XP\_016875493.1 | 173 | CLLFLLAFLSYNRSLDQGCVGGSFPSTVS--PFFLLLSLFLGTCAMLVKETGITVFGVCLVYDLFSLSNKQDKSYLRASS   250 (456) |
|  | Q Consensus | 173 | ~~~~~l~~~~~~~~~~~~~~~~~~~~~~~--~~~~~~~~~~~~la~~~k~~~~~~~~~~~~~~~~~~~~~~~~~~~~~~~   250 (456) |
|  |  |  | .++.+++++++.+..+++ + +++.++++++.+++.++|+. ....+...........++++++..+ |
|  | T Consensus | 159 | ~~~~~l~~~~~~~~~~~~----------~~~~~~~~l~g~~~~l~~~~~~~-~~~~~~~~~~~~~~~~~~~~~~~~~---   224 (458) |
|  | T PF02516.15 | 159 | IIFPLLVIFFLLKATETK----------NNYMFPLLLSSFSLALLSLSWNG-WAYIFYIIIISSILYMTLCKLKGKA---   224 (458) |
|  | T ss\_pred |  | HHHHHHHHHHHHHHhccC----------CCchHHHHHHHHHHHHHHhHhhH-HHHHHHHHHHHHHHHHHHhhcccch--- |
|  |
|  |
|  | Q ss\_pred |  | cchHHHhhHHHHHHHHHHHHHHHHHHHHHHHHhcC-------------CCCccccCCCCcccchhHHHHHHHHHHHHHHH |
|  | Q XP\_016875493.1 | 251 | NRNFLLTMRPFLKRAILVLSYVLVILYFRLWIMGG-------------SMPLFSEQDNPASFSPYILTRFLTYSYLLAFN   317 (456) |
|  | Q Consensus | 251 | ~~~~~~~~~~~~~~~~~~~~~~~~~~~~~~~~~~~-------------~~~~~~~~~~~~~~~~~~~~~~~~~~~~~~~~   317 (456) |
|  |  |  | .................+..... ................................ |
|  | T Consensus | 225 | ------------~~~~~~~~~~~~~~~~~~~~~~~~~~~~~~~~~~~~~~~~~~~~~~~~~~~~~~~~~~~~~~~~~~~~   292 (458) |
|  | T PF02516.15 | 225 | ------------VMGFSRKIAVFVIISLLIIGLAGRLGYALIFPTFFKFTFKSLSAGGWPGIFESISELSAPTFDEFLSL   292 (458) |
|  | T ss\_pred |  | ------------hhHHHHHHHHHHHHHHHHhhhcccccHHHHHHHHHHHhcccccCCCCCcchHHHHHHhcccHHHHhcC |
|  |
|  |
|  | Q ss\_pred |  | HHHHHHhHhhccccccCCCccccccccHHHHHHHHHHHHHHHHHHHHHHHHhc-cccchHHHHHHHHHHHHHhHhccccC |
|  | Q XP\_016875493.1 | 318 | VWLLLAPVTLCYDWQVGSIPLVETIWDMRNLATIFLAVVMALLSLHCLAAFKR-LEHKEVLVGLLFLVFPFIPASNLFFR   396 (456) |
|  | Q Consensus | 318 | ~~~~~~~~~~~~~~~~~~~~~~~~~~~~~~~~~~~~~~~~~~~~~~~~~~~~~-~~~~~~~~~~~~~~~~~~~~~~~~~~   396 (456) |
|  |  |  | ...... .......+..........+++ ++++.......+.............. |
|  | T Consensus | 293 | ~~~~~~--------------------------~~~~~~~~~~~~~~~~~~~~~~~~~~~~~~~~~~~~~~~~~~~~~~~~   346 (458) |
|  | T PF02516.15 | 293 | PGPVNM--------------------------GIGLFGFVIIGSIMLRDEIKRVHLPDFSWYPFILIGIWLIIGLAAYSL   346 (458) |
|  | T ss\_pred |  | chHHHH--------------------------HHHHHHHHHHHHHHHHHHHHhccCCCCccHHHHHHHHHHHHHHHHHHH |
|  |
|  |
|  | Q ss\_pred |  | CCccccccccchHHHHHHHHHHHHHHHHHHHhchhh-----HHHHHHHHHHHHHHHHHHHHHhcC |
|  | Q XP\_016875493.1 | 397 | VGFVVAERVLYMPSMGYCILFVHGLSKLCTWLNRCG-----ATTLIVSTVLLLLLFSWKTVKQNE   456 (456) |
|  | Q Consensus | 397 | ~~~~~~~Ry~~~~~~~~~ll~~~~~~~~~~~~~~~~-----~~~~~~~~~~~~~~~~~~~~~~~~   456 (456) |
|  |  |  | . .||..+..|+++++++.++.+..++.+++. .......+++++............ |
|  | T Consensus | 347 | ~-----~Ry~~~~~p~~~i~~~~~~~~~~~~~~~~~~~~~~~~~~~~~~~~~~~~~~~~~~~~~~   406 (458) |
|  | T PF02516.15 | 347 | S-----TRFALLVIPPLIIFLGLLMGVMASYLKGSPSMRLRRSGNVFILSLVVMLSTISFIQAYE   406 (458) |
|  | T ss\_pred |  | H-----HhHHHhhHHHHHHHHHHHHHHHHHHhcCCccchHHHHHHHHHHHHHHHHHHHHHHHHhh |
|  |
| --- | | | |
|  | Template alignmentCDD | | |
| 3. | PF09852.10 ; DUF2079 ; Predicted membrane protein (DUF2079) | | |
|  | Probability: 99.83%, E-value: 1.1e-17, Score: 153.22, Aligned cols: 342, Identities: 12%, Similarity: -0.012, | | |
|  |
|  | Q ss\_pred |  | CCccccchHHHHhCCCCCCCCCcccccccccccccCCCCCCCcccccchHHHHHHHHHHHhCCCchHHHHHHHHHHHHHH |
|  | Q XP\_016875493.1 | 47 | GEFVHDDVWAIVNNPDVRPGAPLRWGIFTNDFWGKGMAENTSHKSYRPLCVLTFKLNIFLTGMNPFYFHAVNIILHCLVT   126 (456) |
|  | Q Consensus | 47 | ~~~~~De~~~~~~~~~~~~~~~~~~~~~~~~~~~~~~~~~~~~~~~~Pl~~~~~~~~~~l~g~~~~~~rl~~~l~~~~~~   126 (456) |
|  |  |  | ....+||..|...++++.+++....+..+.. ....|||++.++.++.+.++| ++...|+++++++++++ |
|  | T Consensus | 6 | ~~~~~De~~~~~~a~~~~~g~~~~~~~~~~~----------~~~~~~pl~~~l~a~~~~l~g-~~~~~rl~~~l~~~~~~   74 (519) |
|  | T PF09852.10 | 6 | NATAYDLGIYVSILENTMHGHVMYANPLLIN----------SFSEHFSPFLFVIYPIYWFFP-YVKTLLIMQSVMISFSG   74 (519) |
|  | T ss\_pred |  | CccccHHHHHHHHHHHHHcCCccccCccccc----------cccccchhHHHHHHHHHHHcC-ChHHHHHHHHHHHHHHH |
|  |
|  |
|  | Q ss\_pred |  | HHHHHHHHHHhccc--------hHHHHHHHHHHHHccccHHHHHH-HHhHHHHHHHHHHHHHHHHHHHHHhcCCCCCCCC |
|  | Q XP\_016875493.1 | 127 | LVLMYTCDKTVFKN--------RGLAFVTALLFAVHPIHTEAVAG-IVGRADVLACLLFLLAFLSYNRSLDQGCVGGSFP   197 (456) |
|  | Q Consensus | 127 | ~~~~~l~~~~~~~~--------~~~a~~aa~l~a~~p~~~~~~~~-~~~~~~~~~~~~~~l~~~~~~~~~~~~~~~~~~~   197 (456) |
|  |  |  | +++|.++|+.+.++ ++.|++++++++++|..... . ...+.|.+..++++++++.+.+. ++ |
|  | T Consensus | 75 | ~~~y~l~r~~~~~~~~~~~~~~~~~al~a~ll~~~~p~~~~~--~~~~~~~~~~~~~~~~~~~~~~~~~--~~-------   143 (519) |
|  | T PF09852.10 | 75 | LVIYLLAREIFFINNFKKDILLEMLALFISTSYILSPYIESP--LSFDFHLMPFLILFVPLSFYFFMKK--YK-------   143 (519) |
|  | T ss\_pred |  | HHHHHHHHHHhccCcccchhHHHHHHHHHHHHHHHcHHhhhh--hhcCCchHHHHHHHHHHHHHHHHhh--cc------- |
|  |
|  |
|  | Q ss\_pred |  | cchhHHHHHHHHHHHHHHHHHHHHHHHHHHHHHHHHHHh-----------------c----------ccccchhHhhccC |
|  | Q XP\_016875493.1 | 198 | STVSPFFLLLSLFLGTCAMLVKETGITVFGVCLVYDLFS-----------------L----------SNKQDKSYLRASS   250 (456) |
|  | Q Consensus | 198 | ~~~~~~~~~~~~~~~~la~~~k~~~~~~~~~~~~~~~~~-----------------~----------~~~~~~~~~~~~~   250 (456) |
|  |  |  | ..++++++++++++|+.++++.+.+.++.+.. . ++++.+..++ |
|  | T Consensus | 144 | -------~~~~gl~~~la~l~k~~~~~~~~~~~l~~~~~~~~~~~~~~~~~~~~~~~~~~~~~~~~~~~~~~~~~~~---   213 (519) |
|  | T PF09852.10 | 144 | -------ILNLIVLILIISLHSLFVIMVFFIISYQFIFRIRNEGNLNCHKIIRTIANINISDNLKKTPKSKYVLQKI---   213 (519) |
|  | T ss\_pred |  | -------HHHHHHHHHHHHHhcchHHHHHHHHHHHHHHHHhccCCCcchhhhHHhhhcccccccccCCcchhhhhhh--- |
|  |
|  |
|  | Q ss\_pred |  | cchHHHhhHHHHHHHHHHHHHHHHHHHHHHHHhcCCCCccccCCCCcccchhHHHHHHHHHHHHHHHHHHHHHhHhhccc |
|  | Q XP\_016875493.1 | 251 | NRNFLLTMRPFLKRAILVLSYVLVILYFRLWIMGGSMPLFSEQDNPASFSPYILTRFLTYSYLLAFNVWLLLAPVTLCYD   330 (456) |
|  | Q Consensus | 251 | ~~~~~~~~~~~~~~~~~~~~~~~~~~~~~~~~~~~~~~~~~~~~~~~~~~~~~~~~~~~~~~~~~~~~~~~~~~~~~~~~   330 (456) |
|  |  |  | .....................................................+....................... |
|  | T Consensus | 214 | -~~~~~~~~~~~~~~~~~~~~~~~~~~~~~~~~~~~~~~~~~~~~~~~~~~~~~~~~~~~~~~~~~~~~~~~~~~~~~--   290 (519) |
|  | T PF09852.10 | 214 | -VRSKTLIKIIITLILLVGYLYFASLMKTFIASGAVALSPPSTMSTGSVSSSLAGLFTDLFTRPMLIESAFLINFPDK--   290 (519) |
|  | T ss\_pred |  | -hcchHHHHHHHHHHHHHHHHHHHHHHHHHHHccccccCCCcccCCCCcCCcHHHHHHHHhhCchHHHHHHhccchHH-- |
|  |
|  |
|  | Q ss\_pred |  | cccCCCccccccccHHHHHHHHHHHHHHHHHHHHHHHHhccccchHHHHHHHHHHHHHhHhccccCCCccccccccchHH |
|  | Q XP\_016875493.1 | 331 | WQVGSIPLVETIWDMRNLATIFLAVVMALLSLHCLAAFKRLEHKEVLVGLLFLVFPFIPASNLFFRVGFVVAERVLYMPS   410 (456) |
|  | Q Consensus | 331 | ~~~~~~~~~~~~~~~~~~~~~~~~~~~~~~~~~~~~~~~~~~~~~~~~~~~~~~~~~~~~~~~~~~~~~~~~~Ry~~~~~   410 (456) |
|  |  |  | ....+...........+ ....++..+...................||.++.+ |
|  | T Consensus | 291 | ----------------------~~~~~~~~~~~~~~~~~------~~~~~~~~~~~~~~~~~~~~~~~~~~~~ry~~~~~   342 (519) |
|  | T PF09852.10 | 291 | ----------------------IIFAFYAFANTGFLVFL------DPLSLLMDIPYFLYAYLSSYGPYYSLGYQYSTMII   342 (519) |
|  | T ss\_pred |  | ----------------------HHHHHHHHHHHhHHHhc------CHHHHHhHHHHHHHHHhccCccccchhhHHHHhHH |
|  |
|  |
|  | Q ss\_pred |  | HHHHHHHHHHHHHHH-------HHhchhhHHHHHHHHHHHHHHHHHHH |
|  | Q XP\_016875493.1 | 411 | MGYCILFVHGLSKLC-------TWLNRCGATTLIVSTVLLLLLFSWKT   451 (456) |
|  | Q Consensus | 411 | ~~~~ll~~~~~~~~~-------~~~~~~~~~~~~~~~~~~~~~~~~~~   451 (456) |
|  |  |  | |+++++++.++.++. +...++..+.....++++.++..... |
|  | T Consensus | 343 | p~l~i~~~~~~~~~~~~~~~~~~~~~~~~~~~~~~~~~~~~~~~~~~~   390 (519) |
|  | T PF09852.10 | 343 | PFIFIGALFGIRKIVQSARATDSDDVRRTIKKILVGVISIVIVSSLFE   390 (519) |
|  | T ss\_pred |  | HHHHHHHHHHHHHHHHHhccCCCcchhHhHHHHHHHHHHHHHHHHHHh |
|  |
| --- | | | |
|  | Template alignmentCDD | | |
| 4. | PF07220.12 ; DUF1420 ; Protein of unknown function (DUF1420) | | |
|  | Probability: 99.77%, E-value: 5e-16, Score: 146.69, Aligned cols: 366, Identities: 10%, Similarity: -0.067, | | |
|  |
|  | Q ss\_pred |  | CCcchhhhhhHHHHHHHHHHHHHHHHHHhhhcCCCccccc-hHHHHhCCCCCCCCCcccccccccccccCCCCCCCcccc |
|  | Q XP\_016875493.1 | 14 | RTPSRRRGCGLAPAGAAALLAGASCLCYGRSLQGEFVHDD-VWAIVNNPDVRPGAPLRWGIFTNDFWGKGMAENTSHKSY   92 (456) |
|  | Q Consensus | 14 | ~~~~~~~~~~~~~~~~~~~l~~~~~~~~~~~~~~~~~~De-~~~~~~~~~~~~~~~~~~~~~~~~~~~~~~~~~~~~~~~   92 (456) |
|  |  |  | ....+.............+++++...........+..+|| .+|...++++.+++........ .+..+ |
|  | T Consensus | 144 | ~~~~~~~~~~~~~~~l~~~i~~~~~~~~~~~~~p~~~~D~~~yhl~~a~~~~~~g~~~~~~~~------------~~~~~   211 (670) |
|  | T PF07220.12 | 144 | GFIKNKFNLINKNDVLNVFIILLMIGYGFLALCPITNADSLDYHIGVAIEILNQGKMPVFSGW------------FHGRL   211 (670) |
|  | T ss\_pred |  | hhHHhhcccCChhHHHHHHHHHHHHHHHHHHcCCCCCCHHHHHHHHHHHHHHHcCCCCCCCCc------------hhhcC |
|  |
|  |
|  | Q ss\_pred |  | cchHHHHHHHHHHHhCCCchHHHHHHHHHHHHHHHHHHHHHHHHhccchHHHHHHHHHHHHccccHHHHHHHH---hHHH |
|  | Q XP\_016875493.1 | 93 | RPLCVLTFKLNIFLTGMNPFYFHAVNIILHCLVTLVLMYTCDKTVFKNRGLAFVTALLFAVHPIHTEAVAGIV---GRAD   169 (456) |
|  | Q Consensus | 93 | ~Pl~~~~~~~~~~l~g~~~~~~rl~~~l~~~~~~~~~~~l~~~~~~~~~~~a~~aa~l~a~~p~~~~~~~~~~---~~~~   169 (456) |
|  |  |  | ||+...+......+.+.........+.+++.+++.++|.++||...+....+.+++++++++|.+... +.. ...| |
|  | T Consensus | 212 | P~~~~~l~~~~~~l~~~~~~~~~~~~~l~~~l~~~~~y~l~r~~~~~~~~~a~~aall~~~~p~~~~~--s~~~~~~~~d   289 (670) |
|  | T PF07220.12 | 212 | AGSGEVLNALGLAIGAEQFGSLLQFCGLLSIYGILSFYSFAEKFSESDGVWRKIIIIAFLSSPVLVFL--VSSPKPQLLQ   289 (670) |
|  | T ss\_pred |  | CchHHHHHHHHHHHcchhHHHHHHHHHHHHHHHHHHHHHHHHHhcCCCCHHHHHHHHHHHhchHHHHH--hcCCchHHHH |
|  |
|  |
|  | Q ss\_pred |  | HHHHHHHHHHHHHHHHHHhcCCCCCCCCcchhHHHHHHHHHH--HHHHHHHHHHHHHHHHHHHHHHHHhcccccchhHhh |
|  | Q XP\_016875493.1 | 170 | VLACLLFLLAFLSYNRSLDQGCVGGSFPSTVSPFFLLLSLFL--GTCAMLVKETGITVFGVCLVYDLFSLSNKQDKSYLR   247 (456) |
|  | Q Consensus | 170 | ~~~~~~~~l~~~~~~~~~~~~~~~~~~~~~~~~~~~~~~~~~--~~la~~~k~~~~~~~~~~~~~~~~~~~~~~~~~~~~   247 (456) |
|  |  |  | ...+++.+++++.+.+..+++ +.++..+++++ +|+|+.+|++++.+.++..+..+...++++... |
|  | T Consensus | 290 | ~~~~~~~~~~l~~~~~~~~~~----------~~~~~~l~gl~~~~gla~~~K~~~~~~~~~~~~~~l~~~~~~~~~~---   356 (670) |
|  | T PF07220.12 | 290 | IGMTSFAITLLLEIFSKIKTD----------KNKLFAFSLICILIMSATQAKFSFFLSAFLIGLFSIFSLGSIRLFF---   356 (670) |
|  | T ss\_pred |  | HHHHHHHHHHHHHHHHhhccC----------CchHHHHHHHHHHHHHHHhhHHHHHHHHHHHHHHHHHHhcccchhH--- |
|  |
|  |
|  | Q ss\_pred |  | ccCcchHHHhhHHHHH---HHHHHHHHHHHHHHHHHHHhcCCCCccccCCCCcccchhHHHHHHHHHHHHHHHHHHHHHh |
|  | Q XP\_016875493.1 | 248 | ASSNRNFLLTMRPFLK---RAILVLSYVLVILYFRLWIMGGSMPLFSEQDNPASFSPYILTRFLTYSYLLAFNVWLLLAP   324 (456) |
|  | Q Consensus | 248 | ~~~~~~~~~~~~~~~~---~~~~~~~~~~~~~~~~~~~~~~~~~~~~~~~~~~~~~~~~~~~~~~~~~~~~~~~~~~~~~   324 (456) |
|  |  |  | +.... ..+..++....+...+....+....................+..................+ |
|  | T Consensus | 357 | -----------~~~~~~~~~~~~~~~~~~~w~~~~~~~~g~~~~~~~~~~~~~~~~~~~~~~~~~~~~~~~~~~~~~~~~   425 (670) |
|  | T PF07220.12 | 357 | -----------YGLLISLFFFVLINFPAIFWKIKNYNSTFIDVLIHPLPGNTFPGVNEFEVSLRNYQDSALIFPLSLIFP   425 (670) |
|  | T ss\_pred |  | -----------HHHHHHHHHHHHHHHHHHHHHHHHHHccCCcccccCCCCCCCCCccchHHHHHhcccccccCcHHhhcc |
|  |
|  |
|  | Q ss\_pred |  | HhhccccccCCCccccccccHHHHHHHHHHHHHHHHHHHHHHHHhccccchHHHHHHHHHHHHHhHhccccCCCcccccc |
|  | Q XP\_016875493.1 | 325 | VTLCYDWQVGSIPLVETIWDMRNLATIFLAVVMALLSLHCLAAFKRLEHKEVLVGLLFLVFPFIPASNLFFRVGFVVAER   404 (456) |
|  | Q Consensus | 325 | ~~~~~~~~~~~~~~~~~~~~~~~~~~~~~~~~~~~~~~~~~~~~~~~~~~~~~~~~~~~~~~~~~~~~~~~~~~~~~~~R   404 (456) |
|  |  |  | .... ................++++++.......+............... .| |
|  | T Consensus | 426 | ~~~~------------------------~~~~~~~~~~~~~~~~~~~~~~~~~~~~~~~~~~~~~~~~~~~~~-----~R   476 (670) |
|  | T PF07220.12 | 426 | NQFG------------------------VITTVIGLGLFLIIFVKPIVTQKAFLLSVMIILFVILGSLMGQKA-----SR   476 (670) |
|  | T ss\_pred |  | ccch------------------------hhHhHHHHHHHHHHHhcchhchHHHHHHHHHHHHHHHHHHhccch-----HH |
|  |
|  |
|  | Q ss\_pred |  | ccchHHHHHHHHHHHHHHHHHHHhchhhHHHHHHHHHHHHHHH |
|  | Q XP\_016875493.1 | 405 | VLYMPSMGYCILFVHGLSKLCTWLNRCGATTLIVSTVLLLLLF   447 (456) |
|  | Q Consensus | 405 | y~~~~~~~~~ll~~~~~~~~~~~~~~~~~~~~~~~~~~~~~~~   447 (456) |
|  |  |  | |.+|.+|++++ ++.+.....+..++..+.....++++..+.. |
|  | T Consensus | 477 | y~lp~~p~l~l-~~~~~~~~~~~~~~~~~~~~~~~~~~~~~~~   518 (670) |
|  | T PF07220.12 | 477 | FFLEPFVWMLI-SLIGLNSFGKWNIRFVKEAVSTGILLQACAT   518 (670) |
|  | T ss\_pred |  | hhHHHHHHHHH-HHHHHHhhccccHHHHHHHHHHHHHHHHHHH |
|  |
| --- | | | |
|  | Template alignmentCDD | | |
| 5. | PF10131.10 ; PTPS\_related ; 6-pyruvoyl-tetrahydropterin synthase related domain; membrane protein | | |
|  | Probability: 99.73%, E-value: 1.4e-15, Score: 142.47, Aligned cols: 300, Identities: 11%, Similarity: 0.022, | | |
|  |
|  | Q ss\_pred |  | cccccchHHHHHHHHHHHh-CCCchHHHHHHHHHHHHHHHHHHHHHHHHhccchHHHHHHHHHHHHccccHHHHHHHHhH |
|  | Q XP\_016875493.1 | 89 | HKSYRPLCVLTFKLNIFLT-GMNPFYFHAVNIILHCLVTLVLMYTCDKTVFKNRGLAFVTALLFAVHPIHTEAVAGIVGR   167 (456) |
|  | Q Consensus | 89 | ~~~~~Pl~~~~~~~~~~l~-g~~~~~~rl~~~l~~~~~~~~~~~l~~~~~~~~~~~a~~aa~l~a~~p~~~~~~~~~~~~   167 (456) |
|  |  |  | ..+|||+++++.++...++ |.+..+.|+.++++++++++++|.++|+.. ++..|++++++++++|.......+...+ |
|  | T Consensus | 1 | f~~~pPl~~~l~~~~~~l~~g~~~~~~~l~~~l~~~l~~~~~y~l~r~~~--~~~~a~~a~~l~~~~p~~~~~~~~~~~~   78 (616) |
|  | T PF10131.10 | 1 | FRYWGPLSYYIMAGLMFLTSGDLLLAYRLIAFVIFVVGGLPWILWGIHEN--RRVLGTFFGVLWFFMPEHIRIYFTAGNL   78 (616) |
|  | T ss\_pred |  | CCCCchHHHHHHHHHHHHhcCCHHHHHHHHHHHHHHHHHHHHHHHHHHcC--cHHHHHHHHHHHHHCchHHHHHHhcCCH |
|  |
|  |
|  | Q ss\_pred |  | HHHHHHHHHHHHHHHHHHHHhcCCCCCCCCcchhHHHHHHHHHHHHHHHHHHHHHHHHHHHHHHHHHHhcccccchhHhh |
|  | Q XP\_016875493.1 | 168 | ADVLACLLFLLAFLSYNRSLDQGCVGGSFPSTVSPFFLLLSLFLGTCAMLVKETGITVFGVCLVYDLFSLSNKQDKSYLR   247 (456) |
|  | Q Consensus | 168 | ~~~~~~~~~~l~~~~~~~~~~~~~~~~~~~~~~~~~~~~~~~~~~~la~~~k~~~~~~~~~~~~~~~~~~~~~~~~~~~~   247 (456) |
|  |  |  | +|.+..++.+++++++.+..+++ +.++.++++++++++.++|+....+.++..+........+++++ |
|  | T Consensus | 79 | ~~~~~~~~~~l~l~~~~~~~~~~----------~~~~~~~~~l~~~l~~~~~~~~~~~~~~~~~~~~~~~~~~~~~~---   145 (616) |
|  | T PF10131.10 | 79 | PQMVTTMLVPYVIWFLWLYVRKK----------NNRAAVGLFVCMTLMSFTHLMVTAIMGVSAFLYLLIDQIWNKDT---   145 (616) |
|  | T ss\_pred |  | HHHHHHHHHHHHHHHHHHHHhcC----------CHHHHHHHHHHHHHHHHhcHHHHHHHHHHHHHHHHHHHHhCCCh--- |
|  |
|  |
|  | Q ss\_pred |  | ccCcchHHHhhHHHHHHHHHHHHHHHHHHHHHHHHhcCCCCccccCCCCcccchhHHHHHHHHHHHHHHH----HHHHHH |
|  | Q XP\_016875493.1 | 248 | ASSNRNFLLTMRPFLKRAILVLSYVLVILYFRLWIMGGSMPLFSEQDNPASFSPYILTRFLTYSYLLAFN----VWLLLA   323 (456) |
|  | Q Consensus | 248 | ~~~~~~~~~~~~~~~~~~~~~~~~~~~~~~~~~~~~~~~~~~~~~~~~~~~~~~~~~~~~~~~~~~~~~~----~~~~~~   323 (456) |
|  |  |  | +.........++..++...+.................. ................... .... |
|  | T Consensus | 146 | -----------~~~~~~~~~~~~~~~~~~~~~~~~~~~~~~~~~~~~~~--~~~~~~~~~~~~~~~~~~~~~~~~~~~--   210 (616) |
|  | T PF10131.10 | 146 | -----------RRKIFALIYMICGILTAGIWVIPSLKGGLVTSESGDGS--VMSTLIYPLTTSLNPFKRLSAGNDSFY--   210 (616) |
|  | T ss\_pred |  | -----------HHHHHHHHHHHHHHHHHHHHHHHHHccCCccCCCCccc--hhhhheechhhccCcchhccCCCCcch-- |
|  |
|  |
|  | Q ss\_pred |  | hHhhccccccCCCccccccccHHHHHHHHHHHHHHHHHHHHHHHHhccccchHHHHHHHHHHHHHhHhccccCCCccccc |
|  | Q XP\_016875493.1 | 324 | PVTLCYDWQVGSIPLVETIWDMRNLATIFLAVVMALLSLHCLAAFKRLEHKEVLVGLLFLVFPFIPASNLFFRVGFVVAE   403 (456) |
|  | Q Consensus | 324 | ~~~~~~~~~~~~~~~~~~~~~~~~~~~~~~~~~~~~~~~~~~~~~~~~~~~~~~~~~~~~~~~~~~~~~~~~~~~~~~~~   403 (456) |
|  |  |  | ......++.+......+++++......++.+++............+..... |
|  | T Consensus | 211 | -----------------------------~~~~~~~l~~~~~~~~~~~~~~~~~~~~~~~~~~~~~~~~~~~~~~~~~~~   261 (616) |
|  | T PF10131.10 | 211 | -----------------------------FGLAAVLIAIAGILLARGGKKAGFVFLLIMLACTTPAAYRILVKLPFSQLF   261 (616) |
|  | T ss\_pred |  | -----------------------------HHHHHHHHHHHHHHHHcCCChHHHHHHHHHHHhcchHHHHHHhhCCHHHhh |
|  |
|  |
|  | Q ss\_pred |  | ---cccchHHHHHHHHHHHHHHHHHHHhchhhHHHHHHHHHHHHHHHHHHHHHhcC |
|  | Q XP\_016875493.1 | 404 | ---RVLYMPSMGYCILFVHGLSKLCTWLNRCGATTLIVSTVLLLLLFSWKTVKQNE   456 (456) |
|  | Q Consensus | 404 | ---Ry~~~~~~~~~ll~~~~~~~~~~~~~~~~~~~~~~~~~~~~~~~~~~~~~~~~   456 (456) |
|  |  |  | ||..+. +++++++.++.+..++ ....+++++.+........... |
|  | T Consensus | 262 | ~~~r~~~~~--~~~~~~~~~~~~~~~~-------~~~~~~~~~~~~~~~~~~~~~~   308 (616) |
|  | T PF10131.10 | 262 | WMTRFAPMV--YGFFFSACLEWVRLKK-------KYCVLLAALLCVDSISCMNLDF   308 (616) |
|  | T ss\_pred |  | hHhHHHHHH--HHHHHHHHHHhhhhHH-------HHHHHHHHHHHHHHhhhccccc |
|  |
| --- | | | |
|  | Template alignmentCDD | | |
| 6. | PF02366.19 ; PMT ; Dolichyl-phosphate-mannose-protein mannosyltransferase | | |
|  | Probability: 99.64%, E-value: 2.3e-14, Score: 117.29, Aligned cols: 216, Identities: 15%, Similarity: 0.02, | | |
|  |
|  | Q ss\_pred |  | HHHHHHHHHHHHhhhcCCCccccchHHHHhCCCCCCCCCcccccccccccccCCCCCCCcccccchHHHHHHHHHHHhC- |
|  | Q XP\_016875493.1 | 30 | AALLAGASCLCYGRSLQGEFVHDDVWAIVNNPDVRPGAPLRWGIFTNDFWGKGMAENTSHKSYRPLCVLTFKLNIFLTG-   108 (456) |
|  | Q Consensus | 30 | ~~~l~~~~~~~~~~~~~~~~~~De~~~~~~~~~~~~~~~~~~~~~~~~~~~~~~~~~~~~~~~~Pl~~~~~~~~~~l~g-   108 (456) |
|  |  |  | +++++.............+..+||..+...+++..+++..... ++|.+...........| |
|  | T Consensus | 2 | ~~~~~~~~~~~~~~~~~~~~~~D~~~~~~~a~~~~~~~~~~~~-------------------~~~~~~~~~~~~~~~~~~   62 (247) |
|  | T PF02366.19 | 2 | FLTVVAFCVRAQRLMNPAKVVFEELRYYNYAVDYVNNKLLMDV-------------------YPPLGKLLFSLVAALTGN   62 (247) |
|  | T ss\_pred |  | hHHHHHHHHHHHHHhCCcccchHHHHHHHHHHHHHcCccccCC-------------------CCcHHHHHHHHHHHHcCC |
|  |
|  |
|  | Q ss\_pred |  | -----------------CCchHHHHHHHHHHHHHHHHHHHHHHHHhccchHHHHHHHHHHHHccccHHHHHHHHhHHHHH |
|  | Q XP\_016875493.1 | 109 | -----------------MNPFYFHAVNIILHCLVTLVLMYTCDKTVFKNRGLAFVTALLFAVHPIHTEAVAGIVGRADVL   171 (456) |
|  | Q Consensus | 109 | -----------------~~~~~~rl~~~l~~~~~~~~~~~l~~~~~~~~~~~a~~aa~l~a~~p~~~~~~~~~~~~~~~~   171 (456) |
|  |  |  | .+....|+.+.+++.+++..+|.++ |+..++++.+++++++++++|..... ....++|.+ |
|  | T Consensus | 63 | ~~~~~~~~~~~~~~~~~~~~~~~~~~~~~~~~~~~~~~~~~~-~~~~~~~~~a~~~~~~~~~~p~~~~~--~~~~~~d~~   139 (247) |
|  | T PF02366.19 | 63 | KYELNTLDEPGQQYPFTDVAYSMRLFTCLLGSLLVPLMYGTV-YFPTKSKTAASLAALFVIFDNGLITM--SRYIMIEIP   139 (247) |
|  | T ss\_pred |  | CcccccccCCCCCCCcchHHHHHHHHHHHHHHHHHHHHHHHH-hcccCCHHHHHHHHHHHHhchhHHHh--HhhccchHH |
|  |
|  |
|  | Q ss\_pred |  | HHHHHHHHHHHHHHH-------HhcCCCCCCCCcchhHHHHHHHHHHHHHHHHHHHHHHHHHHHHHHHHHHhcccccchh |
|  | Q XP\_016875493.1 | 172 | ACLLFLLAFLSYNRS-------LDQGCVGGSFPSTVSPFFLLLSLFLGTCAMLVKETGITVFGVCLVYDLFSLSNKQDKS   244 (456) |
|  | Q Consensus | 172 | ~~~~~~l~~~~~~~~-------~~~~~~~~~~~~~~~~~~~~~~~~~~~la~~~k~~~~~~~~~~~~~~~~~~~~~~~~~   244 (456) |
|  |  |  | ..++.+++++++.+. .+++ +.++..+++++.+++.++|+.+....+...+.......+++++ |
|  | T Consensus | 140 | ~~~~~~~~~~~~~~~~~~~~~~~~~~----------~~~~~~~~~~~~~l~~~~k~~~~~~~~~~~~~~~~~~~~~~~~-   208 (247) |
|  | T PF02366.19 | 140 | ALYFMSLTAFYWSVYEAQQKRPFSLR----------WHTSLLSTGVALGLALSTKLSAMFTFGWLLILAAFHLWNLLGD-   208 (247) |
|  | T ss\_pred |  | HHHHHHHHHHHHHHHHHhcCCCCCcH----------HHHHHHHHHHHHHHHHHhhHHHHHHHHHHHHHHHHHHHHhcCC- |
|  |
|  |
|  | Q ss\_pred |  | HhhccCcchHHHhhHHHHHHHHHHHHHHHHHHHHHHHHhcC |
|  | Q XP\_016875493.1 | 245 | YLRASSNRNFLLTMRPFLKRAILVLSYVLVILYFRLWIMGG   285 (456) |
|  | Q Consensus | 245 | ~~~~~~~~~~~~~~~~~~~~~~~~~~~~~~~~~~~~~~~~~   285 (456) |
|  |  |  | +......+.........++...++..+.....++ |
|  | T Consensus | 209 | -------~~~~~~~~~~~~~~~~~~~~~~p~~~~~~~~~~~   242 (247) |
|  | T PF02366.19 | 209 | -------LSVPMYRIVKHLFSYIFYLIGVPITVYLAVFAVH   242 (247) |
|  | T ss\_pred |  | -------CCCCHHHHHHHHHHHHHHHHHHHHHHHHHHHHHH |
|  |
| --- | | | |
|  | Template alignmentCDD | | |
| 7. | PF04188.14 ; Mannosyl\_trans2 ; Mannosyltransferase (PIG-V) | | |
|  | Probability: 99.64%, E-value: 6e-14, Score: 124.43, Aligned cols: 326, Identities: 12%, Similarity: -0.005, | | |
|  |
|  | Q ss\_pred |  | ccchHHHH--hCCCC-CCCCCcccccccccccccCCCCCCCcccccchHHHHHHHHHHHhCCC-----------chHHHH |
|  | Q XP\_016875493.1 | 51 | HDDVWAIV--NNPDV-RPGAPLRWGIFTNDFWGKGMAENTSHKSYRPLCVLTFKLNIFLTGMN-----------PFYFHA   116 (456) |
|  | Q Consensus | 51 | ~De~~~~~--~~~~~-~~~~~~~~~~~~~~~~~~~~~~~~~~~~~~Pl~~~~~~~~~~l~g~~-----------~~~~rl   116 (456) |
|  |  |  | |||.+|.. .+++- ...+ ....++|+++++......++|.+ ..+.|+ |
|  | T Consensus | 60 | wD~~~y~~~~ia~~g~y~~~--------------------~~~~f~Pl~p~l~~~~~~l~~~~~~~~~~~~~~~~~~~~~   119 (432) |
|  | T PF04188.14 | 60 | WDSVFFIKNITSKNGKPQFE--------------------HEYAFSQLWTFFVRLFIKSNNDSIYHALRVGVAIENVLFY   119 (432) |
|  | T ss\_pred |  | ccHHHHhhhhHHHcCCCccc--------------------ccccchHHHHHHHHHHHHhcccchHHHHHHHHHHHHHHHH |
|  |
|  |
|  | Q ss\_pred |  | HH-HHHHHHHHHHHHHHHHHHhccchHHHHHHHHHHHHccccHHHHHHHHhHHHHHHHHHHHHHHHHHHHHHhcCCCCCC |
|  | Q XP\_016875493.1 | 117 | VN-IILHCLVTLVLMYTCDKTVFKNRGLAFVTALLFAVHPIHTEAVAGIVGRADVLACLLFLLAFLSYNRSLDQGCVGGS   195 (456) |
|  | Q Consensus | 117 | ~~-~l~~~~~~~~~~~l~~~~~~~~~~~a~~aa~l~a~~p~~~~~~~~~~~~~~~~~~~~~~l~~~~~~~~~~~~~~~~~   195 (456) |
|  |  |  | ++ .+++++++..+|.++ +...++++.|..++++++++|..+.. .... +|.+..++.+++++++.+..++++ |
|  | T Consensus | 120 | ~s~~~~~~~~~~~ly~l~-~~~~~~~~~a~~a~~l~~~~P~~~~~-~~~~--~E~l~~~l~~~~~~~~~~~~~~~~----   191 (432) |
|  | T PF04188.14 | 120 | LSGIVLYFLTKKIFSQNI-RQSQFARTIAKKTSLLFFLTSAAGFL-TSIY--SEPLSFFFAFVGIWSRECSISVPV----   191 (432) |
|  | T ss\_pred |  | HHHHHHHHHHHHHHHhhh-HhhhccHHHHHHHHHHHhhCCchHHh-hccC--cHHHHHHHHHHHHHHHHhccCCCC---- |
|  |
|  |
|  | Q ss\_pred |  | CCcchhHHHHHHHHHHHHHHHH-HHHHHHHHHHHHHHHHHH--------hcccccchhHhhccCcchHHHhhHHHHHHHH |
|  | Q XP\_016875493.1 | 196 | FPSTVSPFFLLLSLFLGTCAML-VKETGITVFGVCLVYDLF--------SLSNKQDKSYLRASSNRNFLLTMRPFLKRAI   266 (456) |
|  | Q Consensus | 196 | ~~~~~~~~~~~~~~~~~~la~~-~k~~~~~~~~~~~~~~~~--------~~~~~~~~~~~~~~~~~~~~~~~~~~~~~~~   266 (456) |
|  |  |  | +.++.+.++++.+++.+ +|.+++...+......+. .+++.++.. +....... |
|  | T Consensus | 192 | -----~~~~~~~~~~~~~la~~~~R~~g~~~~~~~~~~~l~~~~~~~~~~~~~~~~~~--------------~~~~~~~~   252 (432) |
|  | T PF04188.14 | 192 | -----LGQFDISWRYWFPYSFISMACFTLASLNRSNCVLLGIYFIFDLIELTKNRKFV--------------KAICFPLL   252 (432) |
|  | T ss\_pred |  | -----CCchhHHHHHHHHHHHHHHHhHHHHHHHHHHHHHHHHHHHHHHHHhhhhHHHH--------------HHHHHHHH |
|  |
|  |
|  | Q ss\_pred |  | HHHHHHHHHHHHHHHH-hcCCCCccccCCCCcccc--------hhHHHHHHHHHHHHHHHHHHHHHhHhhccccccCCCc |
|  | Q XP\_016875493.1 | 267 | LVLSYVLVILYFRLWI-MGGSMPLFSEQDNPASFS--------PYILTRFLTYSYLLAFNVWLLLAPVTLCYDWQVGSIP   337 (456) |
|  | Q Consensus | 267 | ~~~~~~~~~~~~~~~~-~~~~~~~~~~~~~~~~~~--------~~~~~~~~~~~~~~~~~~~~~~~~~~~~~~~~~~~~~   337 (456) |
|  |  |  | ..++...++....... .................. .....+..........+......|.... |
|  | T Consensus | 253 | ~~~~~~~p~~~~~~~~~~~~f~~~~~~w~~~~~~~~~~~~~~~~~~~~q~~yw~~g~~~~~~~~~~~~~ll---------   323 (432) |
|  | T PF04188.14 | 253 | SGSLMFSALLYQQYYLPYKTFCPQRGEWCKSQLFSSIFITKTSLYSYIQSHYWGVGLLKYWTPNNIPNFLF---------   323 (432) |
|  | T ss\_pred |  | HHHHHHHHHHHHHHHHHHHHHCCCCChhHhCCCCCchhhcccchHHHHHHHcCCCcchhcCCccchhHHHh--------- |
|  |
|  |
|  | Q ss\_pred |  | cccccccHHHHHHHHHHHHHHHHHHHHHHHHhccccchHHHHHHHHHHHHHhHhccccCCCccccccccchHHHHHHHHH |
|  | Q XP\_016875493.1 | 338 | LVETIWDMRNLATIFLAVVMALLSLHCLAAFKRLEHKEVLVGLLFLVFPFIPASNLFFRVGFVVAERVLYMPSMGYCILF   417 (456) |
|  | Q Consensus | 338 | ~~~~~~~~~~~~~~~~~~~~~~~~~~~~~~~~~~~~~~~~~~~~~~~~~~~~~~~~~~~~~~~~~~Ry~~~~~~~~~ll~   417 (456) |
|  |  |  | .....+..........+++++.........+........... ......||.. ..|++.+.+ |
|  | T Consensus | 324 | --------------~~p~~~l~~~~~~~~~~~~~~~~~~~~~~~~~~~~~~~~~~~----~~~~~~R~~~-~~P~l~~~~   384 (432) |
|  | T PF04188.14 | 324 | --------------AVPNIIILIYSSIYFSKIYPSYNLKALVWITRALVVIVCFFA----HVQILNRIAS-FLPLHLWYL   384 (432) |
|  | T ss\_pred |  | --------------HHHHHHHHHHHHHHHHhhCCccccHHHHHHHHHHHHHHHHHH----HHHHHHHHHh-ccHHHHHHH |
|  |
|  |
|  | Q ss\_pred |  | HHHHHHHHHHhchhh-------HHHHHHHHHHHHHHHHHHH |
|  | Q XP\_016875493.1 | 418 | VHGLSKLCTWLNRCG-------ATTLIVSTVLLLLLFSWKT   451 (456) |
|  | Q Consensus | 418 | ~~~~~~~~~~~~~~~-------~~~~~~~~~~~~~~~~~~~   451 (456) |
|  |  |  | +..+.+..++.++.. .+......++......... |
|  | T Consensus | 385 | a~~~~~~~~~~~~~~~~~~~~~~~~~~~~~~~~~~~~~~l~   425 (432) |
|  | T PF04188.14 | 385 | ADRLVKTSDPKKMENPKGDDKIVKFYIYWLAFWIPLQTILF   425 (432) |
|  | T ss\_pred |  | HHHHhcCCCCCcCCCCCCCCHHHHHHHHHHHHHHHHHHHHH |
|  |
| --- | | | |
|  | Template alignmentCDD | | |
| 8. | PF10034.10 ; Dpy19 ; Q-cell neuroblast polarisation | | |
|  | Probability: 99.61%, E-value: 8.4e-13, Score: 124.12, Aligned cols: 362, Identities: 10%, Similarity: -0.034, | | |
|  |
|  | Q ss\_pred |  | CCCccccchHHHHhCCCCCCCCCccccccc--ccccccCCCCCCCcccccchHHHHHHHHHHHhC--------------- |
|  | Q XP\_016875493.1 | 46 | QGEFVHDDVWAIVNNPDVRPGAPLRWGIFT--NDFWGKGMAENTSHKSYRPLCVLTFKLNIFLTG---------------   108 (456) |
|  | Q Consensus | 46 | ~~~~~~De~~~~~~~~~~~~~~~~~~~~~~--~~~~~~~~~~~~~~~~~~Pl~~~~~~~~~~l~g---------------   108 (456) |
|  |  |  | ..++..||..|+..++++.++++...+... .+..............+++....+.+..++++| |
|  | T Consensus | 30 | ~~~~~~d~~~y~~~~~~i~~~~~~~~~~~~~~~d~~~~~p~g~~~~~~~~~~~~~~~a~~~~~~~~~~~~~~~~~~~~~~   109 (651) |
|  | T PF10034.10 | 30 | ELSFRTEMGLYYSYYKTMVEAPTFLDGLHAVMNCNVTEYPDTVNTLKRFNLYPEVILAGKFRIFEWLASKFEYQTKTCYT   109 (651) |
|  | T ss\_pred |  | hhccCcchhhHHhHHhhhccCCCHHHHHHHHHcCCCCcCCCCchHHhhcchHHHHHHHHHHHHHHHHHhhcCcCccchhe |
|  |
|  |
|  | Q ss\_pred |  | -----------------CCchHHHHHHHHHHHHHHHHHHHHHHHHhccchH-HHHHHHHHHHHccccHHHHHHHHhHHHH |
|  | Q XP\_016875493.1 | 109 | -----------------MNPFYFHAVNIILHCLVTLVLMYTCDKTVFKNRG-LAFVTALLFAVHPIHTEAVAGIVGRADV   170 (456) |
|  | Q Consensus | 109 | -----------------~~~~~~rl~~~l~~~~~~~~~~~l~~~~~~~~~~-~a~~aa~l~a~~p~~~~~~~~~~~~~~~   170 (456) |
|  |  |  | ...........++++++++.+|.++|+.. +++ .|++++++++++|.++..+.......|. |
|  | T Consensus | 110 | ~~~~~~~~~~~~~~~~~~p~~~~~~~~~i~~~l~v~~~y~l~~~l~--~~~~~al~aall~a~~p~~~~~~~~g~~~~~~   187 (651) |
|  | T PF10034.10 | 110 | VNRGYGLPPVQSCEGLGELSFFYVYSIFFLTGLMMACFFILCFYLS--GSILGGVLGTLCYFFNHGEATRVMWTPPLRES   187 (651) |
|  | T ss\_pred |  | eecCCCCCCcccCCCcCchHHHHHHHHHHHHHHHHHHHHHHHHHHc--CChHHHHHHHHHHHhChhhhhhHhhCCCcccc |
|  |
|  |
|  | Q ss\_pred |  | HHHHHHHHHHHHHHHHHhcCCCCCCCCcchhHHHHHHHHHHHHHHHHHHHHHHHHHHHHHHHHHHhcccccchhHhhccC |
|  | Q XP\_016875493.1 | 171 | LACLLFLLAFLSYNRSLDQGCVGGSFPSTVSPFFLLLSLFLGTCAMLVKETGITVFGVCLVYDLFSLSNKQDKSYLRASS   250 (456) |
|  | Q Consensus | 171 | ~~~~~~~l~~~~~~~~~~~~~~~~~~~~~~~~~~~~~~~~~~~la~~~k~~~~~~~~~~~~~~~~~~~~~~~~~~~~~~~   250 (456) |
|  |  |  | +..++.+++++++.+..+++++ +.++.++++++.+++..+|...........+..+.....++.++... |
|  | T Consensus | 188 | ~~~~f~~l~l~~~~~~~~~~~~--------~~~~~~l~gl~~~l~~~~~~~~~~~~~~~~~~~~~~~~~~~~~~~~~---   256 (651) |
|  | T PF10034.10 | 188 | FSYPYLVAQLLVVTFTLRSVKV--------TWRHITLVSMTTALFMIPWQFAQFALLTQTCALFVVYIMHFITADKF---   256 (651) |
|  | T ss\_pred |  | CHHHHHHHHHHHHHHHHhCCCC--------CHHHHHHHHHHHHHHHHHhhhHHHHHHHHHHHHHHHHHhhcCCHHHH--- |
|  |
|  |
|  | Q ss\_pred |  | cchHHHhhHH--------------------------------------------------HHHHHHHHHHHHHHHHHHHH |
|  | Q XP\_016875493.1 | 251 | NRNFLLTMRP--------------------------------------------------FLKRAILVLSYVLVILYFRL   280 (456) |
|  | Q Consensus | 251 | ~~~~~~~~~~--------------------------------------------------~~~~~~~~~~~~~~~~~~~~   280 (456) |
|  |  |  | +. .................... |
|  | T Consensus | 257 | --------~~~~~~~~~~~~~~~~~~~~~~~~~~~~~~~~~~~~~~~~~~~~~~~~~~~~~~~~~~~~~~~~~~~~~~~~   328 (651) |
|  | T PF10034.10 | 257 | --------CKILYGLLVAHLLNFAVQFGNSMLLSSFFMSAVISALVVAKAESQIHKLPYQLLIWATQGLGFAAGTLGIKV   328 (651) |
|  | T ss\_pred |  | --------HHHHHHHHHHHHHHHHHHccchHHHHHHHHHHHHHHHHHHHHHhhhccCCHHHHHHHHHHHHHHHHHHHHHH |
|  |
|  |
|  | Q ss\_pred |  | HHhcCCCCcc-ccCCCCcccchhHHHHHHHHHHHHHHHHHHHHHhHhhccccccCCCccccccccHHHHHHHHHHHHHHH |
|  | Q XP\_016875493.1 | 281 | WIMGGSMPLF-SEQDNPASFSPYILTRFLTYSYLLAFNVWLLLAPVTLCYDWQVGSIPLVETIWDMRNLATIFLAVVMAL   359 (456) |
|  | Q Consensus | 281 | ~~~~~~~~~~-~~~~~~~~~~~~~~~~~~~~~~~~~~~~~~~~~~~~~~~~~~~~~~~~~~~~~~~~~~~~~~~~~~~~~   359 (456) |
|  |  |  | .......... ...................................... ............. |
|  | T Consensus | 329 | ~~~~~~~~~~~~~~~~~~~~~~~~~~~~~~~~~~~~~~~~~~~~~~~~~------------------~~~~~~~~~~~~~   390 (651) |
|  | T PF10034.10 | 329 | AVAKVLSIADDSKFTSYRDFHTLLYTCAPEFDFLDQEAPVKLTKTLLLP------------------SAIVAASAVIAKV   390 (651) |
|  | T ss\_pred |  | HHHHhcCcccccccCCCCCHHHHHHhcchhcCCCChHHHHHHHHhcHHH------------------HHHHHHHHHHHHH |
|  |
|  |
|  | Q ss\_pred |  | HHHHHHHHHhccccch------------------------HHHHHHHHHHHHHhHhccccCCCccccccccchHHHHHHH |
|  | Q XP\_016875493.1 | 360 | LSLHCLAAFKRLEHKE------------------------VLVGLLFLVFPFIPASNLFFRVGFVVAERVLYMPSMGYCI   415 (456) |
|  | Q Consensus | 360 | ~~~~~~~~~~~~~~~~------------------------~~~~~~~~~~~~~~~~~~~~~~~~~~~~Ry~~~~~~~~~l   415 (456) |
|  |  |  | ........+++++++. ..+.+++.+......... .||..+..|++++ |
|  | T Consensus | 391 | ~~~~~~~~~~~~~~~~~~~~~~~~~~~~~~~~~~~~~~~~~~~~~~~~~~~~~~~~~~---------~R~~~~~~p~l~i   461 (651) |
|  | T PF10034.10 | 391 | GASEWEYWVRGKKSQVKSDSADEHDEGAQGQEANPRPHAEYVYHVLQAMAFVLMAVII---------MRLKLFGTPALCV   461 (651) |
|  | T ss\_pred |  | HHHHHHHHHhcccccCCCCCccccccccccccCCCCCchHHHHHHHHHHHHHHHHHHH---------HHHHHHHHHHHHH |
|  |
|  |
|  | Q ss\_pred |  | HHHHHHHH-HHHHhchhhHHHHHHHHHHHHHHHHHHHHHhc |
|  | Q XP\_016875493.1 | 416 | LFVHGLSK-LCTWLNRCGATTLIVSTVLLLLLFSWKTVKQN   455 (456) |
|  | Q Consensus | 416 | l~~~~~~~-~~~~~~~~~~~~~~~~~~~~~~~~~~~~~~~~   455 (456) |
|  |  |  | +++.++.+ +.+..+++..+....+++++...........+ |
|  | T Consensus | 462 | l~a~~~~~~~~~~~~~~~~~~~~~~~~~~~~~~~~~~~~~~   502 (651) |
|  | T PF10034.10 | 462 | LASLVASRQFFSFLGDRRRHQAIVIALIAVMSVQGFSNLKT   502 (651) |
|  | T ss\_pred |  | HHHHHhcHHHHHhhhHHHHHHHHHHHHHHHHHhcchhhHHH |
|  |
| --- | | | |
|  | Template alignmentCDD | | |
| 9. | PF12250.9 ; AftA\_N ; Arabinofuranosyltransferase N terminal | | |
|  | Probability: 99.56%, E-value: 3.6e-12, Score: 111.79, Aligned cols: 341, Identities: 10%, Similarity: -0.03, | | |
|  |
|  | Q ss\_pred |  | HHHHHHHHHHHHHHHhhhcCCCccccchHHHHhCCCCCCCCCcccccccccccccCCCCCCCcccccchHHHHHHHHHHH |
|  | Q XP\_016875493.1 | 27 | AGAAALLAGASCLCYGRSLQGEFVHDDVWAIVNNPDVRPGAPLRWGIFTNDFWGKGMAENTSHKSYRPLCVLTFKLNIFL   106 (456) |
|  | Q Consensus | 27 | ~~~~~~l~~~~~~~~~~~~~~~~~~De~~~~~~~~~~~~~~~~~~~~~~~~~~~~~~~~~~~~~~~~Pl~~~~~~~~~~l   106 (456) |
|  |  |  | ...+....+............+...||.++....+.+.++.......+.+ ..+.|||+++++.+....+ |
|  | T Consensus | 76 | ~~~l~~~~l~~~L~~t~~~~~gl~~D~~~~~~~~~~~~~~~~~~d~~~~~-----------~~~~YPPl~~~l~~~~~~l   144 (432) |
|  | T PF12250.9 | 76 | PAALVITTLGIPLSATRLYLDGINVDQGFRTQFLTWMGYTIHLSDMNYID-----------MPSYYPGAWFWIGGRLANL   144 (432) |
|  | T ss\_pred |  | HHHHHHHHHHHHHhcCcccCCCccccHHHHHHHHHHHHhccccccccCCC-----------CcccCChHHHHHHHHHHHH |
|  |
|  |
|  | Q ss\_pred |  | hCCCc-hHHHHHHHHHHHHHHHHHHHHHHHHhccch-HHH-HHHHHHHHHccccHHHHHHHHhHHHHHHHHHHHHHHHHH |
|  | Q XP\_016875493.1 | 107 | TGMNP-FYFHAVNIILHCLVTLVLMYTCDKTVFKNR-GLA-FVTALLFAVHPIHTEAVAGIVGRADVLACLLFLLAFLSY   183 (456) |
|  | Q Consensus | 107 | ~g~~~-~~~rl~~~l~~~~~~~~~~~l~~~~~~~~~-~~a-~~aa~l~a~~p~~~~~~~~~~~~~~~~~~~~~~l~~~~~   183 (456) |
|  |  |  | +|.+. ...|..+++...++....|.+.|+.. ++ ..+ .++++..++.|..... ...+.....+....++.+ |
|  | T Consensus | 145 | ~G~~~~~a~r~~~~l~~~l~~~~~y~l~r~l~--~~~~~al~ia~~~~~~~~~~~~~-----~~y~~l~~~~l~~~l~~~   217 (432) |
|  | T PF12250.9 | 145 | LGLAGWEVFQPWALISLATAGSILVPVWQRIC--GSLTVASGIALVTTSITIVMSAD-----EPYAAIITMGVPAATVMM   217 (432) |
|  | T ss\_pred |  | hCCCHHHHhHHHHHHHHHHHHHHHHHHHHHHH--CCHHHHHHHHHHHHHHHHhcCCC-----CcHHHHHHHHHHHHHHHH |
|  |
|  |
|  | Q ss\_pred |  | HHHHhcCCCCCCCCcchhHHHHHHHHHHHHHHHHHHHHHHHHHHHHHH--HHHHhcccccchhHhhccCcchHHHhhHHH |
|  | Q XP\_016875493.1 | 184 | NRSLDQGCVGGSFPSTVSPFFLLLSLFLGTCAMLVKETGITVFGVCLV--YDLFSLSNKQDKSYLRASSNRNFLLTMRPF   261 (456) |
|  | Q Consensus | 184 | ~~~~~~~~~~~~~~~~~~~~~~~~~~~~~~la~~~k~~~~~~~~~~~~--~~~~~~~~~~~~~~~~~~~~~~~~~~~~~~   261 (456) |
|  |  |  | .|..++ +++..+..+++.+++.++|............ .......+++.++..+ |
|  | T Consensus | 218 | ~r~l~~-----------~~~~~~~~gl~lgl~~l~y~~~~~~~~~~~~~~~~l~~~~~~~~~~~~~--------------   272 (432) |
|  | T PF12250.9 | 218 | RRALTG-----------SLWPLIGLTLYIGVSAAMYTLFTAVVALSVCVMAALFAVVFDHSIKPLL--------------   272 (432) |
|  | T ss\_pred |  | HHHhcC-----------CchHHHHHHHHHHHHHHHhHHHHHHHHHHHHHHHHHHHHHcCCCchHHH-------------- |
|  |
|  |
|  | Q ss\_pred |  | HHHHHHHHHHHHHHHHHHHHHhcCCCCccccCCCCcccchhHHHHHHHHHHHHHHHHHHHHHhHhhccccccCCCccccc |
|  | Q XP\_016875493.1 | 262 | LKRAILVLSYVLVILYFRLWIMGGSMPLFSEQDNPASFSPYILTRFLTYSYLLAFNVWLLLAPVTLCYDWQVGSIPLVET   341 (456) |
|  | Q Consensus | 262 | ~~~~~~~~~~~~~~~~~~~~~~~~~~~~~~~~~~~~~~~~~~~~~~~~~~~~~~~~~~~~~~~~~~~~~~~~~~~~~~~~   341 (456) |
|  |  |  | +......+..+....+...............+...+.-.. +.... |
|  | T Consensus | 273 | -~~~~~~~~a~~~a~~~~~P~l~~~~~~~~~~~~~~~~~~~----------------------------------~~~~~   317 (432) |
|  | T PF12250.9 | 273 | -RLLIIGTGSALIASTVWAPYLTAILSGQPHSGATAMHYLP----------------------------------PTGAQ   317 (432) |
|  | T ss\_pred |  | -HHHHHHHHHHHHHHHHHHHHHHHHHhCCCCCCcchHhcCC----------------------------------ccccC |
|  |
|  |
|  | Q ss\_pred |  | cccHHHHHHHHHHHHHHHHHHHHHHHHhccccchHHHHHHHHHHHHHhHhccccCCCccccccccchHHHHHHHHHHHHH |
|  | Q XP\_016875493.1 | 342 | IWDMRNLATIFLAVVMALLSLHCLAAFKRLEHKEVLVGLLFLVFPFIPASNLFFRVGFVVAERVLYMPSMGYCILFVHGL   421 (456) |
|  | Q Consensus | 342 | ~~~~~~~~~~~~~~~~~~~~~~~~~~~~~~~~~~~~~~~~~~~~~~~~~~~~~~~~~~~~~~Ry~~~~~~~~~ll~~~~~   421 (456) |
|  |  |  | ...+.........+.++..+......+ ++..+.....+.......+...............|+..+..+.+++.++.++ |
|  | T Consensus | 318 | ~~~p~~~~~~~~~L~l~Glv~l~~~~r-~~~~~~l~~~~~~~y~w~~~~~~~~~~~~~ll~~R~~~~l~~~l~~~~a~gi   396 (432) |
|  | T PF12250.9 | 318 | VPMPMLQFNLVGLLCLLGLAYLIVRIA-DPDVRSMLIAQIVFYGWIVTSMIVSLSGKTLLGFRLDAIITIQLATAGMLAL   396 (432) |
|  | T ss\_pred |  | CCCccccccHHHHHHHHHHHHHHHHhc-CHhHHHHHHHHHHHHHHHHHHHHHHHhCCCchhHhHHHHHHHHHHHHHHHHH |
|  |
|  |
|  | Q ss\_pred |  | HHH-----H----HHhchhhHHHHHHHHHHHHHH |
|  | Q XP\_016875493.1 | 422 | SKL-----C----TWLNRCGATTLIVSTVLLLLL   446 (456) |
|  | Q Consensus | 422 | ~~~-----~----~~~~~~~~~~~~~~~~~~~~~   446 (456) |
|  |  |  | .++ . ++.+....+....+...+... |
|  | T Consensus | 397 | ~~l~~~~~~~~~~~~~~~~~~~~~~~~~~~~~~~   430 (432) |
|  | T PF12250.9 | 397 | AELRLVEIPRFYPAVTRPATATTVTRVMVAILAI   430 (432) |
|  | T ss\_pred |  | HHHHHccCccccccccCcccchHHHHHHHHHHHH |
|  |
| --- | | | |
|  | Template alignmentCDD | | |
| 10. | PF11028.9 ; DUF2723 ; Protein of unknown function (DUF2723) | | |
|  | Probability: 99.55%, E-value: 1.5e-13, Score: 106.88, Aligned cols: 155, Identities: 16%, Similarity: 0.157, | | |
|  |
|  | Q ss\_pred |  | cchHHHHhCCCCCCCCCcccccccccccccCCCCCCCcccccchHHHHHHHHHHHh--CCCchHHHHHHHHHHHHHHHHH |
|  | Q XP\_016875493.1 | 52 | DDVWAIVNNPDVRPGAPLRWGIFTNDFWGKGMAENTSHKSYRPLCVLTFKLNIFLT--GMNPFYFHAVNIILHCLVTLVL   129 (456) |
|  | Q Consensus | 52 | De~~~~~~~~~~~~~~~~~~~~~~~~~~~~~~~~~~~~~~~~Pl~~~~~~~~~~l~--g~~~~~~rl~~~l~~~~~~~~~   129 (456) |
|  |  |  | ||..|...+.+....+.. ..|++..+......++ |.+....|+.+.+++.+++.++ |
|  | T Consensus | 1 | D~~~y~~~a~~~~~~~~p----------------------~~~l~~~~~~~~~~~~~~~~~~~~~r~~~~l~~~l~~~~~   58 (188) |
|  | T PF11028.9 | 1 | DCGEYITAANKLEVGHPP----------------------GAPLFMLLGRLFSFFAEPEMVAVWINRLSALCSSFTILFL   58 (188) |
|  | T ss\_pred |  | ChHHHHHHHhhcCCCCCC----------------------ChHHHHHHHHHHHhcCCCCcHHHHHHHHHHHHHHHHHHHH |
|  |
|  |
|  | Q ss\_pred |  | HHHHHHHhccchH------------------HHHHHHHHHHHccccHHHHHHHHhHHHHHHHHHHHHHHHHHHHHHhcCC |
|  | Q XP\_016875493.1 | 130 | MYTCDKTVFKNRG------------------LAFVTALLFAVHPIHTEAVAGIVGRADVLACLLFLLAFLSYNRSLDQGC   191 (456) |
|  | Q Consensus | 130 | ~~l~~~~~~~~~~------------------~a~~aa~l~a~~p~~~~~~~~~~~~~~~~~~~~~~l~~~~~~~~~~~~~   191 (456) |
|  |  |  | |.++|+.. +++ .+++++++++++|..... ....++|.+..++.+++++++.+..+++ |
|  | T Consensus | 59 | ~~~~~~~~--~~~~~~~~~~~~~~~~~~~~~~a~~a~~l~~~~p~~~~~--s~~~~~d~~~~~~~~~~l~~~~~~~~~~-   133 (188) |
|  | T PF11028.9 | 59 | YWSITMFA--KKIMQRKDRDWSRGDQIATLGAGIIGALAYTFSDSFWFS--AVEGEVYAMSSLFTAAIFWMILKWDAEM-   133 (188) |
|  | T ss\_pred |  | HHHHHHHH--HHHHhccccccccchHHHHHHHHHHHHHHHHHchhHHHH--hhhcchhHHHHHHHHHHHHHHHHHHHhc- |
|  |
|  |
|  | Q ss\_pred |  | CCCCCCcchhH-------------HHHHHHHHHHHHHHHHHHHHHHHHHHHHHHHHHhcccccc |
|  | Q XP\_016875493.1 | 192 | VGGSFPSTVSP-------------FFLLLSLFLGTCAMLVKETGITVFGVCLVYDLFSLSNKQD   242 (456) |
|  | Q Consensus | 192 | ~~~~~~~~~~~-------------~~~~~~~~~~~la~~~k~~~~~~~~~~~~~~~~~~~~~~~   242 (456) |
|  |  |  | +. ++.++++++.+++.++|+.++.+++..+++.+...+++++ |
|  | T Consensus | 134 | ---------~~~~~~~~~~~~~~~~~~~l~g~~~~la~~~k~~~~~~~~~~~~~~~~~~~~~~~   188 (188) |
|  | T PF11028.9 | 134 | ---------IGIKHGEIKDSRSPMRWMILIWFMFGLAIGVHLLGLLAVPAIAYVIYFNLWEKTN   188 (188) |
|  | T ss\_pred |  | ---------cCccCCCcCCCCChHHHHHHHHHHHHHHHHHHHHHHHHHHHHHHHHHHhhHHhcC |
|  |
| --- | | | |
|  | Template alignmentCDD | | |
| 11. | PF13231.7 ; PMT\_2 ; Dolichyl-phosphate-mannose-protein mannosyltransferase | | |
|  | Probability: 99.54%, E-value: 3.7e-13, Score: 101.75, Aligned cols: 154, Identities: 16%, Similarity: 0.193, | | |
|  |
|  | Q ss\_pred |  | cccchHHHHHHHHHHHhCCCchHHHHHHHHHHHHHHHHHHHHHHHHhccchHHHHHHHHHHHHccccHHHHHHHHhHHHH |
|  | Q XP\_016875493.1 | 91 | SYRPLCVLTFKLNIFLTGMNPFYFHAVNIILHCLVTLVLMYTCDKTVFKNRGLAFVTALLFAVHPIHTEAVAGIVGRADV   170 (456) |
|  | Q Consensus | 91 | ~~~Pl~~~~~~~~~~l~g~~~~~~rl~~~l~~~~~~~~~~~l~~~~~~~~~~~a~~aa~l~a~~p~~~~~~~~~~~~~~~   170 (456) |
|  |  |  | +|||+++++.+....++|.+....|+.+.+++.+++..+|.+.|+.. +++.+..++.+++++|..... ....++|. |
|  | T Consensus | 1 | ~~~P~~~~~~~~~~~l~g~~~~~~~~~~~~~~~~~~~~~~~~~~~~~--~~~~~~~~~~~~~~~p~~~~~--~~~~~~~~   76 (159) |
|  | T PF13231.7 | 1 | DKPPASLWVMELSTRIFGVNSWAMLVPQALLGVAAVALLYATVRRRF--GAVAGLLAGLILAVTPVAAMM--FRFNNPDA   76 (159) |
|  | T ss\_pred |  | CCChHHHHHHHHHHHHHCCCHHHHHHHHHHHHHHHHHHHHHHHHHHH--HHHHHHHHHHHHHhcHHHHHh--hhcCCHHH |
|  |
|  |
|  | Q ss\_pred |  | HHHHHHHHHHHHHHHHHhcCCCCCCCCcchhHHHHHHHHHHHHHHHHHHHHHHHHHHHHHH--HHHHhcccccchhHhhc |
|  | Q XP\_016875493.1 | 171 | LACLLFLLAFLSYNRSLDQGCVGGSFPSTVSPFFLLLSLFLGTCAMLVKETGITVFGVCLV--YDLFSLSNKQDKSYLRA   248 (456) |
|  | Q Consensus | 171 | ~~~~~~~l~~~~~~~~~~~~~~~~~~~~~~~~~~~~~~~~~~~la~~~k~~~~~~~~~~~~--~~~~~~~~~~~~~~~~~   248 (456) |
|  |  |  | ...++..++++...+..+++ +.+....++++.+++..+|+......+...+ +....+++++++. |
|  | T Consensus | 77 | ~~~~~~~~~~~~~~~~~~~~----------~~~~~~~~~~~~~l~~~~k~~~~~~~~~~~~~~~~~~~~~~~~~~~----   142 (159) |
|  | T PF13231.7 | 77 | LLVLLMIAATWAMLRAVEDG----------RWRWLIVCGAFVGVGFLTKQLAVMLIVPGLALTYLVAGPPKIGVRI----   142 (159) |
|  | T ss\_pred |  | HHHHHHHHHHHHHHHHHHcC----------ChHHHHHHHHHHHHHHHcccchHHhhHHHHHHHHHHhCChhHHHHH---- |
|  |
|  |
|  | Q ss\_pred |  | cCcchHHHhhHHHHHHHHHHHHHHHHHH |
|  | Q XP\_016875493.1 | 249 | SSNRNFLLTMRPFLKRAILVLSYVLVIL   276 (456) |
|  | Q Consensus | 249 | ~~~~~~~~~~~~~~~~~~~~~~~~~~~~   276 (456) |
|  |  |  | +......+...... |
|  | T Consensus | 143 | --------------~~~~~~~~~~~~~~   156 (159) |
|  | T PF13231.7 | 143 | --------------AQLFAAGTSMIVAA   156 (159) |
|  | T ss\_pred |  | --------------HHHHHHHHHHHHHH |
|  |
| --- | | | |
|  | Template alignmentCDD | | |
| 12. | PF09913.10 ; DUF2142 ; Predicted membrane protein (DUF2142) | | |
|  | Probability: 99.53%, E-value: 2.1e-12, Score: 113.5, Aligned cols: 326, Identities: 13%, Similarity: 0.023, | | |
|  |
|  | Q ss\_pred |  | HHHhhhcCCCccccchHHHHhCCCCCCCCCcccccccccccccCCCCCCCccc--------------------------- |
|  | Q XP\_016875493.1 | 39 | LCYGRSLQGEFVHDDVWAIVNNPDVRPGAPLRWGIFTNDFWGKGMAENTSHKS---------------------------   91 (456) |
|  | Q Consensus | 39 | ~~~~~~~~~~~~~De~~~~~~~~~~~~~~~~~~~~~~~~~~~~~~~~~~~~~~---------------------------   91 (456) |
|  |  |  | ..+....+.....||..|+..+..+.+++.. ....+....+.......... |
|  | T Consensus | 4 | ~~~~~~~P~~~~pDE~~H~~~a~~ia~g~~~--~~~~~~~~~~~~~~~~~~~~~~~~~~~~~~~~~~~~~~~~~~~~~~~   81 (405) |
|  | T PF09913.10 | 4 | LAFAVVMPPFQVPDEDGHFIRAYLISRGEFV--GRGAPRVPGTVVLSMMRYPEMGERFGRFKPRELVRDLIPHPGSVSPE   81 (405) |
|  | T ss\_pred |  | eehhhccCCCCCCChHHHHHHHHHHHcCcee--eecCCCCCCcccchhccCcccccccCCCCchHHHhccCCCCCCCCCC |
|  |
|  |
|  | Q ss\_pred |  | -------------------------ccchHHHHHHHHH---HHhCCC----chHHHHHHHHHHHHHHHHHHHHHHHHhcc |
|  | Q XP\_016875493.1 | 92 | -------------------------YRPLCVLTFKLNI---FLTGMN----PFYFHAVNIILHCLVTLVLMYTCDKTVFK   139 (456) |
|  | Q Consensus | 92 | -------------------------~~Pl~~~~~~~~~---~l~g~~----~~~~rl~~~l~~~~~~~~~~~l~~~~~~~   139 (456) |
|  |  |  | +||+++++.+... .++|.+ ....|+.+++++++++.+++.++++.. |
|  | T Consensus | 82 | ~~~~~~~~~~~~~~~~~~~~~~~~~~pPl~y~~~a~~~~l~~~~~~~~~~~~~~~Rl~s~l~~~~~~~~~~~~~~~~~--   159 (405) |
|  | T PF09913.10 | 82 | VPSLNLGNLDVRHRWLPWSIIGSSLYCPLVYMPASLGIATVRILSGSPLLMMYGARLFNVIVFAAALAISFRLAPRYR--   159 (405) |
|  | T ss\_pred |  | ccccccccCCCcccccCccccCccccCHHhHHHHHHHHHHHHHccCCHHHHHHHHHHHHHHHHHHHHHHHHHHChhhH-- |
|  |
|  |
|  | Q ss\_pred |  | chHHHHHHHHHHHHccccHHHHHHHHhHHHHHHHHHHHHHHHHHHHHHhcCCCCCCCCcchhHHHHHHHHHHHHHHHHHH |
|  | Q XP\_016875493.1 | 140 | NRGLAFVTALLFAVHPIHTEAVAGIVGRADVLACLLFLLAFLSYNRSLDQGCVGGSFPSTVSPFFLLLSLFLGTCAMLVK   219 (456) |
|  | Q Consensus | 140 | ~~~~a~~aa~l~a~~p~~~~~~~~~~~~~~~~~~~~~~l~~~~~~~~~~~~~~~~~~~~~~~~~~~~~~~~~~~la~~~k   219 (456) |
|  |  |  | .++++++++|..+..+...+ +|.+..++.+++++++.+..+++. +++.+++.+++.+++.++| |
|  | T Consensus | 160 | ------~~~~~~a~~P~~~~~~~~~~--~D~~~~~~~~~~~~~~~~~~~~~~---------~~~~~~~~~~~~~l~~~~K   222 (405) |
|  | T PF09913.10 | 160 | ------ALFTAVALMPMTLQQAGGIS--ADLVTIAFSFVGFSLVLHSREHFV---------SRRLLILIVLVFVMWVLCK   222 (405) |
|  | T ss\_pred |  | ------HHHHHHHhchHHHHHHhcCC--hHHHHHHHHHHHHHHHHhcCCcCC---------CHHHHHHHHHHHHHHHHHH |
|  |
|  |
|  | Q ss\_pred |  | HHHHHHHHHHHHHHHHhcccccchhHhhccCcchHHHhhHHHHHHHHHHHHHHHHHHHHHHHHhcCCCCccccCCCCccc |
|  | Q XP\_016875493.1 | 220 | ETGITVFGVCLVYDLFSLSNKQDKSYLRASSNRNFLLTMRPFLKRAILVLSYVLVILYFRLWIMGGSMPLFSEQDNPASF   299 (456) |
|  | Q Consensus | 220 | ~~~~~~~~~~~~~~~~~~~~~~~~~~~~~~~~~~~~~~~~~~~~~~~~~~~~~~~~~~~~~~~~~~~~~~~~~~~~~~~~   299 (456) |
|  |  |  | ..++++.+...+..+++++++. +......+..++...+..................... |
|  | T Consensus | 223 | ---~~~~~~~l~~~~~~~~~~~~~~------------------~~~~~~~~~~~~~~~~~~~~~~~~~~~~~~~~~~~~~   281 (405) |
|  | T PF09913.10 | 223 | ---SSIWALPLLLLIPVSAFKNRLT------------------WAAYLGVASVCMVGALLVWNNVTAPNLETFRAVRLTH   281 (405) |
|  | T ss\_pred |  | ---HHHHHHHHHHHHhHHhhCchHH------------------HHHHHHHHHHHHHHHHHHHHhhcCCCccchhhccccC |
|  |
|  |
|  | Q ss\_pred |  | chhHHHHHHHHHHHHHHHHHHHHHhHhhccccccCCCccccccccHHHHHHHHHHHHHHHHHHHHHHHHhcccc------ |
|  | Q XP\_016875493.1 | 300 | SPYILTRFLTYSYLLAFNVWLLLAPVTLCYDWQVGSIPLVETIWDMRNLATIFLAVVMALLSLHCLAAFKRLEH------   373 (456) |
|  | Q Consensus | 300 | ~~~~~~~~~~~~~~~~~~~~~~~~~~~~~~~~~~~~~~~~~~~~~~~~~~~~~~~~~~~~~~~~~~~~~~~~~~------   373 (456) |
|  |  |  | ..+...+.....++...+........................+.+........................+++++ |
|  | T Consensus | 282 | ~~~~~~~~~~~~~~p~~~~~~~~~~~~~~~~~~~~~~~g~~g~~~~~lp~~~~~~~~~~l~~~~~~~~~~~~~~~~~~~~   361 (405) |
|  | T PF09913.10 | 282 | GVDMPANIRLVGAHPLMFVRYLIGVVGSNLKPEIGQFIGAFGWLRFPLPSWVRAAYLLLVLVTAVTEFPAKSFRTWERGV   361 (405) |
|  | T ss\_pred |  | CCChHHHHHHHHhCHHHHHHHHHHHHHHhhHHHHHHHHHHhhccCCCccHHHHHHHHHHHHHHHHccCCccCCCHHHHHH |
|  |
|  |
|  | Q ss\_pred |  | chHHHHHHHHHHHHHhHhccccCCCcc-----------cccccc |
|  | Q XP\_016875493.1 | 374 | KEVLVGLLFLVFPFIPASNLFFRVGFV-----------VAERVL   406 (456) |
|  | Q Consensus | 374 | ~~~~~~~~~~~~~~~~~~~~~~~~~~~-----------~~~Ry~   406 (456) |
|  |  |  | ..........++............+.. .+.||+ |
|  | T Consensus | 362 | ~~~~~~~~~~~i~~~~~~~~~~~~~~~i~G~~~~~~~~~QgRY~   405 (405) |
|  | T PF09913.10 | 362 | LALVLLGGVLFVHAAMCISDTTLCSGTLNSGCRDESIVFQGRYL   405 (405) |
|  | T ss\_pred |  | HHHHHHHHHHHHHHHHHHhccCCCcceeccCCCCccceeceecC |
|  |
| --- | | | |
|  | Template alignmentCDD | | |
| 13. | PF04602.13 ; Arabinose\_trans ; Mycobacterial cell wall arabinan synthesis protein | | |
|  | Probability: 99.47%, E-value: 6.3e-11, Score: 102.85, Aligned cols: 352, Identities: 9%, Similarity: -0.033, | | |
|  |
|  | Q ss\_pred |  | HHHHHHHHhhhcCCCccccchHHHHhCCCCCCCCC---cccccccccccccCCCCCCCcccccchHHHHHHHHHHHhCCC |
|  | Q XP\_016875493.1 | 34 | AGASCLCYGRSLQGEFVHDDVWAIVNNPDVRPGAP---LRWGIFTNDFWGKGMAENTSHKSYRPLCVLTFKLNIFLTGMN   110 (456) |
|  | Q Consensus | 34 | ~~~~~~~~~~~~~~~~~~De~~~~~~~~~~~~~~~---~~~~~~~~~~~~~~~~~~~~~~~~~Pl~~~~~~~~~~l~g~~   110 (456) |
|  |  |  | ..+...+.......+...||.+|...+++..+++. ++..+..+. .|..+.+.......-+|.+ |
|  | T Consensus | 51 | ~~v~~~l~~w~~~gp~~~DEG~Yl~~ar~~~~~G~~~npy~~~~~~~--------------~Pfg~~~~l~~~~~~~g~s   116 (471) |
|  | T PF04602.13 | 51 | LIVGAILLGWYFIGANTADDGYILNMARVAGHAGYMANYYRWYGVPE--------------APFGWFYDVTAALAALSTA   116 (471) |
|  | T ss\_pred |  | HHHHHHHHHHHHhccCCCChHHHHHHHHHHHhcCCccccchhcCCCC--------------CCcHHHHHHHHHHHHhcCC |
|  |
|  |
|  | Q ss\_pred |  | chHHHHHHHHHHHHHHHHHHHHHHHHhc---cchHHHHHHHHHHHHccccHHHHHHHHhHHHHHHHHHHHHHHHHHHHHH |
|  | Q XP\_016875493.1 | 111 | PFYFHAVNIILHCLVTLVLMYTCDKTVF---KNRGLAFVTALLFAVHPIHTEAVAGIVGRADVLACLLFLLAFLSYNRSL   187 (456) |
|  | Q Consensus | 111 | ~~~~rl~~~l~~~~~~~~~~~l~~~~~~---~~~~~a~~aa~l~a~~p~~~~~~~~~~~~~~~~~~~~~~l~~~~~~~~~   187 (456) |
|  |  |  | ....|+++.+++.++.++++....+... .+++.+..++.+..+.....+. ...+.|+...++.++++++..|.. |
|  | T Consensus | 117 | ~~~lRl~~ll~~l~~w~lL~~~vl~rl~~~~~~~~~a~~~aal~~la~wlp~~---~~lr~Ep~~al~~~~~l~l~~ra~   193 (471) |
|  | T PF04602.13 | 117 | SPFVRLTTLIASILCWWIISREVIPRLGRRARHTPAVYWTAAAVFLAFWLPYN---NGLRPEPVIAVGALLTWISVERAI   193 (471) |
|  | T ss\_pred |  | hHHHHHHHHHHHHHHHHHHHHHHHHHhccccCCcHHHHHHHHHHHHHHHcccc---CCCCcHHHHHHHHHHHHHHHHHHH |
|  |
|  |
|  | Q ss\_pred |  | hcCCCCCCCCcchhHHHHHHHHHHHHHHHHHHHHHHHHHHHHHHHHHHhcccccchhHhhccCcchHHHhhHHHHHHH-- |
|  | Q XP\_016875493.1 | 188 | DQGCVGGSFPSTVSPFFLLLSLFLGTCAMLVKETGITVFGVCLVYDLFSLSNKQDKSYLRASSNRNFLLTMRPFLKRA--   265 (456) |
|  | Q Consensus | 188 | ~~~~~~~~~~~~~~~~~~~~~~~~~~la~~~k~~~~~~~~~~~~~~~~~~~~~~~~~~~~~~~~~~~~~~~~~~~~~~--   265 (456) |
|  |  |  | +++ +.....+++++.++++.+|+++......+.+......++.+++. +.. |
|  | T Consensus | 194 | ~~~----------~~~~~alag~~~gla~~aKPtg~~~la~ll~~~~~~~r~~~~r~------------------~~~~~   245 (471) |
|  | T PF04602.13 | 194 | ATG----------RLLPAAIATIIAAFSLAAGPTGLMAVAALLAGSRPLLAILIKRA------------------KQLTP   245 (471) |
|  | T ss\_pred |  | hcC----------CcHHHHHHHHHHHHHHhcCHhHHHHHHHHHHHHHHHHHHHHHHH------------------HhcCC |
|  |
|  |
|  | Q ss\_pred |  | ---------------HHHHHHHHHHHHHHHHHhcCCCCccccCCCCcccchhHHHHHHHHHHHHHHHHHHHHHhHhhccc |
|  | Q XP\_016875493.1 | 266 | ---------------ILVLSYVLVILYFRLWIMGGSMPLFSEQDNPASFSPYILTRFLTYSYLLAFNVWLLLAPVTLCYD   330 (456) |
|  | Q Consensus | 266 | ---------------~~~~~~~~~~~~~~~~~~~~~~~~~~~~~~~~~~~~~~~~~~~~~~~~~~~~~~~~~~~~~~~~~   330 (456) |
|  |  |  | ....+...........+..+......+................+..+....... |
|  | T Consensus | 246 | ~~~~~~~~~~~~~~~~la~~~a~~~~~l~~~f~d~sl~~~~~~~~~~~~~~~~~~~~~e~~Ry~~l~~~-----------   314 (471) |
|  | T PF04602.13 | 246 | NTTTGNKHTPLASGRPHRPLLAAGTAVLFIIFYDQTLAAVSEASRLRTIIGPSNSWYNEFFRYSELFSQ-----------   314 (471) |
|  | T ss\_pred |  | CCCCCCCCCCCCCCccHHHHHHHHHHHHHHHHhcCcHHHHHHHHHHHhhcCCCCHHhhhHHHHHHHhcC----------- |
|  |
|  |
|  | Q ss\_pred |  | cccCCCccccccccHHHHHHHHHHHHHHHHHHHHHHHHhcccc---chHHHHHHHHHHHHHhHhccccCCCccccccccc |
|  | Q XP\_016875493.1 | 331 | WQVGSIPLVETIWDMRNLATIFLAVVMALLSLHCLAAFKRLEH---KEVLVGLLFLVFPFIPASNLFFRVGFVVAERVLY   407 (456) |
|  | Q Consensus | 331 | ~~~~~~~~~~~~~~~~~~~~~~~~~~~~~~~~~~~~~~~~~~~---~~~~~~~~~~~~~~~~~~~~~~~~~~~~~~Ry~~   407 (456) |
|  |  |  | +...+...-......+..+.........++|.+. ......+.+....+....+ +.....+|+- |
|  | T Consensus | 315 | ---------~~~g~~~rr~~vll~~~~l~~~~~~l~r~~r~~~~~~~p~~rl~~~~~~~~~~l~~-----tptKwthhfg   380 (471) |
|  | T PF04602.13 | 315 | ---------TADGSIARRFPVLIMIVCIFTAAAAIIHSASKSKLAKGPTLRLLAVSIMSFGFLAA-----TPTKWVHHFG   380 (471) |
|  | T ss\_pred |  | ---------CCCCcHHHHHHHHHHHHHHHHHHHHHHHHhccccccCChHHHHHHHHHHHHHHHHh-----CCchhhhhHH |
|  |
|  |
|  | Q ss\_pred |  | hHHHHHHHHHHHHHHHHHHHhchhhHHHHHHHHHHHHHHHHHHHHHhcC |
|  | Q XP\_016875493.1 | 408 | MPSMGYCILFVHGLSKLCTWLNRCGATTLIVSTVLLLLLFSWKTVKQNE   456 (456) |
|  | Q Consensus | 408 | ~~~~~~~ll~~~~~~~~~~~~~~~~~~~~~~~~~~~~~~~~~~~~~~~~   456 (456) |
|  |  |  | ...+....+++.......+...+..+ ........+...........|+ |
|  | T Consensus | 381 | ~~a~~~~~~~a~~~~~~~~~~~r~~~-~~~~~~~~~~~~~al~~~g~N~   428 (471) |
|  | T PF04602.13 | 381 | AFAGIGAAIAALAAVALTTPLFQSPR-NRVLFTGIVVIIAAYAATGPNA   428 (471) |
|  | T ss\_pred |  | HHHHHHHHHHHHHHHHhcchhcCCHH-HHHHHHHHHHHHHHHHHhCccc |
|  |
| --- | | | |
|  | Template alignmentCDD | | |
| 14. | PF06728.14 ; PIG-U ; GPI transamidase subunit PIG-U | | |
|  | Probability: 99.44%, E-value: 9.8e-11, Score: 101.42, Aligned cols: 306, Identities: 8%, Similarity: -0.058, | | |
|  |
|  | Q ss\_pred |  | CCccccchHHHHhCCCCCCCCCcccccccccccccCCCCCCCcccccchHHHHHHHHHHHhCCCchHHHHHHHHHHHHHH |
|  | Q XP\_016875493.1 | 47 | GEFVHDDVWAIVNNPDVRPGAPLRWGIFTNDFWGKGMAENTSHKSYRPLCVLTFKLNIFLTGMNPFYFHAVNIILHCLVT   126 (456) |
|  | Q Consensus | 47 | ~~~~~De~~~~~~~~~~~~~~~~~~~~~~~~~~~~~~~~~~~~~~~~Pl~~~~~~~~~~l~g~~~~~~rl~~~l~~~~~~   126 (456) |
|  |  |  | .....|...+...++...++.+.+.+...+ +||+..++..... ++.+....|+++.++..+++ |
|  | T Consensus | 30 | ~~~~~d~~~~~~~~~~~~~G~~py~~~~~~---------------ypP~~~~l~~~~~--~~~~~~~~r~~~~~~~~~~~   92 (363) |
|  | T PF06728.14 | 30 | STPVSGFLRVREGLYLYENGLDPYSGGVFY---------------QSPLLLILNYCCE--LLGGISVTRFVYTSISTMGG   92 (363) |
|  | T ss\_pred |  | cCCcchHHHHHHHHHHHHcCCCCCCCCccc---------------CCcchHHHHhhhh--hcCchHHHHHHHHHHHHHHH |
|  |
|  |
|  | Q ss\_pred |  | HHHHHHHHHHhccch--------HHHHHHHHHHHHccccHHHHHHHHhHHHHHHHHHHHHHHHHHHHHHhcCCCCCCCCc |
|  | Q XP\_016875493.1 | 127 | LVLMYTCDKTVFKNR--------GLAFVTALLFAVHPIHTEAVAGIVGRADVLACLLFLLAFLSYNRSLDQGCVGGSFPS   198 (456) |
|  | Q Consensus | 127 | ~~~~~l~~~~~~~~~--------~~a~~aa~l~a~~p~~~~~~~~~~~~~~~~~~~~~~l~~~~~~~~~~~~~~~~~~~~   198 (456) |
|  |  |  | .++|.++|+.. ++ ..+..++++++++|..... ...++.|....++.+++++++.|.+ |
|  | T Consensus | 93 | ~l~~~~~~~~~--~~~~~~~~~~~~~~~~~~~~~~~p~~~~~--~~~~~~d~~~~~~~~~al~~~~~~~-----------   157 (363) |
|  | T PF06728.14 | 93 | LFVYLIAKQAR--VLDPNQVLSTCSPLWISVIYLLNPLTFLP--GIACSADMILNFTTLMTIYFASCGS-----------   157 (363) |
|  | T ss\_pred |  | HHHHHHHHHhh--hcCcccccccCCcHHHHHHHHhCHHHHHH--HHhcchHHHHHHHHHHHHHHHhCCC----------- |
|  |
|  |
|  | Q ss\_pred |  | chhHHHHHHHHHHHHHHHHHHHHHHHHHHHHHHHHHHhcccccchhHhhccCcchHHHhhHHHHHHHHHHHHHHHHHHHH |
|  | Q XP\_016875493.1 | 199 | TVSPFFLLLSLFLGTCAMLVKETGITVFGVCLVYDLFSLSNKQDKSYLRASSNRNFLLTMRPFLKRAILVLSYVLVILYF   278 (456) |
|  | Q Consensus | 199 | ~~~~~~~~~~~~~~~la~~~k~~~~~~~~~~~~~~~~~~~~~~~~~~~~~~~~~~~~~~~~~~~~~~~~~~~~~~~~~~~   278 (456) |
|  |  |  | ...++++.+++..+|...+...+...++....++.++.. +......+..+..... |
|  | T Consensus | 158 | ------~~~ag~~~gla~~~K~~~~~~~~~~~~~~~~~~~~~~~~-------------------~~~~~~~~~~~~~~~~   212 (363) |
|  | T PF06728.14 | 158 | ------YAIYACCMALTVFINPNALLLFFPSYLILRKCNSSIKFR-------------------QIFVVFLFYLAGLIIT   212 (363) |
|  | T ss\_pred |  | ------HHHHHHHHHHHHhcChHHHHHHHHHHHHHHHcCchHHHH-------------------HHHHHHHHHHHHHHHH |
|  |
|  |
|  | Q ss\_pred |  | HHHHhc------CCCCccccCCCCcccchhHHHHHHHHHHHHHHHHHHHHHhHhhccccccCCCccccccccHHHHHHHH |
|  | Q XP\_016875493.1 | 279 | RLWIMG------GSMPLFSEQDNPASFSPYILTRFLTYSYLLAFNVWLLLAPVTLCYDWQVGSIPLVETIWDMRNLATIF   352 (456) |
|  | Q Consensus | 279 | ~~~~~~------~~~~~~~~~~~~~~~~~~~~~~~~~~~~~~~~~~~~~~~~~~~~~~~~~~~~~~~~~~~~~~~~~~~~   352 (456) |
|  |  |  | .....+ +.................................. |
|  | T Consensus | 213 | ~~~~~~~~~~~~~~~~~~~~~~~~~~~~~~~~~~~~~~~~~~~~~~~---------------------------------   259 (363) |
|  | T PF06728.14 | 213 | SGFFLNSLSFLKIPFRVYLDSHDLTPNLGLWWYFFTEMFNEFRTFFL---------------------------------   259 (363) |
|  | T ss\_pred |  | HHHhhcCHHHHHHHHhhhccccccCCCHHHHHHHHHhHhHHHHHHHH--------------------------------- |
|  |
|  |
|  | Q ss\_pred |  | HHHHHHHHHHHHHHHHhccccchHHHHHHHHHHHHHhHhccccCCCccccccccchHHHHHHHHHHHHHHHHHHHhchhh |
|  | Q XP\_016875493.1 | 353 | LAVVMALLSLHCLAAFKRLEHKEVLVGLLFLVFPFIPASNLFFRVGFVVAERVLYMPSMGYCILFVHGLSKLCTWLNRCG   432 (456) |
|  | Q Consensus | 353 | ~~~~~~~~~~~~~~~~~~~~~~~~~~~~~~~~~~~~~~~~~~~~~~~~~~~Ry~~~~~~~~~ll~~~~~~~~~~~~~~~~   432 (456) |
|  |  |  | ....+...........+++++................-... .+|.....|.+.+... ..++.++.. |
|  | T Consensus | 260 | ~~~~~~~~~~~~~~~~~~~~~~~~~~~~~~~~~~~~~~~~~---------~~y~~~~~p~~~~~~~-----~~~~~~~~~   325 (363) |
|  | T PF06728.14 | 260 | FVFAILPLMFVLPVSIRLYYLPLPITIALIGLHSLFKAYPS---------ICDLSIFLSLLPIFNK-----VQDRMRYSL   325 (363) |
|  | T ss\_pred |  | HHHHHHHHHHHHHHHHHhccChHHHHHHHHHHHHHhCCCCc---------HHHHHHHHHHHHhcHH-----HHHHhhhHH |
|  |
|  |
|  | Q ss\_pred |  | HHHHHHHHHHHHHHHHHHHHHhcC |
|  | Q XP\_016875493.1 | 433 | ATTLIVSTVLLLLLFSWKTVKQNE   456 (456) |
|  | Q Consensus | 433 | ~~~~~~~~~~~~~~~~~~~~~~~~   456 (456) |
|  |  |  | ........................ |
|  | T Consensus | 326 | ~~~~~~~~~~~~~~~~~~~~~~~~   349 (363) |
|  | T PF06728.14 | 326 | LTNNAIVFALVLGSAFYHSWITLG   349 (363) |
|  | T ss\_pred |  | HHHHHHHHHHHHHHHHHHHHHhcC |
|  |
| --- | | | |
|  | Template alignmentCDD | | |
| 15. | PF15971.6 ; Mannosyl\_trans4 ; DolP-mannose mannosyltransferase | | |
|  | Probability: 99.31%, E-value: 9.4e-11, Score: 88.61, Aligned cols: 156, Identities: 16%, Similarity: 0.113, | | |
|  |
|  | Q ss\_pred |  | cCCCCCCCcccccchHHHHHHHHHHHhCCCchHHHHHHH----HHHHHHHHHHHHHHHHHhccch-HHHHHHHHHHHHcc |
|  | Q XP\_016875493.1 | 81 | KGMAENTSHKSYRPLCVLTFKLNIFLTGMNPFYFHAVNI----ILHCLVTLVLMYTCDKTVFKNR-GLAFVTALLFAVHP   155 (456) |
|  | Q Consensus | 81 | ~~~~~~~~~~~~~Pl~~~~~~~~~~l~g~~~~~~rl~~~----l~~~~~~~~~~~l~~~~~~~~~-~~a~~aa~l~a~~p   155 (456) |
|  |  |  | ......+....|||++.++......++|.++...|+.+. +++.+++..+|.+.|+.. ++ +.+..++.+++++| |
|  | T Consensus | 1 | G~~p~~~~~~~~ppl~~~~~~~~~~l~g~~~~~~~~~~~~~~~~~~~~~~~~~~~~~~~~~--~~~~~~~~~~~~~~~~p   78 (163) |
|  | T PF15971.6 | 1 | GGRLYVDAWEPKLPLSYETTGVLALLSGGDMYRLHLLSVVLMSGAVCAIVALVVMLVYDIT--GDDIVAPLAGLSMFLLP   78 (163) |
|  | T ss\_pred |  | CCCccccccCCCCcHHHHHHHHHHHHhCCchHHHHHHHHHHHHHHHHHHHHHHHHHHHHHh--CCCCHHHHHHHHHHHch |
|  |
|  |
|  | Q ss\_pred |  | ccH-HHHHHHHhHHHHHHHHHHHHHHHHHHHHHhcCCCCCCCCcchhHHHHHHHHHHHHHHHHHHHHHHHHHHHHHHHHH |
|  | Q XP\_016875493.1 | 156 | IHT-EAVAGIVGRADVLACLLFLLAFLSYNRSLDQGCVGGSFPSTVSPFFLLLSLFLGTCAMLVKETGITVFGVCLVYDL   234 (456) |
|  | Q Consensus | 156 | ~~~-~~~~~~~~~~~~~~~~~~~l~~~~~~~~~~~~~~~~~~~~~~~~~~~~~~~~~~~la~~~k~~~~~~~~~~~~~~~   234 (456) |
|  |  |  | ... .. ....++|....++.+++++...+ ++ ...++++.+++.++|+.+....+....+.. |
|  | T Consensus | 79 | ~~~~~~--~~~~~~~~~~~~~~~~~~~~~~~---~~--------------~~~~~~~~~l~~~~k~~~~~~~~~~~~~~~   139 (163) |
|  | T PF15971.6 | 79 | GFAVRP--AYGFKAKYLLVLCGLLAIYLYTR---GY--------------PALSGVAAAASVGYWQAGAIFPLIVVGLAV   139 (163) |
|  | T ss\_pred |  | HHhccc--cccccchHHHHHHHHHHHHHHHc---Cc--------------HHHHHHHHHHHHHhhHHHhHHHHHHHHHHH |
|  |
|  |
|  | Q ss\_pred |  | HhcccccchhHhhccCcchHHHhhHHHHHHHHHHHHHHHHHH |
|  | Q XP\_016875493.1 | 235 | FSLSNKQDKSYLRASSNRNFLLTMRPFLKRAILVLSYVLVIL   276 (456) |
|  | Q Consensus | 235 | ~~~~~~~~~~~~~~~~~~~~~~~~~~~~~~~~~~~~~~~~~~   276 (456) |
|  |  |  | ..+++++.. +.........+... |
|  | T Consensus | 140 | ~~~~~~~~~-------------------~~~~~~~~~~~~~~   162 (163) |
|  | T PF15971.6 | 140 | QRRDMRALE-------------------RVVAGGLGFTIVML   162 (163) |
|  | T ss\_pred |  | cccchHHHH-------------------HHHHHHHHHHHHHh |
|  |
| --- | | | |
|  | Template alignmentCDD | | |
| 16. | PF09586.11 ; YfhO ; Bacterial membrane protein YfhO | | |
|  | Probability: 99.14%, E-value: 3.9e-8, Score: 94.94, Aligned cols: 363, Identities: 11%, Similarity: -0.039, | | |
|  |
|  | Q ss\_pred |  | HHHHHHHHHHHHHHHhhhcCCCccc--cchHHH-----HhCCCCC-CCCCcccccccccccccCCCCCCCcccccchHHH |
|  | Q XP\_016875493.1 | 27 | AGAAALLAGASCLCYGRSLQGEFVH--DDVWAI-----VNNPDVR-PGAPLRWGIFTNDFWGKGMAENTSHKSYRPLCVL   98 (456) |
|  | Q Consensus | 27 | ~~~~~~l~~~~~~~~~~~~~~~~~~--De~~~~-----~~~~~~~-~~~~~~~~~~~~~~~~~~~~~~~~~~~~~Pl~~~   98 (456) |
|  |  |  | .+.++++++..........+..... |+..+. ...+.+. ++....++..... ..+..++..++ |
|  | T Consensus | 3 | ~~~~~l~~~~~~~~~~~~~~~~~~~~~D~~~~~~p~~~~~~~~~~~~~~~~~~~~~~~~----------G~~~~~~~~~~   72 (832) |
|  | T PF09586.11 | 3 | LLPFAIIFIYGLSRHVFPFGGQTIMTVDLGQQYIDFFAYFRTTLLQHPDTFFYSFAKGL----------GGDMLGVWAYY   72 (832) |
|  | T ss\_pred |  | HHHHHHHHHHHHHcCCCCCCCCcceeechhHhHHHHHHHHHHHHhhCCccceeccccCC----------CCccHHHHHHH |
|  |
|  |
|  | Q ss\_pred |  | HHHHHHHHhC-----CCchHHHHHHHHHHHHHHHHHHHHHHHHhc-cchHHHHHHHHHHHHccccHHHHHHHHhHHHHHH |
|  | Q XP\_016875493.1 | 99 | TFKLNIFLTG-----MNPFYFHAVNIILHCLVTLVLMYTCDKTVF-KNRGLAFVTALLFAVHPIHTEAVAGIVGRADVLA   172 (456) |
|  | Q Consensus | 99 | ~~~~~~~l~g-----~~~~~~rl~~~l~~~~~~~~~~~l~~~~~~-~~~~~a~~aa~l~a~~p~~~~~~~~~~~~~~~~~   172 (456) |
|  |  |  | +..+...+.. ....+.++..++...++.+.+|.++ |... .++..|++++++++++|......... ..... |
|  | T Consensus | 73 | ~~~p~~~l~~~~~~~~~~~~~~~~~~l~~~l~~~~~y~l~-r~~~~~~~~~a~~~a~~y~~s~~~~~~~~~~---~~~~~   148 (832) |
|  | T PF09586.11 | 73 | LMSPFNLLVLLTPGKWLSFGVWLMVLLKYGFSGLSFAYYL-KKSRLLSGWWLPTLSLTYALSGFAIANQFNV---MWLDA   148 (832) |
|  | T ss\_pred |  | HhCcchhHHhhCCHHHHHHHHHHHHHHHHHHHHHHHHHHH-HHhccccccHHHHHHHHHHHHHHHHHHHhCh---hHHHH |
|  |
|  |
|  | Q ss\_pred |  | HHHHHHHHHHHHHHHhcCCCCCCCCcchhHHHHHHHHHHHHHHHHHHHHHHHHHHHHHHHHHHhcccccchhHhhccCcc |
|  | Q XP\_016875493.1 | 173 | CLLFLLAFLSYNRSLDQGCVGGSFPSTVSPFFLLLSLFLGTCAMLVKETGITVFGVCLVYDLFSLSNKQDKSYLRASSNR   252 (456) |
|  | Q Consensus | 173 | ~~~~~l~~~~~~~~~~~~~~~~~~~~~~~~~~~~~~~~~~~la~~~k~~~~~~~~~~~~~~~~~~~~~~~~~~~~~~~~~   252 (456) |
|  |  |  | ..++.+.++++.+..++++ .....+..++++......-.....+.....++......+++ + |
|  | T Consensus | 149 | ~~~lPl~l~~~~~~~~~~~---------~~~~~~~~~l~~~~~~~~~~~~~~~~~~~~l~~~~~~~~~~----------~   209 (832) |
|  | T PF09586.11 | 149 | MIWLPLVVLGIEQLFERQR---------FWLYPLSLAALLIINYYMGYMVCLFVVAYFFWASVHHFKTW----------R   209 (832) |
|  | T ss\_pred |  | HHHHHHHHHHHHHHHhcCC---------ccHHHHHHHHHHHHHHHHHHHHHHHHHHHHHHHHHhcchhH----------H |
|  |
|  |
|  | Q ss\_pred |  | hHHHhhHHHHHHHHHHHHHHHHHHHHHHHHhcCCCCccccCCCCcccchhHHHHHHHHHHHHHHHHHHHHHhHhhccccc |
|  | Q XP\_016875493.1 | 253 | NFLLTMRPFLKRAILVLSYVLVILYFRLWIMGGSMPLFSEQDNPASFSPYILTRFLTYSYLLAFNVWLLLAPVTLCYDWQ   332 (456) |
|  | Q Consensus | 253 | ~~~~~~~~~~~~~~~~~~~~~~~~~~~~~~~~~~~~~~~~~~~~~~~~~~~~~~~~~~~~~~~~~~~~~~~~~~~~~~~~   332 (456) |
|  |  |  | ...+.........+..++..+..+.-..........................+...... |
|  | T Consensus | 210 | ~~~~~~~~~~~~~~l~~~l~a~~llp~~~~~~~~~~~~~~~~~~~~~~~~~~~~~~~~~---------------------   268 (832) |
|  | T PF09586.11 | 210 | QTCLVYLKFAGGSILAGLLAAWLLLPTFFQLTQSKGQYTIQKIHWKIDYNPLKILSKLV---------------------   268 (832) |
|  | T ss\_pred |  | HHHHHHHHHHHHHHHHHHHHHHHHHHHHHHHhcCcCccCccccccccCCCHHHHHHhhc--------------------- |
|  |
|  |
|  | Q ss\_pred |  | cCCCccccccccHHHHHHHHHHHHHHHHHHHHHHHHhccccchHHHHHHHHHHHHHhHhcc-------ccCCCccccccc |
|  | Q XP\_016875493.1 | 333 | VGSIPLVETIWDMRNLATIFLAVVMALLSLHCLAAFKRLEHKEVLVGLLFLVFPFIPASNL-------FFRVGFVVAERV   405 (456) |
|  | Q Consensus | 333 | ~~~~~~~~~~~~~~~~~~~~~~~~~~~~~~~~~~~~~~~~~~~~~~~~~~~~~~~~~~~~~-------~~~~~~~~~~Ry   405 (456) |
|  |  |  | ....................+.++.+......++++++......+.+++..+..... ..........|+ |
|  | T Consensus | 269 | ----~~~~~~~~~~~~~~~~y~g~~~l~l~~~~~~~~~~~~~~~~~~~~~~~~l~~~~~~~~~~~~~~~~~~~~~~~~R~   344 (832) |
|  | T PF09586.11 | 269 | ----VGNFNFDQMPKGEPNIFVGSLILIGFITYFLTRKIPIKERLAALLVTGFLGLSLCFEPLDLLWHGMQFPVWYPYRF   344 (832) |
|  | T ss\_pred |  | ----cCCCCcccCCCCchHHHHHHHHHHHHHHHHHcCCCCHHHHHHHHHHHHHHHHHhcCHHHHHHHhcCCCCCCCcHHH |
|  |
|  |
|  | Q ss\_pred |  | cchHHHHHHHHHHHHHHHHHHHhchhhHHHHHHHHHHHHHHH |
|  | Q XP\_016875493.1 | 406 | LYMPSMGYCILFVHGLSKLCTWLNRCGATTLIVSTVLLLLLF   447 (456) |
|  | Q Consensus | 406 | ~~~~~~~~~ll~~~~~~~~~~~~~~~~~~~~~~~~~~~~~~~   447 (456) |
|  |  |  | .....+.++++++.++.+..+..++.........++++.... |
|  | T Consensus | 345 | ~~~~~~~~~ila~~~l~~~~~~~~~~~~~~~~~~~~~~~~~~   386 (832) |
|  | T PF09586.11 | 345 | SYVISFWLIVLAVQRLHYQPQFKWYSLLAPLLLLAASLAYTF   386 (832) |
|  | T ss\_pred |  | HHHHHHHHHHHHHHHHhcCCcCChhHHHHHHHHHHHHHHHHH |
|  |
| --- | | | |
|  | Template alignmentCDD | | |
| 17. | PF04922.13 ; DIE2\_ALG10 ; DIE2/ALG10 family | | |
|  | Probability: 99.13%, E-value: 6.2e-9, Score: 89.68, Aligned cols: 209, Identities: 15%, Similarity: 0.098, | | |
|  |
|  | Q ss\_pred |  | CCccccchHHHHhCCCCCCCCCcccccccccccccCCCCCCCcccccchHHHHHHHHHHHhC---------CCchHHHHH |
|  | Q XP\_016875493.1 | 47 | GEFVHDDVWAIVNNPDVRPGAPLRWGIFTNDFWGKGMAENTSHKSYRPLCVLTFKLNIFLTG---------MNPFYFHAV   117 (456) |
|  | Q Consensus | 47 | ~~~~~De~~~~~~~~~~~~~~~~~~~~~~~~~~~~~~~~~~~~~~~~Pl~~~~~~~~~~l~g---------~~~~~~rl~   117 (456) |
|  |  |  | ...+.||..|..+++.+.+++...++...+ .||+++++.+...+++| .+....|+. |
|  | T Consensus | 2 | p~py~DE~fH~~qa~~y~~G~~~~wdp~iT---------------TpPGlyl~~a~~~~l~g~~~~~~~~~~s~~~LR~~   66 (434) |
|  | T PF04922.13 | 2 | PTPYIDEIFHIPQTQQYCKGHWNAWDSKIT---------------TPPGLYIIGYAWARMLTLTGLSESEACSTLSLRAV   66 (434) |
|  | T ss\_pred |  | CCCCcchHhhHHHHHHHHcCCccccCccCC---------------CChhHHHHHHHHHHHHHHhcCCcccCCCHHHHHHH |
|  |
|  |
|  | Q ss\_pred |  | HHHHHH-HHHHHHHHHHHHHhccchHHHHHHHHHHHHccccHHHHHHHHhHHHHHHHHHHHHHHHHHHHHHhcCCCCCCC |
|  | Q XP\_016875493.1 | 118 | NIILHC-LVTLVLMYTCDKTVFKNRGLAFVTALLFAVHPIHTEAVAGIVGRADVLACLLFLLAFLSYNRSLDQGCVGGSF   196 (456) |
|  | Q Consensus | 118 | ~~l~~~-~~~~~~~~l~~~~~~~~~~~a~~aa~l~a~~p~~~~~~~~~~~~~~~~~~~~~~l~~~~~~~~~~~~~~~~~~   196 (456) |
|  |  |  | +++++. +....+|.+. +.. ++..+...++.++++|..... +....+|...++++++++++..+..+++ |
|  | T Consensus | 67 | nll~~~~~~~~~~~~l~-~~~--~~~~a~l~al~l~~~Pl~~~~--sfl~YTDv~Sl~~vll~l~~~l~~~~~~------   135 (434) |
|  | T PF04922.13 | 67 | NLMAVVIYIPATLYIIQ-RRV--WGSQAHFSAFSLVSFPLIWFY--AALYYTDVWSTATVLMALAFALSPRVPF------   135 (434) |
|  | T ss\_pred |  | HHHHHHHHHHHHHHHHH-HHh--cCchHHHHHHHHHhcHHHHHH--HHhhchHHHHHHHHHHHHHHHhCCCCCc------ |
|  |
|  |
|  | Q ss\_pred |  | CcchhHHHHHHHHHHHHHHHHHHHHHHHHHHHHHHHHHHh---cccccchhHhhccCcchHHHhhHHHHH--------HH |
|  | Q XP\_016875493.1 | 197 | PSTVSPFFLLLSLFLGTCAMLVKETGITVFGVCLVYDLFS---LSNKQDKSYLRASSNRNFLLTMRPFLK--------RA   265 (456) |
|  | Q Consensus | 197 | ~~~~~~~~~~~~~~~~~la~~~k~~~~~~~~~~~~~~~~~---~~~~~~~~~~~~~~~~~~~~~~~~~~~--------~~   265 (456) |
|  |  |  | ... .+++++.++|+++|++.++.........+.. ..+++.+ ++..+....... .. |
|  | T Consensus | 136 | ----~~~--~la~l~~~lavl~RQtnIvW~~f~~~~~~~~~~~~~~~~~~--------~~~~~~l~~~~~~~~~~~~~~~   201 (434) |
|  | T PF04922.13 | 136 | ----YMV--QLSALMCAVSLFFRQTNILWAAVVAVIAIENSHYSNGAPPK--------NGALAQIFSTISYTFQIELPIF   201 (434) |
|  | T ss\_pred |  | ----cHH--HHHHHHHHHHHHhchhHHHHHHHHHHHHHHHHhhhCCCCCc--------ccHHHHHHHHHHHHHhhchHHH |
|  |
|  |
|  | Q ss\_pred |  | HHHHHHHHHHHHHHHHHhcCCCCccccCCC |
|  | Q XP\_016875493.1 | 266 | ILVLSYVLVILYFRLWIMGGSMPLFSEQDN   295 (456) |
|  | Q Consensus | 266 | ~~~~~~~~~~~~~~~~~~~~~~~~~~~~~~   295 (456) |
|  |  |  | ......+++......+...+.+-..++.+. |
|  | T Consensus | 202 | ~~~~~~~~v~~~F~~Fv~~NGgIvlGDk~~   231 (434) |
|  | T PF04922.13 | 202 | NILISYASVAVGFSFFLYINGGIALGDKDN   231 (434) |
|  | T ss\_pred |  | HHHHHHHHHHHHHHHHHHhCCCcccCcccc |
|  |
| --- | | | |
|  | Template alignmentCDD | | |
| 18. | PF03155.16 ; Alg6\_Alg8 ; ALG6, ALG8 glycosyltransferase family | | |
|  | Probability: 99.06%, E-value: 3e-8, Score: 88.4, Aligned cols: 327, Identities: 13%, Similarity: -0.006, | | |
|  |
|  | Q ss\_pred |  | CCcc--ccchHHHHhCCCCCCCCCcccccccccccccCCCCCCCcccccchHHHHHHHHHHHh----------------- |
|  | Q XP\_016875493.1 | 47 | GEFV--HDDVWAIVNNPDVRPGAPLRWGIFTNDFWGKGMAENTSHKSYRPLCVLTFKLNIFLT-----------------   107 (456) |
|  | Q Consensus | 47 | ~~~~--~De~~~~~~~~~~~~~~~~~~~~~~~~~~~~~~~~~~~~~~~~Pl~~~~~~~~~~l~-----------------   107 (456) |
|  |  |  | .+.+ .|...+........+.....+...+.++|..+ |||+..+...+...+. |
|  | T Consensus | 9 | ~p~~~s~D~~~~r~w~~~t~~~p~~~wy~~~~~~w~ld---------YPPl~a~~~~~~~~~~~~~~~~~~~l~~~~~~~   79 (470) |
|  | T PF03155.16 | 9 | IPAYHSTDFEVHRNWLAITHSLPLNQWYVDATSEWTLD---------YPPFFAYFEWLLSQVAKYVDPRMLVVDNLNYES   79 (470) |
|  | T ss\_pred |  | ccccCCCcHHHHHHHHHHHhhCCHHHhccCCCccCCCC---------CcHHHHHHHHHHHHHHHHhCHHHhhcccCCCCC |
|  |
|  |
|  | Q ss\_pred |  | CCCchHHHHHHHHHHHHHHHHHHHHHH---HHhccchHHHHHHHHHHHHccccHHHHHHH-HhHHHHHHHHHHHHHHHHH |
|  | Q XP\_016875493.1 | 108 | GMNPFYFHAVNIILHCLVTLVLMYTCD---KTVFKNRGLAFVTALLFAVHPIHTEAVAGI-VGRADVLACLLFLLAFLSY   183 (456) |
|  | Q Consensus | 108 | g~~~~~~rl~~~l~~~~~~~~~~~l~~---~~~~~~~~~a~~aa~l~a~~p~~~~~~~~~-~~~~~~~~~~~~~l~~~~~   183 (456) |
|  |  |  | +......|+..++...+....++...+ +.. +++.+..++++++++|..+.. .. .++.|.....+.+++++++ |
|  | T Consensus | 80 | ~~~~~~~R~~vi~~d~l~~~~v~~~~~~~~~~~--~~~~~~~~~~l~l~~P~li~~--d~~~~q~n~~~~~l~llsl~~~   155 (470) |
|  | T PF03155.16 | 80 | KATVYFQRLSVIATDLVYVLGVRSCLGSLGLAR--DTQQFFAGSMLLLLNVGLLFV--DHIHFQYNGLLFGILLLSIGSL   155 (470) |
|  | T ss\_pred |  | HHHHHHHHHHHHHHHHHHHHHHHHHHHhcCCCC--ChhHHHHHHHHHHHcHHHHHh--hcccccchHHHHHHHHHHHHHH |
|  |
|  |
|  | Q ss\_pred |  | HHHHhcCCCCCCCCcchhHHHHHHHHHHHHHHHHHHHHHHHHHHHHHHHHHHhcccccchhHhhccCcchHHHhhHHHHH |
|  | Q XP\_016875493.1 | 184 | NRSLDQGCVGGSFPSTVSPFFLLLSLFLGTCAMLVKETGITVFGVCLVYDLFSLSNKQDKSYLRASSNRNFLLTMRPFLK   263 (456) |
|  | Q Consensus | 184 | ~~~~~~~~~~~~~~~~~~~~~~~~~~~~~~la~~~k~~~~~~~~~~~~~~~~~~~~~~~~~~~~~~~~~~~~~~~~~~~~   263 (456) |
|  |  |  | .+.+ ...+++++++++.+|...+.+.++..++.+...+.++ +...+..++..+ |
|  | T Consensus | 156 | ~~~~-----------------~~~a~~~~~lal~~K~~~l~~~p~~~~~ll~~~~~~~----------~~~~~~~~~~~~   208 (470) |
|  | T PF03155.16 | 156 | IRQR-----------------FLWSAFAFAVLLNFKHIFLYMAPAFGVYLLRFYCLEQ----------ASVASAVGAVIK   208 (470) |
|  | T ss\_pred |  | HcCc-----------------hHHHHHHHHHHHHcchHHHHHHHHHHHHHHHHhcccC----------CCHhHHHHHHHH |
|  |
|  |
|  | Q ss\_pred |  | HHHHHHHHHHHHHHHHHHHhcCCCCccccCCCCcccchhHHHHHHHHHHHHHHHHHHHHHhHhhccccccCCCccccccc |
|  | Q XP\_016875493.1 | 264 | RAILVLSYVLVILYFRLWIMGGSMPLFSEQDNPASFSPYILTRFLTYSYLLAFNVWLLLAPVTLCYDWQVGSIPLVETIW   343 (456) |
|  | Q Consensus | 264 | ~~~~~~~~~~~~~~~~~~~~~~~~~~~~~~~~~~~~~~~~~~~~~~~~~~~~~~~~~~~~~~~~~~~~~~~~~~~~~~~~   343 (456) |
|  |  |  | .....+...++..... .........................+.................+.........+..+...... |
|  | T Consensus | 209 | ~~~~~~~~~~~~~~Pf-~~~~~~~~~rlfp~~rgl~~~~~a~n~w~~~~~~~~~~~~~~~~~~~~~~~t~g~~~~~~~~~   287 (470) |
|  | T PF03155.16 | 209 | LLVVGLTPFAVSFGPF-WKQLPQVLSRLFPFKRGLTHAYWAPNFWALYNTADKVAAGVLKVHDGGASTTSGLVQEVRHSV   287 (470) |
|  | T ss\_pred |  | HHHHHHHHHHHhcccc-cccHHHHHHHHCCccccccccchhhHHHHHHHHHHHHHHHHHhcCCCCccccCCccccchhhc |
|  |
|  |
|  | Q ss\_pred |  | cHHHHHHHHHHHHHHHHHHHHHHHHhccccchHHHHHHHHHHHHHhHhccccCCCccccccccchHHHHHHHHHH |
|  | Q XP\_016875493.1 | 344 | DMRNLATIFLAVVMALLSLHCLAAFKRLEHKEVLVGLLFLVFPFIPASNLFFRVGFVVAERVLYMPSMGYCILFV   418 (456) |
|  | Q Consensus | 344 | ~~~~~~~~~~~~~~~~~~~~~~~~~~~~~~~~~~~~~~~~~~~~~~~~~~~~~~~~~~~~Ry~~~~~~~~~ll~~   418 (456) |
|  |  |  | .............++..........++++....................+ +...+++|.+...+++++++. |
|  | T Consensus | 288 | ~~~~~~~~~~~~~l~~~~~~~~~l~~~~~~~~~~~~~~~~~~~~l~~flf----~~~vhek~ill~l~Pl~ll~~   358 (470) |
|  | T PF03155.16 | 288 | LPAITPPVTFALTALFMLPILVKLFRSPKKQSPLVFLRAVVLCGCSSFVF----GWHVHEKAILMVLLPLCLLTL   358 (470) |
|  | T ss\_pred |  | CCccHHHHHHHHHHHHHHHHHHHHHcCCCCCCHHHHHHHHHHHHHHHHHh----chhhcchHHHHHHHHHHHHHH |
|  |
| --- | | | |
|  | Template alignmentCDD | | |
| 19. | PF14264.7 ; Glucos\_trans\_II ; Glucosyl transferase GtrII | | |
|  | Probability: 98.99%, E-value: 6.4e-7, Score: 74.96, Aligned cols: 302, Identities: 13%, Similarity: 0.068, | | |
|  |
|  | Q ss\_pred |  | cCCCccccchHHHHhCCCCCCCCCcccccccccccccCCCCCCCcccccchHHHHHHHHHHHhCCCchHHHHHHHHHHHH |
|  | Q XP\_016875493.1 | 45 | LQGEFVHDDVWAIVNNPDVRPGAPLRWGIFTNDFWGKGMAENTSHKSYRPLCVLTFKLNIFLTGMNPFYFHAVNIILHCL   124 (456) |
|  | Q Consensus | 45 | ~~~~~~~De~~~~~~~~~~~~~~~~~~~~~~~~~~~~~~~~~~~~~~~~Pl~~~~~~~~~~l~g~~~~~~rl~~~l~~~~   124 (456) |
|  |  |  | .+..+..||.......+...+. +..++ +|+...+... ....+. ++..++.+.++..+ |
|  | T Consensus | 5 | ~~~~~~~Dd~~~~~~~~~~~~~------~~~~G---------------R~~~~~l~~~-~~~~~~-p~~~~~l~~~~~~~   61 (312) |
|  | T PF14264.7 | 5 | FHSSFSHDSLNALYSDMTEIKW------KLALG---------------RFVVPLIMKI-RGQIAL-PWLIGIVSLFLIAA   61 (312) |
|  | T ss\_pred |  | cccCCCccchhHhhcCchhhhH------HHhcc---------------chhHHHHHHH-ccccch-hHHHHHHHHHHHHH |
|  |
|  |
|  | Q ss\_pred |  | HHHHHHHHHHHHhccchHHHHHHHHHHHHccccHH-HHHHH-HhHHHHHHHHHHHHHHHHHHHHHhcCCCCCCCCcchhH |
|  | Q XP\_016875493.1 | 125 | VTLVLMYTCDKTVFKNRGLAFVTALLFAVHPIHTE-AVAGI-VGRADVLACLLFLLAFLSYNRSLDQGCVGGSFPSTVSP   202 (456) |
|  | Q Consensus | 125 | ~~~~~~~l~~~~~~~~~~~a~~aa~l~a~~p~~~~-~~~~~-~~~~~~~~~~~~~l~~~~~~~~~~~~~~~~~~~~~~~~   202 (456) |
|  |  |  | ++.+.+... +.- ++..+.+++.++..+|.+.+ +..+. .........++..++.+... +++ ++ |
|  | T Consensus | 62 | s~~l~~~~~-~~~--~~~~~~~~~~l~~~~P~~~~~~~~f~~~~~~~~~~~ll~~la~~~~~---~~~----------~~   125 (312) |
|  | T PF14264.7 | 62 | SLYLILETI-QID--SKAMIILVSIFMVTNRTIYSMTATYIYELDYDMLALFFASLAAYILM---KKD----------KP   125 (312) |
|  | T ss\_pred |  | HHHHHHHHh-cCC--cHHHHHHHHHHHHHhHHHHHHHHHHHHhcHHHHHHHHHHHHHHHHHH---hCC----------CC |
|  |
|  |
|  | Q ss\_pred |  | HHHHHHHHHHHHHHHHHHHHHHHHHHHHHHHHHhcccccchhHhhccCcchHHHhhHHHHHHHHHHHHHHHHHHHHHHHH |
|  | Q XP\_016875493.1 | 203 | FFLLLSLFLGTCAMLVKETGITVFGVCLVYDLFSLSNKQDKSYLRASSNRNFLLTMRPFLKRAILVLSYVLVILYFRLWI   282 (456) |
|  | Q Consensus | 203 | ~~~~~~~~~~~la~~~k~~~~~~~~~~~~~~~~~~~~~~~~~~~~~~~~~~~~~~~~~~~~~~~~~~~~~~~~~~~~~~~   282 (456) |
|  |  |  | +....+.++..+++.+++......+...+........+.+++.++ ..+...+... .+...+.+....-.. |
|  | T Consensus | 126 | ~~~~~~~l~~~~sl~~YQ~~~~~~~~~~~~~~l~~~~~~~~~~k~---------~~~~~~~~~~-~~~~~i~y~i~~k~~   195 (312) |
|  | T PF14264.7 | 126 | GWYLLAFLSGVLSLGLYQSYIEVAFAIVIIASLKNLLEGSKYSQV---------LKRGIIAIVS-FVLSVVAYYLIYKLS   195 (312) |
|  | T ss\_pred |  | chHHHHHHHHHHHHHHHHHHHHHHHHHHHHHHHHHHHcCCCcHHH---------HHHHHHHHHH-HHHHHHHHHHHHHHH |
|  |
|  |
|  | Q ss\_pred |  | hcCCCCccccCCCCcccchhH-HHHHHHHHHHHHHHHHHHHHhHhhccccccCCCccccccccHHHHHHHHHHHHHHHHH |
|  | Q XP\_016875493.1 | 283 | MGGSMPLFSEQDNPASFSPYI-LTRFLTYSYLLAFNVWLLLAPVTLCYDWQVGSIPLVETIWDMRNLATIFLAVVMALLS   361 (456) |
|  | Q Consensus | 283 | ~~~~~~~~~~~~~~~~~~~~~-~~~~~~~~~~~~~~~~~~~~~~~~~~~~~~~~~~~~~~~~~~~~~~~~~~~~~~~~~~   361 (456) |
|  |  |  | ..-.....+..++........ .++..+..++...+.. ......................+.... |
|  | T Consensus | 196 | ~~~~~~~~~~~~~~~~~~~~~~~~~i~~~~~~~~~~~~---------------~~~~~~~~~~~~~~~~~~~~~~~~~~~   260 (312) |
|  | T PF14264.7 | 196 | CKFFNVQIEGRTDAFSGEYTSIIVSLKVMLYKLIHDVV---------------KPGTIYELPIVGIADILLIAIGVALCL   260 (312) |
|  | T ss\_pred |  | HHHcCCCCCCCCCccccccccHHHHHHHHHHHHHHHhh---------------CCCCCCCCccHHHHHHHHHHHHHHHHH |
|  |
|  |
|  | Q ss\_pred |  | HHHHHHHhccccchHHHHHHHHHHHHHhHhccccC-CCccccccccchHHHHH |
|  | Q XP\_016875493.1 | 362 | LHCLAAFKRLEHKEVLVGLLFLVFPFIPASNLFFR-VGFVVAERVLYMPSMGY   413 (456) |
|  | Q Consensus | 362 | ~~~~~~~~~~~~~~~~~~~~~~~~~~~~~~~~~~~-~~~~~~~Ry~~~~~~~~   413 (456) |
|  |  |  | ......+++++.+. .++.......|+...... .+....+|-.++....+ |
|  | T Consensus | 261 | ~~~~~~~~~~~~~~---~l~~~~~~~~p~~~~~i~~~~~~~~~r~l~~~~~~~   310 (312) |
|  | T PF14264.7 | 261 | IMIFKLGKGKTGEK---VVSLLLLAALPLSLNLICLTIKSGSEHDLMTYSFNF   310 (312) |
|  | T ss\_pred |  | HHHHHHcCCCcHHH---HHHHHHHHHHHHHHHHHHHHCCCCCcchhhhhhhhe |
|  |
| --- | | | |
|  | Template alignmentCDD | | |
| 20. | PF05208.14 ; ALG3 ; ALG3 protein | | |
|  | Probability: 98.96%, E-value: 6.2e-8, Score: 82.27, Aligned cols: 203, Identities: 12%, Similarity: -0.031, | | |
|  |
|  | Q ss\_pred |  | HHHHHHHHHHHHhhhcCCCccccchHHHHhCCCCCCCCCcccccccccccccCCCCCCCcccccchHHHHHHHHHHHhCC |
|  | Q XP\_016875493.1 | 30 | AALLAGASCLCYGRSLQGEFVHDDVWAIVNNPDVRPGAPLRWGIFTNDFWGKGMAENTSHKSYRPLCVLTFKLNIFLTGM   109 (456) |
|  | Q Consensus | 30 | ~~~l~~~~~~~~~~~~~~~~~~De~~~~~~~~~~~~~~~~~~~~~~~~~~~~~~~~~~~~~~~~Pl~~~~~~~~~~l~g~   109 (456) |
|  |  |  | .+++.-+++.............|+..|...++.+.+|+..+.+...+. ....+||++.++....+.+.+. |
|  | T Consensus | 2 | ~~~~~~~~~~~~~i~~~~yt~iD~~~y~~~~~~i~~G~~pY~~~~~~~----------~p~~Ypp~~~yi~~~l~~l~~~   71 (356) |
|  | T PF05208.14 | 2 | LLVLVDAVLSALIIKKVSYTEIDWTTYMQQIALYQAGERDYTAIKGDT----------GPLVYPASHVYIYSFLYELTNK   71 (356) |
|  | T ss\_pred |  | HHHHHHHHHHHHHHHhCCCCCCcHHHHHHHHHHHHcCCCChhhccCCC----------CCCCCcHHHHHHHHHHHHHhcC |
|  |
|  |
|  | Q ss\_pred |  | --CchHHHHHHHHHHHHHHHHHHHHHHHHhccchHHHHHHHHHHHHccccHHHHHHHHhHHHHHHHHHHHHHHHHHHHHH |
|  | Q XP\_016875493.1 | 110 | --NPFYFHAVNIILHCLVTLVLMYTCDKTVFKNRGLAFVTALLFAVHPIHTEAVAGIVGRADVLACLLFLLAFLSYNRSL   187 (456) |
|  | Q Consensus | 110 | --~~~~~rl~~~l~~~~~~~~~~~l~~~~~~~~~~~a~~aa~l~a~~p~~~~~~~~~~~~~~~~~~~~~~l~~~~~~~~~   187 (456) |
|  |  |  | +....|.....+.+++..+++.+.++ ++..+..++.++..+|.+... ...+++|...+++.+++++++.+.+ |
|  | T Consensus | 72 | ~~~i~~~~~~f~~~~l~~~~li~~i~~~----~~~~~~~~~~l~l~~pl~s~~--~~~g~~D~i~~~~lll~l~~l~~~~   145 (356) |
|  | T PF05208.14 | 72 | GQDIELGQYIFAGIYIATLIVVLSCYIK----AGAPPYLLPLLVLSKRLHSIY--MLRLFNDGIATLAMWVAIFFFQRRQ   145 (356) |
|  | T ss\_pred |  | CCChHHHHHHHHHHHHHHHHHHHHHHHH----cCCChhHHHHHHHccHHHHHH--HHhhhcHHHHHHHHHHHHHHHHhCC |
|  |
|  |
|  | Q ss\_pred |  | hcCCCCCCCCcchhHHHHHHHHHHHHHHHHHHHHHHHHHHHHHHHHHHhcccccchhHhhccCcchHHHhhHHHHHHHHH |
|  | Q XP\_016875493.1 | 188 | DQGCVGGSFPSTVSPFFLLLSLFLGTCAMLVKETGITVFGVCLVYDLFSLSNKQDKSYLRASSNRNFLLTMRPFLKRAIL   267 (456) |
|  | Q Consensus | 188 | ~~~~~~~~~~~~~~~~~~~~~~~~~~la~~~k~~~~~~~~~~~~~~~~~~~~~~~~~~~~~~~~~~~~~~~~~~~~~~~~   267 (456) |
|  |  |  | ...++++.++|+.+|...++..+.+.++....++.++ ..... |
|  | T Consensus | 146 | -----------------~~la~i~~glAv~~K~~~ll~~P~ll~~l~~~~~~~~---------------------~~~~~   187 (356) |
|  | T PF05208.14 | 146 | -----------------LTVATTVWSLGVGVKMSLLLLAPGVAIVIALSGGIWA---------------------AVPLA   187 (356) |
|  | T ss\_pred |  | -----------------HHHHHHHHHHHHHHHHHHHHHHHHHHHHHHHcCCHHH---------------------HHHHH |
|  |
|  |
|  | Q ss\_pred |  | HHHHHHHHHHHHHHHhcCC |
|  | Q XP\_016875493.1 | 268 | VLSYVLVILYFRLWIMGGS   286 (456) |
|  | Q Consensus | 268 | ~~~~~~~~~~~~~~~~~~~   286 (456) |
|  |  |  | ....+........+...++ |
|  | T Consensus | 188 | ~~~~~~~~l~~lPfl~~~~   206 (356) |
|  | T PF05208.14 | 188 | LNAVLTQVLLGIPFLQENA   206 (356) |
|  | T ss\_pred |  | HHHHHHHHHHHHHHHhcCh |
|  |
| --- | | | |
|  | Template alignmentCDD | | |
| 21. | PF09594.11 ; GT87 ; Glycosyltransferase family 87 | | |
|  | Probability: 98.78%, E-value: 3.5e-7, Score: 74.06, Aligned cols: 240, Identities: 9%, Similarity: -0.085, | | |
|  |
|  | Q ss\_pred |  | cccchHHHHHHHHHHHhCC---CchHHHHHHHHHHHHHHHHHHHHHHHHhccch--HHHHHHHHHHHHccccHHHHHHHH |
|  | Q XP\_016875493.1 | 91 | SYRPLCVLTFKLNIFLTGM---NPFYFHAVNIILHCLVTLVLMYTCDKTVFKNR--GLAFVTALLFAVHPIHTEAVAGIV   165 (456) |
|  | Q Consensus | 91 | ~~~Pl~~~~~~~~~~l~g~---~~~~~rl~~~l~~~~~~~~~~~l~~~~~~~~~--~~a~~aa~l~a~~p~~~~~~~~~~   165 (456) |
|  |  |  | .|||...++..+. .+.|+ .....++...+........++.+. |...+++ ......+++++.+|. ... ... |
|  | T Consensus | 2 | ~YpP~~~~l~~~~-~l~~~~~~~~~~~~~~~~~~~~~~~~~~~~~~-r~~~~~~~~~~~~~~~~~~~~~p~-~~~--~~~   76 (251) |
|  | T PF09594.11 | 2 | TYPPFGALVFTPL-WWIHDLFGLLVTERVFALITLLTTYAVAVFLL-RLAGVRDRVWEFVAFAALLVSAPV-YFT--LNI   76 (251) |
|  | T ss\_pred |  | CCChHHHHHHHHH-hhccchhcHHHHHHHHHHHHHHHHHHHHHHHH-HHcCCCCcHHHHHHHHHHHHHHHH-HHH--hhc |
|  |
|  |
|  | Q ss\_pred |  | hHHHHHHHHHHHHHHHHHHHHHhcCCCCCCCCcchhHHHHHHHHHHHHHHHHHHHHHHHHHHHHHHHHHHhcccccchhH |
|  | Q XP\_016875493.1 | 166 | GRADVLACLLFLLAFLSYNRSLDQGCVGGSFPSTVSPFFLLLSLFLGTCAMLVKETGITVFGVCLVYDLFSLSNKQDKSY   245 (456) |
|  | Q Consensus | 166 | ~~~~~~~~~~~~l~~~~~~~~~~~~~~~~~~~~~~~~~~~~~~~~~~~la~~~k~~~~~~~~~~~~~~~~~~~~~~~~~~   245 (456) |
|  |  |  | ++.|.+..++.++++++.. .++++ +.++...++++++++..+|...+.+.+.+..+.+..++.+. |
|  | T Consensus | 77 | g~~~~~~~~~~~~~l~~~~-~~~~~----------~~~~~~~ag~~l~la~~~K~~~~~~~~~ll~~~~~~~~~r~----   141 (251) |
|  | T PF09594.11 | 77 | GQINVMLMALTLFDVALPR-STRHS----------GVLKYVPLGVLTGIAAAIKLTPLVFGLYFLILWVVTKSPRG----   141 (251) |
|  | T ss\_pred |  | CCHHHHHHHHHHHHHHhcc-ccCCC----------CccccHHHHHHHHHHHHhhHHHHHHHHHHHHHHHHcCCHHH---- |
|  |
|  |
|  | Q ss\_pred |  | hhccCcchHHHhhHHHHHHHHHHHHHHHHHHHHHHHHhcCCCCccccCCCCcccchhHHHHHHHHHHHHHHHHHHHHHhH |
|  | Q XP\_016875493.1 | 246 | LRASSNRNFLLTMRPFLKRAILVLSYVLVILYFRLWIMGGSMPLFSEQDNPASFSPYILTRFLTYSYLLAFNVWLLLAPV   325 (456) |
|  | Q Consensus | 246 | ~~~~~~~~~~~~~~~~~~~~~~~~~~~~~~~~~~~~~~~~~~~~~~~~~~~~~~~~~~~~~~~~~~~~~~~~~~~~~~~~   325 (456) |
|  |  |  | .................................. +......+.............. |
|  | T Consensus | 142 | -----------------~~~~~~~~~~~~~~~~~~~~~~~~~~~~~~~~~~-~~~~~~~~~~~~~l~~~~~~~~------   197 (251) |
|  | T PF09594.11 | 142 | -----------------LFGMIGGFLGASGLAIIFRPSISIQYFTDVLFTA-ERIGDLHFARNVSIRAVLERLP------   197 (251) |
|  | T ss\_pred |  | -----------------HHHHHHHHHHHHHHHHHHCChHHHHHHHHHHhcc-cccCCccccccccHHHHHHhcc------ |
|  |
|  |
|  | Q ss\_pred |  | hhccccccCCCccccccccHHHHHHHHHHHHHHHHHHHHHHHHh-----ccccchHHHHHHHHHHHHHh |
|  | Q XP\_016875493.1 | 326 | TLCYDWQVGSIPLVETIWDMRNLATIFLAVVMALLSLHCLAAFK-----RLEHKEVLVGLLFLVFPFIP   389 (456) |
|  | Q Consensus | 326 | ~~~~~~~~~~~~~~~~~~~~~~~~~~~~~~~~~~~~~~~~~~~~-----~~~~~~~~~~~~~~~~~~~~   389 (456) |
|  |  |  | .................+.........++ ++...........+...+.| |
|  | T Consensus | 198 | ---------------~~~~~~~~~~~~~~~~~~~~~~~~~~~~~~~~~~~~~~~~~~~~~~~~~~l~sP   251 (251) |
|  | T PF09594.11 | 198 | ---------------ELGSAASIMWLVAVALVIIAVAVAAYRILRTDLSAHNRLLAVSLVSLVALLCSP   251 (251) |
|  | T ss\_pred |  | ---------------cCCcHHHHHHHHHHHHHHHHHHHHHHHHHhcccccCChHHHHHHHHHHHHHhCC |
|  |
| --- | | | |
|  | Template alignmentCDD | | |
| 22. | PF05007.14 ; Mannosyl\_trans ; Mannosyltransferase (PIG-M) | | |
|  | Probability: 96.49%, E-value: 0.17, Score: 40.29, Aligned cols: 190, Identities: 15%, Similarity: 0.109, | | |
|  |
|  | Q ss\_pred |  | hHHHHHHHHHHHHHHHHHHHHHhcCCCCCCCCcchhHHHHHHHHHHHHHHHHHHHHHHHHHHHHHHHHHHhcccccchhH |
|  | Q XP\_016875493.1 | 166 | GRADVLACLLFLLAFLSYNRSLDQGCVGGSFPSTVSPFFLLLSLFLGTCAMLVKETGITVFGVCLVYDLFSLSNKQDKSY   245 (456) |
|  | Q Consensus | 166 | ~~~~~~~~~~~~l~~~~~~~~~~~~~~~~~~~~~~~~~~~~~~~~~~~la~~~k~~~~~~~~~~~~~~~~~~~~~~~~~~   245 (456) |
|  |  |  | ++.|....+++++++++..|.+ ...++++.|+|..+|...+++.+.+.+.....++++++.+. |
|  | T Consensus | 5 | G~~D~l~~~lvllal~~~~r~~-----------------~~~ag~~lgla~~~Kl~Pii~~~~l~l~~~~~~~~~~~~~~   67 (269) |
|  | T PF05007.14 | 5 | GNADSIVASLVLTTLYLIEKRL-----------------IACAAVFYGFAVHMKMYPVTYILPIALHLRPERDSDEGLRL   67 (269) |
|  | T ss\_pred |  | hhHHHHHHHHHHHHHHHHHcCC-----------------HHHHHHHHHHHHHhchHHHHHHHHHHHHhCccCCCCcchhh |
|  |
|  |
|  | Q ss\_pred |  | hhccCcchHHHhhH---------HHHHHHHHHHHHHHHHHHHHHHHhcCCCCc------cccCCCCcccchhHHHHHHHH |
|  | Q XP\_016875493.1 | 246 | LRASSNRNFLLTMR---------PFLKRAILVLSYVLVILYFRLWIMGGSMPL------FSEQDNPASFSPYILTRFLTY   310 (456) |
|  | Q Consensus | 246 | ~~~~~~~~~~~~~~---------~~~~~~~~~~~~~~~~~~~~~~~~~~~~~~------~~~~~~~~~~~~~~~~~~~~~   310 (456) |
|  |  |  | ++ +......+ .............+......+...+..... ....+...+.+.......... |
|  | T Consensus | 68 | ~~----~~~~~~~~~~~~~~~~~~~~~~~~~~~~~~~~l~~~~~~~~g~~~~~~~~~~~~~r~~~~~n~S~~~~~~~l~~   143 (269) |
|  | T PF05007.14 | 68 | AR----YSFQARLYDFLKRLCSWAVLLFVAIAGLTFLALSFGFYYKYGWEFLEHTYLYHLTRRDIRHNFSPYFYMLYLTA   143 (269) |
|  | T ss\_pred |  | hc----ccHHHHHHHHHHHhccHHHHHHHHHHHHHHHHHHHHHHHHHCHHHHHHHHHHHhccCCCCcCCCHHHHHHHHhc |
|  |
|  |
|  | Q ss\_pred |  | HHHHHHHHHHHHHhHhhccccccCCCccccccccHHHHHHHHHHHHHHHHHHHHHHHHhccccchHHHHHHHHHHHHHhH |
|  | Q XP\_016875493.1 | 311 | SYLLAFNVWLLLAPVTLCYDWQVGSIPLVETIWDMRNLATIFLAVVMALLSLHCLAAFKRLEHKEVLVGLLFLVFPFIPA   390 (456) |
|  | Q Consensus | 311 | ~~~~~~~~~~~~~~~~~~~~~~~~~~~~~~~~~~~~~~~~~~~~~~~~~~~~~~~~~~~~~~~~~~~~~~~~~~~~~~~~   390 (456) |
|  |  |  | ......... ................+. ++...........+.... |
|  | T Consensus | 144 | ~~~~~~~~~---------------------------------~~~~~~~~~~~~~~~~~~-~~~~~~~~~~~~~~f~~~-   188 (269) |
|  | T PF05007.14 | 144 | ESKWSFTLG---------------------------------IAAFLPQFILLSAASFAY-YRDLVFCCFLHTSIFVTF-   188 (269) |
|  | T ss\_pred |  | cCcchhHHH---------------------------------HHHHHHHHHHHHHHHHHc-cCcHHHHHHHHHHHHHHh- |
|  |
|  |
|  | Q ss\_pred |  | hccccCCCccccccccchHHHHHHHHHH |
|  | Q XP\_016875493.1 | 391 | SNLFFRVGFVVAERVLYMPSMGYCILFV   418 (456) |
|  | Q Consensus | 391 | ~~~~~~~~~~~~~Ry~~~~~~~~~ll~~   418 (456) |
|  |  |  | +...+++|.....|++.+... |
|  | T Consensus | 189 | -------~~v~~~qY~~W~lpll~l~~~   209 (269) |
|  | T PF05007.14 | 189 | -------NKVCTSQYFLWYLCLLPLVMP   209 (269) |
|  | T ss\_pred |  | -------chhhcHHHHHHHHHHHHHHch |
|  |
| --- | | | |
|  | Template alignmentCDD | | |
| 23. | PF16192.6 ; PMT\_4TMC ; C-terminal four TMM region of protein-O-mannosyltransferase | | |
|  | Probability: 91.61%, E-value: 2.3, Score: 31.49, Aligned cols: 108, Identities: 6%, Similarity: -0.1, | | |
|  |
|  | Q ss\_pred |  | cccHHHHHHHHHHHHHHHHHHHHHHHHhccccchH------------HHHHHHHHHHHHhHhccccCCCccccccccchH |
|  | Q XP\_016875493.1 | 342 | IWDMRNLATIFLAVVMALLSLHCLAAFKRLEHKEV------------LVGLLFLVFPFIPASNLFFRVGFVVAERVLYMP   409 (456) |
|  | Q Consensus | 342 | ~~~~~~~~~~~~~~~~~~~~~~~~~~~~~~~~~~~------------~~~~~~~~~~~~~~~~~~~~~~~~~~~Ry~~~~   409 (456) |
|  |  |  | ..++..+......++...........++++..... ....+..++..+|.... +......|.+++ |
|  | T Consensus | 58 | ~gNp~iw~~~~~~l~~~~~~~~~~~~~~~r~~~~~~~~~~~~~~~~~~~~~~g~~~~ylP~~~~----~r~~~~~~ylpa   133 (198) |
|  | T PF16192.6 | 58 | LGNPFVYWASTASLGLVGLVVVWYILRWQRGFKDLDSEEVDQIHYAGIYPVLGWFLHYLPFVIM----ARVTYVHHYYPA   133 (198) |
|  | T ss\_pred |  | cCCHHHHHHHHHHHHHHHHHHHHHHHHHhcCCCCCChHHHHHHHHHHHHHHHHHHHHHHHHHhc----cccccHHhHHHH |
|  |
|  |
|  | Q ss\_pred |  | HHHHHHHHHHHHHHHHHHhchhhHHHHHHHHHHHHHHHHHHHHH |
|  | Q XP\_016875493.1 | 410 | SMGYCILFVHGLSKLCTWLNRCGATTLIVSTVLLLLLFSWKTVK   453 (456) |
|  | Q Consensus | 410 | ~~~~~ll~~~~~~~~~~~~~~~~~~~~~~~~~~~~~~~~~~~~~   453 (456) |
|  |  |  | +++.+++.+..++...++..+..+.....+.+++.+........ |
|  | T Consensus | 134 | l~f~~l~~~~~l~~~~~~~~~~~~~~~~~~~~~~~~~~f~~~~P   177 (198) |
|  | T PF16192.6 | 134 | LYFAILALGFFVDWLLRNRSQAIQGAVYGVLYSVIVGLYITFIP   177 (198) |
|  | T ss\_pred |  | HHHHHHHHHHHHHHHHHccchHHHHHHHHHHHHHHHHHHHHhhH |
|  |
| --- | | | |
|  | Template alignmentCDD | | |
| 24. | PF14897.7 ; EpsG ; EpsG family | | |
|  | Probability: 89.45%, E-value: 5.2, Score: 32.12, Aligned cols: 298, Identities: 12%, Similarity: 0.017, | | |
|  |
|  | Q ss\_pred |  | HHHHHHHHHHHHhhhcCC-CccccchHHHHhCCCCCCCCCccccccc------ccccccCCCCCCCcccccchHHHHHHH |
|  | Q XP\_016875493.1 | 30 | AALLAGASCLCYGRSLQG-EFVHDDVWAIVNNPDVRPGAPLRWGIFT------NDFWGKGMAENTSHKSYRPLCVLTFKL   102 (456) |
|  | Q Consensus | 30 | ~~~l~~~~~~~~~~~~~~-~~~~De~~~~~~~~~~~~~~~~~~~~~~------~~~~~~~~~~~~~~~~~~Pl~~~~~~~   102 (456) |
|  |  |  | ....+...........+. +...|...|...-++..+.......... .+ ..|++..+... |
|  | T Consensus | 2 | ~~~~~~~~~l~~~~~~R~~~~g~D~~~Y~~~y~~~~~~~~~~~~~~~~~~~~~~~--------------~E~gf~~l~~~   67 (319) |
|  | T PF14897.7 | 2 | WFVSFATIQWIVLSGFRDVTVGADTAQYKALFLQSQTLPLGAFTDRFFEIVFTES--------------EDPGFYLFQRL   67 (319) |
|  | T ss\_pred |  | HHHHHHHHHHHHHHHHcCcCCCCCHHHHHHHHHHhccCCHHHHhhhhhhccCCCC--------------CCHHHHHHHHH |
|  |
|  |
|  | Q ss\_pred |  | HHHHhCCCchHHHHHHHHHHHHHHHHHHHHHHHHhccchHH---HHHHHHHHHHccccHHHHHHHHhHHHHHHHHH-HHH |
|  | Q XP\_016875493.1 | 103 | NIFLTGMNPFYFHAVNIILHCLVTLVLMYTCDKTVFKNRGL---AFVTALLFAVHPIHTEAVAGIVGRADVLACLL-FLL   178 (456) |
|  | Q Consensus | 103 | ~~~l~g~~~~~~rl~~~l~~~~~~~~~~~l~~~~~~~~~~~---a~~aa~l~a~~p~~~~~~~~~~~~~~~~~~~~-~~l   178 (456) |
|  |  |  | ... +|.| .+....+.+.+.....+... ++. ++.. ...+..++...+..... -..++..+ .+. |
|  | T Consensus | 68 | ~~~-~~~~---~~~~~~~~~~i~~~~~~~~~-~~~--~~~~~~~~~~~~~~~~~~~~~~~~-------Rq~lA~~~~~l~   133 (319) |
|  | T PF14897.7 | 68 | IQY-VITD---YQVYLVLIAMIFMIPLGYFI-YKY--SSEPLISFLLFSVLFYEFFAVTGL-------RQTVATALVVLV   133 (319) |
|  | T ss\_pred |  | HHH-hcCC---HHHHHHHHHHHHHHHHHHHH-HHh--CCchHHHHHHHHHHHHHHHHhHHH-------HHHHHHHHHHHH |
|  |
|  |
|  | Q ss\_pred |  | HHHHHHHHHhcCCCCCCCCcchhHHHHHHHHHHHHHHHHHHHHHHHHHHHHHHHHHHhcccccchhHhhccCcchHHHhh |
|  | Q XP\_016875493.1 | 179 | AFLSYNRSLDQGCVGGSFPSTVSPFFLLLSLFLGTCAMLVKETGITVFGVCLVYDLFSLSNKQDKSYLRASSNRNFLLTM   258 (456) |
|  | Q Consensus | 179 | ~~~~~~~~~~~~~~~~~~~~~~~~~~~~~~~~~~~la~~~k~~~~~~~~~~~~~~~~~~~~~~~~~~~~~~~~~~~~~~~   258 (456) |
|  |  |  | ++.... +++ .....+...+|...|.+++...+. ....+++.+ |
|  | T Consensus | 134 | a~~~~~---~~~--------------~~~~~~~~~la~~~H~sali~i~~----~~~~~~~~~-----------------   175 (319) |
|  | T PF14897.7 | 134 | GYHFVR---ARK--------------LGWFLLLVCIAMTIHKSSLIFVPF----YFLANKQLT-----------------   175 (319) |
|  | T ss\_pred |  | HHHHHH---cCc--------------HHHHHHHHHHHHHHHHHHHHHHHH----HHHhhcCCc----------------- |
|  |
|  |
|  | Q ss\_pred |  | HHHHHHHHHHHHHHHHHHHHHHHHhcC--CCCccccCCCCcccchhHHHHHHHHHHHHHHHHHHHHHhHhhccccccCCC |
|  | Q XP\_016875493.1 | 259 | RPFLKRAILVLSYVLVILYFRLWIMGG--SMPLFSEQDNPASFSPYILTRFLTYSYLLAFNVWLLLAPVTLCYDWQVGSI   336 (456) |
|  | Q Consensus | 259 | ~~~~~~~~~~~~~~~~~~~~~~~~~~~--~~~~~~~~~~~~~~~~~~~~~~~~~~~~~~~~~~~~~~~~~~~~~~~~~~~   336 (456) |
|  |  |  | +.......................... ......+.+........ |
|  | T Consensus | 176 | ~~~~~~~~~~~~~~~~~~~~i~~~~~~~~~~~~Y~~~~~~~~~~~~----------------------------------   221 (319) |
|  | T PF14897.7 | 176 | KAYLMTMFGVIVGLFVFRNPFFDLLVQVSGYDTYSAMDGAGAVNFS----------------------------------   221 (319) |
|  | T ss\_pred |  | HHHHHHHHHHHHHHHHhcHHHHHHHHHHhchhhhhhccCcchHHHH---------------------------------- |
|  |
|  |
|  | Q ss\_pred |  | ccccccccHHHHHHHHHHHHHHHHHHHHHHHHhccccc--hHHHHHHHHHHHHHhHhccccCCCccccccccchHHHHHH |
|  | Q XP\_016875493.1 | 337 | PLVETIWDMRNLATIFLAVVMALLSLHCLAAFKRLEHK--EVLVGLLFLVFPFIPASNLFFRVGFVVAERVLYMPSMGYC   414 (456) |
|  | Q Consensus | 337 | ~~~~~~~~~~~~~~~~~~~~~~~~~~~~~~~~~~~~~~--~~~~~~~~~~~~~~~~~~~~~~~~~~~~~Ry~~~~~~~~~   414 (456) |
|  |  |  | ................++++.+ ....................... .|......+... |
|  | T Consensus | 222 | ----------------~~~~~~~~~~~~~~~~~~~~~~~~~~~~~~~~~~~~~~~~~~~~~~~-----~R~~~~~~~~~~   280 (319) |
|  | T PF14897.7 | 222 | ----------------LMLLSVLFVALWRKEQILANNPSAIHFFNALLLAACLLPLTFLNPSM-----MRLVQYFSLFLL   280 (319) |
|  | T ss\_pred |  | ----------------HHHHHHHHHHHHHHHHHhcCCcchHHHHHHHHHHHHHHHHHhcChhH-----HHHHHHHHHHHH |
|  |
|  |
|  | Q ss\_pred |  | HHHHHHHHHHHHHhchhhHHHHHHHHHHHHHHHHH |
|  | Q XP\_016875493.1 | 415 | ILFVHGLSKLCTWLNRCGATTLIVSTVLLLLLFSW   449 (456) |
|  | Q Consensus | 415 | ll~~~~~~~~~~~~~~~~~~~~~~~~~~~~~~~~~   449 (456) |
|  |  |  | +....+.+..++.+++.................. |
|  | T Consensus | 281 | -~~~~~~~~~~~~~~~~~~~~~~~~~~~~~~~~~~   314 (319) |
|  | T PF14897.7 | 281 | -LMIPEIVGTFERRERLVVYYSAVMLLGLLFIREA   314 (319) |
|  | T ss\_pred |  | -HHHHHHHhcCChHHHHHHHHHHHHHHHHHHHHHc |
|  |
| --- | | | |
|  | Template alignmentCDD | | |
| 25. | PF09971.10 ; DUF2206 ; Predicted membrane protein (DUF2206) | | |
|  | Probability: 83.62%, E-value: 13, Score: 30.82, Aligned cols: 213, Identities: 8%, Similarity: -0.023, | | |
|  |
|  | Q ss\_pred |  | HHHHHHHHHHHHHHHHHHHHHHHHHHHHHHhccccc------chhHhhccCcchHHHhhHHHHH------HHHHHHHHHH |
|  | Q XP\_016875493.1 | 206 | LLSLFLGTCAMLVKETGITVFGVCLVYDLFSLSNKQ------DKSYLRASSNRNFLLTMRPFLK------RAILVLSYVL   273 (456) |
|  | Q Consensus | 206 | ~~~~~~~~la~~~k~~~~~~~~~~~~~~~~~~~~~~------~~~~~~~~~~~~~~~~~~~~~~------~~~~~~~~~~   273 (456) |
|  |  |  | ++..++...-..+|+....+...++........-.+ +++..+ . .....++..+ |
|  | T Consensus | 3 | ~L~~i~~~~lv~sH~~t~~~~~~~l~~~~~~~~~~~~~~~~~~~~~~~---------------~~~~~~~~~~~~~~~~~   67 (390) |
|  | T PF09971.10 | 3 | ILFSVFSCGIIISHYGLTYMVIGLIALSYVLFTFINLVARYINTDKVI---------------IPVTPIRLNFLHICIFI   67 (390) |
|  | T ss\_pred |  | HHHHHHHHHHHHhchHHHHHHHHHHHHHHHHHHHHHHHHHhcCCCCCc---------------CCCCcccccHHHHHHHH |
|  |
|  |
|  | Q ss\_pred |  | HHHHHHHHHhcCCCCccccCCCCcccchhHHHHHHHHHHHHHHHHHHHHHhHhhccccccCCCccccccccHHHHHHHHH |
|  | Q XP\_016875493.1 | 274 | VILYFRLWIMGGSMPLFSEQDNPASFSPYILTRFLTYSYLLAFNVWLLLAPVTLCYDWQVGSIPLVETIWDMRNLATIFL   353 (456) |
|  | Q Consensus | 274 | ~~~~~~~~~~~~~~~~~~~~~~~~~~~~~~~~~~~~~~~~~~~~~~~~~~~~~~~~~~~~~~~~~~~~~~~~~~~~~~~~   353 (456) |
|  |  |  | +....++.... +....+............+...+.................+............. |
|  | T Consensus | 68 | v~~~~W~~~~~---------------~~~~~~~~~~~~~~~~~~~~~~~~~~~~~~~~~~~~~~~~~~~~~~~~~~~~~~   132 (390) |
|  | T PF09971.10 | 68 | FIALSWYIAIT---------------SSTAFYSVSSVIYQVISSMFTESLNPTASQGLAIIQKVPVSQMHLLYTYIYYFN   132 (390) |
|  | T ss\_pred |  | HHHHHHHHHHh---------------cchHHHHHHHHHHHHHHHHhHHhcCCccchhHHHHhcCCCchHHHHHHHHHHHH |
|  |
|  |
|  | Q ss\_pred |  | HHHHHHHHHHH----HHHHhccccchHHHHHHHHHHHHHhHhccccCCC-ccccccccchHHHHHHHHHHHHHHHHHHHh |
|  | Q XP\_016875493.1 | 354 | AVVMALLSLHC----LAAFKRLEHKEVLVGLLFLVFPFIPASNLFFRVG-FVVAERVLYMPSMGYCILFVHGLSKLCTWL   428 (456) |
|  | Q Consensus | 354 | ~~~~~~~~~~~----~~~~~~~~~~~~~~~~~~~~~~~~~~~~~~~~~~-~~~~~Ry~~~~~~~~~ll~~~~~~~~~~~~   428 (456) |
|  |  |  | .+..++..... ...+++++.......+....+..+.......... .....|.+.....+++++++.++..+.+.. |
|  | T Consensus | 133 | ~~l~~iG~~~~~~~~~~~~~~~~~~~~~~~~~~~~~~~~~~~~~~p~~~~~~~~~R~~~~~~~~~~~~a~~g~~~l~~~~   212 (390) |
|  | T PF09971.10 | 133 | QVCIVLGLLYLSYKTFARKNMYNYSIMQLIMCGVAVMVLVGSIVLPYFASALNTTRIYHIMQFFVSPVYIIGFIFALESI   212 (390) |
|  | T ss\_pred |  | HHHHHHHHHHHHHHHHhccCccCCCHHHHHHHHHHHHHHHHHHHHHHHHHhcChHHHHHHHHHHHHHHHHHHHHHHHHhh |
|  |
|  |
|  | Q ss\_pred |  | chh---------------hHHHHHHHHHHHHHHHH |
|  | Q XP\_016875493.1 | 429 | NRC---------------GATTLIVSTVLLLLLFS   448 (456) |
|  | Q Consensus | 429 | ~~~---------------~~~~~~~~~~~~~~~~~   448 (456) |
|  |  |  | ++. ..+...+.+++.++... |
|  | T Consensus | 213 | ~~~~~~~~~~~~~~~~~~~~~~~~~~lv~~~~~~s   247 (390) |
|  | T PF09971.10 | 213 | PKVYARIVKSPFRSNLSFTYGIISLFLCVYLLFNS   247 (390) |
|  | T ss\_pred |  | HHHHHHhhcCccccccchHHHHHHHHHHHHHHHHh |
|  |
| --- | | | |
|  | Template alignmentCDD | | |
| 26. | PF10060.10 ; DUF2298 ; Uncharacterized membrane protein (DUF2298) | | |
|  | Probability: 83.23%, E-value: 18, Score: 32.19, Aligned cols: 364, Identities: 10%, Similarity: -0.005, | | |
|  |
|  | Q ss\_pred |  | hhhhhHHHHHHHHHHHHHHHHHHhhhcCCCccc-cchHHHHhCCCC-CCCCCcccccccccccccCCCCCCCcccccchH |
|  | Q XP\_016875493.1 | 19 | RRGCGLAPAGAAALLAGASCLCYGRSLQGEFVH-DDVWAIVNNPDV-RPGAPLRWGIFTNDFWGKGMAENTSHKSYRPLC   96 (456) |
|  | Q Consensus | 19 | ~~~~~~~~~~~~~~l~~~~~~~~~~~~~~~~~~-De~~~~~~~~~~-~~~~~~~~~~~~~~~~~~~~~~~~~~~~~~Pl~   96 (456) |
|  |  |  | +...+.....-++.+++..........+.+..+ |...+.....+. .+..-+..+.+-.+ ..-.|+.+. |
|  | T Consensus | 49 | ~~~~~~~l~~e~vf~~~f~~~~~~r~~~p~i~~~Ek~md~~~i~s~~~~~~~Pp~dPw~aG----------~~l~Yyyfg   118 (597) |
|  | T PF10060.10 | 49 | LPSLRYILLFELLFLGAFAAWAWVRAHDPAADHTEQPMDLMFMHSIRASLTYPPHDAWLAG----------YPISYYYFG   118 (597) |
|  | T ss\_pred |  | ccchHHHHHHHHHHHHHHHHHHHHHHhCCCCCCCCChhHHHHHHHHHhcCCCCccchhhcC----------CCccccHHH |
|  |
|  |
|  | Q ss\_pred |  | HHHHHHHHHHhC-CCchHHHHHHHHHHHHHHHHHHHHHHHHhccchH--------HHHHHHHHHHHccccHHHHHHHHhH |
|  | Q XP\_016875493.1 | 97 | VLTFKLNIFLTG-MNPFYFHAVNIILHCLVTLVLMYTCDKTVFKNRG--------LAFVTALLFAVHPIHTEAVAGIVGR   167 (456) |
|  | Q Consensus | 97 | ~~~~~~~~~l~g-~~~~~~rl~~~l~~~~~~~~~~~l~~~~~~~~~~--------~a~~aa~l~a~~p~~~~~~~~~~~~   167 (456) |
|  |  |  | +++.+...++.| ....++++.......+.+..+|.++ +.+.++++ .|++++++..+.............. |
|  | T Consensus | 119 | ~~~~A~l~~l~gi~~~~~~nl~~~~~~al~~~~~~~l~-~~l~~~~~~~~~~~~~~g~la~~l~~~~gnl~~~~~~~~~~   197 (597) |
|  | T PF10060.10 | 119 | YWLMNMVGLMAGQSAAVAYNLSQAVWFGLLLSGAFGIG-YNLVAAAGRRFVAALGGGWVATLLVGLSSNLQGLLEWLHAN   197 (597) |
|  | T ss\_pred |  | HHHHHHHHHHhCCCHHHHHHHHHHHHHHHHHHHHHHHH-HHHHHhccccccHHHHHHHHHHHHHHHhcccHHHHHHHHhC |
|  |
|  |
|  | Q ss\_pred |  | H--------------------------------------------------------------------HHHHHHHHHHH |
|  | Q XP\_016875493.1 | 168 | A--------------------------------------------------------------------DVLACLLFLLA   179 (456) |
|  | Q Consensus | 168 | ~--------------------------------------------------------------------~~~~~~~~~l~   179 (456) |
|  |  |  | . +.+...+.+++ |
|  | T Consensus | 198 | ~~~~~~~~~~~~~~~~~~~~~~~~~~~~~~~w~~w~ssRvI~~~~~~~~~~~tI~EFP~fSfl~gDLHpH~~alPf~ll~   277 (597) |
|  | T PF10060.10 | 198 | GVDISWLAAWLQVRGFPENAEVTRQWFISYGWWWWRSSRVLADVSLRGDHIEVIDEFPAFSYILGDNHPHVAAMPFAMLA   277 (597) |
|  | T ss\_pred |  | CCCchhHHHHhhhcCCCcchhhhccccccCccccccceeeeecccCCCCCCcccccCchHHHhcCCCChhhhHHHHHHHH |
|  |
|  |
|  | Q ss\_pred |  | HHHHHHHHhcCCCCCCCCcch-----------hHHHHHHHHHHHHHHHHHHHHHHHHHHHHHHHHHHhccccc------- |
|  | Q XP\_016875493.1 | 180 | FLSYNRSLDQGCVGGSFPSTV-----------SPFFLLLSLFLGTCAMLVKETGITVFGVCLVYDLFSLSNKQ-------   241 (456) |
|  | Q Consensus | 180 | ~~~~~~~~~~~~~~~~~~~~~-----------~~~~~~~~~~~~~la~~~k~~~~~~~~~~~~~~~~~~~~~~-------   241 (456) |
|  |  |  | +.+.....+++++++..++.. .....++.+++.|....++.--......+.+........++ |
|  | T Consensus | 278 | l~l~~~~~~~~~~~~~~~~~~~~~~~~~~~~~~~~~~ll~gll~G~l~~~NtWD~p~~~~l~~~~~~~~~~~~~~~~~~~   357 (597) |
|  | T PF10060.10 | 278 | VAAALVIFLQNSSSNFSSESRAKFNFAPLFPLGWGGFLLVAVITGSLLFLNTWDYPPYWLLTTFSIAVGVVGGVVRVKNF   357 (597) |
|  | T ss\_pred |  | HHHHHHHHhcCCCCCCCchhhhccCCCCccccchHHHHHHHHHHHHHHHHccCCHHHHHHHHHHHHHHHHhccccccccc |
|  |
|  |
|  | Q ss\_pred |  | -------chhHhhccCcchHHHhhHHHHHHHHHHHHHHHHHHHHHHHHhcCCCCccccCCCCcccchhHHHHHHHHHHHH |
|  | Q XP\_016875493.1 | 242 | -------DKSYLRASSNRNFLLTMRPFLKRAILVLSYVLVILYFRLWIMGGSMPLFSEQDNPASFSPYILTRFLTYSYLL   314 (456) |
|  | Q Consensus | 242 | -------~~~~~~~~~~~~~~~~~~~~~~~~~~~~~~~~~~~~~~~~~~~~~~~~~~~~~~~~~~~~~~~~~~~~~~~~~   314 (456) |
|  |  |  | ..+..+ +.....+...+........+..+..+......-.....+...+-.. |
|  | T Consensus | 358 | ~~~~~~~~~~~~~---------------~~~~~~~~~~~~a~ll~lPF~l~f~~~~~gi~~~~~~~T~l~~~l~------   416 (597) |
|  | T PF10060.10 | 358 | LPLQFLPLLPPLL---------------QTTIAGLALFVAALLLYLPYLLTAQSQVGGLIPNLFHPTRFSQYVA------   416 (597) |
|  | T ss\_pred |  | cccccccchHHHH---------------HHHHHHHHHHHHHHHHHHHHHHhccccCCCCcccCCCCCCHHHHHH------ |
|  |
|  |
|  | Q ss\_pred |  | HHHHHHHHHhHhhccccccCCCccccccccHHHHHHHHHHHHHHHHHHHHHHHHhccccchHHHHHHHHHHHHHhHhccc |
|  | Q XP\_016875493.1 | 315 | AFNVWLLLAPVTLCYDWQVGSIPLVETIWDMRNLATIFLAVVMALLSLHCLAAFKRLEHKEVLVGLLFLVFPFIPASNLF   394 (456) |
|  | Q Consensus | 315 | ~~~~~~~~~~~~~~~~~~~~~~~~~~~~~~~~~~~~~~~~~~~~~~~~~~~~~~~~~~~~~~~~~~~~~~~~~~~~~~~~   394 (456) |
|  |  |  | ...+.+...........++.+++..............+..... |
|  | T Consensus | 417 | -------------------------------------i~Glfl~l~~~~l~~~~~~~~~~~~~~~~~~~~~~~~~~~~~~   459 (597) |
|  | T PF10060.10 | 417 | -------------------------------------MFATALLTLTALLTFGWSVFRPRLKVVMICLALTLGTPALLLT   459 (597) |
|  | T ss\_pred |  | -------------------------------------HHHHHHHHHHHHHHHHhhcccchHHHHHHHHHHHHHHHHHHHH |
|  |
|  |
|  | Q ss\_pred |  | c-------------------------CCCccccccccchHHHHHHHHHHHHHHHHHHHhchh------------------ |
|  | Q XP\_016875493.1 | 395 | F-------------------------RVGFVVAERVLYMPSMGYCILFVHGLSKLCTWLNRC------------------   431 (456) |
|  | Q Consensus | 395 | ~-------------------------~~~~~~~~Ry~~~~~~~~~ll~~~~~~~~~~~~~~~------------------   431 (456) |
|  |  |  | . ........|........+.++.........-+.... |
|  | T Consensus | 460 | ~~~~~~~~~~~~~~~~~~~~~~~~~~~~~~~~~~~~~~~~~~~~ll~~l~~~~~~~~~~~~~~~~~~~~~~~~~~~~~~~   539 (597) |
|  | T PF10060.10 | 460 | FIAWVATGTEEGRASLGNVALPDGASSYLPFIVERWTAQPFTFLIVGAMTAVALALLWTGIQHMVGAKNFLPQHFSPQQG   539 (597) |
|  | T ss\_pred |  | HHHHHhcCChhhhhccccccCCCCcccchhHHHHHhccchHHHHHHHHHHHHHHHHHHhhhhhhccccccCcccCCCCCC |
|  |
|  |
|  | Q ss\_pred |  | -----------hHHHHHHHHHHHHHHHHHHH |
|  | Q XP\_016875493.1 | 432 | -----------GATTLIVSTVLLLLLFSWKT   451 (456) |
|  | Q Consensus | 432 | -----------~~~~~~~~~~~~~~~~~~~~   451 (456) |
|  |  |  | .......+++.+........ |
|  | T Consensus | 540 | ~~~~~~~~~~~~~~~f~l~L~~~gl~Lil~~   570 (597) |
|  | T PF10060.10 | 540 | ALDSTPGVAAPTPLLFVLALAVIGLGLTFTP   570 (597) |
|  | T ss\_pred |  | CcCCCCCCCCCHHHHHHHHHHHHHHHHHHHH |
|  |
| --- | | | |
|  | Template alignmentCDD | | |
| 27. | PF01098.20 ; FTSW\_RODA\_SPOVE ; Cell cycle protein | | |
|  | Probability: 32.48%, E-value: 140, Score: 23.84, Aligned cols: 254, Identities: 7%, Similarity: -0.148, | | |
|  |
|  | Q ss\_pred |  | HHHHHHHHHHHHHHHHHHHHHHhccchHHHHHHHHHHHHcccc-------------HHHHHHHHhHHHHHHHHHHHHHHH |
|  | Q XP\_016875493.1 | 115 | HAVNIILHCLVTLVLMYTCDKTVFKNRGLAFVTALLFAVHPIH-------------TEAVAGIVGRADVLACLLFLLAFL   181 (456) |
|  | Q Consensus | 115 | rl~~~l~~~~~~~~~~~l~~~~~~~~~~~a~~aa~l~a~~p~~-------------~~~~~~~~~~~~~~~~~~~~l~~~   181 (456) |
|  |  |  | ++...+.+....+.++++=.+...+..+....++++..+.... +..........|..-..+.+.... |
|  | T Consensus | 40 | q~~~~~iG~~~~~~~~~~dy~~l~~~~~~~~~~~~~ll~~~~~~~~g~~~~G~~~wi~lg~~~~qp~el~~~~~il~~a~   119 (357) |
|  | T PF01098.20 | 40 | QLLFAGIGVIAMFFIMNVDYWTWRTWSKLLMVICFFLLVLVLIPGVGMVRNGSRSWIGVGAFSIQPSEFMKLAMIAFLAK   119 (357) |
|  | T ss\_pred |  | HHHHHHHHHHHHHHHHcCCHHHHHHHHHHHHHHHHHHHHHHHccccccccCccCCeEEcCCcccCHHHHHHHHHHHHHHH |
|  |
|  |
|  | Q ss\_pred |  | HHHHHHhcCCCCCCCCcchhHHHHHHHHHHHHHHHHHHHHHHHHHHHHHHHHHHhcccccchhHhhccCcchHHHhhHHH |
|  | Q XP\_016875493.1 | 182 | SYNRSLDQGCVGGSFPSTVSPFFLLLSLFLGTCAMLVKETGITVFGVCLVYDLFSLSNKQDKSYLRASSNRNFLLTMRPF   261 (456) |
|  | Q Consensus | 182 | ~~~~~~~~~~~~~~~~~~~~~~~~~~~~~~~~la~~~k~~~~~~~~~~~~~~~~~~~~~~~~~~~~~~~~~~~~~~~~~~   261 (456) |
|  |  |  | .+.+.+++++++.+. .........+...+....+..+-.+.............+.+ ++. |
|  | T Consensus | 120 | ~~~~~~~~~~~~~~~----~~~~~~~~~~~~~l~~~~~d~g~~~i~~~~~~~~l~~~~~~-----------------~~~   178 (357) |
|  | T PF01098.20 | 120 | FLSEKQKNITSFRRG----FVPALGIVFSAFLIIMCQPDLGTGTVMVGTCIVMIFVAGAR-----------------IAH   178 (357) |
|  | T ss\_pred |  | HHHHhHhhcccchhc----cHHHHHHHHHHHHHHHhCCCHHHHHHHHHHHHHHHHHcCCC-----------------HHH |
|  |
|  |
|  | Q ss\_pred |  | HHHHHHHHHHHHHHHHHHHHHhcCCCCccccCCCCcccchhHHHHHHHHHHHHHHHHHHHHHhHh--hccccccCCCccc |
|  | Q XP\_016875493.1 | 262 | LKRAILVLSYVLVILYFRLWIMGGSMPLFSEQDNPASFSPYILTRFLTYSYLLAFNVWLLLAPVT--LCYDWQVGSIPLV   339 (456) |
|  | Q Consensus | 262 | ~~~~~~~~~~~~~~~~~~~~~~~~~~~~~~~~~~~~~~~~~~~~~~~~~~~~~~~~~~~~~~~~~--~~~~~~~~~~~~~   339 (456) |
|  |  |  | .......................+-.....+................+...+-...-.....+.. .......+++... |
|  | T Consensus | 179 | ~~~~~~~~~~~~~~~~~~~~~~~~Ri~~~~~p~~~~~~~gyq~~~~~~~i~~~g~~G~G~~~~~~~~~~lp~~~~D~i~~   258 (357) |
|  | T PF01098.20 | 179 | FVFLGLIGLSGFVGLVLSAPYRIKRITSYLNPWEDPLGSGFQIIQSLYAVGPGGLFGMGLGQSRQKFFYLPEPQTDFIFA   258 (357) |
|  | T ss\_pred |  | HHHHHHHHHHHHHHHHHCchHHHHHHHHHhCCCCCCCCCCHHHHHHHHHHHcCCccccCCCCCccccccCCcccCchHHH |
|  |
|  |
|  | Q ss\_pred |  | cccccHHHHHHHHHHHHHHHHHHHHHHHHhccccchHHHHHHHHHHHHHh |
|  | Q XP\_016875493.1 | 340 | ETIWDMRNLATIFLAVVMALLSLHCLAAFKRLEHKEVLVGLLFLVFPFIP   389 (456) |
|  | Q Consensus | 340 | ~~~~~~~~~~~~~~~~~~~~~~~~~~~~~~~~~~~~~~~~~~~~~~~~~~   389 (456) |
|  |  |  | .-...........................++.+++............+.. |
|  | T Consensus | 259 | ~i~~~~G~ig~~~~i~~~~~l~~~~~~~~~~~~~~~~~~l~~g~~~~l~~   308 (357) |
|  | T PF01098.20 | 259 | ILSEELGFIGGTLILLLFSVLLWRGIRIALGAPDLYGSFVAVGIISMIAI   308 (357) |
|  | T ss\_pred |  | HHHHHccHHHHHHHHHHHHHHHHHHHHHHHcCCCHHHHHHHHHHHHHHHH |
|  |
| --- | | | |
|  | Template alignmentCDD | | |
| 28. | PF04973.13 ; NMN\_transporter ; Nicotinamide mononucleotide transporter | | |
|  | Probability: 32.19%, E-value: 95, Score: 21.87, Aligned cols: 97, Identities: 10%, Similarity: 0.063, | | |
|  |
|  | Q ss\_pred |  | HHHHHHHHHHHHHHHHHHHHHhccchHHHHHHHHHHHHccccHHHHHHHHhHHHHHHHHHHHHHHHHHHHHHhcCCCCCC |
|  | Q XP\_016875493.1 | 116 | AVNIILHCLVTLVLMYTCDKTVFKNRGLAFVTALLFAVHPIHTEAVAGIVGRADVLACLLFLLAFLSYNRSLDQGCVGGS   195 (456) |
|  | Q Consensus | 116 | l~~~l~~~~~~~~~~~l~~~~~~~~~~~a~~aa~l~a~~p~~~~~~~~~~~~~~~~~~~~~~l~~~~~~~~~~~~~~~~~   195 (456) |
|  |  |  | ..+.+.+.+++...+... |.-..+...+.+..+++...-..... +. .-.+........++.+.+++++++++++ |
|  | T Consensus | 2 | ~~~~~~~~~g~~~~~l~~-~~~~~~~~~g~~~~~~~~~~~~~~~~--~~---~~~l~~~y~~~~i~G~~~W~k~~~~~~~   75 (180) |
|  | T PF04973.13 | 2 | YIEIFASVMGIINVWLLA-REKVSNFLFGMITVAVFLYIFITQGL--YA---MAVLAAFQFIFNVYGWYHWIARSGEEEV   75 (180) |
|  | T ss\_pred |  | HHHHHHHHHHHHHHHHHH-ccchhHHHHHHHHHHHHHHHHHHCCC--hH---HHHHHHHHHHHHHHHHHHhHhcCCCCCc |
|  |
|  |
|  | Q ss\_pred |  | C--CcchhHHHHHHHHHHHHHHHHH |
|  | Q XP\_016875493.1 | 196 | F--PSTVSPFFLLLSLFLGTCAMLV   218 (456) |
|  | Q Consensus | 196 | ~--~~~~~~~~~~~~~~~~~la~~~   218 (456) |
|  |  |  | . ++.................... |
|  | T Consensus | 76 | v~v~~~~~~~~~~~~~~~~v~~~~~   100 (180) |
|  | T PF04973.13 | 76 | KATVRLDLKGWIFYIIFILVAWIGW   100 (180) |
|  | T ss\_pred |  | ccceecCHHHHHHHHHHHHHHHHHH |
|  |
| --- | | | |
|  | Template alignmentCDD | | |
| 29. | PF09323.11 ; DUF1980 ; Domain of unknown function (DUF1980) | | |
|  | Probability: 28.28%, E-value: 120, Score: 21.6, Aligned cols: 74, Identities: 9%, Similarity: 0.001, | | |
|  |
|  | Q ss\_pred |  | HHHHHHHHHHHHHhHhccccCCCccccccccchHHHHHHHHHHHHHHHHHHHhchh------------------------ |
|  | Q XP\_016875493.1 | 376 | VLVGLLFLVFPFIPASNLFFRVGFVVAERVLYMPSMGYCILFVHGLSKLCTWLNRC------------------------   431 (456) |
|  | Q Consensus | 376 | ~~~~~~~~~~~~~~~~~~~~~~~~~~~~Ry~~~~~~~~~ll~~~~~~~~~~~~~~~------------------------   431 (456) |
|  |  |  | ....++..+..++......-....+.++|+........++++..++..+.+..+.. |
|  | T Consensus | 2 | l~~lill~~~~~l~~l~~tg~i~~yI~Pr~~~~~~~a~~~l~il~~~~~~~~~~~~~~~~~~~~~~~~~~~h~~~~~~~~   81 (187) |
|  | T PF09323.11 | 2 | LRFIVLFGFAYFFMKLHATGDISKYINMKYAYLSFSMIFAMGFLCLYQLVKWVRAGNEAHDHQHGAHHGHSHETDENTWY   81 (187) |
|  | T ss\_pred |  | HHHHHHHHHHHHHHHHHHhCCHHHHcCcchHHHHHHHHHHHHHHHHHHHHHHHHccCcccccccCCCCCCCCCCCcchhH |
|  |
|  |
|  | Q ss\_pred |  | hHHHHHHHHHHHHHHHHH |
|  | Q XP\_016875493.1 | 432 | GATTLIVSTVLLLLLFSW   449 (456) |
|  | Q Consensus | 432 | ~~~~~~~~~~~~~~~~~~   449 (456) |
|  |  |  | .......++++.++.... |
|  | T Consensus | 82 | ~~~~~~~~~~lPlll~~l   99 (187) |
|  | T PF09323.11 | 82 | KKMFTYGMILIPVVTGIF   99 (187) |
|  | T ss\_pred |  | HHHHHHHHHHHHHHHHHh |
|  |
| --- | | | |
|  | Template alignmentCDD | | |
| 30. | PF05562.12 ; WCOR413 ; Cold acclimation protein WCOR413 | | |
|  | Probability: 26.29%, E-value: 130, Score: 21.48, Aligned cols: 70, Identities: 11%, Similarity: 0.117, | | |
|  |
|  | Q ss\_pred |  | HHHHHHHHHhccchHHHHHHHHHHHHccccHHHHHHHHhHHHHHHHHHHHHHHHHHHHHHhcCCCCCCCCcchhHHHHHH |
|  | Q XP\_016875493.1 | 128 | VLMYTCDKTVFKNRGLAFVTALLFAVHPIHTEAVAGIVGRADVLACLLFLLAFLSYNRSLDQGCVGGSFPSTVSPFFLLL   207 (456) |
|  | Q Consensus | 128 | ~~~~l~~~~~~~~~~~a~~aa~l~a~~p~~~~~~~~~~~~~~~~~~~~~~l~~~~~~~~~~~~~~~~~~~~~~~~~~~~~   207 (456) |
|  |  |  | .++.+.|... +++.|+++.++=.+.|.++-. +.. ......+++..+=..+....|.. ... |
|  | T Consensus | 82 | ~~f~~~rge~--G~WiAFlav~lrLFfp~~fP~--~le--lP~s~~LL~vvaP~~~a~~lR~~--------------~~g   141 (182) |
|  | T PF05562.12 | 82 | ILFNFFSGQV--GKWIAFIAVVLRLFFPKRFPD--WLE--MPAALILVIVVAPSLFSSTIRGD--------------WIG   141 (182) |
|  | T ss\_pred |  | HHHHHhcCCc--hhHHHHHHHHHHHhCCCCCch--hhc--CcHHHHHHHHHhHHHHHHHhhcc--------------hHH |
|  |
|  |
|  | Q ss\_pred |  | HHHHHHHHHH |
|  | Q XP\_016875493.1 | 208 | SLFLGTCAML   217 (456) |
|  | Q Consensus | 208 | ~~~~~~la~~   217 (456) |
|  |  |  | ..+++.++.+ |
|  | T Consensus | 142 | ~iicL~Ig~y   151 (182) |
|  | T PF05562.12 | 142 | LVICLAIGCY   151 (182) |
|  | T ss\_pred |  | HHHHHHHHHH |
|  |
| --- | | | |
|  | Template alignmentCDD | | |
| 31. | PF12273.9 ; RCR ; Chitin synthesis regulation, resistance to Congo red | | |
|  | Probability: 23.84%, E-value: 48, Score: 22.45, Aligned cols: 28, Identities: 14%, Similarity: 0.185, | | |
|  |
|  | Q ss\_pred |  | HHHHHHHHHHHHHHHHHHHHHHHhcccc |
|  | Q XP\_016875493.1 | 346 | RNLATIFLAVVMALLSLHCLAAFKRLEH   373 (456) |
|  | Q Consensus | 346 | ~~~~~~~~~~~~~~~~~~~~~~~~~~~~   373 (456) |
|  |  |  | ..++.++++++++++++.....+||+++ |
|  | T Consensus | 2 | Wvl~~iii~~~l~~~~~~~~~~RRRrr~   29 (138) |
|  | T PF12273.9 | 2 | WVVLAGVIVIVLVIFMLCTCTARRRRRR   29 (138) |
|  | T ss\_pred |  | HHHHHHHHHHHHHHHHHHHHHHHHHHHc |
|  |

---

If you use HHpred on our Toolkit for your research, please cite as appropriate:

A Completely Reimplemented MPI Bioinformatics Toolkit
with a New HHpred Server at its Core.  
Zimmermann L, Stephens A, Nam SZ, Rau D,
Kübler J, Lozajic M, Gabler F, Söding J, Lupas AN, Alva V.
J Mol Biol. 2018 Jul 20. S0022-2836(17)30587-9.

  

Protein homology detection by HMM-HMM comparison.  
Söding J. Bioinformatics. 2005 Apr 1;21(7):951-60.  
  
Fast and accurate automatic structure prediction with HHpred.  
Hildebrand A, Remmert M, Biegert A, Söding J. Proteins. 2009;77 Suppl 9:128-32.  
  
Automatic Prediction of Protein 3D Structures by Probabilistic Multi-template Homology Modeling.  
Meier A, Söding J. PLoS Comput Biol. 2015 Oct 23;11(10):e1004343.

Download

---

If you use HHpred on our Toolkit for your research, please cite as appropriate:

A Completely Reimplemented MPI Bioinformatics Toolkit
with a New HHpred Server at its Core.  
Zimmermann L, Stephens A, Nam SZ, Rau D,
Kübler J, Lozajic M, Gabler F, Söding J, Lupas AN, Alva V.
J Mol Biol. 2018 Jul 20. S0022-2836(17)30587-9.

  

Protein homology detection by HMM-HMM comparison.  
Söding J. Bioinformatics. 2005 Apr 1;21(7):951-60.  
  
Fast and accurate automatic structure prediction with HHpred.  
Hildebrand A, Remmert M, Biegert A, Söding J. Proteins. 2009;77 Suppl 9:128-32.  
  
Automatic Prediction of Protein 3D Structures by Probabilistic Multi-template Homology Modeling.  
Meier A, Söding J. PLoS Comput Biol. 2015 Oct 23;11(10):e1004343.

Loading...

---

If you use HHpred on our Toolkit for your research, please cite as appropriate:

A Completely Reimplemented MPI Bioinformatics Toolkit
with a New HHpred Server at its Core.  
Zimmermann L, Stephens A, Nam SZ, Rau D,
Kübler J, Lozajic M, Gabler F, Söding J, Lupas AN, Alva V.
J Mol Biol. 2018 Jul 20. S0022-2836(17)30587-9.

  

Protein homology detection by HMM-HMM comparison.  
Söding J. Bioinformatics. 2005 Apr 1;21(7):951-60.  
  
Fast and accurate automatic structure prediction with HHpred.  
Hildebrand A, Remmert M, Biegert A, Söding J. Proteins. 2009;77 Suppl 9:128-32.  
  
Automatic Prediction of Protein 3D Structures by Probabilistic Multi-template Homology Modeling.  
Meier A, Söding J. PLoS Comput Biol. 2015 Oct 23;11(10):e1004343.

Loading hits...

---

If you use HHpred on our Toolkit for your research, please cite as appropriate:

A Completely Reimplemented MPI Bioinformatics Toolkit
with a New HHpred Server at its Core.  
Zimmermann L, Stephens A, Nam SZ, Rau D,
Kübler J, Lozajic M, Gabler F, Söding J, Lupas AN, Alva V.
J Mol Biol. 2018 Jul 20. S0022-2836(17)30587-9.

  

Protein homology detection by HMM-HMM comparison.  
Söding J. Bioinformatics. 2005 Apr 1;21(7):951-60.  
  
Fast and accurate automatic structure prediction with HHpred.  
Hildebrand A, Remmert M, Biegert A, Söding J. Proteins. 2009;77 Suppl 9:128-32.  
  
Automatic Prediction of Protein 3D Structures by Probabilistic Multi-template Homology Modeling.  
Meier A, Söding J. PLoS Comput Biol. 2015 Oct 23;11(10):e1004343.

Loading hits...

---

If you use HHpred on our Toolkit for your research, please cite as appropriate:

A Completely Reimplemented MPI Bioinformatics Toolkit
with a New HHpred Server at its Core.  
Zimmermann L, Stephens A, Nam SZ, Rau D,
Kübler J, Lozajic M, Gabler F, Söding J, Lupas AN, Alva V.
J Mol Biol. 2018 Jul 20. S0022-2836(17)30587-9.

  

Protein homology detection by HMM-HMM comparison.  
Söding J. Bioinformatics. 2005 Apr 1;21(7):951-60.  
  
Fast and accurate automatic structure prediction with HHpred.  
Hildebrand A, Remmert M, Biegert A, Söding J. Proteins. 2009;77 Suppl 9:128-32.  
  
Automatic Prediction of Protein 3D Structures by Probabilistic Multi-template Homology Modeling.  
Meier A, Söding J. PLoS Comput Biol. 2015 Oct 23;11(10):e1004343.

- Help
- FAQ
- Privacy Policy
- Imprint
- Contact Us
- Cite Us
- Recent Updates

© 2008-2020, Dept. of Protein Evolution, Max Planck Institute for Developmental Biology, Tübingen

Template 3D Structure: 
×

Loading...
