# Supplementary material for: Conserved sequence motifs in human TMTC1, TMTC2, TMTC3, and TMTC4, new O-mannosyltransferases from the GT-C/PMT clan, are rationalized as ligand binding sites
Source: Biol Direct. 2021 Jan 12;16:4. doi: 10.1186/s13062-021-00291-w (PMC7801869; doi:10.1186/s13062-021-00291-w)
Supplement: Supplementary file 3 — Additional file 3. HHPred outputs when searching TMTCs against Pfam or PDB structures. The compressed library file AF3-2020-06-HHPred-TMTCs.zip contains the outputs when running the four human TMTC sequences as input of HHPred against PDB sequences and against Pfam domains (as of 23rd of June 2020). [file 13062_2021_291_MOESM3_ESM.zip › AF3-2020-06-HHPred-TMTCs/HHpred_TMTC2_PDB.html]

(\*) HHpred | Bioinformatics Toolkit          **We're sorry but the Toolkit doesn't work properly without JavaScript enabled. Please enable it to continue.**

Sign In

- Search
- Alignment
- Sequence Analysis
- 2ary Structure
- 3ary Structure
- Classification
- Utils

- HHblits
- HHpred
- HMMER
- PatternSearch
- ProtBLAST/PSI-BLAST

Nothing found.

###### Tools

###### Jobs

ID

Date

Tool

3084060HHPR1780993HHPR5863267HHPR5407837HHPR8665047HHPR2161064HHPR

# HHpred

Job ID: 3084060,Created: 10 minutes ago

- Input
- Parameters
- Results
- Raw Output
- Probability Plot
- Query Template MSA
- Query MSA

>sp|Q8N394|TMTC2\_HUMAN Protein 1..475
MIAELVSSALGLALYLNTLSADFCYDDSRAIKTNQDLLPETPWTHIFYNDFWGTLLTHSG
SHKSYRPLCTLSFRLNHAIGGLNPWSYHLVNVLLHAAVTGLFTSFSKILLGDGYWTFMAG
LMFASHPIHTEAVAGIVGRADVGASLFFLLSLLCYIKHCSTRGYSARTWGWFLGSGLCAG
CSMLWKEQGVTVLAVSAVYDVFVFHRLKIKQILPTIYKRKNLSLFLSISLLIFWGSSLLG
ARLYWMGNKPPSFSNSDNPAADSDSLLTRTLTFFYLPTKNLWLLLCPDTLSFDWSMDAVP
LLKTVCDWRNLHTVAFYTGLLLLAYYGLKSPSVDRECNGKTVTNGKQNANGHSCLSDVEY
QNSETKSSFASKVENGIKNDVSQRTQLPSTENIVVLSLSLLIIPFVPATNLFFYVGFVIA
ERVLYIPSMGFCLLITVGARALYVKVQKRFLKSLIFYATATLIVFYGLKTAIRNG

Paste ExampleUpload File

Protein FASTA

Align two sequences/MSAs

Select structural/domain databases

PDB\_mmCIF70\_29\_May

- PDB\_mmCIF70\_29\_May (default)
- PDB\_mmCIF30\_29\_May
- SCOPe70\_2.07
- ECOD\_ECOD\_F70\_20200207
- COG\_KOG\_v1.0
- Pfam-A\_v33.1
- NCBI\_Conserved\_Domains(CD)\_v3.18
- SMART\_v6.0
- TIGRFAMs\_v15.0
- PRK\_v6.9
- No elements found. Consider changing the search query.
- List is empty.

Select proteomes

Select options

- Euk\_Arabidopsis\_thaliana\_TAIR10\_20\_Jun\_2017
- Euk\_Bombyx\_mori\_p50T\_Dazao\_06\_May\_2019
- Euk\_Brachypodium\_distachyon\_23\_Aug\_2017
- Euk\_Caenorhabditis\_elegans\_18\_Jul\_2017
- Euk\_Capsaspora\_owczarzaki\_ATCC\_30864\_23\_Mar\_2020
- Euk\_Chaetomium\_thermophilum\_29\_Jun\_2017
- Euk\_Chlamydomonas\_reinhardtii\_27\_Jul\_2017
- Euk\_Entamoeba\_histolytica\_HM1\_IMSS\_22\_Mar\_2017
- Euk\_Dictyostelium\_discoideum\_AX4\_19\_Sep\_2017
- Euk\_Drosophila\_melanogaster\_19\_Jul\_2017
- Euk\_Giardia\_lamblia\_ATCC\_50803\_31\_Aug\_2017
- Euk\_Homo\_sapiens\_04\_Jul\_2017
- Euk\_Physcomitrella\_patens\_28\_Aug\_2017
- Euk\_Plasmodium\_falciparum\_3D7\_7\_Jun\_2017
- Euk\_Saccharomyces\_cerevisiae\_S288c\_11\_Mar\_2017
- Euk\_Schizosaccharomyces\_pombe\_19\_Sep\_2017
- Euk\_Solanum\_lycopersicum\_28\_Jul\_2019
- Euk\_Tetrahymena\_thermophila\_SB210\_22\_Aug\_2017
- Euk\_Toxoplasma\_gondii\_ME49\_10\_May\_2018
- Euk\_Trichomonas\_vaginalis\_G3\_21\_Nov\_2018
- Euk\_Trypanosoma\_brucei\_gambiense\_DAL972\_28\_Mar\_2017
- Euk\_Ustilago\_maydis\_521\_29\_May\_2017
- Euk\_Paramecium\_tetraurelia\_9\_Dec\_2018
- Arc\_Archaeoglobus\_fulgidus\_DSM\_4304\_5\_Dec\_2017
- Arc\_Halobacterium\_jilantaiense\_5\_Dec\_2017
- Arc\_Lokiarchaeum\_sp\_GC14\_75\_31\_Oct\_2018
- Arc\_Methanocaldococcus\_jannaschii\_DSM\_2661\_5\_Dec\_2017
- Arc\_Methanosarcina\_mazei\_S\_6\_17\_Mar\_2017
- Arc\_Methanothermus\_fervidus\_DSM\_2088\_5\_Dec\_2017
- Arc\_Pyrococcus\_horikoshii\_OT3\_5\_Dec\_2017
- Arc\_Sulfolobus\_solfataricus\_5\_Dec\_2017
- Arc\_Thermoplasma\_acidophilum\_DSM\_1728\_7\_Dec\_2017
- Bac\_Acinetobacter\_baumannii\_29\_Mar\_2018
- Bac\_Aquifex\_aeolicus\_VF5\_19\_Sep\_2017
- Bac\_Bacillus\_subtilis\_subsp\_subtilis\_str168\_19\_Mar\_2017
- Bac\_Bacteriovorax\_sp\_DB6\_IX\_1\_Jun\_2018
- Bac\_Bdellovibrio\_bacteriovorus\_HD100\_1\_Jun\_2018
- Bac\_Christensenella\_minuta\_2\_Apr\_2019
- Bac\_Deinococcus\_radiodurans\_R1\_19\_Sep\_2017
- Bac\_Enterococcus\_faecalis\_13\_SD\_W\_01\_1\_Jun\_2018
- Bac\_Escherichia\_coli\_K12\_07\_Mar\_2017
- Bac\_Fischerella\_muscicola\_PCC\_7414\_24\_Sep\_2017
- Bac\_Frankia\_alni\_ACN14a\_24\_Sep\_2017
- Bac\_Helicobacter\_pylori\_26695\_1\_Jun\_2018
- Bac\_Leptospira\_interrogans\_serovar\_Lai\_str56601\_1\_Jun\_2018
- Bac\_Mycobacterium\_tuberculosis\_H37Rv\_27\_May\_2017
- Bac\_Neisseria\_gonorrhoeae\_FA\_1090\_1\_Jun\_2018
- Bac\_Neisseria\_meningitidis\_MC58\_9\_Jun\_2017
- Bac\_Nostoc\_punctiforme\_PCC\_73102\_18\_Mar\_2017
- Bac\_Phycisphaerae\_bacterium\_L21\_RPulD3\_1\_Jun\_2018
- Bac\_Plesiocystis\_pacifica\_SIR1\_1\_Jun\_2018
- Bac\_Pseudomonas\_aeruginosa\_PAO1\_5\_Jun\_2017
- Bac\_Salmonella\_ent\_ser\_Typhi\_CT18\_22\_Nov\_2018
- Bac\_Staphylococcus\_aureus\_subsp\_aureus\_NCTC\_8325\_13\_Jun\_2017
- Bac\_Streptomyces\_scabiei\_87.22\_24\_Sep\_2017
- Bac\_Synechocystis\_sp\_PCC\_6803\_6\_Jun\_2017
- Bac\_Tenacibaculum\_dicentrarchi\_27\_Nov\_2017
- Bac\_Tenacibaculum\_maritimum\_NBRC\_15946\_27\_Nov\_2017
- Bac\_Thermus\_aquaticus\_Y51MC23\_24\_Sep\_2017
- Bac\_Thermus\_thermophilus\_HB8\_19\_Sep\_2017
- Bac\_Waddlia\_chondrophila\_WSU\_86\_1044\_1\_Jun\_2018
- Bac\_Yersinia\_pestis\_CO92\_10\_Apr\_2017
- Vir\_SARS-CoV-2\_31\_Mar\_2020
- No elements found. Consider changing the search query.
- List is empty.

Resubmit

MSA generation method

HHblits=>UniRef30

- HHblits=>UniRef30 (default)
- PSI-BLAST=>nr70
- No elements found. Consider changing the search query.
- List is empty.

Maximal no. of MSA generation steps

3

- 0
- 1
- 2
- 3 (default)
- 4
- 5
- 8
- No elements found. Consider changing the search query.
- List is empty.

E-value incl. threshold for MSA generation

1e-3

- 0.1
- 0.05
- 0.02
- 0.01
- 1e-3 (default)
- 1e-6
- 1e-8
- 1e-10
- 1e-15
- 1e-20
- 1e-30
- 1e-40
- 1e-50
- No elements found. Consider changing the search query.
- List is empty.

Min. seq. identity of MSA hits with query (%)

0

- 0 (default)
- 10
- 20
- 30
- 40
- 50
- 60
- 70
- 75
- 80
- 85
- 90
- 95
- 100
- No elements found. Consider changing the search query.
- List is empty.

Min. coverage of MSA hits (%)

20

- 10
- 20 (default)
- 30
- 40
- 50
- 60
- 70
- 80
- 90
- 100
- No elements found. Consider changing the search query.
- List is empty.

Secondary structure scoring

during\_alignment

- none
- after\_alignment
- during\_alignment (default)
- after\_alignment\_pred\_vs\_pred
- during\_alignment\_pred\_vs\_pred
- No elements found. Consider changing the search query.
- List is empty.

Alignment Mode:Realign with MAC

local:norealign

- local:norealign (default)
- local:realign
- global:realign
- No elements found. Consider changing the search query.
- List is empty.

MAC realignment threshold

0.3

- 0.0
- 0.01
- 0.1
- 0.2
- 0.3 (default)
- 0.4
- 0.5
- 0.6
- 0.7
- 0.8
- 0.9
- 0.95
- No elements found. Consider changing the search query.
- List is empty.

No. of target sequences (up to 10000)

250

- 250 (default)
- 500
- 1000
- 2000
- 3000
- 4000
- 5000
- 6000
- 7000
- 8000
- 9000
- 10000
- No elements found. Consider changing the search query.
- List is empty.

Min. probability in hit list (> 10%)

20

- 10
- 20 (default)
- 30
- 40
- 50
- 60
- 70
- 75
- 80
- 85
- 90
- 95
- 100
- No elements found. Consider changing the search query.
- List is empty.

Resubmit

VisHitsAln
Select AllForwardForward Query A3MModel using selectionDownload HHRColor SeqsWrap Seqs

Number of Hits: **15**

Detected sequence features:
**◾Transmembrane segment(s)****◾Signal peptide**

#### Visualization

Resubmit Section

1

475

#### Hitlist

Show102550100AllEntries

Search:

| Nr (Click to sort Ascending) | Hit (Click to sort Ascending) | Name (Click to sort Ascending) | Probability (Click to sort Ascending) | E-value (Click to sort Ascending) | SS (Click to sort Ascending) | Cols (Click to sort Ascending) | Target Length (Click to sort Ascending) |
| --- | --- | --- | --- | --- | --- | --- | --- |
| 1 | 5EZM\_A | 4-amino-4-deoxy-L-arabinose (L-Ara4N) transferase; membrane protein, lipid glycosyltransferase, zinc; HET: PC, DSL, PO4, MPG, EPE; 2.7A {Cupriavidus metallidurans (strain ATCC 43123 / DSM 2839 / NBRC 102507 / CH34)}; Related PDB entries: 5F15\_A ; Related PDB entries: 5F15\_A ; Related PDB entries: 5F15\_A | 99.87 | 5.9e-19 | 35.2 | 368 | 578 |
| 2 | 6S7T\_A | Dolichyl-diphosphooligosaccharide--protein glycosyltransferase subunit STT3B (E.C.2.4.99.18); N-glycosylation, Oligosaccharyltransferase, OSTB, TRANSFERASE; HET: 0K3, KZB, NAG, EGY, MAN, BMA; 3.5A {Homo sapiens} | 99.78 | 2.3e-15 | 39.8 | 371 | 826 |
| 3 | 6EZN\_F | Dolichyl-diphosphooligosaccharide--protein glycosyltransferase subunit 1 (E.C.2.4.99.18); OST complex, oligosaccharyltransferase, N-linked glycosylation; HET: PTY, BMA, CPL, MAN, NAG;{Saccharomyces cerevisiae (strain ATCC 204508 / S288c)}; Related PDB entries: 6C26\_A; Related PDB entries: 6C26\_A; Related PDB entries: 6C26\_A | 99.78 | 3e-15 | 38.2 | 378 | 718 |
| 4 | 6S7O\_A | Dolichyl-diphosphooligosaccharide--protein glycosyltransferase subunit STT3A (E.C.2.4.99.18); N-glycosylation, Oligosaccharyltransferase, OSTA, TRANSFERASE; HET: KZB, NAG, EGY, MAN, KZE, BMA; 3.5A {Homo sapiens}; Related PDB entries: 6FTI\_5 6FTG\_5 6FTJ\_5; Related PDB entries: 6FTG\_5 6FTI\_5 6FTJ\_5; Related PDB entries: 6FTG\_5 6FTI\_5 6FTJ\_5 | 99.77 | 4.9e-15 | 37.8 | 374 | 705 |
| 5 | 3WAJ\_A | Transmembrane oligosaccharyl transferase (E.C.2.4.1.119); oligosaccharyltransferase, N-glycosylation, Archaeoglobus fulgidus, GT-C; 2.501A {Archaeoglobus fulgidus}; Related PDB entries: 5GMY\_A 3WAK\_A; Related PDB entries: 5GMY\_A 3WAK\_A; Related PDB entries: 5GMY\_A 3WAK\_A | 99.74 | 8.6e-15 | 35.9 | 389 | 875 |
| 6 | 5OGL\_A | Peptide-binding protein, Substrate mimicking peptide; Oligosaccharyltransferase, Complex, Protein N-glycosylation, Bacteria; HET: 9UB, PPN; 2.7A {Campylobacter lari (strain RM2100 / D67 / ATCC BAA-1060)}; Related PDB entries: 3RCE\_A 6GXC\_A; Related PDB entries: 6GXC\_A 3RCE\_A ; Related PDB entries: 6GXC\_A 3RCE\_A | 99.72 | 5.7e-14 | 37.7 | 359 | 713 |
| 7 | 6P25\_A | Dolichyl-diphosphooligosaccharide--protein glycosyltransferase subunits (E.C.2.4.99.18); complex, TRANSFERASE, glycosylation; HET: NAG, CPL, NNM; 3.2A {Saccharomyces cerevisiae W303}; Related PDB entries: 6P2R\_A ; Related PDB entries: 6P2R\_A ; Related PDB entries: 6P2R\_A | 99.68 | 7.8e-13 | 39.9 | 394 | 817 |
| 8 | 6P25\_B | Dolichyl-diphosphooligosaccharide--protein glycosyltransferase subunits (E.C.2.4.99.18); complex, TRANSFERASE, glycosylation; HET: NAG, CPL, NNM; 3.2A {Saccharomyces cerevisiae W303}; Related PDB entries: 6P2R\_B ; Related PDB entries: 6P2R\_B ; Related PDB entries: 6P2R\_B | 99.66 | 5.8e-12 | 42.8 | 383 | 759 |
| 9 | 7BVF\_A | Probable arabinosyltransferase B (E.C.2.4.2.-), Probable; Mycobacterium tuberculosis, cell wall synthesis; HET: 95E, DSL, CDL;{Mycolicibacterium smegmatis MC2 155} | 99.52 | 1.2e-10 | 36 | 356 | 1102 |
| 10 | 7BWR\_A | Integral membrane indolylacetylinositol arabinosyltransferase EmbB; Mycobacterium tuberculosis, EmbB, cryo-EM, ethambutol; HET: F8L;{Mycolicibacterium smegmatis MC2 155}; Related PDB entries: 7BVC\_B 7BVG\_B 7BWR\_B 7BX8\_B 7BX8\_A | 99.49 | 2e-10 | 34.8 | 351 | 1082 |
| 11 | 7BVF\_B | Probable arabinosyltransferase B (E.C.2.4.2.-), Probable; Mycobacterium tuberculosis, cell wall synthesis; HET: 95E, DSL, CDL;{Mycolicibacterium smegmatis MC2 155} | 99.47 | 2.6e-10 | 34.4 | 354 | 1116 |
| 12 | 6W98\_A | F5/8 type C domain-containing protein; Glycosyltransferase, lipomannan, lipoarabinomannan, arabinofuranose, membrane; HET: PNS, 6OU; 2.9A {Escherichia coli (strain K12)}; Related PDB entries: 6WBX\_A 6WBY\_A | 99.41 | 6.7e-10 | 34.1 | 379 | 1413 |
| 13 | 7BVE\_B | Integral membrane indolylacetylinositol arabinosyltransferase EmbC; Mycobacterium smegmatis, cell wall synthesis; HET: PO4, PN7, 95E; 2.81A {Mycolicibacterium smegmatis MC2 155}; Related PDB entries: 7BVH\_B 7BVH\_A 7BVE\_A | 99.38 | 4.3e-9 | 35.6 | 351 | 1084 |
| 14 | 6SNI\_X | Dolichyl pyrophosphate Man9GlcNAc2 alpha-1,3-glucosyltransferase (E.C.2.4.1.267); Glycosyltransferase, Glucosyltransferase, GT-C, N-Glycosylation, MEMBRANE; HET: PTY, Y01;{Saccharomyces cerevisiae}; Related PDB entries: 6SNH\_X | 99.28 | 1.3e-8 | 31.4 | 321 | 562 |
| 15 | 7BVC\_A | Integral membrane indolylacetylinositol arabinosyltransferase EmbA; Mycobacterium smegmatis, cell wall synthesis; HET: 95E, PNS, CDL, F8L;{Mycolicibacterium smegmatis MC2 155}; Related PDB entries: 7BVG\_A | 99.27 | 2.2e-8 | 33.3 | 348 | 1088 |

Displaying 1 to 15 of 15 hits

- «
- ‹
- 1
- ›
- »

#### Alignments

|  |  |  |  |
| --- | --- | --- | --- |
|  | | | |
|  | Template alignmentTemplate 3D StructurePDBe | | |
| 1. | 5EZM\_A 4-amino-4-deoxy-L-arabinose (L-Ara4N) transferase; membrane protein, lipid glycosyltransferase, zinc; HET: PC, DSL, PO4, MPG, EPE; 2.7A {Cupriavidus metallidurans (strain ATCC 43123 / DSM 2839 / NBRC 102507 / CH34)}; Related PDB entries: 5F15\_A ; Related PDB entries: 5F15\_A ; Related PDB entries: 5F15\_A | | |
|  | Probability: 99.87%, E-value: 5.9e-19, Score: 180.62, Aligned cols: 368, Identities: 13%, Similarity: 0.044, | | |
|  |
|  | Q ss\_pred |  | CHHHHHHHHHHHHHHHhhcCCCCcccchhHHHhchhhCCCCchhHhhhcccccccccCCCCcccCChHHHHHHHHHHHHh |
|  | Q Q8N394 | 1 | MIAELVSSALGLALYLNTLSADFCYDDSRAIKTNQDLLPETPWTHIFYNDFWGTLLTHSGSHKSYRPLCTLSFRLNHAIG   80 (475) |
|  | Q Consensus | 1 | ~~~~lll~~~~~~~~~~~~~~~~~~De~~~~~~~~~~~~~~~~~~~~~~~~~~~~~~~~~~~~~~~Pl~~~l~~~~~~lf   80 (475) |
|  |  |  | ++.+++++++............+.+||..+...++++.+++++......+ .....+||++.++.+..+.++ |
|  | T Consensus | 33 | ~~~~~~~~~~~~~~~~~~~~~~~~~De~~~~~~a~~~~~~~~~~~~~~~~---------~~~~~~pPl~~~l~~~~~~l~   103 (578) |
|  | T 5EZM\_A | 33 | WVVLFVAVALVVWFVSLDMRHLVGPDEGRYAEISREMFASGDWVTIRYNA---------LKYFEKPPFHMWVTVVGYELF   103 (578) |
|  | T ss\_dssp |  | HHHHHHHHHHHHHHHGGGSSCCCTTHHHHHHHHHHHHHHHCCSSSCEETT---------EECCSSCSHHHHHHHHHHHHH |
|  | T ss\_pred |  | HHHHHHHHHHHHHHhccccccCCCCCHHHHHHHHHHHHHhCCceeEEECC---------EeCCCCCHHHHHHHHHHHHHH |
|  |
|  |
|  | Q ss\_pred |  | CCChHHHHHHHHHHHHHHHHHHHHHHHHHhCChHHHHHHHHHHHHCHHhHHHHHhhhchHHHHHHHHHHHHHHHHHHHHc |
|  | Q Q8N394 | 81 | GLNPWSYHLVNVLLHAAVTGLFTSFSKILLGDGYWTFMAGLMFASHPIHTEAVAGIVGRADVGASLFFLLSLLCYIKHCS   160 (475) |
|  | Q Consensus | 81 | G~~~~~~rl~~~l~~~l~~~lly~l~r~l~~~~~~al~aall~a~~P~~~~~~~~~~~~~~~~~~~f~ll~l~~~l~~~~   160 (475) |
|  |  |  | |.+....|+.++++++++++++|.++|++++ +..+++++++++++|....++..... |.+..++.+++++++.+..+ |
|  | T Consensus | 104 | g~~~~~~rl~~~l~~~l~~~~~~~l~~~~~~-~~~a~~a~~l~~~~p~~~~~~~~~~~--~~~~~~~~~~~~~~~~~~~~   180 (578) |
|  | T 5EZM\_A | 104 | GLGEWQARLAVALSGLLGIGVSMMAARRWFG-ARAAAFTGLALLAAPMWSVAAHFNTL--DMTLAGVMSCVLAFMLMGQH   180 (578) |
|  | T ss\_dssp |  | CSSHHHHTHHHHHHHHHHHHHHHHHHHHHHC-HHHHHHHHHHHHHCHHHHHHHTSCCH--HHHHHHHHHHHHHHHHHHTC |
|  | T ss\_pred |  | CcCHHHHHHHHHHHHHHHHHHHHHHHHHHhC-hHHHHHHHHHHHHHHHHHHHHhhHhh--HHHHHHHHHHHHHHHHHHhC |
|  |
|  |
|  | Q ss\_pred |  | cCCCCcchHHHHHHHHHHHHHHHHhHhHHHHHHHHHHHHHHHHHcccchhhhcchHhhhHHHHHHHHHHHHHHHHHHHHH |
|  | Q Q8N394 | 161 | TRGYSARTWGWFLGSGLCAGCSMLWKEQGVTVLAVSAVYDVFVFHRLKIKQILPTIYKRKNLSLFLSISLLIFWGSSLLG   240 (475) |
|  | Q Consensus | 161 | ~~~~~~~~~~~~~l~~l~~~la~ltk~~~~~~~~~~~~~~l~~~~~~~~~~~~~~~~~~~~~~~~~~~~~~~~~~~~~~~   240 (475) |
|  |  |  | +++++++++++.++++++.+++.++|+....+.+...+......++++++ .+.........+.++....+.. |
|  | T Consensus | 181 | ~~~~~~~~~~~~~l~g~~~gla~~~k~~~~~~~~~~~~~~~~~~~~~~~~--------~~~~~~~~~~~~~~~~~~~~~~   252 (578) |
|  | T 5EZM\_A | 181 | PDASVAARRGWMVACWAAMGVAILTKGLVGIALPGLVLVVYTLVTRDWGL--------WRRLHLALGVVVMLVITVPWFY   252 (578) |
|  | T ss\_dssp |  | TTSCHHHHHHHHHHHHHHHHHHHHHHTTHHHHHHHHHHHHHHHHSCCTTH--------HHHTCHHHHHHHHHHHHHHHHH |
|  | T ss\_pred |  | CCcchhhccHHHHHHHHHHHHHHHccchHHHHHHHHHHHHHHHHcCChHH--------HhhcchHHHHHHHHHHHHHHHH |
|  |
|  |
|  | Q ss\_pred |  | HHHHHhcCCCCCccCCCCCCcCCChHHHHHHHHHHHHHHhHHHhhCcccccccccccccccccccCCHHHHHHHHHHHHH |
|  | Q Q8N394 | 241 | ARLYWMGNKPPSFSNSDNPAADSDSLLTRTLTFFYLPTKNLWLLLCPDTLSFDWSMDAVPLLKTVCDWRNLHTVAFYTGL   320 (475) |
|  | Q Consensus | 241 | ~~~~~~~~~~~~~~~~~~~~~~~~~~~~~~~~~~~~~~~~~~~~~~p~~~~~~~~~~~~~~~~~~~~~~~~~~~~~~~~~   320 (475) |
|  |  |  | ........ ...........................+. ............+ |
|  | T Consensus | 253 | ~~~~~~~~------------------~~~~~~~~~~~~~~~~~~~~~~~~~~~~~------------~~~~~~~~~~~~~   302 (578) |
|  | T 5EZM\_A | 253 | LVSVRNPE------------------FPNFFFIHEHWQRYTSNIHSRSGSVFYFL------------PLVIGGFLPWAGI   302 (578) |
|  | T ss\_dssp |  | HHHHHCTT------------------HHHHHHHCCCCCC-------CCCCTTTHH------------HHHHHHTGGGGGG |
|  | T ss\_pred |  | HHHHHCCc------------------chhhhHHHHHHHHHhcCcccCCCChHHHH------------HHHHHHhhhHHhH |
|  |
|  |
|  | Q ss\_pred |  | HHHHHHHhhCcccccccCCCcccCCccccCCcccccccccccccccccccchhhcCCcCCcccccCCCCCCchHHhhhHH |
|  | Q Q8N394 | 321 | LLLAYYGLKSPSVDRECNGKTVTNGKQNANGHSCLSDVEYQNSETKSSFASKVENGIKNDVSQRTQLPSTENIVVLSLSL   400 (475) |
|  | Q Consensus | 321 | ~~~~~~~~~~~~~~~~~~~~~~~~~~~~~~~~~~~~~~~~~~~~~~~~~~~~~~~~~~~~~~~~~~~~~~~~~~~~~l~~   400 (475) |
|  |  |  | +.......++ .++++..........+.+ |
|  | T Consensus | 303 | ~~~~~~~~~~----------------------------------------------------~~~~~~~~~~~~~~~~~~   330 (578) |
|  | T 5EZM\_A | 303 | FPKLWTAMRA----------------------------------------------------PVEGTQARFRPALMAGIW   330 (578) |
|  | T ss\_dssp |  | HHHHHHHHCC-----------------------------------------------------------CCCHHHHHHHH |
|  | T ss\_pred |  | HHHHHHHhhc----------------------------------------------------ccccCcccccHHHHHHHH |
|  |
|  |
|  | Q ss\_pred |  | HHHHHhhhcCccccCChhhHHHHhHHHHHHHHHHHHHHHHHHHHHhchHHHHHHHHHHHHHHHHHHHHHHHHhcC |
|  | Q Q8N394 | 401 | LIIPFVPATNLFFYVGFVIAERVLYIPSMGFCLLITVGARALYVKVQKRFLKSLIFYATATLIVFYGLKTAIRNG   475 (475) |
|  | Q Consensus | 401 | ~~~~~~~~~~~~~~~~~~~~~Ry~~~~~~~~~ll~~~~l~~~~~~~~~~~~~~~~~~~~~~~~~~~~~~~~~~~~   475 (475) |
|  |  |  | ++..++..... ....+||.++..|+++++++.++.++.++.+++........++++++............ |
|  | T Consensus | 331 | ~~~~~~~~~~~-----~~~~~ry~~~~~p~l~ll~~~~l~~~~~~~~~~~~~~~~~~~~~~~~~~~~~~~~~~~~   400 (578) |
|  | T 5EZM\_A | 331 | AIAIFVFFSIS-----RSKLPGYIVPVIPALGILAGVALDRLSPRSWGKQLIGMAIVAACGLLASPVVATLNANH   400 (578) |
|  | T ss\_dssp |  | HHHHHHHHHTS-----SSCCGGGGTTHHHHHHHHHHHHHHTCCHHHHHHHHHHHHHHHHHHHHHGGGGGGCCCTT |
|  | T ss\_pred |  | HHHHHHHHHHh-----cccChhcHHhHHHHHHHHHHHHHHHhCCCccHHHHHHHHHHHHHHHHHHHHHHHhhhcC |
|  |
| --- | | | |
|  | Template alignmentTemplate 3D StructurePDBe | | |
| 2. | 6S7T\_A Dolichyl-diphosphooligosaccharide--protein glycosyltransferase subunit STT3B (E.C.2.4.99.18); N-glycosylation, Oligosaccharyltransferase, OSTB, TRANSFERASE; HET: 0K3, KZB, NAG, EGY, MAN, BMA; 3.5A {Homo sapiens} | | |
|  | Probability: 99.78%, E-value: 2.3e-15, Score: 161.14, Aligned cols: 371, Identities: 9%, Similarity: -0.021, | | |
|  |
|  | Q ss\_pred |  | HHHHHHHHHHHHHHHhh-cCCCCc---ccchhHHHhchhhCCCCchhHhhhcccccccccCCCCcccCChHHHHHHHHHH |
|  | Q Q8N394 | 2 | IAELVSSALGLALYLNT-LSADFC---YDDSRAIKTNQDLLPETPWTHIFYNDFWGTLLTHSGSHKSYRPLCTLSFRLNH   77 (475) |
|  | Q Consensus | 2 | ~~~lll~~~~~~~~~~~-~~~~~~---~De~~~~~~~~~~~~~~~~~~~~~~~~~~~~~~~~~~~~~~~Pl~~~l~~~~~   77 (475) |
|  |  |  | ++++++++++....... ...+.. .||..+...++.+.+++........+..............++|++.++.+..+ |
|  | T Consensus | 75 | ~~l~~i~~~~~~~rl~~~~~~~~~~~~~D~~~~~~~a~~~~~~g~~~~~~~~~~~~~~p~g~~~~~~~~P~~~~l~a~~~   154 (826) |
|  | T 6S7T\_A | 75 | TILFLAWLAGFSSRLFAVIRFESIIHEFDPWFNYRSTHHLASHGFYEFLNWFDERAWYPLGRIVGGTVYPGLMITAGLIH   154 (826) |
|  | T ss\_dssp |  | HHHHHHHHHHHHTTCTTTTTTCSCCCSTTHHHHHHHHHHHHHHCHHHHHTCEECSSSTTTCEESTTSSCTTHHHHHHHHH |
|  | T ss\_pred |  | HHHHHHHHHHHHHHHHHHHhhhhhhcCCChHHHHHHHHHHHHhCcHHHhccccchhcCCCCCCCCCCCchHHHHHHHHHH |
|  |
|  |
|  | Q ss\_pred |  | HHhC------CChHHHHHHHHHHHHHHHHHHHHHHHHHhCChHHHHHHHHHHHHCHHhHHHHHhhhchHHHHHHHHHHHH |
|  | Q Q8N394 | 78 | AIGG------LNPWSYHLVNVLLHAAVTGLFTSFSKILLGDGYWTFMAGLMFASHPIHTEAVAGIVGRADVGASLFFLLS   151 (475) |
|  | Q Consensus | 78 | ~lfG------~~~~~~rl~~~l~~~l~~~lly~l~r~l~~~~~~al~aall~a~~P~~~~~~~~~~~~~~~~~~~f~ll~   151 (475) |
|  |  |  | .++| ......|++++++++++++++|.++|++.+ +..|++++++++++|.+...+.....++|.+..++.+++ |
|  | T Consensus | 155 | ~l~g~~~~~~~~~~~~~l~~~l~~~l~~~~~y~l~r~l~~-~~~allaall~a~~p~~~~~s~~~~~~~e~~~~~~~~l~   233 (826) |
|  | T 6S7T\_A | 155 | WILNTLNITVHIRDVCVFLAPTFSGLTSISTFLLTRELWN-QGAGLLAACFIAIVPGYISRSVAGSFDNEGIAIFALQFT   233 (826) |
|  | T ss\_dssp |  | HHHHHTTCCCCHHHHHHTHHHHHHHHHHHHHHHHHHHHSC-HHHHHHHHHHTTTCHHHHGGGSTTCCCSHHHHHHHHHHH |
|  | T ss\_pred |  | HHHHhcCCCCcHHHHHHHHHHHHHHHHHHHHHHHHHHHcC-chHHHHHHHHHHHHHHHHHhhhccCchHHHHHHHHHHHH |
|  |
|  |
|  | Q ss\_pred |  | HHHHHHHHccCCCCcchHHHHHHHHHHHHHHHHhHhHHHHHHHHHHHHHHHHHcccchhhhcchHhhh------------ |
|  | Q Q8N394 | 152 | LLCYIKHCSTRGYSARTWGWFLGSGLCAGCSMLWKEQGVTVLAVSAVYDVFVFHRLKIKQILPTIYKR------------   219 (475) |
|  | Q Consensus | 152 | l~~~l~~~~~~~~~~~~~~~~~l~~l~~~la~ltk~~~~~~~~~~~~~~l~~~~~~~~~~~~~~~~~~------------   219 (475) |
|  |  |  | ++++.+..+++ +.++.++++++++++.++|+.+.++++++++..++....++.+. + |
|  | T Consensus | 234 | l~~~~~~~~~~-----~~~~~~l~gl~~gla~~~~~~~~~~~~~~~l~~~~~~~~~~~~~-------~~~~~~~~~~~~~   301 (826) |
|  | T 6S7T\_A | 234 | YYLWVKSVKTG-----SVFWTMCCCLSYFYMVSAWGGYVFIINLIPLHVFVLLLMQRYSK-------RVYIAYSTFYIVG   301 (826) |
|  | T ss\_dssp |  | HHHHHHHHHHC-----CHHHHHHHHHHHHHHHHHCTTHHHHHHHHHHHHHHHHHTTCCCH-------HHHHHHHHHHHHH |
|  | T ss\_pred |  | HHHHHHHhccC-----cHHHHHHHHHHHHHHHHhcccHHHHHHHHHHHHHHHHHhccCCh-------hHHHHHHHHHHHH |
|  |
|  |
|  | Q ss\_pred |  | ---------------------------------------------HHHHHHHHHHHHHHHHHHHHHHHHHHhcCCCCCcc |
|  | Q Q8N394 | 220 | ---------------------------------------------KNLSLFLSISLLIFWGSSLLGARLYWMGNKPPSFS   254 (475) |
|  | Q Consensus | 220 | ---------------------------------------------~~~~~~~~~~~~~~~~~~~~~~~~~~~~~~~~~~~   254 (475) |
|  |  |  | +................................ |
|  | T Consensus | 302 | ~~~~~~~p~~~~~~~~~~~~~~~~~~~~~~~~~~~~~~~~~~~~~~~~~~~~~~~~~~~~~~~~~~~~~~~~~~~~~~--   379 (826) |
|  | T 6S7T\_A | 302 | LILSMQIPFVGFQPIRTSEHMAAAGVFALLQAYAFLQYLRDRLTKQEFQTLFFLGVSLAAGAVFLSVIYLTYTGYIAP--   379 (826) |
|  | T ss\_dssp |  | HHHHTTSTTTTTHHHHBSSTHHHHHHHHHHHHHHHHHHHHHHSCSTTTHHHHHHHHHHHHHHHHHHHHHHHHHTSSBC-- |
|  | T ss\_pred |  | HHHHhccccCCCCcccchHHHHHHHHHHHHHHHHHHHHHHHhcCHHHHHHHHHHHHHHHHHHHHHHHHHHHhcccccc-- |
|  |
|  |
|  | Q ss\_pred |  | CCCCCCcCCChHHHHHHHHHHHHHHhHHHhhCcccccccccccccccccccCCHHHHHHHHHHHHHHHHHHHHhhCcccc |
|  | Q Q8N394 | 255 | NSDNPAADSDSLLTRTLTFFYLPTKNLWLLLCPDTLSFDWSMDAVPLLKTVCDWRNLHTVAFYTGLLLLAYYGLKSPSVD   334 (475) |
|  | Q Consensus | 255 | ~~~~~~~~~~~~~~~~~~~~~~~~~~~~~~~~p~~~~~~~~~~~~~~~~~~~~~~~~~~~~~~~~~~~~~~~~~~~~~~~   334 (475) |
|  |  |  | ..........................................++++++++....+++ |
|  | T Consensus | 380 | -------------------~~~~~~~~~~~~~~~~~~~~~~~~~~~~~~~~~~~~~~~~~~~~~~~~~~~~~~~~~----   436 (826) |
|  | T 6S7T\_A | 380 | -------------------WSGRFYSLWDTGYAKIHIPIIASVSEHQPTTWVSFFFDLHILVCTFPAGLWFCIKNI----   436 (826) |
|  | T ss\_dssp |  | -------------------CCHHHHHHHHSSHHHHTCHHHHHBSTTSCCCHHHHHHSCSSHHHHHHHHHHHHHHSC---- |
|  | T ss\_pred |  | -------------------chHHHHHHhcccccccCccchhcccccCCCCHHHHHHHHHHHHHHHHHHHHHHHhcC---- |
|  |
|  |
|  | Q ss\_pred |  | cccCCCcccCCccccCCcccccccccccccccccccchhhcCCcCCcccccCCCCCCchHHhhhHHHHHHHhhhcCcccc |
|  | Q Q8N394 | 335 | RECNGKTVTNGKQNANGHSCLSDVEYQNSETKSSFASKVENGIKNDVSQRTQLPSTENIVVLSLSLLIIPFVPATNLFFY   414 (475) |
|  | Q Consensus | 335 | ~~~~~~~~~~~~~~~~~~~~~~~~~~~~~~~~~~~~~~~~~~~~~~~~~~~~~~~~~~~~~~~l~~~~~~~~~~~~~~~~   414 (475) |
|  |  |  | +....+.++++++.+.. |
|  | T Consensus | 437 | --------------------------------------------------------~~~~~~~~~~~~~~~~~-------   453 (826) |
|  | T 6S7T\_A | 437 | --------------------------------------------------------NDERVFVALYAISAVYF-------   453 (826) |
|  | T ss\_dssp |  | --------------------------------------------------------CHHHHHHHHHHHHHHHH------- |
|  | T ss\_pred |  | --------------------------------------------------------CHHHHHHHHHHHHHHHH------- |
|  |
|  |
|  | Q ss\_pred |  | CChhhHHHHhHHHHHHHHHHHHHHHHHHHHHhchH--------------------------------------------- |
|  | Q Q8N394 | 415 | VGFVIAERVLYIPSMGFCLLITVGARALYVKVQKR---------------------------------------------   449 (475) |
|  | Q Consensus | 415 | ~~~~~~~Ry~~~~~~~~~ll~~~~l~~~~~~~~~~---------------------------------------------   449 (475) |
|  |  |  | .....||.++..|+++++++.++..+.++..++ |
|  | T Consensus | 454 | --~~~~~Ry~~~~~p~~~ll~a~~l~~l~~~~~~~~~~~~~~~~~~~~~~~~~~~~~~~~~~~~~~~~~~~~~~~~~~~~   531 (826) |
|  | T 6S7T\_A | 454 | --AGVMVRLMLTLTPVVCMLSAIAFSNVFEHYLGDDMKRENPPVEDSSDEDDKRNQGNLYDKAGKVRKHATEQEKTEEGL   531 (826) |
|  | T ss\_dssp |  | --HTTCSTTHHHHHHHHHHHHHHHHHHHHHHTCC---------------------------------------------- |
|  | T ss\_pred |  | --HHHHHHHHHhHHHHHHHHHHHHHHHHHHHHhccccccCCCCCCCCCccchhhccccccccccccccccchhhhhhcCC |
|  |
|  |
|  | Q ss\_pred |  | --HHHHHHHHHHHHHHHHHHHHHHHhcC |
|  | Q Q8N394 | 450 | --FLKSLIFYATATLIVFYGLKTAIRNG   475 (475) |
|  | Q Consensus | 450 | --~~~~~~~~~~~~~~~~~~~~~~~~~~   475 (475) |
|  |  |  | ..+..+.++++++++........... |
|  | T Consensus | 532 | ~~~~~~~~~~~~~~~~~~~~~~~~~~~~   559 (826) |
|  | T 6S7T\_A | 532 | GPNIKSIVTMLMLMLLMMFAVHCTWVTS   559 (826) |
|  | T ss\_dssp |  | -CHHHHHHHHHHHHHHHHHHHHHHHHCC |
|  | T ss\_pred |  | ChHHHHHHHHHHHHHHHHHHHHHHHHhh |
|  |
| --- | | | |
|  | Template alignmentTemplate 3D StructurePDBe | | |
| 3. | 6EZN\_F Dolichyl-diphosphooligosaccharide--protein glycosyltransferase subunit 1 (E.C.2.4.99.18); OST complex, oligosaccharyltransferase, N-linked glycosylation; HET: PTY, BMA, CPL, MAN, NAG;{Saccharomyces cerevisiae (strain ATCC 204508 / S288c)}; Related PDB entries: 6C26\_A; Related PDB entries: 6C26\_A; Related PDB entries: 6C26\_A | | |
|  | Probability: 99.78%, E-value: 3e-15, Score: 157.35, Aligned cols: 378, Identities: 8%, Similarity: -0.075, | | |
|  |
|  | Q ss\_pred |  | CHHHHHHHHHHHHHHHhhcCCCC---cccchhHHHhchhhCCCCchhHhhhcccccccccCCCCcccCChHHHHHHHHHH |
|  | Q Q8N394 | 1 | MIAELVSSALGLALYLNTLSADF---CYDDSRAIKTNQDLLPETPWTHIFYNDFWGTLLTHSGSHKSYRPLCTLSFRLNH   77 (475) |
|  | Q Consensus | 1 | ~~~~lll~~~~~~~~~~~~~~~~---~~De~~~~~~~~~~~~~~~~~~~~~~~~~~~~~~~~~~~~~~~Pl~~~l~~~~~   77 (475) |
|  |  |  | .++++++++............+. ..||..+...++++.+++........+...............+|++.++.+..+ |
|  | T Consensus | 19 | ~~l~~~~~~~~~~~~~~~~~~~~~~~~~D~~~~~~~a~~~~~~g~~~~~~~~~~~~~~~~g~~~~~~~~p~~~~l~a~~~   98 (718) |
|  | T 6EZN\_F | 19 | VIFVAIFGAAISSRLFAVIKFESIIHEFDPWFNYRATKYLVNNSFYKFLNWFDDRTWYPLGRVTGGTLYPGLMTTSAFIW   98 (718) |
|  | T ss\_dssp |  | HHHHHHHHHHHHTTTTTTTTTCCCCCSSSHHHHHHHHHHHHHSCHHHHHSCCCTTSSTTTCCCSSSSCCTTHHHHHHHHH |
|  | T ss\_pred |  | HHHHHHHHHHHHHHHHHHHHchhhhcccChHHHHHHHHHHHHcccHHHhcccCCcccCCCCCCCCCCCChHHHHHHHHHH |
|  |
|  |
|  | Q ss\_pred |  | HH----hC---CChHHHHHHHHHHHHHHHHHHHHHHHHHhCChHHHHHHHHHHHHCHHhHHHHHhhhchHHHHHHHHHHH |
|  | Q Q8N394 | 78 | AI----GG---LNPWSYHLVNVLLHAAVTGLFTSFSKILLGDGYWTFMAGLMFASHPIHTEAVAGIVGRADVGASLFFLL   150 (475) |
|  | Q Consensus | 78 | ~l----fG---~~~~~~rl~~~l~~~l~~~lly~l~r~l~~~~~~al~aall~a~~P~~~~~~~~~~~~~~~~~~~f~ll   150 (475) |
|  |  |  | .+ +| ......|+.++++++++++++|.++|++++ +..|++++++++++|.+...+.....++|.+..++.++ |
|  | T Consensus | 99 | ~l~~~~~G~~~~~~~~~~~~~~l~~~l~~~~~y~l~~~l~~-~~~a~~aa~l~~~~p~~~~~~~~g~~~~~~~~~~~~~~   177 (718) |
|  | T 6EZN\_F | 99 | HALRNWLGLPIDIRNVCVLFAPLFSGVTAWATYEFTKEIKD-ASAGLLAAGFIAIVPGYISRSVAGSYDNEAIAITLLMV   177 (718) |
|  | T ss\_dssp |  | HCCCCCSSCCCCHHHHHHBTHHHHHHHHHHHHHHHHHHHSC-HHHHHHHHHHHHHCHHHHSSSCSBCCSSSTTTHHHHHH |
|  | T ss\_pred |  | HHHHHHhCCCCCHHHHHHHHHHHHHHHHHHHHHHHHHHhcC-hHHHHHHHHHHHHHHHHHHhHhccccchHHHHHHHHHH |
|  |
|  |
|  | Q ss\_pred |  | HHHHHHHHHccCCCCcchHHHHHHHHHHHHHHHHhHhHHHHHHHHHHHHHHHHHcccchhhhcchHhhhHHHHHHHHHHH |
|  | Q Q8N394 | 151 | SLLCYIKHCSTRGYSARTWGWFLGSGLCAGCSMLWKEQGVTVLAVSAVYDVFVFHRLKIKQILPTIYKRKNLSLFLSISL   230 (475) |
|  | Q Consensus | 151 | ~l~~~l~~~~~~~~~~~~~~~~~l~~l~~~la~ltk~~~~~~~~~~~~~~l~~~~~~~~~~~~~~~~~~~~~~~~~~~~~   230 (475) |
|  |  |  | +++++.+..+++ ++++.++++++.+++.++|+.+.++.+++.+..+.....++.+. ++.........+ |
|  | T Consensus | 178 | ~l~~~~~~~~~~-----~~~~~~l~gl~~~l~~~~~~~~~~~~~~~~~~~~~~~~~~~~~~-------~~~~~~~~~~~~   245 (718) |
|  | T 6EZN\_F | 178 | TFMFWIKAQKTG-----SIMHATCAALFYFYMVSAWGGYVFITNLIPLHVFLLILMGRYSS-------KLYSAYTTWYAI   245 (718) |
|  | T ss\_dssp |  | HHHHHHHHHHHC-----CHHHHHHHHHHHHHHHTTCTTGGGGGGTHHHHHHHHHHTTCCCH-------HHHHHHHHHHHH |
|  | T ss\_pred |  | HHHHHHHHhhcC-----CHHHHHHHHHHHHHHHHhccchHHHHHHHHHHHHHHHHccCCCh-------hHHHHHHHHHHH |
|  |
|  |
|  | Q ss\_pred |  | HHHHHHHHHHHHHHHhcCCCCCccCCCCCCcCCChHHH------------------------------------------ |
|  | Q Q8N394 | 231 | LIFWGSSLLGARLYWMGNKPPSFSNSDNPAADSDSLLT------------------------------------------   268 (475) |
|  | Q Consensus | 231 | ~~~~~~~~~~~~~~~~~~~~~~~~~~~~~~~~~~~~~~------------------------------------------   268 (475) |
|  |  |  | .+.+........ ............ |
|  | T Consensus | 246 | ~~~~~~~~p~~~--------------~~~~~~~~~~~~~~~~~~~~~~~~~~~~~~~~~~~~~~~~~~~~~~~~~~~~~~   311 (718) |
|  | T 6EZN\_F | 246 | GTVASMQIPFVG--------------FLPIRSNDHMAALGVFGLIQIVAFGDFVKGQISTAKFKVIMMVSLFLILVLGVV   311 (718) |
|  | T ss\_dssp |  | HHHHTTCSTTSS--------------SHHHHCTTSHHHHHHHHHHHHHHHHHHHHTTSCHHHHTTTC------------- |
|  | T ss\_pred |  | HHHHHhcchhcc--------------CCccCchHHHHHHHHHHHHHHHHHHHHHHhcCChHHHHHHHHHHHHHHHHHHHH |
|  |
|  |
|  | Q ss\_pred |  | --------HHHHHHHHHHHhHHHhhCcccccccccccccccccccCCHHHHHHHHHHHHHHHHHHHHhhCcccccccCCC |
|  | Q Q8N394 | 269 | --------RTLTFFYLPTKNLWLLLCPDTLSFDWSMDAVPLLKTVCDWRNLHTVAFYTGLLLLAYYGLKSPSVDRECNGK   340 (475) |
|  | Q Consensus | 269 | --------~~~~~~~~~~~~~~~~~~p~~~~~~~~~~~~~~~~~~~~~~~~~~~~~~~~~~~~~~~~~~~~~~~~~~~~~   340 (475) |
|  |  |  | ................................................+++++++....+++ |
|  | T Consensus | 312 | ~~~~~~~~~~~~~~~~~~~~~~~~~~~~~~~~~~~~~~~~~~~~~~~~~~~~~~~~~l~~~g~~~~~~~~----------   381 (718) |
|  | T 6EZN\_F | 312 | GLSALTYMGLIAPWTGRFYSLWDTNYAKIHIPIIASVSEHQPVSWPAFFFDTHFLIWLFPAGVFLLFLDL----------   381 (718) |
|  | T ss\_dssp |  | ----------------------------------------CCCCHHHHHHHSSSTTTTHHHHHHHHHTTC---------- |
|  | T ss\_pred |  | HHHHHHHhhhhhhhhHHHHHhhcccccccCCCcccchhHhCCCCHHHHHhhhhHHHHHHHHHHHHHHhcC---------- |
|  |
|  |
|  | Q ss\_pred |  | cccCCccccCCcccccccccccccccccccchhhcCCcCCcccccCCCCCCchHHhhhHHHHHHHhhhcCccccCChhhH |
|  | Q Q8N394 | 341 | TVTNGKQNANGHSCLSDVEYQNSETKSSFASKVENGIKNDVSQRTQLPSTENIVVLSLSLLIIPFVPATNLFFYVGFVIA   420 (475) |
|  | Q Consensus | 341 | ~~~~~~~~~~~~~~~~~~~~~~~~~~~~~~~~~~~~~~~~~~~~~~~~~~~~~~~~~l~~~~~~~~~~~~~~~~~~~~~~   420 (475) |
|  |  |  | ++...+.+++.++.+.. .... |
|  | T Consensus | 382 | --------------------------------------------------~~~~~~~~~~~~~~~~~---------~~~~   402 (718) |
|  | T 6EZN\_F | 382 | --------------------------------------------------KDEHVFVIAYSVLCSYF---------AGVM   402 (718) |
|  | T ss\_dssp |  | --------------------------------------------------CSSHHHHHHHHHHHHHH---------HHHC |
|  | T ss\_pred |  | --------------------------------------------------ChHHHHHHHHHHHHHHH---------HHHH |
|  |
|  |
|  | Q ss\_pred |  | HHHhHHHHHHHHHHHHHHHHHHHHHhchHH-------------HHHHHHHHHHHHHHHHHHHHHHhc |
|  | Q Q8N394 | 421 | ERVLYIPSMGFCLLITVGARALYVKVQKRF-------------LKSLIFYATATLIVFYGLKTAIRN   474 (475) |
|  | Q Consensus | 421 | ~Ry~~~~~~~~~ll~~~~l~~~~~~~~~~~-------------~~~~~~~~~~~~~~~~~~~~~~~~   474 (475) |
|  |  |  | .||..+..|+++++++.++..+.++.+++. ..+.+..+++++++.......... |
|  | T Consensus | 403 | ~R~~~~~~p~~~il~a~~l~~l~~~~~~~~~~~~~~~~~~~~~~~~~~~~~~~~~~~~~~~~~~~~~   469 (718) |
|  | T 6EZN\_F | 403 | VRLMLTLTPVICVSAAVALSKIFDIYLDFKTSDRKYAIKPAALLAKLIVSGSFIFYLYLFVFHSTWV   469 (718) |
|  | T ss\_dssp |  | STTHHHHHHHHHHHHHHHHHHHHHHSCCCC-------CCHHHHHHHHHHHHHHHHHHHHHHHHHHHH |
|  | T ss\_pred |  | HHHHHHHHHHHHHHHHHHHHHHHHHHhccccCCccccccchHHHHHHHHHHHHHHHHHHHHHHHHHH |
|  |
| --- | | | |
|  | Template alignmentTemplate 3D StructurePDBe | | |
| 4. | 6S7O\_A Dolichyl-diphosphooligosaccharide--protein glycosyltransferase subunit STT3A (E.C.2.4.99.18); N-glycosylation, Oligosaccharyltransferase, OSTA, TRANSFERASE; HET: KZB, NAG, EGY, MAN, KZE, BMA; 3.5A {Homo sapiens}; Related PDB entries: 6FTI\_5 6FTG\_5 6FTJ\_5; Related PDB entries: 6FTG\_5 6FTI\_5 6FTJ\_5; Related PDB entries: 6FTG\_5 6FTI\_5 6FTJ\_5 | | |
|  | Probability: 99.77%, E-value: 4.9e-15, Score: 155.32, Aligned cols: 374, Identities: 11%, Similarity: -0.032, | | |
|  |
|  | Q ss\_pred |  | HHHHHHHHHHHHHHHhh----cCCCCcccchhHHHhchhhCCCCchhHhhhcccccccccCCCCcccCChHHHHHHHHHH |
|  | Q Q8N394 | 2 | IAELVSSALGLALYLNT----LSADFCYDDSRAIKTNQDLLPETPWTHIFYNDFWGTLLTHSGSHKSYRPLCTLSFRLNH   77 (475) |
|  | Q Consensus | 2 | ~~~lll~~~~~~~~~~~----~~~~~~~De~~~~~~~~~~~~~~~~~~~~~~~~~~~~~~~~~~~~~~~Pl~~~l~~~~~   77 (475) |
|  |  |  | ++++++++++....... .......||..+...++.+.+++........+..............++|++.++.+..+ |
|  | T Consensus | 21 | ~~l~~~~~~~~~~~~~~~~~~~~~~~~~D~~~~~~~a~~~~~~g~~~~~~~~~~~~~~~~g~~~~~~~~p~~~~~~~~~~   100 (705) |
|  | T 6S7O\_A | 21 | LILSMAAVLSFSTRLFAVLRFESVIHEFDPYFNYRTTRFLAEEGFYKFHNWFDDRAWYPLGRIIGGTIYPGLMITSAAIY   100 (705) |
|  | T ss\_dssp |  | HHHHHHHHHHHHHHCSHHHHSCCCCCSSSHHHHHHHHHHHHHHCSHHHHSCEECSSSTTSCEEHHHHSCCHHHHHHHHHH |
|  | T ss\_pred |  | HHHHHHHHHHHHHHHHHHHhcccccccCChHHHHHHHHHHHHhCChhhhccccccccCCCcCCCCCcCCchHHHHHHHHH |
|  |
|  |
|  | Q ss\_pred |  | HHhC------CChHHHHHHHHHHHHHHHHHHHHHHHHHhCChHHHHHHHHHHHHCHHhHHHHHhhhchHHHHHHHHHHHH |
|  | Q Q8N394 | 78 | AIGG------LNPWSYHLVNVLLHAAVTGLFTSFSKILLGDGYWTFMAGLMFASHPIHTEAVAGIVGRADVGASLFFLLS   151 (475) |
|  | Q Consensus | 78 | ~lfG------~~~~~~rl~~~l~~~l~~~lly~l~r~l~~~~~~al~aall~a~~P~~~~~~~~~~~~~~~~~~~f~ll~   151 (475) |
|  |  |  | .++| .+....|+.++++++++++++|.++|++.+ +..|++++++++++|.+...+.....++|.+..++.+++ |
|  | T Consensus | 101 | ~l~g~~g~~~~~~~~~~~~~~l~~~l~~~~~y~l~~~~~~-~~~al~aa~l~~~~p~~~~~~~~~~~~~~~~~~~~~~~~   179 (705) |
|  | T 6S7O\_A | 101 | HVLHFFHITIDIRNVCVFLAPLFSSFTTIVTYHLTKELKD-AGAGLLAAAMIAVVPGYISRSVAGSYDNEGIAIFCMLLT   179 (705) |
|  | T ss\_dssp |  | HHHHHTTCCCCHHHHHHTHHHHHHHHHHHHHHHHHHHHSC-HHHHHHHHHHHHSCHHHHHSSCTTCCCHHHHHHHHHHHH |
|  | T ss\_pred |  | HHHHHcCCCCCHHHHHHHHHHHHHHHHHHHHHHHHHHHcC-HHHHHHHHHHHHHHHHHHHhhcccchhHHHHHHHHHHHH |
|  |
|  |
|  | Q ss\_pred |  | HHHHHHHHccCCCCcchHHHHHHHHHHHHHHHHhHhHHHHHHHHHHHHHHHHHcccchhhhcc----------------- |
|  | Q Q8N394 | 152 | LLCYIKHCSTRGYSARTWGWFLGSGLCAGCSMLWKEQGVTVLAVSAVYDVFVFHRLKIKQILP-----------------   214 (475) |
|  | Q Consensus | 152 | l~~~l~~~~~~~~~~~~~~~~~l~~l~~~la~ltk~~~~~~~~~~~~~~l~~~~~~~~~~~~~-----------------   214 (475) |
|  |  |  | ++++.+..+++ ++++.++++++.+++.++|+....+.+++.+..+.....++.+.... |
|  | T Consensus | 180 | ~~~~~~~~~~~-----~~~~~~~~gl~~~l~~~~~~~~~~~~~~~~~~~~~~~~~~~~~~~~~~~~~~~~~~~~~~~~~~   254 (705) |
|  | T 6S7O\_A | 180 | YYMWIKAVKTG-----SICWAAKCALAYFYMVSSWGGYVFLINLIPLHVLVLMLTGRFSHRIYVAYCTVYCLGTILSMQI   254 (705) |
|  | T ss\_dssp |  | HHHHHHHHHHC-----CHHHHHHHHHHHHHHHHHCTTHHHHTTTHHHHHHHHHHHTCCCHHHHHHHHHHHHHHHHHHHTT |
|  | T ss\_pred |  | HHHHHHHHhhC-----CHHHHHHHHHHHHHHHHhhchHHHHHHHHHHHHHHHHHhcCCChHHHHHHHHHHHHHHHHHHhc |
|  |
|  |
|  | Q ss\_pred |  | -------------------------------------hHhhhHHHHHHHHHHHHHHHHHHHHHHHHHHhcCCCCCccCCC |
|  | Q Q8N394 | 215 | -------------------------------------TIYKRKNLSLFLSISLLIFWGSSLLGARLYWMGNKPPSFSNSD   257 (475) |
|  | Q Consensus | 215 | -------------------------------------~~~~~~~~~~~~~~~~~~~~~~~~~~~~~~~~~~~~~~~~~~~   257 (475) |
|  |  |  | +...+............................. |
|  | T Consensus | 255 | ~~~~~~~~~~~~~~~~~~~~~~~~~~~~~~~~~~~~~~~~~~~~~~~~~~~~~~~~~~~~~~~~~~~~~~~---------   325 (705) |
|  | T 6S7O\_A | 255 | SFVGFQPVLSSEHMAAFGVFGLCQIHAFVDYLRSKLNPQQFEVLFRSVISLVGFVLLTVGALLMLTGKISP---------   325 (705) |
|  | T ss\_dssp |  | SSSTTHHHHSSTTHHHHHHHHHHHHHHHHHHHHHHSCHHHHHHHC----------------------CCCC--------- |
|  | T ss\_pred |  | cccCCcccCCHHHHHHHHHHHHHHHHHHHHHHHHcCCHHHHHHHHHHHHHHHHHHHHHHHHHHHHcccccc--------- |
|  |
|  |
|  | Q ss\_pred |  | CCCcCCChHHHHHHHHHHHHHHhHHHhhCcccccccccccccccccccCCHHHHHHHHHHHHHHHHHHHHhhCccccccc |
|  | Q Q8N394 | 258 | NPAADSDSLLTRTLTFFYLPTKNLWLLLCPDTLSFDWSMDAVPLLKTVCDWRNLHTVAFYTGLLLLAYYGLKSPSVDREC   337 (475) |
|  | Q Consensus | 258 | ~~~~~~~~~~~~~~~~~~~~~~~~~~~~~p~~~~~~~~~~~~~~~~~~~~~~~~~~~~~~~~~~~~~~~~~~~~~~~~~~   337 (475) |
|  |  |  | ..........................................+++++++.....+++ |
|  | T Consensus | 326 | ----------------~~~~~~~~~~~~~~~~~~~~~~~~~~~~~~~~~~~~~~~~~~~~l~~~~~~~~~~~~-------   382 (705) |
|  | T 6S7O\_A | 326 | ----------------WTGRFYSLLDPSYAKNNIPIIASVSEHQPTTWSSYYFDLQLLVFMFPVGLYYCFSNL-------   382 (705) |
|  | T ss\_dssp |  | ----------------CCSTTHHHHSTTHHHHTCTTTTTSGGGSCCCHHHHHHHCSSSGGGHHHHHHHHHHSC------- |
|  | T ss\_pred |  | ----------------ccHHHHHhhChhHhcCCCCceeeccccCCCCHHHHHHHHHHHHHHHHHHHHHHhcCC------- |
|  |
|  |
|  | Q ss\_pred |  | CCCcccCCccccCCcccccccccccccccccccchhhcCCcCCcccccCCCCCCchHHhhhHHHHHHHhhhcCccccCCh |
|  | Q Q8N394 | 338 | NGKTVTNGKQNANGHSCLSDVEYQNSETKSSFASKVENGIKNDVSQRTQLPSTENIVVLSLSLLIIPFVPATNLFFYVGF   417 (475) |
|  | Q Consensus | 338 | ~~~~~~~~~~~~~~~~~~~~~~~~~~~~~~~~~~~~~~~~~~~~~~~~~~~~~~~~~~~~l~~~~~~~~~~~~~~~~~~~   417 (475) |
|  |  |  | ++.....+++.++.+.. . |
|  | T Consensus | 383 | -----------------------------------------------------~~~~~~~~~~~~~~~~~---------~   400 (705) |
|  | T 6S7O\_A | 383 | -----------------------------------------------------SDARIFIIMYGVTSMYF---------S   400 (705) |
|  | T ss\_dssp |  | -----------------------------------------------------CTTHHHHHHHHHHHHHH---------H |
|  | T ss\_pred |  | -----------------------------------------------------ChhHHHHHHHHHHHHHH---------H |
|  |
|  |
|  | Q ss\_pred |  | hhHHHHhHHHHHHHHHHHHHHHHHHHHHhchHHH----------------------HHHHHHHHHHHHHHHHHHHHHhcC |
|  | Q Q8N394 | 418 | VIAERVLYIPSMGFCLLITVGARALYVKVQKRFL----------------------KSLIFYATATLIVFYGLKTAIRNG   475 (475) |
|  | Q Consensus | 418 | ~~~~Ry~~~~~~~~~ll~~~~l~~~~~~~~~~~~----------------------~~~~~~~~~~~~~~~~~~~~~~~~   475 (475) |
|  |  |  | ....||..+..|+++++++.++..+.++.+++.. ......+++++............. |
|  | T Consensus | 401 | ~~~~R~~~~~~p~~~l~~a~~l~~l~~~~~~~~~~~~~~~~~~~~~~~~~~~~~~~~~~~~~~~~~~~~~~~~~~~~~~~   480 (705) |
|  | T 6S7O\_A | 401 | AVMVRLMLVLAPVMCILSGIGVSQVLSTYMKNLDISRPDKKSKKQQDSTYPIKNEVASGMILVMAFFLITYTFHSTWVTS   480 (705) |
|  | T ss\_dssp |  | HHCSGGGGGGHHHHHHHHHHHHHHHHHHHTTTSSSCC---------------CCHHHHHHHHHHHHHHHHHHHHHHHHCC |
|  | T ss\_pred |  | HHhHHHHHHHHHHHHHHHHHHHHHHHHHHHhhcCCCCCCccCccccCCCCCcCHHHHHHHHHHHHHHHHHHHHhHhHHHH |
|  |
| --- | | | |
|  | Template alignmentTemplate 3D StructurePDBe | | |
| 5. | 3WAJ\_A Transmembrane oligosaccharyl transferase (E.C.2.4.1.119); oligosaccharyltransferase, N-glycosylation, Archaeoglobus fulgidus, GT-C; 2.501A {Archaeoglobus fulgidus}; Related PDB entries: 5GMY\_A 3WAK\_A; Related PDB entries: 5GMY\_A 3WAK\_A; Related PDB entries: 5GMY\_A 3WAK\_A | | |
|  | Probability: 99.74%, E-value: 8.6e-15, Score: 157.74, Aligned cols: 389, Identities: 10%, Similarity: -0.017, | | |
|  |
|  | Q ss\_pred |  | CHHHHHHHHHHHHHH------HhhcCCCCcccchhHHHhchhhCCCCchhHhhhcccccccccCCCCcccCChHHHHHHH |
|  | Q Q8N394 | 1 | MIAELVSSALGLALY------LNTLSADFCYDDSRAIKTNQDLLPETPWTHIFYNDFWGTLLTHSGSHKSYRPLCTLSFR   74 (475) |
|  | Q Consensus | 1 | ~~~~lll~~~~~~~~------~~~~~~~~~~De~~~~~~~~~~~~~~~~~~~~~~~~~~~~~~~~~~~~~~~Pl~~~l~~   74 (475) |
|  |  |  | ++++++++.+..... ..........||..+...++.+.++......+....+.. .+....++|++.++.+ |
|  | T Consensus | 16 | ~l~~l~~~~~~lr~~~~~~~~~~~~~~~~~~D~~~~~~~a~~~~~~~~~~~~~d~~~~~p----~g~~~~~~Pl~~~l~a   91 (875) |
|  | T 3WAJ\_A | 16 | VLVIAALISVKLRILNPWNSVFTWTVRLGGNDPWYYYRLIENTIHNFPHRIWFDPFTYYP----YGSYTHFGPFLVYLGS   91 (875) |
|  | T ss\_dssp |  | TTTHHHHHHHCCCCCTTHHHHBSSSBCCCSSHHHHHHHHHHHHHHTTTCCCSEETTSTTT----TCEECCSCHHHHHHHH |
|  | T ss\_pred |  | HHHHHHHHHHHHHhhCccccccCCceecccCChHHHHHHHHHHHHHCcccCCCCchhcCC----CCcCCCchhHHHHHHH |
|  |
|  |
|  | Q ss\_pred |  | HHHHHhCCC-----hHHHHHHHHHHHHHHHHHHHHHHHHHhCChHHHHHHHHHHHHCHH-hHHHHHhhhchHHHHHHHHH |
|  | Q Q8N394 | 75 | LNHAIGGLN-----PWSYHLVNVLLHAAVTGLFTSFSKILLGDGYWTFMAGLMFASHPI-HTEAVAGIVGRADVGASLFF   148 (475) |
|  | Q Consensus | 75 | ~~~~lfG~~-----~~~~rl~~~l~~~l~~~lly~l~r~l~~~~~~al~aall~a~~P~-~~~~~~~~~~~~~~~~~~f~   148 (475) |
|  |  |  | ....++|.+ ....|+.++++++++++++|.++|++++ +..|++++++++++|. ....+......+|.+..++. |
|  | T Consensus | 92 | ~~~~l~G~~~~~~~~~~~~l~~~l~~~l~~~~~y~l~r~l~~-~~~allaall~a~~p~~~~~~s~~g~~~~~~~~~~~~   170 (875) |
|  | T 3WAJ\_A | 92 | IAGIIFSATSGESLRAVLAFIPAIGGVLAILPVYLLTREVFD-KRAAVIAAFLIAIVPGQFLQRSILGFNDHHIWEAFWQ   170 (875) |
|  | T ss\_dssp |  | HHHHHTTCCSHHHHHHHHHHHHHHHHHTTHHHHHHHHHHHSC-HHHHHHHHHHHTTCCSHHHHTTSTTCCCSHHHHHHHH |
|  | T ss\_pred |  | HHHHHHcCCChHHHHHHHHHHHHHHHHHHHHHHHHHHHHHcC-hHHHHHHHHHHHHcchHHHHHHhccccchHHHHHHHH |
|  |
|  |
|  | Q ss\_pred |  | HHHHHHHHHHHc------------cCCCCcchHHHHHHHHHHHHHHHHhHhHHHHHHHHHHHHHHHHHcccchhhhcchH |
|  | Q Q8N394 | 149 | LLSLLCYIKHCS------------TRGYSARTWGWFLGSGLCAGCSMLWKEQGVTVLAVSAVYDVFVFHRLKIKQILPTI   216 (475) |
|  | Q Consensus | 149 | ll~l~~~l~~~~------------~~~~~~~~~~~~~l~~l~~~la~ltk~~~~~~~~~~~~~~l~~~~~~~~~~~~~~~   216 (475) |
|  |  |  | +++++++.+..+ ++ ++++.++++++++++.++|..+.++.+++++..++....++.++ |
|  | T Consensus | 171 | ~l~l~~~~~~~~~~~~~~~~~~~~~~-----~~~~~~l~gl~~gl~~lt~~~~~~~~~~~~~~~~~~~~~~~~~~-----   240 (875) |
|  | T 3WAJ\_A | 171 | VSALGTFLLAYNRWKGHDLSHNLTAR-----QMAYPVIAGITIGLYVLSWGAGFIIAPIILAFMFFAFVLAGFVN-----   240 (875) |
|  | T ss\_dssp |  | HHHHHHHHHHHTTSSSCCC----CTT-----TSHHHHHHHHHHHHHHHHCGGGGGHHHHHHHHHHHHHHTTTTCC----- |
|  | T ss\_pred |  | HHHHHHHHHHHHHhccCCCCcccccc-----hhHHHHHHHHHHHHHHHHhchHHHHHHHHHHHHHHHHHHHHhCC----- |
|  |
|  |
|  | Q ss\_pred |  | hhhHHHHHHHHHHHHHHHHHHHHHHHHHHhcCCCCCccCCCCCCcCCChHHHHHHHHHHHH------------------- |
|  | Q Q8N394 | 217 | YKRKNLSLFLSISLLIFWGSSLLGARLYWMGNKPPSFSNSDNPAADSDSLLTRTLTFFYLP-------------------   277 (475) |
|  | Q Consensus | 217 | ~~~~~~~~~~~~~~~~~~~~~~~~~~~~~~~~~~~~~~~~~~~~~~~~~~~~~~~~~~~~~-------------------   277 (475) |
|  |  |  | ++.........+.+++..+........................................ |
|  | T Consensus | 241 | --~~~~~~~~~~~~~~~~~~l~~~p~~~~~~~~~~~~~~~~~~~~~~~~~~~~~~~~~~~~~~~~~~~~~~~~~~~~~~~   318 (875) |
|  | T 3WAJ\_A | 241 | --ADRKNLSLVAVVTFAVSALIYLPFAFNYPGFSTIFYSPFQLLVLLGSAVIAAAFYQIEKWNDVGFFERVGLGRKGMPL   318 (875) |
|  | T ss\_dssp |  | --CCHHHHHHHHHHHHHHHHHHHGGGTTSSSSCCSSSSCHHHHHHHHHHHHHHHHHHHHHHHHHHTHHHHTTCGGGHHHH |
|  | T ss\_pred |  | --CCCHhHHHHHHHHHHHHHHHHHHHhcCCCCCChhcccHHHHHHHHHHHHHHHHHHHHHHhcCcchHHhcCCCccchHH |
|  |
|  |
|  | Q ss\_pred |  | -----------------HHhHHHhhCcccccccccccccccccccC--------------CHHHHHHHHHHHHHHHHHHH |
|  | Q Q8N394 | 278 | -----------------TKNLWLLLCPDTLSFDWSMDAVPLLKTVC--------------DWRNLHTVAFYTGLLLLAYY   326 (475) |
|  | Q Consensus | 278 | -----------------~~~~~~~~~p~~~~~~~~~~~~~~~~~~~--------------~~~~~~~~~~~~~~~~~~~~   326 (475) |
|  |  |  | ............................. ........+.+++++..... |
|  | T Consensus | 319 | ~~~~~~~~~~~~~~~~~~~~~~~~~~~~~~~~~~~~~~~i~e~~~~~~~~~~~~~~~~~~~~~~~~~~l~~~~~~~~~~~   398 (875) |
|  | T 3WAJ\_A | 319 | AVIVLTALIMGLFFVISPDFARNLLSVVRVVQPKGGALTIAEVYPFFFTHNGEFTLTNAVLHFGALFFFGMAGILYSAYR   398 (875) |
|  | T ss\_dssp |  | HHHHHHHHHHHHHHC----------------------------------------CTHHHHHHTTHHHHHHHHHHHHHHH |
|  | T ss\_pred |  | HHHHHHHHHHHHHHHHCCHHHHHHHhhcceecCCCCceeeeeccccccccCCCccHHHHHHHhHHHHHHHHHHHHHHHHH |
|  |
|  |
|  | Q ss\_pred |  | HhhCcccccccCCCcccCCccccCCcccccccccccccccccccchhhcCCcCCcccccCCCCCCchHHhhhHHHHHHHh |
|  | Q Q8N394 | 327 | GLKSPSVDRECNGKTVTNGKQNANGHSCLSDVEYQNSETKSSFASKVENGIKNDVSQRTQLPSTENIVVLSLSLLIIPFV   406 (475) |
|  | Q Consensus | 327 | ~~~~~~~~~~~~~~~~~~~~~~~~~~~~~~~~~~~~~~~~~~~~~~~~~~~~~~~~~~~~~~~~~~~~~~~l~~~~~~~~   406 (475) |
|  |  |  | ..++ ......+.+++.++.++ |
|  | T Consensus | 399 | ~~~~-----------------------------------------------------------~~~~~~~l~~~~~~~~~   419 (875) |
|  | T 3WAJ\_A | 399 | FLKR-----------------------------------------------------------RSFPEMALLIWAIAMFI   419 (875) |
|  | T ss\_dssp |  | HHHH-----------------------------------------------------------CCHHHHHHHHHHHHHHH |
|  | T ss\_pred |  | HHcc-----------------------------------------------------------CCchhHHHHHHHHHHHH |
|  |
|  |
|  | Q ss\_pred |  | hhcCccccCChhhHHHHhHHHHHHHHHHHHHHHHHHHHHh------------chHHHHHHHHHHHHHHHHHHHHHHHHhc |
|  | Q Q8N394 | 407 | PATNLFFYVGFVIAERVLYIPSMGFCLLITVGARALYVKV------------QKRFLKSLIFYATATLIVFYGLKTAIRN   474 (475) |
|  | Q Consensus | 407 | ~~~~~~~~~~~~~~~Ry~~~~~~~~~ll~~~~l~~~~~~~------------~~~~~~~~~~~~~~~~~~~~~~~~~~~~   474 (475) |
|  |  |  | . .....||.++..|+++++++.++..+.++. +++........++++++........... |
|  | T Consensus | 420 | ~---------~~~~~Ry~~~~~p~~~il~a~~l~~l~~~~~~~~~~~~~~~~~~~~~~~~~~~~~~~~~~~~~~~~~~~~   490 (875) |
|  | T 3WAJ\_A | 420 | A---------LWGQNRFAYYFAAVSAVYSALALSVVFDKLHLYRALENAIGARNKLSYFRVAFALLIALAAIYPTYILAD   490 (875) |
|  | T ss\_dssp |  | H---------TSSCGGGTHHHHHHHHHHHHHHHHHHGGGCC-----------------CCTTTHHHHHHHHHHHHHHHHH |
|  | T ss\_pred |  | H---------HHHhhhHHHHHHHHHHHHHHHHHHHHHHHHhHHHHHHhhhchhccchHHHHHHHHHHHHHHHHHHHHHHH |
|  |
| --- | | | |
|  | Template alignmentTemplate 3D StructurePDBe | | |
| 6. | 5OGL\_A Peptide-binding protein, Substrate mimicking peptide; Oligosaccharyltransferase, Complex, Protein N-glycosylation, Bacteria; HET: 9UB, PPN; 2.7A {Campylobacter lari (strain RM2100 / D67 / ATCC BAA-1060)}; Related PDB entries: 3RCE\_A 6GXC\_A; Related PDB entries: 6GXC\_A 3RCE\_A ; Related PDB entries: 6GXC\_A 3RCE\_A | | |
|  | Probability: 99.72%, E-value: 5.7e-14, Score: 147.9, Aligned cols: 359, Identities: 13%, Similarity: 0.064, | | |
|  |
|  | Q ss\_pred |  | HHHHHHHHHHHHHHH-----hhcCCCCcccch---------hHHHhchhhCCCCchhHhhhcccccccccCCCCcccCCh |
|  | Q Q8N394 | 2 | IAELVSSALGLALYL-----NTLSADFCYDDS---------RAIKTNQDLLPETPWTHIFYNDFWGTLLTHSGSHKSYRP   67 (475) |
|  | Q Consensus | 2 | ~~~lll~~~~~~~~~-----~~~~~~~~~De~---------~~~~~~~~~~~~~~~~~~~~~~~~~~~~~~~~~~~~~~P   67 (475) |
|  |  |  | +.+++++++++.++. ..-...+..||. .|...+++..++... ......++| |
|  | T Consensus | 18 | ~~l~~i~~~~~~lRl~~~~~~~~~~~~~~~~~~~~~~~D~~~~~~~a~~~~~~~~~---------------~~~~~~~~p   82 (713) |
|  | T 5OGL\_A | 18 | ILILIAFAFSVLARLYWVAWASEFYEFFFNDQLMITTNDGYAFAEGARDMIAGFHQ---------------PNDLSYFGS   82 (713) |
|  | T ss\_dssp |  | HHHHHHHHHHHHHHHHHHHHHTTCGGGEETTEECCSSTTHHHHHHHHHHHHHTCCC---------------TTSCCCTTC |
|  | T ss\_pred |  | HHHHHHHHHHHHHHHHHHHHhhccchhhcCCEEeeccccHHHHHHHHHHHHcCCCC---------------CCCcchhcc |
|  |
|  |
|  | Q ss\_pred |  | HHHHHHHHHHHHhC-CChHHHHHHHHHHHHHHHHHHHHHHHHHhCChHHHHHHHHHHHHCHHhHHHHHhhhchHHHHHHH |
|  | Q Q8N394 | 68 | LCTLSFRLNHAIGG-LNPWSYHLVNVLLHAAVTGLFTSFSKILLGDGYWTFMAGLMFASHPIHTEAVAGIVGRADVGASL   146 (475) |
|  | Q Consensus | 68 | l~~~l~~~~~~lfG-~~~~~~rl~~~l~~~l~~~lly~l~r~l~~~~~~al~aall~a~~P~~~~~~~~~~~~~~~~~~~   146 (475) |
|  |  |  | ++.++.+....++| ......|++++++++++++.+|.++|++++ +..|++++++++++|.+...+......+|.+..+ |
|  | T Consensus | 83 | ~~~~l~~~~~~l~g~~~~~~~~~~~~l~~~l~v~~~y~l~r~l~~-~~~al~aall~a~~p~~~~~s~~g~~~~d~~~~~   161 (713) |
|  | T 5OGL\_A | 83 | SLSTLTYWLYSILPFSFESIILYMSTFFASLIVVPIILIAREYKL-TTYGFIAALLGSIANSYYNRTMSGYYDTDMLVLV   161 (713) |
|  | T ss\_dssp |  | HHHHHHHHHHHHSCSCHHHHHHHHHHHHGGGGHHHHHHHHHHTTC-HHHHHHHHHHHHHCHHHHHTTSTTCCSGGGGTTH |
|  | T ss\_pred |  | hHHHHHHHHHHhCCCCHHHHHHHHHHHHHHHHHHHHHHHHHHhCC-chHHHHHHHHHHHHHHHHHhhccccCchHHHHHH |
|  |
|  |
|  | Q ss\_pred |  | HHHHHHHHHHHHHccCCCCcchHHHHHHHHHHHHHHHHhHhHHH----HHHHHHHHHHHHHHcccch------------- |
|  | Q Q8N394 | 147 | FFLLSLLCYIKHCSTRGYSARTWGWFLGSGLCAGCSMLWKEQGV----TVLAVSAVYDVFVFHRLKI-------------   209 (475) |
|  | Q Consensus | 147 | f~ll~l~~~l~~~~~~~~~~~~~~~~~l~~l~~~la~ltk~~~~----~~~~~~~~~~l~~~~~~~~-------------   209 (475) |
|  |  |  | +.+++++++.+..+++ +.++.++++++.+++.++|..+. .+.+...++.+...++++. |
|  | T Consensus | 162 | ~~~l~~~~~~~~~~~~-----~~~~~~l~gl~~~l~~~~~~~~~~~~~~~~~~~~~~~~~~~~~~~~~~~~~~~~~~~~~   236 (713) |
|  | T 5OGL\_A | 162 | LPMLILLTFIRLTINK-----DIFTLLLSPVFIMIYLWWYPSSYSLNFAMIGLFGLYTLVFHRKEKIFYLTIALMIIALS   236 (713) |
|  | T ss\_dssp |  | HHHHHHHHHHHHHHHC-----CTTHHHHHHHHHHHHHHHCGGGHHHHHHHHHHHHHHHHHHTTTCHHHHHHHHHHHHHHS |
|  | T ss\_pred |  | HHHHHHHHHHHHHcCC-----chHHHHHHHHHHHHHHhhccchHHHHHHHHHHHHHHHHHhCCcchHHHHHHHHHHHHHh |
|  |
|  |
|  | Q ss\_pred |  | --------------------hhhcchHhhhHHHHHHHHHHHHHHHHHHHHHHHHHHhcCCCCCccCCCCCCcCCChHHHH |
|  | Q Q8N394 | 210 | --------------------KQILPTIYKRKNLSLFLSISLLIFWGSSLLGARLYWMGNKPPSFSNSDNPAADSDSLLTR   269 (475) |
|  | Q Consensus | 210 | --------------------~~~~~~~~~~~~~~~~~~~~~~~~~~~~~~~~~~~~~~~~~~~~~~~~~~~~~~~~~~~~   269 (475) |
|  |  |  | +......................................................+.... |
|  | T Consensus | 237 | ~~~~~~~~~~~~~~~~~~~~~~~~~~~~~~~~~~~~~~~~~~~~~~~~~~~~~~~~~~~~~~~~~~~~~~~~~~~~~~~~   316 (713) |
|  | T 5OGL\_A | 237 | MLAWQYKLALIVLLFAIFAFKEEKINFYMIWALIFISILILHLSGGLDPVLYQLKFYVFKASDVQNLKDAAFMYFNVNET   316 (713) |
|  | T ss\_dssp |  | CCCHHHHHHHHHHHHHHHHHCSSCCCHHHHHHHHHHHHHHHHHTTTTHHHHHHHHHHTSCCSCCSCCTTTSCCCCCGGGG |
|  | T ss\_pred |  | hhhHHHHHHHHHHHHHHHHcchhccchHHHHHHHHHHHHHHHHhccchHHHHHHHHHhhcccccccccccccccccHHHH |
|  |
|  |
|  | Q ss\_pred |  | HHHHHHHHHHhHHHhhCcccccccccccccccccccCCHHHHHHHHHHHHHHHHHHHHhhCcccccccCCCcccCCcccc |
|  | Q Q8N394 | 270 | TLTFFYLPTKNLWLLLCPDTLSFDWSMDAVPLLKTVCDWRNLHTVAFYTGLLLLAYYGLKSPSVDRECNGKTVTNGKQNA   349 (475) |
|  | Q Consensus | 270 | ~~~~~~~~~~~~~~~~~p~~~~~~~~~~~~~~~~~~~~~~~~~~~~~~~~~~~~~~~~~~~~~~~~~~~~~~~~~~~~~~   349 (475) |
|  |  |  | ..+.......... ..........+..+.+.....++ |
|  | T Consensus | 317 | ~~~~~~~~~~~~~-------------------------~~~~~~~~~~~l~~~gl~~~~~~-------------------   352 (713) |
|  | T 5OGL\_A | 317 | IMEVNTIDPEVFM-------------------------QRISSSVLVFILSFIGFILLLKD-------------------   352 (713) |
|  | T ss\_dssp |  | BGGGCCCCHHHHH-------------------------HHHHSSHHHHHHHHHHHHHHHTT------------------- |
|  | T ss\_pred |  | HHHhcCCCHHHHH-------------------------HHhcccHHHHHHHHHHHHHHHHc------------------- |
|  |
|  |
|  | Q ss\_pred |  | CCcccccccccccccccccccchhhcCCcCCcccccCCCCCCchHHhhhHHHHHHHhhhcCccccCChhhHHHHhHHHHH |
|  | Q Q8N394 | 350 | NGHSCLSDVEYQNSETKSSFASKVENGIKNDVSQRTQLPSTENIVVLSLSLLIIPFVPATNLFFYVGFVIAERVLYIPSM   429 (475) |
|  | Q Consensus | 350 | ~~~~~~~~~~~~~~~~~~~~~~~~~~~~~~~~~~~~~~~~~~~~~~~~l~~~~~~~~~~~~~~~~~~~~~~~Ry~~~~~~   429 (475) |
|  |  |  | ++...+.+.++++.+.. .....||.++..| |
|  | T Consensus | 353 | -----------------------------------------~~~~~~~l~~~~~~~~~---------~~~~~R~~~~~~p   382 (713) |
|  | T 5OGL\_A | 353 | -----------------------------------------HKSMLLALPMLALGFMA---------LRAGLRFTIYAVP   382 (713) |
|  | T ss\_dssp |  | -----------------------------------------CGGGGGGHHHHHHHHHH---------HHHCGGGGGGGHH |
|  | T ss\_pred |  | -----------------------------------------CHhHHHHHHHHHHHHHH---------HHHhHHHHHHHHH |
|  |
|  |
|  | Q ss\_pred |  | HHHHHHHHHHHHHHHHhchHHH------HHHHHHHHHHHHHHHHHHHHHhcC |
|  | Q Q8N394 | 430 | GFCLLITVGARALYVKVQKRFL------KSLIFYATATLIVFYGLKTAIRNG   475 (475) |
|  | Q Consensus | 430 | ~~~ll~~~~l~~~~~~~~~~~~------~~~~~~~~~~~~~~~~~~~~~~~~   475 (475) |
|  |  |  | +++++++.++..+.++.+++.. +..+.++++++++........... |
|  | T Consensus | 383 | ~~~i~~a~~l~~l~~~~~~~~~~~~~~~~~~~~~~~~~~~~~~~~~~~~~~~   434 (713) |
|  | T 5OGL\_A | 383 | VMALGFGYFLYAFFNFLEKKQIKLSLRNKNILLILIAFFSISPALMHIYYYK   434 (713) |
|  | T ss\_dssp |  | HHHHHHHHHHHHHHHHHHHTTCCCCHHHHHHHHHHHHHHHHHHHHHHHHHCC |
|  | T ss\_pred |  | HHHHHHHHHHHHHHHHHHHhhcccchhHHHHHHHHHHHHHHhHHHHHHHhcC |
|  |
| --- | | | |
|  | Template alignmentTemplate 3D StructurePDBe | | |
| 7. | 6P25\_A Dolichyl-diphosphooligosaccharide--protein glycosyltransferase subunits (E.C.2.4.99.18); complex, TRANSFERASE, glycosylation; HET: NAG, CPL, NNM; 3.2A {Saccharomyces cerevisiae W303}; Related PDB entries: 6P2R\_A ; Related PDB entries: 6P2R\_A ; Related PDB entries: 6P2R\_A | | |
|  | Probability: 99.68%, E-value: 7.8e-13, Score: 140.15, Aligned cols: 394, Identities: 11%, Similarity: -0.012, | | |
|  |
|  | Q ss\_pred |  | HHHHHHHHHHHHHHHhhcCCCCcccchhHHHhchhhCCCCchhHhhhcccccccccCCCCcccCChHHHHHHHHHHHHhC |
|  | Q Q8N394 | 2 | IAELVSSALGLALYLNTLSADFCYDDSRAIKTNQDLLPETPWTHIFYNDFWGTLLTHSGSHKSYRPLCTLSFRLNHAIGG   81 (475) |
|  | Q Consensus | 2 | ~~~lll~~~~~~~~~~~~~~~~~~De~~~~~~~~~~~~~~~~~~~~~~~~~~~~~~~~~~~~~~~Pl~~~l~~~~~~lfG   81 (475) |
|  |  |  | +++++++.+....+.........+||..+...+..+.+++.+.+. +||++.++.+....++| |
|  | T Consensus | 53 | l~~l~ll~~~lrl~~l~~~~~~~~DE~~~~~~a~~~~~g~~~~~~------------------~PPL~~ll~a~~~~l~G   114 (817) |
|  | T 6P25\_A | 53 | VACLAVFTAVIRLHGLAWPDSVVFDEVHFGGFASQYIRGTYFMDV------------------HPPLAKMLYAGVASLGG   114 (817) |
|  | T ss\_dssp |  | HHHHHHHHHHHTTTTTTSSCBCCTTHHHHHHHHHHHHHCBCCCCS------------------SCTHHHHHHHHHHHHTC |
|  | T ss\_pred |  | HHHHHHHHHHHHHhcccCCCcceeeHHHHHHHHHHHHhCCCCCCC------------------CChHHHHHHHHHHHHcC |
|  |
|  |
|  | Q ss\_pred |  | CCh-----------------HHHHHHHHHHHHHHHHHHHHHHHHHhCChHHHHHHHHHHHHCHHhHHHHHhhhchHHHHH |
|  | Q Q8N394 | 82 | LNP-----------------WSYHLVNVLLHAAVTGLFTSFSKILLGDGYWTFMAGLMFASHPIHTEAVAGIVGRADVGA   144 (475) |
|  | Q Consensus | 82 | ~~~-----------------~~~rl~~~l~~~l~~~lly~l~r~l~~~~~~al~aall~a~~P~~~~~~~~~~~~~~~~~   144 (475) |
|  |  |  | .+. ...|++++++++++++++|.++|++..++..|+++++++++.|.....+..... |.+. |
|  | T Consensus | 115 | ~~~~~~f~~ig~~~~~~~~~~~~Rl~~~l~~~l~v~l~y~i~r~l~~~~~~Allaall~~~~p~~i~~s~~~~~--d~~~   192 (817) |
|  | T 6P25\_A | 115 | FQGDFDFENIGDSFPSTTPYVLMRFFSASLGALTVILMYMTLRYSGVRMWVALMSAICFAVENSYVTISRYILL--DAPL   192 (817) |
|  | T ss\_dssp |  | CCSCCCCCSTTCBCCTTSCCHHHHHHHHHHHHHHHHHHHHHHHHTTCCHHHHHHHHHHHHSCHHHHHHHHSSCS--HHHH |
|  | T ss\_pred |  | CCCCCCccccccCCCCCCHHHHHHHHHHHHHHHHHHHHHHHHHHcCCCHHHHHHHHHHHHHcHHHHHHHHHHhh--HHHH |
|  |
|  |
|  | Q ss\_pred |  | HHHHHHHHHHHHHHHccCCCCcchHHHHHHHHHHHHHHHHhHhHHHHHHHHHHHHHHHH------HcccchhhhcchHhh |
|  | Q Q8N394 | 145 | SLFFLLSLLCYIKHCSTRGYSARTWGWFLGSGLCAGCSMLWKEQGVTVLAVSAVYDVFV------FHRLKIKQILPTIYK   218 (475) |
|  | Q Consensus | 145 | ~~f~ll~l~~~l~~~~~~~~~~~~~~~~~l~~l~~~la~ltk~~~~~~~~~~~~~~l~~------~~~~~~~~~~~~~~~   218 (475) |
|  |  |  | +++++++++++.+..++++..++++++++++++++++++++|+.++++++.+++..++. ..+++.+. |
|  | T Consensus | 193 | ~ff~~lal~~~~~~~~~~~~~~~~~~~l~l~gl~lgla~~tK~~gl~~l~~~~l~~l~~l~~~~~~~~~~~~~-------   265 (817) |
|  | T 6P25\_A | 193 | MFFIAAAVYSFKKYEMYPANSLNAYKSLLATGIALGMASSSKWVGLFTVTWVGLLCIWRLWFMIGDLTKSSKS-------   265 (817) |
|  | T ss\_dssp |  | HHHHHHHHHHHHHHHTSCSSSHHHHHHHHHHHHHHHHHHTTCTTHHHHHHHHHHHHHHHHHHHHHCSSSCHHH------- |
|  | T ss\_pred |  | HHHHHHHHHHHHHHHhCCCCCcHHHHHHHHHHHHHHHHHhhhhHHHHHHHHHHHHHHHHHHHHhCCCCCCHHH------- |
|  |
|  |
|  | Q ss\_pred |  | hHHHHHHHHHHHHHHHHHHHHHHHHHHhcC-------------------------------------------------- |
|  | Q Q8N394 | 219 | RKNLSLFLSISLLIFWGSSLLGARLYWMGN--------------------------------------------------   248 (475) |
|  | Q Consensus | 219 | ~~~~~~~~~~~~~~~~~~~~~~~~~~~~~~--------------------------------------------------   248 (475) |
|  |  |  | .........++++++..+.+.......+.. |
|  | T Consensus | 266 | ~~~~~~~~~~~li~ip~~iy~~~~~~~f~~l~~~g~~~~~~s~~f~~~L~g~~~~~~~~~~v~~Gs~itlr~~~~~~gyL   345 (817) |
|  | T 6P25\_A | 266 | IFKVAFAKLAFLLGVPFALYLVFFYIHFQSLTLDGDGASFFSPEFRSTLKNNKIPQNVVADVGIGSIISLRHLSTMGGYL   345 (817) |
|  | T ss\_dssp |  | HHHHHHHHHCCCCCHHHHHHHHHHHHHHHHCCBCCSTTTTSCTTTGGGSBSCCSCCSEESBCBTTCEEEEEESSSSSCCE |
|  | T ss\_pred |  | HHHHHHHHHHHHHHHHHHHHHHHHHHHHHHhccCCCCcccCCHHHHHHhcCCCCCCCceeeeecCceEEEEEccCCCCee |
|  |
|  |
|  | Q ss\_pred |  | -------------------------------------------------------------------------------- |
|  | Q Q8N394 | 249 | --------------------------------------------------------------------------------   248 (475) |
|  | Q Consensus | 249 | --------------------------------------------------------------------------------   248 (475) |
|  |  |  | .+..-...........+..+...+.-....++ .....+|........+.. .......+ |
|  | T Consensus | 346 | hSh~~~yp~gs~qqqvt~y~~~d~nn~w~i~~~~~~~~~~~~~~~v~~g~~irL~h~~t~~~L~sh~~~~pvs~~~~~~~   425 (817) |
|  | T 6P25\_A | 346 | HSHSHNYPAGSEQQQSTLYPHMDANNDWLLELYNAPGESLTTFQNLTDGTKVRLFHTVTRCRLHSHDHKPPVSESSDWQK   425 (817) |
|  | T ss\_dssp |  | EEEEEECSSTTCCEEEEECCSCCGGGCEEEEECC----CCSCCCBCCTTEEEEEEETTTTBCCBCCSCCCSSCCSCSSCE |
|  | T ss\_pred |  | eeecccCCCCCCCccceeecccCCCCcEEEEECCCCCCCCCCceecCCCCEEEEEeCCCCCceeeCCCCCCCCCCCcccc |
|  |
|  |
|  | Q ss\_pred |  | -------------------------------------------------------------------------------- |
|  | Q Q8N394 | 249 | --------------------------------------------------------------------------------   248 (475) |
|  | Q Consensus | 249 | --------------------------------------------------------------------------------   248 (475) |
|  |  |  | ++..++...+.+++.++.... .++ ..+.. |
|  | T Consensus | 426 | evs~~g~~~~~gd~~d~w~v~i~~~~~~~~~~~~~~~~~~t~frl~h~~~~c~L~s~~~~lP~wg~~q~EV~c~~~~~~~   505 (817) |
|  | T 6P25\_A | 426 | EVSCYGYSGFDGDANDDWVVEIDKKNSAPGVAQERVIALDTKFRLRHAMTGCYLFSHEVKLPAWGFEQQEVTCASSGRHD   505 (817) |
|  | T ss\_dssp |  | ECBBCCBTTTBCCGGGCEEEEECTTTSCSSHHHHSCBTTTCCEEEEETTTCCBCEEEEEEECSSSTTEEEEEECSSBCSG |
|  | T ss\_pred |  | EEEeeccCCCCCCcccceEEEEecccCCCCcccccEEEcCCEEEEEeCCCCeEEEECCCcCCcccccceeeeecCCCCCc |
|  |
|  |
|  | Q ss\_pred |  | ----------CCCCccCCCCCCcCCChHHHHHHHHHHHHHHh-----------HHHhhCcccccccccccc-cccccccC |
|  | Q Q8N394 | 249 | ----------KPPSFSNSDNPAADSDSLLTRTLTFFYLPTKN-----------LWLLLCPDTLSFDWSMDA-VPLLKTVC   306 (475) |
|  | Q Consensus | 249 | ----------~~~~~~~~~~~~~~~~~~~~~~~~~~~~~~~~-----------~~~~~~p~~~~~~~~~~~-~~~~~~~~   306 (475) |
|  |  |  | +..............++..++.++|+ +..++....++.+|++++.+++++..+..+.+...++..+.....+++.+ |
|  | T Consensus | 506 | ~~~w~ie~~~~~~~~~~~~~~~~~~~~f~~kf~e~~~~m~~~n~~l~~~h~~~S~p~~Wp~~~r~i~~w~~~~~~i~~lg   585 (817) |
|  | T 6P25\_A | 506 | LTLWYVENNSNPLLPEDTKRISYKPASFISKFIESHKKMWHINKNLVEPHVYESQPTSWPFLLRGISYWGENNRNVYLLG   585 (817) |
|  | T ss\_dssp |  | GGCEEEEEEECSSSCSSCCEECCCCCCHHHHHHHHHHHHHHHHHSCCCCCTTCCCGGGGGGTCSCEEEEEETTEEEEECC |
|  | T ss\_pred |  | CceEEEecCCCCCCCCCcccccCCCCcHHHHHHHHHHHHHHHHhhcCCCCcccCCccccceeecceeccCCCCceeEecc |
|  |
|  |
|  | Q ss\_pred |  | CHHHHHHHHHHHHHHHHHHHH----hhCcccccccCCCcccCCccccCCcccccccccccccccccccchhhcCCcCCcc |
|  | Q Q8N394 | 307 | DWRNLHTVAFYTGLLLLAYYG----LKSPSVDRECNGKTVTNGKQNANGHSCLSDVEYQNSETKSSFASKVENGIKNDVS   382 (475) |
|  | Q Consensus | 307 | ~~~~~~~~~~~~~~~~~~~~~----~~~~~~~~~~~~~~~~~~~~~~~~~~~~~~~~~~~~~~~~~~~~~~~~~~~~~~~   382 (475) |
|  |  |  | .+....+.... |
|  | T Consensus | 586 | Np~~ww~~~~~~~~~~~~~~~~~~~~~~-----------------------------------------------~~~~~   618 (817) |
|  | T 6P25\_A | 586 | NAIVWWAVTAFIGIFGLIVITELFSWQL-----------------------------------------------GKPIL   618 (817) |
|  | T ss\_dssp |  | CHHHHHHHHHHHHHHHHHHHHHHHHHHH-----------------------------------------------TCCCC |
|  | T ss\_pred |  | cHHHHHHHHHHHHHHHHHHHHHHHHHHc-----------------------------------------------CCCCC |
|  |
|  |
|  | Q ss\_pred |  | cccCCCCCCchHHhhhHHHHHHHhhhcCccccCChhhHHHHhHHHHHHHHHHHHHHHHHHHHHhchHHHHHHHHHHHHHH |
|  | Q Q8N394 | 383 | QRTQLPSTENIVVLSLSLLIIPFVPATNLFFYVGFVIAERVLYIPSMGFCLLITVGARALYVKVQKRFLKSLIFYATATL   462 (475) |
|  | Q Consensus | 383 | ~~~~~~~~~~~~~~~l~~~~~~~~~~~~~~~~~~~~~~~Ry~~~~~~~~~ll~~~~l~~~~~~~~~~~~~~~~~~~~~~~   462 (475) |
|  | T Consensus | 619 | ~~~~~~~~~~~~~~~~~gw~~hy~Pf----~~~~R~~fl~hYlpal~F~il~~~~~~~~~~~~~~~~~~~~~~~~~~~~~   694 (817) |
|  | T 6P25\_A | 619 | KDSKVVNFHVQVIHYLLGFAVHYAPS----FLMQRQMFLHHYLPAYYFGILALGHALDIIVSYVFRSKRQMGYAVVITFL   694 (817) |
|  | T ss\_dssp |  | CSHHHHHHHHHHHHHHHHHHHTTGGG----TSCCSCCCGGGSHHHHHHHHHHHHHHHHHHHHTTTSSCHHHHHHHHHHHH |
|  | T ss\_pred |  | CchhHHHHHHHHHHHHHHHHHHHHHH----HHccCccchhhHHHHHHHHHHHHHHHHHHHHHHhccccchHHHHHHHHHH |
|  |
|  |
|  | Q ss\_pred |  | HHHHHHHHHHh |
|  | Q Q8N394 | 463 | IVFYGLKTAIR   473 (475) |
|  | Q Consensus | 463 | ~~~~~~~~~~~   473 (475) |
|  | T Consensus | 695 | ~~~~~~f~~~~   705 (817) |
|  | T 6P25\_A | 695 | AASVYFFKSFS   705 (817) |
|  | T ss\_dssp |  | HHHHHHHHHSG |
|  | T ss\_pred |  | HHHHHHHHHhh |
|  |
| --- | | | |
|  | Template alignmentTemplate 3D StructurePDBe | | |
| 8. | 6P25\_B Dolichyl-diphosphooligosaccharide--protein glycosyltransferase subunits (E.C.2.4.99.18); complex, TRANSFERASE, glycosylation; HET: NAG, CPL, NNM; 3.2A {Saccharomyces cerevisiae W303}; Related PDB entries: 6P2R\_B ; Related PDB entries: 6P2R\_B ; Related PDB entries: 6P2R\_B | | |
|  | Probability: 99.66%, E-value: 5.8e-12, Score: 133.48, Aligned cols: 383, Identities: 11%, Similarity: 0.066, | | |
|  |
|  | Q ss\_pred |  | HHHHHHHHHHHHHHHhhcCCCCcccchhHHHhchhhCCCCchhHhhhcccccccccCCCCcccCChHHHHHHHHHHHHhC |
|  | Q Q8N394 | 2 | IAELVSSALGLALYLNTLSADFCYDDSRAIKTNQDLLPETPWTHIFYNDFWGTLLTHSGSHKSYRPLCTLSFRLNHAIGG   81 (475) |
|  | Q Consensus | 2 | ~~~lll~~~~~~~~~~~~~~~~~~De~~~~~~~~~~~~~~~~~~~~~~~~~~~~~~~~~~~~~~~Pl~~~l~~~~~~lfG   81 (475) |
|  |  |  | +++++++.+....+.........+||..+...+..+.+++.+.+. +||++.++.+....++| |
|  | T Consensus | 68 | l~~l~~~~~~~r~~~l~~~~~~~~DE~~~~~~a~~~~~~~~~~~~------------------~PPl~~~l~a~~~~l~g   129 (759) |
|  | T 6P25\_B | 68 | PVIFTALALFTRMYKIGINNHVVWDEAHFGKFGSYYLRHEFYHDV------------------HPPLGKMLVGLSGYLAG   129 (759) |
|  | T ss\_dssp |  | HHHHHHHHHHHHSSSGGGSCBCCTTHHHHHHHHHHHHTTBCCCCS------------------SCTHHHHHHHHHHHTTT |
|  | T ss\_pred |  | HHHHHHHHHHHHHHhcCCCCceeeeHHHHHHHHHHHHhCCCCcCC------------------CCHHHHHHHHHHHHHhC |
|  |
|  |
|  | Q ss\_pred |  | CCh----------------HHHHHHHHHHHHHHHHHHHHHHHHHhCChHHHHHHHHHHHHCHHhHHHHHhhhchHHHHHH |
|  | Q Q8N394 | 82 | LNP----------------WSYHLVNVLLHAAVTGLFTSFSKILLGDGYWTFMAGLMFASHPIHTEAVAGIVGRADVGAS   145 (475) |
|  | Q Consensus | 82 | ~~~----------------~~~rl~~~l~~~l~~~lly~l~r~l~~~~~~al~aall~a~~P~~~~~~~~~~~~~~~~~~   145 (475) |
|  |  |  | .+. ...|++++++++++++++|.++|++..++..|+++++++++.|.....+..... |.+.. |
|  | T Consensus | 130 | ~~~~~~~~~~~~~~~~~~~~~~R~~~~l~~~l~~~l~y~l~r~l~~~~~~allaall~~~~p~~~~~s~~~~~--d~~~~   207 (759) |
|  | T 6P25\_B | 130 | YNGSWDFPSGEIYPDYLDYVKMRLFNASFSALCVPLAYFTAKAIGFSLPTVWLMTVLVLFENSYSTLGRFILL--DSMLL   207 (759) |
|  | T ss\_dssp |  | CCSCSCCCSSCBCCSSCCHHHHHHHHHHHHHHHHHHHHHHHHHSCCCTHHHHHHHHHHHSCHHHHHHTSSSCS--HHHHH |
|  | T ss\_pred |  | CCCCCCCCCCCCCCCcCCHHHHHHHHHHHHHHHHHHHHHHHHHcCCCHHHHHHHHHHHHhhhHHHHHHHHHHH--HHHHH |
|  |
|  |
|  | Q ss\_pred |  | HHHHHHHHHHHHHHcc-------CCCCcchHHHHHHHHHHHHHHHHhHhHHHHHHHHHHHHHHH--HHcccchhhhcchH |
|  | Q Q8N394 | 146 | LFFLLSLLCYIKHCST-------RGYSARTWGWFLGSGLCAGCSMLWKEQGVTVLAVSAVYDVF--VFHRLKIKQILPTI   216 (475) |
|  | Q Consensus | 146 | ~f~ll~l~~~l~~~~~-------~~~~~~~~~~~~l~~l~~~la~ltk~~~~~~~~~~~~~~l~--~~~~~~~~~~~~~~   216 (475) |
|  |  |  | ++++++++++.+..++ + ++.+++++++++++++++|..++.+++.++++.+. ....++.+...+.. |
|  | T Consensus | 208 | ~f~~l~l~~~~~~~~~~~~~~~~~-----~~~~~~l~gl~lgla~~~K~~~~~~~~~~~l~~l~~~~~~~~~~~~~~~~~   282 (759) |
|  | T 6P25\_B | 208 | FFTVASFFSFVMFHNQRSKPFSRK-----WWKWLLITGISLGCTISVKMVGLFIITMVGIYTVIDLWTFLADKSMSWKTY   282 (759) |
|  | T ss\_dssp |  | HHHHHHHHHHHHHHTTSSSTTSHH-----HHHHHHHHHHHHHHHHHHCGGGHHHHHHHHHHHHHHHHHHTTCSSSCHHHH |
|  | T ss\_pred |  | HHHHHHHHHHHHHhhhcCCCCCHH-----HHHHHHHHHHHHHHHHHhHHHHHHHHHHHHHHHHHHHHHHhcCCCCCHHHH |
|  |
|  |
|  | Q ss\_pred |  | hhhHHHHHHHHHHHHHHHHHHHHHHHHHHhcC------------------------------------------------ |
|  | Q Q8N394 | 217 | YKRKNLSLFLSISLLIFWGSSLLGARLYWMGN------------------------------------------------   248 (475) |
|  | Q Consensus | 217 | ~~~~~~~~~~~~~~~~~~~~~~~~~~~~~~~~------------------------------------------------   248 (475) |
|  |  |  | .+.........+++.+++....+..++..... |
|  | T Consensus | 283 | ~~~~~~~~~~~~~~p~~i~~~~~~~~~~~~~~~g~~~~~~s~~f~~~l~g~~~~~~~~~v~~gs~~vti~~~~~~~~yl~   362 (759) |
|  | T 6P25\_B | 283 | INHWLARIFGLIIVPFCIFLLCFKIHFDLLSHSGTGDANMPSLFQARLVGSDVGQGPRDIALGSSVVSIKNQALGGSLLH   362 (759) |
|  | T ss\_dssp |  | HHHHHHHHCCCCCHHHHHHHHHHHHHHHHCCBCCTTGGGSCHHHHHHSSSCTTCCSSCCCCSSSCEEEEEECSTTCCEEE |
|  | T ss\_pred |  | HHHHHHHHHHHHHHHHHHHHHHHHHHHHhhcCCCCCcccCChHHHHHHCCCCCCCCCceeecCCeeEEEEeCCCCCceeE |
|  |
|  |
|  | Q ss\_pred |  | -------------------------------------------------------------------------------- |
|  | Q Q8N394 | 249 | --------------------------------------------------------------------------------   248 (475) |
|  | Q Consensus | 249 | --------------------------------------------------------------------------------   248 (475) |
|  |  |  | ....-...........+..+...+.-.....+-...... ...+..+.+.... .. |
|  | T Consensus | 363 | s~~~~yp~~~~~q~vt~~~~~d~n~~w~i~~~~~~~~~~~~~~~~~~i~~gd~irL~h~~t~~~L~~~~~~~p~s~~~~e   442 (759) |
|  | T 6P25\_B | 363 | SHIQTYPDGSNQQQVTCYGYKDANNEWFFNRERGLPSWSENETDIEYLKPGTSYRLVHKSTGRNLHTHPVAAPVSKTQWE   442 (759) |
|  | T ss\_dssp |  | EEEEECSSSSSCEEEEEECSCCGGGCEEEECCTTSCCCCTTCCCCCCCCTTSEEEEEESSSCCEEEEEEEECSSCSSSEE |
|  | T ss\_pred |  | EecccCCCCCccceeEecceecCCCeEEEEcCCCCCCCCCCcccceecCCCCEEEEEECCCCCcceeCCCCCCCCCCcee |
|  |
|  |
|  | Q ss\_pred |  | -------------------------------------------------------------------------------- |
|  | Q Q8N394 | 249 | --------------------------------------------------------------------------------   248 (475) |
|  | Q Consensus | 249 | --------------------------------------------------------------------------------   248 (475) |
|  |  |  | ...+++..++...+.+++ ........++ |
|  | T Consensus | 443 | vs~~~~~~~~d~~d~w~i~~~~~~~~~~~~~~~~~~t~frl~~~~~~c~L~~~~~~lp~wg~~q~Ev~c~~~~~~~~~~~   522 (759) |
|  | T 6P25\_B | 443 | VSGYGDNVVGDNKDNWVIEIMDQRGDEDPEKLHTLTTSFRIKNLEMGCYLAQTGNSLPEWGFRQQEVVCMKNPFKRDKRT   522 (759) |
|  | T ss\_dssp |  | EEEECCSSSSCGGGCEEEEEEEECSSSCTTSCCSSSEEEEEEESSSCCEEEEEEEECCGGGTSCEEEEEESSCCTTCSTT |
|  | T ss\_pred |  | EEEecCCCCCCcccCeEEEEecCCCCCCccceEEeeeEEEEEeCCCCeEEEEcCCCCCccccccceeecccCCCcCCcCc |
|  |
|  |
|  | Q ss\_pred |  | --------CCCCccCCCCCCcCCChHHHHHHHHHHHHHHhHHHhhCc-----ccccccccccccc-------------cc |
|  | Q Q8N394 | 249 | --------KPPSFSNSDNPAADSDSLLTRTLTFFYLPTKNLWLLLCP-----DTLSFDWSMDAVP-------------LL   302 (475) |
|  | Q Consensus | 249 | --------~~~~~~~~~~~~~~~~~~~~~~~~~~~~~~~~~~~~~~p-----~~~~~~~~~~~~~-------------~~   302 (475) |
|  |  |  | ...... .+.++..++.++|+ +..++....+|.++++++.+++++..+..+.++. |
|  | T Consensus | 523 | ~w~i~~~~~~~~~~~~~~~~~~~~~f~~~~~e~~~~m~~~~~~l~~~~~~~h~~~S~p~~Wp~~~~~~~~~~~~~~~~~i   602 (759) |
|  | T 6P25\_B | 523 | WWNIETHENERLPPRPEDFQYPKTNFLKDFIHLNLAMMATNNALVPDPDKFDYLASSAWQWPTLNVGLRLCGWGDDNPKY   602 (759) |
|  | T ss\_dssp |  | CEEEEEEEC--------CCCCCCCCHHHHHHHHHHHHHHHHHTTCCCTTBCCSSCBCGGGTTTTCCCEECSCCSSSSCCC |
|  | T ss\_pred |  | eeEEeccccCCCCCCCCcCCCCCCcHHHHHHHHHHHHHHHhhcCCCCccccCcccCChhHcccccCccccccCCCCCCeE |
|  |
|  |
|  | Q ss\_pred |  | cccCCHHHHHHHHHHHHH----HHHHHHHhhCcccccccCCCcccCCccccCCcccccccccccccccccccchhhcCCc |
|  | Q Q8N394 | 303 | KTVCDWRNLHTVAFYTGL----LLLAYYGLKSPSVDRECNGKTVTNGKQNANGHSCLSDVEYQNSETKSSFASKVENGIK   378 (475) |
|  | Q Consensus | 303 | ~~~~~~~~~~~~~~~~~~----~~~~~~~~~~~~~~~~~~~~~~~~~~~~~~~~~~~~~~~~~~~~~~~~~~~~~~~~~~   378 (475) |
|  |  |  | + ++..+..+..+++++.+...+... |
|  | T Consensus | 603 | ~~~gNp~iww~~~~~~~~~~~~~~~~~~~~~r------------------------------------------------   634 (759) |
|  | T 6P25\_B | 603 | FLLGTPASTWASSVAVLAFMATVVILLIRWQR------------------------------------------------   634 (759) |
|  | T ss\_dssp |  | EECCCTTHHHHHHHHHHHHHHHHHHHHHHHHT------------------------------------------------ |
|  | T ss\_pred |  | EEeCCHHHHHHHHHHHHHHHHHHHHHHHHHhc------------------------------------------------ |
|  |
|  |
|  | Q ss\_pred |  | CCcccccCCCCCCchH------------HhhhHHHHHHHhhhcCccccCChhhHHHHhHHHHHHHHHHHHHHHHHHHHHh |
|  | Q Q8N394 | 379 | NDVSQRTQLPSTENIV------------VLSLSLLIIPFVPATNLFFYVGFVIAERVLYIPSMGFCLLITVGARALYVKV   446 (475) |
|  | Q Consensus | 379 | ~~~~~~~~~~~~~~~~------------~~~l~~~~~~~~~~~~~~~~~~~~~~~Ry~~~~~~~~~ll~~~~l~~~~~~~   446 (475) |
|  | T Consensus | 635 | ----------~~~~~~~~~~~~~~~~~~~~~~~g~~~~ylP~----~~~~R~~fl~hYlpal~f~il~~~~~~~~~~~~~   700 (759) |
|  | T 6P25\_B | 635 | ----------QYVDLRNPSNWNVFLMGGFYPLLAWGLHYMPF----VIMSRVTYVHHYLPALYFALIILAYCFDAGLQKW   700 (759) |
|  | T ss\_dssp |  | ----------TCCCCCSHHHHHCCCCCCCHHHHHHHHHHHHH----HHSCSCBCGGGHHHHHHHHHHHHHHHHHTSSSGG |
|  | T ss\_pred |  | ----------CCCCCCCchhHHHHHHHhHHHHHHHHHhcchH----HcccCcccHHHHHHHHHHHHHHHHHHHHHHHHhc |
|  |
|  |
|  | Q ss\_pred |  | c----hHHHHHHHHHHHHHHHHHHHHHHH |
|  | Q Q8N394 | 447 | Q----KRFLKSLIFYATATLIVFYGLKTA   471 (475) |
|  | Q Consensus | 447 | ~----~~~~~~~~~~~~~~~~~~~~~~~~   471 (475) |
|  | T Consensus | 701 | ~~~~~~~~~~~~~~~~~~~~~~~~f~~~~   729 (759) |
|  | T 6P25\_B | 701 | SRSKCGRIMRFVLYAGFMALVIGCFWYFS   729 (759) |
|  | T ss\_dssp |  | GGSHHHHHHHHHHHHHHHHHHHHHHHHTT |
|  | T ss\_pred |  | ccccccHHHHHHHHHHHHHHHHHHHHHHH |
|  |
| --- | | | |
|  | Template alignmentTemplate 3D StructurePDBe | | |
| 9. | 7BVF\_A Probable arabinosyltransferase B (E.C.2.4.2.-), Probable; Mycobacterium tuberculosis, cell wall synthesis; HET: 95E, DSL, CDL;{Mycolicibacterium smegmatis MC2 155} | | |
|  | Probability: 99.52%, E-value: 1.2e-10, Score: 123.82, Aligned cols: 356, Identities: 11%, Similarity: -0.024, | | |
|  |
|  | Q ss\_pred |  | HHHHHHHHHHhhcCCCCcccchhHHHhchhhCCCCchhHhhhcccccccccCCCCcccCChHHHHHHHHHHHHhCCChHH |
|  | Q Q8N394 | 7 | SSALGLALYLNTLSADFCYDDSRAIKTNQDLLPETPWTHIFYNDFWGTLLTHSGSHKSYRPLCTLSFRLNHAIGGLNPWS   86 (475) |
|  | Q Consensus | 7 | l~~~~~~~~~~~~~~~~~~De~~~~~~~~~~~~~~~~~~~~~~~~~~~~~~~~~~~~~~~Pl~~~l~~~~~~lfG~~~~~   86 (475) |
|  |  |  | .++.+.+..+.... +...||.++...+++..+.+.+.+++..- +..+.++|+|++++..+..+ |.++.+ |
|  | T Consensus | 263 | ~~V~~~l~~w~~~g-p~~~DDg~~~~~Ar~~~~~G~~~n~~~~~---------~~~e~p~~lyY~lL~~W~~v-G~s~~~   331 (1102) |
|  | T 7BVF\_A | 263 | AAVIATLLLWHVIG-ATSSDDGYLLTVARVAPKAGYVANYYRYF---------GTTEAPFDWYTSVLAQLAAV-STAGVW   331 (1102) |
|  | T ss\_dssp |  | HHHHHHHHHTTTSC-CCCSTTHHHHHHHHHHHHHTSCBCSSSGG---------GCBCCTTCTTHHHHHHHHHH-CCCHHH |
|  | T ss\_pred |  | HHHHHHHHHHHHhc-ccCCcchHHHHHHhhccccCcHHHHHHHH---------CCCCCCChHHHHHHHHHHhc-cCchHH |
|  |
|  |
|  | Q ss\_pred |  | HHHHHHHHHHHHHHHHHHHHHHHhCCh--------HHHHHHHHHHHHCHHhHHHHHhhhchHHHHHHHHHHHHHHHHHHH |
|  | Q Q8N394 | 87 | YHLVNVLLHAAVTGLFTSFSKILLGDG--------YWTFMAGLMFASHPIHTEAVAGIVGRADVGASLFFLLSLLCYIKH   158 (475) |
|  | Q Consensus | 87 | ~rl~~~l~~~l~~~lly~l~r~l~~~~--------~~al~aall~a~~P~~~~~~~~~~~~~~~~~~~f~ll~l~~~l~~   158 (475) |
|  |  |  | .|++|+++++++..++++..+...+++ ..++.+++++.. .++.+.+.. |+|.+..++.+++++++.+. |
|  | T Consensus | 332 | LRLpSvlagl~t~~ll~r~v~~~lgr~~~~l~~~~~a~~~aal~~la--~~l~y~~~~--Rpyal~al~~~la~~~~~ra   407 (1102) |
|  | T 7BVF\_A | 332 | MRLPATLAGIACWLIVSRFVLRRLGPGPGGLASNRVAVFTAGAVFLS--AWLPFNNGL--RPEPLIALGVLVTWVLVERS   407 (1102) |
|  | T ss\_dssp |  | HTSHHHHHHHHHHHHCCCCCHHHSCCSSSSSSSCSHHHHHHHHHHHH--HHTTTCCSS--SSHHHHHHHHHHHHHHHHHH |
|  | T ss\_pred |  | hHHHHHHHHHHHHHHHHHHHHHHhCCCCCCccccHHHHHHHHHHHHH--HHHHHhcCC--ChHHHHHHHHHHHHHHHHHH |
|  |
|  |
|  | Q ss\_pred |  | HccCCCCcchHHHHHHHHHHHHHHHHhHhHHHHHHHHHHHHHHHHHcccchhhhcchHhhh-HHHHHHHHHHHHHHHHHH |
|  | Q Q8N394 | 159 | CSTRGYSARTWGWFLGSGLCAGCSMLWKEQGVTVLAVSAVYDVFVFHRLKIKQILPTIYKR-KNLSLFLSISLLIFWGSS   237 (475) |
|  | Q Consensus | 159 | ~~~~~~~~~~~~~~~l~~l~~~la~ltk~~~~~~~~~~~~~~l~~~~~~~~~~~~~~~~~~-~~~~~~~~~~~~~~~~~~   237 (475) |
|  |  |  | .+++ +..++.+..++.+++..+|+++++.+....+......+..+.+. + .........++....... |
|  | T Consensus | 408 | ~~~~-----r~~~~al~~~~a~lal~~hptgll~laall~~~~~l~r~lr~r~-------~~~~~~~~la~vla~~~~~l   475 (1102) |
|  | T 7BVF\_A | 408 | IALG-----RLAPAAVAIIVATLTATLAPQGLIALAPLLTGARAIAQRIRRRR-------ATDGLLAPLAVLAAALSLIT   475 (1102) |
|  | T ss\_dssp |  | HHHT-----CSHHHHHHHHHHHHHTTSCGGGGGGGHHHHHTTHHHHHHHHHSC-------SSSCSSHHHHHHHHTGGGTH |
|  | T ss\_pred |  | HHcC-----ChHHHHHHHHHHHHHhccchHHHHHHHHHHHHHHHHHHHHHHhh-------hhcchHHHHHHHHHHHHHHH |
|  |
|  |
|  | Q ss\_pred |  | HHHHHHHHhcCCCCCccCCCCCCcCCChHHHHHHHHHHHHHHhHHHhhCcccccccccccccc-cccccCCHHHHHHHHH |
|  | Q Q8N394 | 238 | LLGARLYWMGNKPPSFSNSDNPAADSDSLLTRTLTFFYLPTKNLWLLLCPDTLSFDWSMDAVP-LLKTVCDWRNLHTVAF   316 (475) |
|  | Q Consensus | 238 | ~~~~~~~~~~~~~~~~~~~~~~~~~~~~~~~~~~~~~~~~~~~~~~~~~p~~~~~~~~~~~~~-~~~~~~~~~~~~~~~~   316 (475) |
|  |  |  | ...+. +.......+.-+....+-...-+......++...... ...+......+...++ |
|  | T Consensus | 476 | ~~~F~---------------------dq~l~~~~~a~~~~~~~g~~~~W~~e~~Ry~~L~~~~~~~Gs~arr~pvLl~l~   534 (1102) |
|  | T 7BVF\_A | 476 | VVVFR---------------------DQTLATVAESARIKYKVGPTIAWYQDFLRYYFLTVESNVEGSMSRRFAVLVLLF   534 (1102) |
|  | T ss\_dssp |  | HHHSS---------------------SSCHHHHHHHHHHHHHSSCCCCGGGTTTTSTTTSCSSCGGGCTTTHHHHHHHHH |
|  | T ss\_pred |  | HHHHc---------------------cCcHHHHHHHHhHHHhhCCCChhHHHHHHHHHHHhccCCCCCchHHHHHHHHHH |
|  |
|  |
|  | Q ss\_pred |  | HHHHHHHHHHHhhCcccccccCCCcccCCccccCCcccccccccccccccccccchhhcCCcCCcccccCCCCCC---ch |
|  | Q Q8N394 | 317 | YTGLLLLAYYGLKSPSVDRECNGKTVTNGKQNANGHSCLSDVEYQNSETKSSFASKVENGIKNDVSQRTQLPSTE---NI   393 (475) |
|  | Q Consensus | 317 | ~~~~~~~~~~~~~~~~~~~~~~~~~~~~~~~~~~~~~~~~~~~~~~~~~~~~~~~~~~~~~~~~~~~~~~~~~~~---~~   393 (475) |
|  |  |  | .+..+......+++ ... .. |
|  | T Consensus | 535 | ~L~~~~~~l~Rrrr----------------------------------------------------------~~g~~~~~   556 (1102) |
|  | T 7BVF\_A | 535 | CLFGVLFVLLRRGR----------------------------------------------------------VAGLASGP   556 (1102) |
|  | T ss\_dssp |  | HHHHHHHHHHSSSC----------------------------------------------------------CSSSCHHH |
|  | T ss\_pred |  | HHHHHHHHHHHcCC----------------------------------------------------------CCCcccCH |
|  |
|  |
|  | Q ss\_pred |  | HHhhhHHHHHHHhhhcCccccCChhhHHHHhHHHHHHHHHHHHHHHHHHHHHhchHHHHHHHHHHHHHHHHHHHHHHHHh |
|  | Q Q8N394 | 394 | VVLSLSLLIIPFVPATNLFFYVGFVIAERVLYIPSMGFCLLITVGARALYVKVQKRFLKSLIFYATATLIVFYGLKTAIR   473 (475) |
|  | Q Consensus | 394 | ~~~~l~~~~~~~~~~~~~~~~~~~~~~~Ry~~~~~~~~~ll~~~~l~~~~~~~~~~~~~~~~~~~~~~~~~~~~~~~~~~   473 (475) |
|  |  |  | ...++++.++.++.+++. ..+..||+....++..++++.++..+.+...+....+......+++++.++.....+ |
|  | T Consensus | 557 | ~~~ll~~~~l~lvll~~t-----PsKwt~hfg~~A~~~~aLlA~~~v~~~~~~~r~~r~~~~~~~~~~~~~als~~g~n~   631 (1102) |
|  | T 7BVF\_A | 557 | AWRLIGTTAVGLLLLTFT-----PTKWAVQFGAFAGLAGVLGAVTAFTFARIGLHSRRNLTLYVTALLFVLAWATSGING   631 (1102) |
|  | T ss\_dssp |  | HHHHHHHHHHHHHHGGGC-----SCCCSGGGGGGHHHHHHHHHHHHHHHHHTTSSCSHHHHHHHHHHHHHHHHHTTSCCC |
|  | T ss\_pred |  | HHHHHHHHHHHHHHHhcC-----ccHHHHHHHHHHHHHHHHHHHHHHHHhhhhhcchHHHHHHHHHHHHHHHHHHhhhcc |
|  |
| --- | | | |
|  | Template alignmentTemplate 3D StructurePDBe | | |
| 10. | 7BWR\_A Integral membrane indolylacetylinositol arabinosyltransferase EmbB; Mycobacterium tuberculosis, EmbB, cryo-EM, ethambutol; HET: F8L;{Mycolicibacterium smegmatis MC2 155}; Related PDB entries: 7BVC\_B 7BVG\_B 7BWR\_B 7BX8\_B 7BX8\_A | | |
|  | Probability: 99.49%, E-value: 2e-10, Score: 121.77, Aligned cols: 351, Identities: 8%, Similarity: -0.086, | | |
|  |
|  | Q ss\_pred |  | HHHHHHHHHhhcCCCCcccchhHHHhchhhCCCCchhHhhhcccccccccCCCCcccCChHHHHHHHHHHHHhCCChHHH |
|  | Q Q8N394 | 8 | SALGLALYLNTLSADFCYDDSRAIKTNQDLLPETPWTHIFYNDFWGTLLTHSGSHKSYRPLCTLSFRLNHAIGGLNPWSY   87 (475) |
|  | Q Consensus | 8 | ~~~~~~~~~~~~~~~~~~De~~~~~~~~~~~~~~~~~~~~~~~~~~~~~~~~~~~~~~~Pl~~~l~~~~~~lfG~~~~~~   87 (475) |
|  |  |  | .++...+.....-.+...||.++...+++..+.+...+++..- +..+.+++++++++..+..+ |.++.+. |
|  | T Consensus | 267 | ~~V~a~L~~w~~ig~~~~DDg~~~~~ar~~~~~G~~~n~~r~~---------~~~e~p~~~yY~lL~~w~~l-G~s~~~l   336 (1082) |
|  | T 7BWR\_A | 267 | GVVVGGMAIWYVIGANSSDDGYILQMARTAEHAGYMANYFRWF---------GSPEDPFGWYYNVLALMTKV-SDASIWI   336 (1082) |
|  | T ss\_dssp |  | HHHTTTTTHHHHHSCCCTTHHHHHHHHHSHHHHSSCBCCSSST---------TCBSCSSCCTHHHHHTTTTS-CCCTTTT |
|  | T ss\_pred |  | HHHHHHHHHHHHhccCCCchHHHHHHHHHHhHhcchHHHHHHh---------CCCCCccHHHHHHHHHHHHh-cCChHHH |
|  |
|  |
|  | Q ss\_pred |  | HHHHHHHHHHHHHHH-----HHHHHHHhCChHHHHHHHHHHHHCHHhHHHHHhhhchHHHHHHHHHHHHHHHHHHHHccC |
|  | Q Q8N394 | 88 | HLVNVLLHAAVTGLF-----TSFSKILLGDGYWTFMAGLMFASHPIHTEAVAGIVGRADVGASLFFLLSLLCYIKHCSTR   162 (475) |
|  | Q Consensus | 88 | rl~~~l~~~l~~~ll-----y~l~r~l~~~~~~al~aall~a~~P~~~~~~~~~~~~~~~~~~~f~ll~l~~~l~~~~~~   162 (475) |
|  |  |  | |++++++++++..++ +.+.++..+++..++.+++.+.. .+..+.+.. |+|.+..++.+++++++.+..+++ |
|  | T Consensus | 337 | RLpS~laglat~~ll~r~v~~~lgr~~~~~~~a~~~Aal~~l~--~~l~y~~~~--Rpy~l~al~~~la~~~l~ra~~~~   412 (1082) |
|  | T 7BWR\_A | 337 | RLPDLICALICWLLLSREVLPRLGPAVAGSRAAMWAAGLVLLG--AWMPFNNGL--RPEGQIATGALITYVLIERAVTSG   412 (1082) |
|  | T ss\_dssp |  | TGGGTGGGTSSHHHHTTSSTGGGCHHHHHCSHHHHHHHHHHHH--HHTTTTTSS--STTHHHHHHHHHHHHHHHHHHHHC |
|  | T ss\_pred |  | HHHHHHHHHHHHHHHHHhhHHHhchhhcccHHHHHHHHHHHHH--HHHHHhcCC--chHHHHHHHHHHHHHHHHHHHhcC |
|  |
|  |
|  | Q ss\_pred |  | CCCcchHHHHHHHHHHHHHHHHhHhHHHHHHHHHHHHH-HHHHcccchhhhcchHhhhHHHHHHHHHHHHHHHHHHHHHH |
|  | Q Q8N394 | 163 | GYSARTWGWFLGSGLCAGCSMLWKEQGVTVLAVSAVYD-VFVFHRLKIKQILPTIYKRKNLSLFLSISLLIFWGSSLLGA   241 (475) |
|  | Q Consensus | 163 | ~~~~~~~~~~~l~~l~~~la~ltk~~~~~~~~~~~~~~-l~~~~~~~~~~~~~~~~~~~~~~~~~~~~~~~~~~~~~~~~   241 (475) |
|  |  |  | +..++.+.+++.++++.+|+++++.+....+.. .+.++.++... ...........+.......+..+ |
|  | T Consensus | 413 | -----r~~~~al~~~~a~lal~~hptgllalaallv~l~~l~r~~r~r~~-------~~~~la~la~~~aa~~~~l~~~F   480 (1082) |
|  | T 7BWR\_A | 413 | -----RLTPAALAITTAAFTLGIQPTGLIAVAALLAGGRPILRIVMRRRR-------LVGTWPLIAPLLAAGTVILAVVF   480 (1082) |
|  | T ss\_dssp |  | -----CSHHHHHHHHHHHHTTSSCTTCHHHHHHHHHTHHHHHHHHHHTTT-------TSCSHHHHHHHHHTTSSTHHHHT |
|  | T ss\_pred |  | -----CCHHHHHHHHHHHHHHHhcHHHHHHHHHHHHccHHHHHHHhhhhc-------cccHHHHHHHHHHHHHHHHHHHH |
|  |
|  |
|  | Q ss\_pred |  | HHHHhcCCCCCccCCCCCCcCCChHHHHHHHHHHHHHHhHHHhhCcccccccccccccccccccCCHHHHHHHHHHHHHH |
|  | Q Q8N394 | 242 | RLYWMGNKPPSFSNSDNPAADSDSLLTRTLTFFYLPTKNLWLLLCPDTLSFDWSMDAVPLLKTVCDWRNLHTVAFYTGLL   321 (475) |
|  | Q Consensus | 242 | ~~~~~~~~~~~~~~~~~~~~~~~~~~~~~~~~~~~~~~~~~~~~~p~~~~~~~~~~~~~~~~~~~~~~~~~~~~~~~~~~   321 (475) |
|  |  |  | . +.......+..+...+.-...-+.....-++.........+..........++.++.+ |
|  | T Consensus | 481 | ~---------------------dqsl~~~~~a~~~~~~~g~~~~W~~e~~Ry~~L~~~~~~Gs~arr~~vLl~l~~L~~~   539 (1082) |
|  | T 7BWR\_A | 481 | A---------------------DQTIATVLEATRIRTAIGPSQEWWTENLRYYYLILPTTDGAISRRVAFVFTAMCLFPS   539 (1082) |
|  | T ss\_dssp |  | S---------------------SCCHHHHHHHHHHHHHHSCCTTTSGGGSSSSCCCCTTCCCHHHHHHTTTTTTTSSHHH |
|  | T ss\_pred |  | h---------------------chHHHHHHHHHHHHhhcCCCccHHHhhHHHHHHHcCCCCCcchHHHHHHHHHHHHHHH |
|  |
|  |
|  | Q ss\_pred |  | HHHHHHhhCcccccccCCCcccCCccccCCcccccccccccccccccccchhhcCCcCCcccccCCCCCC---chHHhhh |
|  | Q Q8N394 | 322 | LLAYYGLKSPSVDRECNGKTVTNGKQNANGHSCLSDVEYQNSETKSSFASKVENGIKNDVSQRTQLPSTE---NIVVLSL   398 (475) |
|  | Q Consensus | 322 | ~~~~~~~~~~~~~~~~~~~~~~~~~~~~~~~~~~~~~~~~~~~~~~~~~~~~~~~~~~~~~~~~~~~~~~---~~~~~~l   398 (475) |
|  |  |  | ......+++ ... .....++ |
|  | T Consensus | 540 | ~~~llR~~r----------------------------------------------------------~~g~~~~~~~~ll   561 (1082) |
|  | T 7BWR\_A | 540 | LFMMLRRKH----------------------------------------------------------IAGVARGPAWRLM   561 (1082) |
|  | T ss\_dssp |  | HHHHHHSSS----------------------------------------------------------CSSSCTTHHHHHH |
|  | T ss\_pred |  | HHHHHhhcc----------------------------------------------------------cCCCcCCHHHHHH |
|  |
|  |
|  | Q ss\_pred |  | HHHHHHHhhhcCccccCChhhHHHHhHHHHHHHHHHHHHHHHHHHHHhchHHHHHHHHHHHHHHHHHHHH |
|  | Q Q8N394 | 399 | SLLIIPFVPATNLFFYVGFVIAERVLYIPSMGFCLLITVGARALYVKVQKRFLKSLIFYATATLIVFYGL   468 (475) |
|  | Q Consensus | 399 | ~~~~~~~~~~~~~~~~~~~~~~~Ry~~~~~~~~~ll~~~~l~~~~~~~~~~~~~~~~~~~~~~~~~~~~~   468 (475) |
|  |  |  | ...+..++...+. ..+..+|+.........+++..+..+.....+....+......++++...+. |
|  | T Consensus | 562 | ~~~~~~~~ll~~t-----PtKwthhfga~ag~g~~l~a~a~v~l~~~~~r~~r~r~~~~~~~~~~~ala~   626 (1082) |
|  | T 7BWR\_A | 562 | GIIFATMFFLMFT-----PTKWIHHFGLFAAVGGAMAALATVLVSPTVLRSARNRMAFLSLVLFVLAFCF   626 (1082) |
|  | T ss\_dssp |  | HHHHHHHHHTBTT-----TBCCCCCTTGGGSHHHHHHHHHHHHSSTTTCCCHHHHHHHHHHHHHHHHHHT |
|  | T ss\_pred |  | HHHHHHHHHHhhC-----cchHHHHHHHHHHHHHHHHHHHHHHHcHHhcccHHHHHHHHHHHHHHHHHHh |
|  |
| --- | | | |
|  | Template alignmentTemplate 3D StructurePDBe | | |
| 11. | 7BVF\_B Probable arabinosyltransferase B (E.C.2.4.2.-), Probable; Mycobacterium tuberculosis, cell wall synthesis; HET: 95E, DSL, CDL;{Mycolicibacterium smegmatis MC2 155} | | |
|  | Probability: 99.47%, E-value: 2.6e-10, Score: 121.64, Aligned cols: 354, Identities: 9%, Similarity: -0.093, | | |
|  |
|  | Q ss\_pred |  | HHHHHHHHHHhhcCCCCcccchhHHHhchhhCCCCchhHhhhcccccccccCCCCcccCChHHHHHHHHHHHHhCCChHH |
|  | Q Q8N394 | 7 | SSALGLALYLNTLSADFCYDDSRAIKTNQDLLPETPWTHIFYNDFWGTLLTHSGSHKSYRPLCTLSFRLNHAIGGLNPWS   86 (475) |
|  | Q Consensus | 7 | l~~~~~~~~~~~~~~~~~~De~~~~~~~~~~~~~~~~~~~~~~~~~~~~~~~~~~~~~~~Pl~~~l~~~~~~lfG~~~~~   86 (475) |
|  |  |  | +.+.+.+..+.... +.+.||.++...+.+..+.+.+.+++..- +..+.+++++++++..+..+ |.++.+ |
|  | T Consensus | 281 | ~~V~~~L~~w~~~g-~~~~DDg~~~~~ar~~~~~G~~~n~~~~~---------~~~e~p~~~yY~lL~~w~~l-G~s~~~   349 (1116) |
|  | T 7BVF\_B | 281 | AVVIFGFLLWHVIG-ANSSDDGYILGMARVADHAGYMSNYFRWF---------GSPEDPFGWYYNLLALMTHV-SDASLW   349 (1116) |
|  | T ss\_dssp |  | HHHHHHHHHHHHHC-CCCSSHHHHHHHHHTTTTSSSCBCCSSSS---------SCBCCSSCSSHHHHHHHTTT-CCCHHH |
|  | T ss\_pred |  | HHHHHHHHHHHHhC-cCCCCchHHHHHhhccccccchHHHHHHh---------CCCCCccHHHHHHHHHHHHc-CCchHH |
|  |
|  |
|  | Q ss\_pred |  | HHHHHHHHHHHHHHHHH-----HHHHHHhCChHHHHHHHHHHHH--CHHhHHHHHhhhchHHHHHHHHHHHHHHHHHHHH |
|  | Q Q8N394 | 87 | YHLVNVLLHAAVTGLFT-----SFSKILLGDGYWTFMAGLMFAS--HPIHTEAVAGIVGRADVGASLFFLLSLLCYIKHC   159 (475) |
|  | Q Consensus | 87 | ~rl~~~l~~~l~~~lly-----~l~r~l~~~~~~al~aall~a~--~P~~~~~~~~~~~~~~~~~~~f~ll~l~~~l~~~   159 (475) |
|  |  |  | .|++++++++++..+++ .+.++..+++..++.+++++.. .|+..+. |+|.+..++.+++++++.+.. |
|  | T Consensus | 350 | lRlpSllagl~t~~ll~r~vl~~lg~~~~~~~~a~~~aal~~l~~~lpy~~~~------Rpyal~al~~~lal~~~~ra~   423 (1116) |
|  | T 7BVF\_B | 350 | MRLPDLAAGLVCWLLLSREVLPRLGPAVEASKPAYWAAAMVLLTAWMPFNNGL------RPEGIIALGSLVTYVLIERSM   423 (1116) |
|  | T ss\_dssp |  | HTSHHHHHHHHHHHHCCCCCGGGSCTTTSSCHHHHHHHHHHHHHHHSSSTTSS------STHHHHHHHHHHHHHHHHHHH |
|  | T ss\_pred |  | hhHHHHHHHHHHHHHHHHHHHHHhchhhcCCHHHHHHHHHHHHHHhchhcCCC------cHHHHHHHHHHHHHHHHHHHh |
|  |
|  |
|  | Q ss\_pred |  | ccCCCCcchHHHHHHHHHHHHHHHHhHhHHHHHHHHHHHHHHHHHcccchhhhcchHhhh-HHHHHHHHHHHHHHHHHHH |
|  | Q Q8N394 | 160 | STRGYSARTWGWFLGSGLCAGCSMLWKEQGVTVLAVSAVYDVFVFHRLKIKQILPTIYKR-KNLSLFLSISLLIFWGSSL   238 (475) |
|  | Q Consensus | 160 | ~~~~~~~~~~~~~~l~~l~~~la~ltk~~~~~~~~~~~~~~l~~~~~~~~~~~~~~~~~~-~~~~~~~~~~~~~~~~~~~   238 (475) |
|  |  |  | +++ +..++.+.+++.++++.+|+++++.+....+......+..+.+. + ..........+.......+ |
|  | T Consensus | 424 | ~~~-----r~~~~al~~~~a~lal~~hptgl~alaall~~l~~l~r~lr~r~-------~~~~~la~la~~la~~~~~l~   491 (1116) |
|  | T 7BVF\_B | 424 | RYS-----RLTPAALAVVTAAFTLGVQPTGLIAVAALVAGGRPMLRILVRRH-------RLVGTLPLVSPMLAAGTVILT   491 (1116) |
|  | T ss\_dssp |  | HHT-----CSSHHHHHHHHHHHHHTTCGGGCCSHHHHHHTTHHHHHHHHHHH-------TTSCSHHHHSHHHHTTTCTHH |
|  | T ss\_pred |  | cCC-----CcHHHHHHHHHHHHHHhccHHHHHHHHHHHHhhHHHHHHHHHhh-------hhcchHHHHHHHHHHHHHHHH |
|  |
|  |
|  | Q ss\_pred |  | HHHHHHHhcCCCCCccCCCCCCcCCChHHHHHHHHHHHHHHhHHHhhCcccccccccccccccccccCCHHHHHHHHHHH |
|  | Q Q8N394 | 239 | LGARLYWMGNKPPSFSNSDNPAADSDSLLTRTLTFFYLPTKNLWLLLCPDTLSFDWSMDAVPLLKTVCDWRNLHTVAFYT   318 (475) |
|  | Q Consensus | 239 | ~~~~~~~~~~~~~~~~~~~~~~~~~~~~~~~~~~~~~~~~~~~~~~~~p~~~~~~~~~~~~~~~~~~~~~~~~~~~~~~~   318 (475) |
|  |  |  | ..+. ........+..+.....-...-+......++.........+......+...++.+ |
|  | T Consensus | 492 | ~~F~---------------------dqsl~~~~~a~~~~~~~g~~~~W~~e~~Ry~~L~~~~~~Gs~arr~~vLl~ll~L   550 (1116) |
|  | T 7BVF\_B | 492 | VVFA---------------------DQTLSTVLEATRVRAKIGPSQAWYTENLRYYYLILPTVDGSLSRRFGFLITALCL   550 (1116) |
|  | T ss\_dssp |  | HHTS---------------------SSCHHHHHHHHHHHHHTSCCCCGGGTHHHHHGGGSSSSSSCHHHHHHHHHHHHHH |
|  | T ss\_pred |  | HHHc---------------------cchHHHHHHHHHHHHHhcCCchHHHHhHHHHHHhccCCCCChHHHHHHHHHHHHH |
|  |
|  |
|  | Q ss\_pred |  | HHHHHHHHHhhCcccccccCCCcccCCccccCCcccccccccccccccccccchhhcCCcCCcccccCCCCCC---chHH |
|  | Q Q8N394 | 319 | GLLLLAYYGLKSPSVDRECNGKTVTNGKQNANGHSCLSDVEYQNSETKSSFASKVENGIKNDVSQRTQLPSTE---NIVV   395 (475) |
|  | Q Consensus | 319 | ~~~~~~~~~~~~~~~~~~~~~~~~~~~~~~~~~~~~~~~~~~~~~~~~~~~~~~~~~~~~~~~~~~~~~~~~~---~~~~   395 (475) |
|  |  |  | ..+.......++ ... .... |
|  | T Consensus | 551 | ~~~~~~l~R~rr----------------------------------------------------------~~g~~~~~~~   572 (1116) |
|  | T 7BVF\_B | 551 | FTAVFIMLRRKR----------------------------------------------------------IPSVARGPAW   572 (1116) |
|  | T ss\_dssp |  | HHHHHHHHHCSC----------------------------------------------------------CTTSCTTHHH |
|  | T ss\_pred |  | HHHHHHHHhcCC----------------------------------------------------------CCcccCcHHH |
|  |
|  |
|  | Q ss\_pred |  | hhhHHHHHHHhhhcCccccCChhhHHHHhHHHHHHHHHHHHHHHHHHHHHhchHHHHHHHHHHHHHHHHHHHHHHHHh |
|  | Q Q8N394 | 396 | LSLSLLIIPFVPATNLFFYVGFVIAERVLYIPSMGFCLLITVGARALYVKVQKRFLKSLIFYATATLIVFYGLKTAIR   473 (475) |
|  | Q Consensus | 396 | ~~l~~~~~~~~~~~~~~~~~~~~~~~Ry~~~~~~~~~ll~~~~l~~~~~~~~~~~~~~~~~~~~~~~~~~~~~~~~~~   473 (475) |
|  |  |  | .++.+.++.++.+.+. ..+..||+....++...+++.++..+.+...+....+......+++++.++.....+ |
|  | T Consensus | 573 | ~ll~~~~~~~~ll~~t-----PtKwthhfg~~a~~~~aLlA~~~~~~~~~~~r~~~~r~~~~~~~~~~~ala~~g~n~   645 (1116) |
|  | T 7BVF\_B | 573 | RLMGVIFGTMFFLMFT-----PTKWVHHFGLFAAVGAAMAALTTVLVSPSVLRWSRNRMAFLAALFFLLALCWATTNG   645 (1116) |
|  | T ss\_dssp |  | HHHHHHHHHHHHTTSC-----SCCCGGGGGGGGTTHHHHHHHHHHHTSTTTSCCHHHHHHHHHHHHHHHHHHTSSCCC |
|  | T ss\_pred |  | HHHHHHHHHHHHHhcC-----chhHHHHHHHHHHHHHHHHHHHHHHhChhhcchHHHHHHHHHHHHHHHHHHHhhhcc |
|  |
| --- | | | |
|  | Template alignmentTemplate 3D StructurePDBe | | |
| 12. | 6W98\_A F5/8 type C domain-containing protein; Glycosyltransferase, lipomannan, lipoarabinomannan, arabinofuranose, membrane; HET: PNS, 6OU; 2.9A {Escherichia coli (strain K12)}; Related PDB entries: 6WBX\_A 6WBY\_A | | |
|  | Probability: 99.41%, E-value: 6.7e-10, Score: 125.05, Aligned cols: 379, Identities: 12%, Similarity: -0.024, | | |
|  |
|  | Q ss\_pred |  | HHHHHHHHHHHHHHhhcCCCCcccchhHHHhchhhCCCCchhHhhhcccccccccCCCCcccCChHHHHHHHHHHHHhCC |
|  | Q Q8N394 | 3 | AELVSSALGLALYLNTLSADFCYDDSRAIKTNQDLLPETPWTHIFYNDFWGTLLTHSGSHKSYRPLCTLSFRLNHAIGGL   82 (475) |
|  | Q Consensus | 3 | ~~lll~~~~~~~~~~~~~~~~~~De~~~~~~~~~~~~~~~~~~~~~~~~~~~~~~~~~~~~~~~Pl~~~l~~~~~~lfG~   82 (475) |
|  |  |  | ..+++++++.+..+.........|+..+........-......+.....+... ......+......++.+...+... |
|  | T Consensus | 17 | ~ll~~~lll~~~~~~~~~g~~~~d~~~~~~~~~~~~l~~~~~~W~~~~~~G~~---~~~~~~y~~P~~~~~~l~~~lg~~   93 (1413) |
|  | T 6W98\_A | 17 | WLAVAAAVSLLLTFSQSPGQISPDTKLDLAINPLRFAARALNLWSSDLPFGQA---QNQAYGYLFPHGAFFSLGHLLGVP   93 (1413) |
|  | T ss\_dssp |  | HHHHHHHHHHHHHTTSSTTCBCCTTCSHHHHCHHHHHHHTTSSEESSSTTSEE---CCSSGGGCCCCCHHHHHHHHHTCC |
|  | T ss\_pred |  | HHHHHHHHHHHHHHhCCCCccCCCCCCccccCHHHHHHHHHhccCCCCCCCCC---chhhhhhhhhHHHHHHHHHHcCCC |
|  |
|  |
|  | Q ss\_pred |  | ChHHHHHHHHHHHHHHHHHHHHHHHHHh-CChHHHHHHHHHHHHCHHhHHHHHhhhchHHHHHHHHHHHHHHHHHHHHcc |
|  | Q Q8N394 | 83 | NPWSYHLVNVLLHAAVTGLFTSFSKILL-GDGYWTFMAGLMFASHPIHTEAVAGIVGRADVGASLFFLLSLLCYIKHCST   161 (475) |
|  | Q Consensus | 83 | ~~~~~rl~~~l~~~l~~~lly~l~r~l~-~~~~~al~aall~a~~P~~~~~~~~~~~~~~~~~~~f~ll~l~~~l~~~~~   161 (475) |
|  |  |  | .....|+..+++.+++.+.+|+++|++. +++..+++++++++++|.++......+. +.+..++....++.+.+..++ |
|  | T Consensus | 94 | ~~~~~rl~~~l~~~la~~g~y~L~r~l~~~~~~~al~Aal~yalsP~~l~~~~~~~~--~~~~~~llp~~ll~l~~~~~~   171 (1413) |
|  | T 6W98\_A | 94 | AWVTQRLWWALLIVAGFWGLIRVAEALGIGTRGSRIIAAVAFALSPRVLTTLGAISS--ETLPMMLAPWVLLPLILTFQG   171 (1413) |
|  | T ss\_dssp |  | HHHHHHHHHHHHHHHHHHHHHHHHHHHTCSCTTHHHHHHHHHHTCHHHHHHHTTCGG--GTHHHHHHHHHHHHHHHHHTT |
|  | T ss\_pred |  | HHHHHHHHHHHHHHHHHHHHHHHHHHhCCCChHHHHHHHHHHHHCHHHHHHhhccCh--hhHHHHHHHHHHHHHHHHHcC |
|  |
|  |
|  | Q ss\_pred |  | --CCCCcchHHHHHHHHHHHHHHHHhHhHHHHHHHHHHHHHHHHHcccchhhhcchHhhhHHHHHHHHHHHHHHHHHHHH |
|  | Q Q8N394 | 162 | --RGYSARTWGWFLGSGLCAGCSMLWKEQGVTVLAVSAVYDVFVFHRLKIKQILPTIYKRKNLSLFLSISLLIFWGSSLL   239 (475) |
|  | Q Consensus | 162 | --~~~~~~~~~~~~l~~l~~~la~ltk~~~~~~~~~~~~~~l~~~~~~~~~~~~~~~~~~~~~~~~~~~~~~~~~~~~~~   239 (475) |
|  |  |  | + ++++.+++++++++...++....++.+.+.+.+++..+++ ++.+.......++.++...++ |
|  | T Consensus | 172 | ~~~-----~~r~~~~~~l~~~l~~~~~~~~~~~~l~~~~l~~l~~~~~-----------~~~~~~~~~~~~~~~l~~~~w   235 (1413) |
|  | T 6W98\_A | 172 | RMS-----PRRAAALSAVAVALMGAVNAVATALACGVAVIWWLAHRPN-----------RTWWRFTAWWIPCLALASTWW   235 (1413) |
|  | T ss\_dssp |  | SSC-----HHHHHHHHHHHHHHSCSSSHHHHHHHSHHHHHHHHSBCCC-----------HHHHHHHHHHHHHHHHHHHHH |
|  | T ss\_pred |  | CCC-----HHHHHHHHHHHHHHhcchhHHHHHHHHHHHHHHHHHCCCC-----------chHHHHHHHHHHHHHHHHHHH |
|  |
|  |
|  | Q ss\_pred |  | HHHHHHhcCCCCCccCCCCCCcCCChHHHHHHH-HHHHHHHhHHHhhCcccccccccccccccccccCCHHHHHHHHHHH |
|  | Q Q8N394 | 240 | GARLYWMGNKPPSFSNSDNPAADSDSLLTRTLT-FFYLPTKNLWLLLCPDTLSFDWSMDAVPLLKTVCDWRNLHTVAFYT   318 (475) |
|  | Q Consensus | 240 | ~~~~~~~~~~~~~~~~~~~~~~~~~~~~~~~~~-~~~~~~~~~~~~~~p~~~~~~~~~~~~~~~~~~~~~~~~~~~~~~~   318 (475) |
|  |  |  | .......+ ............ ............+........+.....................+++ |
|  | T Consensus | 236 | l~Pll~~~-------------~~~~~~~~~~e~~~~~~~~~s~~~~l~~~~~w~~~~~~~~~~~~~~~~~~~~~~~~~~l   302 (1413) |
|  | T 6W98\_A | 236 | IVALLIFG-------------KISPKFLDFIESSGVTTQWTSLTEVLRGTDSWTPFVAPTATAGSSLVTQSAMVIATTML   302 (1413) |
|  | T ss\_dssp |  | HHHHHHHH-------------HHSCCCTTSSCC-------CCHHHHHHTC-----------------CCHHHHHHHHHHH |
|  | T ss\_pred |  | HHHHHHhc-------------ccCHhHHHHHhccccccccccHHHHHhCCCCCccccCCCCcccchHHHhHHHHHHHHHH |
|  |
|  |
|  | Q ss\_pred |  | HHHHHHHHHhhCcccccccCCCcccCCccccCCcccccccccccccccccccchhhcCCcCCcccccCCCCCCchHHhhh |
|  | Q Q8N394 | 319 | GLLLLAYYGLKSPSVDRECNGKTVTNGKQNANGHSCLSDVEYQNSETKSSFASKVENGIKNDVSQRTQLPSTENIVVLSL   398 (475) |
|  | Q Consensus | 319 | ~~~~~~~~~~~~~~~~~~~~~~~~~~~~~~~~~~~~~~~~~~~~~~~~~~~~~~~~~~~~~~~~~~~~~~~~~~~~~~~l   398 (475) |
|  |  |  | .++++....+++ .+...+.+ |
|  | T Consensus | 303 | ~~lgl~~l~~r~------------------------------------------------------------~~~~~~l~   322 (1413) |
|  | T 6W98\_A | 303 | AAAGMAGLAMRG------------------------------------------------------------MPARGRLV   322 (1413) |
|  | T ss\_dssp |  | HHHHHHHHSSTT------------------------------------------------------------CTTHHHHH |
|  | T ss\_pred |  | HHHHHHHHHhCC------------------------------------------------------------CchHHHHH |
|  |
|  |
|  | Q ss\_pred |  | HHHHHHHhhhcCccc-----cCChhhHH-------------HHhHHHHHHHHHHHHHHHHHHHHH------------hch |
|  | Q Q8N394 | 399 | SLLIIPFVPATNLFF-----YVGFVIAE-------------RVLYIPSMGFCLLITVGARALYVK------------VQK   448 (475) |
|  | Q Consensus | 399 | ~~~~~~~~~~~~~~~-----~~~~~~~~-------------Ry~~~~~~~~~ll~~~~l~~~~~~------------~~~   448 (475) |
|  |  |  | +++++.++....... ........ ||..+..++++++++.++..+.++ .++ |
|  | T Consensus | 323 | ~~~l~g~~l~~~~~~~~~~~p~~~~~~~~l~p~~~~~R~~~Rf~~~~~l~lall~a~~l~~l~~~~~~~~~~~~~~~~~~   402 (1413) |
|  | T 6W98\_A | 323 | AVLLIGLVLLTAGYTGALGSPIAQQIQFFLDDGGTPLRNVHKLEPLIRLPLILGLAHALSRIPLPASVPVRQWLSALARP   402 (1413) |
|  | T ss\_dssp |  | HHHHHHHHTC---------------------------CCGGGSHHHHHHHHHHHHHHHTSSSCCTTTSCC---------- |
|  | T ss\_pred |  | HHHHHHHHHHHhcccCCCCCchHHHHHHHhccCchhhcChhhhHHHHHHHHHHHHHHHHHhCCCCCCCCHHHHHHhhcCh |
|  |
|  |
|  | Q ss\_pred |  | HHHHHHHHHHHHHHHHHHHHHHHHhcC |
|  | Q Q8N394 | 449 | RFLKSLIFYATATLIVFYGLKTAIRNG   475 (475) |
|  | Q Consensus | 449 | ~~~~~~~~~~~~~~~~~~~~~~~~~~~   475 (475) |
|  |  |  | ++.+..+..++++++++.......+.. |
|  | T Consensus | 403 | ~~~~~~~~~~~~~~~~~~~~~p~~~~~   429 (1413) |
|  | T 6W98\_A | 403 | ERNRAVAFAIVLLVALAASTSLAWTGR   429 (1413) |
|  | T ss\_dssp |  | --CTTTHHHHHHHHHHHHHTHHHHTTC |
|  | T ss\_pred |  | HHHHHHHHHHHHHHHHHHHHHHHHhCC |
|  |
| --- | | | |
|  | Template alignmentTemplate 3D StructurePDBe | | |
| 13. | 7BVE\_B Integral membrane indolylacetylinositol arabinosyltransferase EmbC; Mycobacterium smegmatis, cell wall synthesis; HET: PO4, PN7, 95E; 2.81A {Mycolicibacterium smegmatis MC2 155}; Related PDB entries: 7BVH\_B 7BVH\_A 7BVE\_A | | |
|  | Probability: 99.38%, E-value: 4.3e-9, Score: 111.84, Aligned cols: 351, Identities: 11%, Similarity: -0.068, | | |
|  |
|  | Q ss\_pred |  | HHHHHHHHHHhhcCCCCcccchhHHHhchhhCCCCchhHhhhcccccccccCCCCcccCChHHHHHHHHHHHHhCCChHH |
|  | Q Q8N394 | 7 | SSALGLALYLNTLSADFCYDDSRAIKTNQDLLPETPWTHIFYNDFWGTLLTHSGSHKSYRPLCTLSFRLNHAIGGLNPWS   86 (475) |
|  | Q Consensus | 7 | l~~~~~~~~~~~~~~~~~~De~~~~~~~~~~~~~~~~~~~~~~~~~~~~~~~~~~~~~~~Pl~~~l~~~~~~lfG~~~~~   86 (475) |
|  |  |  | ..+.+.+..+..... .+.||.++...+++..+.+...+++..- +..+....+++.++..+.. +|.++.+ |
|  | T Consensus | 261 | ~~V~~~l~~w~~ig~-~~~DEg~~l~~ar~~~~~Gy~~n~~~~~---------~~~dapfg~yY~lL~~W~~-vG~s~~~   329 (1084) |
|  | T 7BVE\_B | 261 | GLVSAMLVWWHFVGA-NTADDGYILTMARVSEHAGYMANYYRWF---------GTPESPFGWYYDLLALWAH-VSTASVW   329 (1084) |
|  | T ss\_dssp |  | HHHHHHHHHHHHSCC-CCSSSHHHHHHHHHHHHHSSCBCSSSGG---------GCBCCSSCSSHHHHHHHTT-TCCCHHH |
|  | T ss\_pred |  | HHHHHHHHHHHHhcc-cccchhHHhhhhhchhhcCchHHHHHHH---------CCCCcccHHHHHHHHHHHH-ccccHHH |
|  |
|  |
|  | Q ss\_pred |  | HHHHHHHHHHHHHHHH-----HHHHHHHhCChHHHHHHHHHHHHCHHhHHHHHhhhchHHHHHHHHHHHHHHHHHHHHcc |
|  | Q Q8N394 | 87 | YHLVNVLLHAAVTGLF-----TSFSKILLGDGYWTFMAGLMFASHPIHTEAVAGIVGRADVGASLFFLLSLLCYIKHCST   161 (475) |
|  | Q Consensus | 87 | ~rl~~~l~~~l~~~ll-----y~l~r~l~~~~~~al~aall~a~~P~~~~~~~~~~~~~~~~~~~f~ll~l~~~l~~~~~   161 (475) |
|  |  |  | .|++++++++++..++ ..+.+++.+++...+.+++++.. .+..+.+.. |+|.+..++.+++++++.|..++ |
|  | T Consensus | 330 | LRlpSll~glat~~ll~R~vl~~Lg~~~~~~~~a~~~aal~fl~--~wl~y~~~~--Rpyalvalla~l~~~~~~ra~~~   405 (1084) |
|  | T 7BVE\_B | 330 | MRFPTLLMGLACWWVISREVIPRLGAAAKHSRAAAWTAAGLFLA--FWLPLNNGL--RPEPIIALGILLTWCSVERGVAT   405 (1084) |
|  | T ss\_dssp |  | HHHHHHHHHHHHHHHCCCCCTTTSBSTTTSCSHHHHHHHHHHHH--HHHHHCSSS--SSHHHHHHHHHHHHHHHHHHHHH |
|  | T ss\_pred |  | HHHHHHHHHHHHHHHHHHHhHHHHHHHhccCHHHHHHHHHHHHH--HHHHhcCCC--ChHHHHHHHHHHHHHHHHHHHcC |
|  |
|  |
|  | Q ss\_pred |  | CCCCcchHHHHHHHHHHHHHHHHhHhHHHHHHHHHHHHHHHHHcccchhhhcchHhhh-HHHHHHHHHHHHHHHHHHHHH |
|  | Q Q8N394 | 162 | RGYSARTWGWFLGSGLCAGCSMLWKEQGVTVLAVSAVYDVFVFHRLKIKQILPTIYKR-KNLSLFLSISLLIFWGSSLLG   240 (475) |
|  | Q Consensus | 162 | ~~~~~~~~~~~~l~~l~~~la~ltk~~~~~~~~~~~~~~l~~~~~~~~~~~~~~~~~~-~~~~~~~~~~~~~~~~~~~~~   240 (475) |
|  |  |  | + +..++.+++++.++++++|+++++......+......+..+.+. + ......+...+.......+.. |
|  | T Consensus | 406 | ~-----r~~~~ala~~~a~la~~~~Ptgl~ala~ll~~~~~l~r~lr~r~-------~~~~~la~la~~lAa~~~~l~~~   473 (1084) |
|  | T 7BVE\_B | 406 | S-----RLLPVAVAIIIGALTLFSGPTGIAAVGALLVAIGPLKTIVAAHV-------SRFGYWALLAPIAAAGTVTIFLI   473 (1084) |
|  | T ss\_dssp |  | T-----CSHHHHHHHHHHHHHHTSSGGGGGGHHHHHHTSHHHHHHHHHHT-------TTSCSHHHHHHHHHHHHTTHHHH |
|  | T ss\_pred |  | C-----CcHHHHHHHHHHHHHHhhhHHHHHHHHHHHHHHHHHHHHHHhcc-------chhHHHHHHHHHHHHHHHHHHHH |
|  |
|  |
|  | Q ss\_pred |  | HHHHHhcCCCCCccCCCCCCcCCChHHHHHHHHHHHHHHhHHHhhCcccccccccccccccccccCCHHHHHHHHHHHHH |
|  | Q Q8N394 | 241 | ARLYWMGNKPPSFSNSDNPAADSDSLLTRTLTFFYLPTKNLWLLLCPDTLSFDWSMDAVPLLKTVCDWRNLHTVAFYTGL   320 (475) |
|  | Q Consensus | 241 | ~~~~~~~~~~~~~~~~~~~~~~~~~~~~~~~~~~~~~~~~~~~~~~p~~~~~~~~~~~~~~~~~~~~~~~~~~~~~~~~~   320 (475) |
|  |  |  | +. +.......+..+.....-...-+......+......+...+..........++.++. |
|  | T Consensus | 474 | Fa---------------------dqsl~~~~~a~~v~~~~gp~l~w~~e~~Ry~~l~g~~~~gs~arr~~vLl~l~~l~~   532 (1084) |
|  | T 7BVE\_B | 474 | FR---------------------DQTLAAELQASSFKSAVGPSLAWFDEHIRYSRLFTTSPDGSVARRFAVLTLLLALAV   532 (1084) |
|  | T ss\_dssp |  | TS---------------------SSCHHHHHHHHHHHHHHSCCCCGGGTHHHHHHHTSSSSTTCHHHHHHHHHHHHHHHH |
|  | T ss\_pred |  | HH---------------------hhhHHHHHHHHhhhhccCCCcHhHHHHHHHHHHhcCCCCCCHHHHHHHHHHHHHHHH |
|  |
|  |
|  | Q ss\_pred |  | HHHHHHHhhCcccccccCCCcccCCccccCCcccccccccccccccccccchhhcCCcCCcccccCCCCCC---chHHhh |
|  | Q Q8N394 | 321 | LLLAYYGLKSPSVDRECNGKTVTNGKQNANGHSCLSDVEYQNSETKSSFASKVENGIKNDVSQRTQLPSTE---NIVVLS   397 (475) |
|  | Q Consensus | 321 | ~~~~~~~~~~~~~~~~~~~~~~~~~~~~~~~~~~~~~~~~~~~~~~~~~~~~~~~~~~~~~~~~~~~~~~~---~~~~~~   397 (475) |
|  |  |  | +......+++ ... .....+ |
|  | T Consensus | 533 | ~~~ll~R~rr----------------------------------------------------------~~g~~~~~~~~l   554 (1084) |
|  | T 7BVE\_B | 533 | SIAMTLRKGR----------------------------------------------------------IPGTALGPSRRI   554 (1084) |
|  | T ss\_dssp |  | HHHHHHHSSS----------------------------------------------------------CTTBCHHHHHHH |
|  | T ss\_pred |  | HHHHHhhcCC----------------------------------------------------------CCCCCCCHHHHH |
|  |
|  |
|  | Q ss\_pred |  | hHHHHHHHhhhcCccccCChhhHHHHhHHHHHHHHHHHHHHHHHHHHHhchHHHHHHHHHHHHHHHHHHHH |
|  | Q Q8N394 | 398 | LSLLIIPFVPATNLFFYVGFVIAERVLYIPSMGFCLLITVGARALYVKVQKRFLKSLIFYATATLIVFYGL   468 (475) |
|  | Q Consensus | 398 | l~~~~~~~~~~~~~~~~~~~~~~~Ry~~~~~~~~~ll~~~~l~~~~~~~~~~~~~~~~~~~~~~~~~~~~~   468 (475) |
|  |  |  | +...+..++...+. ..+..+|+........++++..+........+..+.+.+....+++.+.++. |
|  | T Consensus | 555 | ~~~~~~~l~ll~~t-----PtKwthhfg~lag~~~~lla~~~~~~~~~~~r~~r~~~~~~a~~~~~~ala~   620 (1084) |
|  | T 7BVE\_B | 555 | IGITIISFLAMMFT-----PTKWTHHFGVFAGLAGCLGALAAVAVTTTAMKSRRNRTVFGAAVLFVTALSF   620 (1084) |
|  | T ss\_dssp |  | HHHHHHHHHHGGGC-----SSCCSGGGGGGTTHHHHHHHHHHHTTSTTTCCCHHHHHHHHHHHHHHHHHHT |
|  | T ss\_pred |  | HHHHHHHHHHHhcC-----CchHHHHHHhHHHHHHHHHHHHHHHHHHHhccchHHHHHHHHHHHHHHHHHh |
|  |
| --- | | | |
|  | Template alignmentTemplate 3D StructurePDBe | | |
| 14. | 6SNI\_X Dolichyl pyrophosphate Man9GlcNAc2 alpha-1,3-glucosyltransferase (E.C.2.4.1.267); Glycosyltransferase, Glucosyltransferase, GT-C, N-Glycosylation, MEMBRANE; HET: PTY, Y01;{Saccharomyces cerevisiae}; Related PDB entries: 6SNH\_X | | |
|  | Probability: 99.28%, E-value: 1.3e-8, Score: 104.03, Aligned cols: 321, Identities: 12%, Similarity: 0, | | |
|  |
|  | Q ss\_pred |  | CHHHHHHHHHHHHHHHhhcCCCC-------cccchhHHHhchhhCCCCchhHhhhcccccccccCCCCcccCChHHHHHH |
|  | Q Q8N394 | 1 | MIAELVSSALGLALYLNTLSADF-------CYDDSRAIKTNQDLLPETPWTHIFYNDFWGTLLTHSGSHKSYRPLCTLSF   73 (475) |
|  | Q Consensus | 1 | ~~~~lll~~~~~~~~~~~~~~~~-------~~De~~~~~~~~~~~~~~~~~~~~~~~~~~~~~~~~~~~~~~~Pl~~~l~   73 (475) |
|  |  |  | .....++++++++++......++ ..+|........+...+.+..+++.++. +.+...+||+..+.. |
|  | T Consensus | 54 | ~~~l~~i~~~~l~lR~~~~~~~~sg~~~pp~~~D~~~~~~w~~~~~~~~~~~wy~~~~-------~~~~~~YPPl~~~~~   126 (562) |
|  | T 6SNI\_X | 54 | WLPEYIIFVCAVILRCTIGLGPYSGKGSPPLYGDFEAQRHWMEITQHLPLSKWYWYDL-------QYWGLDYPPLTAFHS   126 (562) |
|  | T ss\_dssp |  | --CCSSHHHHHHHHHHHGGGSCCTTSSCSSSCCHHHHHHHHHHHHTTSCTTSTTTSCS-------TTTCCCSCHHHHHHH |
|  | T ss\_pred |  | cHHHHHHHHHHHHHHHHHhcCCCCCCCCCCCCCCHHHHHHHHHHHHhCCHHHhcccCc-------cccCCCChHHHHHHH |
|  |
|  |
|  | Q ss\_pred |  | HHHHHHhC--------------------CChHHHHHHHHHHHHHHHHHH-HHHHHHHhC----ChHHHHHHHHHHHHCHH |
|  | Q Q8N394 | 74 | RLNHAIGG--------------------LNPWSYHLVNVLLHAAVTGLF-TSFSKILLG----DGYWTFMAGLMFASHPI   128 (475) |
|  | Q Consensus | 74 | ~~~~~lfG--------------------~~~~~~rl~~~l~~~l~~~ll-y~l~r~l~~----~~~~al~aall~a~~P~   128 (475) |
|  |  |  | .+...+.+ .+....|+.+++..+++...+ |.+.|+..+ ++..+..++++++++|. |
|  | T Consensus | 127 | ~~~~~i~~~~~~~~~~l~~~~g~~~~~~~~~~~~rl~~i~~~ll~~~~~~~~~~~~~~~~~~~~~~~~~~~~~~~~l~P~   206 (562) |
|  | T 6SNI\_X | 127 | YLLGLIGSFFNPSWFALEKSRGFESPDNGLKTYMRSTVIISDILFYFPAVIYFTKWLGRYRNQSPIGQSIAASAILFQPS   206 (562) |
|  | T ss\_dssp |  | HHHHHHHHHHCTTTTCSSSSTTCCCTTCCSSSHHHHHHHHHHHHHTHHHHHHHHHHHHHHHTCCHHHHHHHHHHHHCCHH |
|  | T ss\_pred |  | HHHHHHHHHhCHHHHHhcccCCCCCcchHHHHHHHHHHHHHHHHHHHHHHHHHHHHHhhcCCCChhHHHHHHHHHHhCHH |
|  |
|  |
|  | Q ss\_pred |  | hHHHHHhhhchHHHHHHHHHHHHHHHHHHHHccCCCCcchHHHHHHHHHHHHHHHHhHhHHHHHHHHHHHHHHHHHcccc |
|  | Q Q8N394 | 129 | HTEAVAGIVGRADVGASLFFLLSLLCYIKHCSTRGYSARTWGWFLGSGLCAGCSMLWKEQGVTVLAVSAVYDVFVFHRLK   208 (475) |
|  | Q Consensus | 129 | ~~~~~~~~~~~~~~~~~~f~ll~l~~~l~~~~~~~~~~~~~~~~~l~~l~~~la~ltk~~~~~~~~~~~~~~l~~~~~~~   208 (475) |
|  |  |  | .+...+.. .+.|.....+.+++++++.+.+ ...+++++++|+.+|+..+.+.+.+.++.+....+++ |
|  | T Consensus | 207 | ~i~~~~~~-~q~d~~~l~l~l~al~~~~~~~------------~~~agi~~~lal~~K~~~l~~~p~~~~~ll~~~~~~~   273 (562) |
|  | T 6SNI\_X | 207 | LMLIDHGH-FQYNSVMLGLTAYAINNLLDEY------------YAMAAVCFVLSICFKQMALYYAPIFFAYLLSRSLLFP   273 (562) |
|  | T ss\_dssp |  | HHHHHTTT-CCCHHHHHHHHHHHHHHHHHTC------------HHHHHHHHHHHHTTCGGGTTSHHHHHHHHHCCCCCSS |
|  | T ss\_pred |  | HHHhhccc-chhHHHHHHHHHHHHHHHHCCC------------hHHHHHHHHHHHHhhHHHHHHHHHHHHHHHHHHcCCC |
|  |
|  |
|  | Q ss\_pred |  | hhhhcchHhhhHHHHHHHHHHHHHHHHHHHHHHHH----HHhcCCCCCccCCCCCCcCCChHHHHHHHHHHHHHHhHHHh |
|  | Q Q8N394 | 209 | IKQILPTIYKRKNLSLFLSISLLIFWGSSLLGARL----YWMGNKPPSFSNSDNPAADSDSLLTRTLTFFYLPTKNLWLL   284 (475) |
|  | Q Consensus | 209 | ~~~~~~~~~~~~~~~~~~~~~~~~~~~~~~~~~~~----~~~~~~~~~~~~~~~~~~~~~~~~~~~~~~~~~~~~~~~~~   284 (475) |
|  |  |  | +++. ++........+....+....+.... .........+..............-...+............ |
|  | T Consensus | 274 | ~~~~------~~~~~~~~~~~~~~~l~~~Pf~~~~~~~~~~~~~~~~~fp~~rgl~~~~~~n~w~~~~~~~~~~~~~~~~   347 (562) |
|  | T 6SNI\_X | 274 | KFNI------ARLTVIAFATLATFAIIFAPLYFLGGGLKNIHQCIHRIFPFARGIFEDKVANFWCVTNVFVKYKERFTIQ   347 (562) |
|  | T ss\_dssp |  | CCCH------HHHHHHHHHHHHHHHHHHHHHHTTTCSHHHHHHHHHHHSCCCCSSSCSCCSSSHHHHTTTSCGGGTSCHH |
|  | T ss\_pred |  | CCcH------HHHHHHHHHHHHHHHHHHHHHHHhcCCHHHHHHHHHHHcCCCcccccchhhhHHHHHHHHHHHHhhCCHH |
|  |
|  |
|  | Q ss\_pred |  | hCcccccccccccccccccccCCHHHHHHHHHHHHHHHHHHHHhhCcccccccCCCcccCCccccCCccccccccccccc |
|  | Q Q8N394 | 285 | LCPDTLSFDWSMDAVPLLKTVCDWRNLHTVAFYTGLLLLAYYGLKSPSVDRECNGKTVTNGKQNANGHSCLSDVEYQNSE   364 (475) |
|  | Q Consensus | 285 | ~~p~~~~~~~~~~~~~~~~~~~~~~~~~~~~~~~~~~~~~~~~~~~~~~~~~~~~~~~~~~~~~~~~~~~~~~~~~~~~~   364 (475) |
|  |  |  | ...........++.+.......++ |
|  | T Consensus | 348 | ----------------------~~~~~~~~~~~l~~l~~~~~~~~~----------------------------------   371 (562) |
|  | T 6SNI\_X | 348 | ----------------------QLQLYSLIATVIGFLPAMIMTLLH----------------------------------   371 (562) |
|  | T ss\_dssp |  | ----------------------HHHHHHHHHHHHHHHHHHHHHHTS---------------------------------- |
|  | T ss\_pred |  | ----------------------HHHHHHHHHHHHHHHHHHHHHHhC---------------------------------- |
|  |
|  |
|  | Q ss\_pred |  | ccccccchhhcCCcCCcccccCCCCCCchHHhhhHHHHHHHhhhcCccccCChhhHHHH-hHHHHHHHHHH |
|  | Q Q8N394 | 365 | TKSSFASKVENGIKNDVSQRTQLPSTENIVVLSLSLLIIPFVPATNLFFYVGFVIAERV-LYIPSMGFCLL   434 (475) |
|  | Q Consensus | 365 | ~~~~~~~~~~~~~~~~~~~~~~~~~~~~~~~~~l~~~~~~~~~~~~~~~~~~~~~~~Ry-~~~~~~~~~ll   434 (475) |
|  |  |  | +..+.....+......+..++ ...+++| +++.+|...+. |
|  | T Consensus | 372 | ------------------------~~~~~~~~~~~~~~l~~flfs-------~~vhekyill~llPl~ll~   411 (562) |
|  | T 6SNI\_X | 372 | ------------------------PKKHLLPYVLIACSMSFFLFS-------FQVHEKTILIPLLPITLLY   411 (562) |
|  | T ss\_dssp |  | ------------------------CCSSSHHHHHHHHHHHHHHHC-------SSCCSSCCHHHHHHHHHGG |
|  | T ss\_pred |  | ------------------------CCcchHHHHHHHHHHHHHHhc-------hhcCchhcHHHHHHHHHHh |
|  |
| --- | | | |
|  | Template alignmentTemplate 3D StructurePDBe | | |
| 15. | 7BVC\_A Integral membrane indolylacetylinositol arabinosyltransferase EmbA; Mycobacterium smegmatis, cell wall synthesis; HET: 95E, PNS, CDL, F8L;{Mycolicibacterium smegmatis MC2 155}; Related PDB entries: 7BVG\_A | | |
|  | Probability: 99.27%, E-value: 2.2e-8, Score: 106.26, Aligned cols: 348, Identities: 10%, Similarity: -0.09, | | |
|  |
|  | Q ss\_pred |  | HHHHHHHHHHhhcCCCCcccchhHHHhchhhCCCCchhHhhhcccccccccCCCCcccCChHHHHHHHHHHHHhCCChHH |
|  | Q Q8N394 | 7 | SSALGLALYLNTLSADFCYDDSRAIKTNQDLLPETPWTHIFYNDFWGTLLTHSGSHKSYRPLCTLSFRLNHAIGGLNPWS   86 (475) |
|  | Q Consensus | 7 | l~~~~~~~~~~~~~~~~~~De~~~~~~~~~~~~~~~~~~~~~~~~~~~~~~~~~~~~~~~Pl~~~l~~~~~~lfG~~~~~   86 (475) |
|  |  |  | ..+.+.+..+.... +...||.++...+++..+.+...+++..- +..+..--+++.++..+..+ |.++.+ |
|  | T Consensus | 251 | ~vV~~~L~~W~~ig-p~~~DDg~~~~~ar~~~~~G~~gny~r~~---------~~~eapf~~yY~ll~~w~~v-g~s~~~   319 (1088) |
|  | T 7BVC\_A | 251 | TGVIGGLLIWHIVG-APTSDDGYNMTIARVASEAGYTTNYYRYF---------GASEAPFDWYQSVLSHLASI-STAGVW   319 (1088) |
|  | T ss\_dssp |  | HHHHHHHHSTTTSC-CCCTTHHHHHHHHHHSSSSSSCBCSSSGG---------GCBCTTSCHHHHHHHHHTTT-CCCHHH |
|  | T ss\_pred |  | HHHHHHHHHHHHhC-CCCcchhHHHHHHHHHHHhcCHHHHHHHh---------cCCCcCCHHHHHHHHHHHhc-ccchHH |
|  |
|  |
|  | Q ss\_pred |  | HHHHHHHHHHHHHHHH-----HHHHHHHhCChHHHHHHHHHHH--HCHHhHHHHHhhhchHHHHHHHHHHHHHHHHHHHH |
|  | Q Q8N394 | 87 | YHLVNVLLHAAVTGLF-----TSFSKILLGDGYWTFMAGLMFA--SHPIHTEAVAGIVGRADVGASLFFLLSLLCYIKHC   159 (475) |
|  | Q Consensus | 87 | ~rl~~~l~~~l~~~ll-----y~l~r~l~~~~~~al~aall~a--~~P~~~~~~~~~~~~~~~~~~~f~ll~l~~~l~~~   159 (475) |
|  |  |  | +|++++++++++..++ ..+.++..+++...+.+++.+. ..|+.... |+|.+..++.+++++++.+.. |
|  | T Consensus | 320 | lRLPSllagl~tw~llsR~vl~~Lg~~~~~~~~a~~aaal~fla~wlPy~~~~------Rpe~~val~~~~a~~~~~ra~   393 (1088) |
|  | T 7BVC\_A | 320 | MRLPATAAAIATWLIISRCVLPRIGRRVAANRVAMLTAGATFLAAWLPFNNGL------RPEPLIAFAVITVWMLVENSI   393 (1088) |
|  | T ss\_dssp |  | HTGGGTHHHHHHHHHCCCCCHHHHCHHHHHCHHHHHHHHHHHHHHHTTTCSSS------SSHHHHHHHHHHHHHHHHHHH |
|  | T ss\_pred |  | hHHHHHHHHHHHHHHHHHHHHHHHhHhhcccHHHHHHHHHHHHHHHHHhcCCC------ChHHHHHHHHHHHHHHHHHHh |
|  |
|  |
|  | Q ss\_pred |  | ccCCCCcchHHHHHHHHHHHHHHHHhHhHHHHHHHHHHHHHHHHHcccchhhhcchHhhhHHHHHHHHHHHHHHHHHHHH |
|  | Q Q8N394 | 160 | STRGYSARTWGWFLGSGLCAGCSMLWKEQGVTVLAVSAVYDVFVFHRLKIKQILPTIYKRKNLSLFLSISLLIFWGSSLL   239 (475) |
|  | Q Consensus | 160 | ~~~~~~~~~~~~~~l~~l~~~la~ltk~~~~~~~~~~~~~~l~~~~~~~~~~~~~~~~~~~~~~~~~~~~~~~~~~~~~~   239 (475) |
|  |  |  | +++ +..++.++.++.+++..+|+++++.+..+++......+.-+.+. +..........++........ |
|  | T Consensus | 394 | ~~~-----r~~~~a~a~~~aala~~~hPtGl~a~a~ll~~~~~l~r~~r~r~-------~~~~~~a~~a~~laa~~~~l~   461 (1088) |
|  | T 7BVC\_A | 394 | GTR-----RLWPAAVAIVIAMFSVTLAPQGLIALAPLLVGARAIGRVVTARR-------AGTGILASLAPLAASVAVVFV   461 (1088) |
|  | T ss\_dssp |  | TTT-----CSHHHHHHHHHHHHHHTTCGGGGGGGHHHHHHHHHHHHHHGGGT-------TTTCSHHHHHHHHHHHTTHHH |
|  | T ss\_pred |  | cCC-----CcHHHHHHHHHHHHHHhhcHHHHHHHHHHHHHHHHHHHHHHHhh-------ccccHHHHHHHHHHHHHHHHH |
|  |
|  |
|  | Q ss\_pred |  | HHHHHHhcCCCCCccCCCCCCcCCChHHHHHHHHHHHHHHhHHHhhCcccccccccccccccccccCCHHHHHHHHHHHH |
|  | Q Q8N394 | 240 | GARLYWMGNKPPSFSNSDNPAADSDSLLTRTLTFFYLPTKNLWLLLCPDTLSFDWSMDAVPLLKTVCDWRNLHTVAFYTG   319 (475) |
|  | Q Consensus | 240 | ~~~~~~~~~~~~~~~~~~~~~~~~~~~~~~~~~~~~~~~~~~~~~~~p~~~~~~~~~~~~~~~~~~~~~~~~~~~~~~~~   319 (475) |
|  |  |  | . ...+.......+..+.....-...-+.....-+...-..........-.....+.+..+ |
|  | T Consensus | 462 | ~--------------------~F~dqtl~~~~~a~r~~~~~gp~~~w~~E~~RY~~L~~~~~~~Gs~arR~~VLl~l~~l   521 (1088) |
|  | T 7BVC\_A | 462 | I--------------------IFRDQTLATVAESVRIKYVVGPTIPWYQEFLRYYFLTVEDSVDGSLTRRFAVLVLLLCL   521 (1088) |
|  | T ss\_dssp |  | H--------------------HTTSSCHHHHHHHHHHHHHHSCCCCTTCCTHHHHHHSCSSCSSSCTTHHHHHHHHHHHH |
|  | T ss\_pred |  | H--------------------HhCCCcHHHHHHHHhHHhhhCCCChHHchhHHHHHHHccCCCCCCHHHHHHHHHHHHHH |
|  |
|  |
|  | Q ss\_pred |  | HHHHHHHHhhCcccccccCCCcccCCccccCCcccccccccccccccccccchhhcCCcCCcccccCCCCCC------ch |
|  | Q Q8N394 | 320 | LLLLAYYGLKSPSVDRECNGKTVTNGKQNANGHSCLSDVEYQNSETKSSFASKVENGIKNDVSQRTQLPSTE------NI   393 (475) |
|  | Q Consensus | 320 | ~~~~~~~~~~~~~~~~~~~~~~~~~~~~~~~~~~~~~~~~~~~~~~~~~~~~~~~~~~~~~~~~~~~~~~~~------~~   393 (475) |
|  |  |  | ........+++ + .. |
|  | T Consensus | 522 | ~~~~~~l~rrr------------------------------------------------------------r~~g~~~~~   541 (1088) |
|  | T 7BVC\_A | 522 | FGLIMVLLRRG------------------------------------------------------------RVPGAVSGP   541 (1088) |
|  | T ss\_dssp |  | HHHHHHHHHSS------------------------------------------------------------CCTTSCHHH |
|  | T ss\_pred |  | HHHHHHHHhcC------------------------------------------------------------CCCCCCccH |
|  |
|  |
|  | Q ss\_pred |  | HHhhhHHHHHHHhhhcCccccCChhhHHHHhHHHHHHHHHHHHHHHHHHHHHhchHHHHHHHHHHHHHHHHHHHH |
|  | Q Q8N394 | 394 | VVLSLSLLIIPFVPATNLFFYVGFVIAERVLYIPSMGFCLLITVGARALYVKVQKRFLKSLIFYATATLIVFYGL   468 (475) |
|  | Q Consensus | 394 | ~~~~l~~~~~~~~~~~~~~~~~~~~~~~Ry~~~~~~~~~ll~~~~l~~~~~~~~~~~~~~~~~~~~~~~~~~~~~   468 (475) |
|  |  |  | ........++.++...+. ..+..+|+-...+....+++.....+.+...+....+.+....+++++.++. |
|  | T Consensus | 542 | ~~~l~~~~~~~~~lL~~t-----PtKWthHfGa~Ag~gaal~a~~~va~~~~~~r~~r~~~~~~a~~~~~~alaf   611 (1088) |
|  | T 7BVC\_A | 542 | LWRLCGSTAIGLLLLILT-----PTKWAIQFGAFAGLAGALGGVTAFAFARVGLHSRRNLALYVTALLFILAWAT   611 (1088) |
|  | T ss\_dssp |  | HHHHHHHHHHHHHHTTSC-----SCCCSGGGGGGHHHHHHHHHHHHHHHHHHTTSCHHHHHHHHHHHHHHHHHHT |
|  | T ss\_pred |  | HHHHHHHHHHHHHHHHHC-----cchHHHHHHHHHHHHHHHHHHHHHHHHHhccChHHHHHHHHHHHHHHHHHHh |
|  |

---

If you use HHpred on our Toolkit for your research, please cite as appropriate:

A Completely Reimplemented MPI Bioinformatics Toolkit
with a New HHpred Server at its Core.  
Zimmermann L, Stephens A, Nam SZ, Rau D,
Kübler J, Lozajic M, Gabler F, Söding J, Lupas AN, Alva V.
J Mol Biol. 2018 Jul 20. S0022-2836(17)30587-9.

  

Protein homology detection by HMM-HMM comparison.  
Söding J. Bioinformatics. 2005 Apr 1;21(7):951-60.  
  
Fast and accurate automatic structure prediction with HHpred.  
Hildebrand A, Remmert M, Biegert A, Söding J. Proteins. 2009;77 Suppl 9:128-32.  
  
Automatic Prediction of Protein 3D Structures by Probabilistic Multi-template Homology Modeling.  
Meier A, Söding J. PLoS Comput Biol. 2015 Oct 23;11(10):e1004343.

Download

---

If you use HHpred on our Toolkit for your research, please cite as appropriate:

A Completely Reimplemented MPI Bioinformatics Toolkit
with a New HHpred Server at its Core.  
Zimmermann L, Stephens A, Nam SZ, Rau D,
Kübler J, Lozajic M, Gabler F, Söding J, Lupas AN, Alva V.
J Mol Biol. 2018 Jul 20. S0022-2836(17)30587-9.

  

Protein homology detection by HMM-HMM comparison.  
Söding J. Bioinformatics. 2005 Apr 1;21(7):951-60.  
  
Fast and accurate automatic structure prediction with HHpred.  
Hildebrand A, Remmert M, Biegert A, Söding J. Proteins. 2009;77 Suppl 9:128-32.  
  
Automatic Prediction of Protein 3D Structures by Probabilistic Multi-template Homology Modeling.  
Meier A, Söding J. PLoS Comput Biol. 2015 Oct 23;11(10):e1004343.

Loading...

---

If you use HHpred on our Toolkit for your research, please cite as appropriate:

A Completely Reimplemented MPI Bioinformatics Toolkit
with a New HHpred Server at its Core.  
Zimmermann L, Stephens A, Nam SZ, Rau D,
Kübler J, Lozajic M, Gabler F, Söding J, Lupas AN, Alva V.
J Mol Biol. 2018 Jul 20. S0022-2836(17)30587-9.

  

Protein homology detection by HMM-HMM comparison.  
Söding J. Bioinformatics. 2005 Apr 1;21(7):951-60.  
  
Fast and accurate automatic structure prediction with HHpred.  
Hildebrand A, Remmert M, Biegert A, Söding J. Proteins. 2009;77 Suppl 9:128-32.  
  
Automatic Prediction of Protein 3D Structures by Probabilistic Multi-template Homology Modeling.  
Meier A, Söding J. PLoS Comput Biol. 2015 Oct 23;11(10):e1004343.

Loading hits...

---

If you use HHpred on our Toolkit for your research, please cite as appropriate:

A Completely Reimplemented MPI Bioinformatics Toolkit
with a New HHpred Server at its Core.  
Zimmermann L, Stephens A, Nam SZ, Rau D,
Kübler J, Lozajic M, Gabler F, Söding J, Lupas AN, Alva V.
J Mol Biol. 2018 Jul 20. S0022-2836(17)30587-9.

  

Protein homology detection by HMM-HMM comparison.  
Söding J. Bioinformatics. 2005 Apr 1;21(7):951-60.  
  
Fast and accurate automatic structure prediction with HHpred.  
Hildebrand A, Remmert M, Biegert A, Söding J. Proteins. 2009;77 Suppl 9:128-32.  
  
Automatic Prediction of Protein 3D Structures by Probabilistic Multi-template Homology Modeling.  
Meier A, Söding J. PLoS Comput Biol. 2015 Oct 23;11(10):e1004343.

Loading hits...

---

If you use HHpred on our Toolkit for your research, please cite as appropriate:

A Completely Reimplemented MPI Bioinformatics Toolkit
with a New HHpred Server at its Core.  
Zimmermann L, Stephens A, Nam SZ, Rau D,
Kübler J, Lozajic M, Gabler F, Söding J, Lupas AN, Alva V.
J Mol Biol. 2018 Jul 20. S0022-2836(17)30587-9.

  

Protein homology detection by HMM-HMM comparison.  
Söding J. Bioinformatics. 2005 Apr 1;21(7):951-60.  
  
Fast and accurate automatic structure prediction with HHpred.  
Hildebrand A, Remmert M, Biegert A, Söding J. Proteins. 2009;77 Suppl 9:128-32.  
  
Automatic Prediction of Protein 3D Structures by Probabilistic Multi-template Homology Modeling.  
Meier A, Söding J. PLoS Comput Biol. 2015 Oct 23;11(10):e1004343.

- Help
- FAQ
- Privacy Policy
- Imprint
- Contact Us
- Cite Us
- Recent Updates

© 2008-2020, Dept. of Protein Evolution, Max Planck Institute for Developmental Biology, Tübingen

Template 3D Structure: 
×

Loading...
