# Supplementary material for: Conserved sequence motifs in human TMTC1, TMTC2, TMTC3, and TMTC4, new O-mannosyltransferases from the GT-C/PMT clan, are rationalized as ligand binding sites
Source: Biol Direct. 2021 Jan 12;16:4. doi: 10.1186/s13062-021-00291-w (PMC7801869; doi:10.1186/s13062-021-00291-w)
Supplement: Supplementary file 3 — Additional file 3. HHPred outputs when searching TMTCs against Pfam or PDB structures. The compressed library file AF3-2020-06-HHPred-TMTCs.zip contains the outputs when running the four human TMTC sequences as input of HHPred against PDB sequences and against Pfam domains (as of 23rd of June 2020). [file 13062_2021_291_MOESM3_ESM.zip › AF3-2020-06-HHPred-TMTCs/HHpred_TMTC2_PFam.html]

HHpred | Bioinformatics Toolkit          **We're sorry but the Toolkit doesn't work properly without JavaScript enabled. Please enable it to continue.**

Sign In

- Search
- Alignment
- Sequence Analysis
- 2ary Structure
- 3ary Structure
- Classification
- Utils

- HHblits
- HHpred
- HMMER
- PatternSearch
- ProtBLAST/PSI-BLAST

Nothing found.

###### Tools

###### Jobs

ID

Date

Tool

8665047HHPR2161064HHPR

# HHpred

Job ID: 8665047,Created: 17 minutes ago

- Input
- Parameters
- Results
- Raw Output
- Probability Plot
- Query Template MSA
- Query MSA

>sp|Q8N394|TMTC2\_HUMAN Protein 1..475
MIAELVSSALGLALYLNTLSADFCYDDSRAIKTNQDLLPETPWTHIFYNDFWGTLLTHSG
SHKSYRPLCTLSFRLNHAIGGLNPWSYHLVNVLLHAAVTGLFTSFSKILLGDGYWTFMAG
LMFASHPIHTEAVAGIVGRADVGASLFFLLSLLCYIKHCSTRGYSARTWGWFLGSGLCAG
CSMLWKEQGVTVLAVSAVYDVFVFHRLKIKQILPTIYKRKNLSLFLSISLLIFWGSSLLG
ARLYWMGNKPPSFSNSDNPAADSDSLLTRTLTFFYLPTKNLWLLLCPDTLSFDWSMDAVP
LLKTVCDWRNLHTVAFYTGLLLLAYYGLKSPSVDRECNGKTVTNGKQNANGHSCLSDVEY
QNSETKSSFASKVENGIKNDVSQRTQLPSTENIVVLSLSLLIIPFVPATNLFFYVGFVIA
ERVLYIPSMGFCLLITVGARALYVKVQKRFLKSLIFYATATLIVFYGLKTAIRNG

Paste ExampleUpload File

Protein FASTA

Align two sequences/MSAs

Select structural/domain databases

Pfam-A\_v33.1

- PDB\_mmCIF70\_29\_May (default)
- PDB\_mmCIF30\_29\_May
- SCOPe70\_2.07
- ECOD\_ECOD\_F70\_20200207
- COG\_KOG\_v1.0
- Pfam-A\_v33.1
- NCBI\_Conserved\_Domains(CD)\_v3.18
- SMART\_v6.0
- TIGRFAMs\_v15.0
- PRK\_v6.9
- No elements found. Consider changing the search query.
- List is empty.

Select proteomes

Select options

- Euk\_Arabidopsis\_thaliana\_TAIR10\_20\_Jun\_2017
- Euk\_Bombyx\_mori\_p50T\_Dazao\_06\_May\_2019
- Euk\_Brachypodium\_distachyon\_23\_Aug\_2017
- Euk\_Caenorhabditis\_elegans\_18\_Jul\_2017
- Euk\_Capsaspora\_owczarzaki\_ATCC\_30864\_23\_Mar\_2020
- Euk\_Chaetomium\_thermophilum\_29\_Jun\_2017
- Euk\_Chlamydomonas\_reinhardtii\_27\_Jul\_2017
- Euk\_Entamoeba\_histolytica\_HM1\_IMSS\_22\_Mar\_2017
- Euk\_Dictyostelium\_discoideum\_AX4\_19\_Sep\_2017
- Euk\_Drosophila\_melanogaster\_19\_Jul\_2017
- Euk\_Giardia\_lamblia\_ATCC\_50803\_31\_Aug\_2017
- Euk\_Homo\_sapiens\_04\_Jul\_2017
- Euk\_Physcomitrella\_patens\_28\_Aug\_2017
- Euk\_Plasmodium\_falciparum\_3D7\_7\_Jun\_2017
- Euk\_Saccharomyces\_cerevisiae\_S288c\_11\_Mar\_2017
- Euk\_Schizosaccharomyces\_pombe\_19\_Sep\_2017
- Euk\_Solanum\_lycopersicum\_28\_Jul\_2019
- Euk\_Tetrahymena\_thermophila\_SB210\_22\_Aug\_2017
- Euk\_Toxoplasma\_gondii\_ME49\_10\_May\_2018
- Euk\_Trichomonas\_vaginalis\_G3\_21\_Nov\_2018
- Euk\_Trypanosoma\_brucei\_gambiense\_DAL972\_28\_Mar\_2017
- Euk\_Ustilago\_maydis\_521\_29\_May\_2017
- Euk\_Paramecium\_tetraurelia\_9\_Dec\_2018
- Arc\_Archaeoglobus\_fulgidus\_DSM\_4304\_5\_Dec\_2017
- Arc\_Halobacterium\_jilantaiense\_5\_Dec\_2017
- Arc\_Lokiarchaeum\_sp\_GC14\_75\_31\_Oct\_2018
- Arc\_Methanocaldococcus\_jannaschii\_DSM\_2661\_5\_Dec\_2017
- Arc\_Methanosarcina\_mazei\_S\_6\_17\_Mar\_2017
- Arc\_Methanothermus\_fervidus\_DSM\_2088\_5\_Dec\_2017
- Arc\_Pyrococcus\_horikoshii\_OT3\_5\_Dec\_2017
- Arc\_Sulfolobus\_solfataricus\_5\_Dec\_2017
- Arc\_Thermoplasma\_acidophilum\_DSM\_1728\_7\_Dec\_2017
- Bac\_Acinetobacter\_baumannii\_29\_Mar\_2018
- Bac\_Aquifex\_aeolicus\_VF5\_19\_Sep\_2017
- Bac\_Bacillus\_subtilis\_subsp\_subtilis\_str168\_19\_Mar\_2017
- Bac\_Bacteriovorax\_sp\_DB6\_IX\_1\_Jun\_2018
- Bac\_Bdellovibrio\_bacteriovorus\_HD100\_1\_Jun\_2018
- Bac\_Christensenella\_minuta\_2\_Apr\_2019
- Bac\_Deinococcus\_radiodurans\_R1\_19\_Sep\_2017
- Bac\_Enterococcus\_faecalis\_13\_SD\_W\_01\_1\_Jun\_2018
- Bac\_Escherichia\_coli\_K12\_07\_Mar\_2017
- Bac\_Fischerella\_muscicola\_PCC\_7414\_24\_Sep\_2017
- Bac\_Frankia\_alni\_ACN14a\_24\_Sep\_2017
- Bac\_Helicobacter\_pylori\_26695\_1\_Jun\_2018
- Bac\_Leptospira\_interrogans\_serovar\_Lai\_str56601\_1\_Jun\_2018
- Bac\_Mycobacterium\_tuberculosis\_H37Rv\_27\_May\_2017
- Bac\_Neisseria\_gonorrhoeae\_FA\_1090\_1\_Jun\_2018
- Bac\_Neisseria\_meningitidis\_MC58\_9\_Jun\_2017
- Bac\_Nostoc\_punctiforme\_PCC\_73102\_18\_Mar\_2017
- Bac\_Phycisphaerae\_bacterium\_L21\_RPulD3\_1\_Jun\_2018
- Bac\_Plesiocystis\_pacifica\_SIR1\_1\_Jun\_2018
- Bac\_Pseudomonas\_aeruginosa\_PAO1\_5\_Jun\_2017
- Bac\_Salmonella\_ent\_ser\_Typhi\_CT18\_22\_Nov\_2018
- Bac\_Staphylococcus\_aureus\_subsp\_aureus\_NCTC\_8325\_13\_Jun\_2017
- Bac\_Streptomyces\_scabiei\_87.22\_24\_Sep\_2017
- Bac\_Synechocystis\_sp\_PCC\_6803\_6\_Jun\_2017
- Bac\_Tenacibaculum\_dicentrarchi\_27\_Nov\_2017
- Bac\_Tenacibaculum\_maritimum\_NBRC\_15946\_27\_Nov\_2017
- Bac\_Thermus\_aquaticus\_Y51MC23\_24\_Sep\_2017
- Bac\_Thermus\_thermophilus\_HB8\_19\_Sep\_2017
- Bac\_Waddlia\_chondrophila\_WSU\_86\_1044\_1\_Jun\_2018
- Bac\_Yersinia\_pestis\_CO92\_10\_Apr\_2017
- Vir\_SARS-CoV-2\_31\_Mar\_2020
- No elements found. Consider changing the search query.
- List is empty.

ResubmitReset

MSA generation method

HHblits=>UniRef30

- HHblits=>UniRef30 (default)
- PSI-BLAST=>nr70
- No elements found. Consider changing the search query.
- List is empty.

Maximal no. of MSA generation steps

3

- 0
- 1
- 2
- 3 (default)
- 4
- 5
- 8
- No elements found. Consider changing the search query.
- List is empty.

E-value incl. threshold for MSA generation

1e-3

- 0.1
- 0.05
- 0.02
- 0.01
- 1e-3 (default)
- 1e-6
- 1e-8
- 1e-10
- 1e-15
- 1e-20
- 1e-30
- 1e-40
- 1e-50
- No elements found. Consider changing the search query.
- List is empty.

Min. seq. identity of MSA hits with query (%)

0

- 0 (default)
- 10
- 20
- 30
- 40
- 50
- 60
- 70
- 75
- 80
- 85
- 90
- 95
- 100
- No elements found. Consider changing the search query.
- List is empty.

Min. coverage of MSA hits (%)

20

- 10
- 20 (default)
- 30
- 40
- 50
- 60
- 70
- 80
- 90
- 100
- No elements found. Consider changing the search query.
- List is empty.

Secondary structure scoring

during\_alignment

- none
- after\_alignment
- during\_alignment (default)
- after\_alignment\_pred\_vs\_pred
- during\_alignment\_pred\_vs\_pred
- No elements found. Consider changing the search query.
- List is empty.

Alignment Mode:Realign with MAC

local:norealign

- local:norealign (default)
- local:realign
- global:realign
- No elements found. Consider changing the search query.
- List is empty.

MAC realignment threshold

0.3

- 0.0
- 0.01
- 0.1
- 0.2
- 0.3 (default)
- 0.4
- 0.5
- 0.6
- 0.7
- 0.8
- 0.9
- 0.95
- No elements found. Consider changing the search query.
- List is empty.

No. of target sequences (up to 10000)

250

- 250 (default)
- 500
- 1000
- 2000
- 3000
- 4000
- 5000
- 6000
- 7000
- 8000
- 9000
- 10000
- No elements found. Consider changing the search query.
- List is empty.

Min. probability in hit list (> 10%)

20

- 10
- 20 (default)
- 30
- 40
- 50
- 60
- 70
- 75
- 80
- 85
- 90
- 95
- 100
- No elements found. Consider changing the search query.
- List is empty.

ResubmitReset

VisHitsAln
Select AllForwardForward Query A3MDownload HHRColor SeqsWrap Seqs

Number of Hits: **28**

Detected sequence features:
**◾Transmembrane segment(s)****◾Signal peptide**

#### Visualization

Resubmit Section

3

475

Prob=99.6% E=1.9E-12 PF04188.14 ; Mannosyl\_trans2 ; Mannosyltransferase (PIG-V)

#### Hitlist

Show102550100AllEntries

Search:

| Nr (Click to sort Ascending) | Hit (Click to sort Ascending) | Name (Click to sort Ascending) | Probability (Click to sort Ascending) | E-value (Click to sort Ascending) | SS (Click to sort Ascending) | Cols (Click to sort Ascending) | Target Length (Click to sort Ascending) |
| --- | --- | --- | --- | --- | --- | --- | --- |
| 1 | PF03901.18 | ; Glyco\_transf\_22 ; Alg9-like mannosyltransferase family | 99.83 | 8.1e-18 | 31.8 | 346 | 388 |
| 2 | PF02516.15 | ; STT3 ; Oligosaccharyl transferase STT3 subunit | 99.82 | 2.1e-17 | 34 | 368 | 458 |
| 3 | PF09852.10 | ; DUF2079 ; Predicted membrane protein (DUF2079) | 99.77 | 1.3e-15 | 36.1 | 341 | 519 |
| 4 | PF07220.12 | ; DUF1420 ; Protein of unknown function (DUF1420) | 99.69 | 5.5e-14 | 35.8 | 363 | 670 |
| 5 | PF10131.10 | ; PTPS\_related ; 6-pyruvoyl-tetrahydropterin synthase related domain; membrane protein | 99.65 | 9.7e-14 | 30.7 | 304 | 616 |
| 6 | PF02366.19 | ; PMT ; Dolichyl-phosphate-mannose-protein mannosyltransferase | 99.61 | 1.3e-13 | 23.9 | 217 | 247 |
| 7 | PF10034.10 | ; Dpy19 ; Q-cell neuroblast polarisation | 99.59 | 1.8e-12 | 33.5 | 349 | 651 |
| 8 | PF04188.14 | ; Mannosyl\_trans2 ; Mannosyltransferase (PIG-V) | 99.59 | 1.9e-12 | 31.7 | 335 | 432 |
| 9 | PF12250.9 | ; AftA\_N ; Arabinofuranosyltransferase N terminal | 99.48 | 3.7e-11 | 30.5 | 339 | 432 |
| 10 | PF13231.7 | ; PMT\_2 ; Dolichyl-phosphate-mannose-protein mannosyltransferase | 99.44 | 1.1e-11 | 20.1 | 156 | 159 |
| 11 | PF09913.10 | ; DUF2142 ; Predicted membrane protein (DUF2142) | 99.42 | 2.7e-11 | 24.4 | 333 | 405 |
| 12 | PF11028.9 | ; DUF2723 ; Protein of unknown function (DUF2723) | 99.4 | 8.1e-12 | 17.1 | 156 | 188 |
| 13 | PF04602.13 | ; Arabinose\_trans ; Mycobacterial cell wall arabinan synthesis protein | 99.29 | 5.9e-9 | 31.8 | 347 | 471 |
| 14 | PF06728.14 | ; PIG-U ; GPI transamidase subunit PIG-U | 99.29 | 7.8e-9 | 32.7 | 328 | 363 |
| 15 | PF15971.6 | ; Mannosyl\_trans4 ; DolP-mannose mannosyltransferase | 99.2 | 1e-9 | 18.7 | 153 | 163 |
| 16 | PF09586.11 | ; YfhO ; Bacterial membrane protein YfhO | 99.07 | 9.4e-8 | 31.4 | 370 | 832 |
| 17 | PF03155.16 | ; Alg6\_Alg8 ; ALG6, ALG8 glycosyltransferase family | 98.91 | 9.4e-7 | 29.2 | 319 | 470 |
| 18 | PF04922.13 | ; DIE2\_ALG10 ; DIE2/ALG10 family | 98.9 | 1.1e-7 | 21.2 | 195 | 434 |
| 19 | PF14264.7 | ; Glucos\_trans\_II ; Glucosyl transferase GtrII | 98.84 | 0.0000028 | 28.2 | 303 | 312 |
| 20 | PF05208.14 | ; ALG3 ; ALG3 protein | 98.69 | 0.0000026 | 22.9 | 200 | 356 |
| 21 | PF09594.11 | ; GT87 ; Glycosyltransferase family 87 | 98.56 | 0.0000064 | 20.7 | 243 | 251 |
| 22 | PF05007.14 | ; Mannosyl\_trans ; Mannosyltransferase (PIG-M) | 95.04 | 0.67 | 16.1 | 192 | 269 |
| 23 | PF14897.7 | ; EpsG ; EpsG family | 88.29 | 8.4 | 34.3 | 298 | 319 |
| 24 | PF16192.6 | ; PMT\_4TMC ; C-terminal four TMM region of protein-O-mannosyltransferase | 81.72 | 13 | 12.1 | 104 | 198 |
| 25 | PF09971.10 | ; DUF2206 ; Predicted membrane protein (DUF2206) | 80.25 | 22 | 19.9 | 222 | 390 |

Displaying 1 to 25 of 28 hits

- «
- ‹
- 1
- 2
- ›
- »

#### Alignments

|  |  |  |  |
| --- | --- | --- | --- |
|  | | | |
|  | Template alignmentCDD | | |
| 1. | PF03901.18 ; Glyco\_transf\_22 ; Alg9-like mannosyltransferase family | | |
|  | Probability: 99.83%, E-value: 8.1e-18, Score: 159.88, Aligned cols: 346, Identities: 10%, Similarity: -0.025, | | |
|  |
|  | Q ss\_pred |  | HHHHHHHHHHHHHHhhcCCCCcccch-hHHHhchhhCCCCchhHhhhcccccccccCCCCcccCChHHHHHHHHHHHHhC |
|  | Q Q8N394 | 3 | AELVSSALGLALYLNTLSADFCYDDS-RAIKTNQDLLPETPWTHIFYNDFWGTLLTHSGSHKSYRPLCTLSFRLNHAIGG   81 (475) |
|  | Q Consensus | 3 | ~~lll~~~~~~~~~~~~~~~~~~De~-~~~~~~~~~~~~~~~~~~~~~~~~~~~~~~~~~~~~~~Pl~~~l~~~~~~lfG   81 (475) |
|  |  |  | +++++.++............+..||. .+...++++.+++......... +.....+||++..+......++| |
|  | T Consensus | 1 | lil~~~~~l~l~~~~~~~~~~~~De~~~~~~~a~~~~~~~~~~~~~~~~--------~~~~~~~p~~~~~~~~~~~~~~~   72 (388) |
|  | T PF03901.18 | 1 | YLLLFTIALRILNCFLVQTSFVPDEYWQSLEVSHHMVFNYGYLTWEWTE--------RLRSYTYPLIFASIYKILHLLGK   72 (388) |
|  | T ss\_pred |  | CHHHHHHHHHHHHHHhhhcCCCchHHHHHHHHHhhhccccCcCCCcccc--------cccCCHHHHHHHHHHHHHHHcCC |
|  |
|  |
|  | Q ss\_pred |  | CCh----HHHHHHHHHHHHHHHHHHHHHHHHHhCChHHHHHHHHHHHHCHHhHHHHHhhhchHHHHHHHHHHHHHHHHHH |
|  | Q Q8N394 | 82 | LNP----WSYHLVNVLLHAAVTGLFTSFSKILLGDGYWTFMAGLMFASHPIHTEAVAGIVGRADVGASLFFLLSLLCYIK   157 (475) |
|  | Q Consensus | 82 | ~~~----~~~rl~~~l~~~l~~~lly~l~r~l~~~~~~al~aall~a~~P~~~~~~~~~~~~~~~~~~~f~ll~l~~~l~   157 (475) |
|  |  |  | .++ ...|+.+.++++++++++|.++|++.+ ++.+++++++++++|....++..... |.+..++.+++++++.+ |
|  | T Consensus | 73 | ~~~~~~~~~~r~~~~l~~~~~~~~~y~l~~~~~~-~~~a~~a~~l~~~~p~~~~~~~~~~~--~~~~~~~~~~~~~~~~~   149 (388) |
|  | T PF03901.18 | 73 | DSVQLLIWIPRLAQALLSAVADVRLYSLMKQLEN-QEVARWVFFCQLCSWFTWYCCTRTLT--NTMETVLTIIALFYYPL   149 (388) |
|  | T ss\_pred |  | CCHHHHHHHHHHHHHHHHHHHHHHHHHHHHHHcC-hhHHHHHHHHHHHhHHHHHHHhhhch--HHHHHHHHHHHHHHHHH |
|  |
|  |
|  | Q ss\_pred |  | HHccCCCCcchHHHHHHHHHHHHHHHHhHhHHHHHHHHHHHHHHHHHcccchhhhcchHhhhHHHHHHHHHHHHHHHHHH |
|  | Q Q8N394 | 158 | HCSTRGYSARTWGWFLGSGLCAGCSMLWKEQGVTVLAVSAVYDVFVFHRLKIKQILPTIYKRKNLSLFLSISLLIFWGSS   237 (475) |
|  | Q Consensus | 158 | ~~~~~~~~~~~~~~~~l~~l~~~la~ltk~~~~~~~~~~~~~~l~~~~~~~~~~~~~~~~~~~~~~~~~~~~~~~~~~~~   237 (475) |
|  |  |  | ..+++ +++.. ++++.+++.++|+.+..+.+...+..+...+++ +++ ..........+........ |
|  | T Consensus | 150 | ~~~~~-----~~~~~--~~~~~~l~~~~k~~~~~~~~~~~~~~~~~~~~~-~~~-------~~~~~~~~~~~~~~~~~~~   214 (388) |
|  | T PF03901.18 | 150 | EGSKS-----MNSVK--YSSLVALAFIIRPTAVILWTPLLFRHFCQEPRK-LDL-------ILHHFLPVGFVTLSLSLMI   214 (388) |
|  | T ss\_pred |  | HhCCC-----CcHHH--HHHHHHHHHHhcchHHHHHHHHHHHHHHcChhc-HHH-------HHHHHHHHHHHHHHHHHHH |
|  |
|  |
|  | Q ss\_pred |  | HHHHHHHHhcCCCCCccCCCCCCcCCChHHHHHHHHHHHHHHhHHHhhCcccccccccccccccccccCCHHHHHHHHHH |
|  | Q Q8N394 | 238 | LLGARLYWMGNKPPSFSNSDNPAADSDSLLTRTLTFFYLPTKNLWLLLCPDTLSFDWSMDAVPLLKTVCDWRNLHTVAFY   317 (475) |
|  | Q Consensus | 238 | ~~~~~~~~~~~~~~~~~~~~~~~~~~~~~~~~~~~~~~~~~~~~~~~~~p~~~~~~~~~~~~~~~~~~~~~~~~~~~~~~   317 (475) |
|  |  |  | ....................................... .......... |
|  | T Consensus | 215 | ~~~~~~~~~~~~~~~~~~~~~~~~~~~~~~~~~~~~~~~-------------------------------~~~~~~~~~~   263 (388) |
|  | T PF03901.18 | 215 | DRIFFGQWTLVQFNFLKFNVLQNWGTFYGSHPWHWYFSQ-------------------------------GFPVILGTHL   263 (388) |
|  | T ss\_pred |  | HHHHhcchhhhhhhhhhhhcccccccccccCchHHHHHh-------------------------------hhhhhHHhHH |
|  |
|  |
|  | Q ss\_pred |  | HHHHHHHHHHhhCcccccccCCCcccCCccccCCcccccccccccccccccccchhhcCCcCCcccccCCCCCCchHHhh |
|  | Q Q8N394 | 318 | TGLLLLAYYGLKSPSVDRECNGKTVTNGKQNANGHSCLSDVEYQNSETKSSFASKVENGIKNDVSQRTQLPSTENIVVLS   397 (475) |
|  | Q Consensus | 318 | ~~~~~~~~~~~~~~~~~~~~~~~~~~~~~~~~~~~~~~~~~~~~~~~~~~~~~~~~~~~~~~~~~~~~~~~~~~~~~~~~   397 (475) |
|  |  |  | +.........+|+ ++ .. |
|  | T Consensus | 264 | ~~~~~~~~~~~~~------------------------------------------------------------~~---~~   280 (388) |
|  | T PF03901.18 | 264 | PFFIHGCYLAPKR------------------------------------------------------------YR---IL   280 (388) |
|  | T ss\_pred |  | HHHHHHHHHchhh------------------------------------------------------------hH---HH |
|  |
|  |
|  | Q ss\_pred |  | hHHHHHHHhhhcCccccCChhhHHHHhHHHHHHHHHHHHHHHHHHHHHhchHHHHHHHHHHHHHHHHHHHHHHHHhcC |
|  | Q Q8N394 | 398 | LSLLIIPFVPATNLFFYVGFVIAERVLYIPSMGFCLLITVGARALYVKVQKRFLKSLIFYATATLIVFYGLKTAIRNG   475 (475) |
|  | Q Consensus | 398 | l~~~~~~~~~~~~~~~~~~~~~~~Ry~~~~~~~~~ll~~~~l~~~~~~~~~~~~~~~~~~~~~~~~~~~~~~~~~~~~   475 (475) |
|  |  |  | ..+.+..++..... ....+||..+..|+++++++.++.++.++.+++. +....++++.............. |
|  | T Consensus | 281 | ~~~~~~~~~~~~~~-----~~~~~ry~~~~~p~~~l~~~~~~~~~~~~~~~~~--~~~~~~~~~~~~~~~~~~~~~~~   351 (388) |
|  | T PF03901.18 | 281 | LVTVLWTLLVYSML-----SHKEFRFIYPVLPFCMVFCGYSLTHLKTWKKPAL--SFLFLSNLFLALYTGLVHQRGTL   351 (388) |
|  | T ss\_pred |  | HHHHHHHHHHHHhh-----cCCCcchHhcHHHHHHHHHHHHHHhhhcccchHH--HHHHHHHHHHHHHHHHHhhccch |
|  |
| --- | | | |
|  | Template alignmentCDD | | |
| 2. | PF02516.15 ; STT3 ; Oligosaccharyl transferase STT3 subunit | | |
|  | Probability: 99.82%, E-value: 2.1e-17, Score: 160.55, Aligned cols: 368, Identities: 10%, Similarity: -0.006, | | |
|  |
|  | Q ss\_pred |  | CHHHHHHHHHHHHHH-----HhhcCCCCcccchhHHHhchhhCCCCc--hhHhhhcccccccccCCCCcccCChHHHHHH |
|  | Q Q8N394 | 1 | MIAELVSSALGLALY-----LNTLSADFCYDDSRAIKTNQDLLPETP--WTHIFYNDFWGTLLTHSGSHKSYRPLCTLSF   73 (475) |
|  | Q Consensus | 1 | ~~~~lll~~~~~~~~-----~~~~~~~~~~De~~~~~~~~~~~~~~~--~~~~~~~~~~~~~~~~~~~~~~~~Pl~~~l~   73 (475) |
|  |  |  | .+++++++++....+ ..........||..+...++++.+++. -......+++.....+.+....++|++.++. |
|  | T Consensus | 4 | ~~~l~~i~~~~~~~r~~~~~~~~~~~~~~~D~~~~~~~a~~~~~~~~~~~~~~~~~~~~~~~~~~~~~~~~~~p~~~~l~   83 (458) |
|  | T PF02516.15 | 4 | RIETAELKGMNTADRAYFTDENGLPYMYEPDSYYNYRLTANILDHGHPGDKIINGTPWDLHSNYPPGNRVNYPPLILWIS   83 (458) |
|  | T ss\_pred |  | hHhHHHHHhhhhhhHHhCCCCCCCCcccCCCcHHHHHHHHHHHHhCCCCCcccCCCCCCchhcCCCCCCCCCCchHHHHH |
|  |
|  |
|  | Q ss\_pred |  | HHHHHHhCCCh-----HHHHHHHHHHHHHHHHHHHHHHHHHhCChHHHHHHHHHHHHCHHhHHHHHhhhchHHHHHHHHH |
|  | Q Q8N394 | 74 | RLNHAIGGLNP-----WSYHLVNVLLHAAVTGLFTSFSKILLGDGYWTFMAGLMFASHPIHTEAVAGIVGRADVGASLFF   148 (475) |
|  | Q Consensus | 74 | ~~~~~lfG~~~-----~~~rl~~~l~~~l~~~lly~l~r~l~~~~~~al~aall~a~~P~~~~~~~~~~~~~~~~~~~f~   148 (475) |
|  |  |  | ....+++|... ...|+.+.++++++++.+|.++|++.+ +..|++++++++++|.+...+......+|.+..++. |
|  | T Consensus | 84 | ~~~~~~~~~~~~~~~~~~~~~~~~~~~~l~~~~~y~l~~~~~~-~~~a~~a~~l~~~~p~~~~~~~~~~~~~~~~~~~~~   162 (458) |
|  | T PF02516.15 | 84 | LLFHNFINLFIPFSLIETCFWLPAIIGPLAGIVMFFMVRRYAG-DLPGLLSGVLLVLAPVYFSRTVPGFFDTDMFNIIFP   162 (458) |
|  | T ss\_pred |  | HHHHHHHHhhCCCcHHHHHhHHHHHHHHHHHHHHHHHHHHHcC-cHHHHHHHHHHHHcHHHHHhhCCCCCCchHHHHHHH |
|  |
|  |
|  | Q ss\_pred |  | HHHHHHHHHHHccCCCCcch--HHHHHHHHHHHHHHHHhHhHHHHHHHHHHHHHHHHHcccchhhhcchHhhhHHHHHHH |
|  | Q Q8N394 | 149 | LLSLLCYIKHCSTRGYSART--WGWFLGSGLCAGCSMLWKEQGVTVLAVSAVYDVFVFHRLKIKQILPTIYKRKNLSLFL   226 (475) |
|  | Q Consensus | 149 | ll~l~~~l~~~~~~~~~~~~--~~~~~l~~l~~~la~ltk~~~~~~~~~~~~~~l~~~~~~~~~~~~~~~~~~~~~~~~~   226 (475) |
|  |  |  | +++++++.+..+++ + +++.++++++.+++.++|+......+...+......++++.++ +....... |
|  | T Consensus | 163 | ~l~~~~~~~~~~~~-----~~~~~~~~l~g~~~~l~~~~~~~~~~~~~~~~~~~~~~~~~~~~~~-------~~~~~~~~   230 (458) |
|  | T PF02516.15 | 163 | LLVIFFLLKATETK-----NNYMFPLLLSSFSLALLSLSWNGWAYIFYIIIISSILYMTLCKLKG-------KAVMGFSR   230 (458) |
|  | T ss\_pred |  | HHHHHHHHHHhccC-----CCchHHHHHHHHHHHHHHhHhhHHHHHHHHHHHHHHHHHHHhhccc-------chhhHHHH |
|  |
|  |
|  | Q ss\_pred |  | HHHHHHHHHHHHHHHHH-------------HHhcCCCCC--ccCCCCCCcCCChHHHHHHHHHHHHHHhHHHhhCccccc |
|  | Q Q8N394 | 227 | SISLLIFWGSSLLGARL-------------YWMGNKPPS--FSNSDNPAADSDSLLTRTLTFFYLPTKNLWLLLCPDTLS   291 (475) |
|  | Q Consensus | 227 | ~~~~~~~~~~~~~~~~~-------------~~~~~~~~~--~~~~~~~~~~~~~~~~~~~~~~~~~~~~~~~~~~p~~~~   291 (475) |
|  |  |  | ...........+..... ......... ........................ |
|  | T Consensus | 231 | ~~~~~~~~~~~~~~~~~~~~~~~~~~~~~~~~~~~~~~~~~~~~~~~~~~~~~~~~~~~~~~~~~---------------   295 (458) |
|  | T PF02516.15 | 231 | KIAVFVIISLLIIGLAGRLGYALIFPTFFKFTFKSLSAGGWPGIFESISELSAPTFDEFLSLPGP---------------   295 (458) |
|  | T ss\_pred |  | HHHHHHHHHHHHhhhcccccHHHHHHHHHHHhcccccCCCCCcchHHHHHHhcccHHHHhcCchH--------------- |
|  |
|  |
|  | Q ss\_pred |  | ccccccccccccccCCHHHHHHHHHHHHHHHHHHHHhhCcccccccCCCcccCCccccCCcccccccccccccccccccc |
|  | Q Q8N394 | 292 | FDWSMDAVPLLKTVCDWRNLHTVAFYTGLLLLAYYGLKSPSVDRECNGKTVTNGKQNANGHSCLSDVEYQNSETKSSFAS   371 (475) |
|  | Q Consensus | 292 | ~~~~~~~~~~~~~~~~~~~~~~~~~~~~~~~~~~~~~~~~~~~~~~~~~~~~~~~~~~~~~~~~~~~~~~~~~~~~~~~~   371 (475) |
|  |  |  | ..........+..........++++++++ |
|  | T Consensus | 296 | ----------------~~~~~~~~~~~~~~~~~~~~~~~~~~~~~-----------------------------------   324 (458) |
|  | T PF02516.15 | 296 | ----------------VNMGIGLFGFVIIGSIMLRDEIKRVHLPD-----------------------------------   324 (458) |
|  | T ss\_pred |  | ----------------HHHHHHHHHHHHHHHHHHHHHHHhccCCC----------------------------------- |
|  |
|  |
|  | Q ss\_pred |  | hhhcCCcCCcccccCCCCCCchHHhhhHHHHHHHhhhcCccccCChhhHHHHhHHHHHHHHHHHHHHHHHHHHHhchHHH |
|  | Q Q8N394 | 372 | KVENGIKNDVSQRTQLPSTENIVVLSLSLLIIPFVPATNLFFYVGFVIAERVLYIPSMGFCLLITVGARALYVKVQKRFL   451 (475) |
|  | Q Consensus | 372 | ~~~~~~~~~~~~~~~~~~~~~~~~~~l~~~~~~~~~~~~~~~~~~~~~~~Ry~~~~~~~~~ll~~~~l~~~~~~~~~~~~   451 (475) |
|  |  |  | ................... .....||.++..|+++++++.++..+.++.+++.. |
|  | T Consensus | 325 | -------------------~~~~~~~~~~~~~~~~~~~-------~~~~~Ry~~~~~p~~~i~~~~~~~~~~~~~~~~~~   378 (458) |
|  | T PF02516.15 | 325 | -------------------FSWYPFILIGIWLIIGLAA-------YSLSTRFALLVIPPLIIFLGLLMGVMASYLKGSPS   378 (458) |
|  | T ss\_pred |  | -------------------CccHHHHHHHHHHHHHHHH-------HHHHHhHHHhhHHHHHHHHHHHHHHHHHHhcCCcc |
|  |
|  |
|  | Q ss\_pred |  | HHHHHHHHHHHHHHHHHHHHHh |
|  | Q Q8N394 | 452 | KSLIFYATATLIVFYGLKTAIR   473 (475) |
|  | Q Consensus | 452 | ~~~~~~~~~~~~~~~~~~~~~~   473 (475) |
|  |  |  | ++.......+.+.......... |
|  | T Consensus | 379 | ~~~~~~~~~~~~~~~~~~~~~~   400 (458) |
|  | T PF02516.15 | 379 | MRLRRSGNVFILSLVVMLSTIS   400 (458) |
|  | T ss\_pred |  | chHHHHHHHHHHHHHHHHHHHH |
|  |
| --- | | | |
|  | Template alignmentCDD | | |
| 3. | PF09852.10 ; DUF2079 ; Predicted membrane protein (DUF2079) | | |
|  | Probability: 99.77%, E-value: 1.3e-15, Score: 150.77, Aligned cols: 341, Identities: 9%, Similarity: -0.034, | | |
|  |
|  | Q ss\_pred |  | CCCcccchhHHHhchhhCCCCchhHhhhcccccccccCCCCcccCChHHHHHHHHHHHHhCCChHHHHHHHHHHHHHHHH |
|  | Q Q8N394 | 21 | ADFCYDDSRAIKTNQDLLPETPWTHIFYNDFWGTLLTHSGSHKSYRPLCTLSFRLNHAIGGLNPWSYHLVNVLLHAAVTG   100 (475) |
|  | Q Consensus | 21 | ~~~~~De~~~~~~~~~~~~~~~~~~~~~~~~~~~~~~~~~~~~~~~Pl~~~l~~~~~~lfG~~~~~~rl~~~l~~~l~~~   100 (475) |
|  |  |  | ....+||..+...++++.+++.+....... .....++|++.++.++.+.++| ++...|++++++++++++ |
|  | T Consensus | 6 | ~~~~~De~~~~~~a~~~~~g~~~~~~~~~~---------~~~~~~~pl~~~l~a~~~~l~g-~~~~~rl~~~l~~~~~~~   75 (519) |
|  | T PF09852.10 | 6 | NATAYDLGIYVSILENTMHGHVMYANPLLI---------NSFSEHFSPFLFVIYPIYWFFP-YVKTLLIMQSVMISFSGL   75 (519) |
|  | T ss\_pred |  | CccccHHHHHHHHHHHHHcCCccccCcccc---------ccccccchhHHHHHHHHHHHcC-ChHHHHHHHHHHHHHHHH |
|  |
|  |
|  | Q ss\_pred |  | HHHHHHHHHhC-C--------hHHHHHHHHHHHHCHHhHHHH-HhhhchHHHHHHHHHHHHHHHHHHHHccCCCCcchHH |
|  | Q Q8N394 | 101 | LFTSFSKILLG-D--------GYWTFMAGLMFASHPIHTEAV-AGIVGRADVGASLFFLLSLLCYIKHCSTRGYSARTWG   170 (475) |
|  | Q Consensus | 101 | lly~l~r~l~~-~--------~~~al~aall~a~~P~~~~~~-~~~~~~~~~~~~~f~ll~l~~~l~~~~~~~~~~~~~~   170 (475) |
|  |  |  | ++|+++|++++ + +..|++++++++++|.....+ ...+. |.+..++++++++++++. ++ |
|  | T Consensus | 76 | ~~y~l~r~~~~~~~~~~~~~~~~~al~a~ll~~~~p~~~~~~~~~~~~--~~~~~~~~~~~~~~~~~~--~~--------   143 (519) |
|  | T PF09852.10 | 76 | VIYLLAREIFFINNFKKDILLEMLALFISTSYILSPYIESPLSFDFHL--MPFLILFVPLSFYFFMKK--YK--------   143 (519) |
|  | T ss\_pred |  | HHHHHHHHHhccCcccchhHHHHHHHHHHHHHHHcHHhhhhhhcCCch--HHHHHHHHHHHHHHHHhh--cc-------- |
|  |
|  |
|  | Q ss\_pred |  | HHHHHHHHHHHHHHhHhHHHHHHHHHHHHHHHH-----------------H----------cccchhhhcchHhhhHHHH |
|  | Q Q8N394 | 171 | WFLGSGLCAGCSMLWKEQGVTVLAVSAVYDVFV-----------------F----------HRLKIKQILPTIYKRKNLS   223 (475) |
|  | Q Consensus | 171 | ~~~l~~l~~~la~ltk~~~~~~~~~~~~~~l~~-----------------~----------~~~~~~~~~~~~~~~~~~~   223 (475) |
|  |  |  | ..++++++++++++|+.+.++.+.+.+..+.. . ++++.....+...+..... |
|  | T Consensus | 144 | -~~~~gl~~~la~l~k~~~~~~~~~~~l~~~~~~~~~~~~~~~~~~~~~~~~~~~~~~~~~~~~~~~~~~~~~~~~~~~~   222 (519) |
|  | T PF09852.10 | 144 | -ILNLIVLILIISLHSLFVIMVFFIISYQFIFRIRNEGNLNCHKIIRTIANINISDNLKKTPKSKYVLQKIVRSKTLIKI   222 (519) |
|  | T ss\_pred |  | -HHHHHHHHHHHHHhcchHHHHHHHHHHHHHHHHhccCCCcchhhhHHhhhcccccccccCCcchhhhhhhhcchHHHHH |
|  |
|  |
|  | Q ss\_pred |  | HHHHHHHHHHHHHHHHHHHHHHhcCCCCCccCCCCCCcCCChHHHHHHHHHHHHHHhHHHhhCccccccccccccccccc |
|  | Q Q8N394 | 224 | LFLSISLLIFWGSSLLGARLYWMGNKPPSFSNSDNPAADSDSLLTRTLTFFYLPTKNLWLLLCPDTLSFDWSMDAVPLLK   303 (475) |
|  | Q Consensus | 224 | ~~~~~~~~~~~~~~~~~~~~~~~~~~~~~~~~~~~~~~~~~~~~~~~~~~~~~~~~~~~~~~~p~~~~~~~~~~~~~~~~   303 (475) |
|  |  |  | ........+..............+.................+..+...+.............. |
|  | T Consensus | 223 | ~~~~~~~~~~~~~~~~~~~~~~~~~~~~~~~~~~~~~~~~~~~~~~~~~~~~~~~~~~~~~~~-----------------   285 (519) |
|  | T PF09852.10 | 223 | IITLILLVGYLYFASLMKTFIASGAVALSPPSTMSTGSVSSSLAGLFTDLFTRPMLIESAFLI-----------------   285 (519) |
|  | T ss\_pred |  | HHHHHHHHHHHHHHHHHHHHHHccccccCCCcccCCCCcCCcHHHHHHHHhhCchHHHHHHhc----------------- |
|  |
|  |
|  | Q ss\_pred |  | ccCCHHHHHHHHHHHHHHHHHHHHhhCcccccccCCCcccCCccccCCcccccccccccccccccccchhhcCCcCCccc |
|  | Q Q8N394 | 304 | TVCDWRNLHTVAFYTGLLLLAYYGLKSPSVDRECNGKTVTNGKQNANGHSCLSDVEYQNSETKSSFASKVENGIKNDVSQ   383 (475) |
|  | Q Consensus | 304 | ~~~~~~~~~~~~~~~~~~~~~~~~~~~~~~~~~~~~~~~~~~~~~~~~~~~~~~~~~~~~~~~~~~~~~~~~~~~~~~~~   383 (475) |
|  |  |  | ............++.......++ |
|  | T Consensus | 286 | ----~~~~~~~~~~~~~~~~~~~~~~~-----------------------------------------------------   308 (519) |
|  | T PF09852.10 | 286 | ----NFPDKIIFAFYAFANTGFLVFLD-----------------------------------------------------   308 (519) |
|  | T ss\_pred |  | ----cchHHHHHHHHHHHHHhHHHhcC----------------------------------------------------- |
|  |
|  |
|  | Q ss\_pred |  | ccCCCCCCchHHhhhHHHHHHHhhhcCccccCChhhHHHHhHHHHHHHHHHHHHHHHHHH-------HHhchHHHHHHHH |
|  | Q Q8N394 | 384 | RTQLPSTENIVVLSLSLLIIPFVPATNLFFYVGFVIAERVLYIPSMGFCLLITVGARALY-------VKVQKRFLKSLIF   456 (475) |
|  | Q Consensus | 384 | ~~~~~~~~~~~~~~l~~~~~~~~~~~~~~~~~~~~~~~Ry~~~~~~~~~ll~~~~l~~~~-------~~~~~~~~~~~~~   456 (475) |
|  |  |  | .......+.+++..++.. .........||.++.+|+++++++.++.++. +..++++.++... |
|  | T Consensus | 309 | -------~~~~~~~~~~~~~~~~~~----~~~~~~~~~ry~~~~~p~l~i~~~~~~~~~~~~~~~~~~~~~~~~~~~~~~   377 (519) |
|  | T PF09852.10 | 309 | -------PLSLLMDIPYFLYAYLSS----YGPYYSLGYQYSTMIIPFIFIGALFGIRKIVQSARATDSDDVRRTIKKILV   377 (519) |
|  | T ss\_pred |  | -------HHHHHhHHHHHHHHHhcc----CccccchhhHHHHhHHHHHHHHHHHHHHHHHHHhccCCCcchhHhHHHHHH |
|  |
|  |
|  | Q ss\_pred |  | HHHHHHHHHHHHH |
|  | Q Q8N394 | 457 | YATATLIVFYGLK   469 (475) |
|  | Q Consensus | 457 | ~~~~~~~~~~~~~   469 (475) |
|  |  |  | .+++++++..... |
|  | T Consensus | 378 | ~~~~~~~~~~~~~   390 (519) |
|  | T PF09852.10 | 378 | GVISIVIVSSLFE   390 (519) |
|  | T ss\_pred |  | HHHHHHHHHHHHh |
|  |
| --- | | | |
|  | Template alignmentCDD | | |
| 4. | PF07220.12 ; DUF1420 ; Protein of unknown function (DUF1420) | | |
|  | Probability: 99.69%, E-value: 5.5e-14, Score: 143.5, Aligned cols: 363, Identities: 12%, Similarity: -0.001, | | |
|  |
|  | Q ss\_pred |  | HHHHHHHHHHHHHHHhhcCCCCcccc-hhHHHhchhhCCCCchhHhhhcccccccccCCCCcccCChHHHHHHHHHHHHh |
|  | Q Q8N394 | 2 | IAELVSSALGLALYLNTLSADFCYDD-SRAIKTNQDLLPETPWTHIFYNDFWGTLLTHSGSHKSYRPLCTLSFRLNHAIG   80 (475) |
|  | Q Consensus | 2 | ~~~lll~~~~~~~~~~~~~~~~~~De-~~~~~~~~~~~~~~~~~~~~~~~~~~~~~~~~~~~~~~~Pl~~~l~~~~~~lf   80 (475) |
|  |  |  | ....++++............+..+|| .+|...++.+.+++.......... ..+||...++......+. |
|  | T Consensus | 158 | ~l~~~i~~~~~~~~~~~~~p~~~~D~~~yhl~~a~~~~~~g~~~~~~~~~~-----------~~~P~~~~~l~~~~~~l~   226 (670) |
|  | T PF07220.12 | 158 | VLNVFIILLMIGYGFLALCPITNADSLDYHIGVAIEILNQGKMPVFSGWFH-----------GRLAGSGEVLNALGLAIG   226 (670) |
|  | T ss\_pred |  | HHHHHHHHHHHHHHHHHcCCCCCCHHHHHHHHHHHHHHHcCCCCCCCCchh-----------hcCCchHHHHHHHHHHHc |
|  |
|  |
|  | Q ss\_pred |  | CCChHHHHHHHHHHHHHHHHHHHHHHHHHhCCh--HHHHHHHHHHHHCHHhHHHHHh---hhchHHHHHHHHHHHHHHHH |
|  | Q Q8N394 | 81 | GLNPWSYHLVNVLLHAAVTGLFTSFSKILLGDG--YWTFMAGLMFASHPIHTEAVAG---IVGRADVGASLFFLLSLLCY   155 (475) |
|  | Q Consensus | 81 | G~~~~~~rl~~~l~~~l~~~lly~l~r~l~~~~--~~al~aall~a~~P~~~~~~~~---~~~~~~~~~~~f~ll~l~~~   155 (475) |
|  |  |  | +.......-.+.+++++++.++|.++|+.++ + ..+.+++++++++|.+...++. .+. |.+.+++.+++++.+ |
|  | T Consensus | 227 | ~~~~~~~~~~~~l~~~l~~~~~y~l~r~~~~-~~~~~a~~aall~~~~p~~~~~s~~~~~~~~--d~~~~~~~~~~l~~~   303 (670) |
|  | T PF07220.12 | 227 | AEQFGSLLQFCGLLSIYGILSFYSFAEKFSE-SDGVWRKIIIIAFLSSPVLVFLVSSPKPQLL--QIGMTSFAITLLLEI   303 (670) |
|  | T ss\_pred |  | chhHHHHHHHHHHHHHHHHHHHHHHHHHhcC-CCCHHHHHHHHHHHhchHHHHHhcCCchHHH--HHHHHHHHHHHHHHH |
|  |
|  |
|  | Q ss\_pred |  | HHHHccCCCCcchHHHHHHHHHH--HHHHHHhHhHHHHHHHHHHHHHHHHHcccchhhhcchHhhhHHHHHHHHHHHHHH |
|  | Q Q8N394 | 156 | IKHCSTRGYSARTWGWFLGSGLC--AGCSMLWKEQGVTVLAVSAVYDVFVFHRLKIKQILPTIYKRKNLSLFLSISLLIF   233 (475) |
|  | Q Consensus | 156 | l~~~~~~~~~~~~~~~~~l~~l~--~~la~ltk~~~~~~~~~~~~~~l~~~~~~~~~~~~~~~~~~~~~~~~~~~~~~~~   233 (475) |
|  |  |  | .+..+++ +.++..+++++ +|+|..+|++++++.+.+.+..+...++++... +.........++..+ |
|  | T Consensus | 304 | ~~~~~~~-----~~~~~~l~gl~~~~gla~~~K~~~~~~~~~~~~~~l~~~~~~~~~~-------~~~~~~~~~~~~~~~   371 (670) |
|  | T PF07220.12 | 304 | FSKIKTD-----KNKLFAFSLICILIMSATQAKFSFFLSAFLIGLFSIFSLGSIRLFF-------YGLLISLFFFVLINF   371 (670) |
|  | T ss\_pred |  | HHhhccC-----CchHHHHHHHHHHHHHHHhhHHHHHHHHHHHHHHHHHHhcccchhH-------HHHHHHHHHHHHHHH |
|  |
|  |
|  | Q ss\_pred |  | HHHHHHHHHHHHhcCCCCCccCCCCCCcCCChHHHHHHHHHHHHHHhHHHhhCcccccccccccccccccccCCHHHHHH |
|  | Q Q8N394 | 234 | WGSSLLGARLYWMGNKPPSFSNSDNPAADSDSLLTRTLTFFYLPTKNLWLLLCPDTLSFDWSMDAVPLLKTVCDWRNLHT   313 (475) |
|  | Q Consensus | 234 | ~~~~~~~~~~~~~~~~~~~~~~~~~~~~~~~~~~~~~~~~~~~~~~~~~~~~~p~~~~~~~~~~~~~~~~~~~~~~~~~~   313 (475) |
|  |  |  | ....+........+....................+..................+........ |
|  | T Consensus | 372 | ~~~~w~~~~~~~~g~~~~~~~~~~~~~~~~~~~~~~~~~~~~~~~~~~~~~~~~~~~~~~~~------------------   433 (670) |
|  | T PF07220.12 | 372 | PAIFWKIKNYNSTFIDVLIHPLPGNTFPGVNEFEVSLRNYQDSALIFPLSLIFPNQFGVITT------------------   433 (670) |
|  | T ss\_pred |  | HHHHHHHHHHHccCCcccccCCCCCCCCCccchHHHHHhcccccccCcHHhhccccchhhHh------------------ |
|  |
|  |
|  | Q ss\_pred |  | HHHHHHHHHHHHHHhhCcccccccCCCcccCCccccCCcccccccccccccccccccchhhcCCcCCcccccCCCCCCch |
|  | Q Q8N394 | 314 | VAFYTGLLLLAYYGLKSPSVDRECNGKTVTNGKQNANGHSCLSDVEYQNSETKSSFASKVENGIKNDVSQRTQLPSTENI   393 (475) |
|  | Q Consensus | 314 | ~~~~~~~~~~~~~~~~~~~~~~~~~~~~~~~~~~~~~~~~~~~~~~~~~~~~~~~~~~~~~~~~~~~~~~~~~~~~~~~~   393 (475) |
|  |  |  | ....+.+......+++ ++. |
|  | T Consensus | 434 | -~~~~~~~~~~~~~~~~------------------------------------------------------------~~~   452 (670) |
|  | T PF07220.12 | 434 | -VIGLGLFLIIFVKPIV------------------------------------------------------------TQK   452 (670) |
|  | T ss\_pred |  | -HHHHHHHHHHHhcchh------------------------------------------------------------chH |
|  |
|  |
|  | Q ss\_pred |  | HHhhhHHHHHHHhhhcCccccCChhhHHHHhHHHHHHHHHHHHHHHHHHHHHhchHHHHHHHHHHHHHHHHHHHHHHHHh |
|  | Q Q8N394 | 394 | VVLSLSLLIIPFVPATNLFFYVGFVIAERVLYIPSMGFCLLITVGARALYVKVQKRFLKSLIFYATATLIVFYGLKTAIR   473 (475) |
|  | Q Consensus | 394 | ~~~~l~~~~~~~~~~~~~~~~~~~~~~~Ry~~~~~~~~~ll~~~~l~~~~~~~~~~~~~~~~~~~~~~~~~~~~~~~~~~   473 (475) |
|  |  |  | ......+.+......... .....||.++.+|++++ ++.+.....+..+++.++.....+++.++.......... |
|  | T Consensus | 453 | ~~~~~~~~~~~~~~~~~~-----~~~~~Ry~lp~~p~l~l-~~~~~~~~~~~~~~~~~~~~~~~~~~~~~~~~~~~~~~~   526 (670) |
|  | T PF07220.12 | 453 | AFLLSVMIILFVILGSLM-----GQKASRFFLEPFVWMLI-SLIGLNSFGKWNIRFVKEAVSTGILLQACATLVIISVGI   526 (670) |
|  | T ss\_pred |  | HHHHHHHHHHHHHHHHHh-----ccchHHhhHHHHHHHHH-HHHHHHhhccccHHHHHHHHHHHHHHHHHHHHHHHHHHH |
|  |
|  |
|  | Q ss\_pred |  | cC |
|  | Q Q8N394 | 474 | NG   475 (475) |
|  | Q Consensus | 474 | ~~   475 (475) |
|  |  |  | .. |
|  | T Consensus | 527 | ~~   528 (670) |
|  | T PF07220.12 | 527 | YQ   528 (670) |
|  | T ss\_pred |  | hh |
|  |
| --- | | | |
|  | Template alignmentCDD | | |
| 5. | PF10131.10 ; PTPS\_related ; 6-pyruvoyl-tetrahydropterin synthase related domain; membrane protein | | |
|  | Probability: 99.65%, E-value: 9.7e-14, Score: 139.99, Aligned cols: 304, Identities: 11%, Similarity: -0.039, | | |
|  |
|  | Q ss\_pred |  | cccCChHHHHHHHHHHHHh-CCChHHHHHHHHHHHHHHHHHHHHHHHHHhCChHHHHHHHHHHHHCHHhHHHHHhhhchH |
|  | Q Q8N394 | 62 | HKSYRPLCTLSFRLNHAIG-GLNPWSYHLVNVLLHAAVTGLFTSFSKILLGDGYWTFMAGLMFASHPIHTEAVAGIVGRA   140 (475) |
|  | Q Consensus | 62 | ~~~~~Pl~~~l~~~~~~lf-G~~~~~~rl~~~l~~~l~~~lly~l~r~l~~~~~~al~aall~a~~P~~~~~~~~~~~~~   140 (475) |
|  |  |  | ..+|||++.++.++...++ |.+..+.|+.++++++++++.+|.++|++.+ +..|++++++++++|.+.........++ |
|  | T Consensus | 1 | f~~~pPl~~~l~~~~~~l~~g~~~~~~~l~~~l~~~l~~~~~y~l~r~~~~-~~~a~~a~~l~~~~p~~~~~~~~~~~~~   79 (616) |
|  | T PF10131.10 | 1 | FRYWGPLSYYIMAGLMFLTSGDLLLAYRLIAFVIFVVGGLPWILWGIHENR-RVLGTFFGVLWFFMPEHIRIYFTAGNLP   79 (616) |
|  | T ss\_pred |  | CCCCchHHHHHHHHHHHHhcCCHHHHHHHHHHHHHHHHHHHHHHHHHHcCc-HHHHHHHHHHHHHCchHHHHHHhcCCHH |
|  |
|  |
|  | Q ss\_pred |  | HHHHHHHHHHHHHHHHHHHccCCCCcchHHHHHHHHHHHHHHHHhHhHHHHHHHHHHHHHHHHHcccchhhhcchHhhhH |
|  | Q Q8N394 | 141 | DVGASLFFLLSLLCYIKHCSTRGYSARTWGWFLGSGLCAGCSMLWKEQGVTVLAVSAVYDVFVFHRLKIKQILPTIYKRK   220 (475) |
|  | Q Consensus | 141 | ~~~~~~f~ll~l~~~l~~~~~~~~~~~~~~~~~l~~l~~~la~ltk~~~~~~~~~~~~~~l~~~~~~~~~~~~~~~~~~~   220 (475) |
|  |  |  | |.+..++.+++++++.+..+++ ++++.++++++.+++.++|+....+.++..+..++....++.+. ++ |
|  | T Consensus | 80 | ~~~~~~~~~l~l~~~~~~~~~~-----~~~~~~~~~l~~~l~~~~~~~~~~~~~~~~~~~~~~~~~~~~~~-------~~   147 (616) |
|  | T PF10131.10 | 80 | QMVTTMLVPYVIWFLWLYVRKK-----NNRAAVGLFVCMTLMSFTHLMVTAIMGVSAFLYLLIDQIWNKDT-------RR   147 (616) |
|  | T ss\_pred |  | HHHHHHHHHHHHHHHHHHHhcC-----CHHHHHHHHHHHHHHHHhcHHHHHHHHHHHHHHHHHHHHhCCCh-------HH |
|  |
|  |
|  | Q ss\_pred |  | HHHHHHHHHHHHHHHHHHHHHHHHHhcCCCCCccCCCCCCcCCChHHHHHHHHHHHHHHhHHHhhCcccccccccccccc |
|  | Q Q8N394 | 221 | NLSLFLSISLLIFWGSSLLGARLYWMGNKPPSFSNSDNPAADSDSLLTRTLTFFYLPTKNLWLLLCPDTLSFDWSMDAVP   300 (475) |
|  | Q Consensus | 221 | ~~~~~~~~~~~~~~~~~~~~~~~~~~~~~~~~~~~~~~~~~~~~~~~~~~~~~~~~~~~~~~~~~~p~~~~~~~~~~~~~   300 (475) |
|  |  |  | ........++.+++...++................. ................... |
|  | T Consensus | 148 | ~~~~~~~~~~~~~~~~~~~~~~~~~~~~~~~~~~~~-~~~~~~~~~~~~~~~~~~~------------------------   202 (616) |
|  | T PF10131.10 | 148 | KIFALIYMICGILTAGIWVIPSLKGGLVTSESGDGS-VMSTLIYPLTTSLNPFKRL------------------------   202 (616) |
|  | T ss\_pred |  | HHHHHHHHHHHHHHHHHHHHHHHccCCccCCCCccc-hhhhheechhhccCcchhc------------------------ |
|  |
|  |
|  | Q ss\_pred |  | cccccCCHHHHHHHHHHHHHHHHHHHHhhCcccccccCCCcccCCccccCCcccccccccccccccccccchhhcCCcCC |
|  | Q Q8N394 | 301 | LLKTVCDWRNLHTVAFYTGLLLLAYYGLKSPSVDRECNGKTVTNGKQNANGHSCLSDVEYQNSETKSSFASKVENGIKND   380 (475) |
|  | Q Consensus | 301 | ~~~~~~~~~~~~~~~~~~~~~~~~~~~~~~~~~~~~~~~~~~~~~~~~~~~~~~~~~~~~~~~~~~~~~~~~~~~~~~~~   380 (475) |
|  |  |  | .............++..+......+++ |
|  | T Consensus | 203 | ---~~~~~~~~~~~~~~~l~~~~~~~~~~~--------------------------------------------------   229 (616) |
|  | T PF10131.10 | 203 | ---SAGNDSFYFGLAAVLIAIAGILLARGG--------------------------------------------------   229 (616) |
|  | T ss\_pred |  | ---cCCCCcchHHHHHHHHHHHHHHHHcCC-------------------------------------------------- |
|  |
|  |
|  | Q ss\_pred |  | cccccCCCCCCchHHhhhHHHHHHHhhhcCccccCChhhHH---HHhHHHHHHHHHHHHHHHHHHHHHhchHHHHHHHHH |
|  | Q Q8N394 | 381 | VSQRTQLPSTENIVVLSLSLLIIPFVPATNLFFYVGFVIAE---RVLYIPSMGFCLLITVGARALYVKVQKRFLKSLIFY   457 (475) |
|  | Q Consensus | 381 | ~~~~~~~~~~~~~~~~~l~~~~~~~~~~~~~~~~~~~~~~~---Ry~~~~~~~~~ll~~~~l~~~~~~~~~~~~~~~~~~   457 (475) |
|  |  |  | +.......++.+++.................. ||..+. +++++++.++.+..++ .... |
|  | T Consensus | 230 | ---------~~~~~~~~~~~~~~~~~~~~~~~~~~~~~~~~~~~r~~~~~--~~~~~~~~~~~~~~~~--------~~~~   290 (616) |
|  | T PF10131.10 | 230 | ---------KKAGFVFLLIMLACTTPAAYRILVKLPFSQLFWMTRFAPMV--YGFFFSACLEWVRLKK--------KYCV   290 (616) |
|  | T ss\_pred |  | ---------ChHHHHHHHHHHHhcchHHHHHHhhCCHHHhhhHhHHHHHH--HHHHHHHHHHhhhhHH--------HHHH |
|  |
|  |
|  | Q ss\_pred |  | HHHHHHHHHHHHHHHhcC |
|  | Q Q8N394 | 458 | ATATLIVFYGLKTAIRNG   475 (475) |
|  | Q Consensus | 458 | ~~~~~~~~~~~~~~~~~~   475 (475) |
|  |  |  | +++++++........... |
|  | T Consensus | 291 | ~~~~~~~~~~~~~~~~~~   308 (616) |
|  | T PF10131.10 | 291 | LLAALLCVDSISCMNLDF   308 (616) |
|  | T ss\_pred |  | HHHHHHHHHHhhhccccc |
|  |
| --- | | | |
|  | Template alignmentCDD | | |
| 6. | PF02366.19 ; PMT ; Dolichyl-phosphate-mannose-protein mannosyltransferase | | |
|  | Probability: 99.61%, E-value: 1.3e-13, Score: 121.28, Aligned cols: 217, Identities: 16%, Similarity: 0.11, | | |
|  |
|  | Q ss\_pred |  | HHHHHHHHHHHHHHhhcCCCCcccchhHHHhchhhCCCCchhHhhhcccccccccCCCCcccCChHHHHHHHHHHHHhC- |
|  | Q Q8N394 | 3 | AELVSSALGLALYLNTLSADFCYDDSRAIKTNQDLLPETPWTHIFYNDFWGTLLTHSGSHKSYRPLCTLSFRLNHAIGG-   81 (475) |
|  | Q Consensus | 3 | ~~lll~~~~~~~~~~~~~~~~~~De~~~~~~~~~~~~~~~~~~~~~~~~~~~~~~~~~~~~~~~Pl~~~l~~~~~~lfG-   81 (475) |
|  |  |  | ++++++......+..........||..+...++++.+++... ..++|............+| |
|  | T Consensus | 1 | ~~~~~~~~~~~~~~~~~~~~~~~D~~~~~~~a~~~~~~~~~~------------------~~~~~~~~~~~~~~~~~~~~   62 (247) |
|  | T PF02366.19 | 1 | IFLTVVAFCVRAQRLMNPAKVVFEELRYYNYAVDYVNNKLLM------------------DVYPPLGKLLFSLVAALTGN   62 (247) |
|  | T ss\_pred |  | ChHHHHHHHHHHHHHhCCcccchHHHHHHHHHHHHHcCcccc------------------CCCCcHHHHHHHHHHHHcCC |
|  |
|  |
|  | Q ss\_pred |  | -----------------CChHHHHHHHHHHHHHHHHHHHHHHHHHhCChHHHHHHHHHHHHCHHhHHHHHhhhchHHHHH |
|  | Q Q8N394 | 82 | -----------------LNPWSYHLVNVLLHAAVTGLFTSFSKILLGDGYWTFMAGLMFASHPIHTEAVAGIVGRADVGA   144 (475) |
|  | Q Consensus | 82 | -----------------~~~~~~rl~~~l~~~l~~~lly~l~r~l~~~~~~al~aall~a~~P~~~~~~~~~~~~~~~~~   144 (475) |
|  |  |  | .+....|+.+.+++.++++.+|.+.|+..+++..+++++++++++|.....+..... |.+. |
|  | T Consensus | 63 | ~~~~~~~~~~~~~~~~~~~~~~~~~~~~~~~~~~~~~~~~~~~~~~~~~~~a~~~~~~~~~~p~~~~~~~~~~~--d~~~   140 (247) |
|  | T PF02366.19 | 63 | KYELNTLDEPGQQYPFTDVAYSMRLFTCLLGSLLVPLMYGTVYFPTKSKTAASLAALFVIFDNGLITMSRYIMI--EIPA   140 (247) |
|  | T ss\_pred |  | CcccccccCCCCCCCcchHHHHHHHHHHHHHHHHHHHHHHHHhcccCCHHHHHHHHHHHHhchhHHHhHhhccc--hHHH |
|  |
|  |
|  | Q ss\_pred |  | HHHHHHHHHHHHHH-------HccCCCCcchHHHHHHHHHHHHHHHHhHhHHHHHHHHHHHHHHHHHcccchhhhcchHh |
|  | Q Q8N394 | 145 | SLFFLLSLLCYIKH-------CSTRGYSARTWGWFLGSGLCAGCSMLWKEQGVTVLAVSAVYDVFVFHRLKIKQILPTIY   217 (475) |
|  | Q Consensus | 145 | ~~f~ll~l~~~l~~-------~~~~~~~~~~~~~~~l~~l~~~la~ltk~~~~~~~~~~~~~~l~~~~~~~~~~~~~~~~   217 (475) |
|  |  |  | .++.+++++++.+. .+++ ++++..+++++.+++.++|+......+...+.......+++.++..+ |
|  | T Consensus | 141 | ~~~~~~~~~~~~~~~~~~~~~~~~~-----~~~~~~~~~~~~~l~~~~k~~~~~~~~~~~~~~~~~~~~~~~~~~~~---   212 (247) |
|  | T PF02366.19 | 141 | LYFMSLTAFYWSVYEAQQKRPFSLR-----WHTSLLSTGVALGLALSTKLSAMFTFGWLLILAAFHLWNLLGDLSVP---   212 (247) |
|  | T ss\_pred |  | HHHHHHHHHHHHHHHHhcCCCCCcH-----HHHHHHHHHHHHHHHHHhhHHHHHHHHHHHHHHHHHHHHhcCCCCCC--- |
|  |
|  |
|  | Q ss\_pred |  | hhHHHHHHHHHHHHHHHHHHHHHHHHHHhcC |
|  | Q Q8N394 | 218 | KRKNLSLFLSISLLIFWGSSLLGARLYWMGN   248 (475) |
|  | Q Consensus | 218 | ~~~~~~~~~~~~~~~~~~~~~~~~~~~~~~~   248 (475) |
|  |  |  | .............+.....+........++ |
|  | T Consensus | 213 | -~~~~~~~~~~~~~~~~~~p~~~~~~~~~~~   242 (247) |
|  | T PF02366.19 | 213 | -MYRIVKHLFSYIFYLIGVPITVYLAVFAVH   242 (247) |
|  | T ss\_pred |  | -HHHHHHHHHHHHHHHHHHHHHHHHHHHHHH |
|  |
| --- | | | |
|  | Template alignmentCDD | | |
| 7. | PF10034.10 ; Dpy19 ; Q-cell neuroblast polarisation | | |
|  | Probability: 99.59%, E-value: 1.8e-12, Score: 131.71, Aligned cols: 349, Identities: 9%, Similarity: -0.051, | | |
|  |
|  | Q ss\_pred |  | CCCCcccchhHHHhchhhCCCCchhHhhhcccccccccCCCCccc---CChHHHHHHHHHHHHhC--------------- |
|  | Q Q8N394 | 20 | SADFCYDDSRAIKTNQDLLPETPWTHIFYNDFWGTLLTHSGSHKS---YRPLCTLSFRLNHAIGG---------------   81 (475) |
|  | Q Consensus | 20 | ~~~~~~De~~~~~~~~~~~~~~~~~~~~~~~~~~~~~~~~~~~~~---~~Pl~~~l~~~~~~lfG---------------   81 (475) |
|  |  |  | +..+..||..|...++++.+++++.+.+....++.....+..... .+|...++.+..++++| |
|  | T Consensus | 30 | ~~~~~~d~~~y~~~~~~i~~~~~~~~~~~~~~~d~~~~~p~g~~~~~~~~~~~~~~~a~~~~~~~~~~~~~~~~~~~~~~   109 (651) |
|  | T PF10034.10 | 30 | ELSFRTEMGLYYSYYKTMVEAPTFLDGLHAVMNCNVTEYPDTVNTLKRFNLYPEVILAGKFRIFEWLASKFEYQTKTCYT   109 (651) |
|  | T ss\_pred |  | hhccCcchhhHHhHHhhhccCCCHHHHHHHHHcCCCCcCCCCchHHhhcchHHHHHHHHHHHHHHHHHhhcCcCccchhe |
|  |
|  |
|  | Q ss\_pred |  | -----------------CChHHHHHHHHHHHHHHHHHHHHHHHHHhCChHHHHHHHHHHHHCHHhHHHHHhhhchHHHHH |
|  | Q Q8N394 | 82 | -----------------LNPWSYHLVNVLLHAAVTGLFTSFSKILLGDGYWTFMAGLMFASHPIHTEAVAGIVGRADVGA   144 (475) |
|  | Q Consensus | 82 | -----------------~~~~~~rl~~~l~~~l~~~lly~l~r~l~~~~~~al~aall~a~~P~~~~~~~~~~~~~~~~~   144 (475) |
|  |  |  | ...........++++++++.+|.++|++++++..|++++++++++|.+...+.......|.+. |
|  | T Consensus | 110 | ~~~~~~~~~~~~~~~~~~p~~~~~~~~~i~~~l~v~~~y~l~~~l~~~~~~al~aall~a~~p~~~~~~~~g~~~~~~~~   189 (651) |
|  | T PF10034.10 | 110 | VNRGYGLPPVQSCEGLGELSFFYVYSIFFLTGLMMACFFILCFYLSGSILGGVLGTLCYFFNHGEATRVMWTPPLRESFS   189 (651) |
|  | T ss\_pred |  | eecCCCCCCcccCCCcCchHHHHHHHHHHHHHHHHHHHHHHHHHHcCChHHHHHHHHHHHhChhhhhhHhhCCCccccCH |
|  |
|  |
|  | Q ss\_pred |  | HHHHHHHHHHHHHHHccCCCCcchH--HHHHHHHHHHHHHHHhHhHHHHHHHHHHHHHHHHHcccchhhhcchHhhhHHH |
|  | Q Q8N394 | 145 | SLFFLLSLLCYIKHCSTRGYSARTW--GWFLGSGLCAGCSMLWKEQGVTVLAVSAVYDVFVFHRLKIKQILPTIYKRKNL   222 (475) |
|  | Q Consensus | 145 | ~~f~ll~l~~~l~~~~~~~~~~~~~--~~~~l~~l~~~la~ltk~~~~~~~~~~~~~~l~~~~~~~~~~~~~~~~~~~~~   222 (475) |
|  |  |  | .++.+++++++.+..+++ +. ++.++++++.+++..+|...........+..+.....++.+ ++.. |
|  | T Consensus | 190 | ~~f~~l~l~~~~~~~~~~-----~~~~~~~~l~gl~~~l~~~~~~~~~~~~~~~~~~~~~~~~~~~~~--------~~~~   256 (651) |
|  | T PF10034.10 | 190 | YPYLVAQLLVVTFTLRSV-----KVTWRHITLVSMTTALFMIPWQFAQFALLTQTCALFVVYIMHFIT--------ADKF   256 (651) |
|  | T ss\_pred |  | HHHHHHHHHHHHHHHhCC-----CCCHHHHHHHHHHHHHHHHHhhhHHHHHHHHHHHHHHHHHhhcCC--------HHHH |
|  |
|  |
|  | Q ss\_pred |  | HHHHHHHHHHHHHHHHHHHHHHH--------------------------------------------------------- |
|  | Q Q8N394 | 223 | SLFLSISLLIFWGSSLLGARLYW---------------------------------------------------------   245 (475) |
|  | Q Consensus | 223 | ~~~~~~~~~~~~~~~~~~~~~~~---------------------------------------------------------   245 (475) |
|  |  |  | ................+...... |
|  | T Consensus | 257 | ~~~~~~~~~~~~~~~~~~~~~~~~~~~~~~~~~~~~~~~~~~~~~~~~~~~~~~~~~~~~~~~~~~~~~~~~~~~~~~~~   336 (651) |
|  | T PF10034.10 | 257 | CKILYGLLVAHLLNFAVQFGNSMLLSSFFMSAVISALVVAKAESQIHKLPYQLLIWATQGLGFAAGTLGIKVAVAKVLSI   336 (651) |
|  | T ss\_pred |  | HHHHHHHHHHHHHHHHHHccchHHHHHHHHHHHHHHHHHHHHHhhhccCCHHHHHHHHHHHHHHHHHHHHHHHHHHhcCc |
|  |
|  |
|  | Q ss\_pred |  | ------hcCCCCCccCCCCCCcCCChHHHHHHHHHHHHHHhHHHhhCcccccccccccccccccccCCHHHHHHHHHHHH |
|  | Q Q8N394 | 246 | ------MGNKPPSFSNSDNPAADSDSLLTRTLTFFYLPTKNLWLLLCPDTLSFDWSMDAVPLLKTVCDWRNLHTVAFYTG   319 (475) |
|  | Q Consensus | 246 | ------~~~~~~~~~~~~~~~~~~~~~~~~~~~~~~~~~~~~~~~~~p~~~~~~~~~~~~~~~~~~~~~~~~~~~~~~~~   319 (475) |
|  |  |  | ............................... .........+... |
|  | T Consensus | 337 | ~~~~~~~~~~~~~~~~~~~~~~~~~~~~~~~~~~~~~------------------------------~~~~~~~~~~~~~   386 (651) |
|  | T PF10034.10 | 337 | ADDSKFTSYRDFHTLLYTCAPEFDFLDQEAPVKLTKT------------------------------LLLPSAIVAASAV   386 (651) |
|  | T ss\_pred |  | ccccccCCCCCHHHHHHhcchhcCCCChHHHHHHHHh------------------------------cHHHHHHHHHHHH |
|  |
|  |
|  | Q ss\_pred |  | HHHHHHHHhhCcccccccCCCcccCCccccCCcccccccccccccccccccchhhcCCcCCcccccCCCCCCch------ |
|  | Q Q8N394 | 320 | LLLLAYYGLKSPSVDRECNGKTVTNGKQNANGHSCLSDVEYQNSETKSSFASKVENGIKNDVSQRTQLPSTENI------   393 (475) |
|  | Q Consensus | 320 | ~~~~~~~~~~~~~~~~~~~~~~~~~~~~~~~~~~~~~~~~~~~~~~~~~~~~~~~~~~~~~~~~~~~~~~~~~~------   393 (475) |
|  |  |  | ++.......++.+++++ .+. |
|  | T Consensus | 387 | ~~~~~~~~~~~~~~~~~------------------------------------------------------~~~~~~~~~   412 (651) |
|  | T PF10034.10 | 387 | IAKVGASEWEYWVRGKK------------------------------------------------------SQVKSDSAD   412 (651) |
|  | T ss\_pred |  | HHHHHHHHHHHHHhccc------------------------------------------------------ccCCCCCcc |
|  |
|  |
|  | Q ss\_pred |  | ------------------HHhhhHHHHHHHhhhcCccccCChhhHHHHhHHHHHHHHHHHHHHHHH-HHHHhchHHHHHH |
|  | Q Q8N394 | 394 | ------------------VVLSLSLLIIPFVPATNLFFYVGFVIAERVLYIPSMGFCLLITVGARA-LYVKVQKRFLKSL   454 (475) |
|  | Q Consensus | 394 | ------------------~~~~l~~~~~~~~~~~~~~~~~~~~~~~Ry~~~~~~~~~ll~~~~l~~-~~~~~~~~~~~~~   454 (475) |
|  |  |  | ..+.+++.+..... .....||..+..|+++++++.++.+ +.++.+++..+.. |
|  | T Consensus | 413 | ~~~~~~~~~~~~~~~~~~~~~~~~~~~~~~~~---------~~~~~R~~~~~~p~l~il~a~~~~~~~~~~~~~~~~~~~   483 (651) |
|  | T PF10034.10 | 413 | EHDEGAQGQEANPRPHAEYVYHVLQAMAFVLM---------AVIIMRLKLFGTPALCVLASLVASRQFFSFLGDRRRHQA   483 (651) |
|  | T ss\_pred |  | ccccccccccCCCCCchHHHHHHHHHHHHHHH---------HHHHHHHHHHHHHHHHHHHHHHhcHHHHHhhhHHHHHHH |
|  |
|  |
|  | Q ss\_pred |  | HHHHHHHHHHHHHHHHHHhc |
|  | Q Q8N394 | 455 | IFYATATLIVFYGLKTAIRN   474 (475) |
|  | Q Consensus | 455 | ~~~~~~~~~~~~~~~~~~~~   474 (475) |
|  |  |  | +..+++++++.......... |
|  | T Consensus | 484 | ~~~~~~~~~~~~~~~~~~~~   503 (651) |
|  | T PF10034.10 | 484 | IVIALIAVMSVQGFSNLKTQ   503 (651) |
|  | T ss\_pred |  | HHHHHHHHHHhcchhhHHHH |
|  |
| --- | | | |
|  | Template alignmentCDD | | |
| 8. | PF04188.14 ; Mannosyl\_trans2 ; Mannosyltransferase (PIG-V) | | |
|  | Probability: 99.59%, E-value: 1.9e-12, Score: 124.6, Aligned cols: 335, Identities: 12%, Similarity: -0.078, | | |
|  |
|  | Q ss\_pred |  | ccchhHHH--hchhh-CCCCchhHhhhcccccccccCCCCcccCChHHHHHHHHHHHHhCCC-----------hHHHHHH |
|  | Q Q8N394 | 25 | YDDSRAIK--TNQDL-LPETPWTHIFYNDFWGTLLTHSGSHKSYRPLCTLSFRLNHAIGGLN-----------PWSYHLV   90 (475) |
|  | Q Consensus | 25 | ~De~~~~~--~~~~~-~~~~~~~~~~~~~~~~~~~~~~~~~~~~~Pl~~~l~~~~~~lfG~~-----------~~~~rl~   90 (475) |
|  |  |  | +||.+|.. .+++- ...+....+ +|++.++......++|.+ ..+.|++ |
|  | T Consensus | 60 | wD~~~y~~~~ia~~g~y~~~~~~~f-------------------~Pl~p~l~~~~~~l~~~~~~~~~~~~~~~~~~~~~~   120 (432) |
|  | T PF04188.14 | 60 | WDSVFFIKNITSKNGKPQFEHEYAF-------------------SQLWTFFVRLFIKSNNDSIYHALRVGVAIENVLFYL   120 (432) |
|  | T ss\_pred |  | ccHHHHhhhhHHHcCCCcccccccc-------------------hHHHHHHHHHHHHhcccchHHHHHHHHHHHHHHHHH |
|  |
|  |
|  | Q ss\_pred |  | H-HHHHHHHHHHHHHHHHHHhCChHHHHHHHHHHHHCHHhHHHHHhhhchHHHHHHHHHHHHHHHHHHHHccCCCCcchH |
|  | Q Q8N394 | 91 | N-VLLHAAVTGLFTSFSKILLGDGYWTFMAGLMFASHPIHTEAVAGIVGRADVGASLFFLLSLLCYIKHCSTRGYSARTW   169 (475) |
|  | Q Consensus | 91 | ~-~l~~~l~~~lly~l~r~l~~~~~~al~aall~a~~P~~~~~~~~~~~~~~~~~~~f~ll~l~~~l~~~~~~~~~~~~~   169 (475) |
|  |  |  | + .+++++++..+|.+.|+..+++..|..++++++++|..... ....+ |.+..++.+++++++.+..++++ ++ |
|  | T Consensus | 121 | s~~~~~~~~~~~ly~l~~~~~~~~~~a~~a~~l~~~~P~~~~~-~~~~~--E~l~~~l~~~~~~~~~~~~~~~~----~~   193 (432) |
|  | T PF04188.14 | 121 | SGIVLYFLTKKIFSQNIRQSQFARTIAKKTSLLFFLTSAAGFL-TSIYS--EPLSFFFAFVGIWSRECSISVPV----LG   193 (432) |
|  | T ss\_pred |  | HHHHHHHHHHHHHHhhhHhhhccHHHHHHHHHHHhhCCchHHh-hccCc--HHHHHHHHHHHHHHHHhccCCCC----CC |
|  |
|  |
|  | Q ss\_pred |  | HHHHHHHHHHHHHHH-hHhHHHHHHHHHHHHHHH--------HHcccchhhhcchHhhhHHHHHHHHHHHHHHHHHHHHH |
|  | Q Q8N394 | 170 | GWFLGSGLCAGCSML-WKEQGVTVLAVSAVYDVF--------VFHRLKIKQILPTIYKRKNLSLFLSISLLIFWGSSLLG   240 (475) |
|  | Q Consensus | 170 | ~~~~l~~l~~~la~l-tk~~~~~~~~~~~~~~l~--------~~~~~~~~~~~~~~~~~~~~~~~~~~~~~~~~~~~~~~   240 (475) |
|  |  |  | ++.+.++++.+++.+ +|..++...+......+. .+++.+... +.........++.......+.. |
|  | T Consensus | 194 | ~~~~~~~~~~~la~~~~R~~g~~~~~~~~~~~l~~~~~~~~~~~~~~~~~~-------~~~~~~~~~~~~~~~p~~~~~~   266 (432) |
|  | T PF04188.14 | 194 | QFDISWRYWFPYSFISMACFTLASLNRSNCVLLGIYFIFDLIELTKNRKFV-------KAICFPLLSGSLMFSALLYQQY   266 (432) |
|  | T ss\_pred |  | chhHHHHHHHHHHHHHHHhHHHHHHHHHHHHHHHHHHHHHHHHhhhhHHHH-------HHHHHHHHHHHHHHHHHHHHHH |
|  |
|  |
|  | Q ss\_pred |  | HH-HHHhcCCCCCccCCCCCCcCCChHHHHHHHHHHHHHHhHHHhhCcccccccccccccccccccCCHHHHHHHHHHHH |
|  | Q Q8N394 | 241 | AR-LYWMGNKPPSFSNSDNPAADSDSLLTRTLTFFYLPTKNLWLLLCPDTLSFDWSMDAVPLLKTVCDWRNLHTVAFYTG   319 (475) |
|  | Q Consensus | 241 | ~~-~~~~~~~~~~~~~~~~~~~~~~~~~~~~~~~~~~~~~~~~~~~~p~~~~~~~~~~~~~~~~~~~~~~~~~~~~~~~~   319 (475) |
|  |  |  | .. ...+......+.....+.. .....+....+.....+.......+.... .+........++. |
|  | T Consensus | 267 | ~~~~~~f~~~~~~w~~~~~~~~--------~~~~~~~~~~~~q~~yw~~g~~~~~~~~~--------~~~~ll~~p~~~l   330 (432) |
|  | T PF04188.14 | 267 | YLPYKTFCPQRGEWCKSQLFSS--------IFITKTSLYSYIQSHYWGVGLLKYWTPNN--------IPNFLFAVPNIII   330 (432) |
|  | T ss\_pred |  | HHHHHHHCCCCChhHhCCCCCc--------hhhcccchHHHHHHHcCCCcchhcCCccc--------hhHHHhHHHHHHH |
|  |
|  |
|  | Q ss\_pred |  | HHHHHHHHhhCcccccccCCCcccCCccccCCcccccccccccccccccccchhhcCCcCCcccccCCCCCCchHHhhhH |
|  | Q Q8N394 | 320 | LLLLAYYGLKSPSVDRECNGKTVTNGKQNANGHSCLSDVEYQNSETKSSFASKVENGIKNDVSQRTQLPSTENIVVLSLS   399 (475) |
|  | Q Consensus | 320 | ~~~~~~~~~~~~~~~~~~~~~~~~~~~~~~~~~~~~~~~~~~~~~~~~~~~~~~~~~~~~~~~~~~~~~~~~~~~~~~l~   399 (475) |
|  |  |  | .+..+....|++++++. ......... |
|  | T Consensus | 331 | ~~~~~~~~~~~~~~~~~------------------------------------------------------~~~~~~~~~   356 (432) |
|  | T PF04188.14 | 331 | LIYSSIYFSKIYPSYNL------------------------------------------------------KALVWITRA   356 (432) |
|  | T ss\_pred |  | HHHHHHHHHhhCCcccc------------------------------------------------------HHHHHHHHH |
|  |
|  |
|  | Q ss\_pred |  | HHHHHHhhhcCccccCChhhHHHHhHHHHHHHHHHHHHHHHHHHHHhchHH------HHHHHHHHHHHHHHHHHHHH |
|  | Q Q8N394 | 400 | LLIIPFVPATNLFFYVGFVIAERVLYIPSMGFCLLITVGARALYVKVQKRF------LKSLIFYATATLIVFYGLKT   470 (475) |
|  | Q Consensus | 400 | ~~~~~~~~~~~~~~~~~~~~~~Ry~~~~~~~~~ll~~~~l~~~~~~~~~~~------~~~~~~~~~~~~~~~~~~~~   470 (475) |
|  |  |  | ..+....... .....||.. ..|++.+.++..+.+..++.++.. ..+......++...+..+.. |
|  | T Consensus | 357 | ~~~~~~~~~~-------~~~~~R~~~-~~P~l~~~~a~~~~~~~~~~~~~~~~~~~~~~~~~~~~~~~~~~~~~~l~   425 (432) |
|  | T PF04188.14 | 357 | LVVIVCFFAH-------VQILNRIAS-FLPLHLWYLADRLVKTSDPKKMENPKGDDKIVKFYIYWLAFWIPLQTILF   425 (432) |
|  | T ss\_pred |  | HHHHHHHHHH-------HHHHHHHHh-ccHHHHHHHHHHHhcCCCCCcCCCCCCCCHHHHHHHHHHHHHHHHHHHHH |
|  |
| --- | | | |
|  | Template alignmentCDD | | |
| 9. | PF12250.9 ; AftA\_N ; Arabinofuranosyltransferase N terminal | | |
|  | Probability: 99.48%, E-value: 3.7e-11, Score: 114.66, Aligned cols: 339, Identities: 10%, Similarity: -0.066, | | |
|  |
|  | Q ss\_pred |  | HHHHHHHHHHHHHHhhcCCCCcccchhHHHhchhhCCCCchhHhhhcccccccccCCCCcccCChHHHHHHHHHHHHhCC |
|  | Q Q8N394 | 3 | AELVSSALGLALYLNTLSADFCYDDSRAIKTNQDLLPETPWTHIFYNDFWGTLLTHSGSHKSYRPLCTLSFRLNHAIGGL   82 (475) |
|  | Q Consensus | 3 | ~~lll~~~~~~~~~~~~~~~~~~De~~~~~~~~~~~~~~~~~~~~~~~~~~~~~~~~~~~~~~~Pl~~~l~~~~~~lfG~   82 (475) |
|  |  |  | ......+............+.+.||.++....+.+.++..+.+....+ ..+.|||+++++.+....++|. |
|  | T Consensus | 78 | ~l~~~~l~~~L~~t~~~~~gl~~D~~~~~~~~~~~~~~~~~~d~~~~~----------~~~~YPPl~~~l~~~~~~l~G~   147 (432) |
|  | T PF12250.9 | 78 | ALVITTLGIPLSATRLYLDGINVDQGFRTQFLTWMGYTIHLSDMNYID----------MPSYYPGAWFWIGGRLANLLGL   147 (432) |
|  | T ss\_pred |  | HHHHHHHHHHHhcCcccCCCccccHHHHHHHHHHHHhccccccccCCC----------CcccCChHHHHHHHHHHHHhCC |
|  |
|  |
|  | Q ss\_pred |  | Ch-HHHHHHHHHHHHHHHHHHHHHHHHHhCChHHH-HHHHHHHHHCHHhHHHHHhhhchHHHHHHHHHHHHHHHHHHHHc |
|  | Q Q8N394 | 83 | NP-WSYHLVNVLLHAAVTGLFTSFSKILLGDGYWT-FMAGLMFASHPIHTEAVAGIVGRADVGASLFFLLSLLCYIKHCS   160 (475) |
|  | Q Consensus | 83 | ~~-~~~rl~~~l~~~l~~~lly~l~r~l~~~~~~a-l~aall~a~~P~~~~~~~~~~~~~~~~~~~f~ll~l~~~l~~~~   160 (475) |
|  |  |  | +. ...|..+++...++....|.+.|++.+++..+ .++++..++.|..... ...+.....+....++.+.|..+ |
|  | T Consensus | 148 | ~~~~a~r~~~~l~~~l~~~~~y~l~r~l~~~~~~al~ia~~~~~~~~~~~~~-----~~y~~l~~~~l~~~l~~~~r~l~   222 (432) |
|  | T PF12250.9 | 148 | AGWEVFQPWALISLATAGSILVPVWQRICGSLTVASGIALVTTSITIVMSAD-----EPYAAIITMGVPAATVMMRRALT   222 (432) |
|  | T ss\_pred |  | CHHHHhHHHHHHHHHHHHHHHHHHHHHHHCCHHHHHHHHHHHHHHHHhcCCC-----CcHHHHHHHHHHHHHHHHHHHhc |
|  |
|  |
|  | Q ss\_pred |  | cCCCCcchHHHHHHHHHHHHHHHHhHhHHHHHHHHHHH----HHHHHHcccchhhhcchHhhhHHHHHHHHHHHHHHHHH |
|  | Q Q8N394 | 161 | TRGYSARTWGWFLGSGLCAGCSMLWKEQGVTVLAVSAV----YDVFVFHRLKIKQILPTIYKRKNLSLFLSISLLIFWGS   236 (475) |
|  | Q Consensus | 161 | ~~~~~~~~~~~~~l~~l~~~la~ltk~~~~~~~~~~~~----~~l~~~~~~~~~~~~~~~~~~~~~~~~~~~~~~~~~~~   236 (475) |
|  |  |  | + +++..+..+++.+++.++|.....+...... ......+++++.. ++................ |
|  | T Consensus | 223 | ~------~~~~~~~~gl~lgl~~l~y~~~~~~~~~~~~~~~~l~~~~~~~~~~~~-------~~~~~~~~~a~~~a~~~~   289 (432) |
|  | T PF12250.9 | 223 | G------SLWPLIGLTLYIGVSAAMYTLFTAVVALSVCVMAALFAVVFDHSIKPL-------LRLLIIGTGSALIASTVW   289 (432) |
|  | T ss\_pred |  | C------CchHHHHHHHHHHHHHHHhHHHHHHHHHHHHHHHHHHHHHcCCCchHH-------HHHHHHHHHHHHHHHHHH |
|  |
|  |
|  | Q ss\_pred |  | HHHHHHHHHhcCCCCCccCCCCCCcCCChHHHHHHHHHHHHHHhHHHhhCcccccccccccccccccccCCHHHHHHHHH |
|  | Q Q8N394 | 237 | SLLGARLYWMGNKPPSFSNSDNPAADSDSLLTRTLTFFYLPTKNLWLLLCPDTLSFDWSMDAVPLLKTVCDWRNLHTVAF   316 (475) |
|  | Q Consensus | 237 | ~~~~~~~~~~~~~~~~~~~~~~~~~~~~~~~~~~~~~~~~~~~~~~~~~~p~~~~~~~~~~~~~~~~~~~~~~~~~~~~~   316 (475) |
|  |  |  | ..+.......+....+......+....+.......... .. |
|  | T Consensus | 290 | ~P~l~~~~~~~~~~~~~~~~~~~~~~~~~~~p~~~~~~----------------------------------------~~   329 (432) |
|  | T PF12250.9 | 290 | APYLTAILSGQPHSGATAMHYLPPTGAQVPMPMLQFNL----------------------------------------VG   329 (432) |
|  | T ss\_pred |  | HHHHHHHHhCCCCCCcchHhcCCccccCCCCccccccH----------------------------------------HH |
|  |
|  |
|  | Q ss\_pred |  | HHHHHHHHHHHhhCcccccccCCCcccCCccccCCcccccccccccccccccccchhhcCCcCCcccccCCCCCCchHHh |
|  | Q Q8N394 | 317 | YTGLLLLAYYGLKSPSVDRECNGKTVTNGKQNANGHSCLSDVEYQNSETKSSFASKVENGIKNDVSQRTQLPSTENIVVL   396 (475) |
|  | Q Consensus | 317 | ~~~~~~~~~~~~~~~~~~~~~~~~~~~~~~~~~~~~~~~~~~~~~~~~~~~~~~~~~~~~~~~~~~~~~~~~~~~~~~~~   396 (475) |
|  |  |  | .+.+++..+...+++++. .+.... |
|  | T Consensus | 330 | ~L~l~Glv~l~~~~r~~~--------------------------------------------------------~~~l~~   353 (432) |
|  | T PF12250.9 | 330 | LLCLLGLAYLIVRIADPD--------------------------------------------------------VRSMLI   353 (432) |
|  | T ss\_pred |  | HHHHHHHHHHHHHhcCHh--------------------------------------------------------HHHHHH |
|  |
|  |
|  | Q ss\_pred |  | hhHHHHHHHhhhcCccccCChhhHHHHhHHHHHHHHHHHHHHHHHH-----H----HHhchHHHHHHHHHHHHHHHHH |
|  | Q Q8N394 | 397 | SLSLLIIPFVPATNLFFYVGFVIAERVLYIPSMGFCLLITVGARAL-----Y----VKVQKRFLKSLIFYATATLIVF   465 (475) |
|  | Q Consensus | 397 | ~l~~~~~~~~~~~~~~~~~~~~~~~Ry~~~~~~~~~ll~~~~l~~~-----~----~~~~~~~~~~~~~~~~~~~~~~   465 (475) |
|  |  |  | .+.......+...............|+..+..+.+++.++.++.++ . ++.+....++.......+..+. |
|  | T Consensus | 354 | ~~~~~y~w~~~~~~~~~~~~~ll~~R~~~~l~~~l~~~~a~gi~~l~~~~~~~~~~~~~~~~~~~~~~~~~~~~~~~~   431 (432) |
|  | T PF12250.9 | 354 | AQIVFYGWIVTSMIVSLSGKTLLGFRLDAIITIQLATAGMLALAELRLVEIPRFYPAVTRPATATTVTRVMVAILAIA   431 (432) |
|  | T ss\_pred |  | HHHHHHHHHHHHHHHHHhCCCchhHhHHHHHHHHHHHHHHHHHHHHHHccCccccccccCcccchHHHHHHHHHHHHh |
|  |
| --- | | | |
|  | Template alignmentCDD | | |
| 10. | PF13231.7 ; PMT\_2 ; Dolichyl-phosphate-mannose-protein mannosyltransferase | | |
|  | Probability: 99.44%, E-value: 1.1e-11, Score: 101.2, Aligned cols: 156, Identities: 19%, Similarity: 0.28, | | |
|  |
|  | Q ss\_pred |  | cCChHHHHHHHHHHHHhCCChHHHHHHHHHHHHHHHHHHHHHHHHHhCChHHHHHHHHHHHHCHHhHHHHHhhhchHHHH |
|  | Q Q8N394 | 64 | SYRPLCTLSFRLNHAIGGLNPWSYHLVNVLLHAAVTGLFTSFSKILLGDGYWTFMAGLMFASHPIHTEAVAGIVGRADVG   143 (475) |
|  | Q Consensus | 64 | ~~~Pl~~~l~~~~~~lfG~~~~~~rl~~~l~~~l~~~lly~l~r~l~~~~~~al~aall~a~~P~~~~~~~~~~~~~~~~   143 (475) |
|  |  |  | ++||++.++.+..+.++|.+....|+.+.+.+.+++..+|.+.|+..+ ++.+..++.+++++|........... |.. |
|  | T Consensus | 1 | ~~~P~~~~~~~~~~~l~g~~~~~~~~~~~~~~~~~~~~~~~~~~~~~~-~~~~~~~~~~~~~~p~~~~~~~~~~~--~~~   77 (159) |
|  | T PF13231.7 | 1 | DKPPASLWVMELSTRIFGVNSWAMLVPQALLGVAAVALLYATVRRRFG-AVAGLLAGLILAVTPVAAMMFRFNNP--DAL   77 (159) |
|  | T ss\_pred |  | CCChHHHHHHHHHHHHHCCCHHHHHHHHHHHHHHHHHHHHHHHHHHHH-HHHHHHHHHHHHhcHHHHHhhhcCCH--HHH |
|  |
|  |
|  | Q ss\_pred |  | HHHHHHHHHHHHHHHHccCCCCcchHHHHHHHHHHHHHHHHhHhHHHHHHHHHHHHHHHHHcccchhhhcchHhhhHHHH |
|  | Q Q8N394 | 144 | ASLFFLLSLLCYIKHCSTRGYSARTWGWFLGSGLCAGCSMLWKEQGVTVLAVSAVYDVFVFHRLKIKQILPTIYKRKNLS   223 (475) |
|  | Q Consensus | 144 | ~~~f~ll~l~~~l~~~~~~~~~~~~~~~~~l~~l~~~la~ltk~~~~~~~~~~~~~~l~~~~~~~~~~~~~~~~~~~~~~   223 (475) |
|  |  |  | ..++..++++...+..+++ +.+....++++.+++..+|+......+...+.......+++.++ .+... |
|  | T Consensus | 78 | ~~~~~~~~~~~~~~~~~~~-----~~~~~~~~~~~~~l~~~~k~~~~~~~~~~~~~~~~~~~~~~~~~-------~~~~~   145 (159) |
|  | T PF13231.7 | 78 | LVLLMIAATWAMLRAVEDG-----RWRWLIVCGAFVGVGFLTKQLAVMLIVPGLALTYLVAGPPKIGV-------RIAQL   145 (159) |
|  | T ss\_pred |  | HHHHHHHHHHHHHHHHHcC-----ChHHHHHHHHHHHHHHHcccchHHhhHHHHHHHHHHhCChhHHH-------HHHHH |
|  |
|  |
|  | Q ss\_pred |  | HHHHHHHHHHH |
|  | Q Q8N394 | 224 | LFLSISLLIFW   234 (475) |
|  | Q Consensus | 224 | ~~~~~~~~~~~   234 (475) |
|  |  |  | ........... |
|  | T Consensus | 146 | ~~~~~~~~~~~   156 (159) |
|  | T PF13231.7 | 146 | FAAGTSMIVAA   156 (159) |
|  | T ss\_pred |  | HHHHHHHHHHH |
|  |
| --- | | | |
|  | Template alignmentCDD | | |
| 11. | PF09913.10 ; DUF2142 ; Predicted membrane protein (DUF2142) | | |
|  | Probability: 99.42%, E-value: 2.7e-11, Score: 115.33, Aligned cols: 333, Identities: 10%, Similarity: -0.116, | | |
|  |
|  | Q ss\_pred |  | HHHHhhcCCCCcccchhHHHhchhhCCCCchhHhhhccc----------------------------------------- |
|  | Q Q8N394 | 13 | ALYLNTLSADFCYDDSRAIKTNQDLLPETPWTHIFYNDF-----------------------------------------   51 (475) |
|  | Q Consensus | 13 | ~~~~~~~~~~~~~De~~~~~~~~~~~~~~~~~~~~~~~~-----------------------------------------   51 (475) |
|  |  |  | ..+....+.....||..|...+..+.+++......+... |
|  | T Consensus | 4 | ~~~~~~~P~~~~pDE~~H~~~a~~ia~g~~~~~~~~~~~~~~~~~~~~~~~~~~~~~~~~~~~~~~~~~~~~~~~~~~~~   83 (405) |
|  | T PF09913.10 | 4 | LAFAVVMPPFQVPDEDGHFIRAYLISRGEFVGRGAPRVPGTVVLSMMRYPEMGERFGRFKPRELVRDLIPHPGSVSPEVP   83 (405) |
|  | T ss\_pred |  | eehhhccCCCCCCChHHHHHHHHHHHcCceeeecCCCCCCcccchhccCcccccccCCCCchHHHhccCCCCCCCCCCcc |
|  |
|  |
|  | Q ss\_pred |  | -----ccccccCCCCccc-----CChHHHHHHHHHH---HHhCCC----hHHHHHHHHHHHHHHHHHHHHHHHHHhCChH |
|  | Q Q8N394 | 52 | -----WGTLLTHSGSHKS-----YRPLCTLSFRLNH---AIGGLN----PWSYHLVNVLLHAAVTGLFTSFSKILLGDGY   114 (475) |
|  | Q Consensus | 52 | -----~~~~~~~~~~~~~-----~~Pl~~~l~~~~~---~lfG~~----~~~~rl~~~l~~~l~~~lly~l~r~l~~~~~   114 (475) |
|  |  |  | -.....++..... +||+++++.+... +++|.+ ....|+.+++++++++.++++++++.. |
|  | T Consensus | 84 | ~~~~~~~~~~~~~~~~~~~~~~~~pPl~y~~~a~~~~l~~~~~~~~~~~~~~~Rl~s~l~~~~~~~~~~~~~~~~~----   159 (405) |
|  | T PF09913.10 | 84 | SLNLGNLDVRHRWLPWSIIGSSLYCPLVYMPASLGIATVRILSGSPLLMMYGARLFNVIVFAAALAISFRLAPRYR----   159 (405) |
|  | T ss\_pred |  | ccccccCCCcccccCccccCccccCHHhHHHHHHHHHHHHHccCCHHHHHHHHHHHHHHHHHHHHHHHHHHChhhH---- |
|  |
|  |
|  | Q ss\_pred |  | HHHHHHHHHHHCHHhHHHHHhhhchHHHHHHHHHHHHHHHHHHHHccCCCCcchHHHHHHHHHHHHHHHHhHhHHHHHHH |
|  | Q Q8N394 | 115 | WTFMAGLMFASHPIHTEAVAGIVGRADVGASLFFLLSLLCYIKHCSTRGYSARTWGWFLGSGLCAGCSMLWKEQGVTVLA   194 (475) |
|  | Q Consensus | 115 | ~al~aall~a~~P~~~~~~~~~~~~~~~~~~~f~ll~l~~~l~~~~~~~~~~~~~~~~~l~~l~~~la~ltk~~~~~~~~   194 (475) |
|  |  |  | .++++++++|.....++..+. |.+..++.+++++++++..+++. +++.+++.+++.+++.++| ..+++ |
|  | T Consensus | 160 | ---~~~~~~a~~P~~~~~~~~~~~--D~~~~~~~~~~~~~~~~~~~~~~----~~~~~~~~~~~~~l~~~~K---~~~~~   227 (405) |
|  | T PF09913.10 | 160 | ---ALFTAVALMPMTLQQAGGISA--DLVTIAFSFVGFSLVLHSREHFV----SRRLLILIVLVFVMWVLCK---SSIWA   227 (405) |
|  | T ss\_pred |  | ---HHHHHHHhchHHHHHHhcCCh--HHHHHHHHHHHHHHHHhcCCcCC----CHHHHHHHHHHHHHHHHHH---HHHHH |
|  |
|  |
|  | Q ss\_pred |  | HHHHHHHHHHcccchhhhcchHhhhHHHHHHHHHHHHHHHHHHHHHHHHHHhcCCCCCccCCCCCCcCCChHHHHHHHHH |
|  | Q Q8N394 | 195 | VSAVYDVFVFHRLKIKQILPTIYKRKNLSLFLSISLLIFWGSSLLGARLYWMGNKPPSFSNSDNPAADSDSLLTRTLTFF   274 (475) |
|  | Q Consensus | 195 | ~~~~~~l~~~~~~~~~~~~~~~~~~~~~~~~~~~~~~~~~~~~~~~~~~~~~~~~~~~~~~~~~~~~~~~~~~~~~~~~~   274 (475) |
|  |  |  | ......+..++++ ++.........+..++...++................................+.. |
|  | T Consensus | 228 | ~~l~~~~~~~~~~-----------~~~~~~~~~~~~~~~~~~~~~~~~~~~~~~~~~~~~~~~~~~~~~~~~~~~~~~~p   296 (405) |
|  | T PF09913.10 | 228 | LPLLLLIPVSAFK-----------NRLTWAAYLGVASVCMVGALLVWNNVTAPNLETFRAVRLTHGVDMPANIRLVGAHP   296 (405) |
|  | T ss\_pred |  | HHHHHHHhHHhhC-----------chHHHHHHHHHHHHHHHHHHHHHHhhcCCCccchhhccccCCCChHHHHHHHHhCH |
|  |
|  |
|  | Q ss\_pred |  | HHHHHhHHHhhCcccccccccccccccccccCCHHHHHHHHHHHHHHHHHHHHhhCcccccccCCCcccCCccccCCccc |
|  | Q Q8N394 | 275 | YLPTKNLWLLLCPDTLSFDWSMDAVPLLKTVCDWRNLHTVAFYTGLLLLAYYGLKSPSVDRECNGKTVTNGKQNANGHSC   354 (475) |
|  | Q Consensus | 275 | ~~~~~~~~~~~~p~~~~~~~~~~~~~~~~~~~~~~~~~~~~~~~~~~~~~~~~~~~~~~~~~~~~~~~~~~~~~~~~~~~   354 (475) |
|  |  |  | ....+.+..........+.......-.......+.........+.+........+++++.+. |
|  | T Consensus | 297 | ~~~~~~~~~~~~~~~~~~~~~~~g~~g~~~~~lp~~~~~~~~~~l~~~~~~~~~~~~~~~~~------------------   358 (405) |
|  | T PF09913.10 | 297 | LMFVRYLIGVVGSNLKPEIGQFIGAFGWLRFPLPSWVRAAYLLLVLVTAVTEFPAKSFRTWE------------------   358 (405) |
|  | T ss\_pred |  | HHHHHHHHHHHHHhhHHHHHHHHHHhhccCCCccHHHHHHHHHHHHHHHHccCCccCCCHHH------------------ |
|  |
|  |
|  | Q ss\_pred |  | ccccccccccccccccchhhcCCcCCcccccCCCCCCchHHhhhHHHHHHHhhhcCccccCChh-----------hHHHH |
|  | Q Q8N394 | 355 | LSDVEYQNSETKSSFASKVENGIKNDVSQRTQLPSTENIVVLSLSLLIIPFVPATNLFFYVGFV-----------IAERV   423 (475) |
|  | Q Consensus | 355 | ~~~~~~~~~~~~~~~~~~~~~~~~~~~~~~~~~~~~~~~~~~~l~~~~~~~~~~~~~~~~~~~~-----------~~~Ry   423 (475) |
|  |  |  | +......+.+..+++....+.......... .+.|| |
|  | T Consensus | 359 | ----------------------------------~~~~~~~~~~~~~~i~~~~~~~~~~~~~~~i~G~~~~~~~~~QgRY   404 (405) |
|  | T PF09913.10 | 359 | ----------------------------------RGVLALVLLGGVLFVHAAMCISDTTLCSGTLNSGCRDESIVFQGRY   404 (405) |
|  | T ss\_pred |  | ----------------------------------HHHHHHHHHHHHHHHHHHHHHhccCCCcceeccCCCCccceeceec |
|  |
|  |
|  | Q ss\_pred |  | h |
|  | Q Q8N394 | 424 | L   424 (475) |
|  | Q Consensus | 424 | ~   424 (475) |
|  |  |  | + |
|  | T Consensus | 405 | ~   405 (405) |
|  | T PF09913.10 | 405 | L   405 (405) |
|  | T ss\_pred |  | C |
|  |
| --- | | | |
|  | Template alignmentCDD | | |
| 12. | PF11028.9 ; DUF2723 ; Protein of unknown function (DUF2723) | | |
|  | Probability: 99.4%, E-value: 8.1e-12, Score: 105.41, Aligned cols: 156, Identities: 13%, Similarity: -0.012, | | |
|  |
|  | Q ss\_pred |  | cchhHHHhchhhCCCCchhHhhhcccccccccCCCCcccCChHHHHHHHHHHHHh--CCChHHHHHHHHHHHHHHHHHHH |
|  | Q Q8N394 | 26 | DDSRAIKTNQDLLPETPWTHIFYNDFWGTLLTHSGSHKSYRPLCTLSFRLNHAIG--GLNPWSYHLVNVLLHAAVTGLFT   103 (475) |
|  | Q Consensus | 26 | De~~~~~~~~~~~~~~~~~~~~~~~~~~~~~~~~~~~~~~~Pl~~~l~~~~~~lf--G~~~~~~rl~~~l~~~l~~~lly   103 (475) |
|  |  |  | ||..|...+......++.. .|++..+......++ |.+....|+.+++++.+++.++| |
|  | T Consensus | 1 | D~~~y~~~a~~~~~~~~p~---------------------~~l~~~~~~~~~~~~~~~~~~~~~r~~~~l~~~l~~~~~~   59 (188) |
|  | T PF11028.9 | 1 | DCGEYITAANKLEVGHPPG---------------------APLFMLLGRLFSFFAEPEMVAVWINRLSALCSSFTILFLY   59 (188) |
|  | T ss\_pred |  | ChHHHHHHHhhcCCCCCCC---------------------hHHHHHHHHHHHhcCCCCcHHHHHHHHHHHHHHHHHHHHH |
|  |
|  |
|  | Q ss\_pred |  | HHHHHHhCChH-----------------HHHHHHHHHHHCHHhHHHHHhhhchHHHHHHHHHHHHHHHHHHHHccCCCCc |
|  | Q Q8N394 | 104 | SFSKILLGDGY-----------------WTFMAGLMFASHPIHTEAVAGIVGRADVGASLFFLLSLLCYIKHCSTRGYSA   166 (475) |
|  | Q Consensus | 104 | ~l~r~l~~~~~-----------------~al~aall~a~~P~~~~~~~~~~~~~~~~~~~f~ll~l~~~l~~~~~~~~~~   166 (475) |
|  |  |  | .+.|++.+++. .+++++++++++|.....+..... |.+..++.+++++++.+..+++ |
|  | T Consensus | 60 | ~~~~~~~~~~~~~~~~~~~~~~~~~~~~~a~~a~~l~~~~p~~~~~s~~~~~--d~~~~~~~~~~l~~~~~~~~~~----   133 (188) |
|  | T PF11028.9 | 60 | WSITMFAKKIMQRKDRDWSRGDQIATLGAGIIGALAYTFSDSFWFSAVEGEV--YAMSSLFTAAIFWMILKWDAEM----   133 (188) |
|  | T ss\_pred |  | HHHHHHHHHHHhccccccccchHHHHHHHHHHHHHHHHHchhHHHHhhhcch--hHHHHHHHHHHHHHHHHHHHhc---- |
|  |
|  |
|  | Q ss\_pred |  | chH-------------HHHHHHHHHHHHHHHhHhHHHHHHHHHHHHHHHHHcccch |
|  | Q Q8N394 | 167 | RTW-------------GWFLGSGLCAGCSMLWKEQGVTVLAVSAVYDVFVFHRLKI   209 (475) |
|  | Q Consensus | 167 | ~~~-------------~~~~l~~l~~~la~ltk~~~~~~~~~~~~~~l~~~~~~~~   209 (475) |
|  |  |  | +. ++.++++++.+++.++|+.+.++++.+++..+...+++++ |
|  | T Consensus | 134 | -~~~~~~~~~~~~~~~~~~~l~g~~~~la~~~k~~~~~~~~~~~~~~~~~~~~~~~   188 (188) |
|  | T PF11028.9 | 134 | -IGIKHGEIKDSRSPMRWMILIWFMFGLAIGVHLLGLLAVPAIAYVIYFNLWEKTN   188 (188) |
|  | T ss\_pred |  | -cCccCCCcCCCCChHHHHHHHHHHHHHHHHHHHHHHHHHHHHHHHHHHhhHHhcC |
|  |
| --- | | | |
|  | Template alignmentCDD | | |
| 13. | PF04602.13 ; Arabinose\_trans ; Mycobacterial cell wall arabinan synthesis protein | | |
|  | Probability: 99.29%, E-value: 5.9e-9, Score: 98.63, Aligned cols: 347, Identities: 10%, Similarity: -0.115, | | |
|  |
|  | Q ss\_pred |  | HHHHHHHHHhhcCCCCcccchhHHHhchhhCCCCchhHhhhcccccccccCCCCcccCChHHHHHHHHHHHH--hCCChH |
|  | Q Q8N394 | 8 | SALGLALYLNTLSADFCYDDSRAIKTNQDLLPETPWTHIFYNDFWGTLLTHSGSHKSYRPLCTLSFRLNHAI--GGLNPW   85 (475) |
|  | Q Consensus | 8 | ~~~~~~~~~~~~~~~~~~De~~~~~~~~~~~~~~~~~~~~~~~~~~~~~~~~~~~~~~~Pl~~~l~~~~~~l--fG~~~~   85 (475) |
|  |  |  | .+....+....+..+...||.+|...+++..+++...+.+.. ++...+|...+ ..+...+ +|.+.. |
|  | T Consensus | 51 | ~~v~~~l~~w~~~gp~~~DEG~Yl~~ar~~~~~G~~~npy~~-----------~~~~~~Pfg~~-~~l~~~~~~~g~s~~   118 (471) |
|  | T PF04602.13 | 51 | LIVGAILLGWYFIGANTADDGYILNMARVAGHAGYMANYYRW-----------YGVPEAPFGWF-YDVTAALAALSTASP   118 (471) |
|  | T ss\_pred |  | HHHHHHHHHHHHhccCCCChHHHHHHHHHHHhcCCccccchh-----------cCCCCCCcHHH-HHHHHHHHHhcCChH |
|  |
|  |
|  | Q ss\_pred |  | HHHHHHHHHHHHHHHHHHHHHHHHh----CChHHHHHHHHHHHHCHHhHHHHHhhhchHHHHHHHHHHHHHHHHHHHHcc |
|  | Q Q8N394 | 86 | SYHLVNVLLHAAVTGLFTSFSKILL----GDGYWTFMAGLMFASHPIHTEAVAGIVGRADVGASLFFLLSLLCYIKHCST   161 (475) |
|  | Q Consensus | 86 | ~~rl~~~l~~~l~~~lly~l~r~l~----~~~~~al~aall~a~~P~~~~~~~~~~~~~~~~~~~f~ll~l~~~l~~~~~   161 (475) |
|  |  |  | ..|++++++++++.+++++...+.. .++..+..++.+..+....... ...+.|....++.++++++..|..++ |
|  | T Consensus | 119 | ~lRl~~ll~~l~~w~lL~~~vl~rl~~~~~~~~~a~~~aal~~la~wlp~~---~~lr~Ep~~al~~~~~l~l~~ra~~~   195 (471) |
|  | T PF04602.13 | 119 | FVRLTTLIASILCWWIISREVIPRLGRRARHTPAVYWTAAAVFLAFWLPYN---NGLRPEPVIAVGALLTWISVERAIAT   195 (471) |
|  | T ss\_pred |  | HHHHHHHHHHHHHHHHHHHHHHHHhccccCCcHHHHHHHHHHHHHHHcccc---CCCCcHHHHHHHHHHHHHHHHHHHhc |
|  |
|  |
|  | Q ss\_pred |  | CCCCcchHHHHHHHHHHHHHHHHhHhHHHHHHHHHHHHHHHHHcccchhhhcchHhhhHH-----------------HHH |
|  | Q Q8N394 | 162 | RGYSARTWGWFLGSGLCAGCSMLWKEQGVTVLAVSAVYDVFVFHRLKIKQILPTIYKRKN-----------------LSL   224 (475) |
|  | Q Consensus | 162 | ~~~~~~~~~~~~l~~l~~~la~ltk~~~~~~~~~~~~~~l~~~~~~~~~~~~~~~~~~~~-----------------~~~   224 (475) |
|  |  |  | + +.....+++++.++++.+|+++......+.+......++.+.+. ++. ... |
|  | T Consensus | 196 | ~-----~~~~~alag~~~gla~~aKPtg~~~la~ll~~~~~~~r~~~~r~-------~~~~~~~~~~~~~~~~~~~~~la   263 (471) |
|  | T PF04602.13 | 196 | G-----RLLPAAIATIIAAFSLAAGPTGLMAVAALLAGSRPLLAILIKRA-------KQLTPNTTTGNKHTPLASGRPHR   263 (471) |
|  | T ss\_pred |  | C-----CcHHHHHHHHHHHHHHhcCHhHHHHHHHHHHHHHHHHHHHHHHH-------HhcCCCCCCCCCCCCCCCCccHH |
|  |
|  |
|  | Q ss\_pred |  | HHHHHHHHHHHHHHHHHHHHHhcCCCCCccCCCCCCcCCChHHHHHHHHHHHHHHhHHHhhCcccccccccccccccccc |
|  | Q Q8N394 | 225 | FLSISLLIFWGSSLLGARLYWMGNKPPSFSNSDNPAADSDSLLTRTLTFFYLPTKNLWLLLCPDTLSFDWSMDAVPLLKT   304 (475) |
|  | Q Consensus | 225 | ~~~~~~~~~~~~~~~~~~~~~~~~~~~~~~~~~~~~~~~~~~~~~~~~~~~~~~~~~~~~~~p~~~~~~~~~~~~~~~~~   304 (475) |
|  |  |  | .............+. ++......+......+.....-+..+..-+...-..+...+ |
|  | T Consensus | 264 | ~~~a~~~~~l~~~f~------------------------d~sl~~~~~~~~~~~~~~~~~~~~~e~~Ry~~l~~~~~~g~   319 (471) |
|  | T PF04602.13 | 264 | PLLAAGTAVLFIIFY------------------------DQTLAAVSEASRLRTIIGPSNSWYNEFFRYSELFSQTADGS   319 (471) |
|  | T ss\_pred |  | HHHHHHHHHHHHHHh------------------------cCcHHHHHHHHHHHhhcCCCCHHhhhHHHHHHHhcCCCCCc |
|  |
|  |
|  | Q ss\_pred |  | cCCHHHHHHHHHHHHHHHHHHHHhhCcccccccCCCcccCCccccCCcccccccccccccccccccchhhcCCcCCcccc |
|  | Q Q8N394 | 305 | VCDWRNLHTVAFYTGLLLLAYYGLKSPSVDRECNGKTVTNGKQNANGHSCLSDVEYQNSETKSSFASKVENGIKNDVSQR   384 (475) |
|  | Q Consensus | 305 | ~~~~~~~~~~~~~~~~~~~~~~~~~~~~~~~~~~~~~~~~~~~~~~~~~~~~~~~~~~~~~~~~~~~~~~~~~~~~~~~~   384 (475) |
|  |  |  | ...-......++.+..+.......+| |
|  | T Consensus | 320 | ~~rr~~vll~~~~l~~~~~~l~r~~r------------------------------------------------------   345 (471) |
|  | T PF04602.13 | 320 | IARRFPVLIMIVCIFTAAAAIIHSAS------------------------------------------------------   345 (471) |
|  | T ss\_pred |  | HHHHHHHHHHHHHHHHHHHHHHHHhc------------------------------------------------------ |
|  |
|  |
|  | Q ss\_pred |  | cCCCCCC---chHHhhhHHHHHHHhhhcCccccCChhhHHHHhHHHHHHHHHHHHHHHHHHHHHhchHHHHHHHHHHHHH |
|  | Q Q8N394 | 385 | TQLPSTE---NIVVLSLSLLIIPFVPATNLFFYVGFVIAERVLYIPSMGFCLLITVGARALYVKVQKRFLKSLIFYATAT   461 (475) |
|  | Q Consensus | 385 | ~~~~~~~---~~~~~~l~~~~~~~~~~~~~~~~~~~~~~~Ry~~~~~~~~~ll~~~~l~~~~~~~~~~~~~~~~~~~~~~   461 (475) |
|  |  |  | .+. ....-.+.+.+..++...+. +.+..+|+-...+....+++..+....+...+....+......++ |
|  | T Consensus | 346 | ----~~~~~~~p~~rl~~~~~~~~~~l~~t-----ptKwthhfg~~a~~~~~~~a~~~~~~~~~~~r~~~~~~~~~~~~~   416 (471) |
|  | T PF04602.13 | 346 | ----KSKLAKGPTLRLLAVSIMSFGFLAAT-----PTKWVHHFGAFAGIGAAIAALAAVALTTPLFQSPRNRVLFTGIVV   416 (471) |
|  | T ss\_pred |  | ----cccccCChHHHHHHHHHHHHHHHHhC-----CchhhhhHHHHHHHHHHHHHHHHHHhcchhcCCHHHHHHHHHHHH |
|  |
|  |
|  | Q ss\_pred |  | HHHHHHH |
|  | Q Q8N394 | 462 | LIVFYGL   468 (475) |
|  | Q Consensus | 462 | ~~~~~~~   468 (475) |
|  |  |  | .....+. |
|  | T Consensus | 417 | ~~~al~~   423 (471) |
|  | T PF04602.13 | 417 | IIAAYAA   423 (471) |
|  | T ss\_pred |  | HHHHHHH |
|  |
| --- | | | |
|  | Template alignmentCDD | | |
| 14. | PF06728.14 ; PIG-U ; GPI transamidase subunit PIG-U | | |
|  | Probability: 99.29%, E-value: 7.8e-9, Score: 96.39, Aligned cols: 328, Identities: 7%, Similarity: -0.154, | | |
|  |
|  | Q ss\_pred |  | HHHHHHHHHHH--hhcCCC------------CcccchhHHHhchhhCCCCchhHhhhcccccccccCCCCcccCChHHHH |
|  | Q Q8N394 | 6 | VSSALGLALYL--NTLSAD------------FCYDDSRAIKTNQDLLPETPWTHIFYNDFWGTLLTHSGSHKSYRPLCTL   71 (475) |
|  | Q Consensus | 6 | ll~~~~~~~~~--~~~~~~------------~~~De~~~~~~~~~~~~~~~~~~~~~~~~~~~~~~~~~~~~~~~Pl~~~   71 (475) |
|  |  |  | ++.++..+... ...... ...|...+...++...++.+..+. ....+||+..+ |
|  | T Consensus | 1 | ~~~~~irl~~~~~~~~~~~~~~~~~~~~~~~~~~d~~~~~~~~~~~~~G~~py~~--------------~~~~ypP~~~~   66 (363) |
|  | T PF06728.14 | 1 | LGLLSISFFLQWYLANTWIAEFLYRRIEVSTPVSGFLRVREGLYLYENGLDPYSG--------------GVFYQSPLLLI   66 (363) |
|  | T ss\_pred |  | CHHHHHHHHHHHHHhcchhHHHHHhcccccCCcchHHHHHHHHHHHHcCCCCCCC--------------CcccCCcchHH |
|  |
|  |
|  | Q ss\_pred |  | HHHHHHHHhCCChHHHHHHHHHHHHHHHHHHHHHHHHHhCCh-------HHHHHHHHHHHHCHHhHHHHHhhhchHHHHH |
|  | Q Q8N394 | 72 | SFRLNHAIGGLNPWSYHLVNVLLHAAVTGLFTSFSKILLGDG-------YWTFMAGLMFASHPIHTEAVAGIVGRADVGA   144 (475) |
|  | Q Consensus | 72 | l~~~~~~lfG~~~~~~rl~~~l~~~l~~~lly~l~r~l~~~~-------~~al~aall~a~~P~~~~~~~~~~~~~~~~~   144 (475) |
|  |  |  | +..... ++.+....|+++.++.++++.++|.+.++..+++ ..+..++++++++|........... |.+. |
|  | T Consensus | 67 | l~~~~~--~~~~~~~~r~~~~~~~~~~~~l~~~~~~~~~~~~~~~~~~~~~~~~~~~~~~~~p~~~~~~~~~~~--d~~~   142 (363) |
|  | T PF06728.14 | 67 | LNYCCE--LLGGISVTRFVYTSISTMGGLFVYLIAKQARVLDPNQVLSTCSPLWISVIYLLNPLTFLPGIACSA--DMIL   142 (363) |
|  | T ss\_pred |  | HHhhhh--hcCchHHHHHHHHHHHHHHHHHHHHHHHHhhhcCcccccccCCcHHHHHHHHhCHHHHHHHHhcch--HHHH |
|  |
|  |
|  | Q ss\_pred |  | HHHHHHHHHHHHHHHccCCCCcchHHHHHHHHHHHHHHHHhHhHHHHHHHHHHHHHHHHHcccchhhhcchHhhhHHHHH |
|  | Q Q8N394 | 145 | SLFFLLSLLCYIKHCSTRGYSARTWGWFLGSGLCAGCSMLWKEQGVTVLAVSAVYDVFVFHRLKIKQILPTIYKRKNLSL   224 (475) |
|  | Q Consensus | 145 | ~~f~ll~l~~~l~~~~~~~~~~~~~~~~~l~~l~~~la~ltk~~~~~~~~~~~~~~l~~~~~~~~~~~~~~~~~~~~~~~   224 (475) |
|  |  |  | .++.+++++++. +++ ...++++.+++..+|...+.+.+....... ++++.++ ...... |
|  | T Consensus | 143 | ~~~~~~al~~~~---~~~---------~~~ag~~~gla~~~K~~~~~~~~~~~~~~~---~~~~~~~-------~~~~~~   200 (363) |
|  | T PF06728.14 | 143 | NFTTLMTIYFAS---CGS---------YAIYACCMALTVFINPNALLLFFPSYLILR---KCNSSIK-------FRQIFV   200 (363) |
|  | T ss\_pred |  | HHHHHHHHHHHh---CCC---------HHHHHHHHHHHHhcChHHHHHHHHHHHHHH---HcCchHH-------HHHHHH |
|  |
|  |
|  | Q ss\_pred |  | HHHHHHHHHHHHHHHHHH-HHHhcCCCCCccCCCCCCcCCChHHHHHHHHHHHHHHhHHHhhCccccccccccccccccc |
|  | Q Q8N394 | 225 | FLSISLLIFWGSSLLGAR-LYWMGNKPPSFSNSDNPAADSDSLLTRTLTFFYLPTKNLWLLLCPDTLSFDWSMDAVPLLK   303 (475) |
|  | Q Consensus | 225 | ~~~~~~~~~~~~~~~~~~-~~~~~~~~~~~~~~~~~~~~~~~~~~~~~~~~~~~~~~~~~~~~p~~~~~~~~~~~~~~~~   303 (475) |
|  |  |  | .............+.... ...................+.............. |
|  | T Consensus | 201 | ~~~~~~~~~~~~~~~~~~~~~~~~~~~~~~~~~~~~~~~~~~~~~~~~~~~~~---------------------------   253 (363) |
|  | T PF06728.14 | 201 | VFLFYLAGLIITSGFFLNSLSFLKIPFRVYLDSHDLTPNLGLWWYFFTEMFNE---------------------------   253 (363) |
|  | T ss\_pred |  | HHHHHHHHHHHHHHHhhcCHHHHHHHHhhhccccccCCCHHHHHHHHHhHhHH--------------------------- |
|  |
|  |
|  | Q ss\_pred |  | ccCCHHHHHHHHHHHHHHHHHHHHhhCcccccccCCCcccCCccccCCcccccccccccccccccccchhhcCCcCCccc |
|  | Q Q8N394 | 304 | TVCDWRNLHTVAFYTGLLLLAYYGLKSPSVDRECNGKTVTNGKQNANGHSCLSDVEYQNSETKSSFASKVENGIKNDVSQ   383 (475) |
|  | Q Consensus | 304 | ~~~~~~~~~~~~~~~~~~~~~~~~~~~~~~~~~~~~~~~~~~~~~~~~~~~~~~~~~~~~~~~~~~~~~~~~~~~~~~~~   383 (475) |
|  |  |  | ..............+.......++ |
|  | T Consensus | 254 | ---~~~~~~~~~~~~~~~~~~~~~~~~-----------------------------------------------------   277 (363) |
|  | T PF06728.14 | 254 | ---FRTFFLFVFAILPLMFVLPVSIRL-----------------------------------------------------   277 (363) |
|  | T ss\_pred |  | ---HHHHHHHHHHHHHHHHHHHHHHHh----------------------------------------------------- |
|  |
|  |
|  | Q ss\_pred |  | ccCCCCCCchHHhhhHHHHHHHhhhcCccccCChhhHHHHhHHHHHHHHHHHHHHHHHHHHHhchHHHHHHHHHHHHHHH |
|  | Q Q8N394 | 384 | RTQLPSTENIVVLSLSLLIIPFVPATNLFFYVGFVIAERVLYIPSMGFCLLITVGARALYVKVQKRFLKSLIFYATATLI   463 (475) |
|  | Q Consensus | 384 | ~~~~~~~~~~~~~~l~~~~~~~~~~~~~~~~~~~~~~~Ry~~~~~~~~~ll~~~~l~~~~~~~~~~~~~~~~~~~~~~~~   463 (475) |
|  |  |  | ++................. .....+|.....|.+.+... ..++.++............... |
|  | T Consensus | 278 | -------~~~~~~~~~~~~~~~~~~~-------~~~~~~y~~~~~p~~~~~~~-----~~~~~~~~~~~~~~~~~~~~~~   338 (363) |
|  | T PF06728.14 | 278 | -------YYLPLPITIALIGLHSLFK-------AYPSICDLSIFLSLLPIFNK-----VQDRMRYSLLTNNAIVFALVLG   338 (363) |
|  | T ss\_pred |  | -------ccChHHHHHHHHHHHHHhC-------CCCcHHHHHHHHHHHHhcHH-----HHHHhhhHHHHHHHHHHHHHHH |
|  |
|  |
|  | Q ss\_pred |  | HHHHHHHHHhcC |
|  | Q Q8N394 | 464 | VFYGLKTAIRNG   475 (475) |
|  | Q Consensus | 464 | ~~~~~~~~~~~~   475 (475) |
|  |  |  | ............ |
|  | T Consensus | 339 | ~~~~~~~~~~~~   350 (363) |
|  | T PF06728.14 | 339 | SAFYHSWITLGC   350 (363) |
|  | T ss\_pred |  | HHHHHHHHhcCC |
|  |
| --- | | | |
|  | Template alignmentCDD | | |
| 15. | PF15971.6 ; Mannosyl\_trans4 ; DolP-mannose mannosyltransferase | | |
|  | Probability: 99.2%, E-value: 1e-9, Score: 89.67, Aligned cols: 153, Identities: 18%, Similarity: 0.098, | | |
|  |
|  | Q ss\_pred |  | CCCcccCChHHHHHHHHHHHHhCCChHHHHHHHH----HHHHHHHHHHHHHHHHHhCChHHHHHHHHHHHHCHHhHHHHH |
|  | Q Q8N394 | 59 | SGSHKSYRPLCTLSFRLNHAIGGLNPWSYHLVNV----LLHAAVTGLFTSFSKILLGDGYWTFMAGLMFASHPIHTEAVA   134 (475) |
|  | Q Consensus | 59 | ~~~~~~~~Pl~~~l~~~~~~lfG~~~~~~rl~~~----l~~~l~~~lly~l~r~l~~~~~~al~aall~a~~P~~~~~~~   134 (475) |
|  |  |  | .+..+++||++.++....++++|.++...|+.+. +.+.+++..+|.+.|+..++++.+..++++++++|....... |
|  | T Consensus | 6 | ~~~~~~~ppl~~~~~~~~~~l~g~~~~~~~~~~~~~~~~~~~~~~~~~~~~~~~~~~~~~~~~~~~~~~~~~p~~~~~~~   85 (163) |
|  | T PF15971.6 | 6 | VDAWEPKLPLSYETTGVLALLSGGDMYRLHLLSVVLMSGAVCAIVALVVMLVYDITGDDIVAPLAGLSMFLLPGFAVRPA   85 (163) |
|  | T ss\_pred |  | ccccCCCCcHHHHHHHHHHHHhCCchHHHHHHHHHHHHHHHHHHHHHHHHHHHHHhCCCCHHHHHHHHHHHchHHhcccc |
|  |
|  |
|  | Q ss\_pred |  | hhhchHHHHHHHHHHHHHHHHHHHHccCCCCcchHHHHHHHHHHHHHHHHhHhHHHHHHHHHHHHHHHHHcccchhhhcc |
|  | Q Q8N394 | 135 | GIVGRADVGASLFFLLSLLCYIKHCSTRGYSARTWGWFLGSGLCAGCSMLWKEQGVTVLAVSAVYDVFVFHRLKIKQILP   214 (475) |
|  | Q Consensus | 135 | ~~~~~~~~~~~~f~ll~l~~~l~~~~~~~~~~~~~~~~~l~~l~~~la~ltk~~~~~~~~~~~~~~l~~~~~~~~~~~~~   214 (475) |
|  |  |  | .. .++|....++.+++++.+.+ ++ ...++++.+++.++|+......+....... +++++++ |
|  | T Consensus | 86 | ~~-~~~~~~~~~~~~~~~~~~~~---~~---------~~~~~~~~~l~~~~k~~~~~~~~~~~~~~~---~~~~~~~---   146 (163) |
|  | T PF15971.6 | 86 | YG-FKAKYLLVLCGLLAIYLYTR---GY---------PALSGVAAAASVGYWQAGAIFPLIVVGLAV---QRRDMRA---   146 (163) |
|  | T ss\_pred |  | cc-ccchHHHHHHHHHHHHHHHc---Cc---------HHHHHHHHHHHHHhhHHHhHHHHHHHHHHH---cccchHH--- |
|  |
|  |
|  | Q ss\_pred |  | hHhhhHHHHHHHHHHHHHHH |
|  | Q Q8N394 | 215 | TIYKRKNLSLFLSISLLIFW   234 (475) |
|  | Q Consensus | 215 | ~~~~~~~~~~~~~~~~~~~~   234 (475) |
|  |  |  | ................ |
|  | T Consensus | 147 | ----~~~~~~~~~~~~~~~~   162 (163) |
|  | T PF15971.6 | 147 | ----LERVVAGGLGFTIVML   162 (163) |
|  | T ss\_pred |  | ----HHHHHHHHHHHHHHHh |
|  |
| --- | | | |
|  | Template alignmentCDD | | |
| 16. | PF09586.11 ; YfhO ; Bacterial membrane protein YfhO | | |
|  | Probability: 99.07%, E-value: 9.4e-8, Score: 99.35, Aligned cols: 370, Identities: 9%, Similarity: -0.029, | | |
|  |
|  | Q ss\_pred |  | CHHHHHHHHHHHHHHHhhcCCCCc--ccchhHHHhchhhCCCCchhHhh--hcccccccccCCCCcccCChHHHHHHHHH |
|  | Q Q8N394 | 1 | MIAELVSSALGLALYLNTLSADFC--YDDSRAIKTNQDLLPETPWTHIF--YNDFWGTLLTHSGSHKSYRPLCTLSFRLN   76 (475) |
|  | Q Consensus | 1 | ~~~~lll~~~~~~~~~~~~~~~~~--~De~~~~~~~~~~~~~~~~~~~~--~~~~~~~~~~~~~~~~~~~Pl~~~l~~~~   76 (475) |
|  |  |  | ++.++++++..........+.... .|+..+....... ..+.. ...........+...+..++..+++..+. |
|  | T Consensus | 3 | ~~~~~l~~~~~~~~~~~~~~~~~~~~~D~~~~~~p~~~~-----~~~~~~~~~~~~~~~~~~~~G~~~~~~~~~~~~~p~   77 (832) |
|  | T PF09586.11 | 3 | LLPFAIIFIYGLSRHVFPFGGQTIMTVDLGQQYIDFFAY-----FRTTLLQHPDTFFYSFAKGLGGDMLGVWAYYLMSPF   77 (832) |
|  | T ss\_pred |  | HHHHHHHHHHHHHcCCCCCCCCcceeechhHhHHHHHHH-----HHHHHhhCCccceeccccCCCCccHHHHHHHHhCcc |
|  |
|  |
|  | Q ss\_pred |  | HHHhC-----CChHHHHHHHHHHHHHHHHHHHHHHHHHh-CChHHHHHHHHHHHHCHHhHHHHHhhhchHHHHHHHHHHH |
|  | Q Q8N394 | 77 | HAIGG-----LNPWSYHLVNVLLHAAVTGLFTSFSKILL-GDGYWTFMAGLMFASHPIHTEAVAGIVGRADVGASLFFLL   150 (475) |
|  | Q Consensus | 77 | ~~lfG-----~~~~~~rl~~~l~~~l~~~lly~l~r~l~-~~~~~al~aall~a~~P~~~~~~~~~~~~~~~~~~~f~ll   150 (475) |
|  |  |  | ..+.. ....+.++..++...++.+.+|.++|++. .++..|++++++++++|........ ........++.+ |
|  | T Consensus | 78 | ~~l~~~~~~~~~~~~~~~~~~l~~~l~~~~~y~l~r~~~~~~~~~a~~~a~~y~~s~~~~~~~~~---~~~~~~~~~lPl   154 (832) |
|  | T PF09586.11 | 78 | NLLVLLTPGKWLSFGVWLMVLLKYGFSGLSFAYYLKKSRLLSGWWLPTLSLTYALSGFAIANQFN---VMWLDAMIWLPL   154 (832) |
|  | T ss\_pred |  | hhHHhhCCHHHHHHHHHHHHHHHHHHHHHHHHHHHHHhccccccHHHHHHHHHHHHHHHHHHHhC---hhHHHHHHHHHH |
|  |
|  |
|  | Q ss\_pred |  | HHHHHHHHHccCCCCcchHHHHHHHHHHHHHHHHhHhHHHHHHHHHHHHHHHHH-cccchhhhcchHhhhHHHHHHHHHH |
|  | Q Q8N394 | 151 | SLLCYIKHCSTRGYSARTWGWFLGSGLCAGCSMLWKEQGVTVLAVSAVYDVFVF-HRLKIKQILPTIYKRKNLSLFLSIS   229 (475) |
|  | Q Consensus | 151 | ~l~~~l~~~~~~~~~~~~~~~~~l~~l~~~la~ltk~~~~~~~~~~~~~~l~~~-~~~~~~~~~~~~~~~~~~~~~~~~~   229 (475) |
|  |  |  | .++++.+..++++ .+...+..+++.......-.....+..+..+...... ++++.+. ++........+ |
|  | T Consensus | 155 | ~l~~~~~~~~~~~----~~~~~~~~~l~~~~~~~~~~~~~~~~~~~~l~~~~~~~~~~~~~~-------~~~~~~~~~~~   223 (832) |
|  | T PF09586.11 | 155 | VVLGIEQLFERQR----FWLYPLSLAALLIINYYMGYMVCLFVVAYFFWASVHHFKTWRQTC-------LVYLKFAGGSI   223 (832) |
|  | T ss\_pred |  | HHHHHHHHHhcCC----ccHHHHHHHHHHHHHHHHHHHHHHHHHHHHHHHHHhcchhHHHHH-------HHHHHHHHHHH |
|  |
|  |
|  | Q ss\_pred |  | HHHHHHHHHHHHHHHHhcCCCCCccCCCCCCcCCChHHHHHHHHHHHHHHhHHHhhCcccccccccccccccccccCCHH |
|  | Q Q8N394 | 230 | LLIFWGSSLLGARLYWMGNKPPSFSNSDNPAADSDSLLTRTLTFFYLPTKNLWLLLCPDTLSFDWSMDAVPLLKTVCDWR   309 (475) |
|  | Q Consensus | 230 | ~~~~~~~~~~~~~~~~~~~~~~~~~~~~~~~~~~~~~~~~~~~~~~~~~~~~~~~~~p~~~~~~~~~~~~~~~~~~~~~~   309 (475) |
|  |  |  | +.+++.+..+.-..........................+........ ........... |
|  | T Consensus | 224 | l~~~l~a~~llp~~~~~~~~~~~~~~~~~~~~~~~~~~~~~~~~~~~----------------------~~~~~~~~~~~   281 (832) |
|  | T PF09586.11 | 224 | LAGLLAAWLLLPTFFQLTQSKGQYTIQKIHWKIDYNPLKILSKLVVG----------------------NFNFDQMPKGE   281 (832) |
|  | T ss\_pred |  | HHHHHHHHHHHHHHHHHhcCcCccCccccccccCCCHHHHHHhhccC----------------------CCCcccCCCCc |
|  |
|  |
|  | Q ss\_pred |  | HHHHHHHHHHHHHHHHHHhhCcccccccCCCcccCCccccCCcccccccccccccccccccchhhcCCcCCcccccCCCC |
|  | Q Q8N394 | 310 | NLHTVAFYTGLLLLAYYGLKSPSVDRECNGKTVTNGKQNANGHSCLSDVEYQNSETKSSFASKVENGIKNDVSQRTQLPS   389 (475) |
|  | Q Consensus | 310 | ~~~~~~~~~~~~~~~~~~~~~~~~~~~~~~~~~~~~~~~~~~~~~~~~~~~~~~~~~~~~~~~~~~~~~~~~~~~~~~~~   389 (475) |
|  |  |  | .......+..+++......++++++.. |
|  | T Consensus | 282 | ~~~y~g~~~l~l~~~~~~~~~~~~~~~-----------------------------------------------------   308 (832) |
|  | T PF09586.11 | 282 | PNIFVGSLILIGFITYFLTRKIPIKER-----------------------------------------------------   308 (832) |
|  | T ss\_pred |  | hHHHHHHHHHHHHHHHHHcCCCCHHHH----------------------------------------------------- |
|  |
|  |
|  | Q ss\_pred |  | CCchHHhhhHHHHHHHhhhcCccccCChhhH---HHHhHHHHHHHHHHHHHHHHHHHHHhchHHHHHHHHHHHHHHHHH |
|  | Q Q8N394 | 390 | TENIVVLSLSLLIIPFVPATNLFFYVGFVIA---ERVLYIPSMGFCLLITVGARALYVKVQKRFLKSLIFYATATLIVF   465 (475) |
|  | Q Consensus | 390 | ~~~~~~~~l~~~~~~~~~~~~~~~~~~~~~~---~Ry~~~~~~~~~ll~~~~l~~~~~~~~~~~~~~~~~~~~~~~~~~   465 (475) |
|  |  |  | .......++.++....+............. .|+.+...+.++++++.++.++.++.+++........+++++... |
|  | T Consensus | 309 | -~~~~~~~~~l~~~~~~~~~~~~~~~~~~~~~~~~R~~~~~~~~~~ila~~~l~~~~~~~~~~~~~~~~~~~~~~~~~~   386 (832) |
|  | T PF09586.11 | 309 | -LAALLVTGFLGLSLCFEPLDLLWHGMQFPVWYPYRFSYVISFWLIVLAVQRLHYQPQFKWYSLLAPLLLLAASLAYTF   386 (832) |
|  | T ss\_pred |  | -HHHHHHHHHHHHHhcCHHHHHHHhcCCCCCCCcHHHHHHHHHHHHHHHHHHHhcCCcCChhHHHHHHHHHHHHHHHHH |
|  |
| --- | | | |
|  | Template alignmentCDD | | |
| 17. | PF03155.16 ; Alg6\_Alg8 ; ALG6, ALG8 glycosyltransferase family | | |
|  | Probability: 98.91%, E-value: 9.4e-7, Score: 85.14, Aligned cols: 319, Identities: 10%, Similarity: -0.021, | | |
|  |
|  | Q ss\_pred |  | CcccchhHHHhchhhCCCCchhHhhhcccccccccCCCCcccCChHHHHHHHHHHHHh-----------------CCChH |
|  | Q Q8N394 | 23 | FCYDDSRAIKTNQDLLPETPWTHIFYNDFWGTLLTHSGSHKSYRPLCTLSFRLNHAIG-----------------GLNPW   85 (475) |
|  | Q Consensus | 23 | ~~~De~~~~~~~~~~~~~~~~~~~~~~~~~~~~~~~~~~~~~~~Pl~~~l~~~~~~lf-----------------G~~~~   85 (475) |
|  |  |  | +..+|........+...+.+..+++.++. +.+...|||+..+...+..++. +.... |
|  | T Consensus | 12 | ~~s~D~~~~r~w~~~t~~~p~~~wy~~~~-------~~w~ldYPPl~a~~~~~~~~~~~~~~~~~~~l~~~~~~~~~~~~   84 (470) |
|  | T PF03155.16 | 12 | YHSTDFEVHRNWLAITHSLPLNQWYVDAT-------SEWTLDYPPFFAYFEWLLSQVAKYVDPRMLVVDNLNYESKATVY   84 (470) |
|  | T ss\_pred |  | cCCCcHHHHHHHHHHHhhCCHHHhccCCC-------ccCCCCCcHHHHHHHHHHHHHHHHhCHHHhhcccCCCCCHHHHH |
|  |
|  |
|  | Q ss\_pred |  | HHHHHHHHHHHHHHHHHHHHHH--HHhCChHHHHHHHHHHHHCHHhHHHHH-hhhchHHHHHHHHHHHHHHHHHHHHccC |
|  | Q Q8N394 | 86 | SYHLVNVLLHAAVTGLFTSFSK--ILLGDGYWTFMAGLMFASHPIHTEAVA-GIVGRADVGASLFFLLSLLCYIKHCSTR   162 (475) |
|  | Q Consensus | 86 | ~~rl~~~l~~~l~~~lly~l~r--~l~~~~~~al~aall~a~~P~~~~~~~-~~~~~~~~~~~~f~ll~l~~~l~~~~~~   162 (475) |
|  |  |  | ..|+..++..++....++.+.+ +...++..+..++++++++|......+ +... |.....+.+++++++.+ ++ |
|  | T Consensus | 85 | ~~R~~vi~~d~l~~~~v~~~~~~~~~~~~~~~~~~~~~l~l~~P~li~~d~~~~q~--n~~~~~l~llsl~~~~~---~~   159 (470) |
|  | T PF03155.16 | 85 | FQRLSVIATDLVYVLGVRSCLGSLGLARDTQQFFAGSMLLLLNVGLLFVDHIHFQY--NGLLFGILLLSIGSLIR---QR   159 (470) |
|  | T ss\_pred |  | HHHHHHHHHHHHHHHHHHHHHHhcCCCCChhHHHHHHHHHHHcHHHHHhhcccccc--hHHHHHHHHHHHHHHHc---Cc |
|  |
|  |
|  | Q ss\_pred |  | CCCcchHHHHHHHHHHHHHHHHhHhHHHHHHHHHHHHHHHHHcccchhhhcchHhhhHHHHHHHHHHHHHHHHHHHHHHH |
|  | Q Q8N394 | 163 | GYSARTWGWFLGSGLCAGCSMLWKEQGVTVLAVSAVYDVFVFHRLKIKQILPTIYKRKNLSLFLSISLLIFWGSSLLGAR   242 (475) |
|  | Q Consensus | 163 | ~~~~~~~~~~~l~~l~~~la~ltk~~~~~~~~~~~~~~l~~~~~~~~~~~~~~~~~~~~~~~~~~~~~~~~~~~~~~~~~   242 (475) |
|  |  |  | ...+++++++++.+|...+.+.+...++.+.....++.+. .+...........+.......... |
|  | T Consensus | 160 | ---------~~~a~~~~~lal~~K~~~l~~~p~~~~~ll~~~~~~~~~~-------~~~~~~~~~~~~~~~~~~~~~~~P   223 (470) |
|  | T PF03155.16 | 160 | ---------FLWSAFAFAVLLNFKHIFLYMAPAFGVYLLRFYCLEQASV-------ASAVGAVIKLLVVGLTPFAVSFGP   223 (470) |
|  | T ss\_pred |  | ---------hHHHHHHHHHHHHcchHHHHHHHHHHHHHHHHhcccCCCH-------hHHHHHHHHHHHHHHHHHHHhccc |
|  |
|  |
|  | Q ss\_pred |  | HHHhcCCCCCcc-----CCCCCCcCCChHHHHHHHHHHHHHHhHHHhhCcccccccccccccccccccCCHHHHHHHHHH |
|  | Q Q8N394 | 243 | LYWMGNKPPSFS-----NSDNPAADSDSLLTRTLTFFYLPTKNLWLLLCPDTLSFDWSMDAVPLLKTVCDWRNLHTVAFY   317 (475) |
|  | Q Consensus | 243 | ~~~~~~~~~~~~-----~~~~~~~~~~~~~~~~~~~~~~~~~~~~~~~~p~~~~~~~~~~~~~~~~~~~~~~~~~~~~~~   317 (475) |
|  |  |  | + .+....... ..+..........-...+..............+.........+...................+ |
|  | T Consensus | 224 | f--~~~~~~~~~rlfp~~rgl~~~~~a~n~w~~~~~~~~~~~~~~~~~~~~~~~t~g~~~~~~~~~~~~~~~~~~~~~~l   301 (470) |
|  | T PF03155.16 | 224 | F--WKQLPQVLSRLFPFKRGLTHAYWAPNFWALYNTADKVAAGVLKVHDGGASTTSGLVQEVRHSVLPAITPPVTFALTA   301 (470) |
|  | T ss\_pred |  | c--cccHHHHHHHHCCccccccccchhhHHHHHHHHHHHHHHHHHhcCCCCccccCCccccchhhcCCccHHHHHHHHHH |
|  |
|  |
|  | Q ss\_pred |  | HHHHHHHHHHhhCcccccccCCCcccCCccccCCcccccccccccccccccccchhhcCCcCCcccccCCCC---CCchH |
|  | Q Q8N394 | 318 | TGLLLLAYYGLKSPSVDRECNGKTVTNGKQNANGHSCLSDVEYQNSETKSSFASKVENGIKNDVSQRTQLPS---TENIV   394 (475) |
|  | Q Consensus | 318 | ~~~~~~~~~~~~~~~~~~~~~~~~~~~~~~~~~~~~~~~~~~~~~~~~~~~~~~~~~~~~~~~~~~~~~~~~---~~~~~   394 (475) |
|  |  |  | +..+.......++ + ..+.. |
|  | T Consensus | 302 | ~~~~~~~~~l~~~----------------------------------------------------------~~~~~~~~~   323 (470) |
|  | T PF03155.16 | 302 | LFMLPILVKLFRS----------------------------------------------------------PKKQSPLVF   323 (470) |
|  | T ss\_pred |  | HHHHHHHHHHHcC----------------------------------------------------------CCCCCHHHH |
|  |
|  |
|  | Q ss\_pred |  | HhhhHHHHHHHhhhcCccccCChhhHHHHhHHHHHHHHHHHH |
|  | Q Q8N394 | 395 | VLSLSLLIIPFVPATNLFFYVGFVIAERVLYIPSMGFCLLIT   436 (475) |
|  | Q Consensus | 395 | ~~~l~~~~~~~~~~~~~~~~~~~~~~~Ry~~~~~~~~~ll~~   436 (475) |
|  |  |  | ...+......+..+. ...+++|.+.+.+++++++. |
|  | T Consensus | 324 | ~~~~~~~~l~~flf~-------~~vhek~ill~l~Pl~ll~~   358 (470) |
|  | T PF03155.16 | 324 | LRAVVLCGCSSFVFG-------WHVHEKAILMVLLPLCLLTL   358 (470) |
|  | T ss\_pred |  | HHHHHHHHHHHHHhc-------hhhcchHHHHHHHHHHHHHH |
|  |
| --- | | | |
|  | Template alignmentCDD | | |
| 18. | PF04922.13 ; DIE2\_ALG10 ; DIE2/ALG10 family | | |
|  | Probability: 98.9%, E-value: 1.1e-7, Score: 89.27, Aligned cols: 195, Identities: 14%, Similarity: 0.025, | | |
|  |
|  | Q ss\_pred |  | CCCcccchhHHHhchhhCCCCchhHhhhcccccccccCCCCcccCChHHHHHHHHHHHHhC---------CChHHHHHHH |
|  | Q Q8N394 | 21 | ADFCYDDSRAIKTNQDLLPETPWTHIFYNDFWGTLLTHSGSHKSYRPLCTLSFRLNHAIGG---------LNPWSYHLVN   91 (475) |
|  | Q Consensus | 21 | ~~~~~De~~~~~~~~~~~~~~~~~~~~~~~~~~~~~~~~~~~~~~~Pl~~~l~~~~~~lfG---------~~~~~~rl~~   91 (475) |
|  |  |  | ...+.||..|..+++.+.+ +++..+ +.....+|+++++.+...+++| .+....|+.+ |
|  | T Consensus | 2 | p~py~DE~fH~~qa~~y~~-G~~~~w-------------dp~iTTpPGlyl~~a~~~~l~g~~~~~~~~~~s~~~LR~~n   67 (434) |
|  | T PF04922.13 | 2 | PTPYIDEIFHIPQTQQYCK-GHWNAW-------------DSKITTPPGLYIIGYAWARMLTLTGLSESEACSTLSLRAVN   67 (434) |
|  | T ss\_pred |  | CCCCcchHhhHHHHHHHHc-CCcccc-------------CccCCCChhHHHHHHHHHHHHHHhcCCcccCCCHHHHHHHH |
|  |
|  |
|  | Q ss\_pred |  | HHHHH-HHHHHHHHHHHHHhCChHHHHHHHHHHHHCHHhHHHHHhhhchHHHHHHHHHHHHHHHHHHHHccCCCCcchHH |
|  | Q Q8N394 | 92 | VLLHA-AVTGLFTSFSKILLGDGYWTFMAGLMFASHPIHTEAVAGIVGRADVGASLFFLLSLLCYIKHCSTRGYSARTWG   170 (475) |
|  | Q Consensus | 92 | ~l~~~-l~~~lly~l~r~l~~~~~~al~aall~a~~P~~~~~~~~~~~~~~~~~~~f~ll~l~~~l~~~~~~~~~~~~~~   170 (475) |
|  |  |  | ++++. +...+++.+.++..++. +...++.++++|.....+...++ |.+.++++++++++..+..+++ ... |
|  | T Consensus | 68 | ll~~~~~~~~~~~~l~~~~~~~~--a~l~al~l~~~Pl~~~~sfl~YT--Dv~Sl~~vll~l~~~l~~~~~~-----~~~   138 (434) |
|  | T PF04922.13 | 68 | LMAVVIYIPATLYIIQRRVWGSQ--AHFSAFSLVSFPLIWFYAALYYT--DVWSTATVLMALAFALSPRVPF-----YMV   138 (434) |
|  | T ss\_pred |  | HHHHHHHHHHHHHHHHHHhcCch--HHHHHHHHHhcHHHHHHHHhhch--HHHHHHHHHHHHHHHhCCCCCc-----cHH |
|  |
|  |
|  | Q ss\_pred |  | HHHHHHHHHHHHHHhHhHHHHHHHHHHHHHHHHHcccchhhhcchHhhhHHHHHHHH---------------HHHHHHHH |
|  | Q Q8N394 | 171 | WFLGSGLCAGCSMLWKEQGVTVLAVSAVYDVFVFHRLKIKQILPTIYKRKNLSLFLS---------------ISLLIFWG   235 (475) |
|  | Q Consensus | 171 | ~~~l~~l~~~la~ltk~~~~~~~~~~~~~~l~~~~~~~~~~~~~~~~~~~~~~~~~~---------------~~~~~~~~   235 (475) |
|  |  |  | .+++++.++++++|++.++.........+....++..+...+.. .+....... .++..+.. |
|  | T Consensus | 139 | --~la~l~~~lavl~RQtnIvW~~f~~~~~~~~~~~~~~~~~~~~~--~~~l~~~~~~~~~~~~~~~~~~~~~~~v~~~F   214 (434) |
|  | T PF04922.13 | 139 | --QLSALMCAVSLFFRQTNILWAAVVAVIAIENSHYSNGAPPKNGA--LAQIFSTISYTFQIELPIFNILISYASVAVGF   214 (434) |
|  | T ss\_pred |  | --HHHHHHHHHHHHhchhHHHHHHHHHHHHHHHHhhhCCCCCcccH--HHHHHHHHHHHHhhchHHHHHHHHHHHHHHHH |
|  |
|  |
|  | Q ss\_pred |  | HHHHHHH |
|  | Q Q8N394 | 236 | SSLLGAR   242 (475) |
|  | Q Consensus | 236 | ~~~~~~~   242 (475) |
|  |  |  | ..+..++ |
|  | T Consensus | 215 | ~~Fv~~N   221 (434) |
|  | T PF04922.13 | 215 | SFFLYIN   221 (434) |
|  | T ss\_pred |  | HHHHHhC |
|  |
| --- | | | |
|  | Template alignmentCDD | | |
| 19. | PF14264.7 ; Glucos\_trans\_II ; Glucosyl transferase GtrII | | |
|  | Probability: 98.84%, E-value: 0.0000028, Score: 76.98, Aligned cols: 303, Identities: 15%, Similarity: 0.046, | | |
|  |
|  | Q ss\_pred |  | cCCCCcccchhHHHhchhhCCCCchhHhhhcccccccccCCCCcccCChHHHHHHHHHHHHhCCChHHHHHHHHHHHHHH |
|  | Q Q8N394 | 19 | LSADFCYDDSRAIKTNQDLLPETPWTHIFYNDFWGTLLTHSGSHKSYRPLCTLSFRLNHAIGGLNPWSYHLVNVLLHAAV   98 (475) |
|  | Q Consensus | 19 | ~~~~~~~De~~~~~~~~~~~~~~~~~~~~~~~~~~~~~~~~~~~~~~~Pl~~~l~~~~~~lfG~~~~~~rl~~~l~~~l~   98 (475) |
|  |  |  | .+..+..||...........+. +..++ ||+...+... ....+. ++..++.++++..++ |
|  | T Consensus | 5 | ~~~~~~~Dd~~~~~~~~~~~~~-----~~~~G---------------R~~~~~l~~~-~~~~~~-p~~~~~l~~~~~~~s   62 (312) |
|  | T PF14264.7 | 5 | FHSSFSHDSLNALYSDMTEIKW-----KLALG---------------RFVVPLIMKI-RGQIAL-PWLIGIVSLFLIAAS   62 (312) |
|  | T ss\_pred |  | cccCCCccchhHhhcCchhhhH-----HHhcc---------------chhHHHHHHH-ccccch-hHHHHHHHHHHHHHH |
|  |
|  |
|  | Q ss\_pred |  | HHHHHHHHHHHhCChHHHHHHHHHHHHCHHhHH-HHHhh-hchHHHHHHHHHHHHHHHHHHHHccCCCCcchHHHHHHHH |
|  | Q Q8N394 | 99 | TGLFTSFSKILLGDGYWTFMAGLMFASHPIHTE-AVAGI-VGRADVGASLFFLLSLLCYIKHCSTRGYSARTWGWFLGSG   176 (475) |
|  | Q Consensus | 99 | ~~lly~l~r~l~~~~~~al~aall~a~~P~~~~-~~~~~-~~~~~~~~~~f~ll~l~~~l~~~~~~~~~~~~~~~~~l~~   176 (475) |
|  |  |  | +.+++...++ +++..+.+++.++..+|.+.+ ...+. .+.+.....++..++.+... +++ +++..+.+. |
|  | T Consensus | 63 | ~~l~~~~~~~--~~~~~~~~~~~l~~~~P~~~~~~~~f~~~~~~~~~~~ll~~la~~~~~---~~~-----~~~~~~~~~   132 (312) |
|  | T PF14264.7 | 63 | LYLILETIQI--DSKAMIILVSIFMVTNRTIYSMTATYIYELDYDMLALFFASLAAYILM---KKD-----KPGWYLLAF   132 (312) |
|  | T ss\_pred |  | HHHHHHHhcC--CcHHHHHHHHHHHHHhHHHHHHHHHHHHhcHHHHHHHHHHHHHHHHHH---hCC-----CCchHHHHH |
|  |
|  |
|  | Q ss\_pred |  | HHHHHHHHhHhHHHHHHHHHHHHHHHHHcccchhhhcchHhhhHHHHHHHHHHHHHHHHHHHHHHHHHHhcCCCCCccCC |
|  | Q Q8N394 | 177 | LCAGCSMLWKEQGVTVLAVSAVYDVFVFHRLKIKQILPTIYKRKNLSLFLSISLLIFWGSSLLGARLYWMGNKPPSFSNS   256 (475) |
|  | Q Consensus | 177 | l~~~la~ltk~~~~~~~~~~~~~~l~~~~~~~~~~~~~~~~~~~~~~~~~~~~~~~~~~~~~~~~~~~~~~~~~~~~~~~   256 (475) |
|  |  |  | ++..+++.+++......+...+...+....+..+..++ .......... .++....-+...+........+..... |
|  | T Consensus | 133 | l~~~~sl~~YQ~~~~~~~~~~~~~~l~~~~~~~~~~k~----~~~~~~~~~~-~~~~~i~y~i~~k~~~~~~~~~~~~~~   207 (312) |
|  | T PF14264.7 | 133 | LSGVLSLGLYQSYIEVAFAIVIIASLKNLLEGSKYSQV----LKRGIIAIVS-FVLSVVAYYLIYKLSCKFFNVQIEGRT   207 (312) |
|  | T ss\_pred |  | HHHHHHHHHHHHHHHHHHHHHHHHHHHHHHcCCCcHHH----HHHHHHHHHH-HHHHHHHHHHHHHHHHHHcCCCCCCCC |
|  |
|  |
|  | Q ss\_pred |  | CCCCcCCChHHHHHHHHHHHHHHhHHHhhCcccccccccccccccccccCCHHHHHHHHHHHHHHHHHHHHhhCcccccc |
|  | Q Q8N394 | 257 | DNPAADSDSLLTRTLTFFYLPTKNLWLLLCPDTLSFDWSMDAVPLLKTVCDWRNLHTVAFYTGLLLLAYYGLKSPSVDRE   336 (475) |
|  | Q Consensus | 257 | ~~~~~~~~~~~~~~~~~~~~~~~~~~~~~~p~~~~~~~~~~~~~~~~~~~~~~~~~~~~~~~~~~~~~~~~~~~~~~~~~   336 (475) |
|  |  |  | +.......+..+...+..++..++............... .........+..........+++ |
|  | T Consensus | 208 | ~~~~~~~~~~~~~i~~~~~~~~~~~~~~~~~~~~~~~~~------------~~~~~~~~~~~~~~~~~~~~~~~------   269 (312) |
|  | T PF14264.7 | 208 | DAFSGEYTSIIVSLKVMLYKLIHDVVKPGTIYELPIVGI------------ADILLIAIGVALCLIMIFKLGKG------   269 (312) |
|  | T ss\_pred |  | CccccccccHHHHHHHHHHHHHHHhhCCCCCCCCccHHH------------HHHHHHHHHHHHHHHHHHHHcCC------ |
|  |
|  |
|  | Q ss\_pred |  | cCCCcccCCccccCCcccccccccccccccccccchhhcCCcCCcccccCCCCCCchHHhhhHHHHHHHhhhcCcccc-C |
|  | Q Q8N394 | 337 | CNGKTVTNGKQNANGHSCLSDVEYQNSETKSSFASKVENGIKNDVSQRTQLPSTENIVVLSLSLLIIPFVPATNLFFY-V   415 (475) |
|  | Q Consensus | 337 | ~~~~~~~~~~~~~~~~~~~~~~~~~~~~~~~~~~~~~~~~~~~~~~~~~~~~~~~~~~~~~l~~~~~~~~~~~~~~~~-~   415 (475) |
|  |  |  | ++... .+..++..+.|+...... . |
|  | T Consensus | 270 | ------------------------------------------------------~~~~~-~l~~~~~~~~p~~~~~i~~~   294 (312) |
|  | T PF14264.7 | 270 | ------------------------------------------------------KTGEK-VVSLLLLAALPLSLNLICLT   294 (312) |
|  | T ss\_pred |  | ------------------------------------------------------CcHHH-HHHHHHHHHHHHHHHHHHHH |
|  |
|  |
|  | Q ss\_pred |  | ChhhHHHHhHHHHHHH |
|  | Q Q8N394 | 416 | GFVIAERVLYIPSMGF   431 (475) |
|  | Q Consensus | 416 | ~~~~~~Ry~~~~~~~~   431 (475) |
|  |  |  | .....+|-.++....+ |
|  | T Consensus | 295 | ~~~~~~r~l~~~~~~~   310 (312) |
|  | T PF14264.7 | 295 | IKSGSEHDLMTYSFNF   310 (312) |
|  | T ss\_pred |  | CCCCCcchhhhhhhhe |
|  |
| --- | | | |
|  | Template alignmentCDD | | |
| 20. | PF05208.14 ; ALG3 ; ALG3 protein | | |
|  | Probability: 98.69%, E-value: 0.0000026, Score: 78.46, Aligned cols: 200, Identities: 10%, Similarity: -0.053, | | |
|  |
|  | Q ss\_pred |  | HHHHHHHHHHHHHhhcCCCCcccchhHHHhchhhCCCCchhHhhhcccccccccCCCCcccCChHHHHHHHHHHHHhCC- |
|  | Q Q8N394 | 4 | ELVSSALGLALYLNTLSADFCYDDSRAIKTNQDLLPETPWTHIFYNDFWGTLLTHSGSHKSYRPLCTLSFRLNHAIGGL-   82 (475) |
|  | Q Consensus | 4 | ~lll~~~~~~~~~~~~~~~~~~De~~~~~~~~~~~~~~~~~~~~~~~~~~~~~~~~~~~~~~~Pl~~~l~~~~~~lfG~-   82 (475) |
|  |  |  | .+++.-+++.............|+..|...++.+.++.........+ .....+||++.+++...+.+.+. |
|  | T Consensus | 2 | ~~~~~~~~~~~~~i~~~~yt~iD~~~y~~~~~~i~~G~~pY~~~~~~---------~~p~~Ypp~~~yi~~~l~~l~~~~   72 (356) |
|  | T PF05208.14 | 2 | LLVLVDAVLSALIIKKVSYTEIDWTTYMQQIALYQAGERDYTAIKGD---------TGPLVYPASHVYIYSFLYELTNKG   72 (356) |
|  | T ss\_pred |  | HHHHHHHHHHHHHHHhCCCCCCcHHHHHHHHHHHHcCCCChhhccCC---------CCCCCCcHHHHHHHHHHHHHhcCC |
|  |
|  |
|  | Q ss\_pred |  | -ChHHHHHHHHHHHHHHHHHHHHHHHHHhCChHHHHHHHHHHHHCHHhHHHHHhhhchHHHHHHHHHHHHHHHHHHHHcc |
|  | Q Q8N394 | 83 | -NPWSYHLVNVLLHAAVTGLFTSFSKILLGDGYWTFMAGLMFASHPIHTEAVAGIVGRADVGASLFFLLSLLCYIKHCST   161 (475) |
|  | Q Consensus | 83 | -~~~~~rl~~~l~~~l~~~lly~l~r~l~~~~~~al~aall~a~~P~~~~~~~~~~~~~~~~~~~f~ll~l~~~l~~~~~   161 (475) |
|  |  |  | +....|..-..+.+++..+++.+.++ ++..+..++.++...|.+.....+..+ |...+++.+++++++.+.+ |
|  | T Consensus | 73 | ~~i~~~~~~f~~~~l~~~~li~~i~~~---~~~~~~~~~~l~l~~pl~s~~~~~g~~--D~i~~~~lll~l~~l~~~~--   145 (356) |
|  | T PF05208.14 | 73 | QDIELGQYIFAGIYIATLIVVLSCYIK---AGAPPYLLPLLVLSKRLHSIYMLRLFN--DGIATLAMWVAIFFFQRRQ--   145 (356) |
|  | T ss\_pred |  | CChHHHHHHHHHHHHHHHHHHHHHHHH---cCCChhHHHHHHHccHHHHHHHHhhhc--HHHHHHHHHHHHHHHHhCC-- |
|  |
|  |
|  | Q ss\_pred |  | CCCCcchHHHHHHHHHHHHHHHHhHhHHHHHHHHHHHHHHHHHcccchhhhcchHhhhHHHHHHHHHHHHHHHHHHHHH |
|  | Q Q8N394 | 162 | RGYSARTWGWFLGSGLCAGCSMLWKEQGVTVLAVSAVYDVFVFHRLKIKQILPTIYKRKNLSLFLSISLLIFWGSSLLG   240 (475) |
|  | Q Consensus | 162 | ~~~~~~~~~~~~l~~l~~~la~ltk~~~~~~~~~~~~~~l~~~~~~~~~~~~~~~~~~~~~~~~~~~~~~~~~~~~~~~   240 (475) |
|  |  |  | ..+++++.++|+.+|...++..+.+.......++.+ +............++....+.. |
|  | T Consensus | 146 | ----------~~la~i~~glAv~~K~~~ll~~P~ll~~l~~~~~~~-----------~~~~~~~~~~~~~~l~~lPfl~   203 (356) |
|  | T PF05208.14 | 146 | ----------LTVATTVWSLGVGVKMSLLLLAPGVAIVIALSGGIW-----------AAVPLALNAVLTQVLLGIPFLQ   203 (356) |
|  | T ss\_pred |  | ----------HHHHHHHHHHHHHHHHHHHHHHHHHHHHHHHcCCHH-----------HHHHHHHHHHHHHHHHHHHHHh |
|  |
| --- | | | |
|  | Template alignmentCDD | | |
| 21. | PF09594.11 ; GT87 ; Glycosyltransferase family 87 | | |
|  | Probability: 98.56%, E-value: 0.0000064, Score: 71.91, Aligned cols: 243, Identities: 10%, Similarity: -0.006, | | |
|  |
|  | Q ss\_pred |  | cCChHHHHHHHHHHHHhCC---ChHHHHHHHHHHHHHHHHHHHHHHHHHhCCh--HHHHHHHHHHHHCHHhHHHHHhhhc |
|  | Q Q8N394 | 64 | SYRPLCTLSFRLNHAIGGL---NPWSYHLVNVLLHAAVTGLFTSFSKILLGDG--YWTFMAGLMFASHPIHTEAVAGIVG   138 (475) |
|  | Q Consensus | 64 | ~~~Pl~~~l~~~~~~lfG~---~~~~~rl~~~l~~~l~~~lly~l~r~l~~~~--~~al~aall~a~~P~~~~~~~~~~~   138 (475) |
|  |  |  | .|||...++..+. .+.|+ .....++..++..+..+..++.+.|+..+++ ......+++++.+|. .......+. |
|  | T Consensus | 2 | ~YpP~~~~l~~~~-~l~~~~~~~~~~~~~~~~~~~~~~~~~~~~~~r~~~~~~~~~~~~~~~~~~~~~p~-~~~~~~g~~   79 (251) |
|  | T PF09594.11 | 2 | TYPPFGALVFTPL-WWIHDLFGLLVTERVFALITLLTTYAVAVFLLRLAGVRDRVWEFVAFAALLVSAPV-YFTLNIGQI   79 (251) |
|  | T ss\_pred |  | CCChHHHHHHHHH-hhccchhcHHHHHHHHHHHHHHHHHHHHHHHHHHcCCCCcHHHHHHHHHHHHHHHH-HHHhhcCCH |
|  |
|  |
|  | Q ss\_pred |  | hHHHHHHHHHHHHHHHHHHHHccCCCCcchHHHHHHHHHHHHHHHHhHhHHHHHHHHHHHHHHHHHcccchhhhcchHhh |
|  | Q Q8N394 | 139 | RADVGASLFFLLSLLCYIKHCSTRGYSARTWGWFLGSGLCAGCSMLWKEQGVTVLAVSAVYDVFVFHRLKIKQILPTIYK   218 (475) |
|  | Q Consensus | 139 | ~~~~~~~~f~ll~l~~~l~~~~~~~~~~~~~~~~~l~~l~~~la~ltk~~~~~~~~~~~~~~l~~~~~~~~~~~~~~~~~   218 (475) |
|  |  |  | |.+..++.++++++.. ..+++ +.++...++++.+++...|.....+.+.+....+..++. |
|  | T Consensus | 80 | --~~~~~~~~~~~l~~~~-~~~~~-----~~~~~~~ag~~l~la~~~K~~~~~~~~~ll~~~~~~~~~------------   139 (251) |
|  | T PF09594.11 | 80 | --NVMLMALTLFDVALPR-STRHS-----GVLKYVPLGVLTGIAAAIKLTPLVFGLYFLILWVVTKSP------------   139 (251) |
|  | T ss\_pred |  | --HHHHHHHHHHHHHhcc-ccCCC-----CccccHHHHHHHHHHHHhhHHHHHHHHHHHHHHHHcCCH------------ |
|  |
|  |
|  | Q ss\_pred |  | hHHHHHHHHHHHHHHHHHHHHHHHHHHhcCCCCCccCCCCCCcCCChHHHHHHHHHHHHHHhHHHhhCcccccccccccc |
|  | Q Q8N394 | 219 | RKNLSLFLSISLLIFWGSSLLGARLYWMGNKPPSFSNSDNPAADSDSLLTRTLTFFYLPTKNLWLLLCPDTLSFDWSMDA   298 (475) |
|  | Q Consensus | 219 | ~~~~~~~~~~~~~~~~~~~~~~~~~~~~~~~~~~~~~~~~~~~~~~~~~~~~~~~~~~~~~~~~~~~~p~~~~~~~~~~~   298 (475) |
|  |  |  | +...............+..... .+......+..... +.......+...+....... |
|  | T Consensus | 140 | r~~~~~~~~~~~~~~~~~~~~~-----------------------~~~~~~~~~~~~~~-~~~~~~~~~~~~~l~~~~~~   195 (251) |
|  | T PF09594.11 | 140 | RGLFGMIGGFLGASGLAIIFRP-----------------------SISIQYFTDVLFTA-ERIGDLHFARNVSIRAVLER   195 (251) |
|  | T ss\_pred |  | HHHHHHHHHHHHHHHHHHHHCC-----------------------hHHHHHHHHHHhcc-cccCCccccccccHHHHHHh |
|  |
|  |
|  | Q ss\_pred |  | cccccccCCHHHHHHHHHHHHHHHHHHHHhhCcccccccCCCcccCCccccCCcccccccccccccccccccchhhcCCc |
|  | Q Q8N394 | 299 | VPLLKTVCDWRNLHTVAFYTGLLLLAYYGLKSPSVDRECNGKTVTNGKQNANGHSCLSDVEYQNSETKSSFASKVENGIK   378 (475) |
|  | Q Consensus | 299 | ~~~~~~~~~~~~~~~~~~~~~~~~~~~~~~~~~~~~~~~~~~~~~~~~~~~~~~~~~~~~~~~~~~~~~~~~~~~~~~~~   378 (475) |
|  |  |  | ...................+.+.+...+..++++.+++ |
|  | T Consensus | 196 | ~~~~~~~~~~~~~~~~~~~~~~~~~~~~~~~~~~~~~~------------------------------------------   233 (251) |
|  | T PF09594.11 | 196 | LPELGSAASIMWLVAVALVIIAVAVAAYRILRTDLSAH------------------------------------------   233 (251) |
|  | T ss\_pred |  | cccCCcHHHHHHHHHHHHHHHHHHHHHHHHHhcccccC------------------------------------------ |
|  |
|  |
|  | Q ss\_pred |  | CCcccccCCCCCCchHHhhhHHHHHHHh |
|  | Q Q8N394 | 379 | NDVSQRTQLPSTENIVVLSLSLLIIPFV   406 (475) |
|  | Q Consensus | 379 | ~~~~~~~~~~~~~~~~~~~l~~~~~~~~   406 (475) |
|  |  |  | .......+..++..++ |
|  | T Consensus | 234 | ------------~~~~~~~~~~~~~~l~   249 (251) |
|  | T PF09594.11 | 234 | ------------NRLLAVSLVSLVALLC   249 (251) |
|  | T ss\_pred |  | ------------ChHHHHHHHHHHHHHh |
|  |
| --- | | | |
|  | Template alignmentCDD | | |
| 22. | PF05007.14 ; Mannosyl\_trans ; Mannosyltransferase (PIG-M) | | |
|  | Probability: 95.04%, E-value: 0.67, Score: 39.57, Aligned cols: 192, Identities: 13%, Similarity: -0.035, | | |
|  |
|  | Q ss\_pred |  | chHHHHHHHHHHHHHHHHHHHHccCCCCcchHHHHHHHHHHHHHHHHhHhHHHHHHHHHHHHHHHHHcccchhhhcchHh |
|  | Q Q8N394 | 138 | GRADVGASLFFLLSLLCYIKHCSTRGYSARTWGWFLGSGLCAGCSMLWKEQGVTVLAVSAVYDVFVFHRLKIKQILPTIY   217 (475) |
|  | Q Consensus | 138 | ~~~~~~~~~f~ll~l~~~l~~~~~~~~~~~~~~~~~l~~l~~~la~ltk~~~~~~~~~~~~~~l~~~~~~~~~~~~~~~~   217 (475) |
|  |  |  | ++.|....+++++++++..|.+ ...++++.|+|..+|...+++.+.+.+...-..++++.....+... |
|  | T Consensus | 5 | G~~D~l~~~lvllal~~~~r~~------------~~~ag~~lgla~~~Kl~Pii~~~~l~l~~~~~~~~~~~~~~~~~~~   72 (269) |
|  | T PF05007.14 | 5 | GNADSIVASLVLTTLYLIEKRL------------IACAAVFYGFAVHMKMYPVTYILPIALHLRPERDSDEGLRLARYSF   72 (269) |
|  | T ss\_pred |  | hhHHHHHHHHHHHHHHHHHcCC------------HHHHHHHHHHHHHhchHHHHHHHHHHHHhCccCCCCcchhhhcccH |
|  |
|  |
|  | Q ss\_pred |  | hhHHH--------H-----HHHHHHHHHHHHHHHHHHHHHHhcCCCCCccCCCCCCcCCChHHHHHHHHHHHHHHhHHHh |
|  | Q Q8N394 | 218 | KRKNL--------S-----LFLSISLLIFWGSSLLGARLYWMGNKPPSFSNSDNPAADSDSLLTRTLTFFYLPTKNLWLL   284 (475) |
|  | Q Consensus | 218 | ~~~~~--------~-----~~~~~~~~~~~~~~~~~~~~~~~~~~~~~~~~~~~~~~~~~~~~~~~~~~~~~~~~~~~~~   284 (475) |
|  |  |  | ..+.. . ...............+.......-.+.-.+.....+.....+.+........... |
|  | T Consensus | 73 | ~~~~~~~~~~~~~~~~~~~~~~~~~~~~~l~~~~~~~~g~~~~~~~~~~~~~r~~~~~n~S~~~~~~~l~~~~~------   146 (269) |
|  | T PF05007.14 | 73 | QARLYDFLKRLCSWAVLLFVAIAGLTFLALSFGFYYKYGWEFLEHTYLYHLTRRDIRHNFSPYFYMLYLTAESK------   146 (269) |
|  | T ss\_pred |  | HHHHHHHHHHhccHHHHHHHHHHHHHHHHHHHHHHHHHCHHHHHHHHHHHhccCCCCcCCCHHHHHHHHhccCc------ |
|  |
|  |
|  | Q ss\_pred |  | hCcccccccccccccccccccCCHHHHHHHHHHHHHHHHHHHHhhCcccccccCCCcccCCccccCCccccccccccccc |
|  | Q Q8N394 | 285 | LCPDTLSFDWSMDAVPLLKTVCDWRNLHTVAFYTGLLLLAYYGLKSPSVDRECNGKTVTNGKQNANGHSCLSDVEYQNSE   364 (475) |
|  | Q Consensus | 285 | ~~p~~~~~~~~~~~~~~~~~~~~~~~~~~~~~~~~~~~~~~~~~~~~~~~~~~~~~~~~~~~~~~~~~~~~~~~~~~~~~   364 (475) |
|  |  |  | .............++...+...+. |
|  | T Consensus | 147 | ----------------------~~~~~~~~~~~~~~~~~~~~~~~~----------------------------------   170 (269) |
|  | T PF05007.14 | 147 | ----------------------WSFTLGIAAFLPQFILLSAASFAY----------------------------------   170 (269) |
|  | T ss\_pred |  | ----------------------chhHHHHHHHHHHHHHHHHHHHHc---------------------------------- |
|  |
|  |
|  | Q ss\_pred |  | ccccccchhhcCCcCCcccccCCCCCCchHHhhhHHHHHHHhhhcCccccCChhhHHHHhHHHHHHHHHHHH |
|  | Q Q8N394 | 365 | TKSSFASKVENGIKNDVSQRTQLPSTENIVVLSLSLLIIPFVPATNLFFYVGFVIAERVLYIPSMGFCLLIT   436 (475) |
|  | Q Consensus | 365 | ~~~~~~~~~~~~~~~~~~~~~~~~~~~~~~~~~l~~~~~~~~~~~~~~~~~~~~~~~Ry~~~~~~~~~ll~~   436 (475) |
|  |  |  | ++............++.+. +...++|.....|.+.+... |
|  | T Consensus | 171 | --------------------------~~~~~~~~~~~~~~f~~~~-------~v~~~qY~~W~lpll~l~~~   209 (269) |
|  | T PF05007.14 | 171 | --------------------------YRDLVFCCFLHTSIFVTFN-------KVCTSQYFLWYLCLLPLVMP   209 (269) |
|  | T ss\_pred |  | --------------------------cCcHHHHHHHHHHHHHHhc-------hhhcHHHHHHHHHHHHHHch |
|  |
| --- | | | |
|  | Template alignmentCDD | | |
| 23. | PF14897.7 ; EpsG ; EpsG family | | |
|  | Probability: 88.29%, E-value: 8.4, Score: 33, Aligned cols: 298, Identities: 12%, Similarity: -0.011, | | |
|  |
|  | Q ss\_pred |  | HHHHHHHHHHHHHhhcCC-CCcccchhHHHhchhhCCCCchhHhhh-------cccccccccCCCCcccCChHHHHHHHH |
|  | Q Q8N394 | 4 | ELVSSALGLALYLNTLSA-DFCYDDSRAIKTNQDLLPETPWTHIFY-------NDFWGTLLTHSGSHKSYRPLCTLSFRL   75 (475) |
|  | Q Consensus | 4 | ~lll~~~~~~~~~~~~~~-~~~~De~~~~~~~~~~~~~~~~~~~~~-------~~~~~~~~~~~~~~~~~~Pl~~~l~~~   75 (475) |
|  |  |  | ...++............. +...|...|...-++..+........+ .+. .|++.++... |
|  | T Consensus | 2 | ~~~~~~~~~l~~~~~~R~~~~g~D~~~Y~~~y~~~~~~~~~~~~~~~~~~~~~~~~--------------E~gf~~l~~~   67 (319) |
|  | T PF14897.7 | 2 | WFVSFATIQWIVLSGFRDVTVGADTAQYKALFLQSQTLPLGAFTDRFFEIVFTESE--------------DPGFYLFQRL   67 (319) |
|  | T ss\_pred |  | HHHHHHHHHHHHHHHHcCcCCCCCHHHHHHHHHHhccCCHHHHhhhhhhccCCCCC--------------CHHHHHHHHH |
|  |
|  |
|  | Q ss\_pred |  | HHHHhCCChHHHHHHHHHHHHHHHHHHHHHHHHHhCChHHHHHHHHHHH--HCHHhHHHHHhhhchHHHHHHHHHHHHHH |
|  | Q Q8N394 | 76 | NHAIGGLNPWSYHLVNVLLHAAVTGLFTSFSKILLGDGYWTFMAGLMFA--SHPIHTEAVAGIVGRADVGASLFFLLSLL   153 (475) |
|  | Q Consensus | 76 | ~~~lfG~~~~~~rl~~~l~~~l~~~lly~l~r~l~~~~~~al~aall~a--~~P~~~~~~~~~~~~~~~~~~~f~ll~l~   153 (475) |
|  |  |  | ... +|.+ .+....+.+.++..+.+...++..++.......++++. ....... |+.....++++.++. |
|  | T Consensus | 68 | ~~~-~~~~---~~~~~~~~~~i~~~~~~~~~~~~~~~~~~~~~~~~~~~~~~~~~~~~-------Rq~lA~~~~~l~a~~   136 (319) |
|  | T PF14897.7 | 68 | IQY-VITD---YQVYLVLIAMIFMIPLGYFIYKYSSEPLISFLLFSVLFYEFFAVTGL-------RQTVATALVVLVGYH   136 (319) |
|  | T ss\_pred |  | HHH-hcCC---HHHHHHHHHHHHHHHHHHHHHHhCCchHHHHHHHHHHHHHHHHhHHH-------HHHHHHHHHHHHHHH |
|  |
|  |
|  | Q ss\_pred |  | HHHHHHccCCCCcchHHHHHHHHHHHHHHHHhHhHHHHHHHHHHHHHHHHHcccchhhhcchHhhhHHHHHHHHHHHHHH |
|  | Q Q8N394 | 154 | CYIKHCSTRGYSARTWGWFLGSGLCAGCSMLWKEQGVTVLAVSAVYDVFVFHRLKIKQILPTIYKRKNLSLFLSISLLIF   233 (475) |
|  | Q Consensus | 154 | ~~l~~~~~~~~~~~~~~~~~l~~l~~~la~ltk~~~~~~~~~~~~~~l~~~~~~~~~~~~~~~~~~~~~~~~~~~~~~~~   233 (475) |
|  |  |  | .+. +++ .....+...+|..+|.+++...+. ....+++. ++............. |
|  | T Consensus | 137 | ~~~---~~~---------~~~~~~~~~la~~~H~sali~i~~----~~~~~~~~-----------~~~~~~~~~~~~~~~   189 (319) |
|  | T PF14897.7 | 137 | FVR---ARK---------LGWFLLLVCIAMTIHKSSLIFVPF----YFLANKQL-----------TKAYLMTMFGVIVGL   189 (319) |
|  | T ss\_pred |  | HHH---cCc---------HHHHHHHHHHHHHHHHHHHHHHHH----HHHhhcCC-----------cHHHHHHHHHHHHHH |
|  |
|  |
|  | Q ss\_pred |  | HHHHHHHHHHHHhcCCCCCccCCCCCCcCCChHHHHHHHHHHHHHHhHHHhhCcccccccccccccccccccCCHHHHHH |
|  | Q Q8N394 | 234 | WGSSLLGARLYWMGNKPPSFSNSDNPAADSDSLLTRTLTFFYLPTKNLWLLLCPDTLSFDWSMDAVPLLKTVCDWRNLHT   313 (475) |
|  | Q Consensus | 234 | ~~~~~~~~~~~~~~~~~~~~~~~~~~~~~~~~~~~~~~~~~~~~~~~~~~~~~p~~~~~~~~~~~~~~~~~~~~~~~~~~   313 (475) |
|  |  |  | ....-...........-..+...+..... ..... |
|  | T Consensus | 190 | ~~~~~~i~~~~~~~~~~~~Y~~~~~~~~~-------~~~~~---------------------------------------   223 (319) |
|  | T PF14897.7 | 190 | FVFRNPFFDLLVQVSGYDTYSAMDGAGAV-------NFSLM---------------------------------------   223 (319) |
|  | T ss\_pred |  | HHhcHHHHHHHHHHhchhhhhhccCcchH-------HHHHH--------------------------------------- |
|  |
|  |
|  | Q ss\_pred |  | HHHHHHHHHHHHHHhhCcccccccCCCcccCCccccCCcccccccccccccccccccchhhcCCcCCcccccCCCCCCc- |
|  | Q Q8N394 | 314 | VAFYTGLLLLAYYGLKSPSVDRECNGKTVTNGKQNANGHSCLSDVEYQNSETKSSFASKVENGIKNDVSQRTQLPSTEN-   392 (475) |
|  | Q Consensus | 314 | ~~~~~~~~~~~~~~~~~~~~~~~~~~~~~~~~~~~~~~~~~~~~~~~~~~~~~~~~~~~~~~~~~~~~~~~~~~~~~~~-   392 (475) |
|  |  |  | ..............++ +..+ |
|  | T Consensus | 224 | -~~~~~~~~~~~~~~~~----------------------------------------------------------~~~~~   244 (319) |
|  | T PF14897.7 | 224 | -LLSVLFVALWRKEQIL----------------------------------------------------------ANNPS   244 (319) |
|  | T ss\_pred |  | -HHHHHHHHHHHHHHHh----------------------------------------------------------cCCcc |
|  |
|  |
|  | Q ss\_pred |  | -hHHhhhHHHHHHHhhhcCccccCChhhHHHHhHHHHHHHHHHHHHHHHHHHHHhchHHHHHHHHHHHHHHHH |
|  | Q Q8N394 | 393 | -IVVLSLSLLIIPFVPATNLFFYVGFVIAERVLYIPSMGFCLLITVGARALYVKVQKRFLKSLIFYATATLIV   464 (475) |
|  | Q Consensus | 393 | -~~~~~l~~~~~~~~~~~~~~~~~~~~~~~Ry~~~~~~~~~ll~~~~l~~~~~~~~~~~~~~~~~~~~~~~~~   464 (475) |
|  |  |  | ..............+.... .....|......+... +....+.+..++.+++...........+... |
|  | T Consensus | 245 | ~~~~~~~~~~~~~~~~~~~~-----~~~~~R~~~~~~~~~~-~~~~~~~~~~~~~~~~~~~~~~~~~~~~~~~   311 (319) |
|  | T PF14897.7 | 245 | AIHFFNALLLAACLLPLTFL-----NPSMMRLVQYFSLFLL-LMIPEIVGTFERRERLVVYYSAVMLLGLLFI   311 (319) |
|  | T ss\_pred |  | hHHHHHHHHHHHHHHHHHhc-----ChhHHHHHHHHHHHHH-HHHHHHHhcCChHHHHHHHHHHHHHHHHHHH |
|  |
| --- | | | |
|  | Template alignmentCDD | | |
| 24. | PF16192.6 ; PMT\_4TMC ; C-terminal four TMM region of protein-O-mannosyltransferase | | |
|  | Probability: 81.72%, E-value: 13, Score: 29.29, Aligned cols: 104, Identities: 9%, Similarity: -0.048, | | |
|  |
|  | Q ss\_pred |  | cCCHHHHHHHHHHHHH---HHHHHHHhhCcccccccCCCcccCCccccCCcccccccccccccccccccchhhcCCcCCc |
|  | Q Q8N394 | 305 | VCDWRNLHTVAFYTGL---LLLAYYGLKSPSVDRECNGKTVTNGKQNANGHSCLSDVEYQNSETKSSFASKVENGIKNDV   381 (475) |
|  | Q Consensus | 305 | ~~~~~~~~~~~~~~~~---~~~~~~~~~~~~~~~~~~~~~~~~~~~~~~~~~~~~~~~~~~~~~~~~~~~~~~~~~~~~~   381 (475) |
|  |  |  | .+++..+......+.. .......+++ |
|  | T Consensus | 58 | ~gNp~iw~~~~~~l~~~~~~~~~~~~~~~---------------------------------------------------   86 (198) |
|  | T PF16192.6 | 58 | LGNPFVYWASTASLGLVGLVVVWYILRWQ---------------------------------------------------   86 (198) |
|  | T ss\_pred |  | cCCHHHHHHHHHHHHHHHHHHHHHHHHHh--------------------------------------------------- |
|  |
|  |
|  | Q ss\_pred |  | ccccCCCCCCchH------------HhhhHHHHHHHhhhcCccccCChhhHHHHhHHHHHHHHHHHHHHHHHHHHHhchH |
|  | Q Q8N394 | 382 | SQRTQLPSTENIV------------VLSLSLLIIPFVPATNLFFYVGFVIAERVLYIPSMGFCLLITVGARALYVKVQKR   449 (475) |
|  | Q Consensus | 382 | ~~~~~~~~~~~~~------------~~~l~~~~~~~~~~~~~~~~~~~~~~~Ry~~~~~~~~~ll~~~~l~~~~~~~~~~   449 (475) |
|  |  |  | +..... .+.++..++..+|+ ...++.....|.++++++.+++.+..+..+.++..++ |
|  | T Consensus | 87 | -------r~~~~~~~~~~~~~~~~~~~~~~g~~~~ylP~----~~~~r~~~~~~ylpal~f~~l~~~~~l~~~~~~~~~~   155 (198) |
|  | T PF16192.6 | 87 | -------RGFKDLDSEEVDQIHYAGIYPVLGWFLHYLPF----VIMARVTYVHHYYPALYFAILALGFFVDWLLRNRSQA   155 (198) |
|  | T ss\_pred |  | -------cCCCCCChHHHHHHHHHHHHHHHHHHHHHHHH----HhccccccHHhHHHHHHHHHHHHHHHHHHHHHccchH |
|  |
|  |
|  | Q ss\_pred |  | HHHHHHHHHHHHHHHHHHHHH |
|  | Q Q8N394 | 450 | FLKSLIFYATATLIVFYGLKT   470 (475) |
|  | Q Consensus | 450 | ~~~~~~~~~~~~~~~~~~~~~   470 (475) |
|  |  |  | ..+......+.+.+..+.... |
|  | T Consensus | 156 | ~~~~~~~~~~~~~~~~f~~~~   176 (198) |
|  | T PF16192.6 | 156 | IQGAVYGVLYSVIVGLYITFI   176 (198) |
|  | T ss\_pred |  | HHHHHHHHHHHHHHHHHHHhh |
|  |
| --- | | | |
|  | Template alignmentCDD | | |
| 25. | PF09971.10 ; DUF2206 ; Predicted membrane protein (DUF2206) | | |
|  | Probability: 80.25%, E-value: 22, Score: 31.28, Aligned cols: 222, Identities: 9%, Similarity: 0.009, | | |
|  |
|  | Q ss\_pred |  | HHHHHHHHHHHHhHhHHHHHHHHHHHHHHHHHcccc-------hhhhcchHhhhHH------HHHHHHHHHHHHHHHHHH |
|  | Q Q8N394 | 173 | LGSGLCAGCSMLWKEQGVTVLAVSAVYDVFVFHRLK-------IKQILPTIYKRKN------LSLFLSISLLIFWGSSLL   239 (475) |
|  | Q Consensus | 173 | ~l~~l~~~la~ltk~~~~~~~~~~~~~~l~~~~~~~-------~~~~~~~~~~~~~------~~~~~~~~~~~~~~~~~~   239 (475) |
|  |  |  | ++..++...-.++|+....+...+.....+...-.+ .+. .+. ......++..+.....+. |
|  | T Consensus | 3 | ~L~~i~~~~lv~sH~~t~~~~~~~l~~~~~~~~~~~~~~~~~~~~~-------~~~~~~~~~~~~~~~~~~~v~~~~W~~   75 (390) |
|  | T PF09971.10 | 3 | ILFSVFSCGIIISHYGLTYMVIGLIALSYVLFTFINLVARYINTDK-------VIIPVTPIRLNFLHICIFIFIALSWYI   75 (390) |
|  | T ss\_pred |  | HHHHHHHHHHHHhchHHHHHHHHHHHHHHHHHHHHHHHHHhcCCCC-------CcCCCCcccccHHHHHHHHHHHHHHHH |
|  |
|  |
|  | Q ss\_pred |  | HHHHHHhcCCCCCccCCCCCCcCCChHHHHHHHHHHHHHHhHHHhhCcccccccccccccccccccCCHHHHHHHHHHHH |
|  | Q Q8N394 | 240 | GARLYWMGNKPPSFSNSDNPAADSDSLLTRTLTFFYLPTKNLWLLLCPDTLSFDWSMDAVPLLKTVCDWRNLHTVAFYTG   319 (475) |
|  | Q Consensus | 240 | ~~~~~~~~~~~~~~~~~~~~~~~~~~~~~~~~~~~~~~~~~~~~~~~p~~~~~~~~~~~~~~~~~~~~~~~~~~~~~~~~   319 (475) |
|  |  |  | . ..+....+............+....................................+. |
|  | T Consensus | 76 | ~--------------------~~~~~~~~~~~~~~~~~~~~~~~~~~~~~~~~~~~~~~~~~~~~~~~~~~~~~~~~~~l   135 (390) |
|  | T PF09971.10 | 76 | A--------------------ITSSTAFYSVSSVIYQVISSMFTESLNPTASQGLAIIQKVPVSQMHLLYTYIYYFNQVC   135 (390) |
|  | T ss\_pred |  | H--------------------HhcchHHHHHHHHHHHHHHHHhHHhcCCccchhHHHHhcCCCchHHHHHHHHHHHHHHH |
|  |
|  |
|  | Q ss\_pred |  | HHHHHHHH----hhCcccccccCCCcccCCccccCCcccccccccccccccccccchhhcCCcCCcccccCCCCCCchHH |
|  | Q Q8N394 | 320 | LLLLAYYG----LKSPSVDRECNGKTVTNGKQNANGHSCLSDVEYQNSETKSSFASKVENGIKNDVSQRTQLPSTENIVV   395 (475) |
|  | Q Consensus | 320 | ~~~~~~~~----~~~~~~~~~~~~~~~~~~~~~~~~~~~~~~~~~~~~~~~~~~~~~~~~~~~~~~~~~~~~~~~~~~~~   395 (475) |
|  |  |  | .+.++... .+++++.+. ..... |
|  | T Consensus | 136 | ~~iG~~~~~~~~~~~~~~~~~------------------------------------------------------~~~~~   161 (390) |
|  | T PF09971.10 | 136 | IVLGLLYLSYKTFARKNMYNY------------------------------------------------------SIMQL   161 (390) |
|  | T ss\_pred |  | HHHHHHHHHHHHHhccCccCC------------------------------------------------------CHHHH |
|  |
|  |
|  | Q ss\_pred |  | hhhHHHHHHHhhhcCccccCChhhHHHHhHHHHHHHHHHHHHHHHHHHHHhchH---------------HHHHHHHHHHH |
|  | Q Q8N394 | 396 | LSLSLLIIPFVPATNLFFYVGFVIAERVLYIPSMGFCLLITVGARALYVKVQKR---------------FLKSLIFYATA   460 (475) |
|  | Q Consensus | 396 | ~~l~~~~~~~~~~~~~~~~~~~~~~~Ry~~~~~~~~~ll~~~~l~~~~~~~~~~---------------~~~~~~~~~~~   460 (475) |
|  |  |  | ......++.+................|......++++++++.++..+.+..++. ..+...+.+++ |
|  | T Consensus | 162 | ~~~~~~~~~~~~~~~~p~~~~~~~~~R~~~~~~~~~~~~a~~g~~~l~~~~~~~~~~~~~~~~~~~~~~~~~~~~~~lv~   241 (390) |
|  | T PF09971.10 | 162 | IMCGVAVMVLVGSIVLPYFASALNTTRIYHIMQFFVSPVYIIGFIFALESIPKVYARIVKSPFRSNLSFTYGIISLFLCV   241 (390) |
|  | T ss\_pred |  | HHHHHHHHHHHHHHHHHHHHHhcChHHHHHHHHHHHHHHHHHHHHHHHHhhHHHHHHhhcCccccccchHHHHHHHHHHH |
|  |
|  |
|  | Q ss\_pred |  | HHHHHHHHHHHHhcC |
|  | Q Q8N394 | 461 | TLIVFYGLKTAIRNG   475 (475) |
|  | Q Consensus | 461 | ~~~~~~~~~~~~~~~   475 (475) |
|  |  |  | ++++..+.......+ |
|  | T Consensus | 242 | ~~~~~sg~~~~~~~~   256 (390) |
|  | T PF09971.10 | 242 | YLLFNSGVIFQILND   256 (390) |
|  | T ss\_pred |  | HHHHHhcHHHHHhCC |
|  |
| --- | | | |
|  | Template alignmentCDD | | |
| 26. | PF10060.10 ; DUF2298 ; Uncharacterized membrane protein (DUF2298) | | |
|  | Probability: 77.17%, E-value: 38, Score: 32.13, Aligned cols: 345, Identities: 11%, Similarity: -0.012, | | |
|  |
|  | Q ss\_pred |  | HHHHHHHHHHHHHhhcCCCCc-ccchhHHHhchhhCCCCchhHhhhcccccccccCCCCcccCChHHHHHHHHHHHHhC- |
|  | Q Q8N394 | 4 | ELVSSALGLALYLNTLSADFC-YDDSRAIKTNQDLLPETPWTHIFYNDFWGTLLTHSGSHKSYRPLCTLSFRLNHAIGG-   81 (475) |
|  | Q Consensus | 4 | ~lll~~~~~~~~~~~~~~~~~-~De~~~~~~~~~~~~~~~~~~~~~~~~~~~~~~~~~~~~~~~Pl~~~l~~~~~~lfG-   81 (475) |
|  |  |  | ++.+++..........+.+.+ .|...+.....+....+.++...+-- .+....|..+.+++.+...++.| |
|  | T Consensus | 60 | ~vf~~~f~~~~~~r~~~p~i~~~Ek~md~~~i~s~~~~~~~Pp~dPw~--------aG~~l~Yyyfg~~~~A~l~~l~gi   131 (597) |
|  | T PF10060.10 | 60 | LLFLGAFAAWAWVRAHDPAADHTEQPMDLMFMHSIRASLTYPPHDAWL--------AGYPISYYYFGYWLMNMVGLMAGQ   131 (597) |
|  | T ss\_pred |  | HHHHHHHHHHHHHHHhCCCCCCCCChhHHHHHHHHHhcCCCCccchhh--------cCCCccccHHHHHHHHHHHHHhCC |
|  |
|  |
|  | Q ss\_pred |  | CChHHHHHHHHHHHHHHHHHHHHHHHHHhCChH--------HHHHHHHHHHHCHHhHHHHHhhhchH------------- |
|  | Q Q8N394 | 82 | LNPWSYHLVNVLLHAAVTGLFTSFSKILLGDGY--------WTFMAGLMFASHPIHTEAVAGIVGRA-------------   140 (475) |
|  | Q Consensus | 82 | ~~~~~~rl~~~l~~~l~~~lly~l~r~l~~~~~--------~al~aall~a~~P~~~~~~~~~~~~~-------------   140 (475) |
|  |  |  | ....++++....+..+.+..+|.+++++.+++. .+++++++..+............... |
|  | T Consensus | 132 | ~~~~~~nl~~~~~~al~~~~~~~l~~~l~~~~~~~~~~~~~~g~la~~l~~~~gnl~~~~~~~~~~~~~~~~~~~~~~~~   211 (597) |
|  | T PF10060.10 | 132 | SAAVAYNLSQAVWFGLLLSGAFGIGYNLVAAAGRRFVAALGGGWVATLLVGLSSNLQGLLEWLHANGVDISWLAAWLQVR   211 (597) |
|  | T ss\_pred |  | CHHHHHHHHHHHHHHHHHHHHHHHHHHHHHhccccccHHHHHHHHHHHHHHHhcccHHHHHHHHhCCCCchhHHHHhhhc |
|  |
|  |
|  | Q ss\_pred |  | -------------------------------------------------------HHHHHHHHHHHHHHHHHHHccCCCC |
|  | Q Q8N394 | 141 | -------------------------------------------------------DVGASLFFLLSLLCYIKHCSTRGYS   165 (475) |
|  | Q Consensus | 141 | -------------------------------------------------------~~~~~~f~ll~l~~~l~~~~~~~~~   165 (475) |
|  |  |  | +.+...+.++++.+.+...+++++. |
|  | T Consensus | 212 | ~~~~~~~~~~~~~~~~~w~~w~ssRvI~~~~~~~~~~~tI~EFP~fSfl~gDLHpH~~alPf~ll~l~l~~~~~~~~~~~   291 (597) |
|  | T PF10060.10 | 212 | GFPENAEVTRQWFISYGWWWWRSSRVLADVSLRGDHIEVIDEFPAFSYILGDNHPHVAAMPFAMLAVAAALVIFLQNSSS   291 (597) |
|  | T ss\_pred |  | CCCcchhhhccccccCccccccceeeeecccCCCCCCcccccCchHHHhcCCCChhhhHHHHHHHHHHHHHHHHhcCCCC |
|  |
|  |
|  | Q ss\_pred |  | cchHH----------------HHHHHHHHHHHHHHhHhHHHHHHHHHHHHHHHHHcccc------------hhhhcchHh |
|  | Q Q8N394 | 166 | ARTWG----------------WFLGSGLCAGCSMLWKEQGVTVLAVSAVYDVFVFHRLK------------IKQILPTIY   217 (475) |
|  | Q Consensus | 166 | ~~~~~----------------~~~l~~l~~~la~ltk~~~~~~~~~~~~~~l~~~~~~~------------~~~~~~~~~   217 (475) |
|  |  |  | ..+.+ ..++.+++.|....++.--......+.+........+. ... |
|  | T Consensus | 292 | ~~~~~~~~~~~~~~~~~~~~~~~ll~gll~G~l~~~NtWD~p~~~~l~~~~~~~~~~~~~~~~~~~~~~~~~~~------   365 (597) |
|  | T PF10060.10 | 292 | NFSSESRAKFNFAPLFPLGWGGFLLVAVITGSLLFLNTWDYPPYWLLTTFSIAVGVVGGVVRVKNFLPLQFLPL------   365 (597) |
|  | T ss\_pred |  | CCCchhhhccCCCCccccchHHHHHHHHHHHHHHHHccCCHHHHHHHHHHHHHHHHhccccccccccccccccc------ |
|  |
|  |
|  | Q ss\_pred |  | hhHHHHHHHHHHHHHHHHHHHHHHHHHHhcCCCCCccCCCCCCcCCChHHHHHHHHHHHHHHhHHHhhCccccccccccc |
|  | Q Q8N394 | 218 | KRKNLSLFLSISLLIFWGSSLLGARLYWMGNKPPSFSNSDNPAADSDSLLTRTLTFFYLPTKNLWLLLCPDTLSFDWSMD   297 (475) |
|  | Q Consensus | 218 | ~~~~~~~~~~~~~~~~~~~~~~~~~~~~~~~~~~~~~~~~~~~~~~~~~~~~~~~~~~~~~~~~~~~~~p~~~~~~~~~~   297 (475) |
|  |  |  | .+.........+.+..........+......+...-..... ..+...+....+. |
|  | T Consensus | 366 | -~~~~~~~~~~~~~~~~~a~ll~lPF~l~f~~~~~gi~~~~~--~~T~l~~~l~i~G-----------------------   419 (597) |
|  | T PF10060.10 | 366 | -LPPLLQTTIAGLALFVAALLLYLPYLLTAQSQVGGLIPNLF--HPTRFSQYVAMFA-----------------------   419 (597) |
|  | T ss\_pred |  | -hHHHHHHHHHHHHHHHHHHHHHHHHHHhccccCCCCcccCC--CCCCHHHHHHHHH----------------------- |
|  |
|  |
|  | Q ss\_pred |  | ccccccccCCHHHHHHHHHHHHHHHHHHHHhhCcccccccCCCcccCCccccCCcccccccccccccccccccchhhcCC |
|  | Q Q8N394 | 298 | AVPLLKTVCDWRNLHTVAFYTGLLLLAYYGLKSPSVDRECNGKTVTNGKQNANGHSCLSDVEYQNSETKSSFASKVENGI   377 (475) |
|  | Q Consensus | 298 | ~~~~~~~~~~~~~~~~~~~~~~~~~~~~~~~~~~~~~~~~~~~~~~~~~~~~~~~~~~~~~~~~~~~~~~~~~~~~~~~~   377 (475) |
|  |  |  | +.++.++.......++ |
|  | T Consensus | 420 | -----------------lfl~l~~~~l~~~~~~-----------------------------------------------   435 (597) |
|  | T PF10060.10 | 420 | -----------------TALLTLTALLTFGWSV-----------------------------------------------   435 (597) |
|  | T ss\_pred |  | -----------------HHHHHHHHHHHHHhhc----------------------------------------------- |
|  |
|  |
|  | Q ss\_pred |  | cCCcccccCCCCCCchHHhhhHHHHHHHhhhcC------------------------------ccccCChhhHHHHhHHH |
|  | Q Q8N394 | 378 | KNDVSQRTQLPSTENIVVLSLSLLIIPFVPATN------------------------------LFFYVGFVIAERVLYIP   427 (475) |
|  | Q Consensus | 378 | ~~~~~~~~~~~~~~~~~~~~l~~~~~~~~~~~~------------------------------~~~~~~~~~~~Ry~~~~   427 (475) |
|  |  |  | .+++..............+... . .....|....+ |
|  | T Consensus | 436 | -----------~~~~~~~~~~~~~~~~~~~~~~~~~~~~~~~~~~~~~~~~~~~~~~~~~~~~~-----~~~~~~~~~~~   499 (597) |
|  | T PF10060.10 | 436 | -----------FRPRLKVVMICLALTLGTPALLLTFIAWVATGTEEGRASLGNVALPDGASSYL-----PFIVERWTAQP   499 (597) |
|  | T ss\_pred |  | -----------ccchHHHHHHHHHHHHHHHHHHHHHHHHHhcCChhhhhccccccCCCCcccch-----hHHHHHhccch |
|  |
|  |
|  | Q ss\_pred |  | HHHHHHHHHHHHHHHHHHhchH-----------------------------HHHHHHHHHHHHHHHHHHH |
|  | Q Q8N394 | 428 | SMGFCLLITVGARALYVKVQKR-----------------------------FLKSLIFYATATLIVFYGL   468 (475) |
|  | Q Consensus | 428 | ~~~~~ll~~~~l~~~~~~~~~~-----------------------------~~~~~~~~~~~~~~~~~~~   468 (475) |
|  |  |  | ...+.++.........-+.+++ ........++.+.+.+... |
|  | T Consensus | 500 | ~~~~ll~~l~~~~~~~~~~~~~~~~~~~~~~~~~~~~~~~~~~~~~~~~~~~~~~f~l~L~~~gl~Lil~   569 (597) |
|  | T PF10060.10 | 500 | FTFLIVGAMTAVALALLWTGIQHMVGAKNFLPQHFSPQQGALDSTPGVAAPTPLLFVLALAVIGLGLTFT   569 (597) |
|  | T ss\_pred |  | HHHHHHHHHHHHHHHHHHhhhhhhccccccCcccCCCCCCCcCCCCCCCCCHHHHHHHHHHHHHHHHHHH |
|  |
| --- | | | |
|  | Template alignmentCDD | | |
| 27. | PF08409.12 ; DUF1736 ; Domain of unknown function (DUF1736) | | |
|  | Probability: 50.61%, E-value: 28, Score: 21.15, Aligned cols: 73, Identities: 55%, Similarity: 0.907, | | |
|  |
|  | Q ss\_pred |  | CCCCCccCCCCCCcCCChHHHHHHHHHHHHHHhHHHhhCcccccccccccccccccccCCHHHHHHHHHHHHH |
|  | Q Q8N394 | 248 | NKPPSFSNSDNPAADSDSLLTRTLTFFYLPTKNLWLLLCPDTLSFDWSMDAVPLLKTVCDWRNLHTVAFYTGL   320 (475) |
|  | Q Consensus | 248 | ~~~~~~~~~~~~~~~~~~~~~~~~~~~~~~~~~~~~~~~p~~~~~~~~~~~~~~~~~~~~~~~~~~~~~~~~~   320 (475) |
|  |  |  | ...+.+...+++.....+...+..........++....+|.....++.++..+...+..+........+..+. |
|  | T Consensus | 1 | ~~~~~~~~~~n~~~~~~~~~~~~~~~~~~~~~yl~ll~~P~~l~~~~~~~~~~~~~~~~~~~~~~~~~~~~~~   73 (74) |
|  | T PF08409.12 | 1 | SGPPVFAAADNPTAKSPSLVTRTLTFLYLPAENFRLLVYPRRLSFDWSMDAIAPVTSVYDPRNALSVALYVAL   73 (74) |
|  | T ss\_pred |  | CCCCCCcccCCchhcCCCHHHHHHHHHHHHHHHHHHHHCccccCCCCCcccCCCCCCCCCHHHHHHHHHHHHh |
|  |
| --- | | | |
|  | Template alignmentCDD | | |
| 28. | PF15102.7 ; TMEM154 ; TMEM154 protein family | | |
|  | Probability: 20.2%, E-value: 110, Score: 22.27, Aligned cols: 52, Identities: 8%, Similarity: -0.047, | | |
|  |
|  | Q ss\_pred |  | HHHHHHHHHHHHHHHHHHHHhhCcccccccCCCcccC-----CccccCCcccccccc |
|  | Q Q8N394 | 308 | WRNLHTVAFYTGLLLLAYYGLKSPSVDRECNGKTVTN-----GKQNANGHSCLSDVE   359 (475) |
|  | Q Consensus | 308 | ~~~~~~~~~~~~~~~~~~~~~~~~~~~~~~~~~~~~~-----~~~~~~~~~~~~~~~   359 (475) |
|  |  |  | ......+++++.+++++.+..||++++++.+...... ...+++.+.+.+..+ |
|  | T Consensus | 57 | lIpvvLLlLLlliIi~lV~~~rRkr~k~~~~sd~~ld~lqt~~~~~e~~~~PmFEeD   113 (140) |
|  | T PF15102.7 | 57 | LIPLILFALLFLSVIILVTRYRRKRAKQEPYSPGSQSALQTYELGSENIKVPIFEED   113 (140) |
|  | T ss\_pred |  | HHHHHHHHHHHHHHHHHHHHHHhhcCCCCCCCCCccchhccccCCCCcCCCCcccCC |
|  |

---

If you use HHpred on our Toolkit for your research, please cite as appropriate:

A Completely Reimplemented MPI Bioinformatics Toolkit
with a New HHpred Server at its Core.  
Zimmermann L, Stephens A, Nam SZ, Rau D,
Kübler J, Lozajic M, Gabler F, Söding J, Lupas AN, Alva V.
J Mol Biol. 2018 Jul 20. S0022-2836(17)30587-9.

  

Protein homology detection by HMM-HMM comparison.  
Söding J. Bioinformatics. 2005 Apr 1;21(7):951-60.  
  
Fast and accurate automatic structure prediction with HHpred.  
Hildebrand A, Remmert M, Biegert A, Söding J. Proteins. 2009;77 Suppl 9:128-32.  
  
Automatic Prediction of Protein 3D Structures by Probabilistic Multi-template Homology Modeling.  
Meier A, Söding J. PLoS Comput Biol. 2015 Oct 23;11(10):e1004343.

Download

---

If you use HHpred on our Toolkit for your research, please cite as appropriate:

A Completely Reimplemented MPI Bioinformatics Toolkit
with a New HHpred Server at its Core.  
Zimmermann L, Stephens A, Nam SZ, Rau D,
Kübler J, Lozajic M, Gabler F, Söding J, Lupas AN, Alva V.
J Mol Biol. 2018 Jul 20. S0022-2836(17)30587-9.

  

Protein homology detection by HMM-HMM comparison.  
Söding J. Bioinformatics. 2005 Apr 1;21(7):951-60.  
  
Fast and accurate automatic structure prediction with HHpred.  
Hildebrand A, Remmert M, Biegert A, Söding J. Proteins. 2009;77 Suppl 9:128-32.  
  
Automatic Prediction of Protein 3D Structures by Probabilistic Multi-template Homology Modeling.  
Meier A, Söding J. PLoS Comput Biol. 2015 Oct 23;11(10):e1004343.

Loading...

---

If you use HHpred on our Toolkit for your research, please cite as appropriate:

A Completely Reimplemented MPI Bioinformatics Toolkit
with a New HHpred Server at its Core.  
Zimmermann L, Stephens A, Nam SZ, Rau D,
Kübler J, Lozajic M, Gabler F, Söding J, Lupas AN, Alva V.
J Mol Biol. 2018 Jul 20. S0022-2836(17)30587-9.

  

Protein homology detection by HMM-HMM comparison.  
Söding J. Bioinformatics. 2005 Apr 1;21(7):951-60.  
  
Fast and accurate automatic structure prediction with HHpred.  
Hildebrand A, Remmert M, Biegert A, Söding J. Proteins. 2009;77 Suppl 9:128-32.  
  
Automatic Prediction of Protein 3D Structures by Probabilistic Multi-template Homology Modeling.  
Meier A, Söding J. PLoS Comput Biol. 2015 Oct 23;11(10):e1004343.

Loading hits...

---

If you use HHpred on our Toolkit for your research, please cite as appropriate:

A Completely Reimplemented MPI Bioinformatics Toolkit
with a New HHpred Server at its Core.  
Zimmermann L, Stephens A, Nam SZ, Rau D,
Kübler J, Lozajic M, Gabler F, Söding J, Lupas AN, Alva V.
J Mol Biol. 2018 Jul 20. S0022-2836(17)30587-9.

  

Protein homology detection by HMM-HMM comparison.  
Söding J. Bioinformatics. 2005 Apr 1;21(7):951-60.  
  
Fast and accurate automatic structure prediction with HHpred.  
Hildebrand A, Remmert M, Biegert A, Söding J. Proteins. 2009;77 Suppl 9:128-32.  
  
Automatic Prediction of Protein 3D Structures by Probabilistic Multi-template Homology Modeling.  
Meier A, Söding J. PLoS Comput Biol. 2015 Oct 23;11(10):e1004343.

Loading hits...

---

If you use HHpred on our Toolkit for your research, please cite as appropriate:

A Completely Reimplemented MPI Bioinformatics Toolkit
with a New HHpred Server at its Core.  
Zimmermann L, Stephens A, Nam SZ, Rau D,
Kübler J, Lozajic M, Gabler F, Söding J, Lupas AN, Alva V.
J Mol Biol. 2018 Jul 20. S0022-2836(17)30587-9.

  

Protein homology detection by HMM-HMM comparison.  
Söding J. Bioinformatics. 2005 Apr 1;21(7):951-60.  
  
Fast and accurate automatic structure prediction with HHpred.  
Hildebrand A, Remmert M, Biegert A, Söding J. Proteins. 2009;77 Suppl 9:128-32.  
  
Automatic Prediction of Protein 3D Structures by Probabilistic Multi-template Homology Modeling.  
Meier A, Söding J. PLoS Comput Biol. 2015 Oct 23;11(10):e1004343.

- Help
- FAQ
- Privacy Policy
- Imprint
- Contact Us
- Cite Us
- Recent Updates

© 2008-2020, Dept. of Protein Evolution, Max Planck Institute for Developmental Biology, Tübingen

Template 3D Structure: 
×

Loading...
