# Supplementary material for: Conserved sequence motifs in human TMTC1, TMTC2, TMTC3, and TMTC4, new O-mannosyltransferases from the GT-C/PMT clan, are rationalized as ligand binding sites
Source: Biol Direct. 2021 Jan 12;16:4. doi: 10.1186/s13062-021-00291-w (PMC7801869; doi:10.1186/s13062-021-00291-w)
Supplement: Supplementary file 3 — Additional file 3. HHPred outputs when searching TMTCs against Pfam or PDB structures. The compressed library file AF3-2020-06-HHPred-TMTCs.zip contains the outputs when running the four human TMTC sequences as input of HHPred against PDB sequences and against Pfam domains (as of 23rd of June 2020). [file 13062_2021_291_MOESM3_ESM.zip › AF3-2020-06-HHPred-TMTCs/HHpred_TMTC3_PDB.html]

HHpred | Bioinformatics Toolkit          **We're sorry but the Toolkit doesn't work properly without JavaScript enabled. Please enable it to continue.**

Sign In

- Search
- Alignment
- Sequence Analysis
- 2ary Structure
- 3ary Structure
- Classification
- Utils

- HHblits
- HHpred
- HMMER
- PatternSearch
- ProtBLAST/PSI-BLAST

Nothing found.

###### Tools

###### Jobs

ID

Date

Tool

8776670HHPR3084060HHPR1780993HHPR5863267HHPR5407837HHPR8665047HHPR2161064HHPR

# HHpred

Job ID: 8776670,Created: 27 minutes ago

- Input
- Parameters
- Results
- Raw Output
- Probability Plot
- Query Template MSA
- Query MSA

>sp|Q6ZXV5|TMTC3\_HUMAN 1..426
MANINLKEITLIVGVVTACYWNSLFCGFVFDDVSAILDNKDLHPSTPLKTLFQNDFWGTP
MSEERSHKSYRPLTVLTFRLNYLLSELKPMSYHLLNMIFHAVVSVIFLKVCKLFLDNKSS
VIASLLFAVHPIHTEAVTGVVGRAELLSSIFFLAAFLSYTRSKGPDNSIIWTPIALTVFL
VAVATLCKEQGITVVGICCVYEVFIAQGYTLPLLCTTAGQFLRGKGSIPFSMLQTLVKLI
VLMFSTLLLVVIRVQVIQSQLPVFTRFDNPAAVSPTPTRQLTFNYLLPVNAWLLLNPSEL
CCDWTMGTIPLIESLLDIRNLATFTFFCFLGMLGVFSIRYSGDSSKTVLMALCLMALPFI
PASNLFFPVGFVVAERVLYVPSMGFCILVAHGWQKISTKSVFKKLSWICLSMVILTHSLK
TFHRNW

Paste ExampleUpload File

Protein FASTA

Align two sequences/MSAs

Select structural/domain databases

PDB\_mmCIF70\_29\_May

- PDB\_mmCIF70\_29\_May (default)
- PDB\_mmCIF30\_29\_May
- SCOPe70\_2.07
- ECOD\_ECOD\_F70\_20200207
- COG\_KOG\_v1.0
- Pfam-A\_v33.1
- NCBI\_Conserved\_Domains(CD)\_v3.18
- SMART\_v6.0
- TIGRFAMs\_v15.0
- PRK\_v6.9
- No elements found. Consider changing the search query.
- List is empty.

Select proteomes

Select options

- Euk\_Arabidopsis\_thaliana\_TAIR10\_20\_Jun\_2017
- Euk\_Bombyx\_mori\_p50T\_Dazao\_06\_May\_2019
- Euk\_Brachypodium\_distachyon\_23\_Aug\_2017
- Euk\_Caenorhabditis\_elegans\_18\_Jul\_2017
- Euk\_Capsaspora\_owczarzaki\_ATCC\_30864\_23\_Mar\_2020
- Euk\_Chaetomium\_thermophilum\_29\_Jun\_2017
- Euk\_Chlamydomonas\_reinhardtii\_27\_Jul\_2017
- Euk\_Entamoeba\_histolytica\_HM1\_IMSS\_22\_Mar\_2017
- Euk\_Dictyostelium\_discoideum\_AX4\_19\_Sep\_2017
- Euk\_Drosophila\_melanogaster\_19\_Jul\_2017
- Euk\_Giardia\_lamblia\_ATCC\_50803\_31\_Aug\_2017
- Euk\_Homo\_sapiens\_04\_Jul\_2017
- Euk\_Physcomitrella\_patens\_28\_Aug\_2017
- Euk\_Plasmodium\_falciparum\_3D7\_7\_Jun\_2017
- Euk\_Saccharomyces\_cerevisiae\_S288c\_11\_Mar\_2017
- Euk\_Schizosaccharomyces\_pombe\_19\_Sep\_2017
- Euk\_Solanum\_lycopersicum\_28\_Jul\_2019
- Euk\_Tetrahymena\_thermophila\_SB210\_22\_Aug\_2017
- Euk\_Toxoplasma\_gondii\_ME49\_10\_May\_2018
- Euk\_Trichomonas\_vaginalis\_G3\_21\_Nov\_2018
- Euk\_Trypanosoma\_brucei\_gambiense\_DAL972\_28\_Mar\_2017
- Euk\_Ustilago\_maydis\_521\_29\_May\_2017
- Euk\_Paramecium\_tetraurelia\_9\_Dec\_2018
- Arc\_Archaeoglobus\_fulgidus\_DSM\_4304\_5\_Dec\_2017
- Arc\_Halobacterium\_jilantaiense\_5\_Dec\_2017
- Arc\_Lokiarchaeum\_sp\_GC14\_75\_31\_Oct\_2018
- Arc\_Methanocaldococcus\_jannaschii\_DSM\_2661\_5\_Dec\_2017
- Arc\_Methanosarcina\_mazei\_S\_6\_17\_Mar\_2017
- Arc\_Methanothermus\_fervidus\_DSM\_2088\_5\_Dec\_2017
- Arc\_Pyrococcus\_horikoshii\_OT3\_5\_Dec\_2017
- Arc\_Sulfolobus\_solfataricus\_5\_Dec\_2017
- Arc\_Thermoplasma\_acidophilum\_DSM\_1728\_7\_Dec\_2017
- Bac\_Acinetobacter\_baumannii\_29\_Mar\_2018
- Bac\_Aquifex\_aeolicus\_VF5\_19\_Sep\_2017
- Bac\_Bacillus\_subtilis\_subsp\_subtilis\_str168\_19\_Mar\_2017
- Bac\_Bacteriovorax\_sp\_DB6\_IX\_1\_Jun\_2018
- Bac\_Bdellovibrio\_bacteriovorus\_HD100\_1\_Jun\_2018
- Bac\_Christensenella\_minuta\_2\_Apr\_2019
- Bac\_Deinococcus\_radiodurans\_R1\_19\_Sep\_2017
- Bac\_Enterococcus\_faecalis\_13\_SD\_W\_01\_1\_Jun\_2018
- Bac\_Escherichia\_coli\_K12\_07\_Mar\_2017
- Bac\_Fischerella\_muscicola\_PCC\_7414\_24\_Sep\_2017
- Bac\_Frankia\_alni\_ACN14a\_24\_Sep\_2017
- Bac\_Helicobacter\_pylori\_26695\_1\_Jun\_2018
- Bac\_Leptospira\_interrogans\_serovar\_Lai\_str56601\_1\_Jun\_2018
- Bac\_Mycobacterium\_tuberculosis\_H37Rv\_27\_May\_2017
- Bac\_Neisseria\_gonorrhoeae\_FA\_1090\_1\_Jun\_2018
- Bac\_Neisseria\_meningitidis\_MC58\_9\_Jun\_2017
- Bac\_Nostoc\_punctiforme\_PCC\_73102\_18\_Mar\_2017
- Bac\_Phycisphaerae\_bacterium\_L21\_RPulD3\_1\_Jun\_2018
- Bac\_Plesiocystis\_pacifica\_SIR1\_1\_Jun\_2018
- Bac\_Pseudomonas\_aeruginosa\_PAO1\_5\_Jun\_2017
- Bac\_Salmonella\_ent\_ser\_Typhi\_CT18\_22\_Nov\_2018
- Bac\_Staphylococcus\_aureus\_subsp\_aureus\_NCTC\_8325\_13\_Jun\_2017
- Bac\_Streptomyces\_scabiei\_87.22\_24\_Sep\_2017
- Bac\_Synechocystis\_sp\_PCC\_6803\_6\_Jun\_2017
- Bac\_Tenacibaculum\_dicentrarchi\_27\_Nov\_2017
- Bac\_Tenacibaculum\_maritimum\_NBRC\_15946\_27\_Nov\_2017
- Bac\_Thermus\_aquaticus\_Y51MC23\_24\_Sep\_2017
- Bac\_Thermus\_thermophilus\_HB8\_19\_Sep\_2017
- Bac\_Waddlia\_chondrophila\_WSU\_86\_1044\_1\_Jun\_2018
- Bac\_Yersinia\_pestis\_CO92\_10\_Apr\_2017
- Vir\_SARS-CoV-2\_31\_Mar\_2020
- No elements found. Consider changing the search query.
- List is empty.

Resubmit

MSA generation method

HHblits=>UniRef30

- HHblits=>UniRef30 (default)
- PSI-BLAST=>nr70
- No elements found. Consider changing the search query.
- List is empty.

Maximal no. of MSA generation steps

3

- 0
- 1
- 2
- 3 (default)
- 4
- 5
- 8
- No elements found. Consider changing the search query.
- List is empty.

E-value incl. threshold for MSA generation

1e-3

- 0.1
- 0.05
- 0.02
- 0.01
- 1e-3 (default)
- 1e-6
- 1e-8
- 1e-10
- 1e-15
- 1e-20
- 1e-30
- 1e-40
- 1e-50
- No elements found. Consider changing the search query.
- List is empty.

Min. seq. identity of MSA hits with query (%)

0

- 0 (default)
- 10
- 20
- 30
- 40
- 50
- 60
- 70
- 75
- 80
- 85
- 90
- 95
- 100
- No elements found. Consider changing the search query.
- List is empty.

Min. coverage of MSA hits (%)

20

- 10
- 20 (default)
- 30
- 40
- 50
- 60
- 70
- 80
- 90
- 100
- No elements found. Consider changing the search query.
- List is empty.

Secondary structure scoring

during\_alignment

- none
- after\_alignment
- during\_alignment (default)
- after\_alignment\_pred\_vs\_pred
- during\_alignment\_pred\_vs\_pred
- No elements found. Consider changing the search query.
- List is empty.

Alignment Mode:Realign with MAC

local:norealign

- local:norealign (default)
- local:realign
- global:realign
- No elements found. Consider changing the search query.
- List is empty.

MAC realignment threshold

0.3

- 0.0
- 0.01
- 0.1
- 0.2
- 0.3 (default)
- 0.4
- 0.5
- 0.6
- 0.7
- 0.8
- 0.9
- 0.95
- No elements found. Consider changing the search query.
- List is empty.

No. of target sequences (up to 10000)

250

- 250 (default)
- 500
- 1000
- 2000
- 3000
- 4000
- 5000
- 6000
- 7000
- 8000
- 9000
- 10000
- No elements found. Consider changing the search query.
- List is empty.

Min. probability in hit list (> 10%)

20

- 10
- 20 (default)
- 30
- 40
- 50
- 60
- 70
- 75
- 80
- 85
- 90
- 95
- 100
- No elements found. Consider changing the search query.
- List is empty.

Resubmit

VisHitsAln
Select AllForwardForward Query A3MModel using selectionDownload HHRColor SeqsWrap Seqs

Number of Hits: **17**

Detected sequence features:
**◾Transmembrane segment(s)**

#### Visualization

Resubmit Section

2

424

Prob=99.5%
E=1.8E-11 6SNI\_X Dolichyl pyrophosphate Man9GlcNAc2
alpha-1,3-glucosyltransferase (E.C.2.4.1.267); Glycosyltransferase,
Glucosyltransferase, GT-C, N-Glycosylation, MEMBRANE; HET: PTY,
Y01;{Saccharomyces cerevisiae}; Related PDB entries: 6SNH\_X

#### Hitlist

Show102550100AllEntries

Search:

| Nr (Click to sort Ascending) | Hit (Click to sort Ascending) | Name (Click to sort Ascending) | Probability (Click to sort Ascending) | E-value (Click to sort Ascending) | SS (Click to sort Ascending) | Cols (Click to sort Ascending) | Target Length (Click to sort Ascending) |
| --- | --- | --- | --- | --- | --- | --- | --- |
| 1 | 5EZM\_A | 4-amino-4-deoxy-L-arabinose (L-Ara4N) transferase; membrane protein, lipid glycosyltransferase, zinc; HET: PC, DSL, PO4, MPG, EPE; 2.7A {Cupriavidus metallidurans (strain ATCC 43123 / DSM 2839 / NBRC 102507 / CH34)}; Related PDB entries: 5F15\_A ; Related PDB entries: 5F15\_A ; Related PDB entries: 5F15\_A | 99.91 | 1.3e-21 | 29.8 | 365 | 578 |
| 2 | 6S7T\_A | Dolichyl-diphosphooligosaccharide--protein glycosyltransferase subunit STT3B (E.C.2.4.99.18); N-glycosylation, Oligosaccharyltransferase, OSTB, TRANSFERASE; HET: 0K3, KZB, NAG, EGY, MAN, BMA; 3.5A {Homo sapiens} | 99.83 | 3.1e-17 | 32.7 | 372 | 826 |
| 3 | 3WAJ\_A | Transmembrane oligosaccharyl transferase (E.C.2.4.1.119); oligosaccharyltransferase, N-glycosylation, Archaeoglobus fulgidus, GT-C; 2.501A {Archaeoglobus fulgidus}; Related PDB entries: 5GMY\_A 3WAK\_A; Related PDB entries: 5GMY\_A 3WAK\_A; Related PDB entries: 5GMY\_A 3WAK\_A | 99.82 | 1.8e-17 | 29.4 | 365 | 875 |
| 4 | 6EZN\_F | Dolichyl-diphosphooligosaccharide--protein glycosyltransferase subunit 1 (E.C.2.4.99.18); OST complex, oligosaccharyltransferase, N-linked glycosylation; HET: PTY, BMA, CPL, MAN, NAG;{Saccharomyces cerevisiae (strain ATCC 204508 / S288c)}; Related PDB entries: 6C26\_A; Related PDB entries: 6C26\_A; Related PDB entries: 6C26\_A | 99.82 | 5.1e-17 | 31.5 | 365 | 718 |
| 5 | 6S7O\_A | Dolichyl-diphosphooligosaccharide--protein glycosyltransferase subunit STT3A (E.C.2.4.99.18); N-glycosylation, Oligosaccharyltransferase, OSTA, TRANSFERASE; HET: KZB, NAG, EGY, MAN, KZE, BMA; 3.5A {Homo sapiens}; Related PDB entries: 6FTI\_5 6FTG\_5 6FTJ\_5; Related PDB entries: 6FTG\_5 6FTI\_5 6FTJ\_5; Related PDB entries: 6FTG\_5 6FTI\_5 6FTJ\_5 | 99.82 | 3.4e-17 | 29.7 | 374 | 705 |
| 6 | 5OGL\_A | Peptide-binding protein, Substrate mimicking peptide; Oligosaccharyltransferase, Complex, Protein N-glycosylation, Bacteria; HET: 9UB, PPN; 2.7A {Campylobacter lari (strain RM2100 / D67 / ATCC BAA-1060)}; Related PDB entries: 3RCE\_A 6GXC\_A; Related PDB entries: 6GXC\_A 3RCE\_A ; Related PDB entries: 6GXC\_A 3RCE\_A | 99.77 | 4.5e-16 | 27.1 | 371 | 713 |
| 7 | 6P25\_A | Dolichyl-diphosphooligosaccharide--protein glycosyltransferase subunits (E.C.2.4.99.18); complex, TRANSFERASE, glycosylation; HET: NAG, CPL, NNM; 3.2A {Saccharomyces cerevisiae W303}; Related PDB entries: 6P2R\_A ; Related PDB entries: 6P2R\_A ; Related PDB entries: 6P2R\_A | 99.66 | 6.3e-14 | 25.6 | 224 | 817 |
| 8 | 6P25\_B | Dolichyl-diphosphooligosaccharide--protein glycosyltransferase subunits (E.C.2.4.99.18); complex, TRANSFERASE, glycosylation; HET: NAG, CPL, NNM; 3.2A {Saccharomyces cerevisiae W303}; Related PDB entries: 6P2R\_B ; Related PDB entries: 6P2R\_B ; Related PDB entries: 6P2R\_B | 99.65 | 8.6e-14 | 25.5 | 241 | 759 |
| 9 | 7BVF\_A | Probable arabinosyltransferase B (E.C.2.4.2.-), Probable; Mycobacterium tuberculosis, cell wall synthesis; HET: 95E, DSL, CDL;{Mycolicibacterium smegmatis MC2 155} | 99.63 | 1.1e-12 | 29.8 | 357 | 1102 |
| 10 | 7BVF\_B | Probable arabinosyltransferase B (E.C.2.4.2.-), Probable; Mycobacterium tuberculosis, cell wall synthesis; HET: 95E, DSL, CDL;{Mycolicibacterium smegmatis MC2 155} | 99.58 | 1.2e-11 | 32 | 355 | 1116 |
| 11 | 7BWR\_A | Integral membrane indolylacetylinositol arabinosyltransferase EmbB; Mycobacterium tuberculosis, EmbB, cryo-EM, ethambutol; HET: F8L;{Mycolicibacterium smegmatis MC2 155}; Related PDB entries: 7BVC\_B 7BVG\_B 7BWR\_B 7BX8\_B 7BX8\_A | 99.55 | 1.4e-11 | 29.3 | 356 | 1082 |
| 12 | 6SNI\_X | Dolichyl pyrophosphate Man9GlcNAc2 alpha-1,3-glucosyltransferase (E.C.2.4.1.267); Glycosyltransferase, Glucosyltransferase, GT-C, N-Glycosylation, MEMBRANE; HET: PTY, Y01;{Saccharomyces cerevisiae}; Related PDB entries: 6SNH\_X | 99.55 | 1.8e-11 | 29.3 | 324 | 562 |
| 13 | 6W98\_A | F5/8 type C domain-containing protein; Glycosyltransferase, lipomannan, lipoarabinomannan, arabinofuranose, membrane; HET: PNS, 6OU; 2.9A {Escherichia coli (strain K12)}; Related PDB entries: 6WBX\_A 6WBY\_A | 99.49 | 8.1e-11 | 31.2 | 384 | 1413 |
| 14 | 7BVE\_B | Integral membrane indolylacetylinositol arabinosyltransferase EmbC; Mycobacterium smegmatis, cell wall synthesis; HET: PO4, PN7, 95E; 2.81A {Mycolicibacterium smegmatis MC2 155}; Related PDB entries: 7BVH\_B 7BVH\_A 7BVE\_A | 99.49 | 4.2e-10 | 33.7 | 353 | 1084 |
| 15 | 7BVC\_A | Integral membrane indolylacetylinositol arabinosyltransferase EmbA; Mycobacterium smegmatis, cell wall synthesis; HET: 95E, PNS, CDL, F8L;{Mycolicibacterium smegmatis MC2 155}; Related PDB entries: 7BVG\_A | 99.35 | 4.2e-9 | 30.3 | 353 | 1088 |
| 16 | 6P25\_B | Dolichyl-diphosphooligosaccharide--protein glycosyltransferase subunits (E.C.2.4.99.18); complex, TRANSFERASE, glycosylation; HET: NAG, CPL, NNM; 3.2A {Saccharomyces cerevisiae W303}; Related PDB entries: 6P2R\_B ; Related PDB entries: 6P2R\_B ; Related PDB entries: 6P2R\_B | 65.1 | 83 | 10.6 | 102 | 759 |
| 17 | 6P25\_A | Dolichyl-diphosphooligosaccharide--protein glycosyltransferase subunits (E.C.2.4.99.18); complex, TRANSFERASE, glycosylation; HET: NAG, CPL, NNM; 3.2A {Saccharomyces cerevisiae W303}; Related PDB entries: 6P2R\_A ; Related PDB entries: 6P2R\_A ; Related PDB entries: 6P2R\_A | 41.55 | 330 | 10.5 | 105 | 817 |

Displaying 1 to 17 of 17 hits

- «
- ‹
- 1
- ›
- »

#### Alignments

|  |  |  |  |
| --- | --- | --- | --- |
|  | | | |
|  | Template alignmentTemplate 3D StructurePDBe | | |
| 1. | 5EZM\_A 4-amino-4-deoxy-L-arabinose (L-Ara4N) transferase; membrane protein, lipid glycosyltransferase, zinc; HET: PC, DSL, PO4, MPG, EPE; 2.7A {Cupriavidus metallidurans (strain ATCC 43123 / DSM 2839 / NBRC 102507 / CH34)}; Related PDB entries: 5F15\_A ; Related PDB entries: 5F15\_A ; Related PDB entries: 5F15\_A | | |
|  | Probability: 99.91%, E-value: 1.3e-21, Score: 183.22, Aligned cols: 365, Identities: 10%, Similarity: -0.004, | | |
|  |
|  | Q ss\_pred |  | cchhHHHHHHHHHHHHHHHHHhhCCCce--eccHHHHhcCCCCCCCCcHHHHhccccCCCCCCcchhcCCCCcchHHHHH |
|  | Q Q6ZXV5 | 2 | ANINLKEITLIVGVVTACYWNSLFCGFV--FDDVSAILDNKDLHPSTPLKTLFQNDFWGTPMSEERSHKSYRPLTVLTFR   79 (426) |
|  | Q Consensus | 2 | ~~~~~~~~~~l~~~~~~~~~~~~~~~~~--~Dd~~~~~~~~~~~~~~~~~~~~~~~~~~~~~~~~~~~~~~~Pl~~~~~~   79 (426) |
|  |  |  | ++.....++++++++...+......... +||..+. ....+..+++.+..+..++..+.++||++.++.+ |
|  | T Consensus | 27 | ~~~~~~~~~~~~~~~~~~~~~~~~~~~~~~~De~~~~---------~~a~~~~~~~~~~~~~~~~~~~~~~pPl~~~l~~   97 (578) |
|  | T 5EZM\_A | 27 | WSAATGWVVLFVAVALVVWFVSLDMRHLVGPDEGRYA---------EISREMFASGDWVTIRYNALKYFEKPPFHMWVTV   97 (578) |
|  | T ss\_dssp |  | -CCTHHHHHHHHHHHHHHHHHGGGSSCCCTTHHHHHH---------HHHHHHHHHCCSSSCEETTEECCSSCSHHHHHHH |
|  | T ss\_pred |  | HHHHHHHHHHHHHHHHHHHHhccccccCCCCCHHHHH---------HHHHHHHHhCCceeEEECCEeCCCCCHHHHHHHH |
|  |
|  |
|  | Q ss\_pred |  | HHHHHhCCCchHHHHHHHHHHHHHHHHHHHHHHHhcCCHHHHHHHHHHHHCcccHHHHHHhhccHHHHHHHHHHHHHHHH |
|  | Q Q6ZXV5 | 80 | LNYLLSELKPMSYHLLNMIFHAVVSVIFLKVCKLFLDNKSSVIASLLFAVHPIHTEAVTGVVGRAELLSSIFFLAAFLSY   159 (426) |
|  | Q Consensus | 80 | ~~~~lfg~~~~~~rl~~~l~~~~~~~~~y~l~~~~~~~~~a~~aall~~~~p~~~~~~~~~~~~~~~~~~~~~~l~~~~~   159 (426) |
|  |  |  | ....++|.++...|+++++++++++.++|.++|+..+++.|++++++++++|......... ++|.+..++.+++++++ |
|  | T Consensus | 98 | ~~~~l~g~~~~~~rl~~~l~~~l~~~~~~~l~~~~~~~~~a~~a~~l~~~~p~~~~~~~~~--~~~~~~~~~~~~~~~~~   175 (578) |
|  | T 5EZM\_A | 98 | VGYELFGLGEWQARLAVALSGLLGIGVSMMAARRWFGARAAAFTGLALLAAPMWSVAAHFN--TLDMTLAGVMSCVLAFM   175 (578) |
|  | T ss\_dssp |  | HHHHHHCSSHHHHTHHHHHHHHHHHHHHHHHHHHHHCHHHHHHHHHHHHHCHHHHHHHTSC--CHHHHHHHHHHHHHHHH |
|  | T ss\_pred |  | HHHHHHCcCHHHHHHHHHHHHHHHHHHHHHHHHHHhChHHHHHHHHHHHHHHHHHHHHhhH--hhHHHHHHHHHHHHHHH |
|  |
|  |
|  | Q ss\_pred |  | HHcCCCC--CcccHHHHHHHHHHHHHHHHhHhHHHHHHHHHHHHHHHHhcCCCccchhcccchhhcCCCCCChHHHHHHH |
|  | Q Q6ZXV5 | 160 | TRSKGPD--NSIIWTPIALTVFLVAVATLCKEQGITVVGICCVYEVFIAQGYTLPLLCTTAGQFLRGKGSIPFSMLQTLV   237 (426) |
|  | Q Consensus | 160 | ~~~~~~~--~~~~~~~~~~~~~~~~la~~~k~~~~~~~~~~~~~~~~~~~~~~~~~~~~~~~~~~~~~~~~~~~~~~~~~   237 (426) |
|  |  |  | .+..+++ +++++++..+++++.+++.++|+....+.+...+......++ +.+....+.... |
|  | T Consensus | 176 | ~~~~~~~~~~~~~~~~~~l~g~~~gla~~~k~~~~~~~~~~~~~~~~~~~~-----------------~~~~~~~~~~~~   238 (578) |
|  | T 5EZM\_A | 176 | LMGQHPDASVAARRGWMVACWAAMGVAILTKGLVGIALPGLVLVVYTLVTR-----------------DWGLWRRLHLAL   238 (578) |
|  | T ss\_dssp |  | HHHTCTTSCHHHHHHHHHHHHHHHHHHHHHHTTHHHHHHHHHHHHHHHHSC-----------------CTTHHHHTCHHH |
|  | T ss\_pred |  | HHHhCCCcchhhccHHHHHHHHHHHHHHHccchHHHHHHHHHHHHHHHHcC-----------------ChHHHhhcchHH |
|  |
|  |
|  | Q ss\_pred |  | HHHHHHHHHHHHHHHHHHHHccCCCccccCCCcccCCCchhhHHhHhhHHHHHHHHHhccccccccCccccCcccccccc |
|  | Q Q6ZXV5 | 238 | KLIVLMFSTLLLVVIRVQVIQSQLPVFTRFDNPAAVSPTPTRQLTFNYLLPVNAWLLLNPSELCCDWTMGTIPLIESLLD   317 (426) |
|  | Q Consensus | 238 | ~~~~~~~~~~~~~~~~~~~~~~~~~~~~~~~~~~~~~~~~~~~~~~~~~~~~~~~~~~~~~~~~~~~~~~~~~~~~~~~~   317 (426) |
|  |  |  | .....++...++.........+.....................................+ |
|  | T Consensus | 239 | ~~~~~~~~~~~~~~~~~~~~~~~~~~~~~~~~~~~~~~~~~~~~~~~~~~~~~~~~~~~~--------------------   298 (578) |
|  | T 5EZM\_A | 239 | GVVVMLVITVPWFYLVSVRNPEFPNFFFIHEHWQRYTSNIHSRSGSVFYFLPLVIGGFLP--------------------   298 (578) |
|  | T ss\_dssp |  | HHHHHHHHHHHHHHHHHHHCTTHHHHHHHCCCCCC-------CCCCTTTHHHHHHHHTGG-------------------- |
|  | T ss\_pred |  | HHHHHHHHHHHHHHHHHHHCCcchhhhHHHHHHHHHhcCcccCCCChHHHHHHHHHHhhh-------------------- |
|  |
|  |
|  | Q ss\_pred |  | HHHHHHHHHHHHHHHHHHHHHHccCCCchhHHHHHHHHHHHHHHHhccCCCcchhhchhhchHHHHHHHHHHHHHHHHhc |
|  | Q Q6ZXV5 | 318 | IRNLATFTFFCFLGMLGVFSIRYSGDSSKTVLMALCLMALPFIPASNLFFPVGFVVAERVLYVPSMGFCILVAHGWQKIS   397 (426) |
|  | Q Consensus | 318 | ~~~~~~~~~~~~~~~~~~~~~~~~~~~~~~~~~~~~~~~~~~~~~~~~~~~~~~~~~~ry~~~~~~~~~il~~~~~~~~~   397 (426) |
|  |  |  | ...++.......++++++++.............+..... ........+||..+..|+++++++.++.+.. |
|  | T Consensus | 299 | ---------~~~~~~~~~~~~~~~~~~~~~~~~~~~~~~~~~~~~~~~-~~~~~~~~~ry~~~~~p~l~ll~~~~l~~~~   368 (578) |
|  | T 5EZM\_A | 299 | ---------WAGIFPKLWTAMRAPVEGTQARFRPALMAGIWAIAIFVF-FSISRSKLPGYIVPVIPALGILAGVALDRLS   368 (578) |
|  | T ss\_dssp |  | ---------GGGGHHHHHHHHCC-------CCCHHHHHHHHHHHHHHH-HHTSSSCCGGGGTTHHHHHHHHHHHHHHTCC |
|  | T ss\_pred |  | ---------HHhHHHHHHHHhhcccccCcccccHHHHHHHHHHHHHHH-HHHhcccChhcHHhHHHHHHHHHHHHHHHhC |
|  |
|  |
|  | Q ss\_pred |  | ccchHHHHHHHHHHHHHHHHHHHhhhh |
|  | Q Q6ZXV5 | 398 | TKSVFKKLSWICLSMVILTHSLKTFHR   424 (426) |
|  | Q Consensus | 398 | ~~~~~~~~~~~~~~~~~~~~~~~~~~~   424 (426) |
|  |  |  | ++.+.++.....++++++......... |
|  | T Consensus | 369 | ~~~~~~~~~~~~~~~~~~~~~~~~~~~   395 (578) |
|  | T 5EZM\_A | 369 | PRSWGKQLIGMAIVAACGLLASPVVAT   395 (578) |
|  | T ss\_dssp |  | HHHHHHHHHHHHHHHHHHHHHGGGGGG |
|  | T ss\_pred |  | CCccHHHHHHHHHHHHHHHHHHHHHHH |
|  |
| --- | | | |
|  | Template alignmentTemplate 3D StructurePDBe | | |
| 2. | 6S7T\_A Dolichyl-diphosphooligosaccharide--protein glycosyltransferase subunit STT3B (E.C.2.4.99.18); N-glycosylation, Oligosaccharyltransferase, OSTB, TRANSFERASE; HET: 0K3, KZB, NAG, EGY, MAN, BMA; 3.5A {Homo sapiens} | | |
|  | Probability: 99.83%, E-value: 3.1e-17, Score: 160.29, Aligned cols: 372, Identities: 8%, Similarity: -0.02, | | |
|  |
|  | Q ss\_pred |  | cchhHHHHHHHHHHHHHHHHHh-hCCCce---eccHHHHhcCCCCCCCCcHHHHhccccCCCCCCcchhcCCCCcchHHH |
|  | Q Q6ZXV5 | 2 | ANINLKEITLIVGVVTACYWNS-LFCGFV---FDDVSAILDNKDLHPSTPLKTLFQNDFWGTPMSEERSHKSYRPLTVLT   77 (426) |
|  | Q Consensus | 2 | ~~~~~~~~~~l~~~~~~~~~~~-~~~~~~---~Dd~~~~~~~~~~~~~~~~~~~~~~~~~~~~~~~~~~~~~~~Pl~~~~   77 (426) |
|  |  |  | .......+++++++++.++... ...+.. .||..+...+..+.+.+.....-..+.......+......++|++.++ |
|  | T Consensus | 70 | ~~~~~~~l~~i~~~~~~~rl~~~~~~~~~~~~~D~~~~~~~a~~~~~~g~~~~~~~~~~~~~~p~g~~~~~~~~P~~~~l   149 (826) |
|  | T 6S7T\_A | 70 | SLLSFTILFLAWLAGFSSRLFAVIRFESIIHEFDPWFNYRSTHHLASHGFYEFLNWFDERAWYPLGRIVGGTVYPGLMIT   149 (826) |
|  | T ss\_dssp |  | HHHHHHHHHHHHHHHHHTTCTTTTTTCSCCCSTTHHHHHHHHHHHHHHCHHHHHTCEECSSSTTTCEESTTSSCTTHHHH |
|  | T ss\_pred |  | HHHHHHHHHHHHHHHHHHHHHHHHhhhhhhcCCChHHHHHHHHHHHHhCcHHHhccccchhcCCCCCCCCCCCchHHHHH |
|  |
|  |
|  | Q ss\_pred |  | HHHHHHHhC------CCchHHHHHHHHHHHHHHHHHHHHHHHhcCCHHHHHHHHHHHHCcccHHHHHHhhccHHHHHHHH |
|  | Q Q6ZXV5 | 78 | FRLNYLLSE------LKPMSYHLLNMIFHAVVSVIFLKVCKLFLDNKSSVIASLLFAVHPIHTEAVTGVVGRAELLSSIF   151 (426) |
|  | Q Consensus | 78 | ~~~~~~lfg------~~~~~~rl~~~l~~~~~~~~~y~l~~~~~~~~~a~~aall~~~~p~~~~~~~~~~~~~~~~~~~~   151 (426) |
|  |  |  | .+..+.++| .+....|+++.++++++++++|.++|++.++..|++++++++++|.++.........+|.+..++ |
|  | T Consensus | 150 | ~a~~~~l~g~~~~~~~~~~~~~l~~~l~~~l~~~~~y~l~r~l~~~~~allaall~a~~p~~~~~s~~~~~~~e~~~~~~   229 (826) |
|  | T 6S7T\_A | 150 | AGLIHWILNTLNITVHIRDVCVFLAPTFSGLTSISTFLLTRELWNQGAGLLAACFIAIVPGYISRSVAGSFDNEGIAIFA   229 (826) |
|  | T ss\_dssp |  | HHHHHHHHHHTTCCCCHHHHHHTHHHHHHHHHHHHHHHHHHHHSCHHHHHHHHHHTTTCHHHHGGGSTTCCCSHHHHHHH |
|  | T ss\_pred |  | HHHHHHHHHhcCCCCcHHHHHHHHHHHHHHHHHHHHHHHHHHHcCchHHHHHHHHHHHHHHHHHhhhccCchHHHHHHHH |
|  |
|  |
|  | Q ss\_pred |  | HHHHHHHHHHcCCCCCcccHHHHHHHHHHHHHHHHhHhHHHHHHHHHHHHHHHHhcCCCccchhcccchhhcCCCCCCh- |
|  | Q Q6ZXV5 | 152 | FLAAFLSYTRSKGPDNSIIWTPIALTVFLVAVATLCKEQGITVVGICCVYEVFIAQGYTLPLLCTTAGQFLRGKGSIPF-   230 (426) |
|  | Q Consensus | 152 | ~~l~~~~~~~~~~~~~~~~~~~~~~~~~~~~la~~~k~~~~~~~~~~~~~~~~~~~~~~~~~~~~~~~~~~~~~~~~~~-   230 (426) |
|  |  |  | .+++++++.+..++++ .++.++++++++++.++|+.+.++.+++.++.++.... ++..++ |
|  | T Consensus | 230 | ~~l~l~~~~~~~~~~~---~~~~~l~gl~~gla~~~~~~~~~~~~~~~l~~~~~~~~----------------~~~~~~~   290 (826) |
|  | T 6S7T\_A | 230 | LQFTYYLWVKSVKTGS---VFWTMCCCLSYFYMVSAWGGYVFIINLIPLHVFVLLLM----------------QRYSKRV   290 (826) |
|  | T ss\_dssp |  | HHHHHHHHHHHHHHCC---HHHHHHHHHHHHHHHHHCTTHHHHHHHHHHHHHHHHHT----------------TCCCHHH |
|  | T ss\_pred |  | HHHHHHHHHHHhccCc---HHHHHHHHHHHHHHHHhcccHHHHHHHHHHHHHHHHHh----------------ccCChhH |
|  |
|  |
|  | Q ss\_pred |  | --------------------------------------------------------HHHHHHHHHHHHHHHHHHHHHHHH |
|  | Q Q6ZXV5 | 231 | --------------------------------------------------------SMLQTLVKLIVLMFSTLLLVVIRV   254 (426) |
|  | Q Consensus | 231 | --------------------------------------------------------~~~~~~~~~~~~~~~~~~~~~~~~   254 (426) |
|  |  |  | ...+.........+..+....... |
|  | T Consensus | 291 | ~~~~~~~~~~~~~~~~~~p~~~~~~~~~~~~~~~~~~~~~~~~~~~~~~~~~~~~~~~~~~~~~~~~~~~~~~~~~~~~~   370 (826) |
|  | T 6S7T\_A | 291 | YIAYSTFYIVGLILSMQIPFVGFQPIRTSEHMAAAGVFALLQAYAFLQYLRDRLTKQEFQTLFFLGVSLAAGAVFLSVIY   370 (826) |
|  | T ss\_dssp |  | HHHHHHHHHHHHHHHTTSTTTTTHHHHBSSTHHHHHHHHHHHHHHHHHHHHHHSCSTTTHHHHHHHHHHHHHHHHHHHHH |
|  | T ss\_pred |  | HHHHHHHHHHHHHHHhccccCCCCcccchHHHHHHHHHHHHHHHHHHHHHHHhcCHHHHHHHHHHHHHHHHHHHHHHHHH |
|  |
|  |
|  | Q ss\_pred |  | HHHccCCCccccCCCcccCCCchhhHHhHhhHHHHHHHHHhccccccccCccccCccccccccHHHHHHHHHHHHHHHHH |
|  | Q Q6ZXV5 | 255 | QVIQSQLPVFTRFDNPAAVSPTPTRQLTFNYLLPVNAWLLLNPSELCCDWTMGTIPLIESLLDIRNLATFTFFCFLGMLG   334 (426) |
|  | Q Consensus | 255 | ~~~~~~~~~~~~~~~~~~~~~~~~~~~~~~~~~~~~~~~~~~~~~~~~~~~~~~~~~~~~~~~~~~~~~~~~~~~~~~~~   334 (426) |
|  |  |  | ................................. ..................++++... |
|  | T Consensus | 371 | ~~~~~~~~~~~~~~~~~~~~~~~~~~~~~~~~~----------------------~~~~~~~~~~~~~~~~~~~~~~~~~   428 (826) |
|  | T 6S7T\_A | 371 | LTYTGYIAPWSGRFYSLWDTGYAKIHIPIIASV----------------------SEHQPTTWVSFFFDLHILVCTFPAG   428 (826) |
|  | T ss\_dssp |  | HHHHTSSBCCCHHHHHHHHSSHHHHTCHHHHHB----------------------STTSCCCHHHHHHSCSSHHHHHHHH |
|  | T ss\_pred |  | HHhccccccchHHHHHHhcccccccCccchhcc----------------------cccCCCCHHHHHHHHHHHHHHHHHH |
|  |
|  |
|  | Q ss\_pred |  | HHHHHccCCCchhHHHHHHHHHHHHHHHhccCCCcchhhchhhchHHHHHHHHHHHHHHHHhcccc-------------- |
|  | Q Q6ZXV5 | 335 | VFSIRYSGDSSKTVLMALCLMALPFIPASNLFFPVGFVVAERVLYVPSMGFCILVAHGWQKISTKS--------------   400 (426) |
|  | Q Consensus | 335 | ~~~~~~~~~~~~~~~~~~~~~~~~~~~~~~~~~~~~~~~~~ry~~~~~~~~~il~~~~~~~~~~~~--------------   400 (426) |
|  |  |  | .....++++.............+.+... ..||..+..|+++++++.++..+.++. |
|  | T Consensus | 429 | ~~~~~~~~~~~~~~~~~~~~~~~~~~~~-----------~~Ry~~~~~p~~~ll~a~~l~~l~~~~~~~~~~~~~~~~~~   497 (826) |
|  | T 6S7T\_A | 429 | LWFCIKNINDERVFVALYAISAVYFAGV-----------MVRLMLTLTPVVCMLSAIAFSNVFEHYLGDDMKRENPPVED   497 (826) |
|  | T ss\_dssp |  | HHHHHHSCCHHHHHHHHHHHHHHHHHTT-----------CSTTHHHHHHHHHHHHHHHHHHHHHHTCC------------ |
|  | T ss\_pred |  | HHHHHhcCCHHHHHHHHHHHHHHHHHHH-----------HHHHHHhHHHHHHHHHHHHHHHHHHHHhccccccCCCCCCC |
|  |
|  |
|  | Q ss\_pred |  | ------------------------------------hHHHHHHHHHHHHHHHHHHHhhhhc |
|  | Q Q6ZXV5 | 401 | ------------------------------------VFKKLSWICLSMVILTHSLKTFHRN   425 (426) |
|  | Q Consensus | 401 | ------------------------------------~~~~~~~~~~~~~~~~~~~~~~~~~   425 (426) |
|  |  |  | ..+.....+++++++.......... |
|  | T Consensus | 498 | ~~~~~~~~~~~~~~~~~~~~~~~~~~~~~~~~~~~~~~~~~~~~~~~~~~~~~~~~~~~~~   558 (826) |
|  | T 6S7T\_A | 498 | SSDEDDKRNQGNLYDKAGKVRKHATEQEKTEEGLGPNIKSIVTMLMLMLLMMFAVHCTWVT   558 (826) |
|  | T ss\_dssp |  | -----------------------------------CHHHHHHHHHHHHHHHHHHHHHHHHC |
|  | T ss\_pred |  | CCccchhhccccccccccccccccchhhhhhcCCChHHHHHHHHHHHHHHHHHHHHHHHHh |
|  |
| --- | | | |
|  | Template alignmentTemplate 3D StructurePDBe | | |
| 3. | 3WAJ\_A Transmembrane oligosaccharyl transferase (E.C.2.4.1.119); oligosaccharyltransferase, N-glycosylation, Archaeoglobus fulgidus, GT-C; 2.501A {Archaeoglobus fulgidus}; Related PDB entries: 5GMY\_A 3WAK\_A; Related PDB entries: 5GMY\_A 3WAK\_A; Related PDB entries: 5GMY\_A 3WAK\_A | | |
|  | Probability: 99.82%, E-value: 1.8e-17, Score: 162.99, Aligned cols: 365, Identities: 11%, Similarity: 0.043, | | |
|  |
|  | Q ss\_pred |  | CcchhHHHHHHHHHHHHHHHH-------HhhCCCce-eccHHHHhcCCCCCCCCcHHHHhccccCCCCCCcchhcCCCCc |
|  | Q Q6ZXV5 | 1 | MANINLKEITLIVGVVTACYW-------NSLFCGFV-FDDVSAILDNKDLHPSTPLKTLFQNDFWGTPMSEERSHKSYRP   72 (426) |
|  | Q Consensus | 1 | ~~~~~~~~~~~l~~~~~~~~~-------~~~~~~~~-~Dd~~~~~~~~~~~~~~~~~~~~~~~~~~~~~~~~~~~~~~~P   72 (426) |
|  |  |  | +++.....+++++++++.++. ........ .||..+...+.++.++.......+........ ....++| |
|  | T Consensus | 9 | ~~~~~~~~l~~l~~~~~~lr~~~~~~~~~~~~~~~~~~D~~~~~~~a~~~~~~~~~~~~~d~~~~~p~g----~~~~~~P   84 (875) |
|  | T 3WAJ\_A | 9 | KKYWHLSVLVIAALISVKLRILNPWNSVFTWTVRLGGNDPWYYYRLIENTIHNFPHRIWFDPFTYYPYG----SYTHFGP   84 (875) |
|  | T ss\_dssp |  | ------CTTTHHHHHHHCCCCCTTHHHHBSSSBCCCSSHHHHHHHHHHHHHHTTTCCCSEETTSTTTTC----EECCSCH |
|  | T ss\_pred |  | hhHHHHHHHHHHHHHHHHHHhhCccccccCCceecccCChHHHHHHHHHHHHHCcccCCCCchhcCCCC----cCCCchh |
|  |
|  |
|  | Q ss\_pred |  | chHHHHHHHHHHhCCC-----chHHHHHHHHHHHHHHHHHHHHHHHhcCCHHHHHHHHHHHHCcc-cHHHHHHhhccHHH |
|  | Q Q6ZXV5 | 73 | LTVLTFRLNYLLSELK-----PMSYHLLNMIFHAVVSVIFLKVCKLFLDNKSSVIASLLFAVHPI-HTEAVTGVVGRAEL   146 (426) |
|  | Q Consensus | 73 | l~~~~~~~~~~lfg~~-----~~~~rl~~~l~~~~~~~~~y~l~~~~~~~~~a~~aall~~~~p~-~~~~~~~~~~~~~~   146 (426) |
|  |  |  | ++.++.+....++|.+ ....|++++++++++++++|.++|++++++.|++++++++++|. ...........+|. |
|  | T Consensus | 85 | l~~~l~a~~~~l~G~~~~~~~~~~~~l~~~l~~~l~~~~~y~l~r~l~~~~~allaall~a~~p~~~~~~s~~g~~~~~~   164 (875) |
|  | T 3WAJ\_A | 85 | FLVYLGSIAGIIFSATSGESLRAVLAFIPAIGGVLAILPVYLLTREVFDKRAAVIAAFLIAIVPGQFLQRSILGFNDHHI   164 (875) |
|  | T ss\_dssp |  | HHHHHHHHHHHHTTCCSHHHHHHHHHHHHHHHHHTTHHHHHHHHHHHSCHHHHHHHHHHHTTCCSHHHHTTSTTCCCSHH |
|  | T ss\_pred |  | HHHHHHHHHHHHHcCCChHHHHHHHHHHHHHHHHHHHHHHHHHHHHHcChHHHHHHHHHHHHcchHHHHHHhccccchHH |
|  |
|  |
|  | Q ss\_pred |  | HHHHHHHHHHHHHHHcCC------------CCCcccHHHHHHHHHHHHHHHHhHhHHHHHHHHHHHHHHHHhcCCCccch |
|  | Q Q6ZXV5 | 147 | LSSIFFLAAFLSYTRSKG------------PDNSIIWTPIALTVFLVAVATLCKEQGITVVGICCVYEVFIAQGYTLPLL   214 (426) |
|  | Q Consensus | 147 | ~~~~~~~l~~~~~~~~~~------------~~~~~~~~~~~~~~~~~~la~~~k~~~~~~~~~~~~~~~~~~~~~~~~~~   214 (426) |
|  |  |  | +..++.+++++++.+..+ +++ +++.++++++++++.++|..+.++.+++.+..++.... |
|  | T Consensus | 165 | ~~~~~~~l~l~~~~~~~~~~~~~~~~~~~~~~~---~~~~~l~gl~~gl~~lt~~~~~~~~~~~~~~~~~~~~~------   235 (875) |
|  | T 3WAJ\_A | 165 | WEAFWQVSALGTFLLAYNRWKGHDLSHNLTARQ---MAYPVIAGITIGLYVLSWGAGFIIAPIILAFMFFAFVL------   235 (875) |
|  | T ss\_dssp |  | HHHHHHHHHHHHHHHHHTTSSSCCC----CTTT---SHHHHHHHHHHHHHHHHCGGGGGHHHHHHHHHHHHHHT------ |
|  | T ss\_pred |  | HHHHHHHHHHHHHHHHHHHhccCCCCcccccch---hHHHHHHHHHHHHHHHHhchHHHHHHHHHHHHHHHHHH------ |
|  |
|  |
|  | Q ss\_pred |  | hcccchhhcCCCCCChHHHHHHHHHHHHHHHHHHHHHHH----------------------------------------- |
|  | Q Q6ZXV5 | 215 | CTTAGQFLRGKGSIPFSMLQTLVKLIVLMFSTLLLVVIR-----------------------------------------   253 (426) |
|  | Q Consensus | 215 | ~~~~~~~~~~~~~~~~~~~~~~~~~~~~~~~~~~~~~~~-----------------------------------------   253 (426) |
|  |  |  | .+...+...+.........++..+....+. |
|  | T Consensus | 236 | ---------~~~~~~~~~~~~~~~~~~~~~~~l~~~p~~~~~~~~~~~~~~~~~~~~~~~~~~~~~~~~~~~~~~~~~~~   306 (875) |
|  | T 3WAJ\_A | 236 | ---------AGFVNADRKNLSLVAVVTFAVSALIYLPFAFNYPGFSTIFYSPFQLLVLLGSAVIAAAFYQIEKWNDVGFF   306 (875) |
|  | T ss\_dssp |  | ---------TTTCCCCHHHHHHHHHHHHHHHHHHHGGGTTSSSSCCSSSSCHHHHHHHHHHHHHHHHHHHHHHHHHHTHH |
|  | T ss\_pred |  | ---------HHhCCCCCHhHHHHHHHHHHHHHHHHHHHhcCCCCCChhcccHHHHHHHHHHHHHHHHHHHHHHhcCcchH |
|  |
|  |
|  | Q ss\_pred |  | -----------------------------HHHHccCCCccccCCCcccCCCchhhHHhHhhHHHHHHHHHhccccccccC |
|  | Q Q6ZXV5 | 254 | -----------------------------VQVIQSQLPVFTRFDNPAAVSPTPTRQLTFNYLLPVNAWLLLNPSELCCDW   304 (426) |
|  | Q Consensus | 254 | -----------------------------~~~~~~~~~~~~~~~~~~~~~~~~~~~~~~~~~~~~~~~~~~~~~~~~~~~   304 (426) |
|  |  |  | ........................+. |
|  | T Consensus | 307 | ~~~~~~~~~~~~~~~~~~~~~~~~~~~~~~~~~~~~~~~~~~~~~~~~~~~i~e~-------------------------   361 (875) |
|  | T 3WAJ\_A | 307 | ERVGLGRKGMPLAVIVLTALIMGLFFVISPDFARNLLSVVRVVQPKGGALTIAEV-------------------------   361 (875) |
|  | T ss\_dssp |  | HHTTCGGGHHHHHHHHHHHHHHHHHHC----------------------------------------------------- |
|  | T ss\_pred |  | HhcCCCccchHHHHHHHHHHHHHHHHHHCCHHHHHHHhhcceecCCCCceeeeec------------------------- |
|  |
|  |
|  | Q ss\_pred |  | ccccCccccc------cccHHHHHHHHHHHHHHHHHHHHHHccCCCchhHHHHHHHHHHHHHHHhccCCCcchhhchhhc |
|  | Q Q6ZXV5 | 305 | TMGTIPLIES------LLDIRNLATFTFFCFLGMLGVFSIRYSGDSSKTVLMALCLMALPFIPASNLFFPVGFVVAERVL   378 (426) |
|  | Q Consensus | 305 | ~~~~~~~~~~------~~~~~~~~~~~~~~~~~~~~~~~~~~~~~~~~~~~~~~~~~~~~~~~~~~~~~~~~~~~~~ry~   378 (426) |
|  |  |  | .+.... .............+.+++......++++++.....+.+++.+..+.. .....||. |
|  | T Consensus | 362 | ----~~~~~~~~~~~~~~~~~~~~~~~~~l~~~~~~~~~~~~~~~~~~~~~~l~~~~~~~~~~---------~~~~~Ry~   428 (875) |
|  | T 3WAJ\_A | 362 | ----YPFFFTHNGEFTLTNAVLHFGALFFFGMAGILYSAYRFLKRRSFPEMALLIWAIAMFIA---------LWGQNRFA   428 (875) |
|  | T ss\_dssp |  | ----------------CTHHHHHHTTHHHHHHHHHHHHHHHHHHHCCHHHHHHHHHHHHHHHH---------TSSCGGGT |
|  | T ss\_pred |  | ----cccccccCCCccHHHHHHHhHHHHHHHHHHHHHHHHHHHccCCchhHHHHHHHHHHHHH---------HHHhhhHH |
|  |
|  |
|  | Q ss\_pred |  | hHHHHHHHHHHHHHHHHhcccc--------------hHHHHHHHHHHHHHHHHHHHhhhhc |
|  | Q Q6ZXV5 | 379 | YVPSMGFCILVAHGWQKISTKS--------------VFKKLSWICLSMVILTHSLKTFHRN   425 (426) |
|  | Q Consensus | 379 | ~~~~~~~~il~~~~~~~~~~~~--------------~~~~~~~~~~~~~~~~~~~~~~~~~   425 (426) |
|  |  |  | .+..|+++++++.++..+.++. +.+.....+++++++.......... |
|  | T Consensus | 429 | ~~~~p~~~il~a~~l~~l~~~~~~~~~~~~~~~~~~~~~~~~~~~~~~~~~~~~~~~~~~~   489 (875) |
|  | T 3WAJ\_A | 429 | YYFAAVSAVYSALALSVVFDKLHLYRALENAIGARNKLSYFRVAFALLIALAAIYPTYILA   489 (875) |
|  | T ss\_dssp |  | HHHHHHHHHHHHHHHHHHGGGCC-----------------CCTTTHHHHHHHHHHHHHHHH |
|  | T ss\_pred |  | HHHHHHHHHHHHHHHHHHHHHHhHHHHHHhhhchhccchHHHHHHHHHHHHHHHHHHHHHH |
|  |
| --- | | | |
|  | Template alignmentTemplate 3D StructurePDBe | | |
| 4. | 6EZN\_F Dolichyl-diphosphooligosaccharide--protein glycosyltransferase subunit 1 (E.C.2.4.99.18); OST complex, oligosaccharyltransferase, N-linked glycosylation; HET: PTY, BMA, CPL, MAN, NAG;{Saccharomyces cerevisiae (strain ATCC 204508 / S288c)}; Related PDB entries: 6C26\_A; Related PDB entries: 6C26\_A; Related PDB entries: 6C26\_A | | |
|  | Probability: 99.82%, E-value: 5.1e-17, Score: 156.42, Aligned cols: 365, Identities: 12%, Similarity: 0.012, | | |
|  |
|  | Q ss\_pred |  | cchhHHHHHHHHHHHHHHHHHh-hCCCc---eeccHHHHhcCCCCCCCCcHHHHhccccCCCCCCcchhcCCCCcchHHH |
|  | Q Q6ZXV5 | 2 | ANINLKEITLIVGVVTACYWNS-LFCGF---VFDDVSAILDNKDLHPSTPLKTLFQNDFWGTPMSEERSHKSYRPLTVLT   77 (426) |
|  | Q Consensus | 2 | ~~~~~~~~~~l~~~~~~~~~~~-~~~~~---~~Dd~~~~~~~~~~~~~~~~~~~~~~~~~~~~~~~~~~~~~~~Pl~~~~   77 (426) |
|  |  |  | +......+++++++++.++... ...+. ..||..+...++++.+++.....-..+.......+......++|.+.++ |
|  | T Consensus | 14 | ~~~~~~~l~~~~~~~~~~~~~~~~~~~~~~~~~D~~~~~~~a~~~~~~g~~~~~~~~~~~~~~~~g~~~~~~~~p~~~~l   93 (718) |
|  | T 6EZN\_F | 14 | TILKLVIFVAIFGAAISSRLFAVIKFESIIHEFDPWFNYRATKYLVNNSFYKFLNWFDDRTWYPLGRVTGGTLYPGLMTT   93 (718) |
|  | T ss\_dssp |  | HHHHHHHHHHHHHHHHHTTTTTTTTTCCCCCSSSHHHHHHHHHHHHHSCHHHHHSCCCTTSSTTTCCCSSSSCCTTHHHH |
|  | T ss\_pred |  | HHHHHHHHHHHHHHHHHHHHHHHHHchhhhcccChHHHHHHHHHHHHcccHHHhcccCCcccCCCCCCCCCCCChHHHHH |
|  |
|  |
|  | Q ss\_pred |  | HHHHHHH----hC---CCchHHHHHHHHHHHHHHHHHHHHHHHhcCCHHHHHHHHHHHHCcccHHHHHHhhccHHHHHHH |
|  | Q Q6ZXV5 | 78 | FRLNYLL----SE---LKPMSYHLLNMIFHAVVSVIFLKVCKLFLDNKSSVIASLLFAVHPIHTEAVTGVVGRAELLSSI   150 (426) |
|  | Q Consensus | 78 | ~~~~~~l----fg---~~~~~~rl~~~l~~~~~~~~~y~l~~~~~~~~~a~~aall~~~~p~~~~~~~~~~~~~~~~~~~   150 (426) |
|  |  |  | .+..+.+ +| ......|+.++++++++++++|.++|++++++.|++++++++++|.+..........+|.+..+ |
|  | T Consensus | 94 | ~a~~~~l~~~~~G~~~~~~~~~~~~~~l~~~l~~~~~y~l~~~l~~~~~a~~aa~l~~~~p~~~~~~~~g~~~~~~~~~~   173 (718) |
|  | T 6EZN\_F | 94 | SAFIWHALRNWLGLPIDIRNVCVLFAPLFSGVTAWATYEFTKEIKDASAGLLAAGFIAIVPGYISRSVAGSYDNEAIAIT   173 (718) |
|  | T ss\_dssp |  | HHHHHHCCCCCSSCCCCHHHHHHBTHHHHHHHHHHHHHHHHHHHSCHHHHHHHHHHHHHCHHHHSSSCSBCCSSSTTTHH |
|  | T ss\_pred |  | HHHHHHHHHHHhCCCCCHHHHHHHHHHHHHHHHHHHHHHHHHHhcChHHHHHHHHHHHHHHHHHHhHhccccchHHHHHH |
|  |
|  |
|  | Q ss\_pred |  | HHHHHHHHHHHcCCCCCcccHHHHHHHHHHHHHHHHhHhHHHHHHHHHHHHHHHHhcCCCccchhcccchhhcCCCCCCh |
|  | Q Q6ZXV5 | 151 | FFLAAFLSYTRSKGPDNSIIWTPIALTVFLVAVATLCKEQGITVVGICCVYEVFIAQGYTLPLLCTTAGQFLRGKGSIPF   230 (426) |
|  | Q Consensus | 151 | ~~~l~~~~~~~~~~~~~~~~~~~~~~~~~~~~la~~~k~~~~~~~~~~~~~~~~~~~~~~~~~~~~~~~~~~~~~~~~~~   230 (426) |
|  |  |  | +.+++++++.+..++++ +++.++++++.+++.++|+.+.++.+++.+..+..... +++..+. |
|  | T Consensus | 174 | ~~~~~l~~~~~~~~~~~---~~~~~l~gl~~~l~~~~~~~~~~~~~~~~~~~~~~~~~---------------~~~~~~~   235 (718) |
|  | T 6EZN\_F | 174 | LLMVTFMFWIKAQKTGS---IMHATCAALFYFYMVSAWGGYVFITNLIPLHVFLLILM---------------GRYSSKL   235 (718) |
|  | T ss\_dssp |  | HHHHHHHHHHHHHHHCC---HHHHHHHHHHHHHHHTTCTTGGGGGGTHHHHHHHHHHT---------------TCCCHHH |
|  | T ss\_pred |  | HHHHHHHHHHHHhhcCC---HHHHHHHHHHHHHHHHhccchHHHHHHHHHHHHHHHHc---------------cCCChhH |
|  |
|  |
|  | Q ss\_pred |  | HHHHHHHHHHHHHHHHHHHHH----------------------------------------------------------- |
|  | Q Q6ZXV5 | 231 | SMLQTLVKLIVLMFSTLLLVV-----------------------------------------------------------   251 (426) |
|  | Q Consensus | 231 | ~~~~~~~~~~~~~~~~~~~~~-----------------------------------------------------------   251 (426) |
|  |  |  | .........+...+....... |
|  | T Consensus | 236 | ~~~~~~~~~~~~~~~~~~p~~~~~~~~~~~~~~~~~~~~~~~~~~~~~~~~~~~~~~~~~~~~~~~~~~~~~~~~~~~~~   315 (718) |
|  | T 6EZN\_F | 236 | YSAYTTWYAIGTVASMQIPFVGFLPIRSNDHMAALGVFGLIQIVAFGDFVKGQISTAKFKVIMMVSLFLILVLGVVGLSA   315 (718) |
|  | T ss\_dssp |  | HHHHHHHHHHHHHHTTCSTTSSSHHHHCTTSHHHHHHHHHHHHHHHHHHHHTTSCHHHHTTTC----------------- |
|  | T ss\_pred |  | HHHHHHHHHHHHHHHhcchhccCCccCchHHHHHHHHHHHHHHHHHHHHHHhcCChHHHHHHHHHHHHHHHHHHHHHHHH |
|  |
|  |
|  | Q ss\_pred |  | ----HHHHHHccCCCccccCCCcccCCCchhhHHhHhhHHHHHHHHHhccccccccCccccCccccccccHHHHHHHHHH |
|  | Q Q6ZXV5 | 252 | ----IRVQVIQSQLPVFTRFDNPAAVSPTPTRQLTFNYLLPVNAWLLLNPSELCCDWTMGTIPLIESLLDIRNLATFTFF   327 (426) |
|  | Q Consensus | 252 | ----~~~~~~~~~~~~~~~~~~~~~~~~~~~~~~~~~~~~~~~~~~~~~~~~~~~~~~~~~~~~~~~~~~~~~~~~~~~~   327 (426) |
|  |  |  | ............................ ................... |
|  | T Consensus | 316 | ~~~~~~~~~~~~~~~~~~~~~~~~~~~~~~~~-----------------------------~~~~~~~~~~~~~~~~~~~   366 (718) |
|  | T 6EZN\_F | 316 | LTYMGLIAPWTGRFYSLWDTNYAKIHIPIIAS-----------------------------VSEHQPVSWPAFFFDTHFL   366 (718) |
|  | T ss\_dssp |  | -----------------------------------------------------------------CCCCHHHHHHHSSST |
|  | T ss\_pred |  | HHHhhhhhhhhHHHHHhhcccccccCCCcccc-----------------------------hhHhCCCCHHHHHhhhhHH |
|  |
|  |
|  | Q ss\_pred |  | HHHHHHHHHHHHccCCCchhHHHHHHHHHHHHHHHhccCCCcchhhchhhchHHHHHHHHHHHHHHHHhcccchHHH--- |
|  | Q Q6ZXV5 | 328 | CFLGMLGVFSIRYSGDSSKTVLMALCLMALPFIPASNLFFPVGFVVAERVLYVPSMGFCILVAHGWQKISTKSVFKK---   404 (426) |
|  | Q Consensus | 328 | ~~~~~~~~~~~~~~~~~~~~~~~~~~~~~~~~~~~~~~~~~~~~~~~~ry~~~~~~~~~il~~~~~~~~~~~~~~~~---   404 (426) |
|  |  |  | ..++..+.....+++++................... .||..+..|+++++++.++..+.++.+.++ |
|  | T Consensus | 367 | ~~l~~~g~~~~~~~~~~~~~~~~~~~~~~~~~~~~~-----------~R~~~~~~p~~~il~a~~l~~l~~~~~~~~~~~   435 (718) |
|  | T 6EZN\_F | 367 | IWLFPAGVFLLFLDLKDEHVFVIAYSVLCSYFAGVM-----------VRLMLTLTPVICVSAAVALSKIFDIYLDFKTSD   435 (718) |
|  | T ss\_dssp |  | TTTHHHHHHHHHTTCCSSHHHHHHHHHHHHHHHHHC-----------STTHHHHHHHHHHHHHHHHHHHHHHSCCCC--- |
|  | T ss\_pred |  | HHHHHHHHHHHHhcCChHHHHHHHHHHHHHHHHHHH-----------HHHHHHHHHHHHHHHHHHHHHHHHHHhccccCC |
|  |
|  |
|  | Q ss\_pred |  | ----------HHHHHHHHHHHHHHHHhhhh |
|  | Q Q6ZXV5 | 405 | ----------LSWICLSMVILTHSLKTFHR   424 (426) |
|  | Q Consensus | 405 | ----------~~~~~~~~~~~~~~~~~~~~   424 (426) |
|  |  |  | .....+++++++........ |
|  | T Consensus | 436 | ~~~~~~~~~~~~~~~~~~~~~~~~~~~~~~   465 (718) |
|  | T 6EZN\_F | 436 | RKYAIKPAALLAKLIVSGSFIFYLYLFVFH   465 (718) |
|  | T ss\_dssp |  | ----CCHHHHHHHHHHHHHHHHHHHHHHHH |
|  | T ss\_pred |  | ccccccchHHHHHHHHHHHHHHHHHHHHHH |
|  |
| --- | | | |
|  | Template alignmentTemplate 3D StructurePDBe | | |
| 5. | 6S7O\_A Dolichyl-diphosphooligosaccharide--protein glycosyltransferase subunit STT3A (E.C.2.4.99.18); N-glycosylation, Oligosaccharyltransferase, OSTA, TRANSFERASE; HET: KZB, NAG, EGY, MAN, KZE, BMA; 3.5A {Homo sapiens}; Related PDB entries: 6FTI\_5 6FTG\_5 6FTJ\_5; Related PDB entries: 6FTG\_5 6FTI\_5 6FTJ\_5; Related PDB entries: 6FTG\_5 6FTI\_5 6FTJ\_5 | | |
|  | Probability: 99.82%, E-value: 3.4e-17, Score: 157.26, Aligned cols: 374, Identities: 11%, Similarity: 0.012, | | |
|  |
|  | Q ss\_pred |  | CcchhHHHHHHHHHHHHHHHHHhhC------CCceeccHHHHhcCCCCCCCCcHHHHhccccCCCCCCcchhcCCCCcch |
|  | Q Q6ZXV5 | 1 | MANINLKEITLIVGVVTACYWNSLF------CGFVFDDVSAILDNKDLHPSTPLKTLFQNDFWGTPMSEERSHKSYRPLT   74 (426) |
|  | Q Consensus | 1 | ~~~~~~~~~~~l~~~~~~~~~~~~~------~~~~~Dd~~~~~~~~~~~~~~~~~~~~~~~~~~~~~~~~~~~~~~~Pl~   74 (426) |
|  |  |  | .++......++++++.+........ .....||..+...+.++.+++........+.......+......++|++ |
|  | T Consensus | 13 | ~~~~~~~~~~l~~~~~~~~~~~~~~~~~~~~~~~~~D~~~~~~~a~~~~~~g~~~~~~~~~~~~~~~~g~~~~~~~~p~~   92 (705) |
|  | T 6S7O\_A | 13 | KQDTLLKLLILSMAAVLSFSTRLFAVLRFESVIHEFDPYFNYRTTRFLAEEGFYKFHNWFDDRAWYPLGRIIGGTIYPGL   92 (705) |
|  | T ss\_dssp |  | HHHHHHHHHHHHHHHHHHHHHHCSHHHHSCCCCCSSSHHHHHHHHHHHHHHCSHHHHSCEECSSSTTSCEEHHHHSCCHH |
|  | T ss\_pred |  | HHHHHHHHHHHHHHHHHHHHHHHHHHHhcccccccCChHHHHHHHHHHHHhCChhhhccccccccCCCcCCCCCcCCchH |
|  |
|  |
|  | Q ss\_pred |  | HHHHHHHHHHhC------CCchHHHHHHHHHHHHHHHHHHHHHHHhcCCHHHHHHHHHHHHCcccHHHHHHhhccHHHHH |
|  | Q Q6ZXV5 | 75 | VLTFRLNYLLSE------LKPMSYHLLNMIFHAVVSVIFLKVCKLFLDNKSSVIASLLFAVHPIHTEAVTGVVGRAELLS   148 (426) |
|  | Q Consensus | 75 | ~~~~~~~~~lfg------~~~~~~rl~~~l~~~~~~~~~y~l~~~~~~~~~a~~aall~~~~p~~~~~~~~~~~~~~~~~   148 (426) |
|  |  |  | .++.+..+.++| ++....|+.++++++++++++|.++|++.+++.|++++++++++|.+.........++|.+. |
|  | T Consensus | 93 | ~~~~~~~~~l~g~~g~~~~~~~~~~~~~~l~~~l~~~~~y~l~~~~~~~~~al~aa~l~~~~p~~~~~~~~~~~~~~~~~   172 (705) |
|  | T 6S7O\_A | 93 | MITSAAIYHVLHFFHITIDIRNVCVFLAPLFSSFTTIVTYHLTKELKDAGAGLLAAAMIAVVPGYISRSVAGSYDNEGIA   172 (705) |
|  | T ss\_dssp |  | HHHHHHHHHHHHHTTCCCCHHHHHHTHHHHHHHHHHHHHHHHHHHHSCHHHHHHHHHHHHSCHHHHHSSCTTCCCHHHHH |
|  | T ss\_pred |  | HHHHHHHHHHHHHcCCCCCHHHHHHHHHHHHHHHHHHHHHHHHHHHcCHHHHHHHHHHHHHHHHHHHhhcccchhHHHHH |
|  |
|  |
|  | Q ss\_pred |  | HHHHHHHHHHHHHcCCCCCcccHHHHHHHHHHHHHHHHhHhHHHHHHHHHHHHHHHHhcCCCccchhcccchhhcCCCCC |
|  | Q Q6ZXV5 | 149 | SIFFLAAFLSYTRSKGPDNSIIWTPIALTVFLVAVATLCKEQGITVVGICCVYEVFIAQGYTLPLLCTTAGQFLRGKGSI   228 (426) |
|  | Q Consensus | 149 | ~~~~~l~~~~~~~~~~~~~~~~~~~~~~~~~~~~la~~~k~~~~~~~~~~~~~~~~~~~~~~~~~~~~~~~~~~~~~~~~   228 (426) |
|  |  |  | .++.+++++++.+..++++ .++.++++++.+++.++|+.+....++..+..+..... ++.. |
|  | T Consensus | 173 | ~~~~~~~~~~~~~~~~~~~---~~~~~~~gl~~~l~~~~~~~~~~~~~~~~~~~~~~~~~----------------~~~~   233 (705) |
|  | T 6S7O\_A | 173 | IFCMLLTYYMWIKAVKTGS---ICWAAKCALAYFYMVSSWGGYVFLINLIPLHVLVLMLT----------------GRFS   233 (705) |
|  | T ss\_dssp |  | HHHHHHHHHHHHHHHHHCC---HHHHHHHHHHHHHHHHHCTTHHHHTTTHHHHHHHHHHH----------------TCCC |
|  | T ss\_pred |  | HHHHHHHHHHHHHHHhhCC---HHHHHHHHHHHHHHHHhhchHHHHHHHHHHHHHHHHHh----------------cCCC |
|  |
|  |
|  | Q ss\_pred |  | Ch---------------------------------------------------------HHHHHHHHHHHHHHHHHHHHH |
|  | Q Q6ZXV5 | 229 | PF---------------------------------------------------------SMLQTLVKLIVLMFSTLLLVV   251 (426) |
|  | Q Consensus | 229 | ~~---------------------------------------------------------~~~~~~~~~~~~~~~~~~~~~   251 (426) |
|  |  |  | ++ +..+................. |
|  | T Consensus | 234 | ~~~~~~~~~~~~~~~~~~~~~~~~~~~~~~~~~~~~~~~~~~~~~~~~~~~~~~~~~~~~~~~~~~~~~~~~~~~~~~~~   313 (705) |
|  | T 6S7O\_A | 234 | HRIYVAYCTVYCLGTILSMQISFVGFQPVLSSEHMAAFGVFGLCQIHAFVDYLRSKLNPQQFEVLFRSVISLVGFVLLTV   313 (705) |
|  | T ss\_dssp |  | HHHHHHHHHHHHHHHHHHHTTSSSTTHHHHSSTTHHHHHHHHHHHHHHHHHHHHHHSCHHHHHHHC-------------- |
|  | T ss\_pred |  | hHHHHHHHHHHHHHHHHHHhccccCCcccCCHHHHHHHHHHHHHHHHHHHHHHHHcCCHHHHHHHHHHHHHHHHHHHHHH |
|  |
|  |
|  | Q ss\_pred |  | HHHHHHccCCCccccCCCcccCCCchhhHHhHhhHHHHHHHHHhccccccccCccccCccccccccHHHHHHHHHHHHHH |
|  | Q Q6ZXV5 | 252 | IRVQVIQSQLPVFTRFDNPAAVSPTPTRQLTFNYLLPVNAWLLLNPSELCCDWTMGTIPLIESLLDIRNLATFTFFCFLG   331 (426) |
|  | Q Consensus | 252 | ~~~~~~~~~~~~~~~~~~~~~~~~~~~~~~~~~~~~~~~~~~~~~~~~~~~~~~~~~~~~~~~~~~~~~~~~~~~~~~~~   331 (426) |
|  |  |  | .................................... ...................++.+ |
|  | T Consensus | 314 | ~~~~~~~~~~~~~~~~~~~~~~~~~~~~~~~~~~~~---------------------~~~~~~~~~~~~~~~~~~~~l~~   372 (705) |
|  | T 6S7O\_A | 314 | GALLMLTGKISPWTGRFYSLLDPSYAKNNIPIIASV---------------------SEHQPTTWSSYYFDLQLLVFMFP   372 (705) |
|  | T ss\_dssp |  | --------CCCCCCSTTHHHHSTTHHHHTCTTTTTS---------------------GGGSCCCHHHHHHHCSSSGGGHH |
|  | T ss\_pred |  | HHHHHHccccccccHHHHHhhChhHhcCCCCceeec---------------------cccCCCCHHHHHHHHHHHHHHHH |
|  |
|  |
|  | Q ss\_pred |  | HHHHHHHHccCCCchhHHHHHHHHHHHHHHHhccCCCcchhhchhhchHHHHHHHHHHHHHHHHhcccchHHHH------ |
|  | Q Q6ZXV5 | 332 | MLGVFSIRYSGDSSKTVLMALCLMALPFIPASNLFFPVGFVVAERVLYVPSMGFCILVAHGWQKISTKSVFKKL------   405 (426) |
|  | Q Consensus | 332 | ~~~~~~~~~~~~~~~~~~~~~~~~~~~~~~~~~~~~~~~~~~~~ry~~~~~~~~~il~~~~~~~~~~~~~~~~~------   405 (426) |
|  |  |  | .+.....+++++.........+......... .||..+..|+++++++.++..+.++...+.. |
|  | T Consensus | 373 | ~~~~~~~~~~~~~~~~~~~~~~~~~~~~~~~------------~R~~~~~~p~~~l~~a~~l~~l~~~~~~~~~~~~~~~   440 (705) |
|  | T 6S7O\_A | 373 | VGLYYCFSNLSDARIFIIMYGVTSMYFSAVM------------VRLMLVLAPVMCILSGIGVSQVLSTYMKNLDISRPDK   440 (705) |
|  | T ss\_dssp |  | HHHHHHHHSCCTTHHHHHHHHHHHHHHHHHC------------SGGGGGGHHHHHHHHHHHHHHHHHHHTTTSSSCC--- |
|  | T ss\_pred |  | HHHHHHhcCCChhHHHHHHHHHHHHHHHHHh------------HHHHHHHHHHHHHHHHHHHHHHHHHHHhhcCCCCCCc |
|  |
|  |
|  | Q ss\_pred |  | ----------------HHHHHHHHHHHHHHHhhhhcC |
|  | Q Q6ZXV5 | 406 | ----------------SWICLSMVILTHSLKTFHRNW   426 (426) |
|  | Q Consensus | 406 | ----------------~~~~~~~~~~~~~~~~~~~~~   426 (426) |
|  |  |  | ....++++++..........+ |
|  | T Consensus | 441 | ~~~~~~~~~~~~~~~~~~~~~~~~~~~~~~~~~~~~~   477 (705) |
|  | T 6S7O\_A | 441 | KSKKQQDSTYPIKNEVASGMILVMAFFLITYTFHSTW   477 (705) |
|  | T ss\_dssp |  | ------------CCHHHHHHHHHHHHHHHHHHHHHHH |
|  | T ss\_pred |  | cCccccCCCCCcCHHHHHHHHHHHHHHHHHHHHhHhH |
|  |
| --- | | | |
|  | Template alignmentTemplate 3D StructurePDBe | | |
| 6. | 5OGL\_A Peptide-binding protein, Substrate mimicking peptide; Oligosaccharyltransferase, Complex, Protein N-glycosylation, Bacteria; HET: 9UB, PPN; 2.7A {Campylobacter lari (strain RM2100 / D67 / ATCC BAA-1060)}; Related PDB entries: 3RCE\_A 6GXC\_A; Related PDB entries: 6GXC\_A 3RCE\_A ; Related PDB entries: 6GXC\_A 3RCE\_A | | |
|  | Probability: 99.77%, E-value: 4.5e-16, Score: 149.62, Aligned cols: 371, Identities: 10%, Similarity: 0.035, | | |
|  |
|  | Q ss\_pred |  | CcchhHHHHHHHHHHHHHHHH-----HhhCCCceeccHHHHhcCCCCCCCCcHHHHhccccCCCCCCcchhcCCCCcchH |
|  | Q Q6ZXV5 | 1 | MANINLKEITLIVGVVTACYW-----NSLFCGFVFDDVSAILDNKDLHPSTPLKTLFQNDFWGTPMSEERSHKSYRPLTV   75 (426) |
|  | Q Consensus | 1 | ~~~~~~~~~~~l~~~~~~~~~-----~~~~~~~~~Dd~~~~~~~~~~~~~~~~~~~~~~~~~~~~~~~~~~~~~~~Pl~~   75 (426) |
|  |  |  | .++.....+++++++++.++. ..-..++..||.......+.........+..++..... ....++|+.. |
|  | T Consensus | 12 | ~~~~~~~~l~~i~~~~~~lRl~~~~~~~~~~~~~~~~~~~~~~~D~~~~~~~a~~~~~~~~~~~------~~~~~~p~~~   85 (713) |
|  | T 5OGL\_A | 12 | SIKYTAILILIAFAFSVLARLYWVAWASEFYEFFFNDQLMITTNDGYAFAEGARDMIAGFHQPN------DLSYFGSSLS   85 (713) |
|  | T ss\_dssp |  | CHHHHHHHHHHHHHHHHHHHHHHHHHHTTCGGGEETTEECCSSTTHHHHHHHHHHHHHTCCCTT------SCCCTTCHHH |
|  | T ss\_pred |  | cHHHHHHHHHHHHHHHHHHHHHHHHHhhccchhhcCCEEeeccccHHHHHHHHHHHHcCCCCCC------CcchhcchHH |
|  |
|  |
|  | Q ss\_pred |  | HHHHHHHHHhC-CCchHHHHHHHHHHHHHHHHHHHHHHHhcCCHHHHHHHHHHHHCcccHHHHHHhhccHHHHHHHHHHH |
|  | Q Q6ZXV5 | 76 | LTFRLNYLLSE-LKPMSYHLLNMIFHAVVSVIFLKVCKLFLDNKSSVIASLLFAVHPIHTEAVTGVVGRAELLSSIFFLA   154 (426) |
|  | Q Consensus | 76 | ~~~~~~~~lfg-~~~~~~rl~~~l~~~~~~~~~y~l~~~~~~~~~a~~aall~~~~p~~~~~~~~~~~~~~~~~~~~~~l   154 (426) |
|  |  |  | ++.+....++| ..+...|++++++++++++.+|.++|++++++.|++++++++++|.++.........+|.+..++.++ |
|  | T Consensus | 86 | ~l~~~~~~l~g~~~~~~~~~~~~l~~~l~v~~~y~l~r~l~~~~~al~aall~a~~p~~~~~s~~g~~~~d~~~~~~~~l   165 (713) |
|  | T 5OGL\_A | 86 | TLTYWLYSILPFSFESIILYMSTFFASLIVVPIILIAREYKLTTYGFIAALLGSIANSYYNRTMSGYYDTDMLVLVLPML   165 (713) |
|  | T ss\_dssp |  | HHHHHHHHHSCSCHHHHHHHHHHHHGGGGHHHHHHHHHHTTCHHHHHHHHHHHHHCHHHHHTTSTTCCSGGGGTTHHHHH |
|  | T ss\_pred |  | HHHHHHHHhCCCCHHHHHHHHHHHHHHHHHHHHHHHHHHhCCchHHHHHHHHHHHHHHHHHhhccccCchHHHHHHHHHH |
|  |
|  |
|  | Q ss\_pred |  | HHHHHHHcCCCCCcccHHHHHHHHHHHHHHHHhHhHHH----HHHHHHHHHHHHHhcCCCccchhcccchhhcCCCC--- |
|  | Q Q6ZXV5 | 155 | AFLSYTRSKGPDNSIIWTPIALTVFLVAVATLCKEQGI----TVVGICCVYEVFIAQGYTLPLLCTTAGQFLRGKGS---   227 (426) |
|  | Q Consensus | 155 | ~~~~~~~~~~~~~~~~~~~~~~~~~~~~la~~~k~~~~----~~~~~~~~~~~~~~~~~~~~~~~~~~~~~~~~~~~---   227 (426) |
|  |  |  | +++++.+..++++ .++.++++++.+++.++|..+. .+.+...++.+..+++ ++. |
|  | T Consensus | 166 | ~~~~~~~~~~~~~---~~~~~l~gl~~~l~~~~~~~~~~~~~~~~~~~~~~~~~~~~~----------------~~~~~~   226 (713) |
|  | T 5OGL\_A | 166 | ILLTFIRLTINKD---IFTLLLSPVFIMIYLWWYPSSYSLNFAMIGLFGLYTLVFHRK----------------EKIFYL   226 (713) |
|  | T ss\_dssp |  | HHHHHHHHHHHCC---TTHHHHHHHHHHHHHHHCGGGHHHHHHHHHHHHHHHHHHTTT----------------CHHHHH |
|  | T ss\_pred |  | HHHHHHHHHcCCc---hHHHHHHHHHHHHHHhhccchHHHHHHHHHHHHHHHHHhCCc----------------chHHHH |
|  |
|  |
|  | Q ss\_pred |  | ------------------------------CChHHHHHHHHHHHHHHHHHHHHHHHHHHHccCCCccccCCCcccCCCch |
|  | Q Q6ZXV5 | 228 | ------------------------------IPFSMLQTLVKLIVLMFSTLLLVVIRVQVIQSQLPVFTRFDNPAAVSPTP   277 (426) |
|  | Q Consensus | 228 | ------------------------------~~~~~~~~~~~~~~~~~~~~~~~~~~~~~~~~~~~~~~~~~~~~~~~~~~   277 (426) |
|  |  |  | ..+..+.........+....................+............. |
|  | T Consensus | 227 | ~~~~~~~~~~~~~~~~~~~~~~~~~~~~~~~~~~~~~~~~~~~~~~~~~~~~~~~~~~~~~~~~~~~~~~~~~~~~~~~~   306 (713) |
|  | T 5OGL\_A | 227 | TIALMIIALSMLAWQYKLALIVLLFAIFAFKEEKINFYMIWALIFISILILHLSGGLDPVLYQLKFYVFKASDVQNLKDA   306 (713) |
|  | T ss\_dssp |  | HHHHHHHHHSCCCHHHHHHHHHHHHHHHHHCSSCCCHHHHHHHHHHHHHHHHHTTTTHHHHHHHHHHTSCCSCCSCCTTT |
|  | T ss\_pred |  | HHHHHHHHHhhhhHHHHHHHHHHHHHHHHcchhccchHHHHHHHHHHHHHHHHhccchHHHHHHHHHhhccccccccccc |
|  |
|  |
|  | Q ss\_pred |  | hhHHhHhhHHHHHHHHHhccccccccCccccCccccccccHHHHHHHHHHHHHHHHHHHHHHccCCCchhHHHHHHHHHH |
|  | Q Q6ZXV5 | 278 | TRQLTFNYLLPVNAWLLLNPSELCCDWTMGTIPLIESLLDIRNLATFTFFCFLGMLGVFSIRYSGDSSKTVLMALCLMAL   357 (426) |
|  | Q Consensus | 278 | ~~~~~~~~~~~~~~~~~~~~~~~~~~~~~~~~~~~~~~~~~~~~~~~~~~~~~~~~~~~~~~~~~~~~~~~~~~~~~~~~   357 (426) |
|  |  |  | .............. .+..................+.+.+.....++ ++...+.+.++++ |
|  | T Consensus | 307 | ~~~~~~~~~~~~~~-----------------~~~~~~~~~~~~~~~~~~~~l~~~gl~~~~~~----~~~~~~~l~~~~~   365 (713) |
|  | T 5OGL\_A | 307 | AFMYFNVNETIMEV-----------------NTIDPEVFMQRISSSVLVFILSFIGFILLLKD----HKSMLLALPMLAL   365 (713) |
|  | T ss\_dssp |  | SCCCCCGGGGBGGG-----------------CCCCHHHHHHHHHSSHHHHHHHHHHHHHHHTT----CGGGGGGHHHHHH |
|  | T ss\_pred |  | ccccccHHHHHHHh-----------------cCCCHHHHHHHhcccHHHHHHHHHHHHHHHHc----CHhHHHHHHHHHH |
|  |
|  |
|  | Q ss\_pred |  | HHHHHhccCCCcchhhchhhchHHHHHHHHHHHHHHHHhcccchHH--------HHHHHHHHHHHHHHHHHhhhhcC |
|  | Q Q6ZXV5 | 358 | PFIPASNLFFPVGFVVAERVLYVPSMGFCILVAHGWQKISTKSVFK--------KLSWICLSMVILTHSLKTFHRNW   426 (426) |
|  | Q Consensus | 358 | ~~~~~~~~~~~~~~~~~~ry~~~~~~~~~il~~~~~~~~~~~~~~~--------~~~~~~~~~~~~~~~~~~~~~~~   426 (426) |
|  |  |  | .+.. .....||..+..|+++++++.++..+.++.+.+ +....++++++..........++ |
|  | T Consensus | 366 | ~~~~---------~~~~~R~~~~~~p~~~i~~a~~l~~l~~~~~~~~~~~~~~~~~~~~~~~~~~~~~~~~~~~~~~   433 (713) |
|  | T 5OGL\_A | 366 | GFMA---------LRAGLRFTIYAVPVMALGFGYFLYAFFNFLEKKQIKLSLRNKNILLILIAFFSISPALMHIYYY   433 (713) |
|  | T ss\_dssp |  | HHHH---------HHHCGGGGGGGHHHHHHHHHHHHHHHHHHHHHTTCCCCHHHHHHHHHHHHHHHHHHHHHHHHHC |
|  | T ss\_pred |  | HHHH---------HHHhHHHHHHHHHHHHHHHHHHHHHHHHHHHHhhcccchhHHHHHHHHHHHHHHhHHHHHHHhc |
|  |
| --- | | | |
|  | Template alignmentTemplate 3D StructurePDBe | | |
| 7. | 6P25\_A Dolichyl-diphosphooligosaccharide--protein glycosyltransferase subunits (E.C.2.4.99.18); complex, TRANSFERASE, glycosylation; HET: NAG, CPL, NNM; 3.2A {Saccharomyces cerevisiae W303}; Related PDB entries: 6P2R\_A ; Related PDB entries: 6P2R\_A ; Related PDB entries: 6P2R\_A | | |
|  | Probability: 99.66%, E-value: 6.3e-14, Score: 135.09, Aligned cols: 224, Identities: 10%, Similarity: 0.044, | | |
|  |
|  | Q ss\_pred |  | CcchhHHHHHHHHHHHHHHHHHhhCCC--ceeccHHHHhcCCCCCCCCcHHHHhccccCCCCCCcchhcCCCCcchHHHH |
|  | Q Q6ZXV5 | 1 | MANINLKEITLIVGVVTACYWNSLFCG--FVFDDVSAILDNKDLHPSTPLKTLFQNDFWGTPMSEERSHKSYRPLTVLTF   78 (426) |
|  | Q Consensus | 1 | ~~~~~~~~~~~l~~~~~~~~~~~~~~~--~~~Dd~~~~~~~~~~~~~~~~~~~~~~~~~~~~~~~~~~~~~~~Pl~~~~~   78 (426) |
|  |  |  | .+......+++++++++.++...+..+ ..+||..+. .....+..... +.+.+||++.++. |
|  | T Consensus | 45 | ~~~~~~~~l~~l~ll~~~lrl~~l~~~~~~~~DE~~~~---------~~a~~~~~g~~---------~~~~~PPL~~ll~   106 (817) |
|  | T 6P25\_A | 45 | VTLKEKLLVACLAVFTAVIRLHGLAWPDSVVFDEVHFG---------GFASQYIRGTY---------FMDVHPPLAKMLY   106 (817) |
|  | T ss\_dssp |  | CSHHHHHHHHHHHHHHHHHTTTTTTSSCBCCTTHHHHH---------HHHHHHHHCBC---------CCCSSCTHHHHHH |
|  | T ss\_pred |  | CcHHHHHHHHHHHHHHHHHHHhcccCCCcceeeHHHHH---------HHHHHHHhCCC---------CCCCCChHHHHHH |
|  |
|  |
|  | Q ss\_pred |  | HHHHHHhCCCc-----------------hHHHHHHHHHHHHHHHHHHHHHHHhc-CCHHHHHHHHHHHHCcccHHHHHHh |
|  | Q Q6ZXV5 | 79 | RLNYLLSELKP-----------------MSYHLLNMIFHAVVSVIFLKVCKLFL-DNKSSVIASLLFAVHPIHTEAVTGV   140 (426) |
|  | Q Consensus | 79 | ~~~~~lfg~~~-----------------~~~rl~~~l~~~~~~~~~y~l~~~~~-~~~~a~~aall~~~~p~~~~~~~~~   140 (426) |
|  |  |  | +....++|.+. ...|++++++++++++++|.++|+.. ++..|++++++++++|..+...... |
|  | T Consensus | 107 | a~~~~l~G~~~~~~f~~ig~~~~~~~~~~~~Rl~~~l~~~l~v~l~y~i~r~l~~~~~~Allaall~~~~p~~i~~s~~~   186 (817) |
|  | T 6P25\_A | 107 | AGVASLGGFQGDFDFENIGDSFPSTTPYVLMRFFSASLGALTVILMYMTLRYSGVRMWVALMSAICFAVENSYVTISRYI   186 (817) |
|  | T ss\_dssp |  | HHHHHHTCCCSCCCCCSTTCBCCTTSCCHHHHHHHHHHHHHHHHHHHHHHHHTTCCHHHHHHHHHHHHSCHHHHHHHHSS |
|  | T ss\_pred |  | HHHHHHcCCCCCCCccccccCCCCCCHHHHHHHHHHHHHHHHHHHHHHHHHHcCCCHHHHHHHHHHHHHcHHHHHHHHHH |
|  |
|  |
|  | Q ss\_pred |  | hccHHHHHHHHHHHHHHHHHHcCCCCC--cccHHHHHHHHHHHHHHHHhHhHHHHHHHHHHHHHHHHhcCCCccchhccc |
|  | Q Q6ZXV5 | 141 | VGRAELLSSIFFLAAFLSYTRSKGPDN--SIIWTPIALTVFLVAVATLCKEQGITVVGICCVYEVFIAQGYTLPLLCTTA   218 (426) |
|  | Q Consensus | 141 | ~~~~~~~~~~~~~l~~~~~~~~~~~~~--~~~~~~~~~~~~~~~la~~~k~~~~~~~~~~~~~~~~~~~~~~~~~~~~~~   218 (426) |
|  |  |  | +.|.+..++++++++++.+..++++ ++++++++++++++++++.+|+.++++++.++++.++...+ |
|  | T Consensus | 187 | --~~d~~~~ff~~lal~~~~~~~~~~~~~~~~~~~l~l~gl~lgla~~tK~~gl~~l~~~~l~~l~~l~~----------   254 (817) |
|  | T 6P25\_A | 187 | --LLDAPLMFFIAAAVYSFKKYEMYPANSLNAYKSLLATGIALGMASSSKWVGLFTVTWVGLLCIWRLWF----------   254 (817) |
|  | T ss\_dssp |  | --CSHHHHHHHHHHHHHHHHHHHTSCSSSHHHHHHHHHHHHHHHHHHTTCTTHHHHHHHHHHHHHHHHHH---------- |
|  | T ss\_pred |  | --hhHHHHHHHHHHHHHHHHHHHhCCCCCcHHHHHHHHHHHHHHHHHhhhhHHHHHHHHHHHHHHHHHHH---------- |
|  |
|  |
|  | Q ss\_pred |  | chhhcCCCCCChHHHHHHHHHHHHHHHHHHHHHHHHHHH |
|  | Q Q6ZXV5 | 219 | GQFLRGKGSIPFSMLQTLVKLIVLMFSTLLLVVIRVQVI   257 (426) |
|  | Q Consensus | 219 | ~~~~~~~~~~~~~~~~~~~~~~~~~~~~~~~~~~~~~~~   257 (426) |
|  |  |  | ..++.....+...........++++++..+...... |
|  | T Consensus | 255 | ---~~~~~~~~~~~~~~~~~~~~~~li~ip~~iy~~~~~   290 (817) |
|  | T 6P25\_A | 255 | ---MIGDLTKSSKSIFKVAFAKLAFLLGVPFALYLVFFY   290 (817) |
|  | T ss\_dssp |  | ---HHHCSSSCHHHHHHHHHHHHCCCCCHHHHHHHHHHH |
|  | T ss\_pred |  | ---HhCCCCCCHHHHHHHHHHHHHHHHHHHHHHHHHHHH |
|  |
| --- | | | |
|  | Template alignmentTemplate 3D StructurePDBe | | |
| 8. | 6P25\_B Dolichyl-diphosphooligosaccharide--protein glycosyltransferase subunits (E.C.2.4.99.18); complex, TRANSFERASE, glycosylation; HET: NAG, CPL, NNM; 3.2A {Saccharomyces cerevisiae W303}; Related PDB entries: 6P2R\_B ; Related PDB entries: 6P2R\_B ; Related PDB entries: 6P2R\_B | | |
|  | Probability: 99.65%, E-value: 8.6e-14, Score: 134.33, Aligned cols: 241, Identities: 12%, Similarity: 0.095, | | |
|  |
|  | Q ss\_pred |  | CcchhHHHHHHHHHHHHHHHHHhhCCCce--eccHHHHhcCCCCCCCCcHHHHhccccCCCCCCcchhcCCCCcchHHHH |
|  | Q Q6ZXV5 | 1 | MANINLKEITLIVGVVTACYWNSLFCGFV--FDDVSAILDNKDLHPSTPLKTLFQNDFWGTPMSEERSHKSYRPLTVLTF   78 (426) |
|  | Q Consensus | 1 | ~~~~~~~~~~~l~~~~~~~~~~~~~~~~~--~Dd~~~~~~~~~~~~~~~~~~~~~~~~~~~~~~~~~~~~~~~Pl~~~~~   78 (426) |
|  |  |  | .+......+++++++++.++......+.. +||..+. ....++....+ +...+||+..++. |
|  | T Consensus | 60 | ~~~~~~~~l~~l~~~~~~~r~~~l~~~~~~~~DE~~~~---------~~a~~~~~~~~---------~~~~~PPl~~~l~   121 (759) |
|  | T 6P25\_B | 60 | LRLESVVMPVIFTALALFTRMYKIGINNHVVWDEAHFG---------KFGSYYLRHEF---------YHDVHPPLGKMLV   121 (759) |
|  | T ss\_dssp |  | HHHHHHHHHHHHHHHHHHHHSSSGGGSCBCCTTHHHHH---------HHHHHHHTTBC---------CCCSSCTHHHHHH |
|  | T ss\_pred |  | hHhHHhHHHHHHHHHHHHHHHHhcCCCCceeeeHHHHH---------HHHHHHHhCCC---------CcCCCCHHHHHHH |
|  |
|  |
|  | Q ss\_pred |  | HHHHHHhCCCc----------------hHHHHHHHHHHHHHHHHHHHHHHHh-cCCHHHHHHHHHHHHCcccHHHHHHhh |
|  | Q Q6ZXV5 | 79 | RLNYLLSELKP----------------MSYHLLNMIFHAVVSVIFLKVCKLF-LDNKSSVIASLLFAVHPIHTEAVTGVV   141 (426) |
|  | Q Consensus | 79 | ~~~~~lfg~~~----------------~~~rl~~~l~~~~~~~~~y~l~~~~-~~~~~a~~aall~~~~p~~~~~~~~~~   141 (426) |
|  |  |  | +....++|.+. ...|++++++++++++++|.++|++ .++..|++++++++++|..+...... |
|  | T Consensus | 122 | a~~~~l~g~~~~~~~~~~~~~~~~~~~~~~R~~~~l~~~l~~~l~y~l~r~l~~~~~~allaall~~~~p~~~~~s~~~-   200 (759) |
|  | T 6P25\_B | 122 | GLSGYLAGYNGSWDFPSGEIYPDYLDYVKMRLFNASFSALCVPLAYFTAKAIGFSLPTVWLMTVLVLFENSYSTLGRFI-   200 (759) |
|  | T ss\_dssp |  | HHHHHTTTCCSCSCCCSSCBCCSSCCHHHHHHHHHHHHHHHHHHHHHHHHHSCCCTHHHHHHHHHHHSCHHHHHHTSSS- |
|  | T ss\_pred |  | HHHHHHhCCCCCCCCCCCCCCCCcCCHHHHHHHHHHHHHHHHHHHHHHHHHcCCCHHHHHHHHHHHHhhhHHHHHHHHH- |
|  |
|  |
|  | Q ss\_pred |  | ccHHHHHHHHHHHHHHHHHHcCCCCCc----ccHHHHHHHHHHHHHHHHhHhHHHHHHHHHHHHHHHHhcCCCccchhcc |
|  | Q Q6ZXV5 | 142 | GRAELLSSIFFLAAFLSYTRSKGPDNS----IIWTPIALTVFLVAVATLCKEQGITVVGICCVYEVFIAQGYTLPLLCTT   217 (426) |
|  | Q Consensus | 142 | ~~~~~~~~~~~~l~~~~~~~~~~~~~~----~~~~~~~~~~~~~~la~~~k~~~~~~~~~~~~~~~~~~~~~~~~~~~~~   217 (426) |
|  |  |  | +.|.+..++++++++++.+..+++++ +++.+++++++++|+++.+|..++.+++.++++.+....+ |
|  | T Consensus | 201 | -~~d~~~~~f~~l~l~~~~~~~~~~~~~~~~~~~~~~~l~gl~lgla~~~K~~~~~~~~~~~l~~l~~~~~---------   270 (759) |
|  | T 6P25\_B | 201 | -LLDSMLLFFTVASFFSFVMFHNQRSKPFSRKWWKWLLITGISLGCTISVKMVGLFIITMVGIYTVIDLWT---------   270 (759) |
|  | T ss\_dssp |  | -CSHHHHHHHHHHHHHHHHHHHTTSSSTTSHHHHHHHHHHHHHHHHHHHHCGGGHHHHHHHHHHHHHHHHH--------- |
|  | T ss\_pred |  | -HHHHHHHHHHHHHHHHHHHHhhhcCCCCCHHHHHHHHHHHHHHHHHHHhHHHHHHHHHHHHHHHHHHHHH--------- |
|  |
|  |
|  | Q ss\_pred |  | cchhhcCCCCCChHHHHHHHHHHHHHHHHHHHHHHHHHHHccCCCccccCCCccc |
|  | Q Q6ZXV5 | 218 | AGQFLRGKGSIPFSMLQTLVKLIVLMFSTLLLVVIRVQVIQSQLPVFTRFDNPAA   272 (426) |
|  | Q Consensus | 218 | ~~~~~~~~~~~~~~~~~~~~~~~~~~~~~~~~~~~~~~~~~~~~~~~~~~~~~~~   272 (426) |
|  |  |  | ...+++...+...+......+..+++........+.............+...+ |
|  | T Consensus | 271 | --~~~~~~~~~~~~~~~~~~~~~~~~~~p~~i~~~~~~~~~~~~~~~g~~~~~~s   323 (759) |
|  | T 6P25\_B | 271 | --FLADKSMSWKTYINHWLARIFGLIIVPFCIFLLCFKIHFDLLSHSGTGDANMP   323 (759) |
|  | T ss\_dssp |  | --HTTCSSSCHHHHHHHHHHHHCCCCCHHHHHHHHHHHHHHHHCCBCCTTGGGSC |
|  | T ss\_pred |  | --HhcCCCCCHHHHHHHHHHHHHHHHHHHHHHHHHHHHHHHHhhcCCCCCcccCC |
|  |
| --- | | | |
|  | Template alignmentTemplate 3D StructurePDBe | | |
| 9. | 7BVF\_A Probable arabinosyltransferase B (E.C.2.4.2.-), Probable; Mycobacterium tuberculosis, cell wall synthesis; HET: 95E, DSL, CDL;{Mycolicibacterium smegmatis MC2 155} | | |
|  | Probability: 99.63%, E-value: 1.1e-12, Score: 126.48, Aligned cols: 357, Identities: 10%, Similarity: -0.013, | | |
|  |
|  | Q ss\_pred |  | HHHHHHHHHHhhCCCceeccHHHHhcCCCCCCCCcHHHHhccccCCCCCCcchhcCCCCcchHHHHHHHHHHhCCCchHH |
|  | Q Q6ZXV5 | 13 | VGVVTACYWNSLFCGFVFDDVSAILDNKDLHPSTPLKTLFQNDFWGTPMSEERSHKSYRPLTVLTFRLNYLLSELKPMSY   92 (426) |
|  | Q Consensus | 13 | ~~~~~~~~~~~~~~~~~~Dd~~~~~~~~~~~~~~~~~~~~~~~~~~~~~~~~~~~~~~~Pl~~~~~~~~~~lfg~~~~~~   92 (426) |
|  |  |  | .++...+-....-.+.+.||.++...+.+..+.+...+++.... ..+.++|+|++++..+..+ |.++... |
|  | T Consensus | 263 | ~~V~~~l~~w~~~gp~~~DDg~~~~~Ar~~~~~G~~~n~~~~~~---------~~e~p~~lyY~lL~~W~~v-G~s~~~L   332 (1102) |
|  | T 7BVF\_A | 263 | AAVIATLLLWHVIGATSSDDGYLLTVARVAPKAGYVANYYRYFG---------TTEAPFDWYTSVLAQLAAV-STAGVWM   332 (1102) |
|  | T ss\_dssp |  | HHHHHHHHHTTTSCCCCSTTHHHHHHHHHHHHHTSCBCSSSGGG---------CBCCTTCTTHHHHHHHHHH-CCCHHHH |
|  | T ss\_pred |  | HHHHHHHHHHHHhcccCCcchHHHHHHhhccccCcHHHHHHHHC---------CCCCCChHHHHHHHHHHhc-cCchHHh |
|  |
|  |
|  | Q ss\_pred |  | HHHHHHHHHHHHHHH-----HHHHHH---hcCCHHHHHHHHHHHHCcccHHHHHHhhccHHHHHHHHHHHHHHHHHHcCC |
|  | Q Q6ZXV5 | 93 | HLLNMIFHAVVSVIF-----LKVCKL---FLDNKSSVIASLLFAVHPIHTEAVTGVVGRAELLSSIFFLAAFLSYTRSKG   164 (426) |
|  | Q Consensus | 93 | rl~~~l~~~~~~~~~-----y~l~~~---~~~~~~a~~aall~~~~p~~~~~~~~~~~~~~~~~~~~~~l~~~~~~~~~~   164 (426) |
|  |  |  | |++|+++++++..++ +.+.++ +.+++.+.+++.+..+. .++.+.... ++|.+..++.+++++++.+..+ |
|  | T Consensus | 333 | RLpSvlagl~t~~ll~r~v~~~lgr~~~~l~~~~~a~~~aal~~la-~~l~y~~~~--Rpyal~al~~~la~~~~~ra~~   409 (1102) |
|  | T 7BVF\_A | 333 | RLPATLAGIACWLIVSRFVLRRLGPGPGGLASNRVAVFTAGAVFLS-AWLPFNNGL--RPEPLIALGVLVTWVLVERSIA   409 (1102) |
|  | T ss\_dssp |  | TSHHHHHHHHHHHHCCCCCHHHSCCSSSSSSSCSHHHHHHHHHHHH-HHTTTCCSS--SSHHHHHHHHHHHHHHHHHHHH |
|  | T ss\_pred |  | HHHHHHHHHHHHHHHHHHHHHHhCCCCCCccccHHHHHHHHHHHHH-HHHHHhcCC--ChHHHHHHHHHHHHHHHHHHHH |
|  |
|  |
|  | Q ss\_pred |  | CCCcccHHHHHHHHHHHHHHHHhHhHHHHHHHHHHHHHHHHhcCCCccchhcccchhhcCCCCCChHHHHHHHHHHHHHH |
|  | Q Q6ZXV5 | 165 | PDNSIIWTPIALTVFLVAVATLCKEQGITVVGICCVYEVFIAQGYTLPLLCTTAGQFLRGKGSIPFSMLQTLVKLIVLMF   244 (426) |
|  | Q Consensus | 165 | ~~~~~~~~~~~~~~~~~~la~~~k~~~~~~~~~~~~~~~~~~~~~~~~~~~~~~~~~~~~~~~~~~~~~~~~~~~~~~~~   244 (426) |
|  |  |  | +++ ..++.+..++.+++..+|+++++.+....+......+. .+.+............+ |
|  | T Consensus | 410 | ~~r---~~~~al~~~~a~lal~~hptgll~laall~~~~~l~r~-------------------lr~r~~~~~~~~~la~v   467 (1102) |
|  | T 7BVF\_A | 410 | LGR---LAPAAVAIIVATLTATLAPQGLIALAPLLTGARAIAQR-------------------IRRRRATDGLLAPLAVL   467 (1102) |
|  | T ss\_dssp |  | HTC---SHHHHHHHHHHHHHTTSCGGGGGGGHHHHHTTHHHHHH-------------------HHHSCSSSCSSHHHHHH |
|  | T ss\_pred |  | cCC---hHHHHHHHHHHHHHhccchHHHHHHHHHHHHHHHHHHH-------------------HHHhhhhcchHHHHHHH |
|  |
|  |
|  | Q ss\_pred |  | HHHHHHHHHHHHHccCCCccccCCCcccCCCchhhHHhHhhHHHHHHHHHhccccccccCccccCccccccccHHHHHHH |
|  | Q Q6ZXV5 | 245 | STLLLVVIRVQVIQSQLPVFTRFDNPAAVSPTPTRQLTFNYLLPVNAWLLLNPSELCCDWTMGTIPLIESLLDIRNLATF   324 (426) |
|  | Q Consensus | 245 | ~~~~~~~~~~~~~~~~~~~~~~~~~~~~~~~~~~~~~~~~~~~~~~~~~~~~~~~~~~~~~~~~~~~~~~~~~~~~~~~~   324 (426) |
|  |  |  | ...........+..+....................+.+....+...+ ......-......... |
|  | T Consensus | 468 | la~~~~~l~~~F~dq~l~~~~~a~~~~~~~g~~~~W~~e~~Ry~~L~-----------------~~~~~~Gs~arr~pvL   530 (1102) |
|  | T 7BVF\_A | 468 | AAALSLITVVVFRDQTLATVAESARIKYKVGPTIAWYQDFLRYYFLT-----------------VESNVEGSMSRRFAVL   530 (1102) |
|  | T ss\_dssp |  | HHTGGGTHHHHSSSSCHHHHHHHHHHHHHSSCCCCGGGTTTTSTTTS-----------------CSSCGGGCTTTHHHHH |
|  | T ss\_pred |  | HHHHHHHHHHHHccCcHHHHHHHHhHHHhhCCCChhHHHHHHHHHHH-----------------hccCCCCCchHHHHHH |
|  |
|  |
|  | Q ss\_pred |  | HHHHHHHHHHHHHHHccCCCc---hhHHHHHHHHHHHHHHHhccCCCcchhhchhhc-hHHHHHHHHHH--HHHHHHhcc |
|  | Q Q6ZXV5 | 325 | TFFCFLGMLGVFSIRYSGDSS---KTVLMALCLMALPFIPASNLFFPVGFVVAERVL-YVPSMGFCILV--AHGWQKIST   398 (426) |
|  | Q Consensus | 325 | ~~~~~~~~~~~~~~~~~~~~~---~~~~~~~~~~~~~~~~~~~~~~~~~~~~~~ry~-~~~~~~~~il~--~~~~~~~~~   398 (426) |
|  |  |  | +.++.+........|+++... ......+.+..+.++.+... ..+..||+ .+..+..++++ +..+.+... |
|  | T Consensus | 531 | l~l~~L~~~~~~l~Rrrr~~g~~~~~~~~ll~~~~l~lvll~~t-----PsKwt~hfg~~A~~~~aLlA~~~v~~~~~~~   605 (1102) |
|  | T 7BVF\_A | 531 | VLLFCLFGVLFVLLRRGRVAGLASGPAWRLIGTTAVGLLLLTFT-----PTKWAVQFGAFAGLAGVLGAVTAFTFARIGL   605 (1102) |
|  | T ss\_dssp |  | HHHHHHHHHHHHHHSSSCCSSSCHHHHHHHHHHHHHHHHHGGGC-----SCCCSGGGGGGHHHHHHHHHHHHHHHHHTTS |
|  | T ss\_pred |  | HHHHHHHHHHHHHHHcCCCCCcccCHHHHHHHHHHHHHHHHhcC-----ccHHHHHHHHHHHHHHHHHHHHHHHHhhhhh |
|  |
|  |
|  | Q ss\_pred |  | cchHHHHHHHHHHHHHHHHHHHhhhhcC |
|  | Q Q6ZXV5 | 399 | KSVFKKLSWICLSMVILTHSLKTFHRNW   426 (426) |
|  | Q Consensus | 399 | ~~~~~~~~~~~~~~~~~~~~~~~~~~~~   426 (426) |
|  |  |  | +..+.+......+++++.++....++-| |
|  | T Consensus | 606 | r~~r~~~~~~~~~~~~~als~~g~n~w~   633 (1102) |
|  | T 7BVF\_A | 606 | HSRRNLTLYVTALLFVLAWATSGINGWF   633 (1102) |
|  | T ss\_dssp |  | SCSHHHHHHHHHHHHHHHHHTTSCCCCS |
|  | T ss\_pred |  | cchHHHHHHHHHHHHHHHHHHhhhcccc |
|  |
| --- | | | |
|  | Template alignmentTemplate 3D StructurePDBe | | |
| 10. | 7BVF\_B Probable arabinosyltransferase B (E.C.2.4.2.-), Probable; Mycobacterium tuberculosis, cell wall synthesis; HET: 95E, DSL, CDL;{Mycolicibacterium smegmatis MC2 155} | | |
|  | Probability: 99.58%, E-value: 1.2e-11, Score: 119.96, Aligned cols: 355, Identities: 11%, Similarity: -0.073, | | |
|  |
|  | Q ss\_pred |  | HHHHHHHHHHhhCCCceeccHHHHhcCCCCCCCCcHHHHhccccCCCCCCcchhcCCCCcchHHHHHHHHHHhCCCchHH |
|  | Q Q6ZXV5 | 13 | VGVVTACYWNSLFCGFVFDDVSAILDNKDLHPSTPLKTLFQNDFWGTPMSEERSHKSYRPLTVLTFRLNYLLSELKPMSY   92 (426) |
|  | Q Consensus | 13 | ~~~~~~~~~~~~~~~~~~Dd~~~~~~~~~~~~~~~~~~~~~~~~~~~~~~~~~~~~~~~Pl~~~~~~~~~~lfg~~~~~~   92 (426) |
|  |  |  | .++...+.......+.+.||.++...+.+..+.+...+++.... ....+++++++++..+..+ |.++... |
|  | T Consensus | 281 | ~~V~~~L~~w~~~g~~~~DDg~~~~~ar~~~~~G~~~n~~~~~~---------~~e~p~~~yY~lL~~w~~l-G~s~~~l   350 (1116) |
|  | T 7BVF\_B | 281 | AVVIFGFLLWHVIGANSSDDGYILGMARVADHAGYMSNYFRWFG---------SPEDPFGWYYNLLALMTHV-SDASLWM   350 (1116) |
|  | T ss\_dssp |  | HHHHHHHHHHHHHCCCCSSHHHHHHHHHTTTTSSSCBCCSSSSS---------CBCCSSCSSHHHHHHHTTT-CCCHHHH |
|  | T ss\_pred |  | HHHHHHHHHHHHhCcCCCCchHHHHHhhccccccchHHHHHHhC---------CCCCccHHHHHHHHHHHHc-CCchHHh |
|  |
|  |
|  | Q ss\_pred |  | HHHHHHHHHHHHHHHHHHHHHhcCC------HHHHHHHHHHHHCcccHHHHHHhhccHHHHHHHHHHHHHHHHHHcCCCC |
|  | Q Q6ZXV5 | 93 | HLLNMIFHAVVSVIFLKVCKLFLDN------KSSVIASLLFAVHPIHTEAVTGVVGRAELLSSIFFLAAFLSYTRSKGPD   166 (426) |
|  | Q Consensus | 93 | rl~~~l~~~~~~~~~y~l~~~~~~~------~~a~~aall~~~~p~~~~~~~~~~~~~~~~~~~~~~l~~~~~~~~~~~~   166 (426) |
|  |  |  | |++|+++++++..++++...+..++ ..++.+++++.... ..+....++|.+..++.+++++++.+..+++ |
|  | T Consensus | 351 | RlpSllagl~t~~ll~r~vl~~lg~~~~~~~~a~~~aal~~l~~~----lpy~~~~Rpyal~al~~~lal~~~~ra~~~~   426 (1116) |
|  | T 7BVF\_B | 351 | RLPDLAAGLVCWLLLSREVLPRLGPAVEASKPAYWAAAMVLLTAW----MPFNNGLRPEGIIALGSLVTYVLIERSMRYS   426 (1116) |
|  | T ss\_dssp |  | TSHHHHHHHHHHHHCCCCCGGGSCTTTSSCHHHHHHHHHHHHHHH----SSSTTSSSTHHHHHHHHHHHHHHHHHHHHHT |
|  | T ss\_pred |  | hHHHHHHHHHHHHHHHHHHHHHhchhhcCCHHHHHHHHHHHHHHh----chhcCCCcHHHHHHHHHHHHHHHHHHHhcCC |
|  |
|  |
|  | Q ss\_pred |  | CcccHHHHHHHHHHHHHHHHhHhHHHHHHHHHHHHHHHHhcCCCccchhcccchhhcCCCCCChHHHHHHHHHHHHHHHH |
|  | Q Q6ZXV5 | 167 | NSIIWTPIALTVFLVAVATLCKEQGITVVGICCVYEVFIAQGYTLPLLCTTAGQFLRGKGSIPFSMLQTLVKLIVLMFST   246 (426) |
|  | Q Consensus | 167 | ~~~~~~~~~~~~~~~~la~~~k~~~~~~~~~~~~~~~~~~~~~~~~~~~~~~~~~~~~~~~~~~~~~~~~~~~~~~~~~~   246 (426) |
|  |  |  | + ..++.+.+++.++++.+|+++++.+....+......+. .+++............+.. |
|  | T Consensus | 427 | r---~~~~al~~~~a~lal~~hptgl~alaall~~l~~l~r~-------------------lr~r~~~~~~la~la~~la   484 (1116) |
|  | T 7BVF\_B | 427 | R---LTPAALAVVTAAFTLGVQPTGLIAVAALVAGGRPMLRI-------------------LVRRHRLVGTLPLVSPMLA   484 (1116) |
|  | T ss\_dssp |  | C---SSHHHHHHHHHHHHHTTCGGGCCSHHHHHHTTHHHHHH-------------------HHHHHTTSCSHHHHSHHHH |
|  | T ss\_pred |  | C---cHHHHHHHHHHHHHHhccHHHHHHHHHHHHhhHHHHHH-------------------HHHhhhhcchHHHHHHHHH |
|  |
|  |
|  | Q ss\_pred |  | HHHHHHHHHHHccCCCccccCCCcccCCCchhhHHhHhhHHHHHHHHHhccccccccCccccCccccccccHHHHHHHHH |
|  | Q Q6ZXV5 | 247 | LLLVVIRVQVIQSQLPVFTRFDNPAAVSPTPTRQLTFNYLLPVNAWLLLNPSELCCDWTMGTIPLIESLLDIRNLATFTF   326 (426) |
|  | Q Consensus | 247 | ~~~~~~~~~~~~~~~~~~~~~~~~~~~~~~~~~~~~~~~~~~~~~~~~~~~~~~~~~~~~~~~~~~~~~~~~~~~~~~~~   326 (426) |
|  |  |  | .........+..+....................+.+....+...+ .....-........... |
|  | T Consensus | 485 | ~~~~~l~~~F~dqsl~~~~~a~~~~~~~g~~~~W~~e~~Ry~~L~------------------~~~~~Gs~arr~~vLl~   546 (1116) |
|  | T 7BVF\_B | 485 | AGTVILTVVFADQTLSTVLEATRVRAKIGPSQAWYTENLRYYYLI------------------LPTVDGSLSRRFGFLIT   546 (1116) |
|  | T ss\_dssp |  | TTTCTHHHHTSSSCHHHHHHHHHHHHHTSCCCCGGGTHHHHHGGG------------------SSSSSSCHHHHHHHHHH |
|  | T ss\_pred |  | HHHHHHHHHHccchHHHHHHHHHHHHHhcCCchHHHHhHHHHHHh------------------ccCCCCChHHHHHHHHH |
|  |
|  |
|  | Q ss\_pred |  | HHHHHHHHHHHHHccCCCc---hhHHHHHHHHHHHHHHHhccCCCcchhhchhhchHHHHHHHHHHHHHHHHhccc--ch |
|  | Q Q6ZXV5 | 327 | FCFLGMLGVFSIRYSGDSS---KTVLMALCLMALPFIPASNLFFPVGFVVAERVLYVPSMGFCILVAHGWQKISTK--SV   401 (426) |
|  | Q Consensus | 327 | ~~~~~~~~~~~~~~~~~~~---~~~~~~~~~~~~~~~~~~~~~~~~~~~~~~ry~~~~~~~~~il~~~~~~~~~~~--~~   401 (426) |
|  |  |  | ++.+........|+++... ......+.+.++.++.+... ..+..||+....++.+.+++.......+. +. |
|  | T Consensus | 547 | ll~L~~~~~~l~R~rr~~g~~~~~~~~ll~~~~~~~~ll~~t-----PtKwthhfg~~a~~~~aLlA~~~~~~~~~~~r~   621 (1116) |
|  | T 7BVF\_B | 547 | ALCLFTAVFIMLRRKRIPSVARGPAWRLMGVIFGTMFFLMFT-----PTKWVHHFGLFAAVGAAMAALTTVLVSPSVLRW   621 (1116) |
|  | T ss\_dssp |  | HHHHHHHHHHHHHCSCCTTSCTTHHHHHHHHHHHHHHHTTSC-----SCCCGGGGGGGGTTHHHHHHHHHHHTSTTTSCC |
|  | T ss\_pred |  | HHHHHHHHHHHHhcCCCCcccCcHHHHHHHHHHHHHHHHhcC-----chhHHHHHHHHHHHHHHHHHHHHHHhChhhcch |
|  |
|  |
|  | Q ss\_pred |  | HH-HHHHHHHHHHHHHHHHHhhhhcC |
|  | Q Q6ZXV5 | 402 | FK-KLSWICLSMVILTHSLKTFHRNW   426 (426) |
|  | Q Consensus | 402 | ~~-~~~~~~~~~~~~~~~~~~~~~~~   426 (426) |
|  |  |  | .+ +......+++++.++....++-| |
|  | T Consensus | 622 | ~~~r~~~~~~~~~~~ala~~g~n~w~   647 (1116) |
|  | T 7BVF\_B | 622 | SRNRMAFLAALFFLLALCWATTNGWW   647 (1116) |
|  | T ss\_dssp |  | HHHHHHHHHHHHHHHHHHTSSCCCCC |
|  | T ss\_pred |  | HHHHHHHHHHHHHHHHHHHhhhcccc |
|  |
| --- | | | |
|  | Template alignmentTemplate 3D StructurePDBe | | |
| 11. | 7BWR\_A Integral membrane indolylacetylinositol arabinosyltransferase EmbB; Mycobacterium tuberculosis, EmbB, cryo-EM, ethambutol; HET: F8L;{Mycolicibacterium smegmatis MC2 155}; Related PDB entries: 7BVC\_B 7BVG\_B 7BWR\_B 7BX8\_B 7BX8\_A | | |
|  | Probability: 99.55%, E-value: 1.4e-11, Score: 118.62, Aligned cols: 356, Identities: 11%, Similarity: -0.039, | | |
|  |
|  | Q ss\_pred |  | HHHHHHHHHHhhCCCceeccHHHHhcCCCCCCCCcHHHHhccccCCCCCCcchhcCCCCcchHHHHHHHHHHhCCCchHH |
|  | Q Q6ZXV5 | 13 | VGVVTACYWNSLFCGFVFDDVSAILDNKDLHPSTPLKTLFQNDFWGTPMSEERSHKSYRPLTVLTFRLNYLLSELKPMSY   92 (426) |
|  | Q Consensus | 13 | ~~~~~~~~~~~~~~~~~~Dd~~~~~~~~~~~~~~~~~~~~~~~~~~~~~~~~~~~~~~~Pl~~~~~~~~~~lfg~~~~~~   92 (426) |
|  |  |  | .++...+-......+.+.||.++...+.+..+.+...+++.... ..+..+++++.++..+..+ |.++... |
|  | T Consensus | 267 | ~~V~a~L~~w~~ig~~~~DDg~~~~~ar~~~~~G~~~n~~r~~~---------~~e~p~~~yY~lL~~w~~l-G~s~~~l   336 (1082) |
|  | T 7BWR\_A | 267 | GVVVGGMAIWYVIGANSSDDGYILQMARTAEHAGYMANYFRWFG---------SPEDPFGWYYNVLALMTKV-SDASIWI   336 (1082) |
|  | T ss\_dssp |  | HHHTTTTTHHHHHSCCCTTHHHHHHHHHSHHHHSSCBCCSSSTT---------CBSCSSCCTHHHHHTTTTS-CCCTTTT |
|  | T ss\_pred |  | HHHHHHHHHHHHhccCCCchHHHHHHHHHHhHhcchHHHHHHhC---------CCCCccHHHHHHHHHHHHh-cCChHHH |
|  |
|  |
|  | Q ss\_pred |  | HHHHHHHHHHHHHHH-----HHHHHHhcCCHHHHHHHHHHHHCcccHHHHHHhhccHHHHHHHHHHHHHHHHHHcCCCCC |
|  | Q Q6ZXV5 | 93 | HLLNMIFHAVVSVIF-----LKVCKLFLDNKSSVIASLLFAVHPIHTEAVTGVVGRAELLSSIFFLAAFLSYTRSKGPDN   167 (426) |
|  | Q Consensus | 93 | rl~~~l~~~~~~~~~-----y~l~~~~~~~~~a~~aall~~~~p~~~~~~~~~~~~~~~~~~~~~~l~~~~~~~~~~~~~   167 (426) |
|  |  |  | |++|+++++++..++ +.++++..+++.+.+++.+.... ..+.+.... ++|.+..++.+++.+++.+..++++ |
|  | T Consensus | 337 | RLpS~laglat~~ll~r~v~~~lgr~~~~~~~a~~~Aal~~l~-~~l~y~~~~--Rpy~l~al~~~la~~~l~ra~~~~r   413 (1082) |
|  | T 7BWR\_A | 337 | RLPDLICALICWLLLSREVLPRLGPAVAGSRAAMWAAGLVLLG-AWMPFNNGL--RPEGQIATGALITYVLIERAVTSGR   413 (1082) |
|  | T ss\_dssp |  | TGGGTGGGTSSHHHHTTSSTGGGCHHHHHCSHHHHHHHHHHHH-HHTTTTTSS--STTHHHHHHHHHHHHHHHHHHHHCC |
|  | T ss\_pred |  | HHHHHHHHHHHHHHHHHhhHHHhchhhcccHHHHHHHHHHHHH-HHHHHhcCC--chHHHHHHHHHHHHHHHHHHHhcCC |
|  |
|  |
|  | Q ss\_pred |  | cccHHHHHHHHHHHHHHHHhHhHHHHHHHHHHHHHHHHhcCCCccchhcccchhhcCCCCCChHHHHHHHHHHHHHHHHH |
|  | Q Q6ZXV5 | 168 | SIIWTPIALTVFLVAVATLCKEQGITVVGICCVYEVFIAQGYTLPLLCTTAGQFLRGKGSIPFSMLQTLVKLIVLMFSTL   247 (426) |
|  | Q Consensus | 168 | ~~~~~~~~~~~~~~~la~~~k~~~~~~~~~~~~~~~~~~~~~~~~~~~~~~~~~~~~~~~~~~~~~~~~~~~~~~~~~~~   247 (426) |
|  |  |  | +.++.+.+++.++++.+|+++++.+....+......+. ..++.+..........+... |
|  | T Consensus | 414 | ---~~~~al~~~~a~lal~~hptgllalaallv~l~~l~r~-------------------~r~r~~~~~~la~la~~~aa   471 (1082) |
|  | T 7BWR\_A | 414 | ---LTPAALAITTAAFTLGIQPTGLIAVAALLAGGRPILRI-------------------VMRRRRLVGTWPLIAPLLAA   471 (1082) |
|  | T ss\_dssp |  | ---SHHHHHHHHHHHHTTSSCTTCHHHHHHHHHTHHHHHHH-------------------HHHTTTTSCSHHHHHHHHHT |
|  | T ss\_pred |  | ---CHHHHHHHHHHHHHHHhcHHHHHHHHHHHHccHHHHHH-------------------HhhhhccccHHHHHHHHHHH |
|  |
|  |
|  | Q ss\_pred |  | HHHHHHHHHHccCCCccccCCCcccCCCchhhHHhHhhHHHHHHHHHhccccccccCccccCccccccccHHHHHHHHHH |
|  | Q Q6ZXV5 | 248 | LLVVIRVQVIQSQLPVFTRFDNPAAVSPTPTRQLTFNYLLPVNAWLLLNPSELCCDWTMGTIPLIESLLDIRNLATFTFF   327 (426) |
|  | Q Consensus | 248 | ~~~~~~~~~~~~~~~~~~~~~~~~~~~~~~~~~~~~~~~~~~~~~~~~~~~~~~~~~~~~~~~~~~~~~~~~~~~~~~~~   327 (426) |
|  |  |  | ........+..+....................+.+....+...+ .....-........+..+ |
|  | T Consensus | 472 | ~~~~l~~~F~dqsl~~~~~a~~~~~~~g~~~~W~~e~~Ry~~L~------------------~~~~~Gs~arr~~vLl~l   533 (1082) |
|  | T 7BWR\_A | 472 | GTVILAVVFADQTIATVLEATRIRTAIGPSQEWWTENLRYYYLI------------------LPTTDGAISRRVAFVFTA   533 (1082) |
|  | T ss\_dssp |  | TSSTHHHHTSSCCHHHHHHHHHHHHHHSCCTTTSGGGSSSSCCC------------------CTTCCCHHHHHHTTTTTT |
|  | T ss\_pred |  | HHHHHHHHHhchHHHHHHHHHHHHhhcCCCccHHHhhHHHHHHH------------------cCCCCCcchHHHHHHHHH |
|  |
|  |
|  | Q ss\_pred |  | HHHHHHHHHHHHccCCCc---hhHHHHHHHHHHHHHHHhccCCCcchhhchhhchHHHHHHHHHHHHHHHHhcc---cch |
|  | Q Q6ZXV5 | 328 | CFLGMLGVFSIRYSGDSS---KTVLMALCLMALPFIPASNLFFPVGFVVAERVLYVPSMGFCILVAHGWQKIST---KSV   401 (426) |
|  | Q Consensus | 328 | ~~~~~~~~~~~~~~~~~~---~~~~~~~~~~~~~~~~~~~~~~~~~~~~~~ry~~~~~~~~~il~~~~~~~~~~---~~~   401 (426) |
|  |  |  | +.++.......|+++... ......+......++..... ..+..||+.........+++..+..+.. +.. |
|  | T Consensus | 534 | ~~L~~~~~~llR~~r~~g~~~~~~~~ll~~~~~~~~ll~~t-----PtKwthhfga~ag~g~~l~a~a~v~l~~~~~r~~   608 (1082) |
|  | T 7BWR\_A | 534 | MCLFPSLFMMLRRKHIAGVARGPAWRLMGIIFATMFFLMFT-----PTKWIHHFGLFAAVGGAMAALATVLVSPTVLRSA   608 (1082) |
|  | T ss\_dssp |  | TSSHHHHHHHHHSSSCSSSCTTHHHHHHHHHHHHHHHTBTT-----TBCCCCCTTGGGSHHHHHHHHHHHHSSTTTCCCH |
|  | T ss\_pred |  | HHHHHHHHHHHhhcccCCCcCCHHHHHHHHHHHHHHHHhhC-----cchHHHHHHHHHHHHHHHHHHHHHHHcHHhcccH |
|  |
|  |
|  | Q ss\_pred |  | HHHHHHHHHHHHHHHHHHHhhhhcC |
|  | Q Q6ZXV5 | 402 | FKKLSWICLSMVILTHSLKTFHRNW   426 (426) |
|  | Q Consensus | 402 | ~~~~~~~~~~~~~~~~~~~~~~~~~   426 (426) |
|  |  |  | +.+......++++..++....++-| |
|  | T Consensus | 609 | r~r~~~~~~~~~~~ala~~g~n~W~   633 (1082) |
|  | T 7BWR\_A | 609 | RNRMAFLSLVLFVLAFCFASTNGWW   633 (1082) |
|  | T ss\_dssp |  | HHHHHHHHHHHHHHHHHTSSCCCCS |
|  | T ss\_pred |  | HHHHHHHHHHHHHHHHHhccccccc |
|  |
| --- | | | |
|  | Template alignmentTemplate 3D StructurePDBe | | |
| 12. | 6SNI\_X Dolichyl pyrophosphate Man9GlcNAc2 alpha-1,3-glucosyltransferase (E.C.2.4.1.267); Glycosyltransferase, Glucosyltransferase, GT-C, N-Glycosylation, MEMBRANE; HET: PTY, Y01;{Saccharomyces cerevisiae}; Related PDB entries: 6SNH\_X | | |
|  | Probability: 99.55%, E-value: 1.8e-11, Score: 113.68, Aligned cols: 324, Identities: 11%, Similarity: -0.018, | | |
|  |
|  | Q ss\_pred |  | CcchhHHHHHHHHHHHHHHHHHhhCCCc-------eeccHHHHhcCCCCCCCCcHHHHhccccCCCCCCcchhcCCCCcc |
|  | Q Q6ZXV5 | 1 | MANINLKEITLIVGVVTACYWNSLFCGF-------VFDDVSAILDNKDLHPSTPLKTLFQNDFWGTPMSEERSHKSYRPL   73 (426) |
|  | Q Consensus | 1 | ~~~~~~~~~~~l~~~~~~~~~~~~~~~~-------~~Dd~~~~~~~~~~~~~~~~~~~~~~~~~~~~~~~~~~~~~~~Pl   73 (426) |
|  |  |  | .+......+.++++++++++......++ ..+|.+...+..+.....+..+++.++... +..+|||+ |
|  | T Consensus | 49 | ~~~~~~~~l~~i~~~~l~lR~~~~~~~~sg~~~pp~~~D~~~~~~w~~~~~~~~~~~wy~~~~~~-------~~~~YPPl   121 (562) |
|  | T 6SNI\_X | 49 | PVGNQWLPEYIIFVCAVILRCTIGLGPYSGKGSPPLYGDFEAQRHWMEITQHLPLSKWYWYDLQY-------WGLDYPPL   121 (562) |
|  | T ss\_dssp |  | -------CCSSHHHHHHHHHHHGGGSCCTTSSCSSSCCHHHHHHHHHHHHTTSCTTSTTTSCSTT-------TCCCSCHH |
|  | T ss\_pred |  | CCCCccHHHHHHHHHHHHHHHHHhcCCCCCCCCCCCCCCHHHHHHHHHHHHhCCHHHhcccCccc-------cCCCChHH |
|  |
|  |
|  | Q ss\_pred |  | hHHHHHHHHHHhC--------------------CCchHHHHHHHHHHHHHHHHH-HHHHHHhc-----CCHHHHHHHHHH |
|  | Q Q6ZXV5 | 74 | TVLTFRLNYLLSE--------------------LKPMSYHLLNMIFHAVVSVIF-LKVCKLFL-----DNKSSVIASLLF   127 (426) |
|  | Q Consensus | 74 | ~~~~~~~~~~lfg--------------------~~~~~~rl~~~l~~~~~~~~~-y~l~~~~~-----~~~~a~~aall~   127 (426) |
|  |  |  | ..+...+...+.+ .+....|+.++++.+++...+ |.+.|+.. +++.+..+++++ |
|  | T Consensus | 122 | ~~~~~~~~~~i~~~~~~~~~~l~~~~g~~~~~~~~~~~~rl~~i~~~ll~~~~~~~~~~~~~~~~~~~~~~~~~~~~~~~   201 (562) |
|  | T 6SNI\_X | 122 | TAFHSYLLGLIGSFFNPSWFALEKSRGFESPDNGLKTYMRSTVIISDILFYFPAVIYFTKWLGRYRNQSPIGQSIAASAI   201 (562) |
|  | T ss\_dssp |  | HHHHHHHHHHHHHHHCTTTTCSSSSTTCCCTTCCSSSHHHHHHHHHHHHHTHHHHHHHHHHHHHHHTCCHHHHHHHHHHH |
|  | T ss\_pred |  | HHHHHHHHHHHHHHhCHHHHHhcccCCCCCcchHHHHHHHHHHHHHHHHHHHHHHHHHHHHHhhcCCCChhHHHHHHHHH |
|  |
|  |
|  | Q ss\_pred |  | HHCcccHHHHHHhhccHHHHHHHHHHHHHHHHHHcCCCCCcccHHHHHHHHHHHHHHHHhHhHHHHHHHHHHHHHHHHhc |
|  | Q Q6ZXV5 | 128 | AVHPIHTEAVTGVVGRAELLSSIFFLAAFLSYTRSKGPDNSIIWTPIALTVFLVAVATLCKEQGITVVGICCVYEVFIAQ   207 (426) |
|  | Q Consensus | 128 | ~~~p~~~~~~~~~~~~~~~~~~~~~~l~~~~~~~~~~~~~~~~~~~~~~~~~~~~la~~~k~~~~~~~~~~~~~~~~~~~   207 (426) |
|  |  |  | +++|..+ ......++.|.....+.+++++++.+.+. ..+++++++|+.+|..++...++.+++.+.... |
|  | T Consensus | 202 | ~l~P~~i-~~~~~~~q~d~~~l~l~l~al~~~~~~~~----------~~agi~~~lal~~K~~~l~~~p~~~~~ll~~~~   270 (562) |
|  | T 6SNI\_X | 202 | LFQPSLM-LIDHGHFQYNSVMLGLTAYAINNLLDEYY----------AMAAVCFVLSICFKQMALYYAPIFFAYLLSRSL   270 (562) |
|  | T ss\_dssp |  | HCCHHHH-HHHTTTCCCHHHHHHHHHHHHHHHHHTCH----------HHHHHHHHHHHTTCGGGTTSHHHHHHHHHCCCC |
|  | T ss\_pred |  | HhCHHHH-HhhcccchhHHHHHHHHHHHHHHHHCCCh----------HHHHHHHHHHHHhhHHHHHHHHHHHHHHHHHHc |
|  |
|  |
|  | Q ss\_pred |  | CCCccchhcccchhhcCCCCCChHHHHHHHHHHHHHHHHHHHHHH-----HHHHHccCCCccccCCCcccCCCchhhHHh |
|  | Q Q6ZXV5 | 208 | GYTLPLLCTTAGQFLRGKGSIPFSMLQTLVKLIVLMFSTLLLVVI-----RVQVIQSQLPVFTRFDNPAAVSPTPTRQLT   282 (426) |
|  | Q Consensus | 208 | ~~~~~~~~~~~~~~~~~~~~~~~~~~~~~~~~~~~~~~~~~~~~~-----~~~~~~~~~~~~~~~~~~~~~~~~~~~~~~   282 (426) |
|  |  |  | + +++++.++..+.........++...++... ......+...............+....... |
|  | T Consensus | 271 | ~--------------~~~~~~~~~~~~~~~~~~~~~l~~~Pf~~~~~~~~~~~~~~~~~fp~~rgl~~~~~~n~w~~~~~   336 (562) |
|  | T 6SNI\_X | 271 | L--------------FPKFNIARLTVIAFATLATFAIIFAPLYFLGGGLKNIHQCIHRIFPFARGIFEDKVANFWCVTNV   336 (562) |
|  | T ss\_dssp |  | C--------------SSCCCHHHHHHHHHHHHHHHHHHHHHHHTTTCSHHHHHHHHHHHSCCCCSSSCSCCSSSHHHHTT |
|  | T ss\_pred |  | C--------------CCCCcHHHHHHHHHHHHHHHHHHHHHHHHhcCCHHHHHHHHHHHcCCCcccccchhhhHHHHHHH |
|  |
|  |
|  | Q ss\_pred |  | HhhHHHHHHHHHhccccccccCccccCccccccccHHHHHHHHHHHHHHHHHHHHHHccCCCchhHHHHHHHHHHHHHHH |
|  | Q Q6ZXV5 | 283 | FNYLLPVNAWLLLNPSELCCDWTMGTIPLIESLLDIRNLATFTFFCFLGMLGVFSIRYSGDSSKTVLMALCLMALPFIPA   362 (426) |
|  | Q Consensus | 283 | ~~~~~~~~~~~~~~~~~~~~~~~~~~~~~~~~~~~~~~~~~~~~~~~~~~~~~~~~~~~~~~~~~~~~~~~~~~~~~~~~   362 (426) |
|  |  |  | ... ...................+..+......++++++...............+.. |
|  | T Consensus | 337 | ~~~-----------------------~~~~~~~~~~~~~~~~~~~l~~l~~~~~~~~~~~~~~~~~~~~~~~l~~flfs-   392 (562) |
|  | T 6SNI\_X | 337 | FVK-----------------------YKERFTIQQLQLYSLIATVIGFLPAMIMTLLHPKKHLLPYVLIACSMSFFLFS-   392 (562) |
|  | T ss\_dssp |  | TSC-----------------------GGGTSCHHHHHHHHHHHHHHHHHHHHHHHHTSCCSSSHHHHHHHHHHHHHHHC- |
|  | T ss\_pred |  | HHH-----------------------HHhhCCHHHHHHHHHHHHHHHHHHHHHHHHhCCCcchHHHHHHHHHHHHHHhc- |
|  |
|  |
|  | Q ss\_pred |  | hccCCCcchhhchhh-chHHHHHHHHH |
|  | Q Q6ZXV5 | 363 | SNLFFPVGFVVAERV-LYVPSMGFCIL   388 (426) |
|  | Q Consensus | 363 | ~~~~~~~~~~~~~ry-~~~~~~~~~il   388 (426) |
|  |  |  | ...+++| +.+.+|...+. |
|  | T Consensus | 393 | --------~~vhekyill~llPl~ll~   411 (562) |
|  | T 6SNI\_X | 393 | --------FQVHEKTILIPLLPITLLY   411 (562) |
|  | T ss\_dssp |  | --------SSCCSSCCHHHHHHHHHGG |
|  | T ss\_pred |  | --------hhcCchhcHHHHHHHHHHh |
|  |
| --- | | | |
|  | Template alignmentTemplate 3D StructurePDBe | | |
| 13. | 6W98\_A F5/8 type C domain-containing protein; Glycosyltransferase, lipomannan, lipoarabinomannan, arabinofuranose, membrane; HET: PNS, 6OU; 2.9A {Escherichia coli (strain K12)}; Related PDB entries: 6WBX\_A 6WBY\_A | | |
|  | Probability: 99.49%, E-value: 8.1e-11, Score: 120.72, Aligned cols: 384, Identities: 12%, Similarity: 0.015, | | |
|  |
|  | Q ss\_pred |  | CcchhHHHHHHHHHHHHHHHHHhhCCCceeccHHHHhcCCCCCCCCcHHHHhccccCCCCCCcchhcCCCCcchHHHHHH |
|  | Q Q6ZXV5 | 1 | MANINLKEITLIVGVVTACYWNSLFCGFVFDDVSAILDNKDLHPSTPLKTLFQNDFWGTPMSEERSHKSYRPLTVLTFRL   80 (426) |
|  | Q Consensus | 1 | ~~~~~~~~~~~l~~~~~~~~~~~~~~~~~~Dd~~~~~~~~~~~~~~~~~~~~~~~~~~~~~~~~~~~~~~~Pl~~~~~~~   80 (426) |
|  |  |  | ........+++++++.+.............+|..++.+...+. ......+++........+..+...+| ...++.+ |
|  | T Consensus | 11 | ~~~~~~~ll~~~lll~~~~~~~~~g~~~~d~~~~~~~~~~~~l--~~~~~~W~~~~~~G~~~~~~~~y~~P--~~~~~~l   86 (1413) |
|  | T 6W98\_A | 11 | SALSRRWLAVAAAVSLLLTFSQSPGQISPDTKLDLAINPLRFA--ARALNLWSSDLPFGQAQNQAYGYLFP--HGAFFSL   86 (1413) |
|  | T ss\_dssp |  | CCCCTHHHHHHHHHHHHHHTTSSTTCBCCTTCSHHHHCHHHHH--HHTTSSEESSSTTSEECCSSGGGCCC--CCHHHHH |
|  | T ss\_pred |  | ccchHHHHHHHHHHHHHHHHhCCCCccCCCCCCccccCHHHHH--HHHHhccCCCCCCCCCchhhhhhhhh--HHHHHHH |
|  |
|  |
|  | Q ss\_pred |  | HHHHhCCCchHHHHHHHHHHHHHHHHHHHHHHHhc--CCHHHHHHHHHHHHCcccHHHHHHhhccHHHHHHHHHHHHHHH |
|  | Q Q6ZXV5 | 81 | NYLLSELKPMSYHLLNMIFHAVVSVIFLKVCKLFL--DNKSSVIASLLFAVHPIHTEAVTGVVGRAELLSSIFFLAAFLS   158 (426) |
|  | Q Consensus | 81 | ~~~lfg~~~~~~rl~~~l~~~~~~~~~y~l~~~~~--~~~~a~~aall~~~~p~~~~~~~~~~~~~~~~~~~~~~l~~~~   158 (426) |
|  |  |  | ...+........|+...++.+++...+|+++|++. ++..+++++++++++|.++...... ..+.+..++...+++. |
|  | T Consensus | 87 | ~~~lg~~~~~~~rl~~~l~~~la~~g~y~L~r~l~~~~~~~al~Aal~yalsP~~l~~~~~~--~~~~~~~~llp~~ll~   164 (1413) |
|  | T 6W98\_A | 87 | GHLLGVPAWVTQRLWWALLIVAGFWGLIRVAEALGIGTRGSRIIAAVAFALSPRVLTTLGAI--SSETLPMMLAPWVLLP   164 (1413) |
|  | T ss\_dssp |  | HHHHTCCHHHHHHHHHHHHHHHHHHHHHHHHHHHTCSCTTHHHHHHHHHHTCHHHHHHHTTC--GGGTHHHHHHHHHHHH |
|  | T ss\_pred |  | HHHcCCCHHHHHHHHHHHHHHHHHHHHHHHHHHhCCCChHHHHHHHHHHHHCHHHHHHhhcc--ChhhHHHHHHHHHHHH |
|  |
|  |
|  | Q ss\_pred |  | HHHcCCC--CCcccHHHHHHHHHHHHHHHHhHhHHHHHHHHHHHHHHHHhcCCCccchhcccchhhcCCCCCChHHHHHH |
|  | Q Q6ZXV5 | 159 | YTRSKGP--DNSIIWTPIALTVFLVAVATLCKEQGITVVGICCVYEVFIAQGYTLPLLCTTAGQFLRGKGSIPFSMLQTL   236 (426) |
|  | Q Consensus | 159 | ~~~~~~~--~~~~~~~~~~~~~~~~~la~~~k~~~~~~~~~~~~~~~~~~~~~~~~~~~~~~~~~~~~~~~~~~~~~~~~   236 (426) |
|  |  |  | +.+..++ ++ +++.+++++++++...++....++........++.+++ .++..+.. |
|  | T Consensus | 165 | l~~~~~~~~~~---~r~~~~~~l~~~l~~~~~~~~~~~~l~~~~l~~l~~~~--------------------~~~~~~~~   221 (1413) |
|  | T 6W98\_A | 165 | LILTFQGRMSP---RRAAALSAVAVALMGAVNAVATALACGVAVIWWLAHRP--------------------NRTWWRFT   221 (1413) |
|  | T ss\_dssp |  | HHHHHTTSSCH---HHHHHHHHHHHHHSCSSSHHHHHHHSHHHHHHHHSBCC--------------------CHHHHHHH |
|  | T ss\_pred |  | HHHHHcCCCCH---HHHHHHHHHHHHHhcchhHHHHHHHHHHHHHHHHHCCC--------------------CchHHHHH |
|  |
|  |
|  | Q ss\_pred |  | HHHHHHHHHHHHHHHHHHHHHccCCCccccCCCcccCCCchhhHHhHhhHHHHHHHHHhccccccccCccccCccccccc |
|  | Q Q6ZXV5 | 237 | VKLIVLMFSTLLLVVIRVQVIQSQLPVFTRFDNPAAVSPTPTRQLTFNYLLPVNAWLLLNPSELCCDWTMGTIPLIESLL   316 (426) |
|  | Q Consensus | 237 | ~~~~~~~~~~~~~~~~~~~~~~~~~~~~~~~~~~~~~~~~~~~~~~~~~~~~~~~~~~~~~~~~~~~~~~~~~~~~~~~~   316 (426) |
|  |  |  | .......++...+++......+.....+............... ....+........+............ |
|  | T Consensus | 222 | ~~~~~~~~l~~~~wl~Pll~~~~~~~~~~~~~e~~~~~~~~~s-----------~~~~l~~~~~w~~~~~~~~~~~~~~~   290 (1413) |
|  | T 6W98\_A | 222 | AWWIPCLALASTWWIVALLIFGKISPKFLDFIESSGVTTQWTS-----------LTEVLRGTDSWTPFVAPTATAGSSLV   290 (1413) |
|  | T ss\_dssp |  | HHHHHHHHHHHHHHHHHHHHHHHHSCCCTTSSCC-------CC-----------HHHHHHTC-----------------C |
|  | T ss\_pred |  | HHHHHHHHHHHHHHHHHHHHhcccCHhHHHHHhcccccccccc-----------HHHHHhCCCCCccccCCCCcccchHH |
|  |
|  |
|  | Q ss\_pred |  | cHHHHHHHHHHHHHHHHHHHHHHccCCCchhHHHHHHHHHHHHHHHh---------------ccCCCcchhhchhhchHH |
|  | Q Q6ZXV5 | 317 | DIRNLATFTFFCFLGMLGVFSIRYSGDSSKTVLMALCLMALPFIPAS---------------NLFFPVGFVVAERVLYVP   381 (426) |
|  | Q Consensus | 317 | ~~~~~~~~~~~~~~~~~~~~~~~~~~~~~~~~~~~~~~~~~~~~~~~---------------~~~~~~~~~~~~ry~~~~   381 (426) |
|  |  |  | ..........++.+++......++++.+.......++.+++...... .- .....+...||..+. |
|  | T Consensus | 291 | ~~~~~~~~~~~l~~lgl~~l~~r~~~~~~~l~~~~l~g~~l~~~~~~~~~~~p~~~~~~~~l~p-~~~~~R~~~Rf~~~~   369 (1413) |
|  | T 6W98\_A | 291 | TQSAMVIATTMLAAAGMAGLAMRGMPARGRLVAVLLIGLVLLTAGYTGALGSPIAQQIQFFLDD-GGTPLRNVHKLEPLI   369 (1413) |
|  | T ss\_dssp |  | CHHHHHHHHHHHHHHHHHHHSSTTCTTHHHHHHHHHHHHHTC----------------------------CCGGGSHHHH |
|  | T ss\_pred |  | HhHHHHHHHHHHHHHHHHHHHhCCCchHHHHHHHHHHHHHHHHhcccCCCCCchHHHHHHHhcc-CchhhcChhhhHHHH |
|  |
|  |
|  | Q ss\_pred |  | HHHHHHHHHHHHHHh---------------cccchHHHHHHHHHHHHHHHHHHHhhhhc |
|  | Q Q6ZXV5 | 382 | SMGFCILVAHGWQKI---------------STKSVFKKLSWICLSMVILTHSLKTFHRN   425 (426) |
|  | Q Consensus | 382 | ~~~~~il~~~~~~~~---------------~~~~~~~~~~~~~~~~~~~~~~~~~~~~~   425 (426) |
|  |  |  | .++++++++.++.++ .++.+.+.....+++++++.......... |
|  | T Consensus | 370 | ~l~lall~a~~l~~l~~~~~~~~~~~~~~~~~~~~~~~~~~~~~~~~~~~~~~~p~~~~   428 (1413) |
|  | T 6W98\_A | 370 | RLPLILGLAHALSRIPLPASVPVRQWLSALARPERNRAVAFAIVLLVALAASTSLAWTG   428 (1413) |
|  | T ss\_dssp |  | HHHHHHHHHHHTSSSCCTTTSCC------------CTTTHHHHHHHHHHHHHTHHHHTT |
|  | T ss\_pred |  | HHHHHHHHHHHHHhCCCCCCCCHHHHHHhhcChHHHHHHHHHHHHHHHHHHHHHHHHhC |
|  |
| --- | | | |
|  | Template alignmentTemplate 3D StructurePDBe | | |
| 14. | 7BVE\_B Integral membrane indolylacetylinositol arabinosyltransferase EmbC; Mycobacterium smegmatis, cell wall synthesis; HET: PO4, PN7, 95E; 2.81A {Mycolicibacterium smegmatis MC2 155}; Related PDB entries: 7BVH\_B 7BVH\_A 7BVE\_A | | |
|  | Probability: 99.49%, E-value: 4.2e-10, Score: 108.83, Aligned cols: 353, Identities: 11%, Similarity: -0.024, | | |
|  |
|  | Q ss\_pred |  | HHHHHHHHHHhhCCCceeccHHHHhcCCCCCCCCcHHHHhc-cccCCCCCCcchhcCCCCcchHHHHHHHHHHhCCCchH |
|  | Q Q6ZXV5 | 13 | VGVVTACYWNSLFCGFVFDDVSAILDNKDLHPSTPLKTLFQ-NDFWGTPMSEERSHKSYRPLTVLTFRLNYLLSELKPMS   91 (426) |
|  | Q Consensus | 13 | ~~~~~~~~~~~~~~~~~~Dd~~~~~~~~~~~~~~~~~~~~~-~~~~~~~~~~~~~~~~~~Pl~~~~~~~~~~lfg~~~~~   91 (426) |
|  |  |  | .++...+-...+..+.+.||.++...+.+....+...+++. .+ ..+....+++.++..+.. +|.++.. |
|  | T Consensus | 261 | ~~V~~~l~~w~~ig~~~~DEg~~l~~ar~~~~~Gy~~n~~~~~~----------~~dapfg~yY~lL~~W~~-vG~s~~~   329 (1084) |
|  | T 7BVE\_B | 261 | GLVSAMLVWWHFVGANTADDGYILTMARVSEHAGYMANYYRWFG----------TPESPFGWYYDLLALWAH-VSTASVW   329 (1084) |
|  | T ss\_dssp |  | HHHHHHHHHHHHSCCCCSSSHHHHHHHHHHHHHSSCBCSSSGGG----------CBCCSSCSSHHHHHHHTT-TCCCHHH |
|  | T ss\_pred |  | HHHHHHHHHHHHhcccccchhHHhhhhhchhhcCchHHHHHHHC----------CCCcccHHHHHHHHHHHH-ccccHHH |
|  |
|  |
|  | Q ss\_pred |  | HHHHHHHHHHHHHHHH-----HHHHHHhc-CCHHHHHHHHHHHHCcccHHHHHHhhccHHHHHHHHHHHHHHHHHHcCCC |
|  | Q Q6ZXV5 | 92 | YHLLNMIFHAVVSVIF-----LKVCKLFL-DNKSSVIASLLFAVHPIHTEAVTGVVGRAELLSSIFFLAAFLSYTRSKGP   165 (426) |
|  | Q Consensus | 92 | ~rl~~~l~~~~~~~~~-----y~l~~~~~-~~~~a~~aall~~~~p~~~~~~~~~~~~~~~~~~~~~~l~~~~~~~~~~~   165 (426) |
|  |  |  | .|++++++++++..++ ..+.++.. ++..++.+++++.. ....+....++|.+..++.+++.+++.|..++ |
|  | T Consensus | 330 | LRlpSll~glat~~ll~R~vl~~Lg~~~~~~~~a~~~aal~fl~----~wl~y~~~~Rpyalvalla~l~~~~~~ra~~~   405 (1084) |
|  | T 7BVE\_B | 330 | MRFPTLLMGLACWWVISREVIPRLGAAAKHSRAAAWTAAGLFLA----FWLPLNNGLRPEPIIALGILLTWCSVERGVAT   405 (1084) |
|  | T ss\_dssp |  | HHHHHHHHHHHHHHHCCCCCTTTSBSTTTSCSHHHHHHHHHHHH----HHHHHCSSSSSHHHHHHHHHHHHHHHHHHHHH |
|  | T ss\_pred |  | HHHHHHHHHHHHHHHHHHHhHHHHHHHhccCHHHHHHHHHHHHH----HHHHhcCCCChHHHHHHHHHHHHHHHHHHHcC |
|  |
|  |
|  | Q ss\_pred |  | CCcccHHHHHHHHHHHHHHHHhHhHHHHHHHHHHHHHHHHhcCCCccchhcccchhhcCCCCCChHHHHHHHHHHHHHHH |
|  | Q Q6ZXV5 | 166 | DNSIIWTPIALTVFLVAVATLCKEQGITVVGICCVYEVFIAQGYTLPLLCTTAGQFLRGKGSIPFSMLQTLVKLIVLMFS   245 (426) |
|  | Q Consensus | 166 | ~~~~~~~~~~~~~~~~~la~~~k~~~~~~~~~~~~~~~~~~~~~~~~~~~~~~~~~~~~~~~~~~~~~~~~~~~~~~~~~   245 (426) |
|  |  |  | ++ ++++.+++++.++++++|+++++......+......+. .+++............+. |
|  | T Consensus | 406 | ~r---~~~~ala~~~a~la~~~~Ptgl~ala~ll~~~~~l~r~-------------------lr~r~~~~~~la~la~~l   463 (1084) |
|  | T 7BVE\_B | 406 | SR---LLPVAVAIIIGALTLFSGPTGIAAVGALLVAIGPLKTI-------------------VAAHVSRFGYWALLAPIA   463 (1084) |
|  | T ss\_dssp |  | TC---SHHHHHHHHHHHHHHTSSGGGGGGHHHHHHTSHHHHHH-------------------HHHHTTTSCSHHHHHHHH |
|  | T ss\_pred |  | CC---cHHHHHHHHHHHHHHhhhHHHHHHHHHHHHHHHHHHHH-------------------HHhccchhHHHHHHHHHH |
|  |
|  |
|  | Q ss\_pred |  | HHHHHHHHHHHHccCCCccccCCCcccCCCchhhHHhHhhHHHHHHHHHhccccccccCccccCccccccccHHHHHHHH |
|  | Q Q6ZXV5 | 246 | TLLLVVIRVQVIQSQLPVFTRFDNPAAVSPTPTRQLTFNYLLPVNAWLLLNPSELCCDWTMGTIPLIESLLDIRNLATFT   325 (426) |
|  | Q Consensus | 246 | ~~~~~~~~~~~~~~~~~~~~~~~~~~~~~~~~~~~~~~~~~~~~~~~~~~~~~~~~~~~~~~~~~~~~~~~~~~~~~~~~   325 (426) |
|  |  |  | .+........+..+...........................+...+ .....-........++ |
|  | T Consensus | 464 | Aa~~~~l~~~Fadqsl~~~~~a~~v~~~~gp~l~w~~e~~Ry~~l~------------------g~~~~gs~arr~~vLl   525 (1084) |
|  | T 7BVE\_B | 464 | AAGTVTIFLIFRDQTLAAELQASSFKSAVGPSLAWFDEHIRYSRLF------------------TTSPDGSVARRFAVLT   525 (1084) |
|  | T ss\_dssp |  | HHHHTTHHHHTSSSCHHHHHHHHHHHHHHSCCCCGGGTHHHHHHHT------------------SSSSTTCHHHHHHHHH |
|  | T ss\_pred |  | HHHHHHHHHHHHhhhHHHHHHHHhhhhccCCCcHhHHHHHHHHHHh------------------cCCCCCCHHHHHHHHH |
|  |
|  |
|  | Q ss\_pred |  | HHHHHHHHHHHHHHccCCCc---hhHHHHHHHHHHHHHHHhccCCCcchhhchhhchHHHHHHHHHHHHHHHH---hccc |
|  | Q Q6ZXV5 | 326 | FFCFLGMLGVFSIRYSGDSS---KTVLMALCLMALPFIPASNLFFPVGFVVAERVLYVPSMGFCILVAHGWQK---ISTK   399 (426) |
|  | Q Consensus | 326 | ~~~~~~~~~~~~~~~~~~~~---~~~~~~~~~~~~~~~~~~~~~~~~~~~~~~ry~~~~~~~~~il~~~~~~~---~~~~   399 (426) |
|  |  |  | .++.++..+....|+++... ..........+..+...... ..+..+|+....+..+.+++..+.. ...+ |
|  | T Consensus | 526 | ~l~~l~~~~~ll~R~rr~~g~~~~~~~~l~~~~~~~l~ll~~t-----PtKwthhfg~lag~~~~lla~~~~~~~~~~~r   600 (1084) |
|  | T 7BVE\_B | 526 | LLLALAVSIAMTLRKGRIPGTALGPSRRIIGITIISFLAMMFT-----PTKWTHHFGVFAGLAGCLGALAAVAVTTTAMK   600 (1084) |
|  | T ss\_dssp |  | HHHHHHHHHHHHHHSSSCTTBCHHHHHHHHHHHHHHHHHGGGC-----SSCCSGGGGGGTTHHHHHHHHHHHTTSTTTCC |
|  | T ss\_pred |  | HHHHHHHHHHHHhhcCCCCCCCCCHHHHHHHHHHHHHHHHhcC-----CchHHHHHHhHHHHHHHHHHHHHHHHHHHhcc |
|  |
|  |
|  | Q ss\_pred |  | chHHHHHHHHHHHHHHHHHHHhhhhcC |
|  | Q Q6ZXV5 | 400 | SVFKKLSWICLSMVILTHSLKTFHRNW   426 (426) |
|  | Q Consensus | 400 | ~~~~~~~~~~~~~~~~~~~~~~~~~~~   426 (426) |
|  |  |  | ..+.+.. ....++++....+.-.+.| |
|  | T Consensus | 601 | ~~r~~~~-~~a~~~~~~ala~~G~N~W   626 (1084) |
|  | T 7BVE\_B | 601 | SRRNRTV-FGAAVLFVTALSFATVNGW   626 (1084) |
|  | T ss\_dssp |  | CHHHHHH-HHHHHHHHHHHHTSSCCCC |
|  | T ss\_pred |  | chHHHHH-HHHHHHHHHHHHhcccccc |
|  |
| --- | | | |
|  | Template alignmentTemplate 3D StructurePDBe | | |
| 15. | 7BVC\_A Integral membrane indolylacetylinositol arabinosyltransferase EmbA; Mycobacterium smegmatis, cell wall synthesis; HET: 95E, PNS, CDL, F8L;{Mycolicibacterium smegmatis MC2 155}; Related PDB entries: 7BVG\_A | | |
|  | Probability: 99.35%, E-value: 4.2e-9, Score: 101.76, Aligned cols: 353, Identities: 8%, Similarity: -0.065, | | |
|  |
|  | Q ss\_pred |  | HHHHHHHHHHhhCCCceeccHHHHhcCCCCCCCCcHHHHhccccCCCCCCcchhcCCCCcchHHHHHHHHHHhCCCchHH |
|  | Q Q6ZXV5 | 13 | VGVVTACYWNSLFCGFVFDDVSAILDNKDLHPSTPLKTLFQNDFWGTPMSEERSHKSYRPLTVLTFRLNYLLSELKPMSY   92 (426) |
|  | Q Consensus | 13 | ~~~~~~~~~~~~~~~~~~Dd~~~~~~~~~~~~~~~~~~~~~~~~~~~~~~~~~~~~~~~Pl~~~~~~~~~~lfg~~~~~~   92 (426) |
|  |  |  | .++...+-...+..+...||.++...+.+..+.....+++..-. ..+...-+++.++..+..+ |.++..+ |
|  | T Consensus | 251 | ~vV~~~L~~W~~igp~~~DDg~~~~~ar~~~~~G~~gny~r~~~---------~~eapf~~yY~ll~~w~~v-g~s~~~l   320 (1088) |
|  | T 7BVC\_A | 251 | TGVIGGLLIWHIVGAPTSDDGYNMTIARVASEAGYTTNYYRYFG---------ASEAPFDWYQSVLSHLASI-STAGVWM   320 (1088) |
|  | T ss\_dssp |  | HHHHHHHHSTTTSCCCCTTHHHHHHHHHHSSSSSSCBCSSSGGG---------CBCTTSCHHHHHHHHHTTT-CCCHHHH |
|  | T ss\_pred |  | HHHHHHHHHHHHhCCCCcchhHHHHHHHHHHHhcCHHHHHHHhc---------CCCcCCHHHHHHHHHHHhc-ccchHHh |
|  |
|  |
|  | Q ss\_pred |  | HHHHHHHHHHHHHHH-----HHHHHHhc-CCHHHHHHHHHHH--HCcccHHHHHHhhccHHHHHHHHHHHHHHHHHHcCC |
|  | Q Q6ZXV5 | 93 | HLLNMIFHAVVSVIF-----LKVCKLFL-DNKSSVIASLLFA--VHPIHTEAVTGVVGRAELLSSIFFLAAFLSYTRSKG   164 (426) |
|  | Q Consensus | 93 | rl~~~l~~~~~~~~~-----y~l~~~~~-~~~~a~~aall~~--~~p~~~~~~~~~~~~~~~~~~~~~~l~~~~~~~~~~   164 (426) |
|  |  |  | |++++++++++..++ ..+.++.. ++...+.+++.+. ..| +....++|.+..++.+++.+++.+..+ |
|  | T Consensus | 321 | RLPSllagl~tw~llsR~vl~~Lg~~~~~~~~a~~aaal~fla~wlP------y~~~~Rpe~~val~~~~a~~~~~ra~~   394 (1088) |
|  | T 7BVC\_A | 321 | RLPATAAAIATWLIISRCVLPRIGRRVAANRVAMLTAGATFLAAWLP------FNNGLRPEPLIAFAVITVWMLVENSIG   394 (1088) |
|  | T ss\_dssp |  | TGGGTHHHHHHHHHCCCCCHHHHCHHHHHCHHHHHHHHHHHHHHHTT------TCSSSSSHHHHHHHHHHHHHHHHHHHT |
|  | T ss\_pred |  | HHHHHHHHHHHHHHHHHHHHHHHhHhhcccHHHHHHHHHHHHHHHHH------hcCCCChHHHHHHHHHHHHHHHHHHhc |
|  |
|  |
|  | Q ss\_pred |  | CCCcccHHHHHHHHHHHHHHHHhHhHHHHHHHHHHHHHHHHhcCCCccchhcccchhhcCCCCCChHHHHHHHHHHHHHH |
|  | Q Q6ZXV5 | 165 | PDNSIIWTPIALTVFLVAVATLCKEQGITVVGICCVYEVFIAQGYTLPLLCTTAGQFLRGKGSIPFSMLQTLVKLIVLMF   244 (426) |
|  | Q Consensus | 165 | ~~~~~~~~~~~~~~~~~~la~~~k~~~~~~~~~~~~~~~~~~~~~~~~~~~~~~~~~~~~~~~~~~~~~~~~~~~~~~~~   244 (426) |
|  |  |  | +++ ..++..+.++.+++..+|+++++....+++......+. .+.+.+..........+ |
|  | T Consensus | 395 | ~~r---~~~~a~a~~~aala~~~hPtGl~a~a~ll~~~~~l~r~-------------------~r~r~~~~~~~a~~a~~   452 (1088) |
|  | T 7BVC\_A | 395 | TRR---LWPAAVAIVIAMFSVTLAPQGLIALAPLLVGARAIGRV-------------------VTARRAGTGILASLAPL   452 (1088) |
|  | T ss\_dssp |  | TTC---SHHHHHHHHHHHHHHTTCGGGGGGGHHHHHHHHHHHHH-------------------HGGGTTTTCSHHHHHHH |
|  | T ss\_pred |  | CCC---cHHHHHHHHHHHHHHhhcHHHHHHHHHHHHHHHHHHHH-------------------HHHhhccccHHHHHHHH |
|  |
|  |
|  | Q ss\_pred |  | HHHHHHHHHHHHHccCCCccccCCCcccCCCchhhHHhHhhHHHHHHHHHhccccccccCccccCccccccccHHHHHHH |
|  | Q Q6ZXV5 | 245 | STLLLVVIRVQVIQSQLPVFTRFDNPAAVSPTPTRQLTFNYLLPVNAWLLLNPSELCCDWTMGTIPLIESLLDIRNLATF   324 (426) |
|  | Q Consensus | 245 | ~~~~~~~~~~~~~~~~~~~~~~~~~~~~~~~~~~~~~~~~~~~~~~~~~~~~~~~~~~~~~~~~~~~~~~~~~~~~~~~~   324 (426) |
|  |  |  | ...........+..+....................+.+....+...+ ...........-..+ |
|  | T Consensus | 453 | laa~~~~l~~~F~dqtl~~~~~a~r~~~~~gp~~~w~~E~~RY~~L~------------------~~~~~~Gs~arR~~V   514 (1088) |
|  | T 7BVC\_A | 453 | AASVAVVFVIIFRDQTLATVAESVRIKYVVGPTIPWYQEFLRYYFLT------------------VEDSVDGSLTRRFAV   514 (1088) |
|  | T ss\_dssp |  | HHHHTTHHHHHTTSSCHHHHHHHHHHHHHHSCCCCTTCCTHHHHHHS------------------CSSCSSSCTTHHHHH |
|  | T ss\_pred |  | HHHHHHHHHHHhCCCcHHHHHHHHhHHhhhCCCChHHchhHHHHHHH------------------ccCCCCCCHHHHHHH |
|  |
|  |
|  | Q ss\_pred |  | HHHHHHHHHHHHHHHccCCCc----hhHHHHHHHHHHHHHHHhccCCCcchhhchhhchHHHHHHHHHHHHHHHHhcc-- |
|  | Q Q6ZXV5 | 325 | TFFCFLGMLGVFSIRYSGDSS----KTVLMALCLMALPFIPASNLFFPVGFVVAERVLYVPSMGFCILVAHGWQKIST--   398 (426) |
|  | Q Consensus | 325 | ~~~~~~~~~~~~~~~~~~~~~----~~~~~~~~~~~~~~~~~~~~~~~~~~~~~~ry~~~~~~~~~il~~~~~~~~~~--   398 (426) |
|  |  |  | ++.++.+........|+++.. ...........+.+....+. ..+..+|+-...+..+.+++.......+ |
|  | T Consensus | 515 | Ll~l~~l~~~~~~l~rrrr~~g~~~~~~~~l~~~~~~~~~lL~~t-----PtKWthHfGa~Ag~gaal~a~~~va~~~~~   589 (1088) |
|  | T 7BVC\_A | 515 | LVLLLCLFGLIMVLLRRGRVPGAVSGPLWRLCGSTAIGLLLLILT-----PTKWAIQFGAFAGLAGALGGVTAFAFARVG   589 (1088) |
|  | T ss\_dssp |  | HHHHHHHHHHHHHHHHSSCCTTSCHHHHHHHHHHHHHHHHHTTSC-----SCCCSGGGGGGHHHHHHHHHHHHHHHHHHT |
|  | T ss\_pred |  | HHHHHHHHHHHHHHHhcCCCCCCCccHHHHHHHHHHHHHHHHHHC-----cchHHHHHHHHHHHHHHHHHHHHHHHHHhc |
|  |
|  |
|  | Q ss\_pred |  | cchHHHHHHHHHHHHHHHHHHHhhhhcC |
|  | Q Q6ZXV5 | 399 | KSVFKKLSWICLSMVILTHSLKTFHRNW   426 (426) |
|  | Q Consensus | 399 | ~~~~~~~~~~~~~~~~~~~~~~~~~~~~   426 (426) |
|  |  |  | .+..+........++++....+.-.+.| |
|  | T Consensus | 590 | ~r~~r~~~~~~a~~~~~~alaf~G~N~W   617 (1088) |
|  | T 7BVC\_A | 590 | LHSRRNLALYVTALLFILAWATSGLNGW   617 (1088) |
|  | T ss\_dssp |  | TSCHHHHHHHHHHHHHHHHHHTTSCCCC |
|  | T ss\_pred |  | cChHHHHHHHHHHHHHHHHHHhhccccc |
|  |
| --- | | | |
|  | Template alignmentTemplate 3D StructurePDBe | | |
| 16. | 6P25\_B Dolichyl-diphosphooligosaccharide--protein glycosyltransferase subunits (E.C.2.4.99.18); complex, TRANSFERASE, glycosylation; HET: NAG, CPL, NNM; 3.2A {Saccharomyces cerevisiae W303}; Related PDB entries: 6P2R\_B ; Related PDB entries: 6P2R\_B ; Related PDB entries: 6P2R\_B | | |
|  | Probability: 65.1%, E-value: 83, Score: 31.58, Aligned cols: 102, Identities: 13%, Similarity: 0.116, | | |
|  |
|  | Q ss\_pred |  | HHHHHHHHHHHHHHHHHHHHHHccCCCchhH------------HHHHHHHHHHHHHHhccCCCcchhhchhhchHHHHHH |
|  | Q Q6ZXV5 | 318 | IRNLATFTFFCFLGMLGVFSIRYSGDSSKTV------------LMALCLMALPFIPASNLFFPVGFVVAERVLYVPSMGF   385 (426) |
|  | Q Consensus | 318 | ~~~~~~~~~~~~~~~~~~~~~~~~~~~~~~~------------~~~~~~~~~~~~~~~~~~~~~~~~~~~ry~~~~~~~~   385 (426) |
|  |  |  | ........++...+..+....+.++...... .+.++..++..+| ++...+....+|.+|++++. |
|  | T Consensus | 610 | iww~~~~~~~~~~~~~~~~~~~~~r~~~~~~~~~~~~~~~~~~~~~~~g~~~~ylP----~~~~~R~~fl~hYlpal~f~   685 (759) |
|  | T 6P25\_B | 610 | STWASSVAVLAFMATVVILLIRWQRQYVDLRNPSNWNVFLMGGFYPLLAWGLHYMP----FVIMSRVTYVHHYLPALYFA   685 (759) |
|  | T ss\_dssp |  | HHHHHHHHHHHHHHHHHHHHHHHHTTCCCCCSHHHHHCCCCCCCHHHHHHHHHHHH----HHHSCSCBCGGGHHHHHHHH |
|  | T ss\_pred |  | HHHHHHHHHHHHHHHHHHHHHHHhcCCCCCCCchhHHHHHHHhHHHHHHHHHhcch----HHcccCcccHHHHHHHHHHH |
|  |
|  |
|  | Q ss\_pred |  | HHHHHHHHHHhcccc------hHHHHHHHHHHHHHHHHHHHhhh |
|  | Q Q6ZXV5 | 386 | CILVAHGWQKISTKS------VFKKLSWICLSMVILTHSLKTFH   423 (426) |
|  | Q Consensus | 386 | ~il~~~~~~~~~~~~------~~~~~~~~~~~~~~~~~~~~~~~   423 (426) |
|  |  |  | +++++..++.+.++. +..+.....+.+++++.+...+. |
|  | T Consensus | 686 | il~~~~~~~~~~~~~~~~~~~~~~~~~~~~~~~~~~~~~f~~~~   729 (759) |
|  | T 6P25\_B | 686 | LIILAYCFDAGLQKWSRSKCGRIMRFVLYAGFMALVIGCFWYFS   729 (759) |
|  | T ss\_dssp |  | HHHHHHHHHTSSSGGGGSHHHHHHHHHHHHHHHHHHHHHHHHTT |
|  | T ss\_pred |  | HHHHHHHHHHHHHhcccccccHHHHHHHHHHHHHHHHHHHHHHH |
|  |
| --- | | | |
|  | Template alignmentTemplate 3D StructurePDBe | | |
| 17. | 6P25\_A Dolichyl-diphosphooligosaccharide--protein glycosyltransferase subunits (E.C.2.4.99.18); complex, TRANSFERASE, glycosylation; HET: NAG, CPL, NNM; 3.2A {Saccharomyces cerevisiae W303}; Related PDB entries: 6P2R\_A ; Related PDB entries: 6P2R\_A ; Related PDB entries: 6P2R\_A | | |
|  | Probability: 41.55%, E-value: 330, Score: 27.85, Aligned cols: 105, Identities: 10%, Similarity: -0.01, | | |
|  |
|  | Q ss\_pred |  | ccHHHHHHHHHHHHHHHHHHHHHHccCCCch------------hHHHHHHHHHHHHHHHhccCCCcchhhchhhchHHHH |
|  | Q Q6ZXV5 | 316 | LDIRNLATFTFFCFLGMLGVFSIRYSGDSSK------------TVLMALCLMALPFIPASNLFFPVGFVVAERVLYVPSM   383 (426) |
|  | Q Consensus | 316 | ~~~~~~~~~~~~~~~~~~~~~~~~~~~~~~~------------~~~~~~~~~~~~~~~~~~~~~~~~~~~~~ry~~~~~~   383 (426) |
|  |  |  | +...++.....+++..........+.++... .....++..++..+| ++...+....++.+|+++ |
|  | T Consensus | 586 | Np~~ww~~~~~~~~~~~~~~~~~~~~~~~~~~~~~~~~~~~~~~~~~~~~gw~~hy~P----f~~~~R~~fl~hYlpal~   661 (817) |
|  | T 6P25\_A | 586 | NAIVWWAVTAFIGIFGLIVITELFSWQLGKPILKDSKVVNFHVQVIHYLLGFAVHYAP----SFLMQRQMFLHHYLPAYY   661 (817) |
|  | T ss\_dssp |  | CHHHHHHHHHHHHHHHHHHHHHHHHHHHTCCCCCSHHHHHHHHHHHHHHHHHHHTTGG----GTSCCSCCCGGGSHHHHH |
|  | T ss\_pred |  | cHHHHHHHHHHHHHHHHHHHHHHHHHHcCCCCCCchhHHHHHHHHHHHHHHHHHHHHH----HHHccCccchhhHHHHHH |
|  |
|  |
|  | Q ss\_pred |  | HHHHHHHHHHHHhcccc---hHHHHHHHHHHHHHHHHHHHhhhh |
|  | Q Q6ZXV5 | 384 | GFCILVAHGWQKISTKS---VFKKLSWICLSMVILTHSLKTFHR   424 (426) |
|  | Q Consensus | 384 | ~~~il~~~~~~~~~~~~---~~~~~~~~~~~~~~~~~~~~~~~~   424 (426) |
|  |  |  | +.+++++..++.+.++. ++......+++.+++....+.... |
|  | T Consensus | 662 | F~il~~~~~~~~~~~~~~~~~~~~~~~~~~~~~~~~~~~f~~~~   705 (817) |
|  | T 6P25\_A | 662 | FGILALGHALDIIVSYVFRSKRQMGYAVVITFLAASVYFFKSFS   705 (817) |
|  | T ss\_dssp |  | HHHHHHHHHHHHHHHTTTSSCHHHHHHHHHHHHHHHHHHHHHSG |
|  | T ss\_pred |  | HHHHHHHHHHHHHHHHhccccchHHHHHHHHHHHHHHHHHHHhh |
|  |

---

If you use HHpred on our Toolkit for your research, please cite as appropriate:

A Completely Reimplemented MPI Bioinformatics Toolkit
with a New HHpred Server at its Core.  
Zimmermann L, Stephens A, Nam SZ, Rau D,
Kübler J, Lozajic M, Gabler F, Söding J, Lupas AN, Alva V.
J Mol Biol. 2018 Jul 20. S0022-2836(17)30587-9.

  

Protein homology detection by HMM-HMM comparison.  
Söding J. Bioinformatics. 2005 Apr 1;21(7):951-60.  
  
Fast and accurate automatic structure prediction with HHpred.  
Hildebrand A, Remmert M, Biegert A, Söding J. Proteins. 2009;77 Suppl 9:128-32.  
  
Automatic Prediction of Protein 3D Structures by Probabilistic Multi-template Homology Modeling.  
Meier A, Söding J. PLoS Comput Biol. 2015 Oct 23;11(10):e1004343.

Download

---

If you use HHpred on our Toolkit for your research, please cite as appropriate:

A Completely Reimplemented MPI Bioinformatics Toolkit
with a New HHpred Server at its Core.  
Zimmermann L, Stephens A, Nam SZ, Rau D,
Kübler J, Lozajic M, Gabler F, Söding J, Lupas AN, Alva V.
J Mol Biol. 2018 Jul 20. S0022-2836(17)30587-9.

  

Protein homology detection by HMM-HMM comparison.  
Söding J. Bioinformatics. 2005 Apr 1;21(7):951-60.  
  
Fast and accurate automatic structure prediction with HHpred.  
Hildebrand A, Remmert M, Biegert A, Söding J. Proteins. 2009;77 Suppl 9:128-32.  
  
Automatic Prediction of Protein 3D Structures by Probabilistic Multi-template Homology Modeling.  
Meier A, Söding J. PLoS Comput Biol. 2015 Oct 23;11(10):e1004343.

Loading...

---

If you use HHpred on our Toolkit for your research, please cite as appropriate:

A Completely Reimplemented MPI Bioinformatics Toolkit
with a New HHpred Server at its Core.  
Zimmermann L, Stephens A, Nam SZ, Rau D,
Kübler J, Lozajic M, Gabler F, Söding J, Lupas AN, Alva V.
J Mol Biol. 2018 Jul 20. S0022-2836(17)30587-9.

  

Protein homology detection by HMM-HMM comparison.  
Söding J. Bioinformatics. 2005 Apr 1;21(7):951-60.  
  
Fast and accurate automatic structure prediction with HHpred.  
Hildebrand A, Remmert M, Biegert A, Söding J. Proteins. 2009;77 Suppl 9:128-32.  
  
Automatic Prediction of Protein 3D Structures by Probabilistic Multi-template Homology Modeling.  
Meier A, Söding J. PLoS Comput Biol. 2015 Oct 23;11(10):e1004343.

Loading hits...

---

If you use HHpred on our Toolkit for your research, please cite as appropriate:

A Completely Reimplemented MPI Bioinformatics Toolkit
with a New HHpred Server at its Core.  
Zimmermann L, Stephens A, Nam SZ, Rau D,
Kübler J, Lozajic M, Gabler F, Söding J, Lupas AN, Alva V.
J Mol Biol. 2018 Jul 20. S0022-2836(17)30587-9.

  

Protein homology detection by HMM-HMM comparison.  
Söding J. Bioinformatics. 2005 Apr 1;21(7):951-60.  
  
Fast and accurate automatic structure prediction with HHpred.  
Hildebrand A, Remmert M, Biegert A, Söding J. Proteins. 2009;77 Suppl 9:128-32.  
  
Automatic Prediction of Protein 3D Structures by Probabilistic Multi-template Homology Modeling.  
Meier A, Söding J. PLoS Comput Biol. 2015 Oct 23;11(10):e1004343.

Loading hits...

---

If you use HHpred on our Toolkit for your research, please cite as appropriate:

A Completely Reimplemented MPI Bioinformatics Toolkit
with a New HHpred Server at its Core.  
Zimmermann L, Stephens A, Nam SZ, Rau D,
Kübler J, Lozajic M, Gabler F, Söding J, Lupas AN, Alva V.
J Mol Biol. 2018 Jul 20. S0022-2836(17)30587-9.

  

Protein homology detection by HMM-HMM comparison.  
Söding J. Bioinformatics. 2005 Apr 1;21(7):951-60.  
  
Fast and accurate automatic structure prediction with HHpred.  
Hildebrand A, Remmert M, Biegert A, Söding J. Proteins. 2009;77 Suppl 9:128-32.  
  
Automatic Prediction of Protein 3D Structures by Probabilistic Multi-template Homology Modeling.  
Meier A, Söding J. PLoS Comput Biol. 2015 Oct 23;11(10):e1004343.

- Help
- FAQ
- Privacy Policy
- Imprint
- Contact Us
- Cite Us
- Recent Updates

© 2008-2020, Dept. of Protein Evolution, Max Planck Institute for Developmental Biology, Tübingen

Template 3D Structure: 
×

Loading...
