# Supplementary material for: Conserved sequence motifs in human TMTC1, TMTC2, TMTC3, and TMTC4, new O-mannosyltransferases from the GT-C/PMT clan, are rationalized as ligand binding sites
Source: Biol Direct. 2021 Jan 12;16:4. doi: 10.1186/s13062-021-00291-w (PMC7801869; doi:10.1186/s13062-021-00291-w)
Supplement: Supplementary file 3 — Additional file 3. HHPred outputs when searching TMTCs against Pfam or PDB structures. The compressed library file AF3-2020-06-HHPred-TMTCs.zip contains the outputs when running the four human TMTC sequences as input of HHPred against PDB sequences and against Pfam domains (as of 23rd of June 2020). [file 13062_2021_291_MOESM3_ESM.zip › AF3-2020-06-HHPred-TMTCs/HHPred_TMTC3_PFam.html]

(\*) HHpred | Bioinformatics Toolkit          **We're sorry but the Toolkit doesn't work properly without JavaScript enabled. Please enable it to continue.**

Sign In

- Search
- Alignment
- Sequence Analysis
- 2ary Structure
- 3ary Structure
- Classification
- Utils

- HHblits
- HHpred
- HMMER
- PatternSearch
- ProtBLAST/PSI-BLAST

Nothing found.

###### Tools

###### Jobs

ID

Date

Tool

5407837HHPR8665047HHPR2161064HHPR

# HHpred

Job ID: 5407837,Created: an hour ago

- Input
- Parameters
- Results
- Raw Output
- Probability Plot
- Query Template MSA
- Query MSA

>sp|Q6ZXV5|TMTC3\_HUMAN 1..426
MANINLKEITLIVGVVTACYWNSLFCGFVFDDVSAILDNKDLHPSTPLKTLFQNDFWGTP
MSEERSHKSYRPLTVLTFRLNYLLSELKPMSYHLLNMIFHAVVSVIFLKVCKLFLDNKSS
VIASLLFAVHPIHTEAVTGVVGRAELLSSIFFLAAFLSYTRSKGPDNSIIWTPIALTVFL
VAVATLCKEQGITVVGICCVYEVFIAQGYTLPLLCTTAGQFLRGKGSIPFSMLQTLVKLI
VLMFSTLLLVVIRVQVIQSQLPVFTRFDNPAAVSPTPTRQLTFNYLLPVNAWLLLNPSEL
CCDWTMGTIPLIESLLDIRNLATFTFFCFLGMLGVFSIRYSGDSSKTVLMALCLMALPFI
PASNLFFPVGFVVAERVLYVPSMGFCILVAHGWQKISTKSVFKKLSWICLSMVILTHSLK
TFHRNW

Paste ExampleUpload File

Protein FASTA

Align two sequences/MSAs

Select structural/domain databases

Pfam-A\_v33.1

- PDB\_mmCIF70\_29\_May (default)
- PDB\_mmCIF30\_29\_May
- SCOPe70\_2.07
- ECOD\_ECOD\_F70\_20200207
- COG\_KOG\_v1.0
- Pfam-A\_v33.1
- NCBI\_Conserved\_Domains(CD)\_v3.18
- SMART\_v6.0
- TIGRFAMs\_v15.0
- PRK\_v6.9
- No elements found. Consider changing the search query.
- List is empty.

Select proteomes

Select options

- Euk\_Arabidopsis\_thaliana\_TAIR10\_20\_Jun\_2017
- Euk\_Bombyx\_mori\_p50T\_Dazao\_06\_May\_2019
- Euk\_Brachypodium\_distachyon\_23\_Aug\_2017
- Euk\_Caenorhabditis\_elegans\_18\_Jul\_2017
- Euk\_Capsaspora\_owczarzaki\_ATCC\_30864\_23\_Mar\_2020
- Euk\_Chaetomium\_thermophilum\_29\_Jun\_2017
- Euk\_Chlamydomonas\_reinhardtii\_27\_Jul\_2017
- Euk\_Entamoeba\_histolytica\_HM1\_IMSS\_22\_Mar\_2017
- Euk\_Dictyostelium\_discoideum\_AX4\_19\_Sep\_2017
- Euk\_Drosophila\_melanogaster\_19\_Jul\_2017
- Euk\_Giardia\_lamblia\_ATCC\_50803\_31\_Aug\_2017
- Euk\_Homo\_sapiens\_04\_Jul\_2017
- Euk\_Physcomitrella\_patens\_28\_Aug\_2017
- Euk\_Plasmodium\_falciparum\_3D7\_7\_Jun\_2017
- Euk\_Saccharomyces\_cerevisiae\_S288c\_11\_Mar\_2017
- Euk\_Schizosaccharomyces\_pombe\_19\_Sep\_2017
- Euk\_Solanum\_lycopersicum\_28\_Jul\_2019
- Euk\_Tetrahymena\_thermophila\_SB210\_22\_Aug\_2017
- Euk\_Toxoplasma\_gondii\_ME49\_10\_May\_2018
- Euk\_Trichomonas\_vaginalis\_G3\_21\_Nov\_2018
- Euk\_Trypanosoma\_brucei\_gambiense\_DAL972\_28\_Mar\_2017
- Euk\_Ustilago\_maydis\_521\_29\_May\_2017
- Euk\_Paramecium\_tetraurelia\_9\_Dec\_2018
- Arc\_Archaeoglobus\_fulgidus\_DSM\_4304\_5\_Dec\_2017
- Arc\_Halobacterium\_jilantaiense\_5\_Dec\_2017
- Arc\_Lokiarchaeum\_sp\_GC14\_75\_31\_Oct\_2018
- Arc\_Methanocaldococcus\_jannaschii\_DSM\_2661\_5\_Dec\_2017
- Arc\_Methanosarcina\_mazei\_S\_6\_17\_Mar\_2017
- Arc\_Methanothermus\_fervidus\_DSM\_2088\_5\_Dec\_2017
- Arc\_Pyrococcus\_horikoshii\_OT3\_5\_Dec\_2017
- Arc\_Sulfolobus\_solfataricus\_5\_Dec\_2017
- Arc\_Thermoplasma\_acidophilum\_DSM\_1728\_7\_Dec\_2017
- Bac\_Acinetobacter\_baumannii\_29\_Mar\_2018
- Bac\_Aquifex\_aeolicus\_VF5\_19\_Sep\_2017
- Bac\_Bacillus\_subtilis\_subsp\_subtilis\_str168\_19\_Mar\_2017
- Bac\_Bacteriovorax\_sp\_DB6\_IX\_1\_Jun\_2018
- Bac\_Bdellovibrio\_bacteriovorus\_HD100\_1\_Jun\_2018
- Bac\_Christensenella\_minuta\_2\_Apr\_2019
- Bac\_Deinococcus\_radiodurans\_R1\_19\_Sep\_2017
- Bac\_Enterococcus\_faecalis\_13\_SD\_W\_01\_1\_Jun\_2018
- Bac\_Escherichia\_coli\_K12\_07\_Mar\_2017
- Bac\_Fischerella\_muscicola\_PCC\_7414\_24\_Sep\_2017
- Bac\_Frankia\_alni\_ACN14a\_24\_Sep\_2017
- Bac\_Helicobacter\_pylori\_26695\_1\_Jun\_2018
- Bac\_Leptospira\_interrogans\_serovar\_Lai\_str56601\_1\_Jun\_2018
- Bac\_Mycobacterium\_tuberculosis\_H37Rv\_27\_May\_2017
- Bac\_Neisseria\_gonorrhoeae\_FA\_1090\_1\_Jun\_2018
- Bac\_Neisseria\_meningitidis\_MC58\_9\_Jun\_2017
- Bac\_Nostoc\_punctiforme\_PCC\_73102\_18\_Mar\_2017
- Bac\_Phycisphaerae\_bacterium\_L21\_RPulD3\_1\_Jun\_2018
- Bac\_Plesiocystis\_pacifica\_SIR1\_1\_Jun\_2018
- Bac\_Pseudomonas\_aeruginosa\_PAO1\_5\_Jun\_2017
- Bac\_Salmonella\_ent\_ser\_Typhi\_CT18\_22\_Nov\_2018
- Bac\_Staphylococcus\_aureus\_subsp\_aureus\_NCTC\_8325\_13\_Jun\_2017
- Bac\_Streptomyces\_scabiei\_87.22\_24\_Sep\_2017
- Bac\_Synechocystis\_sp\_PCC\_6803\_6\_Jun\_2017
- Bac\_Tenacibaculum\_dicentrarchi\_27\_Nov\_2017
- Bac\_Tenacibaculum\_maritimum\_NBRC\_15946\_27\_Nov\_2017
- Bac\_Thermus\_aquaticus\_Y51MC23\_24\_Sep\_2017
- Bac\_Thermus\_thermophilus\_HB8\_19\_Sep\_2017
- Bac\_Waddlia\_chondrophila\_WSU\_86\_1044\_1\_Jun\_2018
- Bac\_Yersinia\_pestis\_CO92\_10\_Apr\_2017
- Vir\_SARS-CoV-2\_31\_Mar\_2020
- No elements found. Consider changing the search query.
- List is empty.

ResubmitReset

MSA generation method

HHblits=>UniRef30

- HHblits=>UniRef30 (default)
- PSI-BLAST=>nr70
- No elements found. Consider changing the search query.
- List is empty.

Maximal no. of MSA generation steps

3

- 0
- 1
- 2
- 3 (default)
- 4
- 5
- 8
- No elements found. Consider changing the search query.
- List is empty.

E-value incl. threshold for MSA generation

1e-3

- 0.1
- 0.05
- 0.02
- 0.01
- 1e-3 (default)
- 1e-6
- 1e-8
- 1e-10
- 1e-15
- 1e-20
- 1e-30
- 1e-40
- 1e-50
- No elements found. Consider changing the search query.
- List is empty.

Min. seq. identity of MSA hits with query (%)

0

- 0 (default)
- 10
- 20
- 30
- 40
- 50
- 60
- 70
- 75
- 80
- 85
- 90
- 95
- 100
- No elements found. Consider changing the search query.
- List is empty.

Min. coverage of MSA hits (%)

20

- 10
- 20 (default)
- 30
- 40
- 50
- 60
- 70
- 80
- 90
- 100
- No elements found. Consider changing the search query.
- List is empty.

Secondary structure scoring

during\_alignment

- none
- after\_alignment
- during\_alignment (default)
- after\_alignment\_pred\_vs\_pred
- during\_alignment\_pred\_vs\_pred
- No elements found. Consider changing the search query.
- List is empty.

Alignment Mode:Realign with MAC

local:norealign

- local:norealign (default)
- local:realign
- global:realign
- No elements found. Consider changing the search query.
- List is empty.

MAC realignment threshold

0.3

- 0.0
- 0.01
- 0.1
- 0.2
- 0.3 (default)
- 0.4
- 0.5
- 0.6
- 0.7
- 0.8
- 0.9
- 0.95
- No elements found. Consider changing the search query.
- List is empty.

No. of target sequences (up to 10000)

250

- 250 (default)
- 500
- 1000
- 2000
- 3000
- 4000
- 5000
- 6000
- 7000
- 8000
- 9000
- 10000
- No elements found. Consider changing the search query.
- List is empty.

Min. probability in hit list (> 10%)

20

- 10
- 20 (default)
- 30
- 40
- 50
- 60
- 70
- 75
- 80
- 85
- 90
- 95
- 100
- No elements found. Consider changing the search query.
- List is empty.

ResubmitReset

VisHitsAln
Select AllForwardForward Query A3MDownload HHRColor SeqsWrap Seqs

Number of Hits: **31**

Detected sequence features:
**◾Transmembrane segment(s)**

#### Visualization

Resubmit Section

9

426

Prob=99.4% E=2.1E-10 PF06728.14 ; PIG-U ; GPI transamidase subunit PIG-U

#### Hitlist

Show102550100AllEntries

Search:

| Nr (Click to sort Ascending) | Hit (Click to sort Ascending) | Name (Click to sort Ascending) | Probability (Click to sort Ascending) | E-value (Click to sort Ascending) | SS (Click to sort Ascending) | Cols (Click to sort Ascending) | Target Length (Click to sort Ascending) |
| --- | --- | --- | --- | --- | --- | --- | --- |
| 1 | PF03901.18 | ; Glyco\_transf\_22 ; Alg9-like mannosyltransferase family | 99.89 | 1.5e-20 | 31 | 344 | 388 |
| 2 | PF02516.15 | ; STT3 ; Oligosaccharyl transferase STT3 subunit | 99.86 | 1.8e-19 | 27.2 | 369 | 458 |
| 3 | PF09852.10 | ; DUF2079 ; Predicted membrane protein (DUF2079) | 99.82 | 8e-17 | 35.6 | 346 | 519 |
| 4 | PF10131.10 | ; PTPS\_related ; 6-pyruvoyl-tetrahydropterin synthase related domain; membrane protein | 99.77 | 2.5e-16 | 28 | 306 | 616 |
| 5 | PF07220.12 | ; DUF1420 ; Protein of unknown function (DUF1420) | 99.76 | 5e-15 | 35.5 | 358 | 670 |
| 6 | PF04188.14 | ; Mannosyl\_trans2 ; Mannosyltransferase (PIG-V) | 99.71 | 3.8e-14 | 31.9 | 336 | 432 |
| 7 | PF02366.19 | ; PMT ; Dolichyl-phosphate-mannose-protein mannosyltransferase | 99.66 | 1.5e-14 | 21.8 | 215 | 247 |
| 8 | PF12250.9 | ; AftA\_N ; Arabinofuranosyltransferase N terminal | 99.63 | 3.1e-13 | 28 | 322 | 432 |
| 9 | PF10034.10 | ; Dpy19 ; Q-cell neuroblast polarisation | 99.62 | 4.4e-13 | 29.2 | 344 | 651 |
| 10 | PF09913.10 | ; DUF2142 ; Predicted membrane protein (DUF2142) | 99.54 | 5.4e-13 | 21.1 | 328 | 405 |
| 11 | PF13231.7 | ; PMT\_2 ; Dolichyl-phosphate-mannose-protein mannosyltransferase | 99.54 | 6.2e-13 | 18.4 | 156 | 159 |
| 12 | PF04602.13 | ; Arabinose\_trans ; Mycobacterial cell wall arabinan synthesis protein | 99.48 | 1.1e-10 | 29.6 | 350 | 471 |
| 13 | PF11028.9 | ; DUF2723 ; Protein of unknown function (DUF2723) | 99.47 | 7.1e-13 | 13.9 | 152 | 188 |
| 14 | PF06728.14 | ; PIG-U ; GPI transamidase subunit PIG-U | 99.42 | 2.1e-10 | 27.6 | 333 | 363 |
| 15 | PF15971.6 | ; Mannosyl\_trans4 ; DolP-mannose mannosyltransferase | 99.35 | 6.2e-11 | 17.5 | 156 | 163 |
| 16 | PF03155.16 | ; Alg6\_Alg8 ; ALG6, ALG8 glycosyltransferase family | 99.25 | 3e-9 | 25.7 | 325 | 470 |
| 17 | PF09586.11 | ; YfhO ; Bacterial membrane protein YfhO | 99.23 | 5.3e-9 | 28.1 | 367 | 832 |
| 18 | PF14264.7 | ; Glucos\_trans\_II ; Glucosyl transferase GtrII | 99.14 | 7.6e-8 | 27.3 | 303 | 312 |
| 19 | PF04922.13 | ; DIE2\_ALG10 ; DIE2/ALG10 family | 99.06 | 1.2e-8 | 19.4 | 207 | 434 |
| 20 | PF05208.14 | ; ALG3 ; ALG3 protein | 99.03 | 2.1e-8 | 19.4 | 198 | 356 |
| 21 | PF09594.11 | ; GT87 ; Glycosyltransferase family 87 | 98.86 | 0.0000011 | 22.9 | 242 | 251 |
| 22 | PF14897.7 | ; EpsG ; EpsG family | 96.65 | 0.15 | 32.5 | 299 | 319 |
| 23 | PF05007.14 | ; Mannosyl\_trans ; Mannosyltransferase (PIG-M) | 96.39 | 0.13 | 15.5 | 191 | 269 |
| 24 | PF10060.10 | ; DUF2298 ; Uncharacterized membrane protein (DUF2298) | 91.75 | 4.9 | 33.4 | 344 | 597 |
| 25 | PF16192.6 | ; PMT\_4TMC ; C-terminal four TMM region of protein-O-mannosyltransferase | 86.81 | 5.3 | 11.4 | 103 | 198 |

Displaying 1 to 25 of 31 hits

- «
- ‹
- 1
- 2
- ›
- »

#### Alignments

|  |  |  |  |
| --- | --- | --- | --- |
|  | | | |
|  | Template alignmentCDD | | |
| 1. | PF03901.18 ; Glyco\_transf\_22 ; Alg9-like mannosyltransferase family | | |
|  | Probability: 99.89%, E-value: 1.5e-20, Score: 164.14, Aligned cols: 344, Identities: 11%, Similarity: -0.052, | | |
|  |
|  | Q ss\_pred |  | HHHHHHHHHHHHHHhhCCCceeccH-HHHhcCCCCCCCCcHHHHhccccCCCCCCcchhcCCCCcchHHHHHHHHHHhCC |
|  | Q Q6ZXV5 | 9 | ITLIVGVVTACYWNSLFCGFVFDDV-SAILDNKDLHPSTPLKTLFQNDFWGTPMSEERSHKSYRPLTVLTFRLNYLLSEL   87 (426) |
|  | Q Consensus | 9 | ~~~l~~~~~~~~~~~~~~~~~~Dd~-~~~~~~~~~~~~~~~~~~~~~~~~~~~~~~~~~~~~~~Pl~~~~~~~~~~lfg~   87 (426) |
|  |  |  | ++++.++.-..........+..||. .+...+++..++........ ..+.....+||+..........++|+ |
|  | T Consensus | 2 | il~~~~~l~l~~~~~~~~~~~~De~~~~~~~a~~~~~~~~~~~~~~--------~~~~~~~~~p~~~~~~~~~~~~~~~~   73 (388) |
|  | T PF03901.18 | 2 | LLLFTIALRILNCFLVQTSFVPDEYWQSLEVSHHMVFNYGYLTWEW--------TERLRSYTYPLIFASIYKILHLLGKD   73 (388) |
|  | T ss\_pred |  | HHHHHHHHHHHHHHhhhcCCCchHHHHHHHHHhhhccccCcCCCcc--------cccccCCHHHHHHHHHHHHHHHcCCC |
|  |
|  |
|  | Q ss\_pred |  | Cc----hHHHHHHHHHHHHHHHHHHHHHHHhcCCHHHHHHHHHHHHCcccHHHHHHhhccHHHHHHHHHHHHHHHHHHcC |
|  | Q Q6ZXV5 | 88 | KP----MSYHLLNMIFHAVVSVIFLKVCKLFLDNKSSVIASLLFAVHPIHTEAVTGVVGRAELLSSIFFLAAFLSYTRSK   163 (426) |
|  | Q Consensus | 88 | ~~----~~~rl~~~l~~~~~~~~~y~l~~~~~~~~~a~~aall~~~~p~~~~~~~~~~~~~~~~~~~~~~l~~~~~~~~~   163 (426) |
|  |  |  | ++ ...|+.+.+++.+++.++|.++|+..+++.|.+++++++++|......... ++|.+..++.+++++++.+.. |
|  | T Consensus | 74 | ~~~~~~~~~r~~~~l~~~~~~~~~y~l~~~~~~~~~a~~a~~l~~~~p~~~~~~~~~--~~~~~~~~~~~~~~~~~~~~~   151 (388) |
|  | T PF03901.18 | 74 | SVQLLIWIPRLAQALLSAVADVRLYSLMKQLENQEVARWVFFCQLCSWFTWYCCTRT--LTNTMETVLTIIALFYYPLEG   151 (388) |
|  | T ss\_pred |  | CHHHHHHHHHHHHHHHHHHHHHHHHHHHHHHcChhHHHHHHHHHHHhHHHHHHHhhh--chHHHHHHHHHHHHHHHHHHh |
|  |
|  |
|  | Q ss\_pred |  | CCCCcccHHHHHHHHHHHHHHHHhHhHHHHHHHHHHHHHHHHhcCCCccchhcccchhhcCCCCCChHHHHHHHHHHHHH |
|  | Q Q6ZXV5 | 164 | GPDNSIIWTPIALTVFLVAVATLCKEQGITVVGICCVYEVFIAQGYTLPLLCTTAGQFLRGKGSIPFSMLQTLVKLIVLM   243 (426) |
|  | Q Consensus | 164 | ~~~~~~~~~~~~~~~~~~~la~~~k~~~~~~~~~~~~~~~~~~~~~~~~~~~~~~~~~~~~~~~~~~~~~~~~~~~~~~~   243 (426) |
|  |  |  | ++++ .+.. ++++.+++.++|+.+..+.+...++.+..+++ ++.+.......... |
|  | T Consensus | 152 | ~~~~---~~~~--~~~~~~l~~~~k~~~~~~~~~~~~~~~~~~~~---------------------~~~~~~~~~~~~~~   205 (388) |
|  | T PF03901.18 | 152 | SKSM---NSVK--YSSLVALAFIIRPTAVILWTPLLFRHFCQEPR---------------------KLDLILHHFLPVGF   205 (388) |
|  | T ss\_pred |  | CCCC---cHHH--HHHHHHHHHHhcchHHHHHHHHHHHHHHcChh---------------------cHHHHHHHHHHHHH |
|  |
|  |
|  | Q ss\_pred |  | HHHHHHHHHHHHHHccCCCccccCCCcccCCCchhhHHhHhhHHHHHHHHHhccccccccCccccCccccccccHHHHHH |
|  | Q Q6ZXV5 | 244 | FSTLLLVVIRVQVIQSQLPVFTRFDNPAAVSPTPTRQLTFNYLLPVNAWLLLNPSELCCDWTMGTIPLIESLLDIRNLAT   323 (426) |
|  | Q Consensus | 244 | ~~~~~~~~~~~~~~~~~~~~~~~~~~~~~~~~~~~~~~~~~~~~~~~~~~~~~~~~~~~~~~~~~~~~~~~~~~~~~~~~   323 (426) |
|  |  |  | +...++........++.................... ............... |
|  | T Consensus | 206 | ~~~~~~~~~~~~~~~~~~~~~~~~~~~~~~~~~~~~-----------------------------~~~~~~~~~~~~~~~   256 (388) |
|  | T PF03901.18 | 206 | VTLSLSLMIDRIFFGQWTLVQFNFLKFNVLQNWGTF-----------------------------YGSHPWHWYFSQGFP   256 (388) |
|  | T ss\_pred |  | HHHHHHHHHHHHHhcchhhhhhhhhhhhcccccccc-----------------------------cccCchHHHHHhhhh |
|  |
|  |
|  | Q ss\_pred |  | HHHHHHHHHHHHHHHHccCCCchhHHHHHHHHHHHHHHHhccCCCcchhhchhhchHHHHHHHHHHHHHHHHhcccchHH |
|  | Q Q6ZXV5 | 324 | FTFFCFLGMLGVFSIRYSGDSSKTVLMALCLMALPFIPASNLFFPVGFVVAERVLYVPSMGFCILVAHGWQKISTKSVFK   403 (426) |
|  | Q Consensus | 324 | ~~~~~~~~~~~~~~~~~~~~~~~~~~~~~~~~~~~~~~~~~~~~~~~~~~~~ry~~~~~~~~~il~~~~~~~~~~~~~~~   403 (426) |
|  |  |  | ......+........+++++++ ....+.+......... ....+||..+..|+++++++.++.+..++.+++ |
|  | T Consensus | 257 | ~~~~~~~~~~~~~~~~~~~~~~----~~~~~~~~~~~~~~~~-----~~~~~ry~~~~~p~~~l~~~~~~~~~~~~~~~~   327 (388) |
|  | T PF03901.18 | 257 | VILGTHLPFFIHGCYLAPKRYR----ILLVTVLWTLLVYSML-----SHKEFRFIYPVLPFCMVFCGYSLTHLKTWKKPA   327 (388) |
|  | T ss\_pred |  | hhHHhHHHHHHHHHHHchhhhH----HHHHHHHHHHHHHHhh-----cCCCcchHhcHHHHHHHHHHHHHHhhhcccchH |
|  |
|  |
|  | Q ss\_pred |  | HHHHHHHHHHHHHHHHHhhhhcC |
|  | Q Q6ZXV5 | 404 | KLSWICLSMVILTHSLKTFHRNW   426 (426) |
|  | Q Consensus | 404 | ~~~~~~~~~~~~~~~~~~~~~~~   426 (426) |
|  |  |  | ......+.++...........+. |
|  | T Consensus | 328 | ~~~~~~~~~~~~~~~~~~~~~~~   350 (388) |
|  | T PF03901.18 | 328 | LSFLFLSNLFLALYTGLVHQRGT   350 (388) |
|  | T ss\_pred |  | HHHHHHHHHHHHHHHHHHhhccc |
|  |
| --- | | | |
|  | Template alignmentCDD | | |
| 2. | PF02516.15 ; STT3 ; Oligosaccharyl transferase STT3 subunit | | |
|  | Probability: 99.86%, E-value: 1.8e-19, Score: 160.81, Aligned cols: 369, Identities: 11%, Similarity: 0.01, | | |
|  |
|  | Q ss\_pred |  | hHHHHHHHHHHHHHHH-----HHhhCCCceeccHHHHhcCCCCCCCCc--HHHHhccccCCCCCCcchhcCCCCcchHHH |
|  | Q Q6ZXV5 | 5 | NLKEITLIVGVVTACY-----WNSLFCGFVFDDVSAILDNKDLHPSTP--LKTLFQNDFWGTPMSEERSHKSYRPLTVLT   77 (426) |
|  | Q Consensus | 5 | ~~~~~~~l~~~~~~~~-----~~~~~~~~~~Dd~~~~~~~~~~~~~~~--~~~~~~~~~~~~~~~~~~~~~~~~Pl~~~~   77 (426) |
|  |  |  | ....+++++++++.++ ..........||..+...+++..+++. .......+.++....+......+||++.++ |
|  | T Consensus | 3 | ~~~~l~~i~~~~~~~r~~~~~~~~~~~~~~~D~~~~~~~a~~~~~~~~~~~~~~~~~~~~~~~~~~~~~~~~~~p~~~~l   82 (458) |
|  | T PF02516.15 | 3 | SRIETAELKGMNTADRAYFTDENGLPYMYEPDSYYNYRLTANILDHGHPGDKIINGTPWDLHSNYPPGNRVNYPPLILWI   82 (458) |
|  | T ss\_pred |  | hhHhHHHHHhhhhhhHHhCCCCCCCCcccCCCcHHHHHHHHHHHHhCCCCCcccCCCCCCchhcCCCCCCCCCCchHHHH |
|  |
|  |
|  | Q ss\_pred |  | HHHHHHHhCCCc-----hHHHHHHHHHHHHHHHHHHHHHHHhcCCHHHHHHHHHHHHCcccHHHHHHhhccHHHHHHHHH |
|  | Q Q6ZXV5 | 78 | FRLNYLLSELKP-----MSYHLLNMIFHAVVSVIFLKVCKLFLDNKSSVIASLLFAVHPIHTEAVTGVVGRAELLSSIFF   152 (426) |
|  | Q Consensus | 78 | ~~~~~~lfg~~~-----~~~rl~~~l~~~~~~~~~y~l~~~~~~~~~a~~aall~~~~p~~~~~~~~~~~~~~~~~~~~~   152 (426) |
|  |  |  | .+....++|... ...|+++.+++++++..+|.++|+..+++.|++++++++++|.+..........+|.+..++. |
|  | T Consensus | 83 | ~~~~~~~~~~~~~~~~~~~~~~~~~~~~~l~~~~~y~l~~~~~~~~~a~~a~~l~~~~p~~~~~~~~~~~~~~~~~~~~~   162 (458) |
|  | T PF02516.15 | 83 | SLLFHNFINLFIPFSLIETCFWLPAIIGPLAGIVMFFMVRRYAGDLPGLLSGVLLVLAPVYFSRTVPGFFDTDMFNIIFP   162 (458) |
|  | T ss\_pred |  | HHHHHHHHHhhCCCcHHHHHhHHHHHHHHHHHHHHHHHHHHHcCcHHHHHHHHHHHHcHHHHHhhCCCCCCchHHHHHHH |
|  |
|  |
|  | Q ss\_pred |  | HHHHHHHHHcCCCCCcccHHHHHHHHHHHHHHHHhHhHHHHHHHHHHHHHHHHhcCCCccchhcccchhhcCCCCCChHH |
|  | Q Q6ZXV5 | 153 | LAAFLSYTRSKGPDNSIIWTPIALTVFLVAVATLCKEQGITVVGICCVYEVFIAQGYTLPLLCTTAGQFLRGKGSIPFSM   232 (426) |
|  | Q Consensus | 153 | ~l~~~~~~~~~~~~~~~~~~~~~~~~~~~~la~~~k~~~~~~~~~~~~~~~~~~~~~~~~~~~~~~~~~~~~~~~~~~~~   232 (426) |
|  |  |  | +++++++.+..++++ +++++..+++++.+++..+|+......+..........++ +++.+... |
|  | T Consensus | 163 | ~l~~~~~~~~~~~~~-~~~~~~~l~g~~~~l~~~~~~~~~~~~~~~~~~~~~~~~~----------------~~~~~~~~   225 (458) |
|  | T PF02516.15 | 163 | LLVIFFLLKATETKN-NYMFPLLLSSFSLALLSLSWNGWAYIFYIIIISSILYMTL----------------CKLKGKAV   225 (458) |
|  | T ss\_pred |  | HHHHHHHHHHhccCC-CchHHHHHHHHHHHHHHhHhhHHHHHHHHHHHHHHHHHHH----------------hhcccchh |
|  |
|  |
|  | Q ss\_pred |  | HHHHHHHHHHHHHHHHHHHHHH-------------HHHccCCCccccCCCcccCCCchhhHHhHhhHHHHHHHHHhcccc |
|  | Q Q6ZXV5 | 233 | LQTLVKLIVLMFSTLLLVVIRV-------------QVIQSQLPVFTRFDNPAAVSPTPTRQLTFNYLLPVNAWLLLNPSE   299 (426) |
|  | Q Consensus | 233 | ~~~~~~~~~~~~~~~~~~~~~~-------------~~~~~~~~~~~~~~~~~~~~~~~~~~~~~~~~~~~~~~~~~~~~~   299 (426) |
|  |  |  | ...............++..... ....+...................... |
|  | T Consensus | 226 | ~~~~~~~~~~~~~~~~~~~~~~~~~~~~~~~~~~~~~~~~~~~~~~~~~~~~~~~~~~~~~~------------------   287 (458) |
|  | T PF02516.15 | 226 | MGFSRKIAVFVIISLLIIGLAGRLGYALIFPTFFKFTFKSLSAGGWPGIFESISELSAPTFD------------------   287 (458) |
|  | T ss\_pred |  | hHHHHHHHHHHHHHHHHhhhcccccHHHHHHHHHHHhcccccCCCCCcchHHHHHHhcccHH------------------ |
|  |
|  |
|  | Q ss\_pred |  | ccccCccccCccccccccHHHHHHHHHHHHHHHHHHHHHHccCCCchhHHHHHHHHHHHHHHHhccCCCcchhhchhhch |
|  | Q Q6ZXV5 | 300 | LCCDWTMGTIPLIESLLDIRNLATFTFFCFLGMLGVFSIRYSGDSSKTVLMALCLMALPFIPASNLFFPVGFVVAERVLY   379 (426) |
|  | Q Consensus | 300 | ~~~~~~~~~~~~~~~~~~~~~~~~~~~~~~~~~~~~~~~~~~~~~~~~~~~~~~~~~~~~~~~~~~~~~~~~~~~~ry~~   379 (426) |
|  |  |  | .............................++++++++.......+........... ....+||.. |
|  | T Consensus | 288 | ----------~~~~~~~~~~~~~~~~~~~~~~~~~~~~~~~~~~~~~~~~~~~~~~~~~~~~~~~~-----~~~~~Ry~~   352 (458) |
|  | T PF02516.15 | 288 | ----------EFLSLPGPVNMGIGLFGFVIIGSIMLRDEIKRVHLPDFSWYPFILIGIWLIIGLAA-----YSLSTRFAL   352 (458) |
|  | T ss\_pred |  | ----------HHhcCchHHHHHHHHHHHHHHHHHHHHHHHHhccCCCCccHHHHHHHHHHHHHHHH-----HHHHHhHHH |
|  |
|  |
|  | Q ss\_pred |  | HHHHHHHHHHHHHHHHhcccchHHH-----HHHHHHHHHHHHHHHHhhh |
|  | Q Q6ZXV5 | 380 | VPSMGFCILVAHGWQKISTKSVFKK-----LSWICLSMVILTHSLKTFH   423 (426) |
|  | Q Consensus | 380 | ~~~~~~~il~~~~~~~~~~~~~~~~-----~~~~~~~~~~~~~~~~~~~   423 (426) |
|  |  |  | +..|+++++++.++.+..++.+.++ .......+++......... |
|  | T Consensus | 353 | ~~~p~~~i~~~~~~~~~~~~~~~~~~~~~~~~~~~~~~~~~~~~~~~~~   401 (458) |
|  | T PF02516.15 | 353 | LVIPPLIIFLGLLMGVMASYLKGSPSMRLRRSGNVFILSLVVMLSTISF   401 (458) |
|  | T ss\_pred |  | hhHHHHHHHHHHHHHHHHHHhcCCccchHHHHHHHHHHHHHHHHHHHHH |
|  |
| --- | | | |
|  | Template alignmentCDD | | |
| 3. | PF09852.10 ; DUF2079 ; Predicted membrane protein (DUF2079) | | |
|  | Probability: 99.82%, E-value: 8e-17, Score: 145.85, Aligned cols: 346, Identities: 10%, Similarity: 0.038, | | |
|  |
|  | Q ss\_pred |  | CCceeccHHHHhcCCCCCCCCcHHHHhccccCCCCCCcchhcCCCCcchHHHHHHHHHHhCCCchHHHHHHHHHHHHHHH |
|  | Q Q6ZXV5 | 26 | CGFVFDDVSAILDNKDLHPSTPLKTLFQNDFWGTPMSEERSHKSYRPLTVLTFRLNYLLSELKPMSYHLLNMIFHAVVSV   105 (426) |
|  | Q Consensus | 26 | ~~~~~Dd~~~~~~~~~~~~~~~~~~~~~~~~~~~~~~~~~~~~~~~Pl~~~~~~~~~~lfg~~~~~~rl~~~l~~~~~~~   105 (426) |
|  |  |  | ....+||..+. ....+..++.....+..+......++|++.++.+..+.++| ++...|++++++++++++ |
|  | T Consensus | 6 | ~~~~~De~~~~---------~~a~~~~~g~~~~~~~~~~~~~~~~~pl~~~l~a~~~~l~g-~~~~~rl~~~l~~~~~~~   75 (519) |
|  | T PF09852.10 | 6 | NATAYDLGIYV---------SILENTMHGHVMYANPLLINSFSEHFSPFLFVIYPIYWFFP-YVKTLLIMQSVMISFSGL   75 (519) |
|  | T ss\_pred |  | CccccHHHHHH---------HHHHHHHcCCccccCccccccccccchhHHHHHHHHHHHcC-ChHHHHHHHHHHHHHHHH |
|  |
|  |
|  | Q ss\_pred |  | HHHHHHHHhcC----------CHHHHHHHHHHHHCcccHHHHHH-hhccHHHHHHHHHHHHHHHHHHcCCCCCcccHHHH |
|  | Q Q6ZXV5 | 106 | IFLKVCKLFLD----------NKSSVIASLLFAVHPIHTEAVTG-VVGRAELLSSIFFLAAFLSYTRSKGPDNSIIWTPI   174 (426) |
|  | Q Consensus | 106 | ~~y~l~~~~~~----------~~~a~~aall~~~~p~~~~~~~~-~~~~~~~~~~~~~~l~~~~~~~~~~~~~~~~~~~~   174 (426) |
|  |  |  | ++|+++|+.++ ++.|++++++++++|..... . ...+.|.+..++.+++++++.+.++ + |
|  | T Consensus | 76 | ~~y~l~r~~~~~~~~~~~~~~~~~al~a~ll~~~~p~~~~~--~~~~~~~~~~~~~~~~~~~~~~~~~~~------~---   144 (519) |
|  | T PF09852.10 | 76 | VIYLLAREIFFINNFKKDILLEMLALFISTSYILSPYIESP--LSFDFHLMPFLILFVPLSFYFFMKKYK------I---   144 (519) |
|  | T ss\_pred |  | HHHHHHHHHhccCcccchhHHHHHHHHHHHHHHHcHHhhhh--hhcCCchHHHHHHHHHHHHHHHHhhcc------H--- |
|  |
|  |
|  | Q ss\_pred |  | HHHHHHHHHHHHhHhHHHHHHHHHHHHHHHH-----------------hcCCCccchhcccchhhcCCCCCChHHHHHHH |
|  | Q Q6ZXV5 | 175 | ALTVFLVAVATLCKEQGITVVGICCVYEVFI-----------------AQGYTLPLLCTTAGQFLRGKGSIPFSMLQTLV   237 (426) |
|  | Q Consensus | 175 | ~~~~~~~~la~~~k~~~~~~~~~~~~~~~~~-----------------~~~~~~~~~~~~~~~~~~~~~~~~~~~~~~~~   237 (426) |
|  |  |  | .++++++++++++|+.++++.+...++.+.. .++......+.++.+...+++...+....... |
|  | T Consensus | 145 | ~~~gl~~~la~l~k~~~~~~~~~~~l~~~~~~~~~~~~~~~~~~~~~~~~~~~~~~~~~~~~~~~~~~~~~~~~~~~~~~   224 (519) |
|  | T PF09852.10 | 145 | LNLIVLILIISLHSLFVIMVFFIISYQFIFRIRNEGNLNCHKIIRTIANINISDNLKKTPKSKYVLQKIVRSKTLIKIII   224 (519) |
|  | T ss\_pred |  | HHHHHHHHHHHHhcchHHHHHHHHHHHHHHHHhccCCCcchhhhHHhhhcccccccccCCcchhhhhhhhcchHHHHHHH |
|  |
|  |
|  | Q ss\_pred |  | HHHHHHHHHHHHHHHHHHHHccCCCccccCCCcccC--CCchhhHHhHhhHHHHHHHHHhccccccccCccccCcccccc |
|  | Q Q6ZXV5 | 238 | KLIVLMFSTLLLVVIRVQVIQSQLPVFTRFDNPAAV--SPTPTRQLTFNYLLPVNAWLLLNPSELCCDWTMGTIPLIESL   315 (426) |
|  | Q Consensus | 238 | ~~~~~~~~~~~~~~~~~~~~~~~~~~~~~~~~~~~~--~~~~~~~~~~~~~~~~~~~~~~~~~~~~~~~~~~~~~~~~~~   315 (426) |
|  |  |  | .....++..+.+........................ .+..+................... |
|  | T Consensus | 225 | ~~~~~~~~~~~~~~~~~~~~~~~~~~~~~~~~~~~~~~~~~~~~~~~~~~~~~~~~~~~~~~------------------   286 (519) |
|  | T PF09852.10 | 225 | TLILLVGYLYFASLMKTFIASGAVALSPPSTMSTGSVSSSLAGLFTDLFTRPMLIESAFLIN------------------   286 (519) |
|  | T ss\_pred |  | HHHHHHHHHHHHHHHHHHHHccccccCCCcccCCCCcCCcHHHHHHHHhhCchHHHHHHhcc------------------ |
|  |
|  |
|  | Q ss\_pred |  | ccHHHHHHHHHHHHHHHHHHHHHHccCCCchhHHHHHHHHHHHHHHHhccCCCcchhhchhhchHHHHHHHHHHHHHHHH |
|  | Q Q6ZXV5 | 316 | LDIRNLATFTFFCFLGMLGVFSIRYSGDSSKTVLMALCLMALPFIPASNLFFPVGFVVAERVLYVPSMGFCILVAHGWQK   395 (426) |
|  | Q Consensus | 316 | ~~~~~~~~~~~~~~~~~~~~~~~~~~~~~~~~~~~~~~~~~~~~~~~~~~~~~~~~~~~~ry~~~~~~~~~il~~~~~~~   395 (426) |
|  |  |  | ..................... +.......+.......+. ..........||..+..|+++++++.++.+ |
|  | T Consensus | 287 | --~~~~~~~~~~~~~~~~~~~~~-----~~~~~~~~~~~~~~~~~~----~~~~~~~~~~ry~~~~~p~l~i~~~~~~~~   355 (519) |
|  | T PF09852.10 | 287 | --FPDKIIFAFYAFANTGFLVFL-----DPLSLLMDIPYFLYAYLS----SYGPYYSLGYQYSTMIIPFIFIGALFGIRK   355 (519) |
|  | T ss\_pred |  | --chHHHHHHHHHHHHHhHHHhc-----CHHHHHhHHHHHHHHHhc----cCccccchhhHHHHhHHHHHHHHHHHHHHH |
|  |
|  |
|  | Q ss\_pred |  | hc---------ccchHHHHHHHHHHHHHHHHHHHh |
|  | Q Q6ZXV5 | 396 | IS---------TKSVFKKLSWICLSMVILTHSLKT   421 (426) |
|  | Q Consensus | 396 | ~~---------~~~~~~~~~~~~~~~~~~~~~~~~   421 (426) |
|  |  |  | +. ++.+..+.....+++++++..... |
|  | T Consensus | 356 | ~~~~~~~~~~~~~~~~~~~~~~~~~~~~~~~~~~~   390 (519) |
|  | T PF09852.10 | 356 | IVQSARATDSDDVRRTIKKILVGVISIVIVSSLFE   390 (519) |
|  | T ss\_pred |  | HHHHhccCCCcchhHhHHHHHHHHHHHHHHHHHHh |
|  |
| --- | | | |
|  | Template alignmentCDD | | |
| 4. | PF10131.10 ; PTPS\_related ; 6-pyruvoyl-tetrahydropterin synthase related domain; membrane protein | | |
|  | Probability: 99.77%, E-value: 2.5e-16, Score: 145.77, Aligned cols: 306, Identities: 9%, Similarity: -0.044, | | |
|  |
|  | Q ss\_pred |  | cCCCCcchHHHHHHHHHHh-CCCchHHHHHHHHHHHHHHHHHHHHHHHhcCCHHHHHHHHHHHHCcccHHHHHHhhccHH |
|  | Q Q6ZXV5 | 67 | HKSYRPLTVLTFRLNYLLS-ELKPMSYHLLNMIFHAVVSVIFLKVCKLFLDNKSSVIASLLFAVHPIHTEAVTGVVGRAE   145 (426) |
|  | Q Consensus | 67 | ~~~~~Pl~~~~~~~~~~lf-g~~~~~~rl~~~l~~~~~~~~~y~l~~~~~~~~~a~~aall~~~~p~~~~~~~~~~~~~~   145 (426) |
|  |  |  | ...|||+++++.+....++ |.+..+.|+.++++++++++.+|.++|+..+++.|++++++++++|.+.....+...++| |
|  | T Consensus | 1 | f~~~pPl~~~l~~~~~~l~~g~~~~~~~l~~~l~~~l~~~~~y~l~r~~~~~~~a~~a~~l~~~~p~~~~~~~~~~~~~~   80 (616) |
|  | T PF10131.10 | 1 | FRYWGPLSYYIMAGLMFLTSGDLLLAYRLIAFVIFVVGGLPWILWGIHENRRVLGTFFGVLWFFMPEHIRIYFTAGNLPQ   80 (616) |
|  | T ss\_pred |  | CCCCchHHHHHHHHHHHHhcCCHHHHHHHHHHHHHHHHHHHHHHHHHHcCcHHHHHHHHHHHHHCchHHHHHHhcCCHHH |
|  |
|  |
|  | Q ss\_pred |  | HHHHHHHHHHHHHHHHcCCCCCcccHHHHHHHHHHHHHHHHhHhHHHHHHHHHHHHHHHHhcCCCccchhcccchhhcCC |
|  | Q Q6ZXV5 | 146 | LLSSIFFLAAFLSYTRSKGPDNSIIWTPIALTVFLVAVATLCKEQGITVVGICCVYEVFIAQGYTLPLLCTTAGQFLRGK   225 (426) |
|  | Q Consensus | 146 | ~~~~~~~~l~~~~~~~~~~~~~~~~~~~~~~~~~~~~la~~~k~~~~~~~~~~~~~~~~~~~~~~~~~~~~~~~~~~~~~   225 (426) |
|  |  |  | .+..++.+++++++.+..++++ +++.++++++.+++.++|+....+.++..+........ |
|  | T Consensus | 81 | ~~~~~~~~l~l~~~~~~~~~~~---~~~~~~~~l~~~l~~~~~~~~~~~~~~~~~~~~~~~~~-----------------   140 (616) |
|  | T PF10131.10 | 81 | MVTTMLVPYVIWFLWLYVRKKN---NRAAVGLFVCMTLMSFTHLMVTAIMGVSAFLYLLIDQI-----------------   140 (616) |
|  | T ss\_pred |  | HHHHHHHHHHHHHHHHHHhcCC---HHHHHHHHHHHHHHHHhcHHHHHHHHHHHHHHHHHHHH----------------- |
|  |
|  |
|  | Q ss\_pred |  | CCCChHHHHHHHHHHHHHHHHHHHHHHHHHHHccCCCccccCCCcccCCCchhhHHhHhhHHHHHHHHHhccccccccCc |
|  | Q Q6ZXV5 | 226 | GSIPFSMLQTLVKLIVLMFSTLLLVVIRVQVIQSQLPVFTRFDNPAAVSPTPTRQLTFNYLLPVNAWLLLNPSELCCDWT   305 (426) |
|  | Q Consensus | 226 | ~~~~~~~~~~~~~~~~~~~~~~~~~~~~~~~~~~~~~~~~~~~~~~~~~~~~~~~~~~~~~~~~~~~~~~~~~~~~~~~~   305 (426) |
|  |  |  | .+++.++........++..+....+.................. .............. |
|  | T Consensus | 141 | --~~~~~~~~~~~~~~~~~~~~~~~~~~~~~~~~~~~~~~~~~~~-~~~~~~~~~~~~~~--------------------   197 (616) |
|  | T PF10131.10 | 141 | --WNKDTRRKIFALIYMICGILTAGIWVIPSLKGGLVTSESGDGS-VMSTLIYPLTTSLN--------------------   197 (616) |
|  | T ss\_pred |  | --hCCChHHHHHHHHHHHHHHHHHHHHHHHHHccCCccCCCCccc-hhhhheechhhccC-------------------- |
|  |
|  |
|  | Q ss\_pred |  | cccCccccccccHHHHHHHHHHHHHHHHHHHHHHccCCCchhHHHHHHHHHHHHHHHhccCCCcchhhchhhchHHHHHH |
|  | Q Q6ZXV5 | 306 | MGTIPLIESLLDIRNLATFTFFCFLGMLGVFSIRYSGDSSKTVLMALCLMALPFIPASNLFFPVGFVVAERVLYVPSMGF   385 (426) |
|  | Q Consensus | 306 | ~~~~~~~~~~~~~~~~~~~~~~~~~~~~~~~~~~~~~~~~~~~~~~~~~~~~~~~~~~~~~~~~~~~~~~ry~~~~~~~~   385 (426) |
|  |  |  | ..................+++.+.......++++.........+.......................||..+. ++ |
|  | T Consensus | 198 | ---~~~~~~~~~~~~~~~~~~~~l~~~~~~~~~~~~~~~~~~~~~~~~~~~~~~~~~~~~~~~~~~~~~~r~~~~~--~~   272 (616) |
|  | T PF10131.10 | 198 | ---PFKRLSAGNDSFYFGLAAVLIAIAGILLARGGKKAGFVFLLIMLACTTPAAYRILVKLPFSQLFWMTRFAPMV--YG   272 (616) |
|  | T ss\_pred |  | ---cchhccCCCCcchHHHHHHHHHHHHHHHHcCCChHHHHHHHHHHHhcchHHHHHHhhCCHHHhhhHhHHHHHH--HH |
|  |
|  |
|  | Q ss\_pred |  | HHHHHHHHHHhcccchHHHHHHHHHHHHHHHHHHHhhhhc |
|  | Q Q6ZXV5 | 386 | CILVAHGWQKISTKSVFKKLSWICLSMVILTHSLKTFHRN   425 (426) |
|  | Q Consensus | 386 | ~il~~~~~~~~~~~~~~~~~~~~~~~~~~~~~~~~~~~~~   425 (426) |
|  |  |  | +++++.++.+..++ ....+++++.+.......... |
|  | T Consensus | 273 | ~~~~~~~~~~~~~~-----~~~~~~~~~~~~~~~~~~~~~   307 (616) |
|  | T PF10131.10 | 273 | FFFSACLEWVRLKK-----KYCVLLAALLCVDSISCMNLD   307 (616) |
|  | T ss\_pred |  | HHHHHHHHhhhhHH-----HHHHHHHHHHHHHHhhhcccc |
|  |
| --- | | | |
|  | Template alignmentCDD | | |
| 5. | PF07220.12 ; DUF1420 ; Protein of unknown function (DUF1420) | | |
|  | Probability: 99.76%, E-value: 5e-15, Score: 138.24, Aligned cols: 358, Identities: 11%, Similarity: 0.035, | | |
|  |
|  | Q ss\_pred |  | cchhHHHHHHHHHHHHHHHHHhhCCCceeccHHHHhcCCCCCCCCcHHHHhccccCCCCCCcchhcCCCCcchHHHHHHH |
|  | Q Q6ZXV5 | 2 | ANINLKEITLIVGVVTACYWNSLFCGFVFDDVSAILDNKDLHPSTPLKTLFQNDFWGTPMSEERSHKSYRPLTVLTFRLN   81 (426) |
|  | Q Consensus | 2 | ~~~~~~~~~~l~~~~~~~~~~~~~~~~~~Dd~~~~~~~~~~~~~~~~~~~~~~~~~~~~~~~~~~~~~~~Pl~~~~~~~~   81 (426) |
|  |  |  | .+.......+++++...........+..+||..|+..... ++.+++.... .+......+||....+.... |
|  | T Consensus | 153 | ~~~~~~l~~~i~~~~~~~~~~~~~p~~~~D~~~yhl~~a~--------~~~~~g~~~~--~~~~~~~~~P~~~~~l~~~~   222 (670) |
|  | T PF07220.12 | 153 | INKNDVLNVFIILLMIGYGFLALCPITNADSLDYHIGVAI--------EILNQGKMPV--FSGWFHGRLAGSGEVLNALG   222 (670) |
|  | T ss\_pred |  | CChhHHHHHHHHHHHHHHHHHHcCCCCCCHHHHHHHHHHH--------HHHHcCCCCC--CCCchhhcCCchHHHHHHHH |
|  |
|  |
|  | Q ss\_pred |  | HHHhCCCchHHHHHHHHHHHHHHHHHHHHHHHhcCC--HHHHHHHHHHHHCcccHHHHHHhhccH---HHHHHHHHHHHH |
|  | Q Q6ZXV5 | 82 | YLLSELKPMSYHLLNMIFHAVVSVIFLKVCKLFLDN--KSSVIASLLFAVHPIHTEAVTGVVGRA---ELLSSIFFLAAF   156 (426) |
|  | Q Consensus | 82 | ~~lfg~~~~~~rl~~~l~~~~~~~~~y~l~~~~~~~--~~a~~aall~~~~p~~~~~~~~~~~~~---~~~~~~~~~l~~   156 (426) |
|  |  |  | ..+.|+......-.+.+++++++.++|.++|+.+++ ..+.+++++++++|.. ......+.+ |...+++.+.++ |
|  | T Consensus | 223 | ~~l~~~~~~~~~~~~~l~~~l~~~~~y~l~r~~~~~~~~~a~~aall~~~~p~~--~~~s~~~~~~~~d~~~~~~~~~~l   300 (670) |
|  | T PF07220.12 | 223 | LAIGAEQFGSLLQFCGLLSIYGILSFYSFAEKFSESDGVWRKIIIIAFLSSPVL--VFLVSSPKPQLLQIGMTSFAITLL   300 (670) |
|  | T ss\_pred |  | HHHcchhHHHHHHHHHHHHHHHHHHHHHHHHHhcCCCCHHHHHHHHHHHhchHH--HHHhcCCchHHHHHHHHHHHHHHH |
|  |
|  |
|  | Q ss\_pred |  | HHHHHcCCCCCcccHHHHHHHHHH--HHHHHHhHhHHHHHHHHHHHHHHHHhcCCCccchhcccchhhcCCCCCChHHHH |
|  | Q Q6ZXV5 | 157 | LSYTRSKGPDNSIIWTPIALTVFL--VAVATLCKEQGITVVGICCVYEVFIAQGYTLPLLCTTAGQFLRGKGSIPFSMLQ   234 (426) |
|  | Q Consensus | 157 | ~~~~~~~~~~~~~~~~~~~~~~~~--~~la~~~k~~~~~~~~~~~~~~~~~~~~~~~~~~~~~~~~~~~~~~~~~~~~~~   234 (426) |
|  |  |  | +.+.+..++++ .++..+++++ +|+|..+|++++.+.++..+..+...++ ++..++.... |
|  | T Consensus | 301 | ~~~~~~~~~~~---~~~~~l~gl~~~~gla~~~K~~~~~~~~~~~~~~l~~~~~----------------~~~~~~~~~~   361 (670) |
|  | T PF07220.12 | 301 | LEIFSKIKTDK---NKLFAFSLICILIMSATQAKFSFFLSAFLIGLFSIFSLGS----------------IRLFFYGLLI   361 (670) |
|  | T ss\_pred |  | HHHHHhhccCC---chHHHHHHHHHHHHHHHhhHHHHHHHHHHHHHHHHHHhcc----------------cchhHHHHHH |
|  |
|  |
|  | Q ss\_pred |  | HHHHHHHHHHHHHHHHHHHHHHHccCCCccccCCCcc-cCCCchhhHHhHhhHHHHHHHHHhccccccccCccccCcccc |
|  | Q Q6ZXV5 | 235 | TLVKLIVLMFSTLLLVVIRVQVIQSQLPVFTRFDNPA-AVSPTPTRQLTFNYLLPVNAWLLLNPSELCCDWTMGTIPLIE   313 (426) |
|  | Q Consensus | 235 | ~~~~~~~~~~~~~~~~~~~~~~~~~~~~~~~~~~~~~-~~~~~~~~~~~~~~~~~~~~~~~~~~~~~~~~~~~~~~~~~~   313 (426) |
|  |  |  | .....+...++..++........+............. ......+..................+...... |
|  | T Consensus | 362 | ~~~~~~~~~~~~~~w~~~~~~~~g~~~~~~~~~~~~~~~~~~~~~~~~~~~~~~~~~~~~~~~~~~~~~~----------   431 (670) |
|  | T PF07220.12 | 362 | SLFFFVLINFPAIFWKIKNYNSTFIDVLIHPLPGNTFPGVNEFEVSLRNYQDSALIFPLSLIFPNQFGVI----------   431 (670) |
|  | T ss\_pred |  | HHHHHHHHHHHHHHHHHHHHHccCCcccccCCCCCCCCCccchHHHHHhcccccccCcHHhhccccchhh---------- |
|  |
|  |
|  | Q ss\_pred |  | ccccHHHHHHHHHHHHHHHHHHHHHHccCCCchhHHHHHHHHHHHHHHHhccCCCcchhhchhhchHHHHHHHHHHHHHH |
|  | Q Q6ZXV5 | 314 | SLLDIRNLATFTFFCFLGMLGVFSIRYSGDSSKTVLMALCLMALPFIPASNLFFPVGFVVAERVLYVPSMGFCILVAHGW   393 (426) |
|  | Q Consensus | 314 | ~~~~~~~~~~~~~~~~~~~~~~~~~~~~~~~~~~~~~~~~~~~~~~~~~~~~~~~~~~~~~~ry~~~~~~~~~il~~~~~   393 (426) |
|  |  |  | ...+..........++++++.......+........... .....||..|..|++++ ++.+. |
|  | T Consensus | 432 | -------------~~~~~~~~~~~~~~~~~~~~~~~~~~~~~~~~~~~~~~~-----~~~~~Ry~lp~~p~l~l-~~~~~   492 (670) |
|  | T PF07220.12 | 432 | -------------TTVIGLGLFLIIFVKPIVTQKAFLLSVMIILFVILGSLM-----GQKASRFFLEPFVWMLI-SLIGL   492 (670) |
|  | T ss\_pred |  | -------------HhHHHHHHHHHHHhcchhchHHHHHHHHHHHHHHHHHHh-----ccchHHhhHHHHHHHHH-HHHHH |
|  |
|  |
|  | Q ss\_pred |  | HHhcccchHHHHHHHHHHHHHHHHHH |
|  | Q Q6ZXV5 | 394 | QKISTKSVFKKLSWICLSMVILTHSL   419 (426) |
|  | Q Consensus | 394 | ~~~~~~~~~~~~~~~~~~~~~~~~~~   419 (426) |
|  |  |  | ....+..+++.......++.+..... |
|  | T Consensus | 493 | ~~~~~~~~~~~~~~~~~~~~~~~~~~   518 (670) |
|  | T PF07220.12 | 493 | NSFGKWNIRFVKEAVSTGILLQACAT   518 (670) |
|  | T ss\_pred |  | HhhccccHHHHHHHHHHHHHHHHHHH |
|  |
| --- | | | |
|  | Template alignmentCDD | | |
| 6. | PF04188.14 ; Mannosyl\_trans2 ; Mannosyltransferase (PIG-V) | | |
|  | Probability: 99.71%, E-value: 3.8e-14, Score: 124.48, Aligned cols: 336, Identities: 11%, Similarity: -0.049, | | |
|  |
|  | Q ss\_pred |  | eccHHHHh--cCCCC-CCCCcHHHHhccccCCCCCCcchhcCCCCcchHHHHHHHHHHhCCC-----------chHHHHH |
|  | Q Q6ZXV5 | 30 | FDDVSAIL--DNKDL-HPSTPLKTLFQNDFWGTPMSEERSHKSYRPLTVLTFRLNYLLSELK-----------PMSYHLL   95 (426) |
|  | Q Consensus | 30 | ~Dd~~~~~--~~~~~-~~~~~~~~~~~~~~~~~~~~~~~~~~~~~Pl~~~~~~~~~~lfg~~-----------~~~~rl~   95 (426) |
|  |  |  | |||.+|.. .+++. ...+....+ +|+++++......++|.+ ..+.|++ |
|  | T Consensus | 60 | wD~~~y~~~~ia~~g~y~~~~~~~f-------------------~Pl~p~l~~~~~~l~~~~~~~~~~~~~~~~~~~~~~   120 (432) |
|  | T PF04188.14 | 60 | WDSVFFIKNITSKNGKPQFEHEYAF-------------------SQLWTFFVRLFIKSNNDSIYHALRVGVAIENVLFYL   120 (432) |
|  | T ss\_pred |  | ccHHHHhhhhHHHcCCCcccccccc-------------------hHHHHHHHHHHHHhcccchHHHHHHHHHHHHHHHHH |
|  |
|  |
|  | Q ss\_pred |  | H-HHHHHHHHHHHHHHHHHhc-CCHHHHHHHHHHHHCcccHHHHHHhhccHHHHHHHHHHHHHHHHHHcCCCCCcccHHH |
|  | Q Q6ZXV5 | 96 | N-MIFHAVVSVIFLKVCKLFL-DNKSSVIASLLFAVHPIHTEAVTGVVGRAELLSSIFFLAAFLSYTRSKGPDNSIIWTP   173 (426) |
|  | Q Consensus | 96 | ~-~l~~~~~~~~~y~l~~~~~-~~~~a~~aall~~~~p~~~~~~~~~~~~~~~~~~~~~~l~~~~~~~~~~~~~~~~~~~   173 (426) |
|  |  |  | + .+++++++..+|.++|+.. +++.|..++++++++|.. ... ....+|.+..++.+++++++.+..+++++ .++ |
|  | T Consensus | 121 | s~~~~~~~~~~~ly~l~~~~~~~~~~a~~a~~l~~~~P~~--~~~-~~~~~E~l~~~l~~~~~~~~~~~~~~~~~--~~~   195 (432) |
|  | T PF04188.14 | 121 | SGIVLYFLTKKIFSQNIRQSQFARTIAKKTSLLFFLTSAA--GFL-TSIYSEPLSFFFAFVGIWSRECSISVPVL--GQF   195 (432) |
|  | T ss\_pred |  | HHHHHHHHHHHHHHhhhHhhhccHHHHHHHHHHHhhCCch--HHh-hccCcHHHHHHHHHHHHHHHHhccCCCCC--Cch |
|  |
|  |
|  | Q ss\_pred |  | HHHHHHHHHHHHH-hHhHHHHHHHHHHHHHHH--------HhcCCCccchhcccchhhcCCCCCChHHHHHHHHHHHHHH |
|  | Q Q6ZXV5 | 174 | IALTVFLVAVATL-CKEQGITVVGICCVYEVF--------IAQGYTLPLLCTTAGQFLRGKGSIPFSMLQTLVKLIVLMF   244 (426) |
|  | Q Consensus | 174 | ~~~~~~~~~la~~-~k~~~~~~~~~~~~~~~~--------~~~~~~~~~~~~~~~~~~~~~~~~~~~~~~~~~~~~~~~~   244 (426) |
|  |  |  | ...++++.+++.+ +|.+++...+......+. .+++ .++..+.........++ |
|  | T Consensus | 196 | ~~~~~~~~~la~~~~R~~g~~~~~~~~~~~l~~~~~~~~~~~~~-------------------~~~~~~~~~~~~~~~~~   256 (432) |
|  | T PF04188.14 | 196 | DISWRYWFPYSFISMACFTLASLNRSNCVLLGIYFIFDLIELTK-------------------NRKFVKAICFPLLSGSL   256 (432) |
|  | T ss\_pred |  | hHHHHHHHHHHHHHHHhHHHHHHHHHHHHHHHHHHHHHHHHhhh-------------------hHHHHHHHHHHHHHHHH |
|  |
|  |
|  | Q ss\_pred |  | HHHHHHHHHHHH-HccCCCccccCCCcccCCCchhhHHhHhhHHHHHHHHHhccccccccCccccCccccccccHHHHHH |
|  | Q Q6ZXV5 | 245 | STLLLVVIRVQV-IQSQLPVFTRFDNPAAVSPTPTRQLTFNYLLPVNAWLLLNPSELCCDWTMGTIPLIESLLDIRNLAT   323 (426) |
|  | Q Consensus | 245 | ~~~~~~~~~~~~-~~~~~~~~~~~~~~~~~~~~~~~~~~~~~~~~~~~~~~~~~~~~~~~~~~~~~~~~~~~~~~~~~~~   323 (426) |
|  |  |  | ...++....... .+................. .....+....+.....+..+....+..... +..... |
|  | T Consensus | 257 | ~~~p~~~~~~~~~~~~f~~~~~~w~~~~~~~~----~~~~~~~~~~~~q~~yw~~g~~~~~~~~~~--------~~~ll~   324 (432) |
|  | T PF04188.14 | 257 | MFSALLYQQYYLPYKTFCPQRGEWCKSQLFSS----IFITKTSLYSYIQSHYWGVGLLKYWTPNNI--------PNFLFA   324 (432) |
|  | T ss\_pred |  | HHHHHHHHHHHHHHHHHCCCCChhHhCCCCCc----hhhcccchHHHHHHHcCCCcchhcCCccch--------hHHHhH |
|  |
|  |
|  | Q ss\_pred |  | HHHHHHHHHHHHHHHHccCCCchhHHHHHHHHHHHHHHHhccCCCcchhhchhhchHHHHHHHHHHHHHHHHhcccchHH |
|  | Q Q6ZXV5 | 324 | FTFFCFLGMLGVFSIRYSGDSSKTVLMALCLMALPFIPASNLFFPVGFVVAERVLYVPSMGFCILVAHGWQKISTKSVFK   403 (426) |
|  | Q Consensus | 324 | ~~~~~~~~~~~~~~~~~~~~~~~~~~~~~~~~~~~~~~~~~~~~~~~~~~~~ry~~~~~~~~~il~~~~~~~~~~~~~~~   403 (426) |
|  |  |  | .......+..+....++++++..........+.......... .....||.. ..|++.+.++..+.+..+++++. |
|  | T Consensus | 325 | ~p~~~l~~~~~~~~~~~~~~~~~~~~~~~~~~~~~~~~~~~~-----~~~~~R~~~-~~P~l~~~~a~~~~~~~~~~~~~   398 (432) |
|  | T PF04188.14 | 325 | VPNIIILIYSSIYFSKIYPSYNLKALVWITRALVVIVCFFAH-----VQILNRIAS-FLPLHLWYLADRLVKTSDPKKME   398 (432) |
|  | T ss\_pred |  | HHHHHHHHHHHHHHHhhCCccccHHHHHHHHHHHHHHHHHHH-----HHHHHHHHh-ccHHHHHHHHHHHhcCCCCCcCC |
|  |
|  |
|  | Q ss\_pred |  | H--------HHHHHHHHHHHHHHHHhhhhcC |
|  | Q Q6ZXV5 | 404 | K--------LSWICLSMVILTHSLKTFHRNW   426 (426) |
|  | Q Consensus | 404 | ~--------~~~~~~~~~~~~~~~~~~~~~~   426 (426) |
|  |  |  | . .......++....+...+..-+ |
|  | T Consensus | 399 | ~~~~~~~~~~~~~~~~~~~~~~~~~l~~~f~   429 (432) |
|  | T PF04188.14 | 399 | NPKGDDKIVKFYIYWLAFWIPLQTILFAAFL   429 (432) |
|  | T ss\_pred |  | CCCCCCHHHHHHHHHHHHHHHHHHHHHHccC |
|  |
| --- | | | |
|  | Template alignmentCDD | | |
| 7. | PF02366.19 ; PMT ; Dolichyl-phosphate-mannose-protein mannosyltransferase | | |
|  | Probability: 99.66%, E-value: 1.5e-14, Score: 117.06, Aligned cols: 215, Identities: 13%, Similarity: 0.083, | | |
|  |
|  | Q ss\_pred |  | HHHHHHHHHHHHHHhhCCCceeccHHHHhcCCCCCCCCcHHHHhccccCCCCCCcchhcCCCCcchHHHHHHHHHHhC-- |
|  | Q Q6ZXV5 | 9 | ITLIVGVVTACYWNSLFCGFVFDDVSAILDNKDLHPSTPLKTLFQNDFWGTPMSEERSHKSYRPLTVLTFRLNYLLSE--   86 (426) |
|  | Q Consensus | 9 | ~~~l~~~~~~~~~~~~~~~~~~Dd~~~~~~~~~~~~~~~~~~~~~~~~~~~~~~~~~~~~~~~Pl~~~~~~~~~~lfg--   86 (426) |
|  |  |  | .++++.............+..+||..+...+++..+++. ....++|............+| |
|  | T Consensus | 2 | ~~~~~~~~~~~~~~~~~~~~~~D~~~~~~~a~~~~~~~~------------------~~~~~~~~~~~~~~~~~~~~~~~   63 (247) |
|  | T PF02366.19 | 2 | FLTVVAFCVRAQRLMNPAKVVFEELRYYNYAVDYVNNKL------------------LMDVYPPLGKLLFSLVAALTGNK   63 (247) |
|  | T ss\_pred |  | hHHHHHHHHHHHHHhCCcccchHHHHHHHHHHHHHcCcc------------------ccCCCCcHHHHHHHHHHHHcCCC |
|  |
|  |
|  | Q ss\_pred |  | ----------------CCchHHHHHHHHHHHHHHHHHHHHHHHhc-CCHHHHHHHHHHHHCcccHHHHHHhhccHHHHHH |
|  | Q Q6ZXV5 | 87 | ----------------LKPMSYHLLNMIFHAVVSVIFLKVCKLFL-DNKSSVIASLLFAVHPIHTEAVTGVVGRAELLSS   149 (426) |
|  | Q Consensus | 87 | ----------------~~~~~~rl~~~l~~~~~~~~~y~l~~~~~-~~~~a~~aall~~~~p~~~~~~~~~~~~~~~~~~   149 (426) |
|  |  |  | .+....|+.+.+++.+++..+|.++|+.. +++.+.+++++++++|......... ++|.+.. |
|  | T Consensus | 64 | ~~~~~~~~~~~~~~~~~~~~~~~~~~~~~~~~~~~~~~~~~~~~~~~~~~a~~~~~~~~~~p~~~~~~~~~--~~d~~~~   141 (247) |
|  | T PF02366.19 | 64 | YELNTLDEPGQQYPFTDVAYSMRLFTCLLGSLLVPLMYGTVYFPTKSKTAASLAALFVIFDNGLITMSRYI--MIEIPAL   141 (247) |
|  | T ss\_pred |  | cccccccCCCCCCCcchHHHHHHHHHHHHHHHHHHHHHHHHhcccCCHHHHHHHHHHHHhchhHHHhHhhc--cchHHHH |
|  |
|  |
|  | Q ss\_pred |  | HHHHHHHHHHHHc-------CCCCCcccHHHHHHHHHHHHHHHHhHhHHHHHHHHHHHHHHHHhcCCCccchhcccchhh |
|  | Q Q6ZXV5 | 150 | IFFLAAFLSYTRS-------KGPDNSIIWTPIALTVFLVAVATLCKEQGITVVGICCVYEVFIAQGYTLPLLCTTAGQFL   222 (426) |
|  | Q Consensus | 150 | ~~~~l~~~~~~~~-------~~~~~~~~~~~~~~~~~~~~la~~~k~~~~~~~~~~~~~~~~~~~~~~~~~~~~~~~~~~   222 (426) |
|  |  |  | ++.+++++++.+. .++++ ++....++++.+++.++|+.+....+...+.......+ + |
|  | T Consensus | 142 | ~~~~~~~~~~~~~~~~~~~~~~~~~---~~~~~~~~~~~~l~~~~k~~~~~~~~~~~~~~~~~~~~-------------~   205 (247) |
|  | T PF02366.19 | 142 | YFMSLTAFYWSVYEAQQKRPFSLRW---HTSLLSTGVALGLALSTKLSAMFTFGWLLILAAFHLWN-------------L   205 (247) |
|  | T ss\_pred |  | HHHHHHHHHHHHHHHhcCCCCCcHH---HHHHHHHHHHHHHHHHhhHHHHHHHHHHHHHHHHHHHH-------------h |
|  |
|  |
|  | Q ss\_pred |  | cCCCCCChHHHHHHHHHHHHHHHHHHHHHHHHHHHcc |
|  | Q Q6ZXV5 | 223 | RGKGSIPFSMLQTLVKLIVLMFSTLLLVVIRVQVIQS   259 (426) |
|  | Q Consensus | 223 | ~~~~~~~~~~~~~~~~~~~~~~~~~~~~~~~~~~~~~   259 (426) |
|  |  |  | ++++..+...+..........+...++........++ |
|  | T Consensus | 206 | ~~~~~~~~~~~~~~~~~~~~~~~~~p~~~~~~~~~~~   242 (247) |
|  | T PF02366.19 | 206 | LGDLSVPMYRIVKHLFSYIFYLIGVPITVYLAVFAVH   242 (247) |
|  | T ss\_pred |  | cCCCCCCHHHHHHHHHHHHHHHHHHHHHHHHHHHHHH |
|  |
| --- | | | |
|  | Template alignmentCDD | | |
| 8. | PF12250.9 ; AftA\_N ; Arabinofuranosyltransferase N terminal | | |
|  | Probability: 99.63%, E-value: 3.1e-13, Score: 117.43, Aligned cols: 322, Identities: 11%, Similarity: -0.04, | | |
|  |
|  | Q ss\_pred |  | HHHHHHHHHHHHHHHHhhCC-CceeccHHHHhcCCCCCCCCcHHHHhccccCCCCCCcchhcCCCCcchHHHHHHHHHHh |
|  | Q Q6ZXV5 | 7 | KEITLIVGVVTACYWNSLFC-GFVFDDVSAILDNKDLHPSTPLKTLFQNDFWGTPMSEERSHKSYRPLTVLTFRLNYLLS   85 (426) |
|  | Q Consensus | 7 | ~~~~~l~~~~~~~~~~~~~~-~~~~Dd~~~~~~~~~~~~~~~~~~~~~~~~~~~~~~~~~~~~~~~Pl~~~~~~~~~~lf   85 (426) |
|  |  |  | ...++...+...++...... +.+.||.++....+.+.++....++...+ ..+.|||+++++.+....++ |
|  | T Consensus | 76 | ~~~l~~~~l~~~L~~t~~~~~gl~~D~~~~~~~~~~~~~~~~~~d~~~~~----------~~~~YPPl~~~l~~~~~~l~   145 (432) |
|  | T PF12250.9 | 76 | PAALVITTLGIPLSATRLYLDGINVDQGFRTQFLTWMGYTIHLSDMNYID----------MPSYYPGAWFWIGGRLANLL   145 (432) |
|  | T ss\_pred |  | HHHHHHHHHHHHHhcCcccCCCccccHHHHHHHHHHHHhccccccccCCC----------CcccCChHHHHHHHHHHHHh |
|  |
|  |
|  | Q ss\_pred |  | CCCc-hHHHHHHHHHHHHHHHHHHHHHHHhcCC-HHH-HHHHHHHHHCcccHHHHHHhhccHHHHHHHHHHHHHHHHHHc |
|  | Q Q6ZXV5 | 86 | ELKP-MSYHLLNMIFHAVVSVIFLKVCKLFLDN-KSS-VIASLLFAVHPIHTEAVTGVVGRAELLSSIFFLAAFLSYTRS   162 (426) |
|  | Q Consensus | 86 | g~~~-~~~rl~~~l~~~~~~~~~y~l~~~~~~~-~~a-~~aall~~~~p~~~~~~~~~~~~~~~~~~~~~~l~~~~~~~~   162 (426) |
|  |  |  | |.++ ...|..+++...++....|.+.|++.++ +.+ .++++..+..|.. ......+.....+....++.+.|. |
|  | T Consensus | 146 | G~~~~~a~r~~~~l~~~l~~~~~y~l~r~l~~~~~~al~ia~~~~~~~~~~-----~~~~~y~~l~~~~l~~~l~~~~r~   220 (432) |
|  | T PF12250.9 | 146 | GLAGWEVFQPWALISLATAGSILVPVWQRICGSLTVASGIALVTTSITIVM-----SADEPYAAIITMGVPAATVMMRRA   220 (432) |
|  | T ss\_pred |  | CCCHHHHhHHHHHHHHHHHHHHHHHHHHHHHCCHHHHHHHHHHHHHHHHhc-----CCCCcHHHHHHHHHHHHHHHHHHH |
|  |
|  |
|  | Q ss\_pred |  | CCCCCcccHHHHHHHHHHHHHHHHhHhHHHHHHHHHHH-HHHHHhcCCCccchhcccchhhcCCCCCChHHHHHHHHHHH |
|  | Q Q6ZXV5 | 163 | KGPDNSIIWTPIALTVFLVAVATLCKEQGITVVGICCV-YEVFIAQGYTLPLLCTTAGQFLRGKGSIPFSMLQTLVKLIV   241 (426) |
|  | Q Consensus | 163 | ~~~~~~~~~~~~~~~~~~~~la~~~k~~~~~~~~~~~~-~~~~~~~~~~~~~~~~~~~~~~~~~~~~~~~~~~~~~~~~~   241 (426) |
|  |  |  | .++++ +.....+++.+++.++|............ ........ .++.++..++....... |
|  | T Consensus | 221 | l~~~~----~~~~~~gl~lgl~~l~y~~~~~~~~~~~~~~~~l~~~~----------------~~~~~~~~~~~~~~~~~   280 (432) |
|  | T PF12250.9 | 221 | LTGSL----WPLIGLTLYIGVSAAMYTLFTAVVALSVCVMAALFAVV----------------FDHSIKPLLRLLIIGTG   280 (432) |
|  | T ss\_pred |  | hcCCc----hHHHHHHHHHHHHHHHhHHHHHHHHHHHHHHHHHHHHH----------------cCCCchHHHHHHHHHHH |
|  |
|  |
|  | Q ss\_pred |  | HHHHHHHHHHHHHHHHccCCCccccCCCcccCCCchhhHHhHhhHHHHHHHHHhccccccccCccccCccccccccHHHH |
|  | Q Q6ZXV5 | 242 | LMFSTLLLVVIRVQVIQSQLPVFTRFDNPAAVSPTPTRQLTFNYLLPVNAWLLLNPSELCCDWTMGTIPLIESLLDIRNL   321 (426) |
|  | Q Consensus | 242 | ~~~~~~~~~~~~~~~~~~~~~~~~~~~~~~~~~~~~~~~~~~~~~~~~~~~~~~~~~~~~~~~~~~~~~~~~~~~~~~~~   321 (426) |
|  |  |  | ..+...+++..............+...+.... +...+...+... |
|  | T Consensus | 281 | a~~~a~~~~~P~l~~~~~~~~~~~~~~~~~~~------------------------------------~~~~~~~~p~~~   324 (432) |
|  | T PF12250.9 | 281 | SALIASTVWAPYLTAILSGQPHSGATAMHYLP------------------------------------PTGAQVPMPMLQ   324 (432) |
|  | T ss\_pred |  | HHHHHHHHHHHHHHHHHhCCCCCCcchHhcCC------------------------------------ccccCCCCcccc |
|  |
|  |
|  | Q ss\_pred |  | HHHHHHHHHHHHHHHHHHccCCCchhHHHHHHHHHHHHHHHhccCCCcchhhchhhchHHHHHHHHHHHHHHHHhccc |
|  | Q Q6ZXV5 | 322 | ATFTFFCFLGMLGVFSIRYSGDSSKTVLMALCLMALPFIPASNLFFPVGFVVAERVLYVPSMGFCILVAHGWQKISTK   399 (426) |
|  | Q Consensus | 322 | ~~~~~~~~~~~~~~~~~~~~~~~~~~~~~~~~~~~~~~~~~~~~~~~~~~~~~~ry~~~~~~~~~il~~~~~~~~~~~   399 (426) |
|  |  |  | ......+.+++.+....+++++..+...........-............+....|+..+..+.+++.++.++.++.++ |
|  | T Consensus | 325 | ~~~~~~L~l~Glv~l~~~~r~~~~~~l~~~~~~~y~w~~~~~~~~~~~~~ll~~R~~~~l~~~l~~~~a~gi~~l~~~   402 (432) |
|  | T PF12250.9 | 325 | FNLVGLLCLLGLAYLIVRIADPDVRSMLIAQIVFYGWIVTSMIVSLSGKTLLGFRLDAIITIQLATAGMLALAELRLV   402 (432) |
|  | T ss\_pred |  | ccHHHHHHHHHHHHHHHHhcCHhHHHHHHHHHHHHHHHHHHHHHHHhCCCchhHhHHHHHHHHHHHHHHHHHHHHHHc |
|  |
| --- | | | |
|  | Template alignmentCDD | | |
| 9. | PF10034.10 ; Dpy19 ; Q-cell neuroblast polarisation | | |
|  | Probability: 99.62%, E-value: 4.4e-13, Score: 124.64, Aligned cols: 344, Identities: 11%, Similarity: 0.001, | | |
|  |
|  | Q ss\_pred |  | CceeccHHHHhcCCCCCCCCcHHHHhcc--ccCCCCCCcchhcCCCCcchHHHH-HHHHHHhC----------------- |
|  | Q Q6ZXV5 | 27 | GFVFDDVSAILDNKDLHPSTPLKTLFQN--DFWGTPMSEERSHKSYRPLTVLTF-RLNYLLSE-----------------   86 (426) |
|  | Q Consensus | 27 | ~~~~Dd~~~~~~~~~~~~~~~~~~~~~~--~~~~~~~~~~~~~~~~~Pl~~~~~-~~~~~lfg-----------------   86 (426) |
|  |  |  | ++..||..|+..++++.+++++.+.... ......+..+.......|.+..+. +..++++| |
|  | T Consensus | 32 | ~~~~d~~~y~~~~~~i~~~~~~~~~~~~~~~d~~~~~p~g~~~~~~~~~~~~~~~a~~~~~~~~~~~~~~~~~~~~~~~~   111 (651) |
|  | T PF10034.10 | 32 | SFRTEMGLYYSYYKTMVEAPTFLDGLHAVMNCNVTEYPDTVNTLKRFNLYPEVILAGKFRIFEWLASKFEYQTKTCYTVN   111 (651) |
|  | T ss\_pred |  | ccCcchhhHHhHHhhhccCCCHHHHHHHHHcCCCCcCCCCchHHhhcchHHHHHHHHHHHHHHHHHhhcCcCccchheee |
|  |
|  |
|  | Q ss\_pred |  | ---------------CCchHHHHHHHHHHHHHHHHHHHHHHHhcCCH-HHHHHHHHHHHCcccHHHHHHhhccHHHHHHH |
|  | Q Q6ZXV5 | 87 | ---------------LKPMSYHLLNMIFHAVVSVIFLKVCKLFLDNK-SSVIASLLFAVHPIHTEAVTGVVGRAELLSSI   150 (426) |
|  | Q Consensus | 87 | ---------------~~~~~~rl~~~l~~~~~~~~~y~l~~~~~~~~-~a~~aall~~~~p~~~~~~~~~~~~~~~~~~~   150 (426) |
|  |  |  | ...........++++++++.+|.++|++++++ .|++++++++++|.++..........|.+..+ |
|  | T Consensus | 112 | ~~~~~~~~~~~~~~~~p~~~~~~~~~i~~~l~v~~~y~l~~~l~~~~~~al~aall~a~~p~~~~~~~~g~~~~~~~~~~   191 (651) |
|  | T PF10034.10 | 112 | RGYGLPPVQSCEGLGELSFFYVYSIFFLTGLMMACFFILCFYLSGSILGGVLGTLCYFFNHGEATRVMWTPPLRESFSYP   191 (651) |
|  | T ss\_pred |  | cCCCCCCcccCCCcCchHHHHHHHHHHHHHHHHHHHHHHHHHHcCChHHHHHHHHHHHhChhhhhhHhhCCCccccCHHH |
|  |
|  |
|  | Q ss\_pred |  | HHHHHHHHHHHcCCCCCcccH--HHHHHHHHHHHHHHHhHhHHHHHHHHHHHHHHHHhcCCCccchhcccchhhcCCCCC |
|  | Q Q6ZXV5 | 151 | FFLAAFLSYTRSKGPDNSIIW--TPIALTVFLVAVATLCKEQGITVVGICCVYEVFIAQGYTLPLLCTTAGQFLRGKGSI   228 (426) |
|  | Q Consensus | 151 | ~~~l~~~~~~~~~~~~~~~~~--~~~~~~~~~~~la~~~k~~~~~~~~~~~~~~~~~~~~~~~~~~~~~~~~~~~~~~~~   228 (426) |
|  |  |  | +.+++++++.+..++++ . ++.++++++.+++..+|...........+..+..... ++.. |
|  | T Consensus | 192 | f~~l~l~~~~~~~~~~~---~~~~~~~l~gl~~~l~~~~~~~~~~~~~~~~~~~~~~~~~----------------~~~~   252 (651) |
|  | T PF10034.10 | 192 | YLVAQLLVVTFTLRSVK---VTWRHITLVSMTTALFMIPWQFAQFALLTQTCALFVVYIM----------------HFIT   252 (651) |
|  | T ss\_pred |  | HHHHHHHHHHHHHhCCC---CCHHHHHHHHHHHHHHHHHhhhHHHHHHHHHHHHHHHHHh----------------hcCC |
|  |
|  |
|  | Q ss\_pred |  | ChHHHHHHHHHHHHHHHHHHHHHHHHHHH--------------------------------------------------- |
|  | Q Q6ZXV5 | 229 | PFSMLQTLVKLIVLMFSTLLLVVIRVQVI---------------------------------------------------   257 (426) |
|  | Q Consensus | 229 | ~~~~~~~~~~~~~~~~~~~~~~~~~~~~~---------------------------------------------------   257 (426) |
|  |  |  | ++..+......+...++............ |
|  | T Consensus | 253 | ~~~~~~~~~~~~~~~~~~~~~~~~~~~~~~~~~~~~~~~~~~~~~~~~~~~~~~~~~~~~~~~~~~~~~~~~~~~~~~~~   332 (651) |
|  | T PF10034.10 | 253 | ADKFCKILYGLLVAHLLNFAVQFGNSMLLSSFFMSAVISALVVAKAESQIHKLPYQLLIWATQGLGFAAGTLGIKVAVAK   332 (651) |
|  | T ss\_pred |  | HHHHHHHHHHHHHHHHHHHHHHccchHHHHHHHHHHHHHHHHHHHHHhhhccCCHHHHHHHHHHHHHHHHHHHHHHHHHH |
|  |
|  |
|  | Q ss\_pred |  | ----------ccCCCccccCCCcccCCCchhhHHhHhhHHHHHHHHHhccccccccCccccCccccccccHHHHHHHHHH |
|  | Q Q6ZXV5 | 258 | ----------QSQLPVFTRFDNPAAVSPTPTRQLTFNYLLPVNAWLLLNPSELCCDWTMGTIPLIESLLDIRNLATFTFF   327 (426) |
|  | Q Consensus | 258 | ----------~~~~~~~~~~~~~~~~~~~~~~~~~~~~~~~~~~~~~~~~~~~~~~~~~~~~~~~~~~~~~~~~~~~~~~   327 (426) |
|  |  |  | .......................... ........+......... |
|  | T Consensus | 333 | ~~~~~~~~~~~~~~~~~~~~~~~~~~~~~~~~~~~~--------------------------~~~~~~~~~~~~~~~~~~   386 (651) |
|  | T PF10034.10 | 333 | VLSIADDSKFTSYRDFHTLLYTCAPEFDFLDQEAPV--------------------------KLTKTLLLPSAIVAASAV   386 (651) |
|  | T ss\_pred |  | hcCcccccccCCCCCHHHHHHhcchhcCCCChHHHH--------------------------HHHHhcHHHHHHHHHHHH |
|  |
|  |
|  | Q ss\_pred |  | HHHHHHHHHHHHccCCCchh------------------------HHHHHHHHHHHHHHHhccCCCcchhhchhhchHHHH |
|  | Q Q6ZXV5 | 328 | CFLGMLGVFSIRYSGDSSKT------------------------VLMALCLMALPFIPASNLFFPVGFVVAERVLYVPSM   383 (426) |
|  | Q Consensus | 328 | ~~~~~~~~~~~~~~~~~~~~------------------------~~~~~~~~~~~~~~~~~~~~~~~~~~~~ry~~~~~~   383 (426) |
|  |  |  | ............|+++..+. ..+.+++.+..... .....||..+..| |
|  | T Consensus | 387 | ~~~~~~~~~~~~~~~~~~~~~~~~~~~~~~~~~~~~~~~~~~~~~~~~~~~~~~~~~~---------~~~~~R~~~~~~p   457 (651) |
|  | T PF10034.10 | 387 | IAKVGASEWEYWVRGKKSQVKSDSADEHDEGAQGQEANPRPHAEYVYHVLQAMAFVLM---------AVIIMRLKLFGTP   457 (651) |
|  | T ss\_pred |  | HHHHHHHHHHHHHhcccccCCCCCccccccccccccCCCCCchHHHHHHHHHHHHHHH---------HHHHHHHHHHHHH |
|  |
|  |
|  | Q ss\_pred |  | HHHHHHHHHHHH-hcccchHHHHHHHHHHHHHHHHHHHhhhh |
|  | Q Q6ZXV5 | 384 | GFCILVAHGWQK-ISTKSVFKKLSWICLSMVILTHSLKTFHR   424 (426) |
|  | Q Consensus | 384 | ~~~il~~~~~~~-~~~~~~~~~~~~~~~~~~~~~~~~~~~~~   424 (426) |
|  |  |  | +++++++.++.+ +.++.+.++....+.+++++......... |
|  | T Consensus | 458 | ~l~il~a~~~~~~~~~~~~~~~~~~~~~~~~~~~~~~~~~~~   499 (651) |
|  | T PF10034.10 | 458 | ALCVLASLVASRQFFSFLGDRRRHQAIVIALIAVMSVQGFSN   499 (651) |
|  | T ss\_pred |  | HHHHHHHHHhcHHHHHhhhHHHHHHHHHHHHHHHHHhcchhh |
|  |
| --- | | | |
|  | Template alignmentCDD | | |
| 10. | PF09913.10 ; DUF2142 ; Predicted membrane protein (DUF2142) | | |
|  | Probability: 99.54%, E-value: 5.4e-13, Score: 116.06, Aligned cols: 328, Identities: 11%, Similarity: -0.026, | | |
|  |
|  | Q ss\_pred |  | HHHHHhhCCCceeccHHHHhcCCCCCCCCcHHHHhcccc----------------------------------------- |
|  | Q Q6ZXV5 | 18 | ACYWNSLFCGFVFDDVSAILDNKDLHPSTPLKTLFQNDF-----------------------------------------   56 (426) |
|  | Q Consensus | 18 | ~~~~~~~~~~~~~Dd~~~~~~~~~~~~~~~~~~~~~~~~-----------------------------------------   56 (426) |
|  |  |  | ..+..........||..|...+....+++......+... |
|  | T Consensus | 4 | ~~~~~~~P~~~~pDE~~H~~~a~~ia~g~~~~~~~~~~~~~~~~~~~~~~~~~~~~~~~~~~~~~~~~~~~~~~~~~~~~   83 (405) |
|  | T PF09913.10 | 4 | LAFAVVMPPFQVPDEDGHFIRAYLISRGEFVGRGAPRVPGTVVLSMMRYPEMGERFGRFKPRELVRDLIPHPGSVSPEVP   83 (405) |
|  | T ss\_pred |  | eehhhccCCCCCCChHHHHHHHHHHHcCceeeecCCCCCCcccchhccCcccccccCCCCchHHHhccCCCCCCCCCCcc |
|  |
|  |
|  | Q ss\_pred |  | -----CCCCCCcchhcCC-----CCcchHHHHHHHH---HHhCCC----chHHHHHHHHHHHHHHHHHHHHHHHhcCCHH |
|  | Q Q6ZXV5 | 57 | -----WGTPMSEERSHKS-----YRPLTVLTFRLNY---LLSELK----PMSYHLLNMIFHAVVSVIFLKVCKLFLDNKS   119 (426) |
|  | Q Consensus | 57 | -----~~~~~~~~~~~~~-----~~Pl~~~~~~~~~---~lfg~~----~~~~rl~~~l~~~~~~~~~y~l~~~~~~~~~   119 (426) |
|  |  |  | ......++..... +||++++..+... +++|.+ ....|+.+++++++++.++|+++++.. |
|  | T Consensus | 84 | ~~~~~~~~~~~~~~~~~~~~~~~~pPl~y~~~a~~~~l~~~~~~~~~~~~~~~Rl~s~l~~~~~~~~~~~~~~~~~----   159 (405) |
|  | T PF09913.10 | 84 | SLNLGNLDVRHRWLPWSIIGSSLYCPLVYMPASLGIATVRILSGSPLLMMYGARLFNVIVFAAALAISFRLAPRYR----   159 (405) |
|  | T ss\_pred |  | ccccccCCCcccccCccccCccccCHHhHHHHHHHHHHHHHccCCHHHHHHHHHHHHHHHHHHHHHHHHHHChhhH---- |
|  |
|  |
|  | Q ss\_pred |  | HHHHHHHHHHCcccHHHHHHhhccHHHHHHHHHHHHHHHHHHcCCCC-CcccHHHHHHHHHHHHHHHHhHhHHHHHHHHH |
|  | Q Q6ZXV5 | 120 | SVIASLLFAVHPIHTEAVTGVVGRAELLSSIFFLAAFLSYTRSKGPD-NSIIWTPIALTVFLVAVATLCKEQGITVVGIC   198 (426) |
|  | Q Consensus | 120 | a~~aall~~~~p~~~~~~~~~~~~~~~~~~~~~~l~~~~~~~~~~~~-~~~~~~~~~~~~~~~~la~~~k~~~~~~~~~~   198 (426) |
|  |  |  | .++++++++|..+...... ++|.+..++.+++++++.+..+++ + ++.++..+++.+++.++| ..++.+. |
|  | T Consensus | 160 | --~~~~~~a~~P~~~~~~~~~--~~D~~~~~~~~~~~~~~~~~~~~~~~---~~~~~~~~~~~~l~~~~K---~~~~~~~   229 (405) |
|  | T PF09913.10 | 160 | --ALFTAVALMPMTLQQAGGI--SADLVTIAFSFVGFSLVLHSREHFVS---RRLLILIVLVFVMWVLCK---SSIWALP   229 (405) |
|  | T ss\_pred |  | --HHHHHHHhchHHHHHHhcC--ChHHHHHHHHHHHHHHHHhcCCcCCC---HHHHHHHHHHHHHHHHHH---HHHHHHH |
|  |
|  |
|  | Q ss\_pred |  | HHHHHHHhcCCCccchhcccchhhcCCCCCChHHHHHHHHHHHHHHHHHHHHHHHHHHHccCCCccccCCCcccCCCchh |
|  | Q Q6ZXV5 | 199 | CVYEVFIAQGYTLPLLCTTAGQFLRGKGSIPFSMLQTLVKLIVLMFSTLLLVVIRVQVIQSQLPVFTRFDNPAAVSPTPT   278 (426) |
|  | Q Consensus | 199 | ~~~~~~~~~~~~~~~~~~~~~~~~~~~~~~~~~~~~~~~~~~~~~~~~~~~~~~~~~~~~~~~~~~~~~~~~~~~~~~~~   278 (426) |
|  |  |  | ....+..+++ .+++.++.....+..++....+............................. |
|  | T Consensus | 230 | l~~~~~~~~~-------------------~~~~~~~~~~~~~~~~~~~~~~~~~~~~~~~~~~~~~~~~~~~~~~~~~~~   290 (405) |
|  | T PF09913.10 | 230 | LLLLIPVSAF-------------------KNRLTWAAYLGVASVCMVGALLVWNNVTAPNLETFRAVRLTHGVDMPANIR   290 (405) |
|  | T ss\_pred |  | HHHHHhHHhh-------------------CchHHHHHHHHHHHHHHHHHHHHHHhhcCCCccchhhccccCCCChHHHHH |
|  |
|  |
|  | Q ss\_pred |  | hHHhHhhHHHHHHHHHhccccccccCccccCccccccccHHHHHHHHHHHHHHHHHHHHHHccCCCchhHHHHHHHHHHH |
|  | Q Q6ZXV5 | 279 | RQLTFNYLLPVNAWLLLNPSELCCDWTMGTIPLIESLLDIRNLATFTFFCFLGMLGVFSIRYSGDSSKTVLMALCLMALP   358 (426) |
|  | Q Consensus | 279 | ~~~~~~~~~~~~~~~~~~~~~~~~~~~~~~~~~~~~~~~~~~~~~~~~~~~~~~~~~~~~~~~~~~~~~~~~~~~~~~~~   358 (426) |
|  |  |  | ...+...................+...............+.........+++........+++++.+.........+... |
|  | T Consensus | 291 | ~~~~~p~~~~~~~~~~~~~~~~~~~~~~~g~~g~~~~~lp~~~~~~~~~~l~~~~~~~~~~~~~~~~~~~~~~~~~~~~~   370 (405) |
|  | T PF09913.10 | 291 | LVGAHPLMFVRYLIGVVGSNLKPEIGQFIGAFGWLRFPLPSWVRAAYLLLVLVTAVTEFPAKSFRTWERGVLALVLLGGV   370 (405) |
|  | T ss\_pred |  | HHHhCHHHHHHHHHHHHHHhhHHHHHHHHHHhhccCCCccHHHHHHHHHHHHHHHHccCCccCCCHHHHHHHHHHHHHHH |
|  |
|  |
|  | Q ss\_pred |  | HHHHhccCCCcchh---------------hchhhc |
|  | Q Q6ZXV5 | 359 | FIPASNLFFPVGFV---------------VAERVL   378 (426) |
|  | Q Consensus | 359 | ~~~~~~~~~~~~~~---------------~~~ry~   378 (426) |
|  |  |  | .+............ .+.||+ |
|  | T Consensus | 371 | ~~i~~~~~~~~~~~~~~~i~G~~~~~~~~~QgRY~   405 (405) |
|  | T PF09913.10 | 371 | LFVHAAMCISDTTLCSGTLNSGCRDESIVFQGRYL   405 (405) |
|  | T ss\_pred |  | HHHHHHHHHhccCCCcceeccCCCCccceeceecC |
|  |
| --- | | | |
|  | Template alignmentCDD | | |
| 11. | PF13231.7 ; PMT\_2 ; Dolichyl-phosphate-mannose-protein mannosyltransferase | | |
|  | Probability: 99.54%, E-value: 6.2e-13, Score: 99.51, Aligned cols: 156, Identities: 13%, Similarity: 0.181, | | |
|  |
|  | Q ss\_pred |  | CCCcchHHHHHHHHHHhCCCchHHHHHHHHHHHHHHHHHHHHHHHhcCCHHHHHHHHHHHHCcccHHHHHHhhccHHHHH |
|  | Q Q6ZXV5 | 69 | SYRPLTVLTFRLNYLLSELKPMSYHLLNMIFHAVVSVIFLKVCKLFLDNKSSVIASLLFAVHPIHTEAVTGVVGRAELLS   148 (426) |
|  | Q Consensus | 69 | ~~~Pl~~~~~~~~~~lfg~~~~~~rl~~~l~~~~~~~~~y~l~~~~~~~~~a~~aall~~~~p~~~~~~~~~~~~~~~~~   148 (426) |
|  |  |  | +|||+++++.+....++|+++...|+.+.+++.++...+|.+.|+..+++.+..++.+++++|.. .......++|... |
|  | T Consensus | 1 | ~~~P~~~~~~~~~~~l~g~~~~~~~~~~~~~~~~~~~~~~~~~~~~~~~~~~~~~~~~~~~~p~~--~~~~~~~~~~~~~   78 (159) |
|  | T PF13231.7 | 1 | DKPPASLWVMELSTRIFGVNSWAMLVPQALLGVAAVALLYATVRRRFGAVAGLLAGLILAVTPVA--AMMFRFNNPDALL   78 (159) |
|  | T ss\_pred |  | CCChHHHHHHHHHHHHHCCCHHHHHHHHHHHHHHHHHHHHHHHHHHHHHHHHHHHHHHHHhcHHH--HHhhhcCCHHHHH |
|  |
|  |
|  | Q ss\_pred |  | HHHHHHHHHHHHHcCCCCCcccHHHHHHHHHHHHHHHHhHhHHHHHHHHHHHHHHHHhcCCCccchhcccchhhcCCCCC |
|  | Q Q6ZXV5 | 149 | SIFFLAAFLSYTRSKGPDNSIIWTPIALTVFLVAVATLCKEQGITVVGICCVYEVFIAQGYTLPLLCTTAGQFLRGKGSI   228 (426) |
|  | Q Consensus | 149 | ~~~~~l~~~~~~~~~~~~~~~~~~~~~~~~~~~~la~~~k~~~~~~~~~~~~~~~~~~~~~~~~~~~~~~~~~~~~~~~~   228 (426) |
|  |  |  | .++..++++...+..++++ .+....++++.+++..+|+......+..........++ |
|  | T Consensus | 79 | ~~~~~~~~~~~~~~~~~~~---~~~~~~~~~~~~l~~~~k~~~~~~~~~~~~~~~~~~~~--------------------   135 (159) |
|  | T PF13231.7 | 79 | VLLMIAATWAMLRAVEDGR---WRWLIVCGAFVGVGFLTKQLAVMLIVPGLALTYLVAGP--------------------   135 (159) |
|  | T ss\_pred |  | HHHHHHHHHHHHHHHHcCC---hHHHHHHHHHHHHHHHcccchHHhhHHHHHHHHHHhCC-------------------- |
|  |
|  |
|  | Q ss\_pred |  | ChHHHHHHHHHHHHHHHHHHHH |
|  | Q Q6ZXV5 | 229 | PFSMLQTLVKLIVLMFSTLLLV   250 (426) |
|  | Q Consensus | 229 | ~~~~~~~~~~~~~~~~~~~~~~   250 (426) |
|  |  |  | ++.++.........+...... |
|  | T Consensus | 136 | -~~~~~~~~~~~~~~~~~~~~~   156 (159) |
|  | T PF13231.7 | 136 | -PKIGVRIAQLFAAGTSMIVAA   156 (159) |
|  | T ss\_pred |  | -hhHHHHHHHHHHHHHHHHHHH |
|  |
| --- | | | |
|  | Template alignmentCDD | | |
| 12. | PF04602.13 ; Arabinose\_trans ; Mycobacterial cell wall arabinan synthesis protein | | |
|  | Probability: 99.48%, E-value: 1.1e-10, Score: 100.55, Aligned cols: 350, Identities: 9%, Similarity: -0.025, | | |
|  |
|  | Q ss\_pred |  | HHHHHHHHHHhhCCCceeccHHHHhcCCCCCCCCcHHHHhccccCCCCCCcchhcCCCCcchHHHHHHHHHH--hCCCch |
|  | Q Q6ZXV5 | 13 | VGVVTACYWNSLFCGFVFDDVSAILDNKDLHPSTPLKTLFQNDFWGTPMSEERSHKSYRPLTVLTFRLNYLL--SELKPM   90 (426) |
|  | Q Consensus | 13 | ~~~~~~~~~~~~~~~~~~Dd~~~~~~~~~~~~~~~~~~~~~~~~~~~~~~~~~~~~~~~Pl~~~~~~~~~~l--fg~~~~   90 (426) |
|  |  |  | .++...+....+..+...||.+|...+++..+++...+.+.. +...++|...+ ..+.... +|.+.. |
|  | T Consensus | 51 | ~~v~~~l~~w~~~gp~~~DEG~Yl~~ar~~~~~G~~~npy~~-----------~~~~~~Pfg~~-~~l~~~~~~~g~s~~   118 (471) |
|  | T PF04602.13 | 51 | LIVGAILLGWYFIGANTADDGYILNMARVAGHAGYMANYYRW-----------YGVPEAPFGWF-YDVTAALAALSTASP   118 (471) |
|  | T ss\_pred |  | HHHHHHHHHHHHhccCCCChHHHHHHHHHHHhcCCccccchh-----------cCCCCCCcHHH-HHHHHHHHHhcCChH |
|  |
|  |
|  | Q ss\_pred |  | HHHHHHHHHHHHHHHHHHHHHHHhcC-----CHHHHHHHHHHHHCcccHHHHHHhhccHHHHHHHHHHHHHHHHHHcCCC |
|  | Q Q6ZXV5 | 91 | SYHLLNMIFHAVVSVIFLKVCKLFLD-----NKSSVIASLLFAVHPIHTEAVTGVVGRAELLSSIFFLAAFLSYTRSKGP   165 (426) |
|  | Q Consensus | 91 | ~~rl~~~l~~~~~~~~~y~l~~~~~~-----~~~a~~aall~~~~p~~~~~~~~~~~~~~~~~~~~~~l~~~~~~~~~~~   165 (426) |
|  |  |  | ..|++++++++++..+++....+..+ ++.+..++.+..+....... ...+.|....++.++++++..|..++ |
|  | T Consensus | 119 | ~lRl~~ll~~l~~w~lL~~~vl~rl~~~~~~~~~a~~~aal~~la~wlp~~---~~lr~Ep~~al~~~~~l~l~~ra~~~   195 (471) |
|  | T PF04602.13 | 119 | FVRLTTLIASILCWWIISREVIPRLGRRARHTPAVYWTAAAVFLAFWLPYN---NGLRPEPVIAVGALLTWISVERAIAT   195 (471) |
|  | T ss\_pred |  | HHHHHHHHHHHHHHHHHHHHHHHHhccccCCcHHHHHHHHHHHHHHHcccc---CCCCcHHHHHHHHHHHHHHHHHHHhc |
|  |
|  |
|  | Q ss\_pred |  | CCcccHHHHHHHHHHHHHHHHhHhHHHHHHHHHHHHHHHHhcCCCccchhcccchhhcCCCCCChHHHHHHHHH------ |
|  | Q Q6ZXV5 | 166 | DNSIIWTPIALTVFLVAVATLCKEQGITVVGICCVYEVFIAQGYTLPLLCTTAGQFLRGKGSIPFSMLQTLVKL------   239 (426) |
|  | Q Consensus | 166 | ~~~~~~~~~~~~~~~~~la~~~k~~~~~~~~~~~~~~~~~~~~~~~~~~~~~~~~~~~~~~~~~~~~~~~~~~~------   239 (426) |
|  |  |  | ++ .....+++++.++++.+|+++......+.+......++ .++..... |
|  | T Consensus | 196 | ~~---~~~~alag~~~gla~~aKPtg~~~la~ll~~~~~~~r~-----------------------~~~r~~~~~~~~~~   249 (471) |
|  | T PF04602.13 | 196 | GR---LLPAAIATIIAAFSLAAGPTGLMAVAALLAGSRPLLAI-----------------------LIKRAKQLTPNTTT   249 (471) |
|  | T ss\_pred |  | CC---cHHHHHHHHHHHHHHhcCHhHHHHHHHHHHHHHHHHHH-----------------------HHHHHHhcCCCCCC |
|  |
|  |
|  | Q ss\_pred |  | -----------HHHHHHHHHHHHHHHHHHccCCCccccCCCcccCCCchhhHHhHhhHHHHHHHHHhccccccccCcccc |
|  | Q Q6ZXV5 | 240 | -----------IVLMFSTLLLVVIRVQVIQSQLPVFTRFDNPAAVSPTPTRQLTFNYLLPVNAWLLLNPSELCCDWTMGT   308 (426) |
|  | Q Consensus | 240 | -----------~~~~~~~~~~~~~~~~~~~~~~~~~~~~~~~~~~~~~~~~~~~~~~~~~~~~~~~~~~~~~~~~~~~~~   308 (426) |
|  |  |  | ....+...........+..+......+...............+....+...+ |
|  | T Consensus | 250 | ~~~~~~~~~~~~la~~~a~~~~~l~~~f~d~sl~~~~~~~~~~~~~~~~~~~~~e~~Ry~~l~-----------------   312 (471) |
|  | T PF04602.13 | 250 | GNKHTPLASGRPHRPLLAAGTAVLFIIFYDQTLAAVSEASRLRTIIGPSNSWYNEFFRYSELF-----------------   312 (471) |
|  | T ss\_pred |  | CCCCCCCCCCccHHHHHHHHHHHHHHHHhcCcHHHHHHHHHHHhhcCCCCHHhhhHHHHHHHh----------------- |
|  |
|  |
|  | Q ss\_pred |  | CccccccccHHHHHHHHHHHHHHHHHHHHHHccCCCc---hhHHHHHHHHHHHHHHHhccCCCcchhhchhhchHHHHHH |
|  | Q Q6ZXV5 | 309 | IPLIESLLDIRNLATFTFFCFLGMLGVFSIRYSGDSS---KTVLMALCLMALPFIPASNLFFPVGFVVAERVLYVPSMGF   385 (426) |
|  | Q Consensus | 309 | ~~~~~~~~~~~~~~~~~~~~~~~~~~~~~~~~~~~~~---~~~~~~~~~~~~~~~~~~~~~~~~~~~~~~ry~~~~~~~~   385 (426) |
|  |  |  | .....-+...-......+..+........++++... ....-.+.+....+...... +.+..+|+....+.. |
|  | T Consensus | 313 | -~~~~~g~~~rr~~vll~~~~l~~~~~~l~r~~r~~~~~~~p~~rl~~~~~~~~~~l~~t-----ptKwthhfg~~a~~~   386 (471) |
|  | T PF04602.13 | 313 | -SQTADGSIARRFPVLIMIVCIFTAAAAIIHSASKSKLAKGPTLRLLAVSIMSFGFLAAT-----PTKWVHHFGAFAGIG   386 (471) |
|  | T ss\_pred |  | -cCCCCCcHHHHHHHHHHHHHHHHHHHHHHHHhccccccCChHHHHHHHHHHHHHHHHhC-----CchhhhhHHHHHHHH |
|  |
|  |
|  | Q ss\_pred |  | HHHHHHHHHHhcc---cchHHHHHHHHHHHHHHHHHHHhhhhcC |
|  | Q Q6ZXV5 | 386 | CILVAHGWQKIST---KSVFKKLSWICLSMVILTHSLKTFHRNW   426 (426) |
|  | Q Consensus | 386 | ~il~~~~~~~~~~---~~~~~~~~~~~~~~~~~~~~~~~~~~~~   426 (426) |
|  |  |  | ..+++.......+ +.+..+......+..+...+...-+.-+ |
|  | T Consensus | 387 | ~~~~a~~~~~~~~~~~r~~~~~~~~~~~~~~~~al~~~g~N~W~   430 (471) |
|  | T PF04602.13 | 387 | AAIAALAAVALTTPLFQSPRNRVLFTGIVVIIAAYAATGPNAYW   430 (471) |
|  | T ss\_pred |  | HHHHHHHHHHhcchhcCCHHHHHHHHHHHHHHHHHHHhCccccc |
|  |
| --- | | | |
|  | Template alignmentCDD | | |
| 13. | PF11028.9 ; DUF2723 ; Protein of unknown function (DUF2723) | | |
|  | Probability: 99.47%, E-value: 7.1e-13, Score: 102.03, Aligned cols: 152, Identities: 18%, Similarity: 0.149, | | |
|  |
|  | Q ss\_pred |  | ccHHHHhcCCCCCCCCcHHHHhccccCCCCCCcchhcCCCCcchHHHHHHHHHHh--CCCchHHHHHHHHHHHHHHHHHH |
|  | Q Q6ZXV5 | 31 | DDVSAILDNKDLHPSTPLKTLFQNDFWGTPMSEERSHKSYRPLTVLTFRLNYLLS--ELKPMSYHLLNMIFHAVVSVIFL   108 (426) |
|  | Q Consensus | 31 | Dd~~~~~~~~~~~~~~~~~~~~~~~~~~~~~~~~~~~~~~~Pl~~~~~~~~~~lf--g~~~~~~rl~~~l~~~~~~~~~y   108 (426) |
|  |  |  | ||..+...+.+.....+. ..|++..+......++ |.++...|+.+.+++.+++.++| |
|  | T Consensus | 1 | D~~~y~~~a~~~~~~~~p---------------------~~~l~~~~~~~~~~~~~~~~~~~~~r~~~~l~~~l~~~~~~   59 (188) |
|  | T PF11028.9 | 1 | DCGEYITAANKLEVGHPP---------------------GAPLFMLLGRLFSFFAEPEMVAVWINRLSALCSSFTILFLY   59 (188) |
|  | T ss\_pred |  | ChHHHHHHHhhcCCCCCC---------------------ChHHHHHHHHHHHhcCCCCcHHHHHHHHHHHHHHHHHHHHH |
|  |
|  |
|  | Q ss\_pred |  | HHHHHhcCCH------------------HHHHHHHHHHHCcccHHHHHHhhccHHHHHHHHHHHHHHHHHHcCCCCCccc |
|  | Q Q6ZXV5 | 109 | KVCKLFLDNK------------------SSVIASLLFAVHPIHTEAVTGVVGRAELLSSIFFLAAFLSYTRSKGPDNSII   170 (426) |
|  | Q Consensus | 109 | ~l~~~~~~~~------------------~a~~aall~~~~p~~~~~~~~~~~~~~~~~~~~~~l~~~~~~~~~~~~~~~~   170 (426) |
|  |  |  | .+.|+..+++ .+++++++++++|.. .......++|.+..++.+++++++.+..++++ |
|  | T Consensus | 60 | ~~~~~~~~~~~~~~~~~~~~~~~~~~~~~a~~a~~l~~~~p~~--~~~s~~~~~d~~~~~~~~~~l~~~~~~~~~~~---   134 (188) |
|  | T PF11028.9 | 60 | WSITMFAKKIMQRKDRDWSRGDQIATLGAGIIGALAYTFSDSF--WFSAVEGEVYAMSSLFTAAIFWMILKWDAEMI---   134 (188) |
|  | T ss\_pred |  | HHHHHHHHHHHhccccccccchHHHHHHHHHHHHHHHHHchhH--HHHhhhcchhHHHHHHHHHHHHHHHHHHHhcc--- |
|  |
|  |
|  | Q ss\_pred |  | H-------------HHHHHHHHHHHHHHHhHhHHHHHHHHHHHHHHHHhcC |
|  | Q Q6ZXV5 | 171 | W-------------TPIALTVFLVAVATLCKEQGITVVGICCVYEVFIAQG   208 (426) |
|  | Q Consensus | 171 | ~-------------~~~~~~~~~~~la~~~k~~~~~~~~~~~~~~~~~~~~   208 (426) |
|  |  |  | . ++..+++++.+++.++|+.++.+.+...++.+...++ |
|  | T Consensus | 135 | ~~~~~~~~~~~~~~~~~~l~g~~~~la~~~k~~~~~~~~~~~~~~~~~~~~   185 (188) |
|  | T PF11028.9 | 135 | GIKHGEIKDSRSPMRWMILIWFMFGLAIGVHLLGLLAVPAIAYVIYFNLWE   185 (188) |
|  | T ss\_pred |  | CccCCCcCCCCChHHHHHHHHHHHHHHHHHHHHHHHHHHHHHHHHHHhhHH |
|  |
| --- | | | |
|  | Template alignmentCDD | | |
| 14. | PF06728.14 ; PIG-U ; GPI transamidase subunit PIG-U | | |
|  | Probability: 99.42%, E-value: 2.1e-10, Score: 98.25, Aligned cols: 333, Identities: 8%, Similarity: -0.072, | | |
|  |
|  | Q ss\_pred |  | HHHHHHHHHh---hCCCce-eccHHHHhcCCCCCCCCcHHHHhccccCCCCCCcchhcCCCCcchHHHHHHHHHHhCCCc |
|  | Q Q6ZXV5 | 14 | GVVTACYWNS---LFCGFV-FDDVSAILDNKDLHPSTPLKTLFQNDFWGTPMSEERSHKSYRPLTVLTFRLNYLLSELKP   89 (426) |
|  | Q Consensus | 14 | ~~~~~~~~~~---~~~~~~-~Dd~~~~~~~~~~~~~~~~~~~~~~~~~~~~~~~~~~~~~~~Pl~~~~~~~~~~lfg~~~   89 (426) |
|  |  |  | +++++++... ...+.. .+.........+..+...+.+..+....+.+..++.. ..+||+..++..... ++.++ |
|  | T Consensus | 1 | ~~~~~irl~~~~~~~~~~~~~~~~~~~~~~~~~~d~~~~~~~~~~~~~G~~py~~~~-~~ypP~~~~l~~~~~--~~~~~   77 (363) |
|  | T PF06728.14 | 1 | LGLLSISFFLQWYLANTWIAEFLYRRIEVSTPVSGFLRVREGLYLYENGLDPYSGGV-FYQSPLLLILNYCCE--LLGGI   77 (363) |
|  | T ss\_pred |  | CHHHHHHHHHHHHHhcchhHHHHHhcccccCCcchHHHHHHHHHHHHcCCCCCCCCc-ccCCcchHHHHhhhh--hcCch |
|  |
|  |
|  | Q ss\_pred |  | hHHHHHHHHHHHHHHHHHHHHHHHhcCC--------HHHHHHHHHHHHCcccHHHHHHhhccHHHHHHHHHHHHHHHHHH |
|  | Q Q6ZXV5 | 90 | MSYHLLNMIFHAVVSVIFLKVCKLFLDN--------KSSVIASLLFAVHPIHTEAVTGVVGRAELLSSIFFLAAFLSYTR   161 (426) |
|  | Q Consensus | 90 | ~~~rl~~~l~~~~~~~~~y~l~~~~~~~--------~~a~~aall~~~~p~~~~~~~~~~~~~~~~~~~~~~l~~~~~~~   161 (426) |
|  |  |  | ...|+++.++.++++.++|.++|+..++ +.+..++++++++|.. ......++.|....++.+++++++.| |
|  | T Consensus | 78 | ~~~r~~~~~~~~~~~~l~~~~~~~~~~~~~~~~~~~~~~~~~~~~~~~~p~~--~~~~~~~~~d~~~~~~~~~al~~~~~   155 (363) |
|  | T PF06728.14 | 78 | SVTRFVYTSISTMGGLFVYLIAKQARVLDPNQVLSTCSPLWISVIYLLNPLT--FLPGIACSADMILNFTTLMTIYFASC   155 (363) |
|  | T ss\_pred |  | HHHHHHHHHHHHHHHHHHHHHHHHhhhcCcccccccCCcHHHHHHHHhCHHH--HHHHHhcchHHHHHHHHHHHHHHHhC |
|  |
|  |
|  | Q ss\_pred |  | cCCCCCcccHHHHHHHHHHHHHHHHhHhHHHHHHHHHHHHHHHHhcCCCccchhcccchhhcCCCCCChHHHHHHHHHHH |
|  | Q Q6ZXV5 | 162 | SKGPDNSIIWTPIALTVFLVAVATLCKEQGITVVGICCVYEVFIAQGYTLPLLCTTAGQFLRGKGSIPFSMLQTLVKLIV   241 (426) |
|  | Q Consensus | 162 | ~~~~~~~~~~~~~~~~~~~~~la~~~k~~~~~~~~~~~~~~~~~~~~~~~~~~~~~~~~~~~~~~~~~~~~~~~~~~~~~   241 (426) |
|  |  |  | .+. ..++++.+++..+|...+...+....+....++. ++....... |
|  | T Consensus | 156 | ~~~----------~~ag~~~gla~~~K~~~~~~~~~~~~~~~~~~~~------------------------~~~~~~~~~   201 (363) |
|  | T PF06728.14 | 156 | GSY----------AIYACCMALTVFINPNALLLFFPSYLILRKCNSS------------------------IKFRQIFVV   201 (363) |
|  | T ss\_pred |  | CCH----------HHHHHHHHHHHhcChHHHHHHHHHHHHHHHcCch------------------------HHHHHHHHH |
|  |
|  |
|  | Q ss\_pred |  | HHHHHHHHHHHHHHHHccCCCccccCCCcccCCCchhhHHhHhhHHHHHHHHHhccccccccCccccCccccccccHHHH |
|  | Q Q6ZXV5 | 242 | LMFSTLLLVVIRVQVIQSQLPVFTRFDNPAAVSPTPTRQLTFNYLLPVNAWLLLNPSELCCDWTMGTIPLIESLLDIRNL   321 (426) |
|  | Q Consensus | 242 | ~~~~~~~~~~~~~~~~~~~~~~~~~~~~~~~~~~~~~~~~~~~~~~~~~~~~~~~~~~~~~~~~~~~~~~~~~~~~~~~~   321 (426) |
|  |  |  | ..+.............+................+...+............ ..... |
|  | T Consensus | 202 | ~~~~~~~~~~~~~~~~~~~~~~~~~~~~~~~~~~~~~~~~~~~~~~~~~~-------------------------~~~~~   256 (363) |
|  | T PF06728.14 | 202 | FLFYLAGLIITSGFFLNSLSFLKIPFRVYLDSHDLTPNLGLWWYFFTEMF-------------------------NEFRT   256 (363) |
|  | T ss\_pred |  | HHHHHHHHHHHHHHhhcCHHHHHHHHhhhccccccCCCHHHHHHHHHhHh-------------------------HHHHH |
|  |
|  |
|  | Q ss\_pred |  | HHHHHHHHHHHHHHHHHHccCCCchhHHHHHHHHHHHHHHHhccCCCcchhhchhhchHHHHHHHHHHHHHHHHhcccch |
|  | Q Q6ZXV5 | 322 | ATFTFFCFLGMLGVFSIRYSGDSSKTVLMALCLMALPFIPASNLFFPVGFVVAERVLYVPSMGFCILVAHGWQKISTKSV   401 (426) |
|  | Q Consensus | 322 | ~~~~~~~~~~~~~~~~~~~~~~~~~~~~~~~~~~~~~~~~~~~~~~~~~~~~~~ry~~~~~~~~~il~~~~~~~~~~~~~   401 (426) |
|  |  |  | ...................+++++................ .....+|.....|.+++... ...++++ |
|  | T Consensus | 257 | ~~~~~~~~~~~~~~~~~~~~~~~~~~~~~~~~~~~~~~~~---------~~~~~~y~~~~~p~~~~~~~----~~~~~~~   323 (363) |
|  | T PF06728.14 | 257 | FFLFVFAILPLMFVLPVSIRLYYLPLPITIALIGLHSLFK---------AYPSICDLSIFLSLLPIFNK----VQDRMRY   323 (363) |
|  | T ss\_pred |  | HHHHHHHHHHHHHHHHHHHHhccChHHHHHHHHHHHHHhC---------CCCcHHHHHHHHHHHHhcHH----HHHHhhh |
|  |
|  |
|  | Q ss\_pred |  | HHHHHHHHHHHHHHHHHHHhhh |
|  | Q Q6ZXV5 | 402 | FKKLSWICLSMVILTHSLKTFH   423 (426) |
|  | Q Consensus | 402 | ~~~~~~~~~~~~~~~~~~~~~~   423 (426) |
|  |  |  | ........+............. |
|  | T Consensus | 324 | ~~~~~~~~~~~~~~~~~~~~~~   345 (363) |
|  | T PF06728.14 | 324 | SLLTNNAIVFALVLGSAFYHSW   345 (363) |
|  | T ss\_pred |  | HHHHHHHHHHHHHHHHHHHHHH |
|  |
| --- | | | |
|  | Template alignmentCDD | | |
| 15. | PF15971.6 ; Mannosyl\_trans4 ; DolP-mannose mannosyltransferase | | |
|  | Probability: 99.35%, E-value: 6.2e-11, Score: 88.77, Aligned cols: 156, Identities: 17%, Similarity: 0.014, | | |
|  |
|  | Q ss\_pred |  | CCCCcchhcCCCCcchHHHHHHHHHHhCCCchHHHHHHH----HHHHHHHHHHHHHHHHhcCC-HHHHHHHHHHHHCccc |
|  | Q Q6ZXV5 | 59 | TPMSEERSHKSYRPLTVLTFRLNYLLSELKPMSYHLLNM----IFHAVVSVIFLKVCKLFLDN-KSSVIASLLFAVHPIH   133 (426) |
|  | Q Consensus | 59 | ~~~~~~~~~~~~~Pl~~~~~~~~~~lfg~~~~~~rl~~~----l~~~~~~~~~y~l~~~~~~~-~~a~~aall~~~~p~~   133 (426) |
|  |  |  | +.....+...++||++.++.....+++|+++...|+.+. +++.+++..+|.+.|+..++ +.+..++++++.+|.. |
|  | T Consensus | 1 | G~~p~~~~~~~~ppl~~~~~~~~~~l~g~~~~~~~~~~~~~~~~~~~~~~~~~~~~~~~~~~~~~~~~~~~~~~~~~p~~   80 (163) |
|  | T PF15971.6 | 1 | GGRLYVDAWEPKLPLSYETTGVLALLSGGDMYRLHLLSVVLMSGAVCAIVALVVMLVYDITGDDIVAPLAGLSMFLLPGF   80 (163) |
|  | T ss\_pred |  | CCCccccccCCCCcHHHHHHHHHHHHhCCchHHHHHHHHHHHHHHHHHHHHHHHHHHHHHhCCCCHHHHHHHHHHHchHH |
|  |
|  |
|  | Q ss\_pred |  | HHH-HHHhhccHHHHHHHHHHHHHHHHHHcCCCCCcccHHHHHHHHHHHHHHHHhHhHHHHHHHHHHHHHHHHhcCCCcc |
|  | Q Q6ZXV5 | 134 | TEA-VTGVVGRAELLSSIFFLAAFLSYTRSKGPDNSIIWTPIALTVFLVAVATLCKEQGITVVGICCVYEVFIAQGYTLP   212 (426) |
|  | Q Consensus | 134 | ~~~-~~~~~~~~~~~~~~~~~l~~~~~~~~~~~~~~~~~~~~~~~~~~~~la~~~k~~~~~~~~~~~~~~~~~~~~~~~~   212 (426) |
|  |  |  | . ......++|....++.+++++...+ ++. ..++++.+++..+|+.+....+.........+++ |
|  | T Consensus | 81 | --~~~~~~~~~~~~~~~~~~~~~~~~~~~---~~~-------~~~~~~~~l~~~~k~~~~~~~~~~~~~~~~~~~~----   144 (163) |
|  | T PF15971.6 | 81 | --AVRPAYGFKAKYLLVLCGLLAIYLYTR---GYP-------ALSGVAAAASVGYWQAGAIFPLIVVGLAVQRRDM----   144 (163) |
|  | T ss\_pred |  | --hccccccccchHHHHHHHHHHHHHHHc---CcH-------HHHHHHHHHHHHhhHHHhHHHHHHHHHHHcccch---- |
|  |
|  |
|  | Q ss\_pred |  | chhcccchhhcCCCCCChHHHHHHHHHHHHHHHHHHHH |
|  | Q Q6ZXV5 | 213 | LLCTTAGQFLRGKGSIPFSMLQTLVKLIVLMFSTLLLV   250 (426) |
|  | Q Consensus | 213 | ~~~~~~~~~~~~~~~~~~~~~~~~~~~~~~~~~~~~~~   250 (426) |
|  |  |  | ++.........+..+... |
|  | T Consensus | 145 | --------------------~~~~~~~~~~~~~~~~~~   162 (163) |
|  | T PF15971.6 | 145 | --------------------RALERVVAGGLGFTIVML   162 (163) |
|  | T ss\_pred |  | --------------------HHHHHHHHHHHHHHHHHh |
|  |
| --- | | | |
|  | Template alignmentCDD | | |
| 16. | PF03155.16 ; Alg6\_Alg8 ; ALG6, ALG8 glycosyltransferase family | | |
|  | Probability: 99.25%, E-value: 3e-9, Score: 93.92, Aligned cols: 325, Identities: 12%, Similarity: 0.009, | | |
|  |
|  | Q ss\_pred |  | ceeccHHHHhcCCCCCCCCcHHHHhccccCCCCCCcchhcCCCCcchHHHHHHHHHHh-----------------CCCch |
|  | Q Q6ZXV5 | 28 | FVFDDVSAILDNKDLHPSTPLKTLFQNDFWGTPMSEERSHKSYRPLTVLTFRLNYLLS-----------------ELKPM   90 (426) |
|  | Q Consensus | 28 | ~~~Dd~~~~~~~~~~~~~~~~~~~~~~~~~~~~~~~~~~~~~~~Pl~~~~~~~~~~lf-----------------g~~~~   90 (426) |
|  |  |  | +..+|.+.+.+..+.....+..+++.++ .+.+..+|||+..+......++. +.... |
|  | T Consensus | 12 | ~~s~D~~~~r~w~~~t~~~p~~~wy~~~-------~~~w~ldYPPl~a~~~~~~~~~~~~~~~~~~~l~~~~~~~~~~~~   84 (470) |
|  | T PF03155.16 | 12 | YHSTDFEVHRNWLAITHSLPLNQWYVDA-------TSEWTLDYPPFFAYFEWLLSQVAKYVDPRMLVVDNLNYESKATVY   84 (470) |
|  | T ss\_pred |  | cCCCcHHHHHHHHHHHhhCCHHHhccCC-------CccCCCCCcHHHHHHHHHHHHHHHHhCHHHhhcccCCCCCHHHHH |
|  |
|  |
|  | Q ss\_pred |  | HHHHHHHHHHHHHHHHHHHHHH---HhcCCHHHHHHHHHHHHCcccHHHHHHhhccHHHHHHHHHHHHHHHHHHcCCCCC |
|  | Q Q6ZXV5 | 91 | SYHLLNMIFHAVVSVIFLKVCK---LFLDNKSSVIASLLFAVHPIHTEAVTGVVGRAELLSSIFFLAAFLSYTRSKGPDN   167 (426) |
|  | Q Consensus | 91 | ~~rl~~~l~~~~~~~~~y~l~~---~~~~~~~a~~aall~~~~p~~~~~~~~~~~~~~~~~~~~~~l~~~~~~~~~~~~~   167 (426) |
|  |  |  | ..|+..++..++....++.+.+ +..+++.+..++++++++|..+ .......+.|.....+.+++++++.+.+. |
|  | T Consensus | 85 | ~~R~~vi~~d~l~~~~v~~~~~~~~~~~~~~~~~~~~~l~l~~P~li-~~d~~~~q~n~~~~~l~llsl~~~~~~~~---   160 (470) |
|  | T PF03155.16 | 85 | FQRLSVIATDLVYVLGVRSCLGSLGLARDTQQFFAGSMLLLLNVGLL-FVDHIHFQYNGLLFGILLLSIGSLIRQRF---   160 (470) |
|  | T ss\_pred |  | HHHHHHHHHHHHHHHHHHHHHHhcCCCCChhHHHHHHHHHHHcHHHH-HhhcccccchHHHHHHHHHHHHHHHcCch--- |
|  |
|  |
|  | Q ss\_pred |  | cccHHHHHHHHHHHHHHHHhHhHHHHHHHHHHHHHHHHhcCCCccchhcccchhhcCCCCCChHHHHHHHHHHHHHHHHH |
|  | Q Q6ZXV5 | 168 | SIIWTPIALTVFLVAVATLCKEQGITVVGICCVYEVFIAQGYTLPLLCTTAGQFLRGKGSIPFSMLQTLVKLIVLMFSTL   247 (426) |
|  | Q Consensus | 168 | ~~~~~~~~~~~~~~~la~~~k~~~~~~~~~~~~~~~~~~~~~~~~~~~~~~~~~~~~~~~~~~~~~~~~~~~~~~~~~~~   247 (426) |
|  |  |  | ..+++++++++.+|...+...+...++.+...+. ++++..+...+.............+ |
|  | T Consensus | 161 | -------~~a~~~~~lal~~K~~~l~~~p~~~~~ll~~~~~--------------~~~~~~~~~~~~~~~~~~~~~~~~~   219 (470) |
|  | T PF03155.16 | 161 | -------LWSAFAFAVLLNFKHIFLYMAPAFGVYLLRFYCL--------------EQASVASAVGAVIKLLVVGLTPFAV   219 (470) |
|  | T ss\_pred |  | -------HHHHHHHHHHHHcchHHHHHHHHHHHHHHHHhcc--------------cCCCHhHHHHHHHHHHHHHHHHHHH |
|  |
|  |
|  | Q ss\_pred |  | HHHHHHHHHHccCCCccccCCCcccCCCchhhHHhHhhHHHHHHHHHhccccccccCccccCccc--cccccHHHHHHHH |
|  | Q Q6ZXV5 | 248 | LLVVIRVQVIQSQLPVFTRFDNPAAVSPTPTRQLTFNYLLPVNAWLLLNPSELCCDWTMGTIPLI--ESLLDIRNLATFT   325 (426) |
|  | Q Consensus | 248 | ~~~~~~~~~~~~~~~~~~~~~~~~~~~~~~~~~~~~~~~~~~~~~~~~~~~~~~~~~~~~~~~~~--~~~~~~~~~~~~~   325 (426) |
|  |  |  | .+..+ .....+...................+.................+.......+.+..+.. ............. |
|  | T Consensus | 220 | ~~~Pf-~~~~~~~~~rlfp~~rgl~~~~~a~n~w~~~~~~~~~~~~~~~~~~~~~~~t~g~~~~~~~~~~~~~~~~~~~~   298 (470) |
|  | T PF03155.16 | 220 | SFGPF-WKQLPQVLSRLFPFKRGLTHAYWAPNFWALYNTADKVAAGVLKVHDGGASTTSGLVQEVRHSVLPAITPPVTFA   298 (470) |
|  | T ss\_pred |  | hcccc-cccHHHHHHHHCCccccccccchhhHHHHHHHHHHHHHHHHHhcCCCCccccCCccccchhhcCCccHHHHHHH |
|  |
|  |
|  | Q ss\_pred |  | HHHHHHHHHHHHHHccCCCchhHHHHHHHHHHHHHHHhccCCCcchhhchhhchHHHHHHHHHHH |
|  | Q Q6ZXV5 | 326 | FFCFLGMLGVFSIRYSGDSSKTVLMALCLMALPFIPASNLFFPVGFVVAERVLYVPSMGFCILVA   390 (426) |
|  | Q Consensus | 326 | ~~~~~~~~~~~~~~~~~~~~~~~~~~~~~~~~~~~~~~~~~~~~~~~~~~ry~~~~~~~~~il~~   390 (426) |
|  |  |  | ..++..........++++......+........+..+... ...+++|...+.+++++++. |
|  | T Consensus | 299 | ~~l~~~~~~~~~l~~~~~~~~~~~~~~~~~~~~l~~flf~-----~~vhek~ill~l~Pl~ll~~   358 (470) |
|  | T PF03155.16 | 299 | LTALFMLPILVKLFRSPKKQSPLVFLRAVVLCGCSSFVFG-----WHVHEKAILMVLLPLCLLTL   358 (470) |
|  | T ss\_pred |  | HHHHHHHHHHHHHHcCCCCCCHHHHHHHHHHHHHHHHHhc-----hhhcchHHHHHHHHHHHHHH |
|  |
| --- | | | |
|  | Template alignmentCDD | | |
| 17. | PF09586.11 ; YfhO ; Bacterial membrane protein YfhO | | |
|  | Probability: 99.23%, E-value: 5.3e-9, Score: 99.82, Aligned cols: 367, Identities: 10%, Similarity: -0.053, | | |
|  |
|  | Q ss\_pred |  | HHHHHHHHHHHHHHHHHhhCCCce--eccHHHHhcCCCCCCCCcHHHHh-ccccCCCCCCcchhcCCCCcchHHHHHHHH |
|  | Q Q6ZXV5 | 6 | LKEITLIVGVVTACYWNSLFCGFV--FDDVSAILDNKDLHPSTPLKTLF-QNDFWGTPMSEERSHKSYRPLTVLTFRLNY   82 (426) |
|  | Q Consensus | 6 | ~~~~~~l~~~~~~~~~~~~~~~~~--~Dd~~~~~~~~~~~~~~~~~~~~-~~~~~~~~~~~~~~~~~~~Pl~~~~~~~~~   82 (426) |
|  |  |  | ...+++++++.+............ .|+..++..... ....... .......++..+......++..++...+.. |
|  | T Consensus | 3 | ~~~~~l~~~~~~~~~~~~~~~~~~~~~D~~~~~~p~~~----~~~~~~~~~~~~~~~~~~~~~G~~~~~~~~~~~~~p~~   78 (832) |
|  | T PF09586.11 | 3 | LLPFAIIFIYGLSRHVFPFGGQTIMTVDLGQQYIDFFA----YFRTTLLQHPDTFFYSFAKGLGGDMLGVWAYYLMSPFN   78 (832) |
|  | T ss\_pred |  | HHHHHHHHHHHHHcCCCCCCCCcceeechhHhHHHHHH----HHHHHHhhCCccceeccccCCCCccHHHHHHHHhCcch |
|  |
|  |
|  | Q ss\_pred |  | HHhC-----CCchHHHHHHHHHHHHHHHHHHHHHHHhc--CCHHHHHHHHHHHHCcccHHHHHHhhccHHHHHHHHHHHH |
|  | Q Q6ZXV5 | 83 | LLSE-----LKPMSYHLLNMIFHAVVSVIFLKVCKLFL--DNKSSVIASLLFAVHPIHTEAVTGVVGRAELLSSIFFLAA   155 (426) |
|  | Q Consensus | 83 | ~lfg-----~~~~~~rl~~~l~~~~~~~~~y~l~~~~~--~~~~a~~aall~~~~p~~~~~~~~~~~~~~~~~~~~~~l~   155 (426) |
|  |  |  | .+.. ..+.+.++..++...++.+.+|.++|+.. ++..|++++++++++|........ ........++.+. |
|  | T Consensus | 79 | ~l~~~~~~~~~~~~~~~~~~l~~~l~~~~~y~l~r~~~~~~~~~a~~~a~~y~~s~~~~~~~~~---~~~~~~~~~lPl~   155 (832) |
|  | T PF09586.11 | 79 | LLVLLTPGKWLSFGVWLMVLLKYGFSGLSFAYYLKKSRLLSGWWLPTLSLTYALSGFAIANQFN---VMWLDAMIWLPLV   155 (832) |
|  | T ss\_pred |  | hHHhhCCHHHHHHHHHHHHHHHHHHHHHHHHHHHHHhccccccHHHHHHHHHHHHHHHHHHHhC---hhHHHHHHHHHHH |
|  |
|  |
|  | Q ss\_pred |  | HHHHHHcCCCCCcccHHHHHHHHHHHHHHHHhHhHHHHHHHHHHHHHHHHh-cCCCccchhcccchhhcCCCCCChHHHH |
|  | Q Q6ZXV5 | 156 | FLSYTRSKGPDNSIIWTPIALTVFLVAVATLCKEQGITVVGICCVYEVFIA-QGYTLPLLCTTAGQFLRGKGSIPFSMLQ   234 (426) |
|  | Q Consensus | 156 | ~~~~~~~~~~~~~~~~~~~~~~~~~~~la~~~k~~~~~~~~~~~~~~~~~~-~~~~~~~~~~~~~~~~~~~~~~~~~~~~   234 (426) |
|  |  |  | ++++.+..++++. +...+..+++.......-.....+..+..++..... ++ ++...+.... |
|  | T Consensus | 156 | l~~~~~~~~~~~~--~~~~~~~~l~~~~~~~~~~~~~~~~~~~~l~~~~~~~~~----------------~~~~~~~~~~   217 (832) |
|  | T PF09586.11 | 156 | VLGIEQLFERQRF--WLYPLSLAALLIINYYMGYMVCLFVVAYFFWASVHHFKT----------------WRQTCLVYLK   217 (832) |
|  | T ss\_pred |  | HHHHHHHHhcCCc--cHHHHHHHHHHHHHHHHHHHHHHHHHHHHHHHHHhcchh----------------HHHHHHHHHH |
|  |
|  |
|  | Q ss\_pred |  | HHHHHHHHHHHHHHHHHHHHHHHccCCCccccCCCcccCCCchhhHHhHhhHHHHHHHHHhccccccccCccccCccccc |
|  | Q Q6ZXV5 | 235 | TLVKLIVLMFSTLLLVVIRVQVIQSQLPVFTRFDNPAAVSPTPTRQLTFNYLLPVNAWLLLNPSELCCDWTMGTIPLIES   314 (426) |
|  | Q Consensus | 235 | ~~~~~~~~~~~~~~~~~~~~~~~~~~~~~~~~~~~~~~~~~~~~~~~~~~~~~~~~~~~~~~~~~~~~~~~~~~~~~~~~   314 (426) |
|  |  |  | .....+..+++.++.++.................................... ...... |
|  | T Consensus | 218 | ~~~~~~l~~~l~a~~llp~~~~~~~~~~~~~~~~~~~~~~~~~~~~~~~~~~~---------------------~~~~~~   276 (832) |
|  | T PF09586.11 | 218 | FAGGSILAGLLAAWLLLPTFFQLTQSKGQYTIQKIHWKIDYNPLKILSKLVVG---------------------NFNFDQ   276 (832) |
|  | T ss\_pred |  | HHHHHHHHHHHHHHHHHHHHHHHhcCcCccCccccccccCCCHHHHHHhhccC---------------------CCCccc |
|  |
|  |
|  | Q ss\_pred |  | cccHHHHHHHHHHHHHHHHHHHHHHccCCCchhHHHHHHHHHHHHHHHhc------cCCCcchhhchhhchHHHHHHHHH |
|  | Q Q6ZXV5 | 315 | LLDIRNLATFTFFCFLGMLGVFSIRYSGDSSKTVLMALCLMALPFIPASN------LFFPVGFVVAERVLYVPSMGFCIL   388 (426) |
|  | Q Consensus | 315 | ~~~~~~~~~~~~~~~~~~~~~~~~~~~~~~~~~~~~~~~~~~~~~~~~~~------~~~~~~~~~~~ry~~~~~~~~~il   388 (426) |
|  |  |  | ............+.++++......++++++.+.....++.+++....... . .........|+.....+.++++ |
|  | T Consensus | 277 | ~~~~~~~~y~g~~~l~l~~~~~~~~~~~~~~~~~~~~~~~~l~~~~~~~~~~~~~~~-~~~~~~~~~R~~~~~~~~~~il   355 (832) |
|  | T PF09586.11 | 277 | MPKGEPNIFVGSLILIGFITYFLTRKIPIKERLAALLVTGFLGLSLCFEPLDLLWHG-MQFPVWYPYRFSYVISFWLIVL   355 (832) |
|  | T ss\_pred |  | CCCCchHHHHHHHHHHHHHHHHHcCCCCHHHHHHHHHHHHHHHHHhcCHHHHHHHhc-CCCCCCCcHHHHHHHHHHHHHH |
|  |
|  |
|  | Q ss\_pred |  | HHHHHHHhcccchHHHHHHHHHHHHHHHHHH |
|  | Q Q6ZXV5 | 389 | VAHGWQKISTKSVFKKLSWICLSMVILTHSL   419 (426) |
|  | Q Consensus | 389 | ~~~~~~~~~~~~~~~~~~~~~~~~~~~~~~~   419 (426) |
|  |  |  | ++.++.+..++++.+......++++++.... |
|  | T Consensus | 356 | a~~~l~~~~~~~~~~~~~~~~~~~~~~~~~~   386 (832) |
|  | T PF09586.11 | 356 | AVQRLHYQPQFKWYSLLAPLLLLAASLAYTF   386 (832) |
|  | T ss\_pred |  | HHHHHhcCCcCChhHHHHHHHHHHHHHHHHH |
|  |
| --- | | | |
|  | Template alignmentCDD | | |
| 18. | PF14264.7 ; Glucos\_trans\_II ; Glucosyl transferase GtrII | | |
|  | Probability: 99.14%, E-value: 7.6e-8, Score: 79.88, Aligned cols: 303, Identities: 12%, Similarity: 0.014, | | |
|  |
|  | Q ss\_pred |  | hCCCceeccHHHHhcCCCCCCCCcHHHHhccccCCCCCCcchhcCCCCcchHHHHHHHHHHhCCCchHHHHHHHHHHHHH |
|  | Q Q6ZXV5 | 24 | LFCGFVFDDVSAILDNKDLHPSTPLKTLFQNDFWGTPMSEERSHKSYRPLTVLTFRLNYLLSELKPMSYHLLNMIFHAVV   103 (426) |
|  | Q Consensus | 24 | ~~~~~~~Dd~~~~~~~~~~~~~~~~~~~~~~~~~~~~~~~~~~~~~~~Pl~~~~~~~~~~lfg~~~~~~rl~~~l~~~~~   103 (426) |
|  |  |  | .+..+..||+..........+. +..++ ||+...+... ....+. ++..++.+.++..++ |
|  | T Consensus | 5 | ~~~~~~~Dd~~~~~~~~~~~~~-----~~~~G---------------R~~~~~l~~~-~~~~~~-p~~~~~l~~~~~~~s   62 (312) |
|  | T PF14264.7 | 5 | FHSSFSHDSLNALYSDMTEIKW-----KLALG---------------RFVVPLIMKI-RGQIAL-PWLIGIVSLFLIAAS   62 (312) |
|  | T ss\_pred |  | cccCCCccchhHhhcCchhhhH-----HHhcc---------------chhHHHHHHH-ccccch-hHHHHHHHHHHHHHH |
|  |
|  |
|  | Q ss\_pred |  | HHHHHHHHHHhcCCHHHHHHHHHHHHCcccHH-HHHHh-hccHHHHHHHHHHHHHHHHHHcCCCCCcccHHHHHHHHHHH |
|  | Q Q6ZXV5 | 104 | SVIFLKVCKLFLDNKSSVIASLLFAVHPIHTE-AVTGV-VGRAELLSSIFFLAAFLSYTRSKGPDNSIIWTPIALTVFLV   181 (426) |
|  | Q Consensus | 104 | ~~~~y~l~~~~~~~~~a~~aall~~~~p~~~~-~~~~~-~~~~~~~~~~~~~l~~~~~~~~~~~~~~~~~~~~~~~~~~~   181 (426) |
|  |  |  | +.+.+...++. ++..+.+++.++..+|...+ ...+. .+.+.....++..++.+... ++++ ++....+.++. |
|  | T Consensus | 63 | ~~l~~~~~~~~-~~~~~~~~~~l~~~~P~~~~~~~~f~~~~~~~~~~~ll~~la~~~~~---~~~~---~~~~~~~~l~~   135 (312) |
|  | T PF14264.7 | 63 | LYLILETIQID-SKAMIILVSIFMVTNRTIYSMTATYIYELDYDMLALFFASLAAYILM---KKDK---PGWYLLAFLSG   135 (312) |
|  | T ss\_pred |  | HHHHHHHhcCC-cHHHHHHHHHHHHHhHHHHHHHHHHHHhcHHHHHHHHHHHHHHHHHH---hCCC---CchHHHHHHHH |
|  |
|  |
|  | Q ss\_pred |  | HHHHHhHhHHHHHHHHHHHHHHHHhcCCCccchhcccchhhcCCCCCChHHHHHHHHHHHHHHHHHHHHHHHHHHHccCC |
|  | Q Q6ZXV5 | 182 | AVATLCKEQGITVVGICCVYEVFIAQGYTLPLLCTTAGQFLRGKGSIPFSMLQTLVKLIVLMFSTLLLVVIRVQVIQSQL   261 (426) |
|  | Q Consensus | 182 | ~la~~~k~~~~~~~~~~~~~~~~~~~~~~~~~~~~~~~~~~~~~~~~~~~~~~~~~~~~~~~~~~~~~~~~~~~~~~~~~   261 (426) |
|  |  |  | .+++.+++......+...+.....+.. +.++..++..++...... .++..+.+...........+ |
|  | T Consensus | 136 | ~~sl~~YQ~~~~~~~~~~~~~~l~~~~--------------~~~~~~k~~~~~~~~~~~-~~~~~i~y~i~~k~~~~~~~   200 (312) |
|  | T PF14264.7 | 136 | VLSLGLYQSYIEVAFAIVIIASLKNLL--------------EGSKYSQVLKRGIIAIVS-FVLSVVAYYLIYKLSCKFFN   200 (312) |
|  | T ss\_pred |  | HHHHHHHHHHHHHHHHHHHHHHHHHHH--------------cCCCcHHHHHHHHHHHHH-HHHHHHHHHHHHHHHHHHcC |
|  |
|  |
|  | Q ss\_pred |  | CccccCCCcccCCCchhhHHhHhhHHHHHHHHHhccccccccCccccCccccccccHHHHHHHHHHHHHHHHHHHHHHcc |
|  | Q Q6ZXV5 | 262 | PVFTRFDNPAAVSPTPTRQLTFNYLLPVNAWLLLNPSELCCDWTMGTIPLIESLLDIRNLATFTFFCFLGMLGVFSIRYS   341 (426) |
|  | Q Consensus | 262 | ~~~~~~~~~~~~~~~~~~~~~~~~~~~~~~~~~~~~~~~~~~~~~~~~~~~~~~~~~~~~~~~~~~~~~~~~~~~~~~~~   341 (426) |
|  |  |  | .+.+...+......... ..+..+...+........ ......................+........++ |
|  | T Consensus | 201 | ~~~~~~~~~~~~~~~~~-~~~i~~~~~~~~~~~~~~-----------~~~~~~~~~~~~~~~~~~~~~~~~~~~~~~~~~   268 (312) |
|  | T PF14264.7 | 201 | VQIEGRTDAFSGEYTSI-IVSLKVMLYKLIHDVVKP-----------GTIYELPIVGIADILLIAIGVALCLIMIFKLGK   268 (312) |
|  | T ss\_pred |  | CCCCCCCCccccccccH-HHHHHHHHHHHHHHhhCC-----------CCCCCCccHHHHHHHHHHHHHHHHHHHHHHHcC |
|  |
|  |
|  | Q ss\_pred |  | CCCchhHHHHHHHHHHHHHHHhccCCC-cchhhchhhchHHHHHH |
|  | Q Q6ZXV5 | 342 | GDSSKTVLMALCLMALPFIPASNLFFP-VGFVVAERVLYVPSMGF   385 (426) |
|  | Q Consensus | 342 | ~~~~~~~~~~~~~~~~~~~~~~~~~~~-~~~~~~~ry~~~~~~~~   385 (426) |
|  |  |  | +++.+. .++.....+.|....... ......+|-..+....+ |
|  | T Consensus | 269 | ~~~~~~---~l~~~~~~~~p~~~~~i~~~~~~~~~r~l~~~~~~~   310 (312) |
|  | T PF14264.7 | 269 | GKTGEK---VVSLLLLAALPLSLNLICLTIKSGSEHDLMTYSFNF   310 (312) |
|  | T ss\_pred |  | CCcHHH---HHHHHHHHHHHHHHHHHHHHCCCCCcchhhhhhhhe |
|  |
| --- | | | |
|  | Template alignmentCDD | | |
| 19. | PF04922.13 ; DIE2\_ALG10 ; DIE2/ALG10 family | | |
|  | Probability: 99.06%, E-value: 1.2e-8, Score: 87.12, Aligned cols: 207, Identities: 9%, Similarity: -0.021, | | |
|  |
|  | Q ss\_pred |  | CceeccHHHHhcCCCCCCCCcH-HHHhccccCCCCCCcchhcCCCCcchHHHHHHHHHHhC---------CCchHHHHHH |
|  | Q Q6ZXV5 | 27 | GFVFDDVSAILDNKDLHPSTPL-KTLFQNDFWGTPMSEERSHKSYRPLTVLTFRLNYLLSE---------LKPMSYHLLN   96 (426) |
|  | Q Consensus | 27 | ~~~~Dd~~~~~~~~~~~~~~~~-~~~~~~~~~~~~~~~~~~~~~~~Pl~~~~~~~~~~lfg---------~~~~~~rl~~   96 (426) |
|  |  |  | ..+.||..|..+++++.+++-. .+...++ ||..+++.+...+++| .+....|..+ |
|  | T Consensus | 3 | ~py~DE~fH~~qa~~y~~G~~~~wdp~iTT---------------pPGlyl~~a~~~~l~g~~~~~~~~~~s~~~LR~~n   67 (434) |
|  | T PF04922.13 | 3 | TPYIDEIFHIPQTQQYCKGHWNAWDSKITT---------------PPGLYIIGYAWARMLTLTGLSESEACSTLSLRAVN   67 (434) |
|  | T ss\_pred |  | CCCcchHhhHHHHHHHHcCCccccCccCCC---------------ChhHHHHHHHHHHHHHHhcCCcccCCCHHHHHHHH |
|  |
|  |
|  | Q ss\_pred |  | HHHHH-HHHHHHHHHHHHhcCCHHHHHHHHHHHHCcccHHHHHHhhccHHHHHHHHHHHHHHHHHHcCCCCCcccHHHHH |
|  | Q Q6ZXV5 | 97 | MIFHA-VVSVIFLKVCKLFLDNKSSVIASLLFAVHPIHTEAVTGVVGRAELLSSIFFLAAFLSYTRSKGPDNSIIWTPIA   175 (426) |
|  | Q Consensus | 97 | ~l~~~-~~~~~~y~l~~~~~~~~~a~~aall~~~~p~~~~~~~~~~~~~~~~~~~~~~l~~~~~~~~~~~~~~~~~~~~~   175 (426) |
|  |  |  | ++++. +...++|.+.++.. ++.+...++.++++|......... .+|...++++++++++..+..+++. .. . |
|  | T Consensus | 68 | ll~~~~~~~~~~~~l~~~~~-~~~a~l~al~l~~~Pl~~~~sfl~--YTDv~Sl~~vll~l~~~l~~~~~~~---~~--~   139 (434) |
|  | T PF04922.13 | 68 | LMAVVIYIPATLYIIQRRVW-GSQAHFSAFSLVSFPLIWFYAALY--YTDVWSTATVLMALAFALSPRVPFY---MV--Q   139 (434) |
|  | T ss\_pred |  | HHHHHHHHHHHHHHHHHHhc-CchHHHHHHHHHhcHHHHHHHHhh--chHHHHHHHHHHHHHHHhCCCCCcc---HH--H |
|  |
|  |
|  | Q ss\_pred |  | HHHHHHHHHHHhHhHHHHHHHHHHHHHHHHhcCCCccchhcccchhhcCCCCCChHHHHHHHHHHH-------------- |
|  | Q Q6ZXV5 | 176 | LTVFLVAVATLCKEQGITVVGICCVYEVFIAQGYTLPLLCTTAGQFLRGKGSIPFSMLQTLVKLIV--------------   241 (426) |
|  | Q Consensus | 176 | ~~~~~~~la~~~k~~~~~~~~~~~~~~~~~~~~~~~~~~~~~~~~~~~~~~~~~~~~~~~~~~~~~--------------   241 (426) |
|  |  |  | .++++.++|+++|++.++..............+ +..+..+....+....... |
|  | T Consensus | 140 | la~l~~~lavl~RQtnIvW~~f~~~~~~~~~~~--------------~~~~~~~~~~~~~l~~~~~~~~~~~~~~~~~~~   205 (434) |
|  | T PF04922.13 | 140 | LSALMCAVSLFFRQTNILWAAVVAVIAIENSHY--------------SNGAPPKNGALAQIFSTISYTFQIELPIFNILI   205 (434) |
|  | T ss\_pred |  | HHHHHHHHHHHhchhHHHHHHHHHHHHHHHHhh--------------hCCCCCcccHHHHHHHHHHHHHhhchHHHHHHH |
|  |
|  |
|  | Q ss\_pred |  | -HHHHHHHHHHHHHHHHccCCCccccCCCc |
|  | Q Q6ZXV5 | 242 | -LMFSTLLLVVIRVQVIQSQLPVFTRFDNP   270 (426) |
|  | Q Consensus | 242 | -~~~~~~~~~~~~~~~~~~~~~~~~~~~~~   270 (426) |
|  |  |  | .+++.+.+..+.....+-..+........ |
|  | T Consensus | 206 | ~~~~v~~~F~~Fv~~NGgIvlGDk~~H~~~   235 (434) |
|  | T PF04922.13 | 206 | SYASVAVGFSFFLYINGGIALGDKDNHVAG   235 (434) |
|  | T ss\_pred |  | HHHHHHHHHHHHHHhCCCcccCccccccCc |
|  |
| --- | | | |
|  | Template alignmentCDD | | |
| 20. | PF05208.14 ; ALG3 ; ALG3 protein | | |
|  | Probability: 99.03%, E-value: 2.1e-8, Score: 84.39, Aligned cols: 198, Identities: 7%, Similarity: 0.008, | | |
|  |
|  | Q ss\_pred |  | HHHHHHHHHHHHHhhCCCce-eccHHHHhcCCCCCCCC-cHHHHhccccCCCCCCcchhcCCCCcchHHHHHHHHHHhCC |
|  | Q Q6ZXV5 | 10 | TLIVGVVTACYWNSLFCGFV-FDDVSAILDNKDLHPST-PLKTLFQNDFWGTPMSEERSHKSYRPLTVLTFRLNYLLSEL   87 (426) |
|  | Q Consensus | 10 | ~~l~~~~~~~~~~~~~~~~~-~Dd~~~~~~~~~~~~~~-~~~~~~~~~~~~~~~~~~~~~~~~~Pl~~~~~~~~~~lfg~   87 (426) |
|  |  |  | .+++.-+.+........++. .|+..|...++.+.+++ ++.+...+. ....+||++.++....+.+.+. |
|  | T Consensus | 2 | ~~~~~~~~~~~~~i~~~~yt~iD~~~y~~~~~~i~~G~~pY~~~~~~~----------~p~~Ypp~~~yi~~~l~~l~~~   71 (356) |
|  | T PF05208.14 | 2 | LLVLVDAVLSALIIKKVSYTEIDWTTYMQQIALYQAGERDYTAIKGDT----------GPLVYPASHVYIYSFLYELTNK   71 (356) |
|  | T ss\_pred |  | HHHHHHHHHHHHHHHhCCCCCCcHHHHHHHHHHHHcCCCChhhccCCC----------CCCCCcHHHHHHHHHHHHHhcC |
|  |
|  |
|  | Q ss\_pred |  | --CchHHHHHHHHHHHHHHHHHHHHHHHhcCCHHHHHHHHHHHHCcccHHHHHHhhccHHHHHHHHHHHHHHHHHHcCCC |
|  | Q Q6ZXV5 | 88 | --KPMSYHLLNMIFHAVVSVIFLKVCKLFLDNKSSVIASLLFAVHPIHTEAVTGVVGRAELLSSIFFLAAFLSYTRSKGP   165 (426) |
|  | Q Consensus | 88 | --~~~~~rl~~~l~~~~~~~~~y~l~~~~~~~~~a~~aall~~~~p~~~~~~~~~~~~~~~~~~~~~~l~~~~~~~~~~~   165 (426) |
|  |  |  | +....|.....+.+++..+++.+.|+ ++..+..++.++...|.+ ......+++|...+++.+++++++.+.+. |
|  | T Consensus | 72 | ~~~i~~~~~~f~~~~l~~~~li~~i~~~--~~~~~~~~~~l~l~~pl~--s~~~~~g~~D~i~~~~lll~l~~l~~~~~-   146 (356) |
|  | T PF05208.14 | 72 | GQDIELGQYIFAGIYIATLIVVLSCYIK--AGAPPYLLPLLVLSKRLH--SIYMLRLFNDGIATLAMWVAIFFFQRRQL-   146 (356) |
|  | T ss\_pred |  | CCChHHHHHHHHHHHHHHHHHHHHHHHH--cCCChhHHHHHHHccHHH--HHHHHhhhcHHHHHHHHHHHHHHHHhCCH- |
|  |
|  |
|  | Q ss\_pred |  | CCcccHHHHHHHHHHHHHHHHhHhHHHHHHHHHHHHHHHHhcCCCccchhcccchhhcCCCCCChHHHHHHHHHHHHHHH |
|  | Q Q6ZXV5 | 166 | DNSIIWTPIALTVFLVAVATLCKEQGITVVGICCVYEVFIAQGYTLPLLCTTAGQFLRGKGSIPFSMLQTLVKLIVLMFS   245 (426) |
|  | Q Consensus | 166 | ~~~~~~~~~~~~~~~~~la~~~k~~~~~~~~~~~~~~~~~~~~~~~~~~~~~~~~~~~~~~~~~~~~~~~~~~~~~~~~~   245 (426) |
|  |  |  | ..++++.++|+.+|...+...+.........++. ++..+......+..+++ |
|  | T Consensus | 147 | ---------~la~i~~glAv~~K~~~ll~~P~ll~~l~~~~~~--------------------~~~~~~~~~~~~~~~l~   197 (356) |
|  | T PF05208.14 | 147 | ---------TVATTVWSLGVGVKMSLLLLAPGVAIVIALSGGI--------------------WAAVPLALNAVLTQVLL   197 (356) |
|  | T ss\_pred |  | ---------HHHHHHHHHHHHHHHHHHHHHHHHHHHHHHcCCH--------------------HHHHHHHHHHHHHHHHH |
|  |
|  |
|  | Q ss\_pred |  | HHHHHH |
|  | Q Q6ZXV5 | 246 | TLLLVV   251 (426) |
|  | Q Consensus | 246 | ~~~~~~   251 (426) |
|  |  |  | ..++.. |
|  | T Consensus | 198 | ~lPfl~   203 (356) |
|  | T PF05208.14 | 198 | GIPFLQ   203 (356) |
|  | T ss\_pred |  | HHHHHh |
|  |
| --- | | | |
|  | Template alignmentCDD | | |
| 21. | PF09594.11 ; GT87 ; Glycosyltransferase family 87 | | |
|  | Probability: 98.86%, E-value: 0.0000011, Score: 70.41, Aligned cols: 242, Identities: 8%, Similarity: -0.054, | | |
|  |
|  | Q ss\_pred |  | CCCCcchHHHHHHHHHHhCC---CchHHHHHHHHHHHHHHHHHHHHHHHhcCC---HHHHHHHHHHHHCcccHHHHHHhh |
|  | Q Q6ZXV5 | 68 | KSYRPLTVLTFRLNYLLSEL---KPMSYHLLNMIFHAVVSVIFLKVCKLFLDN---KSSVIASLLFAVHPIHTEAVTGVV   141 (426) |
|  | Q Consensus | 68 | ~~~~Pl~~~~~~~~~~lfg~---~~~~~rl~~~l~~~~~~~~~y~l~~~~~~~---~~a~~aall~~~~p~~~~~~~~~~   141 (426) |
|  |  |  | ..|||...++.... .+.|+ .....++...+........++.+.|+..++ ......+..++.+|. .+. ... |
|  | T Consensus | 1 | f~YpP~~~~l~~~~-~l~~~~~~~~~~~~~~~~~~~~~~~~~~~~~~r~~~~~~~~~~~~~~~~~~~~~p~-~~~--~~~   76 (251) |
|  | T PF09594.11 | 1 | FTYPPFGALVFTPL-WWIHDLFGLLVTERVFALITLLTTYAVAVFLLRLAGVRDRVWEFVAFAALLVSAPV-YFT--LNI   76 (251) |
|  | T ss\_pred |  | CCCChHHHHHHHHH-hhccchhcHHHHHHHHHHHHHHHHHHHHHHHHHHcCCCCcHHHHHHHHHHHHHHHH-HHH--hhc |
|  |
|  |
|  | Q ss\_pred |  | ccHHHHHHHHHHHHHHHHHHcCCCCCcccHHHHHHHHHHHHHHHHhHhHHHHHHHHHHHHHHHHhcCCCccchhcccchh |
|  | Q Q6ZXV5 | 142 | GRAELLSSIFFLAAFLSYTRSKGPDNSIIWTPIALTVFLVAVATLCKEQGITVVGICCVYEVFIAQGYTLPLLCTTAGQF   221 (426) |
|  | Q Consensus | 142 | ~~~~~~~~~~~~l~~~~~~~~~~~~~~~~~~~~~~~~~~~~la~~~k~~~~~~~~~~~~~~~~~~~~~~~~~~~~~~~~~   221 (426) |
|  |  |  | ++.|.+..++.++++++..+.+++++ ++...++++.+++..+|...+.+.+....+....++. |
|  | T Consensus | 77 | g~~~~~~~~~~~~~l~~~~~~~~~~~----~~~~~ag~~l~la~~~K~~~~~~~~~ll~~~~~~~~~-------------   139 (251) |
|  | T PF09594.11 | 77 | GQINVMLMALTLFDVALPRSTRHSGV----LKYVPLGVLTGIAAAIKLTPLVFGLYFLILWVVTKSP-------------   139 (251) |
|  | T ss\_pred |  | CCHHHHHHHHHHHHHHhccccCCCCc----cccHHHHHHHHHHHHhhHHHHHHHHHHHHHHHHcCCH------------- |
|  |
|  |
|  | Q ss\_pred |  | hcCCCCCChHHHHHHHHHHHHHHHHHHHHHHHHHHHccCCCccccCCCcccCCCchhhHHhHhhHHHHHHHHHhcccccc |
|  | Q Q6ZXV5 | 222 | LRGKGSIPFSMLQTLVKLIVLMFSTLLLVVIRVQVIQSQLPVFTRFDNPAAVSPTPTRQLTFNYLLPVNAWLLLNPSELC   301 (426) |
|  | Q Consensus | 222 | ~~~~~~~~~~~~~~~~~~~~~~~~~~~~~~~~~~~~~~~~~~~~~~~~~~~~~~~~~~~~~~~~~~~~~~~~~~~~~~~~   301 (426) |
|  |  |  | +...........................-....... ............+........ |
|  | T Consensus | 140 | --------r~~~~~~~~~~~~~~~~~~~~~~~~~~~~~~~~~~~-~~~~~~~~~~~~~l~~~~~~~--------------   196 (251) |
|  | T PF09594.11 | 140 | --------RGLFGMIGGFLGASGLAIIFRPSISIQYFTDVLFTA-ERIGDLHFARNVSIRAVLERL--------------   196 (251) |
|  | T ss\_pred |  | --------HHHHHHHHHHHHHHHHHHHHCChHHHHHHHHHHhcc-cccCCccccccccHHHHHHhc-------------- |
|  |
|  |
|  | Q ss\_pred |  | ccCccccCccccccccHHHHHHHHHHHHHHHHHHHHHHccCCCchhHHHHHHHHHHHHH |
|  | Q Q6ZXV5 | 302 | CDWTMGTIPLIESLLDIRNLATFTFFCFLGMLGVFSIRYSGDSSKTVLMALCLMALPFI   360 (426) |
|  | Q Consensus | 302 | ~~~~~~~~~~~~~~~~~~~~~~~~~~~~~~~~~~~~~~~~~~~~~~~~~~~~~~~~~~~   360 (426) |
|  |  |  | .+........................+...+++.++........+...+..+ |
|  | T Consensus | 197 | -------~~~~~~~~~~~~~~~~~~~~~~~~~~~~~~~~~~~~~~~~~~~~~~~~~~~l   248 (251) |
|  | T PF09594.11 | 197 | -------PELGSAASIMWLVAVALVIIAVAVAAYRILRTDLSAHNRLLAVSLVSLVALL   248 (251) |
|  | T ss\_pred |  | -------ccCCcHHHHHHHHHHHHHHHHHHHHHHHHHhcccccCChHHHHHHHHHHHHH |
|  |
| --- | | | |
|  | Template alignmentCDD | | |
| 22. | PF14897.7 ; EpsG ; EpsG family | | |
|  | Probability: 96.65%, E-value: 0.15, Score: 41.42, Aligned cols: 299, Identities: 8%, Similarity: 0.01, | | |
|  |
|  | Q ss\_pred |  | HHHHHHHHHHHHHHhhCC-CceeccHHHHhcCCCCCCCCcHHHHhc-------cccCCCCCCcchhcCCCCcchHHHHHH |
|  | Q Q6ZXV5 | 9 | ITLIVGVVTACYWNSLFC-GFVFDDVSAILDNKDLHPSTPLKTLFQ-------NDFWGTPMSEERSHKSYRPLTVLTFRL   80 (426) |
|  | Q Consensus | 9 | ~~~l~~~~~~~~~~~~~~-~~~~Dd~~~~~~~~~~~~~~~~~~~~~-------~~~~~~~~~~~~~~~~~~Pl~~~~~~~   80 (426) |
|  |  |  | ...++............. +...|...|...-++..+........+ .+ ..|++..+... |
|  | T Consensus | 2 | ~~~~~~~~~l~~~~~~R~~~~g~D~~~Y~~~y~~~~~~~~~~~~~~~~~~~~~~~--------------~E~gf~~l~~~   67 (319) |
|  | T PF14897.7 | 2 | WFVSFATIQWIVLSGFRDVTVGADTAQYKALFLQSQTLPLGAFTDRFFEIVFTES--------------EDPGFYLFQRL   67 (319) |
|  | T ss\_pred |  | HHHHHHHHHHHHHHHHcCcCCCCCHHHHHHHHHHhccCCHHHHhhhhhhccCCCC--------------CCHHHHHHHHH |
|  |
|  |
|  | Q ss\_pred |  | HHHHhCCCchHHHHHHHHHHHHHHHHHHHHHHHhcCCH---HHHHHHHHHHHCcccHHHHHHhhccHHHHHHHH-HHHHH |
|  | Q Q6ZXV5 | 81 | NYLLSELKPMSYHLLNMIFHAVVSVIFLKVCKLFLDNK---SSVIASLLFAVHPIHTEAVTGVVGRAELLSSIF-FLAAF   156 (426) |
|  | Q Consensus | 81 | ~~~lfg~~~~~~rl~~~l~~~~~~~~~y~l~~~~~~~~---~a~~aall~~~~p~~~~~~~~~~~~~~~~~~~~-~~l~~   156 (426) |
|  |  |  | ... +|.+ .+....+.+.++..+.+...+|..++. ..+.............. -..++..+ .+.++ |
|  | T Consensus | 68 | ~~~-~~~~---~~~~~~~~~~i~~~~~~~~~~~~~~~~~~~~~~~~~~~~~~~~~~~~--------Rq~lA~~~~~l~a~   135 (319) |
|  | T PF14897.7 | 68 | IQY-VITD---YQVYLVLIAMIFMIPLGYFIYKYSSEPLISFLLFSVLFYEFFAVTGL--------RQTVATALVVLVGY   135 (319) |
|  | T ss\_pred |  | HHH-hcCC---HHHHHHHHHHHHHHHHHHHHHHhCCchHHHHHHHHHHHHHHHHhHHH--------HHHHHHHHHHHHHH |
|  |
|  |
|  | Q ss\_pred |  | HHHHHcCCCCCcccHHHHHHHHHHHHHHHHhHhHHHHHHHHHHHHHHHHhcCCCccchhcccchhhcCCCCCChHHHHHH |
|  | Q Q6ZXV5 | 157 | LSYTRSKGPDNSIIWTPIALTVFLVAVATLCKEQGITVVGICCVYEVFIAQGYTLPLLCTTAGQFLRGKGSIPFSMLQTL   236 (426) |
|  | Q Consensus | 157 | ~~~~~~~~~~~~~~~~~~~~~~~~~~la~~~k~~~~~~~~~~~~~~~~~~~~~~~~~~~~~~~~~~~~~~~~~~~~~~~~   236 (426) |
|  |  |  | .... ++++ ....+...+|...|.+++...+. ....+++ ..+... |
|  | T Consensus | 136 | ~~~~---~~~~-------~~~~~~~~la~~~H~sali~i~~----~~~~~~~----------------------~~~~~~   179 (319) |
|  | T PF14897.7 | 136 | HFVR---ARKL-------GWFLLLVCIAMTIHKSSLIFVPF----YFLANKQ----------------------LTKAYL   179 (319) |
|  | T ss\_pred |  | HHHH---cCcH-------HHHHHHHHHHHHHHHHHHHHHHH----HHHhhcC----------------------CcHHHH |
|  |
|  |
|  | Q ss\_pred |  | HHHHHHHHHHHHHHHHHHHHHcc--CCCccccCCCcccCCCchhhHHhHhhHHHHHHHHHhccccccccCccccCccccc |
|  | Q Q6ZXV5 | 237 | VKLIVLMFSTLLLVVIRVQVIQS--QLPVFTRFDNPAAVSPTPTRQLTFNYLLPVNAWLLLNPSELCCDWTMGTIPLIES   314 (426) |
|  | Q Consensus | 237 | ~~~~~~~~~~~~~~~~~~~~~~~--~~~~~~~~~~~~~~~~~~~~~~~~~~~~~~~~~~~~~~~~~~~~~~~~~~~~~~~   314 (426) |
|  |  |  | ......................+ ..+.+...+..... ... |
|  | T Consensus | 180 | ~~~~~~~~~~~~~~~~i~~~~~~~~~~~~Y~~~~~~~~~---~~~-----------------------------------   221 (319) |
|  | T PF14897.7 | 180 | MTMFGVIVGLFVFRNPFFDLLVQVSGYDTYSAMDGAGAV---NFS-----------------------------------   221 (319) |
|  | T ss\_pred |  | HHHHHHHHHHHHhcHHHHHHHHHHhchhhhhhccCcchH---HHH----------------------------------- |
|  |
|  |
|  | Q ss\_pred |  | cccHHHHHHHHHHHHHHHHHHHHHHccCCCch-hHHHHHHHHHHHHHHHhccCCCcchhhchhhchHHHHHHHHHHHHHH |
|  | Q Q6ZXV5 | 315 | LLDIRNLATFTFFCFLGMLGVFSIRYSGDSSK-TVLMALCLMALPFIPASNLFFPVGFVVAERVLYVPSMGFCILVAHGW   393 (426) |
|  | Q Consensus | 315 | ~~~~~~~~~~~~~~~~~~~~~~~~~~~~~~~~-~~~~~~~~~~~~~~~~~~~~~~~~~~~~~ry~~~~~~~~~il~~~~~   393 (426) |
|  |  |  | ................++++.+. ..............+.... .....|......+... .....+ |
|  | T Consensus | 222 | ---------~~~~~~~~~~~~~~~~~~~~~~~~~~~~~~~~~~~~~~~~~~~-----~~~~~R~~~~~~~~~~-~~~~~~   286 (319) |
|  | T PF14897.7 | 222 | ---------LMLLSVLFVALWRKEQILANNPSAIHFFNALLLAACLLPLTFL-----NPSMMRLVQYFSLFLL-LMIPEI   286 (319) |
|  | T ss\_pred |  | ---------HHHHHHHHHHHHHHHHHhcCCcchHHHHHHHHHHHHHHHHHhc-----ChhHHHHHHHHHHHHH-HHHHHH |
|  |
|  |
|  | Q ss\_pred |  | HHhcccchHHHHHHHHHHHHHHHHHHHhh |
|  | Q Q6ZXV5 | 394 | QKISTKSVFKKLSWICLSMVILTHSLKTF   422 (426) |
|  | Q Consensus | 394 | ~~~~~~~~~~~~~~~~~~~~~~~~~~~~~   422 (426) |
|  |  |  | .+..++++++......+............ |
|  | T Consensus | 287 | ~~~~~~~~~~~~~~~~~~~~~~~~~~~~~   315 (319) |
|  | T PF14897.7 | 287 | VGTFERRERLVVYYSAVMLLGLLFIREAP   315 (319) |
|  | T ss\_pred |  | HhcCChHHHHHHHHHHHHHHHHHHHHHcc |
|  |
| --- | | | |
|  | Template alignmentCDD | | |
| 23. | PF05007.14 ; Mannosyl\_trans ; Mannosyltransferase (PIG-M) | | |
|  | Probability: 96.39%, E-value: 0.13, Score: 40.53, Aligned cols: 191, Identities: 11%, Similarity: -0.041, | | |
|  |
|  | Q ss\_pred |  | ccHHHHHHHHHHHHHHHHHHcCCCCCcccHHHHHHHHHHHHHHHHhHhHHHHHHHHHHHHHHHHhcCCCccchhcccchh |
|  | Q Q6ZXV5 | 142 | GRAELLSSIFFLAAFLSYTRSKGPDNSIIWTPIALTVFLVAVATLCKEQGITVVGICCVYEVFIAQGYTLPLLCTTAGQF   221 (426) |
|  | Q Consensus | 142 | ~~~~~~~~~~~~l~~~~~~~~~~~~~~~~~~~~~~~~~~~~la~~~k~~~~~~~~~~~~~~~~~~~~~~~~~~~~~~~~~   221 (426) |
|  |  |  | ++.|....+++++++++..|.+. ..++++.|+|..+|...+.+.+...+...-.+++ |
|  | T Consensus | 5 | G~~D~l~~~lvllal~~~~r~~~----------~~ag~~lgla~~~Kl~Pii~~~~l~l~~~~~~~~-------------   61 (269) |
|  | T PF05007.14 | 5 | GNADSIVASLVLTTLYLIEKRLI----------ACAAVFYGFAVHMKMYPVTYILPIALHLRPERDS-------------   61 (269) |
|  | T ss\_pred |  | hhHHHHHHHHHHHHHHHHHcCCH----------HHHHHHHHHHHHhchHHHHHHHHHHHHhCccCCC------------- |
|  |
|  |
|  | Q ss\_pred |  | hcCCCCCChHHHHHHH--------------HHHHHHHHHHHHHHHHHHHHccCCCccccCCCcccCCCchhhHHhHhhHH |
|  | Q Q6ZXV5 | 222 | LRGKGSIPFSMLQTLV--------------KLIVLMFSTLLLVVIRVQVIQSQLPVFTRFDNPAAVSPTPTRQLTFNYLL   287 (426) |
|  | Q Consensus | 222 | ~~~~~~~~~~~~~~~~--------------~~~~~~~~~~~~~~~~~~~~~~~~~~~~~~~~~~~~~~~~~~~~~~~~~~   287 (426) |
|  |  |  | +++....+...+.... .....................+...................+........ |
|  | T Consensus | 62 | ~~~~~~~~~~~~~~~~~~~~~~~~~~~~~~~~~~~~~~~~l~~~~~~~~g~~~~~~~~~~~~~r~~~~~n~S~~~~~~~l   141 (269) |
|  | T PF05007.14 | 62 | DEGLRLARYSFQARLYDFLKRLCSWAVLLFVAIAGLTFLALSFGFYYKYGWEFLEHTYLYHLTRRDIRHNFSPYFYMLYL   141 (269) |
|  | T ss\_pred |  | CcchhhhcccHHHHHHHHHHHhccHHHHHHHHHHHHHHHHHHHHHHHHHCHHHHHHHHHHHhccCCCCcCCCHHHHHHHH |
|  |
|  |
|  | Q ss\_pred |  | HHHHHHHhccccccccCccccCccccccccHHHHHHHHHHHHHHHHHHHHHHccCCCchhHHHHHHHHHHHHHHHhccCC |
|  | Q Q6ZXV5 | 288 | PVNAWLLLNPSELCCDWTMGTIPLIESLLDIRNLATFTFFCFLGMLGVFSIRYSGDSSKTVLMALCLMALPFIPASNLFF   367 (426) |
|  | Q Consensus | 288 | ~~~~~~~~~~~~~~~~~~~~~~~~~~~~~~~~~~~~~~~~~~~~~~~~~~~~~~~~~~~~~~~~~~~~~~~~~~~~~~~~   367 (426) |
|  |  |  | ..... .............++.......+.+++...........++..- |
|  | T Consensus | 142 | ----------------------~~~~~--~~~~~~~~~~~~~~~~~~~~~~~~~~~~~~~~~~~~~~f~~~~--------   189 (269) |
|  | T PF05007.14 | 142 | ----------------------TAESK--WSFTLGIAAFLPQFILLSAASFAYYRDLVFCCFLHTSIFVTFN--------   189 (269) |
|  | T ss\_pred |  | ----------------------hccCc--chhHHHHHHHHHHHHHHHHHHHHccCcHHHHHHHHHHHHHHhc-------- |
|  |
|  |
|  | Q ss\_pred |  | CcchhhchhhchHHHHHHHHHHH |
|  | Q Q6ZXV5 | 368 | PVGFVVAERVLYVPSMGFCILVA   390 (426) |
|  | Q Consensus | 368 | ~~~~~~~~ry~~~~~~~~~il~~   390 (426) |
|  |  |  | +...++|.....|++.+... |
|  | T Consensus | 190 | ---~v~~~qY~~W~lpll~l~~~   209 (269) |
|  | T PF05007.14 | 190 | ---KVCTSQYFLWYLCLLPLVMP   209 (269) |
|  | T ss\_pred |  | ---hhhcHHHHHHHHHHHHHHch |
|  |
| --- | | | |
|  | Template alignmentCDD | | |
| 24. | PF10060.10 ; DUF2298 ; Uncharacterized membrane protein (DUF2298) | | |
|  | Probability: 91.75%, E-value: 4.9, Score: 35.57, Aligned cols: 344, Identities: 13%, Similarity: 0.067, | | |
|  |
|  | Q ss\_pred |  | cchhHHHHHHHHHHHHHHHHHhhCCCce-eccHHHHhcCCCCCCC--CcHHHHhccccCCCCCCcchhcCCCCcchHHHH |
|  | Q Q6ZXV5 | 2 | ANINLKEITLIVGVVTACYWNSLFCGFV-FDDVSAILDNKDLHPS--TPLKTLFQNDFWGTPMSEERSHKSYRPLTVLTF   78 (426) |
|  | Q Consensus | 2 | ~~~~~~~~~~l~~~~~~~~~~~~~~~~~-~Dd~~~~~~~~~~~~~--~~~~~~~~~~~~~~~~~~~~~~~~~~Pl~~~~~   78 (426) |
|  |  |  | +.....-.+.+++....+.....+.+.+ .|...+....+..... -+..+.+-.+ ...+|+.+.+++. |
|  | T Consensus | 53 | ~~~l~~e~vf~~~f~~~~~~r~~~p~i~~~Ek~md~~~i~s~~~~~~~Pp~dPw~aG----------~~l~Yyyfg~~~~   122 (597) |
|  | T PF10060.10 | 53 | RYILLFELLFLGAFAAWAWVRAHDPAADHTEQPMDLMFMHSIRASLTYPPHDAWLAG----------YPISYYYFGYWLM   122 (597) |
|  | T ss\_pred |  | HHHHHHHHHHHHHHHHHHHHHHhCCCCCCCCChhHHHHHHHHHhcCCCCccchhhcC----------CCccccHHHHHHH |
|  |
|  |
|  | Q ss\_pred |  | HHHHHHhC-CCchHHHHHHHHHHHHHHHHHHHHHHHhc-CCH--------HHHHHHHHHHHCcccHH------------- |
|  | Q Q6ZXV5 | 79 | RLNYLLSE-LKPMSYHLLNMIFHAVVSVIFLKVCKLFL-DNK--------SSVIASLLFAVHPIHTE-------------   135 (426) |
|  | Q Consensus | 79 | ~~~~~lfg-~~~~~~rl~~~l~~~~~~~~~y~l~~~~~-~~~--------~a~~aall~~~~p~~~~-------------   135 (426) |
|  |  |  | +...++.| ....++++.......+....+|.+++++. +++ .|.+++++..+...... |
|  | T Consensus | 123 | A~l~~l~gi~~~~~~nl~~~~~~al~~~~~~~l~~~l~~~~~~~~~~~~~~g~la~~l~~~~gnl~~~~~~~~~~~~~~~   202 (597) |
|  | T PF10060.10 | 123 | NMVGLMAGQSAAVAYNLSQAVWFGLLLSGAFGIGYNLVAAAGRRFVAALGGGWVATLLVGLSSNLQGLLEWLHANGVDIS   202 (597) |
|  | T ss\_pred |  | HHHHHHhCCCHHHHHHHHHHHHHHHHHHHHHHHHHHHHHhccccccHHHHHHHHHHHHHHHhcccHHHHHHHHhCCCCch |
|  |
|  |
|  | Q ss\_pred |  | --------------------------HHHHhhccHH--------------------------------HHHHHHHHHHHH |
|  | Q Q6ZXV5 | 136 | --------------------------AVTGVVGRAE--------------------------------LLSSIFFLAAFL   157 (426) |
|  | Q Consensus | 136 | --------------------------~~~~~~~~~~--------------------------------~~~~~~~~l~~~   157 (426) |
|  |  |  | ...|.+++.- .+..++.+..++ |
|  | T Consensus | 203 | ~~~~~~~~~~~~~~~~~~~~~~~~~~w~~w~ssRvI~~~~~~~~~~~tI~EFP~fSfl~gDLHpH~~alPf~ll~l~l~~   282 (597) |
|  | T PF10060.10 | 203 | WLAAWLQVRGFPENAEVTRQWFISYGWWWWRSSRVLADVSLRGDHIEVIDEFPAFSYILGDNHPHVAAMPFAMLAVAAAL   282 (597) |
|  | T ss\_pred |  | hHHHHhhhcCCCcchhhhccccccCccccccceeeeecccCCCCCCcccccCchHHHhcCCCChhhhHHHHHHHHHHHHH |
|  |
|  |
|  | Q ss\_pred |  | HHHHcCCCCCcccHH---------------HHHHHHHHHHHHHHhHhHHHHHHHHHHHHHHHHhcCCCccchhcccchhh |
|  | Q Q6ZXV5 | 158 | SYTRSKGPDNSIIWT---------------PIALTVFLVAVATLCKEQGITVVGICCVYEVFIAQGYTLPLLCTTAGQFL   222 (426) |
|  | Q Consensus | 158 | ~~~~~~~~~~~~~~~---------------~~~~~~~~~~la~~~k~~~~~~~~~~~~~~~~~~~~~~~~~~~~~~~~~~   222 (426) |
|  |  |  | ...+..++++..++. ..++.+++.|....++.--......+.......... |
|  | T Consensus | 283 | ~~~~~~~~~~~~~~~~~~~~~~~~~~~~~~~~ll~gll~G~l~~~NtWD~p~~~~l~~~~~~~~~~--------------   348 (597) |
|  | T PF10060.10 | 283 | VIFLQNSSSNFSSESRAKFNFAPLFPLGWGGFLLVAVITGSLLFLNTWDYPPYWLLTTFSIAVGVV--------------   348 (597) |
|  | T ss\_pred |  | HHHhcCCCCCCCchhhhccCCCCccccchHHHHHHHHHHHHHHHHccCCHHHHHHHHHHHHHHHHh-------------- |
|  |
|  |
|  | Q ss\_pred |  | cCCC-------------CCChHHHHHHHHHHHHHHHHHHHHHHHHHHHccCCCccccCCCcccCCCchhhHHhHhhHHHH |
|  | Q Q6ZXV5 | 223 | RGKG-------------SIPFSMLQTLVKLIVLMFSTLLLVVIRVQVIQSQLPVFTRFDNPAAVSPTPTRQLTFNYLLPV   289 (426) |
|  | Q Consensus | 223 | ~~~~-------------~~~~~~~~~~~~~~~~~~~~~~~~~~~~~~~~~~~~~~~~~~~~~~~~~~~~~~~~~~~~~~~   289 (426) |
|  |  |  | +. ...++.++.....+...+........+...+.+..++ |
|  | T Consensus | 349 | --~~~~~~~~~~~~~~~~~~~~~~~~~~~~~~~~~~a~ll~lPF~l~f~~~~~g--------------------------   400 (597) |
|  | T PF10060.10 | 349 | --GGVVRVKNFLPLQFLPLLPPLLQTTIAGLALFVAALLLYLPYLLTAQSQVGG--------------------------   400 (597) |
|  | T ss\_pred |  | --ccccccccccccccccchHHHHHHHHHHHHHHHHHHHHHHHHHHhccccCCC-------------------------- |
|  |
|  |
|  | Q ss\_pred |  | HHHHHhccccccccCccccCcccc-ccccHHHHHHHHHHHHHHHHHHHHHHccCCCchhHHHHHHHHHHHHHHHhc---- |
|  | Q Q6ZXV5 | 290 | NAWLLLNPSELCCDWTMGTIPLIE-SLLDIRNLATFTFFCFLGMLGVFSIRYSGDSSKTVLMALCLMALPFIPASN----   364 (426) |
|  | Q Consensus | 290 | ~~~~~~~~~~~~~~~~~~~~~~~~-~~~~~~~~~~~~~~~~~~~~~~~~~~~~~~~~~~~~~~~~~~~~~~~~~~~----   364 (426) |
|  |  |  | ..... ..+............+...........++.+++..............+... |
|  | T Consensus | 401 | -------------------i~~~~~~~T~l~~~l~i~Glfl~l~~~~l~~~~~~~~~~~~~~~~~~~~~~~~~~~~~~~~   461 (597) |
|  | T PF10060.10 | 401 | -------------------LIPNLFHPTRFSQYVAMFATALLTLTALLTFGWSVFRPRLKVVMICLALTLGTPALLLTFI   461 (597) |
|  | T ss\_pred |  | -------------------CcccCCCCCCHHHHHHHHHHHHHHHHHHHHHHhhcccchHHHHHHHHHHHHHHHHHHHHHH |
|  |
|  |
|  | Q ss\_pred |  | --------------------------cCCCcchhhchhhchHH--HHHHHHHHHHHHHHhcccch--------------- |
|  | Q Q6ZXV5 | 365 | --------------------------LFFPVGFVVAERVLYVP--SMGFCILVAHGWQKISTKSV---------------   401 (426) |
|  | Q Consensus | 365 | --------------------------~~~~~~~~~~~ry~~~~--~~~~~il~~~~~~~~~~~~~---------------   401 (426) |
|  |  |  | . .....|....+ ...+..+++.......++.+ |
|  | T Consensus | 462 | ~~~~~~~~~~~~~~~~~~~~~~~~~~~-----~~~~~~~~~~~~~~~ll~~l~~~~~~~~~~~~~~~~~~~~~~~~~~~~   536 (597) |
|  | T PF10060.10 | 462 | AWVATGTEEGRASLGNVALPDGASSYL-----PFIVERWTAQPFTFLIVGAMTAVALALLWTGIQHMVGAKNFLPQHFSP   536 (597) |
|  | T ss\_pred |  | HHHhcCChhhhhccccccCCCCcccch-----hHHHHHhccchHHHHHHHHHHHHHHHHHHhhhhhhccccccCcccCCC |
|  |
|  |
|  | Q ss\_pred |  | --------------HHHHHHHHHHHHHHHHHHHh |
|  | Q Q6ZXV5 | 402 | --------------FKKLSWICLSMVILTHSLKT   421 (426) |
|  | Q Consensus | 402 | --------------~~~~~~~~~~~~~~~~~~~~   421 (426) |
|  |  |  | .......+++.+........ |
|  | T Consensus | 537 | ~~~~~~~~~~~~~~~~~~f~l~L~~~gl~Lil~~   570 (597) |
|  | T PF10060.10 | 537 | QQGALDSTPGVAAPTPLLFVLALAVIGLGLTFTP   570 (597) |
|  | T ss\_pred |  | CCCCcCCCCCCCCCHHHHHHHHHHHHHHHHHHHH |
|  |
| --- | | | |
|  | Template alignmentCDD | | |
| 25. | PF16192.6 ; PMT\_4TMC ; C-terminal four TMM region of protein-O-mannosyltransferase | | |
|  | Probability: 86.81%, E-value: 5.3, Score: 29.1, Aligned cols: 103, Identities: 8%, Similarity: -0.068, | | |
|  |
|  | Q ss\_pred |  | HHHHHHHHHHHHHHHHHHHHHHccCCCchh-----------HHHHHHHHHHHHHHHhccCCCcchhhchhhchHHHHHHH |
|  | Q Q6ZXV5 | 318 | IRNLATFTFFCFLGMLGVFSIRYSGDSSKT-----------VLMALCLMALPFIPASNLFFPVGFVVAERVLYVPSMGFC   386 (426) |
|  | Q Consensus | 318 | ~~~~~~~~~~~~~~~~~~~~~~~~~~~~~~-----------~~~~~~~~~~~~~~~~~~~~~~~~~~~~ry~~~~~~~~~   386 (426) |
|  |  |  | ........+....+..+....++++..... .....+..++..+| +..........|.++++++.+ |
|  | T Consensus | 63 | iw~~~~~~l~~~~~~~~~~~~~~~r~~~~~~~~~~~~~~~~~~~~~~g~~~~ylP----~~~~~r~~~~~~ylpal~f~~   138 (198) |
|  | T PF16192.6 | 63 | VYWASTASLGLVGLVVVWYILRWQRGFKDLDSEEVDQIHYAGIYPVLGWFLHYLP----FVIMARVTYVHHYYPALYFAI   138 (198) |
|  | T ss\_pred |  | HHHHHHHHHHHHHHHHHHHHHHHhcCCCCCChHHHHHHHHHHHHHHHHHHHHHHH----HHhccccccHHhHHHHHHHHH |
|  |
|  |
|  | Q ss\_pred |  | HHHHHHHHHhcccchHHHHHHHHHHHHHHHHHHHhhhh |
|  | Q Q6ZXV5 | 387 | ILVAHGWQKISTKSVFKKLSWICLSMVILTHSLKTFHR   424 (426) |
|  | Q Consensus | 387 | il~~~~~~~~~~~~~~~~~~~~~~~~~~~~~~~~~~~~   424 (426) |
|  |  |  | ++.+..++...++..++.......+.+.+....+.... |
|  | T Consensus | 139 | l~~~~~l~~~~~~~~~~~~~~~~~~~~~~~~~~f~~~~   176 (198) |
|  | T PF16192.6 | 139 | LALGFFVDWLLRNRSQAIQGAVYGVLYSVIVGLYITFI   176 (198) |
|  | T ss\_pred |  | HHHHHHHHHHHHccchHHHHHHHHHHHHHHHHHHHHhh |
|  |
| --- | | | |
|  | Template alignmentCDD | | |
| 26. | PF09971.10 ; DUF2206 ; Predicted membrane protein (DUF2206) | | |
|  | Probability: 77.03%, E-value: 21, Score: 29.16, Aligned cols: 212, Identities: 5%, Similarity: -0.079, | | |
|  |
|  | Q ss\_pred |  | CCcccHHHHHHHHHHHHHHHHhHhHHHHHHHHHHHHHHHHhcCCCccchhcccchhhcCCC------CCChHHH------ |
|  | Q Q6ZXV5 | 166 | DNSIIWTPIALTVFLVAVATLCKEQGITVVGICCVYEVFIAQGYTLPLLCTTAGQFLRGKG------SIPFSML------   233 (426) |
|  | Q Consensus | 166 | ~~~~~~~~~~~~~~~~~la~~~k~~~~~~~~~~~~~~~~~~~~~~~~~~~~~~~~~~~~~~------~~~~~~~------   233 (426) |
|  |  |  | +| ++..++...-..+|+....+...++........- .+ ..+.... |
|  | T Consensus | 1 | ~~-------~L~~i~~~~lv~sH~~t~~~~~~~l~~~~~~~~~----------------~~~~~~~~~~~~~~~~~~~~~   57 (390) |
|  | T PF09971.10 | 1 | WG-------ILFSVFSCGIIISHYGLTYMVIGLIALSYVLFTF----------------INLVARYINTDKVIIPVTPIR   57 (390) |
|  | T ss\_pred |  | Ch-------HHHHHHHHHHHHhchHHHHHHHHHHHHHHHHHHH----------------HHHHHHhcCCCCCcCCCCccc |
|  |
|  |
|  | Q ss\_pred |  | HHHHHHHHHHHHHHHHHHHHHHHHccCCCccccCCCcccCCCchhhHHhHhhHHHHHHHHHhccccccccCccccCcccc |
|  | Q Q6ZXV5 | 234 | QTLVKLIVLMFSTLLLVVIRVQVIQSQLPVFTRFDNPAAVSPTPTRQLTFNYLLPVNAWLLLNPSELCCDWTMGTIPLIE   313 (426) |
|  | Q Consensus | 234 | ~~~~~~~~~~~~~~~~~~~~~~~~~~~~~~~~~~~~~~~~~~~~~~~~~~~~~~~~~~~~~~~~~~~~~~~~~~~~~~~~   313 (426) |
|  |  |  | ......+...+....|+.. .......+............+...+.................. |
|  | T Consensus | 58 | ~~~~~~~~~~v~~~~W~~~------------------~~~~~~~~~~~~~~~~~~~~~~~~~~~~~~~~~~~~~~~~~~~   119 (390) |
|  | T PF09971.10 | 58 | LNFLHICIFIFIALSWYIA------------------ITSSTAFYSVSSVIYQVISSMFTESLNPTASQGLAIIQKVPVS   119 (390) |
|  | T ss\_pred |  | ccHHHHHHHHHHHHHHHHH------------------HhcchHHHHHHHHHHHHHHHHhHHhcCCccchhHHHHhcCCCc |
|  |
|  |
|  | Q ss\_pred |  | ccccHHHHHHHHHHHHHHHHHHHH----HHccCCCchhHHHHHHHHHHHHHHHhccCCCcc--hhhchhhchHHHHHHHH |
|  | Q Q6ZXV5 | 314 | SLLDIRNLATFTFFCFLGMLGVFS----IRYSGDSSKTVLMALCLMALPFIPASNLFFPVG--FVVAERVLYVPSMGFCI   387 (426) |
|  | Q Consensus | 314 | ~~~~~~~~~~~~~~~~~~~~~~~~----~~~~~~~~~~~~~~~~~~~~~~~~~~~~~~~~~--~~~~~ry~~~~~~~~~i   387 (426) |
|  |  |  | ...............+.+.+.... .+++++.........+......+.......+.. .....|......+++++ |
|  | T Consensus | 120 | ~~~~~~~~~~~~~~~l~~iG~~~~~~~~~~~~~~~~~~~~~~~~~~~~~~~~~~~~~~p~~~~~~~~~R~~~~~~~~~~~   199 (390) |
|  | T PF09971.10 | 120 | QMHLLYTYIYYFNQVCIVLGLLYLSYKTFARKNMYNYSIMQLIMCGVAVMVLVGSIVLPYFASALNTTRIYHIMQFFVSP   199 (390) |
|  | T ss\_pred |  | hHHHHHHHHHHHHHHHHHHHHHHHHHHHHhccCccCCCHHHHHHHHHHHHHHHHHHHHHHHHHhcChHHHHHHHHHHHHH |
|  |
|  |
|  | Q ss\_pred |  | HHHHHHHHhcccchHH----------------HHHHHHHHHHHHHHH |
|  | Q Q6ZXV5 | 388 | LVAHGWQKISTKSVFK----------------KLSWICLSMVILTHS   418 (426) |
|  | Q Consensus | 388 | l~~~~~~~~~~~~~~~----------------~~~~~~~~~~~~~~~   418 (426) |
|  |  |  | +++.++..+.+..++. .....+++++.+++. |
|  | T Consensus | 200 | ~a~~g~~~l~~~~~~~~~~~~~~~~~~~~~~~~~~~~~~lv~~~~~~   246 (390) |
|  | T PF09971.10 | 200 | VYIIGFIFALESIPKVYARIVKSPFRSNLSFTYGIISLFLCVYLLFN   246 (390) |
|  | T ss\_pred |  | HHHHHHHHHHHhhHHHHHHhhcCccccccchHHHHHHHHHHHHHHHH |
|  |
| --- | | | |
|  | Template alignmentCDD | | |
| 27. | PF16316.6 ; DUF4956 ; Domain of unknown function (DUF4956) | | |
|  | Probability: 40.45%, E-value: 65, Score: 22.35, Aligned cols: 103, Identities: 13%, Similarity: 0.079, | | |
|  |
|  | Q ss\_pred |  | HHHHHHHHHHHHHhcCCHHHHHHHHHHHHCcccHHHHHHhhccHHHHHHHHHHHHHHHHHHcCCCCCcccHHHHHHHHHH |
|  | Q Q6ZXV5 | 101 | AVVSVIFLKVCKLFLDNKSSVIASLLFAVHPIHTEAVTGVVGRAELLSSIFFLAAFLSYTRSKGPDNSIIWTPIALTVFL   180 (426) |
|  | Q Consensus | 101 | ~~~~~~~y~l~~~~~~~~~a~~aall~~~~p~~~~~~~~~~~~~~~~~~~~~~l~~~~~~~~~~~~~~~~~~~~~~~~~~   180 (426) |
|  |  |  | +...++++.+.++..+++....+-.+.......+...... ........+.+++.....|.+.+.+...-...+....+ |
|  | T Consensus | 3 | ~~~~~~~~~~y~~~~~~~~~~~t~~lv~~~~~~i~~~~~~--~~~~i~~g~g~~Ga~~Iir~r~~~~~~~~~a~i~~~~~   80 (168) |
|  | T PF16316.6 | 3 | VAVSILAFGLYLPRHHRRDLVVSYLVVNVGVLAVSTALAS--SAIAAGLGLGLFGVLSIIRLRSAELAHHEIAYYFAALA   80 (168) |
|  | T ss\_pred |  | HHHhHhHHHHhccccCCcchHHHHHHHHHHHHHHHHHHHh--chhhHHHHHHHHHHHHhhhcCCCCCCHHHHHHHHHHHH |
|  |
|  |
|  | Q ss\_pred |  | HHHHHHhH--hHHHHHHHHHHHHHHHH |
|  | Q Q6ZXV5 | 181 | VAVATLCK--EQGITVVGICCVYEVFI   205 (426) |
|  | Q Consensus | 181 | ~~la~~~k--~~~~~~~~~~~~~~~~~   205 (426) |
|  |  |  | .|++.... ...........+..... |
|  | T Consensus | 81 | iGl~~G~g~~~~~~a~~~t~~i~~~l~   107 (168) |
|  | T PF16316.6 | 81 | LGLLGGLGSAAGPLALVGMAVLLLVLA   107 (168) |
|  | T ss\_pred |  | HHHHHhhccchHHHHHHHHHHHHHHHH |
|  |
| --- | | | |
|  | Template alignmentCDD | | |
| 28. | PF11694.9 ; DUF3290 ; Protein of unknown function (DUF3290) | | |
|  | Probability: 23.25%, E-value: 130, Score: 20.29, Aligned cols: 59, Identities: 8%, Similarity: -0.003, | | |
|  |
|  | Q ss\_pred |  | CccccCccccccccHHHHHHHHHHHHHHHHHHHHHHccCCCchhHHHHHHHHHHHHHHH |
|  | Q Q6ZXV5 | 304 | WTMGTIPLIESLLDIRNLATFTFFCFLGMLGVFSIRYSGDSSKTVLMALCLMALPFIPA   362 (426) |
|  | Q Consensus | 304 | ~~~~~~~~~~~~~~~~~~~~~~~~~~~~~~~~~~~~~~~~~~~~~~~~~~~~~~~~~~~   362 (426) |
|  |  |  | |++.-................+.++++++.......|+|...+..=+.++.+++.++.. |
|  | T Consensus | 3 | Ysy~YL~~q~~~~~~~~~~~i~~l~~~li~~~~~y~R~R~~tKYRDL~II~~L~~ll~~   61 (142) |
|  | T PF11694.9 | 3 | YTYDYLQGSQSSWQYARIIILSVLAVIFIGFLVHYLRNRMDSKYKDLTIIVGTLLLLIL   61 (142) |
|  | T ss\_pred |  | cchHHHccccChhHHHHHHHHHHHHHHHHHHHHHHHHhccchhHHHHHHHHHHHHHHHH |
|  |
| --- | | | |
|  | Template alignmentCDD | | |
| 29. | PF01098.20 ; FTSW\_RODA\_SPOVE ; Cell cycle protein | | |
|  | Probability: 22.02%, E-value: 210, Score: 22.44, Aligned cols: 249, Identities: 8%, Similarity: -0.015, | | |
|  |
|  | Q ss\_pred |  | HHHHHHHHHHHHHHHHHHHHHhcCCHHHHHHHHHHHHCcccHH--------------HHHHhhccHHHHHHHHHHHHHHH |
|  | Q Q6ZXV5 | 93 | HLLNMIFHAVVSVIFLKVCKLFLDNKSSVIASLLFAVHPIHTE--------------AVTGVVGRAELLSSIFFLAAFLS   158 (426) |
|  | Q Consensus | 93 | rl~~~l~~~~~~~~~y~l~~~~~~~~~a~~aall~~~~p~~~~--------------~~~~~~~~~~~~~~~~~~l~~~~   158 (426) |
|  |  |  | ++...+.++.....++++-.+...+..-.+..+.....-.... .....+-++.-....+..+.+-. |
|  | T Consensus | 40 | q~~~~~iG~~~~~~~~~~dy~~l~~~~~~~~~~~~~ll~~~~~~~~g~~~~G~~~wi~lg~~~~qp~el~~~~~il~~a~   119 (357) |
|  | T PF01098.20 | 40 | QLLFAGIGVIAMFFIMNVDYWTWRTWSKLLMVICFFLLVLVLIPGVGMVRNGSRSWIGVGAFSIQPSEFMKLAMIAFLAK   119 (357) |
|  | T ss\_pred |  | HHHHHHHHHHHHHHHHcCCHHHHHHHHHHHHHHHHHHHHHHHccccccccCccCCeEEcCCcccCHHHHHHHHHHHHHHH |
|  |
|  |
|  | Q ss\_pred |  | HHHc----CCCCCcccHHHHHHHHHHHHHHHHhHhHHHHHHHHHHHHHHHHhcCCCccchhcccchhhcCCCCCChHHHH |
|  | Q Q6ZXV5 | 159 | YTRS----KGPDNSIIWTPIALTVFLVAVATLCKEQGITVVGICCVYEVFIAQGYTLPLLCTTAGQFLRGKGSIPFSMLQ   234 (426) |
|  | Q Consensus | 159 | ~~~~----~~~~~~~~~~~~~~~~~~~~la~~~k~~~~~~~~~~~~~~~~~~~~~~~~~~~~~~~~~~~~~~~~~~~~~~   234 (426) |
|  |  |  | +... ++++++..........+...+....+..+-.+.............+ .+.+. |
|  | T Consensus | 120 | ~~~~~~~~~~~~~~~~~~~~~~~~~~~~l~~~~~d~g~~~i~~~~~~~~l~~~~---------------------~~~~~   178 (357) |
|  | T PF01098.20 | 120 | FLSEKQKNITSFRRGFVPALGIVFSAFLIIMCQPDLGTGTVMVGTCIVMIFVAG---------------------ARIAH   178 (357) |
|  | T ss\_pred |  | HHHHhHhhcccchhccHHHHHHHHHHHHHHHhCCCHHHHHHHHHHHHHHHHHcC---------------------CCHHH |
|  |
|  |
|  | Q ss\_pred |  | HHHHHHHHHHHHHHHHHHHHHHHccCCCccccCCCcccCCCchhhHHhHhhHHHHHHHHHhcccc--ccccCccccCccc |
|  | Q Q6ZXV5 | 235 | TLVKLIVLMFSTLLLVVIRVQVIQSQLPVFTRFDNPAAVSPTPTRQLTFNYLLPVNAWLLLNPSE--LCCDWTMGTIPLI   312 (426) |
|  | Q Consensus | 235 | ~~~~~~~~~~~~~~~~~~~~~~~~~~~~~~~~~~~~~~~~~~~~~~~~~~~~~~~~~~~~~~~~~--~~~~~~~~~~~~~   312 (426) |
|  |  |  | .........................-....+...++.+......+..+........-........ .......+++... |
|  | T Consensus | 179 | ~~~~~~~~~~~~~~~~~~~~~~~~Ri~~~~~p~~~~~~~gyq~~~~~~~i~~~g~~G~G~~~~~~~~~~lp~~~~D~i~~   258 (357) |
|  | T PF01098.20 | 179 | FVFLGLIGLSGFVGLVLSAPYRIKRITSYLNPWEDPLGSGFQIIQSLYAVGPGGLFGMGLGQSRQKFFYLPEPQTDFIFA   258 (357) |
|  | T ss\_pred |  | HHHHHHHHHHHHHHHHHCchHHHHHHHHHhCCCCCCCCCCHHHHHHHHHHHcCCccccCCCCCccccccCCcccCchHHH |
|  |
|  |
|  | Q ss\_pred |  | cccccHHHHHHHHHHHHHHHHHHHHHHccCCCchhHHHHHHHHHHHHHHH |
|  | Q Q6ZXV5 | 313 | ESLLDIRNLATFTFFCFLGMLGVFSIRYSGDSSKTVLMALCLMALPFIPA   362 (426) |
|  | Q Consensus | 313 | ~~~~~~~~~~~~~~~~~~~~~~~~~~~~~~~~~~~~~~~~~~~~~~~~~~   362 (426) |
|  |  |  | .-..+.........+.+.........+..++.+....-.+.......+.. |
|  | T Consensus | 259 | ~i~~~~G~ig~~~~i~~~~~l~~~~~~~~~~~~~~~~~~l~~g~~~~l~~   308 (357) |
|  | T PF01098.20 | 259 | ILSEELGFIGGTLILLLFSVLLWRGIRIALGAPDLYGSFVAVGIISMIAI   308 (357) |
|  | T ss\_pred |  | HHHHHccHHHHHHHHHHHHHHHHHHHHHHHcCCCHHHHHHHHHHHHHHHH |
|  |
| --- | | | |
|  | Template alignmentCDD | | |
| 30. | PF12273.9 ; RCR ; Chitin synthesis regulation, resistance to Congo red | | |
|  | Probability: 21.8%, E-value: 34, Score: 23.05, Aligned cols: 29, Identities: 10%, Similarity: 0.096, | | |
|  |
|  | Q ss\_pred |  | hhchHHHHHHHHHHHHHHHHhcccchHHH |
|  | Q Q6ZXV5 | 376 | RVLYVPSMGFCILVAHGWQKISTKSVFKK   404 (426) |
|  | Q Consensus | 376 | ry~~~~~~~~~il~~~~~~~~~~~~~~~~   404 (426) |
|  |  |  | |+.+..+..+++++.+++.....++++++ |
|  | T Consensus | 1 | RWvl~~iii~~~l~~~~~~~~~~RRRrr~   29 (138) |
|  | T PF12273.9 | 1 | RWVVLAGVIVIVLVIFMLCTCTARRRRRR   29 (138) |
|  | T ss\_pred |  | CHHHHHHHHHHHHHHHHHHHHHHHHHHHc |
|  |
| --- | | | |
|  | Template alignmentCDD | | |
| 31. | PF10958.9 ; DUF2759 ; Protein of unknown function (DUF2759) | | |
|  | Probability: 20.12%, E-value: 78, Score: 16.74, Aligned cols: 33, Identities: 9%, Similarity: 0.154, | | |
|  |
|  | Q ss\_pred |  | HHHHHHHHHHHHHHcCCCCCcccHHHHHHHHHHHHHHHHh |
|  | Q Q6ZXV5 | 148 | SSIFFLAAFLSYTRSKGPDNSIIWTPIALTVFLVAVATLC   187 (426) |
|  | Q Consensus | 148 | ~~~~~~l~~~~~~~~~~~~~~~~~~~~~~~~~~~~la~~~   187 (426) |
|  |  |  | ..+..+++.+...|..++|+ ..+..+.+.+.+. |
|  | T Consensus | 2 | ~~lvtil~~~g~~r~~k~KN-------~fai~F~~~s~lV   34 (50) |
|  | T PF10958.9 | 2 | FSLVSILSLIGFITAAKNKN-------ILGFIFGIISFAL   34 (50) |
|  | T ss\_pred |  | HHHHHHHHHHHHHHHHHhhh-------HHHHHHHHHHHHH |
|  |

---

If you use HHpred on our Toolkit for your research, please cite as appropriate:

A Completely Reimplemented MPI Bioinformatics Toolkit
with a New HHpred Server at its Core.  
Zimmermann L, Stephens A, Nam SZ, Rau D,
Kübler J, Lozajic M, Gabler F, Söding J, Lupas AN, Alva V.
J Mol Biol. 2018 Jul 20. S0022-2836(17)30587-9.

  

Protein homology detection by HMM-HMM comparison.  
Söding J. Bioinformatics. 2005 Apr 1;21(7):951-60.  
  
Fast and accurate automatic structure prediction with HHpred.  
Hildebrand A, Remmert M, Biegert A, Söding J. Proteins. 2009;77 Suppl 9:128-32.  
  
Automatic Prediction of Protein 3D Structures by Probabilistic Multi-template Homology Modeling.  
Meier A, Söding J. PLoS Comput Biol. 2015 Oct 23;11(10):e1004343.

Download

---

If you use HHpred on our Toolkit for your research, please cite as appropriate:

A Completely Reimplemented MPI Bioinformatics Toolkit
with a New HHpred Server at its Core.  
Zimmermann L, Stephens A, Nam SZ, Rau D,
Kübler J, Lozajic M, Gabler F, Söding J, Lupas AN, Alva V.
J Mol Biol. 2018 Jul 20. S0022-2836(17)30587-9.

  

Protein homology detection by HMM-HMM comparison.  
Söding J. Bioinformatics. 2005 Apr 1;21(7):951-60.  
  
Fast and accurate automatic structure prediction with HHpred.  
Hildebrand A, Remmert M, Biegert A, Söding J. Proteins. 2009;77 Suppl 9:128-32.  
  
Automatic Prediction of Protein 3D Structures by Probabilistic Multi-template Homology Modeling.  
Meier A, Söding J. PLoS Comput Biol. 2015 Oct 23;11(10):e1004343.

Loading...

---

If you use HHpred on our Toolkit for your research, please cite as appropriate:

A Completely Reimplemented MPI Bioinformatics Toolkit
with a New HHpred Server at its Core.  
Zimmermann L, Stephens A, Nam SZ, Rau D,
Kübler J, Lozajic M, Gabler F, Söding J, Lupas AN, Alva V.
J Mol Biol. 2018 Jul 20. S0022-2836(17)30587-9.

  

Protein homology detection by HMM-HMM comparison.  
Söding J. Bioinformatics. 2005 Apr 1;21(7):951-60.  
  
Fast and accurate automatic structure prediction with HHpred.  
Hildebrand A, Remmert M, Biegert A, Söding J. Proteins. 2009;77 Suppl 9:128-32.  
  
Automatic Prediction of Protein 3D Structures by Probabilistic Multi-template Homology Modeling.  
Meier A, Söding J. PLoS Comput Biol. 2015 Oct 23;11(10):e1004343.

Loading hits...

---

If you use HHpred on our Toolkit for your research, please cite as appropriate:

A Completely Reimplemented MPI Bioinformatics Toolkit
with a New HHpred Server at its Core.  
Zimmermann L, Stephens A, Nam SZ, Rau D,
Kübler J, Lozajic M, Gabler F, Söding J, Lupas AN, Alva V.
J Mol Biol. 2018 Jul 20. S0022-2836(17)30587-9.

  

Protein homology detection by HMM-HMM comparison.  
Söding J. Bioinformatics. 2005 Apr 1;21(7):951-60.  
  
Fast and accurate automatic structure prediction with HHpred.  
Hildebrand A, Remmert M, Biegert A, Söding J. Proteins. 2009;77 Suppl 9:128-32.  
  
Automatic Prediction of Protein 3D Structures by Probabilistic Multi-template Homology Modeling.  
Meier A, Söding J. PLoS Comput Biol. 2015 Oct 23;11(10):e1004343.

Loading hits...

---

If you use HHpred on our Toolkit for your research, please cite as appropriate:

A Completely Reimplemented MPI Bioinformatics Toolkit
with a New HHpred Server at its Core.  
Zimmermann L, Stephens A, Nam SZ, Rau D,
Kübler J, Lozajic M, Gabler F, Söding J, Lupas AN, Alva V.
J Mol Biol. 2018 Jul 20. S0022-2836(17)30587-9.

  

Protein homology detection by HMM-HMM comparison.  
Söding J. Bioinformatics. 2005 Apr 1;21(7):951-60.  
  
Fast and accurate automatic structure prediction with HHpred.  
Hildebrand A, Remmert M, Biegert A, Söding J. Proteins. 2009;77 Suppl 9:128-32.  
  
Automatic Prediction of Protein 3D Structures by Probabilistic Multi-template Homology Modeling.  
Meier A, Söding J. PLoS Comput Biol. 2015 Oct 23;11(10):e1004343.

- Help
- FAQ
- Privacy Policy
- Imprint
- Contact Us
- Cite Us
- Recent Updates

© 2008-2020, Dept. of Protein Evolution, Max Planck Institute for Developmental Biology, Tübingen

Template 3D Structure: 
×

Loading...
