# Supplementary material for: Conserved sequence motifs in human TMTC1, TMTC2, TMTC3, and TMTC4, new O-mannosyltransferases from the GT-C/PMT clan, are rationalized as ligand binding sites
Source: Biol Direct. 2021 Jan 12;16:4. doi: 10.1186/s13062-021-00291-w (PMC7801869; doi:10.1186/s13062-021-00291-w)
Supplement: Supplementary file 3 — Additional file 3. HHPred outputs when searching TMTCs against Pfam or PDB structures. The compressed library file AF3-2020-06-HHPred-TMTCs.zip contains the outputs when running the four human TMTC sequences as input of HHPred against PDB sequences and against Pfam domains (as of 23rd of June 2020). [file 13062_2021_291_MOESM3_ESM.zip › AF3-2020-06-HHPred-TMTCs/HHpred_TMTC4_PDB.html]

(\*) HHpred | Bioinformatics Toolkit          **We're sorry but the Toolkit doesn't work properly without JavaScript enabled. Please enable it to continue.**

Sign In

- Search
- Alignment
- Sequence Analysis
- 2ary Structure
- 3ary Structure
- Classification
- Utils

- HHblits
- HHpred
- HMMER
- PatternSearch
- ProtBLAST/PSI-BLAST

Nothing found.

###### Tools

###### Jobs

ID

Date

Tool

8527802HHPR8776670HHPR3084060HHPR1780993HHPR5863267HHPR5407837HHPR8665047HHPR2161064HHPR

# HHpred

Job ID: 8527802,Created: 31 minutes ago

- Input
- Parameters
- Results
- Raw Output
- Probability Plot
- Query Template MSA
- Query MSA

>sp|Q5T4D3|TMTC4\_HUMAN 1..462
MAVLDTDLDHILPSSVLPPFWAKLVVGSVAIVCFARSYDGDFVFDDSEAIVNNKDLQAET
PLGDLWHHDFWGSRLSSNTSHKSYRPLTVLTFRINYYLSGGFHPVGFHVVNILLHSGISV
LMVDVFSVLFGGLQYTSKGRRLHLAPRASLLAALLFAVHPVHTECVAGVVGRADLLCALF
FLLSFLGYCKAFRESNKEGAHSSTFWVLLSIFLGAVAMLCKEQGITVLGLNAVFDILVIG
KFNVLEIVQKVLHKDKSLENLGMLRNGGLLFRMTLLTSGGAGMLYVRWRIMGTGPPAFTE
VDNPASFADSMLVRAVNYNYYYSLNAWLLLCPWWLCFDWSMGCIPLIKSISDWRVIALAA
LWFCLIGLICQALCSEDGHKRRILTLGLGFLVIPFLPASNLFFRVGFVVAERVLYLPSVG
YCVLLTFGFGALSKHTKKKKLIAAVVLGILFINTLRCVLRSG

Paste ExampleUpload File

Protein FASTA

Align two sequences/MSAs

Select structural/domain databases

PDB\_mmCIF70\_29\_May

- PDB\_mmCIF70\_29\_May (default)
- PDB\_mmCIF30\_29\_May
- SCOPe70\_2.07
- ECOD\_ECOD\_F70\_20200207
- COG\_KOG\_v1.0
- Pfam-A\_v33.1
- NCBI\_Conserved\_Domains(CD)\_v3.18
- SMART\_v6.0
- TIGRFAMs\_v15.0
- PRK\_v6.9
- No elements found. Consider changing the search query.
- List is empty.

Select proteomes

Select options

- Euk\_Arabidopsis\_thaliana\_TAIR10\_20\_Jun\_2017
- Euk\_Bombyx\_mori\_p50T\_Dazao\_06\_May\_2019
- Euk\_Brachypodium\_distachyon\_23\_Aug\_2017
- Euk\_Caenorhabditis\_elegans\_18\_Jul\_2017
- Euk\_Capsaspora\_owczarzaki\_ATCC\_30864\_23\_Mar\_2020
- Euk\_Chaetomium\_thermophilum\_29\_Jun\_2017
- Euk\_Chlamydomonas\_reinhardtii\_27\_Jul\_2017
- Euk\_Entamoeba\_histolytica\_HM1\_IMSS\_22\_Mar\_2017
- Euk\_Dictyostelium\_discoideum\_AX4\_19\_Sep\_2017
- Euk\_Drosophila\_melanogaster\_19\_Jul\_2017
- Euk\_Giardia\_lamblia\_ATCC\_50803\_31\_Aug\_2017
- Euk\_Homo\_sapiens\_04\_Jul\_2017
- Euk\_Physcomitrella\_patens\_28\_Aug\_2017
- Euk\_Plasmodium\_falciparum\_3D7\_7\_Jun\_2017
- Euk\_Saccharomyces\_cerevisiae\_S288c\_11\_Mar\_2017
- Euk\_Schizosaccharomyces\_pombe\_19\_Sep\_2017
- Euk\_Solanum\_lycopersicum\_28\_Jul\_2019
- Euk\_Tetrahymena\_thermophila\_SB210\_22\_Aug\_2017
- Euk\_Toxoplasma\_gondii\_ME49\_10\_May\_2018
- Euk\_Trichomonas\_vaginalis\_G3\_21\_Nov\_2018
- Euk\_Trypanosoma\_brucei\_gambiense\_DAL972\_28\_Mar\_2017
- Euk\_Ustilago\_maydis\_521\_29\_May\_2017
- Euk\_Paramecium\_tetraurelia\_9\_Dec\_2018
- Arc\_Archaeoglobus\_fulgidus\_DSM\_4304\_5\_Dec\_2017
- Arc\_Halobacterium\_jilantaiense\_5\_Dec\_2017
- Arc\_Lokiarchaeum\_sp\_GC14\_75\_31\_Oct\_2018
- Arc\_Methanocaldococcus\_jannaschii\_DSM\_2661\_5\_Dec\_2017
- Arc\_Methanosarcina\_mazei\_S\_6\_17\_Mar\_2017
- Arc\_Methanothermus\_fervidus\_DSM\_2088\_5\_Dec\_2017
- Arc\_Pyrococcus\_horikoshii\_OT3\_5\_Dec\_2017
- Arc\_Sulfolobus\_solfataricus\_5\_Dec\_2017
- Arc\_Thermoplasma\_acidophilum\_DSM\_1728\_7\_Dec\_2017
- Bac\_Acinetobacter\_baumannii\_29\_Mar\_2018
- Bac\_Aquifex\_aeolicus\_VF5\_19\_Sep\_2017
- Bac\_Bacillus\_subtilis\_subsp\_subtilis\_str168\_19\_Mar\_2017
- Bac\_Bacteriovorax\_sp\_DB6\_IX\_1\_Jun\_2018
- Bac\_Bdellovibrio\_bacteriovorus\_HD100\_1\_Jun\_2018
- Bac\_Christensenella\_minuta\_2\_Apr\_2019
- Bac\_Deinococcus\_radiodurans\_R1\_19\_Sep\_2017
- Bac\_Enterococcus\_faecalis\_13\_SD\_W\_01\_1\_Jun\_2018
- Bac\_Escherichia\_coli\_K12\_07\_Mar\_2017
- Bac\_Fischerella\_muscicola\_PCC\_7414\_24\_Sep\_2017
- Bac\_Frankia\_alni\_ACN14a\_24\_Sep\_2017
- Bac\_Helicobacter\_pylori\_26695\_1\_Jun\_2018
- Bac\_Leptospira\_interrogans\_serovar\_Lai\_str56601\_1\_Jun\_2018
- Bac\_Mycobacterium\_tuberculosis\_H37Rv\_27\_May\_2017
- Bac\_Neisseria\_gonorrhoeae\_FA\_1090\_1\_Jun\_2018
- Bac\_Neisseria\_meningitidis\_MC58\_9\_Jun\_2017
- Bac\_Nostoc\_punctiforme\_PCC\_73102\_18\_Mar\_2017
- Bac\_Phycisphaerae\_bacterium\_L21\_RPulD3\_1\_Jun\_2018
- Bac\_Plesiocystis\_pacifica\_SIR1\_1\_Jun\_2018
- Bac\_Pseudomonas\_aeruginosa\_PAO1\_5\_Jun\_2017
- Bac\_Salmonella\_ent\_ser\_Typhi\_CT18\_22\_Nov\_2018
- Bac\_Staphylococcus\_aureus\_subsp\_aureus\_NCTC\_8325\_13\_Jun\_2017
- Bac\_Streptomyces\_scabiei\_87.22\_24\_Sep\_2017
- Bac\_Synechocystis\_sp\_PCC\_6803\_6\_Jun\_2017
- Bac\_Tenacibaculum\_dicentrarchi\_27\_Nov\_2017
- Bac\_Tenacibaculum\_maritimum\_NBRC\_15946\_27\_Nov\_2017
- Bac\_Thermus\_aquaticus\_Y51MC23\_24\_Sep\_2017
- Bac\_Thermus\_thermophilus\_HB8\_19\_Sep\_2017
- Bac\_Waddlia\_chondrophila\_WSU\_86\_1044\_1\_Jun\_2018
- Bac\_Yersinia\_pestis\_CO92\_10\_Apr\_2017
- Vir\_SARS-CoV-2\_31\_Mar\_2020
- No elements found. Consider changing the search query.
- List is empty.

Resubmit

MSA generation method

HHblits=>UniRef30

- HHblits=>UniRef30 (default)
- PSI-BLAST=>nr70
- No elements found. Consider changing the search query.
- List is empty.

Maximal no. of MSA generation steps

3

- 0
- 1
- 2
- 3 (default)
- 4
- 5
- 8
- No elements found. Consider changing the search query.
- List is empty.

E-value incl. threshold for MSA generation

1e-3

- 0.1
- 0.05
- 0.02
- 0.01
- 1e-3 (default)
- 1e-6
- 1e-8
- 1e-10
- 1e-15
- 1e-20
- 1e-30
- 1e-40
- 1e-50
- No elements found. Consider changing the search query.
- List is empty.

Min. seq. identity of MSA hits with query (%)

0

- 0 (default)
- 10
- 20
- 30
- 40
- 50
- 60
- 70
- 75
- 80
- 85
- 90
- 95
- 100
- No elements found. Consider changing the search query.
- List is empty.

Min. coverage of MSA hits (%)

20

- 10
- 20 (default)
- 30
- 40
- 50
- 60
- 70
- 80
- 90
- 100
- No elements found. Consider changing the search query.
- List is empty.

Secondary structure scoring

during\_alignment

- none
- after\_alignment
- during\_alignment (default)
- after\_alignment\_pred\_vs\_pred
- during\_alignment\_pred\_vs\_pred
- No elements found. Consider changing the search query.
- List is empty.

Alignment Mode:Realign with MAC

local:norealign

- local:norealign (default)
- local:realign
- global:realign
- No elements found. Consider changing the search query.
- List is empty.

MAC realignment threshold

0.3

- 0.0
- 0.01
- 0.1
- 0.2
- 0.3 (default)
- 0.4
- 0.5
- 0.6
- 0.7
- 0.8
- 0.9
- 0.95
- No elements found. Consider changing the search query.
- List is empty.

No. of target sequences (up to 10000)

250

- 250 (default)
- 500
- 1000
- 2000
- 3000
- 4000
- 5000
- 6000
- 7000
- 8000
- 9000
- 10000
- No elements found. Consider changing the search query.
- List is empty.

Min. probability in hit list (> 10%)

20

- 10
- 20 (default)
- 30
- 40
- 50
- 60
- 70
- 75
- 80
- 85
- 90
- 95
- 100
- No elements found. Consider changing the search query.
- List is empty.

Resubmit

VisHitsAln
Select AllForwardForward Query A3MModel using selectionDownload HHRColor SeqsWrap Seqs

Number of Hits: **17**

Detected sequence features:
**◾Transmembrane segment(s)**

#### Visualization

Resubmit Section

1

460

Prob=99.8%
E=1.7E-16 6EZN\_F Dolichyl-diphosphooligosaccharide--protein
glycosyltransferase subunit 1 (E.C.2.4.99.18); OST complex,
oligosaccharyltransferase, N-linked glycosylation; HET: PTY, BMA, CPL,
MAN, NAG;{Saccharomyces cerevisiae (strain ATCC 204508 / S288c)};
Related PDB entries: 6C26\_A; Related PDB entries: 6C26\_A; Related PDB
entries: 6C26\_A

#### Hitlist

Show102550100AllEntries

Search:

| Nr (Click to sort Ascending) | Hit (Click to sort Ascending) | Name (Click to sort Ascending) | Probability (Click to sort Ascending) | E-value (Click to sort Ascending) | SS (Click to sort Ascending) | Cols (Click to sort Ascending) | Target Length (Click to sort Ascending) |
| --- | --- | --- | --- | --- | --- | --- | --- |
| 1 | 5EZM\_A | 4-amino-4-deoxy-L-arabinose (L-Ara4N) transferase; membrane protein, lipid glycosyltransferase, zinc; HET: PC, DSL, PO4, MPG, EPE; 2.7A {Cupriavidus metallidurans (strain ATCC 43123 / DSM 2839 / NBRC 102507 / CH34)}; Related PDB entries: 5F15\_A ; Related PDB entries: 5F15\_A ; Related PDB entries: 5F15\_A | 99.91 | 4.2e-21 | 33.2 | 377 | 578 |
| 2 | 6S7O\_A | Dolichyl-diphosphooligosaccharide--protein glycosyltransferase subunit STT3A (E.C.2.4.99.18); N-glycosylation, Oligosaccharyltransferase, OSTA, TRANSFERASE; HET: KZB, NAG, EGY, MAN, KZE, BMA; 3.5A {Homo sapiens}; Related PDB entries: 6FTI\_5 6FTG\_5 6FTJ\_5; Related PDB entries: 6FTG\_5 6FTI\_5 6FTJ\_5; Related PDB entries: 6FTG\_5 6FTI\_5 6FTJ\_5 | 99.83 | 4.1e-17 | 35.7 | 388 | 705 |
| 3 | 6S7T\_A | Dolichyl-diphosphooligosaccharide--protein glycosyltransferase subunit STT3B (E.C.2.4.99.18); N-glycosylation, Oligosaccharyltransferase, OSTB, TRANSFERASE; HET: 0K3, KZB, NAG, EGY, MAN, BMA; 3.5A {Homo sapiens} | 99.82 | 1.2e-16 | 36.1 | 388 | 826 |
| 4 | 6EZN\_F | Dolichyl-diphosphooligosaccharide--protein glycosyltransferase subunit 1 (E.C.2.4.99.18); OST complex, oligosaccharyltransferase, N-linked glycosylation; HET: PTY, BMA, CPL, MAN, NAG;{Saccharomyces cerevisiae (strain ATCC 204508 / S288c)}; Related PDB entries: 6C26\_A; Related PDB entries: 6C26\_A; Related PDB entries: 6C26\_A | 99.82 | 1.7e-16 | 36 | 405 | 718 |
| 5 | 3WAJ\_A | Transmembrane oligosaccharyl transferase (E.C.2.4.1.119); oligosaccharyltransferase, N-glycosylation, Archaeoglobus fulgidus, GT-C; 2.501A {Archaeoglobus fulgidus}; Related PDB entries: 5GMY\_A 3WAK\_A; Related PDB entries: 5GMY\_A 3WAK\_A; Related PDB entries: 5GMY\_A 3WAK\_A | 99.8 | 1e-15 | 37.3 | 391 | 875 |
| 6 | 5OGL\_A | Peptide-binding protein, Substrate mimicking peptide; Oligosaccharyltransferase, Complex, Protein N-glycosylation, Bacteria; HET: 9UB, PPN; 2.7A {Campylobacter lari (strain RM2100 / D67 / ATCC BAA-1060)}; Related PDB entries: 3RCE\_A 6GXC\_A; Related PDB entries: 6GXC\_A 3RCE\_A ; Related PDB entries: 6GXC\_A 3RCE\_A | 99.76 | 1.1e-15 | 29.6 | 373 | 713 |
| 7 | 6P25\_A | Dolichyl-diphosphooligosaccharide--protein glycosyltransferase subunits (E.C.2.4.99.18); complex, TRANSFERASE, glycosylation; HET: NAG, CPL, NNM; 3.2A {Saccharomyces cerevisiae W303}; Related PDB entries: 6P2R\_A ; Related PDB entries: 6P2R\_A ; Related PDB entries: 6P2R\_A | 99.64 | 1.8e-13 | 26.6 | 249 | 817 |
| 8 | 6P25\_B | Dolichyl-diphosphooligosaccharide--protein glycosyltransferase subunits (E.C.2.4.99.18); complex, TRANSFERASE, glycosylation; HET: NAG, CPL, NNM; 3.2A {Saccharomyces cerevisiae W303}; Related PDB entries: 6P2R\_B ; Related PDB entries: 6P2R\_B ; Related PDB entries: 6P2R\_B | 99.64 | 1.7e-13 | 26.5 | 266 | 759 |
| 9 | 7BVF\_A | Probable arabinosyltransferase B (E.C.2.4.2.-), Probable; Mycobacterium tuberculosis, cell wall synthesis; HET: 95E, DSL, CDL;{Mycolicibacterium smegmatis MC2 155} | 99.59 | 2.2e-11 | 35.7 | 363 | 1102 |
| 10 | 6W98\_A | F5/8 type C domain-containing protein; Glycosyltransferase, lipomannan, lipoarabinomannan, arabinofuranose, membrane; HET: PNS, 6OU; 2.9A {Escherichia coli (strain K12)}; Related PDB entries: 6WBX\_A 6WBY\_A | 99.53 | 4.1e-11 | 33.6 | 394 | 1413 |
| 11 | 7BVF\_B | Probable arabinosyltransferase B (E.C.2.4.2.-), Probable; Mycobacterium tuberculosis, cell wall synthesis; HET: 95E, DSL, CDL;{Mycolicibacterium smegmatis MC2 155} | 99.52 | 8.6e-11 | 32.9 | 364 | 1116 |
| 12 | 7BWR\_A | Integral membrane indolylacetylinositol arabinosyltransferase EmbB; Mycobacterium tuberculosis, EmbB, cryo-EM, ethambutol; HET: F8L;{Mycolicibacterium smegmatis MC2 155}; Related PDB entries: 7BVC\_B 7BVG\_B 7BWR\_B 7BX8\_B 7BX8\_A | 99.51 | 8e-11 | 31.5 | 358 | 1082 |
| 13 | 7BVE\_B | Integral membrane indolylacetylinositol arabinosyltransferase EmbC; Mycobacterium smegmatis, cell wall synthesis; HET: PO4, PN7, 95E; 2.81A {Mycolicibacterium smegmatis MC2 155}; Related PDB entries: 7BVH\_B 7BVH\_A 7BVE\_A | 99.47 | 4.4e-10 | 33.2 | 356 | 1084 |
| 14 | 6SNI\_X | Dolichyl pyrophosphate Man9GlcNAc2 alpha-1,3-glucosyltransferase (E.C.2.4.1.267); Glycosyltransferase, Glucosyltransferase, GT-C, N-Glycosylation, MEMBRANE; HET: PTY, Y01;{Saccharomyces cerevisiae}; Related PDB entries: 6SNH\_X | 99.41 | 1.2e-9 | 31.3 | 339 | 562 |
| 15 | 7BVC\_A | Integral membrane indolylacetylinositol arabinosyltransferase EmbA; Mycobacterium smegmatis, cell wall synthesis; HET: 95E, PNS, CDL, F8L;{Mycolicibacterium smegmatis MC2 155}; Related PDB entries: 7BVG\_A | 99.4 | 1.3e-9 | 31.3 | 354 | 1088 |
| 16 | 6P25\_B | Dolichyl-diphosphooligosaccharide--protein glycosyltransferase subunits (E.C.2.4.99.18); complex, TRANSFERASE, glycosylation; HET: NAG, CPL, NNM; 3.2A {Saccharomyces cerevisiae W303}; Related PDB entries: 6P2R\_B ; Related PDB entries: 6P2R\_B ; Related PDB entries: 6P2R\_B | 65.59 | 81 | 11 | 104 | 759 |
| 17 | 6P25\_A | Dolichyl-diphosphooligosaccharide--protein glycosyltransferase subunits (E.C.2.4.99.18); complex, TRANSFERASE, glycosylation; HET: NAG, CPL, NNM; 3.2A {Saccharomyces cerevisiae W303}; Related PDB entries: 6P2R\_A ; Related PDB entries: 6P2R\_A ; Related PDB entries: 6P2R\_A | 42.61 | 460 | 12.1 | 104 | 817 |

Displaying 1 to 17 of 17 hits

- «
- ‹
- 1
- ›
- »

#### Alignments

|  |  |  |  |
| --- | --- | --- | --- |
|  | | | |
|  | Template alignmentTemplate 3D StructurePDBe | | |
| 1. | 5EZM\_A 4-amino-4-deoxy-L-arabinose (L-Ara4N) transferase; membrane protein, lipid glycosyltransferase, zinc; HET: PC, DSL, PO4, MPG, EPE; 2.7A {Cupriavidus metallidurans (strain ATCC 43123 / DSM 2839 / NBRC 102507 / CH34)}; Related PDB entries: 5F15\_A ; Related PDB entries: 5F15\_A ; Related PDB entries: 5F15\_A | | |
|  | Probability: 99.91%, E-value: 4.2e-21, Score: 185.34, Aligned cols: 377, Identities: 12%, Similarity: -0.028, | | |
|  |
|  | Q ss\_pred |  | CCcccCChhhcccccCCHHHHHHHHHHHHHHHHHhhhcCCCcc--cCchHHHHhcccccCCCChhhhhcccccccccCCC |
|  | Q Q5T4D3 | 1 | MAVLDTDLDHILPSSVLPPFWAKLVVGSVAIVCFARSYDGDFV--FDDSEAIVNNKDLQAETPLGDLWHHDFWGSRLSSN   78 (462) |
|  | Q Consensus | 1 | ~~~~~~~~~~~~~~~~~~~~~~~~~l~~~~~~~~~~~~~~~~~--~Dd~~~~~~~~~~~~~~~~~~~~~~~~~~~~~~~~   78 (462) |
|  |  |  | +..+.+.................+++++++...+......... +||..+...+.++.+++.+.....++ |
|  | T Consensus | 12 | ~~~~~~~~~~~~~~~~~~~~~~~~~~~~~~~~~~~~~~~~~~~~~~De~~~~~~a~~~~~~~~~~~~~~~~---------   82 (578) |
|  | T 5EZM\_A | 12 | RASVASSQSTQGAVGWSAATGWVVLFVAVALVVWFVSLDMRHLVGPDEGRYAEISREMFASGDWVTIRYNA---------   82 (578) |
|  | T ss\_dssp |  | ----------------CCTHHHHHHHHHHHHHHHHHGGGSSCCCTTHHHHHHHHHHHHHHHCCSSSCEETT--------- |
|  | T ss\_pred |  | ccccccCCCccchhhHHHHHHHHHHHHHHHHHHHHhccccccCCCCCHHHHHHHHHHHHHhCCceeEEECC--------- |
|  |
|  |
|  | Q ss\_pred |  | CCccccCchHHHHHHHHHHHhCCCCchHHHHHHHHHHHHHHHHHHHHHHHHhccccccccccccchHHHHHHHHHHHHHH |
|  | Q Q5T4D3 | 79 | TSHKSYRPLTVLTFRINYYLSGGFHPVGFHVVNILLHSGISVLMVDVFSVLFGGLQYTSKGRRLHLAPRASLLAALLFAV   158 (462) |
|  | Q Consensus | 79 | ~~~~~~~Pl~~~~~~~~~~~~gg~~~~~~rl~~~~~~~l~~~l~~~l~~~l~~~~~~~~~~~~~~~~~~~a~~aall~~~   158 (462) |
|  |  |  | ..+..+||++.++.+....++ |.++...|++++++++++++++|.++|+++ +++.|+++++++++ |
|  | T Consensus | 83 | ~~~~~~pPl~~~l~~~~~~l~-g~~~~~~rl~~~l~~~l~~~~~~~l~~~~~--------------~~~~a~~a~~l~~~   147 (578) |
|  | T 5EZM\_A | 83 | LKYFEKPPFHMWVTVVGYELF-GLGEWQARLAVALSGLLGIGVSMMAARRWF--------------GARAAAFTGLALLA   147 (578) |
|  | T ss\_dssp |  | EECCSSCSHHHHHHHHHHHHH-CSSHHHHTHHHHHHHHHHHHHHHHHHHHHH--------------CHHHHHHHHHHHHH |
|  | T ss\_pred |  | EeCCCCCHHHHHHHHHHHHHH-CcCHHHHHHHHHHHHHHHHHHHHHHHHHHh--------------ChHHHHHHHHHHHH |
|  |
|  |
|  | Q ss\_pred |  | CHHHHHHHHhHhcHHHHHHHHHHHHHHHHHHHHHHHc-----CCCCCchHHHHHHHHHHHHHHHHhchHHHHHHHHHHHH |
|  | Q Q5T4D3 | 159 | HPVHTECVAGVVGRADLLCALFFLLSFLGYCKAFRES-----NKEGAHSSTFWVLLSIFLGAVAMLCKEQGITVLGLNAV   233 (462) |
|  | Q Consensus | 159 | ~p~~~~~~~~~~~~~~~~~~~f~ll~~~~~~~~~~~~-----~~~~~~~~~~~~~~~~~~~~la~l~k~~~~~~~~~~~~   233 (462) |
|  |  |  | +|.....+... ++|.+..++.+++++++.+..+++ + .++..+++++.+++.++|+....+.+...+ |
|  | T Consensus | 148 | ~p~~~~~~~~~--~~~~~~~~~~~~~~~~~~~~~~~~~~~~~~-------~~~~~l~g~~~gla~~~k~~~~~~~~~~~~   218 (578) |
|  | T 5EZM\_A | 148 | APMWSVAAHFN--TLDMTLAGVMSCVLAFMLMGQHPDASVAAR-------RGWMVACWAAMGVAILTKGLVGIALPGLVL   218 (578) |
|  | T ss\_dssp |  | CHHHHHHHTSC--CHHHHHHHHHHHHHHHHHHHTCTTSCHHHH-------HHHHHHHHHHHHHHHHHHTTHHHHHHHHHH |
|  | T ss\_pred |  | HHHHHHHHhhH--hhHHHHHHHHHHHHHHHHHHhCCCcchhhc-------cHHHHHHHHHHHHHHHccchHHHHHHHHHH |
|  |
|  |
|  | Q ss\_pred |  | HHHHHhCCCChHHHHHHHhhhccchHhhchhhhhhHHHHHHHHHHHHHHHHHHHHHHhCCCCCCccccCCcchhcchhhH |
|  | Q Q5T4D3 | 234 | FDILVIGKFNVLEIVQKVLHKDKSLENLGMLRNGGLLFRMTLLTSGGAGMLYVRWRIMGTGPPAFTEVDNPASFADSMLV   313 (462) |
|  | Q Consensus | 234 | ~~~~~~~~~~~~~~~~~~~~~~~~~~~~~~~~~~~~~~~~~~~~~~~~~~~~~~~~~~~~~~~~~~~~~~~~~~~~~~~~   313 (462) |
|  |  |  | ......++++++...+.. ........+....+.............................. |
|  | T Consensus | 219 | ~~~~~~~~~~~~~~~~~~------------------~~~~~~~~~~~~~~~~~~~~~~~~~~~~~~~~~~~~~~~~~~~~   280 (578) |
|  | T 5EZM\_A | 219 | VVYTLVTRDWGLWRRLHL------------------ALGVVVMLVITVPWFYLVSVRNPEFPNFFFIHEHWQRYTSNIHS   280 (578) |
|  | T ss\_dssp |  | HHHHHHSCCTTHHHHTCH------------------HHHHHHHHHHHHHHHHHHHHHCTTHHHHHHHCCCCCC------- |
|  | T ss\_pred |  | HHHHHHcCChHHHhhcch------------------HHHHHHHHHHHHHHHHHHHHHCCcchhhhHHHHHHHHHhcCccc |
|  |
|  |
|  | Q ss\_pred |  | hHhHHHHHHHHHHHHhhccHhhhccccccccCcccccchHHHHHHHHHHHHHHHHHHHHHhcCCCcchHHHHHHHHHHHH |
|  | Q Q5T4D3 | 314 | RAVNYNYYYSLNAWLLLCPWWLCFDWSMGCIPLIKSISDWRVIALAALWFCLIGLICQALCSEDGHKRRILTLGLGFLVI   393 (462) |
|  | Q Consensus | 314 | ~~~~~~~~~~~~~~~~~~p~~~~~~~~~~~~~~~~~~~~~~~~~~~~~~~~~~~~~~~~~~~~~~~~~~~~~~~~~~~~~   393 (462) |
|  |  |  | .......+. ............++..........+++++++.......+.+.+. |
|  | T Consensus | 281 | ~~~~~~~~~---------------------------~~~~~~~~~~~~~~~~~~~~~~~~~~~~~~~~~~~~~~~~~~~~   333 (578) |
|  | T 5EZM\_A | 281 | RSGSVFYFL---------------------------PLVIGGFLPWAGIFPKLWTAMRAPVEGTQARFRPALMAGIWAIA   333 (578) |
|  | T ss\_dssp |  | CCCCTTTHH---------------------------HHHHHHTGGGGGGHHHHHHHHCC-------CCCHHHHHHHHHHH |
|  | T ss\_pred |  | CCCChHHHH---------------------------HHHHHHhhhHHhHHHHHHHHhhcccccCcccccHHHHHHHHHHH |
|  |
|  |
|  | Q ss\_pred |  | HHhhHhccccccchhHHHhhhhHHHHHHHHHHHHHHHHHHhchhhHHHHHHHHHHHHHHHHHHHHHh |
|  | Q Q5T4D3 | 394 | PFLPASNLFFRVGFVVAERVLYLPSVGYCVLLTFGFGALSKHTKKKKLIAAVVLGILFINTLRCVLR   460 (462) |
|  | Q Consensus | 394 | ~~~~~~~~~~~~~~~~~~ry~~~~~~~~~ll~a~~~~~~~~~~~~~~~~~~~~~~~~~~~~~~~~~~   460 (462) |
|  |  |  | ..+..... ....+||+++..|+++++++.++.+..++.+.+......+++++.......... |
|  | T Consensus | 334 | ~~~~~~~~-----~~~~~ry~~~~~p~l~ll~~~~l~~~~~~~~~~~~~~~~~~~~~~~~~~~~~~~   395 (578) |
|  | T 5EZM\_A | 334 | IFVFFSIS-----RSKLPGYIVPVIPALGILAGVALDRLSPRSWGKQLIGMAIVAACGLLASPVVAT   395 (578) |
|  | T ss\_dssp |  | HHHHHHTS-----SSCCGGGGTTHHHHHHHHHHHHHHTCCHHHHHHHHHHHHHHHHHHHHHGGGGGG |
|  | T ss\_pred |  | HHHHHHHh-----cccChhcHHhHHHHHHHHHHHHHHHhCCCccHHHHHHHHHHHHHHHHHHHHHHH |
|  |
| --- | | | |
|  | Template alignmentTemplate 3D StructurePDBe | | |
| 2. | 6S7O\_A Dolichyl-diphosphooligosaccharide--protein glycosyltransferase subunit STT3A (E.C.2.4.99.18); N-glycosylation, Oligosaccharyltransferase, OSTA, TRANSFERASE; HET: KZB, NAG, EGY, MAN, KZE, BMA; 3.5A {Homo sapiens}; Related PDB entries: 6FTI\_5 6FTG\_5 6FTJ\_5; Related PDB entries: 6FTG\_5 6FTI\_5 6FTJ\_5; Related PDB entries: 6FTG\_5 6FTI\_5 6FTJ\_5 | | |
|  | Probability: 99.83%, E-value: 4.1e-17, Score: 161.56, Aligned cols: 388, Identities: 10%, Similarity: -0.087, | | |
|  |
|  | Q ss\_pred |  | CCcccCChhhcccccCCHHHHHHHHHHHHHHHHHhhh----cCCCcccCchHHHHhcccccCCCChhhhhcccccccccC |
|  | Q Q5T4D3 | 1 | MAVLDTDLDHILPSSVLPPFWAKLVVGSVAIVCFARS----YDGDFVFDDSEAIVNNKDLQAETPLGDLWHHDFWGSRLS   76 (462) |
|  | Q Consensus | 1 | ~~~~~~~~~~~~~~~~~~~~~~~~~l~~~~~~~~~~~----~~~~~~~Dd~~~~~~~~~~~~~~~~~~~~~~~~~~~~~~   76 (462) |
|  |  |  | |+++++......+.........+++++++++...... .......||..+...+.++.+++........+....... |
|  | T Consensus | 1 | m~~~~~~~~~~~~~~~~~~~~~l~~~~~~~~~~~~~~~~~~~~~~~~~D~~~~~~~a~~~~~~g~~~~~~~~~~~~~~~~   80 (705) |
|  | T 6S7O\_A | 1 | MTKFGFLRLSYEKQDTLLKLLILSMAAVLSFSTRLFAVLRFESVIHEFDPYFNYRTTRFLAEEGFYKFHNWFDDRAWYPL   80 (705) |
|  | T ss\_dssp |  | ------CCCCHHHHHHHHHHHHHHHHHHHHHHHHCSHHHHSCCCCCSSSHHHHHHHHHHHHHHCSHHHHSCEECSSSTTS |
|  | T ss\_pred |  | CCccccccCChHHHHHHHHHHHHHHHHHHHHHHHHHHHHhcccccccCChHHHHHHHHHHHHhCChhhhccccccccCCC |
|  |
|  |
|  | Q ss\_pred |  | CCCCccccCchHHHHHHHHHHHhCC------CCchHHHHHHHHHHHHHHHHHHHHHHHHhccccccccccccchHHHHHH |
|  | Q Q5T4D3 | 77 | SNTSHKSYRPLTVLTFRINYYLSGG------FHPVGFHVVNILLHSGISVLMVDVFSVLFGGLQYTSKGRRLHLAPRASL   150 (462) |
|  | Q Consensus | 77 | ~~~~~~~~~Pl~~~~~~~~~~~~gg------~~~~~~rl~~~~~~~l~~~l~~~l~~~l~~~~~~~~~~~~~~~~~~~a~   150 (462) |
|  |  |  | .......++|++.++.+..+.++ | ++....|+.++++++++++++|.++|++. +++.|+ |
|  | T Consensus | 81 | g~~~~~~~~p~~~~~~~~~~~l~-g~~g~~~~~~~~~~~~~~l~~~l~~~~~y~l~~~~~--------------~~~~al   145 (705) |
|  | T 6S7O\_A | 81 | GRIIGGTIYPGLMITSAAIYHVL-HFFHITIDIRNVCVFLAPLFSSFTTIVTYHLTKELK--------------DAGAGL   145 (705) |
|  | T ss\_dssp |  | CEEHHHHSCCHHHHHHHHHHHHH-HHTTCCCCHHHHHHTHHHHHHHHHHHHHHHHHHHHS--------------CHHHHH |
|  | T ss\_pred |  | cCCCCCcCCchHHHHHHHHHHHH-HHcCCCCCHHHHHHHHHHHHHHHHHHHHHHHHHHHc--------------CHHHHH |
|  |
|  |
|  | Q ss\_pred |  | HHHHHHHHCHHHHHHHHhHhcHHHHHHHHHHHHHHHHHHHHHHHcCCCCCchHHHHHHHHHHHHHHHHhchHHHHHHHHH |
|  | Q Q5T4D3 | 151 | LAALLFAVHPVHTECVAGVVGRADLLCALFFLLSFLGYCKAFRESNKEGAHSSTFWVLLSIFLGAVAMLCKEQGITVLGL   230 (462) |
|  | Q Consensus | 151 | ~aall~~~~p~~~~~~~~~~~~~~~~~~~f~ll~~~~~~~~~~~~~~~~~~~~~~~~~~~~~~~~la~l~k~~~~~~~~~   230 (462) |
|  |  |  | +++++++++|.+...+......+|.+..++.+++++++.+..++++ .++.++++++.+++.++|+.+....++ |
|  | T Consensus | 146 | ~aa~l~~~~p~~~~~~~~~~~~~~~~~~~~~~~~~~~~~~~~~~~~-------~~~~~~~gl~~~l~~~~~~~~~~~~~~   218 (705) |
|  | T 6S7O\_A | 146 | LAAAMIAVVPGYISRSVAGSYDNEGIAIFCMLLTYYMWIKAVKTGS-------ICWAAKCALAYFYMVSSWGGYVFLINL   218 (705) |
|  | T ss\_dssp |  | HHHHHHHSCHHHHHSSCTTCCCHHHHHHHHHHHHHHHHHHHHHHCC-------HHHHHHHHHHHHHHHHHCTTHHHHTTT |
|  | T ss\_pred |  | HHHHHHHHHHHHHHhhcccchhHHHHHHHHHHHHHHHHHHHHhhCC-------HHHHHHHHHHHHHHHHhhchHHHHHHH |
|  |
|  |
|  | Q ss\_pred |  | HHHHHHHHhCCCChHHH--------------------------------------------------------HHHHhhh |
|  | Q Q5T4D3 | 231 | NAVFDILVIGKFNVLEI--------------------------------------------------------VQKVLHK   254 (462) |
|  | Q Consensus | 231 | ~~~~~~~~~~~~~~~~~--------------------------------------------------------~~~~~~~   254 (462) |
|  |  |  | +.+..+.....++.+.. .++. |
|  | T Consensus | 219 | ~~~~~~~~~~~~~~~~~~~~~~~~~~~~~~~~~~~~~~~~~~~~~~~~~~~~~~~~~~~~~~~~~~~~~~~~~~~~~---   295 (705) |
|  | T 6S7O\_A | 219 | IPLHVLVLMLTGRFSHRIYVAYCTVYCLGTILSMQISFVGFQPVLSSEHMAAFGVFGLCQIHAFVDYLRSKLNPQQF---   295 (705) |
|  | T ss\_dssp |  | HHHHHHHHHHHTCCCHHHHHHHHHHHHHHHHHHHTTSSSTTHHHHSSTTHHHHHHHHHHHHHHHHHHHHHHSCHHHH--- |
|  | T ss\_pred |  | HHHHHHHHHHhcCCChHHHHHHHHHHHHHHHHHHhccccCCcccCCHHHHHHHHHHHHHHHHHHHHHHHHcCCHHHH--- |
|  |
|  |
|  | Q ss\_pred |  | ccchHhhchhhhhhHHHHHHHHHHHHHHHHHHHHHHhCCCCCCccccCCcchhcchhhHhHhHHHHHHHHHHHHhhccHh |
|  | Q Q5T4D3 | 255 | DKSLENLGMLRNGGLLFRMTLLTSGGAGMLYVRWRIMGTGPPAFTEVDNPASFADSMLVRAVNYNYYYSLNAWLLLCPWW   334 (462) |
|  | Q Consensus | 255 | ~~~~~~~~~~~~~~~~~~~~~~~~~~~~~~~~~~~~~~~~~~~~~~~~~~~~~~~~~~~~~~~~~~~~~~~~~~~~~p~~   334 (462) |
|  |  |  | +......................................................+. |
|  | T Consensus | 296 | ----------~~~~~~~~~~~~~~~~~~~~~~~~~~~~~~~~~~~~~~~~~~~~~~~~~~~~~~~~~-------------   352 (705) |
|  | T 6S7O\_A | 296 | ----------EVLFRSVISLVGFVLLTVGALLMLTGKISPWTGRFYSLLDPSYAKNNIPIIASVSEH-------------   352 (705) |
|  | T ss\_dssp |  | ----------HHHC----------------------CCCCCCSTTHHHHSTTHHHHTCTTTTTSGGG------------- |
|  | T ss\_pred |  | ----------HHHHHHHHHHHHHHHHHHHHHHHHccccccccHHHHHhhChhHhcCCCCceeecccc------------- |
|  |
|  |
|  | Q ss\_pred |  | hhccccccccCcccccchHHHHHHHHHHHHHHHHHHHHHhcCCCcchHHHHHHHHHHHHHHhhHhccccccchhHHHhhh |
|  | Q Q5T4D3 | 335 | LCFDWSMGCIPLIKSISDWRVIALAALWFCLIGLICQALCSEDGHKRRILTLGLGFLVIPFLPASNLFFRVGFVVAERVL   414 (462) |
|  | Q Consensus | 335 | ~~~~~~~~~~~~~~~~~~~~~~~~~~~~~~~~~~~~~~~~~~~~~~~~~~~~~~~~~~~~~~~~~~~~~~~~~~~~~ry~   414 (462) |
|  |  |  | ...............+.+.........+++++.........+..+...... .||. |
|  | T Consensus | 353 | -------------~~~~~~~~~~~~~~~~~l~~~~~~~~~~~~~~~~~~~~~~~~~~~~~~~~~------------~R~~   407 (705) |
|  | T 6S7O\_A | 353 | -------------QPTTWSSYYFDLQLLVFMFPVGLYYCFSNLSDARIFIIMYGVTSMYFSAVM------------VRLM   407 (705) |
|  | T ss\_dssp |  | -------------SCCCHHHHHHHCSSSGGGHHHHHHHHHHSCCTTHHHHHHHHHHHHHHHHHC------------SGGG |
|  | T ss\_pred |  | -------------CCCCHHHHHHHHHHHHHHHHHHHHHHhcCCChhHHHHHHHHHHHHHHHHHh------------HHHH |
|  |
|  |
|  | Q ss\_pred |  | hHHHHHHHHHHHHHHHHHHhchhhHHH----------------------HHHHHHHHHHHHHHHHHHhc |
|  | Q Q5T4D3 | 415 | YLPSVGYCVLLTFGFGALSKHTKKKKL----------------------IAAVVLGILFINTLRCVLRS   461 (462) |
|  | Q Consensus | 415 | ~~~~~~~~ll~a~~~~~~~~~~~~~~~----------------------~~~~~~~~~~~~~~~~~~~~   461 (462) |
|  |  |  | .+..|+++++++.++..+.++...+.. ....++++++.......... |
|  | T Consensus | 408 | ~~~~p~~~l~~a~~l~~l~~~~~~~~~~~~~~~~~~~~~~~~~~~~~~~~~~~~~~~~~~~~~~~~~~~   476 (705) |
|  | T 6S7O\_A | 408 | LVLAPVMCILSGIGVSQVLSTYMKNLDISRPDKKSKKQQDSTYPIKNEVASGMILVMAFFLITYTFHST   476 (705) |
|  | T ss\_dssp |  | GGGHHHHHHHHHHHHHHHHHHHTTTSSSCC---------------CCHHHHHHHHHHHHHHHHHHHHHH |
|  | T ss\_pred |  | HHHHHHHHHHHHHHHHHHHHHHHhhcCCCCCCccCccccCCCCCcCHHHHHHHHHHHHHHHHHHHHhHh |
|  |
| --- | | | |
|  | Template alignmentTemplate 3D StructurePDBe | | |
| 3. | 6S7T\_A Dolichyl-diphosphooligosaccharide--protein glycosyltransferase subunit STT3B (E.C.2.4.99.18); N-glycosylation, Oligosaccharyltransferase, OSTB, TRANSFERASE; HET: 0K3, KZB, NAG, EGY, MAN, BMA; 3.5A {Homo sapiens} | | |
|  | Probability: 99.82%, E-value: 1.2e-16, Score: 160.98, Aligned cols: 388, Identities: 10%, Similarity: -0.011, | | |
|  |
|  | Q ss\_pred |  | CCcccCChhhcccccCCHHHHHHHHHHHHHHHHHhhhcC------CCcccCchHHHHhcccccCCCChhhhhcccccccc |
|  | Q Q5T4D3 | 1 | MAVLDTDLDHILPSSVLPPFWAKLVVGSVAIVCFARSYD------GDFVFDDSEAIVNNKDLQAETPLGDLWHHDFWGSR   74 (462) |
|  | Q Consensus | 1 | ~~~~~~~~~~~~~~~~~~~~~~~~~l~~~~~~~~~~~~~------~~~~~Dd~~~~~~~~~~~~~~~~~~~~~~~~~~~~   74 (462) |
|  |  |  | ..++.+................+++++++++........ .....||..+...+.++.+++.....-..+..... |
|  | T Consensus | 53 | ~~~~~~~~~~~~~~~~~~~~~~~~l~~i~~~~~~~rl~~~~~~~~~~~~~D~~~~~~~a~~~~~~g~~~~~~~~~~~~~~   132 (826) |
|  | T 6S7T\_A | 53 | PAPAGLSGGLSQPAGWQSLLSFTILFLAWLAGFSSRLFAVIRFESIIHEFDPWFNYRSTHHLASHGFYEFLNWFDERAWY   132 (826) |
|  | T ss\_dssp |  | ----------CCHHHHHHHHHHHHHHHHHHHHHHTTCTTTTTTCSCCCSTTHHHHHHHHHHHHHHCHHHHHTCEECSSST |
|  | T ss\_pred |  | CCCCCCCCCCCCCchHHHHHHHHHHHHHHHHHHHHHHHHHHhhhhhhcCCChHHHHHHHHHHHHhCcHHHhccccchhcC |
|  |
|  |
|  | Q ss\_pred |  | cCCCCCccccCchHHHHHHHHHHHhCC------CCchHHHHHHHHHHHHHHHHHHHHHHHHhccccccccccccchHHHH |
|  | Q Q5T4D3 | 75 | LSSNTSHKSYRPLTVLTFRINYYLSGG------FHPVGFHVVNILLHSGISVLMVDVFSVLFGGLQYTSKGRRLHLAPRA   148 (462) |
|  | Q Consensus | 75 | ~~~~~~~~~~~Pl~~~~~~~~~~~~gg------~~~~~~rl~~~~~~~l~~~l~~~l~~~l~~~~~~~~~~~~~~~~~~~   148 (462) |
|  |  |  | .........++|++.++.+..+.++ | .+....|++++++++++++++|.++|+++ ++.. |
|  | T Consensus | 133 | p~g~~~~~~~~P~~~~l~a~~~~l~-g~~~~~~~~~~~~~l~~~l~~~l~~~~~y~l~r~l~--------------~~~~   197 (826) |
|  | T 6S7T\_A | 133 | PLGRIVGGTVYPGLMITAGLIHWIL-NTLNITVHIRDVCVFLAPTFSGLTSISTFLLTRELW--------------NQGA   197 (826) |
|  | T ss\_dssp |  | TTCEESTTSSCTTHHHHHHHHHHHH-HHTTCCCCHHHHHHTHHHHHHHHHHHHHHHHHHHHS--------------CHHH |
|  | T ss\_pred |  | CCCCCCCCCCchHHHHHHHHHHHHH-HhcCCCCcHHHHHHHHHHHHHHHHHHHHHHHHHHHc--------------CchH |
|  |
|  |
|  | Q ss\_pred |  | HHHHHHHHHHCHHHHHHHHhHhcHHHHHHHHHHHHHHHHHHHHHHHcCCCCCchHHHHHHHHHHHHHHHHhchHHHHHHH |
|  | Q Q5T4D3 | 149 | SLLAALLFAVHPVHTECVAGVVGRADLLCALFFLLSFLGYCKAFRESNKEGAHSSTFWVLLSIFLGAVAMLCKEQGITVL   228 (462) |
|  | Q Consensus | 149 | a~~aall~~~~p~~~~~~~~~~~~~~~~~~~f~ll~~~~~~~~~~~~~~~~~~~~~~~~~~~~~~~~la~l~k~~~~~~~   228 (462) |
|  |  |  | |++++++++++|.+...+......+|.+..++.+++++++.+..++++ .++.++++++++++.++|+.++++. |
|  | T Consensus | 198 | allaall~a~~p~~~~~s~~~~~~~e~~~~~~~~l~l~~~~~~~~~~~-------~~~~~l~gl~~gla~~~~~~~~~~~   270 (826) |
|  | T 6S7T\_A | 198 | GLLAACFIAIVPGYISRSVAGSFDNEGIAIFALQFTYYLWVKSVKTGS-------VFWTMCCCLSYFYMVSAWGGYVFII   270 (826) |
|  | T ss\_dssp |  | HHHHHHHTTTCHHHHGGGSTTCCCSHHHHHHHHHHHHHHHHHHHHHCC-------HHHHHHHHHHHHHHHHHCTTHHHHH |
|  | T ss\_pred |  | HHHHHHHHHHHHHHHHhhhccCchHHHHHHHHHHHHHHHHHHHhccCc-------HHHHHHHHHHHHHHHHhcccHHHHH |
|  |
|  |
|  | Q ss\_pred |  | HHHHHHHHHHhCCCChHHH---------------------------------------------------------HHHH |
|  | Q Q5T4D3 | 229 | GLNAVFDILVIGKFNVLEI---------------------------------------------------------VQKV   251 (462) |
|  | Q Consensus | 229 | ~~~~~~~~~~~~~~~~~~~---------------------------------------------------------~~~~   251 (462) |
|  |  |  | +++++..++....++.+.. .+.. |
|  | T Consensus | 271 | ~~~~l~~~~~~~~~~~~~~~~~~~~~~~~~~~~~~~~~p~~~~~~~~~~~~~~~~~~~~~~~~~~~~~~~~~~~~~~~~~   350 (826) |
|  | T 6S7T\_A | 271 | NLIPLHVFVLLLMQRYSKRVYIAYSTFYIVGLILSMQIPFVGFQPIRTSEHMAAAGVFALLQAYAFLQYLRDRLTKQEFQ   350 (826) |
|  | T ss\_dssp |  | HHHHHHHHHHHHTTCCCHHHHHHHHHHHHHHHHHHTTSTTTTTHHHHBSSTHHHHHHHHHHHHHHHHHHHHHHSCSTTTH |
|  | T ss\_pred |  | HHHHHHHHHHHHhccCChhHHHHHHHHHHHHHHHHhccccCCCCcccchHHHHHHHHHHHHHHHHHHHHHHHhcCHHHHH |
|  |
|  |
|  | Q ss\_pred |  | hhhccchHhhchhhhhhHHHHHHHHHHHHHHHHHHHHHHhCCCCCCccccCCcchhcchhhHhHhHHHHHHHHHHHHhhc |
|  | Q Q5T4D3 | 252 | LHKDKSLENLGMLRNGGLLFRMTLLTSGGAGMLYVRWRIMGTGPPAFTEVDNPASFADSMLVRAVNYNYYYSLNAWLLLC   331 (462) |
|  | Q Consensus | 252 | ~~~~~~~~~~~~~~~~~~~~~~~~~~~~~~~~~~~~~~~~~~~~~~~~~~~~~~~~~~~~~~~~~~~~~~~~~~~~~~~~   331 (462) |
|  |  |  | ........+............................................ |
|  | T Consensus | 351 | ------------------~~~~~~~~~~~~~~~~~~~~~~~~~~~~~~~~~~~~~~~~~~~~~~~~~~~~~---------   403 (826) |
|  | T 6S7T\_A | 351 | ------------------TLFFLGVSLAAGAVFLSVIYLTYTGYIAPWSGRFYSLWDTGYAKIHIPIIASV---------   403 (826) |
|  | T ss\_dssp |  | ------------------HHHHHHHHHHHHHHHHHHHHHHHHTSSBCCCHHHHHHHHSSHHHHTCHHHHHB--------- |
|  | T ss\_pred |  | ------------------HHHHHHHHHHHHHHHHHHHHHHhccccccchHHHHHHhcccccccCccchhcc--------- |
|  |
|  |
|  | Q ss\_pred |  | cHhhhccccccccCcccccchHHHHHHHHHHHHHHHHHHHHHhcCCCcchHHHHHHHHHHHHHHhhHhccccccchhHHH |
|  | Q Q5T4D3 | 332 | PWWLCFDWSMGCIPLIKSISDWRVIALAALWFCLIGLICQALCSEDGHKRRILTLGLGFLVIPFLPASNLFFRVGFVVAE   411 (462) |
|  | Q Consensus | 332 | p~~~~~~~~~~~~~~~~~~~~~~~~~~~~~~~~~~~~~~~~~~~~~~~~~~~~~~~~~~~~~~~~~~~~~~~~~~~~~~~   411 (462) |
|  |  |  | .................++.++...+....+ +++....+.+++.++.+...... . |
|  | T Consensus | 404 | -------------~~~~~~~~~~~~~~~~~~~~~~~~~~~~~~~---~~~~~~~~~~~~~~~~~~~~~~~---------~   458 (826) |
|  | T 6S7T\_A | 404 | -------------SEHQPTTWVSFFFDLHILVCTFPAGLWFCIK---NINDERVFVALYAISAVYFAGVM---------V   458 (826) |
|  | T ss\_dssp |  | -------------STTSCCCHHHHHHSCSSHHHHHHHHHHHHHH---SCCHHHHHHHHHHHHHHHHHTTC---------S |
|  | T ss\_pred |  | -------------cccCCCCHHHHHHHHHHHHHHHHHHHHHHHh---cCCHHHHHHHHHHHHHHHHHHHH---------H |
|  |
|  |
|  | Q ss\_pred |  | hhhhHHHHHHHHHHHHHHHHHHhch--------------------------------------------------hhHHH |
|  | Q Q5T4D3 | 412 | RVLYLPSVGYCVLLTFGFGALSKHT--------------------------------------------------KKKKL   441 (462) |
|  | Q Consensus | 412 | ry~~~~~~~~~ll~a~~~~~~~~~~--------------------------------------------------~~~~~   441 (462) |
|  |  |  | ||.++..|+++++++.++..+.++. ..... |
|  | T Consensus | 459 | Ry~~~~~p~~~ll~a~~l~~l~~~~~~~~~~~~~~~~~~~~~~~~~~~~~~~~~~~~~~~~~~~~~~~~~~~~~~~~~~~   538 (826) |
|  | T 6S7T\_A | 459 | RLMLTLTPVVCMLSAIAFSNVFEHYLGDDMKRENPPVEDSSDEDDKRNQGNLYDKAGKVRKHATEQEKTEEGLGPNIKSI   538 (826) |
|  | T ss\_dssp |  | TTHHHHHHHHHHHHHHHHHHHHHHTCC-----------------------------------------------CHHHHH |
|  | T ss\_pred |  | HHHHhHHHHHHHHHHHHHHHHHHHHhccccccCCCCCCCCCccchhhccccccccccccccccchhhhhhcCCChHHHHH |
|  |
|  |
|  | Q ss\_pred |  | HHHHHHHHHHHHHHHHHHhcC |
|  | Q Q5T4D3 | 442 | IAAVVLGILFINTLRCVLRSG   462 (462) |
|  | Q Consensus | 442 | ~~~~~~~~~~~~~~~~~~~~~   462 (462) |
|  |  |  | ..++++++++........... |
|  | T Consensus | 539 | ~~~~~~~~~~~~~~~~~~~~~   559 (826) |
|  | T 6S7T\_A | 539 | VTMLMLMLLMMFAVHCTWVTS   559 (826) |
|  | T ss\_dssp |  | HHHHHHHHHHHHHHHHHHHCC |
|  | T ss\_pred |  | HHHHHHHHHHHHHHHHHHHhh |
|  |
| --- | | | |
|  | Template alignmentTemplate 3D StructurePDBe | | |
| 4. | 6EZN\_F Dolichyl-diphosphooligosaccharide--protein glycosyltransferase subunit 1 (E.C.2.4.99.18); OST complex, oligosaccharyltransferase, N-linked glycosylation; HET: PTY, BMA, CPL, MAN, NAG;{Saccharomyces cerevisiae (strain ATCC 204508 / S288c)}; Related PDB entries: 6C26\_A; Related PDB entries: 6C26\_A; Related PDB entries: 6C26\_A | | |
|  | Probability: 99.82%, E-value: 1.7e-16, Score: 157.46, Aligned cols: 405, Identities: 11%, Similarity: -0.037, | | |
|  |
|  | Q ss\_pred |  | cccCChhhcccccCCHHHHHHHHHHHHHHHHHhhh-cCCCc---ccCchHHHHhcccccCCCChhhhhcccccccccCCC |
|  | Q Q5T4D3 | 3 | VLDTDLDHILPSSVLPPFWAKLVVGSVAIVCFARS-YDGDF---VFDDSEAIVNNKDLQAETPLGDLWHHDFWGSRLSSN   78 (462) |
|  | Q Consensus | 3 | ~~~~~~~~~~~~~~~~~~~~~~~l~~~~~~~~~~~-~~~~~---~~Dd~~~~~~~~~~~~~~~~~~~~~~~~~~~~~~~~   78 (462) |
|  |  |  | |+++++..............+++++++++..+... ...+. ..||..|...+.++.+++.....-..+......... |
|  | T Consensus | 1 | m~~~~~~~~~~~~~~~~~~~l~~~~~~~~~~~~~~~~~~~~~~~~~D~~~~~~~a~~~~~~g~~~~~~~~~~~~~~~~g~   80 (718) |
|  | T 6EZN\_F | 1 | MGSDRSCVLSVFQTILKLVIFVAIFGAAISSRLFAVIKFESIIHEFDPWFNYRATKYLVNNSFYKFLNWFDDRTWYPLGR   80 (718) |
|  | T ss\_dssp |  | -----CCSHHHHHHHHHHHHHHHHHHHHHHTTTTTTTTTCCCCCSSSHHHHHHHHHHHHHSCHHHHHSCCCTTSSTTTCC |
|  | T ss\_pred |  | CCcchhHHHHHHHHHHHHHHHHHHHHHHHHHHHHHHHHchhhhcccChHHHHHHHHHHHHcccHHHhcccCCcccCCCCC |
|  |
|  |
|  | Q ss\_pred |  | CCccccCchHHHHHHHHHHH----hCC---CCchHHHHHHHHHHHHHHHHHHHHHHHHhccccccccccccchHHHHHHH |
|  | Q Q5T4D3 | 79 | TSHKSYRPLTVLTFRINYYL----SGG---FHPVGFHVVNILLHSGISVLMVDVFSVLFGGLQYTSKGRRLHLAPRASLL   151 (462) |
|  | Q Consensus | 79 | ~~~~~~~Pl~~~~~~~~~~~----~gg---~~~~~~rl~~~~~~~l~~~l~~~l~~~l~~~~~~~~~~~~~~~~~~~a~~   151 (462) |
|  |  |  | .....++|.+.++.+..+.+ + | +.....|+.++++++++++++|.++|+++ ++..|++ |
|  | T Consensus | 81 | ~~~~~~~p~~~~l~a~~~~l~~~~~-G~~~~~~~~~~~~~~l~~~l~~~~~y~l~~~l~--------------~~~~a~~   145 (718) |
|  | T 6EZN\_F | 81 | VTGGTLYPGLMTTSAFIWHALRNWL-GLPIDIRNVCVLFAPLFSGVTAWATYEFTKEIK--------------DASAGLL   145 (718) |
|  | T ss\_dssp |  | CSSSSCCTTHHHHHHHHHHCCCCCS-SCCCCHHHHHHBTHHHHHHHHHHHHHHHHHHHS--------------CHHHHHH |
|  | T ss\_pred |  | CCCCCCChHHHHHHHHHHHHHHHHh-CCCCCHHHHHHHHHHHHHHHHHHHHHHHHHHhc--------------ChHHHHH |
|  |
|  |
|  | Q ss\_pred |  | HHHHHHHCHHHHHHHHhHhcHHHHHHHHHHHHHHHHHHHHHHHcCCCCCchHHHHHHHHHHHHHHHHhchHHHHHHHHHH |
|  | Q Q5T4D3 | 152 | AALLFAVHPVHTECVAGVVGRADLLCALFFLLSFLGYCKAFRESNKEGAHSSTFWVLLSIFLGAVAMLCKEQGITVLGLN   231 (462) |
|  | Q Consensus | 152 | aall~~~~p~~~~~~~~~~~~~~~~~~~f~ll~~~~~~~~~~~~~~~~~~~~~~~~~~~~~~~~la~l~k~~~~~~~~~~   231 (462) |
|  |  |  | ++++++++|.+...+......+|.+..++.+++++++.+..++++ .++.++++++.+++.++|+.+.++.+++ |
|  | T Consensus | 146 | aa~l~~~~p~~~~~~~~g~~~~~~~~~~~~~~~l~~~~~~~~~~~-------~~~~~l~gl~~~l~~~~~~~~~~~~~~~   218 (718) |
|  | T 6EZN\_F | 146 | AAGFIAIVPGYISRSVAGSYDNEAIAITLLMVTFMFWIKAQKTGS-------IMHATCAALFYFYMVSAWGGYVFITNLI   218 (718) |
|  | T ss\_dssp |  | HHHHHHHCHHHHSSSCSBCCSSSTTTHHHHHHHHHHHHHHHHHCC-------HHHHHHHHHHHHHHHTTCTTGGGGGGTH |
|  | T ss\_pred |  | HHHHHHHHHHHHHhHhccccchHHHHHHHHHHHHHHHHHHhhcCC-------HHHHHHHHHHHHHHHHhccchHHHHHHH |
|  |
|  |
|  | Q ss\_pred |  | HHHHHHHhCCCChHHHHHHHhhhccchHhhchhhhhhHHHHHHHHHHHHHHHHHHHHHHhCCCCCCccccCCcchhcchh |
|  | Q Q5T4D3 | 232 | AVFDILVIGKFNVLEIVQKVLHKDKSLENLGMLRNGGLLFRMTLLTSGGAGMLYVRWRIMGTGPPAFTEVDNPASFADSM   311 (462) |
|  | Q Consensus | 232 | ~~~~~~~~~~~~~~~~~~~~~~~~~~~~~~~~~~~~~~~~~~~~~~~~~~~~~~~~~~~~~~~~~~~~~~~~~~~~~~~~   311 (462) |
|  |  |  | .+..+.....++ +............+..+.............................. |
|  | T Consensus | 219 | ~~~~~~~~~~~~---------------------~~~~~~~~~~~~~~~~~~~~~~~p~~~~~~~~~~~~~~~~~~~~~~~   277 (718) |
|  | T 6EZN\_F | 219 | PLHVFLLILMGR---------------------YSSKLYSAYTTWYAIGTVASMQIPFVGFLPIRSNDHMAALGVFGLIQ   277 (718) |
|  | T ss\_dssp |  | HHHHHHHHHTTC---------------------CCHHHHHHHHHHHHHHHHHTTCSTTSSSHHHHCTTSHHHHHHHHHHH |
|  | T ss\_pred |  | HHHHHHHHHccC---------------------CChhHHHHHHHHHHHHHHHHhcchhccCCccCchHHHHHHHHHHHHH |
|  |
|  |
|  | Q ss\_pred |  | hHhHh-------------------------------------HHHHHHHHHHHHhhccHhhhccccccccCcccccchHH |
|  | Q Q5T4D3 | 312 | LVRAV-------------------------------------NYNYYYSLNAWLLLCPWWLCFDWSMGCIPLIKSISDWR   354 (462) |
|  | Q Consensus | 312 | ~~~~~-------------------------------------~~~~~~~~~~~~~~~p~~~~~~~~~~~~~~~~~~~~~~   354 (462) |
|  |  |  | ..... ...............+...................... |
|  | T Consensus | 278 | ~~~~~~~~~~~~~~~~~~~~~~~~~~~~~~~~~~~~~~~~~~~~~~~~~~~~~~~~~~~~~~~~~~~~~~~~~~~~~~~~   357 (718) |
|  | T 6EZN\_F | 278 | IVAFGDFVKGQISTAKFKVIMMVSLFLILVLGVVGLSALTYMGLIAPWTGRFYSLWDTNYAKIHIPIIASVSEHQPVSWP   357 (718) |
|  | T ss\_dssp |  | HHHHHHHHHTTSCHHHHTTTC-----------------------------------------------------CCCCHH |
|  | T ss\_pred |  | HHHHHHHHHhcCChHHHHHHHHHHHHHHHHHHHHHHHHHHHhhhhhhhhHHHHHhhcccccccCCCcccchhHhCCCCHH |
|  |
|  |
|  | Q ss\_pred |  | HHHHHHHHHHHHHHHHHHHhcCCCcchHHHHHHHHHHHHHHhhHhccccccchhHHHhhhhHHHHHHHHHHHHHHHHHHh |
|  | Q Q5T4D3 | 355 | VIALAALWFCLIGLICQALCSEDGHKRRILTLGLGFLVIPFLPASNLFFRVGFVVAERVLYLPSVGYCVLLTFGFGALSK   434 (462) |
|  | Q Consensus | 355 | ~~~~~~~~~~~~~~~~~~~~~~~~~~~~~~~~~~~~~~~~~~~~~~~~~~~~~~~~~ry~~~~~~~~~ll~a~~~~~~~~   434 (462) |
|  |  |  | ..........++.........+++++................... .||..+..|+++++++.++..+.+ |
|  | T Consensus | 358 | ~~~~~~~~~~~l~~~g~~~~~~~~~~~~~~~~~~~~~~~~~~~~~-----------~R~~~~~~p~~~il~a~~l~~l~~   426 (718) |
|  | T 6EZN\_F | 358 | AFFFDTHFLIWLFPAGVFLLFLDLKDEHVFVIAYSVLCSYFAGVM-----------VRLMLTLTPVICVSAAVALSKIFD   426 (718) |
|  | T ss\_dssp |  | HHHHHSSSTTTTHHHHHHHHHTTCCSSHHHHHHHHHHHHHHHHHC-----------STTHHHHHHHHHHHHHHHHHHHHH |
|  | T ss\_pred |  | HHHhhhhHHHHHHHHHHHHHHhcCChHHHHHHHHHHHHHHHHHHH-----------HHHHHHHHHHHHHHHHHHHHHHHH |
|  |
|  |
|  | Q ss\_pred |  | chhhHH-------------HHHHHHHHHHHHHHHHHHHhc |
|  | Q Q5T4D3 | 435 | HTKKKK-------------LIAAVVLGILFINTLRCVLRS   461 (462) |
|  | Q Consensus | 435 | ~~~~~~-------------~~~~~~~~~~~~~~~~~~~~~   461 (462) |
|  |  |  | +.+..+ ....++++++++......... |
|  | T Consensus | 427 | ~~~~~~~~~~~~~~~~~~~~~~~~~~~~~~~~~~~~~~~~   466 (718) |
|  | T 6EZN\_F | 427 | IYLDFKTSDRKYAIKPAALLAKLIVSGSFIFYLYLFVFHS   466 (718) |
|  | T ss\_dssp |  | HSCCCC-------CCHHHHHHHHHHHHHHHHHHHHHHHHH |
|  | T ss\_pred |  | HHhccccCCccccccchHHHHHHHHHHHHHHHHHHHHHHH |
|  |
| --- | | | |
|  | Template alignmentTemplate 3D StructurePDBe | | |
| 5. | 3WAJ\_A Transmembrane oligosaccharyl transferase (E.C.2.4.1.119); oligosaccharyltransferase, N-glycosylation, Archaeoglobus fulgidus, GT-C; 2.501A {Archaeoglobus fulgidus}; Related PDB entries: 5GMY\_A 3WAK\_A; Related PDB entries: 5GMY\_A 3WAK\_A; Related PDB entries: 5GMY\_A 3WAK\_A | | |
|  | Probability: 99.8%, E-value: 1e-15, Score: 155.35, Aligned cols: 391, Identities: 10%, Similarity: 0.012, | | |
|  |
|  | Q ss\_pred |  | ChhhcccccCCHHHHHHHHHHHHHHHHHh-------hhcCCCcc-cCchHHHHhcccccCCCChhhhhcccccccccCCC |
|  | Q Q5T4D3 | 7 | DLDHILPSSVLPPFWAKLVVGSVAIVCFA-------RSYDGDFV-FDDSEAIVNNKDLQAETPLGDLWHHDFWGSRLSSN   78 (462) |
|  | Q Consensus | 7 | ~~~~~~~~~~~~~~~~~~~l~~~~~~~~~-------~~~~~~~~-~Dd~~~~~~~~~~~~~~~~~~~~~~~~~~~~~~~~   78 (462) |
|  |  |  | ++....+..+......+++++++++..+. ...+.... .||..|...+.++.++......++...... . |
|  | T Consensus | 1 | ~~~~~~~~~~~~~~~~l~~l~~~~~~lr~~~~~~~~~~~~~~~~~~D~~~~~~~a~~~~~~~~~~~~~d~~~~~p----~   76 (875) |
|  | T 3WAJ\_A | 1 | MQNAESWFKKYWHLSVLVIAALISVKLRILNPWNSVFTWTVRLGGNDPWYYYRLIENTIHNFPHRIWFDPFTYYP----Y   76 (875) |
|  | T ss\_dssp |  | --------------CTTTHHHHHHHCCCCCTTHHHHBSSSBCCCSSHHHHHHHHHHHHHHTTTCCCSEETTSTTT----T |
|  | T ss\_pred |  | CcchHHHHhhHHHHHHHHHHHHHHHHHHhhCccccccCCceecccCChHHHHHHHHHHHHHCcccCCCCchhcCC----C |
|  |
|  |
|  | Q ss\_pred |  | CCccccCchHHHHHHHHHHHhCCCC-----chHHHHHHHHHHHHHHHHHHHHHHHHhccccccccccccchHHHHHHHHH |
|  | Q Q5T4D3 | 79 | TSHKSYRPLTVLTFRINYYLSGGFH-----PVGFHVVNILLHSGISVLMVDVFSVLFGGLQYTSKGRRLHLAPRASLLAA   153 (462) |
|  | Q Consensus | 79 | ~~~~~~~Pl~~~~~~~~~~~~gg~~-----~~~~rl~~~~~~~l~~~l~~~l~~~l~~~~~~~~~~~~~~~~~~~a~~aa   153 (462) |
|  |  |  | .....++|++.++.+....++ |.+ ....|+.++++++++++++|.++|+++ +++.|++++ |
|  | T Consensus | 77 | g~~~~~~Pl~~~l~a~~~~l~-G~~~~~~~~~~~~l~~~l~~~l~~~~~y~l~r~l~--------------~~~~allaa   141 (875) |
|  | T 3WAJ\_A | 77 | GSYTHFGPFLVYLGSIAGIIF-SATSGESLRAVLAFIPAIGGVLAILPVYLLTREVF--------------DKRAAVIAA   141 (875) |
|  | T ss\_dssp |  | CEECCSCHHHHHHHHHHHHHT-TCCSHHHHHHHHHHHHHHHHHTTHHHHHHHHHHHS--------------CHHHHHHHH |
|  | T ss\_pred |  | CcCCCchhHHHHHHHHHHHHH-cCCChHHHHHHHHHHHHHHHHHHHHHHHHHHHHHc--------------ChHHHHHHH |
|  |
|  |
|  | Q ss\_pred |  | HHHHHCHH-HHHHHHhHhcHHHHHHHHHHHHHHHHHHHHHH------------HcCCCCCchHHHHHHHHHHHHHHHHhc |
|  | Q Q5T4D3 | 154 | LLFAVHPV-HTECVAGVVGRADLLCALFFLLSFLGYCKAFR------------ESNKEGAHSSTFWVLLSIFLGAVAMLC   220 (462) |
|  | Q Consensus | 154 | ll~~~~p~-~~~~~~~~~~~~~~~~~~f~ll~~~~~~~~~~------------~~~~~~~~~~~~~~~~~~~~~~la~l~   220 (462) |
|  |  |  | ++++++|. ....+......+|.+..++.+++++++.+..+ +++ .++.++++++++++.++ |
|  | T Consensus | 142 | ll~a~~p~~~~~~s~~g~~~~~~~~~~~~~l~l~~~~~~~~~~~~~~~~~~~~~~~-------~~~~~l~gl~~gl~~lt   214 (875) |
|  | T 3WAJ\_A | 142 | FLIAIVPGQFLQRSILGFNDHHIWEAFWQVSALGTFLLAYNRWKGHDLSHNLTARQ-------MAYPVIAGITIGLYVLS   214 (875) |
|  | T ss\_dssp |  | HHHTTCCSHHHHTTSTTCCCSHHHHHHHHHHHHHHHHHHHTTSSSCCC----CTTT-------SHHHHHHHHHHHHHHHH |
|  | T ss\_pred |  | HHHHHcchHHHHHHhccccchHHHHHHHHHHHHHHHHHHHHHhccCCCCcccccch-------hHHHHHHHHHHHHHHHH |
|  |
|  |
|  | Q ss\_pred |  | hHHHHHHHHHHHHHHHH--------HhCCCChHHHHHHHhhhccchHhhchhhhhhHHHHHHHHHHHHHHHHHHHHHHhC |
|  | Q Q5T4D3 | 221 | KEQGITVLGLNAVFDIL--------VIGKFNVLEIVQKVLHKDKSLENLGMLRNGGLLFRMTLLTSGGAGMLYVRWRIMG   292 (462) |
|  | Q Consensus | 221 | k~~~~~~~~~~~~~~~~--------~~~~~~~~~~~~~~~~~~~~~~~~~~~~~~~~~~~~~~~~~~~~~~~~~~~~~~~   292 (462) |
|  |  |  | |..+.++.+++++..++ .+++++ .........++.......+.... |
|  | T Consensus | 215 | ~~~~~~~~~~~~~~~~~~~~~~~~~~~~~~~--------------------------~~~~~~~~~~~~~l~~~p~~~~~   268 (875) |
|  | T 3WAJ\_A | 215 | WGAGFIIAPIILAFMFFAFVLAGFVNADRKN--------------------------LSLVAVVTFAVSALIYLPFAFNY   268 (875) |
|  | T ss\_dssp |  | CGGGGGHHHHHHHHHHHHHHTTTTCCCCHHH--------------------------HHHHHHHHHHHHHHHHGGGTTSS |
|  | T ss\_pred |  | hchHHHHHHHHHHHHHHHHHHHHhCCCCCHh--------------------------HHHHHHHHHHHHHHHHHHHhcCC |
|  |
|  |
|  | Q ss\_pred |  | CCCCCccccC----------------------------CcchhcchhhHhHhHHHHHHHHHHHHhhccHhhhcccccccc |
|  | Q Q5T4D3 | 293 | TGPPAFTEVD----------------------------NPASFADSMLVRAVNYNYYYSLNAWLLLCPWWLCFDWSMGCI   344 (462) |
|  | Q Consensus | 293 | ~~~~~~~~~~----------------------------~~~~~~~~~~~~~~~~~~~~~~~~~~~~~p~~~~~~~~~~~~   344 (462) |
|  |  |  | .......... .............................|............ |
|  | T Consensus | 269 | ~~~~~~~~~~~~~~~~~~~~~~~~~~~~~~~~~~~~~~~~~~~~~~~~~~~~~~~~~~~~~~~~~~~~~~~~~~~~~~~~   348 (875) |
|  | T 3WAJ\_A | 269 | PGFSTIFYSPFQLLVLLGSAVIAAAFYQIEKWNDVGFFERVGLGRKGMPLAVIVLTALIMGLFFVISPDFARNLLSVVRV   348 (875) |
|  | T ss\_dssp |  | SSCCSSSSCHHHHHHHHHHHHHHHHHHHHHHHHHHTHHHHTTCGGGHHHHHHHHHHHHHHHHHHC--------------- |
|  | T ss\_pred |  | CCCChhcccHHHHHHHHHHHHHHHHHHHHHHhcCcchHHhcCCCccchHHHHHHHHHHHHHHHHHHCCHHHHHHHhhcce |
|  |
|  |
|  | Q ss\_pred |  | Cccc--------------------ccchHHHHHHHHHHHHHHHHHHHHHhcCCCcchHHHHHHHHHHHHHHhhHhccccc |
|  | Q Q5T4D3 | 345 | PLIK--------------------SISDWRVIALAALWFCLIGLICQALCSEDGHKRRILTLGLGFLVIPFLPASNLFFR   404 (462) |
|  | Q Consensus | 345 | ~~~~--------------------~~~~~~~~~~~~~~~~~~~~~~~~~~~~~~~~~~~~~~~~~~~~~~~~~~~~~~~~   404 (462) |
|  |  |  | .... ..............++++++.....+++++++.......+..++...... |
|  | T Consensus | 349 | ~~~~~~~~~i~e~~~~~~~~~~~~~~~~~~~~~~~~~~l~~~~~~~~~~~~~~~~~~~~~~l~~~~~~~~~~~~------   422 (875) |
|  | T 3WAJ\_A | 349 | VQPKGGALTIAEVYPFFFTHNGEFTLTNAVLHFGALFFFGMAGILYSAYRFLKRRSFPEMALLIWAIAMFIALW------   422 (875) |
|  | T ss\_dssp |  | -------------------------CTHHHHHHTTHHHHHHHHHHHHHHHHHHHCCHHHHHHHHHHHHHHHHTS------ |
|  | T ss\_pred |  | ecCCCCceeeeeccccccccCCCccHHHHHHHhHHHHHHHHHHHHHHHHHHHccCCchhHHHHHHHHHHHHHHH------ |
|  |
|  |
|  | Q ss\_pred |  | cchhHHHhhhhHHHHHHHHHHHHHHHHHHhch-------------hhHHHHHHHHHHHHHHHHHHHHH |
|  | Q Q5T4D3 | 405 | VGFVVAERVLYLPSVGYCVLLTFGFGALSKHT-------------KKKKLIAAVVLGILFINTLRCVL   459 (462) |
|  | Q Consensus | 405 | ~~~~~~~ry~~~~~~~~~ll~a~~~~~~~~~~-------------~~~~~~~~~~~~~~~~~~~~~~~   459 (462) |
|  |  |  | ...||.++..|+++++++.++..+.++. ++.+....++++++++....... |
|  | T Consensus | 423 | ----~~~Ry~~~~~p~~~il~a~~l~~l~~~~~~~~~~~~~~~~~~~~~~~~~~~~~~~~~~~~~~~~   486 (875) |
|  | T 3WAJ\_A | 423 | ----GQNRFAYYFAAVSAVYSALALSVVFDKLHLYRALENAIGARNKLSYFRVAFALLIALAAIYPTY   486 (875) |
|  | T ss\_dssp |  | ----SCGGGTHHHHHHHHHHHHHHHHHHGGGCC-----------------CCTTTHHHHHHHHHHHHH |
|  | T ss\_pred |  | ----HhhhHHHHHHHHHHHHHHHHHHHHHHHHhHHHHHHhhhchhccchHHHHHHHHHHHHHHHHHHH |
|  |
| --- | | | |
|  | Template alignmentTemplate 3D StructurePDBe | | |
| 6. | 5OGL\_A Peptide-binding protein, Substrate mimicking peptide; Oligosaccharyltransferase, Complex, Protein N-glycosylation, Bacteria; HET: 9UB, PPN; 2.7A {Campylobacter lari (strain RM2100 / D67 / ATCC BAA-1060)}; Related PDB entries: 3RCE\_A 6GXC\_A; Related PDB entries: 6GXC\_A 3RCE\_A ; Related PDB entries: 6GXC\_A 3RCE\_A | | |
|  | Probability: 99.76%, E-value: 1.1e-15, Score: 151.5, Aligned cols: 373, Identities: 12%, Similarity: 0.027, | | |
|  |
|  | Q ss\_pred |  | ccCChhhcccccCCHHHHHHHHHHHHHHHHHh-----hhcCCCcccCch---------HHHHhcccccCCCChhhhhccc |
|  | Q Q5T4D3 | 4 | LDTDLDHILPSSVLPPFWAKLVVGSVAIVCFA-----RSYDGDFVFDDS---------EAIVNNKDLQAETPLGDLWHHD   69 (462) |
|  | Q Consensus | 4 | ~~~~~~~~~~~~~~~~~~~~~~l~~~~~~~~~-----~~~~~~~~~Dd~---------~~~~~~~~~~~~~~~~~~~~~~   69 (462) |
|  |  |  | |+++.....+...+.....+++++++++.++. .....++..||. .|...+.+..++.... |
|  | T Consensus | 1 | m~~~~~~~~~~~~~~~~~~l~~i~~~~~~lRl~~~~~~~~~~~~~~~~~~~~~~~D~~~~~~~a~~~~~~~~~~------   74 (713) |
|  | T 5OGL\_A | 1 | MELQQNFTDNNSIKYTAILILIAFAFSVLARLYWVAWASEFYEFFFNDQLMITTNDGYAFAEGARDMIAGFHQP------   74 (713) |
|  | T ss\_dssp |  | -CCSSCCTTCCCHHHHHHHHHHHHHHHHHHHHHHHHHHTTCGGGEETTEECCSSTTHHHHHHHHHHHHHTCCCT------ |
|  | T ss\_pred |  | CccchhccccccHHHHHHHHHHHHHHHHHHHHHHHHHhhccchhhcCCEEeeccccHHHHHHHHHHHHcCCCCC------ |
|  |
|  |
|  | Q ss\_pred |  | ccccccCCCCCccccCchHHHHHHHHHHHhCC-CCchHHHHHHHHHHHHHHHHHHHHHHHHhccccccccccccchHHHH |
|  | Q Q5T4D3 | 70 | FWGSRLSSNTSHKSYRPLTVLTFRINYYLSGG-FHPVGFHVVNILLHSGISVLMVDVFSVLFGGLQYTSKGRRLHLAPRA   148 (462) |
|  | Q Consensus | 70 | ~~~~~~~~~~~~~~~~Pl~~~~~~~~~~~~gg-~~~~~~rl~~~~~~~l~~~l~~~l~~~l~~~~~~~~~~~~~~~~~~~   148 (462) |
|  |  |  | .....++|++.++.+....++ | +.....|++++++++++++.+|.++|+++ +++. |
|  | T Consensus | 75 | ---------~~~~~~~p~~~~l~~~~~~l~-g~~~~~~~~~~~~l~~~l~v~~~y~l~r~l~--------------~~~~   130 (713) |
|  | T 5OGL\_A | 75 | ---------NDLSYFGSSLSTLTYWLYSIL-PFSFESIILYMSTFFASLIVVPIILIAREYK--------------LTTY   130 (713) |
|  | T ss\_dssp |  | ---------TSCCCTTCHHHHHHHHHHHHS-CSCHHHHHHHHHHHHGGGGHHHHHHHHHHTT--------------CHHH |
|  | T ss\_pred |  | ---------CCcchhcchHHHHHHHHHHhC-CCCHHHHHHHHHHHHHHHHHHHHHHHHHHhC--------------CchH |
|  |
|  |
|  | Q ss\_pred |  | HHHHHHHHHHCHHHHHHHHhHhcHHHHHHHHHHHHHHHHHHHHHHHcCCCCCchHHHHHHHHHHHHHHHHhchHHHH--- |
|  | Q Q5T4D3 | 149 | SLLAALLFAVHPVHTECVAGVVGRADLLCALFFLLSFLGYCKAFRESNKEGAHSSTFWVLLSIFLGAVAMLCKEQGI---   225 (462) |
|  | Q Consensus | 149 | a~~aall~~~~p~~~~~~~~~~~~~~~~~~~f~ll~~~~~~~~~~~~~~~~~~~~~~~~~~~~~~~~la~l~k~~~~---   225 (462) |
|  |  |  | |++++++++++|.++..+......+|.+..++.+++++++.+..++++ .++.++++++.+++.++|..+. |
|  | T Consensus | 131 | al~aall~a~~p~~~~~s~~g~~~~d~~~~~~~~l~~~~~~~~~~~~~-------~~~~~l~gl~~~l~~~~~~~~~~~~   203 (713) |
|  | T 5OGL\_A | 131 | GFIAALLGSIANSYYNRTMSGYYDTDMLVLVLPMLILLTFIRLTINKD-------IFTLLLSPVFIMIYLWWYPSSYSLN   203 (713) |
|  | T ss\_dssp |  | HHHHHHHHHHCHHHHHTTSTTCCSGGGGTTHHHHHHHHHHHHHHHHCC-------TTHHHHHHHHHHHHHHHCGGGHHHH |
|  | T ss\_pred |  | HHHHHHHHHHHHHHHHhhccccCchHHHHHHHHHHHHHHHHHHHcCCc-------hHHHHHHHHHHHHHHhhccchHHHH |
|  |
|  |
|  | Q ss\_pred |  | -HHHHHHHHHHHHHhCCCCh---------------------------------HHHHHHHhhhccchHhhchhhhhhHHH |
|  | Q Q5T4D3 | 226 | -TVLGLNAVFDILVIGKFNV---------------------------------LEIVQKVLHKDKSLENLGMLRNGGLLF   271 (462) |
|  | Q Consensus | 226 | -~~~~~~~~~~~~~~~~~~~---------------------------------~~~~~~~~~~~~~~~~~~~~~~~~~~~   271 (462) |
|  |  |  | .+.+...++.+..+++++. ++...+. ... |
|  | T Consensus | 204 | ~~~~~~~~~~~~~~~~~~~~~~~~~~~~~~~~~~~~~~~~~~~~~~~~~~~~~~~~~~~~-----------------~~~   266 (713) |
|  | T 5OGL\_A | 204 | FAMIGLFGLYTLVFHRKEKIFYLTIALMIIALSMLAWQYKLALIVLLFAIFAFKEEKINF-----------------YMI   266 (713) |
|  | T ss\_dssp |  | HHHHHHHHHHHHHHTTTCHHHHHHHHHHHHHHSCCCHHHHHHHHHHHHHHHHHCSSCCCH-----------------HHH |
|  | T ss\_pred |  | HHHHHHHHHHHHHhCCcchHHHHHHHHHHHHHhhhhHHHHHHHHHHHHHHHHcchhccch-----------------HHH |
|  |
|  |
|  | Q ss\_pred |  | HHHHHHHHHHHHHHHHHHHhCCCCCCccccCCcchhcchhhHhHhHHHHHHHHHHHHhhccHhhhccccccccCcccccc |
|  | Q Q5T4D3 | 272 | RMTLLTSGGAGMLYVRWRIMGTGPPAFTEVDNPASFADSMLVRAVNYNYYYSLNAWLLLCPWWLCFDWSMGCIPLIKSIS   351 (462) |
|  | Q Consensus | 272 | ~~~~~~~~~~~~~~~~~~~~~~~~~~~~~~~~~~~~~~~~~~~~~~~~~~~~~~~~~~~~p~~~~~~~~~~~~~~~~~~~   351 (462) |
|  |  |  | ..........................+..................+.......... ....... |
|  | T Consensus | 267 | ~~~~~~~~~~~~~~~~~~~~~~~~~~~~~~~~~~~~~~~~~~~~~~~~~~~~~~~~-----------------~~~~~~~   329 (713) |
|  | T 5OGL\_A | 267 | WALIFISILILHLSGGLDPVLYQLKFYVFKASDVQNLKDAAFMYFNVNETIMEVNT-----------------IDPEVFM   329 (713) |
|  | T ss\_dssp |  | HHHHHHHHHHHHHTTTTHHHHHHHHHHTSCCSCCSCCTTTSCCCCCGGGGBGGGCC-----------------CCHHHHH |
|  | T ss\_pred |  | HHHHHHHHHHHHHhccchHHHHHHHHHhhcccccccccccccccccHHHHHHHhcC-----------------CCHHHHH |
|  |
|  |
|  | Q ss\_pred |  | hHHHHHHHHHHHHHHHHHHHHHhcCCCcchHHHHHHHHHHHHHHhhHhccccccchhHHHhhhhHHHHHHHHHHHHHHHH |
|  | Q Q5T4D3 | 352 | DWRVIALAALWFCLIGLICQALCSEDGHKRRILTLGLGFLVIPFLPASNLFFRVGFVVAERVLYLPSVGYCVLLTFGFGA   431 (462) |
|  | Q Consensus | 352 | ~~~~~~~~~~~~~~~~~~~~~~~~~~~~~~~~~~~~~~~~~~~~~~~~~~~~~~~~~~~~ry~~~~~~~~~ll~a~~~~~   431 (462) |
|  |  |  | ...........+++++..... ++++...+.+.++++.+...... .||.++..|+++++++.++.. |
|  | T Consensus | 330 | ~~~~~~~~~~~l~~~gl~~~~------~~~~~~~~~l~~~~~~~~~~~~~---------~R~~~~~~p~~~i~~a~~l~~   394 (713) |
|  | T 5OGL\_A | 330 | QRISSSVLVFILSFIGFILLL------KDHKSMLLALPMLALGFMALRAG---------LRFTIYAVPVMALGFGYFLYA   394 (713) |
|  | T ss\_dssp |  | HHHHSSHHHHHHHHHHHHHHH------TTCGGGGGGHHHHHHHHHHHHHC---------GGGGGGGHHHHHHHHHHHHHH |
|  | T ss\_pred |  | HHhcccHHHHHHHHHHHHHHH------HcCHhHHHHHHHHHHHHHHHHHh---------HHHHHHHHHHHHHHHHHHHHH |
|  |
|  |
|  | Q ss\_pred |  | HHhchhh---------HHHHHHHHHHHHHHHHHHHHHhcC |
|  | Q Q5T4D3 | 432 | LSKHTKK---------KKLIAAVVLGILFINTLRCVLRSG   462 (462) |
|  | Q Consensus | 432 | ~~~~~~~---------~~~~~~~~~~~~~~~~~~~~~~~~   462 (462) |
|  |  |  | +.++.++ +.....+++++++........... |
|  | T Consensus | 395 | l~~~~~~~~~~~~~~~~~~~~~~~~~~~~~~~~~~~~~~~   434 (713) |
|  | T 5OGL\_A | 395 | FFNFLEKKQIKLSLRNKNILLILIAFFSISPALMHIYYYK   434 (713) |
|  | T ss\_dssp |  | HHHHHHHTTCCCCHHHHHHHHHHHHHHHHHHHHHHHHHCC |
|  | T ss\_pred |  | HHHHHHHhhcccchhHHHHHHHHHHHHHHhHHHHHHHhcC |
|  |
| --- | | | |
|  | Template alignmentTemplate 3D StructurePDBe | | |
| 7. | 6P25\_A Dolichyl-diphosphooligosaccharide--protein glycosyltransferase subunits (E.C.2.4.99.18); complex, TRANSFERASE, glycosylation; HET: NAG, CPL, NNM; 3.2A {Saccharomyces cerevisiae W303}; Related PDB entries: 6P2R\_A ; Related PDB entries: 6P2R\_A ; Related PDB entries: 6P2R\_A | | |
|  | Probability: 99.64%, E-value: 1.8e-13, Score: 135.93, Aligned cols: 249, Identities: 13%, Similarity: -0.002, | | |
|  |
|  | Q ss\_pred |  | CCcccCChhhcccccCCHHHHHHHHHHHHHHHHHhhhcCCC--cccCchHHHHhcccccCCCChhhhhcccccccccCCC |
|  | Q Q5T4D3 | 1 | MAVLDTDLDHILPSSVLPPFWAKLVVGSVAIVCFARSYDGD--FVFDDSEAIVNNKDLQAETPLGDLWHHDFWGSRLSSN   78 (462) |
|  | Q Consensus | 1 | ~~~~~~~~~~~~~~~~~~~~~~~~~l~~~~~~~~~~~~~~~--~~~Dd~~~~~~~~~~~~~~~~~~~~~~~~~~~~~~~~   78 (462) |
|  |  |  | .......+..............+++++++++.++...+..+ ..+||..+...+..+..++ |
|  | T Consensus | 31 | ~~~~~~~~~~~~~~~~~~~~~~l~~l~ll~~~lrl~~l~~~~~~~~DE~~~~~~a~~~~~g~------------------   92 (817) |
|  | T 6P25\_A | 31 | VTDPSAELASLRTMVTLKEKLLVACLAVFTAVIRLHGLAWPDSVVFDEVHFGGFASQYIRGT------------------   92 (817) |
|  | T ss\_dssp |  | CCCCCHHHHHHSSCCSHHHHHHHHHHHHHHHHHTTTTTTSSCBCCTTHHHHHHHHHHHHHCB------------------ |
|  | T ss\_pred |  | cCCccHHHHhhhccCcHHHHHHHHHHHHHHHHHHHhcccCCCcceeeHHHHHHHHHHHHhCC------------------ |
|  |
|  |
|  | Q ss\_pred |  | CCccccCchHHHHHHHHHHHhCCCCc-----------------hHHHHHHHHHHHHHHHHHHHHHHHHhccccccccccc |
|  | Q Q5T4D3 | 79 | TSHKSYRPLTVLTFRINYYLSGGFHP-----------------VGFHVVNILLHSGISVLMVDVFSVLFGGLQYTSKGRR   141 (462) |
|  | Q Consensus | 79 | ~~~~~~~Pl~~~~~~~~~~~~gg~~~-----------------~~~rl~~~~~~~l~~~l~~~l~~~l~~~~~~~~~~~~   141 (462) |
|  |  |  | .+.+.+||++.++.+....++ |.+. ...|++++++++++++++|.++|++.+ |
|  | T Consensus | 93 | ~~~~~~PPL~~ll~a~~~~l~-G~~~~~~f~~ig~~~~~~~~~~~~Rl~~~l~~~l~v~l~y~i~r~l~~----------   161 (817) |
|  | T 6P25\_A | 93 | YFMDVHPPLAKMLYAGVASLG-GFQGDFDFENIGDSFPSTTPYVLMRFFSASLGALTVILMYMTLRYSGV----------   161 (817) |
|  | T ss\_dssp |  | CCCCSSCTHHHHHHHHHHHHT-CCCSCCCCCSTTCBCCTTSCCHHHHHHHHHHHHHHHHHHHHHHHHTTC---------- |
|  | T ss\_pred |  | CCCCCCChHHHHHHHHHHHHc-CCCCCCCccccccCCCCCCHHHHHHHHHHHHHHHHHHHHHHHHHHcCC---------- |
|  |
|  |
|  | Q ss\_pred |  | cchHHHHHHHHHHHHHHCHHHHHHHHhHhcHHHHHHHHHHHHHHHHHHHHHHHcCCCCCchHHHHHHHHHHHHHHHHhch |
|  | Q Q5T4D3 | 142 | LHLAPRASLLAALLFAVHPVHTECVAGVVGRADLLCALFFLLSFLGYCKAFRESNKEGAHSSTFWVLLSIFLGAVAMLCK   221 (462) |
|  | Q Consensus | 142 | ~~~~~~~a~~aall~~~~p~~~~~~~~~~~~~~~~~~~f~ll~~~~~~~~~~~~~~~~~~~~~~~~~~~~~~~~la~l~k   221 (462) |
|  |  |  | ++..|++++++++++|.....+... ..|.+..++++++++++.+..++++.++.. +++++++++++++++.+| |
|  | T Consensus | 162 | ---~~~~Allaall~~~~p~~i~~s~~~--~~d~~~~ff~~lal~~~~~~~~~~~~~~~~--~~~l~l~gl~lgla~~tK   234 (817) |
|  | T 6P25\_A | 162 | ---RMWVALMSAICFAVENSYVTISRYI--LLDAPLMFFIAAAVYSFKKYEMYPANSLNA--YKSLLATGIALGMASSSK   234 (817) |
|  | T ss\_dssp |  | ---CHHHHHHHHHHHHSCHHHHHHHHSS--CSHHHHHHHHHHHHHHHHHHHTSCSSSHHH--HHHHHHHHHHHHHHHTTC |
|  | T ss\_pred |  | ---CHHHHHHHHHHHHHcHHHHHHHHHH--hhHHHHHHHHHHHHHHHHHHHhCCCCCcHH--HHHHHHHHHHHHHHHhhh |
|  |
|  |
|  | Q ss\_pred |  | HHHHHHHHHHHHHHHHH------hCCCChHHHHHHHhhhccchHhhchhhhhhHHHHHHHHHHHHHHHHHHHHHHhCCCC |
|  | Q Q5T4D3 | 222 | EQGITVLGLNAVFDILV------IGKFNVLEIVQKVLHKDKSLENLGMLRNGGLLFRMTLLTSGGAGMLYVRWRIMGTGP   295 (462) |
|  | Q Consensus | 222 | ~~~~~~~~~~~~~~~~~------~~~~~~~~~~~~~~~~~~~~~~~~~~~~~~~~~~~~~~~~~~~~~~~~~~~~~~~~~   295 (462) |
|  |  |  | +.++++.+.++++.++. ..+++.+...+.. ......++++........+....... |
|  | T Consensus | 235 | ~~gl~~l~~~~l~~l~~l~~~~~~~~~~~~~~~~~~------------------~~~~~~li~ip~~iy~~~~~~~f~~l   296 (817) |
|  | T 6P25\_A | 235 | WVGLFTVTWVGLLCIWRLWFMIGDLTKSSKSIFKVA------------------FAKLAFLLGVPFALYLVFFYIHFQSL   296 (817) |
|  | T ss\_dssp |  | TTHHHHHHHHHHHHHHHHHHHHHCSSSCHHHHHHHH------------------HHHHCCCCCHHHHHHHHHHHHHHHHC |
|  | T ss\_pred |  | hHHHHHHHHHHHHHHHHHHHHhCCCCCCHHHHHHHH------------------HHHHHHHHHHHHHHHHHHHHHHHHHh |
|  |
|  |
|  | Q ss\_pred |  | CCccccCC |
|  | Q Q5T4D3 | 296 | PAFTEVDN   303 (462) |
|  | Q Consensus | 296 | ~~~~~~~~   303 (462) |
|  |  |  | ........ |
|  | T Consensus | 297 | ~~~g~~~~   304 (817) |
|  | T 6P25\_A | 297 | TLDGDGAS   304 (817) |
|  | T ss\_dssp |  | CBCCSTTT |
|  | T ss\_pred |  | ccCCCCcc |
|  |
| --- | | | |
|  | Template alignmentTemplate 3D StructurePDBe | | |
| 8. | 6P25\_B Dolichyl-diphosphooligosaccharide--protein glycosyltransferase subunits (E.C.2.4.99.18); complex, TRANSFERASE, glycosylation; HET: NAG, CPL, NNM; 3.2A {Saccharomyces cerevisiae W303}; Related PDB entries: 6P2R\_B ; Related PDB entries: 6P2R\_B ; Related PDB entries: 6P2R\_B | | |
|  | Probability: 99.64%, E-value: 1.7e-13, Score: 136.37, Aligned cols: 266, Identities: 12%, Similarity: 0.086, | | |
|  |
|  | Q ss\_pred |  | CCcccCChhhcccccCCHHHHHHHHHHHHHHHHHhhhcCCCcc--cCchHHHHhcccccCCCChhhhhcccccccccCCC |
|  | Q Q5T4D3 | 1 | MAVLDTDLDHILPSSVLPPFWAKLVVGSVAIVCFARSYDGDFV--FDDSEAIVNNKDLQAETPLGDLWHHDFWGSRLSSN   78 (462) |
|  | Q Consensus | 1 | ~~~~~~~~~~~~~~~~~~~~~~~~~l~~~~~~~~~~~~~~~~~--~Dd~~~~~~~~~~~~~~~~~~~~~~~~~~~~~~~~   78 (462) |
|  |  |  | +.++..................+++++++++..+......+.. +||..+...+..+.++. |
|  | T Consensus | 46 | ~~~~~~~~~~~~~~~~~~~~~~l~~l~~~~~~~r~~~l~~~~~~~~DE~~~~~~a~~~~~~~------------------   107 (759) |
|  | T 6P25\_B | 46 | EDFSKEKPAAQSSLLRLESVVMPVIFTALALFTRMYKIGINNHVVWDEAHFGKFGSYYLRHE------------------   107 (759) |
|  | T ss\_dssp |  | -----------CCHHHHHHHHHHHHHHHHHHHHHSSSGGGSCBCCTTHHHHHHHHHHHHTTB------------------ |
|  | T ss\_pred |  | hHHhhcCCcccchhhHhHHhHHHHHHHHHHHHHHHHhcCCCCceeeeHHHHHHHHHHHHhCC------------------ |
|  |
|  |
|  | Q ss\_pred |  | CCccccCchHHHHHHHHHHHhCCCCc----------------hHHHHHHHHHHHHHHHHHHHHHHHH-hccccccccccc |
|  | Q Q5T4D3 | 79 | TSHKSYRPLTVLTFRINYYLSGGFHP----------------VGFHVVNILLHSGISVLMVDVFSVL-FGGLQYTSKGRR   141 (462) |
|  | Q Consensus | 79 | ~~~~~~~Pl~~~~~~~~~~~~gg~~~----------------~~~rl~~~~~~~l~~~l~~~l~~~l-~~~~~~~~~~~~   141 (462) |
|  |  |  | .+.+.+||+..++.+....++ |.+. ...|++++++++++++++|.++|++ . |
|  | T Consensus | 108 | ~~~~~~PPl~~~l~a~~~~l~-g~~~~~~~~~~~~~~~~~~~~~~R~~~~l~~~l~~~l~y~l~r~l~~-----------   175 (759) |
|  | T 6P25\_B | 108 | FYHDVHPPLGKMLVGLSGYLA-GYNGSWDFPSGEIYPDYLDYVKMRLFNASFSALCVPLAYFTAKAIGF-----------   175 (759) |
|  | T ss\_dssp |  | CCCCSSCTHHHHHHHHHHHTT-TCCSCSCCCSSCBCCSSCCHHHHHHHHHHHHHHHHHHHHHHHHHSCC----------- |
|  | T ss\_pred |  | CCcCCCCHHHHHHHHHHHHHh-CCCCCCCCCCCCCCCCcCCHHHHHHHHHHHHHHHHHHHHHHHHHcCC----------- |
|  |
|  |
|  | Q ss\_pred |  | cchHHHHHHHHHHHHHHCHHHHHHHHhHhcHHHHHHHHHHHHHHHHHHHHHHHcCCCCCchHHHHHHHHHHHHHHHHhch |
|  | Q Q5T4D3 | 142 | LHLAPRASLLAALLFAVHPVHTECVAGVVGRADLLCALFFLLSFLGYCKAFRESNKEGAHSSTFWVLLSIFLGAVAMLCK   221 (462) |
|  | Q Consensus | 142 | ~~~~~~~a~~aall~~~~p~~~~~~~~~~~~~~~~~~~f~ll~~~~~~~~~~~~~~~~~~~~~~~~~~~~~~~~la~l~k   221 (462) |
|  |  |  | ++..|++++++++++|.....+... ..|.+..++++++++++.+..+++++.....++.+.+++++++++++++| |
|  | T Consensus | 176 | ---~~~~allaall~~~~p~~~~~s~~~--~~d~~~~~f~~l~l~~~~~~~~~~~~~~~~~~~~~~~l~gl~lgla~~~K   250 (759) |
|  | T 6P25\_B | 176 | ---SLPTVWLMTVLVLFENSYSTLGRFI--LLDSMLLFFTVASFFSFVMFHNQRSKPFSRKWWKWLLITGISLGCTISVK   250 (759) |
|  | T ss\_dssp |  | ---CTHHHHHHHHHHHSCHHHHHHTSSS--CSHHHHHHHHHHHHHHHHHHHTTSSSTTSHHHHHHHHHHHHHHHHHHHHC |
|  | T ss\_pred |  | ---CHHHHHHHHHHHHhhhHHHHHHHHH--HHHHHHHHHHHHHHHHHHHHhhhcCCCCCHHHHHHHHHHHHHHHHHHHhH |
|  |
|  |
|  | Q ss\_pred |  | HHHHHHHHHHHHHHHHHhCCCChHHHHHHHhhhccchHhhchhhhhhHHHHHHHHHHHHHHHHHHHHHHhCCCCCCcccc |
|  | Q Q5T4D3 | 222 | EQGITVLGLNAVFDILVIGKFNVLEIVQKVLHKDKSLENLGMLRNGGLLFRMTLLTSGGAGMLYVRWRIMGTGPPAFTEV   301 (462) |
|  | Q Consensus | 222 | ~~~~~~~~~~~~~~~~~~~~~~~~~~~~~~~~~~~~~~~~~~~~~~~~~~~~~~~~~~~~~~~~~~~~~~~~~~~~~~~~   301 (462) |
|  |  |  | ..++.+.++++++.+....++. +++........+..........++..++....+..........+.... |
|  | T Consensus | 251 | ~~~~~~~~~~~l~~l~~~~~~~----------~~~~~~~~~~~~~~~~~~~~~~~~p~~i~~~~~~~~~~~~~~~g~~~~   320 (759) |
|  | T 6P25\_B | 251 | MVGLFIITMVGIYTVIDLWTFL----------ADKSMSWKTYINHWLARIFGLIIVPFCIFLLCFKIHFDLLSHSGTGDA   320 (759) |
|  | T ss\_dssp |  | GGGHHHHHHHHHHHHHHHHHHT----------TCSSSCHHHHHHHHHHHHCCCCCHHHHHHHHHHHHHHHHCCBCCTTGG |
|  | T ss\_pred |  | HHHHHHHHHHHHHHHHHHHHHh----------cCCCCCHHHHHHHHHHHHHHHHHHHHHHHHHHHHHHHHhhcCCCCCcc |
|  |
|  |
|  | Q ss\_pred |  | CCcchhcchh |
|  | Q Q5T4D3 | 302 | DNPASFADSM   311 (462) |
|  | Q Consensus | 302 | ~~~~~~~~~~   311 (462) |
|  |  |  | .....+.... |
|  | T Consensus | 321 | ~~s~~f~~~l   330 (759) |
|  | T 6P25\_B | 321 | NMPSLFQARL   330 (759) |
|  | T ss\_dssp |  | GSCHHHHHHS |
|  | T ss\_pred |  | cCChHHHHHH |
|  |
| --- | | | |
|  | Template alignmentTemplate 3D StructurePDBe | | |
| 9. | 7BVF\_A Probable arabinosyltransferase B (E.C.2.4.2.-), Probable; Mycobacterium tuberculosis, cell wall synthesis; HET: 95E, DSL, CDL;{Mycolicibacterium smegmatis MC2 155} | | |
|  | Probability: 99.59%, E-value: 2.2e-11, Score: 121.16, Aligned cols: 363, Identities: 11%, Similarity: -0.026, | | |
|  |
|  | Q ss\_pred |  | HHHHHHHHHhhhcCCCcccCchHHHHhcccccCCCChhhhhcccccccccCCCCCccccCchHHHHHHHHHHHhCCCCch |
|  | Q Q5T4D3 | 26 | VGSVAIVCFARSYDGDFVFDDSEAIVNNKDLQAETPLGDLWHHDFWGSRLSSNTSHKSYRPLTVLTFRINYYLSGGFHPV   105 (462) |
|  | Q Consensus | 26 | l~~~~~~~~~~~~~~~~~~Dd~~~~~~~~~~~~~~~~~~~~~~~~~~~~~~~~~~~~~~~Pl~~~~~~~~~~~~gg~~~~   105 (462) |
|  |  |  | .++.+.+..+.... +.+.||.++...+.+..+.+...+++..- ...+.++|+|++++..+..+ |.++. |
|  | T Consensus | 263 | ~~V~~~l~~w~~~g-p~~~DDg~~~~~Ar~~~~~G~~~n~~~~~---------~~~e~p~~lyY~lL~~W~~v--G~s~~   330 (1102) |
|  | T 7BVF\_A | 263 | AAVIATLLLWHVIG-ATSSDDGYLLTVARVAPKAGYVANYYRYF---------GTTEAPFDWYTSVLAQLAAV--STAGV   330 (1102) |
|  | T ss\_dssp |  | HHHHHHHHHTTTSC-CCCSTTHHHHHHHHHHHHHTSCBCSSSGG---------GCBCCTTCTTHHHHHHHHHH--CCCHH |
|  | T ss\_pred |  | HHHHHHHHHHHHhc-ccCCcchHHHHHHhhccccCcHHHHHHHH---------CCCCCCChHHHHHHHHHHhc--cCchH |
|  |
|  |
|  | Q ss\_pred |  | HHHHHHHHHHHHHHHHHHHHHHHHhccccccccccc---cchHHHHHHHHHHHHHHCHHHHHHHHhHhcHHHHHHHHHHH |
|  | Q Q5T4D3 | 106 | GFHVVNILLHSGISVLMVDVFSVLFGGLQYTSKGRR---LHLAPRASLLAALLFAVHPVHTECVAGVVGRADLLCALFFL   182 (462) |
|  | Q Consensus | 106 | ~~rl~~~~~~~l~~~l~~~l~~~l~~~~~~~~~~~~---~~~~~~~a~~aall~~~~p~~~~~~~~~~~~~~~~~~~f~l   182 (462) |
|  |  |  | +.|++|+++++++..+++...+.... ++ ...++..++.+++++.. .++.+.+.. ++|.+..++.+ |
|  | T Consensus | 331 | ~LRLpSvlagl~t~~ll~r~v~~~lg--------r~~~~l~~~~~a~~~aal~~la--~~l~y~~~~--Rpyal~al~~~   398 (1102) |
|  | T 7BVF\_A | 331 | WMRLPATLAGIACWLIVSRFVLRRLG--------PGPGGLASNRVAVFTAGAVFLS--AWLPFNNGL--RPEPLIALGVL   398 (1102) |
|  | T ss\_dssp |  | HHTSHHHHHHHHHHHHCCCCCHHHSC--------CSSSSSSSCSHHHHHHHHHHHH--HHTTTCCSS--SSHHHHHHHHH |
|  | T ss\_pred |  | HhHHHHHHHHHHHHHHHHHHHHHHhC--------CCCCCccccHHHHHHHHHHHHH--HHHHHhcCC--ChHHHHHHHHH |
|  |
|  |
|  | Q ss\_pred |  | HHHHHHHHHHHHcCCCCCchHHHHHHHHHHHHHHHHhchHHHHHHHHHHHHHHHHHhCCCChHHHHHHHhhhccchHhhc |
|  | Q Q5T4D3 | 183 | LSFLGYCKAFRESNKEGAHSSTFWVLLSIFLGAVAMLCKEQGITVLGLNAVFDILVIGKFNVLEIVQKVLHKDKSLENLG   262 (462) |
|  | Q Consensus | 183 | l~~~~~~~~~~~~~~~~~~~~~~~~~~~~~~~~la~l~k~~~~~~~~~~~~~~~~~~~~~~~~~~~~~~~~~~~~~~~~~   262 (462) |
|  |  |  | ++++++.+..++++ ..++.+..++.++++.+|+++++....+.+......+..+ |
|  | T Consensus | 399 | la~~~~~ra~~~~r-------~~~~al~~~~a~lal~~hptgll~laall~~~~~l~r~lr-------------------   452 (1102) |
|  | T 7BVF\_A | 399 | VTWVLVERSIALGR-------LAPAAVAIIVATLTATLAPQGLIALAPLLTGARAIAQRIR-------------------   452 (1102) |
|  | T ss\_dssp |  | HHHHHHHHHHHHTC-------SHHHHHHHHHHHHHTTSCGGGGGGGHHHHHTTHHHHHHHH------------------- |
|  | T ss\_pred |  | HHHHHHHHHHHcCC-------hHHHHHHHHHHHHHhccchHHHHHHHHHHHHHHHHHHHHH------------------- |
|  |
|  |
|  | Q ss\_pred |  | hhhhhhHHHHHHHHHHHHHHHHHHHHHHhCCCCCCccccCCcchhcchhhHhHhHHHHHHHHHHHHhhccHhhhcccccc |
|  | Q Q5T4D3 | 263 | MLRNGGLLFRMTLLTSGGAGMLYVRWRIMGTGPPAFTEVDNPASFADSMLVRAVNYNYYYSLNAWLLLCPWWLCFDWSMG   342 (462) |
|  | Q Consensus | 263 | ~~~~~~~~~~~~~~~~~~~~~~~~~~~~~~~~~~~~~~~~~~~~~~~~~~~~~~~~~~~~~~~~~~~~~p~~~~~~~~~~   342 (462) |
|  |  |  | .+.............+..........+..+....................+.+....+...+. |
|  | T Consensus | 453 | --~r~~~~~~~~~la~vla~~~~~l~~~F~dq~l~~~~~a~~~~~~~g~~~~W~~e~~Ry~~L~~---------------   515 (1102) |
|  | T 7BVF\_A | 453 | --RRRATDGLLAPLAVLAAALSLITVVVFRDQTLATVAESARIKYKVGPTIAWYQDFLRYYFLTV---------------   515 (1102) |
|  | T ss\_dssp |  | --HSCSSSCSSHHHHHHHHTGGGTHHHHSSSSCHHHHHHHHHHHHHSSCCCCGGGTTTTSTTTSC--------------- |
|  | T ss\_pred |  | --HhhhhcchHHHHHHHHHHHHHHHHHHHccCcHHHHHHHHhHHHhhCCCChhHHHHHHHHHHHh--------------- |
|  |
|  |
|  | Q ss\_pred |  | ccCcccccchHHHHHHHHHHHHHHHHHHHHHhcCCCcch--HHHHHHHHHHHHHHhhHhccccccchhHHHhhhhHHHHH |
|  | Q Q5T4D3 | 343 | CIPLIKSISDWRVIALAALWFCLIGLICQALCSEDGHKR--RILTLGLGFLVIPFLPASNLFFRVGFVVAERVLYLPSVG   420 (462) |
|  | Q Consensus | 343 | ~~~~~~~~~~~~~~~~~~~~~~~~~~~~~~~~~~~~~~~--~~~~~~~~~~~~~~~~~~~~~~~~~~~~~~ry~~~~~~~   420 (462) |
|  |  |  | .....-+.......++.++++.+......++++.... ......+.++++.++.+.+. ..+..||+....++ |
|  | T Consensus | 516 | --~~~~~Gs~arr~pvLl~l~~L~~~~~~l~Rrrr~~g~~~~~~~~ll~~~~l~lvll~~t-----PsKwt~hfg~~A~~   588 (1102) |
|  | T 7BVF\_A | 516 | --ESNVEGSMSRRFAVLVLLFCLFGVLFVLLRRGRVAGLASGPAWRLIGTTAVGLLLLTFT-----PTKWAVQFGAFAGL   588 (1102) |
|  | T ss\_dssp |  | --SSCGGGCTTTHHHHHHHHHHHHHHHHHHHSSSCCSSSCHHHHHHHHHHHHHHHHHGGGC-----SCCCSGGGGGGHHH |
|  | T ss\_pred |  | --ccCCCCCchHHHHHHHHHHHHHHHHHHHHHcCCCCCcccCHHHHHHHHHHHHHHHHhcC-----ccHHHHHHHHHHHH |
|  |
|  |
|  | Q ss\_pred |  | HHHHHHHHHHHHHhchhhHHHHHHHHHHHHHHHHHHHHHhcC |
|  | Q Q5T4D3 | 421 | YCVLLTFGFGALSKHTKKKKLIAAVVLGILFINTLRCVLRSG   462 (462) |
|  | Q Consensus | 421 | ~~ll~a~~~~~~~~~~~~~~~~~~~~~~~~~~~~~~~~~~~~   462 (462) |
|  |  |  | .+++++.+...+.+...+...........++......+...+ |
|  | T Consensus | 589 | ~~aLlA~~~v~~~~~~~r~~r~~~~~~~~~~~~~als~~g~n   630 (1102) |
|  | T 7BVF\_A | 589 | AGVLGAVTAFTFARIGLHSRRNLTLYVTALLFVLAWATSGIN   630 (1102) |
|  | T ss\_dssp |  | HHHHHHHHHHHHHHTTSSCSHHHHHHHHHHHHHHHHHTTSCC |
|  | T ss\_pred |  | HHHHHHHHHHHHhhhhhcchHHHHHHHHHHHHHHHHHHhhhc |
|  |
| --- | | | |
|  | Template alignmentTemplate 3D StructurePDBe | | |
| 10. | 6W98\_A F5/8 type C domain-containing protein; Glycosyltransferase, lipomannan, lipoarabinomannan, arabinofuranose, membrane; HET: PNS, 6OU; 2.9A {Escherichia coli (strain K12)}; Related PDB entries: 6WBX\_A 6WBY\_A | | |
|  | Probability: 99.53%, E-value: 4.1e-11, Score: 126.55, Aligned cols: 394, Identities: 13%, Similarity: 0.003, | | |
|  |
|  | Q ss\_pred |  | ChhhcccccCCHHHHHHHHHHHHHHHHHhhhcCCCcccCchHHHHhcccccCCCChhhhhcccccccccCCCCCccccCc |
|  | Q Q5T4D3 | 7 | DLDHILPSSVLPPFWAKLVVGSVAIVCFARSYDGDFVFDDSEAIVNNKDLQAETPLGDLWHHDFWGSRLSSNTSHKSYRP   86 (462) |
|  | Q Consensus | 7 | ~~~~~~~~~~~~~~~~~~~l~~~~~~~~~~~~~~~~~~Dd~~~~~~~~~~~~~~~~~~~~~~~~~~~~~~~~~~~~~~~P   86 (462) |
|  |  |  | ++.+............+++++++.+..+....+.-...+|..++.+...+.... ...++.........+..+...+| |
|  | T Consensus | 3 | ~~~~~~~~~~~~~~~ll~~~lll~~~~~~~~~g~~~~d~~~~~~~~~~~~l~~~--~~~W~~~~~~G~~~~~~~~y~~P-   79 (1413) |
|  | T 6W98\_A | 3 | VMTYRLDSSALSRRWLAVAAAVSLLLTFSQSPGQISPDTKLDLAINPLRFAARA--LNLWSSDLPFGQAQNQAYGYLFP-   79 (1413) |
|  | T ss\_dssp |  | --CCCCCCCCCCTHHHHHHHHHHHHHHTTSSTTCBCCTTCSHHHHCHHHHHHHT--TSSEESSSTTSEECCSSGGGCCC- |
|  | T ss\_pred |  | cccccCCcccchHHHHHHHHHHHHHHHHhCCCCccCCCCCCccccCHHHHHHHH--HhccCCCCCCCCCchhhhhhhhh- |
|  |
|  |
|  | Q ss\_pred |  | hHHHHHHHHHHHhCCCCchHHHHHHHHHHHHHHHHHHHHHHHHh-ccccccccccccchHHHHHHHHHHHHHHCHHHHHH |
|  | Q Q5T4D3 | 87 | LTVLTFRINYYLSGGFHPVGFHVVNILLHSGISVLMVDVFSVLF-GGLQYTSKGRRLHLAPRASLLAALLFAVHPVHTEC   165 (462) |
|  | Q Consensus | 87 | l~~~~~~~~~~~~gg~~~~~~rl~~~~~~~l~~~l~~~l~~~l~-~~~~~~~~~~~~~~~~~~a~~aall~~~~p~~~~~   165 (462) |
|  |  |  | ...++.+...+. .......|+..+++.+++.+.+|+++|++. + ++..+++++++++++|.++.. |
|  | T Consensus | 80 | -~~~~~~l~~~lg-~~~~~~~rl~~~l~~~la~~g~y~L~r~l~~~-------------~~~~al~Aal~yalsP~~l~~   144 (1413) |
|  | T 6W98\_A | 80 | -HGAFFSLGHLLG-VPAWVTQRLWWALLIVAGFWGLIRVAEALGIG-------------TRGSRIIAAVAFALSPRVLTT   144 (1413) |
|  | T ss\_dssp |  | -CCHHHHHHHHHT-CCHHHHHHHHHHHHHHHHHHHHHHHHHHHTCS-------------CTTHHHHHHHHHHTCHHHHHH |
|  | T ss\_pred |  | -HHHHHHHHHHcC-CCHHHHHHHHHHHHHHHHHHHHHHHHHHhCCC-------------ChHHHHHHHHHHHHCHHHHHH |
|  |
|  |
|  | Q ss\_pred |  | HHhHhcHHHHHHHHHHHHHHHHHHHHHHH--cCCCCCchHHHHHHHHHHHHHHHHhchHHHHHHHHHHHHHHHHHhCCCC |
|  | Q Q5T4D3 | 166 | VAGVVGRADLLCALFFLLSFLGYCKAFRE--SNKEGAHSSTFWVLLSIFLGAVAMLCKEQGITVLGLNAVFDILVIGKFN   243 (462) |
|  | Q Consensus | 166 | ~~~~~~~~~~~~~~f~ll~~~~~~~~~~~--~~~~~~~~~~~~~~~~~~~~~la~l~k~~~~~~~~~~~~~~~~~~~~~~   243 (462) |
|  |  |  | .... ..+.+..++...+++.+.+..++ ++ +++.+++++++++...++....++.+++...+++..++++ |
|  | T Consensus | 145 | ~~~~--~~~~~~~~llp~~ll~l~~~~~~~~~~-------~r~~~~~~l~~~l~~~~~~~~~~~~l~~~~l~~l~~~~~~   215 (1413) |
|  | T 6W98\_A | 145 | LGAI--SSETLPMMLAPWVLLPLILTFQGRMSP-------RRAAALSAVAVALMGAVNAVATALACGVAVIWWLAHRPNR   215 (1413) |
|  | T ss\_dssp |  | HTTC--GGGTHHHHHHHHHHHHHHHHHTTSSCH-------HHHHHHHHHHHHHSCSSSHHHHHHHSHHHHHHHHSBCCCH |
|  | T ss\_pred |  | hhcc--ChhhHHHHHHHHHHHHHHHHHcCCCCH-------HHHHHHHHHHHHHhcchhHHHHHHHHHHHHHHHHHCCCCc |
|  |
|  |
|  | Q ss\_pred |  | hHHHHHHHhhhccchHhhchhhhhhHHHHHHHHHHHHHHHHHHHHHHhCCCCCCccccCCcchhcchhhHhHhHHHHHHH |
|  | Q Q5T4D3 | 244 | VLEIVQKVLHKDKSLENLGMLRNGGLLFRMTLLTSGGAGMLYVRWRIMGTGPPAFTEVDNPASFADSMLVRAVNYNYYYS   323 (462) |
|  | Q Consensus | 244 | ~~~~~~~~~~~~~~~~~~~~~~~~~~~~~~~~~~~~~~~~~~~~~~~~~~~~~~~~~~~~~~~~~~~~~~~~~~~~~~~~   323 (462) |
|  |  |  | +.++.........+++..+++............+............... |
|  | T Consensus | 216 | ---------------------~~~~~~~~~~~~~~l~~~~wl~Pll~~~~~~~~~~~~~e~~~~~~~~~s----------   264 (1413) |
|  | T 6W98\_A | 216 | ---------------------TWWRFTAWWIPCLALASTWWIVALLIFGKISPKFLDFIESSGVTTQWTS----------   264 (1413) |
|  | T ss\_dssp |  | ---------------------HHHHHHHHHHHHHHHHHHHHHHHHHHHHHHSCCCTTSSCC-------CC---------- |
|  | T ss\_pred |  | ---------------------hHHHHHHHHHHHHHHHHHHHHHHHHHhcccCHhHHHHHhcccccccccc---------- |
|  |
|  |
|  | Q ss\_pred |  | HHHHHhhccHhhhccccccccCcccccchHHHHHHHHHHHHHHHHHHHHHhcCCCcchHHHHHHHHHHHHHHhhHhc--- |
|  | Q Q5T4D3 | 324 | LNAWLLLCPWWLCFDWSMGCIPLIKSISDWRVIALAALWFCLIGLICQALCSEDGHKRRILTLGLGFLVIPFLPASN---   400 (462) |
|  | Q Consensus | 324 | ~~~~~~~~p~~~~~~~~~~~~~~~~~~~~~~~~~~~~~~~~~~~~~~~~~~~~~~~~~~~~~~~~~~~~~~~~~~~~---   400 (462) |
|  |  |  | ..........+..+..................+....+.++.+....+++++.+......++..++....... |
|  | T Consensus | 265 | ----~~~~l~~~~~w~~~~~~~~~~~~~~~~~~~~~~~~~~l~~lgl~~l~~r~~~~~~~l~~~~l~g~~l~~~~~~~~~   340 (1413) |
|  | T 6W98\_A | 265 | ----LTEVLRGTDSWTPFVAPTATAGSSLVTQSAMVIATTMLAAAGMAGLAMRGMPARGRLVAVLLIGLVLLTAGYTGAL   340 (1413) |
|  | T ss\_dssp |  | ----HHHHHHTC-----------------CCHHHHHHHHHHHHHHHHHHHSSTTCTTHHHHHHHHHHHHHTC-------- |
|  | T ss\_pred |  | ----HHHHHhCCCCCccccCCCCcccchHHHhHHHHHHHHHHHHHHHHHHHhCCCchHHHHHHHHHHHHHHHHhcccCCC |
|  |
|  |
|  | Q ss\_pred |  | ------------cccccchhHHHhhhhHHHHHHHHHHHHHHHHH---------------HhchhhHHHHHHHHHHHHHHH |
|  | Q Q5T4D3 | 401 | ------------LFFRVGFVVAERVLYLPSVGYCVLLTFGFGAL---------------SKHTKKKKLIAAVVLGILFIN   453 (462) |
|  | Q Consensus | 401 | ------------~~~~~~~~~~~ry~~~~~~~~~ll~a~~~~~~---------------~~~~~~~~~~~~~~~~~~~~~   453 (462) |
|  |  |  | ...........||..+..++++++++.++..+ .++.+.+.....++++++++. |
|  | T Consensus | 341 | ~~p~~~~~~~~l~p~~~~~R~~~Rf~~~~~l~lall~a~~l~~l~~~~~~~~~~~~~~~~~~~~~~~~~~~~~~~~~~~~   420 (1413) |
|  | T 6W98\_A | 341 | GSPIAQQIQFFLDDGGTPLRNVHKLEPLIRLPLILGLAHALSRIPLPASVPVRQWLSALARPERNRAVAFAIVLLVALAA   420 (1413) |
|  | T ss\_dssp |  | -------------------CCGGGSHHHHHHHHHHHHHHHTSSSCCTTTSCC------------CTTTHHHHHHHHHHHH |
|  | T ss\_pred |  | CCchHHHHHHHhccCchhhcChhhhHHHHHHHHHHHHHHHHHhCCCCCCCCHHHHHHhhcChHHHHHHHHHHHHHHHHHH |
|  |
|  |
|  | Q ss\_pred |  | HHHHHHhcC |
|  | Q Q5T4D3 | 454 | TLRCVLRSG   462 (462) |
|  | Q Consensus | 454 | ~~~~~~~~~   462 (462) |
|  |  |  | ......+.. |
|  | T Consensus | 421 | ~~~p~~~~~   429 (1413) |
|  | T 6W98\_A | 421 | STSLAWTGR   429 (1413) |
|  | T ss\_dssp |  | HTHHHHTTC |
|  | T ss\_pred |  | HHHHHHhCC |
|  |
| --- | | | |
|  | Template alignmentTemplate 3D StructurePDBe | | |
| 11. | 7BVF\_B Probable arabinosyltransferase B (E.C.2.4.2.-), Probable; Mycobacterium tuberculosis, cell wall synthesis; HET: 95E, DSL, CDL;{Mycolicibacterium smegmatis MC2 155} | | |
|  | Probability: 99.52%, E-value: 8.6e-11, Score: 117.39, Aligned cols: 364, Identities: 10%, Similarity: -0.053, | | |
|  |
|  | Q ss\_pred |  | HHHHHHHHHhhhcCCCcccCchHHHHhcccccCCCChhhhhcccccccccCCCCCccccCchHHHHHHHHHHHhCCCCch |
|  | Q Q5T4D3 | 26 | VGSVAIVCFARSYDGDFVFDDSEAIVNNKDLQAETPLGDLWHHDFWGSRLSSNTSHKSYRPLTVLTFRINYYLSGGFHPV   105 (462) |
|  | Q Consensus | 26 | l~~~~~~~~~~~~~~~~~~Dd~~~~~~~~~~~~~~~~~~~~~~~~~~~~~~~~~~~~~~~Pl~~~~~~~~~~~~gg~~~~   105 (462) |
|  |  |  | ..+.+.+..+.... +.+.||.++...+.+....+...+++... ......+++++.++..+.. + |.++. |
|  | T Consensus | 281 | ~~V~~~L~~w~~~g-~~~~DDg~~~~~ar~~~~~G~~~n~~~~~---------~~~e~p~~~yY~lL~~w~~-l-G~s~~   348 (1116) |
|  | T 7BVF\_B | 281 | AVVIFGFLLWHVIG-ANSSDDGYILGMARVADHAGYMSNYFRWF---------GSPEDPFGWYYNLLALMTH-V-SDASL   348 (1116) |
|  | T ss\_dssp |  | HHHHHHHHHHHHHC-CCCSSHHHHHHHHHTTTTSSSCBCCSSSS---------SCBCCSSCSSHHHHHHHTT-T-CCCHH |
|  | T ss\_pred |  | HHHHHHHHHHHHhC-cCCCCchHHHHHhhccccccchHHHHHHh---------CCCCCccHHHHHHHHHHHH-c-CCchH |
|  |
|  |
|  | Q ss\_pred |  | HHHHHHHHHHHHHHHHHHHHHHHHhccccccccccccchHHHHHHHHHHHHHHCHHHHHHHHhHhcHHHHHHHHHHHHHH |
|  | Q Q5T4D3 | 106 | GFHVVNILLHSGISVLMVDVFSVLFGGLQYTSKGRRLHLAPRASLLAALLFAVHPVHTECVAGVVGRADLLCALFFLLSF   185 (462) |
|  | Q Consensus | 106 | ~~rl~~~~~~~l~~~l~~~l~~~l~~~~~~~~~~~~~~~~~~~a~~aall~~~~p~~~~~~~~~~~~~~~~~~~f~ll~~   185 (462) |
|  |  |  | ..|++|+++++++..+++........ ++...++..++.++++++..... +.+.. ++|.+..++.++++ |
|  | T Consensus | 349 | ~lRlpSllagl~t~~ll~r~vl~~lg--------~~~~~~~~a~~~aal~~l~~~lp--y~~~~--Rpyal~al~~~lal   416 (1116) |
|  | T 7BVF\_B | 349 | WMRLPDLAAGLVCWLLLSREVLPRLG--------PAVEASKPAYWAAAMVLLTAWMP--FNNGL--RPEGIIALGSLVTY   416 (1116) |
|  | T ss\_dssp |  | HHTSHHHHHHHHHHHHCCCCCGGGSC--------TTTSSCHHHHHHHHHHHHHHHSS--STTSS--STHHHHHHHHHHHH |
|  | T ss\_pred |  | HhhHHHHHHHHHHHHHHHHHHHHHhc--------hhhcCCHHHHHHHHHHHHHHhch--hcCCC--cHHHHHHHHHHHHH |
|  |
|  |
|  | Q ss\_pred |  | HHHHHHHHHcCCCCCchHHHHHHHHHHHHHHHHhchHHHHHHHHHHHHHHHHHhCCCChHHHHHHHhhhccchHhhchhh |
|  | Q Q5T4D3 | 186 | LGYCKAFRESNKEGAHSSTFWVLLSIFLGAVAMLCKEQGITVLGLNAVFDILVIGKFNVLEIVQKVLHKDKSLENLGMLR   265 (462) |
|  | Q Consensus | 186 | ~~~~~~~~~~~~~~~~~~~~~~~~~~~~~~la~l~k~~~~~~~~~~~~~~~~~~~~~~~~~~~~~~~~~~~~~~~~~~~~   265 (462) |
|  |  |  | +++.+..++++ ..++.+.+++.++++.+|+++++....+.+......+..+ .+ |
|  | T Consensus | 417 | ~~~~ra~~~~r-------~~~~al~~~~a~lal~~hptgl~alaall~~l~~l~r~lr--------------------~r   469 (1116) |
|  | T 7BVF\_B | 417 | VLIERSMRYSR-------LTPAALAVVTAAFTLGVQPTGLIAVAALVAGGRPMLRILV--------------------RR   469 (1116) |
|  | T ss\_dssp |  | HHHHHHHHHTC-------SSHHHHHHHHHHHHHTTCGGGCCSHHHHHHTTHHHHHHHH--------------------HH |
|  | T ss\_pred |  | HHHHHHhcCCC-------cHHHHHHHHHHHHHHhccHHHHHHHHHHHHhhHHHHHHHH--------------------Hh |
|  |
|  |
|  | Q ss\_pred |  | hhhHHHHHHHHHHHHHHHHHHHHHHhCCCCCCccccCCcchhcchhhHhHhHHHHHHHHHHHHhhccHhhhccccccccC |
|  | Q Q5T4D3 | 266 | NGGLLFRMTLLTSGGAGMLYVRWRIMGTGPPAFTEVDNPASFADSMLVRAVNYNYYYSLNAWLLLCPWWLCFDWSMGCIP   345 (462) |
|  | Q Consensus | 266 | ~~~~~~~~~~~~~~~~~~~~~~~~~~~~~~~~~~~~~~~~~~~~~~~~~~~~~~~~~~~~~~~~~~p~~~~~~~~~~~~~   345 (462) |
|  |  |  | .........+..+++.........+..+....................-.....++....-. . |
|  | T Consensus | 470 | ~~~~~~la~la~~la~~~~~l~~~F~dqsl~~~~~a~~~~~~~g~~~~W~~e~~Ry~~L~~~-----------------~   532 (1116) |
|  | T 7BVF\_B | 470 | HRLVGTLPLVSPMLAAGTVILTVVFADQTLSTVLEATRVRAKIGPSQAWYTENLRYYYLILP-----------------T   532 (1116) |
|  | T ss\_dssp |  | HTTSCSHHHHSHHHHTTTCTHHHHTSSSCHHHHHHHHHHHHHTSCCCCGGGTHHHHHGGGSS-----------------S |
|  | T ss\_pred |  | hhhcchHHHHHHHHHHHHHHHHHHHccchHHHHHHHHHHHHHhcCCchHHHHhHHHHHHhcc-----------------C |
|  |
|  |
|  | Q ss\_pred |  | cccccchHHHHHHHHHHHHHHHHHHHHHhcCCCcchHHHHHHHHHHHHHHhhHhccccccchhHHHhhhhHHHHHHHHHH |
|  | Q Q5T4D3 | 346 | LIKSISDWRVIALAALWFCLIGLICQALCSEDGHKRRILTLGLGFLVIPFLPASNLFFRVGFVVAERVLYLPSVGYCVLL   425 (462) |
|  | Q Consensus | 346 | ~~~~~~~~~~~~~~~~~~~~~~~~~~~~~~~~~~~~~~~~~~~~~~~~~~~~~~~~~~~~~~~~~~ry~~~~~~~~~ll~   425 (462) |
|  |  |  | ...+..........++.++.+.......++............+.+.++.++.+.+. ..+..||+....+..+.++ |
|  | T Consensus | 533 | ~~Gs~arr~~vLl~ll~L~~~~~~l~R~rr~~g~~~~~~~~ll~~~~~~~~ll~~t-----PtKwthhfg~~a~~~~aLl   607 (1116) |
|  | T 7BVF\_B | 533 | VDGSLSRRFGFLITALCLFTAVFIMLRRKRIPSVARGPAWRLMGVIFGTMFFLMFT-----PTKWVHHFGLFAAVGAAMA   607 (1116) |
|  | T ss\_dssp |  | SSSCHHHHHHHHHHHHHHHHHHHHHHHCSCCTTSCTTHHHHHHHHHHHHHHHTTSC-----SCCCGGGGGGGGTTHHHHH |
|  | T ss\_pred |  | CCCChHHHHHHHHHHHHHHHHHHHHHhcCCCCcccCcHHHHHHHHHHHHHHHHhcC-----chhHHHHHHHHHHHHHHHH |
|  |
|  |
|  | Q ss\_pred |  | HHHHHHHHhchhhHHHHHHHHHHHHHHHHHHHHHhcC |
|  | Q Q5T4D3 | 426 | TFGFGALSKHTKKKKLIAAVVLGILFINTLRCVLRSG   462 (462) |
|  | Q Consensus | 426 | a~~~~~~~~~~~~~~~~~~~~~~~~~~~~~~~~~~~~   462 (462) |
|  |  |  | +.......+...+.......+...+++.....+...+ |
|  | T Consensus | 608 | A~~~~~~~~~~~r~~~~r~~~~~~~~~~~ala~~g~n   644 (1116) |
|  | T 7BVF\_B | 608 | ALTTVLVSPSVLRWSRNRMAFLAALFFLLALCWATTN   644 (1116) |
|  | T ss\_dssp |  | HHHHHHTSTTTSCCHHHHHHHHHHHHHHHHHHTSSCC |
|  | T ss\_pred |  | HHHHHHhChhhcchHHHHHHHHHHHHHHHHHHHhhhc |
|  |
| --- | | | |
|  | Template alignmentTemplate 3D StructurePDBe | | |
| 12. | 7BWR\_A Integral membrane indolylacetylinositol arabinosyltransferase EmbB; Mycobacterium tuberculosis, EmbB, cryo-EM, ethambutol; HET: F8L;{Mycolicibacterium smegmatis MC2 155}; Related PDB entries: 7BVC\_B 7BVG\_B 7BWR\_B 7BX8\_B 7BX8\_A | | |
|  | Probability: 99.51%, E-value: 8e-11, Score: 116.85, Aligned cols: 358, Identities: 10%, Similarity: -0.042, | | |
|  |
|  | Q ss\_pred |  | HHHHHHHHhhhcCCCcccCchHHHHhcccccCCCChhhhhcccccccccCCCCCccccCchHHHHHHHHHHHhCCCCchH |
|  | Q Q5T4D3 | 27 | GSVAIVCFARSYDGDFVFDDSEAIVNNKDLQAETPLGDLWHHDFWGSRLSSNTSHKSYRPLTVLTFRINYYLSGGFHPVG   106 (462) |
|  | Q Consensus | 27 | ~~~~~~~~~~~~~~~~~~Dd~~~~~~~~~~~~~~~~~~~~~~~~~~~~~~~~~~~~~~~Pl~~~~~~~~~~~~gg~~~~~   106 (462) |
|  |  |  | .++...+.....-.+...||.++...+.+..+.+...+++... ...+..+++++.++..+..+ |.++.+ |
|  | T Consensus | 267 | ~~V~a~L~~w~~ig~~~~DDg~~~~~ar~~~~~G~~~n~~r~~---------~~~e~p~~~yY~lL~~w~~l--G~s~~~   335 (1082) |
|  | T 7BWR\_A | 267 | GVVVGGMAIWYVIGANSSDDGYILQMARTAEHAGYMANYFRWF---------GSPEDPFGWYYNVLALMTKV--SDASIW   335 (1082) |
|  | T ss\_dssp |  | HHHTTTTTHHHHHSCCCTTHHHHHHHHHSHHHHSSCBCCSSST---------TCBSCSSCCTHHHHHTTTTS--CCCTTT |
|  | T ss\_pred |  | HHHHHHHHHHHHhccCCCchHHHHHHHHHHhHhcchHHHHHHh---------CCCCCccHHHHHHHHHHHHh--cCChHH |
|  |
|  |
|  | Q ss\_pred |  | HHHHHHHHHHHHHHHH-----HHHHHHHhccccccccccccchHHHHHHHHHHHHHHCHHHHHHHHhHhcHHHHHHHHHH |
|  | Q Q5T4D3 | 107 | FHVVNILLHSGISVLM-----VDVFSVLFGGLQYTSKGRRLHLAPRASLLAALLFAVHPVHTECVAGVVGRADLLCALFF   181 (462) |
|  | Q Consensus | 107 | ~rl~~~~~~~l~~~l~-----~~l~~~l~~~~~~~~~~~~~~~~~~~a~~aall~~~~p~~~~~~~~~~~~~~~~~~~f~   181 (462) |
|  |  |  | .|++|+++++++..++ +.+.++..+ ++..++.+++.+.. ....+.+.. ++|.+..++. |
|  | T Consensus | 336 | lRLpS~laglat~~ll~r~v~~~lgr~~~~-------------~~~a~~~Aal~~l~--~~l~y~~~~--Rpy~l~al~~   398 (1082) |
|  | T 7BWR\_A | 336 | IRLPDLICALICWLLLSREVLPRLGPAVAG-------------SRAAMWAAGLVLLG--AWMPFNNGL--RPEGQIATGA   398 (1082) |
|  | T ss\_dssp |  | TTGGGTGGGTSSHHHHTTSSTGGGCHHHHH-------------CSHHHHHHHHHHHH--HHTTTTTSS--STTHHHHHHH |
|  | T ss\_pred |  | HHHHHHHHHHHHHHHHHHhhHHHhchhhcc-------------cHHHHHHHHHHHHH--HHHHHhcCC--chHHHHHHHH |
|  |
|  |
|  | Q ss\_pred |  | HHHHHHHHHHHHHcCCCCCchHHHHHHHHHHHHHHHHhchHHHHHHHHHHHHHH-HHHhCCCChHHHHHHHhhhccchHh |
|  | Q Q5T4D3 | 182 | LLSFLGYCKAFRESNKEGAHSSTFWVLLSIFLGAVAMLCKEQGITVLGLNAVFD-ILVIGKFNVLEIVQKVLHKDKSLEN   260 (462) |
|  | Q Consensus | 182 | ll~~~~~~~~~~~~~~~~~~~~~~~~~~~~~~~~la~l~k~~~~~~~~~~~~~~-~~~~~~~~~~~~~~~~~~~~~~~~~   260 (462) |
|  |  |  | +++.+++.+..++++ ..++.+.+++.++++.+|+++++......+.. .+.++.++ |
|  | T Consensus | 399 | ~la~~~l~ra~~~~r-------~~~~al~~~~a~lal~~hptgllalaallv~l~~l~r~~r~-----------------   454 (1082) |
|  | T 7BWR\_A | 399 | LITYVLIERAVTSGR-------LTPAALAITTAAFTLGIQPTGLIAVAALLAGGRPILRIVMR-----------------   454 (1082) |
|  | T ss\_dssp |  | HHHHHHHHHHHHHCC-------SHHHHHHHHHHHHTTSSCTTCHHHHHHHHHTHHHHHHHHHH----------------- |
|  | T ss\_pred |  | HHHHHHHHHHHhcCC-------CHHHHHHHHHHHHHHHhcHHHHHHHHHHHHccHHHHHHHhh----------------- |
|  |
|  |
|  | Q ss\_pred |  | hchhhhhhHHHHHHHHHHHHHHHHHHHHHHhCCCCCCccccCCcchhcchhhHhHhHHHHHHHHHHHHhhccHhhhcccc |
|  | Q Q5T4D3 | 261 | LGMLRNGGLLFRMTLLTSGGAGMLYVRWRIMGTGPPAFTEVDNPASFADSMLVRAVNYNYYYSLNAWLLLCPWWLCFDWS   340 (462) |
|  | Q Consensus | 261 | ~~~~~~~~~~~~~~~~~~~~~~~~~~~~~~~~~~~~~~~~~~~~~~~~~~~~~~~~~~~~~~~~~~~~~~~p~~~~~~~~   340 (462) |
|  |  |  | +.............++.........+..+....................-+....++...+.. |
|  | T Consensus | 455 | ----r~~~~~~la~la~~~aa~~~~l~~~F~dqsl~~~~~a~~~~~~~g~~~~W~~e~~Ry~~L~~~-------------   517 (1082) |
|  | T 7BWR\_A | 455 | ----RRRLVGTWPLIAPLLAAGTVILAVVFADQTIATVLEATRIRTAIGPSQEWWTENLRYYYLILP-------------   517 (1082) |
|  | T ss\_dssp |  | ----TTTTSCSHHHHHHHHHTTSSTHHHHTSSCCHHHHHHHHHHHHHHSCCTTTSGGGSSSSCCCCT------------- |
|  | T ss\_pred |  | ----hhccccHHHHHHHHHHHHHHHHHHHHhchHHHHHHHHHHHHhhcCCCccHHHhhHHHHHHHcC------------- |
|  |
|  |
|  | Q ss\_pred |  | ccccCcccccchHHHHHHHHHHHHHHHHHHHHHhcCCCcchHHHHHHHHHHHHHHhhHhccccccchhHHHhhhhHHHHH |
|  | Q Q5T4D3 | 341 | MGCIPLIKSISDWRVIALAALWFCLIGLICQALCSEDGHKRRILTLGLGFLVIPFLPASNLFFRVGFVVAERVLYLPSVG   420 (462) |
|  | Q Consensus | 341 | ~~~~~~~~~~~~~~~~~~~~~~~~~~~~~~~~~~~~~~~~~~~~~~~~~~~~~~~~~~~~~~~~~~~~~~~ry~~~~~~~   420 (462) |
|  |  |  | ....+..........+..++.+.......++............+...++.++...+. ..+..||+...... |
|  | T Consensus | 518 | ----~~~Gs~arr~~vLl~l~~L~~~~~~llR~~r~~g~~~~~~~~ll~~~~~~~~ll~~t-----PtKwthhfga~ag~   588 (1082) |
|  | T 7BWR\_A | 518 | ----TTDGAISRRVAFVFTAMCLFPSLFMMLRRKHIAGVARGPAWRLMGIIFATMFFLMFT-----PTKWIHHFGLFAAV   588 (1082) |
|  | T ss\_dssp |  | ----TCCCHHHHHHTTTTTTTSSHHHHHHHHHSSSCSSSCTTHHHHHHHHHHHHHHHTBTT-----TBCCCCCTTGGGSH |
|  | T ss\_pred |  | ----CCCCcchHHHHHHHHHHHHHHHHHHHHhhcccCCCcCCHHHHHHHHHHHHHHHHhhC-----cchHHHHHHHHHHH |
|  |
|  |
|  | Q ss\_pred |  | HHHHHHHHHHHHHhchhhHHHHHHHHHHHHHHHHHHHHHhcC |
|  | Q Q5T4D3 | 421 | YCVLLTFGFGALSKHTKKKKLIAAVVLGILFINTLRCVLRSG   462 (462) |
|  | Q Consensus | 421 | ~~ll~a~~~~~~~~~~~~~~~~~~~~~~~~~~~~~~~~~~~~   462 (462) |
|  |  |  | ...+++..+..+.....+...-...+...+++.....+...| |
|  | T Consensus | 589 | g~~l~a~a~v~l~~~~~r~~r~r~~~~~~~~~~~ala~~g~n   630 (1082) |
|  | T 7BWR\_A | 589 | GGAMAALATVLVSPTVLRSARNRMAFLSLVLFVLAFCFASTN   630 (1082) |
|  | T ss\_dssp |  | HHHHHHHHHHHSSTTTCCCHHHHHHHHHHHHHHHHHHTSSCC |
|  | T ss\_pred |  | HHHHHHHHHHHHcHHhcccHHHHHHHHHHHHHHHHHHhcccc |
|  |
| --- | | | |
|  | Template alignmentTemplate 3D StructurePDBe | | |
| 13. | 7BVE\_B Integral membrane indolylacetylinositol arabinosyltransferase EmbC; Mycobacterium smegmatis, cell wall synthesis; HET: PO4, PN7, 95E; 2.81A {Mycolicibacterium smegmatis MC2 155}; Related PDB entries: 7BVH\_B 7BVH\_A 7BVE\_A | | |
|  | Probability: 99.47%, E-value: 4.4e-10, Score: 111.73, Aligned cols: 356, Identities: 12%, Similarity: -0.001, | | |
|  |
|  | Q ss\_pred |  | HHHHHHHHhhhcCCCcccCchHHHHhcccccCCCChhhhhcccccccccCCCCCccccCchHHHHHHHHHHHhCCCCchH |
|  | Q Q5T4D3 | 27 | GSVAIVCFARSYDGDFVFDDSEAIVNNKDLQAETPLGDLWHHDFWGSRLSSNTSHKSYRPLTVLTFRINYYLSGGFHPVG   106 (462) |
|  | Q Consensus | 27 | ~~~~~~~~~~~~~~~~~~Dd~~~~~~~~~~~~~~~~~~~~~~~~~~~~~~~~~~~~~~~Pl~~~~~~~~~~~~gg~~~~~   106 (462) |
|  |  |  | .++...+-...+..+.+.||.++...+.+....+...+++..- ...+....+++.++..+.. + |.++.. |
|  | T Consensus | 261 | ~~V~~~l~~w~~ig~~~~DEg~~l~~ar~~~~~Gy~~n~~~~~---------~~~dapfg~yY~lL~~W~~-v-G~s~~~   329 (1084) |
|  | T 7BVE\_B | 261 | GLVSAMLVWWHFVGANTADDGYILTMARVSEHAGYMANYYRWF---------GTPESPFGWYYDLLALWAH-V-STASVW   329 (1084) |
|  | T ss\_dssp |  | HHHHHHHHHHHHSCCCCSSSHHHHHHHHHHHHHSSCBCSSSGG---------GCBCCSSCSSHHHHHHHTT-T-CCCHHH |
|  | T ss\_pred |  | HHHHHHHHHHHHhcccccchhHHhhhhhchhhcCchHHHHHHH---------CCCCcccHHHHHHHHHHHH-c-cccHHH |
|  |
|  |
|  | Q ss\_pred |  | HHHHHHHHHHHHHHHH-----HHHHHHHhccccccccccccchHHHHHHHHHHHHHHCHHHHHHHHhHhcHHHHHHHHHH |
|  | Q Q5T4D3 | 107 | FHVVNILLHSGISVLM-----VDVFSVLFGGLQYTSKGRRLHLAPRASLLAALLFAVHPVHTECVAGVVGRADLLCALFF   181 (462) |
|  | Q Consensus | 107 | ~rl~~~~~~~l~~~l~-----~~l~~~l~~~~~~~~~~~~~~~~~~~a~~aall~~~~p~~~~~~~~~~~~~~~~~~~f~   181 (462) |
|  |  |  | .|++++++++++..++ ..+.++..+ ++...+.+++++.. ....+.+.. ++|.+..++. |
|  | T Consensus | 330 | LRlpSll~glat~~ll~R~vl~~Lg~~~~~-------------~~~a~~~aal~fl~--~wl~y~~~~--Rpyalvalla   392 (1084) |
|  | T 7BVE\_B | 330 | MRFPTLLMGLACWWVISREVIPRLGAAAKH-------------SRAAAWTAAGLFLA--FWLPLNNGL--RPEPIIALGI   392 (1084) |
|  | T ss\_dssp |  | HHHHHHHHHHHHHHHCCCCCTTTSBSTTTS-------------CSHHHHHHHHHHHH--HHHHHCSSS--SSHHHHHHHH |
|  | T ss\_pred |  | HHHHHHHHHHHHHHHHHHHhHHHHHHHhcc-------------CHHHHHHHHHHHHH--HHHHhcCCC--ChHHHHHHHH |
|  |
|  |
|  | Q ss\_pred |  | HHHHHHHHHHHHHcCCCCCchHHHHHHHHHHHHHHHHhchHHHHHHHHHHHHHHHHHhCCCChHHHHHHHhhhccchHhh |
|  | Q Q5T4D3 | 182 | LLSFLGYCKAFRESNKEGAHSSTFWVLLSIFLGAVAMLCKEQGITVLGLNAVFDILVIGKFNVLEIVQKVLHKDKSLENL   261 (462) |
|  | Q Consensus | 182 | ll~~~~~~~~~~~~~~~~~~~~~~~~~~~~~~~~la~l~k~~~~~~~~~~~~~~~~~~~~~~~~~~~~~~~~~~~~~~~~   261 (462) |
|  |  |  | +++++++.+..++++ ..++.+++++.++++.+|+++++......+......+..+ |
|  | T Consensus | 393 | ~l~~~~~~ra~~~~r-------~~~~ala~~~a~la~~~~Ptgl~ala~ll~~~~~l~r~lr------------------   447 (1084) |
|  | T 7BVE\_B | 393 | LLTWCSVERGVATSR-------LLPVAVAIIIGALTLFSGPTGIAAVGALLVAIGPLKTIVA------------------   447 (1084) |
|  | T ss\_dssp |  | HHHHHHHHHHHHHTC-------SHHHHHHHHHHHHHHTSSGGGGGGHHHHHHTSHHHHHHHH------------------ |
|  | T ss\_pred |  | HHHHHHHHHHHcCCC-------cHHHHHHHHHHHHHHhhhHHHHHHHHHHHHHHHHHHHHHH------------------ |
|  |
|  |
|  | Q ss\_pred |  | chhhhhhHHHHHHHHHHHHHHHHHHHHHHhCCCCCCccccCCcchhcchhhHhHhHHHHHHHHHHHHhhccHhhhccccc |
|  | Q Q5T4D3 | 262 | GMLRNGGLLFRMTLLTSGGAGMLYVRWRIMGTGPPAFTEVDNPASFADSMLVRAVNYNYYYSLNAWLLLCPWWLCFDWSM   341 (462) |
|  | Q Consensus | 262 | ~~~~~~~~~~~~~~~~~~~~~~~~~~~~~~~~~~~~~~~~~~~~~~~~~~~~~~~~~~~~~~~~~~~~~~p~~~~~~~~~   341 (462) |
|  |  |  | .+.........+..++..........+..+......+.............-.....++.. |
|  | T Consensus | 448 | --~r~~~~~~la~la~~lAa~~~~l~~~Fadqsl~~~~~a~~v~~~~gp~l~w~~e~~Ry~~------------------   507 (1084) |
|  | T 7BVE\_B | 448 | --AHVSRFGYWALLAPIAAAGTVTIFLIFRDQTLAAELQASSFKSAVGPSLAWFDEHIRYSR------------------   507 (1084) |
|  | T ss\_dssp |  | --HHTTTSCSHHHHHHHHHHHHTTHHHHTSSSCHHHHHHHHHHHHHHSCCCCGGGTHHHHHH------------------ |
|  | T ss\_pred |  | --hccchhHHHHHHHHHHHHHHHHHHHHHHhhhHHHHHHHHhhhhccCCCcHhHHHHHHHHH------------------ |
|  |
|  |
|  | Q ss\_pred |  | cccCcccccchHHHHHHHHHHHHHHHHHHHHHhcCCCcch---HHHHHHHHHHHHHHhhHhccccccchhHHHhhhhHHH |
|  | Q Q5T4D3 | 342 | GCIPLIKSISDWRVIALAALWFCLIGLICQALCSEDGHKR---RILTLGLGFLVIPFLPASNLFFRVGFVVAERVLYLPS   418 (462) |
|  | Q Consensus | 342 | ~~~~~~~~~~~~~~~~~~~~~~~~~~~~~~~~~~~~~~~~---~~~~~~~~~~~~~~~~~~~~~~~~~~~~~~ry~~~~~   418 (462) |
|  |  |  | ...........--...++.++.+........++++... ......+...+..++.+.+. ..+..+|+.... |
|  | T Consensus | 508 | --l~g~~~~gs~arr~~vLl~l~~l~~~~~ll~R~rr~~g~~~~~~~~l~~~~~~~l~ll~~t-----PtKwthhfg~la   580 (1084) |
|  | T 7BVE\_B | 508 | --LFTTSPDGSVARRFAVLTLLLALAVSIAMTLRKGRIPGTALGPSRRIIGITIISFLAMMFT-----PTKWTHHFGVFA   580 (1084) |
|  | T ss\_dssp |  | --HTSSSSTTCHHHHHHHHHHHHHHHHHHHHHHHSSSCTTBCHHHHHHHHHHHHHHHHHGGGC-----SSCCSGGGGGGT |
|  | T ss\_pred |  | --HhcCCCCCCHHHHHHHHHHHHHHHHHHHHHhhcCCCCCCCCCHHHHHHHHHHHHHHHHhcC-----CchHHHHHHhHH |
|  |
|  |
|  | Q ss\_pred |  | HHHHHHHHHHHHHHHhchhhHHHHHHHHHHHHHHHHHHHHHhcC |
|  | Q Q5T4D3 | 419 | VGYCVLLTFGFGALSKHTKKKKLIAAVVLGILFINTLRCVLRSG   462 (462) |
|  | Q Consensus | 419 | ~~~~ll~a~~~~~~~~~~~~~~~~~~~~~~~~~~~~~~~~~~~~   462 (462) |
|  |  |  | +..+++++..+....+...+...........++......+...| |
|  | T Consensus | 581 | g~~~~lla~~~~~~~~~~~r~~r~~~~~~a~~~~~~ala~~G~N   624 (1084) |
|  | T 7BVE\_B | 581 | GLAGCLGALAAVAVTTTAMKSRRNRTVFGAAVLFVTALSFATVN   624 (1084) |
|  | T ss\_dssp |  | THHHHHHHHHHHTTSTTTCCCHHHHHHHHHHHHHHHHHHTSSCC |
|  | T ss\_pred |  | HHHHHHHHHHHHHHHHHhccchHHHHHHHHHHHHHHHHHhcccc |
|  |
| --- | | | |
|  | Template alignmentTemplate 3D StructurePDBe | | |
| 14. | 6SNI\_X Dolichyl pyrophosphate Man9GlcNAc2 alpha-1,3-glucosyltransferase (E.C.2.4.1.267); Glycosyltransferase, Glucosyltransferase, GT-C, N-Glycosylation, MEMBRANE; HET: PTY, Y01;{Saccharomyces cerevisiae}; Related PDB entries: 6SNH\_X | | |
|  | Probability: 99.41%, E-value: 1.2e-9, Score: 104.58, Aligned cols: 339, Identities: 11%, Similarity: 0.019, | | |
|  |
|  | Q ss\_pred |  | CCcccCChhhcccccCCHHHHHHHHHHHHHHHHHhhhcCCCc-------ccCchHHHHhcccccCCCChhhhhccccccc |
|  | Q Q5T4D3 | 1 | MAVLDTDLDHILPSSVLPPFWAKLVVGSVAIVCFARSYDGDF-------VFDDSEAIVNNKDLQAETPLGDLWHHDFWGS   73 (462) |
|  | Q Consensus | 1 | ~~~~~~~~~~~~~~~~~~~~~~~~~l~~~~~~~~~~~~~~~~-------~~Dd~~~~~~~~~~~~~~~~~~~~~~~~~~~   73 (462) |
|  |  |  | +.......................++++++++++......++ ..+|+..+....+.....+..+++.++ |
|  | T Consensus | 35 | ~~~~~~~~~~~~~~~~~~~~~~l~~i~~~~l~lR~~~~~~~~sg~~~pp~~~D~~~~~~w~~~~~~~~~~~wy~~~----   110 (562) |
|  | T 6SNI\_X | 35 | FYASPMYDFLYPFRPVGNQWLPEYIIFVCAVILRCTIGLGPYSGKGSPPLYGDFEAQRHWMEITQHLPLSKWYWYD----   110 (562) |
|  | T ss\_dssp |  | ---------------------CCSSHHHHHHHHHHHGGGSCCTTSSCSSSCCHHHHHHHHHHHHTTSCTTSTTTSC---- |
|  | T ss\_pred |  | cCCCchhHhhhccCCCCCccHHHHHHHHHHHHHHHHHhcCCCCCCCCCCCCCCHHHHHHHHHHHHhCCHHHhcccC---- |
|  |
|  |
|  | Q ss\_pred |  | ccCCCCCccccCchHHHHHHHHHHHhCC--------------------CCchHHHHHHHHHHHHHHHHH-HHHHHHHhcc |
|  | Q Q5T4D3 | 74 | RLSSNTSHKSYRPLTVLTFRINYYLSGG--------------------FHPVGFHVVNILLHSGISVLM-VDVFSVLFGG   132 (462) |
|  | Q Consensus | 74 | ~~~~~~~~~~~~Pl~~~~~~~~~~~~gg--------------------~~~~~~rl~~~~~~~l~~~l~-~~l~~~l~~~   132 (462) |
|  |  |  | .+.+..+|||+..+...+...+. + .+....|+.++++.+++...+ |.+.|+.. |
|  | T Consensus | 111 | ---~~~~~~~YPPl~~~~~~~~~~i~-~~~~~~~~~l~~~~g~~~~~~~~~~~~rl~~i~~~ll~~~~~~~~~~~~~~--   184 (562) |
|  | T 6SNI\_X | 111 | ---LQYWGLDYPPLTAFHSYLLGLIG-SFFNPSWFALEKSRGFESPDNGLKTYMRSTVIISDILFYFPAVIYFTKWLG--   184 (562) |
|  | T ss\_dssp |  | ---STTTCCCSCHHHHHHHHHHHHHH-HHHCTTTTCSSSSTTCCCTTCCSSSHHHHHHHHHHHHHTHHHHHHHHHHHH-- |
|  | T ss\_pred |  | ---ccccCCCChHHHHHHHHHHHHHH-HHhCHHHHHhcccCCCCCcchHHHHHHHHHHHHHHHHHHHHHHHHHHHHHh-- |
|  |
|  |
|  | Q ss\_pred |  | ccccccccccchHHHHHHHHHHHHHHCHHHHHHHHhHhcHHHHHHHHHHHHHHHHHHHHHHHcCCCCCchHHHHHHHHHH |
|  | Q Q5T4D3 | 133 | LQYTSKGRRLHLAPRASLLAALLFAVHPVHTECVAGVVGRADLLCALFFLLSFLGYCKAFRESNKEGAHSSTFWVLLSIF   212 (462) |
|  | Q Consensus | 133 | ~~~~~~~~~~~~~~~~a~~aall~~~~p~~~~~~~~~~~~~~~~~~~f~ll~~~~~~~~~~~~~~~~~~~~~~~~~~~~~   212 (462) |
|  |  |  | ++.+.+++.+..++++++++|..+...... ++.|.....+.+++++++.+ ++ ...+++ |
|  | T Consensus | 185 | -------~~~~~~~~~~~~~~~~~~l~P~~i~~~~~~-~q~d~~~l~l~l~al~~~~~----~~----------~~~agi   242 (562) |
|  | T 6SNI\_X | 185 | -------RYRNQSPIGQSIAASAILFQPSLMLIDHGH-FQYNSVMLGLTAYAINNLLD----EY----------YAMAAV   242 (562) |
|  | T ss\_dssp |  | -------HHHTCCHHHHHHHHHHHHCCHHHHHHHTTT-CCCHHHHHHHHHHHHHHHHH----TC----------HHHHHH |
|  | T ss\_pred |  | -------hcCCCChhHHHHHHHHHHhCHHHHHhhccc-chhHHHHHHHHHHHHHHHHC----CC----------hHHHHH |
|  |
|  |
|  | Q ss\_pred |  | HHHHHHhchHHHHHHHHHHHHHHHHHhCCCChHHHHHHHhhhccchHhhchhhhhhHHHHHHHHHHHHHHHHHHHHHHhC |
|  | Q Q5T4D3 | 213 | LGAVAMLCKEQGITVLGLNAVFDILVIGKFNVLEIVQKVLHKDKSLENLGMLRNGGLLFRMTLLTSGGAGMLYVRWRIMG   292 (462) |
|  | Q Consensus | 213 | ~~~la~l~k~~~~~~~~~~~~~~~~~~~~~~~~~~~~~~~~~~~~~~~~~~~~~~~~~~~~~~~~~~~~~~~~~~~~~~~   292 (462) |
|  |  |  | ++++|+.+|...+...++.+++.+....++++++..+ ........++.......++...+ |
|  | T Consensus | 243 | ~~~lal~~K~~~l~~~p~~~~~ll~~~~~~~~~~~~~--------------------~~~~~~~~~~~~~l~~~Pf~~~~   302 (562) |
|  | T 6SNI\_X | 243 | CFVLSICFKQMALYYAPIFFAYLLSRSLLFPKFNIAR--------------------LTVIAFATLATFAIIFAPLYFLG   302 (562) |
|  | T ss\_dssp |  | HHHHHHTTCGGGTTSHHHHHHHHHCCCCCSSCCCHHH--------------------HHHHHHHHHHHHHHHHHHHHTTT |
|  | T ss\_pred |  | HHHHHHHhhHHHHHHHHHHHHHHHHHHcCCCCCcHHH--------------------HHHHHHHHHHHHHHHHHHHHHhc |
|  |
|  |
|  | Q ss\_pred |  | CCCCCccc---------cCCcchhcchhhHhHhHHHHHHHHHHHHhhccHhhhccccccccCcccccchHHHHHHHHHHH |
|  | Q Q5T4D3 | 293 | TGPPAFTE---------VDNPASFADSMLVRAVNYNYYYSLNAWLLLCPWWLCFDWSMGCIPLIKSISDWRVIALAALWF   363 (462) |
|  | Q Consensus | 293 | ~~~~~~~~---------~~~~~~~~~~~~~~~~~~~~~~~~~~~~~~~p~~~~~~~~~~~~~~~~~~~~~~~~~~~~~~~   363 (462) |
|  |  |  | .+.....+ .+...+...+........... ..................+ |
|  | T Consensus | 303 | ~~~~~~~~~~~~~fp~~rgl~~~~~~n~w~~~~~~~~~-----------------------~~~~~~~~~~~~~~~~~~l   359 (562) |
|  | T 6SNI\_X | 303 | GGLKNIHQCIHRIFPFARGIFEDKVANFWCVTNVFVKY-----------------------KERFTIQQLQLYSLIATVI   359 (562) |
|  | T ss\_dssp |  | CSHHHHHHHHHHHSCCCCSSSCSCCSSSHHHHTTTSCG-----------------------GGTSCHHHHHHHHHHHHHH |
|  | T ss\_pred |  | CCHHHHHHHHHHHcCCCcccccchhhhHHHHHHHHHHH-----------------------HhhCCHHHHHHHHHHHHHH |
|  |
|  |
|  | Q ss\_pred |  | HHHHHHHHHHhcCCCcchHHHHHHHHHHHHHHhhHhccccccchhHHHhh-hhHHHHHHHHH |
|  | Q Q5T4D3 | 364 | CLIGLICQALCSEDGHKRRILTLGLGFLVIPFLPASNLFFRVGFVVAERV-LYLPSVGYCVL   424 (462) |
|  | Q Consensus | 364 | ~~~~~~~~~~~~~~~~~~~~~~~~~~~~~~~~~~~~~~~~~~~~~~~~ry-~~~~~~~~~ll   424 (462) |
|  |  |  | +.+.......++++++...............+.+ ..+++| +++.+|...+. |
|  | T Consensus | 360 | ~~l~~~~~~~~~~~~~~~~~~~~~~~l~~flfs~----------~vhekyill~llPl~ll~   411 (562) |
|  | T 6SNI\_X | 360 | GFLPAMIMTLLHPKKHLLPYVLIACSMSFFLFSF----------QVHEKTILIPLLPITLLY   411 (562) |
|  | T ss\_dssp |  | HHHHHHHHHHTSCCSSSHHHHHHHHHHHHHHHCS----------SCCSSCCHHHHHHHHHGG |
|  | T ss\_pred |  | HHHHHHHHHHhCCCcchHHHHHHHHHHHHHHhch----------hcCchhcHHHHHHHHHHh |
|  |
| --- | | | |
|  | Template alignmentTemplate 3D StructurePDBe | | |
| 15. | 7BVC\_A Integral membrane indolylacetylinositol arabinosyltransferase EmbA; Mycobacterium smegmatis, cell wall synthesis; HET: 95E, PNS, CDL, F8L;{Mycolicibacterium smegmatis MC2 155}; Related PDB entries: 7BVG\_A | | |
|  | Probability: 99.4%, E-value: 1.3e-9, Score: 108.13, Aligned cols: 354, Identities: 12%, Similarity: -0.026, | | |
|  |
|  | Q ss\_pred |  | HHHHHHHHHhhhcCCCcccCchHHHHhcccccCCCChhhhhcccccccccCCCCCccccCch--HHHHHHHHHHHhCCCC |
|  | Q Q5T4D3 | 26 | VGSVAIVCFARSYDGDFVFDDSEAIVNNKDLQAETPLGDLWHHDFWGSRLSSNTSHKSYRPL--TVLTFRINYYLSGGFH   103 (462) |
|  | Q Consensus | 26 | l~~~~~~~~~~~~~~~~~~Dd~~~~~~~~~~~~~~~~~~~~~~~~~~~~~~~~~~~~~~~Pl--~~~~~~~~~~~~gg~~   103 (462) |
|  |  |  | ..+.+.+..+.... +...||.++...+.+....+...+++. .......|+ ++.++..+..+ |.+ |
|  | T Consensus | 251 | ~vV~~~L~~W~~ig-p~~~DDg~~~~~ar~~~~~G~~gny~r-----------~~~~~eapf~~yY~ll~~w~~v--g~s   316 (1088) |
|  | T 7BVC\_A | 251 | TGVIGGLLIWHIVG-APTSDDGYNMTIARVASEAGYTTNYYR-----------YFGASEAPFDWYQSVLSHLASI--STA   316 (1088) |
|  | T ss\_dssp |  | HHHHHHHHSTTTSC-CCCTTHHHHHHHHHHSSSSSSCBCSSS-----------GGGCBCTTSCHHHHHHHHHTTT--CCC |
|  | T ss\_pred |  | HHHHHHHHHHHHhC-CCCcchhHHHHHHHHHHHhcCHHHHHH-----------HhcCCCcCCHHHHHHHHHHHhc--ccc |
|  |
|  |
|  | Q ss\_pred |  | chHHHHHHHHHHHHHHHHH-----HHHHHHHhccccccccccccchHHHHHHHHHHHHH--HCHHHHHHHHhHhcHHHHH |
|  | Q Q5T4D3 | 104 | PVGFHVVNILLHSGISVLM-----VDVFSVLFGGLQYTSKGRRLHLAPRASLLAALLFA--VHPVHTECVAGVVGRADLL   176 (462) |
|  | Q Consensus | 104 | ~~~~rl~~~~~~~l~~~l~-----~~l~~~l~~~~~~~~~~~~~~~~~~~a~~aall~~--~~p~~~~~~~~~~~~~~~~   176 (462) |
|  |  |  | +.+.|++++++++++..++ ..+.++..+ ++...+.+++.+. ..|+..+. ++|.+ |
|  | T Consensus | 317 | ~~~lRLPSllagl~tw~llsR~vl~~Lg~~~~~-------------~~~a~~aaal~fla~wlPy~~~~------Rpe~~   377 (1088) |
|  | T 7BVC\_A | 317 | GVWMRLPATAAAIATWLIISRCVLPRIGRRVAA-------------NRVAMLTAGATFLAAWLPFNNGL------RPEPL   377 (1088) |
|  | T ss\_dssp |  | HHHHTGGGTHHHHHHHHHCCCCCHHHHCHHHHH-------------CHHHHHHHHHHHHHHHTTTCSSS------SSHHH |
|  | T ss\_pred |  | hHHhHHHHHHHHHHHHHHHHHHHHHHHhHhhcc-------------cHHHHHHHHHHHHHHHHHhcCCC------ChHHH |
|  |
|  |
|  | Q ss\_pred |  | HHHHHHHHHHHHHHHHHHcCCCCCchHHHHHHHHHHHHHHHHhchHHHHHHHHHHHHHHHHHhCCCChHHHHHHHhhhcc |
|  | Q Q5T4D3 | 177 | CALFFLLSFLGYCKAFRESNKEGAHSSTFWVLLSIFLGAVAMLCKEQGITVLGLNAVFDILVIGKFNVLEIVQKVLHKDK   256 (462) |
|  | Q Consensus | 177 | ~~~f~ll~~~~~~~~~~~~~~~~~~~~~~~~~~~~~~~~la~l~k~~~~~~~~~~~~~~~~~~~~~~~~~~~~~~~~~~~   256 (462) |
|  |  |  | ..++.+++.+++.+..++++ ..+...+.++.+++..+|+++++....+++......+..+ |
|  | T Consensus | 378 | val~~~~a~~~~~ra~~~~r-------~~~~a~a~~~aala~~~hPtGl~a~a~ll~~~~~l~r~~r-------------   437 (1088) |
|  | T 7BVC\_A | 378 | IAFAVITVWMLVENSIGTRR-------LWPAAVAIVIAMFSVTLAPQGLIALAPLLVGARAIGRVVT-------------   437 (1088) |
|  | T ss\_dssp |  | HHHHHHHHHHHHHHHHTTTC-------SHHHHHHHHHHHHHHTTCGGGGGGGHHHHHHHHHHHHHHG------------- |
|  | T ss\_pred |  | HHHHHHHHHHHHHHHhcCCC-------cHHHHHHHHHHHHHHhhcHHHHHHHHHHHHHHHHHHHHHH------------- |
|  |
|  |
|  | Q ss\_pred |  | chHhhchhhhhhHHHHHHHHHHHHHHHHHHHHHHhCCCCCCccccCCcchhcchhhHhHhHHHHHHHHHHHHhhccHhhh |
|  | Q Q5T4D3 | 257 | SLENLGMLRNGGLLFRMTLLTSGGAGMLYVRWRIMGTGPPAFTEVDNPASFADSMLVRAVNYNYYYSLNAWLLLCPWWLC   336 (462) |
|  | Q Consensus | 257 | ~~~~~~~~~~~~~~~~~~~~~~~~~~~~~~~~~~~~~~~~~~~~~~~~~~~~~~~~~~~~~~~~~~~~~~~~~~~p~~~~   336 (462) |
|  |  |  | .+.+.......+..++..........+..+......+.............-+....+| ...+. |
|  | T Consensus | 438 | -------~r~~~~~~~a~~a~~laa~~~~l~~~F~dqtl~~~~~a~r~~~~~gp~~~w~~E~~RY------~~L~~----   500 (1088) |
|  | T 7BVC\_A | 438 | -------ARRAGTGILASLAPLAASVAVVFVIIFRDQTLATVAESVRIKYVVGPTIPWYQEFLRY------YFLTV----   500 (1088) |
|  | T ss\_dssp |  | -------GGTTTTCSHHHHHHHHHHHTTHHHHHTTSSCHHHHHHHHHHHHHHSCCCCTTCCTHHH------HHHSC---- |
|  | T ss\_pred |  | -------HhhccccHHHHHHHHHHHHHHHHHHHhCCCcHHHHHHHHhHHhhhCCCChHHchhHHH------HHHHc---- |
|  |
|  |
|  | Q ss\_pred |  | ccccccccCcccccchHHHHHHHHHHHHHHHHHHHHHhcCCCcch--HHHHHHHHHHHHHHhhHhccccccchhHHHhhh |
|  | Q Q5T4D3 | 337 | FDWSMGCIPLIKSISDWRVIALAALWFCLIGLICQALCSEDGHKR--RILTLGLGFLVIPFLPASNLFFRVGFVVAERVL   414 (462) |
|  | Q Consensus | 337 | ~~~~~~~~~~~~~~~~~~~~~~~~~~~~~~~~~~~~~~~~~~~~~--~~~~~~~~~~~~~~~~~~~~~~~~~~~~~~ry~   414 (462) |
|  |  |  | .....-....-...++.++++++......++++.... ..........++.++.+.+. ..+..+|+ |
|  | T Consensus | 501 | --------~~~~~Gs~arR~~VLl~l~~l~~~~~~l~rrrr~~g~~~~~~~~l~~~~~~~~~lL~~t-----PtKWthHf   567 (1088) |
|  | T 7BVC\_A | 501 | --------EDSVDGSLTRRFAVLVLLLCLFGLIMVLLRRGRVPGAVSGPLWRLCGSTAIGLLLLILT-----PTKWAIQF   567 (1088) |
|  | T ss\_dssp |  | --------SSCSSSCTTHHHHHHHHHHHHHHHHHHHHHSSCCTTSCHHHHHHHHHHHHHHHHHTTSC-----SCCCSGGG |
|  | T ss\_pred |  | --------cCCCCCCHHHHHHHHHHHHHHHHHHHHHHhcCCCCCCCccHHHHHHHHHHHHHHHHHHC-----cchHHHHH |
|  |
|  |
|  | Q ss\_pred |  | hHHHHHHHHHHHHHHHHHHhchhhHHHHHHHHHHHHHHHHHHHHHhcC |
|  | Q Q5T4D3 | 415 | YLPSVGYCVLLTFGFGALSKHTKKKKLIAAVVLGILFINTLRCVLRSG   462 (462) |
|  | Q Consensus | 415 | ~~~~~~~~ll~a~~~~~~~~~~~~~~~~~~~~~~~~~~~~~~~~~~~~   462 (462) |
|  |  |  | -...+..+.+++.......+...+...........++......+...| |
|  | T Consensus | 568 | Ga~Ag~gaal~a~~~va~~~~~~r~~r~~~~~~a~~~~~~alaf~G~N   615 (1088) |
|  | T 7BVC\_A | 568 | GAFAGLAGALGGVTAFAFARVGLHSRRNLALYVTALLFILAWATSGLN   615 (1088) |
|  | T ss\_dssp |  | GGGHHHHHHHHHHHHHHHHHHTTSCHHHHHHHHHHHHHHHHHHTTSCC |
|  | T ss\_pred |  | HHHHHHHHHHHHHHHHHHHHhccChHHHHHHHHHHHHHHHHHHhhccc |
|  |
| --- | | | |
|  | Template alignmentTemplate 3D StructurePDBe | | |
| 16. | 6P25\_B Dolichyl-diphosphooligosaccharide--protein glycosyltransferase subunits (E.C.2.4.99.18); complex, TRANSFERASE, glycosylation; HET: NAG, CPL, NNM; 3.2A {Saccharomyces cerevisiae W303}; Related PDB entries: 6P2R\_B ; Related PDB entries: 6P2R\_B ; Related PDB entries: 6P2R\_B | | |
|  | Probability: 65.59%, E-value: 81, Score: 32.48, Aligned cols: 104, Identities: 10%, Similarity: -0.006, | | |
|  |
|  | Q ss\_pred |  | chHHHHHHHHHHHHHHHHHHHHHhcCCCcchHHH------------HHHHHHHHHHHhhHhccccccchhHHHhhhhHHH |
|  | Q Q5T4D3 | 351 | SDWRVIALAALWFCLIGLICQALCSEDGHKRRIL------------TLGLGFLVIPFLPASNLFFRVGFVVAERVLYLPS   418 (462) |
|  | Q Consensus | 351 | ~~~~~~~~~~~~~~~~~~~~~~~~~~~~~~~~~~------------~~~~~~~~~~~~~~~~~~~~~~~~~~~ry~~~~~   418 (462) |
|  |  |  | ++...+...+.++...+..+....+.+++..... .+.++..++.++|+.... .....+|.+|++ |
|  | T Consensus | 607 | Np~iww~~~~~~~~~~~~~~~~~~~~~r~~~~~~~~~~~~~~~~~~~~~~~g~~~~ylP~~~~~----R~~fl~hYlpal   682 (759) |
|  | T 6P25\_B | 607 | TPASTWASSVAVLAFMATVVILLIRWQRQYVDLRNPSNWNVFLMGGFYPLLAWGLHYMPFVIMS----RVTYVHHYLPAL   682 (759) |
|  | T ss\_dssp |  | CTTHHHHHHHHHHHHHHHHHHHHHHHHTTCCCCCSHHHHHCCCCCCCHHHHHHHHHHHHHHHSC----SCBCGGGHHHHH |
|  | T ss\_pred |  | CHHHHHHHHHHHHHHHHHHHHHHHHHhcCCCCCCCchhHHHHHHHhHHHHHHHHHhcchHHccc----CcccHHHHHHHH |
|  |
|  |
|  | Q ss\_pred |  | HHHHHHHHHHHHHHHhch------hhHHHHHHHHHHHHHHHHHHHH |
|  | Q Q5T4D3 | 419 | VGYCVLLTFGFGALSKHT------KKKKLIAAVVLGILFINTLRCV   458 (462) |
|  | Q Consensus | 419 | ~~~~ll~a~~~~~~~~~~------~~~~~~~~~~~~~~~~~~~~~~   458 (462) |
|  |  |  | ++.+++++..+..+.++. +..+.....+++++++.....+ |
|  | T Consensus | 683 | ~f~il~~~~~~~~~~~~~~~~~~~~~~~~~~~~~~~~~~~~~f~~~   728 (759) |
|  | T 6P25\_B | 683 | YFALIILAYCFDAGLQKWSRSKCGRIMRFVLYAGFMALVIGCFWYF   728 (759) |
|  | T ss\_dssp |  | HHHHHHHHHHHHTSSSGGGGSHHHHHHHHHHHHHHHHHHHHHHHHT |
|  | T ss\_pred |  | HHHHHHHHHHHHHHHHhcccccccHHHHHHHHHHHHHHHHHHHHHH |
|  |
| --- | | | |
|  | Template alignmentTemplate 3D StructurePDBe | | |
| 17. | 6P25\_A Dolichyl-diphosphooligosaccharide--protein glycosyltransferase subunits (E.C.2.4.99.18); complex, TRANSFERASE, glycosylation; HET: NAG, CPL, NNM; 3.2A {Saccharomyces cerevisiae W303}; Related PDB entries: 6P2R\_A ; Related PDB entries: 6P2R\_A ; Related PDB entries: 6P2R\_A | | |
|  | Probability: 42.61%, E-value: 460, Score: 27.55, Aligned cols: 104, Identities: 8%, Similarity: -0.1, | | |
|  |
|  | Q ss\_pred |  | chHHHHHHHHHHHHHHHHHHHHHhcCCCcchH-----------HHHHHHHHHHHHHhhHhccccccchhHHHhhhhHHHH |
|  | Q Q5T4D3 | 351 | SDWRVIALAALWFCLIGLICQALCSEDGHKRR-----------ILTLGLGFLVIPFLPASNLFFRVGFVVAERVLYLPSV   419 (462) |
|  | Q Consensus | 351 | ~~~~~~~~~~~~~~~~~~~~~~~~~~~~~~~~-----------~~~~~~~~~~~~~~~~~~~~~~~~~~~~~ry~~~~~~   419 (462) |
|  |  |  | ++...+...+.++..+........+.++.... .....++..++-++|+.... .....++.+|+++ |
|  | T Consensus | 586 | Np~~ww~~~~~~~~~~~~~~~~~~~~~~~~~~~~~~~~~~~~~~~~~~~~gw~~hy~Pf~~~~----R~~fl~hYlpal~   661 (817) |
|  | T 6P25\_A | 586 | NAIVWWAVTAFIGIFGLIVITELFSWQLGKPILKDSKVVNFHVQVIHYLLGFAVHYAPSFLMQ----RQMFLHHYLPAYY   661 (817) |
|  | T ss\_dssp |  | CHHHHHHHHHHHHHHHHHHHHHHHHHHHTCCCCCSHHHHHHHHHHHHHHHHHHHTTGGGTSCC----SCCCGGGSHHHHH |
|  | T ss\_pred |  | cHHHHHHHHHHHHHHHHHHHHHHHHHHcCCCCCCchhHHHHHHHHHHHHHHHHHHHHHHHHcc----CccchhhHHHHHH |
|  |
|  |
|  | Q ss\_pred |  | HHHHHHHHHHHHHHhc----hhhHHHHHHHHHHHHHHHHHHHH |
|  | Q Q5T4D3 | 420 | GYCVLLTFGFGALSKH----TKKKKLIAAVVLGILFINTLRCV   458 (462) |
|  | Q Consensus | 420 | ~~~ll~a~~~~~~~~~----~~~~~~~~~~~~~~~~~~~~~~~   458 (462) |
|  |  |  | +.+++++..+..+.+. .+......+++.+++++.....+ |
|  | T Consensus | 662 | F~il~~~~~~~~~~~~~~~~~~~~~~~~~~~~~~~~~~~f~~~   704 (817) |
|  | T 6P25\_A | 662 | FGILALGHALDIIVSYVFRSKRQMGYAVVITFLAASVYFFKSF   704 (817) |
|  | T ss\_dssp |  | HHHHHHHHHHHHHHHTTTSSCHHHHHHHHHHHHHHHHHHHHHS |
|  | T ss\_pred |  | HHHHHHHHHHHHHHHHhccccchHHHHHHHHHHHHHHHHHHHh |
|  |

---

If you use HHpred on our Toolkit for your research, please cite as appropriate:

A Completely Reimplemented MPI Bioinformatics Toolkit
with a New HHpred Server at its Core.  
Zimmermann L, Stephens A, Nam SZ, Rau D,
Kübler J, Lozajic M, Gabler F, Söding J, Lupas AN, Alva V.
J Mol Biol. 2018 Jul 20. S0022-2836(17)30587-9.

  

Protein homology detection by HMM-HMM comparison.  
Söding J. Bioinformatics. 2005 Apr 1;21(7):951-60.  
  
Fast and accurate automatic structure prediction with HHpred.  
Hildebrand A, Remmert M, Biegert A, Söding J. Proteins. 2009;77 Suppl 9:128-32.  
  
Automatic Prediction of Protein 3D Structures by Probabilistic Multi-template Homology Modeling.  
Meier A, Söding J. PLoS Comput Biol. 2015 Oct 23;11(10):e1004343.

Download

---

If you use HHpred on our Toolkit for your research, please cite as appropriate:

A Completely Reimplemented MPI Bioinformatics Toolkit
with a New HHpred Server at its Core.  
Zimmermann L, Stephens A, Nam SZ, Rau D,
Kübler J, Lozajic M, Gabler F, Söding J, Lupas AN, Alva V.
J Mol Biol. 2018 Jul 20. S0022-2836(17)30587-9.

  

Protein homology detection by HMM-HMM comparison.  
Söding J. Bioinformatics. 2005 Apr 1;21(7):951-60.  
  
Fast and accurate automatic structure prediction with HHpred.  
Hildebrand A, Remmert M, Biegert A, Söding J. Proteins. 2009;77 Suppl 9:128-32.  
  
Automatic Prediction of Protein 3D Structures by Probabilistic Multi-template Homology Modeling.  
Meier A, Söding J. PLoS Comput Biol. 2015 Oct 23;11(10):e1004343.

Loading...

---

If you use HHpred on our Toolkit for your research, please cite as appropriate:

A Completely Reimplemented MPI Bioinformatics Toolkit
with a New HHpred Server at its Core.  
Zimmermann L, Stephens A, Nam SZ, Rau D,
Kübler J, Lozajic M, Gabler F, Söding J, Lupas AN, Alva V.
J Mol Biol. 2018 Jul 20. S0022-2836(17)30587-9.

  

Protein homology detection by HMM-HMM comparison.  
Söding J. Bioinformatics. 2005 Apr 1;21(7):951-60.  
  
Fast and accurate automatic structure prediction with HHpred.  
Hildebrand A, Remmert M, Biegert A, Söding J. Proteins. 2009;77 Suppl 9:128-32.  
  
Automatic Prediction of Protein 3D Structures by Probabilistic Multi-template Homology Modeling.  
Meier A, Söding J. PLoS Comput Biol. 2015 Oct 23;11(10):e1004343.

Loading hits...

---

If you use HHpred on our Toolkit for your research, please cite as appropriate:

A Completely Reimplemented MPI Bioinformatics Toolkit
with a New HHpred Server at its Core.  
Zimmermann L, Stephens A, Nam SZ, Rau D,
Kübler J, Lozajic M, Gabler F, Söding J, Lupas AN, Alva V.
J Mol Biol. 2018 Jul 20. S0022-2836(17)30587-9.

  

Protein homology detection by HMM-HMM comparison.  
Söding J. Bioinformatics. 2005 Apr 1;21(7):951-60.  
  
Fast and accurate automatic structure prediction with HHpred.  
Hildebrand A, Remmert M, Biegert A, Söding J. Proteins. 2009;77 Suppl 9:128-32.  
  
Automatic Prediction of Protein 3D Structures by Probabilistic Multi-template Homology Modeling.  
Meier A, Söding J. PLoS Comput Biol. 2015 Oct 23;11(10):e1004343.

Loading hits...

---

If you use HHpred on our Toolkit for your research, please cite as appropriate:

A Completely Reimplemented MPI Bioinformatics Toolkit
with a New HHpred Server at its Core.  
Zimmermann L, Stephens A, Nam SZ, Rau D,
Kübler J, Lozajic M, Gabler F, Söding J, Lupas AN, Alva V.
J Mol Biol. 2018 Jul 20. S0022-2836(17)30587-9.

  

Protein homology detection by HMM-HMM comparison.  
Söding J. Bioinformatics. 2005 Apr 1;21(7):951-60.  
  
Fast and accurate automatic structure prediction with HHpred.  
Hildebrand A, Remmert M, Biegert A, Söding J. Proteins. 2009;77 Suppl 9:128-32.  
  
Automatic Prediction of Protein 3D Structures by Probabilistic Multi-template Homology Modeling.  
Meier A, Söding J. PLoS Comput Biol. 2015 Oct 23;11(10):e1004343.

- Help
- FAQ
- Privacy Policy
- Imprint
- Contact Us
- Cite Us
- Recent Updates

© 2008-2020, Dept. of Protein Evolution, Max Planck Institute for Developmental Biology, Tübingen

Template 3D Structure: 
×

Loading...
