# Supplementary material for: Conserved sequence motifs in human TMTC1, TMTC2, TMTC3, and TMTC4, new O-mannosyltransferases from the GT-C/PMT clan, are rationalized as ligand binding sites
Source: Biol Direct. 2021 Jan 12;16:4. doi: 10.1186/s13062-021-00291-w (PMC7801869; doi:10.1186/s13062-021-00291-w)
Supplement: Supplementary file 3 — Additional file 3. HHPred outputs when searching TMTCs against Pfam or PDB structures. The compressed library file AF3-2020-06-HHPred-TMTCs.zip contains the outputs when running the four human TMTC sequences as input of HHPred against PDB sequences and against Pfam domains (as of 23rd of June 2020). [file 13062_2021_291_MOESM3_ESM.zip › AF3-2020-06-HHPred-TMTCs/HHpred_TMTC4_Pfam.html]

(\*) HHpred | Bioinformatics Toolkit          **We're sorry but the Toolkit doesn't work properly without JavaScript enabled. Please enable it to continue.**

Sign In

- Search
- Alignment
- Sequence Analysis
- 2ary Structure
- 3ary Structure
- Classification
- Utils

- HHblits
- HHpred
- HMMER
- PatternSearch
- ProtBLAST/PSI-BLAST

Nothing found.

###### Tools

###### Jobs

ID

Date

Tool

5863267HHPR5407837HHPR8665047HHPR2161064HHPR

# HHpred

Job ID: 5863267,Created: 16 minutes ago

- Input
- Parameters
- Results
- Raw Output
- Probability Plot
- Query Template MSA
- Query MSA

>sp|Q5T4D3|TMTC4\_HUMAN 1..462
MAVLDTDLDHILPSSVLPPFWAKLVVGSVAIVCFARSYDGDFVFDDSEAIVNNKDLQAET
PLGDLWHHDFWGSRLSSNTSHKSYRPLTVLTFRINYYLSGGFHPVGFHVVNILLHSGISV
LMVDVFSVLFGGLQYTSKGRRLHLAPRASLLAALLFAVHPVHTECVAGVVGRADLLCALF
FLLSFLGYCKAFRESNKEGAHSSTFWVLLSIFLGAVAMLCKEQGITVLGLNAVFDILVIG
KFNVLEIVQKVLHKDKSLENLGMLRNGGLLFRMTLLTSGGAGMLYVRWRIMGTGPPAFTE
VDNPASFADSMLVRAVNYNYYYSLNAWLLLCPWWLCFDWSMGCIPLIKSISDWRVIALAA
LWFCLIGLICQALCSEDGHKRRILTLGLGFLVIPFLPASNLFFRVGFVVAERVLYLPSVG
YCVLLTFGFGALSKHTKKKKLIAAVVLGILFINTLRCVLRSG

Paste ExampleUpload File

Protein FASTA

Align two sequences/MSAs

Select structural/domain databases

Pfam-A\_v33.1

- PDB\_mmCIF70\_29\_May (default)
- PDB\_mmCIF30\_29\_May
- SCOPe70\_2.07
- ECOD\_ECOD\_F70\_20200207
- COG\_KOG\_v1.0
- Pfam-A\_v33.1
- NCBI\_Conserved\_Domains(CD)\_v3.18
- SMART\_v6.0
- TIGRFAMs\_v15.0
- PRK\_v6.9
- No elements found. Consider changing the search query.
- List is empty.

Select proteomes

Select options

- Euk\_Arabidopsis\_thaliana\_TAIR10\_20\_Jun\_2017
- Euk\_Bombyx\_mori\_p50T\_Dazao\_06\_May\_2019
- Euk\_Brachypodium\_distachyon\_23\_Aug\_2017
- Euk\_Caenorhabditis\_elegans\_18\_Jul\_2017
- Euk\_Capsaspora\_owczarzaki\_ATCC\_30864\_23\_Mar\_2020
- Euk\_Chaetomium\_thermophilum\_29\_Jun\_2017
- Euk\_Chlamydomonas\_reinhardtii\_27\_Jul\_2017
- Euk\_Entamoeba\_histolytica\_HM1\_IMSS\_22\_Mar\_2017
- Euk\_Dictyostelium\_discoideum\_AX4\_19\_Sep\_2017
- Euk\_Drosophila\_melanogaster\_19\_Jul\_2017
- Euk\_Giardia\_lamblia\_ATCC\_50803\_31\_Aug\_2017
- Euk\_Homo\_sapiens\_04\_Jul\_2017
- Euk\_Physcomitrella\_patens\_28\_Aug\_2017
- Euk\_Plasmodium\_falciparum\_3D7\_7\_Jun\_2017
- Euk\_Saccharomyces\_cerevisiae\_S288c\_11\_Mar\_2017
- Euk\_Schizosaccharomyces\_pombe\_19\_Sep\_2017
- Euk\_Solanum\_lycopersicum\_28\_Jul\_2019
- Euk\_Tetrahymena\_thermophila\_SB210\_22\_Aug\_2017
- Euk\_Toxoplasma\_gondii\_ME49\_10\_May\_2018
- Euk\_Trichomonas\_vaginalis\_G3\_21\_Nov\_2018
- Euk\_Trypanosoma\_brucei\_gambiense\_DAL972\_28\_Mar\_2017
- Euk\_Ustilago\_maydis\_521\_29\_May\_2017
- Euk\_Paramecium\_tetraurelia\_9\_Dec\_2018
- Arc\_Archaeoglobus\_fulgidus\_DSM\_4304\_5\_Dec\_2017
- Arc\_Halobacterium\_jilantaiense\_5\_Dec\_2017
- Arc\_Lokiarchaeum\_sp\_GC14\_75\_31\_Oct\_2018
- Arc\_Methanocaldococcus\_jannaschii\_DSM\_2661\_5\_Dec\_2017
- Arc\_Methanosarcina\_mazei\_S\_6\_17\_Mar\_2017
- Arc\_Methanothermus\_fervidus\_DSM\_2088\_5\_Dec\_2017
- Arc\_Pyrococcus\_horikoshii\_OT3\_5\_Dec\_2017
- Arc\_Sulfolobus\_solfataricus\_5\_Dec\_2017
- Arc\_Thermoplasma\_acidophilum\_DSM\_1728\_7\_Dec\_2017
- Bac\_Acinetobacter\_baumannii\_29\_Mar\_2018
- Bac\_Aquifex\_aeolicus\_VF5\_19\_Sep\_2017
- Bac\_Bacillus\_subtilis\_subsp\_subtilis\_str168\_19\_Mar\_2017
- Bac\_Bacteriovorax\_sp\_DB6\_IX\_1\_Jun\_2018
- Bac\_Bdellovibrio\_bacteriovorus\_HD100\_1\_Jun\_2018
- Bac\_Christensenella\_minuta\_2\_Apr\_2019
- Bac\_Deinococcus\_radiodurans\_R1\_19\_Sep\_2017
- Bac\_Enterococcus\_faecalis\_13\_SD\_W\_01\_1\_Jun\_2018
- Bac\_Escherichia\_coli\_K12\_07\_Mar\_2017
- Bac\_Fischerella\_muscicola\_PCC\_7414\_24\_Sep\_2017
- Bac\_Frankia\_alni\_ACN14a\_24\_Sep\_2017
- Bac\_Helicobacter\_pylori\_26695\_1\_Jun\_2018
- Bac\_Leptospira\_interrogans\_serovar\_Lai\_str56601\_1\_Jun\_2018
- Bac\_Mycobacterium\_tuberculosis\_H37Rv\_27\_May\_2017
- Bac\_Neisseria\_gonorrhoeae\_FA\_1090\_1\_Jun\_2018
- Bac\_Neisseria\_meningitidis\_MC58\_9\_Jun\_2017
- Bac\_Nostoc\_punctiforme\_PCC\_73102\_18\_Mar\_2017
- Bac\_Phycisphaerae\_bacterium\_L21\_RPulD3\_1\_Jun\_2018
- Bac\_Plesiocystis\_pacifica\_SIR1\_1\_Jun\_2018
- Bac\_Pseudomonas\_aeruginosa\_PAO1\_5\_Jun\_2017
- Bac\_Salmonella\_ent\_ser\_Typhi\_CT18\_22\_Nov\_2018
- Bac\_Staphylococcus\_aureus\_subsp\_aureus\_NCTC\_8325\_13\_Jun\_2017
- Bac\_Streptomyces\_scabiei\_87.22\_24\_Sep\_2017
- Bac\_Synechocystis\_sp\_PCC\_6803\_6\_Jun\_2017
- Bac\_Tenacibaculum\_dicentrarchi\_27\_Nov\_2017
- Bac\_Tenacibaculum\_maritimum\_NBRC\_15946\_27\_Nov\_2017
- Bac\_Thermus\_aquaticus\_Y51MC23\_24\_Sep\_2017
- Bac\_Thermus\_thermophilus\_HB8\_19\_Sep\_2017
- Bac\_Waddlia\_chondrophila\_WSU\_86\_1044\_1\_Jun\_2018
- Bac\_Yersinia\_pestis\_CO92\_10\_Apr\_2017
- Vir\_SARS-CoV-2\_31\_Mar\_2020
- No elements found. Consider changing the search query.
- List is empty.

ResubmitReset

MSA generation method

HHblits=>UniRef30

- HHblits=>UniRef30 (default)
- PSI-BLAST=>nr70
- No elements found. Consider changing the search query.
- List is empty.

Maximal no. of MSA generation steps

3

- 0
- 1
- 2
- 3 (default)
- 4
- 5
- 8
- No elements found. Consider changing the search query.
- List is empty.

E-value incl. threshold for MSA generation

1e-3

- 0.1
- 0.05
- 0.02
- 0.01
- 1e-3 (default)
- 1e-6
- 1e-8
- 1e-10
- 1e-15
- 1e-20
- 1e-30
- 1e-40
- 1e-50
- No elements found. Consider changing the search query.
- List is empty.

Min. seq. identity of MSA hits with query (%)

0

- 0 (default)
- 10
- 20
- 30
- 40
- 50
- 60
- 70
- 75
- 80
- 85
- 90
- 95
- 100
- No elements found. Consider changing the search query.
- List is empty.

Min. coverage of MSA hits (%)

20

- 10
- 20 (default)
- 30
- 40
- 50
- 60
- 70
- 80
- 90
- 100
- No elements found. Consider changing the search query.
- List is empty.

Secondary structure scoring

during\_alignment

- none
- after\_alignment
- during\_alignment (default)
- after\_alignment\_pred\_vs\_pred
- during\_alignment\_pred\_vs\_pred
- No elements found. Consider changing the search query.
- List is empty.

Alignment Mode:Realign with MAC

local:norealign

- local:norealign (default)
- local:realign
- global:realign
- No elements found. Consider changing the search query.
- List is empty.

MAC realignment threshold

0.3

- 0.0
- 0.01
- 0.1
- 0.2
- 0.3 (default)
- 0.4
- 0.5
- 0.6
- 0.7
- 0.8
- 0.9
- 0.95
- No elements found. Consider changing the search query.
- List is empty.

No. of target sequences (up to 10000)

250

- 250 (default)
- 500
- 1000
- 2000
- 3000
- 4000
- 5000
- 6000
- 7000
- 8000
- 9000
- 10000
- No elements found. Consider changing the search query.
- List is empty.

Min. probability in hit list (> 10%)

20

- 10
- 20 (default)
- 30
- 40
- 50
- 60
- 70
- 75
- 80
- 85
- 90
- 95
- 100
- No elements found. Consider changing the search query.
- List is empty.

ResubmitReset

VisHitsAln
Select AllForwardForward Query A3MDownload HHRColor SeqsWrap Seqs

Number of Hits: **33**

Detected sequence features:
**◾Transmembrane segment(s)**

#### Visualization

Resubmit Section

22

462

Prob=98.6% E=1.5E-06 PF09594.11 ; GT87 ; Glycosyltransferase family 87

#### Hitlist

Show102550100AllEntries

Search:

| Nr (Click to sort Ascending) | Hit (Click to sort Ascending) | Name (Click to sort Ascending) | Probability (Click to sort Ascending) | E-value (Click to sort Ascending) | SS (Click to sort Ascending) | Cols (Click to sort Ascending) | Target Length (Click to sort Ascending) |
| --- | --- | --- | --- | --- | --- | --- | --- |
| 1 | PF03901.18 | ; Glyco\_transf\_22 ; Alg9-like mannosyltransferase family | 99.86 | 6.4e-19 | 31.6 | 345 | 388 |
| 2 | PF02516.15 | ; STT3 ; Oligosaccharyl transferase STT3 subunit | 99.83 | 9.5e-18 | 32.1 | 369 | 458 |
| 3 | PF09852.10 | ; DUF2079 ; Predicted membrane protein (DUF2079) | 99.78 | 6.6e-16 | 33.5 | 328 | 519 |
| 4 | PF10131.10 | ; PTPS\_related ; 6-pyruvoyl-tetrahydropterin synthase related domain; membrane protein | 99.73 | 4.3e-15 | 31.2 | 303 | 616 |
| 5 | PF07220.12 | ; DUF1420 ; Protein of unknown function (DUF1420) | 99.72 | 4.3e-14 | 36.6 | 365 | 670 |
| 6 | PF02366.19 | ; PMT ; Dolichyl-phosphate-mannose-protein mannosyltransferase | 99.6 | 2.1e-13 | 23.2 | 223 | 247 |
| 7 | PF10034.10 | ; Dpy19 ; Q-cell neuroblast polarisation | 99.58 | 3e-12 | 32.2 | 346 | 651 |
| 8 | PF04188.14 | ; Mannosyl\_trans2 ; Mannosyltransferase (PIG-V) | 99.58 | 1.4e-12 | 27.8 | 331 | 432 |
| 9 | PF12250.9 | ; AftA\_N ; Arabinofuranosyltransferase N terminal | 99.53 | 1.6e-11 | 29.7 | 321 | 432 |
| 10 | PF09913.10 | ; DUF2142 ; Predicted membrane protein (DUF2142) | 99.5 | 8.8e-12 | 26 | 324 | 405 |
| 11 | PF13231.7 | ; PMT\_2 ; Dolichyl-phosphate-mannose-protein mannosyltransferase | 99.5 | 1.6e-12 | 18.3 | 157 | 159 |
| 12 | PF11028.9 | ; DUF2723 ; Protein of unknown function (DUF2723) | 99.48 | 1.8e-12 | 17.6 | 155 | 188 |
| 13 | PF04602.13 | ; Arabinose\_trans ; Mycobacterial cell wall arabinan synthesis protein | 99.45 | 1.6e-10 | 29.6 | 356 | 471 |
| 14 | PF06728.14 | ; PIG-U ; GPI transamidase subunit PIG-U | 99.28 | 5e-9 | 29 | 306 | 363 |
| 15 | PF15971.6 | ; Mannosyl\_trans4 ; DolP-mannose mannosyltransferase | 99.22 | 3.9e-10 | 15.8 | 151 | 163 |
| 16 | PF09586.11 | ; YfhO ; Bacterial membrane protein YfhO | 99.18 | 2e-8 | 30 | 371 | 832 |
| 17 | PF14264.7 | ; Glucos\_trans\_II ; Glucosyl transferase GtrII | 99 | 5.6e-7 | 27.8 | 300 | 312 |
| 18 | PF03155.16 | ; Alg6\_Alg8 ; ALG6, ALG8 glycosyltransferase family | 98.98 | 3.8e-7 | 27.3 | 319 | 470 |
| 19 | PF04922.13 | ; DIE2\_ALG10 ; DIE2/ALG10 family | 98.97 | 2.5e-8 | 18.1 | 228 | 434 |
| 20 | PF05208.14 | ; ALG3 ; ALG3 protein | 98.7 | 0.0000012 | 19.4 | 198 | 356 |
| 21 | PF09594.11 | ; GT87 ; Glycosyltransferase family 87 | 98.63 | 0.0000015 | 17.5 | 239 | 251 |
| 22 | PF05007.14 | ; Mannosyl\_trans ; Mannosyltransferase (PIG-M) | 94.9 | 0.87 | 15.5 | 202 | 269 |
| 23 | PF14897.7 | ; EpsG ; EpsG family | 93.55 | 1.9 | 34.4 | 306 | 319 |
| 24 | PF10060.10 | ; DUF2298 ; Uncharacterized membrane protein (DUF2298) | 88.03 | 11 | 35.8 | 363 | 597 |
| 25 | PF16192.6 | ; PMT\_4TMC ; C-terminal four TMM region of protein-O-mannosyltransferase | 79.5 | 12 | 12 | 106 | 198 |

Displaying 1 to 25 of 33 hits

- «
- ‹
- 1
- 2
- ›
- »

#### Alignments

|  |  |  |  |
| --- | --- | --- | --- |
|  | | | |
|  | Template alignmentCDD | | |
| 1. | PF03901.18 ; Glyco\_transf\_22 ; Alg9-like mannosyltransferase family | | |
|  | Probability: 99.86%, E-value: 6.4e-19, Score: 158.24, Aligned cols: 345, Identities: 10%, Similarity: -0.046, | | |
|  |
|  | Q ss\_pred |  | HHHHHHHHHHHHHhhhcCCCcccCch-HHHHhcccccCCCChhhhhcccccccccCCCCCccccCchHHHHHHHHHHHhC |
|  | Q Q5T4D3 | 22 | AKLVVGSVAIVCFARSYDGDFVFDDS-EAIVNNKDLQAETPLGDLWHHDFWGSRLSSNTSHKSYRPLTVLTFRINYYLSG   100 (462) |
|  | Q Consensus | 22 | ~~~~l~~~~~~~~~~~~~~~~~~Dd~-~~~~~~~~~~~~~~~~~~~~~~~~~~~~~~~~~~~~~~Pl~~~~~~~~~~~~g   100 (462) |
|  |  |  | .++++.++.-..........+..||. .+...+++..+++........+ +.....+||+..........++ |
|  | T Consensus | 1 | lil~~~~~l~l~~~~~~~~~~~~De~~~~~~~a~~~~~~~~~~~~~~~~--------~~~~~~~p~~~~~~~~~~~~~~-   71 (388) |
|  | T PF03901.18 | 1 | YLLLFTIALRILNCFLVQTSFVPDEYWQSLEVSHHMVFNYGYLTWEWTE--------RLRSYTYPLIFASIYKILHLLG-   71 (388) |
|  | T ss\_pred |  | CHHHHHHHHHHHHHHhhhcCCCchHHHHHHHHHhhhccccCcCCCcccc--------cccCCHHHHHHHHHHHHHHHcC- |
|  |
|  |
|  | Q ss\_pred |  | CCCc----hHHHHHHHHHHHHHHHHHHHHHHHHhccccccccccccchHHHHHHHHHHHHHHCHHHHHHHHhHhcHHHHH |
|  | Q Q5T4D3 | 101 | GFHP----VGFHVVNILLHSGISVLMVDVFSVLFGGLQYTSKGRRLHLAPRASLLAALLFAVHPVHTECVAGVVGRADLL   176 (462) |
|  | Q Consensus | 101 | g~~~----~~~rl~~~~~~~l~~~l~~~l~~~l~~~~~~~~~~~~~~~~~~~a~~aall~~~~p~~~~~~~~~~~~~~~~   176 (462) |
|  |  |  | |+++ ...|+.+.+++++++.++|.++|+.+ +++.|++++++++++|.....+... ++|.+ |
|  | T Consensus | 72 | ~~~~~~~~~~~r~~~~l~~~~~~~~~y~l~~~~~--------------~~~~a~~a~~l~~~~p~~~~~~~~~--~~~~~   135 (388) |
|  | T PF03901.18 | 72 | KDSVQLLIWIPRLAQALLSAVADVRLYSLMKQLE--------------NQEVARWVFFCQLCSWFTWYCCTRT--LTNTM   135 (388) |
|  | T ss\_pred |  | CCCHHHHHHHHHHHHHHHHHHHHHHHHHHHHHHc--------------ChhHHHHHHHHHHHhHHHHHHHhhh--chHHH |
|  |
|  |
|  | Q ss\_pred |  | HHHHHHHHHHHHHHHHHHcCCCCCchHHHHHHHHHHHHHHHHhchHHHHHHHHHHHHHHHHHhCCCChHHHHHHHhhhcc |
|  | Q Q5T4D3 | 177 | CALFFLLSFLGYCKAFRESNKEGAHSSTFWVLLSIFLGAVAMLCKEQGITVLGLNAVFDILVIGKFNVLEIVQKVLHKDK   256 (462) |
|  | Q Consensus | 177 | ~~~f~ll~~~~~~~~~~~~~~~~~~~~~~~~~~~~~~~~la~l~k~~~~~~~~~~~~~~~~~~~~~~~~~~~~~~~~~~~   256 (462) |
|  |  |  | ..++.+++++++.+..++++ .+.. ++++.+++.++|+.++.+.+...+..+...++++ |
|  | T Consensus | 136 | ~~~~~~~~~~~~~~~~~~~~-------~~~~--~~~~~~l~~~~k~~~~~~~~~~~~~~~~~~~~~~-------------   193 (388) |
|  | T PF03901.18 | 136 | ETVLTIIALFYYPLEGSKSM-------NSVK--YSSLVALAFIIRPTAVILWTPLLFRHFCQEPRKL-------------   193 (388) |
|  | T ss\_pred |  | HHHHHHHHHHHHHHHhCCCC-------cHHH--HHHHHHHHHHhcchHHHHHHHHHHHHHHcChhcH------------- |
|  |
|  |
|  | Q ss\_pred |  | chHhhchhhhhhHHHHHHHHHHHHHHHHHHHHHHhCCCCCCccccCCcchhcchhhHhHhHHHHHHHHHHHHhhccHhhh |
|  | Q Q5T4D3 | 257 | SLENLGMLRNGGLLFRMTLLTSGGAGMLYVRWRIMGTGPPAFTEVDNPASFADSMLVRAVNYNYYYSLNAWLLLCPWWLC   336 (462) |
|  | Q Consensus | 257 | ~~~~~~~~~~~~~~~~~~~~~~~~~~~~~~~~~~~~~~~~~~~~~~~~~~~~~~~~~~~~~~~~~~~~~~~~~~~p~~~~   336 (462) |
|  |  |  | +.......................................................+...... |
|  | T Consensus | 194 | --------~~~~~~~~~~~~~~~~~~~~~~~~~~~~~~~~~~~~~~~~~~~~~~~~~~~~~~~~~~~~~~~---------   256 (388) |
|  | T PF03901.18 | 194 | --------DLILHHFLPVGFVTLSLSLMIDRIFFGQWTLVQFNFLKFNVLQNWGTFYGSHPWHWYFSQGFP---------   256 (388) |
|  | T ss\_pred |  | --------HHHHHHHHHHHHHHHHHHHHHHHHHhcchhhhhhhhhhhhcccccccccccCchHHHHHhhhh--------- |
|  |
|  |
|  | Q ss\_pred |  | ccccccccCcccccchHHHHHHHHHHHHHHHHHHHHHhcCCCcchHHHHHHHHHHHHHHhhHhccccccchhHHHhhhhH |
|  | Q Q5T4D3 | 337 | FDWSMGCIPLIKSISDWRVIALAALWFCLIGLICQALCSEDGHKRRILTLGLGFLVIPFLPASNLFFRVGFVVAERVLYL   416 (462) |
|  | Q Consensus | 337 | ~~~~~~~~~~~~~~~~~~~~~~~~~~~~~~~~~~~~~~~~~~~~~~~~~~~~~~~~~~~~~~~~~~~~~~~~~~~ry~~~   416 (462) |
|  |  |  | ...............+.+++++ ....+.+......... ....+||..+ |
|  | T Consensus | 257 | -----------------------~~~~~~~~~~~~~~~~~~~~~~----~~~~~~~~~~~~~~~~-----~~~~~ry~~~   304 (388) |
|  | T PF03901.18 | 257 | -----------------------VILGTHLPFFIHGCYLAPKRYR----ILLVTVLWTLLVYSML-----SHKEFRFIYP   304 (388) |
|  | T ss\_pred |  | -----------------------hhHHhHHHHHHHHHHHchhhhH----HHHHHHHHHHHHHHhh-----cCCCcchHhc |
|  |
|  |
|  | Q ss\_pred |  | HHHHHHHHHHHHHHHHHhchhhHHHHHHHHHHHHHHHHHHHHHhcC |
|  | Q Q5T4D3 | 417 | PSVGYCVLLTFGFGALSKHTKKKKLIAAVVLGILFINTLRCVLRSG   462 (462) |
|  | Q Consensus | 417 | ~~~~~~ll~a~~~~~~~~~~~~~~~~~~~~~~~~~~~~~~~~~~~~   462 (462) |
|  |  |  | ..|+++++++.++.+..++.++.......+++.............. |
|  | T Consensus | 305 | ~~p~~~l~~~~~~~~~~~~~~~~~~~~~~~~~~~~~~~~~~~~~~~   350 (388) |
|  | T PF03901.18 | 305 | VLPFCMVFCGYSLTHLKTWKKPALSFLFLSNLFLALYTGLVHQRGT   350 (388) |
|  | T ss\_pred |  | HHHHHHHHHHHHHHhhhcccchHHHHHHHHHHHHHHHHHHHhhccc |
|  |
| --- | | | |
|  | Template alignmentCDD | | |
| 2. | PF02516.15 ; STT3 ; Oligosaccharyl transferase STT3 subunit | | |
|  | Probability: 99.83%, E-value: 9.5e-18, Score: 154.02, Aligned cols: 369, Identities: 10%, Similarity: -0.065, | | |
|  |
|  | Q ss\_pred |  | HHHHHHHHHHHHHHHH-----hhhcCCCcccCchHHHHhcccccCCCC--hhhhhcccccccccCCCCCccccCchHHHH |
|  | Q Q5T4D3 | 19 | PFWAKLVVGSVAIVCF-----ARSYDGDFVFDDSEAIVNNKDLQAETP--LGDLWHHDFWGSRLSSNTSHKSYRPLTVLT   91 (462) |
|  | Q Consensus | 19 | ~~~~~~~l~~~~~~~~-----~~~~~~~~~~Dd~~~~~~~~~~~~~~~--~~~~~~~~~~~~~~~~~~~~~~~~Pl~~~~   91 (462) |
|  |  |  | ....+++++++++..+ ..........||..|...+++..+++. .......++++....+......++|++.++ |
|  | T Consensus | 3 | ~~~~l~~i~~~~~~~r~~~~~~~~~~~~~~~D~~~~~~~a~~~~~~~~~~~~~~~~~~~~~~~~~~~~~~~~~~p~~~~l   82 (458) |
|  | T PF02516.15 | 3 | SRIETAELKGMNTADRAYFTDENGLPYMYEPDSYYNYRLTANILDHGHPGDKIINGTPWDLHSNYPPGNRVNYPPLILWI   82 (458) |
|  | T ss\_pred |  | hhHhHHHHHhhhhhhHHhCCCCCCCCcccCCCcHHHHHHHHHHHHhCCCCCcccCCCCCCchhcCCCCCCCCCCchHHHH |
|  |
|  |
|  | Q ss\_pred |  | HHHHHHHhCCCCc-----hHHHHHHHHHHHHHHHHHHHHHHHHhccccccccccccchHHHHHHHHHHHHHHCHHHHHHH |
|  | Q Q5T4D3 | 92 | FRINYYLSGGFHP-----VGFHVVNILLHSGISVLMVDVFSVLFGGLQYTSKGRRLHLAPRASLLAALLFAVHPVHTECV   166 (462) |
|  | Q Consensus | 92 | ~~~~~~~~gg~~~-----~~~rl~~~~~~~l~~~l~~~l~~~l~~~~~~~~~~~~~~~~~~~a~~aall~~~~p~~~~~~   166 (462) |
|  |  |  | ......++ |... ...|+++.++++++++++|.++|++. +++.|++++++++++|.+...+ |
|  | T Consensus | 83 | ~~~~~~~~-~~~~~~~~~~~~~~~~~~~~~l~~~~~y~l~~~~~--------------~~~~a~~a~~l~~~~p~~~~~~   147 (458) |
|  | T PF02516.15 | 83 | SLLFHNFI-NLFIPFSLIETCFWLPAIIGPLAGIVMFFMVRRYA--------------GDLPGLLSGVLLVLAPVYFSRT   147 (458) |
|  | T ss\_pred |  | HHHHHHHH-HhhCCCcHHHHHhHHHHHHHHHHHHHHHHHHHHHc--------------CcHHHHHHHHHHHHcHHHHHhh |
|  |
|  |
|  | Q ss\_pred |  | HhHhcHHHHHHHHHHHHHHHHHHHHHHHcCCCCCchHHHHHHHHHHHHHHHHhchHHHHHHHHHHHHHHHHHhCCCChHH |
|  | Q Q5T4D3 | 167 | AGVVGRADLLCALFFLLSFLGYCKAFRESNKEGAHSSTFWVLLSIFLGAVAMLCKEQGITVLGLNAVFDILVIGKFNVLE   246 (462) |
|  | Q Consensus | 167 | ~~~~~~~~~~~~~f~ll~~~~~~~~~~~~~~~~~~~~~~~~~~~~~~~~la~l~k~~~~~~~~~~~~~~~~~~~~~~~~~   246 (462) |
|  |  |  | ......+|.+..++.+++++++.+..++++++ +++..+++++.+++.++|+......+...+......+++++++ |
|  | T Consensus | 148 | ~~~~~~~~~~~~~~~~l~~~~~~~~~~~~~~~-----~~~~~l~g~~~~l~~~~~~~~~~~~~~~~~~~~~~~~~~~~~~   222 (458) |
|  | T PF02516.15 | 148 | VPGFFDTDMFNIIFPLLVIFFLLKATETKNNY-----MFPLLLSSFSLALLSLSWNGWAYIFYIIIISSILYMTLCKLKG   222 (458) |
|  | T ss\_pred |  | CCCCCCchHHHHHHHHHHHHHHHHHhccCCCc-----hHHHHHHHHHHHHHHhHhhHHHHHHHHHHHHHHHHHHHhhccc |
|  |
|  |
|  | Q ss\_pred |  | HHHHHhhhccchHhhchhhhhhHHHHHHHHHHHHHHHHHHHHH-------------HhCCCCCCccccCCcchhcchhhH |
|  | Q Q5T4D3 | 247 | IVQKVLHKDKSLENLGMLRNGGLLFRMTLLTSGGAGMLYVRWR-------------IMGTGPPAFTEVDNPASFADSMLV   313 (462) |
|  | Q Consensus | 247 | ~~~~~~~~~~~~~~~~~~~~~~~~~~~~~~~~~~~~~~~~~~~-------------~~~~~~~~~~~~~~~~~~~~~~~~   313 (462) |
|  |  |  | ..... .................... ........................ |
|  | T Consensus | 223 | ~~~~~------------------~~~~~~~~~~~~~~~~~~~~~~~~~~~~~~~~~~~~~~~~~~~~~~~~~~~~~~~~~   284 (458) |
|  | T PF02516.15 | 223 | KAVMG------------------FSRKIAVFVIISLLIIGLAGRLGYALIFPTFFKFTFKSLSAGGWPGIFESISELSAP   284 (458) |
|  | T ss\_pred |  | chhhH------------------HHHHHHHHHHHHHHHhhhcccccHHHHHHHHHHHhcccccCCCCCcchHHHHHHhcc |
|  |
|  |
|  | Q ss\_pred |  | hHhHHHHHHHHHHHHhhccHhhhccccccccCcccccchHHHHHHHHHHHHHHHHHHHHHhcCCCcchHHHHHHHHHHHH |
|  | Q Q5T4D3 | 314 | RAVNYNYYYSLNAWLLLCPWWLCFDWSMGCIPLIKSISDWRVIALAALWFCLIGLICQALCSEDGHKRRILTLGLGFLVI   393 (462) |
|  | Q Consensus | 314 | ~~~~~~~~~~~~~~~~~~p~~~~~~~~~~~~~~~~~~~~~~~~~~~~~~~~~~~~~~~~~~~~~~~~~~~~~~~~~~~~~   393 (462) |
|  |  |  | ........ .............+..........++++++++............ |
|  | T Consensus | 285 | ~~~~~~~~----------------------------~~~~~~~~~~~~~~~~~~~~~~~~~~~~~~~~~~~~~~~~~~~~   336 (458) |
|  | T PF02516.15 | 285 | TFDEFLSL----------------------------PGPVNMGIGLFGFVIIGSIMLRDEIKRVHLPDFSWYPFILIGIW   336 (458) |
|  | T ss\_pred |  | cHHHHhcC----------------------------chHHHHHHHHHHHHHHHHHHHHHHHHhccCCCCccHHHHHHHHH |
|  |
|  |
|  | Q ss\_pred |  | HHhhHhccccccchhHHHhhhhHHHHHHHHHHHHHHHHHHhchhhHH-----HHHHHHHHHHHHHHHHHHH |
|  | Q Q5T4D3 | 394 | PFLPASNLFFRVGFVVAERVLYLPSVGYCVLLTFGFGALSKHTKKKK-----LIAAVVLGILFINTLRCVL   459 (462) |
|  | Q Consensus | 394 | ~~~~~~~~~~~~~~~~~~ry~~~~~~~~~ll~a~~~~~~~~~~~~~~-----~~~~~~~~~~~~~~~~~~~   459 (462) |
|  |  |  | .... .......+||..+..|.++++++.++.+..++.+.++ .......+++......... |
|  | T Consensus | 337 | ~~~~------~~~~~~~~Ry~~~~~p~~~i~~~~~~~~~~~~~~~~~~~~~~~~~~~~~~~~~~~~~~~~~   401 (458) |
|  | T PF02516.15 | 337 | LIIG------LAAYSLSTRFALLVIPPLIIFLGLLMGVMASYLKGSPSMRLRRSGNVFILSLVVMLSTISF   401 (458) |
|  | T ss\_pred |  | HHHH------HHHHHHHHhHHHhhHHHHHHHHHHHHHHHHHHhcCCccchHHHHHHHHHHHHHHHHHHHHH |
|  |
| --- | | | |
|  | Template alignmentCDD | | |
| 3. | PF09852.10 ; DUF2079 ; Predicted membrane protein (DUF2079) | | |
|  | Probability: 99.78%, E-value: 6.6e-16, Score: 144.01, Aligned cols: 328, Identities: 13%, Similarity: 0.039, | | |
|  |
|  | Q ss\_pred |  | CCcccCchHHHHhcccccCCCChhhhhcccccccccCCCCCccccCchHHHHHHHHHHHhCCCCchHHHHHHHHHHHHHH |
|  | Q Q5T4D3 | 40 | GDFVFDDSEAIVNNKDLQAETPLGDLWHHDFWGSRLSSNTSHKSYRPLTVLTFRINYYLSGGFHPVGFHVVNILLHSGIS   119 (462) |
|  | Q Consensus | 40 | ~~~~~Dd~~~~~~~~~~~~~~~~~~~~~~~~~~~~~~~~~~~~~~~Pl~~~~~~~~~~~~gg~~~~~~rl~~~~~~~l~~   119 (462) |
|  |  |  | ....+||..+...+.++.+++.......+. .....++|++.++.++.+.++ | ++...|+++++++++++ |
|  | T Consensus | 6 | ~~~~~De~~~~~~a~~~~~g~~~~~~~~~~---------~~~~~~~pl~~~l~a~~~~l~-g-~~~~~rl~~~l~~~~~~   74 (519) |
|  | T PF09852.10 | 6 | NATAYDLGIYVSILENTMHGHVMYANPLLI---------NSFSEHFSPFLFVIYPIYWFF-P-YVKTLLIMQSVMISFSG   74 (519) |
|  | T ss\_pred |  | CccccHHHHHHHHHHHHHcCCccccCcccc---------ccccccchhHHHHHHHHHHHc-C-ChHHHHHHHHHHHHHHH |
|  |
|  |
|  | Q ss\_pred |  | HHHHHHHHHHhccccccccccccchH----------HHHHHHHHHHHHHCHHHHHHH-HhHhcHHHHHHHHHHHHHHHHH |
|  | Q Q5T4D3 | 120 | VLMVDVFSVLFGGLQYTSKGRRLHLA----------PRASLLAALLFAVHPVHTECV-AGVVGRADLLCALFFLLSFLGY   188 (462) |
|  | Q Consensus | 120 | ~l~~~l~~~l~~~~~~~~~~~~~~~~----------~~~a~~aall~~~~p~~~~~~-~~~~~~~~~~~~~f~ll~~~~~   188 (462) |
|  |  |  | +++|+++|+++ + ++.|++++++++++|...... ... +.|.+..++.+++++++ |
|  | T Consensus | 75 | ~~~y~l~r~~~--------------~~~~~~~~~~~~~~al~a~ll~~~~p~~~~~~~~~~--~~~~~~~~~~~~~~~~~   138 (519) |
|  | T PF09852.10 | 75 | LVIYLLAREIF--------------FINNFKKDILLEMLALFISTSYILSPYIESPLSFDF--HLMPFLILFVPLSFYFF   138 (519) |
|  | T ss\_pred |  | HHHHHHHHHHh--------------ccCcccchhHHHHHHHHHHHHHHHcHHhhhhhhcCC--chHHHHHHHHHHHHHHH |
|  |
|  |
|  | Q ss\_pred |  | HHHHHHcCCCCCchHHHHHHHHHHHHHHHHhchHHHHHHHHHHHHHHHHH-----------------h----------CC |
|  | Q Q5T4D3 | 189 | CKAFRESNKEGAHSSTFWVLLSIFLGAVAMLCKEQGITVLGLNAVFDILV-----------------I----------GK   241 (462) |
|  | Q Consensus | 189 | ~~~~~~~~~~~~~~~~~~~~~~~~~~~la~l~k~~~~~~~~~~~~~~~~~-----------------~----------~~   241 (462) |
|  |  |  | .+. ++ ..++++++++++++|+.++++.+.+.+..+.. . ++ |
|  | T Consensus | 139 | ~~~---~~----------~~~~gl~~~la~l~k~~~~~~~~~~~l~~~~~~~~~~~~~~~~~~~~~~~~~~~~~~~~~~~   205 (519) |
|  | T PF09852.10 | 139 | MKK---YK----------ILNLIVLILIISLHSLFVIMVFFIISYQFIFRIRNEGNLNCHKIIRTIANINISDNLKKTPK   205 (519) |
|  | T ss\_pred |  | Hhh---cc----------HHHHHHHHHHHHHhcchHHHHHHHHHHHHHHHHhccCCCcchhhhHHhhhcccccccccCCc |
|  |
|  |
|  | Q ss\_pred |  | CChHHHHHHHhhhccchHhhchhhhhhHHHHHHHHHHHHHHHHHHHHHHhCCC--------CCCccccCCcchhcchhhH |
|  | Q Q5T4D3 | 242 | FNVLEIVQKVLHKDKSLENLGMLRNGGLLFRMTLLTSGGAGMLYVRWRIMGTG--------PPAFTEVDNPASFADSMLV   313 (462) |
|  | Q Consensus | 242 | ~~~~~~~~~~~~~~~~~~~~~~~~~~~~~~~~~~~~~~~~~~~~~~~~~~~~~--------~~~~~~~~~~~~~~~~~~~   313 (462) |
|  |  |  | ++.+...+...+. ...........+....+........ .+................. |
|  | T Consensus | 206 | ~~~~~~~~~~~~~---------------~~~~~~~~~~~~~~~~~~~~~~~~~~~~~~~~~~~~~~~~~~~~~~~~~~~~   270 (519) |
|  | T PF09852.10 | 206 | SKYVLQKIVRSKT---------------LIKIIITLILLVGYLYFASLMKTFIASGAVALSPPSTMSTGSVSSSLAGLFT   270 (519) |
|  | T ss\_pred |  | chhhhhhhhcchH---------------HHHHHHHHHHHHHHHHHHHHHHHHHHccccccCCCcccCCCCcCCcHHHHHH |
|  |
|  |
|  | Q ss\_pred |  | hHhHHHHHHHHHHHHhhccHhhhccccccccCcccccchHHHHHHHHHHHHHHHHHHHHHhcCCCcchHHHHHHHHHHHH |
|  | Q Q5T4D3 | 314 | RAVNYNYYYSLNAWLLLCPWWLCFDWSMGCIPLIKSISDWRVIALAALWFCLIGLICQALCSEDGHKRRILTLGLGFLVI   393 (462) |
|  | Q Consensus | 314 | ~~~~~~~~~~~~~~~~~~p~~~~~~~~~~~~~~~~~~~~~~~~~~~~~~~~~~~~~~~~~~~~~~~~~~~~~~~~~~~~~   393 (462) |
|  |  |  | .............. .............++..+.... .+.......+.+.+. |
|  | T Consensus | 271 | ~~~~~~~~~~~~~~----------------------~~~~~~~~~~~~~~~~~~~~~~-------~~~~~~~~~~~~~~~   321 (519) |
|  | T PF09852.10 | 271 | DLFTRPMLIESAFL----------------------INFPDKIIFAFYAFANTGFLVF-------LDPLSLLMDIPYFLY   321 (519) |
|  | T ss\_pred |  | HHhhCchHHHHHHh----------------------ccchHHHHHHHHHHHHHhHHHh-------cCHHHHHhHHHHHHH |
|  |
|  |
|  | Q ss\_pred |  | HHhhHhccccccchhHHHhhhhHHHHHHHHHHHHHHHHHHhc-----------hhhHHHHHHHHHHHHHHHHH |
|  | Q Q5T4D3 | 394 | PFLPASNLFFRVGFVVAERVLYLPSVGYCVLLTFGFGALSKH-----------TKKKKLIAAVVLGILFINTL   455 (462) |
|  | Q Consensus | 394 | ~~~~~~~~~~~~~~~~~~ry~~~~~~~~~ll~a~~~~~~~~~-----------~~~~~~~~~~~~~~~~~~~~   455 (462) |
|  |  |  | ..+...... .....||..+.+|+++++++.++.++.+. ..++.....++++++..... |
|  | T Consensus | 322 | ~~~~~~~~~----~~~~~ry~~~~~p~l~i~~~~~~~~~~~~~~~~~~~~~~~~~~~~~~~~~~~~~~~~~~~   390 (519) |
|  | T PF09852.10 | 322 | AYLSSYGPY----YSLGYQYSTMIIPFIFIGALFGIRKIVQSARATDSDDVRRTIKKILVGVISIVIVSSLFE   390 (519) |
|  | T ss\_pred |  | HHhccCccc----cchhhHHHHhHHHHHHHHHHHHHHHHHHHhccCCCcchhHhHHHHHHHHHHHHHHHHHHh |
|  |
| --- | | | |
|  | Template alignmentCDD | | |
| 4. | PF10131.10 ; PTPS\_related ; 6-pyruvoyl-tetrahydropterin synthase related domain; membrane protein | | |
|  | Probability: 99.73%, E-value: 4.3e-15, Score: 141.66, Aligned cols: 303, Identities: 6%, Similarity: -0.106, | | |
|  |
|  | Q ss\_pred |  | ccccCchHHHHHHHHHHHhCCCCchHHHHHHHHHHHHHHHHHHHHHHHHhccccccccccccchHHHHHHHHHHHHHHCH |
|  | Q Q5T4D3 | 81 | HKSYRPLTVLTFRINYYLSGGFHPVGFHVVNILLHSGISVLMVDVFSVLFGGLQYTSKGRRLHLAPRASLLAALLFAVHP   160 (462) |
|  | Q Consensus | 81 | ~~~~~Pl~~~~~~~~~~~~gg~~~~~~rl~~~~~~~l~~~l~~~l~~~l~~~~~~~~~~~~~~~~~~~a~~aall~~~~p   160 (462) |
|  |  |  | ...|||++.++.++...++||.+..+.|+.++++++++++++|.++|++. ++..|++++++++++| |
|  | T Consensus | 1 | f~~~pPl~~~l~~~~~~l~~g~~~~~~~l~~~l~~~l~~~~~y~l~r~~~--------------~~~~a~~a~~l~~~~p   66 (616) |
|  | T PF10131.10 | 1 | FRYWGPLSYYIMAGLMFLTSGDLLLAYRLIAFVIFVVGGLPWILWGIHEN--------------RRVLGTFFGVLWFFMP   66 (616) |
|  | T ss\_pred |  | CCCCchHHHHHHHHHHHHhcCCHHHHHHHHHHHHHHHHHHHHHHHHHHcC--------------cHHHHHHHHHHHHHCc |
|  |
|  |
|  | Q ss\_pred |  | HHHHHHHhHhcHHHHHHHHHHHHHHHHHHHHHHHcCCCCCchHHHHHHHHHHHHHHHHhch-HHHHHHHHHHHHHHHHHh |
|  | Q Q5T4D3 | 161 | VHTECVAGVVGRADLLCALFFLLSFLGYCKAFRESNKEGAHSSTFWVLLSIFLGAVAMLCK-EQGITVLGLNAVFDILVI   239 (462) |
|  | Q Consensus | 161 | ~~~~~~~~~~~~~~~~~~~f~ll~~~~~~~~~~~~~~~~~~~~~~~~~~~~~~~~la~l~k-~~~~~~~~~~~~~~~~~~   239 (462) |
|  |  |  | .......+....+|.+..++.+++++++.+..++++ .++.++++++.++++++| .....+.+..+++.+... |
|  | T Consensus | 67 | ~~~~~~~~~~~~~~~~~~~~~~l~l~~~~~~~~~~~-------~~~~~~~~l~~~l~~~~~~~~~~~~~~~~~~~~~~~~   139 (616) |
|  | T PF10131.10 | 67 | EHIRIYFTAGNLPQMVTTMLVPYVIWFLWLYVRKKN-------NRAAVGLFVCMTLMSFTHLMVTAIMGVSAFLYLLIDQ   139 (616) |
|  | T ss\_pred |  | hHHHHHHhcCCHHHHHHHHHHHHHHHHHHHHHhcCC-------HHHHHHHHHHHHHHHHhcHHHHHHHHHHHHHHHHHHH |
|  |
|  |
|  | Q ss\_pred |  | CCCChHHHHHHHhhhccchHhhchhhhhhHHHHHHHHHHHHHHHHHHHHHHhCCCCCCccccCCcchhcchhhHhHhHHH |
|  | Q Q5T4D3 | 240 | GKFNVLEIVQKVLHKDKSLENLGMLRNGGLLFRMTLLTSGGAGMLYVRWRIMGTGPPAFTEVDNPASFADSMLVRAVNYN   319 (462) |
|  | Q Consensus | 240 | ~~~~~~~~~~~~~~~~~~~~~~~~~~~~~~~~~~~~~~~~~~~~~~~~~~~~~~~~~~~~~~~~~~~~~~~~~~~~~~~~   319 (462) |
|  |  |  | .+++.++..++ .....+..++....+................................... |
|  | T Consensus | 140 | ~~~~~~~~~~~-------------------~~~~~~~~~~~~~~~~~~~~~~~~~~~~~~~~~~~~~~~~~~~~~~~~~~   200 (616) |
|  | T PF10131.10 | 140 | IWNKDTRRKIF-------------------ALIYMICGILTAGIWVIPSLKGGLVTSESGDGSVMSTLIYPLTTSLNPFK   200 (616) |
|  | T ss\_pred |  | HhCCChHHHHH-------------------HHHHHHHHHHHHHHHHHHHHccCCccCCCCccchhhhheechhhccCcch |
|  |
|  |
|  | Q ss\_pred |  | HH----HHHHHHHhhccHhhhccccccccCcccccchHHHHHHHHHHHHHHHHHHHHHhcCCCcchHHHHHHHHHHHHHH |
|  | Q Q5T4D3 | 320 | YY----YSLNAWLLLCPWWLCFDWSMGCIPLIKSISDWRVIALAALWFCLIGLICQALCSEDGHKRRILTLGLGFLVIPF   395 (462) |
|  | Q Consensus | 320 | ~~----~~~~~~~~~~p~~~~~~~~~~~~~~~~~~~~~~~~~~~~~~~~~~~~~~~~~~~~~~~~~~~~~~~~~~~~~~~   395 (462) |
|  |  |  | .. ...... ...+.++.......++++++........++...... |
|  | T Consensus | 201 | ~~~~~~~~~~~~--------------------------------~~~~~l~~~~~~~~~~~~~~~~~~~~~~~~~~~~~~   248 (616) |
|  | T PF10131.10 | 201 | RLSAGNDSFYFG--------------------------------LAAVLIAIAGILLARGGKKAGFVFLLIMLACTTPAA   248 (616) |
|  | T ss\_pred |  | hccCCCCcchHH--------------------------------HHHHHHHHHHHHHHcCCChHHHHHHHHHHHhcchHH |
|  |
|  |
|  | Q ss\_pred |  | hhHhccccccchhHHHhhhhHHHHHHHHHHHHHHHHHHhchhhHHHHHHHHHHHHHHHHHHHHHhcC |
|  | Q Q5T4D3 | 396 | LPASNLFFRVGFVVAERVLYLPSVGYCVLLTFGFGALSKHTKKKKLIAAVVLGILFINTLRCVLRSG   462 (462) |
|  | Q Consensus | 396 | ~~~~~~~~~~~~~~~~ry~~~~~~~~~ll~a~~~~~~~~~~~~~~~~~~~~~~~~~~~~~~~~~~~~   462 (462) |
|  |  |  | ................||+.+. +++++++.++.+..++ ....+++++.+........... |
|  | T Consensus | 249 | ~~~~~~~~~~~~~~~~r~~~~~--~~~~~~~~~~~~~~~~-----~~~~~~~~~~~~~~~~~~~~~~   308 (616) |
|  | T PF10131.10 | 249 | YRILVKLPFSQLFWMTRFAPMV--YGFFFSACLEWVRLKK-----KYCVLLAALLCVDSISCMNLDF   308 (616) |
|  | T ss\_pred |  | HHHHhhCCHHHhhhHhHHHHHH--HHHHHHHHHHhhhhHH-----HHHHHHHHHHHHHHhhhccccc |
|  |
| --- | | | |
|  | Template alignmentCDD | | |
| 5. | PF07220.12 ; DUF1420 ; Protein of unknown function (DUF1420) | | |
|  | Probability: 99.72%, E-value: 4.3e-14, Score: 135.91, Aligned cols: 365, Identities: 12%, Similarity: -0.067, | | |
|  |
|  | Q ss\_pred |  | ChhhcccccCCHHHHHHHHHHHHHHHHHhhhcCCCcccCchHHHHhcccccCCCChhhhhcccccccccCCCCCccccCc |
|  | Q Q5T4D3 | 7 | DLDHILPSSVLPPFWAKLVVGSVAIVCFARSYDGDFVFDDSEAIVNNKDLQAETPLGDLWHHDFWGSRLSSNTSHKSYRP   86 (462) |
|  | Q Consensus | 7 | ~~~~~~~~~~~~~~~~~~~l~~~~~~~~~~~~~~~~~~Dd~~~~~~~~~~~~~~~~~~~~~~~~~~~~~~~~~~~~~~~P   86 (462) |
|  |  |  | .......+..........+++++.+.........+..+||..|+.... .++.+++.... .+......+|| |
|  | T Consensus | 144 | ~~~~~~~~~~~~~~~l~~~i~~~~~~~~~~~~~p~~~~D~~~yhl~~a--------~~~~~~g~~~~--~~~~~~~~~P~   213 (670) |
|  | T PF07220.12 | 144 | GFIKNKFNLINKNDVLNVFIILLMIGYGFLALCPITNADSLDYHIGVA--------IEILNQGKMPV--FSGWFHGRLAG   213 (670) |
|  | T ss\_pred |  | hhHHhhcccCChhHHHHHHHHHHHHHHHHHHcCCCCCCHHHHHHHHHH--------HHHHHcCCCCC--CCCchhhcCCc |
|  |
|  |
|  | Q ss\_pred |  | hHHHHHHHHHHHhCCCCchHHHHHHHHHHHHHHHHHHHHHHHHhccccccccccccchHH--HHHHHHHHHHHHCHHHHH |
|  | Q Q5T4D3 | 87 | LTVLTFRINYYLSGGFHPVGFHVVNILLHSGISVLMVDVFSVLFGGLQYTSKGRRLHLAP--RASLLAALLFAVHPVHTE   164 (462) |
|  | Q Consensus | 87 | l~~~~~~~~~~~~gg~~~~~~rl~~~~~~~l~~~l~~~l~~~l~~~~~~~~~~~~~~~~~--~~a~~aall~~~~p~~~~   164 (462) |
|  |  |  | ....+......+. ++........+.++++++++++|.++|+.+ ++ ..+.+++++++++|.+.. |
|  | T Consensus | 214 | ~~~~l~~~~~~l~-~~~~~~~~~~~~l~~~l~~~~~y~l~r~~~--------------~~~~~~a~~aall~~~~p~~~~   278 (670) |
|  | T PF07220.12 | 214 | SGEVLNALGLAIG-AEQFGSLLQFCGLLSIYGILSFYSFAEKFS--------------ESDGVWRKIIIIAFLSSPVLVF   278 (670) |
|  | T ss\_pred |  | hHHHHHHHHHHHc-chhHHHHHHHHHHHHHHHHHHHHHHHHHhc--------------CCCCHHHHHHHHHHHhchHHHH |
|  |
|  |
|  | Q ss\_pred |  | HHHh---HhcHHHHHHHHHHHHHHHHHHHHHHHcCCCCCchHHHHHHHHHHH--HHHHHhchHHHHHHHHHHHHHHHHHh |
|  | Q Q5T4D3 | 165 | CVAG---VVGRADLLCALFFLLSFLGYCKAFRESNKEGAHSSTFWVLLSIFL--GAVAMLCKEQGITVLGLNAVFDILVI   239 (462) |
|  | Q Consensus | 165 | ~~~~---~~~~~~~~~~~f~ll~~~~~~~~~~~~~~~~~~~~~~~~~~~~~~--~~la~l~k~~~~~~~~~~~~~~~~~~   239 (462) |
|  |  |  | .+.. . ..|.+.+++.+++++++.+..++++ .++..+++++ +|+|+.+|++++++.++..+..+... |
|  | T Consensus | 279 | ~s~~~~~~--~~d~~~~~~~~~~l~~~~~~~~~~~-------~~~~~l~gl~~~~gla~~~K~~~~~~~~~~~~~~l~~~   349 (670) |
|  | T PF07220.12 | 279 | LVSSPKPQ--LLQIGMTSFAITLLLEIFSKIKTDK-------NKLFAFSLICILIMSATQAKFSFFLSAFLIGLFSIFSL   349 (670) |
|  | T ss\_pred |  | HhcCCchH--HHHHHHHHHHHHHHHHHHHhhccCC-------chHHHHHHHHHHHHHHHhhHHHHHHHHHHHHHHHHHHh |
|  |
|  |
|  | Q ss\_pred |  | CCCChHHHHHHHhhhccchHhhchhhhhhHHH---HHHHHHHHHHHHHHHHHHHhCCCCCCccccCCcchhcchhhHhHh |
|  | Q Q5T4D3 | 240 | GKFNVLEIVQKVLHKDKSLENLGMLRNGGLLF---RMTLLTSGGAGMLYVRWRIMGTGPPAFTEVDNPASFADSMLVRAV   316 (462) |
|  | Q Consensus | 240 | ~~~~~~~~~~~~~~~~~~~~~~~~~~~~~~~~---~~~~~~~~~~~~~~~~~~~~~~~~~~~~~~~~~~~~~~~~~~~~~   316 (462) |
|  |  |  | ++++ ...+.... ......++...+........+....................+... |
|  | T Consensus | 350 | ~~~~--------------------~~~~~~~~~~~~~~~~~~~~~~w~~~~~~~~g~~~~~~~~~~~~~~~~~~~~~~~~   409 (670) |
|  | T PF07220.12 | 350 | GSIR--------------------LFFYGLLISLFFFVLINFPAIFWKIKNYNSTFIDVLIHPLPGNTFPGVNEFEVSLR   409 (670) |
|  | T ss\_pred |  | cccc--------------------hhHHHHHHHHHHHHHHHHHHHHHHHHHHHccCCcccccCCCCCCCCCccchHHHHH |
|  |
|  |
|  | Q ss\_pred |  | HHHHHHHHHHHHhhccHhhhccccccccCcccccchHHHHHHHHHHHHHHHHHHHHHhcCCCcchHHHHHHHHHHHHHHh |
|  | Q Q5T4D3 | 317 | NYNYYYSLNAWLLLCPWWLCFDWSMGCIPLIKSISDWRVIALAALWFCLIGLICQALCSEDGHKRRILTLGLGFLVIPFL   396 (462) |
|  | Q Consensus | 317 | ~~~~~~~~~~~~~~~p~~~~~~~~~~~~~~~~~~~~~~~~~~~~~~~~~~~~~~~~~~~~~~~~~~~~~~~~~~~~~~~~   396 (462) |
|  |  |  | ...............|...... ...++.........++++++................. |
|  | T Consensus | 410 | ~~~~~~~~~~~~~~~~~~~~~~---------------------~~~~~~~~~~~~~~~~~~~~~~~~~~~~~~~~~~~~~   468 (670) |
|  | T PF07220.12 | 410 | NYQDSALIFPLSLIFPNQFGVI---------------------TTVIGLGLFLIIFVKPIVTQKAFLLSVMIILFVILGS   468 (670) |
|  | T ss\_pred |  | hcccccccCcHHhhccccchhh---------------------HhHHHHHHHHHHHhcchhchHHHHHHHHHHHHHHHHH |
|  |
|  |
|  | Q ss\_pred |  | hHhccccccchhHHHhhhhHHHHHHHHHHHHHHHHHHhchhhHHHHHHHHHHHHHHHHH |
|  | Q Q5T4D3 | 397 | PASNLFFRVGFVVAERVLYLPSVGYCVLLTFGFGALSKHTKKKKLIAAVVLGILFINTL   455 (462) |
|  | Q Consensus | 397 | ~~~~~~~~~~~~~~~ry~~~~~~~~~ll~a~~~~~~~~~~~~~~~~~~~~~~~~~~~~~   455 (462) |
|  |  |  | ...... .||++|.+|++++ ++.+.....+..++........++++..... |
|  | T Consensus | 469 | ~~~~~~--------~Ry~lp~~p~l~l-~~~~~~~~~~~~~~~~~~~~~~~~~~~~~~~   518 (670) |
|  | T PF07220.12 | 469 | LMGQKA--------SRFFLEPFVWMLI-SLIGLNSFGKWNIRFVKEAVSTGILLQACAT   518 (670) |
|  | T ss\_pred |  | Hhccch--------HHhhHHHHHHHHH-HHHHHHhhccccHHHHHHHHHHHHHHHHHHH |
|  |
| --- | | | |
|  | Template alignmentCDD | | |
| 6. | PF02366.19 ; PMT ; Dolichyl-phosphate-mannose-protein mannosyltransferase | | |
|  | Probability: 99.6%, E-value: 2.1e-13, Score: 113.37, Aligned cols: 223, Identities: 13%, Similarity: 0.008, | | |
|  |
|  | Q ss\_pred |  | HHHHHHHHHHHHhhhcCCCcccCchHHHHhcccccCCCChhhhhcccccccccCCCCCccccCchHHHHHHHHHHHhCC- |
|  | Q Q5T4D3 | 23 | KLVVGSVAIVCFARSYDGDFVFDDSEAIVNNKDLQAETPLGDLWHHDFWGSRLSSNTSHKSYRPLTVLTFRINYYLSGG-   101 (462) |
|  | Q Consensus | 23 | ~~~l~~~~~~~~~~~~~~~~~~Dd~~~~~~~~~~~~~~~~~~~~~~~~~~~~~~~~~~~~~~~Pl~~~~~~~~~~~~gg-   101 (462) |
|  |  |  | .++++.............+..+||..+...+.+..+++ .....++|............+ + |
|  | T Consensus | 2 | ~~~~~~~~~~~~~~~~~~~~~~D~~~~~~~a~~~~~~~------------------~~~~~~~~~~~~~~~~~~~~~-~~   62 (247) |
|  | T PF02366.19 | 2 | FLTVVAFCVRAQRLMNPAKVVFEELRYYNYAVDYVNNK------------------LLMDVYPPLGKLLFSLVAALT-GN   62 (247) |
|  | T ss\_pred |  | hHHHHHHHHHHHHHhCCcccchHHHHHHHHHHHHHcCc------------------cccCCCCcHHHHHHHHHHHHc-CC |
|  |
|  |
|  | Q ss\_pred |  | -----------------CCchHHHHHHHHHHHHHHHHHHHHHHHHhccccccccccccchHHHHHHHHHHHHHHCHHHHH |
|  | Q Q5T4D3 | 102 | -----------------FHPVGFHVVNILLHSGISVLMVDVFSVLFGGLQYTSKGRRLHLAPRASLLAALLFAVHPVHTE   164 (462) |
|  | Q Consensus | 102 | -----------------~~~~~~rl~~~~~~~l~~~l~~~l~~~l~~~~~~~~~~~~~~~~~~~a~~aall~~~~p~~~~   164 (462) |
|  |  |  | .+....|+.+.+++.+++.++|.++|+..+ +++.+++++++++++|.... |
|  | T Consensus | 63 | ~~~~~~~~~~~~~~~~~~~~~~~~~~~~~~~~~~~~~~~~~~~~~~~-------------~~~~a~~~~~~~~~~p~~~~   129 (247) |
|  | T PF02366.19 | 63 | KYELNTLDEPGQQYPFTDVAYSMRLFTCLLGSLLVPLMYGTVYFPTK-------------SKTAASLAALFVIFDNGLIT   129 (247) |
|  | T ss\_pred |  | CcccccccCCCCCCCcchHHHHHHHHHHHHHHHHHHHHHHHHhcccC-------------CHHHHHHHHHHHHhchhHHH |
|  |
|  |
|  | Q ss\_pred |  | HHHhHhcHHHHHHHHHHHHHHHHHHHHHHHcCCCCCchHHHHHHHHHHHHHHHHhchHHHHHHHHHHHHHHHHHhCCCCh |
|  | Q Q5T4D3 | 165 | CVAGVVGRADLLCALFFLLSFLGYCKAFRESNKEGAHSSTFWVLLSIFLGAVAMLCKEQGITVLGLNAVFDILVIGKFNV   244 (462) |
|  | Q Consensus | 165 | ~~~~~~~~~~~~~~~f~ll~~~~~~~~~~~~~~~~~~~~~~~~~~~~~~~~la~l~k~~~~~~~~~~~~~~~~~~~~~~~   244 (462) |
|  |  |  | ..... .+|.+..++.+++++++.+..++++++....++++...++++.+++.++|+.+....+...+.......+++ |
|  | T Consensus | 130 | ~~~~~--~~d~~~~~~~~~~~~~~~~~~~~~~~~~~~~~~~~~~~~~~~~~l~~~~k~~~~~~~~~~~~~~~~~~~~~~-   206 (247) |
|  | T PF02366.19 | 130 | MSRYI--MIEIPALYFMSLTAFYWSVYEAQQKRPFSLRWHTSLLSTGVALGLALSTKLSAMFTFGWLLILAAFHLWNLL-   206 (247) |
|  | T ss\_pred |  | hHhhc--cchHHHHHHHHHHHHHHHHHHHhcCCCCCcHHHHHHHHHHHHHHHHHHhhHHHHHHHHHHHHHHHHHHHHhc- |
|  |
|  |
|  | Q ss\_pred |  | HHHHHHHhhhccchHhhchhhhhhHHHHHHHHHHHHHHHHHHHHHHhCC |
|  | Q Q5T4D3 | 245 | LEIVQKVLHKDKSLENLGMLRNGGLLFRMTLLTSGGAGMLYVRWRIMGT   293 (462) |
|  | Q Consensus | 245 | ~~~~~~~~~~~~~~~~~~~~~~~~~~~~~~~~~~~~~~~~~~~~~~~~~   293 (462) |
|  |  |  | ....................+....+........++ |
|  | T Consensus | 207 | -------------~~~~~~~~~~~~~~~~~~~~~~~~p~~~~~~~~~~~   242 (247) |
|  | T PF02366.19 | 207 | -------------GDLSVPMYRIVKHLFSYIFYLIGVPITVYLAVFAVH   242 (247) |
|  | T ss\_pred |  | -------------CCCCCCHHHHHHHHHHHHHHHHHHHHHHHHHHHHHH |
|  |
| --- | | | |
|  | Template alignmentCDD | | |
| 7. | PF10034.10 ; Dpy19 ; Q-cell neuroblast polarisation | | |
|  | Probability: 99.58%, E-value: 3e-12, Score: 122.51, Aligned cols: 346, Identities: 9%, Similarity: -0.055, | | |
|  |
|  | Q ss\_pred |  | CCCcccCchHHHHhcccccCCCChhh-hhc--ccccccccCCCCCccccCchHHHHHHHHHHHhCC-------------- |
|  | Q Q5T4D3 | 39 | DGDFVFDDSEAIVNNKDLQAETPLGD-LWH--HDFWGSRLSSNTSHKSYRPLTVLTFRINYYLSGG--------------   101 (462) |
|  | Q Consensus | 39 | ~~~~~~Dd~~~~~~~~~~~~~~~~~~-~~~--~~~~~~~~~~~~~~~~~~Pl~~~~~~~~~~~~gg--------------   101 (462) |
|  |  |  | +.++..||..|+..++++.+++++.+ ... .+..............++|....+.+..++++ + |
|  | T Consensus | 30 | ~~~~~~d~~~y~~~~~~i~~~~~~~~~~~~~~~d~~~~~p~g~~~~~~~~~~~~~~~a~~~~~~-~~~~~~~~~~~~~~~   108 (651) |
|  | T PF10034.10 | 30 | ELSFRTEMGLYYSYYKTMVEAPTFLDGLHAVMNCNVTEYPDTVNTLKRFNLYPEVILAGKFRIF-EWLASKFEYQTKTCY   108 (651) |
|  | T ss\_pred |  | hhccCcchhhHHhHHhhhccCCCHHHHHHHHHcCCCCcCCCCchHHhhcchHHHHHHHHHHHHH-HHHHhhcCcCccchh |
|  |
|  |
|  | Q ss\_pred |  | ------------------CCchHHHHHHHHHHHHHHHHHHHHHHHHhccccccccccccchHHH-HHHHHHHHHHHCHHH |
|  | Q Q5T4D3 | 102 | ------------------FHPVGFHVVNILLHSGISVLMVDVFSVLFGGLQYTSKGRRLHLAPR-ASLLAALLFAVHPVH   162 (462) |
|  | Q Consensus | 102 | ------------------~~~~~~rl~~~~~~~l~~~l~~~l~~~l~~~~~~~~~~~~~~~~~~-~a~~aall~~~~p~~   162 (462) |
|  |  |  | .....+.....++++++++.+|.++|+++ +++ .|++++++++++|.+ |
|  | T Consensus | 109 | ~~~~~~~~~~~~~~~~~~~p~~~~~~~~~i~~~l~v~~~y~l~~~l~--------------~~~~~al~aall~a~~p~~   174 (651) |
|  | T PF10034.10 | 109 | TVNRGYGLPPVQSCEGLGELSFFYVYSIFFLTGLMMACFFILCFYLS--------------GSILGGVLGTLCYFFNHGE   174 (651) |
|  | T ss\_pred |  | eeecCCCCCCcccCCCcCchHHHHHHHHHHHHHHHHHHHHHHHHHHc--------------CChHHHHHHHHHHHhChhh |
|  |
|  |
|  | Q ss\_pred |  | HHHHHhHhcHHHHHHHHHHHHHHHHHHHHHHHcCCCCCchHHHHHHHHHHHHHHHHhchHHHHHHHHHHHHHHHHHhCCC |
|  | Q Q5T4D3 | 163 | TECVAGVVGRADLLCALFFLLSFLGYCKAFRESNKEGAHSSTFWVLLSIFLGAVAMLCKEQGITVLGLNAVFDILVIGKF   242 (462) |
|  | Q Consensus | 163 | ~~~~~~~~~~~~~~~~~f~ll~~~~~~~~~~~~~~~~~~~~~~~~~~~~~~~~la~l~k~~~~~~~~~~~~~~~~~~~~~   242 (462) |
|  |  |  | ...........|.+..++.+++++++.+..++++++ .++.++++++.+++.++|...........+..+.....+ |
|  | T Consensus | 175 | ~~~~~~g~~~~~~~~~~f~~l~l~~~~~~~~~~~~~-----~~~~~l~gl~~~l~~~~~~~~~~~~~~~~~~~~~~~~~~   249 (651) |
|  | T PF10034.10 | 175 | ATRVMWTPPLRESFSYPYLVAQLLVVTFTLRSVKVT-----WRHITLVSMTTALFMIPWQFAQFALLTQTCALFVVYIMH   249 (651) |
|  | T ss\_pred |  | hhhHhhCCCccccCHHHHHHHHHHHHHHHHhCCCCC-----HHHHHHHHHHHHHHHHHhhhHHHHHHHHHHHHHHHHHhh |
|  |
|  |
|  | Q ss\_pred |  | ChHHHHHHHhhhccchHhhchhhhhhHHHHHHHHHHHHHHHHHHHHHH-------------------------------- |
|  | Q Q5T4D3 | 243 | NVLEIVQKVLHKDKSLENLGMLRNGGLLFRMTLLTSGGAGMLYVRWRI--------------------------------   290 (462) |
|  | Q Consensus | 243 | ~~~~~~~~~~~~~~~~~~~~~~~~~~~~~~~~~~~~~~~~~~~~~~~~--------------------------------   290 (462) |
|  |  |  | +.++...+. ..................... |
|  | T Consensus | 250 | ~~~~~~~~~------------------~~~~~~~~~~~~~~~~~~~~~~~~~~~~~~~~~~~~~~~~~~~~~~~~~~~~~   311 (651) |
|  | T PF10034.10 | 250 | FITADKFCK------------------ILYGLLVAHLLNFAVQFGNSMLLSSFFMSAVISALVVAKAESQIHKLPYQLLI   311 (651) |
|  | T ss\_pred |  | cCCHHHHHH------------------HHHHHHHHHHHHHHHHccchHHHHHHHHHHHHHHHHHHHHHhhhccCCHHHHH |
|  |
|  |
|  | Q ss\_pred |  | -------------------------------hCCCCCCccccCCcchhcchhhHhHhHHHHHHHHHHHHhhccHhhhccc |
|  | Q Q5T4D3 | 291 | -------------------------------MGTGPPAFTEVDNPASFADSMLVRAVNYNYYYSLNAWLLLCPWWLCFDW   339 (462) |
|  | Q Consensus | 291 | -------------------------------~~~~~~~~~~~~~~~~~~~~~~~~~~~~~~~~~~~~~~~~~p~~~~~~~   339 (462) |
|  |  |  | ................................. |
|  | T Consensus | 312 | ~~~~~~~~~~~~~~~~~~~~~~~~~~~~~~~~~~~~~~~~~~~~~~~~~~~~~~~~~~~~~~~~----------------   375 (651) |
|  | T PF10034.10 | 312 | WATQGLGFAAGTLGIKVAVAKVLSIADDSKFTSYRDFHTLLYTCAPEFDFLDQEAPVKLTKTLL----------------   375 (651) |
|  | T ss\_pred |  | HHHHHHHHHHHHHHHHHHHHHhcCcccccccCCCCCHHHHHHhcchhcCCCChHHHHHHHHhcH---------------- |
|  |
|  |
|  | Q ss\_pred |  | cccccCcccccchHHHHHHHHHHHHHHHHHHHHHhcCCCcchHH------------------------HHHHHHHHHHHH |
|  | Q Q5T4D3 | 340 | SMGCIPLIKSISDWRVIALAALWFCLIGLICQALCSEDGHKRRI------------------------LTLGLGFLVIPF   395 (462) |
|  | Q Consensus | 340 | ~~~~~~~~~~~~~~~~~~~~~~~~~~~~~~~~~~~~~~~~~~~~------------------------~~~~~~~~~~~~   395 (462) |
|  |  |  | .......+..+++........+.+++++.+. ..+.+++.+... |
|  | T Consensus | 376 | -------------~~~~~~~~~~~~~~~~~~~~~~~~~~~~~~~~~~~~~~~~~~~~~~~~~~~~~~~~~~~~~~~~~~~   442 (651) |
|  | T PF10034.10 | 376 | -------------LPSAIVAASAVIAKVGASEWEYWVRGKKSQVKSDSADEHDEGAQGQEANPRPHAEYVYHVLQAMAFV   442 (651) |
|  | T ss\_pred |  | -------------HHHHHHHHHHHHHHHHHHHHHHHHhcccccCCCCCccccccccccccCCCCCchHHHHHHHHHHHHH |
|  |
|  |
|  | Q ss\_pred |  | hhHhccccccchhHHHhhhhHHHHHHHHHHHHHHHH-HHhchhhHHHHHHHHHHHHHHHHHHHHHh |
|  | Q Q5T4D3 | 396 | LPASNLFFRVGFVVAERVLYLPSVGYCVLLTFGFGA-LSKHTKKKKLIAAVVLGILFINTLRCVLR   460 (462) |
|  | Q Consensus | 396 | ~~~~~~~~~~~~~~~~ry~~~~~~~~~ll~a~~~~~-~~~~~~~~~~~~~~~~~~~~~~~~~~~~~   460 (462) |
|  |  |  | .. .....||..+..|+++++++.++.+ +.+..+++.....+.+++++......... |
|  | T Consensus | 443 | ~~---------~~~~~R~~~~~~p~l~il~a~~~~~~~~~~~~~~~~~~~~~~~~~~~~~~~~~~~   499 (651) |
|  | T PF10034.10 | 443 | LM---------AVIIMRLKLFGTPALCVLASLVASRQFFSFLGDRRRHQAIVIALIAVMSVQGFSN   499 (651) |
|  | T ss\_pred |  | HH---------HHHHHHHHHHHHHHHHHHHHHHhcHHHHHhhhHHHHHHHHHHHHHHHHHhcchhh |
|  |
| --- | | | |
|  | Template alignmentCDD | | |
| 8. | PF04188.14 ; Mannosyl\_trans2 ; Mannosyltransferase (PIG-V) | | |
|  | Probability: 99.58%, E-value: 1.4e-12, Score: 117.76, Aligned cols: 331, Identities: 10%, Similarity: -0.047, | | |
|  |
|  | Q ss\_pred |  | cCchHHHH--hcccc-cCCCChhhhhcccccccccCCCCCccccCchHHHHHHHHHHHhCCCC-----------chHHHH |
|  | Q Q5T4D3 | 44 | FDDSEAIV--NNKDL-QAETPLGDLWHHDFWGSRLSSNTSHKSYRPLTVLTFRINYYLSGGFH-----------PVGFHV   109 (462) |
|  | Q Consensus | 44 | ~Dd~~~~~--~~~~~-~~~~~~~~~~~~~~~~~~~~~~~~~~~~~Pl~~~~~~~~~~~~gg~~-----------~~~~rl   109 (462) |
|  |  |  | |||.+|.. .+++- ...+....+ +|+++++......++ |.+ ..+.|+ |
|  | T Consensus | 60 | wD~~~y~~~~ia~~g~y~~~~~~~f-------------------~Pl~p~l~~~~~~l~-~~~~~~~~~~~~~~~~~~~~   119 (432) |
|  | T PF04188.14 | 60 | WDSVFFIKNITSKNGKPQFEHEYAF-------------------SQLWTFFVRLFIKSN-NDSIYHALRVGVAIENVLFY   119 (432) |
|  | T ss\_pred |  | ccHHHHhhhhHHHcCCCcccccccc-------------------hHHHHHHHHHHHHhc-ccchHHHHHHHHHHHHHHHH |
|  |
|  |
|  | Q ss\_pred |  | HH-HHHHHHHHHHHHHHHHHHhccccccccccccchHHHHHHHHHHHHHHCHHHHHHHHhHhcHHHHHHHHHHHHHHHHH |
|  | Q Q5T4D3 | 110 | VN-ILLHSGISVLMVDVFSVLFGGLQYTSKGRRLHLAPRASLLAALLFAVHPVHTECVAGVVGRADLLCALFFLLSFLGY   188 (462) |
|  | Q Consensus | 110 | ~~-~~~~~l~~~l~~~l~~~l~~~~~~~~~~~~~~~~~~~a~~aall~~~~p~~~~~~~~~~~~~~~~~~~f~ll~~~~~   188 (462) |
|  |  |  | ++ .+++++++..+|.++++.++ +++.|..++++++++|..... ... .+|.+..++.+++++++ |
|  | T Consensus | 120 | ~s~~~~~~~~~~~ly~l~~~~~~-------------~~~~a~~a~~l~~~~P~~~~~-~~~--~~E~l~~~l~~~~~~~~   183 (432) |
|  | T PF04188.14 | 120 | LSGIVLYFLTKKIFSQNIRQSQF-------------ARTIAKKTSLLFFLTSAAGFL-TSI--YSEPLSFFFAFVGIWSR   183 (432) |
|  | T ss\_pred |  | HHHHHHHHHHHHHHHhhhHhhhc-------------cHHHHHHHHHHHhhCCchHHh-hcc--CcHHHHHHHHHHHHHHH |
|  |
|  |
|  | Q ss\_pred |  | HHHHHHcCCCCCchHHHHHHHHHHHHHHHHh-chHHHHHHHHHHHHHHHHHh------CCCChHHHHHHHhhhccchHhh |
|  | Q Q5T4D3 | 189 | CKAFRESNKEGAHSSTFWVLLSIFLGAVAML-CKEQGITVLGLNAVFDILVI------GKFNVLEIVQKVLHKDKSLENL   261 (462) |
|  | Q Consensus | 189 | ~~~~~~~~~~~~~~~~~~~~~~~~~~~la~l-~k~~~~~~~~~~~~~~~~~~------~~~~~~~~~~~~~~~~~~~~~~   261 (462) |
|  |  |  | .+..+++++ .++...++++.+++.+ +|.+++...+......+... ++++.++..+.. |
|  | T Consensus | 184 | ~~~~~~~~~------~~~~~~~~~~~~la~~~~R~~g~~~~~~~~~~~l~~~~~~~~~~~~~~~~~~~~~----------   247 (432) |
|  | T PF04188.14 | 184 | ECSISVPVL------GQFDISWRYWFPYSFISMACFTLASLNRSNCVLLGIYFIFDLIELTKNRKFVKAI----------   247 (432) |
|  | T ss\_pred |  | HhccCCCCC------CchhHHHHHHHHHHHHHHHhHHHHHHHHHHHHHHHHHHHHHHHHhhhhHHHHHHH---------- |
|  |
|  |
|  | Q ss\_pred |  | chhhhhhHHHHHHHHHHHHHHHHHHHHHH-hCCCCCCccccCCcchhc--------chhhHhHhHHHHHHHHHHHHhhcc |
|  | Q Q5T4D3 | 262 | GMLRNGGLLFRMTLLTSGGAGMLYVRWRI-MGTGPPAFTEVDNPASFA--------DSMLVRAVNYNYYYSLNAWLLLCP   332 (462) |
|  | Q Consensus | 262 | ~~~~~~~~~~~~~~~~~~~~~~~~~~~~~-~~~~~~~~~~~~~~~~~~--------~~~~~~~~~~~~~~~~~~~~~~~p   332 (462) |
|  |  |  | ........+....+....... .................. .....+......... |
|  | T Consensus | 248 | --------~~~~~~~~~~~~p~~~~~~~~~~~~f~~~~~~w~~~~~~~~~~~~~~~~~~~~q~~yw~~g~~---------   310 (432) |
|  | T PF04188.14 | 248 | --------CFPLLSGSLMFSALLYQQYYLPYKTFCPQRGEWCKSQLFSSIFITKTSLYSYIQSHYWGVGLL---------   310 (432) |
|  | T ss\_pred |  | --------HHHHHHHHHHHHHHHHHHHHHHHHHHCCCCChhHhCCCCCchhhcccchHHHHHHHcCCCcch--------- |
|  |
|  |
|  | Q ss\_pred |  | HhhhccccccccCcccccchHHHHHHHHHHHHHHHHHHHHHhcCCCcchHHHHHHHHHHHHHHhhHhccccccchhHHHh |
|  | Q Q5T4D3 | 333 | WWLCFDWSMGCIPLIKSISDWRVIALAALWFCLIGLICQALCSEDGHKRRILTLGLGFLVIPFLPASNLFFRVGFVVAER   412 (462) |
|  | Q Consensus | 333 | ~~~~~~~~~~~~~~~~~~~~~~~~~~~~~~~~~~~~~~~~~~~~~~~~~~~~~~~~~~~~~~~~~~~~~~~~~~~~~~~r   412 (462) |
|  |  |  | ........+.........+..+..+....++++++.................. ........| |
|  | T Consensus | 311 | ------------~~~~~~~~~~~ll~~p~~~l~~~~~~~~~~~~~~~~~~~~~~~~~~~~~~~~~------~~~~~~~~R   372 (432) |
|  | T PF04188.14 | 311 | ------------KYWTPNNIPNFLFAVPNIIILIYSSIYFSKIYPSYNLKALVWITRALVVIVCF------FAHVQILNR   372 (432) |
|  | T ss\_pred |  | ------------hcCCccchhHHHhHHHHHHHHHHHHHHHHhhCCccccHHHHHHHHHHHHHHHH------HHHHHHHHH |
|  |
|  |
|  | Q ss\_pred |  | hhhHHHHHHHHHHHHHHHHHHhchhhHH------HHHHHHHHHHHHHHHHHHHhcC |
|  | Q Q5T4D3 | 413 | VLYLPSVGYCVLLTFGFGALSKHTKKKK------LIAAVVLGILFINTLRCVLRSG   462 (462) |
|  | Q Consensus | 413 | y~~~~~~~~~ll~a~~~~~~~~~~~~~~------~~~~~~~~~~~~~~~~~~~~~~   462 (462) |
|  |  |  | |.. ..|++.+.++..+.+..++.++.. .....+...+....+....-.+ |
|  | T Consensus | 373 | ~~~-~~P~l~~~~a~~~~~~~~~~~~~~~~~~~~~~~~~~~~~~~~~~~~~~l~~~   427 (432) |
|  | T PF04188.14 | 373 | IAS-FLPLHLWYLADRLVKTSDPKKMENPKGDDKIVKFYIYWLAFWIPLQTILFAA   427 (432) |
|  | T ss\_pred |  | HHh-ccHHHHHHHHHHHhcCCCCCcCCCCCCCCHHHHHHHHHHHHHHHHHHHHHHc |
|  |
| --- | | | |
|  | Template alignmentCDD | | |
| 9. | PF12250.9 ; AftA\_N ; Arabinofuranosyltransferase N terminal | | |
|  | Probability: 99.53%, E-value: 1.6e-11, Score: 109.68, Aligned cols: 321, Identities: 10%, Similarity: -0.035, | | |
|  |
|  | Q ss\_pred |  | HHHHHHHHHHHHHHHhhhcCC-CcccCchHHHHhcccccCCCChhhhhcccccccccCCCCCccccCchHHHHHHHHHHH |
|  | Q Q5T4D3 | 20 | FWAKLVVGSVAIVCFARSYDG-DFVFDDSEAIVNNKDLQAETPLGDLWHHDFWGSRLSSNTSHKSYRPLTVLTFRINYYL   98 (462) |
|  | Q Consensus | 20 | ~~~~~~l~~~~~~~~~~~~~~-~~~~Dd~~~~~~~~~~~~~~~~~~~~~~~~~~~~~~~~~~~~~~~Pl~~~~~~~~~~~   98 (462) |
|  |  |  | ....++...+...++...... +.+.||..+....+.+.++....++.. .+..+.|||+++++.+....+ |
|  | T Consensus | 75 | ~~~~l~~~~l~~~L~~t~~~~~gl~~D~~~~~~~~~~~~~~~~~~d~~~----------~~~~~~YPPl~~~l~~~~~~l   144 (432) |
|  | T PF12250.9 | 75 | SPAALVITTLGIPLSATRLYLDGINVDQGFRTQFLTWMGYTIHLSDMNY----------IDMPSYYPGAWFWIGGRLANL   144 (432) |
|  | T ss\_pred |  | HHHHHHHHHHHHHHhcCcccCCCccccHHHHHHHHHHHHhccccccccC----------CCCcccCChHHHHHHHHHHHH |
|  |
|  |
|  | Q ss\_pred |  | hCCCCc-hHHHHHHHHHHHHHHHHHHHHHHHHhccccccccccccchHHHHH-HHHHHHHHHCHHHHHHHHhHhcHHHHH |
|  | Q Q5T4D3 | 99 | SGGFHP-VGFHVVNILLHSGISVLMVDVFSVLFGGLQYTSKGRRLHLAPRAS-LLAALLFAVHPVHTECVAGVVGRADLL   176 (462) |
|  | Q Consensus | 99 | ~gg~~~-~~~rl~~~~~~~l~~~l~~~l~~~l~~~~~~~~~~~~~~~~~~~a-~~aall~~~~p~~~~~~~~~~~~~~~~   176 (462) |
|  |  |  | + |.++ ...|..+++...++...+|.+.|++++ ++..+ .++++..++.|.. ......+.. |
|  | T Consensus | 145 | ~-G~~~~~a~r~~~~l~~~l~~~~~y~l~r~l~~-------------~~~~al~ia~~~~~~~~~~-----~~~~~y~~l   205 (432) |
|  | T PF12250.9 | 145 | L-GLAGWEVFQPWALISLATAGSILVPVWQRICG-------------SLTVASGIALVTTSITIVM-----SADEPYAAI   205 (432) |
|  | T ss\_pred |  | h-CCCHHHHhHHHHHHHHHHHHHHHHHHHHHHHC-------------CHHHHHHHHHHHHHHHHhc-----CCCCcHHHH |
|  |
|  |
|  | Q ss\_pred |  | HHHHHHHHHHHHHHHHHHcCCCCCchHHHHHHHHHHHHHHHHhchHHHHHHHHHHHH----HHHHHhCCCChHHHHHHHh |
|  | Q Q5T4D3 | 177 | CALFFLLSFLGYCKAFRESNKEGAHSSTFWVLLSIFLGAVAMLCKEQGITVLGLNAV----FDILVIGKFNVLEIVQKVL   252 (462) |
|  | Q Consensus | 177 | ~~~f~ll~~~~~~~~~~~~~~~~~~~~~~~~~~~~~~~~la~l~k~~~~~~~~~~~~----~~~~~~~~~~~~~~~~~~~   252 (462) |
|  |  |  | ...+....++++.+..+++ ++.....+++.+++.++|............ ......++++ |
|  | T Consensus | 206 | ~~~~l~~~l~~~~r~l~~~--------~~~~~~~gl~lgl~~l~y~~~~~~~~~~~~~~~~l~~~~~~~~~---------   268 (432) |
|  | T PF12250.9 | 206 | ITMGVPAATVMMRRALTGS--------LWPLIGLTLYIGVSAAMYTLFTAVVALSVCVMAALFAVVFDHSI---------   268 (432) |
|  | T ss\_pred |  | HHHHHHHHHHHHHHHhcCC--------chHHHHHHHHHHHHHHHhHHHHHHHHHHHHHHHHHHHHHcCCCc--------- |
|  |
|  |
|  | Q ss\_pred |  | hhccchHhhchhhhhhHHHHHHHHHHHHHHHHHHHHHHhCCCCCCccccCCcchhcchhhHhHhHHHHHHHHHHHHhhcc |
|  | Q Q5T4D3 | 253 | HKDKSLENLGMLRNGGLLFRMTLLTSGGAGMLYVRWRIMGTGPPAFTEVDNPASFADSMLVRAVNYNYYYSLNAWLLLCP   332 (462) |
|  | Q Consensus | 253 | ~~~~~~~~~~~~~~~~~~~~~~~~~~~~~~~~~~~~~~~~~~~~~~~~~~~~~~~~~~~~~~~~~~~~~~~~~~~~~~~p   332 (462) |
|  |  |  | +.....................++.......+...+.....-...+..+........ |
|  | T Consensus | 269 | ------------~~~~~~~~~~~~a~~~a~~~~~P~l~~~~~~~~~~~~~~~~~~~~~~~~~~~p~~~~-----------   325 (432) |
|  | T PF12250.9 | 269 | ------------KPLLRLLIIGTGSALIASTVWAPYLTAILSGQPHSGATAMHYLPPTGAQVPMPMLQF-----------   325 (432) |
|  | T ss\_pred |  | ------------hHHHHHHHHHHHHHHHHHHHHHHHHHHHHhCCCCCCcchHhcCCccccCCCCccccc----------- |
|  |
|  |
|  | Q ss\_pred |  | HhhhccccccccCcccccchHHHHHHHHHHHHHHHHHHHHHhcCCCcchHHHHHHHHHHHHHHhhHhccccccchhHHHh |
|  | Q Q5T4D3 | 333 | WWLCFDWSMGCIPLIKSISDWRVIALAALWFCLIGLICQALCSEDGHKRRILTLGLGFLVIPFLPASNLFFRVGFVVAER   412 (462) |
|  | Q Consensus | 333 | ~~~~~~~~~~~~~~~~~~~~~~~~~~~~~~~~~~~~~~~~~~~~~~~~~~~~~~~~~~~~~~~~~~~~~~~~~~~~~~~r   412 (462) |
|  |  |  | .....+.+++......+++ ++..+.....+....+..+...............| |
|  | T Consensus | 326 | -------------------------~~~~~L~l~Glv~l~~~~r-~~~~~~l~~~~~~~y~w~~~~~~~~~~~~~ll~~R   379 (432) |
|  | T PF12250.9 | 326 | -------------------------NLVGLLCLLGLAYLIVRIA-DPDVRSMLIAQIVFYGWIVTSMIVSLSGKTLLGFR   379 (432) |
|  | T ss\_pred |  | -------------------------cHHHHHHHHHHHHHHHHhc-CHhHHHHHHHHHHHHHHHHHHHHHHHhCCCchhHh |
|  |
|  |
|  | Q ss\_pred |  | hhhHHHHHHHHHHHHHHHHHHhc |
|  | Q Q5T4D3 | 413 | VLYLPSVGYCVLLTFGFGALSKH   435 (462) |
|  | Q Consensus | 413 | y~~~~~~~~~ll~a~~~~~~~~~   435 (462) |
|  |  |  | +..+..+.+++.++.++.++.+. |
|  | T Consensus | 380 | ~~~~l~~~l~~~~a~gi~~l~~~   402 (432) |
|  | T PF12250.9 | 380 | LDAIITIQLATAGMLALAELRLV   402 (432) |
|  | T ss\_pred |  | HHHHHHHHHHHHHHHHHHHHHHc |
|  |
| --- | | | |
|  | Template alignmentCDD | | |
| 10. | PF09913.10 ; DUF2142 ; Predicted membrane protein (DUF2142) | | |
|  | Probability: 99.5%, E-value: 8.8e-12, Score: 111.53, Aligned cols: 324, Identities: 12%, Similarity: -0.019, | | |
|  |
|  | Q ss\_pred |  | HHHhhhcCCCcccCchHHHHhcccccCCCChhhhhcccc----------------------------------------- |
|  | Q Q5T4D3 | 32 | VCFARSYDGDFVFDDSEAIVNNKDLQAETPLGDLWHHDF-----------------------------------------   70 (462) |
|  | Q Consensus | 32 | ~~~~~~~~~~~~~Dd~~~~~~~~~~~~~~~~~~~~~~~~-----------------------------------------   70 (462) |
|  |  |  | ..+..........||..|+..+....+++......+... |
|  | T Consensus | 4 | ~~~~~~~P~~~~pDE~~H~~~a~~ia~g~~~~~~~~~~~~~~~~~~~~~~~~~~~~~~~~~~~~~~~~~~~~~~~~~~~~   83 (405) |
|  | T PF09913.10 | 4 | LAFAVVMPPFQVPDEDGHFIRAYLISRGEFVGRGAPRVPGTVVLSMMRYPEMGERFGRFKPRELVRDLIPHPGSVSPEVP   83 (405) |
|  | T ss\_pred |  | eehhhccCCCCCCChHHHHHHHHHHHcCceeeecCCCCCCcccchhccCcccccccCCCCchHHHhccCCCCCCCCCCcc |
|  |
|  |
|  | Q ss\_pred |  | -----cccccCCCCCccc-----cCchHHHHHHHHH---HHhCCCC----chHHHHHHHHHHHHHHHHHHHHHHHHhccc |
|  | Q Q5T4D3 | 71 | -----WGSRLSSNTSHKS-----YRPLTVLTFRINY---YLSGGFH----PVGFHVVNILLHSGISVLMVDVFSVLFGGL   133 (462) |
|  | Q Consensus | 71 | -----~~~~~~~~~~~~~-----~~Pl~~~~~~~~~---~~~gg~~----~~~~rl~~~~~~~l~~~l~~~l~~~l~~~~   133 (462) |
|  |  |  | .......+..... +||++++..+... +++ |.+ ....|+.+++++++++.+++.++++.. |
|  | T Consensus | 84 | ~~~~~~~~~~~~~~~~~~~~~~~~pPl~y~~~a~~~~l~~~~-~~~~~~~~~~~Rl~s~l~~~~~~~~~~~~~~~~~---   159 (405) |
|  | T PF09913.10 | 84 | SLNLGNLDVRHRWLPWSIIGSSLYCPLVYMPASLGIATVRIL-SGSPLLMMYGARLFNVIVFAAALAISFRLAPRYR---   159 (405) |
|  | T ss\_pred |  | ccccccCCCcccccCccccCccccCHHhHHHHHHHHHHHHHc-cCCHHHHHHHHHHHHHHHHHHHHHHHHHHChhhH--- |
|  |
|  |
|  | Q ss\_pred |  | cccccccccchHHHHHHHHHHHHHHCHHHHHHHHhHhcHHHHHHHHHHHHHHHHHHHHHHHc-CCCCCchHHHHHHHHHH |
|  | Q Q5T4D3 | 134 | QYTSKGRRLHLAPRASLLAALLFAVHPVHTECVAGVVGRADLLCALFFLLSFLGYCKAFRES-NKEGAHSSTFWVLLSIF   212 (462) |
|  | Q Consensus | 134 | ~~~~~~~~~~~~~~~a~~aall~~~~p~~~~~~~~~~~~~~~~~~~f~ll~~~~~~~~~~~~-~~~~~~~~~~~~~~~~~   212 (462) |
|  |  |  | .++++++++|.....+... ++|.+..++.+++++++.+..+++ + ++..++.++ |
|  | T Consensus | 160 | -----------------~~~~~~a~~P~~~~~~~~~--~~D~~~~~~~~~~~~~~~~~~~~~~~-------~~~~~~~~~   213 (405) |
|  | T PF09913.10 | 160 | -----------------ALFTAVALMPMTLQQAGGI--SADLVTIAFSFVGFSLVLHSREHFVS-------RRLLILIVL   213 (405) |
|  | T ss\_pred |  | -----------------HHHHHHHhchHHHHHHhcC--ChHHHHHHHHHHHHHHHHhcCCcCCC-------HHHHHHHHH |
|  |
|  |
|  | Q ss\_pred |  | HHHHHHhchHHHHHHHHHHHHHHHHHhCCCChHHHHHHHhhhccchHhhchhhhhhHHHHHHHHHHHHHHHHHHHHHHhC |
|  | Q Q5T4D3 | 213 | LGAVAMLCKEQGITVLGLNAVFDILVIGKFNVLEIVQKVLHKDKSLENLGMLRNGGLLFRMTLLTSGGAGMLYVRWRIMG   292 (462) |
|  | Q Consensus | 213 | ~~~la~l~k~~~~~~~~~~~~~~~~~~~~~~~~~~~~~~~~~~~~~~~~~~~~~~~~~~~~~~~~~~~~~~~~~~~~~~~   292 (462) |
|  |  |  | +.+++.++| ..++.+..+..+..+++++ ++...........++....+........ |
|  | T Consensus | 214 | ~~~l~~~~K---~~~~~~~l~~~~~~~~~~~---------------------~~~~~~~~~~~~~~~~~~~~~~~~~~~~   269 (405) |
|  | T PF09913.10 | 214 | VFVMWVLCK---SSIWALPLLLLIPVSAFKN---------------------RLTWAAYLGVASVCMVGALLVWNNVTAP   269 (405) |
|  | T ss\_pred |  | HHHHHHHHH---HHHHHHHHHHHHhHHhhCc---------------------hHHHHHHHHHHHHHHHHHHHHHHhhcCC |
|  |
|  |
|  | Q ss\_pred |  | CCCCCc----cccCCcchhcchhhHhHhHHHHHHHHHHHHhhccHhhhccccccccCcccccchHHHHHHHHHHHHHHHH |
|  | Q Q5T4D3 | 293 | TGPPAF----TEVDNPASFADSMLVRAVNYNYYYSLNAWLLLCPWWLCFDWSMGCIPLIKSISDWRVIALAALWFCLIGL   368 (462) |
|  | Q Consensus | 293 | ~~~~~~----~~~~~~~~~~~~~~~~~~~~~~~~~~~~~~~~~p~~~~~~~~~~~~~~~~~~~~~~~~~~~~~~~~~~~~   368 (462) |
|  |  |  | ...... ....+.........++.........+.+............. ..................+.++.. |
|  | T Consensus | 270 | ~~~~~~~~~~~~~~~~~~~~~~~~~~p~~~~~~~~~~~~~~~~~~~~~~~g-----~~g~~~~~lp~~~~~~~~~~l~~~   344 (405) |
|  | T PF09913.10 | 270 | NLETFRAVRLTHGVDMPANIRLVGAHPLMFVRYLIGVVGSNLKPEIGQFIG-----AFGWLRFPLPSWVRAAYLLLVLVT   344 (405) |
|  | T ss\_pred |  | CccchhhccccCCCChHHHHHHHHhCHHHHHHHHHHHHHHhhHHHHHHHHH-----HhhccCCCccHHHHHHHHHHHHHH |
|  |
|  |
|  | Q ss\_pred |  | HHHHHhcCCCcchHHHHHHHHHHHHHHhhHhccccccchh---------------HHHhhh |
|  | Q Q5T4D3 | 369 | ICQALCSEDGHKRRILTLGLGFLVIPFLPASNLFFRVGFV---------------VAERVL   414 (462) |
|  | Q Consensus | 369 | ~~~~~~~~~~~~~~~~~~~~~~~~~~~~~~~~~~~~~~~~---------------~~~ry~   414 (462) |
|  |  |  | .....++++++.+.........++...+............ .+.||+ |
|  | T Consensus | 345 | ~~~~~~~~~~~~~~~~~~~~~~~~~~~~i~~~~~~~~~~~~~~~i~G~~~~~~~~~QgRY~   405 (405) |
|  | T PF09913.10 | 345 | AVTEFPAKSFRTWERGVLALVLLGGVLFVHAAMCISDTTLCSGTLNSGCRDESIVFQGRYL   405 (405) |
|  | T ss\_pred |  | HHccCCccCCCHHHHHHHHHHHHHHHHHHHHHHHHhccCCCcceeccCCCCccceeceecC |
|  |
| --- | | | |
|  | Template alignmentCDD | | |
| 11. | PF13231.7 ; PMT\_2 ; Dolichyl-phosphate-mannose-protein mannosyltransferase | | |
|  | Probability: 99.5%, E-value: 1.6e-12, Score: 99.97, Aligned cols: 157, Identities: 18%, Similarity: 0.208, | | |
|  |
|  | Q ss\_pred |  | ccCchHHHHHHHHHHHhCCCCchHHHHHHHHHHHHHHHHHHHHHHHHhccccccccccccchHHHHHHHHHHHHHHCHHH |
|  | Q Q5T4D3 | 83 | SYRPLTVLTFRINYYLSGGFHPVGFHVVNILLHSGISVLMVDVFSVLFGGLQYTSKGRRLHLAPRASLLAALLFAVHPVH   162 (462) |
|  | Q Consensus | 83 | ~~~Pl~~~~~~~~~~~~gg~~~~~~rl~~~~~~~l~~~l~~~l~~~l~~~~~~~~~~~~~~~~~~~a~~aall~~~~p~~   162 (462) |
|  |  |  | +|||++.++.+....++ |++....|+.+.+++.+++..+|.+.|+.. +++.+..++.+++++|.. |
|  | T Consensus | 1 | ~~~P~~~~~~~~~~~l~-g~~~~~~~~~~~~~~~~~~~~~~~~~~~~~--------------~~~~~~~~~~~~~~~p~~   65 (159) |
|  | T PF13231.7 | 1 | DKPPASLWVMELSTRIF-GVNSWAMLVPQALLGVAAVALLYATVRRRF--------------GAVAGLLAGLILAVTPVA   65 (159) |
|  | T ss\_pred |  | CCChHHHHHHHHHHHHH-CCCHHHHHHHHHHHHHHHHHHHHHHHHHHH--------------HHHHHHHHHHHHHhcHHH |
|  |
|  |
|  | Q ss\_pred |  | HHHHHhHhcHHHHHHHHHHHHHHHHHHHHHHHcCCCCCchHHHHHHHHHHHHHHHHhchHHHHHHHHHHHH--HHHHHhC |
|  | Q Q5T4D3 | 163 | TECVAGVVGRADLLCALFFLLSFLGYCKAFRESNKEGAHSSTFWVLLSIFLGAVAMLCKEQGITVLGLNAV--FDILVIG   240 (462) |
|  | Q Consensus | 163 | ~~~~~~~~~~~~~~~~~f~ll~~~~~~~~~~~~~~~~~~~~~~~~~~~~~~~~la~l~k~~~~~~~~~~~~--~~~~~~~   240 (462) |
|  |  |  | ....... .+|....++..+++++..+..++++ .+....++++.+++..+|+.+....+...+ .....++ |
|  | T Consensus | 66 | ~~~~~~~--~~~~~~~~~~~~~~~~~~~~~~~~~-------~~~~~~~~~~~~l~~~~k~~~~~~~~~~~~~~~~~~~~~   136 (159) |
|  | T PF13231.7 | 66 | AMMFRFN--NPDALLVLLMIAATWAMLRAVEDGR-------WRWLIVCGAFVGVGFLTKQLAVMLIVPGLALTYLVAGPP   136 (159) |
|  | T ss\_pred |  | HHhhhcC--CHHHHHHHHHHHHHHHHHHHHHcCC-------hHHHHHHHHHHHHHHHcccchHHhhHHHHHHHHHHhCCh |
|  |
|  |
|  | Q ss\_pred |  | CCChHHHHHHHhhhccchHhhchhhhhhHHHHHHHHHHHHHHHH |
|  | Q Q5T4D3 | 241 | KFNVLEIVQKVLHKDKSLENLGMLRNGGLLFRMTLLTSGGAGML   284 (462) |
|  | Q Consensus | 241 | ~~~~~~~~~~~~~~~~~~~~~~~~~~~~~~~~~~~~~~~~~~~~   284 (462) |
|  |  |  | +++ +..................+ |
|  | T Consensus | 137 | ~~~---------------------~~~~~~~~~~~~~~~~~~~w   159 (159) |
|  | T PF13231.7 | 137 | KIG---------------------VRIAQLFAAGTSMIVAAGWW   159 (159) |
|  | T ss\_pred |  | hHH---------------------HHHHHHHHHHHHHHHHHhcC |
|  |
| --- | | | |
|  | Template alignmentCDD | | |
| 12. | PF11028.9 ; DUF2723 ; Protein of unknown function (DUF2723) | | |
|  | Probability: 99.48%, E-value: 1.8e-12, Score: 102.57, Aligned cols: 155, Identities: 17%, Similarity: 0.135, | | |
|  |
|  | Q ss\_pred |  | CchHHHHhcccccCCCChhhhhcccccccccCCCCCccccCchHHHHHHHHHHHh-CCCCchHHHHHHHHHHHHHHHHHH |
|  | Q Q5T4D3 | 45 | DDSEAIVNNKDLQAETPLGDLWHHDFWGSRLSSNTSHKSYRPLTVLTFRINYYLS-GGFHPVGFHVVNILLHSGISVLMV   123 (462) |
|  | Q Consensus | 45 | Dd~~~~~~~~~~~~~~~~~~~~~~~~~~~~~~~~~~~~~~~Pl~~~~~~~~~~~~-gg~~~~~~rl~~~~~~~l~~~l~~   123 (462) |
|  |  |  | ||..|...+.+........ .|++..+......++ .|.++...|+.+.+++.+++.++| |
|  | T Consensus | 1 | D~~~y~~~a~~~~~~~~p~---------------------~~l~~~~~~~~~~~~~~~~~~~~~r~~~~l~~~l~~~~~~   59 (188) |
|  | T PF11028.9 | 1 | DCGEYITAANKLEVGHPPG---------------------APLFMLLGRLFSFFAEPEMVAVWINRLSALCSSFTILFLY   59 (188) |
|  | T ss\_pred |  | ChHHHHHHHhhcCCCCCCC---------------------hHHHHHHHHHHHhcCCCCcHHHHHHHHHHHHHHHHHHHHH |
|  |
|  |
|  | Q ss\_pred |  | HHHHHHhccccccccccccchHHH------------------HHHHHHHHHHHCHHHHHHHHhHhcHHHHHHHHHHHHHH |
|  | Q Q5T4D3 | 124 | DVFSVLFGGLQYTSKGRRLHLAPR------------------ASLLAALLFAVHPVHTECVAGVVGRADLLCALFFLLSF   185 (462) |
|  | Q Consensus | 124 | ~l~~~l~~~~~~~~~~~~~~~~~~------------------~a~~aall~~~~p~~~~~~~~~~~~~~~~~~~f~ll~~   185 (462) |
|  |  |  | .++|+.. +++ .+++++++++++|.....+... .+|.+..++.++++ |
|  | T Consensus | 60 | ~~~~~~~--------------~~~~~~~~~~~~~~~~~~~~~~a~~a~~l~~~~p~~~~~s~~~--~~d~~~~~~~~~~l   123 (188) |
|  | T PF11028.9 | 60 | WSITMFA--------------KKIMQRKDRDWSRGDQIATLGAGIIGALAYTFSDSFWFSAVEG--EVYAMSSLFTAAIF   123 (188) |
|  | T ss\_pred |  | HHHHHHH--------------HHHHhccccccccchHHHHHHHHHHHHHHHHHchhHHHHhhhc--chhHHHHHHHHHHH |
|  |
|  |
|  | Q ss\_pred |  | HHHHHHHHHcCCCCCchHH-------------HHHHHHHHHHHHHHhchHHHHHHHHHHHHHHHHHhCCCC |
|  | Q Q5T4D3 | 186 | LGYCKAFRESNKEGAHSST-------------FWVLLSIFLGAVAMLCKEQGITVLGLNAVFDILVIGKFN   243 (462) |
|  | Q Consensus | 186 | ~~~~~~~~~~~~~~~~~~~-------------~~~~~~~~~~~la~l~k~~~~~~~~~~~~~~~~~~~~~~   243 (462) |
|  |  |  | +++.+..++++ . ++..+++++.+++.++|+.++.+.+..++..+...++++ |
|  | T Consensus | 124 | ~~~~~~~~~~~-------~~~~~~~~~~~~~~~~~~l~g~~~~la~~~k~~~~~~~~~~~~~~~~~~~~~~   187 (188) |
|  | T PF11028.9 | 124 | WMILKWDAEMI-------GIKHGEIKDSRSPMRWMILIWFMFGLAIGVHLLGLLAVPAIAYVIYFNLWEKT   187 (188) |
|  | T ss\_pred |  | HHHHHHHHhcc-------CccCCCcCCCCChHHHHHHHHHHHHHHHHHHHHHHHHHHHHHHHHHHhhHHhc |
|  |
| --- | | | |
|  | Template alignmentCDD | | |
| 13. | PF04602.13 ; Arabinose\_trans ; Mycobacterial cell wall arabinan synthesis protein | | |
|  | Probability: 99.45%, E-value: 1.6e-10, Score: 102.36, Aligned cols: 356, Identities: 9%, Similarity: -0.032, | | |
|  |
|  | Q ss\_pred |  | HHHHHHHHhhhcCCCcccCchHHHHhcccccCCCChhhhhcccccccccCCCCCccccCchHHHHHHHHHHH--hCCCCc |
|  | Q Q5T4D3 | 27 | GSVAIVCFARSYDGDFVFDDSEAIVNNKDLQAETPLGDLWHHDFWGSRLSSNTSHKSYRPLTVLTFRINYYL--SGGFHP   104 (462) |
|  | Q Consensus | 27 | ~~~~~~~~~~~~~~~~~~Dd~~~~~~~~~~~~~~~~~~~~~~~~~~~~~~~~~~~~~~~Pl~~~~~~~~~~~--~gg~~~   104 (462) |
|  |  |  | .++...+....+..+...||.+|...+++..+++...+.+. .+....+|+... +.+.... + |.+. |
|  | T Consensus | 51 | ~~v~~~l~~w~~~gp~~~DEG~Yl~~ar~~~~~G~~~npy~-----------~~~~~~~Pfg~~-~~l~~~~~~~-g~s~   117 (471) |
|  | T PF04602.13 | 51 | LIVGAILLGWYFIGANTADDGYILNMARVAGHAGYMANYYR-----------WYGVPEAPFGWF-YDVTAALAAL-STAS   117 (471) |
|  | T ss\_pred |  | HHHHHHHHHHHHhccCCCChHHHHHHHHHHHhcCCccccch-----------hcCCCCCCcHHH-HHHHHHHHHh-cCCh |
|  |
|  |
|  | Q ss\_pred |  | hHHHHHHHHHHHHHHHHHHHHHHHHhccccccccccccchHHHHHHHHHHHHHHCHHHHHHHHhHhcHHHHHHHHHHHHH |
|  | Q Q5T4D3 | 105 | VGFHVVNILLHSGISVLMVDVFSVLFGGLQYTSKGRRLHLAPRASLLAALLFAVHPVHTECVAGVVGRADLLCALFFLLS   184 (462) |
|  | Q Consensus | 105 | ~~~rl~~~~~~~l~~~l~~~l~~~l~~~~~~~~~~~~~~~~~~~a~~aall~~~~p~~~~~~~~~~~~~~~~~~~f~ll~   184 (462) |
|  |  |  | ...|++++++++++..+++....+.. .....+++.+..++.+..+....... ... +.|....++.+++ |
|  | T Consensus | 118 | ~~lRl~~ll~~l~~w~lL~~~vl~rl---------~~~~~~~~~a~~~aal~~la~wlp~~-~~l--r~Ep~~al~~~~~   185 (471) |
|  | T PF04602.13 | 118 | PFVRLTTLIASILCWWIISREVIPRL---------GRRARHTPAVYWTAAAVFLAFWLPYN-NGL--RPEPVIAVGALLT   185 (471) |
|  | T ss\_pred |  | HHHHHHHHHHHHHHHHHHHHHHHHHh---------ccccCCcHHHHHHHHHHHHHHHcccc-CCC--CcHHHHHHHHHHH |
|  |
|  |
|  | Q ss\_pred |  | HHHHHHHHHHcCCCCCchHHHHHHHHHHHHHHHHhchHHHHHHHHHHHHHHHHHhCCCChHHHHHHHhhhccchHhhchh |
|  | Q Q5T4D3 | 185 | FLGYCKAFRESNKEGAHSSTFWVLLSIFLGAVAMLCKEQGITVLGLNAVFDILVIGKFNVLEIVQKVLHKDKSLENLGML   264 (462) |
|  | Q Consensus | 185 | ~~~~~~~~~~~~~~~~~~~~~~~~~~~~~~~la~l~k~~~~~~~~~~~~~~~~~~~~~~~~~~~~~~~~~~~~~~~~~~~   264 (462) |
|  |  |  | +++..+..++++ .....+++++.++++.+|++++.....+.+......++.+ |
|  | T Consensus | 186 | l~l~~ra~~~~~-------~~~~alag~~~gla~~aKPtg~~~la~ll~~~~~~~r~~~---------------------   237 (471) |
|  | T PF04602.13 | 186 | WISVERAIATGR-------LLPAAIATIIAAFSLAAGPTGLMAVAALLAGSRPLLAILI---------------------   237 (471) |
|  | T ss\_pred |  | HHHHHHHHhcCC-------cHHHHHHHHHHHHHHhcCHhHHHHHHHHHHHHHHHHHHHH--------------------- |
|  |
|  |
|  | Q ss\_pred |  | hhhhHHHH--H-----------------HHHHHHHHHHHHHHHHHhCCCCCCccccCCcchhcchhhHhHhHHHHHHHHH |
|  | Q Q5T4D3 | 265 | RNGGLLFR--M-----------------TLLTSGGAGMLYVRWRIMGTGPPAFTEVDNPASFADSMLVRAVNYNYYYSLN   325 (462) |
|  | Q Consensus | 265 | ~~~~~~~~--~-----------------~~~~~~~~~~~~~~~~~~~~~~~~~~~~~~~~~~~~~~~~~~~~~~~~~~~~   325 (462) |
|  |  |  | ... . ....+...........+..++..+..+................+..++.... |
|  | T Consensus | 238 | -----~r~~~~~~~~~~~~~~~~~~~~~~la~~~a~~~~~l~~~f~d~sl~~~~~~~~~~~~~~~~~~~~~e~~Ry~~l~   312 (471) |
|  | T PF04602.13 | 238 | -----KRAKQLTPNTTTGNKHTPLASGRPHRPLLAAGTAVLFIIFYDQTLAAVSEASRLRTIIGPSNSWYNEFFRYSELF   312 (471) |
|  | T ss\_pred |  | -----HHHHhcCCCCCCCCCCCCCCCCccHHHHHHHHHHHHHHHHhcCcHHHHHHHHHHHhhcCCCCHHhhhHHHHHHHh |
|  |
|  |
|  | Q ss\_pred |  | HHHhhccHhhhccccccccCcccccchHHHHHHHHHHHHHHHHHHHHHhcCCCcchHHHHHHHHHHHHHHhhHhcccccc |
|  | Q Q5T4D3 | 326 | AWLLLCPWWLCFDWSMGCIPLIKSISDWRVIALAALWFCLIGLICQALCSEDGHKRRILTLGLGFLVIPFLPASNLFFRV   405 (462) |
|  | Q Consensus | 326 | ~~~~~~p~~~~~~~~~~~~~~~~~~~~~~~~~~~~~~~~~~~~~~~~~~~~~~~~~~~~~~~~~~~~~~~~~~~~~~~~~   405 (462) |
|  |  |  | .. ....+...-......+..+..+.......++.++.........+.+....+....+. |
|  | T Consensus | 313 | ~~-----------------~~~g~~~rr~~vll~~~~l~~~~~~l~r~~r~~~~~~~p~~rl~~~~~~~~~~l~~t----   371 (471) |
|  | T PF04602.13 | 313 | SQ-----------------TADGSIARRFPVLIMIVCIFTAAAAIIHSASKSKLAKGPTLRLLAVSIMSFGFLAAT----   371 (471) |
|  | T ss\_pred |  | cC-----------------CCCCcHHHHHHHHHHHHHHHHHHHHHHHHhccccccCChHHHHHHHHHHHHHHHHhC---- |
|  |
|  |
|  | Q ss\_pred |  | chhHHHhhhhHHHHHHHHHHHHHHHHHHhchhhHHHHHHHHHHHHHHHHHHHHHhcC |
|  | Q Q5T4D3 | 406 | GFVVAERVLYLPSVGYCVLLTFGFGALSKHTKKKKLIAAVVLGILFINTLRCVLRSG   462 (462) |
|  | Q Consensus | 406 | ~~~~~~ry~~~~~~~~~ll~a~~~~~~~~~~~~~~~~~~~~~~~~~~~~~~~~~~~~   462 (462) |
|  |  |  | ..+..+|+-...+....+++.......+...+...........+.......+...| |
|  | T Consensus | 372 | -ptKwthhfg~~a~~~~~~~a~~~~~~~~~~~r~~~~~~~~~~~~~~~~al~~~g~N   427 (471) |
|  | T PF04602.13 | 372 | -PTKWVHHFGAFAGIGAAIAALAAVALTTPLFQSPRNRVLFTGIVVIIAAYAATGPN   427 (471) |
|  | T ss\_pred |  | -CchhhhhHHHHHHHHHHHHHHHHHHhcchhcCCHHHHHHHHHHHHHHHHHHHhCcc |
|  |
| --- | | | |
|  | Template alignmentCDD | | |
| 14. | PF06728.14 ; PIG-U ; GPI transamidase subunit PIG-U | | |
|  | Probability: 99.28%, E-value: 5e-9, Score: 92.14, Aligned cols: 306, Identities: 7%, Similarity: -0.126, | | |
|  |
|  | Q ss\_pred |  | CchHHHHhcccccCCCChhhhhcccccccccCCCCCccccCchHHHHHHHHHHHhCCCCchHHHHHHHHHHHHHHHHHHH |
|  | Q Q5T4D3 | 45 | DDSEAIVNNKDLQAETPLGDLWHHDFWGSRLSSNTSHKSYRPLTVLTFRINYYLSGGFHPVGFHVVNILLHSGISVLMVD   124 (462) |
|  | Q Consensus | 45 | Dd~~~~~~~~~~~~~~~~~~~~~~~~~~~~~~~~~~~~~~~Pl~~~~~~~~~~~~gg~~~~~~rl~~~~~~~l~~~l~~~   124 (462) |
|  |  |  | |...+........++.+..+ .....+||+..++..... + +.+....|+++.++.++++.++|. |
|  | T Consensus | 35 | d~~~~~~~~~~~~~G~~py~--------------~~~~~ypP~~~~l~~~~~--~-~~~~~~~r~~~~~~~~~~~~l~~~   97 (363) |
|  | T PF06728.14 | 35 | GFLRVREGLYLYENGLDPYS--------------GGVFYQSPLLLILNYCCE--L-LGGISVTRFVYTSISTMGGLFVYL   97 (363) |
|  | T ss\_pred |  | hHHHHHHHHHHHHcCCCCCC--------------CCcccCCcchHHHHhhhh--h-cCchHHHHHHHHHHHHHHHHHHHH |
|  |
|  |
|  | Q ss\_pred |  | HHHHHhccccccccccccchHH--------HHHHHHHHHHHHCHHHHHHHHhHhcHHHHHHHHHHHHHHHHHHHHHHHcC |
|  | Q Q5T4D3 | 125 | VFSVLFGGLQYTSKGRRLHLAP--------RASLLAALLFAVHPVHTECVAGVVGRADLLCALFFLLSFLGYCKAFRESN   196 (462) |
|  | Q Consensus | 125 | l~~~l~~~~~~~~~~~~~~~~~--------~~a~~aall~~~~p~~~~~~~~~~~~~~~~~~~f~ll~~~~~~~~~~~~~   196 (462) |
|  |  |  | ++++.. ++ ..+..++++++++|......... +.|.+..++.+++++++.| ++ |
|  | T Consensus | 98 | ~~~~~~--------------~~~~~~~~~~~~~~~~~~~~~~~p~~~~~~~~~--~~d~~~~~~~~~al~~~~~----~~   157 (363) |
|  | T PF06728.14 | 98 | IAKQAR--------------VLDPNQVLSTCSPLWISVIYLLNPLTFLPGIAC--SADMILNFTTLMTIYFASC----GS   157 (363) |
|  | T ss\_pred |  | HHHHhh--------------hcCcccccccCCcHHHHHHHHhCHHHHHHHHhc--chHHHHHHHHHHHHHHHhC----CC |
|  |
|  |
|  | Q ss\_pred |  | CCCCchHHHHHHHHHHHHHHHHhchHHHHHHHHHHHHHHHHHhCCCChHHHHHHHhhhccchHhhchhhhhhHHHHHHHH |
|  | Q Q5T4D3 | 197 | KEGAHSSTFWVLLSIFLGAVAMLCKEQGITVLGLNAVFDILVIGKFNVLEIVQKVLHKDKSLENLGMLRNGGLLFRMTLL   276 (462) |
|  | Q Consensus | 197 | ~~~~~~~~~~~~~~~~~~~la~l~k~~~~~~~~~~~~~~~~~~~~~~~~~~~~~~~~~~~~~~~~~~~~~~~~~~~~~~~   276 (462) |
|  |  |  | ...++++.+++..+|...+...+.........++.++ ..+.......... |
|  | T Consensus | 158 | ----------~~~ag~~~gla~~~K~~~~~~~~~~~~~~~~~~~~~~--------------------~~~~~~~~~~~~~   207 (363) |
|  | T PF06728.14 | 158 | ----------YAIYACCMALTVFINPNALLLFFPSYLILRKCNSSIK--------------------FRQIFVVFLFYLA   207 (363) |
|  | T ss\_pred |  | ----------HHHHHHHHHHHHhcChHHHHHHHHHHHHHHHcCchHH--------------------HHHHHHHHHHHHH |
|  |
|  |
|  | Q ss\_pred |  | HHHHHHHHHHH-HHHhCCCCCCccccCCcchhcchhhHhHhHHHHHHHHHHHHhhccHhhhccccccccCcccccchHHH |
|  | Q Q5T4D3 | 277 | TSGGAGMLYVR-WRIMGTGPPAFTEVDNPASFADSMLVRAVNYNYYYSLNAWLLLCPWWLCFDWSMGCIPLIKSISDWRV   355 (462) |
|  | Q Consensus | 277 | ~~~~~~~~~~~-~~~~~~~~~~~~~~~~~~~~~~~~~~~~~~~~~~~~~~~~~~~~p~~~~~~~~~~~~~~~~~~~~~~~   355 (462) |
|  |  |  | ......+.... ........................................ |
|  | T Consensus | 208 | ~~~~~~~~~~~~~~~~~~~~~~~~~~~~~~~~~~~~~~~~~~~~~~~~~~~~----------------------------   259 (363) |
|  | T PF06728.14 | 208 | GLIITSGFFLNSLSFLKIPFRVYLDSHDLTPNLGLWWYFFTEMFNEFRTFFL----------------------------   259 (363) |
|  | T ss\_pred |  | HHHHHHHHhhcCHHHHHHHHhhhccccccCCCHHHHHHHHHhHhHHHHHHHH---------------------------- |
|  |
|  |
|  | Q ss\_pred |  | HHHHHHHHHHHHHHHHHHhcCCCcchHHHHHHHHHHHHHHhhHhccccccchhHHHhhhhHHHHHHHHHHHHHHHHHHhc |
|  | Q Q5T4D3 | 356 | IALAALWFCLIGLICQALCSEDGHKRRILTLGLGFLVIPFLPASNLFFRVGFVVAERVLYLPSVGYCVLLTFGFGALSKH   435 (462) |
|  | Q Consensus | 356 | ~~~~~~~~~~~~~~~~~~~~~~~~~~~~~~~~~~~~~~~~~~~~~~~~~~~~~~~~ry~~~~~~~~~ll~a~~~~~~~~~   435 (462) |
|  |  |  | ................+++++................+... .+|.....|.+.+... .....+ |
|  | T Consensus | 260 | ----~~~~~~~~~~~~~~~~~~~~~~~~~~~~~~~~~~~~~~~~~----------~~y~~~~~p~~~~~~~---~~~~~~   322 (363) |
|  | T PF06728.14 | 260 | ----FVFAILPLMFVLPVSIRLYYLPLPITIALIGLHSLFKAYPS----------ICDLSIFLSLLPIFNK---VQDRMR   322 (363) |
|  | T ss\_pred |  | ----HHHHHHHHHHHHHHHHHhccChHHHHHHHHHHHHHhCCCCc----------HHHHHHHHHHHHhcHH---HHHHhh |
|  |
|  |
|  | Q ss\_pred |  | hhhHHHHHHHHHHHHHHHHHHHHHhcC |
|  | Q Q5T4D3 | 436 | TKKKKLIAAVVLGILFINTLRCVLRSG   462 (462) |
|  | Q Consensus | 436 | ~~~~~~~~~~~~~~~~~~~~~~~~~~~   462 (462) |
|  |  |  | ........................... |
|  | T Consensus | 323 | ~~~~~~~~~~~~~~~~~~~~~~~~~~~   349 (363) |
|  | T PF06728.14 | 323 | YSLLTNNAIVFALVLGSAFYHSWITLG   349 (363) |
|  | T ss\_pred |  | hHHHHHHHHHHHHHHHHHHHHHHHhcC |
|  |
| --- | | | |
|  | Template alignmentCDD | | |
| 15. | PF15971.6 ; Mannosyl\_trans4 ; DolP-mannose mannosyltransferase | | |
|  | Probability: 99.22%, E-value: 3.9e-10, Score: 86.72, Aligned cols: 151, Identities: 19%, Similarity: 0.087, | | |
|  |
|  | Q ss\_pred |  | CCCccccCchHHHHHHHHHHHhCCCCchHHHHHHH----HHHHHHHHHHHHHHHHHhccccccccccccchHH-HHHHHH |
|  | Q Q5T4D3 | 78 | NTSHKSYRPLTVLTFRINYYLSGGFHPVGFHVVNI----LLHSGISVLMVDVFSVLFGGLQYTSKGRRLHLAP-RASLLA   152 (462) |
|  | Q Consensus | 78 | ~~~~~~~~Pl~~~~~~~~~~~~gg~~~~~~rl~~~----~~~~l~~~l~~~l~~~l~~~~~~~~~~~~~~~~~-~~a~~a   152 (462) |
|  |  |  | .+..+++||++.++......++ |+++...|+.+. +++.+++..+|.+.|+.. ++ +.+..+ |
|  | T Consensus | 6 | ~~~~~~~ppl~~~~~~~~~~l~-g~~~~~~~~~~~~~~~~~~~~~~~~~~~~~~~~~--------------~~~~~~~~~   70 (163) |
|  | T PF15971.6 | 6 | VDAWEPKLPLSYETTGVLALLS-GGDMYRLHLLSVVLMSGAVCAIVALVVMLVYDIT--------------GDDIVAPLA   70 (163) |
|  | T ss\_pred |  | ccccCCCCcHHHHHHHHHHHHh-CCchHHHHHHHHHHHHHHHHHHHHHHHHHHHHHh--------------CCCCHHHHH |
|  |
|  |
|  | Q ss\_pred |  | HHHHHHCHHHH-HHHHhHhcHHHHHHHHHHHHHHHHHHHHHHHcCCCCCchHHHHHHHHHHHHHHHHhchHHHHHHHHHH |
|  | Q Q5T4D3 | 153 | ALLFAVHPVHT-ECVAGVVGRADLLCALFFLLSFLGYCKAFRESNKEGAHSSTFWVLLSIFLGAVAMLCKEQGITVLGLN   231 (462) |
|  | Q Consensus | 153 | all~~~~p~~~-~~~~~~~~~~~~~~~~f~ll~~~~~~~~~~~~~~~~~~~~~~~~~~~~~~~~la~l~k~~~~~~~~~~   231 (462) |
|  |  |  | +++++++|... ...... .+|....++.+++++...+ ++ ...++++.+++.++|+.+....+.. |
|  | T Consensus | 71 | ~~~~~~~p~~~~~~~~~~--~~~~~~~~~~~~~~~~~~~----~~----------~~~~~~~~~l~~~~k~~~~~~~~~~   134 (163) |
|  | T PF15971.6 | 71 | GLSMFLLPGFAVRPAYGF--KAKYLLVLCGLLAIYLYTR----GY----------PALSGVAAAASVGYWQAGAIFPLIV   134 (163) |
|  | T ss\_pred |  | HHHHHHchHHhccccccc--cchHHHHHHHHHHHHHHHc----Cc----------HHHHHHHHHHHHHhhHHHhHHHHHH |
|  |
|  |
|  | Q ss\_pred |  | HHHHHHHhCCCChHHHHHHHhhhccchHhhchhhhhhHHHHHHHHHHHHHHHHH |
|  | Q Q5T4D3 | 232 | AVFDILVIGKFNVLEIVQKVLHKDKSLENLGMLRNGGLLFRMTLLTSGGAGMLY   285 (462) |
|  | Q Consensus | 232 | ~~~~~~~~~~~~~~~~~~~~~~~~~~~~~~~~~~~~~~~~~~~~~~~~~~~~~~   285 (462) |
|  |  |  | .......+++++ ................ |
|  | T Consensus | 135 | ~~~~~~~~~~~~--------------------------~~~~~~~~~~~~~~~~   162 (163) |
|  | T PF15971.6 | 135 | VGLAVQRRDMRA--------------------------LERVVAGGLGFTIVML   162 (163) |
|  | T ss\_pred |  | HHHHHcccchHH--------------------------HHHHHHHHHHHHHHHh |
|  |
| --- | | | |
|  | Template alignmentCDD | | |
| 16. | PF09586.11 ; YfhO ; Bacterial membrane protein YfhO | | |
|  | Probability: 99.18%, E-value: 2e-8, Score: 98.69, Aligned cols: 371, Identities: 11%, Similarity: -0.045, | | |
|  |
|  | Q ss\_pred |  | HHHHHHHHHHHHHHHhhhcCCCcc--cCchHHHHhcccccCCCChhhhh-cccccccccCCCCCccccCchHHHHHHHHH |
|  | Q Q5T4D3 | 20 | FWAKLVVGSVAIVCFARSYDGDFV--FDDSEAIVNNKDLQAETPLGDLW-HHDFWGSRLSSNTSHKSYRPLTVLTFRINY   96 (462) |
|  | Q Consensus | 20 | ~~~~~~l~~~~~~~~~~~~~~~~~--~Dd~~~~~~~~~~~~~~~~~~~~-~~~~~~~~~~~~~~~~~~~Pl~~~~~~~~~   96 (462) |
|  |  |  | .+.+++++++.........+.... .|+..++........ .... ........+.........++..+....+.. |
|  | T Consensus | 3 | ~~~~~l~~~~~~~~~~~~~~~~~~~~~D~~~~~~p~~~~~~----~~~~~~~~~~~~~~~~~~G~~~~~~~~~~~~~p~~   78 (832) |
|  | T PF09586.11 | 3 | LLPFAIIFIYGLSRHVFPFGGQTIMTVDLGQQYIDFFAYFR----TTLLQHPDTFFYSFAKGLGGDMLGVWAYYLMSPFN   78 (832) |
|  | T ss\_pred |  | HHHHHHHHHHHHHcCCCCCCCCcceeechhHhHHHHHHHHH----HHHhhCCccceeccccCCCCccHHHHHHHHhCcch |
|  |
|  |
|  | Q ss\_pred |  | HHhCC-----CCchHHHHHHHHHHHHHHHHHHHHHHHHh-ccccccccccccchHHHHHHHHHHHHHHCHHHHHHHHhHh |
|  | Q Q5T4D3 | 97 | YLSGG-----FHPVGFHVVNILLHSGISVLMVDVFSVLF-GGLQYTSKGRRLHLAPRASLLAALLFAVHPVHTECVAGVV   170 (462) |
|  | Q Consensus | 97 | ~~~gg-----~~~~~~rl~~~~~~~l~~~l~~~l~~~l~-~~~~~~~~~~~~~~~~~~a~~aall~~~~p~~~~~~~~~~   170 (462) |
|  |  |  | .+. . ..+.+.++..++...++.+.+|.++|++. . ++..|+++++++++++......... |
|  | T Consensus | 79 | ~l~-~~~~~~~~~~~~~~~~~l~~~l~~~~~y~l~r~~~~~-------------~~~~a~~~a~~y~~s~~~~~~~~~~-   143 (832) |
|  | T PF09586.11 | 79 | LLV-LLTPGKWLSFGVWLMVLLKYGFSGLSFAYYLKKSRLL-------------SGWWLPTLSLTYALSGFAIANQFNV-   143 (832) |
|  | T ss\_pred |  | hHH-hhCCHHHHHHHHHHHHHHHHHHHHHHHHHHHHHhccc-------------cccHHHHHHHHHHHHHHHHHHHhCh- |
|  |
|  |
|  | Q ss\_pred |  | cHHHHHHHHHHHHHHHHHHHHHHHcCCCCCchHHHHHHHHHHHHHHHHhchHHHHHHHHHHHHHHHHHhCCCChHHHHHH |
|  | Q Q5T4D3 | 171 | GRADLLCALFFLLSFLGYCKAFRESNKEGAHSSTFWVLLSIFLGAVAMLCKEQGITVLGLNAVFDILVIGKFNVLEIVQK   250 (462) |
|  | Q Consensus | 171 | ~~~~~~~~~f~ll~~~~~~~~~~~~~~~~~~~~~~~~~~~~~~~~la~l~k~~~~~~~~~~~~~~~~~~~~~~~~~~~~~   250 (462) |
|  |  |  | .......++-+.++++.+..++++ ++...++..+..++-..-.....+...+..........++.++..++ |
|  | T Consensus | 144 | --~~~~~~~~lPl~l~~~~~~~~~~~-------~~~~~~~~~l~~~~~~~~~~~~~~~~~~~~l~~~~~~~~~~~~~~~~   214 (832) |
|  | T PF09586.11 | 144 | --MWLDAMIWLPLVVLGIEQLFERQR-------FWLYPLSLAALLIINYYMGYMVCLFVVAYFFWASVHHFKTWRQTCLV   214 (832) |
|  | T ss\_pred |  | --hHHHHHHHHHHHHHHHHHHHhcCC-------ccHHHHHHHHHHHHHHHHHHHHHHHHHHHHHHHHHhcchhHHHHHHH |
|  |
|  |
|  | Q ss\_pred |  | HhhhccchHhhchhhhhhHHHHHHHHHHHHHHHHHHHHHHhCCCCCCccccCCcchhcchhhHhHhHHHHHHHHHHHHhh |
|  | Q Q5T4D3 | 251 | VLHKDKSLENLGMLRNGGLLFRMTLLTSGGAGMLYVRWRIMGTGPPAFTEVDNPASFADSMLVRAVNYNYYYSLNAWLLL   330 (462) |
|  | Q Consensus | 251 | ~~~~~~~~~~~~~~~~~~~~~~~~~~~~~~~~~~~~~~~~~~~~~~~~~~~~~~~~~~~~~~~~~~~~~~~~~~~~~~~~   330 (462) |
|  |  |  | ...- ....++.+++.+..+.-..........................+........ |
|  | T Consensus | 215 | ~~~~---------------~~~~~l~~~l~a~~llp~~~~~~~~~~~~~~~~~~~~~~~~~~~~~~~~~~~---------   270 (832) |
|  | T PF09586.11 | 215 | YLKF---------------AGGSILAGLLAAWLLLPTFFQLTQSKGQYTIQKIHWKIDYNPLKILSKLVVG---------   270 (832) |
|  | T ss\_pred |  | HHHH---------------HHHHHHHHHHHHHHHHHHHHHHhcCcCccCccccccccCCCHHHHHHhhccC--------- |
|  |
|  |
|  | Q ss\_pred |  | ccHhhhccccccccCcccccchHHHHHHHHHHHHHHHHHHHHHhcCCCcchHHHHHHHHHHHHHHhhHhccccccchhHH |
|  | Q Q5T4D3 | 331 | CPWWLCFDWSMGCIPLIKSISDWRVIALAALWFCLIGLICQALCSEDGHKRRILTLGLGFLVIPFLPASNLFFRVGFVVA   410 (462) |
|  | Q Consensus | 331 | ~p~~~~~~~~~~~~~~~~~~~~~~~~~~~~~~~~~~~~~~~~~~~~~~~~~~~~~~~~~~~~~~~~~~~~~~~~~~~~~~   410 (462) |
|  |  |  | ..................+..+++......++++++.+......++.+++................. |
|  | T Consensus | 271 | -------------~~~~~~~~~~~~~~y~g~~~l~l~~~~~~~~~~~~~~~~~~~~~~~~l~~~~~~~~~~~~~~~~~~~   337 (832) |
|  | T PF09586.11 | 271 | -------------NFNFDQMPKGEPNIFVGSLILIGFITYFLTRKIPIKERLAALLVTGFLGLSLCFEPLDLLWHGMQFP   337 (832) |
|  | T ss\_pred |  | -------------CCCcccCCCCchHHHHHHHHHHHHHHHHHcCCCCHHHHHHHHHHHHHHHHHhcCHHHHHHHhcCCCC |
|  |
|  |
|  | Q ss\_pred |  | H----hhhhHHHHHHHHHHHHHHHHHHhchhhHHHHHHHHHHHHHHHHH |
|  | Q Q5T4D3 | 411 | E----RVLYLPSVGYCVLLTFGFGALSKHTKKKKLIAAVVLGILFINTL   455 (462) |
|  | Q Consensus | 411 | ~----ry~~~~~~~~~ll~a~~~~~~~~~~~~~~~~~~~~~~~~~~~~~   455 (462) |
|  |  |  | . |+.....+.++++++.++.+..+.++........+++++..... |
|  | T Consensus | 338 | ~~~~~R~~~~~~~~~~ila~~~l~~~~~~~~~~~~~~~~~~~~~~~~~~   386 (832) |
|  | T PF09586.11 | 338 | VWYPYRFSYVISFWLIVLAVQRLHYQPQFKWYSLLAPLLLLAASLAYTF   386 (832) |
|  | T ss\_pred |  | CCCcHHHHHHHHHHHHHHHHHHHhcCCcCChhHHHHHHHHHHHHHHHHH |
|  |
| --- | | | |
|  | Template alignmentCDD | | |
| 17. | PF14264.7 ; Glucos\_trans\_II ; Glucosyl transferase GtrII | | |
|  | Probability: 99%, E-value: 5.6e-7, Score: 76.74, Aligned cols: 300, Identities: 14%, Similarity: 0.073, | | |
|  |
|  | Q ss\_pred |  | cCCCcccCchHHHHhcccccCCCChhhhhcccccccccCCCCCccccCchHHHHHHHHHHHhCCCCchHHHHHHHHHHHH |
|  | Q Q5T4D3 | 38 | YDGDFVFDDSEAIVNNKDLQAETPLGDLWHHDFWGSRLSSNTSHKSYRPLTVLTFRINYYLSGGFHPVGFHVVNILLHSG   117 (462) |
|  | Q Consensus | 38 | ~~~~~~~Dd~~~~~~~~~~~~~~~~~~~~~~~~~~~~~~~~~~~~~~~Pl~~~~~~~~~~~~gg~~~~~~rl~~~~~~~l   117 (462) |
|  |  |  | .+..+..||+..........+. +..++ ||+...+... .... +. ++..++.++++..+ |
|  | T Consensus | 5 | ~~~~~~~Dd~~~~~~~~~~~~~-----~~~~G---------------R~~~~~l~~~-~~~~-~~-p~~~~~l~~~~~~~   61 (312) |
|  | T PF14264.7 | 5 | FHSSFSHDSLNALYSDMTEIKW-----KLALG---------------RFVVPLIMKI-RGQI-AL-PWLIGIVSLFLIAA   61 (312) |
|  | T ss\_pred |  | cccCCCccchhHhhcCchhhhH-----HHhcc---------------chhHHHHHHH-cccc-ch-hHHHHHHHHHHHHH |
|  |
|  |
|  | Q ss\_pred |  | HHHHHHHHHHHHhccccccccccccchHHHHHHHHHHHHHHCHHHHH-HHHhH-hcHHHHHHHHHHHHHHHHHHHHHHHc |
|  | Q Q5T4D3 | 118 | ISVLMVDVFSVLFGGLQYTSKGRRLHLAPRASLLAALLFAVHPVHTE-CVAGV-VGRADLLCALFFLLSFLGYCKAFRES   195 (462) |
|  | Q Consensus | 118 | ~~~l~~~l~~~l~~~~~~~~~~~~~~~~~~~a~~aall~~~~p~~~~-~~~~~-~~~~~~~~~~f~ll~~~~~~~~~~~~   195 (462) |
|  |  |  | ++.+.+...++. ++..+.+++.++.++|.+.+ ...+. .+.+.....++..++.++.. +++ |
|  | T Consensus | 62 | s~~l~~~~~~~~---------------~~~~~~~~~~l~~~~P~~~~~~~~f~~~~~~~~~~~ll~~la~~~~~---~~~   123 (312) |
|  | T PF14264.7 | 62 | SLYLILETIQID---------------SKAMIILVSIFMVTNRTIYSMTATYIYELDYDMLALFFASLAAYILM---KKD   123 (312) |
|  | T ss\_pred |  | HHHHHHHHhcCC---------------cHHHHHHHHHHHHHhHHHHHHHHHHHHhcHHHHHHHHHHHHHHHHHH---hCC |
|  |
|  |
|  | Q ss\_pred |  | CCCCCchHHHHHHHHHHHHHHHHhchHHHHHHHHHHHHHHHH---HhCCCChHHHHHHHhhhccchHhhchhhhhhHHHH |
|  | Q Q5T4D3 | 196 | NKEGAHSSTFWVLLSIFLGAVAMLCKEQGITVLGLNAVFDIL---VIGKFNVLEIVQKVLHKDKSLENLGMLRNGGLLFR   272 (462) |
|  | Q Consensus | 196 | ~~~~~~~~~~~~~~~~~~~~la~l~k~~~~~~~~~~~~~~~~---~~~~~~~~~~~~~~~~~~~~~~~~~~~~~~~~~~~   272 (462) |
|  |  |  | + ++....+.++..+++.+++..+...+...+.... .+.+++.|+..++. .... |
|  | T Consensus | 124 | ~-------~~~~~~~~l~~~~sl~~YQ~~~~~~~~~~~~~~l~~~~~~~~~~k~~~~~~-----------------~~~~   179 (312) |
|  | T PF14264.7 | 124 | K-------PGWYLLAFLSGVLSLGLYQSYIEVAFAIVIIASLKNLLEGSKYSQVLKRGI-----------------IAIV   179 (312) |
|  | T ss\_pred |  | C-------CchHHHHHHHHHHHHHHHHHHHHHHHHHHHHHHHHHHHcCCCcHHHHHHHH-----------------HHHH |
|  |
|  |
|  | Q ss\_pred |  | HHHHHHHHHHHHHHHHHHhCCCCCCccccCCcchhcchhhHhHhHHHHHHHHHHHHhhccHhhhccccccccCcccccch |
|  | Q Q5T4D3 | 273 | MTLLTSGGAGMLYVRWRIMGTGPPAFTEVDNPASFADSMLVRAVNYNYYYSLNAWLLLCPWWLCFDWSMGCIPLIKSISD   352 (462) |
|  | Q Consensus | 273 | ~~~~~~~~~~~~~~~~~~~~~~~~~~~~~~~~~~~~~~~~~~~~~~~~~~~~~~~~~~~p~~~~~~~~~~~~~~~~~~~~   352 (462) |
|  |  |  | . .....+.-...........+.+.....+.......+..++..+..+....+...... ........ |
|  | T Consensus | 180 | ~-~~~~~i~y~i~~k~~~~~~~~~~~~~~~~~~~~~~~~~~~i~~~~~~~~~~~~~~~~-------------~~~~~~~~   245 (312) |
|  | T PF14264.7 | 180 | S-FVLSVVAYYLIYKLSCKFFNVQIEGRTDAFSGEYTSIIVSLKVMLYKLIHDVVKPGT-------------IYELPIVG   245 (312) |
|  | T ss\_pred |  | H-HHHHHHHHHHHHHHHHHHcCCCCCCCCCccccccccHHHHHHHHHHHHHHHhhCCCC-------------CCCCccHH |
|  |
|  |
|  | Q ss\_pred |  | HHHHHHHHHHHHHHHHHHHHHhcCCCcchHHHHHHHHHHHHHHhhHhccccc-cchhHHHhhhhHHHHHH |
|  | Q Q5T4D3 | 353 | WRVIALAALWFCLIGLICQALCSEDGHKRRILTLGLGFLVIPFLPASNLFFR-VGFVVAERVLYLPSVGY   421 (462) |
|  | Q Consensus | 353 | ~~~~~~~~~~~~~~~~~~~~~~~~~~~~~~~~~~~~~~~~~~~~~~~~~~~~-~~~~~~~ry~~~~~~~~   421 (462) |
|  |  |  | ..........+.+........+++++.+. .++.......|+...... .+....+|-.++....+ |
|  | T Consensus | 246 | ~~~~~~~~~~~~~~~~~~~~~~~~~~~~~-----~l~~~~~~~~p~~~~~i~~~~~~~~~r~l~~~~~~~   310 (312) |
|  | T PF14264.7 | 246 | IADILLIAIGVALCLIMIFKLGKGKTGEK-----VVSLLLLAALPLSLNLICLTIKSGSEHDLMTYSFNF   310 (312) |
|  | T ss\_pred |  | HHHHHHHHHHHHHHHHHHHHHcCCCcHHH-----HHHHHHHHHHHHHHHHHHHHCCCCCcchhhhhhhhe |
|  |
| --- | | | |
|  | Template alignmentCDD | | |
| 18. | PF03155.16 ; Alg6\_Alg8 ; ALG6, ALG8 glycosyltransferase family | | |
|  | Probability: 98.98%, E-value: 3.8e-7, Score: 82.73, Aligned cols: 319, Identities: 9%, Similarity: -0.014, | | |
|  |
|  | Q ss\_pred |  | cccCchHHHHhcccccCCCChhhhhcccccccccCCCCCccccCchHHHHHHHHHHHhC-----------------CCCc |
|  | Q Q5T4D3 | 42 | FVFDDSEAIVNNKDLQAETPLGDLWHHDFWGSRLSSNTSHKSYRPLTVLTFRINYYLSG-----------------GFHP   104 (462) |
|  | Q Consensus | 42 | ~~~Dd~~~~~~~~~~~~~~~~~~~~~~~~~~~~~~~~~~~~~~~Pl~~~~~~~~~~~~g-----------------g~~~   104 (462) |
|  |  |  | +..+|...+.+..+...+....+++.++ .+.+..+|||+..+...+...+ . +... |
|  | T Consensus | 12 | ~~s~D~~~~r~w~~~t~~~p~~~wy~~~-------~~~w~ldYPPl~a~~~~~~~~~-~~~~~~~~~~l~~~~~~~~~~~   83 (470) |
|  | T PF03155.16 | 12 | YHSTDFEVHRNWLAITHSLPLNQWYVDA-------TSEWTLDYPPFFAYFEWLLSQV-AKYVDPRMLVVDNLNYESKATV   83 (470) |
|  | T ss\_pred |  | cCCCcHHHHHHHHHHHhhCCHHHhccCC-------CccCCCCCcHHHHHHHHHHHHH-HHHhCHHHhhcccCCCCCHHHH |
|  |
|  |
|  | Q ss\_pred |  | hHHHHHHHHHHHHHHHHHHHHHH---HHhccccccccccccchHHHHHHHHHHHHHHCHHHHHHHH-hHhcHHHHHHHHH |
|  | Q Q5T4D3 | 105 | VGFHVVNILLHSGISVLMVDVFS---VLFGGLQYTSKGRRLHLAPRASLLAALLFAVHPVHTECVA-GVVGRADLLCALF   180 (462) |
|  | Q Consensus | 105 | ~~~rl~~~~~~~l~~~l~~~l~~---~l~~~~~~~~~~~~~~~~~~~a~~aall~~~~p~~~~~~~-~~~~~~~~~~~~f   180 (462) |
|  |  |  | ...|+..++..++....++.+.+ +.. +++.+..++++++++|....... .. +.|.....+ |
|  | T Consensus | 84 | ~~~R~~vi~~d~l~~~~v~~~~~~~~~~~--------------~~~~~~~~~~l~l~~P~li~~d~~~~--q~n~~~~~l   147 (470) |
|  | T PF03155.16 | 84 | YFQRLSVIATDLVYVLGVRSCLGSLGLAR--------------DTQQFFAGSMLLLLNVGLLFVDHIHF--QYNGLLFGI   147 (470) |
|  | T ss\_pred |  | HHHHHHHHHHHHHHHHHHHHHHHhcCCCC--------------ChhHHHHHHHHHHHcHHHHHhhcccc--cchHHHHHH |
|  |
|  |
|  | Q ss\_pred |  | HHHHHHHHHHHHHHcCCCCCchHHHHHHHHHHHHHHHHhchHHHHHHHHHHHHHHHHHhCCCChH--HHHHHHhhhccch |
|  | Q Q5T4D3 | 181 | FLLSFLGYCKAFRESNKEGAHSSTFWVLLSIFLGAVAMLCKEQGITVLGLNAVFDILVIGKFNVL--EIVQKVLHKDKSL   258 (462) |
|  | Q Consensus | 181 | ~ll~~~~~~~~~~~~~~~~~~~~~~~~~~~~~~~~la~l~k~~~~~~~~~~~~~~~~~~~~~~~~--~~~~~~~~~~~~~   258 (462) |
|  |  |  | .+++++++.+ ++ ...+++++++++.+|...+...++..++.+...+.++.+ +..++ |
|  | T Consensus | 148 | ~llsl~~~~~----~~----------~~~a~~~~~lal~~K~~~l~~~p~~~~~ll~~~~~~~~~~~~~~~~--------   205 (470) |
|  | T PF03155.16 | 148 | LLLSIGSLIR----QR----------FLWSAFAFAVLLNFKHIFLYMAPAFGVYLLRFYCLEQASVASAVGA--------   205 (470) |
|  | T ss\_pred |  | HHHHHHHHHc----Cc----------hHHHHHHHHHHHHcchHHHHHHHHHHHHHHHHhcccCCCHhHHHHH-------- |
|  |
|  |
|  | Q ss\_pred |  | HhhchhhhhhHHHHHHHHHHHHHHHHHHHHHHhCCCCCCcc-----ccCCcchhcchhhHhHhHHHHHHHHHHHHhhccH |
|  | Q Q5T4D3 | 259 | ENLGMLRNGGLLFRMTLLTSGGAGMLYVRWRIMGTGPPAFT-----EVDNPASFADSMLVRAVNYNYYYSLNAWLLLCPW   333 (462) |
|  | Q Consensus | 259 | ~~~~~~~~~~~~~~~~~~~~~~~~~~~~~~~~~~~~~~~~~-----~~~~~~~~~~~~~~~~~~~~~~~~~~~~~~~~p~   333 (462) |
|  |  |  | ........+........++ .++...... ......+......-...+..............+. |
|  | T Consensus | 206 | -----------~~~~~~~~~~~~~~~~~Pf--~~~~~~~~~rlfp~~rgl~~~~~a~n~w~~~~~~~~~~~~~~~~~~~~   272 (470) |
|  | T PF03155.16 | 206 | -----------VIKLLVVGLTPFAVSFGPF--WKQLPQVLSRLFPFKRGLTHAYWAPNFWALYNTADKVAAGVLKVHDGG   272 (470) |
|  | T ss\_pred |  | -----------HHHHHHHHHHHHHHhcccc--cccHHHHHHHHCCccccccccchhhHHHHHHHHHHHHHHHHHhcCCCC |
|  |
|  |
|  | Q ss\_pred |  | hhhccccccccCcccccchHHHHHHHHHHHHHHHHHHHHHhcCCCcchHHHHHHHHHHHHHHhhHhccccccchhHHHhh |
|  | Q Q5T4D3 | 334 | WLCFDWSMGCIPLIKSISDWRVIALAALWFCLIGLICQALCSEDGHKRRILTLGLGFLVIPFLPASNLFFRVGFVVAERV   413 (462) |
|  | Q Consensus | 334 | ~~~~~~~~~~~~~~~~~~~~~~~~~~~~~~~~~~~~~~~~~~~~~~~~~~~~~~~~~~~~~~~~~~~~~~~~~~~~~~ry   413 (462) |
|  |  |  | ........+...................++..+.......+++++...+.....+.........+ +...+++| |
|  | T Consensus | 273 | ~~~t~g~~~~~~~~~~~~~~~~~~~~~~l~~~~~~~~~l~~~~~~~~~~~~~~~~~~~~l~~flf-------~~~vhek~   345 (470) |
|  | T PF03155.16 | 273 | ASTTSGLVQEVRHSVLPAITPPVTFALTALFMLPILVKLFRSPKKQSPLVFLRAVVLCGCSSFVF-------GWHVHEKA   345 (470) |
|  | T ss\_pred |  | ccccCCccccchhhcCCccHHHHHHHHHHHHHHHHHHHHHcCCCCCCHHHHHHHHHHHHHHHHHh-------chhhcchH |
|  |
|  |
|  | Q ss\_pred |  | hhHHHHHHHHHHH |
|  | Q Q5T4D3 | 414 | LYLPSVGYCVLLT   426 (462) |
|  | Q Consensus | 414 | ~~~~~~~~~ll~a   426 (462) |
|  |  |  | .+.+.+++++++. |
|  | T Consensus | 346 | ill~l~Pl~ll~~   358 (470) |
|  | T PF03155.16 | 346 | ILMVLLPLCLLTL   358 (470) |
|  | T ss\_pred |  | HHHHHHHHHHHHH |
|  |
| --- | | | |
|  | Template alignmentCDD | | |
| 19. | PF04922.13 ; DIE2\_ALG10 ; DIE2/ALG10 family | | |
|  | Probability: 98.97%, E-value: 2.5e-8, Score: 87.54, Aligned cols: 228, Identities: 12%, Similarity: -0.021, | | |
|  |
|  | Q ss\_pred |  | CcccCchHHHHhcccccCCCChhhhhcccccccccCCCCCccccCchHHHHHHHHHHHhCC---------CCchHHHHHH |
|  | Q Q5T4D3 | 41 | DFVFDDSEAIVNNKDLQAETPLGDLWHHDFWGSRLSSNTSHKSYRPLTVLTFRINYYLSGG---------FHPVGFHVVN   111 (462) |
|  | Q Consensus | 41 | ~~~~Dd~~~~~~~~~~~~~~~~~~~~~~~~~~~~~~~~~~~~~~~Pl~~~~~~~~~~~~gg---------~~~~~~rl~~   111 (462) |
|  |  |  | ..+.||..|..+++.+.+ +++..+ ++....+|..+++.+...+++ | .+....|..| |
|  | T Consensus | 3 | ~py~DE~fH~~qa~~y~~-G~~~~w-------------dp~iTTpPGlyl~~a~~~~l~-g~~~~~~~~~~s~~~LR~~n   67 (434) |
|  | T PF04922.13 | 3 | TPYIDEIFHIPQTQQYCK-GHWNAW-------------DSKITTPPGLYIIGYAWARML-TLTGLSESEACSTLSLRAVN   67 (434) |
|  | T ss\_pred |  | CCCcchHhhHHHHHHHHc-CCcccc-------------CccCCCChhHHHHHHHHHHHH-HHhcCCcccCCCHHHHHHHH |
|  |
|  |
|  | Q ss\_pred |  | HHHHH-HHHHHHHHHHHHHhccccccccccccchHHHHHHHHHHHHHHCHHHHHHHHhHhcHHHHHHHHHHHHHHHHHHH |
|  | Q Q5T4D3 | 112 | ILLHS-GISVLMVDVFSVLFGGLQYTSKGRRLHLAPRASLLAALLFAVHPVHTECVAGVVGRADLLCALFFLLSFLGYCK   190 (462) |
|  | Q Consensus | 112 | ~~~~~-l~~~l~~~l~~~l~~~~~~~~~~~~~~~~~~~a~~aall~~~~p~~~~~~~~~~~~~~~~~~~f~ll~~~~~~~   190 (462) |
|  |  |  | ++++. +...++|.+.++. ++..+...++.++++|..+..+... .+|...++++++++++..+ |
|  | T Consensus | 68 | ll~~~~~~~~~~~~l~~~~---------------~~~~a~l~al~l~~~Pl~~~~sfl~--YTDv~Sl~~vll~l~~~l~   130 (434) |
|  | T PF04922.13 | 68 | LMAVVIYIPATLYIIQRRV---------------WGSQAHFSAFSLVSFPLIWFYAALY--YTDVWSTATVLMALAFALS   130 (434) |
|  | T ss\_pred |  | HHHHHHHHHHHHHHHHHHh---------------cCchHHHHHHHHHhcHHHHHHHHhh--chHHHHHHHHHHHHHHHhC |
|  |
|  |
|  | Q ss\_pred |  | HHHHcCCCCCchHHHHHHHHHHHHHHHHhchHHHHHHHHHHHHHHHHHhCCCChHHHHHHHhhhccchHhhchhhhhhHH |
|  | Q Q5T4D3 | 191 | AFRESNKEGAHSSTFWVLLSIFLGAVAMLCKEQGITVLGLNAVFDILVIGKFNVLEIVQKVLHKDKSLENLGMLRNGGLL   270 (462) |
|  | Q Consensus | 191 | ~~~~~~~~~~~~~~~~~~~~~~~~~la~l~k~~~~~~~~~~~~~~~~~~~~~~~~~~~~~~~~~~~~~~~~~~~~~~~~~   270 (462) |
|  |  |  | ..+++. ...+++++.++|+++|++.++.........+....+...++..+...+.-............... |
|  | T Consensus | 131 | ~~~~~~---------~~~la~l~~~lavl~RQtnIvW~~f~~~~~~~~~~~~~~~~~~~~~~~~l~~~~~~~~~~~~~~~   201 (434) |
|  | T PF04922.13 | 131 | PRVPFY---------MVQLSALMCAVSLFFRQTNILWAAVVAVIAIENSHYSNGAPPKNGALAQIFSTISYTFQIELPIF   201 (434) |
|  | T ss\_pred |  | CCCCcc---------HHHHHHHHHHHHHHhchhHHHHHHHHHHHHHHHHhhhCCCCCcccHHHHHHHHHHHHHhhchHHH |
|  |
|  |
|  | Q ss\_pred |  | HHHHHHHHHHHHHHHHHHHHhCCCCCCccccCCcchhcc |
|  | Q Q5T4D3 | 271 | FRMTLLTSGGAGMLYVRWRIMGTGPPAFTEVDNPASFAD   309 (462) |
|  | Q Consensus | 271 | ~~~~~~~~~~~~~~~~~~~~~~~~~~~~~~~~~~~~~~~   309 (462) |
|  |  |  | ......+++.+.+..+...-.+-..++...........+ |
|  | T Consensus | 202 | ~~~~~~~~v~~~F~~Fv~~NGgIvlGDk~~H~~~~H~~q   240 (434) |
|  | T PF04922.13 | 202 | NILISYASVAVGFSFFLYINGGIALGDKDNHVAGNHIPQ   240 (434) |
|  | T ss\_pred |  | HHHHHHHHHHHHHHHHHHhCCCcccCccccccCcccHHH |
|  |
| --- | | | |
|  | Template alignmentCDD | | |
| 20. | PF05208.14 ; ALG3 ; ALG3 protein | | |
|  | Probability: 98.7%, E-value: 0.0000012, Score: 75.65, Aligned cols: 198, Identities: 13%, Similarity: 0.03, | | |
|  |
|  | Q ss\_pred |  | HHHHHHHHHHHhhhcCCCcc-cCchHHHHhcccccCCCChhhhhcccccccccCCCCCccccCchHHHHHHHHHHHhCCC |
|  | Q Q5T4D3 | 24 | LVVGSVAIVCFARSYDGDFV-FDDSEAIVNNKDLQAETPLGDLWHHDFWGSRLSSNTSHKSYRPLTVLTFRINYYLSGGF   102 (462) |
|  | Q Consensus | 24 | ~~l~~~~~~~~~~~~~~~~~-~Dd~~~~~~~~~~~~~~~~~~~~~~~~~~~~~~~~~~~~~~~Pl~~~~~~~~~~~~gg~   102 (462) |
|  |  |  | .++++-+..........++. .|+..|...+..+.+++..-+....+ .....+||++.+++.+.+.+. +. |
|  | T Consensus | 2 | ~~~~~~~~~~~~~i~~~~yt~iD~~~y~~~~~~i~~G~~pY~~~~~~---------~~p~~Ypp~~~yi~~~l~~l~-~~   71 (356) |
|  | T PF05208.14 | 2 | LLVLVDAVLSALIIKKVSYTEIDWTTYMQQIALYQAGERDYTAIKGD---------TGPLVYPASHVYIYSFLYELT-NK   71 (356) |
|  | T ss\_pred |  | HHHHHHHHHHHHHHHhCCCCCCcHHHHHHHHHHHHcCCCChhhccCC---------CCCCCCcHHHHHHHHHHHHHh-cC |
|  |
|  |
|  | Q ss\_pred |  | --CchHHHHHHHHHHHHHHHHHHHHHHHHhccccccccccccchHHHHHHHHHHHHHHCHHHHHHHHhHhcHHHHHHHHH |
|  | Q Q5T4D3 | 103 | --HPVGFHVVNILLHSGISVLMVDVFSVLFGGLQYTSKGRRLHLAPRASLLAALLFAVHPVHTECVAGVVGRADLLCALF   180 (462) |
|  | Q Consensus | 103 | --~~~~~rl~~~~~~~l~~~l~~~l~~~l~~~~~~~~~~~~~~~~~~~a~~aall~~~~p~~~~~~~~~~~~~~~~~~~f   180 (462) |
|  |  |  | +....|.....+.+++..+++.+.++ ++..+..++.++...|.......+. ++|...+++ |
|  | T Consensus | 72 | ~~~i~~~~~~f~~~~l~~~~li~~i~~~----------------~~~~~~~~~~l~l~~pl~s~~~~~g--~~D~i~~~~   133 (356) |
|  | T PF05208.14 | 72 | GQDIELGQYIFAGIYIATLIVVLSCYIK----------------AGAPPYLLPLLVLSKRLHSIYMLRL--FNDGIATLA   133 (356) |
|  | T ss\_pred |  | CCChHHHHHHHHHHHHHHHHHHHHHHHH----------------cCCChhHHHHHHHccHHHHHHHHhh--hcHHHHHHH |
|  |
|  |
|  | Q ss\_pred |  | HHHHHHHHHHHHHHcCCCCCchHHHHHHHHHHHHHHHHhchHHHHHHHHHHHHHHHHHhCCCChHHHHHHHhhhccchHh |
|  | Q Q5T4D3 | 181 | FLLSFLGYCKAFRESNKEGAHSSTFWVLLSIFLGAVAMLCKEQGITVLGLNAVFDILVIGKFNVLEIVQKVLHKDKSLEN   260 (462) |
|  | Q Consensus | 181 | ~ll~~~~~~~~~~~~~~~~~~~~~~~~~~~~~~~~la~l~k~~~~~~~~~~~~~~~~~~~~~~~~~~~~~~~~~~~~~~~   260 (462) |
|  |  |  | .+++++++.+.+ ...++++.++|+.+|...++..+.+.+.....++.++ |
|  | T Consensus | 134 | lll~l~~l~~~~--------------~~la~i~~glAv~~K~~~ll~~P~ll~~l~~~~~~~~-----------------   182 (356) |
|  | T PF05208.14 | 134 | MWVAIFFFQRRQ--------------LTVATTVWSLGVGVKMSLLLLAPGVAIVIALSGGIWA-----------------   182 (356) |
|  | T ss\_pred |  | HHHHHHHHHhCC--------------HHHHHHHHHHHHHHHHHHHHHHHHHHHHHHHcCCHHH----------------- |
|  |
|  |
|  | Q ss\_pred |  | hchhhhhhHHHHHHHHHHHHHHHHHHHHH |
|  | Q Q5T4D3 | 261 | LGMLRNGGLLFRMTLLTSGGAGMLYVRWR   289 (462) |
|  | Q Consensus | 261 | ~~~~~~~~~~~~~~~~~~~~~~~~~~~~~   289 (462) |
|  |  |  | .........+.......++. |
|  | T Consensus | 183 | ---------~~~~~~~~~~~~~l~~lPfl   202 (356) |
|  | T PF05208.14 | 183 | ---------AVPLALNAVLTQVLLGIPFL   202 (356) |
|  | T ss\_pred |  | ---------HHHHHHHHHHHHHHHHHHHH |
|  |
| --- | | | |
|  | Template alignmentCDD | | |
| 21. | PF09594.11 ; GT87 ; Glycosyltransferase family 87 | | |
|  | Probability: 98.63%, E-value: 0.0000015, Score: 71.46, Aligned cols: 239, Identities: 12%, Similarity: -0.034, | | |
|  |
|  | Q ss\_pred |  | cccCchHHHHHHHHHHHhCCC---CchHHHHHHHHHHHHHHHHHHHHHHHHhccccccccccccchHH--HHHHHHHHHH |
|  | Q Q5T4D3 | 82 | KSYRPLTVLTFRINYYLSGGF---HPVGFHVVNILLHSGISVLMVDVFSVLFGGLQYTSKGRRLHLAP--RASLLAALLF   156 (462) |
|  | Q Consensus | 82 | ~~~~Pl~~~~~~~~~~~~gg~---~~~~~rl~~~~~~~l~~~l~~~l~~~l~~~~~~~~~~~~~~~~~--~~a~~aall~   156 (462) |
|  |  |  | ..|||...++.... .+. |+ .....++..++..+..+..++.+.|+..+ ++ ......++.+ |
|  | T Consensus | 1 | f~YpP~~~~l~~~~-~l~-~~~~~~~~~~~~~~~~~~~~~~~~~~~~~r~~~~-------------~~~~~~~~~~~~~~   65 (251) |
|  | T PF09594.11 | 1 | FTYPPFGALVFTPL-WWI-HDLFGLLVTERVFALITLLTTYAVAVFLLRLAGV-------------RDRVWEFVAFAALL   65 (251) |
|  | T ss\_pred |  | CCCChHHHHHHHHH-hhc-cchhcHHHHHHHHHHHHHHHHHHHHHHHHHHcCC-------------CCcHHHHHHHHHHH |
|  |
|  |
|  | Q ss\_pred |  | HHCHHHHHHHHhHhcHHHHHHHHHHHHHHHHHHHHHHHcCCCCCchHHHHHHHHHHHHHHHHhchHHHHHHHHHHHHHHH |
|  | Q Q5T4D3 | 157 | AVHPVHTECVAGVVGRADLLCALFFLLSFLGYCKAFRESNKEGAHSSTFWVLLSIFLGAVAMLCKEQGITVLGLNAVFDI   236 (462) |
|  | Q Consensus | 157 | ~~~p~~~~~~~~~~~~~~~~~~~f~ll~~~~~~~~~~~~~~~~~~~~~~~~~~~~~~~~la~l~k~~~~~~~~~~~~~~~   236 (462) |
|  |  |  | +.+|. .+..... +.|.+..++.++++++.. ..++++ .++...++++.+++...|...+.+.+.+....+ |
|  | T Consensus | 66 | ~~~p~-~~~~~~g--~~~~~~~~~~~~~l~~~~-~~~~~~-------~~~~~~ag~~l~la~~~K~~~~~~~~~ll~~~~   134 (251) |
|  | T PF09594.11 | 66 | VSAPV-YFTLNIG--QINVMLMALTLFDVALPR-STRHSG-------VLKYVPLGVLTGIAAAIKLTPLVFGLYFLILWV   134 (251) |
|  | T ss\_pred |  | HHHHH-HHHhhcC--CHHHHHHHHHHHHHHhcc-ccCCCC-------ccccHHHHHHHHHHHHhhHHHHHHHHHHHHHHH |
|  |
|  |
|  | Q ss\_pred |  | HHhCCCChHHHHHHHhhhccchHhhchhhhhhHHHHHHHHHHHHHHHHHHHHHHhCCCCCCccccCCcch----hcchhh |
|  | Q Q5T4D3 | 237 | LVIGKFNVLEIVQKVLHKDKSLENLGMLRNGGLLFRMTLLTSGGAGMLYVRWRIMGTGPPAFTEVDNPAS----FADSML   312 (462) |
|  | Q Consensus | 237 | ~~~~~~~~~~~~~~~~~~~~~~~~~~~~~~~~~~~~~~~~~~~~~~~~~~~~~~~~~~~~~~~~~~~~~~----~~~~~~   312 (462) |
|  |  |  | ..++.+. .........+...........+....+.+.....+ ...... |
|  | T Consensus | 135 | ~~~~~r~----------------------------~~~~~~~~~~~~~~~~~~~~~~~~~~~~~~~~~~~~~~~~~~~~~   186 (251) |
|  | T PF09594.11 | 135 | VTKSPRG----------------------------LFGMIGGFLGASGLAIIFRPSISIQYFTDVLFTAERIGDLHFARN   186 (251) |
|  | T ss\_pred |  | HcCCHHH----------------------------HHHHHHHHHHHHHHHHHHCChHHHHHHHHHHhcccccCCcccccc |
|  |
|  |
|  | Q ss\_pred |  | HhHhHHHHHHHHHHHHhhccHhhhccccccccCcccccchHHHHHHHHHHHHHHHHHHHHHhcCCCcchHHHHHHHHHHH |
|  | Q Q5T4D3 | 313 | VRAVNYNYYYSLNAWLLLCPWWLCFDWSMGCIPLIKSISDWRVIALAALWFCLIGLICQALCSEDGHKRRILTLGLGFLV   392 (462) |
|  | Q Consensus | 313 | ~~~~~~~~~~~~~~~~~~~p~~~~~~~~~~~~~~~~~~~~~~~~~~~~~~~~~~~~~~~~~~~~~~~~~~~~~~~~~~~~   392 (462) |
|  |  |  | ......... ...................+.+.+...+..++++.+++.......+..+ |
|  | T Consensus | 187 | ~~l~~~~~~----------------------~~~~~~~~~~~~~~~~~~~~~~~~~~~~~~~~~~~~~~~~~~~~~~~~~   244 (251) |
|  | T PF09594.11 | 187 | VSIRAVLER----------------------LPELGSAASIMWLVAVALVIIAVAVAAYRILRTDLSAHNRLLAVSLVSL   244 (251) |
|  | T ss\_pred |  | ccHHHHHHh----------------------cccCCcHHHHHHHHHHHHHHHHHHHHHHHHHhcccccCChHHHHHHHHH |
|  |
|  |
|  | Q ss\_pred |  | HHHh |
|  | Q Q5T4D3 | 393 | IPFL   396 (462) |
|  | Q Consensus | 393 | ~~~~   396 (462) |
|  |  |  | +..+ |
|  | T Consensus | 245 | ~~~l   248 (251) |
|  | T PF09594.11 | 245 | VALL   248 (251) |
|  | T ss\_pred |  | HHHH |
|  |
| --- | | | |
|  | Template alignmentCDD | | |
| 22. | PF05007.14 ; Mannosyl\_trans ; Mannosyltransferase (PIG-M) | | |
|  | Probability: 94.9%, E-value: 0.87, Score: 36.64, Aligned cols: 202, Identities: 11%, Similarity: -0.067, | | |
|  |
|  | Q ss\_pred |  | cHHHHHHHHHHHHHHHHHHHHHHHcCCCCCchHHHHHHHHHHHHHHHHhchHHHHHHHHHHHHHHHHHhCCCChHHHHHH |
|  | Q Q5T4D3 | 171 | GRADLLCALFFLLSFLGYCKAFRESNKEGAHSSTFWVLLSIFLGAVAMLCKEQGITVLGLNAVFDILVIGKFNVLEIVQK   250 (462) |
|  | Q Consensus | 171 | ~~~~~~~~~f~ll~~~~~~~~~~~~~~~~~~~~~~~~~~~~~~~~la~l~k~~~~~~~~~~~~~~~~~~~~~~~~~~~~~   250 (462) |
|  |  |  | ++.|....+++++++++..|.+ ...++++.|+|..+|...+++.+.+.+...-.+++++..+..+. |
|  | T Consensus | 5 | G~~D~l~~~lvllal~~~~r~~--------------~~~ag~~lgla~~~Kl~Pii~~~~l~l~~~~~~~~~~~~~~~~~   70 (269) |
|  | T PF05007.14 | 5 | GNADSIVASLVLTTLYLIEKRL--------------IACAAVFYGFAVHMKMYPVTYILPIALHLRPERDSDEGLRLARY   70 (269) |
|  | T ss\_pred |  | hhHHHHHHHHHHHHHHHHHcCC--------------HHHHHHHHHHHHHhchHHHHHHHHHHHHhCccCCCCcchhhhcc |
|  |
|  |
|  | Q ss\_pred |  | HhhhccchHhhchhhhhhHHHHHHHHHHHHHHHHHHHHHHhCCCCC---CccccCCcchhcchhhHhHhHHHHHHHHHHH |
|  | Q Q5T4D3 | 251 | VLHKDKSLENLGMLRNGGLLFRMTLLTSGGAGMLYVRWRIMGTGPP---AFTEVDNPASFADSMLVRAVNYNYYYSLNAW   327 (462) |
|  | Q Consensus | 251 | ~~~~~~~~~~~~~~~~~~~~~~~~~~~~~~~~~~~~~~~~~~~~~~---~~~~~~~~~~~~~~~~~~~~~~~~~~~~~~~   327 (462) |
|  |  |  | ..+++.........+...........................+... .+.........+.+........... |
|  | T Consensus | 71 | ~~~~~~~~~~~~~~~~~~~~~~~~~~~~~~~l~~~~~~~~g~~~~~~~~~~~~~r~~~~~n~S~~~~~~~l~~~------   144 (269) |
|  | T PF05007.14 | 71 | SFQARLYDFLKRLCSWAVLLFVAIAGLTFLALSFGFYYKYGWEFLEHTYLYHLTRRDIRHNFSPYFYMLYLTAE------   144 (269) |
|  | T ss\_pred |  | cHHHHHHHHHHHhccHHHHHHHHHHHHHHHHHHHHHHHHHCHHHHHHHHHHHhccCCCCcCCCHHHHHHHHhcc------ |
|  |
|  |
|  | Q ss\_pred |  | HhhccHhhhccccccccCcccccchHHHHHHHHHHHHHHHHHHHHHhcCCCcchHHHHHHHHHHHHHHhhHhccccccch |
|  | Q Q5T4D3 | 328 | LLLCPWWLCFDWSMGCIPLIKSISDWRVIALAALWFCLIGLICQALCSEDGHKRRILTLGLGFLVIPFLPASNLFFRVGF   407 (462) |
|  | Q Consensus | 328 | ~~~~p~~~~~~~~~~~~~~~~~~~~~~~~~~~~~~~~~~~~~~~~~~~~~~~~~~~~~~~~~~~~~~~~~~~~~~~~~~~   407 (462) |
|  |  |  | ........................+. +++................ . |
|  | T Consensus | 145 | -----------------------~~~~~~~~~~~~~~~~~~~~~~~~~~--~~~~~~~~~~~~~~f~~~~---------~   190 (269) |
|  | T PF05007.14 | 145 | -----------------------SKWSFTLGIAAFLPQFILLSAASFAY--YRDLVFCCFLHTSIFVTFN---------K   190 (269) |
|  | T ss\_pred |  | -----------------------CcchhHHHHHHHHHHHHHHHHHHHHc--cCcHHHHHHHHHHHHHHhc---------h |
|  |
|  |
|  | Q ss\_pred |  | hHHHhhhhHHHHHHHHHHH |
|  | Q Q5T4D3 | 408 | VVAERVLYLPSVGYCVLLT   426 (462) |
|  | Q Consensus | 408 | ~~~~ry~~~~~~~~~ll~a   426 (462) |
|  |  |  | ...++|.....|.+.+... |
|  | T Consensus | 191 | v~~~qY~~W~lpll~l~~~   209 (269) |
|  | T PF05007.14 | 191 | VCTSQYFLWYLCLLPLVMP   209 (269) |
|  | T ss\_pred |  | hhcHHHHHHHHHHHHHHch |
|  |
| --- | | | |
|  | Template alignmentCDD | | |
| 23. | PF14897.7 ; EpsG ; EpsG family | | |
|  | Probability: 93.55%, E-value: 1.9, Score: 35.4, Aligned cols: 306, Identities: 10%, Similarity: -0.032, | | |
|  |
|  | Q ss\_pred |  | HHHHHHHHHHHHhhhcCC-CcccCchHHHHhcccccCCCChhhhhcccccccccCCCCCc----cccCchHHHHHHHHHH |
|  | Q Q5T4D3 | 23 | KLVVGSVAIVCFARSYDG-DFVFDDSEAIVNNKDLQAETPLGDLWHHDFWGSRLSSNTSH----KSYRPLTVLTFRINYY   97 (462) |
|  | Q Consensus | 23 | ~~~l~~~~~~~~~~~~~~-~~~~Dd~~~~~~~~~~~~~~~~~~~~~~~~~~~~~~~~~~~----~~~~Pl~~~~~~~~~~   97 (462) |
|  |  |  | ...++............. +...|...|...-++..+.+......+ ... ....|++..+...... |
|  | T Consensus | 2 | ~~~~~~~~~l~~~~~~R~~~~g~D~~~Y~~~y~~~~~~~~~~~~~~-----------~~~~~~~~~~E~gf~~l~~~~~~   70 (319) |
|  | T PF14897.7 | 2 | WFVSFATIQWIVLSGFRDVTVGADTAQYKALFLQSQTLPLGAFTDR-----------FFEIVFTESEDPGFYLFQRLIQY   70 (319) |
|  | T ss\_pred |  | HHHHHHHHHHHHHHHHcCcCCCCCHHHHHHHHHHhccCCHHHHhhh-----------hhhccCCCCCCHHHHHHHHHHHH |
|  |
|  |
|  | Q ss\_pred |  | HhCCCCchHHHHHHHHHHHHHHHHHHHHHHHHhccccccccccccchHHHHHHHHHHHHHHCHHHHHHHHhHhcHHHHHH |
|  | Q Q5T4D3 | 98 | LSGGFHPVGFHVVNILLHSGISVLMVDVFSVLFGGLQYTSKGRRLHLAPRASLLAALLFAVHPVHTECVAGVVGRADLLC   177 (462) |
|  | Q Consensus | 98 | ~~gg~~~~~~rl~~~~~~~l~~~l~~~l~~~l~~~~~~~~~~~~~~~~~~~a~~aall~~~~p~~~~~~~~~~~~~~~~~   177 (462) |
|  |  |  | + |.+ .+....+.+.++..+.+...++..+ +.. ....+.+........... .-..++ |
|  | T Consensus | 71 | ~--~~~---~~~~~~~~~~i~~~~~~~~~~~~~~-------------~~~----~~~~~~~~~~~~~~~~~~--~Rq~lA   126 (319) |
|  | T PF14897.7 | 71 | V--ITD---YQVYLVLIAMIFMIPLGYFIYKYSS-------------EPL----ISFLLFSVLFYEFFAVTG--LRQTVA   126 (319) |
|  | T ss\_pred |  | h--cCC---HHHHHHHHHHHHHHHHHHHHHHhCC-------------chH----HHHHHHHHHHHHHHHhHH--HHHHHH |
|  |
|  |
|  | Q ss\_pred |  | HHH-HHHHHHHHHHHHHHcCCCCCchHHHHHHHHHHHHHHHHhchHHHHHHHHHHHHHHHHHhCCCChHHHHHHHhhhcc |
|  | Q Q5T4D3 | 178 | ALF-FLLSFLGYCKAFRESNKEGAHSSTFWVLLSIFLGAVAMLCKEQGITVLGLNAVFDILVIGKFNVLEIVQKVLHKDK   256 (462) |
|  | Q Consensus | 178 | ~~f-~ll~~~~~~~~~~~~~~~~~~~~~~~~~~~~~~~~la~l~k~~~~~~~~~~~~~~~~~~~~~~~~~~~~~~~~~~~   256 (462) |
|  |  |  | ..+ .+.++.... ++| .....+...+|...|.+++...+. ....+++.+ |
|  | T Consensus | 127 | ~~~~~l~a~~~~~----~~~----------~~~~~~~~~la~~~H~sali~i~~----~~~~~~~~~-------------   175 (319) |
|  | T PF14897.7 | 127 | TALVVLVGYHFVR----ARK----------LGWFLLLVCIAMTIHKSSLIFVPF----YFLANKQLT-------------   175 (319) |
|  | T ss\_pred |  | HHHHHHHHHHHHH----cCc----------HHHHHHHHHHHHHHHHHHHHHHHH----HHHhhcCCc------------- |
|  |
|  |
|  | Q ss\_pred |  | chHhhchhhhhhHHHHHHHHHHHHHHHHHHHHHHhCCCCCCccccCCcchhcchhhHhHhHHHHHHHHHHHHhhccHhhh |
|  | Q Q5T4D3 | 257 | SLENLGMLRNGGLLFRMTLLTSGGAGMLYVRWRIMGTGPPAFTEVDNPASFADSMLVRAVNYNYYYSLNAWLLLCPWWLC   336 (462) |
|  | Q Consensus | 257 | ~~~~~~~~~~~~~~~~~~~~~~~~~~~~~~~~~~~~~~~~~~~~~~~~~~~~~~~~~~~~~~~~~~~~~~~~~~~p~~~~   336 (462) |
|  |  |  | +................................+.+.+..... .... |
|  | T Consensus | 176 | --------~~~~~~~~~~~~~~~~~~~~i~~~~~~~~~~~~Y~~~~~~~~~----------~~~~---------------   222 (319) |
|  | T PF14897.7 | 176 | --------KAYLMTMFGVIVGLFVFRNPFFDLLVQVSGYDTYSAMDGAGAV----------NFSL---------------   222 (319) |
|  | T ss\_pred |  | --------HHHHHHHHHHHHHHHHhcHHHHHHHHHHhchhhhhhccCcchH----------HHHH--------------- |
|  |
|  |
|  | Q ss\_pred |  | ccccccccCcccccchHHHHHHHHHHHHHHHHHHHHHhcCCCcchHHHHHHHHHHHHHHhhHhccccccchhHHHhhhhH |
|  | Q Q5T4D3 | 337 | FDWSMGCIPLIKSISDWRVIALAALWFCLIGLICQALCSEDGHKRRILTLGLGFLVIPFLPASNLFFRVGFVVAERVLYL   416 (462) |
|  | Q Consensus | 337 | ~~~~~~~~~~~~~~~~~~~~~~~~~~~~~~~~~~~~~~~~~~~~~~~~~~~~~~~~~~~~~~~~~~~~~~~~~~~ry~~~   416 (462) |
|  |  |  | ...............++++.+................+.... .....|.... |
|  | T Consensus | 223 | -----------------------~~~~~~~~~~~~~~~~~~~~~~~~~~~~~~~~~~~~~~~~~~-----~~~~~R~~~~   274 (319) |
|  | T PF14897.7 | 223 | -----------------------MLLSVLFVALWRKEQILANNPSAIHFFNALLLAACLLPLTFL-----NPSMMRLVQY   274 (319) |
|  | T ss\_pred |  | -----------------------HHHHHHHHHHHHHHHHhcCCcchHHHHHHHHHHHHHHHHHhc-----ChhHHHHHHH |
|  |
|  |
|  | Q ss\_pred |  | HHHHHHHHHHHHHHHHHhchhhHHHHHHHHHHHHHHHHHH |
|  | Q Q5T4D3 | 417 | PSVGYCVLLTFGFGALSKHTKKKKLIAAVVLGILFINTLR   456 (462) |
|  | Q Consensus | 417 | ~~~~~~ll~a~~~~~~~~~~~~~~~~~~~~~~~~~~~~~~   456 (462) |
|  |  |  | ..+... .....+.+..++++++......++......... |
|  | T Consensus | 275 | ~~~~~~-~~~~~~~~~~~~~~~~~~~~~~~~~~~~~~~~~   313 (319) |
|  | T PF14897.7 | 275 | FSLFLL-LMIPEIVGTFERRERLVVYYSAVMLLGLLFIRE   313 (319) |
|  | T ss\_pred |  | HHHHHH-HHHHHHHhcCChHHHHHHHHHHHHHHHHHHHHH |
|  |
| --- | | | |
|  | Template alignmentCDD | | |
| 24. | PF10060.10 ; DUF2298 ; Uncharacterized membrane protein (DUF2298) | | |
|  | Probability: 88.03%, E-value: 11, Score: 34.1, Aligned cols: 363, Identities: 12%, Similarity: 0, | | |
|  |
|  | Q ss\_pred |  | ccccCCHHHHHHHHHHHHHHHHHhhhcCCCcc-cCchHHHHhcccccCCCChhhhhcccccccccCCCCCccccCchHHH |
|  | Q Q5T4D3 | 12 | LPSSVLPPFWAKLVVGSVAIVCFARSYDGDFV-FDDSEAIVNNKDLQAETPLGDLWHHDFWGSRLSSNTSHKSYRPLTVL   90 (462) |
|  | Q Consensus | 12 | ~~~~~~~~~~~~~~l~~~~~~~~~~~~~~~~~-~Dd~~~~~~~~~~~~~~~~~~~~~~~~~~~~~~~~~~~~~~~Pl~~~   90 (462) |
|  |  |  | .++.+.....-++.+++..........+.+.+ .|...+..........+.++.... +..+.... |+.+.++ |
|  | T Consensus | 49 | ~~~~~~~l~~e~vf~~~f~~~~~~r~~~p~i~~~Ek~md~~~i~s~~~~~~~Pp~dP-------w~aG~~l~-Yyyfg~~   120 (597) |
|  | T PF10060.10 | 49 | LPSLRYILLFELLFLGAFAAWAWVRAHDPAADHTEQPMDLMFMHSIRASLTYPPHDA-------WLAGYPIS-YYYFGYW   120 (597) |
|  | T ss\_pred |  | ccchHHHHHHHHHHHHHHHHHHHHHHhCCCCCCCCChhHHHHHHHHHhcCCCCccch-------hhcCCCcc-ccHHHHH |
|  |
|  |
|  | Q ss\_pred |  | HHHHHHHHhCCCCchHHHHHHHHHHHHHHHHHHHHHHHHhccccccccccccchHHH--------HHHHHHHHHHHCHHH |
|  | Q Q5T4D3 | 91 | TFRINYYLSGGFHPVGFHVVNILLHSGISVLMVDVFSVLFGGLQYTSKGRRLHLAPR--------ASLLAALLFAVHPVH   162 (462) |
|  | Q Consensus | 91 | ~~~~~~~~~gg~~~~~~rl~~~~~~~l~~~l~~~l~~~l~~~~~~~~~~~~~~~~~~--------~a~~aall~~~~p~~   162 (462) |
|  |  |  | +.+...++.|-....++++....+..+.+..+|.+++++.+ +++ .|++++++..+.... |
|  | T Consensus | 121 | ~~A~l~~l~gi~~~~~~nl~~~~~~al~~~~~~~l~~~l~~-------------~~~~~~~~~~~~g~la~~l~~~~gnl   187 (597) |
|  | T PF10060.10 | 121 | LMNMVGLMAGQSAAVAYNLSQAVWFGLLLSGAFGIGYNLVA-------------AAGRRFVAALGGGWVATLLVGLSSNL   187 (597) |
|  | T ss\_pred |  | HHHHHHHHhCCCHHHHHHHHHHHHHHHHHHHHHHHHHHHHH-------------hccccccHHHHHHHHHHHHHHHhccc |
|  |
|  |
|  | Q ss\_pred |  | -----------------------------------------H---------------------------HHHHhHhcHHH |
|  | Q Q5T4D3 | 163 | -----------------------------------------T---------------------------ECVAGVVGRAD   174 (462) |
|  | Q Consensus | 163 | -----------------------------------------~---------------------------~~~~~~~~~~~   174 (462) |
|  |  |  | . .+.....-.++ |
|  | T Consensus | 188 | ~~~~~~~~~~~~~~~~~~~~~~~~~~~~~~~~~~~~~~~~~w~~w~ssRvI~~~~~~~~~~~tI~EFP~fSfl~gDLHpH   267 (597) |
|  | T PF10060.10 | 188 | QGLLEWLHANGVDISWLAAWLQVRGFPENAEVTRQWFISYGWWWWRSSRVLADVSLRGDHIEVIDEFPAFSYILGDNHPH   267 (597) |
|  | T ss\_pred |  | HHHHHHHHhCCCCchhHHHHhhhcCCCcchhhhccccccCccccccceeeeecccCCCCCCcccccCchHHHhcCCCChh |
|  |
|  |
|  | Q ss\_pred |  | HHHHHHHHHHHHHHHHHHHHcCCCCCchHHH--------------HHHHHHHHHHHHHhchHHHHHHHHHHHHHHHHHhC |
|  | Q Q5T4D3 | 175 | LLCALFFLLSFLGYCKAFRESNKEGAHSSTF--------------WVLLSIFLGAVAMLCKEQGITVLGLNAVFDILVIG   240 (462) |
|  | Q Consensus | 175 | ~~~~~f~ll~~~~~~~~~~~~~~~~~~~~~~--------------~~~~~~~~~~la~l~k~~~~~~~~~~~~~~~~~~~   240 (462) |
|  |  |  | .+...+.++++.+.+...+++++++.....+ ..++.+++.|....++.--......+......... |
|  | T Consensus | 268 | ~~alPf~ll~l~l~~~~~~~~~~~~~~~~~~~~~~~~~~~~~~~~~~ll~gll~G~l~~~NtWD~p~~~~l~~~~~~~~~   347 (597) |
|  | T PF10060.10 | 268 | VAAMPFAMLAVAAALVIFLQNSSSNFSSESRAKFNFAPLFPLGWGGFLLVAVITGSLLFLNTWDYPPYWLLTTFSIAVGV   347 (597) |
|  | T ss\_pred |  | hhHHHHHHHHHHHHHHHHhcCCCCCCCchhhhccCCCCccccchHHHHHHHHHHHHHHHHccCCHHHHHHHHHHHHHHHH |
|  |
|  |
|  | Q ss\_pred |  | CCC--------------hHHHHHHHhhhccchHhhchhhhhhHHHHHHHHHHHHHHHHHHHHHHhCCCCCCccccCC-cc |
|  | Q Q5T4D3 | 241 | KFN--------------VLEIVQKVLHKDKSLENLGMLRNGGLLFRMTLLTSGGAGMLYVRWRIMGTGPPAFTEVDN-PA   305 (462) |
|  | Q Consensus | 241 | ~~~--------------~~~~~~~~~~~~~~~~~~~~~~~~~~~~~~~~~~~~~~~~~~~~~~~~~~~~~~~~~~~~-~~   305 (462) |
|  |  |  | .++ ..+..++ .........+.......++.....+......... .. |
|  | T Consensus | 348 | ~~~~~~~~~~~~~~~~~~~~~~~~-------------------~~~~~~~~~~~a~ll~lPF~l~f~~~~~gi~~~~~~~   408 (597) |
|  | T PF10060.10 | 348 | VGGVVRVKNFLPLQFLPLLPPLLQ-------------------TTIAGLALFVAALLLYLPYLLTAQSQVGGLIPNLFHP   408 (597) |
|  | T ss\_pred |  | hccccccccccccccccchHHHHH-------------------HHHHHHHHHHHHHHHHHHHHHhccccCCCCcccCCCC |
|  |
|  |
|  | Q ss\_pred |  | hhcchhhHhHhHHHHHHHHHHHHhhccHhhhccccccccCcccccchHHHHHHHHHHHHHHHHHHHHHhcCCCcchHHHH |
|  | Q Q5T4D3 | 306 | SFADSMLVRAVNYNYYYSLNAWLLLCPWWLCFDWSMGCIPLIKSISDWRVIALAALWFCLIGLICQALCSEDGHKRRILT   385 (462) |
|  | Q Consensus | 306 | ~~~~~~~~~~~~~~~~~~~~~~~~~~p~~~~~~~~~~~~~~~~~~~~~~~~~~~~~~~~~~~~~~~~~~~~~~~~~~~~~   385 (462) |
|  |  |  | +........+. ..+..++.......++.+++...... |
|  | T Consensus | 409 | T~l~~~l~i~G-------------------------------------------lfl~l~~~~l~~~~~~~~~~~~~~~~   445 (597) |
|  | T PF10060.10 | 409 | TRFSQYVAMFA-------------------------------------------TALLTLTALLTFGWSVFRPRLKVVMI   445 (597) |
|  | T ss\_pred |  | CCHHHHHHHHH-------------------------------------------HHHHHHHHHHHHHhhcccchHHHHHH |
|  |
|  |
|  | Q ss\_pred |  | HHHHHHHHHHhhHhc----------------------cccccchhHHHhhhhHH--HHHHHHHHHHHHHHHHhchh---- |
|  | Q Q5T4D3 | 386 | LGLGFLVIPFLPASN----------------------LFFRVGFVVAERVLYLP--SVGYCVLLTFGFGALSKHTK----   437 (462) |
|  | Q Consensus | 386 | ~~~~~~~~~~~~~~~----------------------~~~~~~~~~~~ry~~~~--~~~~~ll~a~~~~~~~~~~~----   437 (462) |
|  |  |  | ............... -..........|....+ ...+..+++.......++.+ |
|  | T Consensus | 446 | ~~~~~~~~~~~~~~~~~~~~~~~~~~~~~~~~~~~~~~~~~~~~~~~~~~~~~~~~~~ll~~l~~~~~~~~~~~~~~~~~   525 (597) |
|  | T PF10060.10 | 446 | CLALTLGTPALLLTFIAWVATGTEEGRASLGNVALPDGASSYLPFIVERWTAQPFTFLIVGAMTAVALALLWTGIQHMVG   525 (597) |
|  | T ss\_pred |  | HHHHHHHHHHHHHHHHHHHhcCChhhhhccccccCCCCcccchhHHHHHhccchHHHHHHHHHHHHHHHHHHhhhhhhcc |
|  |
|  |
|  | Q ss\_pred |  | -------------------------hHHHHHHHHHHHHHHHHHHH |
|  | Q Q5T4D3 | 438 | -------------------------KKKLIAAVVLGILFINTLRC   457 (462) |
|  | Q Consensus | 438 | -------------------------~~~~~~~~~~~~~~~~~~~~   457 (462) |
|  |  |  | .......+++.+........ |
|  | T Consensus | 526 | ~~~~~~~~~~~~~~~~~~~~~~~~~~~~~f~l~L~~~gl~Lil~~   570 (597) |
|  | T PF10060.10 | 526 | AKNFLPQHFSPQQGALDSTPGVAAPTPLLFVLALAVIGLGLTFTP   570 (597) |
|  | T ss\_pred |  | ccccCcccCCCCCCCcCCCCCCCCCHHHHHHHHHHHHHHHHHHHH |
|  |
| --- | | | |
|  | Template alignmentCDD | | |
| 25. | PF16192.6 ; PMT\_4TMC ; C-terminal four TMM region of protein-O-mannosyltransferase | | |
|  | Probability: 79.5%, E-value: 12, Score: 27.76, Aligned cols: 106, Identities: 11%, Similarity: -0.11, | | |
|  |
|  | Q ss\_pred |  | chHHHHHHHHHHHHHHHHHHHHHhcCCCcchHH-----------HHHHHHHHHHHHhhHhccccccchhHHHhhhhHHHH |
|  | Q Q5T4D3 | 351 | SDWRVIALAALWFCLIGLICQALCSEDGHKRRI-----------LTLGLGFLVIPFLPASNLFFRVGFVVAERVLYLPSV   419 (462) |
|  | Q Consensus | 351 | ~~~~~~~~~~~~~~~~~~~~~~~~~~~~~~~~~-----------~~~~~~~~~~~~~~~~~~~~~~~~~~~~ry~~~~~~   419 (462) |
|  |  |  | ++.........++...........++++..... ....++..++..+|..... ......|.+++++ |
|  | T Consensus | 60 | Np~iw~~~~~~l~~~~~~~~~~~~~~~r~~~~~~~~~~~~~~~~~~~~~~g~~~~ylP~~~~~----r~~~~~~ylpal~   135 (198) |
|  | T PF16192.6 | 60 | NPFVYWASTASLGLVGLVVVWYILRWQRGFKDLDSEEVDQIHYAGIYPVLGWFLHYLPFVIMA----RVTYVHHYYPALY   135 (198) |
|  | T ss\_pred |  | CHHHHHHHHHHHHHHHHHHHHHHHHHhcCCCCCChHHHHHHHHHHHHHHHHHHHHHHHHHhcc----ccccHHhHHHHHH |
|  |
|  |
|  | Q ss\_pred |  | HHHHHHHHHHHHHHhchhhHHHHHHHHHHHHHHHHHHHHHh |
|  | Q Q5T4D3 | 420 | GYCVLLTFGFGALSKHTKKKKLIAAVVLGILFINTLRCVLR   460 (462) |
|  | Q Consensus | 420 | ~~~ll~a~~~~~~~~~~~~~~~~~~~~~~~~~~~~~~~~~~   460 (462) |
|  |  |  | +.+++.+..++...++..+........+.+.+......... |
|  | T Consensus | 136 | f~~l~~~~~l~~~~~~~~~~~~~~~~~~~~~~~~~~f~~~~   176 (198) |
|  | T PF16192.6 | 136 | FAILALGFFVDWLLRNRSQAIQGAVYGVLYSVIVGLYITFI   176 (198) |
|  | T ss\_pred |  | HHHHHHHHHHHHHHHccchHHHHHHHHHHHHHHHHHHHHhh |
|  |
| --- | | | |
|  | Template alignmentCDD | | |
| 26. | PF09971.10 ; DUF2206 ; Predicted membrane protein (DUF2206) | | |
|  | Probability: 49.01%, E-value: 83, Score: 26.03, Aligned cols: 206, Identities: 7%, Similarity: -0.073, | | |
|  |
|  | Q ss\_pred |  | HHHHHHHHHHHhchHHHHHHHHHHHHHHH----HHh-------CCCChHHHHHHHhhhccchHhhchhhhhhHH------ |
|  | Q Q5T4D3 | 208 | LLSIFLGAVAMLCKEQGITVLGLNAVFDI----LVI-------GKFNVLEIVQKVLHKDKSLENLGMLRNGGLL------   270 (462) |
|  | Q Consensus | 208 | ~~~~~~~~la~l~k~~~~~~~~~~~~~~~----~~~-------~~~~~~~~~~~~~~~~~~~~~~~~~~~~~~~------   270 (462) |
|  |  |  | ++..++...-+++|+....+...++.... +.+ ++.. .. |
|  | T Consensus | 3 | ~L~~i~~~~lv~sH~~t~~~~~~~l~~~~~~~~~~~~~~~~~~~~~~-------------------------~~~~~~~~   57 (390) |
|  | T PF09971.10 | 3 | ILFSVFSCGIIISHYGLTYMVIGLIALSYVLFTFINLVARYINTDKV-------------------------IIPVTPIR   57 (390) |
|  | T ss\_pred |  | HHHHHHHHHHHHhchHHHHHHHHHHHHHHHHHHHHHHHHHhcCCCCC-------------------------cCCCCccc |
|  |
|  |
|  | Q ss\_pred |  | HHHHHHHHHHHHHHHHHHHHhCCCCCCccccCCcchhcchhhHhHhHHHHHHHHHHHHhhccHhhhccccccccCccccc |
|  | Q Q5T4D3 | 271 | FRMTLLTSGGAGMLYVRWRIMGTGPPAFTEVDNPASFADSMLVRAVNYNYYYSLNAWLLLCPWWLCFDWSMGCIPLIKSI   350 (462) |
|  | Q Consensus | 271 | ~~~~~~~~~~~~~~~~~~~~~~~~~~~~~~~~~~~~~~~~~~~~~~~~~~~~~~~~~~~~~p~~~~~~~~~~~~~~~~~~   350 (462) |
|  |  |  | ......++..+....|.....+.. ..+............+...+.................... |
|  | T Consensus | 58 | ~~~~~~~~~~v~~~~W~~~~~~~~----------------~~~~~~~~~~~~~~~~~~~~~~~~~~~~~~~~~~~~~~~~   121 (390) |
|  | T PF09971.10 | 58 | LNFLHICIFIFIALSWYIAITSST----------------AFYSVSSVIYQVISSMFTESLNPTASQGLAIIQKVPVSQM   121 (390) |
|  | T ss\_pred |  | ccHHHHHHHHHHHHHHHHHHhcch----------------HHHHHHHHHHHHHHHHhHHhcCCccchhHHHHhcCCCchH |
|  |
|  |
|  | Q ss\_pred |  | chHHHHHHHHHHHHHHHHHHHH----HhcCCCcchHHHHHHHHHHHHHHhhHhccccccc-hhHHHhhhhHHHHHHHHHH |
|  | Q Q5T4D3 | 351 | SDWRVIALAALWFCLIGLICQA----LCSEDGHKRRILTLGLGFLVIPFLPASNLFFRVG-FVVAERVLYLPSVGYCVLL   425 (462) |
|  | Q Consensus | 351 | ~~~~~~~~~~~~~~~~~~~~~~----~~~~~~~~~~~~~~~~~~~~~~~~~~~~~~~~~~-~~~~~ry~~~~~~~~~ll~   425 (462) |
|  |  |  | ............+..+...... ++++++++.......+....+..+.......... .....|.+....+++++++ |
|  | T Consensus | 122 | ~~~~~~~~~~~~~l~~iG~~~~~~~~~~~~~~~~~~~~~~~~~~~~~~~~~~~~~~p~~~~~~~~~R~~~~~~~~~~~~a   201 (390) |
|  | T PF09971.10 | 122 | HLLYTYIYYFNQVCIVLGLLYLSYKTFARKNMYNYSIMQLIMCGVAVMVLVGSIVLPYFASALNTTRIYHIMQFFVSPVY   201 (390) |
|  | T ss\_pred |  | HHHHHHHHHHHHHHHHHHHHHHHHHHHhccCccCCCHHHHHHHHHHHHHHHHHHHHHHHHHhcChHHHHHHHHHHHHHHH |
|  |
|  |
|  | Q ss\_pred |  | HHHHHHHHhchhhH----------------HHHHHHHHHHHHHHH |
|  | Q Q5T4D3 | 426 | TFGFGALSKHTKKK----------------KLIAAVVLGILFINT   454 (462) |
|  | Q Consensus | 426 | a~~~~~~~~~~~~~----------------~~~~~~~~~~~~~~~   454 (462) |
|  |  |  | +.++..+.+..++. .....+++++.++.. |
|  | T Consensus | 202 | ~~g~~~l~~~~~~~~~~~~~~~~~~~~~~~~~~~~~~lv~~~~~~   246 (390) |
|  | T PF09971.10 | 202 | IIGFIFALESIPKVYARIVKSPFRSNLSFTYGIISLFLCVYLLFN   246 (390) |
|  | T ss\_pred |  | HHHHHHHHHhhHHHHHHhhcCccccccchHHHHHHHHHHHHHHHH |
|  |
| --- | | | |
|  | Template alignmentCDD | | |
| 27. | PF11694.9 ; DUF3290 ; Protein of unknown function (DUF3290) | | |
|  | Probability: 33.44%, E-value: 78, Score: 21.87, Aligned cols: 60, Identities: 3%, Similarity: -0.13, | | |
|  |
|  | Q ss\_pred |  | cccccccCcccccchHHHHHHHHHHHHHHHHHHHHHhcCCCcchHHHHHHHHHHHHHHhh |
|  | Q Q5T4D3 | 338 | DWSMGCIPLIKSISDWRVIALAALWFCLIGLICQALCSEDGHKRRILTLGLGFLVIPFLP   397 (462) |
|  | Q Consensus | 338 | ~~~~~~~~~~~~~~~~~~~~~~~~~~~~~~~~~~~~~~~~~~~~~~~~~~~~~~~~~~~~   397 (462) |
|  |  |  | +|++.............-....++++++++.......|.+...+-+-+..++.+++.++. |
|  | T Consensus | 2 | FYsy~YL~~q~~~~~~~~~~~i~~l~~~li~~~~~y~R~R~~tKYRDL~II~~L~~ll~~   61 (142) |
|  | T PF11694.9 | 2 | FYTYDYLQGSQSSWQYARIIILSVLAVIFIGFLVHYLRNRMDSKYKDLTIIVGTLLLLIL   61 (142) |
|  | T ss\_pred |  | ccchHHHccccChhHHHHHHHHHHHHHHHHHHHHHHHHhccchhHHHHHHHHHHHHHHHH |
|  |
| --- | | | |
|  | Template alignmentCDD | | |
| 28. | PF08409.12 ; DUF1736 ; Domain of unknown function (DUF1736) | | |
|  | Probability: 30.66%, E-value: 53, Score: 18.49, Aligned cols: 73, Identities: 37%, Similarity: 0.631, | | |
|  |
|  | Q ss\_pred |  | CCCCCccccCCcchhcchhhHhHhHHHHHHHHHHHHhhccHhhhccccccccCcccccchHHHHHHHHHHHHH |
|  | Q Q5T4D3 | 293 | TGPPAFTEVDNPASFADSMLVRAVNYNYYYSLNAWLLLCPWWLCFDWSMGCIPLIKSISDWRVIALAALWFCL   365 (462) |
|  | Q Consensus | 293 | ~~~~~~~~~~~~~~~~~~~~~~~~~~~~~~~~~~~~~~~p~~~~~~~~~~~~~~~~~~~~~~~~~~~~~~~~~   365 (462) |
|  |  |  | ...+.....+++.........+..........++....+|..+..++.....+...+..+...........++ |
|  | T Consensus | 1 | ~~~~~~~~~~n~~~~~~~~~~~~~~~~~~~~~yl~ll~~P~~l~~~~~~~~~~~~~~~~~~~~~~~~~~~~~~   73 (74) |
|  | T PF08409.12 | 1 | SGPPVFAAADNPTAKSPSLVTRTLTFLYLPAENFRLLVYPRRLSFDWSMDAIAPVTSVYDPRNALSVALYVAL   73 (74) |
|  | T ss\_pred |  | CCCCCCcccCCchhcCCCHHHHHHHHHHHHHHHHHHHHCccccCCCCCcccCCCCCCCCCHHHHHHHHHHHHh |
|  |
| --- | | | |
|  | Template alignmentCDD | | |
| 29. | PF12273.9 ; RCR ; Chitin synthesis regulation, resistance to Congo red | | |
|  | Probability: 26.77%, E-value: 35, Score: 23.57, Aligned cols: 29, Identities: 21%, Similarity: 0.32, | | |
|  |
|  | Q ss\_pred |  | hhhhHHHHHHHHHHHHHHHHHHhchhhHH |
|  | Q Q5T4D3 | 412 | RVLYLPSVGYCVLLTFGFGALSKHTKKKK   440 (462) |
|  | Q Consensus | 412 | ry~~~~~~~~~ll~a~~~~~~~~~~~~~~   440 (462) |
|  |  |  | |+.+..++.+++++.+++..+..++++++ |
|  | T Consensus | 1 | RWvl~~iii~~~l~~~~~~~~~~RRRrr~   29 (138) |
|  | T PF12273.9 | 1 | RWVVLAGVIVIVLVIFMLCTCTARRRRRR   29 (138) |
|  | T ss\_pred |  | CHHHHHHHHHHHHHHHHHHHHHHHHHHHc |
|  |
| --- | | | |
|  | Template alignmentCDD | | |
| 30. | PF04973.13 ; NMN\_transporter ; Nicotinamide mononucleotide transporter | | |
|  | Probability: 25.76%, E-value: 130, Score: 21.41, Aligned cols: 93, Identities: 12%, Similarity: 0.079, | | |
|  |
|  | Q ss\_pred |  | HHHHHHHHHHHHHHHHHHHHHhccccccccccccchHHHHHHHHHHHHHHCHHHHHHHHhHhcHHHHHHHHHHHHHHHHH |
|  | Q Q5T4D3 | 109 | VVNILLHSGISVLMVDVFSVLFGGLQYTSKGRRLHLAPRASLLAALLFAVHPVHTECVAGVVGRADLLCALFFLLSFLGY   188 (462) |
|  | Q Consensus | 109 | l~~~~~~~l~~~l~~~l~~~l~~~~~~~~~~~~~~~~~~~a~~aall~~~~p~~~~~~~~~~~~~~~~~~~f~ll~~~~~   188 (462) |
|  |  |  | ..+.+..+.++..++...|+... +...+.+..+++...... ...+. .-.+...+...+++.+ |
|  | T Consensus | 2 | ~~~~~~~~~g~~~~~l~~~~~~~-------------~~~~g~~~~~~~~~~~~~--~~~~~---~~~l~~~y~~~~i~G~   63 (180) |
|  | T PF04973.13 | 2 | YIEIFASVMGIINVWLLAREKVS-------------NFLFGMITVAVFLYIFIT--QGLYA---MAVLAAFQFIFNVYGW   63 (180) |
|  | T ss\_pred |  | HHHHHHHHHHHHHHHHHHccchh-------------HHHHHHHHHHHHHHHHHH--CCChH---HHHHHHHHHHHHHHHH |
|  |
|  |
|  | Q ss\_pred |  | HHHHHHcCCC-----CCchHHHHHHHHHHHHHHHHh |
|  | Q Q5T4D3 | 189 | CKAFRESNKE-----GAHSSTFWVLLSIFLGAVAML   219 (462) |
|  | Q Consensus | 189 | ~~~~~~~~~~-----~~~~~~~~~~~~~~~~~la~l   219 (462) |
|  |  |  | .++.++++++ +.................... |
|  | T Consensus | 64 | ~~W~k~~~~~~~v~v~~~~~~~~~~~~~~~~v~~~~   99 (180) |
|  | T PF04973.13 | 64 | YHWIARSGEEEVKATVRLDLKGWIFYIIFILVAWIG   99 (180) |
|  | T ss\_pred |  | HHhHhcCCCCCcccceecCHHHHHHHHHHHHHHHHH |
|  |
| --- | | | |
|  | Template alignmentCDD | | |
| 31. | PF05767.13 ; Pox\_A14 ; Poxvirus virion envelope protein A14 | | |
|  | Probability: 25.21%, E-value: 84, Score: 19.6, Aligned cols: 37, Identities: 19%, Similarity: 0.325, | | |
|  |
|  | Q ss\_pred |  | HHHHHHHHHHHHHHHHHcCCCCCchHHHHHHHHHHHHHHHHh |
|  | Q Q5T4D3 | 178 | ALFFLLSFLGYCKAFRESNKEGAHSSTFWVLLSIFLGAVAML   219 (462) |
|  | Q Consensus | 178 | ~~f~ll~~~~~~~~~~~~~~~~~~~~~~~~~~~~~~~~la~l   219 (462) |
|  |  |  | .++.+.+++++....|+++.. ..|-.++.+++.++.. |
|  | T Consensus | 20 | ~LL~~aCIFAfidFSK~~~~~-----~~WRaLSI~~Filgii   56 (92) |
|  | T PF05767.13 | 20 | ALLVLACIFAFVEFSKVTTSD-----YTWRALCIVCFITGMV   56 (92) |
|  | T ss\_pred |  | HHHHHHHHHHHHHhcCCCCCh-----HHHHHHHHHHHHHHHH |
|  |
| --- | | | |
|  | Template alignmentCDD | | |
| 32. | PF09323.11 ; DUF1980 ; Domain of unknown function (DUF1980) | | |
|  | Probability: 22.74%, E-value: 160, Score: 21.19, Aligned cols: 73, Identities: 12%, Similarity: 0.022, | | |
|  |
|  | Q ss\_pred |  | HHHHHHHHHHHHhhHhccccccchhHHHhhhhHHHHHHHHHHHHHHHHHHhchhhH------------------------ |
|  | Q Q5T4D3 | 384 | LTLGLGFLVIPFLPASNLFFRVGFVVAERVLYLPSVGYCVLLTFGFGALSKHTKKK------------------------   439 (462) |
|  | Q Consensus | 384 | ~~~~~~~~~~~~~~~~~~~~~~~~~~~~ry~~~~~~~~~ll~a~~~~~~~~~~~~~------------------------   439 (462) |
|  |  |  | ....++..+..++......-....+..+|+........++++..++..+.+..+.. |
|  | T Consensus | 2 | l~~lill~~~~~l~~l~~tg~i~~yI~Pr~~~~~~~a~~~l~il~~~~~~~~~~~~~~~~~~~~~~~~~~~h~~~~~~~~   81 (187) |
|  | T PF09323.11 | 2 | LRFIVLFGFAYFFMKLHATGDISKYINMKYAYLSFSMIFAMGFLCLYQLVKWVRAGNEAHDHQHGAHHGHSHETDENTWY   81 (187) |
|  | T ss\_pred |  | HHHHHHHHHHHHHHHHHHhCCHHHHcCcchHHHHHHHHHHHHHHHHHHHHHHHHccCcccccccCCCCCCCCCCCcchhH |
|  |
|  |
|  | Q ss\_pred |  | -HHHHHHHHHHHHHHHHH |
|  | Q Q5T4D3 | 440 | -KLIAAVVLGILFINTLR   456 (462) |
|  | Q Consensus | 440 | -~~~~~~~~~~~~~~~~~   456 (462) |
|  |  |  | ......++++-++..+. |
|  | T Consensus | 82 | ~~~~~~~~~~lPlll~~l   99 (187) |
|  | T PF09323.11 | 82 | KKMFTYGMILIPVVTGIF   99 (187) |
|  | T ss\_pred |  | HHHHHHHHHHHHHHHHHh |
|  |
| --- | | | |
|  | Template alignmentCDD | | |
| 33. | PF12273.9 ; RCR ; Chitin synthesis regulation, resistance to Congo red | | |
|  | Probability: 21.99%, E-value: 32, Score: 23.79, Aligned cols: 28, Identities: 18%, Similarity: 0.405, | | |
|  |
|  | Q ss\_pred |  | HHHHHHHHHHHHHHHHHHHHHhcCCCcc |
|  | Q Q5T4D3 | 353 | WRVIALAALWFCLIGLICQALCSEDGHK   380 (462) |
|  | Q Consensus | 353 | ~~~~~~~~~~~~~~~~~~~~~~~~~~~~   380 (462) |
|  |  |  | |..+..++++++++++++.+..|+++++ |
|  | T Consensus | 2 | Wvl~~iii~~~l~~~~~~~~~~RRRrr~   29 (138) |
|  | T PF12273.9 | 2 | WVVLAGVIVIVLVIFMLCTCTARRRRRR   29 (138) |
|  | T ss\_pred |  | HHHHHHHHHHHHHHHHHHHHHHHHHHHc |
|  |

---

If you use HHpred on our Toolkit for your research, please cite as appropriate:

A Completely Reimplemented MPI Bioinformatics Toolkit
with a New HHpred Server at its Core.  
Zimmermann L, Stephens A, Nam SZ, Rau D,
Kübler J, Lozajic M, Gabler F, Söding J, Lupas AN, Alva V.
J Mol Biol. 2018 Jul 20. S0022-2836(17)30587-9.

  

Protein homology detection by HMM-HMM comparison.  
Söding J. Bioinformatics. 2005 Apr 1;21(7):951-60.  
  
Fast and accurate automatic structure prediction with HHpred.  
Hildebrand A, Remmert M, Biegert A, Söding J. Proteins. 2009;77 Suppl 9:128-32.  
  
Automatic Prediction of Protein 3D Structures by Probabilistic Multi-template Homology Modeling.  
Meier A, Söding J. PLoS Comput Biol. 2015 Oct 23;11(10):e1004343.

Download

---

If you use HHpred on our Toolkit for your research, please cite as appropriate:

A Completely Reimplemented MPI Bioinformatics Toolkit
with a New HHpred Server at its Core.  
Zimmermann L, Stephens A, Nam SZ, Rau D,
Kübler J, Lozajic M, Gabler F, Söding J, Lupas AN, Alva V.
J Mol Biol. 2018 Jul 20. S0022-2836(17)30587-9.

  

Protein homology detection by HMM-HMM comparison.  
Söding J. Bioinformatics. 2005 Apr 1;21(7):951-60.  
  
Fast and accurate automatic structure prediction with HHpred.  
Hildebrand A, Remmert M, Biegert A, Söding J. Proteins. 2009;77 Suppl 9:128-32.  
  
Automatic Prediction of Protein 3D Structures by Probabilistic Multi-template Homology Modeling.  
Meier A, Söding J. PLoS Comput Biol. 2015 Oct 23;11(10):e1004343.

Loading...

---

If you use HHpred on our Toolkit for your research, please cite as appropriate:

A Completely Reimplemented MPI Bioinformatics Toolkit
with a New HHpred Server at its Core.  
Zimmermann L, Stephens A, Nam SZ, Rau D,
Kübler J, Lozajic M, Gabler F, Söding J, Lupas AN, Alva V.
J Mol Biol. 2018 Jul 20. S0022-2836(17)30587-9.

  

Protein homology detection by HMM-HMM comparison.  
Söding J. Bioinformatics. 2005 Apr 1;21(7):951-60.  
  
Fast and accurate automatic structure prediction with HHpred.  
Hildebrand A, Remmert M, Biegert A, Söding J. Proteins. 2009;77 Suppl 9:128-32.  
  
Automatic Prediction of Protein 3D Structures by Probabilistic Multi-template Homology Modeling.  
Meier A, Söding J. PLoS Comput Biol. 2015 Oct 23;11(10):e1004343.

Loading hits...

---

If you use HHpred on our Toolkit for your research, please cite as appropriate:

A Completely Reimplemented MPI Bioinformatics Toolkit
with a New HHpred Server at its Core.  
Zimmermann L, Stephens A, Nam SZ, Rau D,
Kübler J, Lozajic M, Gabler F, Söding J, Lupas AN, Alva V.
J Mol Biol. 2018 Jul 20. S0022-2836(17)30587-9.

  

Protein homology detection by HMM-HMM comparison.  
Söding J. Bioinformatics. 2005 Apr 1;21(7):951-60.  
  
Fast and accurate automatic structure prediction with HHpred.  
Hildebrand A, Remmert M, Biegert A, Söding J. Proteins. 2009;77 Suppl 9:128-32.  
  
Automatic Prediction of Protein 3D Structures by Probabilistic Multi-template Homology Modeling.  
Meier A, Söding J. PLoS Comput Biol. 2015 Oct 23;11(10):e1004343.

Loading hits...

---

If you use HHpred on our Toolkit for your research, please cite as appropriate:

A Completely Reimplemented MPI Bioinformatics Toolkit
with a New HHpred Server at its Core.  
Zimmermann L, Stephens A, Nam SZ, Rau D,
Kübler J, Lozajic M, Gabler F, Söding J, Lupas AN, Alva V.
J Mol Biol. 2018 Jul 20. S0022-2836(17)30587-9.

  

Protein homology detection by HMM-HMM comparison.  
Söding J. Bioinformatics. 2005 Apr 1;21(7):951-60.  
  
Fast and accurate automatic structure prediction with HHpred.  
Hildebrand A, Remmert M, Biegert A, Söding J. Proteins. 2009;77 Suppl 9:128-32.  
  
Automatic Prediction of Protein 3D Structures by Probabilistic Multi-template Homology Modeling.  
Meier A, Söding J. PLoS Comput Biol. 2015 Oct 23;11(10):e1004343.

- Help
- FAQ
- Privacy Policy
- Imprint
- Contact Us
- Cite Us
- Recent Updates

© 2008-2020, Dept. of Protein Evolution, Max Planck Institute for Developmental Biology, Tübingen

Template 3D Structure: 
×

Loading...
