# Supplementary material for: Conserved sequence motifs in human TMTC1, TMTC2, TMTC3, and TMTC4, new O-mannosyltransferases from the GT-C/PMT clan, are rationalized as ligand binding sites
Source: Biol Direct. 2021 Jan 12;16:4. doi: 10.1186/s13062-021-00291-w (PMC7801869; doi:10.1186/s13062-021-00291-w)
Supplement: Supplementary file 3 — Additional file 3. HHPred outputs when searching TMTCs against Pfam or PDB structures. The compressed library file AF3-2020-06-HHPred-TMTCs.zip contains the outputs when running the four human TMTC sequences as input of HHPred against PDB sequences and against Pfam domains (as of 23rd of June 2020). [file 13062_2021_291_MOESM3_ESM.zip › AF3-2020-06-HHPred-TMTCs/index.html]

Supporting Information


**Additional File 3   
  
 Supporting Information for HHpred Analysis of TMTCs**
  
  
Filename HHpred\_TMTC1\_PDB.html : download
  
  
Filename HHpred\_TMTC2\_PDB.html : download
  
  
Filename HHpred\_TMTC3\_PDB.html : download
  
  
Filename HHpred\_TMTC4\_PDB.html : download
  
  
Filename HHpred\_TMTC1\_Pfam.html : download
  
  
Filename HHpred\_TMTC2\_Pfam.html : download
  
  
Filename HHpred\_TMTC3\_Pfam.html : download
  
  
Filename HHpred\_TMTC4\_Pfam.html : download

Additional information for the publication
  
**"Conserved sequence motifs in human TMTC1, TMTC2, TMTC3, and TMTC4, new O-mannosyltransferases from the GT-C/PMT clan, are rationalized as ligand binding sites"**
  
by Birgit Eisenhaber, Swati Sinha, Chaitanya K. Jadalanki, Vladimir A. Shitov, Qiao Wen Tan, Fernanda L. Sirota, Frank Eisenhaber
