# Supplementary figures and images for: Conserved sequence motifs in human TMTC1, TMTC2, TMTC3, and TMTC4, new O-mannosyltransferases from the GT-C/PMT clan, are rationalized as ligand binding sites
Source: Biol Direct. 2021 Jan 12;16:4. doi: 10.1186/s13062-021-00291-w (PMC7801869; doi:10.1186/s13062-021-00291-w)

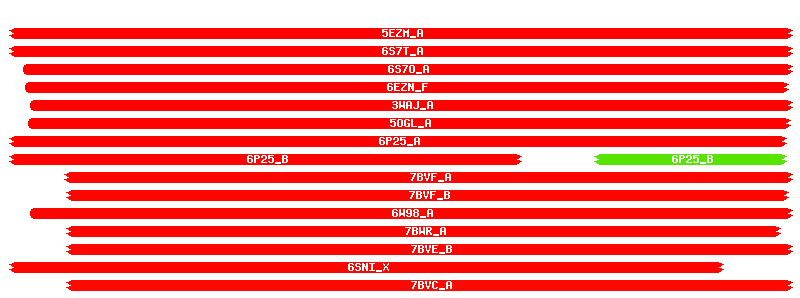

Supplement: Supplementary file 3 — Additional file 3. HHPred outputs when searching TMTCs against Pfam or PDB structures. The compressed library file AF3-2020-06-HHPred-TMTCs.zip contains the outputs when running the four human TMTC sequences as input of HHPred against PDB sequences and against Pfam domains (as of 23rd of June 2020). [file 13062_2021_291_MOESM3_ESM.zip › AF3-2020-06-HHPred-TMTCs/HHpred_TMTC1_PDB_files/1780993.txt]

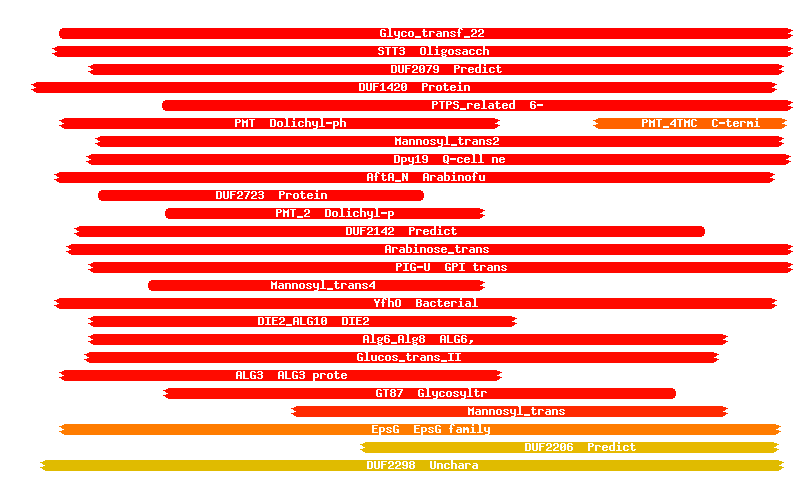

Supplement: Supplementary file 3 — Additional file 3. HHPred outputs when searching TMTCs against Pfam or PDB structures. The compressed library file AF3-2020-06-HHPred-TMTCs.zip contains the outputs when running the four human TMTC sequences as input of HHPred against PDB sequences and against Pfam domains (as of 23rd of June 2020). [file 13062_2021_291_MOESM3_ESM.zip › AF3-2020-06-HHPred-TMTCs/HHPred_TMTC1_PFam_files/2161064.txt]

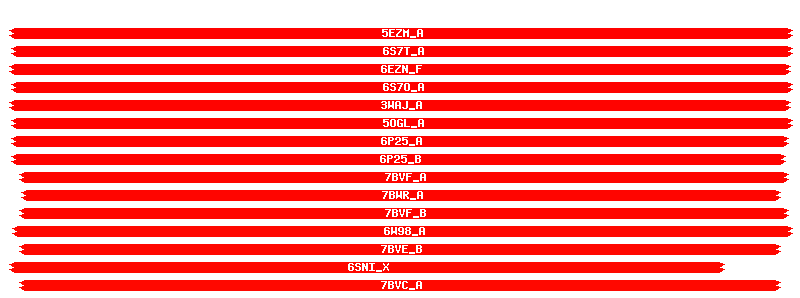

Supplement: Supplementary file 3 — Additional file 3. HHPred outputs when searching TMTCs against Pfam or PDB structures. The compressed library file AF3-2020-06-HHPred-TMTCs.zip contains the outputs when running the four human TMTC sequences as input of HHPred against PDB sequences and against Pfam domains (as of 23rd of June 2020). [file 13062_2021_291_MOESM3_ESM.zip › AF3-2020-06-HHPred-TMTCs/HHpred_TMTC2_PDB_files/3084060.txt]

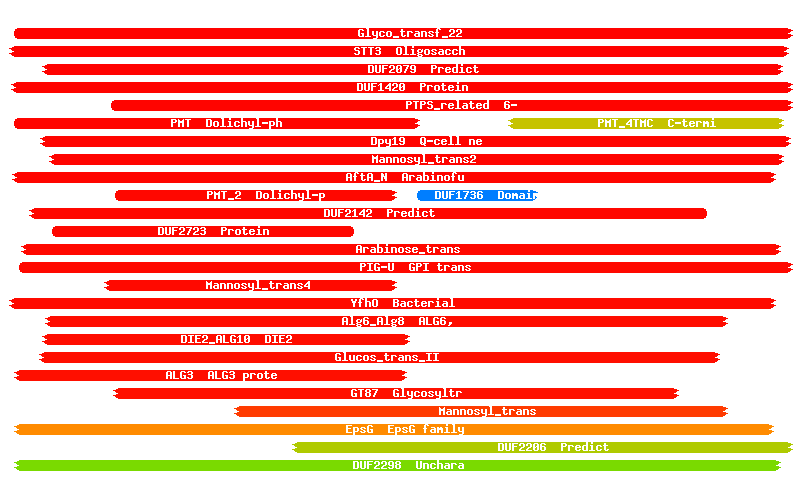

Supplement: Supplementary file 3 — Additional file 3. HHPred outputs when searching TMTCs against Pfam or PDB structures. The compressed library file AF3-2020-06-HHPred-TMTCs.zip contains the outputs when running the four human TMTC sequences as input of HHPred against PDB sequences and against Pfam domains (as of 23rd of June 2020). [file 13062_2021_291_MOESM3_ESM.zip › AF3-2020-06-HHPred-TMTCs/HHpred_TMTC2_PFam_files/8665047.txt]

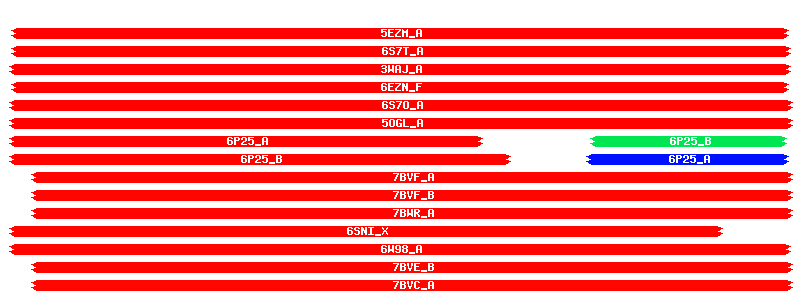

Supplement: Supplementary file 3 — Additional file 3. HHPred outputs when searching TMTCs against Pfam or PDB structures. The compressed library file AF3-2020-06-HHPred-TMTCs.zip contains the outputs when running the four human TMTC sequences as input of HHPred against PDB sequences and against Pfam domains (as of 23rd of June 2020). [file 13062_2021_291_MOESM3_ESM.zip › AF3-2020-06-HHPred-TMTCs/HHpred_TMTC3_PDB_files/8776670.txt]

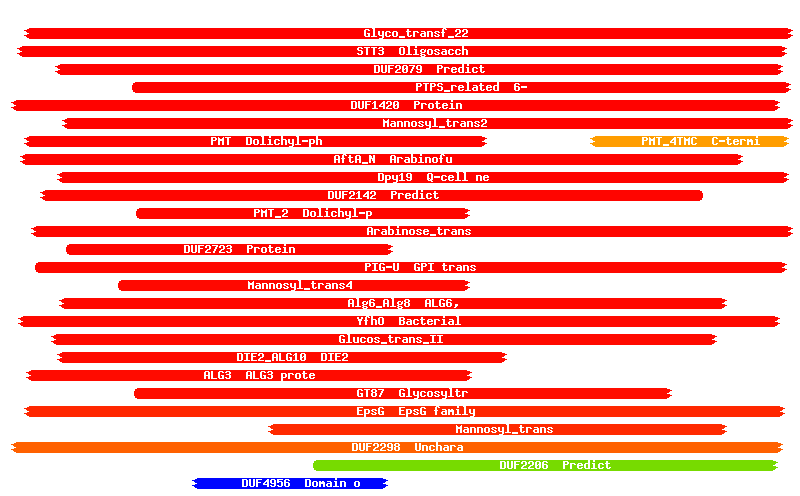

Supplement: Supplementary file 3 — Additional file 3. HHPred outputs when searching TMTCs against Pfam or PDB structures. The compressed library file AF3-2020-06-HHPred-TMTCs.zip contains the outputs when running the four human TMTC sequences as input of HHPred against PDB sequences and against Pfam domains (as of 23rd of June 2020). [file 13062_2021_291_MOESM3_ESM.zip › AF3-2020-06-HHPred-TMTCs/HHPred_TMTC3_PFam_files/5407837.txt]

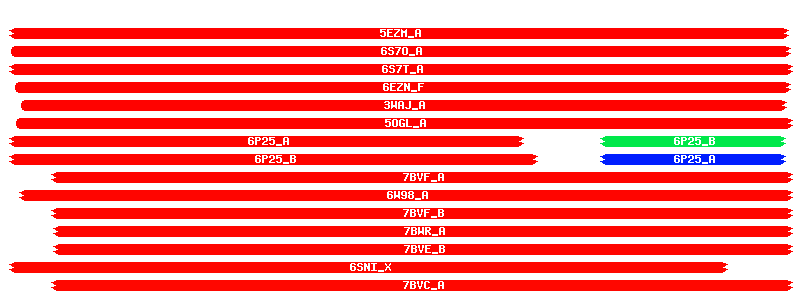

Supplement: Supplementary file 3 — Additional file 3. HHPred outputs when searching TMTCs against Pfam or PDB structures. The compressed library file AF3-2020-06-HHPred-TMTCs.zip contains the outputs when running the four human TMTC sequences as input of HHPred against PDB sequences and against Pfam domains (as of 23rd of June 2020). [file 13062_2021_291_MOESM3_ESM.zip › AF3-2020-06-HHPred-TMTCs/HHpred_TMTC4_PDB_files/8527802.txt]

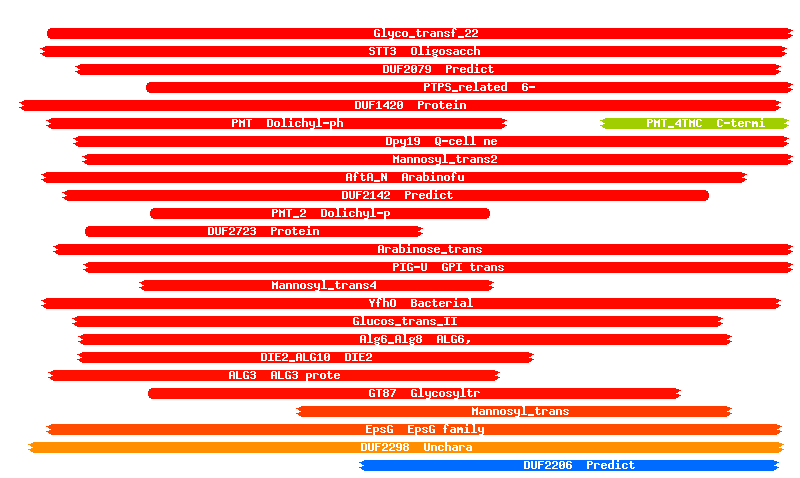

Supplement: Supplementary file 3 — Additional file 3. HHPred outputs when searching TMTCs against Pfam or PDB structures. The compressed library file AF3-2020-06-HHPred-TMTCs.zip contains the outputs when running the four human TMTC sequences as input of HHPred against PDB sequences and against Pfam domains (as of 23rd of June 2020). [file 13062_2021_291_MOESM3_ESM.zip › AF3-2020-06-HHPred-TMTCs/HHpred_TMTC4_Pfam_files/5863267.txt]
