# Supplementary material for: Conserved sequence motifs in human TMTC1, TMTC2, TMTC3, and TMTC4, new O-mannosyltransferases from the GT-C/PMT clan, are rationalized as ligand binding sites
Source: Biol Direct. 2021 Jan 12;16:4. doi: 10.1186/s13062-021-00291-w (PMC7801869; doi:10.1186/s13062-021-00291-w)
Supplement: Supplementary file 5 — Additional file 5. Atomic coordinates of 3D structural models of all four TMTCs with ligands (divalent metal ion and dolichyl-phosphate-mannose. The file AF5-2020-10-3Dmodel-TMTCs.zip provides the atomic coordinates for the 3D structural models of the four human TMTCs generated with template structures 5ezm and 5f15. [file 13062_2021_291_MOESM5_ESM.zip › AF5-2020-10-3Dmodel-TMTCs/index.html]

Supporting Information


**Additional File 5   
  
 Supporting Information for 3D Structural Models of TMTCs**
  
  
atomic coordinates in classical PDB format
  
  
Model for TMTC1 :  structure including hydrogens
  
  
Model for TMTC1 :  structure without hydrogens
  
  
Model for TMTC2 :  structure including hydrogens
  
  
Model for TMTC2 :  structure without hydrogens
  
  
Model for TMTC3 :  structure including hydrogens
  
  
Model for TMTC3 :  structure without hydrogens
  
  
Model for TMTC4 :  structure including hydrogens
  
  
Model for TMTC4 :  structure without hydrogens

Additional information for the publication
  
**"Conserved sequence motifs in human TMTC1, TMTC2, TMTC3, and TMTC4, new O-mannosyltransferases from the GT-C/PMT clan, are rationalized as ligand binding sites"**
  
by Birgit Eisenhaber, Swati Sinha, Chaitanya K. Jadalanki, Vladimir A. Shitov, Qiao Wen Tan, Fernanda L. Sirota, Frank Eisenhaber
